# Downregulated Salt-inducible Kinase 3 Expression Promotes Chemoresistance in Serous Ovarian Cancer via the ATP‐binding Cassette Protein ABCG2

**DOI:** 10.7150/jca.34886

**Published:** 2019-10-15

**Authors:** Yu-Ling Liang, Chin-Han Wu, Chieh-Yi Kang, Chang-Ni Lin, Neng-Yao Shih, Sheng-Hsiang Lin, Yeong-Chang Chen, Keng-Fu Hsu

**Affiliations:** 1 Department of Obstetrics and Gynecology, National Cheng Kung University Hospital, College of Medicine, National Cheng Kung University, Tainan, Taiwan;; 2 Department of Dental Technology, Shu Zen Junior College of Medicine and Management;; 3 Department of Obstetrics and Gynecology, Chi Mei Medical Center, Yongkang, Tainan, Taiwan;; 4 National Institute of Cancer Research, National Health Research Institutes;; 5 Graduate Institute of Clinical Medicine, National Cheng Kung University Hospital, College of Medicine, National Cheng Kung University, Tainan, Taiwan.

**Keywords:** ovarian cancer, SIK3, ABCG2, chemoresistance

## Abstract

**Background:** Epithelial ovarian cancer (EOC) has a high tumor-associated mortality rate among gynecological cancers. Although CA125 is a well-studied biomarker for ovarian cancer, it is also elevated under numerous conditions, resulting in decreased specificity. Recently, we identified a novel tumor-associated antigen, salt-inducible kinase 3 (SIK3), during tumorigenesis in ovarian cancer. However, the association between SIK3 expression and patient outcomes in ovarian cancer remains unclear.

**Materials and Methods:** We collected EOC samples from 204 patients and examined tumor SIK3 expression by immunohistochemistry (IHC) and CA125 expression in tumors and serum. The expression levels of SIK3 and CA125 were correlated with patient survival. SIK3 expression was silenced with SIK3-specific shRNAs to investigate the possible mechanisms related to chemoresistance in serous-type ovarian cancer cell lines OVCAR4 and SKOV3.

**Results:** In advanced-stage serous ovarian cancer, patients with low SIK3 expression have poorer overall survival (OS) and progression-free survival (PFS) than patients with high SIK3 expression. Ovarian cancer cells with SIK3 knockdown display increased chemoresistance to Taxol plus cisplatin treatment, which is associated with the upregulation of the ABCG2 transporter. In addition, in serous ovarian cancer, SIK3 expression is inversely correlated to ABCG2 expression, and patients with low SIK3 and high ABCG2 expression have worse prognosis than patients with high SIK3 and low ABCG2 expression.

**Conclusion:** Our results demonstrated that serous EOC patients with low SIK3 expression have poor prognosis, which is associated with chemoresistance mediated by ABCG2 upregulation. SIK3 and ABCG2 expression levels may be potential prognostic markers to predict the outcome in serous EOC patients.

## Introduction

Epithelial ovarian cancer (EOC) has a high tumor-associated mortality rate because EOC is usually diagnosed at an advanced stage (stage III or IV). The five-year survival rate is approximately 40% for patients who are initially diagnosed with advanced-stage disease 1. Most women with EOC achieve remission with a combination of surgical resection and platinum-based chemotherapy. More than 70% of these patients relapse after debulking surgery and chemotherapy, often due to the development of resistance against platinum-containing regimens 2. Cancer antigen 125 (CA125), a member of the mucin family of glycoproteins 3, is widely used as a surrogate serum biomarker for monitoring the efficacy of treatment response in ovarian cancer 4, 5. However, elevated CA125 is also observed in other diseases, i.e., pancreatic, lung, breast, colorectal and gastrointestinal cancer or some benign conditions, i.e., cirrhosis, hepatitis 6, which compromises the specificity of CA125 in ovarian cancer. Although early decrease in serum CA125 has been reported as a potential surrogate for better survival 7, there are still no reliable biomarkers to identify patients who are most likely to experience disease recurrence or who are likely to respond to primary therapy. Therefore, more specific prognostic markers are urgently needed.

Salt-inducible kinases (SIKs) are highly conserved serine/threonine protein kinases that belong to a family of AMP-activated protein kinases (AMPKs) 8 and may have a role in steroidogenesis, adipogenesis or regulation of tumor malignancy 9, 10. The SIK family contains three isoforms: SIK1, which is expressed in the adrenal cortex; SIK2, which is more specific to adipose tissues; and SIK3, which is ubiquitously expressed. Loss of SIK1 expression facilitates lung metastasis in mice and is correlated with the development of distant metastasis in human breast cancer 11. Downregulation of SIK1 accelerates the growth and invasion of hepatocellular carcinoma through RNF2 12. SIK2 has been reported to be overexpressed in high-grade serous ovarian cancer in which it functions as a centrosome kinase during cell cycle progression 13. Higher expression of SIK2 significantly correlated with poorer survival 13. Moreover, overexpression of SIK2 may promote omental ovarian cancer metastasis by activating the phosphatidylinositol 3-kinase pathway 14. Unlike the biological functions of SIK1 and SIK2, the functions of SIK3 are unknown, particularly in cancer. SIK3 has been reported to be overexpressed in high salt/IL-17 environments and mediate cell proliferation, inflammation and metastasis in MCF-7 breast cancer cells 15. Previously, through a phage display system from ovarian cancer ascites, we identified SIK3 as a novel EOC-specific tumor-associated antigen 16. SIK3 overexpression markedly promoted cell proliferation and enabled cells to grow in mice. Decreased SIK3 expression in SKOV3 cells consistently abolished SKOV3 tumorigenic potency through modulation of the protein levels of cell cycle regulators.

Despite therapeutic advances in EOC, the development of chemoresistance in tumors is still an important problem. Most studies of multidrug resistance (MDR) in cancer are on ATP‐binding cassette proteins (ABC proteins) that increase drug efflux and decrease the accumulation and efficiency of drugs inside cancer cells 17, 18. High expression levels of ABCB1 (P-glycoprotein, P-gp), ABCC1 (multidrug resistance-associated protein 1, MRP1), and ABCG2 (breast cancer resistance protein, BCRP) have been reported to be associated with chemoresistance and adverse outcomes in EOC 19-21. Although we have observed *in vitro* that SIK3 is important for ovarian cancer growth, the associations between SIK3 and chemoresistance-associated proteins i.e., ABC proteins, as well as clinical survival in ovarian cancer, are still largely unknown.

## Patients, Materials and Methods

### Patients

After approval by the Institutional Review Board of the National Cheng Kung University Hospital (NCKUH), patients who underwent primary surgery for EOC from NCKUH were consecutively enrolled between July 1999 and October 2011. Patient clinical data were collected and included the FIGO stage, presurgery serum CA125 level, clinicopathologic characteristics, surgery record, treatment modality, recurrence status and survival status. Optimal cytoreduction was considered when the maximum diameter of residual disease was less than 1 centimeter. Survival time was calculated from the date of surgery. Overall survival (OS) was determined based on the date of death or the date of last contact for living patients. Progression-free survival (PFS) was determined based on the date of first progression or death, whichever occurred first, or the date of last contact for living patients with or without recurrent disease. Disease progression was based on the Response Evaluation Criteria in Solid Tumors (RECIST) or serially increasing CA125 levels or any clinical or radiographic evidence of new lesions as either local/regional relapse or distant metastasis 22, 23. All patients received adjuvant platinum-based chemotherapy except patients with stage IA, grade 1 disease. Patients with disease progression or disease recurrence <6 months after discontinuing chemotherapy were defined as chemoresistant, whereas patients without a recurrence or with recurrence ≥6 months after discontinuing chemotherapy were defined as chemosensitive 24. All procedures were carried out in accordance with the approved guidelines of NCKUH. The exclusion criteria were as follows: no histologic confirmation of the diagnosis or inadequate data in the medical record.

### Immunohistochemistry and quantification of CA125, SIK3 and ABCG2

Immunohistochemistry (IHC) was performed using a conventional method as described previously 16. Formalin-fixed paraffin-embedded tissue samples were obtained and stained with a GST control antibody or anti-CA125, anti-SIK3 (obtained from Dr. Shih's lab) and anti-ABCG2 (Abcam) antibodies. H&E staining was performed to verify that the tumor cell composition of the paraffin sections was at least sixty percent. Then, serial sections of the tissues were used to determine the percentage of SIK3 and CA125 protein expression. Briefly, the sections were serially dewaxed, rehydrated, and treated for antigen retrieval by heating with 10 mM sodium citrate (pH 6.0) for 20 min. After blocking endogenous peroxidases with 3% hydrogen peroxide, the tumor sections were incubated with the primary antibody (1:20 for CA125, 1:4000 for SIK3, and 1:2000 for ABCG2) overnight at 4°C. The bound primary antibodies were detected using the LSAB kit (Dako, Carpinteria, CA), and the slides were counterstained with hematoxylin.

For the observer-assisted analysis of CA125, SIK3 and ABCG2 localization and staining intensity in ovarian cancer, a quantitative evaluation was performed with a score based on the percentage of positive cells. All optical fields were examined, and five representative fields at ×200 magnification were evaluated in each case. For each case, the percentage of positively stained tumor cells was recorded by two experienced gynecologic oncologists (YLL and KFH). The mean values of the results from both observers were used for all further calculations. Based on time-dependent receiver operating characteristic (ROC) curves, we defined the best cut-off point for SIK3 and CA125. Patients with more positively stained tumor cells than the cut-off point were defined to have “high expression”, and patients with less positively stained tumor cells than the cut-off point were defined to have “low expression”.

### Cell culture and gene modulation

Two serous-type ovarian cancer cell lines, namely, OVCAR4, purchased from American Type Culture Collection (ATCC; Manassas, VA USA), and SKOV3, purchased from the European Collection of Cell Cultures (ECACC; Salisbury, Scotland), were used. OVCAR4 and SKOV3 cells were cultured in RPMI 1640 and McCoy's 5A medium, respectively, supplemented with 10% fetal bovine serum (FBS), penicillin (100 U/ml)/streptomycin (100 mg/ml) (Invitrogen, Carlsbad, CA, USA). To generate cells with stable knockdown of SIK3, we transfected OVCAR4 and SKOV3 cells with SIK3-specific small hairpin RNA (strain#01: 5'-CCGGGAAGCATTGGTGCGCTATTTGCTCGAGCAAATAGCGCACCAATGCTTCTTTTTG-3' and strain#61: 5'-CCGGGCCAGGCTTTATCTTATCAAACTCGAGTTTGATAAGATAAAGCCTGGCTTTTTG-3') (National RNAi Core Facility, Academia Sinica, Taiwan). Simultaneously, their corresponding control cells were also established by transfection with the pLKO.1-luciferase vector (Luc) and selection in medium containing 2.5 mg/ml puromycin. After limiting dilution, the expression levels in individual cell clones were confirmed by immunoblotting analyses.

### RNA isolation, reverse transcription and real-time quantitative PCR

Total RNA was extracted from cultured ovarian cancer cell lines using TRIzol reagent (Invitrogen, Carlsbad, CA). One microgram of total RNA was reverse transcribed using the GoScript^TM^ transcription system (Promega, Madison, WI, USA) according to the manufacturer's specification. Quantitative real-time PCR was performed using the QuantiFast SYBR Green PCR kit (Qiagen), and the results were normalized according to the expression levels of beta-actin RNA. Fold changes for the target gene were calculated as 2^-∆∆CT^. The primers used for real-time PCR are shown in Supplementary Table 1.

### Gene expression profiling and data normalization

TRIzol-isolated RNA samples were shipped on dry ice to Welgene Biotech (Taiwan), where the gene expression microarray experiments were performed as a contract service. The labeling of 100 ng of total RNA was performed using the Low Input Quick Amp Labeling Kit (Agilent Technologies). A total of 0.6 μg of cyanin-3-labeled cRNA probe was fragmented and hybridized to the Agilent SurePrint G3 Human GE v2 8x60K Microarray using a Gene Expression Hybridization Kit (Agilent) according to the manufacturer's instructions. Then, the microarrays were scanned using an Agilent G2565CA Microarray Scanner with Scan Control software v8.5. The background-subtracted and normalized expression data were normalized by deducting group mean values. The fold change for each gene was calculated by dividing the SIK3-silenced group mean RNA expression by the luciferase-silenced group mean RNA expression levels. To identify relevant dysregulated RNA transcripts, we considered differential expression if the fold-change was > 1.5, and the adjusted p-value (false discovery rate) was < 0.05.

### Western blotting analysis

In brief, cells were lysed in lysis buffer (50 mM Tris-HCl, pH 7.4, 5 mM MgCl2, 1% Nonidet P-40, 150 mM NaCl, 1 mM phenylmethylsulfonyl fluoride) containing protease inhibitors (Roche, Molecular Biochemicals, Mannheim, Germany). The cell lysates (50 mg) were subjected to 10% SDS-PAGE and subsequently transferred onto a PVDF membrane (Millipore, Billerica, MA). After blocking with 5% nonfat milk, the membranes were incubated with anti-SIK3 (obtained from Dr. Shih's lab) or anti-ABCG2 antibody at 4°C overnight. Monoclonal α-tubulin antibody (Sigma-Aldrich) was used as a loading control. The protein expression was probed with peroxidase-coupled secondary antibodies and then detected by enhanced chemiluminescence (Amersham Pharmacia, Uppsala, Sweden).

### ABCG2 functional assay

To detect the multidrug resistant phenotype of ABCG2 overexpression in OVCAR4 and SKOV3 cells with SIK3 knockdown, we used an MDR Assay Kit (Abcam) to check the function of ABCG2 by flow cytometry. According to the manufacturer's protocol, 2 x 10^5^ suspended cells were pretreated with the inhibitor novobiocin (50 nM) or DMSO and incubated at 37°C for 5 minutes. Then, freshly diluted Efflux Gold Detection Reagent was added to each tube and incubated at 37°C for 30 minutes. Cell viability was monitored by propidium iodide (PI) staining. The cellular orange fluorescence signal of the Efflux Gold Detection Reagent was measured immediately by flow cytometry in the living (PI-negative) cell population with identical equipment settings. The multidrug resistance activity factor (MAF) was calculated using the following formula: MAFBCRP (or ABCG2) = 100 × (FBCRP - F0)/FBCRP. MAF values ≥25 indicated multidrug resistance positivity.

### Animal studies

Female, 6- to 8-week-old severe combined immunodeficient (NOD/SCID) mice were obtained from the Laboratory Animal Center of the National Cheng Kung University. The animals were maintained in specific pathogen-free animal care facility under isothermal conditions with regular photoperiods. The experimental protocol adhered to the rules of the Animal Protection Act of Taiwan and was approved by the Laboratory Animal Care and Use Committee of the National Cheng Kung University. To examine the effect of chemoresistance of SIK3 silencing on tumor growth, SKOV3 cells expressing either a control shRNA (shLuc) or shRNA targeting SIK3 (shSIK3#01 and shSIK3#61) were subcutaneously inoculated into the posterior flank of NOD/SCID mice. One week after tumor cell injection, all mice were intraperitoneally (i.p.) injected with cisplatin (1 mg/kg) twice per week. Tumors were measured twice a week with calipers to determine the length (L) and width (W), and the volume was calculated using the formula (L×W×W×0.5). At day 45 after tumor injection, mice were sacrificed and tumor weights were measured.

### Statistical analyses

Statistical analysis of OS and PFS were performed using SPSS software (Version 22.0; IBM Corp, Armonk, NY). Survival curves were generated using the Kaplan-Meier method, and differences in survival were assessed by the log-rank test. Univariate and multivariate survival analyses were performed using Cox proportional hazards models to identify prognostic factors. Factors that were prognostically relevant in the univariate analysis were included in the multivariate Cox analysis. The optimal cut-off values for tumor SIK3 and CA125 expression and presurgery serum CA125 level were determined using time-dependent ROC curve analysis. C-statistics provided the overall measures of predictive accuracy, and time-dependent ROC curves and areas under the curves (AUCs) were used to summarize the predictive accuracy at specific times. Time-dependent ROC analysis was performed using R software (R-3.3.2, R Foundation for Statistical Computing, http://www.r-project.org/). SIK3 expression was categorized into two groups as <47.5% and ≥47.5%, while tissue CA125 expression was categorized as <14% and ≥14%. A presurgery serum CA125 level of ≥314.8 U/ml was considered to be elevated. Effects were expressed as hazard ratios (HRs) with 95% confidence intervals (CIs). The relationship between ABCG2 and SIK3 was determined by Ingenuity Pathway Analysis (IPA), which is a web-based software application for the analysis, integration, and interpretation of data derived from microarrays. Numerical measurements for the intensity of ABCG2 and SIK3 were performed using Pearson's correlation coefficients. Linear regression was used to model the relationship between ABCG2 and SIK3. A P value <0.05 was considered statistically significant.

## Results

A total of 204 patients with FIGO stage I-IV ovarian cancer were enrolled in this study. The demographic information of the patients is shown in Table 1. The mean age at diagnosis was 52.8 years (range 25-82 years). Most patients (61.3%) had stage IIIC or IV disease and serous histology (51.5%). Approximately 30.9% of the patients had stage I disease, 7.8% had stage II disease, 52.4% had stage III disease, and 8.9% had stage IV disease. There were 151 patients (74%) who underwent optimal debulking (tumor<1 cm) surgery. At the time of the last follow-up, 78 patients (78/204=38%) were alive without evidence of disease, 27 (27/204=13%) were alive with disease, 91 (91/204=45%) were dead from disease, and 8 (8/204=4%) had died from other causes. The median OS and PFS were 49.5 months (range 0.25-205 months) and 28.4 months (range 0.25-182.4 months), respectively.

### Immunostaining of SIK3 and CA125 in ovarian cancer; high SIK3 expression was significantly associated with increased chemosensitivity

**Figure 1:**
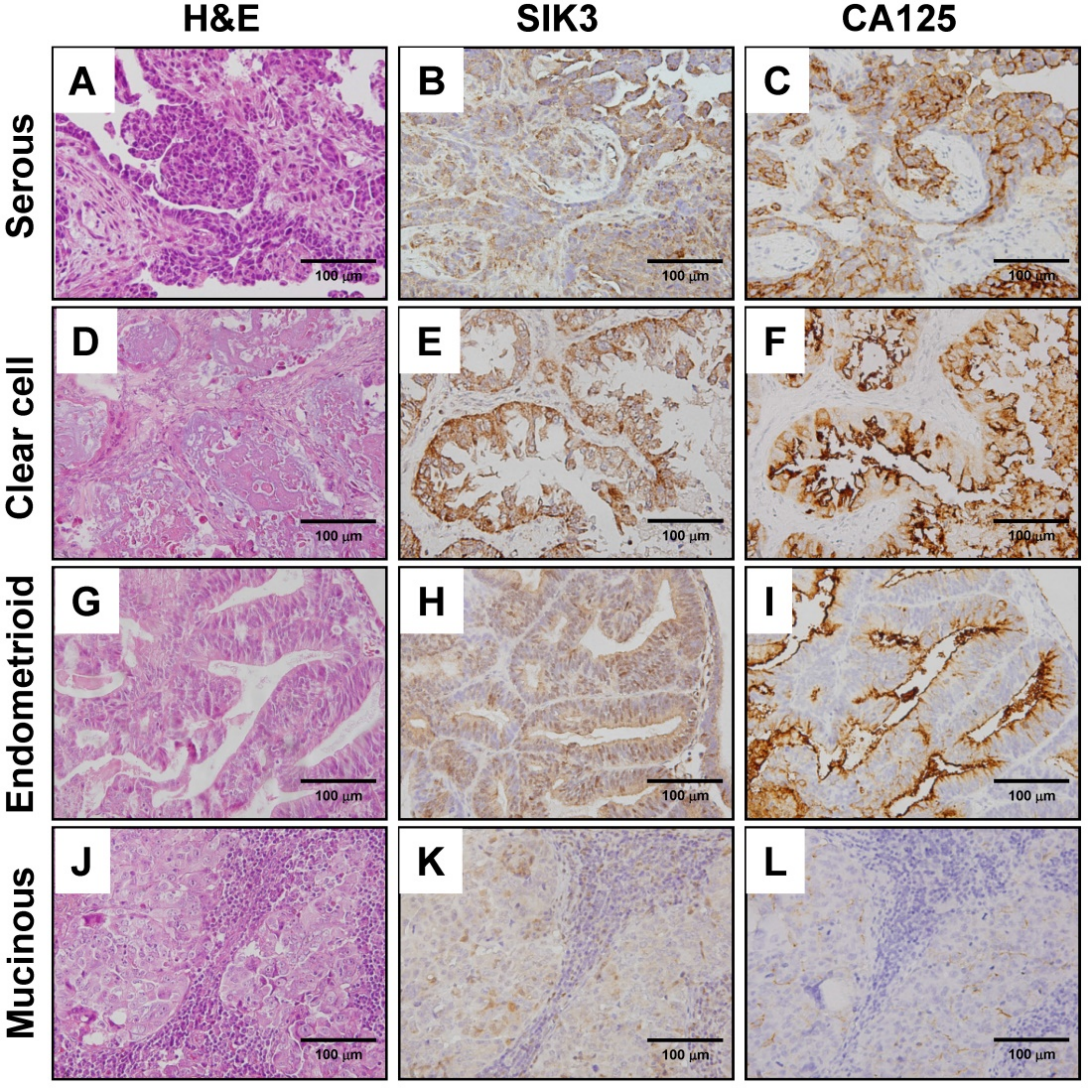
** Immunohistochemical staining of SIK3 and CA125 in different types of ovarian cancer.** H&E (A, D, G, J), SIK3 (B, E, H, K) and CA125 (C, F, I, L) Immunohistochemical staining in serous, clear, endometrioid and mucinous ovarian cancer, respectively. Note that SIK3 express mainly in tumor cell cytoplasm while CA125 in tumor cell membrane predominately.

The tumor sections of patients were used to detect SIK3 and CA125 expression by immunohistochemistry. The expression of SIK3 was predominantly localized to the cytoplasm of cancer cells (Figure 1B, 1E, 1H, 1K). In contrast to that of SIK3, the expression pattern of CA125, a membrane protein, was typically confined to the surfaces of tumor cells (Figure 1C, 1F, 1I, 1L). The cut-off points for high SIK3 and high CA125 expression based on time-dependent ROC curves were 47.5% and 14%, respectively. The best cut-off point for high presurgery serum CA125 level was 314.8 U/ml (Table 1). Regarding the sensitivity and specificity analyses of these markers for prognostic prediction, tissue expression of SIK3 showed better specificity than tissue and serum expression of CA125 (80.0%, 56%, and 50%, respectively) by IHC in ovarian cancer patients.

EOC is divided into four major types based on histological classification by cell type: serous (51.5%), endometrioid (14.2%), clear cell (20.6%) and mucinous (9.3%) (Table 1). In our cohort, we observed that most of the patients with high expression levels of SIK3 and CA125 had stage III disease (45.6% for SIK3 and 67.3% for CA125) and serous-type ovarian cancer (46.8% for SIK3 and 70.1% for CA125). Of the 125 patients with advanced-stage disease (3 stage III and 4 stage IV), 115 (92%) received chemotherapy after surgery, including 90 serous EOC patients, and 63 (50.4%) were chemosensitive. When chemosensitivity was analyzed based on the factors, only tumors with high expression of SIK3 were significantly associated with increased chemosensitivity (67.5% vs 32.5%, P=0.01, 95% CI, 1.00-5.48, HR=2.23). By Cox regression model analysis, clinicopathological parameters including age, stage, histologic types, residual tumor, CA125 expression, SIK3 expression, optimal debulking surgery, early-stage ovarian cancer, and high SIK3 expression were independently associated with better OS (Table 2).

### Low tissue expression levels of SIK3 are associated with poor prognoses in serous EOC patients

**Figure 2:**
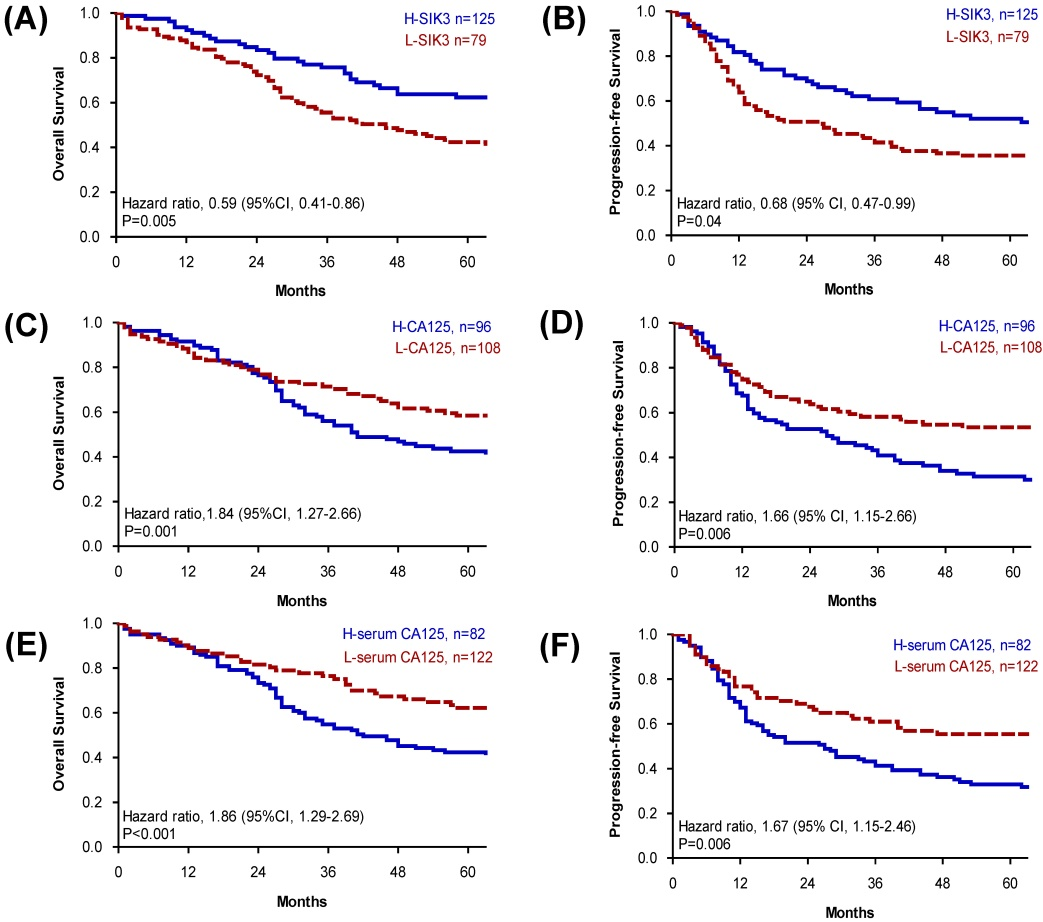
** Patients with low SIK3, high CA125 expression or high serum CA125 level have poor prognosis.** (A, B) Patients with lower level of SIK3 expression (<47.5%) were associated with a poor OS (median survival: 46 vs 127 months, *p* = 0.005) and PFS (median survival: 26 vs 66 months, *p* = 0.04). (C, D) Patients with higher CA125 expression (≥14%) had worse OS (median survival: 41 vs 135 months,* p* =0.001) and PFS (median survival: 27 vs 98 months,* p* =0.006). (E, F) Patients with a higher serum CA125 (≥314.8 u/ml) level had worse OS (median survival: 42 vs 135 months,* p* <0.001) and PFS (median survival: 27 vs 98 months, *p* =0.006) than patients with a lower serum CA125 level.

Based on the cut-off point, we divided all 204 EOC patients into groups based on high or low SIK3 expression, CA125 expression, and presurgical serum CA125 level. Patients with lower SIK3 expression (<47.5%) had poorer OS (median survival: 46 vs 127 months, *p* = 0.005) and PFS (median survival: 26 vs 66 months, *p* = 0.04) (Figure 2A, 2B). Patients with higher CA125 expression (≥14%) had worse OS (median survival: 41 vs 135 months,* p* =0.001) and PFS (median survival: 27 vs 98 months,* p* =0.006) (Figure 2C, 2D). Patients with a high serum CA125 (≥314.8 U/ml) level had worse OS (median survival: 42 vs 135 months,* p* <0.001) and PFS (median survival: 27 vs 98 months, *p* =0.006) than patients with a low serum CA125 level (Figure 2E, 2F). Since the serous type is the most frequently found histologic type in EOC, we further analyzed 105 serous EOC patients. We found that patients with low expression of SIK3 (<47.5%) had poorer OS (median survival: 34 vs 75 months, *p*=0.02) and PFS (median survival: 13 vs 44 months=0.006) than patients with low SIK3 expression (Supplementary Figure 1A, 1B). Patients with high expression CA125 (≥14%) had worse OS than patients with low CA125 expression (median survival: 32 vs 105 months, p<0.001) and PFS (median survival: 15 vs 36 months=0.03) (Supplementary Figure 1C, 1D). No significant survival differences were observed by presurgery serum CA125 levels (<314.8 U/ml or not) (Supplementary Figure 1E, 1F).

**Figure 3:**
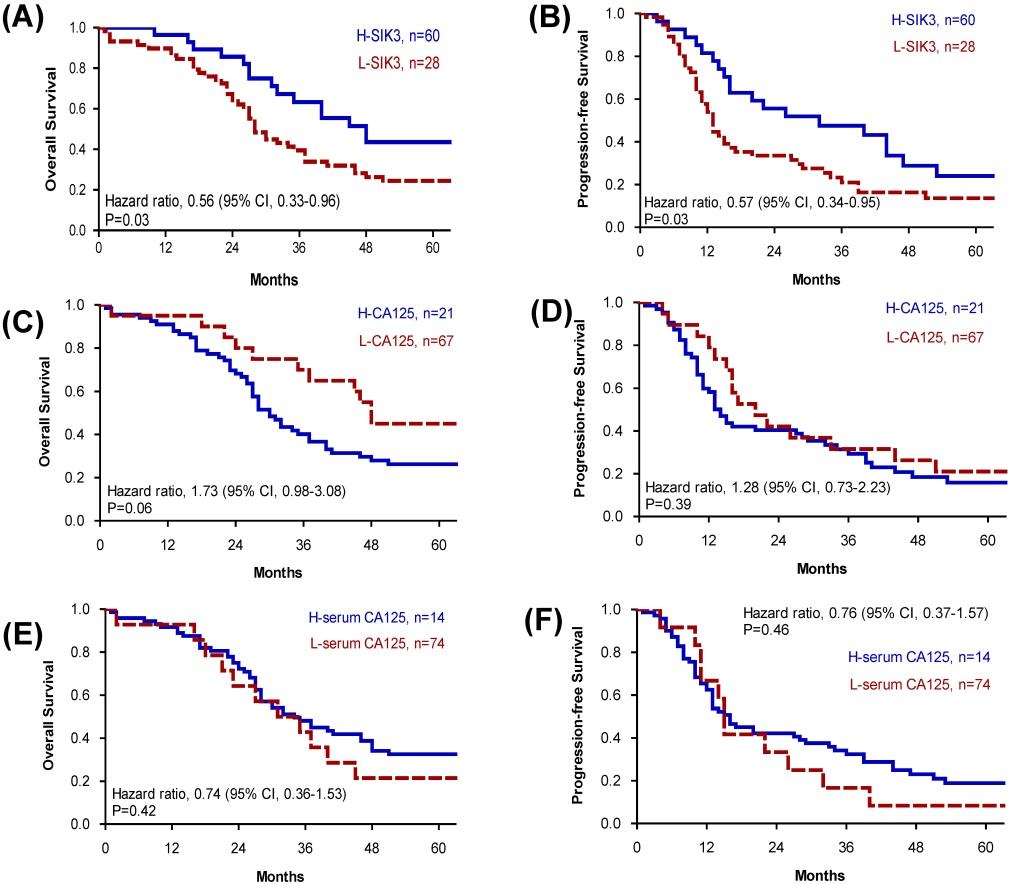
** Patients in advanced-stage serous ovarian cancer with low SIK3 expression have poor OS and PFS.** (A, B) In advanced-stage serous ovarian cancer (n=88), patients with low SIK3 expression (<47.5%) had worse OS (median survival: 28 vs 48 months, *p*=0.03) (A) and PFS (median survival 13 vs 32 months, *p*=0.03) (B) than patients with high SIK3 expression. CA125 expression (C, D) and serum CA125 level (E, F) were not significantly associated with patient survival.

Because advanced-stage serous EOC patients are the most difficult to clinically manage, we further analyzed tissue SIK3 and CA125 expression and serum CA125 levels for prognosis in patients with advanced serous EOC. We found that for advanced- stage serous EOC, patients with low SIK3 expression had worse OS (median survival: 28 vs 48 months, p=0.03) and PFS (median survival 13 vs 32 months, p=0.03) (Figure 3A, 3B). Levels of CA125 expression and serum CA125 were not significantly associated with patient survival (Figure 3).

### Inhibition of SIK3 expression in ovarian cancer cells increases chemoresistance to Taxol and cisplatin treatment

**Figure 4:**
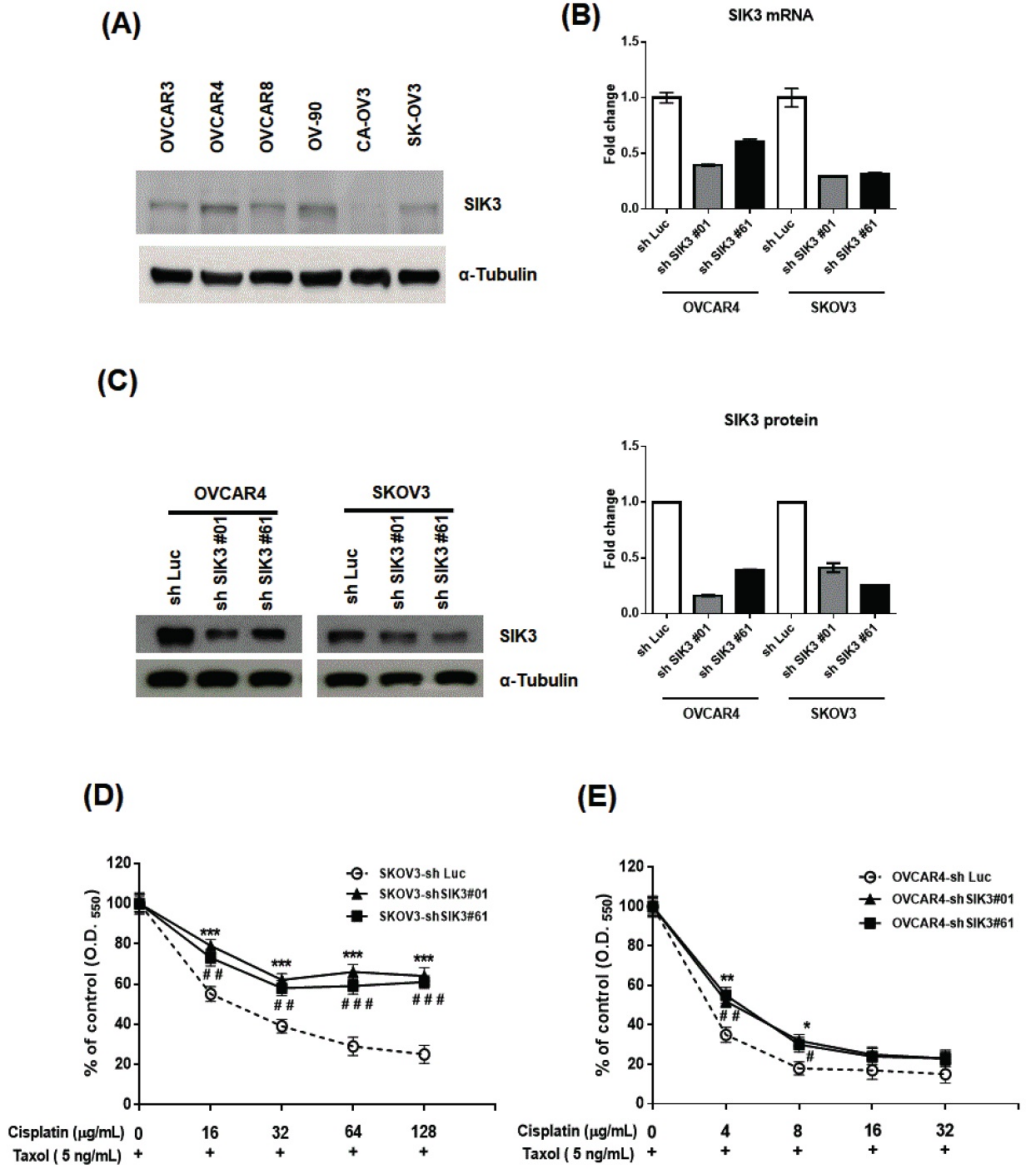
** Knockdown of SIK3 expression promotes chemoresistance to Taxol/cisplatin treatment in ovarian cancer cells. (**A) Endogenous SIK3 expression was detected in six serous ovarian cancer cell lines. Lentiviruses with siRNAs (shSIK3#01 and #061) efficiently knocked down SIK3 expression at mRNA (B) and protein levels (C) compared to letiviruses with control shLuc. SKOV3 (D) and OVCAR4 (E) with or without SIK3 knockdown were seeded and treated with Taxol (5 ng/ml) and cisplatin at the indicated concentrations. Cell viabilities were measured by MTT assays. Data are presented as the mean±SEM from three independent experiments and analyzed by *t* tests. *: p<0.05, **: p<0.01, ***: p<0.001 (shSIK3#01 compared to the shLuc control). **^#^**: p<0.05, **^# #^**: p<0.01, **^# # #^**: p<0.001 (shSIK3#61 compared to the shLuc control).

To confirm the clinical observation of chemosensitivity related to SIK3 expression, we selected SKOV3 and OVCAR4 cells from six serous ovarian cancer cell lines for further SIK3 knockdown experiments (Figure 4A). The two cell lines were infected with lentiviruses carrying specifically designed siRNAs (#01 or #61) to suppress SIK3 expression. The knockdown of SIK3 was examined by real-time RT-PCR and Western blotting, showing at least 50% downregulation at mRNA (Figure 4B) and protein levels (Figure 4C) of SIK3 in both cell lines.

We hypothesized that poor survival in serous EOC patients with low SIK3 expression is associated with chemoresistance to anticancer agents. Thus, the OVCAR4 and SKOV3 cells with/without SIK3 knockdown were treated with two first-line therapeutic agents, cisplatin/Taxol, and cell viabilities were measured by MTT assays. In SKOV3 cells, the IC_50_ of cisplatin was 21 ug/mL in SKOV3-sh Luc, while up to 128 ug/mL in SKOV3-shSIK3#01, -shSIK3#61(Figure 4D). In OVCAR4 cells, the IC_50_ of cisplatin was 3.1 ug/mL in OVCAR4-shLuc, and elevated to 4.1 and 4.6 ug/mL in OVCAR4-shSIK3 #01 and -shSIK3#61 respectively (Figure 4D). Therefore, ovarian cancer cells with SIK3 knockdown displayed significant chemoresistance to cisplatin and Taxol compared to control cells (Figure 4D, 4E). Animal experiments also showed the similar results (Supplementary Figure 2). The average volume and weight of tumors in NOD/SCID mice subcutaneously injected with SKOV3-shSIK3#01, shSIK3#61 cells were significantly higher after cisplatin treatment compared to those in mice injected with SKOV3-shLuc control cells.

### Suppression of SIK3 in ovarian cancer cells promotes the activation of ABCG2

**Figure 5:**
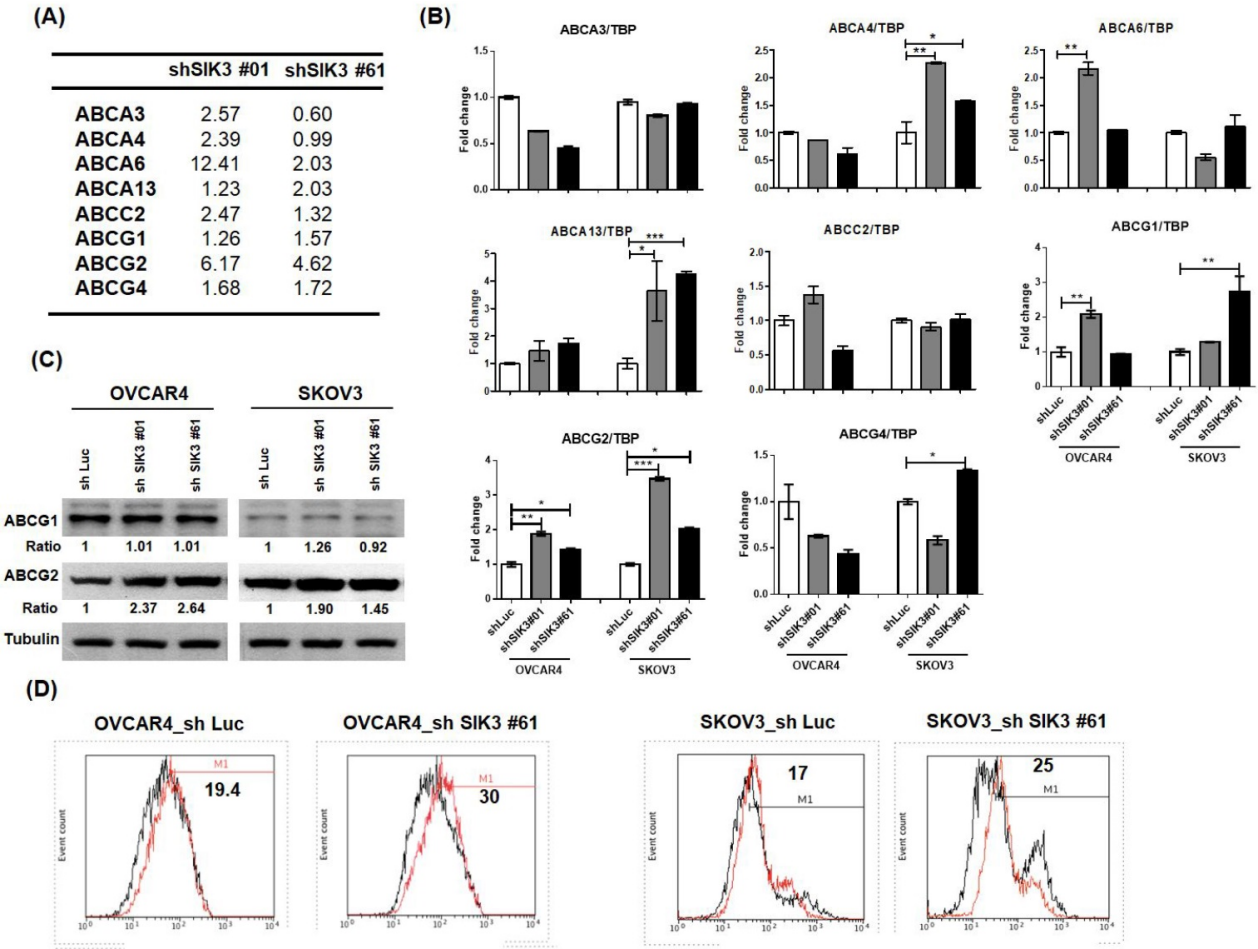
** ABCG2 upregulation is associated with SIK3 attenuation.** (A) List of ABC family proteins from microarray data for which the expression changed more than 1.5-fold compared with control cells. (B) The mRNA expression levels of ABC proteins were verified by real-time PCR in OVCAR4 and SKOV3 cells. The white bar (shLuc) represents control cells, and the gray bar (shSIK3#01) and black bar (shSIK3#61) represent indicated cells with SIK3 knockdown. Data are represented as the mean ± SEM from three independent experiments and analyzed by one-way ANOVA. *: *p*<0.05 and **: *p*<0.01 (compared to the shLuc control). Note that ABCG1 and ABCG2 were both significantly upregulated in cells with SIK3 knocked down by shSIK3#01 and shSIK3#61. (C) The protein expression levels of ABCG1 and ABCG2 were further verified by Western blotting. Note that only ABCG2 was upregulated in OVCAR4 and SKOV3 cells with SIK3 knocked down by shSIK3#01 and shSIK3#61. (D) The functional MDR activity of the ABCG2 protein was determined using an MDR assay kit (Abcam). A total of 2 x 10^5^ suspended cells were pretreated with the inhibitor novobiocin (50 nM) or DMSO. Then, diluted Efflux Gold Detection Reagent was added at 37°C for 30 minutes. The cellular orange fluorescence signal of the Efflux Gold Detection Reagent was measured immediately by flow cytometry in the living (PI-negative) cell population. The numbers in the upper left corners are MAF values.

To explore the mechanism of SIK3 suppression-induced chemoresistance in ovarian cancer, we performed microarray analysis in two SKOV3 cell lines with SIK3 knockdown (shSIK3#01 and #61, Supplementary Tables 2 and 3). Expression changes in the ABC family members by more than 1.5-fold compared with control cells are listed in Figure 5A. Three genes (ABCA6, ABCG2 and ABCG4) showed an increase of more than 1.5-fold in shSIK3#01- and #61-transfected SKOV3 cells. Based on real-time PCR, ABCG1 and ABCG2 mRNA levels were increased in both SKOV3 and OVCAR4 cells (Figure 5B), while only ABCG2 protein levels were elevated in both OVCAR4 and SKOV3 cells with SIK3 knockdown (Figure 5C). To determine ABCG2 activity, we treated cells with SIK3 knockdown with novobiocin (an ABCG2 inhibitor). The increased fluorescence intensity in shSIK3#61-transfected SKOV3 and OVCAR4 cells with MAF values of 30 and 25, respectively, suggested that the function of SIK3-induced ABCG2 was increased (Figure 5D).

### SIK3 expression is inversely correlated with ABCG2; serous ovarian cancer patients with low SIK3 and high ABCG2 expression have poor prognosis

**Figure 6:**
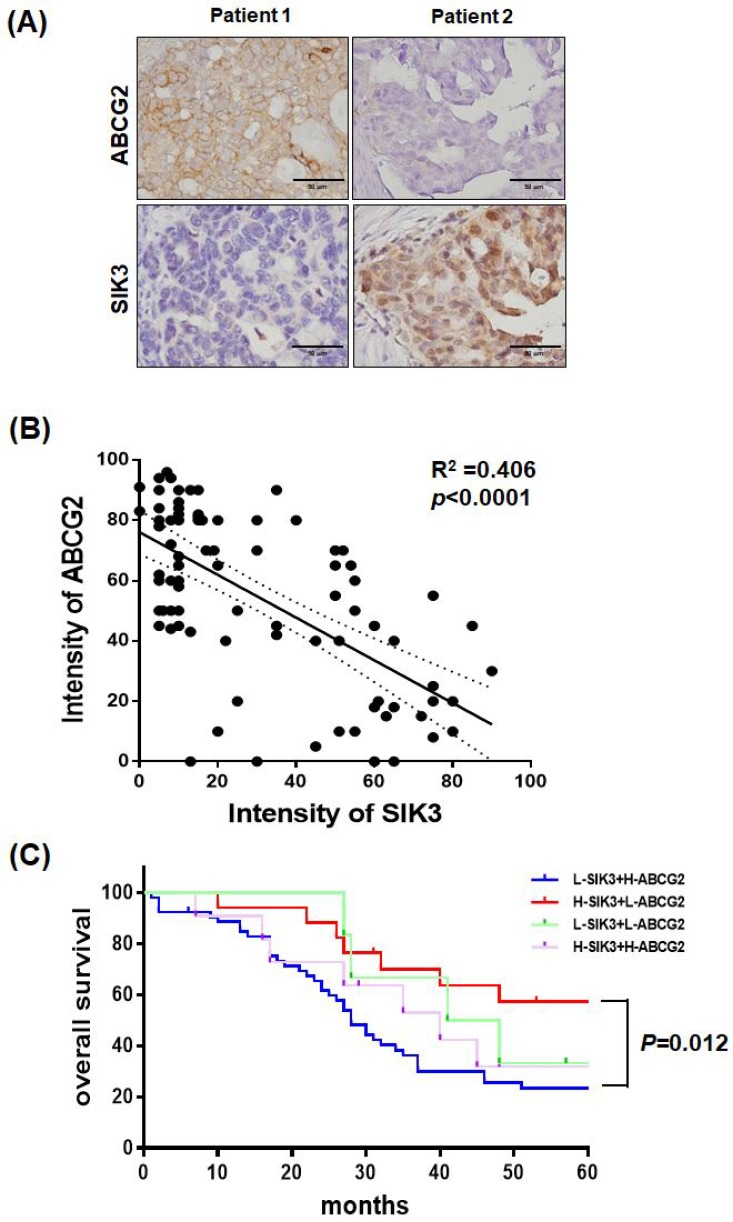
** Serous ovarian cancer patients with low SIK3 and high ABCG2 expression have poor prognosis.** (A, B) SIK3 expression is inversely correlated with ABCG2 in serous ovarian cancer. (A) Representative IHC staining for ABCG2 and SIK3 from the serial section of two serous ovarian cancer patients. (B) Regression analysis of SIK3 and ABCG2 expression in 88 advanced-stage serous ovarian cancer patients, r^2^=0.406 (*p*<0.0001). (C) In serous ovarian cancer, patients with low SIK3 (<47.5%) and high ABCG2 expression (≥41%) had worse OS than patients with high SIK3 and low ABCG2 expression. The 5-year survival rate was 57.35% vs 23.50% (*p*=0.012).

Serial sections of paraffin-embedded serous-type ovarian cancer lesions were used to detect ABCG2 and SIK3 protein levels by immunohistochemistry. In Figure 6A, tumors from two patients showed opposite expression levels of SIK3 and ABCG2. In 88 advanced-stage serous EOC samples, the percentage of cells positively stained for ABCG2 and SIK3 in the tumor region was scored and analyzed. Numerical measurements for the intensity of ABCG2 and SIK3 were performed using Pearson's correlation coefficients (Figure 6B). Linear regression was used to model the relationship between ABCG2 and SIK3. The value of r2 (coefficient of determination) was 0.406, which indicated a moderate relationship. The cut-off points for high SIK3 and high ABCG2 expression based on time-dependent ROC curves were 47.5% and 41%, respectively. Based on the cut-off points, we grouped patients into high or low SIK3 and ABCG2 expression groups. Patients with low SIK3 (<47.5%) and high ABCG2 expression (≥41%) had worse OS than patients with high SIK3 and low ABCG2 expression (p=0.012, log-rank test) (Figure 6C).

## Discussion

In this study, we demonstrated that serous EOC patients with low SIK3 expression had poor prognosis, which may be due to chemoresistance development mediated by ABCG2 activation.

The SIK family is a novel protein kinase family that comprises SIK1, SIK2 and SIK3. SIK1 is an important regulator of early-phase ACTH signaling in the adrenal cortex, and SIK2 is an important regulator of early-phase insulin signaling in adipose tissues 9. SIK2 has been reported as a centrosome kinase that initiates mitosis. High expression of SIK2 was significantly correlated with poor survival in patients with high-grade serous ovarian cancer 13, 14. SIK3 has also been shown to be an important mitotic regulator and a druggable antimitotic target 25. From our previous study, we identified SIK3 as a novel ovarian TAA that is highly and preferentially expressed in ovarian cancer but not in benign gynecologic diseases, i.e., adenomyosis or endometriosis, with elevated CA125 levels. Increased expression of SIK3 promotes G1/S phase cell cycle progression and induces cell proliferation via the activation of the c-Src-phosphoinositide 3-kinase (PI3K) linkage, with subsequent downregulation of p21 Waf/Cip1 and consequent increase in tumorigenesis in mice 16. However, in our clinical cohort analysis, we found that EOC patients with high SIK3 expression had better survival outcomes (Figure 2, Supplementary Figure 1, and Table 2), especially in advanced serous EOC (Figure 3). Among the factors including presurgery serum CA125, tissue CA125, and SIK3 expression, only high tissue SIK3 expression predicted a better prognosis in advanced EOC patients. Because almost all advanced EOC patients received adjuvant Taxol/platinum chemotherapy, chemosensitivity likely influenced the prognosis. Indeed, we found that stage III/IV ovarian cancer patients with high expression of SIK3 are more chemosensitive than patients with low expression of SIK3 (Table 1, P=0.01, Fisher's exact test). This observation may explain why advanced serous EOC patients with high SIK3 expression showed a better prognosis.

From our *in vitro* studies, we found that the ovarian cancer cell lines SKOV3 and OVCAR4 with SIK3 knockdown displayed significant chemoresistance to Taxol/cisplatin, which was specifically associated with the upregulation of ABCG2 (Figure 4 and Figure 5). The human genome contains 49 ABC genes, which are arranged into 7 different subfamilies from ABCA to ABCG. Many studies with drug-selected models have shown that the overexpression of ABC transporters, including ABCB1, ABCC1 and ABCG2, is the main mechanism related to the multidrug resistance phenotype 19-21, 26, 27. Moreover, ABCG2 is a universal marker of stem cells and promotes stem cell proliferation 21, 28, which increases chemoresistance.

Baber et al. reported that high salt treatment induced ABCB1 expression and paclitaxel resistance in MCF-7 cells and that knockdown of SIK3 downregulated ABCB1 expression. SIK3 may mediate ABCB1-associated drug resistance in breast cancer cells 29. However, the authors did not show a correlation between clinical outcomes and SIK3 expression in breast cancer patients. In our study, ovarian cancer cells with SIK3 knockdown and ABCG2 upregulation showed significant chemoresistance to Taxol/cisplatin treatment. Clinically, SIK3 expression is inversely correlated with ABCG2 (Figure 6B), and serous EOC patients with low SIK3 and high ABCG2 expression had significantly worse prognosis than patients with high SIK3 and low ABCG2 expression (Figure 6C). Since SIKs have been reported to have diverse oncogenic and tumor-suppressive roles in cancer 30, further study will be needed to answer these questions.

In conclusion, we demonstrate that EOC patients with low expression of SIK3 have poor prognostic outcomes, especially in advanced serous disease, which may be due to chemoresistance development mediated by the upregulation of ABCG2. This is the first report describing a clinical association between SIK3 expression and survival outcomes in EOC patients. Because SIK3 functions have been reported to be associated with glucose tolerance/energy metabolism 31,32, further studies may be needed to our current observation, identify the regulation of ABCG2 in EOC as well as glycolytic activity/metabolic change in SIK3-associated ovarian cancer.

## Supplementary Material

Supplementary figures and tables.

## Figures and Tables

**Table 1:** Clinicopathological characteristics of the patients

Characteristic	Total	Tissue SIK3 ≥ 47.5 % n ( % )	Tissue CA125 ≥ 14% n ( % )	Mean pre-surgery Serum CA125 (u/ml)	Pre-surgery serum CA125 ≥ 314(u/ml) n ( % )
**Number**	204	79( 38.7 )	107( 52.5)		110( 53.9 )
**Age (mean)**	52.8	54.6	55.2		54.7
**Stage**					
I	63 (30.9)	29 (36.7)	17 (15.9)	406.4	13 (11.8)
II	16 (7.8)	8 (10.1)	7 (6.5)	357.4	5 (4.5)
III	107 (52.4)	36 (45.6)	72 (67.3)	1339.2	77 (70.0)
IV	18 (8.9)	6 (7.6)	11 (10.3)	1833.1	15 (13.6)
**Cell type**					
Serous	105 (51.5)	37 (46.8)	75 (70.1)	1451.8	74 (67.3)
Clear cell	42 (20.6)	22 (27.8)	11 (10.3)	574.6	14 (12.7)
Endometrioid	29 (14.2)	14 (17.7)	14 (13.1)	585.1	13 (11.8)
Mucinous	19 (9.3)	4 (5.1)	2 (1.9)	497.0	5 (4.5)
Others^#^	9 (4.4)	2 (2.5)	5 (4.7)	568.6	4 (3.6)
**Chemotherapy Response in Stage III+IV**					
Number	115	40	77		84
Sensitive	63 (54.8)	27 (67.5)*	40 (51.9)	1223.4	47 (56.0)
Resistant	52 (45.2)	13 (32.5)	37 (48.1)	1792.5	37 (44.0)

#: 2 undifferentiated carcinoma, 2 mixed type of endometrioid and mucinous adenocarcinoma, 1 mixed type of endometrioid and clear cell adenocarcinoma, 1 mixed type of endometrioid and serous adenocarcinoma, 2 MMMT, 1 poorly-differentiated tumor; *: Comparison between chemosensitive and chemoresistant ovarian cancer patients, Fisher's exact test, p=0.01; Sensitive was defined as a recurrence after a platinum-free interval ≥ 6 months, Resistant was defined as a recurrence after a platinum-free interval < 6 months.

**Table 2:** Univariate and multivariate Cox proportional hazards regression model for overall survival (n= 204)

Factor	Univariate P	HR (95% CI)	Multivariate P	HR (95% CI)
Age	0.001	1.030 (1.013-1.047)	0.062	1.038 (0.998-1.079)
Stage (advanced vs. early )	<0.0001	4.242 (2.537-7.092)	0.004	3.513 (1.480-8.399)
Histology (serous vs. non-serous)	0.002	0.531 (0.352-0.802)	0.150	0.571 (0.267-1.225)
Residual tumor (suboptimal debulking vs. optimal)	<0.0001	3.391 (2.259-5.088)	0.006	3.349 (1.426-7.865)
CA125 staining ( H-CA125 vs.L-CA125)	0.001	1.838 (1.269-2.663)	0.416	1.482(0.574-3.830)
SIK3 staining (H-SIK3 vs. L-SIK3)	0.007	0.540 (0.410-0.860)	0.015	0.415 (0.204-0.843)
Pre-surgery serum CA125 (H-serum CA125 vs. L-serum CA125)	0.001	1.862 (1.288-2.691)	0.106	2.220 (0.844-5.835)

H-CA125: Tissue CA125 ≥ 14%, L-CA125:Tissue CA125 <14%; H-SIK3:Tissue SIK3 ≥ 47.5 %, L-SIK3:Tissue SIK3 < 47.5 %; H-serum CA125:Pre-surgery serum CA125 ≥ 314(u/ml), L-serum CA125:Pre-surgery serum CA125 ≥ 314(u/ml)
