# Supplementary material for: Downregulated Salt-inducible Kinase 3 Expression Promotes Chemoresistance in Serous Ovarian Cancer via the ATP‐binding Cassette Protein ABCG2
Source: J Cancer. 2019 Oct 15;10(24):6025–36. doi: 10.7150/jca.34886 (PMC6856590; doi:10.7150/jca.34886)
Supplement: Supplementary file 1 — Supplementary figures and tables. [file jcav10p6025s1.pdf]

Suppl Fig 1

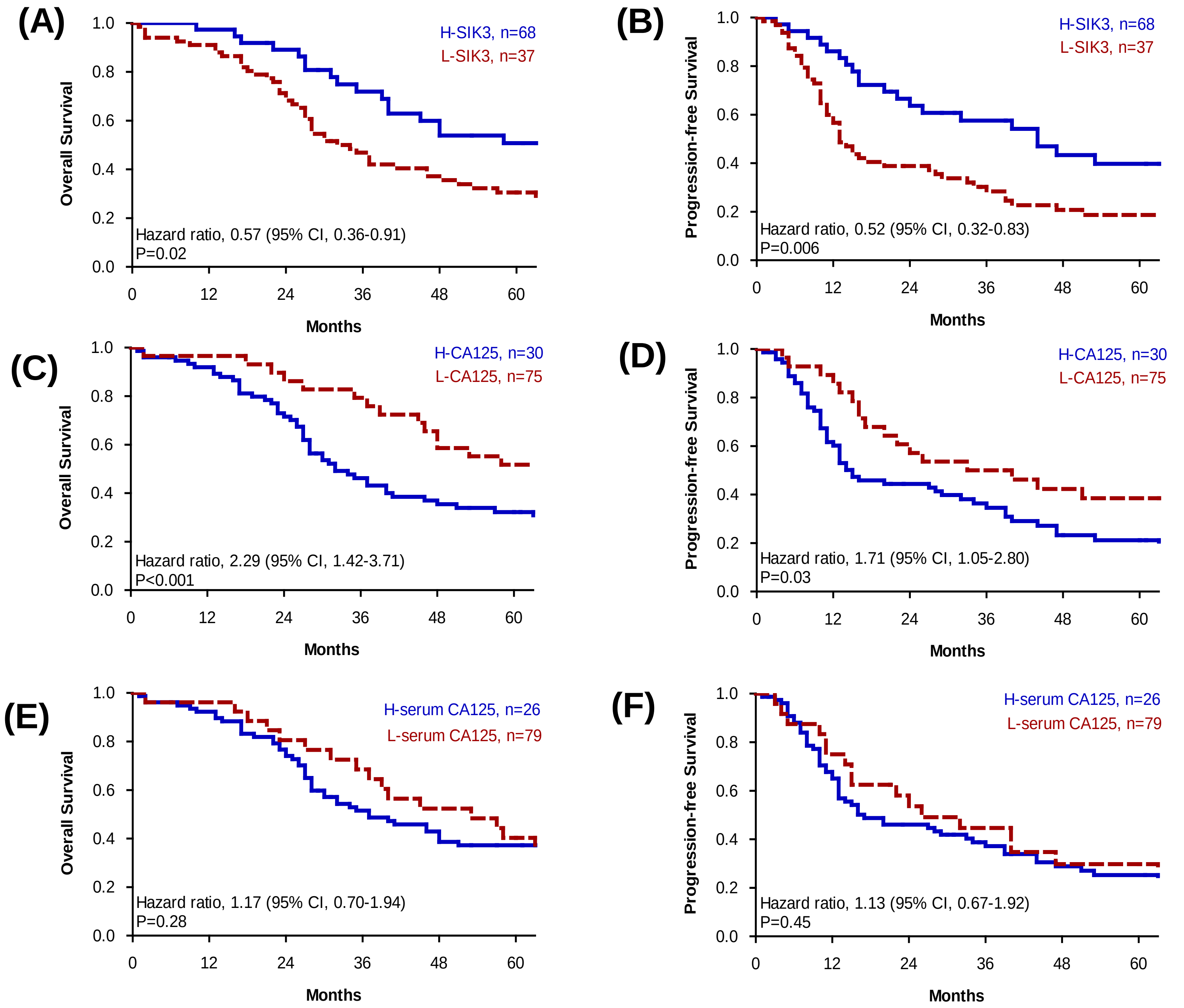

(A)
Overall Survival
H-SIK3, n=68
L-SIK3, n=37
Hazard ratio, 0.57 (95% CI, 0.36-0.91)
P=0.02
Months
(B)
Progression-free Survival
H-SIK3, n=68
L-SIK3, n=37
Hazard ratio, 0.52 (95% CI, 0.32-0.83)
P=0.006
Months
(C)
Overall Survival
H-CA125, n=30
L-CA125, n=75
Hazard ratio, 2.29 (95% CI, 1.42-3.71)
P<0.001
Months
(D)
Progression-free Survival
H-CA125, n=30
L-CA125, n=75
Hazard ratio, 1.71 (95% CI, 1.05-2.80)
P=0.03
Months
(E)
Overall Survival
H-serum CA125, n=26
L-serum CA125, n=79
Hazard ratio, 1.17 (95% CI, 0.70-1.94)
P=0.28
Months
(F)
Progression-free Survival
H-serum CA125, n=26
L-serum CA125, n=79
Hazard ratio, 1.13 (95% CI, 0.67-1.92)
P=0.45
Months

Suppl Fig 2

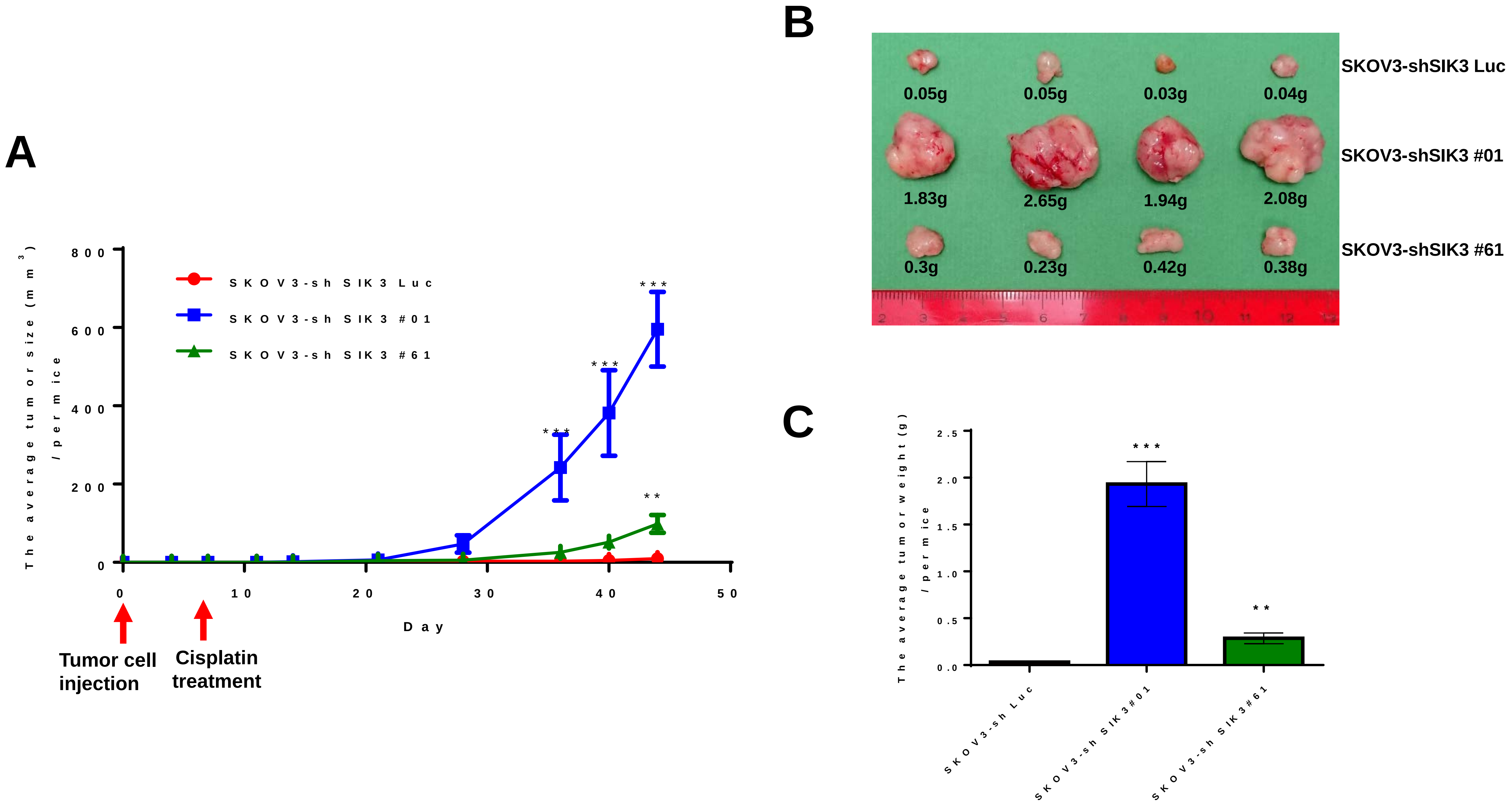
A
B
C
SKOV3-shSIK3 Luc
SKOV3-shSIK3 #01
SKOV3-shSIK3 #61
0.05g
0.05g
0.03g
0.04g
1.83g
2.65g
1.94g
2.08g
0.3g
0.23g
0.42g
0.38g
The average tumor size (mm3) / per mice
SKOV3-sh SIK3 Luc
SKOV3-sh SIK3 #01
SKOV3-sh SIK3 #61
800
600
400
200
0
0
10
20
30
40
50
Day
***
***
***
**
Tumor cell injection
Cisplatin treatment
The average tumor weight (g) / per mice
2.5
2.0
1.5
1.0
0.5
0.0
***
**
SKOV3-sh Luc
SKOV3-sh SIK3#01
SKOV3-sh SIK3#61

Supplementary Table 1

	forward sequence (5' – 3')	reverse sequence (5' – 3')
**ABCA3**	CATTAGGTACGACAACTGCTCG	TTTCTCCGTGTGTAACTGAACC
**ABCA4**	ACCCGGAGAGAATTGCAGGA	AGACAGGCCGATGTTTTTAATGA
**ABCA6**	TGTCATTGGGGCACCAAATAAA	AAGTGGACTGTTATATCCCTGGA
**ABCA13**	CAGCTATGAAGGGTCAATGGAG	TGGATCGTTCTACCCAAAGTCT
**ABCC1**	TTACTCATTCAGCTCGTCTTGTC	CAGGGATTAGGGTCGTGGAT
**ABCC2**	TCTCTCGATACTCTGTGGCAC	CTGGAATCCGTAGGAGATGAAGA
**ABCG1**	ATTCAGGGACCTTTCCTATTCGG	CTCACCACTATTGAACTTCCCG
**ABCG2**	TGAGCCTACAACTGGCTTAGA	CCCTGCTTAGACATCCTTTTCAG
**ABCG4**	CCTCTCAGGTAAATTCTGCCG	CTCCCTGTATCCTGCCAAGAT
**SIK3**	AGTTGTCCTCTACGTGCTTGT	ATGGCGGATCAAATGCTCACA
**TBP**	TTCGGAGAGTTCTGGGATTGTA	TGGACTGTTCTTCACTCTTGGC

Supplementary Table 2 SKOV3 shSIK3 01 vs SKOV3-shLuc.result

Gene	PPEE	PPDE	PostFC	RealFC	C1Mean	C2Mean
ABCA12	0	1	0.067594	0.065508	16.67114	254.6316
ADAMTS1	0	1	10.57688	10.76628	310.0833	28.79227
CA9	0	1	9.974287	10.15749	283.4094	27.89251
CCL22	0	1	15.24485	15.67672	294.5235	18.77796
CFH	0	1	12.30891	12.43673	626.7572	50.38647
COL11A1	0	1	14.02238	14.30655	373.4336	26.09299
COL13A1	0	1	8.177631	8.264995	386.7705	46.78743
COL5A1	0	1	7.072475	7.090607	1352.585	190.7488
DOK5	0	1	0.059488	0.058213	24.45101	420.1871
FLRT1	0	1	0.056784	0.055012	16.67114	303.2185
FN1	0	1	25.66138	25.69432	10958.5	426.4854
IGFBP5	0	1	6.908424	6.914418	3882.154	561.4492
ITGA2	0	1	7.813607	7.851112	812.4404	103.4722
KRTAP2-3	0	1	14.64838	15.22398	205.6108	13.49637
LONRF2	0	1	0.00741	0.004926	1.11141	227.6389
MCC	0	1	16.58812	17.01697	352.3168	20.69444
MMP7	0	1	17.06444	17.12644	2527.345	147.5604
MT2A	0	1	5.944297	5.945711	11844.29	1992.065
NCALD	0	1	11.13474	11.26303	506.8028	44.98792
PI3	0	1	7.362594	7.410539	560.1504	75.5797
PRODH	0	1	0.084649	0.082882	24.45101	295.1207
RHOU	0	1	0.051324	0.050531	34.4537	682.0168
SERPINE1	0	1	12.74917	12.75386	18211.56	1427.916
SLC30A2	0	1	0.025342	0.021734	3.334229	153.8587
SLCO2A1	0	1	7.534782	7.589207	519.0283	68.38163
TXNIP	0	1	8.379228	8.382276	11561.99	1379.329
C8orf4	0	1	11.531	11.5604	2402.868	207.8442
CPED1	0	1	33.48283	36.47123	230.0618	6.298308
HEPHL1	0	1	91.57278	93.58653	2442.878	26.09299
KRT16	0	1	9.209733	9.304275	468.8926	50.38647
NPPB	0	1	21.40846	21.54415	1880.505	87.27656
PMEPA1	0	1	7.01268	7.020643	3076.382	438.1823
TGFB2	0	1	7.182829	7.193756	2362.123	328.3488
TGFBI	0	1	15.25124	15.26661	8214.428	538.0555
THBS1	0	1	5.946059	5.946737	25180.1	4234.263
ALDH1A3	0	1	23.14599	23.39749	1158.089	49.48671
BIRC3	0	1	12.63247	12.64817	5269.193	416.5881
LAMA1	0	1	0.106199	0.10581	136.7034	1292.053
LOXL2	0	1	9.859064	9.887738	1717.128	173.6534
MMP2	0	1	10.89247	10.99376	603.4954	54.88526
SDCBP2	0	1	19.67009	21.19056	146.2504	6.892149
KRT6A	1.11E-16	1	7.45803	7.516451	473.2715	62.95609
CD44	2.22E-16	1	5.89515	5.899896	3466.486	587.5422
COL8A1	2.22E-16	1	5.805157	5.810304	3089.719	531.7572
LAMC2	2.22E-16	1	5.692184	5.693037	17851.46	3135.658
BTBD11	2.22E-16	1	0.08121	0.078982	18.89396	239.3357
TGM2	3.33E-16	1	5.687808	5.688969	13329.14	2342.971
DYX1C1-C	3.33E-16	1	0.085415	0.083334	20.57219	246.9747
ITGB3	4.44E-16	1	5.660301	5.661827	9714.831	1715.839
NT5E	6.66E-16	1	5.571408	5.57216	19307.41	3464.969
NOD2	7.77E-16	1	7.290173	7.352383	423.4471	57.58453
NLRP1	8.88E-16	1	8.830641	8.980812	266.7383	29.69202
PDCD1LG	1.11E-15	1	7.666269	7.749	355.6511	45.88767
IRAK3	1.11E-15	1	8.171229	8.284017	305.671	36.89009
RAB4B-EC	1.33E-15	1	128.803	7408.545	74.07545	0

KCNJ5	1.78E-15	1	0.150601	0.150143	158.487	1055.632
CXCL12	2.22E-15	1	0.109481	0.107893	34.4537	319.4142
VCAN	2.55E-15	1	5.424549	5.426945	5708.2	1051.817
SLCO2B1	5.55E-15	1	37.2629	48.52752	87.80136	1.799517
DSCAML1	6.33E-15	1	7.181773	7.252913	358.9853	49.48671
AFAP1L1	7.33E-15	1	5.468276	5.475839	1842.717	336.5096
ZBED2	1.02E-14	1	5.965083	5.98858	711.3021	118.7681
SPP1	1.04E-14	1	5.332831	5.337717	2660.715	498.4661
ADD2	1.09E-14	1	14.92413	15.90236	128.9235	8.097825
ST6GAL1	1.55E-14	1	6.246542	6.281782	525.6967	83.67752
ATP13A4	1.72E-14	1	0.007267	0.000126	0	79.17873
ATP2B2	3.59E-14	1	0.15231	0.151521	94.46981	623.5325
IGFL1	3.84E-14	1	5.409694	5.421374	1165.869	215.0422
TGFBR1	4.77E-14	1	5.192586	5.19901	1969.418	378.7982
ERBB4	4.85E-14	1	0.148254	0.147318	77.79867	528.1581
IQGAP2	5.5E-14	1	0.144572	0.14354	67.79598	472.3731
HBEGF	5.75E-14	1	5.222237	5.229962	1628.215	311.3164
MT1L	5.92E-14	1	5.241976	5.250553	1507.071	287.0229
RELN	7.36E-14	1	0.121432	0.119694	34.4537	287.9227
ANTXR2	8.5E-14	1	14.01144	14.94281	121.1436	8.097825
CCL26	1.02E-13	1	8.908471	9.126043	188.9396	20.69444
PDK4	1.07E-13	1	5.942058	5.976331	500.1343	83.67752
DEFB4A	1.13E-13	1	26.45696	31.73862	85.97865	2.699275
SRPX2	1.52E-13	1	6.633819	6.7011	325.643	48.58695
MAMLD1	1.85E-13	1	7.655775	7.783415	231.1732	29.69202
C15orf48	2.05E-13	1	8.322665	8.500886	198.9423	23.39372
DAPP1	2.05E-13	1	5.181563	5.192456	1135.327	218.6413
ABLIM3	2.28E-13	1	6.317849	6.371277	361.2081	56.68477
PGM2L1	2.43E-13	1	4.994156	5.000299	1851.808	370.3315
RGPD6	2.95E-13	1	103.9025	6075.965	60.74965	0
PPP2R2C	3.05E-13	1	9.574971	9.876369	160.043	16.19565
TNF	3.16E-13	1	5.686375	5.715458	524.5853	91.77535
CA8	3.33E-13	1	6.302408	6.357427	348.9826	54.88526
PAPSS2	3.73E-13	1	4.861818	4.865704	2806.309	576.7451
RAB3B	4.51E-13	1	4.977406	4.985124	1463.449	293.5551
CCL2	5.14E-13	1	5.140756	5.153669	941.3639	182.6509
GAS6	6.84E-13	1	4.773603	4.77684	3171.963	664.0216
FOXA1	1.13E-12	1	5.58654	5.617758	470.1263	83.67752
ARRDC4	1.25E-12	1	5.084144	5.098507	825.7773	161.9565
RBP4	1.56E-12	1	9.61231	9.952833	143.3718	14.39613
PRUNE2	1.62E-12	1	4.777944	4.784373	1631.549	341.0084
ULBP2	1.75E-12	1	5.304753	5.328115	569.7641	106.9273
COL17A1	2.68E-12	1	6.365433	6.437686	272.2953	42.28864
MUC3A	3.28E-12	1	5.146835	5.166873	609.0525	117.8683
EDN1	3.29E-12	1	4.548026	4.549597	5853.794	1286.654
SAA2-SAA	4.35E-12	1	5.887165	5.938387	322.72	54.3364
B3GNT5	4.5E-12	1	5.370898	5.400331	456.7893	84.57728
ARNT2	4.75E-12	1	0.179323	0.178557	108.9181	610.0361
TMTC1	6.79E-12	1	0.198575	0.198233	268.9611	1356.836
MAP1LC3	7.2E-12	1	0.177119	0.176243	93.0361	527.9332
TACR1	7.34E-12	1	9.737065	10.13929	127.8121	12.59662
PRPH	7.78E-12	1	0.13046	0.128176	27.78524	216.8418
RGCC	9.5E-12	1	5.605735	5.648607	345.6484	61.18356
TGFA	1.37E-11	1	4.466788	4.471216	2031.657	454.3779
UGT1A6	1.7E-11	1	4.755817	4.769659	751.0573	157.4577
NID1	1.84E-11	1	4.566966	4.575117	1140.306	249.2331

AZGP1	1.92E-11	1	8.647476	8.932076	136.7034	15.29589
CD40	2.67E-11	1	4.361047	4.364424	2473.998	566.8477
BMP7	2.69E-11	1	0.215076	0.214964	875.8574	4074.466
F3	2.8E-11	1	4.302095	4.302763	12109.92	2814.444
RNF180	2.93E-11	1	0.087432	0.083039	10.00269	120.5676
CACNB4	3.1E-11	1	10.4513	10.99469	108.9181	9.897341
RFTN1	3.57E-11	1	4.803826	4.821662	585.7128	121.4674
MOXD1	4.04E-11	1	4.840909	4.860887	542.3679	111.57
GPR1	5.71E-11	1	0.148054	0.145931	33.34229	228.5386
AOX1	6.09E-11	1	15.65251	17.5036	78.91008	4.498792
SLAMF7	6.37E-11	1	8.112847	8.367574	135.592	16.19565
JPH3	6.55E-11	1	5.90779	5.978385	236.7302	39.58937
FILIP1L	8.15E-11	1	4.579478	4.593046	690.1853	150.2596
RND1	8.42E-11	1	5.901641	5.973787	231.1732	38.68961
IL4I1	9.8E-11	1	4.796928	4.81853	482.4073	100.1071
WNK4	9.95E-11	1	7.502329	7.701943	145.5947	18.89492
STOX1	1.03E-10	1	0.166434	0.164727	46.6792	283.4239
MUC15	1.27E-10	1	0.049631	0.042716	3.334229	78.27897
NFE2L3	1.41E-10	1	4.121801	4.123133	5609.284	1360.435
IFFO2	1.45E-10	1	4.164831	4.167904	2445.101	586.6424
TMEM156	1.93E-10	1	5.919745	5.999582	210.5788	35.09057
NPTXR	2.08E-10	1	4.115228	4.11796	2675.163	649.6255
FABP3	2.08E-10	1	0.206582	0.205884	133.3691	647.826
ATP10A	2.18E-10	1	4.54522	4.56125	574.5988	125.9662
TMEM255	2.21E-10	1	12.34745	13.39141	84.46713	6.298308
TM4SF1	2.32E-10	1	4.096729	4.099175	2917.45	711.7088
ADORA2A	2.49E-10	1	7.18219	7.36552	144.1387	19.56075
ATP6V1G	2.74E-10	1	78.09214	4552.222	45.51222	0
PIK3IP1	2.75E-10	1	4.741898	4.765823	424.5585	89.07607
SYTL4	3.83E-10	1	5.709136	5.783201	213.3906	36.89009
PROM1	4.1E-10	1	4.00359	4.004373	8910.171	2225.102
VNN1	4.18E-10	1	14.55826	16.27111	73.35303	4.498792
ANGPTL4	5.39E-10	1	4.579407	4.600586	451.2323	98.07366
TLL2	5.52E-10	1	4.563757	4.584463	457.9007	99.87317
EYA2	5.53E-10	1	0.212342	0.211612	130.0349	614.5349
SGCG	5.55E-10	1	0.152626	0.150119	28.89665	192.5483
TMEM45A	5.63E-10	1	4.231001	4.240605	828.0001	195.2476
ADGRF4	5.83E-10	1	10.44131	11.1046	90.02418	8.097825
VAV3	6.32E-10	1	0.120674	0.116913	15.55973	133.1642
COL6A1	6.64E-10	1	4.064459	4.069472	1417.047	348.2065
FRMD3	6.68E-10	1	5.738266	5.81718	198.9423	34.19082
IL1B	7.64E-10	1	5.757936	5.840856	194.4967	33.29106
TNFSF10	8.22E-10	1	5.017942	5.059736	282.298	55.78501
PRKCQ	9.07E-10	1	0.129455	0.125945	17.78255	141.2621
TRIM55	1.01E-09	1	5.803763	5.892264	185.6054	31.49154
CTSB	1.04E-09	1	3.942716	3.942893	37251.75	9447.813
ROBO1	1.16E-09	1	0.064589	0.057574	4.445638	77.37921
RASGRF2	1.19E-09	1	4.101081	4.108997	916.9129	223.1401
ZNF425	1.53E-09	1	0.228005	0.227482	191.1624	840.3743
FUT2	1.65E-09	1	0.172479	0.170361	37.88795	222.4472
FBN1	1.81E-09	1	5.986729	6.091921	164.4886	26.99275
TRPV6	1.86E-09	1	0.187399	0.185718	51.12484	275.326
UPP1	2.05E-09	1	0.244838	0.244804	3178.631	12984.41
RUNDC3A	2.25E-09	1	5.562567	5.640608	187.8282	33.29106
NICN1	2.52E-09	1	0.194188	0.192638	57.03754	296.1285
GLDC	2.6E-09	1	0.244969	0.244791	602.384	2460.839

NLRP2	2.76E-09	1	4.888214	4.929926	261.7481	53.08574
SEC11C	2.88E-09	1	0.21476	0.21376	95.58122	447.1799
ADAMTS1	3.39E-09	1	0.244808	0.244568	437.8954	1790.519
GALNT14	3.39E-09	1	4.138519	4.151094	590.1585	142.1618
PABPC1L	3.52E-09	1	0.231454	0.230852	167.8228	727.0047
FAT3	4.21E-09	1	0.189614	0.187801	47.80173	254.5776
NKD1	4.38E-09	1	6.192324	6.322018	142.2604	22.49396
SHOX2	4.41E-09	1	4.796473	4.837278	261.1813	53.9855
FGF1	4.43E-09	1	4.815521	4.856454	257.847	53.08574
ADAM12	4.49E-09	1	9.662095	10.28213	83.35572	8.097825
C4orf19	5.51E-09	1	3.927514	3.935315	857.0524	217.7775
EHF	5.88E-09	1	3.778127	3.781503	1772.698	468.7741
MFAP3L	6.19E-09	1	0.217812	0.216726	88.91277	410.2898
RNF39	6.32E-09	1	4.162639	4.17828	481.2403	115.1691
THBS3	6.61E-09	1	0.231197	0.230438	135.592	588.4419
LTB	6.82E-09	1	4.939578	4.989447	224.5047	44.98792
BHLHE41	7.77E-09	1	4.498574	4.528658	305.6376	67.48187
KCNK3	9.04E-09	1	4.1554	4.172502	446.7867	107.0712
CREB5	9.81E-09	1	3.721234	3.724603	1746.024	468.7741
TNXB	1E-08	1	18.72912	23.21172	53.26986	2.285386
CD274	1.01E-08	1	3.716217	3.719413	1800.484	484.07
SCGB2A1	1.03E-08	1	0.047447	0.038172	2.222819	58.48429
SESN3	1.06E-08	1	0.229404	0.228511	112.2524	491.268
TGFB1	1.07E-08	1	3.761798	3.766692	1233.665	327.512
PSMG3-A	1.07E-08	1	0.071159	0.063479	4.445638	70.18115
FGF21	1.4E-08	1	0.194273	0.192289	44.45638	231.2379
SPON1	1.47E-08	1	3.626846	3.627447	9017.977	2486.032
SAA4	1.61E-08	1	65.7298	3704.217	37.03217	0
DMBT1	1.68E-08	1	7.226344	7.488928	101.1383	13.49637
ENOSF1	1.69E-08	1	0.233372	0.232476	113.3638	487.669
STXBP6	1.74E-08	1	0.090998	0.084336	6.668457	79.17873
LGALS9	1.83E-08	1	7.963973	8.331021	90.02418	10.7971
SEMA3C	1.86E-08	1	3.835206	3.844126	709.0793	184.4505
LPCAT1	1.87E-08	1	3.611231	3.612955	3075.27	851.1714
C5orf46	1.99E-08	1	64.2783	3668.652	36.67652	0
RSPH1	2.07E-08	1	0.155995	0.15246	21.11678	138.5628
STARD8	2.08E-08	1	5.296538	5.375459	164.4886	30.59178
SAA2	2.26E-08	1	3.770222	3.77775	806.7611	213.5486
RORC	2.39E-08	1	0.248242	0.24766	182.2712	736.0023
FHOD3	2.4E-08	1	4.612618	4.65345	234.5074	50.38647
SCN5A	2.45E-08	1	6.757264	6.97152	106.6953	15.29589
MT1A	2.68E-08	1	4.719187	4.767155	214.502	44.98792
EFNB1	3.03E-08	1	3.798755	3.807935	661.2887	173.6534
CASC2	3.14E-08	1	0.120736	0.115534	11.1141	96.27414
WRB-SH3	3.56E-08	1	8.015364	8.41186	84.76721	10.0683
KU-MEL-3	3.59E-08	1	0.014113	0.000247	0	40.48912
TMEM256	3.62E-08	1	0.014346	0.000246	0	40.5611
DYNC2H1	3.83E-08	1	0.261497	0.261149	321.1974	1229.97
MPZL2	3.96E-08	1	3.522086	3.523237	4400.071	1248.865
NPIPA7	3.96E-08	1	26.0214	38.78429	43.30052	1.106703
DNER	4.02E-08	1	0.132688	0.127866	13.33691	104.372
IL11	4.33E-08	1	3.814765	3.825651	563.4069	147.2634
BTN3A2	4.57E-08	1	3.616679	3.621629	1090.448	301.0861
VPREB3	4.87E-08	1	0.138442	0.133897	14.44832	107.971
BIRC7	4.88E-08	1	0.138527	0.133897	14.44832	107.971
B3GNT7	5.04E-08	1	4.356557	4.389242	256.7356	58.48429

SIK3	5.05E-08	1	0.268804	0.268601	562.3732	2093.738
YPEL4	5.24E-08	1	11.93205	13.31313	60.01612	4.498792
CSF2	5.27E-08	1	19.86396	25.80203	46.6792	1.799517
NELL2	5.4E-08	1	0.25559	0.254996	185.6054	727.9045
GLIPR1	5.42E-08	1	3.54021	3.543363	1625.992	458.8767
RASD2	5.48E-08	1	4.069219	4.090504	342.3142	83.67752
SPHK1	5.55E-08	1	3.520581	3.523171	1952.747	554.2511
ARHGEF6	5.7E-08	1	6.257939	6.433027	110.0295	17.09541
BEX2	6.35E-08	1	0.27259	0.272456	860.1866	3157.18
GBP5	6.78E-08	1	3.594377	3.599881	966.6151	268.5059
ESRRG	7.04E-08	1	0.145073	0.140673	15.55973	110.6703
ADAMTS9	7.18E-08	1	3.955753	3.973404	378.9907	95.37438
INHBE	7.41E-08	1	0.273832	0.273698	844.6713	3086.171
DYSF	7.42E-08	1	3.497684	3.500508	1763.807	503.8647
ELOVL4	7.68E-08	1	0.205466	0.203303	43.34497	213.2427
PTPN22	8.47E-08	1	19.45501	25.18783	45.56779	1.799517
IGFBP7	8.5E-08	1	3.444097	3.444687	8287.781	2405.954
MLLT3	9.08E-08	1	3.839019	3.853668	433.4497	112.4698
UBD	9.34E-08	1	9.077061	9.715338	70.0188	7.198066
CPVL	9.34E-08	1	0.276195	0.276149	2476.221	8966.991
IGFBPL1	9.39E-08	1	15.42997	18.46377	50.01343	2.699275
PIK3AP1	9.66E-08	1	3.519521	3.523867	1163.646	330.2113
RAB42	9.71E-08	1	4.015495	4.036699	326.9878	80.99624
VTCN1	1.04E-07	1	0.27751	0.277403	1084.736	3910.35
TBXAS1	1.05E-07	1	8.510798	9.048424	73.35303	8.097825
CCAT1	1.11E-07	1	4.360474	4.398494	225.6161	51.28622
SLIT1	1.19E-07	1	0.114858	0.108701	8.891277	81.87801
NID2	1.3E-07	1	3.398398	3.399328	4994.675	1469.305
ADGRB1	1.35E-07	1	8.934163	9.561149	68.90739	7.198066
CTSS	1.37E-07	1	3.620489	3.629201	633.5146	174.5531
SERPINE2	1.38E-07	1	3.472371	3.476405	1213.659	349.1062
C6orf223	1.46E-07	1	0.278337	0.278135	566.8189	2037.953
GPR176	1.54E-07	1	3.466981	3.471294	1152.532	332.0108
CLGN	1.57E-07	1	0.278435	0.278214	517.9169	1861.6
MAP3K7C	1.67E-07	1	5.355608	5.461616	127.8121	23.39372
STON2	1.7E-07	1	0.277518	0.277236	412.333	1487.327
LCN2	1.71E-07	1	0.278531	0.278517	8400.034	30159.9
PROCR	1.75E-07	1	3.49281	3.498317	919.1357	262.7294
KCP	1.87E-07	1	0.17024	0.166515	21.11678	126.8659
PBX1	2.01E-07	1	0.274045	0.273634	272.2953	995.1327
COL4A1	2.08E-07	1	3.380388	3.380594	22259.31	6584.431
GBP3	2.1E-07	1	3.394949	3.397978	1559.308	458.8857
COL4A4	2.31E-07	1	3.49818	3.50473	775.7639	221.3405
TRIM15	2.31E-07	1	18.46251	23.95942	43.34497	1.799517
NLRP3	2.33E-07	1	6.346893	6.558305	94.46981	14.39613
HLA-F-AS	2.37E-07	1	6.515723	6.748338	91.13559	13.49637
GJB3	2.4E-07	1	3.742747	3.757337	402.3303	107.0712
GJA1	2.78E-07	1	3.364492	3.367453	1560.419	463.3755
SLC22A31	2.82E-07	1	0.212995	0.210656	41.12215	195.2476
KLHL14	2.91E-07	1	0.252756	0.25164	97.80404	388.6956
CDKN1A	3.07E-07	1	3.342057	3.34452	1811.598	541.6545
DLC1	3.12E-07	1	3.784445	3.801332	352.3168	92.67511
GRAMD2	3.21E-07	1	0.24421	0.242847	76.68726	315.8152
FLI1	3.23E-07	1	4.329819	4.371969	196.7195	44.98792
SYTL2	3.24E-07	1	3.343168	3.345936	1643.775	491.268
KCNJ1	3.55E-07	1	0.083969	0.075018	4.445638	59.38405

DUSP4	3.69E-07	1	4.197613	4.234437	213.3906	50.38647
TMEM159	3.7E-07	1	3.335512	3.338477	1525.965	457.0772
SMIM11A	3.74E-07	1	54.5181	3102.944	31.01944	0
ALDH3B2	3.75E-07	1	0.232937	0.231189	57.7933	250.0158
MIPEPP3	4.08E-07	1	0.121486	0.11502	8.891277	77.37921
ZP1	4.14E-07	1	8.505471	9.098579	65.57317	7.198066
FAM86JP	4.21E-07	1	0.197977	0.195009	30.00806	153.9217
TGM3	4.33E-07	1	0.039279	0.026512	1.11141	42.28864
VIM	4.65E-07	1	3.275267	3.276733	2895.222	883.5627
SDCBP2-A	4.67E-07	1	0.135808	0.130126	11.1141	85.47704
LINC0083	4.76E-07	1	6.503383	6.750632	85.57854	12.6686
SFTA1P	4.86E-07	1	3.884998	3.90855	277.8524	71.08091
TMEM37	4.92E-07	1	0.199218	0.196236	30.00806	152.9589
LINC0121	5.13E-07	0.999999	0.05801	0.046816	2.233933	47.92113
C1orf228	5.24E-07	0.999999	0.122904	0.116373	8.891277	76.47946
COL15A1	5.28E-07	0.999999	12.10754	13.86062	50.01343	3.599033
GBP2	5.29E-07	0.999999	3.263146	3.264605	2846.32	871.8658
PLCH1	5.39E-07	0.999999	0.07293	0.062985	3.334229	53.08574
ADGRB2	5.42E-07	0.999999	3.323517	3.327241	1182.54	355.4045
PCK2	5.53E-07	0.999999	0.292511	0.292358	770.2068	2634.492
SERF2-C1	5.59E-07	0.999999	14.85977	18.04907	44.66755	2.465338
SEMA3D	5.64E-07	0.999999	0.232628	0.230738	53.34766	231.2379
PPARGC1	5.68E-07	0.999999	0.283251	0.282801	256.7356	907.8561
CD68	5.77E-07	0.999999	3.513396	3.52326	511.3595	145.131
GALNT1	5.92E-07	0.999999	3.310526	3.31412	1213.659	366.2016
RENBP	5.92E-07	0.999999	0.01667	0.000292	0	34.19082
TMEM101	5.94E-07	0.999999	0.137428	0.13151	11.1141	84.57728
LXN	6.08E-07	0.999999	4.063955	4.096277	221.1705	53.9855
POU2F2	6.34E-07	0.999999	6.889365	7.199774	77.79867	10.7971
LOC10192	7E-07	0.999999	0.116654	0.109576	7.779867	71.08091
AMZ1	7.28E-07	0.999999	3.524103	3.535123	461.235	130.465
PHYH	7.71E-07	0.999999	0.292098	0.291811	410.1101	1405.422
TMEM59L	7.97E-07	0.999999	3.771493	3.792521	290.0779	76.47946
SAA1	8.34E-07	0.999999	3.269815	3.273316	1231.909	376.3419
SLC35F3	8.4E-07	0.999999	4.712749	4.785206	137.8148	28.79227
NPIPA8	8.51E-07	0.999999	0.116226	0.108997	7.579813	69.6233
PPP1R16I	8.79E-07	0.999999	8.219676	8.7902	63.35035	7.198066
COL4A2	8.92E-07	0.999999	3.26246	3.26255	46771.45	14335.85
MFI2	9.02E-07	0.999999	3.585618	3.599871	378.9907	105.2717
PPP3CB-A	9.06E-07	0.999999	0.196974	0.193593	26.67383	137.825
TMEM74B	9.09E-07	0.999999	7.371516	7.774428	70.0188	8.997583
SERPING	9.82E-07	0.999999	3.353855	3.360362	692.4082	206.0447
PCDH19	1E-06	0.999999	5.975823	6.172563	88.91277	14.39613
ABCA5	1.01E-06	0.999999	0.262793	0.261617	93.3584	356.8801
ADM	1.03E-06	0.999999	3.231736	3.234602	1434.83	443.5808
DDR2	1.05E-06	0.999999	0.195528	0.192022	25.56242	133.1642
SOX6	1.11E-06	0.999999	0.127278	0.12063	8.891277	73.78018
KCNQ3	1.14E-06	0.999999	4.852297	4.939361	124.4779	25.19323
ANKRD35	1.15E-06	0.999999	0.176471	0.172198	18.89396	109.7705
TFPI	1.17E-06	0.999999	3.713716	3.734067	288.9665	77.37921
LINC0067	1.23E-06	0.999999	5.334869	5.467945	103.3611	18.89492
PDLIM3	1.24E-06	0.999999	4.302074	4.351833	164.4886	37.78985
FAM86C1	1.3E-06	0.999999	0.237275	0.235244	50.3024	213.8636
ADGRF1	1.33E-06	0.999999	3.614953	3.631287	326.7544	89.97583
RCN3	1.35E-06	0.999999	3.231256	3.235064	1100.295	340.1086
CX3CL1	1.37E-06	0.999999	3.180837	3.182916	1901.622	597.4395

UST	1.4E-06	0.999999	0.235649	0.233539	48.16849	206.2876
SLC27A6	1.44E-06	0.999999	0.129053	0.122119	8.891277	72.88042
NOTCH3	1.45E-06	0.999999	3.15451	3.155233	5351.437	1696.044
FAM86HP	1.45E-06	0.999999	0.186988	0.183056	21.55023	117.7694
TLL1	1.45E-06	0.999999	3.623428	3.641053	314.5289	86.3768
ELL2	1.48E-06	0.999999	3.183894	3.186367	1602.653	502.9649
SCN4B	1.48E-06	0.999999	4.272191	4.32243	163.3772	37.78985
CIRBP	1.48E-06	0.999999	0.290404	0.289881	221.1705	762.995
UPK1B	1.5E-06	0.999998	3.340012	3.347443	600.3835	179.3488
MOV10L1	1.55E-06	0.999998	0.238621	0.23657	50.01343	211.4432
FGD6	1.56E-06	0.999998	3.228111	3.232176	1009.16	312.2161
GLIPR2	1.56E-06	0.999998	3.242308	3.246814	920.2471	283.4239
EDNRB	1.61E-06	0.999998	13.35568	16.00243	43.34497	2.699275
NEBL	1.63E-06	0.999998	0.26144	0.260073	80.02149	307.7173
TMEM178	1.69E-06	0.999998	3.320929	3.328304	607.941	182.6509
ITGA5	1.7E-06	0.999998	3.138271	3.139264	3849.923	1226.371
WNT6	1.75E-06	0.999998	3.541847	3.556745	345.6484	97.1739
TINAGL1	1.77E-06	0.999998	3.147059	3.147402	11435.29	3633.242
DNAJA4	1.79E-06	0.999998	4.766051	4.851166	122.2551	25.19323
PNMA2	1.81E-06	0.999998	6.653531	6.959143	73.31969	10.52717
ZNF497	1.84E-06	0.999998	0.130524	0.123645	8.891277	71.98066
GALNT10	1.84E-06	0.999998	3.135399	3.135888	7798.761	2486.932
C17orf58	1.84E-06	0.999998	0.254566	0.252977	66.68457	263.6292
SCNN1A	1.93E-06	0.999998	0.234284	0.232008	44.45638	191.6485
FLJ23867	1.96E-06	0.999998	3.14623	3.146476	15927.61	5062.04
ITGB6	1.97E-06	0.999998	3.122761	3.123662	4193.348	1342.439
FKBP1A-S	2.25E-06	0.999998	10.6555	12.03186	48.06846	3.985929
GPNMB	2.33E-06	0.999998	0.30751	0.307461	2472.886	8042.939
MMD	2.45E-06	0.999998	3.193058	3.197452	909.133	284.3236
FAM20A	2.46E-06	0.999998	0.232152	0.229709	41.12215	179.0519
SEPT5-GP	2.52E-06	0.999997	48.4012	2748.404	27.47404	0
RNF157-A	2.84E-06	0.999997	0.16004	0.154502	13.33691	86.3768
LCK	2.86E-06	0.999997	4.990283	5.100061	105.5839	20.69444
GSTO1	2.89E-06	0.999997	3.136138	3.139254	1225.885	390.4951
CTTNBP2	2.93E-06	0.999997	0.183971	0.179556	18.89396	105.2717
CACNA1G	2.97E-06	0.999997	5.236793	5.370827	96.69263	17.99517
WBSCR27	3.12E-06	0.999997	0.064029	0.051688	2.222819	43.1884
DOCK4	3.15E-06	0.999997	3.204984	3.210523	727.9733	226.7391
PPP1R12B	3.17E-06	0.999997	3.080318	3.081618	2771.9	899.4884
CLDN11	3.18E-06	0.999997	4.605276	4.683948	122.2551	26.09299
KLK7	3.46E-06	0.999997	0.304746	0.304339	302.3034	993.3332
LOC10192	3.61E-06	0.999996	5.401037	5.555728	90.02418	16.19565
KLF7	3.62E-06	0.999996	3.131576	3.135263	1032.5	329.3115
FUZ	3.7E-06	0.999996	0.297299	0.296675	190.051	640.6279
ENPP1	3.81E-06	0.999996	0.29178	0.290977	148.9622	511.9625
ATG9B	4.03E-06	0.999996	7.648086	8.173441	58.90471	7.198066
SH3RF3	4.34E-06	0.999996	3.27727	3.286168	479.0175	145.7608
ACPP	4.38E-06	0.999996	0.220741	0.21758	31.11947	143.0616
MSN	4.38E-06	0.999996	3.096667	3.096769	37022.16	11955.09
IDNK	4.4E-06	0.999996	0.195024	0.190881	21.11678	110.6703
ALDH3B1	4.45E-06	0.999996	0.306595	0.30617	284.6098	929.6033
CRLF1	4.53E-06	0.999995	3.827571	3.85953	194.4967	50.38647
AQP1	4.6E-06	0.999995	3.854013	3.888097	188.9396	48.58695
CCL20	4.63E-06	0.999995	6.303215	6.582729	71.13021	10.7971
CARD11	4.77E-06	0.999995	3.36192	3.374076	373.4336	110.6703
TNFSF9	4.98E-06	0.999995	0.31125	0.310914	370.0994	1190.38

CFAP74	5.11E-06	0.999995	0.137336	0.130152	8.891277	68.38163
ABTB2	5.14E-06	0.999995	3.134397	3.139274	782.4323	249.2331
PTGES3L-	5.23E-06	0.999995	45.41034	2622.815	26.21815	0
VGLL3	5.25E-06	0.999995	3.15607	3.161686	691.2968	218.6413
CST6	5.3E-06	0.999995	3.263681	3.272809	462.3464	141.2621
FIBIN	5.59E-06	0.999994	0.097077	0.086861	4.445638	51.28622
VIL1	5.68E-06	0.999994	0.145841	0.139083	10.00269	71.98066
JPH2	5.71E-06	0.999994	3.027907	3.029572	2101.676	693.7137
FOSL1	5.93E-06	0.999994	3.08781	3.091601	956.9236	309.5169
CH17-360	5.94E-06	0.999994	45.71666	2592.807	25.91807	0
ENTPD2	6.21E-06	0.999994	4.580992	4.664921	113.3638	24.29347
PRKACB	6.31E-06	0.999994	3.095726	3.100034	859.1196	277.1256
OSBPL6	6.39E-06	0.999994	0.262447	0.260589	60.01612	230.3381
ST3GAL1	6.46E-06	0.999994	3.010127	3.010606	7191.931	2388.858
MUTYH	6.91E-06	0.999993	0.288625	0.287555	108.9181	378.7982
KLF15	7.41E-06	0.999993	0.28427	0.283026	94.46981	333.8103
HSD17B8	7.63E-06	0.999992	0.297049	0.296182	136.7034	461.576
LOC28562	8.14E-06	0.999992	15.19245	19.66001	35.56511	1.799517
TRAF1	8.23E-06	0.999992	4.019779	4.066394	150.0403	36.89009
SVOPL	8.79E-06	0.999991	0.122875	0.114173	6.668457	58.48429
GPSM3	8.88E-06	0.999991	4.688792	4.784768	103.3611	21.5942
NOG	9.14E-06	0.999991	8.462983	9.248954	50.01343	5.39855
SLC8A3	9.14E-06	0.999991	8.462983	9.248954	50.01343	5.39855
H1F0	9.91E-06	0.99999	0.324434	0.324341	1340.36	4132.59
ARSJ	1.04E-05	0.99999	3.586867	3.611676	214.502	59.38405
GPR158	1.05E-05	0.999989	3.556513	3.579965	222.2819	62.08332
NRIP3	1.08E-05	0.999989	3.124932	3.132023	544.6685	173.8963
SAT1	1.16E-05	0.999988	2.958443	2.958795	9373.628	3168.049
QSOX1	1.18E-05	0.999988	2.994858	2.994966	32156.41	10736.82
EGFL7	1.19E-05	0.999988	3.050606	3.055486	731.3075	239.3357
P3H2	1.27E-05	0.999987	2.938181	2.939237	3073.047	1045.519
MYPN	1.28E-05	0.999987	10.26235	11.70495	42.23356	3.599033
SOX9	1.29E-05	0.999987	3.382789	3.400056	272.2953	80.07849
SPEG	1.29E-05	0.999987	2.992248	2.995621	1026.942	342.8079
C1QTNF3	1.3E-05	0.999987	9.673258	10.90131	43.84511	4.012922
LCA5L	1.31E-05	0.999987	0.11435	0.104849	5.557048	53.08574
MAP2K6	1.32E-05	0.999987	0.236117	0.233116	33.34229	143.0616
SEMA3F	1.33E-05	0.999987	2.995808	2.999298	976.929	325.7125
NOVA2	1.35E-05	0.999987	43.18553	2446.101	24.45101	0
PLSCR4	1.35E-05	0.999987	43.18553	2446.101	24.45101	0
STK17A	1.44E-05	0.999986	2.926478	2.927163	4630.132	1581.775
HRK	1.44E-05	0.999986	0.21625	0.212374	24.45101	115.1691
TRHDE	1.44E-05	0.999986	0.311477	0.310774	176.7141	568.6472
KCNK6	1.46E-05	0.999985	3.329497	3.345146	288.9665	86.3768
CDRT4	1.49E-05	0.999985	42.87873	2428.319	24.27319	0
PRR5-ARI	1.52E-05	0.999985	42.8212	2424.984	24.23984	0
SDSL	1.52E-05	0.999985	0.311957	0.311267	176.7141	567.7475
CH17-340	1.56E-05	0.999984	0.264839	0.262733	52.23625	198.8466
TICAM2	1.58E-05	0.999984	6.488378	6.848105	60.62739	8.844624
AK7	1.69E-05	0.999983	0.253628	0.251054	42.23356	168.2548
LRCH2	1.72E-05	0.999983	0.330165	0.329987	705.7451	2138.725
WNT7B	1.75E-05	0.999983	2.922284	2.924174	1693.81	579.2374
TCAF2	1.78E-05	0.999982	7.56214	8.165853	51.50272	6.298308
RPS6KL1	1.8E-05	0.999982	0.281622	0.279955	70.0188	250.1328
FAM156B	1.89E-05	0.999981	41.51338	2392.753	23.91753	0
GSDMB	1.95E-05	0.99998	3.509789	3.534228	206.7222	58.48429

FAM172BI	1.97E-05	0.99998	5.230645	5.40108	77.79867	14.39613
OGDHL	1.97E-05	0.99998	0.328257	0.327943	400.1074	1220.072
KCNIP1	2.03E-05	0.99998	2.929246	2.93193	1200.322	409.39
FBLN7	2.05E-05	0.99998	7.507284	8.105952	51.12484	6.298308
LINC0141	2.05E-05	0.99998	7.507284	8.105952	51.12484	6.298308
ULBP1	2.12E-05	0.999979	4.093364	4.153807	123.3665	29.69202
REN	2.15E-05	0.999979	8.868149	9.862151	44.45638	4.498792
TMPRSS4	2.15E-05	0.999979	0.331601	0.331357	526.8081	1589.873
RUNX2	2.15E-05	0.999978	3.125489	3.134768	408.9987	130.465
NRP1	2.21E-05	0.999978	3.054399	3.061391	512.3598	167.355
PANX2	2.23E-05	0.999978	3.291068	3.306956	276.741	83.67752
EXOSC6	2.25E-05	0.999978	0.287014	0.285458	74.46444	260.8849
CD70	2.3E-05	0.999977	0.317376	0.316688	178.9369	565.0482
LTBP4	2.31E-05	0.999977	2.877337	2.878091	4158.895	1445.012
LOC10192	2.41E-05	0.999976	0.118501	0.108527	5.557048	51.28622
FUT3	2.47E-05	0.999975	2.96908	2.973686	723.9833	243.4566
SOD2	2.5E-05	0.999975	2.886454	2.886754	10335	3580.138
IL20RB	2.67E-05	0.999973	0.315813	0.315009	155.5973	493.9673
S1PR3	2.68E-05	0.999973	2.933704	2.937542	859.0196	292.4214
LGALS14	2.69E-05	0.999973	0.212185	0.207772	21.11678	101.6727
MGAT4A	2.82E-05	0.999972	2.931489	2.935319	843.3154	287.2928
ALDH1L1	2.88E-05	0.999971	0.2695	0.267277	50.01343	187.1497
NYNRIN	2.96E-05	0.99997	3.624108	3.656674	167.8228	45.88767
ADGRA2	3.08E-05	0.999969	0.294051	0.292581	80.02149	273.5265
DENND3	3.11E-05	0.999969	2.87973	2.882163	1268.118	439.9818
DCBLD2	3.17E-05	0.999968	2.860345	2.860672	9451.427	3303.912
SNAI1	3.22E-05	0.999968	2.933012	2.937417	747.9786	254.6316
FOXL1	3.24E-05	0.999968	6.211041	6.540568	58.90471	8.997583
AKAP12	3.31E-05	0.999967	2.855058	2.856782	1750.47	612.7354
TPK1	3.37E-05	0.999966	0.337368	0.337135	545.7021	1618.665
ZNF774	3.38E-05	0.999966	0.248623	0.245516	34.4537	140.3623
EPHX2	3.5E-05	0.999965	0.298666	0.297252	85.57854	287.9227
CHN2	3.52E-05	0.999965	0.322595	0.321897	177.8255	552.4516
TPM2	3.57E-05	0.999964	2.854822	2.855087	11351.94	3976.032
TTC12	3.61E-05	0.999964	0.282241	0.280296	60.01612	214.1425
MLLT11	3.74E-05	0.999963	2.918835	2.923158	739.0874	252.8321
PMS2	3.81E-05	0.999962	0.333763	0.333355	310.061	930.1431
TUBB3	3.83E-05	0.999962	2.876362	2.879386	1017.718	353.4431
GAS5	3.9E-05	0.999961	0.340154	0.3401	2391.753	7032.511
CD34	3.94E-05	0.999961	13.791	17.8174	32.23088	1.799517
FSCN1	4.02E-05	0.99996	2.826161	2.826723	5229.182	1849.903
FGF18	4.12E-05	0.999959	0.328626	0.328039	213.3906	650.5253
SPOCD1	4.19E-05	0.999958	2.912859	2.917463	703.5223	241.1352
SPIRE1	4.19E-05	0.999958	0.342201	0.342108	1395.93	4080.404
ADGRG6	4.21E-05	0.999958	2.977355	2.983943	510.137	170.9541
FOXC1	4.22E-05	0.999958	2.8776	2.880977	912.4673	316.7149
GBP1	4.51E-05	0.999955	2.812051	2.812979	3130.841	1112.992
FIBCD1	4.62E-05	0.999954	7.184607	7.753588	48.90202	6.298308
ITGA11	4.75E-05	0.999953	39.35048	2223.819	22.22819	0
CCDC3	4.77E-05	0.999952	0.304533	0.303217	90.02418	296.9202
RP1	4.87E-05	0.999951	11.02349	13.13086	35.56511	2.699275
FLRT2	4.87E-05	0.999951	0.243443	0.239998	30.00806	125.0664
MTMR10	5.03E-05	0.99995	0.342125	0.341872	505.6914	1479.203
IL23A	5.09E-05	0.999949	3.081893	3.09299	336.7571	108.8708
CCDC80	5.3E-05	0.999947	2.795882	2.796753	3286.438	1175.084
GNAL	5.38E-05	0.999946	0.178826	0.172241	12.22551	71.02692

C19orf38	5.43E-05	0.999946	0.206884	0.201761	17.78255	88.17631
PRSS23	5.59E-05	0.999944	2.78966	2.790537	3304.243	1184.082
PLEKHH1	5.63E-05	0.999944	0.31041	0.309152	98.4042	318.3255
PCSK5	5.66E-05	0.999943	0.245264	0.241737	30.00806	124.1666
MIR31HG	5.72E-05	0.999943	2.989437	2.997458	423.4471	141.2621
KCNK15	5.8E-05	0.999942	0.323443	0.322576	143.3718	444.4806
FXYD5	6.07E-05	0.999939	2.782646	2.783575	3043.039	1093.206
SCNN1B	6.11E-05	0.999939	6.647804	7.094113	51.12484	7.198066
COX6B2	6.18E-05	0.999938	5.522703	5.754657	63.06138	10.95006
SDS	6.21E-05	0.999938	0.234985	0.231048	25.56242	110.6703
LAMA3	6.46E-05	0.999935	2.817275	2.817415	20911.17	7422.106
PLAUR	6.65E-05	0.999934	2.778637	2.779987	2086.116	750.3984
SNAPC1	6.74E-05	0.999933	2.771213	2.772024	3514.277	1267.759
FRMD6	6.77E-05	0.999932	2.794762	2.79698	1313.686	469.6738
EMB	6.78E-05	0.999932	3.161803	3.176878	259.4808	81.67106
RAMP1	6.9E-05	0.999931	5.521681	5.760004	62.23894	10.7971
HINT2	6.9E-05	0.999931	0.336082	0.335505	220.0591	655.9238
DACT3	7.04E-05	0.99993	3.611316	3.648878	144.4832	39.58937
GALNT18	7.05E-05	0.99993	2.88974	2.895153	575.7102	198.8466
TNFAIP3	7.06E-05	0.999929	2.771049	2.772385	2132.795	769.2934
GLI1	7.07E-05	0.999929	0.229878	0.225639	23.3396	103.4722
RSPO4	7.51E-05	0.999925	2.990806	2.999689	377.8793	125.9662
TRAK2	7.67E-05	0.999923	0.350089	0.349973	1110.298	3172.548
SLC16A6	7.76E-05	0.999922	0.19494	0.189024	14.44832	76.47946
LOX	7.8E-05	0.999922	2.757136	2.757883	3764.344	1364.933
DOCK2	7.82E-05	0.999922	6.214658	6.581008	53.34766	8.097825
PTGS2	7.82E-05	0.999922	6.214658	6.581008	53.34766	8.097825
CFAP206	8.01E-05	0.99992	0.078371	0.063612	2.222819	35.09057
PPT2-EGI	8.04E-05	0.99992	4.983397	5.149181	70.46337	13.67633
MAP3K12	8.27E-05	0.999917	0.334227	0.333525	180.0484	539.855
TGM1	8.65E-05	0.999914	0.220829	0.21595	20.00537	92.67511
APH1B	8.68E-05	0.999913	0.283067	0.280775	50.01343	178.1521
CKS2	8.75E-05	0.999912	2.747555	2.748213	4156.672	1512.494
TGM5	8.93E-05	0.999911	4.1661	4.247752	95.58122	22.49396
NEURL1B	9E-05	0.99991	2.789967	2.792925	962.4807	344.6074
APBA1	9.12E-05	0.999909	0.284981	0.282729	51.12484	180.8514
GCOM1	9.52E-05	0.999905	3.234247	3.254394	209.4451	64.35071
FAM72A	9.62E-05	0.999904	2.805475	2.809163	783.2103	278.7991
HLA-DPB1	9.65E-05	0.999904	3.797038	3.850083	117.8094	30.59178
NSG1	9.7E-05	0.999903	5.423989	5.657163	61.12753	10.7971
TNS1	9.71E-05	0.999903	2.735805	2.736823	2706.282	988.8344
TAGLN	9.71E-05	0.999903	2.795265	2.79535	34291.43	12267.3
MALT1	9.74E-05	0.999903	2.828377	2.832846	660.1773	233.0374
SMPD3	9.75E-05	0.999903	0.114815	0.103144	4.445638	43.1884
LBH	9.84E-05	0.999902	2.735859	2.736913	2568.468	938.4479
RCAN2	9.89E-05	0.999901	3.660927	3.704923	130.0349	35.09057
KCNMB4	1E-04	0.9999	3.860847	3.917726	112.2301	28.63931
PADI1	0.000102	0.999898	3.145153	3.161318	238.9531	75.5797
HSPE1-M	0.000105	0.999895	6.92565	7.481914	46.24575	6.172342
TUBA1A	0.000106	0.999894	2.738293	2.73992	1666.981	608.3986
SGK1	0.000106	0.999894	2.845155	2.850441	566.8189	198.8466
KIAA1462	0.000108	0.999892	2.779465	2.782763	871.3451	313.1159
UNKL	0.000111	0.999889	2.971635	2.981384	343.4367	115.1871
C6orf15	0.000114	0.999886	6.086577	6.443929	52.23625	8.097825
KCNK13	0.000116	0.999884	3.31032	3.334692	180.0484	53.9855
CYTIP	0.000117	0.999883	0.171481	0.163623	10.00269	61.18356

ABCA1	0.000118	0.999882	0.343329	0.342743	218.9477	638.8284
FAM219A	0.000118	0.999882	2.80128	2.805599	679.0713	242.035
ACSL5	0.000119	0.999881	2.825179	2.830202	585.7128	206.9444
FAM86EP	0.000122	0.999878	0.280465	0.277904	44.47861	160.076
ELOVL5	0.000128	0.999872	2.710083	2.710714	4263.367	1572.778
SFXN2	0.000128	0.999872	0.279529	0.276908	43.34497	156.5579
ITIH5	0.00013	0.99987	3.998217	4.068461	98.90434	24.30247
APBA2	0.000134	0.999866	2.926917	2.935756	364.5423	124.1666
C12orf75	0.000136	0.999864	2.706026	2.706581	4739.05	1750.93
LTF	0.000137	0.999863	5.326298	5.554323	60.01612	10.7971
WNT3A	0.000137	0.999863	17.67112	28.10903	25.56242	0.899758
DAB2	0.000145	0.999855	2.803757	2.808721	581.2672	206.9444
PIR	0.000145	0.999855	0.347114	0.346548	227.839	657.4714
RHOV	0.000146	0.999854	3.239244	3.26139	187.8282	57.58453
SEC14L2	0.000146	0.999854	2.725924	2.728326	1116.967	409.39
RARB	0.000147	0.999853	0.057332	0.038935	1.11141	28.79227
CAMK2N1	0.00015	0.99985	2.713591	2.715623	1304.795	480.4709
DGAT2	0.000152	0.999848	2.799829	2.804804	577.933	206.0447
RXFP1	0.000156	0.999844	6.709504	7.225042	45.56779	6.298308
CXCL2	0.000159	0.999841	0.267339	0.26414	34.4537	130.465
LOC10013	0.000159	0.999841	0.102004	0.088472	3.334229	37.78985
HPGD	0.000168	0.999832	0.02512	0.000444	0	22.49396
CD200	0.00017	0.99983	3.473436	3.508546	138.9262	39.58937
COL6A2	0.000177	0.999823	2.677399	2.678107	3612.081	1348.738
FAM86C2	0.00018	0.99982	0.287709	0.285193	45.97901	161.2457
LINC0049	0.000182	0.999818	0.279139	0.276251	40.01074	144.8611
XG	0.000188	0.999812	0.29345	0.291064	50.01343	171.8538
UPK1A	0.000189	0.999811	0.204689	0.198357	14.44832	72.88042
ACVR2A	0.000199	0.999801	0.320018	0.318518	82.24431	258.2306
KIAA1024	0.0002	0.9998	3.593766	3.638132	121.1436	33.29106
SLC9A5	0.000201	0.999799	5.630803	5.923638	53.34766	8.997583
SERPINA3	0.000219	0.999781	0.292735	0.290289	47.79061	164.6558
PYY	0.000223	0.999777	0.104274	0.090629	3.334229	36.89009
PLXNB3	0.000225	0.999775	2.87032	2.879025	352.3168	122.3671
SHANK1	0.000237	0.999763	5.354923	5.61003	55.57048	9.897341
PLEKHG4	0.000241	0.999759	0.354023	0.353438	222.2819	628.9311
CMTM3	0.000253	0.999747	2.97191	2.984912	257.847	86.3768
CKMT1A	0.000255	0.999745	0.324418	0.322956	83.86697	259.7062
ADRB2	0.000262	0.999738	2.951981	2.964337	266.7383	89.97583
S100A8	0.000262	0.999738	4.407244	4.527004	73.35303	16.19565
SLC17A7	0.000275	0.999725	2.920455	2.932031	282.298	96.27414
PRSS3P2	0.000277	0.999723	0.026611	0.000463	0	21.5942
GEM	0.000277	0.999723	6.975689	7.605025	41.12215	5.39855
PCOLCE2	0.000279	0.999721	3.176467	3.199051	175.6027	54.88526
ZNF70	0.000286	0.999714	0.334539	0.333281	102.2497	306.8176
DNAJC27-	0.000292	0.999708	0.137856	0.126682	5.557048	43.9352
SAMD5	0.000292	0.999708	0.104365	0.090304	3.223088	35.79239
SMTN	0.000294	0.999706	2.649797	2.651928	1169.203	440.8816
LMLN	0.000297	0.999703	0.363639	0.363239	335.6457	924.0518
CGB7	0.0003	0.9997	10.14655	12.20637	30.86384	2.519323
KCNS3	0.000304	0.999696	3.925372	4.000815	90.02418	22.49396
APOL1	0.000316	0.999684	2.648333	2.648533	12428.62	4692.635
NRCAM	0.000316	0.999684	2.659375	2.659519	17463.58	6566.436
ZBTB20	0.000327	0.999673	2.909967	2.921592	273.4068	93.57486
MYLK-AS1	0.000331	0.999669	0.217044	0.211	15.55973	73.78018
CEACAM1	0.000333	0.999667	2.855534	2.865162	314.5289	109.7705

GALNT5	0.000336	0.999664	5.253312	5.497849	54.45907	9.897341
LINC0154	0.000336	0.999664	0.342079	0.340994	117.8094	345.5072
DGCR6	0.000343	0.999657	0.347741	0.346792	138.2816	398.7639
OSCP1	0.000353	0.999647	0.313669	0.311676	61.12753	196.1473
ZEB1	0.000357	0.999643	5.702336	6.032693	48.90202	8.097825
ZC3H12A	0.00036	0.99964	2.613527	2.614949	1689.343	646.0265
SCEL	0.000363	0.999637	2.627417	2.62949	1175.871	447.1799
SH3TC2	0.000366	0.999634	2.882209	2.893449	281.1866	97.1739
MIR181A2	0.00037	0.99963	9.639312	11.48996	31.11947	2.699275
NKX3-1	0.000373	0.999627	4.24577	4.353854	74.46444	17.09541
PXDN	0.00038	0.99962	2.622276	2.622534	9391.411	3581.038
CSGALNA	0.00038	0.99962	5.035884	5.2458	56.68189	10.7971
RDH12	0.000382	0.999618	0.14042	0.128872	5.557048	43.1884
KLHDC9	0.000384	0.999616	0.247046	0.242285	22.22819	91.77535
STBD1	0.000388	0.999612	2.635231	2.637914	933.1284	353.731
MXRA5	0.00039	0.99961	0.369248	0.368917	395.6618	1072.512
GLS	0.000393	0.999607	2.609244	2.609618	6398.385	2451.841
PLA2R1	0.000396	0.999604	4.159775	4.259736	76.68726	17.99517
LDHD	0.000406	0.999594	0.207209	0.200428	13.33691	66.58211
LRRN1	0.000407	0.999593	4.489038	4.629596	66.68457	14.39613
UBASH3B	0.00041	0.99959	2.611145	2.613044	1262.561	483.1702
CCBL1	0.000419	0.999581	2.642084	2.645275	790.2122	298.7198
DKK3	0.000423	0.999577	2.590332	2.591121	3030.814	1169.686
PCDHGC3	0.000425	0.999575	2.590761	2.591621	2732.2	1054.238
TENM1	0.00043	0.99957	2.882209	2.89411	265.6269	91.77535
GPC4	0.000434	0.999566	2.589877	2.590543	3524.28	1360.435
MLH1	0.000447	0.999553	0.368444	0.368006	302.3034	821.4793
FBF1	0.000452	0.999548	0.33409	0.332588	85.57854	257.3309
DSE	0.000454	0.999546	2.674305	2.678862	571.2645	213.2427
TUBB2A	0.000459	0.999541	2.583371	2.584249	2654.58	1027.209
PTRF	0.000461	0.999539	2.59361	2.593979	6371.711	2456.34
MTMR11	0.000463	0.999537	2.820963	2.830569	305.6376	107.971
BCO2	0.000479	0.999521	5.147231	5.385669	53.34766	9.897341
MGAT5B	0.000479	0.999521	2.976735	2.992776	210.0564	70.18115
GAD1	0.000484	0.999516	2.705226	2.710907	463.4578	170.9541
KIF3C	0.000492	0.999508	2.589812	2.591575	1331.469	513.762
RAP2C-AS	0.000496	0.999504	0.195013	0.187293	11.1141	59.38405
ZCWPW1	0.000501	0.999499	0.321787	0.31987	64.46176	201.5459
SYNDIG1L	0.000505	0.999495	7.322806	8.136663	36.67652	4.498792
ZNF559-Z	0.000505	0.999495	32.68496	1813.709	18.12709	0
BTC	0.000506	0.999494	0.330772	0.329106	76.68726	233.0374
CC2D1B	0.000508	0.999492	0.373818	0.373494	411.2438	1101.088
EPHA7	0.000513	0.999487	0.063399	0.042961	1.11141	26.09299
ACADSB	0.000518	0.999482	0.357415	0.356576	155.5973	436.3828
FAM20C	0.000519	0.999481	2.691781	2.697245	475.6833	176.3526
TLE6	0.000521	0.999479	0.30238	0.299718	45.56779	152.0592
TMEM150	0.000525	0.999475	0.089685	0.072964	2.222819	30.59178
CYGB	0.000532	0.999468	2.573918	2.574384	5038.02	1956.974
LTBP2	0.000544	0.999456	2.574501	2.57595	1573.756	610.9359
EEF1A2	0.00056	0.99944	2.571176	2.571607	5340.323	2076.642
DAW1	0.000565	0.999435	3.337563	3.373647	124.4779	36.89009
STEAP1	0.000585	0.999415	2.75833	2.766711	336.8794	121.7553
HLA-B	0.000599	0.999401	2.587321	2.587509	12437	4806.545
TMX2-CTN	0.000604	0.999396	0.02815	0.000499	0	20.01062
LOC10272	0.000606	0.999394	9.328689	11.07974	30.00806	2.699275
KATNAL1	0.00061	0.99939	2.679374	2.684969	459.0233	170.9541

ACAD11	0.000647	0.999353	0.374049	0.373588	294.0679	787.1626
PLEKHO1	0.000652	0.999348	2.585706	2.588369	878.0136	339.2089
ACTBL2	0.000655	0.999345	4.12001	4.223905	72.24162	17.09541
GNG2	0.000657	0.999343	6.603866	7.194042	38.89934	5.39855
DIXDC1	0.000672	0.999328	2.633493	2.637874	560.1504	212.343
ITGB5	0.000672	0.999328	2.557769	2.558121	6453.955	2522.922
DISC1	0.000679	0.999321	2.930072	2.945713	207.0445	70.28012
PRSS12	0.000681	0.999319	2.622566	2.626626	597.9384	227.6389
RAC2	0.000696	0.999304	2.578025	2.580647	884.682	342.8079
COL4A3	0.000698	0.999302	3.251627	3.284015	130.0349	39.58937
MURC	0.000707	0.999293	0.247046	0.241767	20.00537	82.77776
MMP15	0.000715	0.999285	2.546749	2.548217	1529.3	600.1388
CASC8	0.000729	0.999271	3.132435	3.15888	147.8175	46.78743
UBXN10	0.000734	0.999266	0.321736	0.3196	57.7933	180.8514
STXBP4	0.000754	0.999246	0.29952	0.296508	40.01074	134.9637
BARX1	0.000763	0.999237	2.98602	3.004782	181.1598	60.28381
ECSCR	0.000767	0.999233	3.840948	3.919106	81.1329	20.69444
TMEM154	0.000769	0.999231	2.810012	2.821457	258.9584	91.77535
SLC6A17	0.000771	0.999229	5.440671	5.758536	46.6792	8.097825
CYP24A1	0.000772	0.999228	2.902911	2.917967	210.0564	71.98066
THAP7-AS	0.000787	0.999213	0.182773	0.173527	8.891277	51.28622
AOC1	0.000795	0.999205	7.104418	7.890164	35.56511	4.498792
RAB27B	0.000803	0.999197	15.41729	24.44407	22.22819	0.899758
ICT1	0.000819	0.999181	0.378541	0.378112	312.3061	825.9781
ACSS1	0.000823	0.999177	0.367564	0.366797	172.2685	469.6738
NCCRP1	0.00083	0.99917	3.686638	3.752026	87.80136	23.39372
CLCN5	0.000831	0.999169	0.377056	0.376572	275.7963	732.4033
EFNB3	0.000833	0.999167	2.718459	2.726516	331.2001	121.4674
KRT81	0.000838	0.999162	2.560273	2.562918	861.3091	336.0597
IDO1	0.00084	0.99916	2.734367	2.743228	311.5837	113.5765
ZNF404	0.000853	0.999147	3.13469	3.161712	142.2604	44.98792
DNAJB4	0.000853	0.999147	2.526158	2.527378	1776.033	702.7112
IFI44	0.000855	0.999145	3.121828	3.148161	144.4832	45.88767
TMCC2	0.000863	0.999137	2.892547	2.907998	206.7222	71.08091
AIM1	0.000866	0.999134	2.547453	2.549829	963.5921	377.8985
P2RX5	0.00087	0.99913	2.539981	2.542008	1100.407	432.8827
CTSK	0.000881	0.999119	3.564282	3.619501	94.46981	26.09299
EDN2	0.000888	0.999112	2.575889	2.579271	684.6283	265.4287
DBP	0.000892	0.999108	0.362033	0.361039	134.4806	372.4999
FAM72C	0.000897	0.999103	2.539869	2.54201	1041.535	409.7229
AARD	0.000898	0.999102	0.254741	0.24963	21.11678	84.62227
CLMP	0.000904	0.999096	3.978547	4.074555	73.35303	17.99517
PFN4	0.000917	0.999083	0.193939	0.185436	10.00269	53.9855
CCDC57	0.000922	0.999078	0.35517	0.353976	108.9181	307.7173
DSEL	0.000942	0.999058	0.273634	0.269456	26.67383	99.0184
PHYHIPL	0.000954	0.999046	0.259694	0.254772	22.22819	87.27656
B4GALNT	0.000955	0.999045	2.611896	2.616732	496.8001	189.849
ZNF771	0.000962	0.999038	0.313355	0.310703	46.6792	150.2596
RASGRP1	0.000973	0.999027	5.647522	6.014786	43.34497	7.198066
MZF1-AS1	0.000986	0.999014	0.334272	0.332382	66.68457	200.6461
WFDC21P	0.001	0.999	2.651172	2.657507	394.5504	148.4601
TFR2	0.001014	0.998986	3.733888	3.80733	82.24431	21.5942
C15orf54	0.001024	0.998976	8.963608	10.66951	28.89665	2.699275
EPHA1-AS	0.001024	0.998976	8.963608	10.66951	28.89665	2.699275
SUCO	0.001027	0.998973	2.507666	2.508196	4062.202	1619.565
TTC8	0.001041	0.998959	0.375982	0.375347	210.0564	559.6497

ZNF777	0.001053	0.998947	0.385627	0.385288	404.5531	1050.018
PCDHGC5	0.001128	0.998872	8.136242	9.383845	30.56376	3.248127
MORN1	0.001132	0.998868	0.293131	0.289569	33.34229	115.1691
HARBI1	0.001138	0.998862	0.307625	0.304742	41.12215	134.9637
RAB6B	0.001155	0.998845	2.583648	2.588291	512.3598	197.9468
NHSL1	0.001159	0.998841	0.384216	0.383782	310.0833	807.983
STK32A	0.001197	0.998803	0.197351	0.188578	10.00269	53.08574
COL7A1	0.001208	0.998792	2.819973	2.833526	216.7249	76.47946
FLNC	0.001228	0.998772	2.499244	2.499583	6295.024	2518.423
LANCL3	0.001235	0.998765	4.43017	4.585156	57.7933	12.59662
SEMA7A	0.001254	0.998746	2.493797	2.495389	1333.691	534.4564
PDGFRL	0.001255	0.998745	0.289621	0.285904	31.11947	108.8708
EFNA1	0.001266	0.998734	2.502099	2.504092	1074.733	429.1847
CLIC5	0.001269	0.998731	2.502501	2.50452	1061.396	423.7862
SCG5	0.001269	0.998731	5.046021	5.306708	47.79061	8.997583
TRIM54	0.001292	0.998708	29.23888	1668.114	16.67114	0
TEN1-CDK	0.001295	0.998705	4.712693	4.915276	52.26959	10.62615
SYTL1	0.001298	0.998702	0.390179	0.389881	462.3464	1185.881
FAM169A	0.001317	0.998683	0.365763	0.364675	123.3665	338.3091
SLC47A2	0.001328	0.998672	0.152771	0.140584	5.557048	39.58937
GPR143	0.001354	0.998646	0.367841	0.366825	130.0349	354.5048
GCSHP3	0.001381	0.998619	0.246937	0.241124	17.78255	73.78018
LRRC4B	0.001383	0.998617	3.480224	3.534346	92.247	26.09299
CXCL6	0.001418	0.998582	3.1724	3.205136	121.1436	37.78985
EFHD2	0.001432	0.998568	2.475885	2.476383	4177.789	1687.047
GAS6-AS2	0.001433	0.998567	3.221241	3.25714	114.7864	35.23454
CHMP1B2	0.001465	0.998535	0.311803	0.30886	41.12215	133.1642
GPR156	0.001481	0.998519	2.894716	2.912616	175.6027	60.28381
GJA5	0.001482	0.998518	7.585979	8.625431	31.11947	3.599033
MMP1	0.0015	0.9985	7.569072	8.625431	31.11947	3.599033
TACSTD2	0.001512	0.998488	2.48272	2.483005	7477.564	3011.491
NFAM1	0.001513	0.998487	4.784017	5.003686	50.01343	9.987317
SYT11	0.001513	0.998487	4.639016	4.834438	52.23625	10.7971
SSFA2	0.00152	0.99848	2.465513	2.466168	3199.748	1297.451
ZNF658	0.001529	0.998471	0.261582	0.256316	20.85004	81.37414
C1orf56	0.001532	0.998468	0.353811	0.352296	85.57854	242.9347
GOLGA8G	0.00157	0.99843	29.09173	1629.215	16.28215	0
PGBD5	0.001586	0.998414	2.744686	2.756311	235.6188	85.47704
ZNF90	0.001588	0.998412	6.899684	7.672775	33.34229	4.336835
PLPPR3	0.001604	0.998396	3.005438	3.029387	144.4832	47.68719
QPCTL	0.001628	0.998372	2.501629	2.504531	738.3427	294.7968
OLFML2A	0.001639	0.998361	2.59758	2.60394	379.5686	145.7608
ZCCHC8	0.001657	0.998343	0.390575	0.390146	315.6848	809.1616
CXorf38	0.001657	0.998343	2.467416	2.469014	1274.987	516.3893
IGSF3	0.001658	0.998342	2.473628	2.475629	1058.062	427.3852
RNFT1	0.001661	0.998339	0.376584	0.37572	154.4859	411.1895
IGFBP3	0.001667	0.998333	2.472879	2.473172	7087.459	2865.73
SHISA2	0.001667	0.998333	2.864797	2.881903	178.9369	62.08332
KLK10	0.001685	0.998315	2.458107	2.458636	3931.056	1598.871
UBA7	0.00169	0.99831	2.876456	2.894183	174.4913	60.28381
ADAMTS7	0.001705	0.998295	3.547557	3.60956	84.46713	23.39372
SLC16A2	0.001709	0.998291	8.625756	10.25929	27.78524	2.699275
KCNC4	0.001725	0.998275	3.213092	3.249951	111.141	34.19082
ZSCAN31	0.001737	0.998263	0.3822	0.381481	186.7168	489.4685
NLRP4	0.001741	0.998259	10.52094	13.51798	24.45101	1.799517
TSPAN5	0.001772	0.998228	2.58138	2.587303	393.439	152.0592

TACO1	0.001781	0.998219	0.385788	0.385148	214.502	556.9504
CADPS2	0.001786	0.998214	4.225944	4.361993	58.90471	13.49637
BORCS7-/	0.001807	0.998193	28.29758	1612.544	16.11544	0
TMX3	0.001813	0.998187	2.629597	2.6375	315.6403	119.6679
ERRFI1	0.001814	0.998186	2.45206	2.452572	3963.287	1615.966
NCEH1	0.001824	0.998176	2.454488	2.456049	1328.134	540.7547
KLHL22	0.001824	0.998176	0.389372	0.388847	257.847	663.1219
FAM134B	0.001826	0.998174	0.364379	0.363094	102.2497	281.6243
GOLGA1	0.001844	0.998156	0.388276	0.38771	238.9531	616.3344
RAB19	0.001849	0.998151	0.261037	0.25566	20.00537	78.27897
SLC44A3	0.001866	0.998134	2.458999	2.460799	1135.861	461.576
ZNF805	0.001867	0.998133	2.483245	2.486064	759.2039	305.378
ABCG2	0.001872	0.998128	5.739958	6.167951	38.89934	6.298308
KYNU	0.001872	0.998128	5.739958	6.167951	38.89934	6.298308
FHL2	0.001881	0.998119	2.443925	2.44462	2945.235	1204.776
MAPK8IP1	0.001889	0.998111	0.348572	0.346763	71.13021	205.1449
DPYD	0.001903	0.998097	2.548	2.553147	445.6752	174.5531
ZNF397	0.001906	0.998094	0.317974	0.315077	42.23356	134.064
CES1	0.001909	0.998091	14.26158	22.72153	20.6611	0.899758
TMEM8B	0.001911	0.998089	2.59442	2.60123	353.4282	135.8635
CD59	0.001912	0.998088	2.454486	2.454838	5764.881	2348.369
FAM110B	0.001912	0.998088	0.140881	0.126939	4.445638	35.09057
CRYAB	0.001914	0.998086	2.478608	2.478767	12933.47	5217.698
ARHGEF2	0.001931	0.998069	0.359533	0.358064	88.91277	248.3333
CORO7-P	0.001952	0.998048	0.031556	0.000562	0	17.78822
TMEM80	0.002005	0.997995	0.336201	0.333886	55.57048	166.4553
CCDC51	0.002029	0.997971	0.39404	0.393607	313.4175	796.2861
HKDC1	0.002051	0.997949	0.393574	0.393548	5280.307	13417.2
TMEM158	0.002056	0.997944	3.800768	3.889373	70.0188	17.99517
STYK1	0.002061	0.997939	2.909818	2.930694	155.5973	53.08574
TCTN1	0.002077	0.997923	0.376025	0.374999	132.2577	352.7053
SORCS2	0.002077	0.997923	2.744417	2.757498	213.3906	77.37921
NCAM1	0.002087	0.997913	0.371029	0.369872	114.4752	309.5169
PLEKHA7	0.002101	0.997899	0.380743	0.379876	154.4859	406.6908
KDM7A	0.002117	0.997883	2.446282	2.448152	1099.184	448.9794
AURKC	0.002155	0.997845	5.361727	5.706406	41.12215	7.198066
MAGEC2	0.002166	0.997834	2.575484	2.581918	360.0967	139.4625
MDP1	0.002196	0.997804	0.305789	0.302301	34.26476	113.3695
DUSP2	0.002197	0.997803	0.394549	0.39407	288.9665	733.303
LYRM4-AS	0.0023	0.9977	0.299468	0.295678	31.11947	105.2717
LINC0095	0.002314	0.997686	3.02045	3.048172	128.9235	42.28864
HLA-F	0.002327	0.997673	2.725509	2.7383	214.3687	78.27897
ARHGDIB	0.002436	0.997564	2.419168	2.42003	2240.602	925.8513
TCEANC	0.002446	0.997554	0.250236	0.243874	16.67114	68.39063
UNK	0.002506	0.997494	0.385047	0.38419	160.043	416.5881
LIF	0.00251	0.99749	2.418519	2.419747	1591.539	657.7233
THBD	0.002531	0.997469	27.35629	1556.973	15.55973	0
CKMT1B	0.002534	0.997466	0.376283	0.375193	120.6324	321.5376
BTN3A1	0.002549	0.997451	2.446514	2.449125	787.4781	321.5286
ZMAT1	0.002599	0.997401	0.232083	0.224718	13.33691	59.38405
HNRNPA1	0.002642	0.997358	13.92225	22.01297	20.01649	0.899758
DRAXIN	0.002658	0.997342	13.91474	22.00076	20.00537	0.899758
NTNG1	0.002701	0.997299	2.727786	2.741375	202.2765	73.78018
TFF3	0.002721	0.997279	13.82153	22.00076	20.00537	0.899758
ETS1	0.002742	0.997258	2.412795	2.41326	4175.566	1730.253
SLC31A2	0.002744	0.997256	3.419228	3.475928	84.46713	24.29347

ITGB1	0.002749	0.997251	2.498935	2.498972	57671.04	23077.9
GGT6	0.002761	0.997239	0.032709	0.000583	0	17.1404
NCF2	0.002772	0.997228	2.415503	2.417115	1209.214	500.2656
TPRG1L	0.002813	0.997187	2.404707	2.405836	1738.245	722.5059
STEAP4	0.002819	0.997181	2.798249	2.815304	172.2685	61.18356
TMOD1	0.002822	0.997178	2.426324	2.428552	902.4646	371.6002
ZFP91-CN	0.002859	0.997141	27.01742	1536.968	15.35968	0
MYLK3	0.002903	0.997097	0.349164	0.347055	62.15002	179.0969
DNAJC28	0.002904	0.997096	0.280884	0.276042	23.3396	84.57728
SALL1	0.002915	0.997085	3.055959	3.087526	116.698	37.78985
PLOD2	0.002939	0.997061	2.416966	2.417256	6716.248	2778.454
EIF5A2	0.002999	0.997001	2.542759	2.549214	347.0488	136.1334
LOC10065	0.003043	0.996957	0.298596	0.294525	28.89665	98.13664
CHKB-AS1	0.003045	0.996955	0.199947	0.190209	8.891277	46.78743
FRMD4B	0.003046	0.996954	2.562296	2.569514	316.7517	123.2669
PYM1	0.003051	0.996949	0.395346	0.394713	214.502	543.454
PLCE1	0.003072	0.996928	2.412472	2.414403	1005.826	416.5881
C2	0.003087	0.996913	0.373566	0.372229	101.1383	271.727
PLTP	0.003095	0.996905	0.406243	0.406158	1615.99	3978.731
LINC0127	0.003114	0.996886	2.939566	2.964118	133.3691	44.98792
RAB5B	0.003126	0.996874	2.407201	2.407535	5789.377	2404.685
AOC3	0.003194	0.996806	3.092473	3.126429	109.7295	35.09057
ERICH2	0.003203	0.996797	0.399575	0.399031	255.6242	640.6279
SEC23A	0.003212	0.996788	2.400588	2.400981	4877.977	2031.654
ADAMTS1	0.003237	0.996763	2.85451	2.874748	150.0403	52.18598
CAV1	0.003241	0.996759	2.39588	2.396354	4025.526	1679.849
CDH16	0.003338	0.996662	4.068029	4.197417	56.68189	13.49637
ADRBK2	0.003348	0.996652	2.550068	2.557196	316.7517	123.8607
PIK3CD-A	0.003429	0.996571	0.163776	0.150868	5.557048	36.89009
PLPPR2	0.003455	0.996545	2.383915	2.384849	2012.763	843.9733
TLR2	0.003506	0.996494	2.57422	2.582611	281.1866	108.8708
WDR86-A	0.003568	0.996432	0.295094	0.29072	26.67383	91.77535
FLJ37035	0.003582	0.996418	0.191194	0.180328	7.779867	43.1884
FANCE	0.003637	0.996363	0.405228	0.404796	323.4202	798.9854
FOLR1	0.003639	0.996361	0.407935	0.407621	439.0068	1077.011
SCML1	0.003656	0.996344	2.383914	2.385318	1339.249	561.4492
PCLO	0.00372	0.99628	0.349465	0.347239	57.7933	166.4553
FREM1	0.003726	0.996274	0.256854	0.250497	16.67114	66.58211
RASSF2	0.003728	0.996272	4.71419	4.946432	44.45638	8.979588
SENCR	0.003731	0.996269	5.860219	6.372077	34.4537	5.39855
IRAK2	0.003751	0.996249	3.102879	3.138848	104.517	33.29106
GBP6	0.003772	0.996228	7.062366	8.037243	28.99668	3.599033
GRAMD1E	0.00378	0.99622	2.93346	2.958955	127.8121	43.1884
MIA-RAB4	0.003802	0.996198	0.034306	0.000601	0	16.61854
ATP6V0D2	0.003856	0.996144	7.064921	8.009527	28.89665	3.599033
KLF6	0.003878	0.996122	2.373836	2.374468	2941.901	1238.967
COQ10B	0.003897	0.996103	2.376727	2.378085	1373.702	577.6448
PEAR1	0.003913	0.996087	2.780648	2.798259	161.1544	57.58453
LIPG	0.003963	0.996037	2.373446	2.374634	1542.636	649.6255
LINC0155	0.003988	0.996012	0.311219	0.30738	31.99748	104.12
HPN	0.004	0.996	0.203818	0.193937	8.891277	45.88767
ENG	0.004003	0.995997	2.860018	2.881819	140.0376	48.58695
B3GALNT	0.004041	0.995959	0.407782	0.407384	345.6484	848.4721
ANXA8L1	0.004052	0.995948	13.38885	21.1456	19.22739	0.899758
FAM161A	0.004058	0.995942	0.380538	0.379279	106.3508	280.4187
IL4R	0.004081	0.995919	2.3759	2.377461	1193.654	502.0651

POLR3C	0.004088	0.995912	0.412141	0.412018	1121.412	2721.769
TPBG	0.004092	0.995908	2.375532	2.377166	1161.423	488.5688
ETV7	0.004106	0.995894	2.985959	3.015741	116.698	38.68961
IL18BP	0.004106	0.995894	2.985959	3.015741	116.698	38.68961
RMND1	0.004134	0.995866	0.409384	0.409031	390.1048	953.7438
TTC39A	0.004192	0.995808	0.398211	0.397477	185.6054	466.9746
LYRM9	0.004209	0.995791	0.297998	0.293598	26.67383	90.87559
LOC90784	0.004278	0.995722	0.388313	0.38728	128.9235	332.9106
PLAU	0.004286	0.995714	2.365032	2.365605	3209.751	1356.836
MAT1A	0.00436	0.99564	0.390416	0.389419	135.592	348.2065
DOPEY1	0.004369	0.995631	0.397203	0.396413	172.2685	434.5833
SUN2	0.004433	0.995567	2.358271	2.359024	2470.663	1047.319
CSPG4	0.004444	0.995556	0.404942	0.404368	243.3987	601.9383
DNAJC25	0.004461	0.995539	2.425205	2.428965	524.5298	215.942
SAMD9L	0.00447	0.99553	2.712677	2.727536	176.7141	64.7826
KRT15	0.004475	0.995525	4.092771	4.232512	53.34766	12.59662
ZNF883	0.004534	0.995466	3.433298	3.498665	75.57585	21.5942
LOC10537	0.004545	0.995455	0.371248	0.369586	81.1329	219.541
TTC30A	0.004589	0.995411	0.305784	0.301655	28.87442	95.74328
CHAT	0.004594	0.995406	0.034551	0.000617	0	16.19565
UBXN11	0.00462	0.99538	0.388431	0.387338	123.3665	318.5144
YBEY	0.004625	0.995375	0.385091	0.383893	112.2524	292.4214
COL27A1	0.004663	0.995337	2.464938	2.470369	386.7705	156.5579
CLDN3	0.004673	0.995327	0.397495	0.396673	165.6	417.4879
GOLGA7B	0.004679	0.995321	3.08508	3.121735	101.1383	32.3913
HSPA1B	0.004687	0.995313	2.378356	2.378582	8156.935	3429.321
HOPX	0.004705	0.995295	7.972849	9.438843	25.56242	2.699275
CAPN3	0.004709	0.995291	0.167806	0.154639	5.557048	35.99033
FOS	0.004729	0.995271	0.414738	0.414529	662.4001	1597.971
COL11A2	0.004753	0.995247	2.844253	2.866277	136.7034	47.68719
RFESD	0.004789	0.995211	0.271857	0.265913	18.89396	71.08091
APCDD1L	0.004798	0.995202	25.92781	1445.832	14.44832	0
CCL11	0.004798	0.995202	25.92781	1445.832	14.44832	0
INSL4	0.004798	0.995202	25.92781	1445.832	14.44832	0
GIMAP2	0.004856	0.995144	13.16347	20.7791	18.89396	0.899758
HRAT92	0.004856	0.995144	13.16347	20.7791	18.89396	0.899758
LDLRAD4	0.004856	0.995144	13.16347	20.7791	18.89396	0.899758
SQSTM1	0.004865	0.995135	0.399758	0.399751	19115.13	47817.66
DRP2	0.004886	0.995114	6.241944	6.904171	31.11947	4.498792
ILDR2	0.004886	0.995114	6.241944	6.904171	31.11947	4.498792
XIRP1	0.004886	0.995114	6.241944	6.904171	31.11947	4.498792
PPIL6	0.004905	0.995095	0.367896	0.366108	73.70868	201.3479
LOC15222	0.004972	0.995028	13.07568	20.7791	18.89396	0.899758
IL34	0.004994	0.995006	0.234676	0.226602	12.22551	53.9855
KCNMA1	0.005044	0.994956	3.160391	3.203116	92.247	28.79227
BMF	0.0051	0.9949	2.489402	2.495904	325.643	130.465
GPRIN1	0.005112	0.994888	2.344667	2.345536	2066.11	880.8634
MYL9	0.005113	0.994887	2.374962	2.375151	9804.855	4128.091
ZNF365	0.005184	0.994816	2.567647	2.577189	241.1759	93.57486
GPRC5A	0.005274	0.994726	2.34581	2.346328	3466.486	1477.403
CYP27B1	0.005327	0.994673	3.229313	3.278879	85.57854	26.09299
THNSL1	0.005425	0.994575	0.41484	0.414482	393.439	949.245
LYRM5	0.005479	0.994521	0.40254	0.401776	178.9369	445.3804
HERC5	0.00548	0.99452	2.37332	2.375979	699.0766	294.221
MYO7A	0.005567	0.994433	0.377525	0.375967	85.57854	227.6389
LINC0046	0.005577	0.994423	0.357161	0.354913	57.7933	162.8563

SKIL	0.005604	0.994396	2.336356	2.337126	2279.501	975.338
KISS1	0.005621	0.994379	3.314718	3.372118	78.91008	23.39372
NKAIN4	0.005623	0.994377	3.315756	3.372118	78.91008	23.39372
STRIP2	0.005642	0.994358	2.459726	2.465676	351.0721	142.3778
MATN2	0.005799	0.994201	0.418991	0.418851	1004.714	2398.756
TMEM116	0.005818	0.994182	0.375405	0.373713	80.02149	214.1425
RIN1	0.005823	0.994177	2.681377	2.696366	172.2685	63.88284
GPC2	0.005827	0.994173	3.067669	3.105056	97.80404	31.49154
SELENBP	0.00588	0.99412	0.280325	0.274595	20.00537	72.88042
ERP27	0.005892	0.994108	0.419107	0.418919	735.7531	1756.328
CDKN2D	0.005919	0.994081	2.404212	2.408347	474.5719	197.0471
ZNF341	0.005938	0.994062	2.491258	2.49841	296.7464	118.7681
RHBDD3	0.005969	0.994031	0.408968	0.408335	221.1705	541.6545
CA2	0.005984	0.994016	0.398252	0.397277	142.2604	358.1038
SP140L	0.006056	0.993944	2.648826	2.662818	182.1045	68.38163
PDGFB	0.006058	0.993942	2.327803	2.328587	2243.936	963.6411
SLC9A4	0.00606	0.99394	0.332689	0.329383	38.57703	117.1395
ABCA3	0.006088	0.993912	2.559366	2.569107	231.1732	89.97583
KCNJ15	0.006165	0.993835	4.008422	4.144352	52.23625	12.59662
CDA	0.006347	0.993653	2.346612	2.348821	815.7746	347.3067
MALL	0.006375	0.993625	2.332366	2.332758	4523.937	1939.303
ALDH1B1	0.006391	0.993609	2.332552	2.332936	4612.172	1976.976
DPP4	0.006428	0.993572	2.975658	3.008324	105.5839	35.09057
TACC2	0.006448	0.993552	0.420057	0.419787	522.3625	1244.366
TP53AIP1	0.006491	0.993509	0.079487	0.054163	1.11141	20.69444
EPHB2	0.006524	0.993476	2.326024	2.327433	1248.113	536.2559
MTG1	0.006555	0.993445	0.416509	0.41607	321.1974	771.9926
OAS1	0.006699	0.993301	2.492772	2.500455	276.741	110.6703
ABHD14A	0.006838	0.993162	25.16683	1383.594	13.82594	0
CXCL8	0.007025	0.992975	2.368558	2.368652	19651.94	8296.671
ZBED5-AS	0.007062	0.992938	0.33407	0.33075	37.78793	114.2693
ALDH1A1	0.007079	0.992921	0.273518	0.267187	17.78255	66.58211
MAP3K9	0.007162	0.992838	2.309908	2.310746	2095.385	906.7944
PDZK1IP1	0.007279	0.992721	2.30936	2.310151	2178.363	942.9467
AKAP2	0.007339	0.992661	2.327545	2.327818	6439.096	2766.145
STK36	0.007595	0.992405	0.41813	0.417642	288.9665	691.9141
ROR1	0.007708	0.992292	2.398822	2.403609	400.1074	166.4553
P4HA3	0.00771	0.99229	6.023093	6.657673	30.00806	4.498792
LAT2	0.007745	0.992255	3.410524	3.479853	68.90739	19.79468
SYT3	0.007751	0.992249	2.94451	2.976661	104.4725	35.09057
ZNF268	0.007772	0.992228	0.394295	0.39305	108.9181	277.1256
ITGB2-AS	0.007828	0.992172	0.368148	0.366032	62.23894	170.0543
PPP1R1C	0.007932	0.992068	3.452361	3.526216	66.68457	18.90392
SH3BGR	0.007945	0.992055	0.408607	0.407773	167.9451	411.8734
CDHR2	0.00798	0.99202	0.137462	0.119854	3.334229	27.89251
LOC10192	0.00798	0.99202	0.137462	0.119854	3.334229	27.89251
PHOSPH(	0.007992	0.992008	0.406909	0.40604	157.8202	388.6956
ARHGEF1	0.008133	0.991867	2.311927	2.312252	5409.23	2339.372
RSRP1	0.008179	0.991821	2.383459	2.388035	419.0014	175.4529
IL12A	0.00821	0.99179	2.970313	3.004015	100.0269	33.29106
IFT88	0.008211	0.991789	0.365976	0.363746	58.90471	161.9565
SGIP1	0.008271	0.991729	0.419726	0.419248	290.0779	691.9141
CCDC115	0.00829	0.99171	0.421241	0.420816	326.7544	776.4914
RWDD2B	0.008313	0.991687	0.426027	0.425835	739.0874	1735.634
ABHD11	0.008406	0.991594	0.425218	0.42494	510.7594	1201.969
ARNTL2	0.008454	0.991546	2.294449	2.295213	2216.173	965.5576

RLTPR	0.008461	0.991539	3.74743	3.858112	55.57048	14.39613
BTN3A3	0.008663	0.991337	2.417976	2.423881	331.5557	136.7813
HPS4	0.008718	0.991282	0.426192	0.425946	566.8189	1330.743
ACOT13	0.008756	0.991244	0.426825	0.426629	712.4135	1669.879
TMEM51-/	0.008757	0.991243	2.751757	2.773072	132.2577	47.68719
IP6K3	0.008878	0.991122	0.176491	0.162775	5.557048	34.19082
TCF7	0.008895	0.991105	2.398499	2.4038	361.2081	150.2596
BCL6	0.008931	0.991069	2.291674	2.292911	1363.7	594.7402
GSTZ1	0.009015	0.990985	0.39657	0.395296	106.6953	269.9275
MDGA1	0.009058	0.990942	2.636325	2.651844	162.2658	61.18356
NLGN4X	0.009061	0.990939	0.216388	0.206056	8.891277	43.1884
KCNJ16	0.009125	0.990875	9.119489	11.67537	21.11678	1.799517
NPNT	0.009393	0.990607	2.484448	2.493396	240.0645	96.27414
UPK3B	0.009466	0.990534	0.410923	0.410068	160.8099	392.1687
RUSC2	0.009525	0.990475	2.282315	2.283247	1789.369	783.6895
TNC	0.009596	0.990404	2.297829	2.299753	883.5706	384.1968
BEAN1	0.009752	0.990248	5.098064	5.463223	34.4537	6.298308
WSCD1	0.009887	0.990113	23.5911	1334.691	13.33691	0
ITGAV	0.009938	0.990062	2.30445	2.304662	8234.434	3572.94
ZNF572	0.009959	0.990041	0.237722	0.228905	11.1141	48.58695
LOC10192	0.009972	0.990028	0.082998	0.056623	1.11141	19.79468
FAM160A1	0.009995	0.990005	2.342064	2.34579	481.2403	205.1449
NKX2-1	0.01002	0.98998	3.355974	3.423735	67.79598	19.79468
PRR15	0.010145	0.989855	0.40843	0.40745	140.0376	343.7077
SLC37A2	0.010203	0.989797	2.719455	2.739556	133.4692	48.71291
TDRD7	0.010254	0.989746	2.320855	2.32401	564.5961	242.9347
MCOLN3	0.010256	0.989744	0.422985	0.422448	263.4041	623.5325
SVEP1	0.010335	0.989665	6.507907	7.393623	26.67383	3.599033
FAS	0.010349	0.989651	2.279426	2.280916	1136.972	498.4661
ERI2	0.010387	0.989613	0.428733	0.428427	456.7893	1066.214
ZNF34	0.010457	0.989543	0.405315	0.404217	124.6112	308.2932
CADM4	0.010539	0.989461	2.273398	2.27451	1483.732	652.3248
LOC10012	0.01059	0.98941	0.262431	0.25502	14.44832	56.68477
ANLN	0.010653	0.989347	2.310401	2.310547	11770.94	5094.432
TLN2	0.010778	0.989222	0.431211	0.431009	704.6337	1634.861
SMIM10L2	0.010778	0.989222	2.570674	2.584002	176.7141	68.38163
LINC0106	0.010819	0.989181	0.288747	0.282911	19.37187	68.4986
DHRS4-AS	0.010916	0.989084	0.402322	0.401071	111.141	277.1256
GBAP1	0.010929	0.989071	0.397813	0.396445	99.39336	250.7266
LINC0113	0.010985	0.989015	2.376897	2.382343	349.5383	146.7146
CCIN	0.011034	0.988966	4.549612	4.798986	38.89934	8.097825
PPFIBP2	0.011085	0.988915	0.414498	0.413647	162.2658	392.2946
IFI30	0.011138	0.988862	0.426166	0.426126	3468.709	8140.113
CCDC71	0.011182	0.988818	0.411661	0.410715	145.5947	354.5048
SLC9A8	0.011199	0.988801	0.422915	0.422329	236.7302	560.5494
PAMR1	0.011218	0.988782	0.20832	0.196717	7.779867	39.58937
SOX5	0.011218	0.988782	0.20832	0.196717	7.779867	39.58937
SQRDL	0.011343	0.988657	2.286911	2.289167	757.9813	331.1111
DNAJC30	0.011347	0.988653	0.387093	0.385345	76.96511	199.7463
GAN	0.011395	0.988605	2.395595	2.40173	311.1947	129.5652
SLC20A1	0.011465	0.988535	0.432145	0.431996	924.6928	2140.525
ZC2HC1C	0.011531	0.988469	0.313614	0.308891	25.56242	82.77776
TINCR	0.011562	0.988438	0.406978	0.405836	122.2551	301.2571
PRKD1	0.011599	0.988401	4.192498	4.376045	43.34497	9.897341
HOXC10	0.011658	0.988342	2.89345	2.924985	100.0269	34.19082
SPATA17	0.011731	0.988269	0.116898	0.095404	2.222819	23.39372

ACTA2	0.011733	0.988267	2.294717	2.297327	649.0632	282.5241
SYTL3	0.011814	0.988186	2.376972	2.382702	332.3115	139.4625
QPCT	0.011843	0.988157	0.37968	0.377645	65.57317	173.6534
MGLL	0.011976	0.988024	2.281806	2.282045	7094.127	3108.665
MYO1E	0.011983	0.988017	2.259175	2.260131	1696.011	750.3984
DGCR6L	0.012029	0.987971	0.432126	0.431838	485.2192	1123.627
TBC1D30	0.01203	0.98797	0.414696	0.413773	152.2631	368.0011
FAM86B1	0.012079	0.987921	0.405544	0.404324	114.3307	282.785
RBM24	0.012101	0.987899	3.026054	3.067414	85.57854	27.89251
KRTAP5-A	0.012183	0.987817	0.181179	0.167173	5.557048	33.29106
BRWD1-IT	0.012236	0.987764	0.038703	0.000694	0	14.39613
NDST3	0.012236	0.987764	0.038703	0.000694	0	14.39613
TSHR	0.012236	0.987764	0.038703	0.000694	0	14.39613
CCDC125	0.01236	0.98764	0.403277	0.401996	106.6953	265.4287
EPHA4	0.012823	0.987177	0.242272	0.233223	11.1141	47.68719
ABCA4	0.012937	0.987063	2.379147	2.385168	311.1947	130.465
LRRC31	0.012956	0.987044	3.062706	3.108567	81.1329	26.09299
SCNN1G	0.013136	0.986864	7.294929	8.618395	23.3396	2.699275
FAM86DP	0.013144	0.986856	0.399836	0.3984	94.82547	238.0311
MFAP5	0.013192	0.986808	2.249838	2.25055	2250.26	999.8654
PLXNA2	0.013266	0.986734	2.289213	2.292086	585.7128	255.5314
IL18	0.013298	0.986702	2.250525	2.251703	1361.477	604.6376
C8orf44-S	0.013383	0.986617	2.613183	2.629414	148.8511	56.60379
EME1	0.013707	0.986293	0.413906	0.41289	135.592	328.4118
APOL3	0.013777	0.986223	2.487454	2.498033	200.0537	80.07849
SLC47A1	0.013794	0.986206	0.198433	0.185511	6.668457	35.99033
TTC26	0.014034	0.985966	0.433091	0.432696	353.8839	817.8713
GRK5	0.014035	0.985965	2.86091	2.892488	98.91545	34.19082
CYP20A1	0.014103	0.985897	0.422375	0.421607	180.6041	428.3839
ESR1	0.014295	0.985705	0.310401	0.305266	23.3396	76.47946
PDLIM5	0.014302	0.985698	2.25615	2.256467	5086.522	2254.191
ELAC1	0.014431	0.985569	0.354763	0.351621	41.12215	116.9686
GNGT2	0.014432	0.985568	3.130315	3.182163	74.46444	23.39372
CFAP36	0.014465	0.985535	0.436136	0.435854	495.6887	1137.294
CSRP2BP	0.014536	0.985464	0.429901	0.429342	254.2905	592.2929
FSBP	0.014619	0.985381	0.383855	0.381797	65.09526	170.5132
CCDC106	0.014621	0.985379	0.415069	0.414043	134.4806	324.8127
GTF2IP20	0.014639	0.985361	0.35992	0.356971	44.11185	123.5908
CYFIP2	0.014697	0.985303	0.437451	0.437219	616.8323	1410.821
LDLR	0.014962	0.985038	2.255809	2.256082	5920.501	2624.235
FGFR2	0.01505	0.98495	0.43802	0.437837	764.6498	1746.44
IL32	0.015126	0.984874	2.235548	2.236305	2080.559	930.3501
ARSB	0.015359	0.984641	2.262282	2.264711	670.4134	296.0205
STX12	0.015466	0.984534	2.232988	2.233883	1752.693	784.5892
ECM1	0.015524	0.984476	3.182807	3.241444	70.0188	21.5942
ZNF33B	0.015559	0.984441	0.424108	0.423325	177.3365	418.9275
LY96	0.015572	0.984428	4.64724	4.935458	35.56511	7.198066
TPI1P2	0.01569	0.98431	0.225865	0.215012	8.891277	41.38888
ADAMTS9	0.015844	0.984156	8.652339	11.06117	20.00537	1.799517
LINC0084	0.015844	0.984156	8.652339	11.06117	20.00537	1.799517
AURKAPS	0.016075	0.983925	11.67594	18.36023	16.69337	0.899758
CYTH4	0.016129	0.983871	8.617902	11.06117	20.00537	1.799517
MEF2B	0.016191	0.983809	0.382642	0.380455	61.1831	160.8318
GLIS1	0.01627	0.98373	11.66092	18.3358	16.67114	0.899758
C5orf56	0.01645	0.98355	2.488894	2.500387	184.494	73.78018
LVCAT1	0.016487	0.983513	6.256109	7.085671	25.56242	3.599033

TUBB4A	0.01667	0.98333	0.43667	0.436268	347.8712	797.3928
SLC6A2	0.016693	0.983307	6.242616	7.085671	25.56242	3.599033
AKR1B10	0.016724	0.983276	2.580375	2.596253	147.184	56.68477
UNC5B	0.016871	0.983129	0.43948	0.439198	497.9115	1133.695
SULT1A2	0.016966	0.983034	0.361948	0.358877	43.25606	120.5496
GLRX2	0.017021	0.982979	0.437983	0.437617	382.3249	873.6653
SIAE	0.017101	0.982899	0.44078	0.440557	640.1719	1453.11
EMP1	0.017368	0.982632	2.229744	2.230161	3744.339	1678.949
SRR	0.017616	0.982384	0.383427	0.381196	60.01612	157.4577
PDE5A	0.017617	0.982383	0.43786	0.437456	346.7598	792.6871
FAM129B	0.017736	0.982264	2.266823	2.26694	14286.06	6301.907
SUMF2	0.017841	0.982159	0.440108	0.440018	1597.096	3629.625
NRAV	0.018008	0.981992	0.411631	0.410342	106.6953	260.0302
FNDC1	0.018246	0.981754	2.403667	2.412049	230.0618	95.37438
TSPAN2	0.018352	0.981648	2.236189	2.238392	729.0847	325.7125
KLLN	0.018461	0.981539	0.30237	0.29656	20.00537	67.48187
LRRC3	0.018573	0.981427	2.378413	2.385871	251.1786	105.2717
IDH1	0.018649	0.981351	0.432942	0.432918	5703.754	13175.16
RGS2	0.018672	0.981328	2.403034	2.411493	227.839	94.47462
EGLN3	0.018709	0.981291	2.222744	2.22315	3816.58	1716.739
MT1X	0.018747	0.981253	2.26297	2.266194	505.6914	223.1401
PPM1M	0.01883	0.98117	0.437515	0.437039	294.5235	673.919
GNG13	0.018897	0.981103	22.09277	1223.551	12.22551	0
NLRP5	0.018897	0.981103	22.09277	1223.551	12.22551	0
TUNAR	0.018897	0.981103	22.09277	1223.551	12.22551	0
SCLY	0.018901	0.981099	0.437966	0.437505	303.4148	693.5247
ALOX5	0.019283	0.980717	5.575702	6.164676	27.78524	4.498792
SYT14	0.019339	0.980661	0.432566	0.431878	206.7222	478.6714
SLC3A2	0.019403	0.980597	0.434857	0.434829	5031.351	11570.89
MEGF6	0.019649	0.980351	2.248251	2.251114	559.039	248.3333
FTL	0.019665	0.980335	0.411113	0.411112	108479.1	263867.6
ASPH	0.019807	0.980193	2.273364	2.273438	22224.86	9775.874
AFG3L2	0.019836	0.980164	0.442357	0.442265	1519.297	3435.277
CT47A1	0.019912	0.980088	21.67086	1221.328	12.20328	0
LGI2	0.020043	0.979957	2.35478	2.361641	265.6269	112.4698
KANK4	0.020081	0.979919	0.171184	0.154504	4.445638	28.82826
MYCBP	0.020215	0.979785	2.206219	2.207259	1457.736	660.4226
PRKXP1	0.020292	0.979708	0.27523	0.267601	14.47055	54.10247
FAM175A	0.020526	0.979474	0.406496	0.404937	87.80136	216.8418
LRRN4	0.020622	0.979378	3.526761	3.626667	52.23625	14.39613
CNTF	0.020816	0.979184	0.041909	0.00074	0	13.49637
LINC0088	0.020816	0.979184	0.041909	0.00074	0	13.49637
KCNE1	0.020938	0.979062	2.553933	2.569797	143.3718	55.78501
GNAO1	0.020955	0.979045	5.116572	5.550112	30.00806	5.39855
CCDC89	0.020961	0.979039	0.268469	0.260193	13.33691	51.28622
ENO2	0.021229	0.978771	2.200974	2.201988	1485.955	674.8187
SLC52A3	0.021237	0.978763	2.490904	2.504075	164.4886	65.68236
ABI3BP	0.02133	0.97867	3.592301	3.70369	50.01343	13.49637
TMCC3	0.021359	0.978641	0.437742	0.437154	242.2873	554.2511
PSMA5	0.021366	0.978634	2.201959	2.20257	2467.329	1120.199
PFKFB2	0.021493	0.978507	0.445625	0.445411	656.8431	1474.704
BSCL2	0.021514	0.978486	0.440881	0.440418	302.9703	687.9282
CDH18	0.021652	0.978348	0.330602	0.325858	26.67383	81.87801
CXCL16	0.021759	0.978241	2.197893	2.198753	1744.913	793.5868
BCDIN3D	0.021774	0.978226	0.409078	0.407581	90.46874	221.9794
CLUAP1	0.021785	0.978215	0.437304	0.436702	232.6069	532.6569

PHF11	0.021854	0.978146	2.380501	2.388745	227.839	95.37438
HLA-H	0.021855	0.978145	3.901609	4.057529	43.00044	10.59016
MARCH4	0.021869	0.978131	6.955969	8.208171	22.22819	2.699275
PTN	0.021869	0.978131	6.955969	8.208171	22.22819	2.699275
PAQR5	0.021903	0.978097	2.332948	2.33934	277.8524	118.7681
CPA6	0.021927	0.978073	3.016207	3.064354	74.46444	24.29347
NBPF3	0.022155	0.977845	2.243548	2.246701	504.7578	224.6607
MEGF9	0.022288	0.977712	2.194478	2.195306	1837.227	836.8832
ZNF93	0.022311	0.977689	2.59504	2.613824	128.8013	49.27076
SPSB2	0.022507	0.977493	0.41736	0.416055	107.8067	259.1304
PLEKHA4	0.022591	0.977409	0.446246	0.445983	535.6994	1201.177
NCS1	0.02268	0.97732	2.198724	2.199226	2991.915	1360.435
EGR1	0.0228	0.9772	0.439992	0.439955	3784.35	8601.689
DTD2	0.022908	0.977092	0.438168	0.437556	228.6836	522.6516
HOXA11-A	0.022979	0.977021	4.504343	4.781268	34.4537	7.198066
TMEM56	0.022982	0.977018	2.192469	2.193589	1355.253	617.819
SYT16	0.022993	0.977007	3.85822	4.011712	43.34497	10.7971
DHFRL1	0.023012	0.976988	0.302092	0.29587	18.89396	63.88284
SUMF1	0.023089	0.976911	0.433794	0.433009	181.1598	418.3876
PLA2G4A	0.023261	0.976739	2.825087	2.858088	90.02418	31.49154
TBC1D19	0.023436	0.976564	2.258465	2.262483	411.2215	181.7512
SIM2	0.023902	0.976098	2.193517	2.194922	1062.508	484.07
LAMP3	0.023924	0.976076	0.444824	0.444758	2156.135	4847.898
SLC29A3	0.023941	0.976059	0.44046	0.439873	243.3987	553.3514
TMEM106	0.023972	0.976028	2.920456	2.96169	80.04372	27.01974
LOC10012	0.02408	0.97592	0.091564	0.062283	1.11141	17.99517
SETDB2	0.024132	0.975868	0.437482	0.436805	206.7222	473.2729
HIF1A	0.024153	0.975847	2.266281	2.266334	30564.87	13486.48
MAMDC2	0.024334	0.975666	2.835323	2.869485	87.80136	30.59178
MAGI2-AS	0.024477	0.975523	0.396042	0.393975	65.57317	166.4553
BBC3	0.024658	0.975342	2.708944	2.734696	103.3611	37.78985
NFU1	0.024928	0.975072	0.447254	0.446918	419.0014	937.5482
SACS	0.024945	0.975055	2.182862	2.183675	1844.94	844.873
IL12RB1	0.025042	0.974958	0.126129	0.103136	2.222819	21.63919
BMP1	0.025185	0.974815	2.203834	2.205981	704.6337	319.4142
NUP210L	0.0255	0.9745	0.363755	0.360335	38.89934	107.971
APOA1	0.025531	0.974469	0.126385	0.103351	2.222819	21.5942
ZNF625-Z	0.025664	0.974336	20.9743	1180.206	11.79206	0
GSN	0.025731	0.974269	2.198358	2.19865	5151.383	2342.971
LOC72973	0.025779	0.974221	4.932847	5.329234	30.10809	5.641485
LIMD2	0.026021	0.973979	2.492981	2.507085	151.1517	60.28381
VSTM1	0.026175	0.973825	2.954873	2.999054	75.57585	25.19323
MAGEH1	0.026225	0.973775	2.329509	2.336522	252.29	107.971
SECTM1	0.026409	0.973591	2.260081	2.264513	366.7652	161.9565
CDK3	0.026522	0.973478	0.30199	0.295546	18.22712	61.69643
LAMB1	0.026543	0.973457	2.252706	2.252765	27315.11	12125.14
IL2RG	0.026607	0.973393	3.395264	3.486086	53.34766	15.29589
DMBX1	0.026688	0.973312	2.406025	2.416267	188.9396	78.189
MORN3	0.026909	0.973091	0.256624	0.247214	11.1141	44.98792
CLU	0.027072	0.972928	0.447152	0.447089	2207.259	4936.974
RASA3	0.027503	0.972497	2.180628	2.182215	942.4753	431.884
TM7SF2	0.027579	0.972421	0.448144	0.447724	341.2027	762.0953
CASP1	0.027602	0.972398	2.314658	2.321311	261.2368	112.5328
HS6ST2	0.027694	0.972306	2.193415	2.195543	701.2994	319.4142
ZNF362	0.027768	0.972232	0.422964	0.421705	110.0295	260.9299
NRG1	0.027778	0.972222	0.446779	0.446296	296.7464	664.9214

HOXC11	0.027893	0.972107	8.152853	10.44697	18.89396	1.799517
NKILA	0.02793	0.97207	2.674719	2.699834	104.4725	38.68961
TMSB10	0.027999	0.972001	2.244014	2.244077	25614.66	11414.33
NHS	0.028004	0.971996	2.294352	2.30029	285.6323	124.1666
CDH5	0.028222	0.971778	2.790433	2.822807	88.91277	31.49154
NUPR1	0.028252	0.971748	0.445557	0.445514	3269.767	7339.328
HSH2D	0.028256	0.971744	2.51491	2.530923	138.9484	54.89425
RRP1B	0.028337	0.971663	0.450052	0.449963	1618.212	3596.334
ANXA2P3	0.028446	0.971554	12.90921	24.89097	14.3483	0.566848
WARS	0.028809	0.971191	0.442123	0.442098	5541.488	12534.53
SNX7	0.028967	0.971033	2.172714	2.174074	1068.065	491.268
PSG4	0.029011	0.970989	0.446067	0.445536	264.5155	593.7145
WDR31	0.029345	0.970655	0.382903	0.380119	48.90202	128.6654
CES1P2	0.02943	0.97057	20.59778	1157.977	11.56977	0
PPP4R1L	0.029493	0.970507	2.515948	2.53194	136.7034	53.9855
MCMDC2	0.029946	0.970054	0.296587	0.289631	16.67114	57.58453
NGFR	0.03015	0.96985	5.357314	5.918178	26.67383	4.498792
ENC1	0.030169	0.969831	2.168805	2.169296	2994.137	1380.229
TRIM10	0.030373	0.969627	10.83812	17.11414	15.55973	0.899758
SERTAD4	0.030468	0.969532	2.440827	2.45332	161.1544	65.68236
SLCO3A1	0.030518	0.969482	0.443495	0.44283	211.1678	476.8719
COBLL1	0.030539	0.969461	0.453635	0.453424	669.0686	1475.604
TP53	0.030562	0.969438	0.453086	0.452963	1172.537	2588.605
SLIT2	0.030676	0.969324	2.685009	2.711019	100.0269	36.89009
POU5F1	0.030711	0.969289	2.605827	2.627455	113.2082	43.08043
ISM2	0.03095	0.96905	2.896863	2.938256	76.68726	26.09299
KCNG3	0.031117	0.968883	0.318776	0.313027	21.11678	67.48187
IL33	0.031464	0.968536	0.409592	0.407778	75.57585	185.3502
CD14	0.031855	0.968145	3.763231	3.908872	42.23356	10.7971
CCDC88B	0.032151	0.967849	2.690288	2.717032	97.80404	35.99033
CHCHD10	0.032208	0.967792	0.452726	0.452639	1611.544	3560.344
CCDC191	0.032559	0.967441	0.332591	0.327501	24.45101	74.67994
PSKH1	0.03264	0.96736	0.452383	0.451989	357.8739	791.7873
DCBLD1	0.032765	0.967235	2.243678	2.248482	337.8685	150.2596
SOBP	0.032784	0.967216	0.043989	0.000793	0	12.59662
PDZD2	0.03291	0.96709	2.155314	2.15605	1928.296	894.3598
ACTN1	0.033244	0.966756	2.233268	2.233322	29265.64	13104.08
FAM198A	0.033366	0.966634	4.006137	4.196234	37.78793	8.997583
NAV1	0.033498	0.966502	2.189617	2.192478	529.031	241.2882
THEM4	0.033564	0.966436	0.437108	0.43613	145.5947	333.8463
ACCS	0.033685	0.966315	0.270338	0.261457	12.22551	46.78743
TSPAN13	0.033707	0.966293	2.1603	2.160734	3287.55	1521.491
ROS1	0.033996	0.966004	4.356803	4.627078	33.34229	7.198066
ZMYND12	0.034049	0.965951	0.044769	0.000793	0	12.59662
SLAIN1	0.034218	0.965782	0.261669	0.252258	11.1141	44.08816
PALD1	0.034492	0.965508	0.407266	0.405243	68.90739	170.0543
ADPRH	0.034506	0.965494	2.191342	2.1943	503.4685	229.4384
UBLCP1	0.034656	0.965344	0.455726	0.455606	1181.428	2593.103
JMJD7-PL	0.034804	0.965196	0.306589	0.299849	17.89369	59.69896
EEFSEC	0.035068	0.964932	0.45455	0.454175	376.7678	829.5772
FLT3LG	0.035203	0.964797	3.326879	3.413473	52.23625	15.29589
C9orf47	0.035258	0.964742	20.34782	1122.412	11.21412	0
ARF4	0.035401	0.964599	2.212628	2.212704	20112.07	9089.358
SNAPC3	0.035489	0.964511	2.146295	2.147052	1887.407	879.0639
NCOA5	0.035712	0.964288	0.455261	0.454881	378.9907	833.1762
GADD45B	0.035792	0.964208	2.14597	2.1468	1688.231	786.3888

PGF	0.035807	0.964193	2.327466	2.335785	212.2792	90.87559
DZIP3	0.03591	0.96409	0.457256	0.457014	583.49	1276.757
ANKRD31	0.036065	0.963935	0.095673	0.065559	1.11141	17.09541
CAPN6	0.036196	0.963804	6.617009	7.797947	21.11678	2.699275
CDK14	0.036196	0.963804	6.617009	7.797947	21.11678	2.699275
ZNF747	0.036379	0.963621	0.409461	0.407467	70.0188	171.8538
TLE4	0.036719	0.963281	6.598645	7.797947	21.11678	2.699275
GLB1L	0.036824	0.963176	0.383825	0.380837	45.56779	119.6679
HOTAIR	0.03749	0.96251	20.17524	1112.41	11.1141	0
LINC0096	0.03749	0.96251	20.17524	1112.41	11.1141	0
KIDINS22	0.037522	0.962478	0.458065	0.457925	1010.271	2206.207
C11orf85	0.037524	0.962476	0.13173	0.107843	2.222819	20.69444
CATIP	0.037524	0.962476	0.13173	0.107843	2.222819	20.69444
ECT2L	0.037524	0.962476	0.13173	0.107843	2.222819	20.69444
GCNT2	0.038408	0.961592	2.14277	2.14328	2788.527	1301.051
STK39	0.038486	0.961514	2.156825	2.158963	677.9598	314.0156
MDFI	0.038679	0.961321	2.264658	2.270817	265.6269	116.9686
TLCD1	0.038748	0.961252	0.419124	0.417414	82.24431	197.0471
NTF3	0.038984	0.961016	2.315159	2.323557	211.1678	90.87559
TET1	0.039388	0.960612	0.420793	0.419125	84.46713	201.5459
MSR1	0.039493	0.960507	3.141351	3.210373	57.7933	17.99517
ICA1L	0.039755	0.960245	0.421881	0.420279	86.37875	205.5408
RHBDL2	0.040009	0.959991	2.844905	2.885881	75.39803	26.11998
NLRX1	0.040067	0.959933	0.456363	0.455923	321.1974	704.5108
MAST4	0.040378	0.959622	2.313772	2.322088	208.945	89.97583
RASGRP3	0.041116	0.958884	0.283049	0.274645	13.33691	48.58695
UAP1	0.04121	0.95879	2.154926	2.155166	5910.476	2742.463
NRP2	0.041304	0.958696	2.32844	2.337482	195.6081	83.67752
ARPC4-TT	0.041642	0.958358	2.586797	2.609145	105.5172	40.43514
XPA	0.041725	0.958275	0.435964	0.43479	118.9208	273.5265
ZNF697	0.042099	0.957901	2.267951	2.274766	245.6215	107.971
EFCAB2	0.042334	0.957666	0.41578	0.413867	73.35303	177.2524
NPC1L1	0.042383	0.957617	5.724161	6.469766	23.3396	3.599033
WNT2B	0.042383	0.957617	5.724161	6.469766	23.3396	3.599033
YPEL2	0.04247	0.95753	2.215492	2.220302	325.643	146.6606
FBXO32	0.042585	0.957415	2.310436	2.31906	204.4994	88.17631
LRP1	0.042909	0.957091	2.136699	2.137087	3626.529	1696.944
RNF144A	0.043697	0.956303	0.449353	0.448558	180.3929	402.174
DHX58	0.044116	0.955884	3.665539	3.806031	41.12215	10.7971
SH3GL3	0.044583	0.955417	2.801628	2.84035	76.68726	26.99275
SFRP1	0.044585	0.955415	2.125139	2.126371	1105.853	520.0603
LRRC10B	0.044674	0.955326	0.257673	0.247232	10.00269	40.48912
RBM45	0.044681	0.955319	0.434936	0.433667	110.0295	253.7318
ZC3H10	0.045312	0.954688	0.419631	0.417831	76.68726	183.5507
ADRA1B	0.045357	0.954643	2.332811	2.342508	183.3826	78.27897
CCDC103	0.045379	0.954621	0.360807	0.356635	31.11947	87.27656
TMEM132	0.045677	0.954323	2.129898	2.130299	3417.584	1604.269
LRRCC1	0.045719	0.954281	0.445636	0.4447	150.0403	337.4094
LACC1	0.045749	0.954251	2.184827	2.188787	380.1021	173.6534
MYO5C	0.046122	0.953878	0.462866	0.462556	464.5692	1004.364
WISP3	0.046289	0.953711	2.59027	2.613677	101.1383	38.68961
COL16A1	0.046313	0.953687	2.212077	2.216995	311.1947	140.3623
IGSF9	0.0467	0.9533	2.375031	2.386793	158.9316	66.58211
PATZ1	0.046797	0.953203	0.463485	0.463216	526.8081	1137.294
HSPB3	0.04704	0.95296	7.718037	9.832766	17.78255	1.799517
TCEB3-AS	0.047165	0.952835	0.415909	0.413969	70.0188	169.1546

TMEM189	0.047402	0.952598	19.27997	1080.179	10.79179	0
SYNE1	0.047431	0.952569	0.452522	0.451799	192.2739	425.5857
ICA1	0.047497	0.952503	0.426291	0.424639	85.57854	201.5459
ARHGDIG	0.047857	0.952143	7.687804	9.832766	17.78255	1.799517
RPGR	0.04794	0.95206	0.349677	0.3448	26.67383	77.37921
PITPNM2	0.048514	0.951486	2.361405	2.372744	162.2658	68.38163
ENPP4	0.048524	0.951476	2.165247	2.16871	423.4471	195.2476
APOL2	0.048554	0.951446	2.114984	2.115684	1894.098	895.2595
FAM114A1	0.04899	0.95101	2.112562	2.113369	1659.279	785.1291
AP4S1	0.048994	0.951006	0.452364	0.451591	182.3156	403.7305
SRPX	0.049118	0.950882	3.304119	3.395222	48.90202	14.39613
RCAN1	0.049227	0.950773	2.115259	2.115801	2526.234	1193.979
LINGO1	0.049262	0.950738	4.218548	4.472889	32.23088	7.198066
FHL1	0.049456	0.950544	2.111717	2.112489	1731.576	819.6798
XDH	0.049512	0.950488	2.238651	2.244874	254.5128	113.3695
EDARADD	0.049576	0.950424	2.527158	2.54729	110.0518	43.1974
SH3PXD2	0.049702	0.950298	2.49961	2.518572	115.5866	45.88767
TMEM71	0.049746	0.950254	4.441848	4.758496	30.00806	6.298308
SLC16A4	0.049786	0.950214	2.208891	2.214124	296.8464	134.064
NXPH3	0.049817	0.950183	2.30994	2.319327	187.8282	80.97825
GLT8D2	0.050003	0.949997	2.199671	2.204569	313.4175	142.1618
IGFBP6	0.050102	0.949898	2.310113	2.319327	187.8282	80.97825
SEPP1	0.050229	0.949771	0.357555	0.353002	28.89665	81.87801
YOD1	0.050447	0.949553	2.114663	2.116088	942.4753	445.3804
HEXDC	0.050631	0.949369	0.434279	0.432842	98.91545	228.5386
CCDC18-A	0.050713	0.949287	0.429465	0.427896	88.66826	207.2323
MATN3	0.050986	0.949014	3.081551	3.148646	56.68189	17.99517
STYX	0.051074	0.948926	2.108686	2.109576	1477.119	700.1919
ZNF346	0.051308	0.948692	0.464254	0.463887	385.6591	831.3767
CD58	0.051493	0.948507	2.285551	2.293854	202.2765	88.17631
KCNG1	0.051545	0.948455	0.464414	0.464049	387.8819	835.8755
TNFRSF1	0.051579	0.948421	2.4718	2.489452	120.4546	48.38
PIGM	0.051651	0.948349	0.443146	0.442019	124.4779	281.6243
LINC0146	0.052059	0.947941	0.04663	0.000854	0	11.69686
OAS3	0.052063	0.947937	2.117947	2.119725	759.0927	358.1038
SULT2B1	0.052534	0.947466	0.411589	0.409303	61.12753	149.3599
NUDT12	0.052821	0.947179	0.460199	0.459631	248.9557	541.6545
ENTPD1	0.053062	0.946938	0.44625	0.445202	134.0027	301.0051
ATP8B3	0.053156	0.946844	2.838346	2.881432	70.0188	24.29347
RNF38	0.053159	0.946841	2.105259	2.106417	1144.752	543.454
NPY4R	0.053213	0.946787	5.077659	5.5928	25.20677	4.498792
CCM2L	0.053362	0.946638	2.228898	2.235071	253.4014	113.3695
ATP10D	0.053577	0.946423	2.124527	2.126675	625.7236	294.221
PAQR9	0.053698	0.946302	10.15837	15.89249	14.44832	0.899758
TYRO3	0.05384	0.94616	2.105698	2.107001	1018.051	483.1702
ZNF736	0.053918	0.946082	2.345079	2.356493	161.1544	68.38163
VAMP5	0.054143	0.945857	2.599113	2.624415	94.46981	35.99033
EHBP1L1	0.054668	0.945332	2.099914	2.100692	1723.796	820.5796
SYT2	0.054907	0.945093	10.09227	15.89249	14.44832	0.899758
WDR17	0.054985	0.945015	0.100804	0.069199	1.11141	16.19565
RPH3AL	0.055229	0.944771	2.24965	2.256977	223.3933	98.97341
LOC10192	0.055481	0.944519	0.048047	0.000854	0	11.69686
TBCK	0.055497	0.944503	0.467101	0.466758	421.2242	902.4576
KLK6	0.055882	0.944118	0.468379	0.468172	682.4055	1457.608
ARHGEF2	0.05618	0.94382	2.266703	2.274642	206.7222	90.87559
KRT14	0.056218	0.943782	6.315335	7.432848	20.12763	2.699275

ADAL	0.056417	0.943583	0.462528	0.461982	258.9584	560.5494
EMX2OS	0.056856	0.943144	0.468683	0.468526	924.7595	1973.773
ZNF134	0.057178	0.942822	2.602359	2.628361	92.247	35.09057
PHTF1	0.057374	0.942626	2.197192	2.202592	283.4094	128.6654
TAPT1-AS	0.05757	0.94243	0.263642	0.25285	10.00269	39.58937
ZNF415	0.05769	0.94231	2.79661	2.837215	71.49698	25.19323
LOC38989	0.059231	0.940769	0.226	0.212004	6.668457	31.49154
DLG1-AS1	0.059731	0.940269	0.226259	0.212004	6.668457	31.49154
APPL2	0.059767	0.940233	0.464942	0.46441	271.1839	583.9431
SOX3	0.059853	0.940147	0.240654	0.227768	7.779867	34.19082
PTGIS	0.060205	0.939795	2.204409	2.210165	263.4041	119.173
EDA	0.060456	0.939544	2.605772	2.632515	90.02418	34.19082
ABHD6	0.060519	0.939481	0.458944	0.458201	190.051	414.7886
DNAJC22	0.060586	0.939414	0.458724	0.457972	187.8504	410.1908
MEIS1	0.061014	0.938986	0.460449	0.459751	202.2765	439.9818
RNASEH1	0.061639	0.938361	0.359693	0.355034	27.78524	78.27897
BMP6	0.062297	0.937703	2.12087	2.1236	496.8001	233.9372
ZNF517	0.062699	0.937301	0.394382	0.391268	43.34497	110.7962
PDCD4	0.063078	0.936922	2.086775	2.08787	1164.879	557.9221
TRPM4	0.063252	0.936748	2.08848	2.089815	970.2606	464.2753
CCDC136	0.063458	0.936542	2.452469	2.470148	115.5866	46.78743
LINC0028	0.063623	0.936377	2.657049	2.687893	82.24431	30.59178
HLA-A	0.064169	0.935831	2.12083	2.120991	8409.036	3964.668
BCKDHB	0.06448	0.93552	0.466017	0.465466	256.7356	551.5788
PECR	0.064492	0.935508	0.471781	0.471512	529.031	1121.999
LRRC37A	0.064686	0.935314	0.341878	0.336219	22.26153	66.23121
KIAA1161	0.064887	0.935113	2.096108	2.097999	692.7749	330.2023
GABPB2	0.065043	0.934957	0.432889	0.431195	82.24431	190.7488
LOC10192	0.065289	0.934711	2.583559	2.609523	90.64657	34.73067
TNFRSF2	0.065323	0.934677	2.089743	2.090159	3176.409	1519.692
TMEM86A	0.065448	0.934552	0.47082	0.47044	380.1021	807.983
DPYSL4	0.066445	0.933555	5.466206	6.161814	22.22819	3.599033
LOC10012	0.067003	0.932997	5.458187	6.161814	22.22819	3.599033
BBOX1	0.067212	0.932788	0.169624	0.148606	3.334229	22.49396
GBA	0.067507	0.932493	0.468781	0.468714	2113.423	4508.995
GTF2H2	0.067526	0.932474	0.457728	0.456839	158.7649	347.5406
TMSB15B	0.067606	0.932394	2.504109	2.524985	102.2497	40.48912
CCDC53	0.067617	0.932383	0.463288	0.462589	202.2765	437.2825
SLC7A8	0.067759	0.932241	0.46506	0.464402	218.9477	471.4734
HKR1	0.067877	0.932123	2.132201	2.135732	390.1048	182.6509
REEP3	0.067968	0.932032	2.080917	2.081458	2372.859	1139.994
FAM122A	0.068126	0.931874	0.449236	0.448054	118.9208	265.4287
RPP25	0.068712	0.931288	0.472722	0.472375	415.6672	879.9636
SLC46A3	0.068824	0.931176	0.419134	0.416836	61.12753	146.6606
VPS13C	0.068988	0.931012	0.47394	0.473764	823.5545	1738.333
COQ4	0.069467	0.930533	0.46226	0.461498	185.6054	402.192
C9orf3	0.0698	0.9302	2.10141	2.103874	535.7994	254.6676
PMP22	0.070096	0.929904	2.360269	2.373722	136.7034	57.58453
TMEM44-/	0.070446	0.929554	0.278815	0.268705	11.1141	41.38888
PTPRR	0.071052	0.928948	2.088279	2.090356	635.7263	304.1183
CCDC134	0.071175	0.928825	2.155033	2.159631	308.9719	143.0616
CDK15	0.071256	0.928744	4.07565	4.318699	31.11947	7.198066
TMCC1-A	0.071477	0.928523	0.329825	0.323176	18.89396	58.48429
NSFP1	0.071827	0.928173	0.163771	0.141613	3.034148	21.48623
HCP5	0.071945	0.928055	2.179032	2.184602	263.4041	120.5676
RTP3	0.072601	0.927399	4.285638	4.582314	28.89665	6.298308

GBE1	0.07272	0.92728	2.084886	2.085209	3994.406	1915.585
DLGAP1-A	0.072829	0.927171	0.423616	0.42147	64.46176	152.9589
CD53	0.073559	0.926441	18.25772	1001.269	10.00269	0
EPB41L3	0.073559	0.926441	18.25772	1001.269	10.00269	0
LINC0099	0.073696	0.926304	2.240313	2.248491	194.2299	86.3768
LOC72855	0.073966	0.926034	2.072025	2.073424	904.7985	436.3738
SRBD1	0.073993	0.926007	0.464011	0.463225	183.3826	395.8937
SIGIRR	0.074182	0.925818	0.464348	0.463572	185.6054	400.3924
TRIM16	0.074516	0.925484	2.086606	2.086883	4675.233	2240.29
ZC3H4	0.074706	0.925294	0.472927	0.472492	325.643	689.2149
RGS9	0.07475	0.92525	2.3307	2.343087	143.3718	61.18356
SEMA3B	0.074954	0.925046	0.473362	0.472942	337.8685	714.4081
RGPD2	0.074989	0.925011	3.061864	3.1328	51.85837	16.54656
CPT2	0.075048	0.924952	0.476195	0.475931	546.8135	1148.946
LOC10192	0.075225	0.924775	0.341338	0.335383	21.11678	62.98308
RECK	0.075779	0.924221	2.123841	2.127636	365.6538	171.8538
ACAP1	0.076273	0.923727	17.94333	1001.269	10.00269	0
MRAP2	0.076273	0.923727	17.94333	1001.269	10.00269	0
SYN2	0.076273	0.923727	17.94333	1001.269	10.00269	0
TCP10L	0.076374	0.923626	2.674106	2.708927	75.57585	27.89251
DACT1	0.077652	0.922348	0.215532	0.199518	5.557048	27.89251
KLHL36	0.077749	0.922251	2.063077	2.063749	1860.6	901.5578
CCDC150	0.077898	0.922102	0.36957	0.365035	28.89665	79.17873
SEPSECS	0.078314	0.921686	0.457739	0.45668	135.592	296.9202
UCHL1	0.078357	0.921643	0.469788	0.469172	230.0618	490.3683
SKAP1	0.078385	0.921615	3.394789	3.513509	41.12215	11.69686
B4GALT1	0.078476	0.921524	2.090125	2.090331	6295.024	3011.491
HMBS	0.078631	0.921369	0.477233	0.477058	809.1062	1696.044
ASS1	0.079071	0.920929	0.471936	0.471877	2398.422	5082.735
GALR2	0.079602	0.920398	7.250886	9.218563	16.67114	1.799517
CD22	0.079637	0.920363	0.143902	0.118108	2.222819	18.89492
PLIN5	0.079637	0.920363	0.143902	0.118108	2.222819	18.89492
ANO2	0.079759	0.920241	0.428032	0.425978	66.68457	156.5579
PIK3R5	0.079794	0.920206	3.116855	3.195634	48.90202	15.29589
STC2	0.079849	0.920151	2.140931	2.140983	26620.48	12433.76
RBAKDN	0.079988	0.920012	3.605552	3.757563	36.67652	9.75338
ANOS1	0.080103	0.919897	2.108258	2.111598	398.996	188.9492
CARD6	0.080122	0.919878	2.200329	2.207257	217.8363	98.68549
MAPK13	0.080453	0.919547	2.076471	2.078566	608.4078	292.7004
IER5	0.080892	0.919108	2.064224	2.06469	2686.277	1301.051
DUSP10	0.081026	0.918974	2.156334	2.161562	272.2953	125.9662
PALMD	0.081272	0.918728	2.120287	2.124258	347.8712	163.756
KCNH7	0.083111	0.916889	0.106517	0.073267	1.11141	15.29589
NLGN1	0.083111	0.916889	0.106517	0.073267	1.11141	15.29589
SH2B3	0.084162	0.915838	2.116232	2.120095	348.9826	164.6018
RFX3-AS1	0.084898	0.915102	0.107122	0.073267	1.11141	15.29589
C10orf54	0.085051	0.914949	2.054305	2.055571	974.7062	474.1726
PLEKHB1	0.085596	0.914404	0.334927	0.328225	18.89396	57.58453
ZNF433	0.086366	0.913634	0.424684	0.422381	60.09392	142.2878
CLDN22	0.086438	0.913562	0.050947	0.000925	0	10.7971
CGRRF1	0.086451	0.913549	0.468826	0.468072	187.8282	401.2922
IFT74	0.086598	0.913402	0.472111	0.471474	222.2819	471.4734
DACT2	0.086768	0.913232	0.477619	0.477183	332.3115	696.4129
DUSP14	0.08678	0.91322	2.049242	2.05016	1333.691	650.5253
CRISPLD2	0.087451	0.912549	2.222915	2.231238	186.7168	83.67752
ZNF85	0.087557	0.912443	2.562629	2.590008	84.18928	32.49927

TMEM40	0.088017	0.911983	2.287173	2.298702	148.9289	64.7826
COL25A1	0.088195	0.911805	2.351031	2.365525	125.5893	53.08574
CHPT1	0.088815	0.911185	0.45347	0.452164	107.8067	238.436
KCNJ11	0.089274	0.910726	0.199918	0.181533	4.490095	24.77934
HSD3B7	0.089449	0.910551	2.366224	2.381957	120.0322	50.38647
HSCB	0.089536	0.910464	0.467456	0.466635	170.0457	364.4201
RRP15	0.089586	0.910414	0.477599	0.477154	314.5511	659.2349
ZNF280A	0.089682	0.910318	2.988165	3.05437	52.23625	17.09541
GALE	0.08973	0.91027	0.480364	0.480219	976.929	2034.354
ZNF25	0.090085	0.909915	2.220085	2.228638	184.494	82.77776
TAP1	0.091269	0.908731	2.044136	2.044805	1852.72	906.0566
ACP2	0.091486	0.908514	0.476809	0.47629	273.4068	574.0458
JAK2	0.091714	0.908286	2.093444	2.096821	386.7705	184.4505
HOXA13	0.091989	0.908011	2.865028	2.918668	57.7933	19.79468
ZSWIM4	0.092752	0.907248	2.046918	2.048445	814.6632	397.6932
PAK6	0.092991	0.907009	0.33133	0.324232	17.71587	54.66032
GATM	0.093205	0.906795	3.655013	3.826076	34.4537	8.997583
TMEM115	0.093446	0.906554	0.476339	0.475755	247.8443	520.9601
MAP1LC3	0.0947	0.9053	2.237446	2.247061	167.8228	74.67994
MUC1	0.094749	0.905251	0.481508	0.481164	413.4444	859.2692
ECE1	0.09481	0.90519	2.083242	2.08338	9342.709	4484.395
NACAD	0.095003	0.904997	2.084374	2.087658	400.1074	191.6485
IPO5P1	0.095275	0.904725	0.470114	0.469302	174.5691	371.9871
COL24A1	0.095653	0.904347	5.939089	6.977499	18.89396	2.699275
MMP10	0.095653	0.904347	5.939089	6.977499	18.89396	2.699275
MGAT3	0.095807	0.904193	0.482074	0.481746	433.4497	899.7583
E2F2	0.09613	0.90387	0.471223	0.470441	181.1598	385.0966
CDC42EP	0.096215	0.903785	2.05005	2.051881	670.18	326.6123
FAM214B	0.096227	0.903773	2.04045	2.041742	936.9183	458.8767
C2orf42	0.096303	0.903697	0.465584	0.46458	143.3718	308.6171
CDK5R1	0.096309	0.903691	2.382078	2.3989	112.2524	46.78743
ZNF564	0.09661	0.90339	0.464283	0.46323	136.7034	295.1207
TRAPPC2	0.097173	0.902827	2.078322	2.081472	413.511	198.6576
SLC41A1	0.097414	0.902586	2.038504	2.039085	2117.235	1038.321
CFAP45	0.097474	0.902526	3.781129	3.976514	32.23088	8.097825
COG8	0.097503	0.902497	0.475777	0.475144	223.9824	471.4104
SP2-AS1	0.097795	0.902205	0.327069	0.319587	16.67114	52.18598
LOC44166	0.098056	0.901944	3.777686	3.976514	32.23088	8.097825
IQCK	0.098268	0.901732	0.473338	0.472623	195.6081	413.8888
LRP4	0.098358	0.901642	2.163279	2.169598	224.5047	103.4722
HEG1	0.098734	0.901266	2.033501	2.034482	1244.779	611.8356
NEIL2	0.098783	0.901217	2.119789	2.124514	286.7437	134.9637
PNCK	0.10036	0.89964	2.241252	2.251385	160.043	71.08091
SLC9B2	0.100516	0.899484	2.509412	2.535026	86.68995	34.19082
LOC10192	0.100641	0.899359	0.253541	0.240418	7.779867	32.3913
DCLRE1A	0.100653	0.899347	0.484132	0.483856	514.5826	1063.514
GCLM	0.101058	0.898942	2.044579	2.044949	3245.349	1587.003
USP46-AS	0.101384	0.898616	0.202035	0.183333	4.445638	24.29347
NAT16	0.101825	0.898175	3.932753	4.164509	30.00806	7.198066
LAMC3	0.102165	0.897835	2.344706	2.359904	118.9208	50.38647
MARK1	0.102492	0.897508	0.20238	0.183333	4.445638	24.29347
CD6	0.102508	0.897492	3.928703	4.164509	30.00806	7.198066
SMOC2	0.102542	0.897458	0.456713	0.455364	104.4725	229.4384
RBPJ	0.103391	0.896609	2.035785	2.036247	2652.935	1302.85
CAPN13	0.104051	0.895949	2.032774	2.034165	860.231	422.8864
RARRES3	0.104165	0.895835	2.208789	2.217648	175.6027	79.17873

CD163L1	0.10421	0.89579	5.192213	5.853862	21.11678	3.599033
CCDC13	0.104774	0.895226	0.222869	0.206166	5.557048	26.99275
ZNF546	0.104774	0.895226	0.222869	0.206166	5.557048	26.99275
ZNF596	0.104966	0.895034	0.307695	0.29871	13.34803	44.70899
BDKRB2	0.105385	0.894615	4.124047	4.406132	27.78524	6.298308
NKX3-2	0.106945	0.893055	4.377163	4.728147	25.56242	5.39855
RYR2	0.107126	0.892874	2.286445	2.299208	134.4806	58.48429
C14orf79	0.10715	0.89285	0.445249	0.443448	77.79867	175.4529
PPFIA4	0.107182	0.892818	2.061047	2.063881	440.1182	213.2427
AGA	0.107463	0.892537	0.459216	0.45787	106.6953	233.0374
RNF122	0.107584	0.892416	2.286679	2.299208	134.4806	58.48429
SLCO1B3	0.10759	0.89241	4.372925	4.728147	25.56242	5.39855
DAAM2	0.107606	0.892394	4.709991	5.178683	23.3396	4.498792
DEPDC1B	0.108063	0.891937	0.48634	0.48615	747.9786	1538.587
TTC30B	0.109133	0.890867	0.373173	0.36825	26.69606	72.51152
PTTG1	0.111405	0.888595	2.05085	2.051053	6049.402	2949.408
UPF3A	0.111805	0.888195	0.487721	0.487475	589.0471	1208.375
TP53RK	0.111933	0.888067	0.487018	0.486687	437.8954	899.7583
LPCAT2	0.112622	0.887378	2.075842	2.079484	349.894	168.2548
LRRC7	0.112976	0.887024	0.348639	0.342177	20.00537	58.48429
PNMA3	0.113041	0.886959	2.376737	2.39452	105.5839	44.08816
ROGDI	0.114053	0.885947	0.475669	0.474872	175.6027	369.8007
PLA2G12A	0.114252	0.885748	0.487624	0.487471	949.1438	1947.086
EBI3	0.114639	0.885361	2.721731	2.766133	62.23894	22.49396
LMOD1	0.115139	0.884861	2.016552	2.017527	1198.1	593.8405
PLAT	0.115147	0.884853	2.810478	2.86255	56.68189	19.79468
MRPL1	0.115157	0.884843	0.487283	0.486934	407.8873	837.675
NMB	0.115218	0.884782	2.809523	2.86255	56.68189	19.79468
GFY	0.116055	0.883945	0.151364	0.12401	2.222819	17.99517
SMAD3	0.116162	0.883838	2.019066	2.019609	2200.591	1089.607
PLSCR3	0.117581	0.882419	2.019783	2.021267	791.3236	391.4938
ZNF583	0.118151	0.881849	0.428881	0.426287	54.45907	127.7657
FAM101B	0.119029	0.880971	2.061324	2.064764	369.6548	179.0249
TNFRSF1	0.11962	0.88038	3.449928	3.590782	35.56511	9.897341
ZSWIM7	0.1207	0.8793	0.463027	0.46168	106.8065	231.3549
IRF1	0.120989	0.879011	2.01223	2.012849	1910.969	949.38
TNIP1	0.121018	0.878982	2.055007	2.055145	8953.516	4356.63
B3GALT6	0.12113	0.87887	0.483272	0.482666	234.5074	485.8695
MCTP2	0.121413	0.878587	2.082519	2.086775	302.3034	144.8611
KIAA0754	0.121425	0.878575	2.010076	2.010965	1301.083	646.9892
COL23A1	0.121706	0.878294	2.513197	2.540558	80.02149	31.49154
MAPK8IP3	0.122459	0.877541	2.012658	2.013997	868.0109	430.9842
RAB38	0.122648	0.877352	0.484617	0.484028	245.6215	507.4637
SLC9A2	0.122736	0.877264	0.49057	0.490332	607.941	1239.867
EPS8	0.123174	0.876826	2.011206	2.012488	903.576	448.9794
LRRC2	0.123252	0.876748	2.732762	2.778447	60.01612	21.5942
PRMT6	0.123329	0.876671	0.480652	0.479926	195.6081	407.5905
PKIA	0.123904	0.876096	0.112917	0.077843	1.11141	14.39613
LGALS1	0.124276	0.875724	2.073082	2.073162	15792.02	7617.354
PRKAR2A	0.124322	0.875678	0.333538	0.326048	16.51555	50.67439
N6AMT2	0.12456	0.87544	0.407614	0.40411	38.89934	96.27414
CORO6	0.12515	0.87485	2.080429	2.084868	294.5235	141.2621
MBNL3	0.125161	0.874839	0.462127	0.460705	101.1383	219.541
METTL7B	0.125909	0.874091	0.398757	0.394834	34.4537	87.27656
WHAMMP	0.126076	0.873924	2.934105	3.00134	49.16876	16.3756
DECR2	0.12624	0.87376	0.446133	0.444163	71.13021	160.157

ALDH3A2	0.126302	0.873698	0.490756	0.490587	839.1142	1710.441
ALOX12B	0.126447	0.873553	0.113554	0.077843	1.11141	14.39613
ZNF311	0.126729	0.873271	0.424195	0.421369	48.90202	116.0688
SLC25A33	0.12685	0.87315	0.491135	0.490907	615.7209	1254.263
SEPW1	0.128073	0.871927	2.033304	2.035889	463.4578	227.6389
PCDH9	0.128195	0.871805	0.305113	0.295552	12.22551	41.38888
DNMBP	0.12837	0.87163	2.021704	2.023811	559.039	276.2258
C4orf48	0.128563	0.871437	2.317498	2.33294	113.3638	48.58695
ZNF446	0.128581	0.871419	0.478199	0.477337	164.4886	344.6074
SURF2	0.128734	0.871266	0.484546	0.483891	221.1705	457.0772
FMNL1	0.128998	0.871002	2.011084	2.012709	713.525	354.5048
SPA17	0.129186	0.870814	0.462752	0.461315	100.0269	216.8418
CCNB3	0.129493	0.870507	0.260654	0.247285	7.779867	31.49154
SMIM3	0.129831	0.870169	2.002198	2.003357	1005.826	502.0651
EGF	0.129933	0.870067	2.51443	2.542619	77.79867	30.59178
LENG9	0.13037	0.86963	0.458459	0.456894	90.02418	197.0471
ZFP90	0.130564	0.869436	0.48626	0.485676	243.3987	501.1654
RNF214	0.130676	0.869324	0.49123	0.49088	406.7759	828.6774
PCDHB2	0.131071	0.868929	2.252421	2.264565	133.0246	58.73622
ACADL	0.131257	0.868743	0.440057	0.437843	62.23894	142.1618
LINC0155	0.131535	0.868465	6.783735	8.604361	15.55973	1.799517
AP1M1	0.132602	0.867398	2.001302	2.001959	1738.245	868.2668
GRIN1	0.132684	0.867316	2.743855	2.791831	57.7933	20.69444
F12	0.132769	0.867231	2.126981	2.133467	211.1678	98.97341
LINC0158	0.132866	0.867134	2.97684	3.050407	46.6792	15.29589
ZNF556	0.132916	0.867084	2.742337	2.791155	57.80441	20.70344
COLGALT	0.133249	0.866751	0.40315	0.399334	35.56511	89.07607
LOC10192	0.133275	0.866725	0.354031	0.347522	20.00537	57.58453
CASC9	0.133506	0.866494	0.325478	0.317268	14.83732	46.78743
COA6	0.134053	0.865947	0.490631	0.490522	1312.575	2675.881
MBD6	0.134078	0.865922	0.49269	0.492362	441.2296	896.1593
KCNC3	0.134134	0.865866	0.490587	0.490479	1327.023	2705.573
LRRC46	0.13443	0.86557	0.246769	0.231873	6.668457	28.79227
DVL3	0.134826	0.865174	0.485287	0.485236	2825.203	5822.336
SOWAHA	0.135615	0.864385	0.465274	0.463866	102.2497	220.4408
GPM6B	0.135676	0.864324	0.323409	0.315012	14.44832	45.88767
KCNH2	0.135717	0.864283	2.107269	2.113062	230.0618	108.8708
YPEL5	0.135879	0.864121	2.00852	2.0089	3033.037	1509.794
KRCC1	0.135939	0.864061	0.493474	0.493256	652.3974	1322.645
CYP1B1	0.136428	0.863572	2.150458	2.158269	184.494	85.47704
SPEF2	0.136792	0.863208	0.209618	0.190382	4.445638	23.39372
PNPLA4	0.136822	0.863178	0.491285	0.490854	330.0886	672.4884
HS1BP3	0.137572	0.862428	0.492445	0.492067	376.7678	765.6943
LOC10028	0.138205	0.861795	0.429948	0.427271	51.12484	119.6679
NDUFAF4	0.138229	0.861771	0.209973	0.190382	4.445638	23.39372
MYO15B	0.138593	0.861407	0.230384	0.213272	5.557048	26.09299
VAV2	0.139767	0.860233	1.996116	1.996713	1897.176	950.1448
FCGR2A	0.140293	0.859707	16.34019	890.1277	8.891277	0
LOC72968	0.140293	0.859707	16.34019	890.1277	8.891277	0
RASGRF2	0.140293	0.859707	16.34019	890.1277	8.891277	0
SLCO1A2	0.140293	0.859707	16.34019	890.1277	8.891277	0
CREB3L1	0.140443	0.859557	2.674365	2.716746	61.12753	22.49396
CRABP1	0.140485	0.859515	2.109674	2.115897	218.9477	103.4722
CCDC122	0.141483	0.858517	0.433661	0.431097	53.51437	124.1487
LTB4R2	0.142107	0.857893	0.056291	0.001009	0	9.897341
PATL2	0.142107	0.857893	0.056291	0.001009	0	9.897341

RPUSD3	0.142562	0.857438	0.4944	0.494098	464.5692	940.2474
FAM174B	0.142653	0.857347	2.026783	2.029647	414.5558	204.2451
PDE1C	0.142748	0.857252	1.997047	1.998583	739.0874	369.8007
MOBP	0.142935	0.857065	16.28267	886.7934	8.857934	0
LOC10050	0.143027	0.856973	2.755929	2.806431	55.57048	19.79468
MYB	0.143145	0.856855	0.397907	0.393719	32.23088	81.87801
CYP4F3	0.143177	0.856823	3.789855	4.010319	28.89665	7.198066
RYR1	0.143403	0.856597	0.47617	0.475108	133.3691	280.7246
RNFT2	0.14342	0.85658	0.351097	0.344364	18.89396	54.88526
CNGB1	0.143756	0.856244	2.054727	2.058649	311.1947	151.1594
ARHGEF1	0.143999	0.856001	1.995018	1.995511	2339.517	1172.385
ATP2C1	0.145036	0.854964	2.032298	2.032439	8653.435	4257.656
KRTDAP	0.145073	0.854927	16.06073	890.1277	8.891277	0
EGFL6	0.145229	0.854771	0.430403	0.427629	50.01343	116.9686
IQCH	0.145592	0.854408	0.379083	0.373912	25.56242	68.38163
AMT	0.145798	0.854202	0.374565	0.369041	24.23984	65.70035
NLRP7	0.145938	0.854062	2.460654	2.486816	80.56608	32.3913
SLC2A9	0.146382	0.853618	2.167407	2.176204	164.4886	75.5797
LINC0135	0.146413	0.853587	0.289371	0.278128	10.00269	35.99033
GBP4	0.14646	0.85354	2.177722	2.186994	158.2203	72.34057
TIGD2	0.1468	0.8532	0.493138	0.492662	304.5262	618.134
NT5C3B	0.146888	0.853112	0.490808	0.490249	254.5128	519.1605
RASSF5	0.147187	0.852813	2.039453	2.043077	340.0913	166.4553
SLC24A1	0.147616	0.852384	0.411495	0.40781	37.78793	92.67511
POLR3B	0.148538	0.851462	0.493863	0.493424	324.5316	657.7233
C1orf116	0.148638	0.851362	2.000454	2.002421	580.1558	289.7222
GK	0.14875	0.85125	0.490099	0.489472	231.1732	472.3011
HEXIM2	0.149176	0.850824	0.483238	0.482375	164.4886	341.0084
KIAA1211	0.149667	0.850333	2.09402	2.099791	226.7276	107.971
FAM155B	0.150242	0.849758	3.962456	4.22995	26.67383	6.298308
MCTP1	0.15055	0.84945	2.065344	2.069995	270.0725	130.465
ANKLE1	0.151323	0.848677	3.957831	4.22995	26.67383	6.298308
CATSPER	0.151529	0.848471	0.334378	0.326429	15.55973	47.68719
KIAA1683	0.151898	0.848102	0.334526	0.326429	15.55973	47.68719
RHNO1	0.152182	0.847818	0.497132	0.496892	594.6152	1196.679
FBN2	0.152467	0.847533	1.99619	1.996545	3188.634	1597.071
DUXAP10	0.152811	0.847189	1.985797	1.98716	818.9866	412.1343
PTPRJ	0.153391	0.846609	1.994865	1.99522	3240.859	1624.307
EVA1A	0.153393	0.846607	2.015878	2.018764	412.333	204.2451
CYP2E1	0.1536	0.8464	2.563515	2.597633	67.79598	26.09299
HS2ST1	0.154754	0.845246	0.494988	0.494884	1393.708	2816.243
HDDC2	0.15499	0.84501	0.496042	0.495644	356.7625	719.8066
LOC10272	0.155034	0.844966	3.041576	3.127676	42.23356	13.49637
MYL7	0.156026	0.843974	2.883427	2.949626	47.79061	16.19565
PLD2	0.156143	0.843857	2.009009	2.011616	443.4524	220.4408
HOXC13	0.156157	0.843843	2.275849	2.290532	113.3638	49.48671
ZFP37	0.156284	0.843716	2.88222	2.949626	47.79061	16.19565
SFXN4	0.156965	0.843035	0.497197	0.496844	402.3303	809.7825
C6orf52	0.157647	0.842353	0.425465	0.422356	44.45638	105.2717
SLC43A1	0.157664	0.842336	0.478993	0.477912	131.1463	274.4263
EEF2KMT	0.157883	0.842117	0.48497	0.484097	162.7326	336.1677
DLX5	0.15883	0.84117	0.460262	0.458467	80.02149	174.5531
PAPPA	0.158887	0.841113	2.305108	2.321941	104.4725	44.98792
TEX264	0.159028	0.840972	0.498186	0.497902	494.5773	993.3332
GSDMC	0.159184	0.840816	4.49114	4.932184	22.22819	4.498792
PRDM1	0.159184	0.840816	4.49114	4.932184	22.22819	4.498792

ZBED6CL	0.15961	0.84039	0.491819	0.491186	224.5047	457.0772
PECAM1	0.159953	0.840047	4.91616	5.54591	20.00537	3.599033
LINC0152	0.160159	0.839841	0.425014	0.42186	43.80065	103.8411
PQLC2L	0.160504	0.839496	0.487604	0.486805	177.8255	365.3019
WNT9A	0.160657	0.839343	4.483762	4.932184	22.22819	4.498792
TRERF1	0.16079	0.83921	2.145732	2.154324	166.7114	77.37921
KCND1	0.160884	0.839116	2.247481	2.260864	120.0322	53.08574
C6orf203	0.161623	0.838377	0.483538	0.482585	148.9289	308.6171
S100P	0.161819	0.838181	2.413549	2.437536	83.35572	34.19082
SEPT4	0.161819	0.838181	2.413549	2.437536	83.35572	34.19082
DDX60L	0.162042	0.837958	1.985903	1.987697	622.3894	313.1159
LOC10050	0.162639	0.837361	15.87999	863.4538	8.624538	0
CCDC40	0.162792	0.837208	0.450027	0.44781	64.46176	143.9613
MAN2B2	0.163685	0.836315	1.980748	1.981217	2358.411	1190.38
KLHDC7A	0.163688	0.836312	0.396938	0.392447	30.00806	76.47946
CALB1	0.164081	0.835919	8.655817	13.44918	12.22551	0.899758
RET	0.164081	0.835919	8.655817	13.44918	12.22551	0.899758
SETBP1	0.166036	0.833964	0.486086	0.485162	156.7088	323.0132
ACY1	0.167147	0.832853	0.500168	0.499894	520.3953	1041.02
TRPM8	0.167869	0.832131	0.483871	0.482881	143.3718	296.9202
BCDIN3D-	0.168684	0.831316	0.441294	0.438731	54.47018	124.1666
COTL1	0.168799	0.831201	2.025836	2.025939	11746.49	5798.043
SIRPB2	0.169071	0.830929	8.585645	13.42475	12.20328	0.899758
HDAC10	0.169103	0.830897	2.004362	2.007201	404.5531	201.5459
SSPO	0.169137	0.830863	2.905541	2.977794	45.56779	15.29589
KIF20A	0.17016	0.82984	1.986855	1.987158	3683.211	1853.502
ADSS	0.170236	0.829764	0.498512	0.498397	1235.887	2479.734
ELK3	0.170297	0.829703	1.96884	1.969907	1019.163	517.361
NIPAL4	0.170716	0.829284	2.626197	2.667358	60.01612	22.49396
ZNF587	0.171855	0.828145	1.970122	1.970697	1930.741	979.7198
SLC35B1	0.172146	0.827854	0.501461	0.501245	670.18	1337.041
FLJ22447	0.172629	0.827371	2.21849	2.231165	124.4779	55.78501
NMNAT1	0.173873	0.826127	0.500982	0.500621	400.7854	800.5869
COL12A1	0.17401	0.82599	1.977368	1.977755	2841.874	1436.914
CACNB3	0.174485	0.825515	1.979845	1.981808	563.4847	284.3236
DUS2	0.175036	0.824964	0.490598	0.489794	176.7141	360.8031
SYBU	0.175424	0.824576	0.49613	0.495512	234.5074	473.2729
LOC28402	0.175497	0.824503	0.442419	0.439839	54.13676	123.0959
SLC30A6	0.176004	0.823996	0.499868	0.499747	1182.229	2365.663
PMS2P7	0.17698	0.82302	0.391605	0.386581	27.16285	70.28012
IGDCC3	0.177623	0.822377	0.192097	0.168861	3.334229	19.79468
COX15	0.177964	0.822036	0.501494	0.501331	862.4538	1720.338
SLC16A12	0.178014	0.821986	2.148209	2.157583	153.3745	71.08091
LRRC28	0.178336	0.821664	0.456021	0.453947	67.79598	149.3599
FSTL3	0.178441	0.821559	1.963048	1.963913	1228.108	625.332
TRIB3	0.17867	0.82133	0.488443	0.488415	5085.81	10412.9
SEPHS2	0.179486	0.820514	0.493576	0.493528	2910.782	5897.916
ZNF606	0.179699	0.820301	0.484793	0.483718	134.4806	278.0253
CES4A	0.179739	0.820261	0.192523	0.168861	3.334229	19.79468
LINC0148	0.179739	0.820261	0.192523	0.168861	3.334229	19.79468
C1orf168	0.180952	0.819048	0.120135	0.083028	1.11141	13.49637
MICB	0.181271	0.818729	1.963242	1.963848	1779.367	906.0566
BIVM	0.181962	0.818038	0.495278	0.494595	208.3115	421.1859
FGF9	0.182582	0.817418	0.365211	0.35873	20.00537	55.78501
NPHP3	0.18289	0.81711	0.502091	0.501708	371.5553	740.5911
SLCO4A1	0.183571	0.816429	1.959201	1.959888	1587.093	809.7825

ERO1A	0.183752	0.816248	2.011358	2.011471	10457.25	5198.803
FCHSD1	0.184003	0.815997	1.963211	1.963747	2007.206	1022.125
PDIK1L	0.184301	0.815699	0.496344	0.495661	212.2792	428.285
TRDMT1	0.185578	0.814422	0.456873	0.454724	66.68457	146.6606
IL15	0.185797	0.814203	2.11713	2.125339	167.834	78.96279
ZNF589	0.18601	0.81399	0.463449	0.461548	75.57585	163.756
SSBP2	0.186551	0.813449	0.473205	0.47166	93.3584	197.9468
C1orf131	0.186884	0.813116	0.498048	0.497435	232.2846	466.9746
MATK	0.187071	0.812929	2.182548	2.19372	132.2577	60.28381
SNHG15	0.188985	0.811015	0.500237	0.499678	258.9584	518.2608
CCDC109	0.189122	0.810878	1.989079	1.9919	397.8846	199.7463
CGN	0.189173	0.810827	0.499657	0.499577	1822.712	3648.52
ZNF688	0.189555	0.810445	0.440571	0.43773	50.01343	114.2693
DES	0.190436	0.809564	2.711103	2.763632	52.23625	18.89492
ZSCAN21	0.190464	0.809536	0.499374	0.498764	237.8417	476.8719
FHAD1	0.191001	0.808999	2.27721	2.293707	101.1383	44.08816
PCDHGB5	0.191498	0.808502	1.95351	1.954571	999.9908	511.6116
FRMD4A	0.191697	0.808303	1.99369	1.996704	370.0994	185.3502
PMS2P5	0.191776	0.808224	0.481796	0.480556	114.3085	237.8781
IFT172	0.191803	0.808197	0.497839	0.497179	215.6135	433.6835
KIAA1147	0.192131	0.807869	1.966141	1.966505	2970.798	1510.694
MAGED4	0.192373	0.807627	1.978288	1.980756	447.3424	225.8393
MAGED4E	0.192373	0.807627	1.978288	1.980756	447.3424	225.8393
MAGIX	0.193032	0.806968	0.454408	0.452151	62.23894	137.663
LOC10013	0.193594	0.806406	0.059294	0.001086	0	9.19553
LOC10013	0.193594	0.806406	0.059294	0.001086	0	9.19553
NR1D1	0.194782	0.805218	2.100245	2.107815	174.4913	82.77776
ASB3	0.195004	0.804996	2.019153	2.023343	280.2975	138.5268
ATCAY	0.195247	0.804753	0.059403	0.001088	0	9.177535
HEY2	0.19608	0.80392	0.37302	0.366819	21.11678	57.58453
NEK3	0.196222	0.803778	0.373136	0.366819	21.11678	57.58453
SH3RF2	0.196675	0.803325	1.948434	1.949363	1155.866	592.9407
LACTB	0.197149	0.802851	1.950457	1.951221	1365.922	700.03
CAPN14	0.197159	0.802841	3.646958	3.85613	27.78524	7.198066
FLJ26245	0.197159	0.802841	3.646958	3.85613	27.78524	7.198066
PKD2	0.198433	0.801567	0.504719	0.504313	351.2054	696.4129
LCP1	0.198755	0.801245	2.037939	2.037986	25746.91	12633.51
IFT46	0.199469	0.800531	0.50317	0.502674	286.7437	570.4468
C11orf70	0.199888	0.800112	0.478488	0.477071	100.0269	209.6797
GPR161	0.200648	0.799352	1.945815	1.946698	1210.458	621.796
LDHA	0.201274	0.798726	2.039168	2.039212	27575.18	13522.47
ANXA9	0.201897	0.798103	0.505899	0.505528	383.4363	758.4963
TMEM123	0.202227	0.797773	2.034361	2.034409	25454.61	12512.04
C1QL4	0.202289	0.797711	0.395199	0.390162	26.67383	68.38163
ESPN	0.202794	0.797206	0.445899	0.443222	52.23625	117.8683
ADCK1	0.202869	0.797131	0.496717	0.495932	181.1598	365.3019
SAMD9	0.20305	0.79695	2.040182	2.04545	230.0618	112.4698
NEDD4	0.203731	0.796269	0.505917	0.505771	972.4834	1922.783
PLEKHA8	0.203968	0.796032	1.968677	1.971161	446.0976	226.3072
ZNF81	0.205108	0.794892	0.498835	0.49809	194.4967	390.4951
BCL2L15	0.206985	0.793015	3.802229	4.054535	25.64022	6.316303
TMEM181	0.207324	0.792676	1.942988	1.943681	1533.745	789.088
PLEK2	0.207341	0.792659	1.963867	1.966152	472.3491	240.2355
ETV5	0.20766	0.79234	1.943314	1.944031	1457.058	749.4987
MDM1	0.207681	0.792319	0.494315	0.493409	156.7088	317.6147
GGACT	0.208136	0.791864	0.420083	0.416146	35.56511	85.47704

CCT6B	0.208892	0.791108	0.276406	0.262267	7.779867	29.69202
CRTAC1	0.209199	0.790801	3.800865	4.053768	25.56242	6.298308
IL31RA	0.209394	0.790606	6.316584	7.990159	14.44832	1.799517
PTPRN2	0.209394	0.790606	6.316584	7.990159	14.44832	1.799517
PCDHGB1	0.209942	0.790058	3.169146	3.285509	34.87603	10.60815
RDH10	0.209955	0.790045	0.507352	0.507194	898.0634	1770.661
TTLL7	0.211726	0.788274	2.525563	2.561318	62.23894	24.29347
FMO4	0.211734	0.788266	2.836824	2.905181	44.45638	15.29589
ZNF252P	0.2118	0.7882	0.506772	0.50664	1082.424	2136.485
CLHC1	0.212156	0.787844	0.331595	0.322398	13.33691	41.38888
DNAH11	0.212156	0.787844	0.331595	0.322398	13.33691	41.38888
GP1BB	0.212391	0.787609	0.060519	0.00111	0	8.997583
MYO7B	0.212391	0.787609	0.060519	0.00111	0	8.997583
PDE4A	0.212641	0.787359	1.946001	1.947585	662.4001	340.1086
N4BP2	0.213213	0.786787	0.495715	0.494821	158.9427	321.2227
GTF2H3	0.214335	0.785665	0.509359	0.50913	632.481	1242.287
FAM24B-(	0.215213	0.784787	14.81822	816.7746	8.157746	0
SLC35B4	0.216058	0.783942	0.509437	0.50919	576.8216	1132.832
CIPC	0.217088	0.782912	0.509407	0.509104	469.0593	921.3525
LOC28458	0.217452	0.782548	1.993249	1.997014	300.0806	150.2596
FKBP7	0.217468	0.782532	2.087856	2.095593	167.8228	80.07849
KDELR3	0.21758	0.78242	1.944692	1.945137	2353.965	1210.175
CPSF2	0.21887	0.78113	0.501149	0.501096	2615.147	5218.868
RPAP2	0.219945	0.780055	0.508751	0.508352	356.7625	701.8115
MASP1	0.220091	0.779909	4.001101	4.317165	23.3396	5.39855
MYOM3	0.220091	0.779909	4.001101	4.317165	23.3396	5.39855
DANCR	0.220963	0.779037	0.510144	0.50993	665.7343	1305.549
TMEM44	0.221265	0.778735	2.023976	2.029218	230.0618	113.3695
CASS4	0.221748	0.778252	3.995712	4.317165	23.3396	5.39855
HLA-DPA1	0.221785	0.778215	2.582394	2.624114	56.68189	21.5942
VEGFB	0.221853	0.778147	0.510332	0.510113	651.286	1276.757
GCFC2	0.222033	0.777967	0.496472	0.495545	153.3745	309.5169
PREPL	0.222493	0.777507	0.500948	0.500899	2911.893	5813.338
LGR5	0.222743	0.777257	0.167057	0.13778	2.222819	16.19565
ST6GAL2	0.222743	0.777257	0.167057	0.13778	2.222819	16.19565
RP9	0.222807	0.777193	0.509952	0.509574	383.2363	752.081
LHX2	0.222843	0.777157	2.736644	2.794474	47.79061	17.09541
SFXN3	0.224564	0.775436	1.9314	1.932558	902.4646	466.9746
SAPCD2	0.226309	0.773691	1.931488	1.93221	1420.381	735.1025
FAM188B	0.226434	0.773566	0.48643	0.485114	107.8067	222.2403
TBX19	0.227262	0.772738	2.146027	2.156854	130.0349	60.28381
ZNF613	0.227423	0.772577	0.494348	0.493309	136.7034	277.1256
FBXL16	0.228373	0.771627	1.966202	1.969157	366.7652	186.25
LRRC23	0.229335	0.770665	0.484992	0.483604	102.2497	211.4432
CHST9	0.229595	0.770405	2.858835	2.932332	42.23356	14.39613
ZSWIM6	0.229692	0.770308	1.967021	1.97014	354.5397	179.9517
SGPP2	0.229868	0.770132	2.044485	2.05084	195.6081	95.37438
RNF34	0.229975	0.770025	0.50924	0.508747	293.4121	576.7451
LOC15476	0.230485	0.769515	3.750772	3.999152	25.21788	6.298308
SPSB4	0.230705	0.769295	0.246719	0.229064	5.557048	24.29347
RNPEPL1	0.232277	0.767723	1.92764	1.928381	1375.925	713.5083
LINC0151	0.232381	0.767619	0.343354	0.334696	14.44832	43.1884
PYCARD	0.232381	0.767619	0.343354	0.334696	14.44832	43.1884
CCNT2-AS	0.232485	0.767515	4.660265	5.237958	18.89396	3.599033
KIAA1549	0.232485	0.767515	4.660265	5.237958	18.89396	3.599033
LINC0052	0.232485	0.767515	4.660265	5.237958	18.89396	3.599033

RNF175	0.232485	0.767515	4.660265	5.237958	18.89396	3.599033
POFUT2	0.232494	0.767506	1.938714	1.939104	2660.715	1372.131
NUDT3	0.232903	0.767097	0.511478	0.511111	387.7263	758.6042
C1orf21	0.23316	0.76684	0.512373	0.512051	450.1209	879.0639
TRIM66	0.233895	0.766105	0.411347	0.406805	30.01917	73.80717
NFIA	0.234171	0.765829	1.939031	1.941043	525.6967	270.8272
S1PR1	0.234369	0.765631	2.180489	2.193372	114.4752	52.18598
TM4SF1-A	0.23476	0.76524	4.650869	5.237958	18.89396	3.599033
ZNF768	0.235211	0.764789	0.505867	0.505799	2093.896	4139.788
SORBS1	0.235493	0.764507	1.956716	1.959554	383.4363	195.6704
SMIM19	0.236294	0.763706	0.50423	0.503472	191.1624	379.698
BDNF-AS	0.237192	0.762808	0.200972	0.176897	3.334229	18.89492
KLK13	0.237192	0.762808	0.200972	0.176897	3.334229	18.89492
LINC0113	0.237273	0.762727	2.439551	2.469918	66.68457	26.99275
COL8A2	0.237367	0.762633	2.278595	2.297769	88.91277	38.68961
LRRK1	0.239108	0.760892	1.980079	1.98379	294.5235	148.4601
EMP3	0.23916	0.76084	1.996207	2.000648	255.6242	127.7657
GARS	0.239299	0.760701	0.490806	0.490792	10509.49	21413.35
MSH5-SAI	0.2393	0.7607	0.418477	0.414237	32.35313	78.11702
C1orf95	0.239819	0.760181	0.227005	0.206239	4.445638	21.5942
ST3GAL5	0.239857	0.760143	0.460213	0.457855	58.90471	128.6654
NFIB	0.240414	0.759586	1.930195	1.930664	2150.578	1113.901
LIAS	0.240803	0.759197	0.503911	0.503135	183.1936	364.1142
AMZ2	0.241631	0.758369	0.510846	0.510734	1295.881	2537.3
TMEM64	0.241635	0.758365	1.947443	1.950044	412.333	211.4432
ALG5	0.241884	0.758116	0.512146	0.511722	335.6457	655.9238
ULBP3	0.242115	0.757885	1.986467	1.990702	269.0945	135.1707
CAPS	0.242629	0.757371	0.508812	0.50822	240.0645	472.3731
GCNT4	0.243201	0.756799	2.074608	2.082517	161.1544	77.37921
NEDD4L	0.24325	0.75675	1.922346	1.923003	1538.191	799.8851
FKBP1B	0.244275	0.755725	2.047504	2.054398	181.1598	88.17631
ZNF84	0.244833	0.755167	0.513692	0.51331	380.1021	740.5011
GDA	0.245857	0.754143	1.967922	1.96805	8467.83	4302.644
ATG14	0.245935	0.754065	1.939366	1.941724	449.0095	231.2379
CDH4	0.246294	0.753706	3.396887	3.565278	28.89665	8.097825
XKR6	0.246294	0.753706	3.396887	3.565278	28.89665	8.097825
OSGEP	0.246475	0.753525	0.507062	0.506346	202.2765	399.4927
ADAMTS2	0.247536	0.752464	3.39392	3.565278	28.89665	8.097825
PLEKHG1	0.247643	0.752357	0.511407	0.510893	276.741	541.6905
GTF2H2B	0.247734	0.752266	0.459886	0.457364	56.83749	124.2836
SIRPB1	0.249052	0.750948	14.32875	787.878	7.86878	0
EPDR1	0.249063	0.750937	2.883661	2.9631	40.01074	13.49637
WNT4	0.249123	0.750877	0.366893	0.359469	17.78255	49.48671
METTL7A	0.249218	0.750782	0.319763	0.309	11.1141	35.99033
CEP85L	0.249769	0.750231	0.438715	0.435332	41.12215	94.47462
NBPF25P	0.25039	0.74961	2.035955	2.042348	188.4951	92.28821
SCRN3	0.250952	0.749048	0.508208	0.507531	210.0564	413.8888
PARP3	0.251399	0.748601	1.963242	1.966649	316.7517	161.0567
RRAS	0.251744	0.748256	1.917775	1.918379	1691.565	881.7631
ANKRD34	0.252737	0.747263	14.42267	778.9867	7.779867	0
CLEC4E	0.252737	0.747263	14.42267	778.9867	7.779867	0
GSG1L	0.252737	0.747263	14.42267	778.9867	7.779867	0
KCNE2	0.252737	0.747263	14.42267	778.9867	7.779867	0
LINC0031	0.252737	0.747263	14.42267	778.9867	7.779867	0
MSC-AS1	0.252737	0.747263	14.42267	778.9867	7.779867	0
PDE10A	0.252737	0.747263	14.42267	778.9867	7.779867	0

FLJ37201	0.253012	0.746988	0.339032	0.329561	13.33691	40.48912
ACSL4	0.25428	0.74572	1.945332	1.945539	5050.245	2595.803
ZNF264	0.25506	0.74494	1.916049	1.917355	765.9613	399.4837
FCHSD2	0.255587	0.744413	1.912771	1.913543	1313.686	686.5156
ZNF667-A	0.256209	0.743791	2.20757	2.223144	100.0269	44.98792
ZNF213	0.25634	0.74366	0.507659	0.506924	193.3853	381.4975
FAM181A	0.257593	0.742407	0.284608	0.27046	7.779867	28.79227
MME	0.257593	0.742407	0.284608	0.27046	7.779867	28.79227
RBM43	0.257668	0.742332	0.46254	0.460106	57.84887	125.7412
PLIN3	0.258813	0.741187	1.923214	1.923609	2565.133	1333.496
CDCP1	0.259848	0.740152	1.939657	1.93988	4644.581	2394.257
LOC38960	0.260174	0.739826	14.17814	778.9867	7.779867	0
PCK1	0.260174	0.739826	14.17814	778.9867	7.779867	0
DNASE1L	0.260417	0.739583	1.910571	1.911399	1196.988	626.2318
SHANK3	0.260856	0.739144	1.939097	1.941825	387.8819	199.7463
PPP1R18	0.260872	0.739128	1.915322	1.915859	1858.277	969.9395
ABTB1	0.262549	0.737451	1.942401	1.945152	374.545	192.5483
SNX29	0.263057	0.736943	0.506598	0.505778	173.3799	342.8079
CSNK2A3	0.26443	0.73557	14.12167	775.6525	7.746525	0
FAM114A	0.264437	0.735563	0.516491	0.516107	370.0994	717.1074
CXCR4	0.264517	0.735483	3.50406	3.70194	26.67383	7.198066
LST1	0.264517	0.735483	3.50406	3.70194	26.67383	7.198066
MGARP	0.264517	0.735483	3.50406	3.70194	26.67383	7.198066
ZFP57	0.264517	0.735483	3.50406	3.70194	26.67383	7.198066
FAT2	0.26455	0.73545	0.506227	0.506183	3238.648	6398.181
DNAJC18	0.264699	0.735301	0.517232	0.51691	441.5741	854.2665
LINC0102	0.264951	0.735049	2.314046	2.336523	77.79867	33.29106
ZFP41	0.265081	0.734919	0.516088	0.51567	340.0913	659.5228
CENPBD1	0.265568	0.734432	0.489243	0.487774	96.54815	197.9468
NT5C1A	0.267648	0.732352	7.904543	12.22753	11.1141	0.899758
ZPLD1	0.267648	0.732352	7.904543	12.22753	11.1141	0.899758
EVPL	0.268339	0.731661	2.2503	2.26905	87.80136	38.68961
EPB41L4E	0.269167	0.730833	0.51535	0.514855	286.7437	556.9504
INSIG2	0.27015	0.72985	1.914694	1.916449	569.0417	296.9202
FAR2	0.270308	0.729692	0.364337	0.356454	16.67114	46.78743
LRRC8A	0.270801	0.729199	1.927812	1.928079	3816.58	1979.468
MIPEP	0.270961	0.729039	0.512322	0.511676	220.0591	430.0845
B3GNT4	0.27145	0.72855	2.673764	2.7295	46.6792	17.09541
PIM1	0.271518	0.728482	1.904971	1.905726	1301.461	682.9166
ZNF594	0.271586	0.728414	0.457289	0.454577	51.12484	112.4788
TAGAP	0.272282	0.727718	7.854709	12.22753	11.1141	0.899758
POF1B	0.273379	0.726621	3.131683	3.254241	32.23088	9.897341
LRRN2	0.273917	0.726083	0.333732	0.323692	12.22551	37.78985
AGRN	0.276307	0.723693	1.975217	1.975288	15840.92	8019.546
FLYWCH2	0.277378	0.722622	0.518913	0.518723	749.0901	1444.112
ZNF395	0.277649	0.722351	1.900809	1.90175	1036.945	545.2535
RAB11A	0.277935	0.722065	1.921681	1.921969	3512.232	1827.409
RPLP0P2	0.278796	0.721204	0.507418	0.506503	158.1425	312.2341
GTF2H2C	0.280468	0.719532	0.507584	0.506665	157.4089	310.6865
LRP12	0.280502	0.719498	1.899966	1.901079	875.7907	460.6763
AMDHD2	0.280597	0.719403	0.499077	0.497871	117.8094	236.6364
METTL20	0.281002	0.718998	0.314269	0.30247	10.00269	33.09311
MIR548XH	0.28269	0.71731	2.580876	2.627553	50.68028	19.28182
IL1A	0.282787	0.717213	1.898997	1.900144	848.0055	446.2801
CLCN4	0.283627	0.716373	0.520341	0.520038	467.9034	899.7583
FGFBP1	0.283673	0.716327	2.784621	2.855184	41.12215	14.39613

ERCC5	0.284204	0.715796	1.903391	1.904942	631.8363	331.6779
AOC2	0.28477	0.71523	2.096769	2.106978	128.9235	61.18356
GAL3ST4	0.285382	0.714618	3.619173	3.853002	24.57327	6.370289
EDNRA	0.2854	0.7146	1.985319	1.990571	216.7249	108.8708
HIST2H4A	0.285592	0.714408	0.340236	0.330526	12.78121	38.68961
HIST2H4B	0.285592	0.714408	0.340236	0.330526	12.78121	38.68961
ZC3H12C	0.285739	0.714261	2.128947	2.140856	115.5866	53.9855
VGLL1	0.285923	0.714077	2.138363	2.150786	112.2524	52.18598
SEMA6B	0.286354	0.713646	1.920706	1.923165	410.1101	213.2427
PHF19	0.286673	0.713327	1.894916	1.89583	1078.067	568.6472
TGS1	0.286706	0.713294	0.519865	0.519689	799.1146	1537.687
C10orf67	0.287039	0.712961	0.373469	0.366125	17.78255	48.58695
UTP20	0.288359	0.711641	0.520971	0.520651	444.5638	853.8706
CHMP1B	0.288419	0.711581	0.521141	0.52085	488.12	937.1703
LOC64516	0.289455	0.710545	2.302346	2.325196	75.47582	32.45428
SLC6A8	0.29034	0.70966	1.896364	1.897761	706.8565	372.4639
TMEM179	0.290577	0.709423	0.5213	0.520939	401.2189	770.1931
CALM3	0.290829	0.709171	1.954242	1.954341	10807.35	5529.915
ADHFE1	0.291093	0.708907	0.255978	0.23787	5.557048	23.39372
OSMR	0.291899	0.708101	1.906192	1.906574	2577.07	1351.671
LRRC8D	0.292568	0.707432	0.514081	0.514015	2145.02	4173.079
HUNK	0.292643	0.707357	0.472874	0.470604	62.23894	132.2645
MRPL40	0.292694	0.707306	0.521827	0.521547	506.8028	971.739
PTGER4	0.292771	0.707229	2.04114	2.048894	156.7088	76.47946
LOC10050	0.293246	0.706754	0.256312	0.23787	5.557048	23.39372
TSPAN18	0.293981	0.706019	2.942526	3.038826	35.56511	11.69686
FAM86B3I	0.294268	0.705732	0.374703	0.367349	17.78255	48.42499
KHNYN	0.29444	0.70556	1.892081	1.892913	1154.755	610.0361
LMO2	0.294777	0.705223	0.391174	0.384856	21.11678	54.88526
OCIAD2	0.29487	0.70513	1.906389	1.906747	2751.85	1443.212
SRGAP1	0.295103	0.704897	0.522184	0.521933	566.8189	1086.008
C19orf66	0.295296	0.704704	1.929746	1.932795	335.6457	173.6534
LONRF3	0.295296	0.704704	1.929746	1.932795	335.6457	173.6534
GJB6	0.295383	0.704617	0.484905	0.483083	77.79867	161.0567
KCNN3	0.295539	0.704461	3.02773	3.136231	33.37563	10.63514
PNP	0.29643	0.70357	0.520696	0.520547	953.5894	1831.908
LRTOMT	0.296724	0.703276	0.462136	0.459338	51.2471	111.579
E2F5	0.297217	0.702783	0.522831	0.522561	534.588	1023.025
STAG3L4	0.298454	0.701546	0.495597	0.494118	97.80404	197.9468
PIK3R1	0.298731	0.701269	0.522784	0.522448	430.1155	823.2788
ERV3-1	0.299238	0.700762	2.067618	2.076806	137.448	66.17722
TSPAN15	0.299715	0.700285	1.909271	1.909568	3329.783	1743.732
UBE2F-SC	0.299797	0.700203	0.065906	0.001216	0	8.214793
SVIL	0.299961	0.700039	1.919154	1.919369	4745.719	2472.536
BORCS7	0.300181	0.699819	0.419879	0.41503	28.80774	69.42535
RAET1G	0.300386	0.699614	3.815189	4.111674	22.22819	5.39855
FIRRE	0.30118	0.69882	13.78989	742.3102	7.413102	0
MKX	0.301317	0.698683	0.523117	0.522829	493.4659	943.8465
ZNF788	0.301828	0.698172	3.192124	3.334501	29.78578	8.925602
SAMD4B	0.302656	0.697344	1.903438	1.903782	2845.209	1494.499
PRX	0.302766	0.697234	1.981511	1.987016	205.6108	103.4722
DLEC1	0.302825	0.697175	0.177083	0.14588	2.222819	15.29589
FJX1	0.303197	0.696803	0.493279	0.491721	91.13559	185.3502
LINC0100	0.303274	0.696726	0.382089	0.375105	18.89396	50.38647
RIBC1	0.303677	0.696323	0.236215	0.215202	4.445638	20.69444
ZNF629	0.305016	0.694984	0.522588	0.522426	893.5733	1710.441

ST3GAL6	0.305075	0.694925	1.925563	1.928755	324.5316	168.2548
ATPAF2	0.30561	0.69439	0.504425	0.503263	122.2551	242.9347
IFITM10	0.305751	0.694249	1.988659	1.9944	195.6081	98.07366
SLC35E3	0.305822	0.694178	0.493454	0.491887	90.56877	184.1355
QTRT1	0.305893	0.694107	0.523723	0.523489	605.7182	1157.089
ZSCAN26	0.306375	0.693625	0.523699	0.523335	397.8846	760.2958
TM6SF1	0.307348	0.692652	2.055307	2.063981	141.149	68.38163
SYDE1	0.307465	0.692535	2.002168	2.008697	178.9369	89.07607
ZBTB43	0.307488	0.692512	1.885806	1.886932	845.5048	448.0796
PRRT2	0.307523	0.692477	2.804703	2.880813	38.89934	13.49637
ZDHHC1	0.308624	0.691376	0.398289	0.392244	22.22819	56.68477
ZNF235	0.308624	0.691376	0.398289	0.392244	22.22819	56.68477
ANK3	0.308698	0.691302	0.524195	0.523951	581.2672	1109.402
SPATA7	0.308791	0.691209	0.438416	0.434436	35.56511	81.87801
CTNNAL1	0.308838	0.691162	1.90847	1.908733	3757.676	1968.671
KLF9	0.309156	0.690844	1.963799	1.968566	226.7276	115.1691
CRIP3	0.310893	0.689107	0.371144	0.363442	16.67114	45.88767
DEPDC4	0.311166	0.688834	0.422016	0.417175	28.89665	69.28139
TAF5	0.31207	0.68793	2.10836	2.120273	114.4752	53.9855
SLC27A2	0.314209	0.685791	4.057957	4.439188	20.00537	4.498792
APOD	0.314573	0.685427	0.066795	0.001233	0	8.097825
ATE1-AS1	0.314573	0.685427	0.066795	0.001233	0	8.097825
NALT1	0.314573	0.685427	0.066795	0.001233	0	8.097825
WBSCR28	0.314573	0.685427	0.066795	0.001233	0	8.097825
XKR4	0.314573	0.685427	0.066795	0.001233	0	8.097825
SYK	0.31522	0.68478	0.42277	0.41793	28.91888	69.20941
DCUN1D3	0.315389	0.684611	1.910983	1.913691	366.7652	191.6485
C8orf46	0.315895	0.684105	4.053439	4.439188	20.00537	4.498792
ZNF430	0.315904	0.684096	1.906687	1.909311	382.8473	200.5111
ASPRV1	0.315987	0.684013	5.849433	7.375956	13.33691	1.799517
ATP12A	0.315987	0.684013	5.849433	7.375956	13.33691	1.799517
GPR83	0.315987	0.684013	5.849433	7.375956	13.33691	1.799517
NXF3	0.315987	0.684013	5.849433	7.375956	13.33691	1.799517
TG	0.315987	0.684013	5.849433	7.375956	13.33691	1.799517
SAMD13	0.316166	0.683834	0.293869	0.279182	7.779867	27.89251
SRD5A1	0.316177	0.683823	1.880267	1.881409	841.3371	447.1799
RAET1L	0.316306	0.683694	5.669852	7.031514	13.72591	1.943478
LOC28434	0.317074	0.682926	0.443599	0.439877	37.44339	85.13513
MID2	0.317144	0.682856	0.501701	0.50037	106.6953	213.2427
LARGE	0.318286	0.681714	4.046987	4.439188	20.00537	4.498792
PLEKHH2	0.319019	0.680981	1.890645	1.892592	502.3571	265.4287
PLCXD2	0.320163	0.679837	2.196475	2.213538	88.40152	39.93127
COL5A2	0.320173	0.679827	0.456508	0.453352	44.45638	98.07366
APPBP2	0.320467	0.679533	0.523655	0.523523	1091.404	2084.74
RASSF1	0.320918	0.679082	1.890681	1.892592	502.3571	265.4287
MAP2K3	0.321359	0.678641	1.881101	1.881722	1520.408	807.983
ZBTB48	0.32159	0.67841	0.518173	0.517434	192.2739	371.6002
RNLS	0.322431	0.677569	0.341746	0.331585	12.22551	36.89009
ITIH4	0.322489	0.677511	0.320959	0.309021	10.00269	32.3913
KCNA5	0.323019	0.676981	0.06795	0.001233	0	8.097825
ATP7A	0.323157	0.676843	0.522281	0.521708	247.8443	475.0724
NPR3	0.324758	0.675242	1.875229	1.876156	1028.054	547.9528
PADI3	0.324912	0.675088	1.933687	1.93755	266.7383	137.663
GAS7	0.325005	0.674995	0.506683	0.505489	118.9208	235.2688
ARL15	0.325211	0.674789	0.517359	0.516565	178.9369	346.4069
LAPTM5	0.325666	0.674334	2.610883	2.664525	45.56779	17.09541

DOCK5	0.325893	0.674107	1.879356	1.880755	673.5142	358.1038
ECM2	0.327066	0.672934	4.394291	4.930005	17.78255	3.599033
AP4B1	0.328762	0.671238	1.927176	1.930961	274.5182	142.1618
LIPH	0.328936	0.671064	2.111173	2.123512	108.9181	51.28622
LINC0162	0.329795	0.670205	4.922209	5.746827	15.55973	2.699275
LOC28333	0.329819	0.670181	0.487015	0.485053	72.24162	148.946
FAM229B	0.329894	0.670106	0.495789	0.494121	86.68995	175.4529
PPARD	0.329945	0.670055	1.880632	1.881132	1887.218	1003.231
KIF26B	0.330423	0.669577	1.944414	1.948842	236.7302	121.4674
ACOX3	0.330765	0.669235	1.913754	1.916915	315.6403	164.6558
HES1	0.330915	0.669085	1.873705	1.874798	853.5626	455.2777
ARHGAP2	0.331413	0.668587	1.958976	1.959031	19264.06	9833.459
DUSP7	0.332989	0.667011	1.872876	1.873652	1200.322	640.6279
LBP	0.334081	0.665919	2.826036	2.9101	36.67652	12.59662
GYLTL1B	0.334981	0.665019	0.516478	0.51559	160.043	310.4166
PHKA1	0.335068	0.664932	2.165393	2.181087	92.247	42.28864
CAMTA2	0.335831	0.664169	1.880633	1.882414	530.1424	281.6243
OPRK1	0.337418	0.662582	0.281229	0.264984	6.668457	25.19323
MPV17L	0.337427	0.662573	0.42632	0.421554	28.89665	68.56158
TMEM236	0.338076	0.661924	3.972387	4.347501	19.98314	4.588767
PPM1E	0.338432	0.661568	2.073646	2.084264	120.0322	57.58453
ITPRIP	0.339358	0.660642	1.869031	1.869986	969.1492	518.2608
MOB3C	0.340372	0.659628	1.90423	1.907196	331.2001	173.6534
VEGFC	0.340429	0.659571	2.355172	2.384743	62.23894	26.09299
AXIN2	0.341004	0.658996	0.464867	0.461921	47.79061	103.4722
ITGAL	0.341208	0.658792	4.855588	5.652959	15.55973	2.744263
PDE6B	0.342422	0.657578	0.482377	0.480151	63.58374	132.4354
CHSY3	0.343352	0.656648	3.027054	3.142061	31.11947	9.897341
RNASET2	0.343399	0.656601	1.889577	1.889856	3433.144	1816.612
SLC38A4	0.343827	0.656173	0.476244	0.473704	56.68189	119.6679
IL10RA	0.344135	0.655865	2.710407	2.778035	40.01074	14.39613
HOXC8	0.344185	0.655815	3.025602	3.142061	31.11947	9.897341
CFAP58	0.345388	0.654612	3.023525	3.142061	31.11947	9.897341
AMIGO3	0.346375	0.653625	0.446945	0.443139	36.67652	82.77776
RELT	0.347822	0.652178	1.865039	1.866252	772.4297	413.8888
HIST2H2A	0.347885	0.652115	0.182714	0.15132	2.233933	14.81902
QSOX2	0.348076	0.651924	1.865375	1.866163	1166.98	625.332
AKR1E2	0.349178	0.650822	0.312863	0.299686	8.891277	29.69202
NABP1	0.350635	0.649365	1.873972	1.874401	2173.917	1159.788
CARNS1	0.35073	0.64927	0.137745	0.095791	1.11141	11.69686
LRRC73	0.35073	0.64927	0.137745	0.095791	1.11141	11.69686
PLA2G4E-	0.35073	0.64927	0.137745	0.095791	1.11141	11.69686
HHLA2	0.351345	0.648655	13.19545	707.8565	7.068565	0
ITPR2	0.351359	0.648641	1.868194	1.869695	615.7209	329.3115
PDE2A	0.351454	0.648546	2.060874	2.071205	121.1436	58.48429
SOWAHB	0.354221	0.645779	1.991268	1.998452	160.043	80.07849
TMEM199	0.355352	0.644648	0.529019	0.528516	286.7437	542.5543
LOC10028	0.35562	0.64438	0.378576	0.370709	16.67114	44.98792
TMEM27	0.35562	0.64438	0.378576	0.370709	16.67114	44.98792
HPCAL4	0.355865	0.644135	1.994427	2.001799	156.7088	78.27897
IFNAR2	0.356008	0.643992	1.867339	1.868931	580.1558	310.4166
INSIG1	0.356612	0.643388	1.863615	1.864247	1452.612	779.1907
SPOPL	0.356844	0.643156	1.860513	1.861271	1225.885	658.6231
CYP4F11	0.357155	0.642845	1.863852	1.864489	1422.604	762.995
DYDC2	0.360241	0.639759	2.85483	2.94389	34.4537	11.69686
ZC3H12B	0.360527	0.639473	3.960236	4.335708	19.46078	4.480796

TMPRSS3	0.360591	0.639409	0.530129	0.529977	946.921	1786.731
LOC10028	0.360693	0.639307	0.280333	0.263459	6.390605	24.28448
ALOX5AP	0.361871	0.638129	2.852074	2.94389	34.4537	11.69686
SLC28A3	0.361995	0.638005	2.264846	2.28839	70.0188	30.59178
TPCN2	0.36335	0.63665	0.504971	0.503485	95.58122	189.849
ATHL1	0.363979	0.636021	1.941917	1.94697	206.7222	106.1715
MGST3	0.364864	0.635136	0.532056	0.531839	654.6202	1230.869
AGAP7P	0.365071	0.634929	4.660855	5.369348	15.79313	2.933212
OAS2	0.365421	0.634579	2.054343	2.064967	118.9208	57.58453
MCCC1	0.365874	0.634126	0.532331	0.532104	622.3894	1169.686
HIST2H2A	0.36657	0.63343	2.0956	2.108261	103.9057	49.27976
HIST2H2A	0.367266	0.632734	2.095765	2.108261	103.9057	49.27976
ME3	0.370243	0.629757	1.902632	1.906159	277.8524	145.7608
MPP7	0.370939	0.629061	0.532123	0.531954	855.7854	1608.768
TTLL1	0.371972	0.628028	0.471471	0.468587	48.90202	104.372
DEDD2	0.372055	0.627945	1.85297	1.854138	785.7666	423.7862
KCCAT21	0.372221	0.627779	3.077756	3.209146	28.89665	8.997583
PTHLH	0.372221	0.627779	3.077756	3.209146	28.89665	8.997583
GJC1	0.372294	0.627706	1.859807	1.86142	565.1962	303.6324
ZNF714	0.372341	0.627659	1.879081	1.881737	361.3859	192.0444
ZNF623	0.372399	0.627601	0.532111	0.531949	890.2391	1673.55
ANKRD26	0.373657	0.626343	0.533675	0.533372	467.9034	877.2643
SPTB	0.374152	0.625848	2.165736	2.182894	84.46713	38.68961
LOC34488	0.374716	0.625284	0.350154	0.339872	12.22551	35.99033
CCDC151	0.37533	0.62467	0.451893	0.448008	36.67652	81.87801
LAT	0.375791	0.624209	0.350345	0.339872	12.22551	35.99033
ZNF804A	0.375791	0.624209	0.350345	0.339872	12.22551	35.99033
ORC5	0.376957	0.623043	0.532013	0.531864	954.7008	1795.018
SALL2	0.377	0.623	0.522964	0.522048	157.8202	302.3188
WDR63	0.377058	0.622942	0.303183	0.288484	7.779867	26.99275
LOC10050	0.37734	0.62266	0.460511	0.457096	41.12215	89.97583
MEOX1	0.377725	0.622275	1.853013	1.854334	682.4055	368.0011
USP51	0.378006	0.621994	0.467774	0.464683	45.56779	98.07366
PAFAH2	0.379268	0.620732	0.532669	0.532199	301.192	565.948
ANKRD44	0.379589	0.620411	2.042057	2.052204	120.0322	58.48429
MYBPC2	0.380187	0.619813	2.21522	2.236399	74.46444	33.29106
WNT5A	0.381333	0.618667	0.533924	0.533518	348.9826	654.1243
PKN2-AS1	0.381375	0.618625	0.247044	0.224979	4.445638	19.79468
EBF4	0.381925	0.618075	1.945282	1.950874	187.8282	96.27414
ZNF780A	0.382012	0.617988	0.533757	0.533302	316.6962	593.8495
CCDC159	0.384428	0.615572	0.463572	0.460243	42.23356	91.77535
FBXO42	0.384765	0.615235	0.533722	0.533556	853.5626	1599.77
SRC	0.385197	0.614803	1.846012	1.847035	885.7934	479.5712
PMVK	0.385917	0.614083	0.535621	0.535373	582.3786	1087.808
EEF1E1-B	0.387092	0.612908	0.534725	0.534316	346.4375	648.3838
PRR19	0.38716	0.61284	2.483768	2.528474	47.79061	18.89492
OSTM1	0.387162	0.612838	0.535307	0.535081	626.835	1171.485
NOCT	0.388684	0.611316	1.857486	1.859404	473.4716	254.6316
HSD17B6	0.388696	0.611304	2.429677	2.469752	51.12484	20.69444
DMTN	0.389024	0.610976	1.874682	1.877503	337.8685	179.9517
NFKBIE	0.391543	0.608457	1.851236	1.852819	560.1504	302.3188
LAYN	0.392081	0.607919	1.843817	1.844808	894.6847	484.9697
FLJ36777	0.392449	0.607551	0.187714	0.154991	2.222819	14.39613
ZNHIT2	0.392517	0.607483	0.516381	0.515118	114.4752	222.2403
LGALS7	0.392787	0.607213	0.071542	0.001328	0	7.521979
TNS4	0.393386	0.606614	1.845032	1.845774	1182.595	640.6999

ZNF782	0.393423	0.606577	0.428861	0.423599	26.67383	62.98308
ACTG2	0.394	0.606	3.629278	3.906182	21.11678	5.39855
GPR173	0.394	0.606	3.629278	3.906182	21.11678	5.39855
ZNF324B	0.39404	0.60596	0.529358	0.528624	193.1074	365.3109
LOC10028	0.394232	0.605768	0.221907	0.195507	3.334229	17.09541
DDX28	0.394379	0.605621	0.523581	0.522615	146.7061	280.7246
DUSP9	0.395131	0.604869	1.977695	1.985063	150.0403	75.5797
PTPRM	0.395289	0.604711	1.861483	1.861792	3005.252	1614.166
COBL	0.395333	0.604667	2.03543	2.04567	117.8094	57.58453
HTATSF1	0.395741	0.604259	2.357785	2.390743	56.09284	23.4567
ZBTB47	0.396747	0.603253	1.918869	1.923637	211.1678	109.7705
ALX4	0.396794	0.603206	0.362698	0.353094	13.33691	37.78985
SPG11	0.398357	0.601643	0.536894	0.536684	689.0739	1283.955
STK10	0.399931	0.600069	1.844542	1.845124	1526.132	827.1118
LINC0142	0.400155	0.599845	0.256515	0.235268	4.767947	20.29855
TRIO	0.400854	0.599146	1.875842	1.876033	4900.205	2611.998
ATP8B1	0.402114	0.597886	1.839383	1.840126	1206.991	655.9238
PIGW	0.402118	0.597882	0.523148	0.522119	137.8148	263.9621
RTEL1-TN	0.40291	0.59709	0.436287	0.431444	28.59657	66.29419
PRH1-TAS	0.403422	0.596578	0.294818	0.278363	6.912968	24.86032
ZNF879	0.403516	0.596484	0.434057	0.428988	27.78524	64.7826
BCL2A1	0.4047	0.5953	7.153268	11.00588	10.00269	0.899758
PHF21B	0.4047	0.5953	7.153268	11.00588	10.00269	0.899758
ANKRD29	0.406468	0.593532	0.504271	0.502498	80.02149	159.2572
GPR75-AS	0.406704	0.593296	0.413646	0.407464	22.00591	54.02149
TFCP2L1	0.407282	0.592718	1.906104	1.910669	220.0591	115.1691
CORO2B	0.407404	0.592596	12.65603	667.8457	6.668457	0
PARS2	0.407664	0.592336	0.532868	0.532198	211.1678	396.7934
TUBA4A	0.408639	0.591361	1.868008	1.86822	4427.622	2369.963
PKI55	0.409303	0.590697	0.480261	0.477533	51.12484	107.0712
CXCL3	0.409749	0.590251	0.491119	0.488801	61.12753	125.0664
STAT5A	0.409763	0.590237	1.877524	1.880905	283.2761	150.6015
PTPRD	0.410382	0.589618	7.108857	11.00588	10.00269	0.899758
FAM86FP	0.410397	0.589603	0.365497	0.355915	13.41471	37.70887
TCF3	0.410445	0.589555	0.536511	0.536368	1012.494	1887.693
CBR3-AS1	0.410625	0.589375	0.527629	0.526694	154.4859	293.3212
UGCG	0.410722	0.589278	1.881881	1.882026	6522.863	3465.869
CLSTN1	0.411917	0.588083	1.895787	1.895888	9545.897	5035.047
PDSS1	0.411947	0.588053	0.528185	0.527294	158.9316	301.419
ANKRD1	0.413288	0.586712	1.908989	1.909062	13889.29	7275.446
TRAPPC8	0.413453	0.586547	0.538485	0.538288	720.1934	1337.941
STRADA	0.413805	0.586195	0.53512	0.53451	232.2846	434.5833
TMEFF1	0.413906	0.586094	0.521949	0.520773	122.7885	235.7907
LINC0015	0.41397	0.58603	1.834358	1.835652	687.2735	374.3984
ADAMTSL	0.414191	0.585809	12.50515	667.8457	6.668457	0
AZGP1P1	0.414191	0.585809	12.50515	667.8457	6.668457	0
CD33	0.414191	0.585809	12.50515	667.8457	6.668457	0
JAZF1-AS	0.414191	0.585809	12.50515	667.8457	6.668457	0
LINC0125	0.414191	0.585809	12.50515	667.8457	6.668457	0
LOC10192	0.414191	0.585809	12.50515	667.8457	6.668457	0
LOC10246	0.414191	0.585809	12.50515	667.8457	6.668457	0
WDFY4	0.414191	0.585809	12.50515	667.8457	6.668457	0
ACSL6	0.414407	0.585593	5.478351	6.881089	12.50336	1.808514
CYB5RL	0.414411	0.585589	0.522681	0.521562	126.7007	242.9347
PARVA	0.414886	0.585114	1.839377	1.839906	1660.446	902.4576
LOC10050	0.415097	0.584903	0.411184	0.404759	21.11678	52.18598

THAP7	0.415517	0.584483	0.536047	0.535443	238.9531	446.2801
LOC14569	0.415908	0.584092	2.381903	2.418338	52.23625	21.5942
SUCLG2-/	0.415985	0.584015	2.381233	2.418338	52.23625	21.5942
PRR5L	0.416063	0.583937	1.846014	1.848096	435.6726	235.7367
SRP14-AS	0.416316	0.583684	0.504815	0.502958	77.83201	154.7584
GTF2IP1	0.416413	0.583587	1.870467	1.87065	5076.152	2713.572
TTC25	0.416589	0.583411	0.374118	0.365115	14.44832	39.58937
EFNA3	0.416747	0.583253	1.893431	1.897529	235.6188	124.1666
CWH43	0.417273	0.582727	3.834589	4.192689	18.89396	4.498792
GAB3	0.417273	0.582727	3.834589	4.192689	18.89396	4.498792
RFX3	0.417731	0.582269	0.536489	0.535918	247.8443	462.4758
NBL1	0.4186	0.5814	1.840417	1.840869	1984.5	1078.018
CYP27A1	0.419974	0.580026	3.828599	4.192689	18.89396	4.498792
TIMP4	0.419974	0.580026	3.828599	4.192689	18.89396	4.498792
TBC1D8B	0.421211	0.578789	1.934819	1.940971	171.1571	88.17631
SPHK2	0.423091	0.576909	0.540404	0.539999	355.6511	658.6231
EID1	0.423332	0.576668	0.539701	0.539511	744.6444	1380.229
CYP3A4	0.423664	0.576336	12.29555	667.8457	6.668457	0
KRTAP3-1	0.423664	0.576336	12.29555	667.8457	6.668457	0
RFPL4AL1	0.423664	0.576336	12.29555	667.8457	6.668457	0
SPANXN3	0.423664	0.576336	12.29555	667.8457	6.668457	0
TRPC1	0.423899	0.576101	1.936515	1.942807	167.8228	86.3768
CRELD2	0.423979	0.576021	0.537399	0.537271	1103.63	2054.148
TNNC1	0.424136	0.575864	0.525491	0.525454	3862.148	7350.126
ALCAM	0.424394	0.575606	1.907759	1.907827	14712.84	7711.828
CPM	0.426369	0.573631	1.832959	1.833523	1542.025	841.0131
SLC7A2	0.426561	0.573439	0.540884	0.540517	385.6591	713.5083
ZNF816-Z	0.426651	0.573349	0.07367	0.00137	0	7.288042
FUCA1	0.426653	0.573347	1.842641	1.84301	2396.199	1300.151
MAP4K4	0.426927	0.573073	1.866833	1.867008	5346.991	2863.931
ITPR1	0.426927	0.573073	0.504878	0.503001	75.57585	150.2596
OXLD1	0.427739	0.572261	0.541151	0.540796	398.996	737.8018
ZNF18	0.428442	0.571558	0.497973	0.495811	65.57317	132.2645
EBAG9	0.428563	0.571437	0.532599	0.531803	175.6027	330.2113
DGAT1	0.430534	0.569466	1.83683	1.837261	2031.657	1105.803
SLC44A4	0.430616	0.569384	0.073923	0.001375	0	7.26105
ZCCHC6	0.430672	0.569328	1.830637	1.831189	1598.207	872.7656
ARID3B	0.431446	0.568554	1.863914	1.867154	288.9665	154.7584
HELQ	0.431689	0.568311	0.521907	0.520657	113.3638	217.7415
VNN2	0.4323	0.5677	3.218266	3.393561	24.45101	7.198066
CCDC73	0.433021	0.566979	0.073628	0.001387	0	7.198066
LINC0020	0.433435	0.566565	0.276752	0.257684	5.557048	21.5942
PSD2	0.434173	0.565827	3.215202	3.393561	24.45101	7.198066
SLC26A9	0.434173	0.565827	3.215202	3.393561	24.45101	7.198066
MUC12	0.434647	0.565353	4.133944	4.622053	16.67114	3.599033
DPYSL2	0.43515	0.56485	1.830297	1.831915	535.6994	292.4214
ODC1	0.435432	0.564568	0.537802	0.537691	1274.787	2370.863
TMPRSS2	0.435697	0.564303	0.516236	0.514706	94.46981	183.5507
ACO1	0.436273	0.563727	1.843104	1.843418	2826.315	1533.188
PCDHGC4	0.436478	0.563522	2.302167	2.332517	56.99308	24.42844
CTH	0.436594	0.563406	0.540739	0.540581	912.4673	1687.947
MOCS3	0.436667	0.563333	0.541334	0.540882	312.0727	576.979
CD248	0.43688	0.56312	4.128317	4.622053	16.67114	3.599033
RASGRP2	0.43688	0.56312	4.128317	4.622053	16.67114	3.599033
SERPINB5	0.43688	0.56312	4.128317	4.622053	16.67114	3.599033
ZNF770	0.437016	0.562984	0.538284	0.538171	1283.678	2385.268

RAB26	0.437647	0.562353	2.004423	2.014204	117.8094	58.48429
TNFSF13	0.43827	0.56173	1.838443	1.840667	402.7193	218.7852
CLSTN2	0.438708	0.561292	0.339328	0.327193	10.00269	30.59178
DGKA	0.439136	0.560864	1.822253	1.823006	1154.755	633.4298
PHOSPHO	0.439897	0.560103	0.462851	0.459045	36.8988	80.3934
AANAT	0.439905	0.560095	0.074522	0.001387	0	7.198066
PRCAT47	0.440033	0.559967	4.120286	4.622053	16.67114	3.599033
CHAC1	0.44077	0.55923	0.542134	0.541943	740.1988	1365.833
C9orf84	0.441652	0.558348	2.64553	2.716237	36.67652	13.49637
STEAP2	0.441703	0.558297	1.840481	1.842724	387.982	210.5434
RNASE4	0.442513	0.557487	1.930226	1.936655	162.6214	83.96545
HNRNPA1	0.443208	0.556792	0.513753	0.512121	86.89	169.6764
HDDC3	0.443883	0.556117	0.529818	0.528798	138.9262	262.7294
SP110	0.444512	0.555488	2.014062	2.024783	111.141	54.88526
FAM26E	0.445223	0.554777	5.382282	6.761754	12.22551	1.799517
SPATA6	0.445425	0.554575	0.313687	0.298428	7.779867	26.09299
AQP11	0.445708	0.554292	0.411154	0.404378	20.00537	49.48671
UNC5CL	0.445874	0.554126	2.485123	2.534577	43.34497	17.09541
SLC22A17	0.445923	0.554077	2.425911	2.469685	46.6792	18.89492
REEP1	0.447701	0.552299	1.913095	1.918707	177.8255	92.67511
NEXN	0.447873	0.552127	1.830317	1.830716	2207.259	1205.676
TMEM198	0.448151	0.551849	0.501409	0.499207	65.57317	131.3647
TMOD3	0.448357	0.551643	1.839908	1.840195	3124.372	1697.844
WIPF1	0.448613	0.551387	1.81672	1.81763	946.921	520.9601
TTC9	0.448674	0.551326	1.846882	1.849631	324.5316	175.4529
C6orf222	0.448922	0.551078	4.583249	5.336603	14.44832	2.699275
HIC1	0.448922	0.551078	4.583249	5.336603	14.44832	2.699275
BVES	0.449154	0.550846	1.947245	1.954465	146.0392	74.71593
PCNX	0.449197	0.550803	0.539762	0.539647	1219.216	2259.293
AK9	0.449293	0.550707	0.430345	0.424711	24.45101	57.58453
ELFN1	0.449496	0.550504	0.0758	0.001387	0	7.198066
LINC0064	0.449496	0.550504	0.0758	0.001387	0	7.198066
LOC10012	0.449496	0.550504	0.0758	0.001387	0	7.198066
SLC26A1	0.449496	0.550504	0.0758	0.001387	0	7.198066
CCL3	0.449722	0.550278	5.362561	6.761754	12.22551	1.799517
TSHZ3	0.449722	0.550278	5.362561	6.761754	12.22551	1.799517
FAR2P1	0.450162	0.549838	0.314429	0.29915	7.779867	26.03001
LIPT2	0.450412	0.549588	0.394141	0.386152	16.67114	43.1884
SOX4	0.451626	0.548374	1.83643	1.836736	2915.227	1587.174
TSEN2	0.451646	0.548354	0.536097	0.535281	173.3799	323.913
KRBOX1	0.452645	0.547355	4.571534	5.336603	14.44832	2.699275
SLC25A21	0.452645	0.547355	4.571534	5.336603	14.44832	2.699275
PITX2	0.453757	0.546243	0.148077	0.103766	1.11141	10.7971
FUT11	0.454035	0.545965	1.940181	1.947182	148.9289	76.47946
PIANP	0.454502	0.545498	2.961709	3.08576	27.78524	8.997583
CD46	0.455487	0.544513	1.85287	1.853054	4900.205	2644.39
TMSB15A	0.456003	0.543997	2.959508	3.08576	27.78524	8.997583
ADAP2	0.456765	0.543235	1.958344	1.966477	134.4806	68.38163
STK26	0.457649	0.542351	1.816225	1.816851	1374.814	756.6967
RBP2	0.457872	0.542128	0.148638	0.103766	1.11141	10.7971
CDK6	0.458058	0.541942	1.856455	1.856616	5744.876	3094.269
TMX4	0.458315	0.541685	0.540464	0.539813	214.2575	396.9194
TTYH1	0.458937	0.541063	2.237525	2.264122	61.12753	26.99275
CFAP70	0.458989	0.541011	0.258042	0.235687	4.445638	18.89492
TYMSOS	0.458989	0.541011	0.258042	0.235687	4.445638	18.89492
HSD17B13	0.459641	0.540359	4.071675	4.555265	16.47109	3.608031

GAS8	0.460045	0.539955	0.53169	0.530646	135.592	255.5314
TRAF2	0.460576	0.539424	1.812239	1.813209	864.6767	476.8719
GMDS-AS	0.460861	0.539139	0.463713	0.459691	35.56511	77.37921
CDH24	0.461809	0.538191	1.817348	1.818926	536.8108	295.1207
ZMYND11	0.461828	0.538172	0.541224	0.541117	1340.36	2477.035
PBXIP1	0.461986	0.538014	1.818658	1.819172	1651.555	907.8561
KCTD21-A	0.462022	0.537978	0.258451	0.235687	4.445638	18.89492
LPAR1	0.462412	0.537588	1.899381	1.904702	183.3826	96.27414
NHLRC3	0.462601	0.537399	1.835496	1.838027	345.6484	188.0495
ANGEL2	0.463076	0.536924	0.53882	0.538045	182.4712	339.1459
LOC10574	0.463535	0.536465	3.316092	3.525223	22.22819	6.298308
PPM1L	0.463639	0.536361	0.149443	0.103766	1.11141	10.7971
TIMP1	0.465031	0.534969	1.826507	1.826862	2422.873	1326.244
TCEANC2	0.465566	0.534434	0.544693	0.544154	266.7383	490.1973
SEMA4B	0.466074	0.533926	1.809945	1.810703	1122.524	619.9335
INTS2	0.466109	0.533891	0.536662	0.535778	160.043	298.7198
RPL17-C1	0.466828	0.533172	1.864318	1.868199	236.9859	126.8479
NIPSNAP3	0.467196	0.532804	2.786314	2.880465	31.11947	10.7971
GAL	0.467926	0.532074	2.089556	2.105229	83.35572	39.58937
LINC0047	0.468472	0.531528	0.38269	0.373604	14.44832	38.68961
CHCHD3	0.469006	0.530994	0.53248	0.532437	3324.226	6243.423
SSR4P1	0.469243	0.530757	0.425116	0.418832	22.22819	53.08574
SLC38A6	0.469603	0.530397	0.541709	0.54099	200.0537	369.8007
MACROD2	0.470124	0.529876	1.818006	1.818465	1822.712	1002.331
ETV4	0.472195	0.527805	1.817501	1.819369	453.4551	249.2331
IL12RB2	0.473166	0.526834	2.100658	2.117244	80.02149	37.78985
TREX1	0.473385	0.526615	1.951726	1.959758	132.2577	67.48187
IQCA1	0.47371	0.52629	0.332461	0.319014	8.891277	27.89251
RAD51B	0.474359	0.525641	0.534153	0.533118	136.7034	256.4311
EPHX4	0.47445	0.52555	1.924094	1.930817	151.1517	78.27897
BCL7A	0.475451	0.524549	0.546289	0.545757	270.0725	494.8671
PTGS1	0.475769	0.524231	1.853359	1.853502	6260.57	3377.693
RNF182	0.475804	0.524196	0.542917	0.542238	207.8336	383.297
RAD52	0.475812	0.524188	0.518634	0.516947	85.57854	165.5555
ITGB7	0.476342	0.523658	2.424578	2.469646	44.45638	17.99517
KIF5A	0.476342	0.523658	2.424578	2.469646	44.45638	17.99517
CLIP4	0.476568	0.523432	0.537574	0.537512	2289.504	4259.456
DNAH17	0.47752	0.52248	2.485909	2.538137	41.12215	16.19565
RAB11FIP	0.478009	0.521991	0.543577	0.543465	1262.561	2323.176
ADGRL3	0.478674	0.521326	0.23331	0.206362	3.334229	16.19565
AKAP3	0.478674	0.521326	0.23331	0.206362	3.334229	16.19565
ARF4-AS1	0.478674	0.521326	0.23331	0.206362	3.334229	16.19565
CCDC173	0.478674	0.521326	0.23331	0.206362	3.334229	16.19565
ATE1	0.479637	0.520363	0.548742	0.548467	511.2484	932.1496
SEMA3A	0.479824	0.520176	1.804714	1.805465	1101.407	610.0361
ATXN1	0.480183	0.519817	1.813807	1.814238	1984.978	1094.106
ARFGEF3	0.480297	0.519703	1.80499	1.80627	646.8404	358.1038
SIX5	0.480941	0.519059	1.81369	1.815663	434.5611	239.3357
MXD3	0.480998	0.519002	1.812147	1.813944	466.792	257.3309
ZNF658B	0.482316	0.517684	0.307317	0.290502	6.935196	23.89758
GIPR	0.482324	0.517676	1.866091	1.870371	215.569	115.25
SEMA3G	0.48318	0.51682	2.030121	2.04266	95.58122	46.78743
STAC	0.483377	0.516623	1.813094	1.815095	427.8927	235.7367
CEP95	0.483586	0.516414	0.547872	0.547394	294.5235	538.0555
MYADM	0.483887	0.516113	1.875243	1.875322	11978.69	6387.537
STEAP3	0.484148	0.515852	1.803149	1.804546	602.384	333.8103

TRIM4	0.484182	0.515818	1.801474	1.802436	854.674	474.1726
ZNF841	0.484191	0.515809	1.802709	1.803814	736.8646	408.4993
TUB	0.484584	0.515416	1.888469	1.893927	178.9369	94.47462
EGR2	0.485032	0.514968	0.199093	0.165316	2.222819	13.49637
FAM227B	0.485032	0.514968	0.199093	0.165316	2.222819	13.49637
ATP6V1E2	0.48515	0.51485	0.450968	0.446141	28.89665	64.7826
GOLGA2	0.486078	0.513922	1.841536	1.84171	5090.467	2763.986
PTPRE	0.486233	0.513767	1.821803	1.824211	354.5397	194.3478
WDR5B	0.486237	0.513763	0.536029	0.534995	136.7034	255.5314
TST	0.486419	0.513581	0.549638	0.549375	536.8108	977.1375
COQ9	0.486509	0.513491	0.54922	0.548996	626.835	1141.793
MORN4	0.489247	0.510753	0.549229	0.548787	318.9745	581.2439
CHRNE	0.489464	0.510536	0.199704	0.165316	2.222819	13.49637
LOC73020	0.489464	0.510536	0.199704	0.165316	2.222819	13.49637
APLN	0.489891	0.510109	2.141234	2.161296	70.0188	32.3913
S100A3	0.489891	0.510109	2.141234	2.161296	70.0188	32.3913
NEK8	0.489911	0.510089	0.438456	0.43276	24.75109	57.20663
FAT4	0.49002	0.50998	1.829802	1.832657	303.4148	165.5555
OXCT1	0.490053	0.509947	0.549516	0.549303	661.2887	1203.877
PDGFRB	0.490335	0.509665	3.010154	3.154042	25.56242	8.097825
MEIS3	0.491321	0.508679	2.089243	2.105751	79.13236	37.57391
RARRES2	0.491429	0.508571	0.540628	0.539717	157.8202	292.4214
SHISA9	0.491957	0.508043	0.550519	0.550278	594.6264	1080.601
LINC0033	0.492445	0.507555	0.496794	0.494139	53.34766	107.971
CPNE7	0.492701	0.507299	1.908604	1.915188	153.3745	80.07849
KATNAL2	0.492768	0.507232	0.455433	0.450775	30.00806	66.58211
NAB2	0.493147	0.506853	0.548848	0.548682	866.8995	1579.976
ARSD	0.494138	0.505862	0.54884	0.548672	840.2256	1531.389
CACNG4	0.494675	0.505325	0.546936	0.546312	225.6161	412.9891
BAHCC1	0.494677	0.505323	3.443366	3.700691	20.00537	5.39855
BRCAT54	0.494677	0.505323	3.443366	3.700691	20.00537	5.39855
CLDND2	0.494677	0.505323	3.443366	3.700691	20.00537	5.39855
GLP2R	0.494677	0.505323	3.443366	3.700691	20.00537	5.39855
LOC38828	0.494677	0.505323	3.443366	3.700691	20.00537	5.39855
VILL	0.494677	0.505323	3.443366	3.700691	20.00537	5.39855
DNAL1	0.498072	0.501928	0.55105	0.550793	540.2673	980.8985
EPN3	0.498263	0.501737	0.532169	0.530909	112.2524	211.4432
LOC14578	0.499067	0.500933	0.402413	0.394366	16.67114	42.28864
LAGE3	0.499602	0.500398	0.551583	0.551228	396.7732	719.8066
AADAT	0.499612	0.500388	0.459749	0.455165	31.11947	68.38163
UBE2F	0.500424	0.499576	1.79303	1.793963	884.682	493.1395
THOP1	0.500873	0.499127	0.55221	0.551917	490.1316	888.0614
H19	0.501141	0.498859	0.349578	0.337105	10.00269	29.69202
NRM	0.501706	0.498294	0.544568	0.543784	180.0484	331.1111
C6orf99	0.501761	0.498239	2.176571	2.199839	63.35035	28.79227
FNDC5	0.502056	0.497944	2.813439	2.9177	28.89665	9.897341
GDAP1	0.502513	0.497487	0.507199	0.504952	62.23894	123.2669
SLC3A1	0.502658	0.497342	0.349809	0.337105	10.00269	29.69202
CD82	0.502942	0.497058	1.792901	1.793883	825.2772	460.0464
DOLK	0.503164	0.496836	0.551455	0.550989	307.8605	558.7499
DBNDD1	0.503789	0.496211	1.797184	1.798709	535.6994	297.82
ZNF507	0.504065	0.495935	0.548738	0.548602	1038.057	1892.192
GCLC	0.506249	0.493751	0.54935	0.549205	963.5921	1754.529
ZNF681	0.5069	0.4931	2.053738	2.068286	84.20039	40.70507
PSD3	0.507218	0.492782	1.791325	1.792438	725.7505	404.8912
CCDC107	0.507736	0.492264	1.817935	1.820632	314.5289	172.7536

ENDOU	0.508303	0.491697	2.422242	2.469603	42.23356	17.09541
ERCC4	0.508359	0.491641	0.552806	0.552544	537.9334	973.5655
BBOF1	0.508465	0.491535	0.496911	0.494141	51.12484	103.4722
KRT23	0.509138	0.490862	1.798021	1.799757	471.2377	261.8297
MFAP2	0.50935	0.49065	2.028393	2.041677	90.02418	44.08816
TMEM245	0.509896	0.490104	1.834868	1.835024	5719.358	3116.772
DNAH2	0.51008	0.48992	2.487487	2.542115	38.89934	15.29589
CACNA2D	0.510496	0.489504	2.486785	2.542115	38.89934	15.29589
PROC	0.510496	0.489504	2.486785	2.542115	38.89934	15.29589
LRRC6	0.510912	0.489088	2.567829	2.633949	35.56511	13.49637
PCSK1N	0.512388	0.487612	0.51184	0.509679	65.57317	128.6654
TRANK1	0.512451	0.487549	0.543494	0.542594	156.7088	288.8224
SEMA5B	0.513307	0.486693	0.324634	0.309082	7.779867	25.19323
MSX2	0.513896	0.486104	0.552433	0.551921	280.0752	507.4637
GPRC5B	0.514157	0.485843	1.794509	1.795038	1535.968	855.6701
ERAP2	0.514467	0.485533	1.885116	1.890951	163.3772	86.39479
PKP2	0.514494	0.485506	0.543731	0.543663	2116.124	3892.354
WDR35	0.514568	0.485432	0.546819	0.546046	182.2712	333.8103
ZNF786	0.514747	0.485253	0.542175	0.541205	145.5947	269.0277
LINC0068	0.515035	0.484965	0.531238	0.529835	100.8271	190.3079
ZNF853	0.515184	0.484816	0.531667	0.530242	101.1383	190.7488
ZMYM6NE	0.515646	0.484354	1.797719	1.799611	432.3383	240.2355
ZFP62	0.516059	0.483941	0.553294	0.553078	652.3974	1179.583
DTWD2	0.516512	0.483488	0.550497	0.549903	233.7294	425.0458
EBLN3	0.516959	0.483041	1.801493	1.80187	2180.586	1210.175
SSPN	0.518219	0.481781	1.807863	1.810417	332.3115	183.5507
EPHB1	0.519359	0.480641	2.082461	2.099588	75.57585	35.99033
UFSP2	0.519446	0.480554	0.55444	0.554068	377.8793	682.0168
ALOX12P2	0.520006	0.479994	2.24891	2.27988	53.34766	23.39372
SMIM5	0.520111	0.479889	0.438361	0.432436	23.3396	53.9855
ARID2	0.520516	0.479484	0.551733	0.551584	946.921	1716.739
SLC6A15	0.520907	0.479093	0.457353	0.452424	28.89665	63.88284
SLX4IP	0.520907	0.479093	0.457353	0.452424	28.89665	63.88284
PCMTD1	0.522799	0.477201	1.785547	1.786222	1206.991	675.7185
TGIF2-C2	0.523276	0.476724	1.936269	1.945015	120.8324	62.11931
MSX1	0.523317	0.476683	1.843625	1.84791	211.1678	114.2693
HJURP	0.523532	0.476468	1.791163	1.791653	1677.206	936.1175
AKR1C3	0.523793	0.476207	1.78603	1.787412	579.0222	323.94
SMPD2	0.524373	0.475627	0.544611	0.543678	151.1517	278.0253
CASQ1	0.524624	0.475376	3.615739	3.946191	17.78255	4.498792
FAM43B	0.524624	0.475376	3.615739	3.946191	17.78255	4.498792
LRRC4C	0.524624	0.475376	3.615739	3.946191	17.78255	4.498792
AKAP7	0.524995	0.475005	0.487373	0.483964	42.23356	87.27656
ZNF429	0.525197	0.474803	3.352541	3.593098	20.00537	5.560506
PERP	0.525702	0.474298	1.806366	1.806649	2934.121	1624.064
DLGAP5	0.52641	0.47359	1.807905	1.808169	3216.419	1778.822
HPS1	0.526454	0.473546	0.555436	0.555177	543.4793	978.937
TATDN3	0.526466	0.473534	0.532945	0.531497	99.46004	187.1407
DHRS4L1	0.526483	0.473517	0.081143	0.001494	0	6.685204
RAB11FIP	0.526912	0.473088	1.797171	1.797552	2113.901	1175.984
LOC10019	0.527292	0.472708	0.508651	0.506249	58.98251	116.5187
BMP2	0.528219	0.471781	2.166842	2.191112	61.12753	27.89251
C4orf26	0.528687	0.471313	4.322273	5.002681	13.90373	2.771256
TLR1	0.52881	0.47119	2.466889	2.522509	38.59925	15.29589
ZNF784	0.528985	0.471015	0.548665	0.547843	174.4913	318.5144
RRS1	0.529021	0.470979	0.554671	0.554469	687.9625	1240.767

ANGPTL2	0.53027	0.46973	0.477895	0.474052	36.67652	77.37921
AKAP6	0.530331	0.469669	0.489925	0.486664	43.34497	89.07607
PALB2	0.531216	0.468784	0.556038	0.555809	624.6122	1123.798
FOXF2	0.531489	0.468511	1.888453	1.894844	150.0403	79.17873
WNT11	0.531664	0.468336	2.319532	2.357483	46.6792	19.79468
PER2	0.532758	0.467242	0.536923	0.535628	109.096	203.6873
ABHD4	0.53348	0.46652	1.778904	1.779829	853.5626	479.5712
VANGL2	0.533958	0.466042	1.785774	1.787472	471.2377	263.6292
CRIM1	0.53405	0.46595	1.85442	1.854496	11862.07	6396.382
CAPN5	0.53443	0.46557	1.779316	1.78052	656.8431	368.9009
ENPP2	0.534431	0.465569	0.426611	0.419634	20.00537	47.68719
GPAT3	0.534649	0.465351	0.550143	0.549375	183.3826	333.8103
NIPBL-AS	0.534761	0.465239	0.401786	0.393181	15.55973	39.58937
SEC13	0.535806	0.464194	1.817095	1.817281	4486.76	2468.937
ARHGEF2	0.535979	0.464021	1.789821	1.790243	1907.179	1065.314
POLH	0.536626	0.463374	1.796232	1.796565	2445.879	1361.415
SLC25A27	0.536836	0.463164	1.953959	1.964045	107.8067	54.88526
HLA-DOB	0.536877	0.463123	2.364744	2.407919	43.34497	17.99517
EXT2	0.538563	0.461437	1.793751	1.794102	2308.398	1286.654
GOLGA6L	0.539258	0.460742	0.37426	0.363338	11.84763	32.62524
TMEM249	0.53959	0.46041	2.843591	2.962374	26.67383	8.997583
TPM1	0.539629	0.460371	1.871137	1.871187	18822.83	10059.3
LINC0045	0.539863	0.460137	11.20123	592.2699	5.912699	0
KCNJ4	0.540922	0.459078	0.270541	0.247464	4.445638	17.99517
NFIX	0.541524	0.458476	1.783272	1.783784	1535.968	861.0687
ZNF554	0.542153	0.457847	0.539095	0.537788	110.0295	204.605
SPTBN5	0.543059	0.456941	0.378256	0.367421	12.22551	33.29106
P2RY11	0.543417	0.456583	2.710129	2.802652	29.49681	10.51817
RTN4RL1	0.543626	0.456374	0.454499	0.449268	26.67383	59.38405
CTF1	0.543934	0.456066	0.54036	0.539127	114.4752	212.343
HELZ2	0.544629	0.455371	1.784331	1.784779	1777.144	995.7175
OSER1-AS	0.544895	0.455105	0.542511	0.541357	122.2551	225.8393
RMI1	0.545283	0.454717	0.558245	0.557853	357.8739	641.5277
KIF5C	0.54579	0.45421	0.473369	0.46915	33.34229	71.08091
LY6K	0.545974	0.454026	2.574478	2.645618	33.34229	12.59662
C8orf58	0.546198	0.453802	0.53364	0.532126	93.3584	175.4529
GEMIN8	0.546783	0.453217	0.540818	0.539549	113.2638	209.9316
C17orf97	0.547294	0.452706	0.411041	0.402937	16.67114	41.38888
DHRS7	0.547726	0.452274	0.557282	0.557086	705.7451	1266.86
LHX4-AS1	0.547921	0.452079	0.411191	0.402937	16.67114	41.38888
FAM186B	0.548326	0.451674	0.314128	0.296768	6.668457	22.49396
RBM5	0.548524	0.451476	0.556573	0.556407	843.5599	1516.093
ATP6V0E2	0.548673	0.451327	0.559416	0.55908	424.5585	759.396
TIMM9	0.548993	0.451007	0.556525	0.556361	858.0082	1542.186
SMN2	0.549498	0.450502	1.772052	1.773379	598.6497	337.5713
LOC10192	0.549699	0.450301	1.847697	1.852759	180.0484	97.1739
FBLIM1	0.550954	0.449046	1.827688	1.827808	7216.727	3948.292
TNFRSF1	0.55163	0.44837	3.862343	4.314101	15.55973	3.599033
TSKS	0.55163	0.44837	3.862343	4.314101	15.55973	3.599033
PTX3	0.551919	0.448081	1.77025	1.771212	807.9948	456.1775
COQ2	0.552541	0.447459	0.544825	0.543712	126.7007	233.0374
ZNF83	0.552715	0.447285	1.769812	1.770608	993.3334	561.0083
SPR	0.553867	0.446133	1.770581	1.771965	572.3759	323.0132
XKR8	0.554016	0.445984	1.795229	1.797829	308.9719	171.8538
GLCE	0.55427	0.44573	1.769184	1.770218	750.2015	423.7862
IQGAP3	0.554612	0.445388	1.800848	1.801075	3615.415	2007.361

SNX31	0.554813	0.445187	3.854995	4.314101	15.55973	3.599033
MBOAT2	0.555396	0.444604	1.771191	1.772573	563.5291	317.9116
XYLB	0.555585	0.444415	0.530114	0.528395	82.24431	155.6582
CEBPA	0.555801	0.444199	0.55381	0.553053	185.6054	335.6098
DUS4L	0.555831	0.444169	0.560113	0.559857	560.1504	1000.531
PC	0.555937	0.444063	0.560063	0.559695	381.2135	681.117
DIRC3-AS	0.557754	0.442246	6.401993	9.784221	8.891277	0.899758
HBE1	0.557754	0.442246	6.401993	9.784221	8.891277	0.899758
WTAPP1	0.557754	0.442246	6.401993	9.784221	8.891277	0.899758
VWA8	0.557786	0.442214	0.560467	0.560135	422.3356	753.9975
OVGP1	0.559066	0.440934	3.154501	3.349041	21.11678	6.298308
PNPO	0.559131	0.440869	0.560367	0.560073	471.4933	841.8499
LIMA1	0.5598	0.4402	1.812909	1.813064	5394.782	2975.501
LINC0034	0.560188	0.439812	0.44571	0.439764	23.3396	53.08574
GPD2	0.560209	0.439791	0.552892	0.552802	1559.308	2820.742
RNF169	0.560671	0.439329	1.77029	1.770878	1344.806	759.396
MAP1B	0.560975	0.439025	1.819253	1.819384	6466.181	3554.045
ASCC2	0.56171	0.43829	0.553807	0.553712	1507.071	2721.769
PSG8	0.562431	0.437569	0.382889	0.372299	12.34776	33.18309
VPS9D1	0.562612	0.437388	0.533037	0.531386	85.57854	161.0567
DCPS	0.563016	0.436984	0.561262	0.560918	406.7759	725.2052
APAF1	0.563145	0.436855	1.763849	1.76485	781.3209	442.7081
ENTPD5	0.563158	0.436842	0.542401	0.541119	110.0295	203.3454
LINC0060	0.56356	0.43644	6.363004	9.784221	8.891277	0.899758
MKRN2OS	0.56356	0.43644	6.363004	9.784221	8.891277	0.899758
CLIC3	0.564309	0.435691	1.765619	1.766868	616.8323	349.1062
PTGR2	0.565041	0.434959	0.561393	0.561008	363.4309	647.826
MBOAT1	0.565128	0.434872	0.561512	0.561225	488.1978	869.8863
SMIM24	0.565939	0.434061	0.530145	0.528353	78.91008	149.3599
DLG4	0.566215	0.433785	1.776453	1.778492	385.6591	216.8418
FEM1A	0.566413	0.433587	0.561228	0.560987	583.49	1040.121
RBM14-RI	0.5665	0.4335	0.347099	0.333188	8.780136	26.37192
LOC10527	0.567144	0.432856	0.246533	0.218493	3.334229	15.29589
ZNF620	0.567444	0.432556	0.538677	0.537217	96.69263	179.9966
TLCD2	0.567818	0.432182	0.561249	0.561007	572.0425	1019.678
ARL10	0.567936	0.432064	0.083271	0.001585	0	6.298308
SUGT1P3	0.567936	0.432064	0.083271	0.001585	0	6.298308
HORMAD1	0.568351	0.431649	0.427356	0.420108	18.89396	44.98792
TMEM209	0.568422	0.431578	0.559518	0.559356	886.8604	1585.509
ZNF155	0.568509	0.431491	1.827394	1.831832	194.4967	106.1715
AUTS2	0.568792	0.431208	0.161404	0.11319	1.11141	9.897341
RAB39B	0.568792	0.431208	0.161404	0.11319	1.11141	9.897341
SCGB1D2	0.568792	0.431208	0.161404	0.11319	1.11141	9.897341
UAP1L1	0.568832	0.431168	0.561701	0.561316	359.0853	639.7282
NGF	0.568938	0.431062	1.951981	1.962944	98.91545	50.38647
RAB3IL1	0.569015	0.430985	0.562019	0.561632	362.3195	645.1267
C6orf120	0.569118	0.430882	0.561615	0.561376	587.9357	1047.319
USP40	0.569573	0.430427	0.560864	0.560663	700.188	1248.865
B4GALNT	0.569924	0.430076	1.952182	1.962944	98.91545	50.38647
KLHL2	0.57251	0.42749	1.76027	1.761499	632.3921	359.0036
AFP	0.572587	0.427413	4.244289	4.926379	13.33691	2.699275
BORCS8	0.573146	0.426854	0.559187	0.558551	220.6148	394.9849
APOL6	0.573387	0.426613	1.791021	1.791256	3433.278	1916.683
ARPIN	0.573585	0.426415	0.56146	0.560954	276.8966	493.6254
SENP3-EI	0.574022	0.425978	3.373681	3.641012	18.40494	5.047644
SNAP91	0.57448	0.42552	0.162263	0.11319	1.11141	9.897341

PRIM1	0.574559	0.425441	0.563086	0.56274	404.5531	718.9069
SERHL	0.574709	0.425291	2.555184	2.627042	32.37536	12.31769
ERVFRD-1	0.574781	0.425219	0.084271	0.001585	0	6.298308
GUCY1A2	0.574781	0.425219	0.084271	0.001585	0	6.298308
ARHGAP2	0.574863	0.425137	2.884565	3.016963	24.45101	8.097825
C1QTNF6	0.574866	0.425134	0.541424	0.539984	99.72678	184.6934
H3F3A	0.57496	0.42504	1.843833	1.849172	166.4558	90.01182
SAP25	0.575026	0.424974	2.032455	2.048392	75.57585	36.89009
AHRR	0.575177	0.424823	2.416777	2.469502	37.78793	15.29589
CAMK2N2	0.575582	0.424418	1.853575	1.859385	155.5973	83.67752
TGFBR3L	0.575688	0.424312	1.908477	1.917222	115.5866	60.28381
LINGO2	0.576281	0.423719	4.233683	4.926379	13.33691	2.699275
LOC10012	0.576516	0.423484	0.299887	0.279879	5.568162	19.92065
SEMA4F	0.576893	0.423107	1.762919	1.76446	497.8115	282.1282
R3HDM2	0.57776	0.42224	0.56259	0.562371	639.0605	1136.377
TRHDE-AS	0.578185	0.421815	0.517981	0.515533	58.90471	114.2693
SLC9A7	0.578613	0.421387	1.771315	1.771726	1869.391	1055.12
IL17C	0.57921	0.42079	0.21224	0.177115	2.222819	12.59662
PINK1-AS	0.579543	0.420457	0.54851	0.54736	122.3106	223.464
SP100	0.579722	0.420278	1.756444	1.757307	893.5955	508.4984
BRSK2	0.579984	0.420016	0.336372	0.320525	7.779867	24.29347
TPTE2P5	0.579984	0.420016	0.336372	0.320525	7.779867	24.29347
GPRASP1	0.580632	0.419368	0.375142	0.363511	11.1141	30.59178
SP140	0.58076	0.41924	10.69535	573.3759	5.723759	0
AQP4	0.580884	0.419116	4.915131	6.147551	11.1141	1.799517
COL14A1	0.580884	0.419116	4.915131	6.147551	11.1141	1.799517
NMNAT3	0.5811	0.4189	0.510197	0.507376	51.12484	100.7729
SIK1	0.581119	0.418881	1.784387	1.784643	3111.947	1743.732
HES2	0.581237	0.418763	1.782567	1.785268	294.5569	164.9887
ACVRL1	0.581894	0.418106	2.582101	2.65908	31.11947	11.69686
SERGEF	0.582286	0.417714	1.807144	1.810986	216.7249	119.6679
DNASE1L	0.582293	0.417707	0.212688	0.177115	2.222819	12.59662
IGFBP4	0.582657	0.417343	1.800017	1.800182	4957.998	2754.16
LDOC1	0.582775	0.417225	2.041202	2.058417	72.24162	35.09057
TRIM24	0.582846	0.417154	0.559528	0.559403	1148.086	2052.349
PTP4A3	0.582913	0.417087	0.56397	0.563546	330.0886	585.7427
CSAD	0.583144	0.416856	0.525359	0.523241	66.79572	127.6667
SERPINA5	0.583443	0.416557	1.910337	1.919202	112.2524	58.48429
FOXE1	0.583556	0.416444	2.146829	2.171854	56.68189	26.09299
DDX53	0.584232	0.415768	0.0857	0.001585	0	6.298308
SLC9B1	0.584232	0.415768	0.0857	0.001585	0	6.298308
SNORA73	0.584232	0.415768	0.0857	0.001585	0	6.298308
KLF2	0.584615	0.415385	1.95973	1.971411	92.247	46.78743
FUT9	0.584968	0.415032	0.301196	0.281098	5.557048	19.79468
HSD17B3	0.584968	0.415032	0.301196	0.281098	5.557048	19.79468
RFPL2	0.584968	0.415032	0.301196	0.281098	5.557048	19.79468
HIST1H2A	0.584993	0.415007	0.551616	0.550552	132.2577	240.2355
CLIC2	0.585324	0.414676	4.897512	6.147551	11.1141	1.799517
MAEL	0.585324	0.414676	4.897512	6.147551	11.1141	1.799517
OLFML2B	0.585324	0.414676	4.897512	6.147551	11.1141	1.799517
TMEM211	0.585324	0.414676	4.897512	6.147551	11.1141	1.799517
ARHGAP4	0.585462	0.414538	0.547895	0.546677	115.5866	211.4432
TAF9B	0.585688	0.414312	0.563989	0.563486	283.4094	502.9649
STK32C	0.585819	0.414181	1.809418	1.813561	205.6108	113.3695
ZNF528	0.585828	0.414172	1.900751	1.909377	115.6977	60.58972
FAM172A	0.58611	0.41389	1.775461	1.777941	316.7517	178.1521

OVOL1	0.586561	0.413439	1.753829	1.754881	716.8592	408.4903
SNRNP48	0.586749	0.413251	1.763803	1.764268	1649.332	934.8489
SESTD1	0.586815	0.413185	1.761888	1.762392	1515.963	860.1689
HSPG2	0.586861	0.413139	1.844206	1.844262	15760.9	8545.904
NOS1	0.587712	0.412288	2.264982	2.301364	45.56779	19.79468
MZF1	0.587917	0.412083	1.764057	1.765999	395.6618	224.0398
CDK1	0.588611	0.411389	1.802781	1.802924	5880.468	3261.624
SLC25A13	0.589302	0.410698	0.551544	0.551489	2618.481	4748.025
FBXW4	0.589682	0.410318	0.56449	0.564012	292.3007	518.2608
HDGFRP3	0.590201	0.409799	1.764771	1.766814	376.7678	213.2427
SDK1	0.590405	0.409595	1.754026	1.7555	521.2511	296.9202
LOC72934	0.590502	0.409498	0.565568	0.565193	373.6892	661.1784
C9orf69	0.591132	0.408868	0.565234	0.565002	614.6095	1087.808
PIK3CD	0.5915	0.4085	1.753373	1.754096	1041.424	593.7055
LOC28368	0.59151	0.40849	3.262152	3.497255	18.90508	5.39855
SEZ6L2	0.592013	0.407987	1.772991	1.7733	2527.345	1425.217
LOC10192	0.592036	0.407964	0.085729	0.001615	0	6.18134
LBHD1	0.592064	0.407936	2.141609	2.166668	56.2151	25.94003
HIVEP2	0.592262	0.407738	1.750146	1.751052	841.3371	480.4709
HRASLS	0.592546	0.407454	0.447509	0.441265	22.22819	50.38647
BUB3	0.592783	0.407217	1.792502	1.792679	4553.445	2540.018
WDR78	0.592816	0.407184	0.532447	0.530549	74.46444	140.3623
ABAT	0.593278	0.406722	1.81212	1.81644	194.4967	107.0712
CNTNAP3	0.593578	0.406422	4.377215	5.192743	12.47002	2.393357
BHMT2	0.593683	0.406317	0.164656	0.115605	1.11141	9.690397
CACNA1F	0.594151	0.405849	3.257454	3.4952	18.89396	5.39855
LOC10050	0.594151	0.405849	3.257454	3.4952	18.89396	5.39855
IRF6	0.594489	0.405511	1.763609	1.764015	1887.173	1069.813
FUT1	0.595494	0.404506	0.566486	0.566148	413.1109	729.695
RNF103-C	0.596117	0.403883	0.08672	0.001606	0	6.21733
TSLP	0.596309	0.403691	0.388376	0.377624	12.22551	32.3913
ZNF2	0.596517	0.403483	0.546705	0.545359	104.6059	191.8195
ALOX15B	0.596524	0.403476	3.2534	3.4952	18.89396	5.39855
NHLH1	0.596524	0.403476	3.2534	3.4952	18.89396	5.39855
C15orf38-/	0.59666	0.40334	2.160732	2.187717	53.59217	24.49142
SLC4A7	0.597138	0.402862	1.76139	1.761801	1892.731	1074.311
LMCD1	0.597306	0.402694	1.77131	1.773957	300.0806	169.1546
C2orf72	0.597328	0.402672	1.914068	1.923553	105.5839	54.88526
FAM86B2	0.598016	0.401984	0.287151	0.26422	4.756833	18.03116
PAX9	0.598211	0.401789	1.834278	1.839883	158.9316	86.3768
APOBEC3	0.59846	0.40154	1.797901	1.801699	215.6135	119.6679
CEACAM6	0.598581	0.401419	10.58762	556.7048	5.557048	0
DEFB1	0.598581	0.401419	10.58762	556.7048	5.557048	0
EBF3	0.598581	0.401419	10.58762	556.7048	5.557048	0
FAM132A	0.598581	0.401419	10.58762	556.7048	5.557048	0
FAM71A	0.598581	0.401419	10.58762	556.7048	5.557048	0
HIST1H1B	0.598581	0.401419	10.58762	556.7048	5.557048	0
HIST1H1D	0.598581	0.401419	10.58762	556.7048	5.557048	0
KCNH4	0.598581	0.401419	10.58762	556.7048	5.557048	0
LINC0086	0.598581	0.401419	10.58762	556.7048	5.557048	0
LOC10099	0.598581	0.401419	10.58762	556.7048	5.557048	0
LOC10192	0.598581	0.401419	10.58762	556.7048	5.557048	0
LOC10272	0.598581	0.401419	10.58762	556.7048	5.557048	0
MYOZ2	0.598581	0.401419	10.58762	556.7048	5.557048	0
PLA2G2F	0.598581	0.401419	10.58762	556.7048	5.557048	0
PRELP	0.598581	0.401419	10.58762	556.7048	5.557048	0

SFTPA2	0.598581	0.401419	10.58762	556.7048	5.557048	0
RELL2	0.598729	0.401271	1.756694	1.758522	414.5558	235.7367
PRSS1	0.599396	0.400604	0.340146	0.324489	7.779867	23.99655
HOTAIRM	0.60032	0.39968	1.768393	1.770875	312.3061	176.3526
POMZP3	0.600418	0.399582	0.557001	0.556049	147.3618	265.0238
TAB3	0.600571	0.399429	1.757548	1.757995	1730.465	984.3356
HHIPL1	0.601042	0.398958	1.968685	1.98129	85.57854	43.1884
TIMM8B	0.601133	0.398867	0.567335	0.567072	542.3679	956.4431
WWC1	0.601143	0.398857	1.829433	1.8295	13107.96	7164.775
MYLK	0.601248	0.398752	1.769054	1.769359	2544.017	1437.814
SNX30	0.601612	0.398388	1.749813	1.750468	1140.306	651.425
SERINC2	0.601831	0.398169	1.786593	1.78678	4223.356	2363.665
EFEMP2	0.602196	0.397804	2.117081	2.140645	57.7933	26.99275
KCNJ18	0.602203	0.397797	3.386357	3.67672	17.52693	4.759721
GPS2	0.603232	0.396768	0.566944	0.566722	643.5061	1135.495
ADGRF2	0.60429	0.39571	10.45061	558.9276	5.579276	0
PCDHA4	0.60429	0.39571	10.45061	558.9276	5.579276	0
AMZ2P1	0.60472	0.39528	0.525104	0.522748	61.14976	116.9866
CAPN2	0.605027	0.394973	1.810396	1.810496	8338.906	4605.863
LINC0090	0.605165	0.394835	0.458729	0.453019	24.45101	53.9855
NEAT1	0.605544	0.394456	0.555523	0.55546	2285.069	4113.839
SLC8A1	0.605829	0.394171	0.568459	0.568158	472.3491	831.3767
RSG1	0.606174	0.393826	0.524921	0.522639	61.12753	116.9686
LINC0131	0.606217	0.393783	4.831995	6.067163	10.91404	1.790519
ZNF79	0.606331	0.393669	0.548442	0.547055	103.3611	188.9492
ARHGAP2	0.606841	0.393159	2.030097	2.047577	70.0188	34.19082
FPGT	0.607061	0.392939	0.566325	0.565734	241.1203	426.2155
SCN4A	0.607209	0.392791	0.553508	0.552359	122.2551	221.3405
C9orf173-/	0.607874	0.392126	10.41296	556.7048	5.557048	0
CDH10	0.607874	0.392126	10.41296	556.7048	5.557048	0
RP1L1	0.607874	0.392126	10.41296	556.7048	5.557048	0
TNN	0.607874	0.392126	10.41296	556.7048	5.557048	0
THEMIS2	0.608133	0.391867	1.74739	1.747987	1269.23	726.105
RNF146	0.608606	0.391394	0.568097	0.567655	315.6403	556.0506
ZNF12	0.609383	0.390617	0.568666	0.568371	474.5719	834.9757
POGLUT1	0.609525	0.390475	0.568818	0.568484	417.89	735.1025
LUC7L	0.609697	0.390303	0.555052	0.553913	125.5893	226.7391
GIN1	0.610782	0.389218	0.552007	0.550796	114.4752	207.8442
ARL4C	0.611615	0.388385	0.568733	0.568472	533.4766	938.4479
CBFA2T3	0.611951	0.388049	2.932471	3.085181	22.22819	7.198066
VPS54	0.611977	0.388023	1.747649	1.748206	1337.026	764.7946
INPP5J	0.612313	0.387687	0.550973	0.549644	107.8067	196.1473
FLCN	0.612464	0.387536	0.569304	0.568979	429.0041	753.9975
ZNF284	0.612553	0.387447	1.861795	1.868973	127.8121	68.38163
C2CD2L	0.61274	0.38726	1.743898	1.744575	1092.516	626.2318
COMMD1	0.612754	0.387246	0.566536	0.565932	231.1732	408.4903
TCAF2P1	0.613229	0.386771	10.35648	553.3706	5.523706	0
STX18	0.613529	0.386471	0.569517	0.569089	332.3115	583.9431
PXDC1	0.614204	0.385796	0.556812	0.556749	2216.151	3980.531
PRMT3	0.61443	0.38557	0.56964	0.569312	425.6699	747.6992
TAF15	0.614805	0.385195	0.562424	0.562322	1401.487	2492.331
ZBED3	0.615296	0.384704	0.521594	0.51905	55.57048	107.0712
ZNF285	0.615389	0.384611	1.904893	1.914439	103.3611	53.9855
TOR1B	0.615663	0.384337	1.739221	1.740051	900.2418	517.361
FAXDC2	0.616088	0.383912	1.766833	1.769726	272.2953	153.8587
TEX22	0.617792	0.382208	0.28401	0.260481	4.445638	17.09541

SYNE4	0.618092	0.381908	0.568882	0.568394	282.298	496.6666
PPP1R3G	0.619174	0.380826	2.255488	2.293316	43.34497	18.89492
TMEM17	0.619679	0.380321	0.513154	0.510233	47.79061	93.67384
LGALS8-A	0.619845	0.380155	0.284313	0.260481	4.445638	17.09541
LMO7-AS1	0.619845	0.380155	0.284313	0.260481	4.445638	17.09541
FAM72B	0.620508	0.379492	1.739318	1.740032	1026.42	589.8815
FOXD1	0.621135	0.378865	2.156231	2.184903	51.12484	23.39372
ADAM8	0.621509	0.378491	1.957984	1.970884	83.35572	42.28864
MAGI2	0.621564	0.378436	0.371909	0.358845	10.00269	27.89251
TMEM241	0.621722	0.378278	0.569844	0.569316	268.9611	472.4361
RAPGEF3	0.621863	0.378137	1.765606	1.765854	3158.626	1788.72
SFMBT1	0.621937	0.378063	0.570253	0.569795	304.5262	534.4564
ABLIM2	0.621953	0.378047	2.010358	2.026754	71.13021	35.09057
ZNF133	0.622324	0.377676	0.567079	0.56641	208.945	368.9009
WDR53	0.622612	0.377388	0.56489	0.564079	175.6027	311.3164
HNF1A-AS	0.622847	0.377153	0.169284	0.119863	1.122524	9.438465
CHD1L	0.622999	0.377001	0.568925	0.568737	743.533	1307.349
NME7	0.623237	0.376763	1.735984	1.737118	642.3947	369.8007
PHOSPHO	0.624333	0.375667	1.748038	1.750109	360.0411	205.7207
PAM	0.624374	0.375626	1.812501	1.812578	11106.32	6127.354
CCDC47	0.624408	0.375592	0.558251	0.558188	2233.933	4002.125
EFR3B	0.625916	0.374084	0.50063	0.496996	38.89934	78.27897
DHRS3	0.626899	0.373101	1.739203	1.74093	432.3383	248.3333
HLA-E	0.627238	0.372762	1.780672	1.780831	4998.965	2807.093
SBF2-AS1	0.627582	0.372418	0.530347	0.528078	62.23894	117.8683
KCTD18	0.627725	0.372275	0.570457	0.569933	265.6269	466.0748
STARD4	0.627774	0.372226	0.571432	0.571191	587.9357	1029.324
MYOZ3	0.627982	0.372018	2.296482	2.339655	40.01074	17.09541
NPTN-IT1	0.627995	0.372005	3.396888	3.699693	16.67114	4.498792
SHANK2	0.628392	0.371608	1.75442	1.757022	295.6349	168.2548
CATSPER	0.628724	0.371276	0.48122	0.476529	30.00806	62.98308
CPNE5	0.629509	0.370491	2.10516	2.129276	55.57048	26.09299
ALG6	0.629631	0.370369	0.572072	0.571669	345.6484	604.6376
IPCEF1	0.629686	0.370314	0.257325	0.22843	3.334229	14.63007
DZANK1	0.629711	0.370289	0.491047	0.486879	33.85354	69.54232
LPIN2	0.630478	0.369522	0.566313	0.566194	1174.76	2074.843
MDFIC	0.630618	0.369382	3.391823	3.699693	16.67114	4.498792
FOSB	0.631039	0.368961	1.784572	1.788545	201.1651	112.4698
SEMA6C	0.632462	0.367538	1.895231	1.905017	101.1383	53.08574
RGS19	0.632809	0.367191	1.734082	1.735552	493.4659	284.3236
TMX1	0.633316	0.366684	1.74537	1.745758	1947.201	1115.385
SRSF8	0.633522	0.366478	0.568457	0.568312	953.9895	1678.643
CASZ1	0.634024	0.365976	1.736582	1.738277	430.1155	247.4335
AFAP1L2	0.634178	0.365822	1.740299	1.740771	1553.751	892.5602
USB1	0.635499	0.364501	1.7544	1.754683	2721.842	1551.183
GABRE	0.636546	0.363454	1.925922	1.937413	88.91277	45.88767
ZSCAN16	0.636668	0.363332	0.571535	0.570938	237.8417	416.5881
ZNF542P	0.637096	0.362904	1.789349	1.793694	185.6054	103.4722
CNTNAP3	0.637592	0.362408	10.16577	532.2538	5.312538	0
CDCA5	0.639439	0.360561	1.748822	1.749142	2333.96	1334.342
PLEKHA1	0.640293	0.359707	1.735742	1.736244	1448.167	834.0759
PRKD2	0.640556	0.359444	1.737828	1.738286	1593.761	916.8537
SEPT8	0.640946	0.359054	1.787275	1.78739	6965.204	3896.853
KNDC1	0.641112	0.358888	0.570806	0.570119	206.7222	362.6026
JMJD6	0.641178	0.358822	1.739873	1.740292	1750.603	1005.921
ZDHHC6	0.641179	0.358821	1.73458	1.735098	1400.376	807.0832

NFKBIZ	0.642128	0.357872	1.7337	1.734227	1373.147	791.7873
C2orf88	0.642525	0.357475	0.566052	0.565154	155.5973	275.326
CENPV	0.642549	0.357451	1.946997	1.960026	81.1329	41.38888
HIVEP3	0.642974	0.357026	1.78107	1.785303	191.1624	107.0712
TMEM139	0.644264	0.355736	0.573583	0.57335	590.1585	1029.324
OR2A1-AS	0.64442	0.35558	0.422077	0.413201	15.31522	37.07904
SATB2	0.646243	0.353757	2.076772	2.099486	56.68189	26.99275
MMP17	0.646246	0.353754	0.437523	0.429783	17.78255	41.38888
ZAK	0.646499	0.353501	1.766999	1.767175	4390.112	2484.251
TPM4	0.646543	0.353457	1.867502	1.86752	49718.91	26622.95
LRRC37A	0.646796	0.353204	0.45372	0.4471	21.00564	46.99438
LHFP	0.646873	0.353127	1.72513	1.726453	539.0336	312.2161
TP53TG1	0.647064	0.352936	0.534407	0.53214	62.23894	116.9686
C2orf15	0.647203	0.352797	0.520562	0.517672	48.90202	94.47462
ZNF506	0.647281	0.352719	1.746436	1.749141	280.0974	160.13
CTGF	0.647834	0.352166	1.797913	1.797996	9800.41	5450.736
CD19	0.648781	0.351219	2.99291	3.172859	20.00537	6.298308
ZFYVE9	0.648822	0.351178	0.5686	0.568489	1260.338	2217.004
ARHGAP2	0.649091	0.350909	0.522365	0.51954	50.01343	96.27414
ZSCAN5A	0.649244	0.350756	0.521006	0.518116	48.90202	94.39364
NHSL2	0.649986	0.350014	2.144516	2.173485	48.90202	22.49396
NES	0.650263	0.349737	1.720626	1.721654	700.188	406.6908
FAM65B	0.650542	0.349458	2.49157	2.564143	30.00806	11.69686
GHRLOS	0.650542	0.349458	2.49157	2.564143	30.00806	11.69686
MAFB	0.650542	0.349458	2.49157	2.564143	30.00806	11.69686
APOBR	0.650646	0.349354	0.261345	0.232139	3.334229	14.39613
LAMA4	0.650646	0.349354	0.261345	0.232139	3.334229	14.39613
MBTPS2	0.650694	0.349306	0.572573	0.572409	851.3397	1487.3
ZNF502	0.650775	0.349225	2.989798	3.172859	20.00537	6.298308
CA11	0.651163	0.348837	0.570083	0.569294	176.7141	310.4166
SIRT1	0.651405	0.348595	0.575349	0.575116	609.0525	1059.016
CASC15	0.651535	0.348465	2.490264	2.564143	30.00806	11.69686
CTU2	0.65165	0.34835	1.729387	1.731315	380.1021	219.541
ASRGL1	0.651708	0.348292	0.575755	0.575261	286.7437	498.4661
GULP1	0.652157	0.347843	0.576073	0.575809	535.6994	930.3501
SGOL2	0.652246	0.347754	1.73879	1.739141	2125.015	1221.872
NECAP2	0.652286	0.347714	1.734595	1.734998	1832.714	1056.316
RFT1	0.65267	0.34733	0.572552	0.572396	889.1943	1553.469
EFHC2	0.652682	0.347318	0.444927	0.437608	18.89396	43.1884
RFXAP	0.652987	0.347013	0.56467	0.563622	133.3691	236.6364
HNF4A	0.653951	0.346049	0.261875	0.232139	3.334229	14.39613
RRAGC	0.654179	0.345821	0.57121	0.571081	1104.741	1934.48
THTPA	0.654919	0.345081	0.55542	0.553969	96.69263	174.5531
SRD5A3	0.655068	0.344932	0.576636	0.576166	301.192	522.7596
TANGO2	0.655507	0.344493	0.575274	0.57507	696.9094	1211.875
FAM167A	0.655786	0.344214	1.749249	1.752213	252.29	143.9793
PTCD2	0.656031	0.343969	0.566624	0.5656	138.9262	245.634
PALM3	0.656249	0.343751	0.573079	0.572358	196.7195	343.7077
ANKLE2	0.656873	0.343127	1.738392	1.738721	2260.607	1300.151
MYH14	0.656904	0.343096	0.550032	0.550009	6143.872	11170.5
IER3	0.6576	0.3424	1.781199	1.781309	7250.836	4070.507
GOLGA3	0.658145	0.341855	1.734147	1.734525	1922.794	1108.538
TSPAN10	0.658329	0.341671	0.543485	0.541506	71.13021	131.3647
C18orf32	0.658745	0.341255	0.57267	0.572532	1029.576	1798.293
LOC1019	0.659174	0.340826	0.411059	0.400796	13.33691	33.29106
AGT	0.659293	0.340707	3.596369	4.006149	14.44832	3.599033

CPA5	0.659293	0.340707	3.596369	4.006149	14.44832	3.599033
SLC6A13	0.659293	0.340707	3.596369	4.006149	14.44832	3.599033
LOC10192	0.659795	0.340205	0.533358	0.530925	57.99335	109.2397
LOC10361	0.66091	0.33909	0.562194	0.561	115.5866	206.0447
ZNF780B	0.660937	0.339063	0.570685	0.5698	160.4653	281.6243
LOC10028	0.661101	0.338899	0.564621	0.563501	124.7668	221.4215
MARVELD	0.66125	0.33875	0.57561	0.574976	223.3933	388.5336
KIAA1211	0.662111	0.337889	0.568403	0.567438	144.4832	254.6316
DTX3	0.662291	0.337709	0.578004	0.577592	336.7571	583.0434
PPCDC	0.662359	0.337641	0.554369	0.552811	90.02418	162.8563
MIR99AH(	0.662478	0.337522	0.49183	0.487214	31.11947	63.88284
NNMT	0.663133	0.336867	1.75886	1.759034	4355.614	2476.135
F8	0.663484	0.336516	1.853886	1.862201	108.9181	58.48429
AMPD3	0.663743	0.336257	1.751541	1.754822	228.9504	130.465
CHPF2	0.663766	0.336234	1.74281	1.743075	2788.527	1599.77
SLC9A3R	0.664731	0.335269	1.713078	1.714026	747.9786	436.3828
C21orf59	0.665362	0.334638	0.573019	0.572889	1074.733	1875.996
PON2	0.66562	0.33438	1.752052	1.752253	3736.559	2132.427
LOXL1	0.666439	0.333561	0.578965	0.578614	395.6618	683.8163
KCNK1	0.666754	0.333246	1.713402	1.714185	906.9102	529.0579
LOC10050	0.667223	0.332777	0.53973	0.537506	63.35035	117.8683
TMEM200	0.667546	0.332454	2.109891	2.137414	50.01343	23.39372
KIFC3	0.667644	0.332356	1.738682	1.738963	2605.144	1498.098
NEURL2	0.668088	0.331912	0.464006	0.457605	22.22819	48.58695
FAM149B	0.668301	0.331699	0.57962	0.579297	436.784	753.9975
ZNF778	0.668688	0.331312	0.578057	0.577487	247.8443	429.1847
PPP1R14(	0.668968	0.331032	1.711793	1.712766	713.525	416.5881
CABYR	0.669211	0.330789	0.57391	0.573132	178.9369	312.2161
PIGH	0.669515	0.330485	0.578948	0.578479	295.6349	511.0627
TNFRSF2	0.669568	0.330432	1.803737	1.809653	140.0376	77.37921
KHDC1	0.669645	0.330355	0.542027	0.53988	65.57317	121.4674
PRDM11	0.669645	0.330355	0.542027	0.53988	65.57317	121.4674
L3MBTL3	0.669952	0.330048	0.57416	0.573387	180.0484	314.0156
BTD	0.67033	0.32967	1.864156	1.873272	101.1383	53.9855
KCNN4	0.67033	0.32967	1.864156	1.873272	101.1383	53.9855
HBP1	0.67085	0.32915	1.741457	1.741704	2969.686	1705.042
FTH1	0.670971	0.329029	0.508864	0.508863	170475.8	335013
WDR7	0.670988	0.329012	0.570078	0.569106	143.3718	251.9323
RASSF9	0.67123	0.32877	2.198419	2.234527	42.23356	18.89492
PLEKHF1	0.671432	0.328568	0.579889	0.579555	414.5558	715.3079
LYRM7	0.671461	0.328539	0.577708	0.57751	701.3884	1214.512
SIRT3	0.671723	0.328277	0.568552	0.567506	133.3691	235.0169
ADRA2B	0.671968	0.328032	0.469316	0.463318	23.3396	50.38647
NSL1	0.672126	0.327874	0.577744	0.577144	231.1732	400.5544
FARSB	0.672335	0.327665	0.570455	0.570363	1529.3	2681.28
FANK1	0.672635	0.327365	2.063491	2.086698	54.45907	26.09299
XKR7	0.672635	0.327365	2.063491	2.086698	54.45907	26.09299
AK8	0.672726	0.327274	0.228276	0.190727	2.222819	11.69686
NLRC4	0.672726	0.327274	0.228276	0.190727	2.222819	11.69686
ENO1-AS	0.672748	0.327252	0.176567	0.124496	1.11141	8.997583
PLCH2	0.672748	0.327252	0.176567	0.124496	1.11141	8.997583
SBSPON	0.672748	0.327252	0.176567	0.124496	1.11141	8.997583
VPS36	0.67282	0.32718	0.580062	0.579748	442.341	762.995
FGD3	0.673632	0.326368	0.55537	0.553753	86.68995	156.5579
SLC25A1(	0.674017	0.325983	0.574789	0.574656	1048.059	1823.81
LOC10050	0.674033	0.325967	0.430616	0.421943	15.55973	36.89009

RBPMS	0.674244	0.325756	1.71087	1.711663	862.4538	503.8647
KSR2	0.674407	0.325593	0.383801	0.370802	10.00269	26.99275
USP19	0.674583	0.325417	0.5766	0.576444	910.2444	1579.076
SLC2A14	0.674758	0.325242	3.779227	4.299599	13.07018	3.032185
SLC43A2	0.674861	0.325139	0.567224	0.566093	123.3442	217.8945
FLJ31104	0.675131	0.324869	2.439009	2.507089	30.17477	12.02977
FAM171A1	0.675172	0.324828	0.580557	0.580235	431.2269	743.2004
DPH6	0.676378	0.323622	0.533568	0.530978	54.45907	102.5724
RILPL1	0.676415	0.323585	0.57714	0.576453	202.2765	350.9057
EXOC3-AS	0.676636	0.323364	0.228952	0.190727	2.222819	11.69686
STK4-AS1	0.676636	0.323364	0.228952	0.190727	2.222819	11.69686
EYA4	0.67708	0.32292	0.579928	0.5797	620.1665	1069.813
MSTO1	0.677519	0.322481	0.577843	0.577669	799.4369	1383.909
LOC10050	0.677846	0.322154	0.177491	0.124496	1.11141	8.997583
SLCO6A1	0.677846	0.322154	0.177491	0.124496	1.11141	8.997583
IFT81	0.678177	0.321823	0.580442	0.579951	282.298	486.7692
CACNA1D	0.678599	0.321401	2.13129	2.161117	46.6792	21.5942
SCAI	0.679984	0.320016	1.70644	1.707832	503.1018	294.5809
PIM2	0.680176	0.319824	1.706058	1.706938	780.2095	457.0772
RBKS	0.680436	0.319564	0.573649	0.572773	156.6643	273.5265
XKR9	0.680556	0.319444	1.735144	1.738104	245.8327	141.433
ACSF3	0.680652	0.319348	0.574108	0.573227	157.8202	275.326
GPR146	0.680674	0.319326	0.3407	0.322562	6.668457	20.69444
HEATR4	0.680674	0.319326	0.3407	0.322562	6.668457	20.69444
TRAF3	0.680735	0.319265	1.722496	1.722869	1900.51	1103.104
LINC0031	0.680875	0.319125	0.439295	0.431042	16.67114	38.68961
CYLD	0.680969	0.319031	1.742905	1.743109	3680.989	2111.733
KIAA0753	0.681405	0.318595	0.581863	0.581494	375.6564	646.0265
TNFAIP2	0.681919	0.318081	1.783778	1.783856	10244.97	5743.157
UNC5A	0.681943	0.318057	2.233602	2.27468	38.89934	17.09541
FAM71F1	0.683196	0.316804	3.074145	3.289709	17.78255	5.39855
CHST6	0.684513	0.315487	0.577969	0.577237	189.562	328.4028
UPK1A-AS	0.684553	0.315447	0.521316	0.518058	43.34497	83.67752
TMEM98	0.684617	0.315383	0.567573	0.566393	116.698	206.0447
GRID1	0.684713	0.315287	3.071542	3.289709	17.78255	5.39855
PCDHGA6	0.684713	0.315287	3.071542	3.289709	17.78255	5.39855
MSH5	0.685829	0.314171	0.550307	0.548359	72.11937	131.5267
MAP6	0.685934	0.314066	3.905329	4.516155	12.22551	2.699275
CEBPA-AS	0.686056	0.313944	0.398126	0.386223	11.1141	28.79227
LURAP1	0.686389	0.313611	0.447108	0.439332	17.78255	40.48912
TNFRSF9	0.687021	0.312979	0.566033	0.5647	106.6953	188.9492
IGF2BP1	0.687416	0.312584	1.81918	1.826621	116.698	63.88284
NAF1	0.687581	0.312419	0.578144	0.577388	183.3826	317.6147
CKAP2L	0.688521	0.311479	1.707592	1.708156	1220.328	714.4081
THSD4	0.688823	0.311177	1.701974	1.702712	939.1411	551.5518
LINC0141	0.6892	0.3108	3.895831	4.516155	12.22551	2.699275
NAV3	0.6892	0.3108	3.895831	4.516155	12.22551	2.699275
PP7080	0.689412	0.310588	0.495241	0.490543	30.00806	61.18356
COCH	0.689955	0.310045	2.001733	2.020944	60.01612	29.69202
GPR153	0.690053	0.309947	1.717849	1.720228	295.6349	171.8538
TMEM63B	0.690312	0.309688	1.708337	1.708842	1391.485	814.2813
DARS2	0.690704	0.309296	0.574459	0.574362	1431.496	2492.331
RBM11	0.691207	0.308793	0.532307	0.529435	50.01343	94.47462
RPTOR	0.691625	0.308375	0.581586	0.581393	731.3075	1257.862
INE1	0.691975	0.308025	0.299563	0.274944	4.445638	16.19565
FKBP9P1	0.692218	0.307782	2.421705	2.489781	29.60795	11.88581

BATF3	0.69227	0.30773	2.611756	2.715602	24.45101	8.997583
CMPK2	0.692531	0.307469	2.276761	2.324275	35.56511	15.29589
PJA2	0.692785	0.307215	1.764881	1.764985	7557.585	4281.95
IFIT2	0.693436	0.306564	1.698106	1.699163	639.0605	376.099
TRIP6	0.69352	0.30648	0.575113	0.575016	1462.615	2543.617
TMED9	0.693727	0.306273	1.789365	1.789425	13421.38	7500.385
LONRF1	0.693777	0.306223	0.582372	0.581782	234.5074	403.0917
TTLL3	0.693926	0.306074	1.836205	1.844812	104.0168	56.37886
SH3BP5	0.694093	0.305907	0.582571	0.582356	642.3947	1103.104
SEC14L1	0.694173	0.305827	1.775882	1.775963	9782.738	5508.41
TRIQK	0.69424	0.30576	1.697172	1.698421	550.1477	323.913
MFSD10	0.694406	0.305594	1.704005	1.704577	1194.765	700.9117
LOC64785	0.694804	0.305196	0.09683	0.001813	0	5.506521
CRYL1	0.694853	0.305147	0.583429	0.5829	261.1813	448.0796
NPR2	0.695111	0.304889	2.096705	2.124098	47.79061	22.49396
FAM208A	0.695421	0.304579	0.570036	0.569972	2153.912	3778.985
SLC2A12	0.695636	0.304364	1.705072	1.707024	357.8739	209.6437
KANK3	0.695668	0.304332	0.460976	0.453882	20.00537	44.08816
EDAR	0.695913	0.304087	5.674425	8.562568	7.779867	0.899758
TLX1	0.695913	0.304087	5.674425	8.562568	7.779867	0.899758
SLTM	0.6962	0.3038	0.571253	0.571185	2085.004	3650.319
MAOB	0.696499	0.303501	0.411	0.39983	12.22551	30.59178
GPHN	0.697016	0.302984	0.583379	0.582772	232.2846	398.5929
RND2	0.698536	0.301464	0.456181	0.448826	18.89396	42.10869
CAP1	0.698611	0.301389	1.794174	1.794225	15903.16	8863.519
CHURC1	0.698917	0.301083	0.582489	0.582302	741.877	1274.049
BTBD3	0.699153	0.300847	0.579339	0.579207	1055.839	1822.91
BANCR	0.699476	0.300524	5.650718	8.562568	7.779867	0.899758
CCDC129	0.699476	0.300524	5.650718	8.562568	7.779867	0.899758
CSF2RB	0.699476	0.300524	5.650718	8.562568	7.779867	0.899758
MGAM	0.699476	0.300524	5.650718	8.562568	7.779867	0.899758
P2RX6	0.699476	0.300524	5.650718	8.562568	7.779867	0.899758
HLA-DMB	0.700138	0.299862	0.585117	0.584838	495.6887	847.5723
BFSP2	0.700587	0.299413	0.096954	0.001849	0	5.39855
C16orf96	0.700587	0.299413	0.096954	0.001849	0	5.39855
GATA3	0.700587	0.299413	0.096954	0.001849	0	5.39855
LINC0062	0.700587	0.299413	0.096954	0.001849	0	5.39855
PPP1R42	0.700587	0.299413	0.096954	0.001849	0	5.39855
ROPN1L	0.700587	0.299413	0.096954	0.001849	0	5.39855
ROPN1L-A	0.700587	0.299413	0.096954	0.001849	0	5.39855
SERPINF2	0.700587	0.299413	0.096954	0.001849	0	5.39855
SPDYC	0.700587	0.299413	0.096954	0.001849	0	5.39855
WDR87	0.700587	0.299413	0.096954	0.001849	0	5.39855
NUDT7	0.700798	0.299202	0.362594	0.346155	7.779867	22.49396
BDH1	0.701339	0.298661	0.583704	0.583498	671.2914	1150.467
TMEM61	0.702457	0.297543	0.472653	0.466237	22.22819	47.68719
GJA9-MY(	0.702488	0.297512	9.417931	488.9088	4.879088	0
MAP4K1	0.702655	0.297345	0.421953	0.411925	13.33691	32.3913
IL10	0.702896	0.297104	0.097247	0.001855	0	5.380555
IDH1-AS1	0.703247	0.296753	0.422085	0.411925	13.33691	32.3913
CCDC28B	0.703271	0.296729	0.581017	0.580199	172.2685	296.9202
CD55	0.703447	0.296553	1.7062	1.706624	1621.547	950.1448
ANKRD33	0.703592	0.296408	4.44798	5.533349	10.00269	1.799517
GPC6	0.703592	0.296408	4.44798	5.533349	10.00269	1.799517
GRIN2B	0.703592	0.296408	4.44798	5.533349	10.00269	1.799517
LINC0111	0.703592	0.296408	4.44798	5.533349	10.00269	1.799517

LOC10099	0.703592	0.296408	4.44798	5.533349	10.00269	1.799517
PITX1	0.703924	0.296076	1.692056	1.69338	513.4712	303.2185
ERMARD	0.704202	0.295798	0.57177	0.570554	114.4752	200.6461
APC2	0.704421	0.295579	5.617152	8.562568	7.779867	0.899758
MYO18B	0.704421	0.295579	5.617152	8.562568	7.779867	0.899758
PTGIR	0.704421	0.295579	5.617152	8.562568	7.779867	0.899758
SAMD14	0.704421	0.295579	5.617152	8.562568	7.779867	0.899758
SHC4	0.704421	0.295579	5.617152	8.562568	7.779867	0.899758
KIF19	0.704598	0.295402	1.911015	1.924424	74.46444	38.68961
WHAMMP	0.704914	0.295086	2.227361	2.270168	37.06551	16.32162
ZNF655	0.705296	0.294704	0.579569	0.579451	1198.6	2068.517
IL15RA	0.706015	0.293985	1.693544	1.69513	430.1155	253.7318
TEX2	0.706184	0.293816	0.580756	0.580626	1090.293	1877.796
NCOA1	0.706351	0.293649	0.582795	0.58263	823.5545	1413.52
AGPAT2	0.706446	0.293554	1.694577	1.695202	1093.627	645.1267
CCDC126	0.70677	0.29323	1.733353	1.73698	200.0537	115.1691
EP300-AS	0.706777	0.293223	0.534692	0.531814	48.97982	92.10826
NOL9	0.707003	0.292997	0.587008	0.586679	419.0236	714.2371
TPCN1	0.707161	0.292839	1.741547	1.741697	4911.319	2819.843
TAPBP	0.70722	0.29278	1.738395	1.738554	4659.963	2680.362
C2orf54	0.707368	0.292632	0.567598	0.566173	97.80404	172.7536
ANKRD18	0.70742	0.29258	2.20689	2.247528	38.01021	16.90646
UMAD1	0.707814	0.292186	0.58498	0.584324	214.502	367.1014
CECR1	0.708647	0.291353	0.098576	0.001849	0	5.39855
DPH6-AS1	0.708647	0.291353	0.098576	0.001849	0	5.39855
FAM83F	0.708647	0.291353	0.098576	0.001849	0	5.39855
IDE	0.708759	0.291241	0.579141	0.579032	1275.898	2203.508
TMA16	0.709072	0.290928	0.581914	0.581073	167.8228	288.8224
ZMYM1	0.709108	0.290892	0.586573	0.586318	541.2565	923.152
CNNM4	0.709402	0.290598	0.58465	0.584461	734.6417	1256.962
TBPL1	0.709954	0.290046	0.588097	0.58775	404.5531	688.3151
TMEM133	0.710017	0.289983	0.570067	0.568719	103.3611	181.7512
FAM129A	0.710117	0.289883	0.558757	0.558734	6211.668	11117.41
CLDN9	0.710177	0.289823	0.558922	0.557097	76.68726	137.663
SPINT2	0.710205	0.289795	1.799302	1.799342	20501.06	11393.64
ZNRD1-AS	0.710412	0.289588	0.43238	0.422748	14.44832	34.19082
ZNF619	0.710497	0.289503	0.577212	0.576134	131.1463	227.6389
ACER2	0.710843	0.289157	1.808854	1.816383	111.141	61.18356
HMBOX1	0.711256	0.288744	1.72586	1.729264	210.0564	121.4674
C7orf55	0.711756	0.288244	0.572858	0.571612	111.6633	195.3555
TRAF3IP2	0.712125	0.287875	0.491364	0.486086	26.67383	54.88526
FAM120C	0.71219	0.28781	0.584048	0.583317	188.9396	323.913
CEP295NI	0.712272	0.287728	2.401038	2.469206	28.89665	11.69686
HDX	0.712272	0.287728	2.401038	2.469206	28.89665	11.69686
GSS	0.712503	0.287497	0.58352	0.583366	899.1304	1541.286
LOC10050	0.713339	0.286661	1.854119	1.864319	88.91277	47.68719
ZNF91	0.713722	0.286278	1.701283	1.70364	294.179	172.6726
BCAR3	0.714084	0.285916	1.711689	1.711982	2370.637	1384.728
C15orf59	0.714441	0.285559	0.27541	0.245154	3.334229	13.63134
TLK2	0.71453	0.28547	0.585826	0.585635	722.4162	1233.569
IFI16	0.714768	0.285232	1.702456	1.70284	1772.698	1041.02
ATG2B	0.714795	0.285205	0.581512	0.58139	1114.744	1917.385
FAM41C	0.715418	0.284582	0.435067	0.425707	14.62615	34.37077
IL6	0.715656	0.284344	1.685711	1.686951	531.2538	314.9154
AMIGO1	0.715676	0.284324	0.499057	0.494179	28.89665	58.48429
ZNF343	0.71586	0.28414	0.587515	0.586946	242.2873	412.8001

LYPD6B	0.715916	0.284084	0.588923	0.5885	325.643	553.3514
TIAF1	0.716522	0.283478	9.245354	478.9061	4.779061	0
ZNF41	0.716956	0.283044	0.589133	0.588805	420.1128	713.5083
CDK7	0.717199	0.282801	1.684424	1.685342	715.7478	424.6859
CNPY3	0.717206	0.282794	1.688716	1.689325	1108.075	655.9238
CALCOCO	0.717223	0.282777	1.702628	1.702993	1869.391	1097.705
GKAP1	0.717604	0.282396	1.691741	1.69369	348.9826	206.0447
OXCT2	0.717792	0.282208	2.285198	2.337084	32.96441	14.09921
ENOX1	0.718583	0.281417	2.141349	2.175738	41.12215	18.89492
BICC1	0.718702	0.281298	1.722506	1.722707	3589.853	2083.84
PCDHGB2	0.718737	0.281263	3.706583	4.248569	12.3033	2.888224
PDE8B	0.718856	0.281144	0.330376	0.309192	5.557048	17.99517
TPPP3	0.718856	0.281144	0.330376	0.309192	5.557048	17.99517
TPPP	0.719009	0.280991	3.178038	3.453195	15.55973	4.498792
DAPK3	0.719083	0.280917	1.690493	1.691044	1208.102	714.4081
NRSN2-AS	0.719125	0.280875	0.578884	0.577788	128.9235	223.1401
CLCF1	0.71929	0.28071	1.704855	1.707479	261.1813	152.9589
C21orf62	0.719365	0.280635	0.387138	0.373102	9.269156	24.86032
LOC10544	0.720124	0.279876	0.44975	0.441302	16.67114	37.78985
DNAJC6	0.720376	0.279624	0.583918	0.583784	1054.728	1806.715
EXT1	0.720476	0.279524	1.712855	1.71311	2725.176	1590.773
USP37	0.720616	0.279384	0.588979	0.588726	543.4793	923.152
ZNF202	0.720876	0.279124	0.589858	0.589548	443.4524	752.1979
TRIP11	0.721501	0.278499	0.57921	0.579121	1564.865	2702.145
SLC16A5	0.721593	0.278407	0.590586	0.590151	322.3088	546.1533
TBCD	0.721868	0.278132	0.579472	0.579383	1591.539	2746.962
WDR89	0.722412	0.277588	0.590412	0.589998	332.3115	563.2487
PVRL4	0.723094	0.276906	1.696107	1.698403	293.4121	172.7536
EPB41L4A	0.723289	0.276711	1.683027	1.68379	846.8941	502.9649
C18orf21	0.723379	0.276621	0.589508	0.58896	251.1786	426.4854
ZFP30	0.723445	0.276555	0.582032	0.581033	141.149	242.9347
MAPK12	0.723458	0.276542	1.750148	1.75524	150.0403	85.47704
ATP5H	0.723808	0.276192	0.573962	0.573904	2429.541	4233.363
BTN2A3P	0.724095	0.275905	2.033601	2.058283	50.01343	24.29347
ZFYVE28	0.724099	0.275901	1.897644	1.911426	72.24162	37.78985
PHYKPL	0.724501	0.275499	0.591342	0.590936	344.537	583.0434
CTSE	0.724562	0.275438	2.264215	2.315145	33.34229	14.39613
IFI27L2	0.724629	0.275371	1.680219	1.681463	523.4739	311.3164
LRRC8C	0.724641	0.275359	1.682496	1.683991	437.8954	260.0302
ST6GALN	0.724761	0.275239	1.684052	1.685595	420.1128	249.2331
MAFG-AS	0.72483	0.27517	0.565357	0.563605	81.1329	143.9613
LOC10014	0.724866	0.275134	0.278051	0.247604	3.334229	13.49637
MIR503HG	0.724866	0.275134	0.278051	0.247604	3.334229	13.49637
OGFOD2	0.724911	0.275089	0.589516	0.5889	227.839	386.8961
TMEM161	0.725012	0.274988	1.995766	2.017168	54.45907	26.99275
BEND3	0.726441	0.273559	0.590977	0.59045	265.6269	449.8792
DLL1	0.726764	0.273236	1.713167	1.71655	210.0564	122.3671
WDR19	0.726786	0.273214	0.590856	0.590359	276.741	468.7741
ZNF121	0.727399	0.272601	1.709136	1.712231	223.3933	130.465
CACNA1I	0.727427	0.272573	2.831319	2.996677	18.89396	6.298308
PRKAG2-A	0.727427	0.272573	2.831319	2.996677	18.89396	6.298308
STK31	0.727623	0.272377	2.626389	2.742806	22.22819	8.097825
C12orf80	0.727772	0.272228	0.278602	0.247604	3.334229	13.49637
DMGDH	0.727772	0.272228	0.278602	0.247604	3.334229	13.49637
ERICH5	0.728839	0.271161	0.579332	0.578213	122.2551	211.4432
TMEM125	0.729315	0.270685	0.590668	0.590436	603.4954	1022.125

CCDC189	0.729318	0.270682	0.534465	0.531338	44.45638	83.67752
GOLGA8F	0.729727	0.270273	2.789719	2.9474	19.40521	6.577233
CLN6	0.729812	0.270188	0.591891	0.591435	301.192	509.2632
LYN	0.730139	0.269861	1.706654	1.706912	2658.492	1557.482
TADA2A	0.730195	0.269805	0.586413	0.585547	162.2658	277.1256
LRCH3	0.730513	0.269487	0.590871	0.590298	240.0645	406.6908
TMEM117	0.730794	0.269206	0.590082	0.589411	208.945	354.5048
C5	0.730915	0.269085	0.543998	0.541197	51.12484	94.47462
C7orf61	0.73175	0.26825	1.854221	1.865255	82.24431	44.08816
C15orf40	0.731838	0.268162	0.581766	0.580696	129.2903	222.6542
RABEP2	0.731896	0.268104	1.67729	1.678729	450.1209	268.128
TMEM191	0.732411	0.267589	0.25033	0.21279	2.456215	11.57989
YEATS4	0.733279	0.266721	0.592673	0.592212	297.8578	502.9649
KLK8	0.733626	0.266374	0.593413	0.593053	388.9934	655.9238
LRP10	0.733753	0.266247	1.708819	1.709049	2998.583	1754.529
CSF1	0.733864	0.266136	0.584224	0.584115	1269.23	2172.916
ISCU	0.733879	0.266121	0.589387	0.589213	806.8834	1369.432
CTBP1-AS	0.733987	0.266013	0.310384	0.285237	4.445638	15.61081
C16orf59	0.733989	0.266011	0.577737	0.576464	108.9181	188.9492
TMEM67	0.734068	0.265932	0.590334	0.589629	198.9423	337.4094
UROS	0.734151	0.265849	0.588765	0.588603	868.0109	1474.704
CNIH2	0.73421	0.26579	2.101333	2.133009	42.23356	19.79468
LOC10013	0.73421	0.26579	2.101333	2.133009	42.23356	19.79468
UBE2Q2	0.734503	0.265497	1.68209	1.682617	1241.445	737.8018
PXYLP1	0.734769	0.265231	0.592761	0.592469	471.2377	795.3863
LEPR	0.735599	0.264401	1.686958	1.687384	1553.073	920.3988
CPAMD8	0.735971	0.264029	1.674509	1.67522	922.47	550.6521
CLTB	0.736054	0.263946	1.72598	1.726125	5041.354	2920.615
PRSS53	0.73689	0.26311	2.00616	2.0289	51.12484	25.19323
TMEM30B	0.736993	0.263007	1.680585	1.68111	1240.333	737.8018
PLEKHG5	0.737153	0.262847	1.671992	1.672917	692.4082	413.8888
YIPF2	0.737217	0.262783	1.699988	1.700264	2458.438	1445.912
KHDRBS3	0.737292	0.262708	1.671803	1.672971	547.9249	327.512
IBTK	0.737429	0.262571	0.581772	0.581689	1700.457	2923.315
ZFP3	0.737591	0.262409	0.567646	0.565847	78.91008	139.4625
RPP38	0.73803	0.26197	0.593611	0.593174	310.0833	522.7596
IGDCC4	0.738042	0.261958	1.672093	1.673394	492.3544	294.221
COL26A1	0.738489	0.261511	0.594237	0.593924	445.6752	750.3984
NIFK-AS1	0.739002	0.260998	0.49554	0.489931	25.56242	52.18598
BASP1	0.739103	0.260897	1.704631	1.704869	2836.317	1663.653
PPM1D	0.739908	0.260092	0.59361	0.59303	241.1759	406.6908
ZNF165	0.740083	0.259917	1.669857	1.670841	647.9518	387.7958
CGB	0.740492	0.259508	8.93855	461.1236	4.601236	0
TIGD7	0.740888	0.259112	1.705474	1.708832	204.4994	119.6679
CPZ	0.741607	0.258393	0.586112	0.585127	140.0376	239.3357
STC1	0.742158	0.257842	0.594516	0.594221	465.6806	783.6895
PCDHB16	0.742472	0.257528	2.074353	2.104052	43.34497	20.59547
TMEM2	0.742565	0.257435	1.727869	1.727995	5791.555	3351.6
PHLDB3	0.7426	0.2574	1.679907	1.682111	301.192	179.0519
EIF2D	0.742684	0.257316	0.593956	0.593701	537.9222	906.0566
DHRS4	0.742718	0.257282	0.590219	0.589387	168.3786	285.6913
YPEL1	0.742727	0.257273	1.868209	1.880698	74.46444	39.58937
NPAS1	0.742782	0.257218	1.803051	1.811522	97.80404	53.9855
CNOT10	0.742937	0.257063	0.588779	0.588642	1004.714	1706.842
MOSPD2	0.743156	0.256844	0.591308	0.591131	781.3209	1321.745
STRA6	0.743164	0.256836	0.510103	0.505405	30.00806	59.38405

IGSF8	0.743195	0.256805	1.703072	1.703301	2968.575	1742.832
CACHD1	0.74326	0.25674	0.578633	0.577292	103.3611	179.0519
IFT43	0.74327	0.25673	0.595325	0.594949	364.5423	612.7354
DGUOK	0.743457	0.256543	1.672601	1.673232	1014.717	606.4371
GPLD1	0.744027	0.255973	0.433927	0.423691	13.33691	31.49154
ZNF253	0.74413	0.25587	1.810377	1.819543	93.3584	51.30422
TRIM61	0.744156	0.255844	1.884147	1.897795	70.0188	36.89009
PBDC1	0.744434	0.255566	0.595551	0.595127	323.4202	543.454
NOP14-AS	0.744591	0.255409	0.516191	0.511816	32.23088	62.98308
NFATC4	0.745094	0.254906	2.395547	2.469102	26.67383	10.7971
RTP4	0.745094	0.254906	2.395547	2.469102	26.67383	10.7971
WDR44	0.745348	0.254652	1.667442	1.668208	843.5599	505.6642
RPS10P7	0.745791	0.254209	0.52647	0.522556	36.68763	70.21714
CPB2-AS1	0.745908	0.254092	0.284107	0.253321	3.345343	13.23544
SPDEF	0.746047	0.253953	2.394229	2.469102	26.67383	10.7971
BTG3	0.746792	0.253208	1.694965	1.695233	2498.449	1473.804
TSPAN33	0.747049	0.252951	1.666403	1.667734	475.6833	285.2234
ADAMTS1	0.747174	0.252826	0.533032	0.529447	40.01074	75.5797
REPS2	0.747998	0.252002	0.596022	0.595742	499.034	837.675
VASH2	0.748327	0.251673	2.064703	2.093994	43.34497	20.69444
LCAT	0.748641	0.251359	1.892269	1.906749	66.91797	35.09057
AGR2	0.74904	0.25096	0.443862	0.43417	14.44832	33.29106
TNK1	0.749249	0.250751	0.586427	0.585389	131.1463	224.0398
LINC0156	0.749423	0.250577	0.547811	0.545005	50.01343	91.77535
ZNF514	0.749445	0.250555	0.58787	0.586871	137.8148	234.8369
ADORA1	0.750009	0.249991	0.597339	0.596977	385.6591	646.0265
SLC2A6	0.750056	0.249944	1.663526	1.664347	780.2095	468.7741
PCDHAC2	0.750258	0.249742	1.914541	1.930754	62.02777	32.12137
PRRT3	0.750401	0.249599	0.559194	0.556907	61.12753	109.7705
C11orf96	0.750784	0.249216	3.330395	3.698197	13.33691	3.599033
RAET1E	0.750784	0.249216	3.330395	3.698197	13.33691	3.599033
GPM6A	0.750997	0.249003	0.24633	0.206607	2.222819	10.7971
LOC28408	0.750997	0.249003	0.24633	0.206607	2.222819	10.7971
TIRAP	0.751368	0.248632	0.569413	0.567571	75.57585	133.1642
CDCA3	0.751416	0.248584	1.6626	1.663539	669.0686	402.192
SCN2B	0.751619	0.248381	4.063763	4.921554	10.00269	2.024456
ENOX2	0.751635	0.248365	0.597325	0.596898	320.086	536.2559
TEX261	0.751716	0.248284	0.583134	0.583059	1828.269	3135.658
RPL13AP2	0.751818	0.248182	2.49024	2.582903	23.6508	9.150542
ATP5S	0.751845	0.248155	0.573014	0.571324	82.24431	143.9613
ODF3B	0.751903	0.248097	0.45246	0.443575	15.55973	35.09057
PHLPP2	0.751998	0.248002	0.597472	0.597068	338.9799	567.7475
SLC43A3	0.752217	0.247783	1.666768	1.667377	1059.173	635.2294
SLC35A1	0.752582	0.247418	0.597811	0.597337	294.5235	493.0676
GLS2	0.752649	0.247351	0.577508	0.576005	92.247	160.157
KLRD1	0.752654	0.247346	0.286403	0.255548	3.356457	13.16346
SIL1	0.752773	0.247227	0.588712	0.588598	1194.765	2029.855
TSPAN3	0.752794	0.247206	1.703553	1.703746	3613.193	2120.73
CSRNP2	0.75318	0.24682	0.585519	0.585433	1597.096	2728.067
CA12	0.753242	0.246758	1.702323	1.702523	3417.584	2007.361
MYO5A	0.75336	0.24664	0.597791	0.597373	327.8658	548.8526
TMEM185	0.753511	0.246489	1.661556	1.662354	800.2149	481.3707
FAM64A	0.753562	0.246438	1.663363	1.66415	789.1008	474.1726
EIF4EBP3	0.75372	0.24628	0.526609	0.522655	35.63179	68.18368
KCNK9	0.753931	0.246069	2.497518	2.592216	23.3396	8.997583
AMACR	0.754007	0.245993	0.553867	0.551284	54.22567	98.37058

TNFRSF1	0.754571	0.245429	0.582274	0.582208	2147.243	3688.109
DNAJB5	0.754741	0.245259	1.661146	1.662621	432.3383	260.0302
CFAP53	0.754848	0.245152	0.316541	0.291106	4.445638	15.29589
LOC44046	0.754848	0.245152	0.316541	0.291106	4.445638	15.29589
ITK	0.755267	0.244733	8.770684	445.5638	4.445638	0
LINC0070	0.755267	0.244733	8.770684	445.5638	4.445638	0
PNMAL2	0.755267	0.244733	8.770684	445.5638	4.445638	0
SERPINB	0.755267	0.244733	8.770684	445.5638	4.445638	0
SHC2	0.755267	0.244733	8.770684	445.5638	4.445638	0
CCBL2	0.755662	0.244338	0.596454	0.596232	626.6238	1050.981
ST3GAL3	0.75573	0.24427	1.683087	1.685771	241.1759	143.0616
ARHGAP3	0.755973	0.244027	0.597341	0.597076	516.8055	865.5675
H2BFXP	0.756061	0.243939	5.296872	7.951741	7.224162	0.899758
ZFP36	0.757131	0.242869	1.660549	1.661316	814.6632	490.3683
HSPA12B	0.757132	0.242868	0.317004	0.291106	4.445638	15.29589
LOC10050	0.757132	0.242868	0.317004	0.291106	4.445638	15.29589
MYO15A	0.757132	0.242868	0.317004	0.291106	4.445638	15.29589
GDPGP1	0.757217	0.242783	0.467597	0.459761	17.78255	38.68961
KIAA0087	0.757266	0.242734	0.105186	0.002024	0	4.930676
CHST15	0.757685	0.242315	1.674471	1.674864	1668.226	996.0324
SMARCB1	0.757879	0.242121	0.591599	0.591469	1055.839	1785.12
ALDH4A1	0.758371	0.241629	1.659013	1.659828	764.6498	460.6763
ST6GALN	0.758517	0.241483	1.666647	1.668714	312.3061	187.1497
NUDT22	0.758681	0.241319	0.59802	0.597742	484.5746	810.6822
ATP1A3	0.759086	0.240914	1.705415	1.709401	172.2685	100.7729
LOC10010	0.75917	0.24083	5.275081	7.951741	7.224162	0.899758
IGFLR1	0.759206	0.240794	0.47417	0.466775	18.89396	40.48912
IFITM2	0.759738	0.240262	1.664364	1.664924	1126.569	676.6452
GRAMD3	0.76006	0.23994	1.679162	1.679492	1970.529	1173.285
MNX1-AS	0.760099	0.239901	0.395059	0.380336	8.891277	23.39372
MAPKAPK	0.760237	0.239763	1.692324	1.692553	2916.339	1723.037
ACHE	0.760384	0.239616	1.682951	1.685872	224.5047	133.1642
AKR1B15	0.76039	0.23961	8.670097	445.5638	4.445638	0
C20orf202	0.76039	0.23961	8.670097	445.5638	4.445638	0
C2orf66	0.76039	0.23961	8.670097	445.5638	4.445638	0
CD5L	0.76039	0.23961	8.670097	445.5638	4.445638	0
COL22A1	0.76039	0.23961	8.670097	445.5638	4.445638	0
DACT3-AS	0.76039	0.23961	8.670097	445.5638	4.445638	0
DPRXP4	0.76039	0.23961	8.670097	445.5638	4.445638	0
GP5	0.76039	0.23961	8.670097	445.5638	4.445638	0
GRIK4	0.76039	0.23961	8.670097	445.5638	4.445638	0
HAPLN2	0.76039	0.23961	8.670097	445.5638	4.445638	0
HIST3H2E	0.76039	0.23961	8.670097	445.5638	4.445638	0
HSD52	0.76039	0.23961	8.670097	445.5638	4.445638	0
HTR2C	0.76039	0.23961	8.670097	445.5638	4.445638	0
IL22RA2	0.76039	0.23961	8.670097	445.5638	4.445638	0
KIF25	0.76039	0.23961	8.670097	445.5638	4.445638	0
LHX1	0.76039	0.23961	8.670097	445.5638	4.445638	0
LINC0110	0.76039	0.23961	8.670097	445.5638	4.445638	0
LINC0153	0.76039	0.23961	8.670097	445.5638	4.445638	0
LOC10012	0.76039	0.23961	8.670097	445.5638	4.445638	0
LRRC36	0.76039	0.23961	8.670097	445.5638	4.445638	0
MGC3280	0.76039	0.23961	8.670097	445.5638	4.445638	0
MSH4	0.76039	0.23961	8.670097	445.5638	4.445638	0
PLA2G7	0.76039	0.23961	8.670097	445.5638	4.445638	0
SAMSN1	0.76039	0.23961	8.670097	445.5638	4.445638	0

TEX40	0.76039	0.23961	8.670097	445.5638	4.445638	0
ZNF334	0.76039	0.23961	8.670097	445.5638	4.445638	0
ETNK2	0.760581	0.239419	1.662612	1.663202	1062.508	638.8284
HAP1	0.760595	0.239405	2.084697	2.116948	40.01074	18.89492
ARMCX5-(	0.760714	0.239286	0.578363	0.576829	89.05725	154.3985
METTL8	0.760819	0.239181	0.599385	0.598901	281.909	470.7176
C2CD5	0.760847	0.239153	0.597976	0.597735	567.9303	950.1448
COA7	0.761151	0.238849	0.598762	0.598491	503.4908	841.274
LOC72839	0.761382	0.238618	2.88563	3.084217	16.67114	5.39855
SLC10A5	0.76139	0.23861	0.395331	0.380336	8.891277	23.39372
GTF2IRD2	0.761435	0.238565	0.584295	0.582981	105.0615	180.2216
CDIPT-AS	0.761488	0.238512	0.194875	0.138312	1.11141	8.097825
CSTF3-AS	0.761488	0.238512	0.194875	0.138312	1.11141	8.097825
LOC10192	0.761488	0.238512	0.194875	0.138312	1.11141	8.097825
NME5	0.761488	0.238512	0.194875	0.138312	1.11141	8.097825
ZCCHC7	0.7616	0.2384	1.656717	1.657539	754.6471	455.2777
PTPRC	0.761649	0.238351	2.644402	2.776802	20.00537	7.198066
FAM66E	0.761772	0.238228	8.650922	444.4524	4.434524	0
NPIPB11	0.762582	0.237418	1.733733	1.73927	131.2575	75.46273
TMEM54	0.762917	0.237083	1.671126	1.671517	1664.892	996.0324
CARNMT1	0.763105	0.236895	1.655795	1.657316	414.5558	250.1328
KDM5B	0.763111	0.236889	1.706452	1.706606	4442.304	2603.001
CDK5R2	0.763201	0.236799	2.882244	3.084217	16.67114	5.39855
FAM110C	0.763558	0.236442	1.658186	1.659813	382.3249	230.3381
CCDC153	0.763679	0.236321	0.582723	0.58131	97.80404	168.2548
CAND2	0.763714	0.236286	1.738815	1.744661	125.5893	71.98066
DLX6-AS1	0.76374	0.23626	0.560617	0.558152	57.74884	103.4722
ADAM20P	0.764018	0.235982	1.817609	1.827958	82.24431	44.98792
LOC10537	0.764142	0.235858	0.573862	0.572052	76.68726	134.064
TSPO	0.764282	0.235718	1.690554	1.690778	2974.132	1759.027
STX5	0.764397	0.235603	1.669007	1.669413	1598.207	957.3428
DNAJC25-	0.764519	0.235481	8.612571	442.2296	4.412296	0
TMEM140	0.764995	0.235005	1.674826	1.677428	244.5101	145.7608
DAPL1	0.765693	0.234307	0.195872	0.138312	1.11141	8.097825
LINC0123	0.765693	0.234307	0.195872	0.138312	1.11141	8.097825
CCDC110	0.7657	0.2343	0.504135	0.498524	25.56242	51.28622
SPC25	0.766082	0.233918	1.654778	1.655567	772.563	466.6416
CCDC121	0.766102	0.233898	0.5579	0.555333	54.45907	98.07366
STAMBPL	0.766728	0.233272	1.828498	1.839508	77.79867	42.28864
PARD3B	0.766866	0.233134	0.600859	0.600513	390.1048	649.6255
MPHOSPH	0.767129	0.232871	0.594867	0.594722	929.9831	1563.735
UTP15	0.767231	0.232769	0.60121	0.600869	401.23	667.7556
ZDBF2	0.767336	0.232664	0.410599	0.397278	10.00269	25.19323
EPS8L3	0.767377	0.232623	8.530367	445.5638	4.445638	0
FAM196B	0.767377	0.232623	8.530367	445.5638	4.445638	0
FXYD1	0.767377	0.232623	8.530367	445.5638	4.445638	0
HYAL4	0.767377	0.232623	8.530367	445.5638	4.445638	0
KEL	0.767377	0.232623	8.530367	445.5638	4.445638	0
MGAT4EF	0.767377	0.232623	8.530367	445.5638	4.445638	0
MUM1L1	0.767377	0.232623	8.530367	445.5638	4.445638	0
NCKAP1L	0.767377	0.232623	8.530367	445.5638	4.445638	0
NRXN2	0.767377	0.232623	8.530367	445.5638	4.445638	0
ODF3L1	0.767377	0.232623	8.530367	445.5638	4.445638	0
PGLYRP4	0.767377	0.232623	8.530367	445.5638	4.445638	0
SNAP25-A	0.767377	0.232623	8.530367	445.5638	4.445638	0
TREM1	0.767377	0.232623	8.530367	445.5638	4.445638	0

SERPINI1	0.767687	0.232313	0.537421	0.533806	38.89934	72.88042
CCNY	0.767899	0.232101	1.690437	1.690648	3151.88	1864.299
CBWD3	0.768502	0.231498	1.656302	1.658054	353.8728	213.4227
FOXO4	0.768646	0.231354	1.655302	1.656988	366.7652	221.3405
WNT10A	0.768977	0.231023	1.664884	1.667184	274.5182	164.6558
HYI	0.769247	0.230753	1.750352	1.757041	112.2524	63.88284
SMPD1	0.769393	0.230607	0.601921	0.601557	374.545	622.6327
MAP3K5	0.769572	0.230428	1.652739	1.653463	848.0055	512.8622
SPPL2B	0.769691	0.230309	0.602369	0.601903	297.8578	494.8671
IMPDH1	0.77008	0.22992	1.699694	1.699857	4167.786	2451.841
HOXB5	0.770214	0.229786	1.806145	1.816339	83.35572	45.88767
ZNF850	0.770274	0.229726	2.241811	2.296895	30.0414	13.07349
ANKRD16	0.770791	0.229209	0.590536	0.58938	118.9208	201.7798
SLC15A2	0.770837	0.229163	2.398595	2.480152	24.67329	9.942329
FZD7	0.770875	0.229125	1.657599	1.658137	1154.755	696.4129
CDKL1	0.771266	0.228734	1.963037	1.984802	50.01343	25.19323
CCDC169	0.771295	0.228705	8.516695	436.6726	4.356726	0
TOP3B	0.771463	0.228537	0.601902	0.601579	416.7786	692.8139
SCHLAP1	0.771552	0.228448	0.488874	0.482222	21.09455	43.75525
HLA-L	0.771743	0.228257	2.398602	2.477953	24.53992	9.897341
ADO	0.771891	0.228109	0.599321	0.599127	720.1934	1202.077
SPRED3	0.771974	0.228026	1.681809	1.685218	195.6081	116.0688
ZNF226	0.771983	0.228017	1.652013	1.653751	360.0967	217.7415
TNFSF15	0.772443	0.227557	1.742084	1.748351	116.3535	66.54612
KSR1	0.772594	0.227406	0.597089	0.596186	154.4859	259.1304
ITPA	0.772805	0.227195	0.602457	0.602142	432.3383	718.0071
PTGFRN	0.772941	0.227059	1.664677	1.665071	1601.541	961.8416
CYP2S1	0.773029	0.226971	0.603401	0.602968	320.086	530.8574
MOSPD3	0.773533	0.226467	1.646961	1.648102	532.3652	323.0132
ADGRF3	0.773661	0.226339	0.347194	0.325455	5.557048	17.09541
FTX	0.773661	0.226339	0.347194	0.325455	5.557048	17.09541
HSD11B2	0.773742	0.226258	2.046231	2.07689	41.12215	19.79468
NUDT18	0.774309	0.225691	0.580734	0.579044	83.35572	143.9613
WARS2	0.774562	0.225438	0.598403	0.597578	165.6	277.1256
FAM104B	0.774633	0.225367	0.598527	0.597707	166.7114	278.9251
ZBTB40	0.77465	0.22535	0.600944	0.600721	612.3867	1019.426
SAMD10	0.774687	0.225313	1.64844	1.649922	411.2215	249.2331
SLC7A14	0.774731	0.225269	0.198372	0.140972	1.11141	7.944866
LINC0048	0.774791	0.225209	0.20038	0.1437	1.144752	8.025844
C9orf64	0.774884	0.225116	1.644737	1.645852	552.3706	335.6098
NLK	0.77498	0.22502	1.646959	1.648301	452.3437	274.4263
ACOT1	0.775142	0.224858	0.560045	0.557442	53.64774	96.24715
SIDT2	0.775468	0.224532	1.655207	1.655726	1190.32	718.9069
S100A6	0.775489	0.224511	1.680572	1.68081	2741.847	1631.262
DCHS2	0.775523	0.224477	0.347591	0.325455	5.557048	17.09541
PMS2P9	0.776045	0.223955	0.598363	0.597474	156.6421	262.1806
TNNI3	0.7762	0.2238	0.604139	0.603782	387.8819	642.4274
MCAT	0.776227	0.223773	0.602857	0.60229	240.0645	398.5929
CDON	0.776296	0.223704	0.598381	0.597527	160.043	267.849
C1GALT1	0.776405	0.223595	0.603538	0.603213	419.0014	694.6224
ATP2A1	0.776563	0.223437	2.389113	2.468978	24.45101	9.897341
XXYLT1	0.776936	0.223064	0.603801	0.603316	281.1866	466.0748
CAT	0.777134	0.222866	1.645222	1.646087	699.0766	424.6859
MAP2K1	0.777682	0.222318	1.67965	1.679884	2779.635	1654.656
SLC25A53	0.778228	0.221772	4.102289	5.078839	9.180243	1.799517
UHRF2	0.778242	0.221758	1.692827	1.692993	4102.213	2423.049

MYH9	0.778447	0.221553	1.813581	1.813594	66216.67	36511.29
DYNC2LI1	0.778739	0.221261	0.604676	0.604171	274.5182	454.3779
AZU1	0.778797	0.221203	3.566369	4.105931	11.1141	2.699275
CT62	0.778797	0.221203	3.566369	4.105931	11.1141	2.699275
GCKR	0.778797	0.221203	3.566369	4.105931	11.1141	2.699275
VN1R1	0.778797	0.221203	3.566369	4.105931	11.1141	2.699275
CHMP4C	0.779028	0.220972	1.661893	1.662268	1671.204	1005.372
C11orf74	0.779036	0.220964	0.570042	0.567847	63.35035	111.57
TNFAIP8	0.77919	0.22081	1.650879	1.652907	307.8605	186.25
MACROD1	0.779556	0.220444	1.650806	1.65274	316.7517	191.6485
CREB3L4	0.779973	0.220027	0.604564	0.604064	272.2953	450.7789
ADAMTS1	0.780817	0.219183	1.648818	1.650685	326.7544	197.9468
IRF2	0.781191	0.218809	1.646954	1.648711	345.6484	209.6437
CXCL10	0.781427	0.218573	3.557979	4.105931	11.1141	2.699275
GGN	0.781427	0.218573	3.557979	4.105931	11.1141	2.699275
FBN3	0.781516	0.218484	0.603561	0.602883	204.4994	339.2089
PCYT2	0.782124	0.217876	0.59594	0.595826	1200.322	2014.559
GDF15	0.782361	0.217639	0.565086	0.565072	10284.98	18201.21
HOXB-AS	0.782921	0.217079	0.455794	0.446227	14.44832	32.3913
TNFAIP8L	0.783265	0.216735	8.304456	432.2269	4.312269	0
YAE1D1	0.783434	0.216566	0.60548	0.604969	265.6269	439.0821
MIPOL1	0.784181	0.215819	1.672344	1.675643	194.4967	116.0688
OR7E14P	0.785179	0.214821	0.296229	0.264521	3.334229	12.63261
COQ5	0.785702	0.214298	0.604811	0.604559	540.1451	893.46
TCIRG1	0.785783	0.214217	1.748669	1.756213	101.1383	57.58453
PLCB2	0.785867	0.214133	2.500324	2.605728	21.11678	8.097825
WNT5B	0.785867	0.214133	2.500324	2.605728	21.11678	8.097825
PLA2G10	0.785954	0.214046	2.136745	2.179049	33.34229	15.29589
ME1	0.786024	0.213976	1.663128	1.663439	2020.543	1214.674
MRPL38	0.786031	0.213969	0.603924	0.603711	651.286	1078.81
DNM1P35	0.78616	0.21384	0.372182	0.353265	6.668457	18.89492
SNORA18	0.786414	0.213586	2.135976	2.179049	33.34229	15.29589
SALL4	0.786436	0.213564	0.471719	0.463361	16.67114	35.99033
RASSF10	0.786659	0.213341	0.544085	0.540478	38.89934	71.98066
RAB39A	0.786885	0.213115	0.471875	0.463361	16.67114	35.99033
CPS1	0.786969	0.213031	0.544038	0.540478	38.89934	71.98066
KIAA1919	0.787063	0.212937	0.592644	0.591353	106.2619	179.6997
ZFP28	0.787142	0.212858	1.644901	1.64694	302.3034	183.5507
ZNF143	0.787167	0.212833	1.636743	1.637856	533.4766	325.7125
C16orf74	0.78717	0.21283	1.657391	1.660041	234.5074	141.2621
JHDM1D-/	0.787264	0.212736	1.724746	1.730862	116.8091	67.48187
GPATCH3	0.787328	0.212672	0.603155	0.602379	175.6027	291.5217
ESPNL	0.787448	0.212552	0.297038	0.265276	3.334229	12.59662
LINC0112	0.78761	0.21239	0.478562	0.470704	17.78255	37.78985
SCNN1D	0.78761	0.21239	0.478562	0.470704	17.78255	37.78985
SNORA81	0.787716	0.212284	0.372528	0.353265	6.668457	18.89492
SOX30	0.787716	0.212284	0.372528	0.353265	6.668457	18.89492
NUP50-AS	0.787725	0.212275	0.604452	0.603749	193.3853	320.314
ARIH2OS	0.787819	0.212181	0.471725	0.463323	16.58223	35.80138
ASB16-AS	0.787928	0.212072	0.599604	0.598629	140.0376	233.9372
THY1	0.787986	0.212014	0.478708	0.470704	17.78255	37.78985
ING3	0.787999	0.212001	0.60495	0.604274	201.1651	332.9106
SERTAD4	0.788246	0.211754	1.639027	1.640589	382.3249	233.0374
FMN1	0.788266	0.211734	1.637959	1.639557	379.7242	231.5978
GOLGA8K	0.788281	0.211719	8.267417	422.2242	4.212242	0
HSPB2-C1	0.788281	0.211719	8.267417	422.2242	4.212242	0

PRKAA1	0.788347	0.211653	0.600702	0.600553	919.1357	1530.489
EPC2	0.788372	0.211628	0.607036	0.606698	401.2189	661.3224
MRPS31	0.788476	0.211524	0.605598	0.604958	212.2792	350.9057
ARHGAP3	0.7885	0.2115	1.650831	1.651261	1424.827	862.8682
ALKBH8	0.788528	0.211472	0.596552	0.595379	118.9208	199.7463
KCTD1	0.788622	0.211378	0.603964	0.603767	702.4109	1163.387
DHRSX	0.788747	0.211253	0.605898	0.605274	217.8363	359.9033
DOCK10	0.788793	0.211207	1.83933	1.85261	66.68457	35.99033
RGS5	0.78895	0.21105	0.528723	0.524118	31.11947	59.38405
TTLL11	0.789174	0.210826	1.753763	1.761585	96.69263	54.88526
C12orf4	0.789281	0.210719	0.606402	0.605806	227.839	376.099
SUSD6	0.78949	0.21051	1.647415	1.647875	1344.806	816.0808
ZNF192P1	0.789492	0.210508	0.495627	0.489064	21.11678	43.1884
LINC0109	0.78955	0.21045	8.248242	421.1128	4.201128	0
BACE1-AS	0.789607	0.210393	0.495713	0.489064	21.11678	43.1884
GTPBP8	0.789635	0.210365	0.60816	0.607757	342.3142	563.2487
PPIL2	0.789722	0.210278	0.602966	0.602789	767.984	1274.058
SPOCK2	0.789783	0.210217	1.775096	1.784062	86.68995	48.58695
TCONS_0	0.789896	0.210104	0.297611	0.265276	3.334229	12.59662
CCDC74A	0.789994	0.210006	0.22236	0.170763	1.455947	8.574697
LINC0131	0.790046	0.209954	0.500645	0.494205	22.22819	44.98792
UFSP1	0.790083	0.209917	0.504976	0.49895	23.3396	46.78743
SMURF2	0.790412	0.209588	1.651565	1.651957	1594.873	965.4407
TGFB2-OT	0.790469	0.209531	2.792995	2.979652	16.29326	5.461533
SLC25A16	0.790585	0.209415	0.608134	0.607623	270.0725	444.4806
NEDD8-M	0.790918	0.209082	8.191501	425.5585	4.245585	0
LRIG3	0.790992	0.209008	0.608089	0.607644	304.5262	501.1654
PTRH1	0.791233	0.208767	1.652053	1.654698	236.7302	143.0616
TMEM205	0.791389	0.208611	0.593339	0.59326	1746.024	2943.109
LOC64455	0.7914	0.2086	2.671551	2.820495	17.78255	6.298308
SP5	0.7914	0.2086	2.671551	2.820495	17.78255	6.298308
TFAP2E	0.7914	0.2086	2.671551	2.820495	17.78255	6.298308
CD24	0.791545	0.208455	1.727525	1.727589	11298.03	6539.767
CD302	0.791678	0.208322	1.635126	1.636572	409.5433	250.2408
ADGRG4	0.791884	0.208116	2.962086	3.206696	14.44832	4.498792
TKFC	0.792064	0.207936	0.604464	0.603639	167.8228	278.0253
KBTBD11	0.792151	0.207849	1.64484	1.646938	284.5209	172.7536
CMTM7	0.792216	0.207784	1.648253	1.64868	1427.05	865.5675
JAZF1	0.792476	0.207524	1.644995	1.64548	1230.33	747.6992
DOK1	0.792635	0.207365	1.689091	1.693588	147.8175	87.27656
LOC64373	0.793217	0.206783	2.959187	3.206696	14.44832	4.498792
LOC33916	0.79353	0.20647	1.999399	2.026894	41.95571	20.69444
NSMCE1	0.793733	0.206267	0.602546	0.602395	923.5814	1533.188
N4BP2L1	0.793763	0.206237	1.729672	1.736366	107.8067	62.08332
TEX36	0.793908	0.206092	2.667121	2.820495	17.78255	6.298308
PER1	0.794024	0.205976	1.639374	1.641223	318.9745	194.3478
RNF219	0.794358	0.205642	1.630344	1.63131	617.9437	378.7982
LOC65484	0.794785	0.205215	0.392942	0.376241	7.779867	20.69444
TMEM81	0.794943	0.205057	1.719473	1.725829	113.3638	65.68236
PI4K2A	0.795044	0.204956	1.656499	1.65681	1996.092	1204.776
IRF8	0.795208	0.204792	1.872644	1.888887	57.7933	30.59178
SH2D3C	0.795285	0.204715	2.297855	2.366261	25.56242	10.7971
LGALS3	0.795498	0.204502	0.588765	0.588712	2648.489	4498.792
GTF2H1	0.795752	0.204248	0.605241	0.605053	722.4162	1193.979
DENND6B	0.795828	0.204172	0.567381	0.564723	53.34766	94.47462
PPP2R5B	0.796114	0.203886	1.639449	1.639978	1130.304	689.2149

STX1A	0.796124	0.203876	1.700325	1.705702	128.9235	75.5797
DNAJB2	0.796147	0.203853	1.675219	1.675404	3536.505	2110.833
UBE2Q2P	0.796683	0.203317	0.112734	0.002187	0	4.561775
MAFK	0.797055	0.202945	1.644017	1.644442	1447.055	879.9636
SRXN1	0.797092	0.202908	0.585746	0.585704	3233.09	5520.017
ZNF703	0.797185	0.202815	0.610178	0.609826	391.2162	641.5277
RUNDC3A	0.797507	0.202493	1.909982	1.92949	51.25821	26.56087
CTSO	0.797583	0.202417	1.668111	1.671757	174.4913	104.372
CBX4	0.79765	0.20235	0.589486	0.589432	2545.128	4317.94
WDPCP	0.798093	0.201907	0.564304	0.561518	50.01343	89.07607
HEIH	0.798095	0.201905	0.563265	0.560361	48.90202	87.27656
COMTD1	0.798286	0.201714	0.60818	0.607503	200.0537	329.3115
MBLAC2	0.798373	0.201627	0.598533	0.597339	114.4752	191.6485
INTS5	0.798394	0.201606	0.608193	0.607951	559.039	919.553
DIO1	0.798483	0.201517	0.112821	0.002218	0	4.498792
RS1	0.798483	0.201517	0.112821	0.002218	0	4.498792
POLG	0.798531	0.201469	1.644925	1.645331	1489.289	905.1569
FLT4	0.798552	0.201448	2.17304	2.22251	30.00806	13.49637
KPNA7	0.798626	0.201374	3.990259	4.919146	8.891277	1.799517
S100A9	0.798688	0.201312	1.64144	1.641895	1319.243	803.4842
BAG3	0.799144	0.200856	1.656098	1.656386	2150.578	1298.351
SPATA13	0.799235	0.200765	0.610614	0.610072	254.5128	417.1909
ZNF837	0.79924	0.20076	0.562074	0.559156	47.79061	85.47704
LOC10192	0.799251	0.200749	0.576921	0.574721	62.92801	109.5006
LPXN	0.799831	0.200169	0.560888	0.557899	46.6792	83.67752
MAGEA6	0.799889	0.200111	1.863638	1.879322	58.16006	30.94269
NAPG	0.800141	0.199859	0.607234	0.607037	701.2994	1155.29
PITPNM1	0.800208	0.199792	1.666521	1.666733	3030.814	1818.412
HECA	0.800314	0.199686	1.635101	1.635654	1071.399	655.024
USP36	0.800467	0.199533	0.593887	0.593818	1982.755	3339.003
HGH1	0.800555	0.199445	0.605078	0.60492	873.5679	1444.112
CD38	0.800737	0.199263	3.980829	4.919146	8.891277	1.799517
EDIL3	0.800737	0.199263	3.980829	4.919146	8.891277	1.799517
ENPP6	0.800737	0.199263	3.980829	4.919146	8.891277	1.799517
MIR155HC	0.800737	0.199263	3.980829	4.919146	8.891277	1.799517
RASD1	0.800737	0.199263	3.980829	4.919146	8.891277	1.799517
RBM44	0.800737	0.199263	3.980829	4.919146	8.891277	1.799517
SERP2	0.800737	0.199263	3.980829	4.919146	8.891277	1.799517
RABL2A	0.800818	0.199182	0.578718	0.576519	64.09499	111.1831
LACE1	0.80082	0.19918	0.55961	0.556587	45.56779	81.87801
NAPA-AS1	0.800885	0.199115	0.410465	0.395543	8.891277	22.49396
ZBED3-AS	0.800885	0.199115	0.410465	0.395543	8.891277	22.49396
MAGI3	0.801114	0.198886	1.633268	1.635177	308.9719	188.9492
FIZ1	0.801126	0.198874	0.611707	0.611278	321.1974	525.4588
RALB	0.801145	0.198855	1.642863	1.64327	1481.509	901.5578
JMJD7	0.801286	0.198714	0.592766	0.59126	91.03556	153.9756
TBC1D10A	0.801569	0.198431	1.626803	1.627718	626.835	385.0966
NUAK1	0.801726	0.198274	1.651588	1.651897	1987.2	1202.977
RPRD1A	0.801797	0.198203	0.597918	0.59783	1578.202	2639.891
C3orf52	0.801813	0.198187	1.627179	1.628649	395.6618	242.9347
ZNF383	0.801837	0.198163	1.691407	1.696437	132.8023	78.27897
ZCCHC24	0.801979	0.198021	1.635275	1.635795	1139.195	696.4129
LYPLA2P2	0.801992	0.198008	0.114386	0.002183	0	4.570772
PCDHA3	0.802046	0.197954	0.11393	0.002213	0	4.507789
TIGD1	0.802109	0.197891	1.660473	1.663928	181.1598	108.8708
MCEE	0.802449	0.197551	1.688257	1.693152	135.592	80.07849

BAIAP3	0.802536	0.197464	1.636178	1.638269	280.0752	170.9541
ANO4	0.80293	0.19707	0.114132	0.002218	0	4.498792
CCDC65	0.80293	0.19707	0.114132	0.002218	0	4.498792
CHRM5	0.80293	0.19707	0.114132	0.002218	0	4.498792
CRB2	0.80293	0.19707	0.114132	0.002218	0	4.498792
ERI3-IT1	0.80293	0.19707	0.114132	0.002218	0	4.498792
GYG2P1	0.80293	0.19707	0.114132	0.002218	0	4.498792
ITIH6	0.80293	0.19707	0.114132	0.002218	0	4.498792
KCNIP4	0.80293	0.19707	0.114132	0.002218	0	4.498792
LAMTOR5	0.80293	0.19707	0.114132	0.002218	0	4.498792
LINC0047(	0.80293	0.19707	0.114132	0.002218	0	4.498792
LOC10013	0.80293	0.19707	0.114132	0.002218	0	4.498792
LOC10192	0.80293	0.19707	0.114132	0.002218	0	4.498792
TM4SF19-	0.80293	0.19707	0.114132	0.002218	0	4.498792
WDR88	0.80293	0.19707	0.114132	0.002218	0	4.498792
HR	0.803453	0.196547	1.625736	1.627198	396.7732	243.8345
PTCD3	0.803457	0.196543	0.600891	0.600783	1269.23	2112.632
PCDHAC1	0.803564	0.196436	0.282173	0.245321	2.711839	11.08502
DUSP5P1	0.803671	0.196329	3.967415	4.919146	8.891277	1.799517
DHODH	0.803942	0.196058	0.602099	0.600943	120.0322	199.7463
FAM212B	0.804163	0.195837	0.593863	0.59236	91.13559	153.8587
SPRY4	0.804194	0.195806	1.644437	1.644796	1682.674	1023.025
EFCAB13	0.804368	0.195632	0.602486	0.601382	123.3665	205.1449
NUDT4P1	0.804511	0.195489	0.612291	0.611837	297.3021	485.9235
SPIRE2	0.80461	0.19539	0.549268	0.545492	37.78793	69.28139
NUDT6	0.804749	0.195251	0.592732	0.59112	86.68995	146.6606
THAP6	0.804973	0.195027	0.606388	0.605431	144.4832	238.6519
SLC13A5	0.805022	0.194978	1.9514	1.975936	44.45638	22.49396
ATOH8	0.805519	0.194481	1.63364	1.635809	272.2953	166.4553
TRO	0.806326	0.193674	0.545482	0.541541	35.56511	65.68236
ABCC2	0.806332	0.193668	2.381471	2.46883	22.22819	8.997583
DLGAP3	0.806332	0.193668	2.381471	2.46883	22.22819	8.997583
PRR4	0.806379	0.193621	0.115392	0.002205	0	4.525784
LINC0086(	0.806642	0.193358	1.637073	1.637499	1396.097	852.575
LINC0101	0.806727	0.193273	1.768828	1.77856	80.02149	44.98792
LINC0088	0.807032	0.192968	3.128255	3.464153	12.49224	3.599033
C7orf65	0.807036	0.192964	0.33503	0.308325	4.445638	14.44112
TRIM8	0.807373	0.192627	1.625172	1.625848	855.7854	526.3586
LOC1005(	0.807478	0.192522	0.335559	0.309288	4.445638	14.39613
LPL	0.807478	0.192522	0.335559	0.309288	4.445638	14.39613
TSNAXIP1	0.807478	0.192522	0.335559	0.309288	4.445638	14.39613
DBF4B	0.807676	0.192324	1.62443	1.626098	346.7598	213.2427
C2orf81	0.807707	0.192293	0.541339	0.537132	33.34229	62.08332
SUFU	0.808339	0.191661	1.6388	1.64151	224.5047	136.7633
ADTRP	0.808397	0.191603	0.539084	0.53473	32.23088	60.28381
GPRIN2	0.808415	0.191585	1.632961	1.635238	258.9584	158.3575
MORN2	0.808486	0.191514	0.612	0.611388	217.8363	356.3043
ZNF665	0.808643	0.191357	3.597401	4.234156	10.0138	2.357367
ZNF239	0.80885	0.19115	0.599303	0.597928	101.1383	169.1546
CATIP-AS	0.808977	0.191023	0.116004	0.002218	0	4.498792
GUCA2B	0.808977	0.191023	0.116004	0.002218	0	4.498792
LOC10193	0.808977	0.191023	0.116004	0.002218	0	4.498792
PRSS33	0.808977	0.191023	0.116004	0.002218	0	4.498792
SNORD17	0.808977	0.191023	0.116004	0.002218	0	4.498792
FGF7	0.809035	0.190965	0.115565	0.002249	0	4.435808
C14orf132	0.809286	0.190714	1.634393	1.636903	240.0756	146.6606

SLC45A1	0.809406	0.190594	0.336036	0.309288	4.445638	14.39613
ASB5	0.809792	0.190208	4.899443	7.340914	6.668457	0.899758
EFCC1	0.809792	0.190208	4.899443	7.340914	6.668457	0.899758
EN2	0.809792	0.190208	4.899443	7.340914	6.668457	0.899758
GPR78	0.809792	0.190208	4.899443	7.340914	6.668457	0.899758
LINC0144	0.809792	0.190208	4.899443	7.340914	6.668457	0.899758
LOC55422	0.809792	0.190208	4.899443	7.340914	6.668457	0.899758
MAGEC3	0.809792	0.190208	4.899443	7.340914	6.668457	0.899758
MCTS2P	0.809792	0.190208	4.899443	7.340914	6.668457	0.899758
NEUROG2	0.809792	0.190208	4.899443	7.340914	6.668457	0.899758
PDE6G	0.809792	0.190208	4.899443	7.340914	6.668457	0.899758
PITX3	0.809792	0.190208	4.899443	7.340914	6.668457	0.899758
CAMK2B	0.809905	0.190095	1.896817	1.916387	50.01343	26.09299
FSD2	0.810455	0.189545	2.018459	2.050834	37.43227	18.2471
RNF185	0.810828	0.189172	0.596321	0.596254	2093.896	3511.757
PDLIM4	0.810944	0.189056	1.620566	1.622143	363.4309	224.0398
PHEX	0.810958	0.189042	2.219974	2.279333	26.67383	11.69686
ZNF764	0.811121	0.188879	0.606893	0.605848	132.2577	218.3084
MKNK2	0.811131	0.188869	1.701537	1.701618	8410.036	4942.372
SH3TC1	0.811199	0.188801	0.58842	0.586546	73.35303	125.0664
ZNF385A	0.81126	0.18874	1.617705	1.61897	450.1209	278.0253
C14orf169	0.811428	0.188572	0.614548	0.614081	288.9665	470.5736
CARD8-AS	0.811579	0.188421	1.949283	1.973901	43.34497	21.9541
ESAM	0.811583	0.188417	2.116695	2.160848	31.11947	14.39613
NANOG	0.811656	0.188344	4.869825	7.269023	6.668457	0.908756
S100A1	0.811662	0.188338	0.598251	0.596822	95.58122	160.157
TRPT1	0.811688	0.188312	0.613942	0.613626	426.7813	695.5132
AKT3	0.812068	0.187932	2.115894	2.160848	31.11947	14.39613
TPST2	0.812643	0.187357	1.620819	1.621494	849.1169	523.6593
IGFBP2	0.812753	0.187247	1.692022	1.692118	6915.19	4086.702
TMED7-TI	0.812972	0.187028	1.625329	1.62587	1071.31	658.911
ZFP36L1	0.812978	0.187022	1.6644	1.664578	3537.617	2125.229
PLD6	0.81302	0.18698	0.609165	0.608203	143.3718	235.7367
NSUN5P2	0.813286	0.186714	0.601892	0.600605	105.8062	176.1727
HTR1D	0.813422	0.186578	4.871299	7.340914	6.668457	0.899758
KRT34	0.813422	0.186578	4.871299	7.340914	6.668457	0.899758
LINC0070	0.813422	0.186578	4.871299	7.340914	6.668457	0.899758
USP27X	0.813719	0.186281	0.597103	0.595585	90.02418	151.1594
LOC44124	0.813848	0.186152	0.51061	0.504288	22.22819	44.08816
WEE2-AS	0.813943	0.186057	0.468759	0.458972	14.44832	31.49154
RTN4	0.814042	0.185958	1.689758	1.689857	6700.688	3965.235
DNAJC15	0.814099	0.185901	0.476499	0.467545	15.55973	33.29106
PILRB	0.814184	0.185816	1.628854	1.631189	250.245	153.4088
NKX1-2	0.814226	0.185774	0.506118	0.499467	21.11678	42.28864
CASC18	0.814704	0.185296	0.267485	0.22537	2.222819	9.897341
ERICH6-A	0.814704	0.185296	0.267485	0.22537	2.222819	9.897341
KLHDC8A	0.814704	0.185296	0.267485	0.22537	2.222819	9.897341
SLC22A2	0.814704	0.185296	0.267485	0.22537	2.222819	9.897341
RTN4IP1	0.814786	0.185214	0.603797	0.602573	111.141	184.4505
MED19	0.815189	0.184811	0.615411	0.614868	247.8443	403.0917
MAGEA2E	0.815778	0.184222	0.117668	0.002254	0	4.426811
ARL14EP	0.81578	0.18422	0.616012	0.615617	341.2027	554.2511
SLK	0.815842	0.184158	0.599623	0.599547	1843.828	3075.374
MROH2A	0.815901	0.184099	2.503778	2.622612	18.89396	7.198066
ZNF229	0.815953	0.184047	1.658731	1.66274	155.5973	93.57486
MLYCD	0.816145	0.183855	0.608524	0.607509	133.3691	219.541

TGIF1	0.816163	0.183837	0.596993	0.59693	2212.816	3707.004
RNF112	0.817112	0.182888	1.960218	1.986634	41.12215	20.69444
TLR9	0.81737	0.18263	1.875228	1.89369	51.12484	26.99275
PCOLCE-/	0.817397	0.182603	0.268236	0.22537	2.222819	9.897341
RUNDC3E	0.817397	0.182603	0.268236	0.22537	2.222819	9.897341
CNTNAP1	0.817625	0.182375	1.621972	1.624188	264.5155	162.8563
ANXA2	0.817809	0.182191	1.738727	1.73876	21840.64	12561.04
EPS8L1	0.818032	0.181968	0.601005	0.600925	1700.457	2829.74
FLYWCH1	0.818045	0.181955	0.608263	0.608127	996.9344	1639.36
AK4	0.818082	0.181918	1.688043	1.688139	6897.408	4085.802
LTBP1	0.818445	0.181555	1.60997	1.611011	547.9249	340.1086
GCNT3	0.818468	0.181532	1.637496	1.640634	193.3853	117.8683
RPL23AP8	0.818619	0.181381	0.523731	0.51804	25.1512	48.55996
TRABD2A	0.81868	0.18132	1.690454	1.696281	114.4752	67.48187
RBL1	0.818742	0.181258	0.614721	0.614466	520.6954	847.4014
C8orf31	0.818819	0.181181	0.560811	0.55742	41.12215	73.78018
CREM	0.819179	0.180821	0.615764	0.615483	480.1289	780.0905
KRT5	0.819291	0.180709	3.07772	3.405642	12.28108	3.599033
APOBEC3	0.819309	0.180691	0.221397	0.163956	1.255893	7.710929
BIK	0.819372	0.180628	1.773155	1.784029	72.24162	40.48912
HMX1	0.819496	0.180504	0.607535	0.606406	120.0322	197.9468
RNF125	0.819934	0.180066	0.585768	0.58355	63.22809	108.3579
HYPK	0.82004	0.17996	0.617571	0.617142	313.7065	508.3275
SNHG17	0.820166	0.179834	0.618081	0.617624	298.9692	484.07
LOC10192	0.820243	0.179757	1.659446	1.663706	146.7061	88.17631
MIR22HG	0.82031	0.17969	1.61356	1.614261	804.6605	498.4661
NPRL2	0.820479	0.179521	1.609997	1.611334	419.0014	260.0302
RIC1	0.820733	0.179267	1.658736	1.658909	3655.426	2203.508
MTHFSD	0.820884	0.179116	0.607334	0.606199	117.8094	194.3478
RNF138	0.821007	0.178993	0.616532	0.61626	503.4685	816.9805
SHC3	0.821027	0.178973	3.068134	3.390245	12.22551	3.599033
NMNAT2	0.821506	0.178494	0.61714	0.61685	471.2377	763.9488
PALM2-AK	0.822122	0.177878	1.741889	1.751143	81.46632	46.5175
ITGA4	0.822401	0.177599	3.064421	3.390245	12.22551	3.599033
LOC10192	0.822401	0.177599	3.064421	3.390245	12.22551	3.599033
LOC10192	0.822401	0.177599	3.064421	3.390245	12.22551	3.599033
EPHA6	0.822646	0.177354	2.067242	2.106436	32.23088	15.29589
ZNF704	0.822646	0.177354	0.578328	0.575688	53.34766	92.67511
AGO3	0.822652	0.177348	1.606075	1.607185	509.0256	316.7149
DPH5	0.822871	0.177129	0.618113	0.617624	271.1839	439.0821
CHST1	0.822937	0.177063	2.283032	2.356798	23.3396	9.897341
COPZ2	0.823115	0.176885	0.553065	0.549061	35.56511	64.7826
ZNF443	0.823359	0.176641	1.711913	1.719219	95.4034	55.48809
FOXN3-AS	0.823465	0.176535	0.591536	0.589577	70.0188	118.7681
AHI1	0.823689	0.176311	1.607209	1.608115	613.4981	381.4975
WHAMM	0.823911	0.176089	1.608581	1.610122	362.1862	224.9396
ATP6V1G	0.823958	0.176042	0.346418	0.320088	4.601236	14.39613
PCDH10	0.82401	0.17599	2.696666	2.878726	15.55973	5.39855
SIGLEC15	0.82401	0.17599	2.696666	2.878726	15.55973	5.39855
RAB36	0.824072	0.175928	0.615182	0.614325	160.0652	260.561
IMPACT	0.824291	0.175709	0.617902	0.61762	485.686	786.3888
ITGA1	0.824616	0.175384	1.605737	1.606978	446.7867	278.0253
GARNL3	0.824617	0.175383	1.6942	1.700568	105.5839	62.08332
TESK2	0.825039	0.174961	1.678624	1.684307	116.698	69.28139
VPS9D1-A	0.825058	0.174942	0.618249	0.617627	215.6135	349.1062
RIMBP3	0.825207	0.174793	2.947971	3.223785	12.95904	4.012922

ATRNL1	0.825487	0.174513	1.604688	1.605781	505.6914	314.9154
ABCB6	0.82551	0.17449	0.607237	0.607132	1287.012	2119.831
PRUNE	0.825513	0.174487	0.610399	0.610265	1014.717	1662.753
TUBA8	0.82555	0.17445	0.618794	0.618469	413.4555	668.5204
GRHL3	0.825768	0.174232	0.575862	0.573094	50.01343	87.27656
MAST2	0.82595	0.17405	1.644276	1.644499	2710.728	1648.357
REPS1	0.826019	0.173981	0.615782	0.615577	670.18	1088.708
UBIAD1	0.826185	0.173815	0.617709	0.617446	512.3598	829.8111
GFOD1	0.826205	0.173795	1.610713	1.611332	904.6874	561.4492
AARSD1	0.826457	0.173543	0.619892	0.619452	304.8708	492.1678
MICAL2	0.826654	0.173346	1.673863	1.673974	5902.696	3526.153
ANO6	0.826714	0.173286	1.675633	1.67574	5999.389	3580.138
HTRA4	0.826722	0.173278	0.256315	0.208399	1.867168	8.997583
LOC10028	0.827219	0.172781	0.39021	0.370919	6.668457	17.99517
PXMP2	0.827347	0.172653	0.62062	0.620125	275.6296	444.4806
VDR	0.827355	0.172645	1.621305	1.621708	1427.05	879.9636
TMEM234	0.827451	0.172549	0.613592	0.612571	134.4806	219.541
SLX4	0.82749	0.17251	0.618365	0.617628	185.6054	300.5193
TMEM45B	0.827573	0.172427	0.613642	0.612621	134.4806	219.523
LOC10099	0.827757	0.172243	0.215921	0.15442	1.11141	7.252052
GRB7	0.827828	0.172172	1.616935	1.619316	238.9531	147.5604
SLC25A23	0.827841	0.172159	1.671433	1.671548	5543.711	3316.509
PXMP4	0.827878	0.172122	0.620889	0.620425	294.1234	474.0737
SLC23A2	0.828005	0.171995	0.619359	0.61907	474.5719	766.5941
HNF4G	0.828176	0.171824	0.542762	0.538006	30.00806	55.78501
C15orf65	0.828309	0.171691	0.542675	0.538006	30.00806	55.78501
EGOT	0.828309	0.171691	0.542675	0.538006	30.00806	55.78501
BCAT1	0.828741	0.171259	0.595498	0.595452	3000.806	5039.546
L3MBTL2	0.828779	0.171221	0.61943	0.619135	454.5665	734.2028
C1orf74	0.828813	0.171187	0.60488	0.603443	94.46981	156.5579
FAH	0.828823	0.171177	0.615851	0.615658	697.9652	1133.695
AQP6	0.829256	0.170744	0.34586	0.319231	4.456752	13.98224
CNNM2	0.829287	0.170713	0.587958	0.58571	61.12753	104.372
LPAR6	0.82966	0.17034	0.21599	0.154035	1.100295	7.198066
RANBP10	0.829908	0.170092	0.620599	0.620257	392.3276	632.5301
SLC7A7	0.830095	0.169905	0.604297	0.60276	90.02418	149.3599
TMEM184	0.830347	0.169653	0.620024	0.619743	486.7974	785.489
MPHOSPH	0.830379	0.169621	0.62134	0.620914	314.5289	506.5639
STYXL1	0.830594	0.169406	0.615489	0.615309	740.1988	1202.977
NUDT16P	0.830622	0.169378	1.836017	1.852551	53.34766	28.79227
ZNF529-A	0.830622	0.169378	1.836017	1.852551	53.34766	28.79227
ERVV-1	0.830655	0.169345	1.827573	1.843758	54.70358	29.66503
MRPL12	0.830753	0.169247	0.600078	0.60002	2330.626	3884.257
GUSB	0.830928	0.169072	0.619184	0.618929	525.6967	849.3718
PIWIL4	0.831176	0.168824	1.639134	1.643023	156.7088	95.37438
BLNK	0.831374	0.168626	0.586874	0.58457	58.90471	100.7729
A2M	0.831418	0.168582	0.217419	0.155577	1.11141	7.198066
CX3CR1	0.831418	0.168582	0.217419	0.155577	1.11141	7.198066
FKBP10	0.831418	0.168582	0.217419	0.155577	1.11141	7.198066
LOC10192	0.831418	0.168582	0.217419	0.155577	1.11141	7.198066
NXPH2	0.831418	0.168582	0.217419	0.155577	1.11141	7.198066
STKLD1	0.831418	0.168582	0.217419	0.155577	1.11141	7.198066
ZNF98	0.831551	0.168449	4.19533	5.586883	7.43533	1.322645
CYB5D2	0.831573	0.168427	0.587229	0.584895	58.92694	100.7549
PTPRH	0.831706	0.168294	1.803883	1.818282	58.90471	32.3913
SULF2	0.831981	0.168019	1.618355	1.618735	1529.3	944.7462

ITFG2	0.83225	0.16775	0.616175	0.615229	142.2604	231.2379
ALKBH4	0.832452	0.167548	0.62186	0.621325	250.0672	402.4799
PHF14	0.832493	0.167507	0.621951	0.621569	350.094	563.2487
PEX10	0.83259	0.16741	0.622036	0.621646	343.4256	552.4516
ZNF550	0.832601	0.167399	1.609337	1.609838	1137.906	706.8411
NIPSNAP3	0.832637	0.167363	1.599564	1.601024	374.545	233.9372
HIF3A	0.833023	0.166977	1.903676	1.926549	43.34497	22.49396
SLC35E2B	0.833093	0.166907	0.622822	0.622328	275.6963	443.014
LRRC27	0.833138	0.166862	0.569887	0.566635	42.68924	75.34576
NDUFAF4	0.83329	0.16671	0.6199	0.619641	511.2484	825.0784
ZZZ3	0.83332	0.16668	0.605752	0.605673	1747.136	2884.625
BPIFB1	0.833403	0.166597	2.373431	2.468649	20.00537	8.097825
CT45A3	0.833611	0.166389	0.122138	0.002395	0	4.165881
SKP1P2	0.83407	0.16593	0.218248	0.155383	1.11141	7.207064
BZRAP1-A	0.834104	0.165896	2.372243	2.468649	20.00537	8.097825
NUCB2	0.834244	0.165756	0.616093	0.615926	809.1062	1313.647
ZMAT3	0.8343	0.1657	1.598668	1.599422	737.4981	461.0991
TEP1	0.834388	0.165612	1.601699	1.602389	785.7666	490.3683
OARD1	0.834397	0.165603	0.622895	0.62243	287.8551	462.4758
ACOX1	0.834552	0.165448	0.620469	0.620215	527.3527	850.2806
PI4KA	0.834557	0.165443	0.594882	0.594842	3479.723	5849.833
C1orf167	0.834667	0.165333	0.2185	0.155577	1.11141	7.198066
MID1IP1-A	0.834667	0.165333	0.2185	0.155577	1.11141	7.198066
SLC51B	0.834667	0.165333	0.2185	0.155577	1.11141	7.198066
TMEM88	0.834667	0.165333	0.2185	0.155577	1.11141	7.198066
ACKR2	0.834692	0.165308	0.620357	0.619604	177.8366	287.0229
ZNF48	0.835045	0.164955	0.620403	0.620156	543.4793	876.3646
MAGEA12	0.8351	0.1649	2.370545	2.468649	20.00537	8.097825
NEK1	0.835111	0.164889	0.622642	0.622069	233.396	375.1992
AOAH	0.835217	0.164783	2.094665	2.140223	28.89665	13.49637
MIF4GD	0.835322	0.164678	0.621747	0.621078	200.0537	322.1135
PCDHB11	0.835429	0.164571	0.31785	0.284862	3.345343	11.76884
GATS	0.835443	0.164557	1.603878	1.604453	960.0134	598.3393
CREBZF	0.83558	0.16442	1.61481	1.6152	1450.39	897.9588
DNAJB11	0.83564	0.16436	0.595269	0.59523	3560.956	5982.493
CT45A5	0.835928	0.164072	0.122837	0.00241	0	4.138888
MAP3K3	0.835967	0.164033	0.623469	0.62305	318.9745	511.9625
BRCA2	0.836274	0.163726	0.623617	0.623187	311.1947	499.3659
SLC1A1	0.836291	0.163709	1.597814	1.598522	782.4323	489.4685
RALY-AS1	0.836361	0.163639	0.517092	0.510323	21.11678	41.38888
FAM13B	0.836445	0.163555	0.617803	0.617619	722.4162	1169.686
ACTN4	0.836467	0.163533	1.758034	1.758051	45665.6	25975.12
LOC15506	0.836605	0.163395	1.638743	1.642786	147.8175	89.97583
CAPN10-A	0.836623	0.163377	2.200164	2.26342	24.45101	10.7971
C11orf68	0.83684	0.16316	1.594003	1.595056	512.3598	321.2137
GPR160	0.836912	0.163088	1.610397	1.610819	1353.697	840.3743
KREMEN2	0.83708	0.16292	1.622981	1.62633	175.6027	107.971
FAM216A	0.837261	0.162739	0.622165	0.621418	182.2712	293.3212
SEPN1	0.837396	0.162604	1.612557	1.61296	1378.615	854.7074
UNC13A	0.837409	0.162591	1.937684	1.964653	38.89934	19.79468
ARRDC2	0.837579	0.162421	1.593199	1.594291	493.4659	309.5169
CNKSR3	0.837589	0.162411	0.623838	0.623291	244.5101	392.2946
EXOC3L4	0.83764	0.16236	0.44142	0.427825	10.00269	23.39372
LINC0063	0.83764	0.16236	0.44142	0.427825	10.00269	23.39372
WDR25	0.837853	0.162147	0.623401	0.622731	203.388	326.6123
LINC0142	0.837966	0.162034	0.318808	0.285664	3.334229	11.69686

PRRG2	0.838126	0.161874	0.622124	0.621394	183.3826	295.1207
LPCAT4	0.838406	0.161594	1.611915	1.612308	1430.384	887.1617
SMG1P2	0.838418	0.161582	1.601195	1.603253	266.7605	166.3833
LOC10050	0.838499	0.161501	0.44167	0.427825	10.00269	23.39372
TGFB3	0.838588	0.161412	0.502111	0.494233	17.78255	35.99033
ZNF843	0.838588	0.161412	0.502111	0.494233	17.78255	35.99033
HYAL3	0.838744	0.161256	1.677979	1.68446	102.2497	60.6977
HMGN2	0.838782	0.161218	0.590683	0.590655	4876.71	8256.452
HOXB6	0.838814	0.161186	1.68462	1.691198	98.91545	58.48429
RBM26-AS	0.838953	0.161047	0.453722	0.441376	11.1141	25.19323
LEMD1	0.83901	0.16099	1.63849	1.642657	143.3718	87.27656
PML	0.839129	0.160871	1.620152	1.620457	1879.394	1159.788
PRR3	0.839369	0.160631	0.61591	0.614789	120.0322	195.2476
LOC38869	0.839437	0.160563	1.747676	1.758391	69.87432	39.73333
FAM133D	0.839479	0.160521	0.618151	0.617145	133.5247	216.3649
ZDHHC4	0.839653	0.160347	0.624522	0.624163	371.2108	594.7402
CIAPIN1	0.839675	0.160325	0.617365	0.617205	830.223	1345.139
TUBE1	0.839727	0.160273	0.624585	0.624222	367.8766	589.3417
LINC0118	0.839795	0.160205	0.615894	0.614763	118.9208	193.448
HNRNPKP	0.839938	0.160062	0.482107	0.472467	14.44832	30.59178
USP27X-A	0.839938	0.160062	0.482107	0.472467	14.44832	30.59178
ALOX15	0.839961	0.160039	0.319404	0.285664	3.334229	11.69686
LOC10013	0.839961	0.160039	0.319404	0.285664	3.334229	11.69686
C5AR1	0.839983	0.160017	0.454108	0.441533	11.1141	25.18423
KANSL1-A	0.840225	0.159775	0.559075	0.555031	34.4537	62.08332
ERLIN2	0.840309	0.159691	0.61125	0.611151	1367.645	2237.825
LOC10537	0.840334	0.159666	0.464627	0.453121	12.22551	26.99275
SETD7	0.840803	0.159197	0.613428	0.613315	1188.097	1937.18
HLA-C	0.841013	0.158987	1.68116	1.681233	8873.616	5278.036
AP5S1	0.841119	0.158881	0.615845	0.614681	115.5866	188.0495
SOX8	0.84113	0.15887	1.677669	1.684291	100.0269	59.38405
MTERF2	0.841396	0.158604	0.622544	0.621747	167.8228	269.9275
SLC25A30	0.84149	0.15851	0.619648	0.619468	744.6444	1202.077
GDF11	0.841534	0.158466	1.591292	1.592063	709.0793	445.3804
SIRT5	0.841935	0.158065	0.623995	0.623728	510.137	817.8893
LENG1	0.84216	0.15784	0.62599	0.625556	306.749	490.3683
CRYZL1	0.842251	0.157749	0.625977	0.625475	265.6269	424.6859
GRHL2	0.842324	0.157676	0.617769	0.617618	893.5733	1446.811
C10orf35	0.842451	0.157549	1.588674	1.589964	413.4444	260.0302
UQCR11	0.842581	0.157419	0.619564	0.619391	776.8753	1254.263
MNS1	0.842604	0.157396	0.620943	0.620017	144.4832	233.0374
LOC55424	0.842716	0.157284	0.329254	0.297662	3.589853	12.08375
ZNF182	0.842744	0.157256	0.622713	0.62189	162.2658	260.9299
HGD	0.843029	0.156971	0.624663	0.624375	462.3464	740.5011
GALNT3	0.843156	0.156844	1.635273	1.635464	3106.39	1899.39
PPP1R13B	0.843281	0.156719	0.621157	0.620968	721.3048	1161.588
CYTH1	0.843349	0.156651	1.600554	1.601041	1123.635	701.8115
KLF10	0.843456	0.156544	1.620756	1.621025	2129.461	1313.647
FAM72D	0.843551	0.156449	0.557613	0.553381	32.94218	59.53701
AKAP5	0.843602	0.156398	1.599792	1.602104	236.7302	147.7583
CBY1	0.84367	0.15633	0.619486	0.61932	809.1062	1306.449
IZUMO4	0.843731	0.156269	2.508137	2.644313	16.67114	6.298308
ROBO3	0.843731	0.156269	2.508137	2.644313	16.67114	6.298308
CTAGE5	0.843762	0.156238	0.610667	0.610579	1542.636	2526.521
GPR180	0.843896	0.156104	1.592747	1.593376	870.2337	546.1533
KIAA1656	0.844026	0.155974	0.538181	0.532716	25.6291	48.11907

KCNIP3	0.844093	0.155907	1.627063	1.630917	151.1517	92.67511
TMEM135	0.844266	0.155734	0.627314	0.626803	265.6269	423.7862
BACH2	0.844306	0.155694	1.618448	1.621852	167.8228	103.4722
CLDN23	0.844988	0.155012	1.671248	1.67759	101.1383	60.28381
EVA1B	0.845014	0.154986	1.688173	1.695695	90.02418	53.08574
ULK4	0.845084	0.154916	0.618784	0.617635	118.9208	192.5483
NAPEPLD	0.845216	0.154784	0.627654	0.627127	256.7356	409.39
TSEN54	0.845245	0.154755	0.626015	0.625705	430.1155	687.4153
MARVELD	0.845252	0.154748	0.627679	0.627154	257.847	411.1446
DYNC1I1	0.845768	0.154232	2.041733	2.0837	30.00806	14.39613
HIST1H3D	0.84595	0.15405	0.365245	0.340147	4.890202	14.39613
ZNF251	0.846077	0.153923	0.620909	0.620736	774.6525	1247.965
UBXN2B	0.846152	0.153848	0.621094	0.620918	764.4386	1231.148
ALG10	0.846654	0.153346	0.616327	0.615059	106.0285	172.3937
MRPL10	0.846703	0.153297	0.625254	0.624997	517.9169	828.6774
KCTD13	0.847236	0.152764	0.627962	0.627428	248.9557	396.7934
ZNF558	0.847579	0.152421	0.624881	0.624651	592.3813	948.3453
POR	0.847644	0.152356	0.604927	0.604871	2426.207	4011.123
PIGY	0.847768	0.152232	0.628065	0.627681	346.7598	552.4516
PYURF	0.847768	0.152232	0.628065	0.627681	346.7598	552.4516
ITGAX	0.848059	0.151941	2.129443	2.184397	25.56242	11.69686
HFE	0.848243	0.151757	0.628544	0.628048	267.8497	426.4854
BLOC1S4	0.848261	0.151739	0.621464	0.620379	125.5893	202.4456
EPB41L4A	0.848327	0.151673	3.227409	3.695707	10.00269	2.699275
FADS6	0.848327	0.151673	3.227409	3.695707	10.00269	2.699275
HRASLS2	0.848327	0.151673	3.227409	3.695707	10.00269	2.699275
KRT9	0.848327	0.151673	3.227409	3.695707	10.00269	2.699275
RAD21L1	0.848327	0.151673	3.227409	3.695707	10.00269	2.699275
SCN1A	0.848327	0.151673	3.227409	3.695707	10.00269	2.699275
UCP1	0.848327	0.151673	3.227409	3.695707	10.00269	2.699275
SMUG1	0.848364	0.151636	0.627941	0.627618	419.0014	667.6117
PMS2P4	0.848393	0.151607	0.554163	0.549565	30.34148	55.21817
ABHD13	0.848607	0.151393	0.626747	0.626465	472.3491	753.9975
C1orf204	0.848658	0.151342	2.128454	2.184397	25.56242	11.69686
ZMAT5	0.848672	0.151328	0.628094	0.627714	345.6484	550.6521
RGAG4	0.848876	0.151124	0.618831	0.617637	112.2524	181.7512
FAM173B	0.849065	0.150935	0.627375	0.626684	192.2739	306.8176
LOXL1-AS	0.849326	0.150674	0.627936	0.627327	215.6135	343.7077
LRRFIP1	0.849389	0.150611	1.643342	1.643481	4335.542	2638.019
TSC22D1	0.849863	0.150137	1.614703	1.614971	2112.79	1308.249
LOC10192	0.8501	0.1499	0.357008	0.329892	4.445638	13.49637
NPY1R	0.8501	0.1499	0.357008	0.329892	4.445638	13.49637
RBM20	0.8501	0.1499	0.357008	0.329892	4.445638	13.49637
DIO2	0.850163	0.149837	2.740337	2.960198	13.33691	4.498792
FAM83A	0.850163	0.149837	2.740337	2.960198	13.33691	4.498792
HHIP-AS1	0.850163	0.149837	2.740337	2.960198	13.33691	4.498792
LHX4	0.850163	0.149837	2.740337	2.960198	13.33691	4.498792
NLRP10	0.850163	0.149837	2.740337	2.960198	13.33691	4.498792
RNF152	0.850163	0.149837	2.740337	2.960198	13.33691	4.498792
TUBAL3	0.850163	0.149837	2.740337	2.960198	13.33691	4.498792
AR	0.85031	0.14969	3.220127	3.695707	10.00269	2.699275
LCTL	0.85031	0.14969	3.220127	3.695707	10.00269	2.699275
SYCE1L	0.85031	0.14969	3.220127	3.695707	10.00269	2.699275
LOC10192	0.850353	0.149647	0.541615	0.536141	25.56242	47.68719
DYRK1A	0.850531	0.149469	0.614748	0.61465	1381.482	2247.596
SLC6A10F	0.85056	0.14944	2.256942	2.33611	21.11678	9.033573

KIF12	0.850578	0.149422	1.589002	1.591108	253.4014	159.2572
ADAMTSL	0.850612	0.149388	1.585387	1.58724	285.6323	179.9517
ZNF691	0.850698	0.149302	0.618918	0.617638	106.6953	172.7536
ARSG	0.850755	0.149245	0.571271	0.567604	37.78793	66.58211
TET2	0.850765	0.149235	0.629727	0.629349	357.8739	568.6472
FN3K	0.851054	0.148946	0.629605	0.629062	244.5101	388.6956
CCR6	0.851175	0.148825	0.226074	0.16142	1.11141	6.937137
S100A13	0.851205	0.148795	1.640301	1.640444	4188.903	2553.514
HDHD2	0.851379	0.148621	0.628625	0.628312	424.5585	675.7185
FNBP1	0.851565	0.148435	1.614136	1.614396	2171.694	1345.202
RIT1	0.851641	0.148359	0.620255	0.620116	965.8149	1557.482
NR2F2	0.851651	0.148349	0.626951	0.626703	536.8108	856.5699
BCL2L10	0.85169	0.14831	0.357499	0.329892	4.445638	13.49637
SOX2	0.85169	0.14831	0.357499	0.329892	4.445638	13.49637
ZNF624	0.851722	0.148278	0.538779	0.532947	24.45101	45.88767
HOXB9	0.851841	0.148159	1.594042	1.594486	1238.11	776.4914
ISL1	0.851847	0.148153	1.770437	1.783981	57.7933	32.3913
KIAA0226	0.851847	0.148153	1.770437	1.783981	57.7933	32.3913
IL11RA	0.852069	0.147931	1.798178	1.813963	52.23625	28.79227
TMEM53	0.852286	0.147714	0.610904	0.609185	80.02149	131.3647
PARG	0.852385	0.147615	0.617364	0.617253	1212.359	1964.127
NVL	0.852528	0.147472	0.62976	0.629434	414.5558	658.6231
ZNF579	0.852528	0.147472	0.62976	0.629434	414.5558	658.6231
HERC4	0.852696	0.147304	1.615238	1.615481	2375.138	1470.232
MANEA	0.853055	0.146945	1.577264	1.578332	485.686	307.7173
MARCKSL	0.853094	0.146906	1.614067	1.614318	2252.827	1395.525
MKS1	0.853108	0.146892	0.625975	0.625014	141.149	225.8393
RPP40	0.853137	0.146863	0.628318	0.628038	469.0148	746.7994
CPD	0.853206	0.146794	1.65244	1.652546	5822.675	3523.454
LGALS7B	0.853352	0.146648	0.327448	0.293793	3.334229	11.37294
KANSL1L	0.853393	0.146607	0.629978	0.629343	208.945	332.0108
GUSBP3	0.853478	0.146522	2.179519	2.244957	23.03952	10.25724
ARAP2	0.853597	0.146403	1.576953	1.577956	516.8055	327.512
TRIM69	0.853617	0.146383	1.997201	2.033823	31.11947	15.29589
GTPBP6	0.853701	0.146299	0.631492	0.631024	287.8551	456.1775
CHMP6	0.854187	0.145813	0.62767	0.626781	152.2631	242.9347
TIMELESS	0.854202	0.145798	0.612148	0.612073	1840.494	3006.992
NBEAL2	0.854258	0.145742	1.585423	1.585989	934.6955	589.3417
LOC28445	0.854664	0.145336	1.656792	1.662977	100.5715	60.47276
IMMP1L	0.854821	0.145179	0.626092	0.625134	138.9262	222.2403
PASK	0.854821	0.145179	0.626092	0.625134	138.9262	222.2403
APOBEC3	0.854876	0.145124	7.116902	355.5397	3.545397	0
ATP5J2	0.854949	0.145051	1.657287	1.657379	6751.88	4073.827
SEC22B	0.855019	0.144981	1.64543	1.645548	5134.712	3120.362
ARHGAP3	0.855629	0.144371	1.577592	1.579262	311.1947	197.0471
NELFB	0.855639	0.144361	1.597032	1.597399	1480.398	926.7511
SUPT20H	0.855968	0.144032	0.623652	0.623493	825.7773	1324.444
ZNF878	0.856129	0.143871	2.374809	2.485365	17.92704	7.207064
ADAP1	0.856156	0.143844	1.577075	1.577802	726.8619	460.6763
KLHL15	0.856193	0.143807	1.581808	1.58239	916.9129	579.4443
RHBDL1	0.856317	0.143683	1.913069	1.94058	36.67652	18.89492
SNHG11	0.856326	0.143674	0.624316	0.623249	123.3665	197.9468
MCM9	0.856345	0.143655	0.610413	0.608565	74.46444	122.3671
GLTSCR1	0.856752	0.143248	0.631446	0.631107	390.1048	618.134
LOC10099	0.856974	0.143026	0.386549	0.363719	5.557048	15.29589
DISP1	0.856993	0.143007	0.610379	0.608565	74.46444	122.3671

FOSL2	0.857186	0.142814	1.619138	1.619339	2844.097	1756.328
DTL	0.857234	0.142766	0.626556	0.626361	676.8484	1080.61
ABHD14B	0.8573	0.1427	0.622008	0.621873	974.7062	1567.379
BAZ1A	0.85732	0.14268	1.593863	1.594245	1411.49	885.3622
FRMD8	0.85735	0.14265	1.576476	1.577176	753.5357	477.7717
VEGFA	0.857489	0.142511	0.593378	0.593355	5952.71	10032.31
SDHAF1	0.857739	0.142261	1.575644	1.577354	302.3034	191.6485
STAG3L3	0.857963	0.142037	1.636424	1.641834	112.0857	68.26466
PXN-AS1	0.858516	0.141484	0.519853	0.512301	18.89396	36.89009
MYCL	0.858601	0.141399	0.633203	0.632688	256.7356	405.791
TMTC3	0.858623	0.141377	0.630608	0.63034	495.6887	786.3888
CNFN	0.858707	0.141293	0.61016	0.608291	72.24162	118.7681
PYROXD2	0.858707	0.141293	0.61016	0.608291	72.24162	118.7681
LOC10028	0.858835	0.141165	0.408618	0.388997	6.668457	17.15839
P2RX5-TA	0.859313	0.140687	1.737208	1.749047	62.02777	35.45947
ANAPC1P	0.859574	0.140426	7.021026	349.9826	3.489826	0
LTV1	0.859765	0.140235	0.626188	0.626019	786.878	1256.962
LINC0105	0.85984	0.14016	1.612023	1.612246	2528.457	1568.279
STMN3	0.859844	0.140156	1.626649	1.631639	118.9208	72.88042
TBCCD1	0.860096	0.139904	0.633907	0.633529	356.5402	562.7898
VEPH1	0.860224	0.139776	0.631213	0.630408	164.4886	260.9299
ZFC3H1	0.860387	0.139613	1.613696	1.613907	2676.274	1658.255
CRYZ	0.860458	0.139542	1.612596	1.612812	2606.255	1615.966
ZNF577	0.860498	0.139502	1.586622	1.589404	194.4967	122.3671
C1QTNF3	0.86059	0.13941	4.191321	5.845808	6.412833	1.088708
LOC55310	0.860724	0.139276	0.509312	0.500919	16.67114	33.29106
MAML1	0.860797	0.139203	0.618422	0.618328	1450.39	2345.67
ZNF783	0.860904	0.139096	0.63459	0.63418	327.9992	517.2081
APOBEC3	0.860966	0.139034	1.612772	1.61693	135.7142	83.92945
SCN8A	0.860968	0.139032	1.686741	1.695264	77.79867	45.88767
DNAJB6	0.860983	0.139017	1.645447	1.64555	5994.943	3643.121
EIF4E3	0.861158	0.138842	1.568358	1.569657	391.2162	249.2331
C7orf25	0.861223	0.138777	0.633567	0.633223	383.4363	605.5373
CATSPER	0.861323	0.138677	1.955886	1.989484	32.23088	16.19565
GNG7	0.861404	0.138596	0.609807	0.607847	68.90739	113.3695
RARA-AS	0.861622	0.138378	1.955273	1.989484	32.23088	16.19565
USP35	0.862047	0.137953	1.573757	1.57572	262.2927	166.4553
PCAT7	0.862119	0.137881	1.822341	1.841978	44.75646	24.29347
DCLK2	0.862186	0.137814	0.580048	0.576504	38.89934	67.48187
PRKCQ-A	0.862186	0.137814	0.580048	0.576504	38.89934	67.48187
SUOX	0.86234	0.13766	0.634648	0.634259	338.9799	534.4564
NATD1	0.862437	0.137563	1.581686	1.584314	203.5102	128.4495
HIST1H1C	0.862442	0.137558	0.632638	0.632361	476.7947	753.9975
ZBTB11-A	0.862502	0.137498	0.594302	0.591455	48.90202	82.68779
ZNF280C	0.862561	0.137439	0.635534	0.6351	308.9719	486.4993
CD72	0.862576	0.137424	0.429032	0.412055	7.779867	18.89492
HIST2H2E	0.862576	0.137424	0.429032	0.412055	7.779867	18.89492
MUC16	0.862697	0.137303	0.630162	0.629949	624.6122	991.5337
FOXD2-AS	0.862834	0.137166	1.622004	1.626805	120.0322	73.78018
NEIL1	0.86286	0.13714	0.496436	0.486779	14.44832	29.69202
RBMS2	0.863069	0.136931	1.625943	1.626093	3865.594	2377.224
CLEC11A	0.863163	0.136837	0.625306	0.624071	107.8067	172.7536
ANKRD34	0.863218	0.136782	1.585319	1.588105	190.051	119.6679
C16orf62	0.863218	0.136782	0.622603	0.622486	1136.972	1826.509
EBLN2	0.863262	0.136738	0.429252	0.412055	7.779867	18.89492
ITGA9-AS	0.863262	0.136738	0.429252	0.412055	7.779867	18.89492

LOC64898	0.863425	0.136575	0.605909	0.603638	60.78299	100.7009
DIS3L	0.863722	0.136278	0.629427	0.629236	693.5196	1102.168
LOC10537	0.863735	0.136265	0.488777	0.478341	13.33691	27.89251
TP53INP2	0.863878	0.136122	1.581797	1.582231	1231.442	778.2909
RNF103	0.863972	0.136028	1.57678	1.577304	989.1545	627.1135
CISH	0.863996	0.136004	1.599711	1.603361	150.0403	93.57486
SFTA2	0.864041	0.135959	0.636359	0.635896	290.0779	456.1775
ANKRD30	0.864056	0.135944	0.292615	0.247882	2.222819	8.997583
CASC1	0.864056	0.135944	0.292615	0.247882	2.222819	8.997583
HIST2H2E	0.864056	0.135944	0.292615	0.247882	2.222819	8.997583
HS3ST6	0.864056	0.135944	0.292615	0.247882	2.222819	8.997583
LOC10192	0.864056	0.135944	0.292615	0.247882	2.222819	8.997583
EPB41L4A	0.86412	0.13588	0.634102	0.633364	178.9369	282.5241
STX18-AS	0.864232	0.135768	0.429567	0.412055	7.779867	18.89492
UVSSA	0.864368	0.135632	0.625469	0.624208	105.5839	169.1546
NRSN2	0.864545	0.135455	1.564499	1.565827	378.9907	242.035
ZSCAN25	0.864552	0.135448	0.634749	0.634443	440.1182	693.7137
IGFN1	0.864552	0.135448	0.636102	0.635587	255.6242	402.192
LINC0023	0.86484	0.13516	0.470135	0.457716	11.1141	24.29347
GPR162	0.864899	0.135101	0.458633	0.44493	10.00269	22.49396
JAG1	0.864981	0.135019	1.596267	1.596552	1900.51	1190.38
SMG1P7	0.865365	0.134635	0.631611	0.630612	134.9918	214.0705
MPPED2	0.865474	0.134526	0.234219	0.168628	1.11141	6.640216
PINX1	0.865768	0.134232	0.635333	0.634627	186.7168	294.221
TLR3	0.865851	0.134149	1.640606	1.646866	97.80404	59.38405
FEZ1	0.865899	0.134101	0.510225	0.501452	16.23769	32.3913
PHF20L1	0.866113	0.133887	0.614659	0.614593	2032.768	3307.512
KIAA0319	0.866161	0.133839	0.293408	0.247882	2.222819	8.997583
NUTM2D	0.866291	0.133709	0.591874	0.588772	44.9454	76.34449
PRR16	0.866323	0.133677	1.702011	1.7123	67.79598	39.58937
ZBTB5	0.866591	0.133409	1.573993	1.574513	990.2659	628.9311
SGSM1	0.866765	0.133235	1.941653	1.975357	32.01971	16.20465
ZNF548	0.867043	0.132957	1.562607	1.563963	364.6646	233.1634
GPX8	0.867092	0.132908	1.601366	1.601604	2310.754	1442.771
EIF2A	0.867111	0.132889	1.603761	1.603986	2460.661	1534.088
MLPH	0.86713	0.13287	1.602875	1.607056	134.4806	83.67752
MAL	0.86721	0.13279	1.59111	1.594511	157.8202	98.97341
TMEM56-I	0.867247	0.132753	1.782132	1.798864	48.79088	27.11872
FAM98A	0.867411	0.132589	1.588276	1.588598	1683.786	1059.915
RPUSD2	0.86761	0.13239	0.634183	0.633352	156.7088	247.4335
PCGF3	0.86765	0.13235	1.559399	1.560466	474.5719	304.1183
PRICKLE1	0.867698	0.132302	0.627876	0.627731	931.3612	1483.701
LINC0049	0.867715	0.132285	6.848449	339.9799	3.389799	0
ALPK3	0.867742	0.132258	1.717553	1.729121	62.23894	35.99033
DDX42	0.867805	0.132195	0.615681	0.615614	2049.439	3329.106
ASPSCR1	0.867972	0.132028	0.63082	0.630641	753.5357	1194.879
SUN3	0.868116	0.131884	1.679886	1.688833	74.46444	44.08816
LYRM2	0.868261	0.131739	0.629638	0.629475	833.7239	1324.48
TSC22D1-	0.868323	0.131677	3.527693	4.32337	7.813209	1.799517
GAGE6	0.868355	0.131645	0.134648	0.002677	0	3.724999
SNX20	0.868518	0.131482	3.52163	4.304944	7.779867	1.799517
EXTL3-AS	0.868573	0.131427	2.523582	2.685658	14.37053	5.344564
GTF2H2C	0.868705	0.131295	0.40496	0.383302	6.101639	15.93472
FKBP14	0.868742	0.131258	1.596107	1.596365	2102.754	1317.21
ADCK5	0.868815	0.131185	0.638543	0.637952	226.7276	355.4045
CD7	0.868927	0.131073	6.828013	334.4229	3.334229	0

MIR646HC	0.868927	0.131073	6.828013	334.4229	3.334229	0
TF	0.868927	0.131073	6.828013	334.4229	3.334229	0
ITCH	0.86897	0.13103	1.630236	1.630355	4906.873	3009.692
DNAH10	0.869132	0.130868	2.513807	2.673235	14.44832	5.39855
SCUBE2	0.869132	0.130868	2.513807	2.673235	14.44832	5.39855
HERC3	0.869319	0.130681	1.560342	1.561143	633.5035	405.791
ZC3H14	0.869441	0.130559	0.613724	0.613666	2330.626	3797.88
PHLDA2	0.869452	0.130548	1.562031	1.563784	285.6323	182.6509
CHODL	0.869475	0.130525	0.600819	0.598159	51.12484	85.47704
PCDHB3	0.86955	0.13045	0.135393	0.002646	0	3.769987
CLDN16	0.869772	0.130228	0.618268	0.618193	1788.258	2892.723
OLFM2	0.869813	0.130187	1.566388	1.568629	230.0618	146.6606
C4BPB	0.87003	0.12997	3.513678	4.304944	7.779867	1.799517
DISP2	0.87003	0.12997	3.513678	4.304944	7.779867	1.799517
ZNF442	0.87003	0.12997	3.513678	4.304944	7.779867	1.799517
SLC39A13	0.870039	0.129961	1.562778	1.563479	714.6364	457.0772
DHRS11	0.870543	0.129457	0.638616	0.637988	208.945	327.512
NADK2	0.870848	0.129152	0.631052	0.630887	819.1089	1298.351
PPP1R12C	0.871052	0.128948	0.608322	0.608281	3258.653	5357.161
C3orf38	0.871077	0.128923	0.638673	0.638308	360.0967	564.1485
BEX5	0.871299	0.128701	1.628907	1.634761	100.0269	61.18356
STK17B	0.871451	0.128549	1.574598	1.57502	1220.161	774.6919
AMOTL2	0.871544	0.128456	0.586559	0.586548	11907.64	20301.25
SPECC1L	0.871915	0.128085	0.620224	0.620144	1677.662	2705.285
DLL4	0.871977	0.128023	2.244163	2.33157	18.89396	8.097825
LOC54147	0.871977	0.128023	2.244163	2.33157	18.89396	8.097825
ADRA2A	0.872067	0.127933	6.752573	334.4229	3.334229	0
APCDD1L	0.872067	0.127933	6.752573	334.4229	3.334229	0
AQP7P1	0.872067	0.127933	6.752573	334.4229	3.334229	0
ASAP1-IT1	0.872067	0.127933	6.752573	334.4229	3.334229	0
AVPR1A	0.872067	0.127933	6.752573	334.4229	3.334229	0
CCDC190	0.872067	0.127933	6.752573	334.4229	3.334229	0
CD69	0.872067	0.127933	6.752573	334.4229	3.334229	0
CEACAM2	0.872067	0.127933	6.752573	334.4229	3.334229	0
CFHR3	0.872067	0.127933	6.752573	334.4229	3.334229	0
CLPSL2	0.872067	0.127933	6.752573	334.4229	3.334229	0
CMAHP	0.872067	0.127933	6.752573	334.4229	3.334229	0
COX7B2	0.872067	0.127933	6.752573	334.4229	3.334229	0
CSMD2	0.872067	0.127933	6.752573	334.4229	3.334229	0
CTAGE1	0.872067	0.127933	6.752573	334.4229	3.334229	0
CXCL11	0.872067	0.127933	6.752573	334.4229	3.334229	0
CXCR2P1	0.872067	0.127933	6.752573	334.4229	3.334229	0
DGKI	0.872067	0.127933	6.752573	334.4229	3.334229	0
DNMBP-A	0.872067	0.127933	6.752573	334.4229	3.334229	0
ESX1	0.872067	0.127933	6.752573	334.4229	3.334229	0
FAM184A	0.872067	0.127933	6.752573	334.4229	3.334229	0
FAM57B	0.872067	0.127933	6.752573	334.4229	3.334229	0
GMFG	0.872067	0.127933	6.752573	334.4229	3.334229	0
GRIK1-AS	0.872067	0.127933	6.752573	334.4229	3.334229	0
HLA-G	0.872067	0.127933	6.752573	334.4229	3.334229	0
IL1RAPL1	0.872067	0.127933	6.752573	334.4229	3.334229	0
IL7	0.872067	0.127933	6.752573	334.4229	3.334229	0
INSRR	0.872067	0.127933	6.752573	334.4229	3.334229	0
JAKMIP3	0.872067	0.127933	6.752573	334.4229	3.334229	0
KIAA1210	0.872067	0.127933	6.752573	334.4229	3.334229	0
LINC0033	0.872067	0.127933	6.752573	334.4229	3.334229	0

LINC0045	0.872067	0.127933	6.752573	334.4229	3.334229	0
LINC0062	0.872067	0.127933	6.752573	334.4229	3.334229	0
LINC0093	0.872067	0.127933	6.752573	334.4229	3.334229	0
LINC0102	0.872067	0.127933	6.752573	334.4229	3.334229	0
LINC0109	0.872067	0.127933	6.752573	334.4229	3.334229	0
LINC0126	0.872067	0.127933	6.752573	334.4229	3.334229	0
LINC0139	0.872067	0.127933	6.752573	334.4229	3.334229	0
LINC0152	0.872067	0.127933	6.752573	334.4229	3.334229	0
LOC10028	0.872067	0.127933	6.752573	334.4229	3.334229	0
LOC10105	0.872067	0.127933	6.752573	334.4229	3.334229	0
LOC10192	0.872067	0.127933	6.752573	334.4229	3.334229	0
LOC10192	0.872067	0.127933	6.752573	334.4229	3.334229	0
LOC10192	0.872067	0.127933	6.752573	334.4229	3.334229	0
LOC28464	0.872067	0.127933	6.752573	334.4229	3.334229	0
LOC57453	0.872067	0.127933	6.752573	334.4229	3.334229	0
LOC64594	0.872067	0.127933	6.752573	334.4229	3.334229	0
MEP1B	0.872067	0.127933	6.752573	334.4229	3.334229	0
MEX3B	0.872067	0.127933	6.752573	334.4229	3.334229	0
PRDM12	0.872067	0.127933	6.752573	334.4229	3.334229	0
PRDM8	0.872067	0.127933	6.752573	334.4229	3.334229	0
SERPINA9	0.872067	0.127933	6.752573	334.4229	3.334229	0
SOGA3	0.872067	0.127933	6.752573	334.4229	3.334229	0
TMEM31	0.872067	0.127933	6.752573	334.4229	3.334229	0
TPH2	0.872067	0.127933	6.752573	334.4229	3.334229	0
VSIG8	0.872067	0.127933	6.752573	334.4229	3.334229	0
LEAP2	0.872128	0.127872	3.502366	4.304944	7.779867	1.799517
SRL	0.872128	0.127872	3.502366	4.304944	7.779867	1.799517
TDRD9	0.872128	0.127872	3.502366	4.304944	7.779867	1.799517
FAM234A	0.872189	0.127811	0.625495	0.625386	1252.559	2002.862
HEATR6	0.872456	0.127544	0.640133	0.639681	290.0779	453.4782
BUB1B	0.872581	0.127419	1.588228	1.588509	1896.209	1193.7
RNASEK-(	0.872764	0.127236	0.432689	0.414919	7.535357	18.17512
NKX2-5	0.872794	0.127206	2.242623	2.33157	18.89396	8.097825
NEU3	0.872824	0.127176	1.557287	1.559	288.9665	185.3502
IP6K1	0.87288	0.12712	0.631505	0.631343	817.9975	1295.652
GSDMA	0.872923	0.127077	6.733397	333.3115	3.323115	0
DGKB	0.873023	0.126977	0.375358	0.347877	4.579007	13.18146
FAM32A	0.873052	0.126948	1.602498	1.602694	2869.66	1790.519
HSPB8	0.873135	0.126865	1.596749	1.596974	2413.982	1511.594
BID	0.873189	0.126811	1.604139	1.604328	2913.005	1815.712
LINC0094	0.873506	0.126494	1.629217	1.635277	96.70375	59.13212
KIAA0125	0.873538	0.126462	1.967572	2.006552	28.89665	14.39613
TFEB	0.873548	0.126452	0.641225	0.640769	292.3007	456.1775
SMAD1	0.873549	0.126451	0.64099	0.64038	218.9477	341.9082
NAPB	0.873587	0.126413	0.639965	0.639298	196.7195	307.7173
ALDH5A1	0.873614	0.126386	0.634927	0.634721	640.7943	1009.574
NDUFB10	0.873926	0.126074	0.629483	0.629349	1013.606	1610.567
SIPA1L3	0.873931	0.126069	1.591502	1.591754	2103.898	1321.745
ZKSCAN2	0.874179	0.125821	0.640228	0.639555	194.4967	304.1183
HTR7P1	0.874531	0.125469	0.599286	0.59637	46.6792	78.27897
LIPE	0.874565	0.125435	1.578735	1.581906	162.2658	102.5724
PRDM16	0.874669	0.125331	0.536624	0.529509	20.00537	37.78985
BSN	0.874684	0.125316	1.744731	1.759013	52.23625	29.69202
ANKRD6	0.874859	0.125141	0.64088	0.640497	342.3142	534.4564
POLR1A	0.874958	0.125042	0.62479	0.624693	1373.702	2199.009
PI4KAP2	0.874963	0.125037	0.641976	0.641406	233.8183	364.5461

MRPL57	0.874978	0.125022	0.640623	0.640263	364.7313	569.664
KRT4	0.875083	0.124917	2.798447	3.082292	11.1141	3.599033
LINC0017(	0.875083	0.124917	2.798447	3.082292	11.1141	3.599033
LOC65316	0.875083	0.124917	2.798447	3.082292	11.1141	3.599033
LRRC15	0.875083	0.124917	2.798447	3.082292	11.1141	3.599033
MYOM2	0.875083	0.124917	2.798447	3.082292	11.1141	3.599033
PMS1	0.875309	0.124691	0.637746	0.6375	534.588	838.5747
SFT2D2	0.875538	0.124462	1.551791	1.55329	325.643	209.6437
KCTD17	0.875591	0.124409	1.551926	1.553452	320.086	206.0447
CCDC9	0.875595	0.124405	1.55047	1.55171	392.3276	252.8321
ZNF600	0.875786	0.124214	1.56013	1.560708	862.4316	552.5866
ARHGEF3	0.87588	0.12412	1.550852	1.552279	341.2694	219.8469
TSGA13	0.875989	0.124011	0.461685	0.4473	9.52478	21.30628
TET3	0.876221	0.123779	0.633042	0.632883	852.4511	1346.938
CKMT2	0.876223	0.123777	0.343589	0.309447	3.334229	10.7971
HELLS	0.876241	0.123759	0.640559	0.640238	408.9987	638.8284
DUSP28	0.876278	0.123722	1.7715	1.788653	46.6792	26.09299
FAM155A	0.876317	0.123683	6.647776	334.4229	3.334229	0
FAM71E2	0.876317	0.123683	6.647776	334.4229	3.334229	0
FAP	0.876317	0.123683	6.647776	334.4229	3.334229	0
GSTA1	0.876317	0.123683	6.647776	334.4229	3.334229	0
HIST1H1T	0.876317	0.123683	6.647776	334.4229	3.334229	0
LHX5	0.876317	0.123683	6.647776	334.4229	3.334229	0
LINC0032	0.876317	0.123683	6.647776	334.4229	3.334229	0
NRN1L	0.876317	0.123683	6.647776	334.4229	3.334229	0
PDCL2	0.876317	0.123683	6.647776	334.4229	3.334229	0
RGS4	0.876317	0.123683	6.647776	334.4229	3.334229	0
SIGLEC1(	0.876317	0.123683	6.647776	334.4229	3.334229	0
SLC12A3	0.876317	0.123683	6.647776	334.4229	3.334229	0
SLC13A1	0.876317	0.123683	6.647776	334.4229	3.334229	0
SLC35D3	0.876317	0.123683	6.647776	334.4229	3.334229	0
SNORA16	0.876317	0.123683	6.647776	334.4229	3.334229	0
TBX4	0.876317	0.123683	6.647776	334.4229	3.334229	0
THBS2	0.876317	0.123683	6.647776	334.4229	3.334229	0
TLX1NB	0.876317	0.123683	6.647776	334.4229	3.334229	0
ZCCHC18	0.876317	0.123683	6.647776	334.4229	3.334229	0
ZNF648	0.876317	0.123683	6.647776	334.4229	3.334229	0
PCNXL4	0.876416	0.123584	0.639344	0.639071	480.1289	751.2982
ZNF484	0.876782	0.123218	0.636028	0.634951	122.2551	192.5483
KCTD6	0.877006	0.122994	0.641498	0.640789	184.494	287.9227
LINC0066	0.877089	0.122911	1.862354	1.888081	35.56511	18.83194
ADCY2	0.877111	0.122889	0.138707	0.002771	0	3.599033
ADGRF5	0.877111	0.122889	0.138707	0.002771	0	3.599033
C11orf16	0.877111	0.122889	0.138707	0.002771	0	3.599033
CLEC7A	0.877111	0.122889	0.138707	0.002771	0	3.599033
CRB1	0.877111	0.122889	0.138707	0.002771	0	3.599033
FOLR2	0.877111	0.122889	0.138707	0.002771	0	3.599033
GABRD	0.877111	0.122889	0.138707	0.002771	0	3.599033
LCN12	0.877111	0.122889	0.138707	0.002771	0	3.599033
LINC0147	0.877111	0.122889	0.138707	0.002771	0	3.599033
LOC10192	0.877111	0.122889	0.138707	0.002771	0	3.599033
LOC10192	0.877111	0.122889	0.138707	0.002771	0	3.599033
LPAR4	0.877111	0.122889	0.138707	0.002771	0	3.599033
LRRIQ1	0.877111	0.122889	0.138707	0.002771	0	3.599033
NBAT1	0.877111	0.122889	0.138707	0.002771	0	3.599033
NPFF	0.877111	0.122889	0.138707	0.002771	0	3.599033

PCA3	0.877111	0.122889	0.138707	0.002771	0	3.599033
PIK3C2G	0.877111	0.122889	0.138707	0.002771	0	3.599033
RFTN2	0.877111	0.122889	0.138707	0.002771	0	3.599033
UCN	0.877111	0.122889	0.138707	0.002771	0	3.599033
ZNF366	0.877111	0.122889	0.138707	0.002771	0	3.599033
NKAP	0.877157	0.122843	0.635871	0.635678	684.6283	1077.011
ZNF205	0.87716	0.12284	0.64198	0.641312	195.6081	305.0181
MRPL48	0.877225	0.122775	0.640494	0.640197	441.2296	689.2149
CD83	0.877322	0.122678	1.55113	1.551943	599.0498	385.9963
FGG	0.877364	0.122636	0.344022	0.309447	3.334229	10.7971
STAC3	0.877364	0.122636	0.344022	0.309447	3.334229	10.7971
DNAJB13	0.877385	0.122615	0.566546	0.561557	27.78524	49.48671
PIGQ	0.877412	0.122588	0.635142	0.634962	732.4189	1153.49
JADE3	0.877445	0.122555	1.55901	1.559567	892.4619	572.2463
TRAM1L1	0.877586	0.122414	0.527995	0.520238	17.78255	34.19082
IQUB	0.877684	0.122316	0.308861	0.265156	2.378417	8.997583
GABPB1-A	0.87772	0.12228	1.860297	1.886279	35.56511	18.84994
C14orf37	0.877836	0.122164	1.807339	1.827774	41.12215	22.49396
SPATA6L	0.877888	0.122112	1.806963	1.827774	41.12215	22.49396
LOC64338	0.877896	0.122104	4.209653	6.180262	5.723759	0.917753
HIF1A-AS	0.878006	0.121994	2.038911	2.08946	24.45101	11.69686
PCSK4	0.878011	0.121989	0.528243	0.520238	17.78255	34.19082
TMEM263	0.878075	0.121925	1.578705	1.579003	1781.59	1128.297
ZNF271P	0.878093	0.121907	0.63921	0.638963	532.3652	833.1762
LOC10028	0.878103	0.121897	1.547434	1.548767	362.3195	233.9372
LOC10272	0.878268	0.121732	0.242374	0.175021	1.11141	6.397282
PCGF6	0.878474	0.121526	0.636557	0.635451	118.9208	187.1497
EFTUD1	0.878545	0.121455	0.641861	0.641538	404.5531	630.6046
C11orf72	0.878593	0.121407	1.55701	1.55929	217.0472	139.1926
ABCB10	0.878667	0.121333	0.643594	0.643048	238.9531	371.6002
MB21D1	0.878721	0.121279	0.607167	0.604522	51.12484	84.57728
NDUFV3	0.878753	0.121247	0.628202	0.628094	1236.999	1969.454
CCDC77	0.878767	0.121233	1.548504	1.549361	564.5961	364.4021
PRKAA2	0.878888	0.121112	0.63052	0.630396	1069.176	1696.044
ZNF185	0.879007	0.120993	1.586526	1.586768	2187.254	1378.43
DHRS4L2	0.879094	0.120906	0.638389	0.63732	125.0336	196.1923
RBP7	0.879103	0.120897	0.641514	0.641229	467.9034	729.704
MLKL	0.879111	0.120889	1.582275	1.586228	135.592	85.47704
MED18	0.879173	0.120827	0.643908	0.643429	272.6843	423.8042
SULT4A1	0.87918	0.12082	1.855527	1.88179	35.56511	18.89492
MZT1	0.879343	0.120657	1.565259	1.565677	1205.879	770.1931
LINC-ROF	0.879525	0.120475	1.814515	1.83599	39.65509	21.5942
ZNF720	0.879623	0.120377	1.543878	1.545052	415.6672	269.0277
TOP1P1	0.879867	0.120133	4.191451	6.144011	5.634847	0.908756
BCL2	0.879989	0.120011	0.606941	0.604237	50.01343	82.77776
C12orf29	0.880014	0.119986	1.546967	1.547818	566.8189	366.2016
RABEP1	0.880123	0.119877	0.629562	0.629449	1182.54	1878.695
DTX4	0.880219	0.119781	0.640647	0.640393	515.694	805.2837
CPEB3	0.880295	0.119705	0.640125	0.639115	132.2577	206.9444
DCAF11	0.880604	0.119396	1.596427	1.596612	2936.344	1839.106
ABCG4	0.880624	0.119376	1.674531	1.684233	66.68457	39.58937
PIH1D2	0.880792	0.119208	0.56259	0.557162	25.56242	45.88767
HINT3	0.880795	0.119205	0.635554	0.635393	836.8914	1317.129
VOPP1	0.881106	0.118894	1.60151	1.601673	3366.46	2101.835
BORCS5	0.881148	0.118852	0.62428	0.622436	72.24162	116.0688
LAMTOR1	0.881191	0.118809	0.627361	0.627267	1444.832	2303.381

FRG2B	0.881213	0.118787	0.140918	0.002771	0	3.599033
HIST2H2E	0.881213	0.118787	0.140918	0.002771	0	3.599033
LOC10537	0.881213	0.118787	0.140918	0.002771	0	3.599033
LOC10666	0.881213	0.118787	0.140918	0.002771	0	3.599033
MAG	0.881213	0.118787	0.140918	0.002771	0	3.599033
PAK7	0.881213	0.118787	0.140918	0.002771	0	3.599033
PPP1R14I	0.881213	0.118787	0.140918	0.002771	0	3.599033
SPATA12	0.881213	0.118787	0.140918	0.002771	0	3.599033
TCF4	0.881213	0.118787	0.140918	0.002771	0	3.599033
TMEM72	0.881213	0.118787	0.140918	0.002771	0	3.599033
WNT8B	0.881213	0.118787	0.140918	0.002771	0	3.599033
GSTO2	0.881225	0.118775	0.645055	0.644588	278.9638	432.7837
ERLEC1	0.881435	0.118565	1.560398	1.560835	1161.423	744.1001
PITPNA-A	0.881448	0.118552	0.637511	0.636352	113.3638	178.1521
ZNF211	0.881494	0.118506	0.643769	0.643432	387.8819	602.8381
AKNA	0.881655	0.118345	0.632254	0.632126	1016.94	1608.768
RGPD8	0.881735	0.118265	1.541006	1.542253	388.4932	251.8963
HABP2	0.881776	0.118224	2.149376	2.222058	20.00537	8.997583
INHA	0.881776	0.118224	2.149376	2.222058	20.00537	8.997583
OLAH	0.881776	0.118224	2.149376	2.222058	20.00537	8.997583
SDF2L1	0.882053	0.117947	0.629769	0.629663	1278.121	2029.855
ADSL	0.882291	0.117709	0.623939	0.623863	1776.033	2846.835
PHLDB2	0.882306	0.117694	1.642487	1.642555	8689.511	5290.237
ATRAID	0.882352	0.117648	0.629734	0.629627	1223.662	1943.478
LCMT2	0.882404	0.117596	0.645696	0.645241	285.6323	442.6811
C7orf49	0.882453	0.117547	0.628597	0.628498	1348.14	2145.024
SNAI2	0.882463	0.117537	2.148086	2.222058	20.00537	8.997583
NBPF10	0.882466	0.117534	1.588606	1.588813	2625.527	1652.505
CELF5	0.8826	0.1174	0.60646	0.603629	47.79061	79.17873
RASAL2-A	0.882648	0.117352	1.687373	1.698249	61.12753	35.99033
LUCAT1	0.882713	0.117287	0.245541	0.177514	1.11141	6.307306
PVRL1	0.882733	0.117267	0.644067	0.643763	437.8954	680.2173
FILIP1	0.882762	0.117238	1.544158	1.54496	596.8269	386.3022
LINC0102	0.882811	0.117189	0.381035	0.353436	4.445638	12.59662
TAF1A-AS	0.882886	0.117114	0.511822	0.501986	14.44832	28.79227
MMACHC	0.88297	0.11703	0.644156	0.643364	164.4886	255.6753
VSTM2L	0.882976	0.117024	4.164216	6.11926	5.557048	0.899758
SMYD2	0.882999	0.117001	1.545183	1.545891	691.2968	447.1799
FLVCR1	0.883062	0.116938	0.645534	0.644869	195.6081	303.3355
FOXI3	0.883149	0.116851	0.245862	0.177767	1.11141	6.298308
IDI2-AS1	0.883149	0.116851	0.245862	0.177767	1.11141	6.298308
KIF6	0.883149	0.116851	0.245862	0.177767	1.11141	6.298308
LOC10155	0.883149	0.116851	0.245862	0.177767	1.11141	6.298308
LOC10192	0.883149	0.116851	0.245862	0.177767	1.11141	6.298308
LOC10272	0.883149	0.116851	0.245862	0.177767	1.11141	6.298308
P2RY1	0.883149	0.116851	0.245862	0.177767	1.11141	6.298308
SH3BP1	0.883185	0.116815	1.552899	1.553415	965.8149	621.733
INPP4B	0.883232	0.116768	1.543206	1.544025	583.49	377.8985
FAM27E3	0.883306	0.116694	0.532652	0.524655	17.78255	33.90289
SLC31A1	0.883448	0.116552	1.590065	1.590262	2717.608	1708.902
TNFRSF1	0.883511	0.116489	1.541117	1.54264	312.3061	202.4456
SNX10	0.883534	0.116466	1.541329	1.542733	334.5343	216.8418
ZNF821	0.883612	0.116388	0.606257	0.603304	46.6792	77.37921
LHCGR	0.883733	0.116267	0.381387	0.353436	4.445638	12.59662
MROH8	0.883733	0.116267	0.381387	0.353436	4.445638	12.59662
RPGRIP1	0.883733	0.116267	0.381387	0.353436	4.445638	12.59662

SNX27	0.883768	0.116232	0.628077	0.627985	1447.055	2304.29
TERF1	0.883784	0.116216	0.635495	0.635351	933.7952	1469.737
BTG1	0.883803	0.116197	1.547358	1.547994	759.0927	490.3683
RBM15	0.884103	0.115897	1.53782	1.538957	422.3356	274.4263
NFYC-AS1	0.884214	0.115786	0.406778	0.383086	5.557048	14.5221
TAP2	0.884447	0.115553	1.601407	1.601555	3775.458	2357.367
VIPAS39	0.88445	0.11555	0.643028	0.642772	511.2484	795.3863
LCOR	0.884505	0.115495	1.557811	1.558238	1158.089	743.2004
BNC1	0.88456	0.11544	1.984591	2.028492	25.56242	12.59662
HOXC4	0.88456	0.11544	1.984591	2.028492	25.56242	12.59662
ADCY10P	0.884731	0.115269	4.148168	6.11926	5.557048	0.899758
C10orf105	0.884731	0.115269	4.148168	6.11926	5.557048	0.899758
CABP7	0.884731	0.115269	4.148168	6.11926	5.557048	0.899758
CLEC2B	0.884731	0.115269	4.148168	6.11926	5.557048	0.899758
FLJ16779	0.884731	0.115269	4.148168	6.11926	5.557048	0.899758
INHBA	0.884731	0.115269	4.148168	6.11926	5.557048	0.899758
LINC0121	0.884731	0.115269	4.148168	6.11926	5.557048	0.899758
LOC10574	0.884731	0.115269	4.148168	6.11926	5.557048	0.899758
MYRFL	0.884731	0.115269	4.148168	6.11926	5.557048	0.899758
NPAS4	0.884731	0.115269	4.148168	6.11926	5.557048	0.899758
PRDM9	0.884731	0.115269	4.148168	6.11926	5.557048	0.899758
SPRY4-IT	0.884731	0.115269	4.148168	6.11926	5.557048	0.899758
ZEB2	0.884731	0.115269	4.148168	6.11926	5.557048	0.899758
ZNF423	0.884731	0.115269	4.148168	6.11926	5.557048	0.899758
PEA15	0.88484	0.11516	1.681257	1.681286	22491.6	13377.61
ZC2HC1A	0.884886	0.115114	0.646901	0.646484	311.1947	481.3707
WBP2	0.884923	0.115077	0.619804	0.619749	2422.873	3909.45
ZRANB1	0.885009	0.114991	1.587119	1.587317	2720.731	1714.04
TAF6L	0.885104	0.114896	0.644957	0.644066	148.9289	231.2379
TAGLN3	0.885294	0.114706	1.983228	2.028492	25.56242	12.59662
FABP5	0.885294	0.114706	1.53843	1.539931	314.5289	204.2451
PDPR	0.885372	0.114628	1.552244	1.552735	995.2339	640.9518
SUSD1	0.885392	0.114608	1.536352	1.53741	452.3437	294.221
SLC50A1	0.885412	0.114588	0.619841	0.619786	2400.711	3873.46
KAT6A	0.885451	0.114549	0.637873	0.637711	811.329	1272.258
STAU2-AS	0.885484	0.114516	4.158012	6.192723	5.568162	0.890761
EIF1AD	0.88553	0.11447	1.555011	1.555458	1101.44	708.1098
ADGRG3	0.885548	0.114452	0.247039	0.177767	1.11141	6.298308
DCDC1	0.885548	0.114452	0.247039	0.177767	1.11141	6.298308
LOC1005C	0.885548	0.114452	0.247039	0.177767	1.11141	6.298308
RPL29P2	0.885548	0.114452	0.247039	0.177767	1.11141	6.298308
TLDC2	0.885548	0.114452	0.247039	0.177767	1.11141	6.298308
REEP2	0.885583	0.114417	0.640505	0.639361	114.4752	179.0519
TRIP10	0.885663	0.114337	1.612824	1.612938	4845.746	3004.293
SENP8	0.885724	0.114276	0.59526	0.591583	37.78793	63.88284
NOTCH2N	0.885754	0.114246	1.558294	1.558695	1234.876	792.2462
COG6	0.885898	0.114102	1.557175	1.557579	1245.89	799.8851
VPS11	0.885911	0.114089	0.641245	0.641046	669.0686	1043.72
CYP39A1	0.885948	0.114052	1.655695	1.664716	68.90739	41.38888
UTP23	0.88604	0.11396	0.647504	0.647096	317.8298	491.1691
RAB8B	0.886089	0.113911	1.555607	1.556043	1115.855	717.1074
RADIL	0.886116	0.113884	0.555357	0.549103	22.22819	40.48912
LOC10026	0.886265	0.113735	1.541369	1.543307	245.6215	159.1492
RFXANK	0.886444	0.113556	0.618661	0.618611	2765.187	4469.999
LOC10192	0.886563	0.113437	0.48778	0.475313	11.1141	23.39372
FCHO1	0.886605	0.113395	1.545036	1.545634	802.4377	519.1605

CACNA1H	0.886661	0.113339	1.543778	1.545943	221.1705	143.0616
SLFNL1-A	0.886821	0.113179	1.807184	1.829849	37.81015	20.65845
ARL6IP5	0.886943	0.113057	1.585964	1.586161	2657.38	1675.35
LOC28507	0.886951	0.113049	1.535112	1.536432	354.6508	230.824
AATK	0.887132	0.112868	0.488009	0.475313	11.1141	23.39372
BVES-AS1	0.887149	0.112851	4.125447	6.11926	5.557048	0.899758
GABRQ	0.887149	0.112851	4.125447	6.11926	5.557048	0.899758
LOC28493	0.887149	0.112851	4.125447	6.11926	5.557048	0.899758
MEGF11	0.887149	0.112851	4.125447	6.11926	5.557048	0.899758
PAGE1	0.887149	0.112851	4.125447	6.11926	5.557048	0.899758
SCARNA1	0.887149	0.112851	4.125447	6.11926	5.557048	0.899758
GREM1	0.887261	0.112739	1.546862	1.547402	892.4619	576.7451
ROCK1P1	0.88727	0.11273	0.633706	0.63213	83.6558	132.3454
PEX1	0.887274	0.112726	0.635718	0.635586	1002.491	1577.276
ARMCX5	0.887276	0.112724	0.648792	0.648251	239.8311	369.9716
USP44	0.887302	0.112698	0.646334	0.645493	154.4859	239.3357
TIMM44	0.88732	0.11268	0.627961	0.627879	1666.003	2653.387
BLOC1S1-	0.887352	0.112648	0.443542	0.425156	7.457558	17.55428
GUSBP11	0.887399	0.112601	0.637772	0.636356	94.46981	148.4601
ICAM5	0.887471	0.112529	1.567571	1.567866	1718.239	1095.906
UBE2E3	0.887506	0.112494	1.550746	1.551205	1081.513	697.2047
PP14571	0.887587	0.112413	0.477243	0.46346	10.00269	21.5942
STX11	0.887643	0.112357	1.565103	1.56868	141.149	89.97583
URGCP-M	0.887948	0.112052	1.856367	1.884912	32.80881	17.40133
SLC8A2	0.887958	0.112042	0.410197	0.386436	5.557048	14.39613
SMARCD2	0.888104	0.111896	0.628762	0.628678	1579.313	2512.125
MEIOC	0.888127	0.111873	0.605353	0.60223	43.34497	71.98066
NKX6-1	0.888157	0.111843	0.578351	0.573585	28.89665	50.38647
BMP2K	0.888178	0.111822	0.649453	0.649027	310.0833	477.7717
PCIF1	0.888243	0.111757	0.645561	0.645296	492.3544	762.995
TSACC	0.888261	0.111739	0.432069	0.412107	6.668457	16.19565
WAS	0.888261	0.111739	0.432069	0.412107	6.668457	16.19565
GPR27	0.888318	0.111682	0.464845	0.449453	8.891277	19.79468
WNK3	0.8884	0.1116	0.644549	0.643483	124.5001	193.484
C2CD3	0.888441	0.111559	0.640598	0.640423	749.0901	1169.686
NEMP2	0.888582	0.111418	0.613564	0.610876	50.01343	81.87801
INTS6-AS	0.888617	0.111383	0.450044	0.432646	7.779867	17.99517
LOC10050	0.888617	0.111383	0.450044	0.432646	7.779867	17.99517
MMEL1	0.888617	0.111383	0.450044	0.432646	7.779867	17.99517
NR2F2-AS	0.888617	0.111383	0.450044	0.432646	7.779867	17.99517
FAM135A	0.888688	0.111312	0.637733	0.637591	922.47	1446.811
STOM	0.88875	0.11125	1.584579	1.584769	2766.298	1745.549
SMCHD1	0.888765	0.111235	0.632824	0.632721	1303.683	2060.447
MSMO1	0.888791	0.111209	1.57775	1.577972	2338.406	1481.902
LHX6	0.889017	0.110983	1.577081	1.58162	116.698	73.78018
MESP1	0.889042	0.110958	0.465127	0.449453	8.891277	19.79468
TGIF2LY	0.889062	0.110938	6.346561	316.6403	3.156403	0
AP1S1	0.889166	0.110834	1.607145	1.60726	4887.979	3041.183
KIAA1257	0.889199	0.110801	0.432433	0.412107	6.668457	16.19565
LOC22072	0.889209	0.110791	1.530603	1.531962	347.1266	226.5861
ANKRD24	0.889445	0.110555	1.747109	1.764313	44.45638	25.19323
HLCS	0.889519	0.110481	0.630177	0.63009	1515.963	2405.954
EYA3	0.88953	0.11047	1.557383	1.55774	1385.239	889.2581
ALG1	0.889712	0.110288	0.650178	0.649576	215.1133	331.165
ASTE1	0.88973	0.11027	0.650023	0.64939	204.4994	314.9154
HOXC13-A	0.88975	0.11025	1.85632	1.884835	32.23088	17.09541

LOC10028	0.88988	0.11012	0.145604	0.002932	0	3.401086
LOC44089	0.88988	0.11012	0.145604	0.002932	0	3.401086
PHF7	0.889913	0.110087	0.576913	0.571954	27.78524	48.58695
KIF13A	0.889934	0.110066	0.628881	0.6288	1656	2633.593
TIGD4	0.88995	0.11005	0.549506	0.542421	20.00537	36.89009
WWC2	0.889974	0.110026	1.6133	1.613401	5590.39	3464.969
ANO10	0.890078	0.109922	1.54441	1.54491	975.8176	631.6303
UTP18	0.89013	0.10987	0.640827	0.640661	787.9894	1229.97
CETN3	0.890138	0.109862	0.649579	0.64918	320.086	493.0676
LINC0108	0.890184	0.109816	1.708017	1.721594	51.12484	29.69202
SPRY3	0.890394	0.109606	1.611659	1.61846	84.46713	52.18598
PCBD2	0.890462	0.109538	0.649639	0.648899	175.047	269.7655
RECQL5	0.890467	0.109533	0.647863	0.647571	444.5638	686.5156
PDCD6IPI	0.890692	0.109308	2.798279	3.123087	10.02491	3.20314
LFNG	0.890808	0.109192	1.60161	1.607654	91.13559	56.68477
POU4F1	0.890828	0.109172	0.593091	0.589181	34.4537	58.48429
COQ6	0.890829	0.109171	0.650545	0.650129	311.1947	478.6714
WASF1	0.891029	0.108971	0.650982	0.650529	285.6323	439.0821
SLC39A11	0.891087	0.108913	1.535848	1.536496	736.8646	479.5712
PRTG	0.891311	0.108689	0.651361	0.650797	228.9504	351.8055
FKBPL	0.891333	0.108667	0.651409	0.650892	250.0672	384.1968
C12orf43	0.891525	0.108475	0.651585	0.650912	195.6081	300.5193
GOLGA6D	0.891637	0.108363	2.752206	3.050306	10.20274	3.338103
KTN1-AS1	0.891702	0.108298	0.575367	0.570199	26.67383	46.78743
PRSS36	0.891841	0.108159	1.780431	1.801008	38.89934	21.5942
DDX11L2	0.891924	0.108076	1.704131	1.717671	50.86922	29.61105
GRIK5	0.891967	0.108033	2.521486	2.7137	12.22551	4.498792
LINC0051	0.891967	0.108033	2.521486	2.7137	12.22551	4.498792
MPZ	0.891967	0.108033	2.521486	2.7137	12.22551	4.498792
PINLYP	0.891967	0.108033	2.521486	2.7137	12.22551	4.498792
RNASE1	0.891967	0.108033	2.521486	2.7137	12.22551	4.498792
UTS2R	0.891967	0.108033	2.521486	2.7137	12.22551	4.498792
BCAS1	0.892122	0.107878	2.174711	2.259691	17.78255	7.863888
C5orf30	0.892422	0.107578	0.646532	0.646302	563.4847	871.8658
GSR	0.892546	0.107454	0.628702	0.628629	1808.263	2876.527
FNDC4	0.892622	0.107378	1.546291	1.549221	164.4886	106.1715
FGF11	0.892666	0.107334	1.595942	1.601836	92.247	57.58453
AARS2	0.892721	0.107279	0.647712	0.64746	516.1831	797.2488
ATG16L1	0.89274	0.10726	1.549652	1.550035	1291.458	833.1762
SUZ12	0.892824	0.107176	0.63345	0.633355	1384.583	2186.116
GLIDR	0.893058	0.106942	0.600423	0.596782	37.28779	62.48821
LINC0064	0.893086	0.106914	2.217983	2.314233	16.67114	7.198066
LOC64320	0.893086	0.106914	2.217983	2.314233	16.67114	7.198066
TMEM173	0.893093	0.106907	1.576079	1.576279	2635.152	1671.751
ISPD	0.893098	0.106902	0.610799	0.607757	44.28967	72.88042
GPR3	0.893131	0.106869	1.66762	1.678454	58.90471	35.09057
EFNB2	0.893146	0.106854	0.63762	0.637499	1086.959	1705.042
ITGA3	0.893188	0.106812	1.628965	1.629032	8611.201	5286.08
ZNF398	0.893215	0.106785	0.646013	0.6458	611.2753	946.5457
TCN2	0.893231	0.106769	0.649722	0.648772	138.9262	214.1425
CIART	0.893256	0.106744	0.651146	0.650365	165.6	254.6316
ZNF547	0.893293	0.106707	1.706463	1.72043	49.55775	28.80126
GPR107	0.893455	0.106545	1.613831	1.613922	6205	3844.667
SLC35E2	0.893643	0.106357	0.649577	0.649302	481.1737	741.0679
C1orf198	0.893698	0.106302	1.571494	1.571711	2398.422	1525.99
CAMKMT	0.893823	0.106177	0.650967	0.650136	155.5973	239.3357

MT1M	0.893874	0.106126	2.216302	2.314233	16.67114	7.198066
TTF1	0.893978	0.106022	1.526525	1.527421	511.2484	334.7101
MYL5	0.894032	0.105968	0.591554	0.587316	32.23088	54.88526
ZNF549	0.894043	0.105957	1.559573	1.563435	129.0013	82.50784
PLEKHS1	0.894079	0.105921	1.826235	1.852373	33.34229	17.99517
CMC1	0.894145	0.105855	0.651237	0.650906	391.2162	601.0385
FBXO16	0.894198	0.105802	0.637211	0.635547	78.91008	124.1666
PRKCG	0.894204	0.105796	1.570327	1.57484	113.3638	71.98066
TRIL	0.894226	0.105774	0.613138	0.610227	45.56779	74.67994
LOC10013	0.894254	0.105746	0.632636	0.630728	69.14079	109.6266
PSORS1C	0.894262	0.105738	1.825757	1.852373	33.34229	17.99517
SYN1	0.894262	0.105738	1.825757	1.852373	33.34229	17.99517
COLGALT	0.894339	0.105661	1.599572	1.59969	4611.216	2882.565
ZNF639	0.894352	0.105648	0.644441	0.644265	752.4243	1167.886
BOLA3	0.894477	0.105523	0.648508	0.648262	529.031	816.0808
FBXO4	0.894547	0.105453	0.634358	0.632471	71.13021	112.4698
VCP	0.894582	0.105418	1.626307	1.626376	8424.485	5179.909
PNRC1	0.894637	0.105363	1.556698	1.557	1634.883	1050.018
KIAA0586	0.894762	0.105238	0.650657	0.650373	463.4578	712.6086
CYB561A3	0.894919	0.105081	0.648541	0.648301	540.1451	833.1762
NTAN1	0.894925	0.105075	1.544691	1.545093	1213.659	785.489
SNCA	0.894966	0.105034	0.654356	0.653763	221.1705	338.3091
PARP14	0.895175	0.104825	1.586757	1.586908	3515.333	2215.205
PPP1R3F	0.895298	0.104702	0.653806	0.653353	284.5209	435.483
RIMKLB	0.895372	0.104628	0.650751	0.650464	451.2323	693.7137
UBE2C	0.895418	0.104582	1.582821	1.582985	3161.96	1997.463
LBR	0.895517	0.104483	0.643134	0.642976	831.3344	1292.953
ZNF561-A	0.895647	0.104353	0.590763	0.586289	31.11947	53.08574
CCDC157	0.895694	0.104306	0.538045	0.529534	16.67114	31.49154
LOC44002	0.895694	0.104306	0.538045	0.529534	16.67114	31.49154
TMEM223	0.895732	0.104268	0.654734	0.654262	277.8524	424.6859
LOC14870	0.895744	0.104256	0.590681	0.586289	31.11947	53.08574
ZNF552	0.895794	0.104206	0.654576	0.653905	195.4191	298.8547
RAB30-AS	0.895803	0.104197	0.652627	0.651815	158.9316	243.8345
INA	0.895828	0.104172	1.52541	1.526209	571.2645	374.2995
GNG4	0.896048	0.103952	0.613101	0.610049	44.03405	72.18761
GBP1P1	0.896061	0.103939	2.89278	3.285483	8.891277	2.699275
IPP	0.896139	0.103861	0.655238	0.654682	235.6188	359.9033
CRIPAK	0.896198	0.103802	0.621146	0.618536	51.12484	82.6608
MIER3	0.896225	0.103775	0.648233	0.648014	592.3813	914.1544
ALDH1L2	0.896275	0.103725	0.621434	0.62139	3016.366	4854.232
PIGP	0.896292	0.103708	0.652738	0.652404	385.6591	591.1412
ANO7	0.896314	0.103686	2.00478	2.057739	22.22819	10.7971
ZNF627	0.896328	0.103672	0.653727	0.653368	365.6538	559.6497
PDXK	0.896419	0.103581	1.621643	1.621715	7984.366	4923.405
RNF5	0.896502	0.103498	1.539359	1.542257	163.4439	105.9735
CBR3	0.896531	0.103469	1.53434	1.536922	181.1598	117.8683
NEDD9	0.896538	0.103462	1.598745	1.598858	4813.515	3010.591
FAM78A	0.896813	0.103187	2.003831	2.057739	22.22819	10.7971
SOX12	0.896912	0.103088	0.648626	0.648413	620.1665	956.4431
SNF8	0.896943	0.103057	0.641685	0.641551	998.0458	1555.682
A2M-AS1	0.897097	0.102903	2.888449	3.285483	8.891277	2.699275
ABCA17P	0.897097	0.102903	2.888449	3.285483	8.891277	2.699275
AMBP	0.897097	0.102903	2.888449	3.285483	8.891277	2.699275
ASIC4	0.897097	0.102903	2.888449	3.285483	8.891277	2.699275
PKDCC	0.897097	0.102903	2.888449	3.285483	8.891277	2.699275

TPRG1	0.897097	0.102903	2.888449	3.285483	8.891277	2.699275
STX16-NF	0.897113	0.102887	1.760017	1.780099	39.08827	21.9541
KLHL29	0.897229	0.102771	0.650374	0.650126	522.3625	803.4842
LINC0130	0.897263	0.102737	0.653964	0.653176	163.3772	250.1328
ARMC5	0.897495	0.102505	1.51977	1.521263	301.7366	198.3427
LOC49414	0.897578	0.102422	1.886121	1.921005	27.78524	14.45912
SEPT6	0.897832	0.102168	0.648252	0.648049	639.0605	986.1351
ANKRD20	0.897866	0.102134	6.11979	297.7464	2.967464	0
LOC65360	0.897867	0.102133	6.12065	303.3034	3.023034	0
CAPZB	0.897917	0.102083	1.618206	1.61828	7709.848	4764.22
CAB39	0.898024	0.101976	1.575467	1.575642	3019.7	1916.485
ABCD4	0.898026	0.101974	0.65534	0.654957	341.2027	520.9601
SCARB2	0.898076	0.101924	1.598305	1.598415	4882.422	3054.535
NIPAL3	0.898179	0.101821	1.576901	1.577071	3050.819	1934.48
SBNO2	0.898264	0.101736	1.553136	1.553422	1747.136	1124.698
ACOT4	0.898345	0.101655	0.631252	0.629091	61.12753	97.1739
DYRK3	0.898412	0.101588	0.648884	0.647683	107.8067	166.4553
C9orf40	0.89847	0.10153	1.518756	1.519714	469.0148	308.6171
CCDC68	0.898508	0.101492	1.698599	1.71313	47.79061	27.89251
ADAMTS6	0.898537	0.101463	2.882275	3.285483	8.891277	2.699275
HBQ1	0.898537	0.101463	2.882275	3.285483	8.891277	2.699275
JAM2	0.898537	0.101463	2.882275	3.285483	8.891277	2.699275
RBP1	0.898551	0.101449	1.555797	1.556066	1832.714	1177.784
EAF2	0.898574	0.101426	1.698863	1.71313	47.79061	27.89251
FZD9	0.898574	0.101426	1.698863	1.71313	47.79061	27.89251
SHMT2	0.89861	0.10139	0.62999	0.629925	2076.113	3295.815
ALG1L	0.898621	0.101379	0.258723	0.187954	1.11141	5.9564
CD164L2	0.898633	0.101367	1.699047	1.71313	47.79061	27.89251
ZNF256	0.898642	0.101358	1.529863	1.532385	183.3826	119.6679
PMM1	0.898794	0.101206	1.527026	1.527609	801.3263	524.5591
MED12	0.898806	0.101194	0.646264	0.646095	769.0954	1190.38
ADAMTS4	0.898945	0.101055	2.344848	2.483936	13.67034	5.497523
RAB12	0.898956	0.101044	0.640078	0.639964	1145.863	1790.519
CAB39L	0.899084	0.100916	1.568633	1.573563	103.3611	65.68236
SGSH	0.899166	0.100834	1.52791	1.528464	845.7827	553.3514
RHPN1-AS	0.899181	0.100819	0.567796	0.561579	22.22819	39.58937
C1orf54	0.8992	0.1008	0.527812	0.518173	14.44832	27.89251
ERVK13-1	0.899207	0.100793	0.646395	0.645013	94.01414	145.7608
ZBTB37	0.899212	0.100788	0.588833	0.584012	28.89665	49.48671
CERS6	0.899291	0.100709	0.631849	0.631778	1859.388	2943.109
LMO7	0.899321	0.100679	1.537088	1.53751	1113.632	724.3054
PTPDC1	0.899323	0.100677	0.656126	0.655719	315.6403	481.3707
PRR29-AS	0.899366	0.100634	0.324848	0.278496	2.300618	8.286774
HCAR1	0.899395	0.100605	0.532077	0.522823	15.04849	28.79227
LNX1	0.899549	0.100451	0.527991	0.518173	14.44832	27.89251
FGF13	0.899561	0.100439	1.529426	1.532024	177.8255	116.0688
COLCA1	0.89957	0.10043	3.936408	5.756911	5.279196	0.908756
RAB3D	0.899579	0.100421	1.56532	1.565527	2485.868	1587.875
PRSS3	0.89971	0.10029	0.363906	0.327729	3.334229	10.19426
GABRG3	0.899715	0.100285	0.32237	0.275391	2.222819	8.097825
MAGEA3	0.899817	0.100183	1.591707	1.598331	82.85558	51.83508
PDE8A	0.899837	0.100163	1.522685	1.523357	675.737	443.5808
PCSK6	0.899868	0.100132	1.637023	1.646804	62.23894	37.78985
GPATCH4	0.899966	0.100034	0.649032	0.648834	646.8404	996.9322
TMEM201	0.899984	0.100016	0.652098	0.651859	550.1477	843.9733
LOC10272	0.899985	0.100015	1.614856	1.622862	70.98573	43.73725

ZNF667	0.900019	0.099981	1.582393	1.588163	90.10197	56.72976
LAMB4	0.900118	0.099882	1.857119	1.888596	28.89665	15.29589
TC2N	0.900141	0.099859	1.559432	1.559663	2190.588	1404.523
GPT2	0.900191	0.099809	1.626719	1.626777	9877.097	6071.569
SUDS3	0.90024	0.09976	0.637262	0.637168	1412.602	2217.004
WDFY2	0.900383	0.099617	0.653647	0.652593	124.4779	190.7488
SLC25A19	0.900476	0.099524	0.658262	0.657641	210.0564	319.4142
ZFP2	0.900476	0.099524	0.429811	0.407266	5.857128	14.39613
NPIPB3	0.9005	0.0995	0.654495	0.654199	434.0277	663.4548
CR2	0.900506	0.099494	1.657571	1.66903	55.57048	33.29106
LINC0095	0.900572	0.099428	1.856134	1.888596	28.89665	15.29589
FXYD3	0.900602	0.099398	0.648769	0.647404	96.69263	149.3599
MLXIPL	0.900656	0.099344	0.631774	0.629532	58.90471	93.57486
SYNJ2BP-	0.900665	0.099335	6.043089	293.3007	2.923007	0
BCL2L1	0.900788	0.099212	1.605655	1.605743	6333.923	3944.54
C11orf65	0.900876	0.099124	0.322956	0.275391	2.222819	8.097825
LOC10192	0.900876	0.099124	0.322956	0.275391	2.222819	8.097825
LTK	0.900876	0.099124	0.322956	0.275391	2.222819	8.097825
ORAI1	0.900982	0.099018	0.65259	0.652348	532.3652	816.0808
LOC1005	0.901062	0.098938	0.521754	0.511317	13.33691	26.09299
TLX3	0.901062	0.098938	0.521754	0.511317	13.33691	26.09299
TTC41P	0.901062	0.098938	0.521754	0.511317	13.33691	26.09299
ASB9	0.901072	0.098928	2.116082	2.194492	17.78255	8.097825
SLC16A1-	0.901108	0.098892	1.68441	1.6982	48.90202	28.79227
MEIOB	0.901188	0.098812	1.68466	1.6982	48.90202	28.79227
CHRM4	0.901192	0.098808	1.612864	1.621097	70.0188	43.1884
ZNF814	0.901376	0.098624	1.550082	1.554286	118.0095	75.92161
SCN3B	0.901461	0.098539	1.774036	1.796298	35.56511	19.79468
CPE	0.901473	0.098527	0.649468	0.649278	672.4028	1035.622
C1orf210	0.901557	0.098443	0.658417	0.657993	307.8605	467.8833
CMSS1	0.901676	0.098324	0.644639	0.644503	959.1465	1488.2
ZNF512	0.901703	0.098297	0.653298	0.653051	522.3625	799.8851
ACTL8	0.901843	0.098157	1.948379	1.994523	23.3396	11.69686
NR4A1	0.901845	0.098155	1.514708	1.515566	517.9169	341.7282
ZNF407	0.901913	0.098087	0.639767	0.637898	70.0188	109.7705
CEP41	0.901936	0.098064	0.652503	0.652275	564.5961	865.5855
LOC10192	0.901986	0.098014	0.653764	0.652686	119.4098	182.9569
TOP2A	0.902004	0.097996	1.619173	1.619238	8651.212	5342.765
ADGRE2	0.902019	0.097981	2.307372	2.438514	13.83705	5.668477
S100A2	0.902201	0.097799	1.511783	1.512838	417.89	276.2258
ZNF792	0.902277	0.097723	1.594008	1.601102	77.79867	48.58695
PLA2G4B	0.902456	0.097544	0.634247	0.632053	60.005	94.9425
SLC35G6	0.902486	0.097514	0.323794	0.275391	2.222819	8.097825
SPERT	0.902486	0.097514	0.323794	0.275391	2.222819	8.097825
PCDHGB7	0.9025	0.0975	0.153956	0.00313	0	3.185144
DHRS12	0.902538	0.097462	0.627043	0.624448	51.12484	81.87801
ZNRF3-AS	0.902649	0.097351	0.543419	0.53474	16.31549	30.5198
HIST1H2A	0.90272	0.09728	5.985563	289.9665	2.889665	0
PRAME	0.902857	0.097143	1.556255	1.556484	2153.912	1383.828
ETV6	0.902881	0.097119	1.509537	1.510583	426.7813	282.5241
TXNRD3	0.902885	0.097115	1.510065	1.511378	334.5343	221.3405
CCDC171	0.902909	0.097091	0.586604	0.581377	26.67383	45.88767
CBR4	0.902952	0.097048	0.658407	0.657679	173.3799	263.6292
CRABP2	0.903049	0.096951	0.643826	0.643705	1096.961	1704.142
C8orf82	0.903211	0.096789	0.658117	0.657776	382.3249	581.2439
TMC8	0.903212	0.096788	1.578883	1.585105	85.57854	53.9855

ZFPM2-AS	0.903212	0.096788	1.578883	1.585105	85.57854	53.9855
GYG2	0.903296	0.096704	0.648617	0.648454	794.6578	1225.471
ACYP2	0.903368	0.096632	1.555583	1.56021	106.6953	68.38163
ATP9B	0.903463	0.096537	0.649906	0.648521	93.3584	143.9613
DDX58	0.903473	0.096527	1.547542	1.547812	1821.6	1176.884
SREBF1	0.903477	0.096523	0.648637	0.648477	829.1115	1278.557
EFNA2	0.903501	0.096499	2.327895	2.467744	13.33691	5.39855
LOC10050	0.903501	0.096499	2.327895	2.467744	13.33691	5.39855
MAP2	0.903527	0.096473	0.636629	0.636549	1681.563	2641.69
CCDC149	0.903547	0.096453	0.659572	0.659133	291.1893	441.7813
CCND3	0.903556	0.096444	1.558634	1.558846	2339.517	1500.797
BAIAP2-AS	0.903565	0.096435	0.654887	0.654634	508.3143	776.4914
SAT2	0.903598	0.096402	0.650578	0.650394	705.7451	1085.109
PDRG1	0.903699	0.096301	1.539191	1.539523	1422.604	924.0518
NME3	0.903703	0.096297	1.671468	1.684176	50.01343	29.69202
CTC-338M	0.903852	0.096148	1.543564	1.547519	121.1436	78.27897
FLOT1	0.903935	0.096065	0.624878	0.624835	3126.395	5003.556
EMSY	0.903989	0.096011	0.651021	0.650835	696.8538	1070.712
ANKRD34	0.904112	0.095888	1.619194	1.628101	64.46176	39.58937
TMEM65	0.904125	0.095875	1.520348	1.520894	840.2256	552.4516
CROCCP3	0.90413	0.09587	1.788625	1.813303	32.99775	18.19311
SURF1	0.904238	0.095762	0.657159	0.656872	455.6779	693.7137
IFIH1	0.904242	0.095758	1.515975	1.516633	676.8484	446.2801
LRRC75A	0.904272	0.095728	0.612351	0.608728	37.78793	62.08332
PARP11	0.904285	0.095715	1.61931	1.628101	64.46176	39.58937
HDAC5	0.904342	0.095658	1.510667	1.511518	516.8055	341.9082
HLX	0.904411	0.095589	2.325511	2.467744	13.33691	5.39855
PER3	0.904422	0.095578	0.66138	0.660792	221.1705	334.7101
MAP3K11	0.904426	0.095574	1.533854	1.534224	1260.338	821.4793
LYPD3	0.904463	0.095537	1.515997	1.518265	196.7195	129.5652
CETN4P	0.904643	0.095357	0.506801	0.494317	11.1141	22.49396
MAGEC1	0.904727	0.095273	1.750868	1.771915	36.67652	20.69444
CKB	0.904818	0.095182	1.540987	1.541291	1561.53	1013.128
PPP2R4	0.904976	0.095024	1.574403	1.574546	3656.538	2322.276
LOC10272	0.90511	0.09489	0.560061	0.552734	18.89396	34.19082
PMEL	0.905191	0.094809	1.504855	1.506229	321.1974	213.2427
KAT6B	0.905287	0.094713	0.653538	0.65333	620.1665	949.245
TMEM230	0.905368	0.094632	1.564724	1.564898	2959.684	1891.292
GRM6	0.90558	0.09442	3.639457	4.975028	5.59039	1.1157
KCTD14	0.905634	0.094366	1.57231	1.578257	86.50101	54.80428
LINC0100	0.905874	0.094126	0.56058	0.553225	18.81616	34.01986
RABL2B	0.905885	0.094115	0.661832	0.66133	253.7681	383.7289
CD99P1	0.905904	0.094096	1.658715	1.670976	51.12484	30.59178
LZTFL1	0.906041	0.093959	0.662085	0.6615	217.8363	329.3115
LOC10050	0.906066	0.093934	0.655974	0.654761	107.8067	164.6558
LOC64242	0.906114	0.093886	0.586635	0.581276	25.52908	43.9262
NOTCH1	0.906246	0.093754	1.521407	1.52188	973.5948	639.7282
CCNA2	0.906323	0.093677	1.549823	1.550057	2073.89	1337.941
SART3	0.906432	0.093568	0.647589	0.647454	964.7035	1490
S100A5	0.906552	0.093448	1.899681	1.940331	24.45101	12.59662
LCLAT1	0.906613	0.093387	0.659219	0.658913	416.7786	632.5301
TMED3	0.906684	0.093316	1.540224	1.540508	1699.345	1103.104
LARP6	0.906691	0.093309	0.650375	0.650217	820.2203	1261.461
LOC43999	0.906735	0.093265	0.587087	0.581605	25.41794	43.71026
ELK4	0.906745	0.093255	1.537956	1.53826	1552.95	1009.547
C12orf45	0.906907	0.093093	0.662646	0.662006	198.9423	300.5193

SNAI3	0.906922	0.093078	1.898947	1.940331	24.45101	12.59662
TSPAN9	0.90703	0.09297	1.52149	1.521944	1014.717	666.7209
IQCH-AS1	0.907122	0.092878	0.649877	0.648305	82.24431	126.8659
SH3D21	0.907224	0.092776	1.50755	1.50969	207.8336	137.663
PLAGL2	0.907226	0.092774	0.6403	0.640213	1511.517	2360.966
AMDHD1	0.907301	0.092699	0.373566	0.337551	3.334229	9.897341
CYP26C1	0.907301	0.092699	0.373566	0.337551	3.334229	9.897341
ESR2	0.907301	0.092699	0.373566	0.337551	3.334229	9.897341
MDH1B	0.907301	0.092699	0.373566	0.337551	3.334229	9.897341
PRIMA1	0.907446	0.092554	2.033328	2.098672	18.89396	8.997583
TATDN2	0.907448	0.092552	1.541892	1.542157	1826.046	1184.082
LOC10063	0.90745	0.09255	0.66065	0.660316	382.625	579.4623
LOC10050	0.907592	0.092408	1.729955	1.749564	37.78793	21.5942
ZNF436-A	0.907741	0.092259	1.602731	1.611024	66.68457	41.38888
CILP2	0.907864	0.092136	1.547618	1.552302	103.3611	66.58211
FKRP	0.908008	0.091992	0.663694	0.663134	226.7276	341.9082
MIA	0.908033	0.091967	2.59543	2.8599	10.00269	3.491062
B4GALT4	0.908034	0.091966	1.536996	1.537287	1645.998	1070.712
JAGN1	0.908062	0.091938	0.658656	0.658392	484.5746	736.0023
ATP5G3	0.908125	0.091875	0.626549	0.626508	3249.762	5187.107
CACNG8	0.908173	0.091827	1.555853	1.561089	94.3809	60.45476
MAPK11	0.90819	0.09181	0.660258	0.659204	123.3665	187.1497
HILPDA	0.908193	0.091807	1.50667	1.507376	627.9464	416.5791
MBTD1	0.908199	0.091801	0.661469	0.661141	396.7732	600.1388
TRIM45	0.908213	0.091787	0.658283	0.65715	112.9303	171.8538
BBS10	0.908334	0.091666	0.663827	0.663319	250.0672	376.9987
MFHAS1	0.908377	0.091623	0.655098	0.654903	673.5142	1028.424
AIMP2	0.908388	0.091612	0.655216	0.655016	642.3947	980.7366
GTF3C6	0.90848	0.09152	0.661257	0.66093	390.1048	590.2414
NANOS1	0.908529	0.091471	0.66237	0.661982	323.4202	488.5688
LOC40086	0.908553	0.091447	0.374209	0.337551	3.334229	9.897341
SIX3-AS1	0.908553	0.091447	0.374209	0.337551	3.334229	9.897341
SAP30	0.90857	0.09143	0.662501	0.662125	338.9799	511.9625
LOC10050	0.908645	0.091355	0.582419	0.576546	23.3396	40.48912
FBXO10	0.908656	0.091344	0.664544	0.664051	262.2927	394.9939
PCDHA2	0.908665	0.091335	5.812986	279.9638	2.789638	0
C6orf226	0.908704	0.091296	0.582528	0.576546	23.3396	40.48912
GTF2IRD1	0.908865	0.091135	0.653715	0.653538	727.9733	1113.901
DSC3	0.908956	0.091044	1.501379	1.50309	250.8785	166.9052
MAPK8IP2	0.908974	0.091026	0.659934	0.658809	115.5866	175.4529
DPY19L1F	0.908995	0.091005	1.786368	1.812362	31.2195	17.22137
FDX1L	0.909031	0.090969	0.659058	0.658795	480.1289	728.8042
FOXK2	0.909038	0.090962	0.626085	0.626046	3425.364	5471.43
HSBP1L1	0.909083	0.090917	0.65723	0.65602	104.4725	159.2572
LOC10192	0.909095	0.090905	0.472954	0.455404	7.779867	17.09541
GJA3	0.909175	0.090825	0.599518	0.594824	28.89665	48.58695
ZNF587B	0.909222	0.090778	0.64812	0.646281	71.74149	111.0122
NXT2	0.909252	0.090748	1.497451	1.498793	322.3088	215.0422
TUBBP5	0.909256	0.090744	3.563059	4.829601	5.557048	1.142693
JAG2	0.909267	0.090733	1.518234	1.518691	982.4861	646.9262
NBPF9	0.909278	0.090722	1.545167	1.5454	2057.508	1331.372
DNAJB14	0.909342	0.090658	1.522644	1.523045	1129.192	741.4008
UBE2Q1	0.909349	0.090651	0.633261	0.633205	2325.069	3671.914
SLC16A10	0.909432	0.090568	0.611731	0.607881	34.4537	56.68477
ZP3	0.9095	0.0905	0.633617	0.630943	50.15791	79.50264
ASNS	0.90955	0.09045	0.596781	0.596772	16737.83	28047.27

SFXN5	0.909616	0.090384	0.664278	0.663599	184.494	278.0253
CXCL5	0.909624	0.090376	1.502249	1.503061	530.1424	352.7053
CCNA1	0.909676	0.090324	1.498787	1.500327	276.741	184.4505
LIMS2	0.909709	0.090291	1.498122	1.499264	372.3222	248.3333
CYB561D2	0.909715	0.090285	0.664567	0.663844	175.6027	264.5289
LOC10192	0.909753	0.090247	0.40934	0.380601	4.445638	11.69686
MCIDAS	0.909753	0.090247	0.40934	0.380601	4.445638	11.69686
OXTR	0.909896	0.090104	1.577802	1.584702	75.57585	47.68719
LINC0095	0.90996	0.09004	0.435177	0.411356	5.545934	13.49637
EVI5	0.909983	0.090017	1.508681	1.50926	769.9734	510.163
L1CAM	0.910009	0.089991	1.659244	1.659268	26102.57	15731.37
FAM66C	0.910022	0.089978	0.456557	0.436332	6.668457	15.29589
LOC10012	0.910142	0.089858	1.877408	1.917009	24.63995	12.84855
TSNAX	0.910153	0.089847	0.651079	0.650939	941.1083	1445.777
EFEMP1	0.910342	0.089658	1.523256	1.523633	1203.657	789.9878
DENND2A	0.910371	0.089629	1.496685	1.498023	316.7517	211.4432
PRRX2	0.91039	0.08961	0.662132	0.661127	126.7007	191.6485
CYP4V2	0.910485	0.089515	0.650962	0.649271	76.3205	117.5534
LOC10029	0.9105	0.0895	0.661345	0.661071	473.4605	716.2076
RORA	0.910611	0.089389	1.857208	1.893359	25.56242	13.49637
MYEOV	0.910613	0.089387	1.608236	1.617401	61.12753	37.78985
LOC10004	0.910701	0.089299	0.580921	0.574636	22.22819	38.68961
LOC10192	0.910793	0.089207	0.409857	0.380601	4.445638	11.69686
GPR179	0.910803	0.089197	0.456925	0.436332	6.668457	15.29589
UQCC2	0.910839	0.089161	0.648502	0.648383	1089.181	1679.849
HHAT	0.910881	0.089119	1.511635	1.514451	156.7088	103.4722
BRD9	0.910892	0.089108	0.611668	0.607562	33.34229	54.88526
TPST1	0.910902	0.089098	1.498255	1.499154	473.4605	315.8152
CA5BP1	0.910949	0.089051	1.500599	1.502612	212.8349	141.64
ELF3	0.910957	0.089043	0.632639	0.632587	2582.916	4083.103
DGCR8	0.911036	0.088964	0.658686	0.658469	600.1612	911.4552
OSBP2	0.911055	0.088945	0.665844	0.665137	178.9369	269.0277
LINC0011	0.911269	0.088731	0.436397	0.412179	5.557048	13.49637
SHBG	0.911269	0.088731	0.436397	0.412179	5.557048	13.49637
ZNF660	0.911269	0.088731	0.436397	0.412179	5.557048	13.49637
GOLGA6L	0.911279	0.088721	1.728362	1.74847	36.06524	20.62246
ZNF17	0.911398	0.088602	0.665697	0.664927	164.522	247.4335
SLC35G2	0.911429	0.088571	1.498103	1.498955	499.0229	332.9106
FAM89A	0.911439	0.088561	1.528698	1.532806	114.4752	74.67994
HACD4	0.911482	0.088518	1.535548	1.539951	106.6953	69.28139
MCCC2	0.91149	0.08851	0.642665	0.642581	1544.859	2404.154
ZNF823	0.911496	0.088504	1.495642	1.497428	241.1759	161.0567
IREB2	0.911599	0.088401	0.648692	0.648575	1110.332	1711.961
LOC10050	0.911652	0.088348	0.643578	0.641408	60.01612	93.57486
PTOV1	0.911695	0.088305	1.560458	1.560614	3156.17	2022.387
LINC0031	0.911847	0.088153	2.184955	2.29195	14.44832	6.298308
SAMD12	0.91192	0.08808	0.663618	0.663303	403.4417	608.2366
PLA2G15	0.911976	0.088024	1.501688	1.502368	631.2806	420.1871
VMA21	0.911993	0.088007	0.650569	0.650441	1012.461	1556.582
MEPCE	0.912083	0.087917	0.642863	0.642779	1561.53	2429.347
UBXN1	0.912084	0.087916	0.649203	0.649086	1134.749	1748.23
LETM2	0.912097	0.087903	0.663284	0.662983	422.3356	637.0289
PANK1	0.912108	0.087892	0.661218	0.660966	504.78	763.7059
LINC0032	0.912187	0.087813	2.532473	2.77434	10.00269	3.599033
LPO	0.912187	0.087813	2.532473	2.77434	10.00269	3.599033
TREML2	0.912187	0.087813	2.532473	2.77434	10.00269	3.599033

BMP8B	0.912193	0.087807	1.498745	1.50095	197.0418	131.2747
GLI2	0.912372	0.087628	1.539003	1.543741	100.0269	64.7916
POLR2J2	0.912393	0.087607	0.548028	0.538843	15.09294	28.01847
OBSL1	0.912439	0.087561	0.647326	0.647221	1239.222	1914.686
COPS7A	0.912498	0.087502	0.658843	0.658635	615.7209	934.8489
RMDN1	0.91251	0.08749	0.662043	0.661787	507.9142	767.4938
EPHA8	0.912598	0.087402	2.183105	2.29195	14.44832	6.298308
LOC44249	0.912598	0.087402	2.183105	2.29195	14.44832	6.298308
SNORA8	0.912598	0.087402	2.183105	2.29195	14.44832	6.298308
SBK2	0.912647	0.087353	1.964788	2.020257	20.00537	9.897341
IGIP	0.912656	0.087344	1.506936	1.509679	158.9316	105.2717
BRMS1L	0.912673	0.087327	0.665325	0.664972	358.9853	539.855
CLEC16A	0.91272	0.08728	0.654307	0.654153	835.78	1277.657
PTPRG-AS	0.912736	0.087264	0.624737	0.621328	39.68844	63.88284
FAM171B	0.912932	0.087068	0.667646	0.66715	254.5128	381.4975
LIMS1	0.913069	0.086931	1.570461	1.570582	4229.236	2692.779
TUBGCP5	0.913118	0.086882	0.655127	0.654971	838.0028	1279.456
ZNF471	0.913174	0.086826	1.540781	1.545741	95.97022	62.08332
OBFC1	0.913211	0.086789	1.543423	1.548557	93.3584	60.28381
TMEM47	0.913216	0.086784	0.6526	0.652465	978.0404	1498.997
PPP2R2A	0.913234	0.086766	1.550412	1.550593	2694.057	1737.433
MMP25	0.913271	0.086729	2.528539	2.77434	10.00269	3.599033
TESC	0.913271	0.086729	2.528539	2.77434	10.00269	3.599033
ZIK1	0.913271	0.086729	2.528539	2.77434	10.00269	3.599033
ZNF726	0.913271	0.086729	2.528539	2.77434	10.00269	3.599033
MIR205HG	0.913345	0.086655	0.545036	0.535439	14.44832	26.99275
NWD1	0.913378	0.086622	1.962115	2.018424	20.00537	9.906339
FNDC3B	0.913385	0.086615	1.566196	1.566327	3885.488	2480.634
EP300	0.913413	0.086587	0.649634	0.649519	1131.415	1741.932
ZNF74	0.913445	0.086555	0.667018	0.666639	340.0913	510.163
SNX12	0.91349	0.08651	0.651202	0.651078	1038.057	1594.372
RBM15B	0.913494	0.086506	0.644495	0.64441	1550.416	2405.954
FRG1DP	0.913668	0.086332	5.668828	276.6296	2.756296	0
SNX16	0.91373	0.08627	1.500336	1.500967	677.9598	451.6787
DDX20	0.913787	0.086213	0.667146	0.666775	346.7598	520.0603
RSRC2	0.914006	0.085994	1.527575	1.527867	1549.305	1014.028
NBR2	0.914034	0.085966	0.621312	0.61768	36.67652	59.38405
LOC40105	0.914045	0.085955	0.599943	0.594886	26.79608	45.0509
GS1-259H	0.914145	0.085855	1.819839	1.852255	26.67383	14.39613
ABR	0.914172	0.085828	1.5303	1.530566	1768.253	1155.29
GPSM1	0.914219	0.085781	1.491088	1.491977	477.9061	320.314
FAM177A1	0.914236	0.085764	1.515958	1.51634	1169.203	771.0659
C19orf44	0.914263	0.085737	0.666884	0.665967	137.8148	206.9444
ST3GAL4-	0.914274	0.085726	0.651532	0.649595	67.79598	104.372
FAM76A	0.914291	0.085709	0.668759	0.668046	176.7141	264.5289
ARHGEF3	0.914293	0.085707	0.661449	0.661224	564.5961	853.8706
SH3BGRL	0.914371	0.085629	1.574262	1.574368	4932.436	3132.958
LOC10192	0.914376	0.085624	0.546463	0.53687	14.44832	26.92077
ZNF582	0.914419	0.085581	1.819251	1.852255	26.67383	14.39613
ATP6V1B1	0.914445	0.085555	0.611175	0.606857	31.11947	51.28622
KBTBD12	0.914456	0.085544	1.741164	1.763699	33.03109	18.72397
EMC10	0.914457	0.085543	0.645758	0.645668	1458.169	2258.393
ASPHD1	0.914483	0.085517	1.513273	1.51367	1135.861	750.3984
MPND	0.91466	0.08534	0.668469	0.668041	294.5235	440.8816
JMJD4	0.914685	0.085315	0.669003	0.668573	298.9692	447.1799
GAGE10	0.914732	0.085268	0.164157	0.003377	0	2.951207

ACOT2	0.914782	0.085218	0.659003	0.657525	86.38987	131.3917
POM121L	0.914865	0.085135	0.280347	0.207929	1.16698	5.650482
COL9A2	0.914922	0.085078	0.648979	0.648874	1245.89	1920.084
LINC0053(	0.914943	0.085057	3.053002	3.690741	6.668457	1.799517
CDC6	0.915088	0.084912	0.644537	0.644456	1640.441	2545.47
KIAA1324	0.915112	0.084888	1.941827	1.995487	20.31657	10.17627
C17orf96	0.915116	0.084884	1.487658	1.488832	352.3168	236.6364
ZNF227	0.915121	0.084879	1.490881	1.491791	452.3437	303.2185
ARHGAP1	0.915321	0.084679	1.559668	1.566356	76.05376	48.55096
TGFB1I1	0.915461	0.084539	1.492772	1.493494	591.2699	395.8937
LINC0155	0.915482	0.084518	5.61235	273.2953	2.722953	0
LOC1001	0.915546	0.084454	0.641032	0.638479	51.12484	80.07849
DMPK	0.915587	0.084413	1.505356	1.505832	909.133	603.7378
FGF12	0.915605	0.084395	0.668459	0.667566	141.149	211.4432
TIGD6	0.915651	0.084349	0.670285	0.669751	235.6188	351.8055
ZNF136	0.915664	0.084336	0.659016	0.657485	83.35572	126.7849
RHOC	0.915757	0.084243	1.573579	1.573683	4955.775	3149.154
TTC4	0.915786	0.084214	0.66441	0.664153	494.5995	744.712
RPL7A	0.915797	0.084203	1.646214	1.646239	24052.01	14610.28
ZDHHC14	0.915804	0.084196	1.505278	1.508353	141.149	93.57486
LOC1002	0.915864	0.084136	0.657352	0.655652	76.68726	116.9686
NADK	0.915917	0.084083	0.659794	0.659613	715.7478	1085.109
TMBIM1	0.915934	0.084066	1.578074	1.578169	5395.894	3419.082
BTN2A1	0.915935	0.084065	1.517289	1.51763	1313.097	865.2256
ANGPTL6	0.915987	0.084013	3.046527	3.690741	6.668457	1.799517
C9orf152	0.915987	0.084013	3.046527	3.690741	6.668457	1.799517
LCAL1	0.915987	0.084013	3.046527	3.690741	6.668457	1.799517
LOC4406(	0.915987	0.084013	3.046527	3.690741	6.668457	1.799517
OLFM1	0.915987	0.084013	3.046527	3.690741	6.668457	1.799517
USP30-AS	0.915987	0.084013	3.046527	3.690741	6.668457	1.799517
EXOSC9	0.916124	0.083876	0.666202	0.665912	436.784	655.9238
TMEM14A	0.916134	0.083866	1.498144	1.498737	707.9679	472.3731
LINC0093	0.916164	0.083836	0.66473	0.663517	104.4725	157.4577
SNX17	0.916175	0.083825	0.648787	0.648689	1322.577	2038.852
OTULIN	0.916332	0.083668	1.519782	1.520097	1429.273	940.2474
DOCK8	0.916358	0.083642	0.645727	0.643391	55.57048	86.3768
OSBPL5	0.916391	0.083609	1.517304	1.521373	112.2524	73.78018
TMEM175	0.916416	0.083584	0.671502	0.6708	182.2712	271.727
TRAPPC2	0.916446	0.083554	0.671675	0.671119	230.0618	342.8079
ANKDD1A	0.91645	0.08355	1.56382	1.570802	72.24162	45.98665
SRP68	0.916559	0.083441	0.643645	0.643571	1763.807	2740.664
CDH2	0.916614	0.083386	0.670406	0.669964	284.5209	424.6859
HECW1	0.91668	0.08332	1.907687	1.954898	21.11678	10.7971
TRAPPC6	0.916694	0.083306	0.66386	0.663633	569.0417	857.4697
CEP290	0.916743	0.083257	0.667373	0.667066	412.333	618.134
MAFA	0.916934	0.083066	1.622845	1.634657	50.01343	30.59178
IGF2	0.917251	0.082749	1.786706	1.815983	27.78524	15.29589
LOC6132(	0.917255	0.082745	0.671163	0.670384	161.1433	240.3794
LOC1053	0.91739	0.08261	1.906232	1.954898	21.11678	10.7971
C9orf163	0.917433	0.082567	3.037318	3.690741	6.668457	1.799517
KBTBD11-	0.917433	0.082567	3.037318	3.690741	6.668457	1.799517
LINC0106	0.917433	0.082567	3.037318	3.690741	6.668457	1.799517
NFASC	0.917433	0.082567	3.037318	3.690741	6.668457	1.799517
PHYHIP	0.917433	0.082567	3.037318	3.690741	6.668457	1.799517
DCAF13	0.917466	0.082534	1.556243	1.55638	3678.766	2363.665
ZNF208	0.917503	0.082497	2.952953	3.517208	6.924082	1.961473

NDUFAF1	0.917518	0.082482	0.67174	0.671251	256.7356	382.4783
C19orf73	0.917523	0.082477	0.53414	0.522802	12.22551	23.39372
ZNF157	0.917523	0.082477	0.53414	0.522802	12.22551	23.39372
LOC10192	0.917705	0.082295	2.076124	2.160043	15.55973	7.198066
MAP3K8	0.917811	0.082189	1.49482	1.495385	760.2042	508.3634
RRP1	0.917817	0.082183	0.659506	0.659343	799.1035	1211.974
LOC40092	0.91782	0.08218	1.609047	1.619668	52.89198	32.65223
DUOX2	0.917862	0.082138	5.525357	263.2927	2.622927	0
C19orf54	0.917902	0.082098	0.672321	0.671802	241.1759	359.0036
TROVE2	0.917956	0.082044	1.504266	1.504698	999.1572	664.0216
PHF20	0.918012	0.081988	0.652838	0.652725	1142.062	1749.688
ACKR3	0.918152	0.081848	2.075086	2.160043	15.55973	7.198066
NRL	0.918152	0.081848	2.075086	2.160043	15.55973	7.198066
PSMD6-A	0.918152	0.081848	2.075086	2.160043	15.55973	7.198066
CNNM1	0.918169	0.081831	3.001128	3.618766	6.668457	1.835507
ZC3H18	0.91825	0.08175	0.65883	0.658674	820.2203	1245.265
CREB3L2	0.918265	0.081735	0.642231	0.642166	2020.543	3146.455
FOXO3B	0.918314	0.081686	0.625165	0.621452	35.7207	57.48556
COL21A1	0.918407	0.081593	0.281965	0.20734	1.11141	5.39855
LOC10192	0.918407	0.081593	0.281965	0.20734	1.11141	5.39855
LOC38970	0.918407	0.081593	0.281965	0.20734	1.11141	5.39855
H6PD	0.91842	0.08158	0.659775	0.659611	781.3209	1184.523
THOC3	0.918499	0.081501	1.563982	1.564096	4323.272	2764.067
SLC25A46	0.918524	0.081476	0.643433	0.643366	1971.641	3064.577
ZBTB8B	0.918549	0.081451	1.699005	1.718236	35.56511	20.69444
LRRC61	0.918565	0.081435	0.670933	0.670561	337.8685	503.8647
TPM3P9	0.918575	0.081425	1.482072	1.48306	412.333	278.0253
LINC0062	0.918622	0.081378	0.595465	0.589646	23.3396	39.58937
DDX5	0.918627	0.081373	0.618697	0.618677	6804.049	10997.75
NECAB2	0.918716	0.081284	1.538154	1.543938	83.35572	53.9855
RGS16	0.918752	0.081248	1.522211	1.526802	98.91545	64.7826
HMCN2	0.918785	0.081215	2.073601	2.160043	15.55973	7.198066
HSD17B1	0.918818	0.081182	0.644661	0.64459	1819.377	2822.542
LOC6543	0.918881	0.081119	0.624091	0.620177	34.55372	55.72203
TRAF3IP1	0.918923	0.081077	0.672133	0.671718	302.3034	450.0501
NRGN	0.918957	0.081043	0.668846	0.667713	111.141	166.4553
ARL17A	0.919003	0.080997	0.672914	0.672134	160.4209	238.6789
FGFR1OP	0.919074	0.080926	0.658436	0.658289	872.4565	1325.344
STARD13	0.919096	0.080904	1.478108	1.479327	336.7571	227.6389
CT45A10	0.919125	0.080875	3.111643	3.862189	6.179437	1.592572
METTL18	0.919141	0.080859	0.673723	0.672938	162.2658	241.1352
CEP85	0.91919	0.08081	1.515964	1.516263	1517.074	1000.531
ZNF593	0.919284	0.080716	0.667627	0.667366	484.5746	726.105
NEU1	0.919298	0.080702	0.639718	0.639664	2450.658	3831.171
ID2	0.919465	0.080535	0.655279	0.65516	1095.85	1672.651
ATF5	0.919614	0.080386	0.662839	0.662653	686.8511	1036.522
EML2	0.919627	0.080373	1.560085	1.560203	4210.019	2698.375
METTL1	0.919645	0.080355	0.671453	0.6711	355.6511	529.9576
DBIL5P	0.919662	0.080338	0.282866	0.20734	1.11141	5.39855
DEGS2	0.919662	0.080338	0.282866	0.20734	1.11141	5.39855
EEF1DP3	0.919662	0.080338	0.282866	0.20734	1.11141	5.39855
FAM184B	0.919662	0.080338	0.282866	0.20734	1.11141	5.39855
HELT	0.919662	0.080338	0.282866	0.20734	1.11141	5.39855
HIST4H4	0.919662	0.080338	0.282866	0.20734	1.11141	5.39855
LINC0138	0.919662	0.080338	0.282866	0.20734	1.11141	5.39855
LOC10192	0.919662	0.080338	0.282866	0.20734	1.11141	5.39855

LOC10192	0.919662	0.080338	0.282866	0.20734	1.11141	5.39855
LOC10669	0.919662	0.080338	0.282866	0.20734	1.11141	5.39855
MATN1	0.919662	0.080338	0.282866	0.20734	1.11141	5.39855
MYRIP	0.919662	0.080338	0.282866	0.20734	1.11141	5.39855
SERPINF1	0.919662	0.080338	0.282866	0.20734	1.11141	5.39855
WFDC3	0.919662	0.080338	0.282866	0.20734	1.11141	5.39855
RPS6KB1	0.919693	0.080307	0.6613	0.661134	785.7666	1188.518
DSG2	0.919707	0.080293	1.591821	1.591885	8381.995	5265.449
SMG8	0.919768	0.080232	0.667394	0.667146	509.0256	762.995
E2F7	0.919794	0.080206	0.675021	0.674418	211.1678	313.1159
RCOR3	0.919875	0.080125	0.666988	0.666748	527.9196	791.7873
PRDM4	0.919921	0.080079	0.66515	0.664939	601.2726	904.2571
TECPR2	0.919971	0.080029	0.669715	0.669431	452.3437	675.7185
EML6	0.920003	0.079997	1.757127	1.783739	28.89665	16.19565
RASL11B	0.920003	0.079997	1.757127	1.783739	28.89665	16.19565
SPECC1L	0.920015	0.079985	1.477301	1.478617	305.4376	206.5665
SULT1A3	0.920182	0.079818	1.491343	1.494269	145.6391	97.46182
SULT1A4	0.920182	0.079818	1.491343	1.494269	145.6391	97.46182
LMBRD2	0.920191	0.079809	0.674471	0.674014	278.9638	413.8888
REM1	0.920205	0.079795	1.756642	1.783739	28.89665	16.19565
MIS12	0.920227	0.079773	0.673978	0.673523	274.5182	407.5905
CCDC94	0.920365	0.079635	0.672664	0.671709	131.1463	195.2476
DZIP1	0.92043	0.07957	1.491952	1.492477	812.4404	544.3538
ZNF189	0.920445	0.079555	0.67503	0.674547	263.4041	390.4951
ATP2B1	0.920447	0.079553	0.667422	0.66719	553.482	829.5772
MAP1A	0.920604	0.079396	0.594426	0.588314	22.22819	37.78985
HIST3H2A	0.920668	0.079332	1.680186	1.69812	36.67652	21.5942
PGLS	0.920672	0.079328	0.650476	0.650387	1444.832	2221.503
SYNPO2	0.920678	0.079322	1.632595	1.646187	44.45638	27.00175
UBE2J1	0.920692	0.079308	1.565352	1.565454	5036.908	3217.536
SCYL3	0.920703	0.079297	0.675891	0.675218	188.9396	279.8248
ADAM11	0.920797	0.079203	1.555641	1.562818	68.90739	44.08816
LOC10192	0.920821	0.079179	0.643327	0.64054	46.6792	72.88042
HEATR3	0.920833	0.079167	0.661864	0.6617	775.7639	1172.385
MBOAT7	0.920868	0.079132	1.586442	1.58651	7971.03	5024.25
NUDT13	0.920946	0.079054	0.569865	0.561616	16.67114	29.69202
SCARF1	0.920962	0.079038	0.559024	0.549845	14.83732	26.99275
AFG3L1P	0.92103	0.07897	0.660612	0.658822	71.13021	107.971
STK25	0.921058	0.078942	1.525062	1.525288	2057.219	1348.738
RNF6	0.921117	0.078883	0.654866	0.654756	1165.869	1780.622
FXN	0.921133	0.078867	0.675284	0.674526	164.4997	243.8795
CRX	0.921163	0.078837	0.658717	0.656807	66.82906	101.7537
GRTP1	0.921188	0.078812	0.676397	0.675814	217.7585	322.2214
SLFN11	0.921193	0.078807	3.511808	4.956946	4.990229	0.998732
OSGEPL1	0.921268	0.078732	0.316962	0.255287	1.60043	6.298308
AQR	0.921287	0.078713	0.66118	0.661028	854.674	1292.953
HMOX1	0.921298	0.078702	1.498236	1.498654	1036.945	691.9141
CRYGN	0.921387	0.078613	0.284154	0.20734	1.11141	5.39855
HIST2H3D	0.921387	0.078613	0.284154	0.20734	1.11141	5.39855
NPFFR1	0.921387	0.078613	0.284154	0.20734	1.11141	5.39855
OLIG2	0.921387	0.078613	0.284154	0.20734	1.11141	5.39855
PTCSC3	0.921387	0.078613	0.284154	0.20734	1.11141	5.39855
TDRD5	0.921387	0.078613	0.284154	0.20734	1.11141	5.39855
NINL	0.921469	0.078531	0.673695	0.672725	128.9235	191.6485
PBRM1	0.921481	0.078519	0.657241	0.657117	1016.94	1547.584
SYT12	0.92149	0.07851	0.669476	0.668136	95.58122	143.0616

KIAA0907	0.921581	0.078419	1.52876	1.528967	2227.265	1456.709
PRR18	0.921634	0.078366	0.519394	0.505572	10.00269	19.79468
SELPLG	0.921656	0.078344	0.610137	0.605101	26.67383	44.08816
NCOA3	0.921677	0.078323	0.631191	0.631157	3988.849	6319.902
ANKRD19	0.921731	0.078269	2.302636	2.467201	11.1141	4.498792
CYS1	0.921731	0.078269	2.302636	2.467201	11.1141	4.498792
GABRR2	0.921731	0.078269	2.302636	2.467201	11.1141	4.498792
LINC0122	0.921731	0.078269	2.302636	2.467201	11.1141	4.498792
SSSCA1-/	0.921731	0.078269	2.302636	2.467201	11.1141	4.498792
AKR1C1	0.921767	0.078233	1.486476	1.489186	152.2853	102.2575
ANKRD50	0.921801	0.078199	1.502044	1.50242	1143.64	761.1955
KIF21B	0.9221	0.0779	1.508599	1.508914	1384.816	917.7535
METTL21I	0.922189	0.077811	1.474621	1.476399	224.5047	152.0592
DDB2	0.922222	0.077778	1.498281	1.501866	118.9208	79.17873
KCNJ14	0.922251	0.077749	1.620793	1.633466	45.56779	27.89251
AGL	0.92228	0.07772	0.669834	0.669583	501.2457	748.5989
ADM2	0.922445	0.077555	0.659875	0.659741	971.1386	1472.005
APBB1	0.92246	0.07754	1.73056	1.754887	30.00806	17.09541
PARD6B	0.922487	0.077513	0.677805	0.677185	204.5216	302.0219
MYLIP	0.922577	0.077423	1.507367	1.507685	1371.479	909.6556
CR1L	0.922592	0.077408	2.299883	2.467201	11.1141	4.498792
KRT13	0.92266	0.07734	1.662526	1.679612	37.78793	22.49396
GALNT2	0.922695	0.077305	1.528396	1.528595	2310.621	1511.594
IGSF23	0.922699	0.077301	1.988857	2.057413	16.67114	8.097825
SNX24	0.922842	0.077158	0.65317	0.653076	1371.479	2100.036
SLC4A3	0.922896	0.077104	1.474106	1.475065	410.1101	278.0253
NPHP1	0.922908	0.077092	0.666034	0.664433	78.91008	118.7681
SDHA	0.922935	0.077065	1.51703	1.517282	1779.267	1172.664
FAM120A(	0.922942	0.077058	0.666452	0.666259	654.6202	982.5361
CREG2	0.922944	0.077056	0.653223	0.650862	54.45907	83.67752
AOC4P	0.922975	0.077025	5.35278	253.29	2.5229	0
MGC5734	0.922975	0.077025	5.35278	253.29	2.5229	0
MTPAP	0.923027	0.076973	0.667949	0.667735	580.4114	869.2295
CNTN1	0.92308	0.07692	1.988002	2.057413	16.67114	8.097825
NKRF	0.923116	0.076884	0.669036	0.668811	559.039	835.8755
RCHY1	0.92312	0.07688	0.666758	0.666566	673.5142	1010.429
MYO16	0.923148	0.076852	1.582917	1.592625	54.45907	34.19082
LIPT1	0.923156	0.076844	0.675315	0.674322	125.5893	186.25
SULT1A1	0.923159	0.076841	0.678474	0.677882	213.6796	315.2213
RNF24	0.923172	0.076828	1.485683	1.486231	750.2015	504.7644
SLC26A4	0.923212	0.076788	1.741617	1.76767	28.65214	16.20465
THNSL2	0.923224	0.076776	0.638588	0.63532	40.01074	62.98308
WDR34	0.923294	0.076706	0.670031	0.669793	527.9196	788.1883
ARVCF	0.923379	0.076621	0.677405	0.676927	260.0698	384.1968
PRR5	0.923402	0.076598	0.678062	0.677495	218.8365	323.0132
MSI1	0.923472	0.076528	0.674903	0.673782	113.3638	168.2548
EZH2	0.923578	0.076422	0.6574	0.657287	1132.526	1723.037
DIP2C	0.923597	0.076403	1.471967	1.472909	420.1128	285.2234
MPRIP	0.923598	0.076402	1.557363	1.557471	4579.007	2940.023
PARP16	0.92361	0.07639	1.47646	1.478632	182.2712	123.2669
BTNL9	0.923612	0.076388	0.509879	0.494373	8.891277	17.99517
MYOM1	0.923682	0.076318	1.527531	1.533288	80.02149	52.18598
LINC0100(	0.923767	0.076233	0.677335	0.676893	281.2089	415.4454
MSTO2P	0.923836	0.076164	0.665876	0.664196	75.24243	113.2886
CALM2	0.923942	0.076058	1.613858	1.613895	15399.69	9541.937
TRMT13	0.923963	0.076037	0.678597	0.678044	224.5047	331.1111

DHPS	0.924054	0.075946	1.520948	1.521168	2051.662	1348.738
EML1	0.924055	0.075945	1.471721	1.472617	441.2296	299.6195
LOC72898	0.924095	0.075905	2.365098	2.562804	10.06937	3.922946
MLXIP	0.924113	0.075887	1.530953	1.531132	2596.253	1695.64
AMPD2	0.92412	0.07588	1.501464	1.501803	1264.784	842.1738
ZMIZ1-AS	0.924138	0.075862	0.510162	0.494373	8.891277	17.99517
COG4	0.924144	0.075856	0.657162	0.657053	1175.871	1789.619
SGK2	0.924151	0.075849	0.654	0.651593	53.34766	81.87801
GRIP1	0.924184	0.075816	0.677244	0.676319	134.4806	198.8466
ALDOC	0.924215	0.075785	1.482154	1.48271	745.7558	502.9649
U2SURP	0.924336	0.075664	0.629988	0.629959	4651.249	7383.417
CDC42P3	0.924449	0.075551	0.174882	0.003691	0	2.699275
LINC0046	0.924449	0.075551	0.174882	0.003691	0	2.699275
LINC0129	0.924449	0.075551	0.174882	0.003691	0	2.699275
SLC9C2	0.924449	0.075551	0.174882	0.003691	0	2.699275
TSPEAR	0.924449	0.075551	0.174882	0.003691	0	2.699275
DHRS13	0.924593	0.075407	0.679441	0.678831	203.388	299.6195
CROT	0.924661	0.075339	0.679437	0.678862	215.6135	317.6147
FLJ42969	0.924667	0.075333	1.706566	1.728919	31.11947	17.99517
G0S2	0.924667	0.075333	1.706566	1.728919	31.11947	17.99517
LIPC	0.924667	0.075333	1.706566	1.728919	31.11947	17.99517
SORBS2	0.924698	0.075302	0.66754	0.665896	76.68726	115.1691
NAPSA	0.924812	0.075188	1.706157	1.728919	31.11947	17.99517
VPS37C	0.924877	0.075123	1.47922	1.479821	672.4028	454.3779
PQLC3	0.92488	0.07512	0.670538	0.669109	86.68995	129.5652
EFNA5	0.924976	0.075024	1.473658	1.474393	541.2565	367.1014
SMYD3	0.924976	0.075024	1.489845	1.490272	993.6002	666.7209
UPF1	0.925215	0.074785	1.563704	1.563794	5718.202	3656.618
LOC15147	0.925216	0.074784	3.517097	5.093071	4.623464	0.899758
FOXK1	0.925372	0.074628	1.495872	1.496236	1159.356	774.8449
TGIF2LX	0.925474	0.074526	2.603723	2.940895	7.957693	2.699275
LOC10013	0.92549	0.07451	0.498322	0.480688	7.779867	16.19565
ZNF233	0.92549	0.07451	0.498322	0.480688	7.779867	16.19565
MRAS	0.925557	0.074443	0.651088	0.651013	1754.916	2695.676
MRPL28	0.925579	0.074421	0.66299	0.66285	895.7961	1351.437
LOC10028	0.925586	0.074414	0.175805	0.003667	0	2.71727
ASF1A	0.925639	0.074361	0.674666	0.674384	442.341	655.9238
LOC10192	0.92567	0.07433	1.792627	1.827136	23.89531	13.07349
FAM46C	0.925729	0.074271	1.565808	1.57476	56.68189	35.99033
DUBR	0.925803	0.074197	0.661705	0.659626	61.12753	92.67511
SLC2A8	0.925829	0.074171	1.479444	1.480001	725.7505	490.3683
PLA1A	0.92585	0.07415	0.676774	0.675644	110.0295	162.8563
TMEM213	0.925886	0.074114	0.175618	0.003595	0	2.771256
DDX56	0.925962	0.074038	0.652926	0.652846	1627.104	2492.331
VIPR2	0.925975	0.074025	2.592882	2.925565	7.968807	2.71727
UQCRHL	0.925977	0.074023	0.176286	0.003679	0	2.708272
FUNDC2	0.92598	0.07402	1.489462	1.489877	1001.38	672.1195
ADCY3	0.926047	0.073953	1.502539	1.50284	1429.273	951.0445
TUBB4B	0.92605	0.07395	1.596091	1.596138	11687.56	7322.395
LOC10192	0.926074	0.073926	0.498643	0.480688	7.779867	16.19565
LRP11	0.926189	0.073811	1.495722	1.496073	1204.768	805.2837
CDHR1	0.926256	0.073744	1.463397	1.464871	262.2927	179.0519
MROH7-T	0.926273	0.073727	2.301512	2.477526	10.42502	4.201871
LOC72832	0.92628	0.07372	0.680974	0.680211	162.1102	238.328
KCNT2	0.92631	0.07369	1.513207	1.518632	83.35572	54.88526
MRPS6	0.926316	0.073684	0.64823	0.648166	2028.923	3130.259

NOS1AP	0.92632	0.07368	1.526323	1.532492	74.46444	48.58695
A2ML1	0.926367	0.073633	0.17677	0.003691	0	2.699275
ABHD12B	0.926367	0.073633	0.17677	0.003691	0	2.699275
C10orf107	0.926367	0.073633	0.17677	0.003691	0	2.699275
C5orf47	0.926367	0.073633	0.17677	0.003691	0	2.699275
CALR3	0.926367	0.073633	0.17677	0.003691	0	2.699275
CDO1	0.926367	0.073633	0.17677	0.003691	0	2.699275
CHST4	0.926367	0.073633	0.17677	0.003691	0	2.699275
FRMD1	0.926367	0.073633	0.17677	0.003691	0	2.699275
HAAO	0.926367	0.073633	0.17677	0.003691	0	2.699275
KL	0.926367	0.073633	0.17677	0.003691	0	2.699275
LINC0020	0.926367	0.073633	0.17677	0.003691	0	2.699275
LINC0026	0.926367	0.073633	0.17677	0.003691	0	2.699275
LINC0031	0.926367	0.073633	0.17677	0.003691	0	2.699275
LINC0051	0.926367	0.073633	0.17677	0.003691	0	2.699275
LINC0122	0.926367	0.073633	0.17677	0.003691	0	2.699275
LINC0142	0.926367	0.073633	0.17677	0.003691	0	2.699275
LINC0152	0.926367	0.073633	0.17677	0.003691	0	2.699275
LOC10050	0.926367	0.073633	0.17677	0.003691	0	2.699275
LOC10192	0.926367	0.073633	0.17677	0.003691	0	2.699275
LOC28457	0.926367	0.073633	0.17677	0.003691	0	2.699275
MORC2-A	0.926367	0.073633	0.17677	0.003691	0	2.699275
MYBPHL	0.926367	0.073633	0.17677	0.003691	0	2.699275
NAALAD2	0.926367	0.073633	0.17677	0.003691	0	2.699275
NMUR1	0.926367	0.073633	0.17677	0.003691	0	2.699275
OR7E12P	0.926367	0.073633	0.17677	0.003691	0	2.699275
PARD6G-/	0.926367	0.073633	0.17677	0.003691	0	2.699275
PART1	0.926367	0.073633	0.17677	0.003691	0	2.699275
PDCD1	0.926367	0.073633	0.17677	0.003691	0	2.699275
PERM1	0.926367	0.073633	0.17677	0.003691	0	2.699275
PGAM1P5	0.926367	0.073633	0.17677	0.003691	0	2.699275
PGR	0.926367	0.073633	0.17677	0.003691	0	2.699275
PRLR	0.926367	0.073633	0.17677	0.003691	0	2.699275
PRND	0.926367	0.073633	0.17677	0.003691	0	2.699275
RAB40AL	0.926367	0.073633	0.17677	0.003691	0	2.699275
RIMS2	0.926367	0.073633	0.17677	0.003691	0	2.699275
SNORA6	0.926367	0.073633	0.17677	0.003691	0	2.699275
SPATA45	0.926367	0.073633	0.17677	0.003691	0	2.699275
SPINK5	0.926367	0.073633	0.17677	0.003691	0	2.699275
TTLL9	0.926367	0.073633	0.17677	0.003691	0	2.699275
TXLNB	0.926367	0.073633	0.17677	0.003691	0	2.699275
VMO1	0.926367	0.073633	0.17677	0.003691	0	2.699275
KCTD16	0.92646	0.07354	1.77825	1.811072	24.45101	13.49637
DENND4C	0.926487	0.073513	1.502099	1.502397	1443.721	960.9419
FAT1	0.926565	0.073435	1.528536	1.528705	2715.174	1776.123
ZC3HAV1	0.92658	0.07342	1.525114	1.525295	2513.619	1647.952
SPINT1	0.926641	0.073359	1.53576	1.535906	3201.971	2084.74
PLA2G16	0.926655	0.073345	1.478652	1.479191	747.9786	505.6642
PSG9	0.926662	0.073338	1.684789	1.705422	32.23088	18.89492
ZNF26	0.926674	0.073326	0.681115	0.680209	138.9262	204.2451
STON1-G	0.926697	0.073303	1.809263	1.847613	22.61718	12.23671
CELF4	0.92671	0.07329	1.918	1.975286	17.78255	8.997583
C1R	0.926733	0.073267	1.777656	1.811072	24.45101	13.49637
GEN1	0.926748	0.073252	0.675565	0.675287	449.0095	664.9214
HAX1	0.926785	0.073215	0.638808	0.638768	3373.128	5280.681
ALG10B	0.92682	0.07318	0.676267	0.675976	427.8149	632.89

MDN1	0.926822	0.073178	0.661408	0.661288	1060.285	1603.369
GTF2IRD2	0.926824	0.073176	0.665509	0.663622	66.09553	99.60324
PCDHGA1	0.926838	0.073162	3.010911	3.737899	5.879357	1.565579
SH3BGRL	0.926851	0.073149	1.488164	1.488565	1051.393	706.3103
TMEM192	0.92691	0.07309	0.659205	0.659099	1231.731	1868.816
SIX2	0.926915	0.073085	1.536703	1.543913	66.68457	43.1884
CPT1B	0.926926	0.073074	1.470201	1.472632	162.2658	110.1844
RNF157	0.926996	0.073004	0.674329	0.674079	499.7898	741.4458
TMEM167	0.927089	0.072911	1.506992	1.507249	1730.465	1148.092
HNRNPDL	0.927133	0.072867	0.647859	0.647798	2135.018	3295.815
GPR35	0.927202	0.072798	0.483987	0.463584	6.668457	14.39613
PRR13	0.927256	0.072744	0.640196	0.640154	3125.284	4882.089
PPAT	0.927301	0.072699	1.509687	1.509929	1808.263	1197.578
GPR19	0.927465	0.072535	0.559501	0.549177	13.33691	24.29347
UBXN8	0.927482	0.072518	0.677672	0.676458	102.2497	151.1594
POLL	0.927547	0.072453	0.68149	0.681039	274.5182	403.0917
BTK	0.927595	0.072405	0.360317	0.309767	2.222819	7.198066
CA3-AS1	0.927595	0.072405	0.360317	0.309767	2.222819	7.198066
CASP5	0.927595	0.072405	0.360317	0.309767	2.222819	7.198066
CLUL1	0.927595	0.072405	0.360317	0.309767	2.222819	7.198066
FCMR	0.927595	0.072405	0.360317	0.309767	2.222819	7.198066
LINC0143	0.927595	0.072405	0.360317	0.309767	2.222819	7.198066
TTC39C-A	0.927595	0.072405	0.360317	0.309767	2.222819	7.198066
ASAH2B	0.927607	0.072393	0.616783	0.611483	25.65133	41.95573
NME1	0.927643	0.072357	0.651792	0.65172	1794.526	2753.53
HSPB1	0.927662	0.072338	1.586519	1.586572	9877.097	6225.428
MTFMT	0.92772	0.07228	0.682435	0.681923	241.2203	353.74
SHD	0.92777	0.07223	0.559692	0.549177	13.33691	24.29347
UGT1A10	0.927782	0.072218	5.19818	248.8443	2.478443	0
SCAND1	0.927828	0.072172	0.683246	0.68272	238.9531	350.006
POPDC2	0.927854	0.072146	0.484357	0.463584	6.668457	14.39613
USH1G	0.927854	0.072146	0.484357	0.463584	6.668457	14.39613
BAIAP2L2	0.927871	0.072129	0.682789	0.682014	158.9316	233.0374
LYNX1	0.928055	0.071945	1.550304	1.558596	58.90471	37.78985
SLC25A34	0.928168	0.071832	0.620511	0.615525	26.67383	43.34136
PDE4D	0.928213	0.071787	1.495281	1.499844	94.46981	62.98308
SLC27A3	0.928279	0.071721	0.682976	0.682474	245.6215	359.9033
KLRAP1	0.928287	0.071713	2.141983	2.262253	12.22551	5.39855
TMEM35	0.928287	0.071713	2.141983	2.262253	12.22551	5.39855
ETFB	0.928334	0.071666	0.647505	0.647448	2279.501	3520.754
CCDC113	0.928357	0.071643	0.683312	0.682526	156.4531	229.2314
NTHL1	0.928411	0.071589	1.457029	1.458597	246.7329	169.1546
ZNF273	0.928475	0.071525	1.458018	1.459795	218.3809	149.5938
MRPL24	0.928481	0.071519	0.661732	0.66162	1161.423	1755.428
SRGAP2D	0.928576	0.071424	0.684653	0.68399	189.0619	276.4147
DIDO1	0.928629	0.071371	0.662628	0.66251	1082.513	1633.961
HIST1H4K	0.928637	0.071363	0.465737	0.441597	5.557048	12.59662
HIST1H2B	0.92864	0.07136	0.440648	0.411603	4.445638	10.81509
FAM168A	0.928661	0.071339	1.515622	1.515823	2209.482	1457.608
BMP4	0.928683	0.071317	1.467721	1.468387	587.9357	400.3924
LINC0111	0.928816	0.071184	0.3612	0.309767	2.222819	7.198066
ZRANB2-A	0.928816	0.071184	0.3612	0.309767	2.222819	7.198066
MED31	0.928824	0.071176	1.478894	1.482218	120.0322	80.97825
GADD45G	0.928831	0.071169	1.745625	1.775107	25.56242	14.39613
HIST1H2A	0.928905	0.071095	0.408199	0.371268	3.334229	8.997583
RFNG	0.928919	0.071081	0.664735	0.664606	982.4861	1478.303

CSF1R	0.928926	0.071074	1.45674	1.458151	268.9611	184.4505
ANKRD61	0.928953	0.071047	0.179461	0.003691	0	2.699275
DOC2GP	0.928953	0.071047	0.179461	0.003691	0	2.699275
ELF5	0.928953	0.071047	0.179461	0.003691	0	2.699275
FAM90A2I	0.928953	0.071047	0.179461	0.003691	0	2.699275
OGFR-AS	0.928953	0.071047	0.179461	0.003691	0	2.699275
OR10S1	0.928953	0.071047	0.179461	0.003691	0	2.699275
POMC	0.928953	0.071047	0.179461	0.003691	0	2.699275
RBPJL	0.928953	0.071047	0.179461	0.003691	0	2.699275
RLBP1	0.928953	0.071047	0.179461	0.003691	0	2.699275
SLC13A4	0.928953	0.071047	0.179461	0.003691	0	2.699275
SLC9A3	0.928953	0.071047	0.179461	0.003691	0	2.699275
SPG20-AS	0.928953	0.071047	0.179461	0.003691	0	2.699275
TFEC	0.928953	0.071047	0.179461	0.003691	0	2.699275
TRIM49	0.928953	0.071047	0.179461	0.003691	0	2.699275
TSPAN8	0.928953	0.071047	0.179461	0.003691	0	2.699275
ZNF300P1	0.928953	0.071047	0.179461	0.003691	0	2.699275
ILF3-AS1	0.928964	0.071036	0.669241	0.667356	66.69569	99.94515
DIRC3	0.92909	0.07091	2.553043	2.875259	7.779867	2.699275
GDAP1L1	0.92909	0.07091	2.553043	2.875259	7.779867	2.699275
NFKB2	0.929104	0.070896	1.511714	1.511926	2119.458	1401.823
GEMIN6	0.929131	0.070869	0.683748	0.683264	254.5128	372.4999
FBLN5	0.929203	0.070797	1.466683	1.467344	590.1585	402.192
HEMK1	0.929225	0.070775	0.684262	0.68374	235.6188	344.6074
ADAM1A	0.929303	0.070697	0.6087	0.60266	22.22819	36.89009
MEFV	0.929305	0.070695	2.851491	3.426634	6.190551	1.799517
CHAC2	0.929359	0.070641	0.682342	0.681306	118.9208	174.5531
SGSM3	0.929359	0.070641	1.506658	1.50689	1904.956	1264.16
ZHX2	0.92937	0.07063	0.677498	0.677236	473.4605	699.1122
DTNBP1	0.929374	0.070626	1.463804	1.464531	532.3652	363.5024
AMIGO2	0.929387	0.070613	1.515016	1.515214	2249.493	1484.601
SYNGR1	0.929402	0.070598	0.682023	0.681663	348.9826	511.9625
IL18R1	0.929429	0.070571	0.673648	0.671995	75.57585	112.4698
USP2	0.929465	0.070535	1.458892	1.459803	419.0014	287.0229
FDXR	0.929486	0.070514	0.685105	0.684451	187.8282	274.4263
MPZL3	0.929528	0.070472	1.488699	1.489041	1232.587	827.7686
KRT18P55	0.929587	0.070413	0.441714	0.412288	4.445638	10.7971
CCDC87	0.929612	0.070388	0.408662	0.371268	3.334229	8.997583
GJB1	0.929612	0.070388	0.408662	0.371268	3.334229	8.997583
LINC0110	0.929612	0.070388	0.408662	0.371268	3.334229	8.997583
LOC10254	0.929612	0.070388	0.408662	0.371268	3.334229	8.997583
TBC1D23	0.929646	0.070354	1.50114	1.501398	1692.677	1127.397
PDE3A	0.929675	0.070325	0.554969	0.543705	12.22551	22.49396
ANKRD30	0.929682	0.070318	3.426945	4.946473	4.490095	0.899758
DUS3L	0.929729	0.070271	0.678311	0.678048	480.1289	708.1098
METTL23	0.929736	0.070264	0.667816	0.667673	896.9075	1343.339
ASIC1	0.929745	0.070255	1.455698	1.456799	343.4256	235.7367
CDH26	0.929836	0.070164	2.549489	2.875259	7.779867	2.699275
LINC0093	0.929836	0.070164	2.549489	2.875259	7.779867	2.699275
LINC0135	0.929836	0.070164	2.549489	2.875259	7.779867	2.699275
LINC0153	0.929836	0.070164	2.549489	2.875259	7.779867	2.699275
LINC0160	0.929836	0.070164	2.549489	2.875259	7.779867	2.699275
NMUR2	0.929836	0.070164	2.549489	2.875259	7.779867	2.699275
PSORS1C	0.929836	0.070164	2.549489	2.875259	7.779867	2.699275
SLC30A3	0.929836	0.070164	2.549489	2.875259	7.779867	2.699275
NOP56	0.929853	0.070147	0.645448	0.645399	2749.627	4260.356

OSGIN1	0.929892	0.070108	0.683525	0.683108	295.6349	432.7837
EFR3A	0.929897	0.070103	0.635691	0.635661	4306.712	6775.18
MSANTD2	0.929936	0.070064	1.456473	1.458337	203.388	139.4625
CLCA3P	0.929989	0.070011	3.409111	4.897607	4.445638	0.899758
FAM19A4	0.929989	0.070011	3.409111	4.897607	4.445638	0.899758
GIMAP8	0.929989	0.070011	3.409111	4.897607	4.445638	0.899758
PI16	0.929989	0.070011	3.409111	4.897607	4.445638	0.899758
PCYOX1L	0.929994	0.070006	0.680451	0.68014	398.996	586.6424
TGFBR3	0.930009	0.069991	0.6808	0.680492	408.9987	601.0385
BLOC1S2	0.930107	0.069893	0.677379	0.677127	487.3976	719.8066
PTAFR	0.930135	0.069865	1.473343	1.473842	796.9918	540.7547
FLJ42102	0.930154	0.069846	0.524095	0.508864	9.046874	17.78822
SGMS1-A	0.930182	0.069818	0.409817	0.372384	3.334229	8.97059
STAT3	0.930185	0.069815	1.545117	1.545222	4642.791	3004.608
ANKMY1	0.930226	0.069774	0.665393	0.663164	56.68189	85.47704
CDKL3	0.930328	0.069672	0.674554	0.672879	74.46444	110.6703
LINC0117	0.930361	0.069639	5.084327	237.7302	2.367302	0
SLC15A1	0.93042	0.06958	0.676231	0.674654	78.91008	116.9686
TAS1R3	0.930425	0.069575	0.442241	0.412288	4.445638	10.7971
RAB28	0.930466	0.069534	0.683065	0.682694	332.3115	486.7692
MRPS18B	0.930595	0.069405	0.664661	0.664543	1070.287	1610.567
GPR37	0.930596	0.069404	0.409325	0.371268	3.334229	8.997583
LOC10050	0.930596	0.069404	0.409325	0.371268	3.334229	8.997583
OLFML1	0.930596	0.069404	0.409325	0.371268	3.334229	8.997583
PCDHB17	0.930628	0.069372	3.39876	4.885934	4.478981	0.908756
SMARCC1	0.930652	0.069348	0.647391	0.64734	2517.343	3888.755
LOC25739	0.93068	0.06932	0.648648	0.645329	38.89934	60.28381
FAM24B	0.930705	0.069295	1.49661	1.5016	85.12286	56.68477
CRLF2	0.930754	0.069246	3.404406	4.909823	4.456752	0.899758
MANEAL	0.930792	0.069208	1.503093	1.508549	79.23239	52.51889
PKD1L2	0.930822	0.069178	0.676669	0.67644	536.8108	793.5868
CDCA8	0.930826	0.069174	1.501334	1.501577	1798.261	1197.578
RASL10A	0.930871	0.069129	2.544424	2.875259	7.779867	2.699275
SLC38A5	0.930871	0.069129	2.544424	2.875259	7.779867	2.699275
STARD4-A	0.930871	0.069129	2.544424	2.875259	7.779867	2.699275
FMR1	0.930885	0.069115	0.656215	0.656138	1703.791	2596.702
PDGFD	0.930901	0.069099	0.673671	0.671824	68.90739	102.5724
MNX1	0.930925	0.069075	1.716698	1.74337	26.67383	15.29589
CCDC97	0.930929	0.069071	0.674062	0.67387	652.3974	968.1399
NPDC1	0.930948	0.069052	1.48974	1.490055	1319.243	885.3622
LOC10013	0.930954	0.069046	0.587322	0.579161	16.67114	28.79227
RD3	0.930954	0.069046	0.587322	0.579161	16.67114	28.79227
SUSD3	0.930981	0.069019	1.450178	1.451516	283.4094	195.2476
FAM181B	0.931034	0.068966	0.633802	0.629346	30.00806	47.68719
TLR7	0.931047	0.068953	2.041139	2.135148	13.45917	6.298308
HSPA1A	0.931049	0.068951	1.478291	1.481997	107.5066	72.53851
SLC25A35	0.931105	0.068895	0.666456	0.664143	55.57048	83.67752
ADRA1D	0.931108	0.068892	3.396894	4.897607	4.445638	0.899758
ARID3C	0.931108	0.068892	3.396894	4.897607	4.445638	0.899758
C10orf95	0.931108	0.068892	3.396894	4.897607	4.445638	0.899758
CACNA2D	0.931108	0.068892	3.396894	4.897607	4.445638	0.899758
CACNG7	0.931108	0.068892	3.396894	4.897607	4.445638	0.899758
CHRDL1	0.931108	0.068892	3.396894	4.897607	4.445638	0.899758
FAM19A3	0.931108	0.068892	3.396894	4.897607	4.445638	0.899758
FRZB	0.931108	0.068892	3.396894	4.897607	4.445638	0.899758
LAG3	0.931108	0.068892	3.396894	4.897607	4.445638	0.899758

LOC10012	0.931108	0.068892	3.396894	4.897607	4.445638	0.899758
LOC10192	0.931108	0.068892	3.396894	4.897607	4.445638	0.899758
LOC10192	0.931108	0.068892	3.396894	4.897607	4.445638	0.899758
MYL2	0.931108	0.068892	3.396894	4.897607	4.445638	0.899758
OR10H1	0.931108	0.068892	3.396894	4.897607	4.445638	0.899758
TYRP1	0.931108	0.068892	3.396894	4.897607	4.445638	0.899758
ZNF474	0.931108	0.068892	3.396894	4.897607	4.445638	0.899758
C7orf57	0.931147	0.068853	0.587473	0.579161	16.67114	28.79227
TDRD3	0.931147	0.068853	0.587473	0.579161	16.67114	28.79227
ZNF571	0.931161	0.068839	0.669716	0.667667	61.27201	91.77535
DMAP1	0.931163	0.068837	0.681879	0.681575	414.5558	608.2366
TPTEP1	0.931201	0.068799	1.459199	1.461651	155.953	106.6933
C14orf93	0.931214	0.068786	0.673637	0.671824	68.90739	102.5724
MRI1	0.931244	0.068756	1.477624	1.478038	970.3495	656.5086
DDX49	0.931246	0.068754	0.656391	0.656314	1668.226	2541.817
AJAP1	0.931246	0.068754	1.465639	1.466219	670.18	457.0772
HID1	0.931404	0.068596	0.670019	0.669871	869.1223	1297.451
CDKN2A	0.931461	0.068539	1.521194	1.521353	2830.76	1860.682
NUTM2B	0.931467	0.068533	0.609909	0.603662	21.46132	35.55845
TOX	0.9315	0.0685	0.688026	0.687443	213.3906	310.4166
ARFGAP3	0.931504	0.068496	1.524413	1.524562	3045.262	1997.463
MRPS7	0.931541	0.068459	0.666784	0.666658	1004.714	1507.095
ZRANB3	0.931581	0.068419	0.688357	0.687666	180.0484	261.8297
ACE	0.931612	0.068388	1.581758	1.593634	44.45638	27.89251
MIR100HC	0.931612	0.068388	1.581758	1.593634	44.45638	27.89251
NANS	0.931653	0.068347	1.518371	1.518538	2680.72	1765.326
AQP3	0.93166	0.06834	0.659482	0.656754	46.6792	71.08091
HNRNPU	0.931751	0.068249	0.623641	0.623625	8362.246	13409.1
MARCH3	0.931778	0.068222	1.459116	1.461832	143.3718	98.07366
ZNF716	0.931809	0.068191	3.381868	4.873174	4.42341	0.899758
PRR7	0.931842	0.068158	0.681152	0.679816	91.13559	134.064
RPS27L	0.931843	0.068157	1.485735	1.486068	1218.105	819.6798
CLN5	0.931893	0.068107	1.449508	1.450718	306.749	211.4432
SNX22	0.931897	0.068103	0.54967	0.537281	11.1141	20.69444
TPRA1	0.931947	0.068053	1.522865	1.523017	2999.694	1969.571
PGM5P2	0.931972	0.068028	1.644979	1.663676	33.29783	20.01062
P2RX4	0.931983	0.068017	0.688113	0.687509	202.2765	294.221
MBD3	0.932007	0.067993	0.677156	0.676945	602.384	889.861
IFNGR2	0.93202	0.06798	1.490779	1.491071	1431.496	960.0421
ATG9A	0.932055	0.067945	1.510188	1.510381	2269.498	1502.596
NUP62CL	0.932084	0.067916	1.523285	1.530484	63.35035	41.38888
RNF19B	0.932091	0.067909	1.471499	1.47196	856.8968	582.1436
GTF3A	0.932125	0.067875	0.665658	0.665542	1095.85	1646.558
FLVCR2	0.932146	0.067854	0.676458	0.674737	72.24162	107.0712
SLC11A2	0.932215	0.067785	1.52045	1.520605	2957.95	1945.241
LOC10192	0.932262	0.067738	0.350914	0.292885	1.856054	6.361291
NOV	0.932326	0.067674	0.687273	0.686839	286.7437	417.4879
NQO2	0.93234	0.06766	1.47588	1.476285	986.9317	668.5204
GGT3P	0.93235	0.06765	5.007626	233.2846	2.322846	0
KIF26A	0.932353	0.067647	0.688681	0.688039	190.051	276.2258
MARK4	0.932366	0.067634	1.500257	1.500488	1886.062	1256.962
CUZD1	0.932366	0.067634	1.815465	1.859257	20.08317	10.7971
STARD7-A	0.932376	0.067624	0.642952	0.638982	33.34229	52.18598
FAM204A	0.932456	0.067544	0.677645	0.677431	579.0444	854.7704
FOXO6	0.932499	0.067501	0.667478	0.665165	54.45907	81.87801
ASB8	0.932519	0.067481	0.682877	0.682576	410.1101	600.8316

C16orf70	0.932546	0.067454	0.685836	0.685453	320.086	466.9746
C1orf226	0.932628	0.067372	1.44961	1.451365	208.945	143.9613
BST1	0.932645	0.067355	3.379594	4.897607	4.445638	0.899758
HSD17B2	0.932645	0.067355	3.379594	4.897607	4.445638	0.899758
LINC0064:	0.932645	0.067355	3.379594	4.897607	4.445638	0.899758
LOC10013	0.932645	0.067355	3.379594	4.897607	4.445638	0.899758
LOC10192	0.932645	0.067355	3.379594	4.897607	4.445638	0.899758
MCF2	0.932645	0.067355	3.379594	4.897607	4.445638	0.899758
SH2D1B	0.932645	0.067355	3.379594	4.897607	4.445638	0.899758
SNORA10	0.932645	0.067355	3.379594	4.897607	4.445638	0.899758
SNORD15	0.932645	0.067355	3.379594	4.897607	4.445638	0.899758
ST8SIA2	0.932645	0.067355	3.379594	4.897607	4.445638	0.899758
TAS2R4	0.932645	0.067355	3.379594	4.897607	4.445638	0.899758
ZNF418	0.932645	0.067355	3.379594	4.897607	4.445638	0.899758
SPARC	0.93267	0.06733	1.691183	1.715157	27.78524	16.19565
DMRT3	0.932802	0.067198	0.685828	0.684691	107.8067	157.4577
MAP7D2	0.932854	0.067146	0.68839	0.687497	136.7034	198.8466
FAM160B1	0.932902	0.067098	1.477593	1.477969	1069.176	723.4057
KLHL7	0.932916	0.067084	1.472271	1.4727	923.5814	627.1315
DDRGK1	0.932967	0.067033	0.66962	0.669485	932.4726	1392.826
DNAJC5	0.932999	0.067001	1.518343	1.5185	2841.874	1871.497
GNRH1	0.933003	0.066997	1.809397	1.852058	20.00537	10.7971
LINC0143	0.933003	0.066997	1.809397	1.852058	20.00537	10.7971
SCFD2	0.933027	0.066973	1.449975	1.450834	441.2296	304.1183
VLDLR-AS	0.933052	0.066948	1.614667	1.630225	36.67652	22.49396
ZNF575	0.93306	0.06694	2.023363	2.115768	13.33691	6.298308
SLC25A45	0.933099	0.066901	1.689526	1.714206	27.78524	16.20465
LOC10192	0.933099	0.066901	0.623133	0.617712	24.45101	39.58937
ZBTB2	0.933103	0.066897	0.679887	0.679657	547.9249	806.1834
EID2B	0.933109	0.066891	0.585345	0.576598	15.55973	26.99275
AGMAT	0.933172	0.066828	1.446951	1.448697	211.1678	145.7608
C9orf78	0.933186	0.066814	1.487297	1.487589	1440.387	968.2659
BBS1	0.933238	0.066762	0.688298	0.68784	266.7383	387.7958
CLDN15	0.933275	0.066725	1.455154	1.45775	145.5947	99.87317
FBXO9	0.933286	0.066714	0.670376	0.67024	941.3639	1404.523
GHET1	0.933286	0.066714	0.607752	0.601043	20.00537	33.29106
DCX	0.933304	0.066696	4.991095	236.6188	2.356188	0
EPM2AIP1	0.933351	0.066649	0.689352	0.688825	231.1732	335.6098
PSG2	0.933357	0.066643	3.316216	4.711229	4.445638	0.935749
LINC0125	0.933358	0.066642	0.417622	0.380387	3.334229	8.781641
RAC3	0.933421	0.066579	0.68662	0.686246	327.8658	477.7717
CLSTN3	0.933423	0.066577	1.474009	1.474407	1000.269	678.4178
HSF2BP	0.933424	0.066576	1.449892	1.452066	171.1571	117.8683
FAM192A	0.933435	0.066565	0.670554	0.670416	910.2444	1357.735
KALRN	0.933445	0.066555	0.688707	0.687672	120.0322	174.5531
SMAD9	0.933487	0.066513	1.444233	1.445918	221.1705	152.9589
ST7L	0.933563	0.066437	0.690517	0.689685	148.9289	215.942
ANK2	0.933603	0.066397	0.690113	0.689527	207.8336	301.419
WWOX	0.933624	0.066376	0.671261	0.669122	57.7933	86.3768
PTGR1	0.933692	0.066308	1.5081	1.508287	2332.849	1546.685
PMAIP1	0.933745	0.066255	1.470166	1.470588	951.3666	646.9262
ENTPD7	0.933809	0.066191	1.457784	1.458393	624.6122	428.285
NIPAL2	0.933835	0.066165	0.683825	0.682425	87.80136	128.6654
ZNF137P	0.933835	0.066165	1.511498	1.518244	65.56205	43.1794
CDK18	0.933858	0.066142	1.47175	1.472165	939.1411	637.9286
LRRC37B	0.93387	0.06613	1.454564	1.45524	557.283	382.9461

LOC64377	0.933898	0.066102	3.296642	4.66683	4.445638	0.944746
ZNF483	0.93391	0.06609	1.779452	1.817671	21.10567	11.60688
HOXB8	0.933918	0.066082	1.485159	1.490034	84.46713	56.68477
ANKRD39	0.933971	0.066029	1.44622	1.448168	188.9396	130.465
SMIM17	0.933971	0.066029	1.939143	2.011248	15.19297	7.548972
SNAP29	0.934059	0.065941	0.660713	0.66063	1578.868	2389.947
MED12L	0.934121	0.065879	0.543391	0.529634	10.00269	18.89492
SCAMP1	0.934124	0.065876	1.479452	1.479788	1203.69	813.4175
TOX2	0.934149	0.065851	1.452161	1.452855	549.0363	377.8985
EOGT	0.93426	0.06574	1.444636	1.446488	197.8309	136.7633
MRPL42P	0.934287	0.065713	4.930925	228.839	2.27839	0
TRIP13	0.934424	0.065576	1.449948	1.450683	515.7274	355.5035
PSMB8-AS	0.93452	0.06548	0.635018	0.630304	27.78524	44.08816
PAQR6	0.934525	0.065475	0.672424	0.670218	56.68189	84.57728
CFAP54	0.93453	0.06547	0.543643	0.529634	10.00269	18.89492
RGPD1	0.934612	0.065388	2.120679	2.248026	11.25858	5.002656
LRPPRC	0.934617	0.065383	0.626165	0.626149	8441.156	13481.08
INGX	0.934625	0.065375	0.563261	0.551813	11.981	21.72017
KATNA1	0.934657	0.065343	0.687059	0.686718	358.9853	522.7596
SLC35F6	0.934695	0.065305	0.658201	0.65813	1832.748	2784.788
TSTD1	0.93482	0.06518	0.671371	0.671237	939.1411	1399.124
PSMC5	0.934833	0.065167	0.659565	0.659489	1680.451	2548.116
PRR36	0.93486	0.06514	1.441926	1.443852	192.2739	133.1642
TOM1L1	0.934868	0.065132	0.679224	0.679026	639.0605	941.1472
MTIF2	0.934868	0.065132	0.672969	0.672824	866.8995	1288.454
CMAS	0.934885	0.065115	1.489794	1.490051	1618.212	1086.008
MICU2	0.934903	0.065097	0.683884	0.683625	483.4632	707.21
FHIT	0.934905	0.065095	0.689582	0.688508	113.3638	164.6558
TRIM59	0.934917	0.065083	1.441425	1.443049	223.3933	154.8034
HOMEZ	0.934947	0.065053	0.68626	0.685947	392.8055	572.6512
GNG10	0.93495	0.06505	1.513751	1.513909	2801.952	1850.803
CFAP65	0.934968	0.065032	4.885342	223.2819	2.222819	0
FGF14	0.934968	0.065032	4.885342	223.2819	2.222819	0
LEFTY1	0.934968	0.065032	4.885342	223.2819	2.222819	0
LOC40054	0.934968	0.065032	4.885342	223.2819	2.222819	0
LUZP4	0.934968	0.065032	4.885342	223.2819	2.222819	0
MS4A7	0.934968	0.065032	4.885342	223.2819	2.222819	0
RHOJ	0.934968	0.065032	4.885342	223.2819	2.222819	0
SMC5-AS1	0.934968	0.065032	4.885342	223.2819	2.222819	0
TGM4	0.934968	0.065032	4.885342	223.2819	2.222819	0
TYR	0.934968	0.065032	4.885342	223.2819	2.222819	0
SGCB	0.934983	0.065017	1.441459	1.44313	217.0694	150.4126
RFX5	0.935024	0.064976	1.501449	1.50165	2136.129	1422.518
C12orf57	0.935032	0.064968	1.460671	1.461179	754.6471	516.4613
KDELC1	0.935039	0.064961	0.692211	0.691581	192.2739	278.0253
WRAP73	0.935094	0.064906	1.45444	1.45505	617.9437	424.6859
PRC1	0.935173	0.064827	1.510755	1.510921	2649.6	1753.629
CHSY1	0.935175	0.064825	1.472752	1.473121	1073.622	728.8042
BIRC2	0.935309	0.064691	1.552772	1.552846	6710.691	4321.539
PEX3	0.935331	0.064669	0.688027	0.687683	354.5397	515.5615
DHX57	0.935336	0.064664	0.671748	0.671615	946.921	1409.921
TECPR1	0.935355	0.064645	1.467938	1.468341	988.3321	673.0912
OVCA2	0.935371	0.064629	0.692523	0.691733	153.3856	221.7454
PTGES2-A	0.935419	0.064581	1.501628	1.508035	67.25139	44.59202
PEX11G	0.935449	0.064551	0.607331	0.600096	18.89396	31.49154
FTSJ2	0.935465	0.064535	0.688	0.68766	360.0967	523.6593

MAPK6	0.935471	0.064529	0.656697	0.656632	1982.755	3019.589
KIZ	0.935495	0.064505	0.693483	0.692849	194.4967	280.7246
RBM23	0.935529	0.064471	0.682498	0.68227	551.2592	807.983
FANCF	0.935543	0.064457	0.683479	0.681876	76.68726	112.4698
SEPT3	0.93555	0.06445	0.675151	0.674997	830.223	1229.97
CIITA	0.93558	0.06442	1.510076	1.517157	62.23894	41.01998
UBL3	0.935595	0.064405	1.4595	1.460005	757.9813	519.1605
MPHOSPH	0.935778	0.064222	0.693317	0.692624	174.4913	251.9323
TEFM	0.935845	0.064155	0.692264	0.691235	120.0322	173.6534
UBALD2	0.93585	0.06415	1.469439	1.469823	1023.608	696.4129
OR7D2	0.935854	0.064146	0.338583	0.271385	1.511517	5.596497
METRN	0.935888	0.064112	1.449237	1.449914	547.9249	377.8985
TMEM178	0.935907	0.064093	1.542871	1.552687	48.90202	31.49154
IVD	0.935909	0.064091	0.674491	0.674346	876.4687	1299.737
SLC22A18	0.935924	0.064076	0.69174	0.691308	286.7437	414.7886
GLTSCR1	0.935925	0.064075	0.692441	0.691509	130.0349	188.0495
MVD	0.935993	0.064007	1.469104	1.469492	996.9344	678.4178
CALD1	0.936023	0.063977	1.617824	1.617846	26584.92	16432.29
ZNF385C	0.936075	0.063925	0.658381	0.655105	38.89934	59.38405
SMARCA1	0.9361	0.0639	1.511382	1.511538	2881.885	1906.588
SH3PXD2	0.936113	0.063887	1.505836	1.506009	2552.908	1695.145
PROSER2	0.936138	0.063862	1.464143	1.464572	901.3532	615.4347
SYT1	0.936163	0.063837	1.481114	1.486373	78.91008	53.08574
LOC10192	0.936173	0.063827	4.854224	224.3933	2.233933	0
RNF144A-	0.936173	0.063827	4.854224	224.3933	2.233933	0
TNK2	0.936199	0.063801	1.455904	1.456425	739.0874	507.4637
CLDND1	0.936267	0.063733	1.502216	1.502403	2306.175	1534.988
LOC10050	0.93632	0.06368	1.644342	1.664696	30.00806	18.02216
SLC44A2	0.936325	0.063675	1.558507	1.558571	7687.62	4932.475
MMP28	0.936335	0.063665	0.535835	0.520378	8.891277	17.09541
PLK1	0.936336	0.063664	1.506638	1.506809	2552.908	1694.245
RTBDN	0.936336	0.063664	0.604245	0.596685	17.78255	29.80899
PIP4K2B	0.936338	0.063662	1.479043	1.479343	1347.028	910.5554
CUL4A	0.936368	0.063632	1.514077	1.514225	2949.681	1947.977
TRPM6	0.936373	0.063627	1.935047	2.008937	14.47055	7.198066
SRSF12	0.936403	0.063597	0.689449	0.688105	92.247	134.064
FAM58A	0.93647	0.06353	1.458766	1.45925	789.1008	540.7547
FAM188A	0.936484	0.063516	0.68079	0.680592	624.6122	917.7535
RBM28	0.936512	0.063488	0.683062	0.682842	570.1531	834.9757
REEP4	0.936516	0.063484	1.463612	1.464037	910.2444	621.733
KIF3B	0.936523	0.063477	0.660255	0.660182	1758.25	2663.285
MRPS16	0.936589	0.063411	0.655953	0.655893	2159.469	3292.414
NFIL3	0.936595	0.063405	0.667996	0.667892	1218.105	1823.81
ADAM18	0.936637	0.063363	4.835048	223.2819	2.222819	0
ADCY10	0.936637	0.063363	4.835048	223.2819	2.222819	0
AGMO	0.936637	0.063363	4.835048	223.2819	2.222819	0
ANKRD26	0.936637	0.063363	4.835048	223.2819	2.222819	0
AQP7P3	0.936637	0.063363	4.835048	223.2819	2.222819	0
ASCL5	0.936637	0.063363	4.835048	223.2819	2.222819	0
B3GAT1	0.936637	0.063363	4.835048	223.2819	2.222819	0
BEND5	0.936637	0.063363	4.835048	223.2819	2.222819	0
C12orf74	0.936637	0.063363	4.835048	223.2819	2.222819	0
C1orf140	0.936637	0.063363	4.835048	223.2819	2.222819	0
C20orf141	0.936637	0.063363	4.835048	223.2819	2.222819	0
C2CD4C	0.936637	0.063363	4.835048	223.2819	2.222819	0
C4BPA	0.936637	0.063363	4.835048	223.2819	2.222819	0

CCDC166	0.936637	0.063363	4.835048	223.2819	2.222819	0
CERKL	0.936637	0.063363	4.835048	223.2819	2.222819	0
CHI3L2	0.936637	0.063363	4.835048	223.2819	2.222819	0
CIDEA	0.936637	0.063363	4.835048	223.2819	2.222819	0
CLLU1OS	0.936637	0.063363	4.835048	223.2819	2.222819	0
COL20A1	0.936637	0.063363	4.835048	223.2819	2.222819	0
CREB3L3	0.936637	0.063363	4.835048	223.2819	2.222819	0
CRNN	0.936637	0.063363	4.835048	223.2819	2.222819	0
CYP2B7P	0.936637	0.063363	4.835048	223.2819	2.222819	0
CYP4F12	0.936637	0.063363	4.835048	223.2819	2.222819	0
DBH-AS1	0.936637	0.063363	4.835048	223.2819	2.222819	0
DIO3	0.936637	0.063363	4.835048	223.2819	2.222819	0
DKFZp451	0.936637	0.063363	4.835048	223.2819	2.222819	0
EFS	0.936637	0.063363	4.835048	223.2819	2.222819	0
FAM151A	0.936637	0.063363	4.835048	223.2819	2.222819	0
FCN3	0.936637	0.063363	4.835048	223.2819	2.222819	0
FGF8	0.936637	0.063363	4.835048	223.2819	2.222819	0
FRRS1L	0.936637	0.063363	4.835048	223.2819	2.222819	0
FZD10	0.936637	0.063363	4.835048	223.2819	2.222819	0
GALR3	0.936637	0.063363	4.835048	223.2819	2.222819	0
GAST	0.936637	0.063363	4.835048	223.2819	2.222819	0
GDF5	0.936637	0.063363	4.835048	223.2819	2.222819	0
GIPC3	0.936637	0.063363	4.835048	223.2819	2.222819	0
GJA4	0.936637	0.063363	4.835048	223.2819	2.222819	0
GNG12-AS	0.936637	0.063363	4.835048	223.2819	2.222819	0
GPR45	0.936637	0.063363	4.835048	223.2819	2.222819	0
GSTA5	0.936637	0.063363	4.835048	223.2819	2.222819	0
GTSF1L	0.936637	0.063363	4.835048	223.2819	2.222819	0
HAR1A	0.936637	0.063363	4.835048	223.2819	2.222819	0
HIST1H2A	0.936637	0.063363	4.835048	223.2819	2.222819	0
HIST1H2B	0.936637	0.063363	4.835048	223.2819	2.222819	0
HOXC-AS	0.936637	0.063363	4.835048	223.2819	2.222819	0
HOXC-AS	0.936637	0.063363	4.835048	223.2819	2.222819	0
HRAT17	0.936637	0.063363	4.835048	223.2819	2.222819	0
IRX1	0.936637	0.063363	4.835048	223.2819	2.222819	0
ITIH3	0.936637	0.063363	4.835048	223.2819	2.222819	0
IZUMO2	0.936637	0.063363	4.835048	223.2819	2.222819	0
KCNJ6	0.936637	0.063363	4.835048	223.2819	2.222819	0
KLF17	0.936637	0.063363	4.835048	223.2819	2.222819	0
KLRC1	0.936637	0.063363	4.835048	223.2819	2.222819	0
KRT79	0.936637	0.063363	4.835048	223.2819	2.222819	0
LHB	0.936637	0.063363	4.835048	223.2819	2.222819	0
LINC0030	0.936637	0.063363	4.835048	223.2819	2.222819	0
LINC0057	0.936637	0.063363	4.835048	223.2819	2.222819	0
LINC0090	0.936637	0.063363	4.835048	223.2819	2.222819	0
LINC0122	0.936637	0.063363	4.835048	223.2819	2.222819	0
LINC0139	0.936637	0.063363	4.835048	223.2819	2.222819	0
LOC10013	0.936637	0.063363	4.835048	223.2819	2.222819	0
LOC10050	0.936637	0.063363	4.835048	223.2819	2.222819	0
LOC10192	0.936637	0.063363	4.835048	223.2819	2.222819	0
LOC10192	0.936637	0.063363	4.835048	223.2819	2.222819	0
LOC10192	0.936637	0.063363	4.835048	223.2819	2.222819	0
LOC10192	0.936637	0.063363	4.835048	223.2819	2.222819	0
LOC10192	0.936637	0.063363	4.835048	223.2819	2.222819	0
LOC10272	0.936637	0.063363	4.835048	223.2819	2.222819	0
LOC25518	0.936637	0.063363	4.835048	223.2819	2.222819	0

LOC28482	0.936637	0.063363	4.835048	223.2819	2.222819	0
LOC28562	0.936637	0.063363	4.835048	223.2819	2.222819	0
LOC72873	0.936637	0.063363	4.835048	223.2819	2.222819	0
LOC72960	0.936637	0.063363	4.835048	223.2819	2.222819	0
LRRC38	0.936637	0.063363	4.835048	223.2819	2.222819	0
LSP1	0.936637	0.063363	4.835048	223.2819	2.222819	0
MEF2C	0.936637	0.063363	4.835048	223.2819	2.222819	0
MSC	0.936637	0.063363	4.835048	223.2819	2.222819	0
MUC13	0.936637	0.063363	4.835048	223.2819	2.222819	0
MUSK	0.936637	0.063363	4.835048	223.2819	2.222819	0
MYO1H	0.936637	0.063363	4.835048	223.2819	2.222819	0
NAP1L5	0.936637	0.063363	4.835048	223.2819	2.222819	0
NCAN	0.936637	0.063363	4.835048	223.2819	2.222819	0
OIT3	0.936637	0.063363	4.835048	223.2819	2.222819	0
OSM	0.936637	0.063363	4.835048	223.2819	2.222819	0
OXCT1-AS	0.936637	0.063363	4.835048	223.2819	2.222819	0
P2RX3	0.936637	0.063363	4.835048	223.2819	2.222819	0
PHACTR3	0.936637	0.063363	4.835048	223.2819	2.222819	0
PLA2G5	0.936637	0.063363	4.835048	223.2819	2.222819	0
PLCL1	0.936637	0.063363	4.835048	223.2819	2.222819	0
POU3F3	0.936637	0.063363	4.835048	223.2819	2.222819	0
PTPRO	0.936637	0.063363	4.835048	223.2819	2.222819	0
RUFY4	0.936637	0.063363	4.835048	223.2819	2.222819	0
RXFP4	0.936637	0.063363	4.835048	223.2819	2.222819	0
SCARNA2	0.936637	0.063363	4.835048	223.2819	2.222819	0
SCARNA5	0.936637	0.063363	4.835048	223.2819	2.222819	0
SLC1A6	0.936637	0.063363	4.835048	223.2819	2.222819	0
SLC6A14	0.936637	0.063363	4.835048	223.2819	2.222819	0
SLCO1B7	0.936637	0.063363	4.835048	223.2819	2.222819	0
SOST	0.936637	0.063363	4.835048	223.2819	2.222819	0
SSTR3	0.936637	0.063363	4.835048	223.2819	2.222819	0
TM4SF4	0.936637	0.063363	4.835048	223.2819	2.222819	0
ZNF473	0.936654	0.063346	0.676393	0.676238	801.3263	1184.982
LINC0032	0.936691	0.063309	1.932189	2.005853	14.44832	7.198066
SWSAP1	0.936788	0.063212	0.664292	0.661244	42.24468	63.89184
JUNB	0.936824	0.063176	1.513643	1.513788	3128.618	2066.745
DPH7	0.936827	0.063173	1.442529	1.443292	485.686	336.5096
RNF165	0.936835	0.063165	1.435849	1.437463	221.1705	153.8587
CRACR2E	0.936888	0.063112	1.444209	1.446938	136.7034	94.47462
SCN1B	0.936904	0.063096	1.470572	1.470923	1123.635	763.8948
GPALPP1	0.936923	0.063077	0.686513	0.686244	455.6779	664.0216
PCAT6	0.936923	0.063077	1.434425	1.436113	214.502	149.3599
ARHGAP3	0.936993	0.063007	2.268777	2.466388	8.891277	3.599033
HHIPL2	0.936993	0.063007	2.268777	2.466388	8.891277	3.599033
SOX9-AS1	0.936993	0.063007	2.268777	2.466388	8.891277	3.599033
A1BG	0.937058	0.062942	1.436118	1.438127	181.1598	125.9662
GRN	0.937154	0.062846	1.533227	1.533326	4693.483	3060.978
FBXW4P1	0.937201	0.062799	1.930901	2.005853	14.44832	7.198066
NUFIP1	0.937226	0.062774	0.694549	0.694062	252.29	363.5024
TLDC1	0.937242	0.062758	1.442595	1.443323	508.9811	352.6423
ZNRF2P2	0.937335	0.062665	0.314309	0.233173	1.133638	4.894685
TMEM169	0.937375	0.062625	0.694278	0.693377	132.2577	190.7488
MTAP	0.937514	0.062486	1.495367	1.495568	2100.564	1404.523
STARD5	0.937525	0.062475	0.60672	0.599035	17.78255	29.69202
EFHB	0.937572	0.062428	2.266499	2.466388	8.891277	3.599033
ENTPD3	0.937572	0.062428	2.266499	2.466388	8.891277	3.599033

KRT78	0.937572	0.062428	2.266499	2.466388	8.891277	3.599033
LOC10272	0.937572	0.062428	2.266499	2.466388	8.891277	3.599033
PAPPA2	0.937572	0.062428	2.266499	2.466388	8.891277	3.599033
MOCOS	0.937659	0.062341	0.68236	0.682164	640.1719	938.4479
TRIM65	0.937668	0.062332	0.689938	0.689629	402.3414	583.4213
CECR5	0.937687	0.062313	0.690186	0.689863	376.7678	546.1533
AMBRA1	0.937711	0.062289	0.685645	0.685404	511.2706	745.9446
RPL13P5	0.937716	0.062284	1.526526	1.535532	51.12484	33.29106
GATA2-AS	0.937723	0.062277	0.696114	0.695402	168.9343	242.9347
ZNF529	0.937737	0.062263	1.43301	1.434309	272.3065	189.849
TMEM144	0.937774	0.062226	0.696723	0.696086	192.2739	276.2258
MMAB	0.937786	0.062214	0.687979	0.68771	463.4578	673.919
KIAA1551	0.937809	0.062191	0.677362	0.677209	814.6632	1202.977
PSMB10	0.937823	0.062177	1.442477	1.443191	509.0256	352.7053
AREL1	0.937951	0.062049	1.496815	1.497004	2281.724	1524.191
MIR3945H	0.937968	0.062032	2.160337	2.313018	10.00269	4.31884
CFB	0.937972	0.062028	0.661972	0.661899	1736.022	2622.795
EMX2	0.93804	0.06196	0.68632	0.686082	523.4739	762.995
DFNA5	0.938075	0.061925	1.462949	1.463345	962.4807	657.7233
FBXO33	0.93814	0.06186	1.440341	1.44109	482.3518	334.7101
CCDC174	0.938145	0.061855	0.689838	0.689529	388.9934	564.1485
RBMS3	0.93817	0.06183	1.444504	1.445129	596.8269	412.9891
DLX4	0.938175	0.061825	1.434107	1.436278	166.7114	116.0688
ZFYVE26	0.938216	0.061784	0.683025	0.682826	616.8323	903.3573
TRIM39-R	0.938232	0.061768	0.194371	0.004145	0	2.402355
KIAA1644	0.93838	0.06162	2.263248	2.466388	8.891277	3.599033
LINC0129	0.93838	0.06162	2.263248	2.466388	8.891277	3.599033
LRFN4	0.938408	0.061592	1.432936	1.433986	336.7571	234.8369
DHDH	0.938422	0.061578	1.460395	1.464558	92.247	62.98308
ARMCX1	0.938452	0.061548	1.443747	1.44439	567.9303	393.1944
LOC10013	0.938461	0.061539	4.758347	218.8363	2.178363	0
EFCAB6	0.938524	0.061476	0.526565	0.508946	7.779867	15.29589
PRR22	0.938524	0.061476	0.526565	0.508946	7.779867	15.29589
EPGN	0.938543	0.061457	0.394537	0.345909	2.489557	7.216062
GPSM2	0.938552	0.061448	1.45624	1.456687	848.0055	582.1436
MRC2	0.938569	0.061431	1.454842	1.455304	815.7746	560.5494
ELOVL2	0.938595	0.061405	0.69245	0.691062	88.91277	128.6654
TBC1D8	0.938703	0.061297	1.440907	1.441576	551.2592	382.3973
NOC4L	0.938734	0.061266	0.693552	0.693175	321.1974	463.3755
TRIB2	0.938756	0.061244	1.447328	1.45062	112.2524	77.37921
LINC0017	0.938784	0.061216	0.62399	0.617727	21.11678	34.19082
ZNF444	0.938845	0.061155	0.68428	0.684072	583.49	852.9709
CHMP7	0.938873	0.061127	1.457498	1.457914	931.3612	638.8284
WDR60	0.938875	0.061125	0.693983	0.693599	314.5289	453.4782
PSD4	0.938882	0.061118	0.692697	0.692351	350.094	505.6642
AADACP1	0.938887	0.061113	4.765184	223.2819	2.222819	0
ANKRD20	0.938887	0.061113	4.765184	223.2819	2.222819	0
C1orf61	0.938887	0.061113	4.765184	223.2819	2.222819	0
CCL3L3	0.938887	0.061113	4.765184	223.2819	2.222819	0
CUX2	0.938887	0.061113	4.765184	223.2819	2.222819	0
FAM95C	0.938887	0.061113	4.765184	223.2819	2.222819	0
FAR2P2	0.938887	0.061113	4.765184	223.2819	2.222819	0
IVL	0.938887	0.061113	4.765184	223.2819	2.222819	0
KCTD19	0.938887	0.061113	4.765184	223.2819	2.222819	0
LINC0032	0.938887	0.061113	4.765184	223.2819	2.222819	0
LINC0070	0.938887	0.061113	4.765184	223.2819	2.222819	0

LINC0070	0.938887	0.061113	4.765184	223.2819	2.222819	0
LINC0092	0.938887	0.061113	4.765184	223.2819	2.222819	0
LINC0161	0.938887	0.061113	4.765184	223.2819	2.222819	0
LOC10012	0.938887	0.061113	4.765184	223.2819	2.222819	0
LOC10192	0.938887	0.061113	4.765184	223.2819	2.222819	0
LOC64493	0.938887	0.061113	4.765184	223.2819	2.222819	0
MIR181A1	0.938887	0.061113	4.765184	223.2819	2.222819	0
MPEG1	0.938887	0.061113	4.765184	223.2819	2.222819	0
NCF1B	0.938887	0.061113	4.765184	223.2819	2.222819	0
NGB	0.938887	0.061113	4.765184	223.2819	2.222819	0
NOX5	0.938887	0.061113	4.765184	223.2819	2.222819	0
PLD5	0.938887	0.061113	4.765184	223.2819	2.222819	0
PLXNA4	0.938887	0.061113	4.765184	223.2819	2.222819	0
POU4F3	0.938887	0.061113	4.765184	223.2819	2.222819	0
RNF186	0.938887	0.061113	4.765184	223.2819	2.222819	0
SH3PXD2	0.938887	0.061113	4.765184	223.2819	2.222819	0
SLAMF9	0.938887	0.061113	4.765184	223.2819	2.222819	0
SLC10A6	0.938887	0.061113	4.765184	223.2819	2.222819	0
SLC13A3	0.938887	0.061113	4.765184	223.2819	2.222819	0
SLC17A9	0.938887	0.061113	4.765184	223.2819	2.222819	0
SMTNL2	0.938887	0.061113	4.765184	223.2819	2.222819	0
SMYD1	0.938887	0.061113	4.765184	223.2819	2.222819	0
SNORA53	0.938887	0.061113	4.765184	223.2819	2.222819	0
SPAG5-AS	0.938887	0.061113	4.765184	223.2819	2.222819	0
SPEM1	0.938887	0.061113	4.765184	223.2819	2.222819	0
STMN2	0.938887	0.061113	4.765184	223.2819	2.222819	0
TEK	0.938887	0.061113	4.765184	223.2819	2.222819	0
TFF2	0.938887	0.061113	4.765184	223.2819	2.222819	0
TMEM179	0.938887	0.061113	4.765184	223.2819	2.222819	0
TMPRSS6	0.938887	0.061113	4.765184	223.2819	2.222819	0
TMPRSS7	0.938887	0.061113	4.765184	223.2819	2.222819	0
TRPC5OS	0.938887	0.061113	4.765184	223.2819	2.222819	0
UBQLNL	0.938887	0.061113	4.765184	223.2819	2.222819	0
XKR5	0.938887	0.061113	4.765184	223.2819	2.222819	0
ZNF534	0.938887	0.061113	4.765184	223.2819	2.222819	0
GOLGA6L	0.93891	0.06109	4.739172	217.7249	2.167249	0
LOC10192	0.93891	0.06109	4.739172	217.7249	2.167249	0
ZNF385B	0.938932	0.061068	0.660984	0.657523	36.67652	55.78501
GADD45A	0.938959	0.061041	1.5425	1.542577	6163.878	3995.827
PLAC8	0.938999	0.061001	0.666933	0.663865	41.04436	61.83139
IGFBP1	0.93903	0.06097	1.699292	1.728784	23.3396	13.49637
KCNAB3	0.93903	0.06097	1.699292	1.728784	23.3396	13.49637
PARVG	0.93903	0.06097	1.699292	1.728784	23.3396	13.49637
RGS10	0.939045	0.060955	0.69206	0.691735	373.4336	539.855
LOH12CR	0.939065	0.060935	0.500729	0.478336	6.179437	12.92953
PTS	0.939182	0.060818	1.449454	1.449953	757.9813	522.7596
ASAH2	0.939208	0.060792	0.615433	0.608218	18.80505	30.92469
BOLA3-AS	0.939237	0.060763	1.44249	1.445431	122.2551	84.57728
ZNF234	0.939244	0.060756	1.427745	1.429509	201.0651	140.6502
HNRNPD	0.939269	0.060731	0.660092	0.660029	2014.986	3052.88
SRP54	0.939325	0.060675	1.508171	1.508315	3038.594	2014.559
TCF25	0.939364	0.060636	0.683756	0.683567	662.4001	969.0397
FASTKD3	0.939399	0.060601	0.697355	0.696882	258.9584	371.6002
ZSCAN20	0.939447	0.060553	0.696472	0.6953	104.4725	150.2596
DDN	0.939451	0.060549	0.605882	0.597837	16.67114	27.89251
IL3RA	0.939451	0.060549	0.605882	0.597837	16.67114	27.89251

NARR	0.939459	0.060541	0.195503	0.004099	0	2.429347
LOC10192	0.939545	0.060455	0.196746	0.004208	0	2.366364
STRADB	0.939548	0.060452	0.678211	0.678069	891.3505	1314.547
TPT1-AS1	0.939563	0.060437	0.665341	0.662093	38.84376	58.67324
LOC33980	0.939581	0.060419	0.689194	0.687451	71.14133	103.4902
C2orf69	0.939582	0.060418	0.684537	0.684339	621.278	907.8561
PGM3	0.939596	0.060404	1.504399	1.504551	2837.429	1885.893
SPPL3	0.939667	0.060333	0.678573	0.678429	881.4589	1299.269
SETDB1	0.939669	0.060331	0.672968	0.672857	1123.635	1669.951
ELP3	0.939711	0.060289	0.685187	0.684984	604.6068	882.6629
STARD3	0.939711	0.060289	1.449746	1.450222	794.6578	547.9528
C4orf36	0.939724	0.060276	0.577488	0.566348	12.22551	21.5942
FAM65C	0.939724	0.060276	0.577488	0.566348	12.22551	21.5942
APOM	0.939774	0.060226	0.655289	0.651374	32.23088	49.48671
CRHR1-IT	0.939814	0.060186	1.486048	1.492457	64.46176	43.1884
ISG20	0.939814	0.060186	1.486048	1.492457	64.46176	43.1884
NPIPA2	0.939865	0.060135	0.692663	0.691129	80.37714	116.3028
ATRIP	0.939941	0.060059	0.699408	0.698628	153.3745	219.541
HCG8	0.940021	0.059979	0.577693	0.566348	12.22551	21.5942
ZNF724P	0.940021	0.059979	0.577693	0.566348	12.22551	21.5942
LOC10192	0.940118	0.059882	0.699756	0.69902	162.2658	232.1376
TP53I11	0.940158	0.059842	1.462908	1.463247	1162.534	794.4866
KLHL17	0.940165	0.059835	1.434453	1.437097	134.4806	93.57486
ZNF738	0.940185	0.059815	1.430495	1.432948	145.9281	101.8346
RALGPS2	0.940218	0.059782	1.522072	1.522178	4256.699	2796.449
PRKCDBP	0.940284	0.059716	0.662481	0.658853	35.56511	53.9855
ARHGEF1	0.940332	0.059668	1.428945	1.429834	400.1074	279.8248
PURB	0.940351	0.059649	0.661013	0.66095	2033.29	3076.319
CA5B	0.940367	0.059633	0.699954	0.699321	188.7062	269.8465
COQ7	0.940393	0.059607	0.699558	0.698988	210.0564	300.5193
EXD2	0.940398	0.059602	1.458121	1.458498	1009.16	691.9141
D2HGDH	0.940407	0.059593	0.691328	0.6897	74.46444	107.971
DNLZ	0.940407	0.059593	0.691328	0.6897	74.46444	107.971
CDH3	0.940424	0.059576	1.530506	1.530597	5125.821	3348.9
UNC13D	0.940465	0.059535	0.694027	0.693711	390.1048	562.3489
NPPA-AS1	0.940513	0.059487	0.198565	0.004256	0	2.339372
MIR34AHG	0.940524	0.059476	0.67867	0.67611	49.23544	72.82644
HAUS6	0.94053	0.05947	1.51241	1.512536	3522.057	2328.574
SETD1B	0.940549	0.059451	0.695781	0.695413	327.8658	471.4734
FER1L4	0.940554	0.059446	1.426791	1.427822	336.8127	235.8896
LOC10192	0.940554	0.059446	1.671411	1.697958	24.45101	14.39613
OSCAR	0.940554	0.059446	1.671411	1.697958	24.45101	14.39613
DPH1	0.940604	0.059396	0.685018	0.684828	647.9407	946.1408
BICD2	0.940626	0.059374	1.488355	1.48855	2161.692	1452.21
GABBR1	0.940632	0.059368	1.424433	1.425689	274.5182	192.5483
MC1R	0.940648	0.059352	0.68237	0.680111	54.45907	80.07849
GEMIN8P4	0.940658	0.059342	0.686878	0.684915	62.23894	90.87559
ACVR1C	0.940659	0.059341	0.514924	0.494467	6.668457	13.49637
OAZ3	0.940659	0.059341	0.514924	0.494467	6.668457	13.49637
SOSTDC1	0.940761	0.059239	1.670929	1.697958	24.45101	14.39613
DICER1-A	0.940804	0.059196	1.579941	1.595231	34.4537	21.5942
OPRL1	0.940804	0.059196	1.579941	1.595231	34.4537	21.5942
BLVRA	0.940819	0.059181	1.475127	1.475382	1570.422	1064.414
GPR82	0.940832	0.059168	0.199179	0.004273	0	2.330374
YWHAH	0.940842	0.059158	1.515614	1.51573	3927.721	2591.304
COQ10A	0.940889	0.059111	1.422784	1.424602	192.2739	134.9637

DPY19L1F	0.940893	0.059107	2.624361	3.109166	5.923813	1.89849
DET1	0.940902	0.059098	0.689457	0.687567	65.57317	95.37438
SHB	0.940929	0.059071	1.466915	1.467212	1342.583	915.0542
DDX60	0.940991	0.059009	1.430781	1.431569	445.6752	311.3164
PCDHB9	0.941091	0.058909	1.736251	1.773535	19.93869	11.23798
BLVRB	0.941109	0.058891	0.651775	0.651734	3216.419	4935.174
PANK4	0.941158	0.058842	1.425287	1.42623	373.4336	261.8297
SNRNP70	0.941196	0.058804	1.512481	1.512603	3569.848	2360.066
TMEM186	0.94121	0.05879	0.702133	0.701447	176.7141	251.9323
SLC27A1	0.941217	0.058783	1.452125	1.452529	942.2975	648.7257
USP6	0.941269	0.058731	0.43835	0.400649	3.389799	8.475723
ZKSCAN3	0.941317	0.058683	0.698428	0.69801	286.5214	410.4877
SLC2A4	0.941341	0.058659	0.691709	0.689918	68.90739	99.88217
PHC2	0.941342	0.058658	1.494942	1.495108	2589.584	1732.035
NINJ1	0.941366	0.058634	1.466381	1.466677	1317.02	897.9588
GOLGA8M	0.941376	0.058624	1.780844	1.826737	17.69364	9.681399
DNAH6	0.941555	0.058445	0.605091	0.596473	15.55973	26.09299
HYMAI	0.941574	0.058426	2.092361	2.22954	10.10271	4.525784
NKD2	0.941614	0.058386	1.492468	1.499791	56.68189	37.78985
ZCCHC10	0.941629	0.058371	1.43021	1.430931	495.6887	346.4069
PBX3	0.941638	0.058362	1.421581	1.423348	193.3853	135.8635
NSUN5P1	0.941655	0.058345	0.699901	0.699441	259.9031	371.5912
ABCB8	0.941684	0.058316	0.69064	0.690407	532.3652	771.0929
LOC72908	0.941728	0.058272	0.636795	0.630957	22.77278	36.0983
NGEF	0.941744	0.058256	0.702888	0.702232	184.494	262.7294
FAHD2CP	0.941795	0.058205	0.676608	0.673872	44.76758	66.43815
ANKAR	0.941907	0.058093	1.64669	1.670757	25.56242	15.29589
LVRN	0.941907	0.058093	1.64669	1.670757	25.56242	15.29589
LOC10192	0.941975	0.058025	0.573944	0.56169	11.1141	19.79468
NKPD1	0.941975	0.058025	0.573944	0.56169	11.1141	19.79468
ART5	0.942016	0.057984	2.085389	2.220703	10.00269	4.498792
GOLGA6E	0.942076	0.057924	3.05052	4.188106	4.290041	1.016727
LGR4	0.942085	0.057915	0.67157	0.671477	1364.811	2032.554
KIAA1107	0.942091	0.057909	1.440095	1.443713	100.0269	69.28139
GOLGA8F	0.942095	0.057905	0.31798	0.230541	1.033611	4.516787
CSRNP3	0.942126	0.057874	0.702604	0.70172	134.4806	191.6485
TICAM1	0.942126	0.057874	1.433116	1.43374	577.933	403.0917
TIPIN	0.942133	0.057867	0.702979	0.702199	152.2631	216.8418
LOC72874	0.942179	0.057821	1.421622	1.422661	328.9772	231.2379
PIF1	0.94219	0.05781	1.433012	1.436259	111.141	77.37921
MICA	0.942212	0.057788	1.450945	1.451346	918.0243	632.5301
BZRAP1	0.942235	0.057765	1.445891	1.449982	90.02418	62.08332
MFSD12	0.942315	0.057685	0.678485	0.678361	999.1572	1472.904
IFITM1	0.942353	0.057647	1.44342	1.443891	774.897	536.6698
LOC10192	0.942365	0.057635	0.624436	0.61738	19.06067	30.87971
C2orf68	0.942375	0.057625	0.694541	0.694259	427.8927	616.3344
PLS3	0.942387	0.057613	1.56535	1.565394	11466.41	7324.932
CPSF3	0.942419	0.057581	0.680096	0.679961	909.133	1337.041
ATP13A3	0.942433	0.057567	1.570493	1.570533	12764.54	8127.517
TRMT10C	0.942437	0.057563	0.683496	0.683342	814.6632	1192.18
DPCR1	0.942489	0.057511	2.083786	2.220703	10.00269	4.498792
INCA1	0.942489	0.057511	2.083786	2.220703	10.00269	4.498792
RNF224	0.942489	0.057511	2.083786	2.220703	10.00269	4.498792
TRIM25	0.94253	0.05747	1.520708	1.520805	4729.048	3109.565
NDRG4	0.942612	0.057388	1.426554	1.427292	479.0175	335.6098
TVP23B	0.942653	0.057347	1.465565	1.465844	1393.296	950.5047

LINC0063	0.942691	0.057309	0.499871	0.475537	5.557048	11.69686
LOC10192	0.942691	0.057309	0.499871	0.475537	5.557048	11.69686
MST1	0.942691	0.057309	0.499871	0.475537	5.557048	11.69686
PKHD1L1	0.942691	0.057309	0.499871	0.475537	5.557048	11.69686
PCCB	0.942829	0.057171	0.674071	0.673973	1302.572	1932.681
FAM228B	0.942841	0.057159	0.658262	0.654021	30.00806	45.88767
MVB12B	0.942841	0.057159	0.658262	0.654021	30.00806	45.88767
FOXN3	0.942956	0.057044	1.499343	1.499485	3001.917	2001.962
HCFC1	0.942959	0.057041	1.501929	1.502064	3171.963	2111.733
CASP6	0.942971	0.057029	1.421622	1.422523	378.9907	266.4184
TTK	0.94299	0.05701	1.48733	1.487509	2273.944	1528.689
ZBTB21	0.943006	0.056994	0.686416	0.686243	705.7451	1028.424
MUM1	0.94305	0.05695	0.702222	0.701779	273.4068	389.5953
NBPF7	0.943055	0.056945	0.473283	0.443	4.378954	9.897341
LOC10192	0.94307	0.05693	1.857027	1.922386	14.3483	7.458996
SIPA1	0.943119	0.056881	1.448436	1.448824	972.4834	671.2197
SCARF2	0.943137	0.056863	1.427407	1.430217	122.2551	85.47704
SLC19A2	0.943141	0.056859	0.685368	0.685203	732.4189	1068.913
FAM13A-A	0.94315	0.05685	2.081495	2.220703	10.00269	4.498792
ZNF582-A	0.94315	0.05685	2.081495	2.220703	10.00269	4.498792
FLJ46906	0.943208	0.056792	1.440011	1.443933	92.247	63.88284
LOC72997	0.943231	0.056769	0.665649	0.661798	33.34229	50.38647
ATP5G1	0.943264	0.056736	0.671026	0.670942	1489.289	2219.704
PTER	0.943293	0.056707	0.68796	0.687776	664.6229	966.3404
LINC0160	0.943296	0.056704	0.500309	0.475537	5.557048	11.69686
RUSC1-AS	0.943296	0.056704	0.500309	0.475537	5.557048	11.69686
SLC23A1	0.943296	0.056704	0.500309	0.475537	5.557048	11.69686
USP49	0.943301	0.056699	1.414629	1.415873	273.9402	193.475
PEX11B	0.943409	0.056591	0.688436	0.688252	676.8484	983.4358
ZNF250	0.943421	0.056579	0.690666	0.690461	603.2287	873.6653
KIAA1468	0.943441	0.056559	0.701889	0.701462	278.9638	397.6932
ARHGEF4	0.94347	0.05653	0.705109	0.704264	140.0376	198.8466
MIEN1	0.94347	0.05653	0.6898	0.689604	633.5035	918.6532
ZIC1	0.943474	0.056526	0.701023	0.6997	90.02418	128.6654
PDE4DIP	0.943499	0.056501	1.506924	1.507043	3717.598	2466.813
HOXA7	0.943541	0.056459	1.542532	1.555248	37.78793	24.29347
ARHGAP3	0.943552	0.056448	0.6986	0.698272	365.6538	523.6593
MKRN1	0.943555	0.056445	1.522426	1.522516	5012.457	3292.216
GNAS-AS	0.943559	0.056441	1.542298	1.555248	37.78793	24.29347
NSD1	0.943614	0.056386	0.655384	0.655342	3069.713	4684.142
PPP1R15A	0.943633	0.056367	0.649309	0.649277	4045.531	6230.826
RASAL3	0.943667	0.056333	0.331995	0.248716	1.11141	4.498792
NLGN2	0.943681	0.056319	1.449109	1.449483	992.4888	684.7161
MIOX	0.943682	0.056318	0.604185	0.594908	14.44832	24.29347
ZNRF3	0.943688	0.056312	0.690393	0.690194	623.6008	903.5193
NEXN-AS1	0.943746	0.056254	1.751028	1.793973	17.86035	9.951327
WDR81	0.943748	0.056252	0.702011	0.701597	287.8551	410.2898
ZNF839	0.943748	0.056252	0.706371	0.705677	173.3799	245.697
SLX1A-SU	0.943753	0.056247	1.413698	1.415615	177.2921	125.2374
SLX1B-SU	0.943753	0.056247	1.413698	1.415615	177.2921	125.2374
PPP1R32	0.943758	0.056242	1.752625	1.795896	17.78255	9.897341
LOC10192	0.943766	0.056234	3.096384	4.408945	4.001074	0.899758
SPATA33	0.943775	0.056225	1.442995	1.447459	83.35572	57.58453
RAB3GAP	0.94378	0.05622	1.49904	1.499176	3194.191	2130.628
LOC10192	0.943787	0.056213	0.338294	0.25852	1.211436	4.714734
CCND1	0.943796	0.056204	1.549534	1.549589	8842.375	5706.267

CCDC34	0.943878	0.056122	0.705897	0.705312	205.6108	291.5217
CNTROB	0.943965	0.056035	1.433631	1.434155	689.0739	480.4709
MZT2A	0.944033	0.055967	1.421253	1.422043	431.7826	303.6324
ATP1A2	0.944061	0.055939	0.676916	0.673817	40.01074	59.38405
PRRG4	0.944099	0.055901	1.44063	1.441072	816.886	566.8567
PMS2CL	0.944118	0.055882	0.700815	0.699298	80.04372	114.4673
ZBTB3	0.944121	0.055879	0.68284	0.680139	45.48999	66.88803
CFAP221	0.944244	0.055756	0.569713	0.556101	10.00269	17.99517
TRMT5	0.944273	0.055727	0.691972	0.691761	576.9661	834.058
RABGAP1	0.944289	0.055711	1.43926	1.439712	797.9921	554.2691
WHAMMP	0.944294	0.055706	1.539914	1.552724	37.26556	23.99655
RELB	0.944327	0.055673	1.445522	1.44591	945.8096	654.1243
ZFP1	0.94433	0.05567	0.706505	0.705858	182.2712	258.2306
P2RY8	0.944333	0.055667	2.584373	3.076539	5.557048	1.799517
RNU6-2	0.944345	0.055655	1.719559	1.757328	19.06067	10.84209
SLC6A16	0.944348	0.055652	1.604431	1.624939	27.78524	17.09541
ZNF296	0.944355	0.055645	1.412322	1.414352	166.7114	117.8683
LOC10192	0.944362	0.055638	0.332393	0.248221	1.11141	4.507789
ZNF181	0.944375	0.055625	0.703697	0.703258	270.0725	384.0348
METAP1D	0.944395	0.055605	0.681398	0.678558	43.34497	63.88284
TRIP4	0.944411	0.055589	0.701626	0.701269	340.0913	484.9697
TMEM138	0.944444	0.055556	0.692795	0.692582	580.1558	837.675
WDR11-A	0.944456	0.055544	2.588274	3.085763	5.601504	1.808514
TP53I3	0.944521	0.055479	1.410411	1.411671	266.7383	188.9492
KLHL7-AS	0.944534	0.055466	0.479648	0.449731	4.445638	9.897341
SPDYA	0.944534	0.055466	0.479648	0.449731	4.445638	9.897341
DCP1B	0.944543	0.055457	0.700197	0.698642	76.68726	109.7705
ADAM32	0.944565	0.055435	0.332982	0.248716	1.11141	4.498792
AGAP1-IT	0.944565	0.055435	0.332982	0.248716	1.11141	4.498792
ATOH7	0.944565	0.055435	0.332982	0.248716	1.11141	4.498792
CCDC170	0.944565	0.055435	0.332982	0.248716	1.11141	4.498792
CELF6	0.944565	0.055435	0.332982	0.248716	1.11141	4.498792
FXYD4	0.944565	0.055435	0.332982	0.248716	1.11141	4.498792
GPR63	0.944565	0.055435	0.332982	0.248716	1.11141	4.498792
LOC10050	0.944565	0.055435	0.332982	0.248716	1.11141	4.498792
LOC28437	0.944565	0.055435	0.332982	0.248716	1.11141	4.498792
MADCAM1	0.944565	0.055435	0.332982	0.248716	1.11141	4.498792
OR7E47P	0.944565	0.055435	0.332982	0.248716	1.11141	4.498792
SAMD12-A	0.944565	0.055435	0.332982	0.248716	1.11141	4.498792
SAXO2	0.944565	0.055435	0.332982	0.248716	1.11141	4.498792
SH3GL2	0.944565	0.055435	0.332982	0.248716	1.11141	4.498792
TRIM63	0.944565	0.055435	0.332982	0.248716	1.11141	4.498792
UGT3A2	0.944565	0.055435	0.332982	0.248716	1.11141	4.498792
VWA8-AS	0.944565	0.055435	0.332982	0.248716	1.11141	4.498792
ABHD11-A	0.9446	0.0554	0.569962	0.556101	10.00269	17.99517
PCMTD2	0.944625	0.055375	0.697065	0.696802	464.5692	666.7209
CARF	0.944637	0.055363	0.657719	0.653117	27.66298	42.36062
PORCN	0.944647	0.055353	1.434995	1.435474	756.8699	527.2584
ZBTB26	0.94467	0.05533	0.69799	0.696252	68.90739	98.97341
NCRUPAR	0.9447	0.0553	0.351257	0.276464	1.35592	4.930676
GRASP	0.944711	0.055289	0.685295	0.682674	46.6792	68.38163
IKBKE	0.944719	0.055281	0.70744	0.706631	145.5947	206.0447
ZSCAN5B	0.944724	0.055276	0.446866	0.40839	3.334229	8.178803
DEPDC5	0.944759	0.055241	0.703995	0.703586	295.6349	420.1871
CENPM	0.944786	0.055214	0.696685	0.69643	481.2403	691.0144
PPIH	0.944805	0.055195	1.438346	1.43879	809.1062	562.3489

LINC0124	0.94484	0.05516	0.525927	0.505084	6.657343	13.19046
DMBT1P1	0.944845	0.055155	0.20752	0.004498	0	2.213405
TNRC6C-/	0.944873	0.055127	0.705398	0.704944	265.6269	376.8098
AASDHPF	0.944962	0.055038	0.676827	0.676729	1291.458	1908.387
ORMDL3	0.944968	0.055032	1.445102	1.445477	978.0404	676.6182
ZNF610	0.945012	0.054988	0.688715	0.686283	50.01343	72.88042
PLCD1	0.945031	0.054969	1.409897	1.411662	186.7168	132.2645
PJA1	0.945041	0.054959	1.450861	1.451185	1172.537	807.983
RNF13	0.945045	0.054955	0.69672	0.696465	471.2377	676.6182
MEF2D	0.945051	0.054949	1.474759	1.474963	1928.296	1307.349
ABI3	0.945052	0.054948	2.579377	3.076539	5.557048	1.799517
ADCY4	0.945052	0.054948	2.579377	3.076539	5.557048	1.799517
ANKFN1	0.945052	0.054948	2.579377	3.076539	5.557048	1.799517
C8G	0.945052	0.054948	2.579377	3.076539	5.557048	1.799517
GBGT1	0.945052	0.054948	2.579377	3.076539	5.557048	1.799517
GCM1	0.945052	0.054948	2.579377	3.076539	5.557048	1.799517
LINC0101	0.945052	0.054948	2.579377	3.076539	5.557048	1.799517
LOC10192	0.945052	0.054948	2.579377	3.076539	5.557048	1.799517
MAGEB2	0.945052	0.054948	2.579377	3.076539	5.557048	1.799517
NR1I2	0.945052	0.054948	2.579377	3.076539	5.557048	1.799517
PLEKHG7	0.945052	0.054948	2.579377	3.076539	5.557048	1.799517
RASGEF1	0.945052	0.054948	2.579377	3.076539	5.557048	1.799517
RFPL4A	0.945052	0.054948	2.579377	3.076539	5.557048	1.799517
UXT-AS1	0.945052	0.054948	2.579377	3.076539	5.557048	1.799517
C2orf27B	0.94506	0.05494	0.206582	0.004391	0	2.267391
CDC26	0.945094	0.054906	1.44507	1.445442	985.8203	682.0168
ST6GALN	0.945114	0.054886	0.70289	0.702529	335.6457	477.7717
PALLD	0.94514	0.05486	1.528772	1.528846	6319.475	4133.49
SUPT6H	0.945175	0.054825	0.668125	0.668058	1928.296	2886.425
WWP2	0.945196	0.054804	0.692825	0.69262	590.1585	852.0711
ZNF540	0.945212	0.054788	0.480181	0.449731	4.445638	9.897341
AGAP2-AS	0.945222	0.054778	1.460734	1.466729	63.35035	43.1884
METTL25	0.945267	0.054733	0.640566	0.634418	21.11678	33.29106
DYNLT3	0.945301	0.054699	1.454495	1.454794	1259.227	865.5675
MILR1	0.945312	0.054688	2.046581	2.178798	9.813747	4.498792
CEACAM1	0.945335	0.054665	0.701566	0.699992	75.57585	107.971
FGD5P1	0.945362	0.054638	2.569658	3.061317	5.557048	1.808514
AGAP5	0.945363	0.054637	0.688509	0.685932	48.12403	70.16315
CPNE2	0.945429	0.054571	1.418747	1.419479	462.3464	325.7125
HESX1	0.945436	0.054564	0.450561	0.412469	3.334229	8.097825
RBAK-RB/	0.94544	0.05456	0.208866	0.004534	0	2.19541
LINC-PINT	0.94544	0.05456	1.41135	1.412359	327.8658	232.1376
TTC38	0.945514	0.054486	1.419804	1.420504	485.686	341.9082
PDE4C	0.945529	0.054471	0.629527	0.622309	18.18266	29.22415
LOC1005C	0.945673	0.054327	0.65229	0.647119	24.45101	37.78985
NIPAL1	0.94572	0.05428	0.707943	0.706875	110.0407	155.6762
HOMER3	0.945736	0.054264	1.465688	1.46592	1673.783	1141.793
SGF29	0.945774	0.054226	0.705872	0.705422	258.9584	367.1014
CDC37L1-	0.945798	0.054202	0.334392	0.248716	1.11141	4.498792
COL6A4P	0.945798	0.054202	0.334392	0.248716	1.11141	4.498792
GH2	0.945798	0.054202	0.334392	0.248716	1.11141	4.498792
NUPR2	0.945798	0.054202	0.334392	0.248716	1.11141	4.498792
RRAD	0.945798	0.054202	0.334392	0.248716	1.11141	4.498792
SCARNA1	0.945798	0.054202	0.334392	0.248716	1.11141	4.498792
SEPT4-AS	0.945798	0.054202	0.334392	0.248716	1.11141	4.498792
SLC7A3	0.945798	0.054202	0.334392	0.248716	1.11141	4.498792

STX17-AS	0.945798	0.054202	0.334392	0.248716	1.11141	4.498792
TOLLIP-AS	0.945798	0.054202	0.334392	0.248716	1.11141	4.498792
DKFZP434	0.945832	0.054168	0.603137	0.593092	13.33691	22.49396
LINC0085	0.945832	0.054168	0.603137	0.593092	13.33691	22.49396
TMEM136	0.945857	0.054143	0.710114	0.709232	135.3141	190.7937
OSBPL2	0.945863	0.054137	1.475406	1.475598	2046.905	1387.166
AK3	0.945877	0.054123	1.490649	1.490793	2900.346	1945.502
FAM229A	0.945883	0.054117	1.956072	2.056761	11.1141	5.39855
FLJ42351	0.945883	0.054117	1.956072	2.056761	11.1141	5.39855
GFI1	0.945883	0.054117	1.956072	2.056761	11.1141	5.39855
REV3L	0.945898	0.054102	0.689087	0.688922	730.1961	1059.915
ABCC10	0.945947	0.054053	0.698589	0.698327	456.7893	654.1243
C17orf80	0.945958	0.054042	0.692195	0.69201	666.8457	963.6411
ZFP42	0.945984	0.054016	0.375983	0.310591	1.678228	5.425543
MUC5B	0.945995	0.054005	0.451036	0.412469	3.334229	8.097825
OGFRP1	0.945995	0.054005	0.451036	0.412469	3.334229	8.097825
PHACTR1	0.945995	0.054005	0.451036	0.412469	3.334229	8.097825
RERG	0.945995	0.054005	0.451036	0.412469	3.334229	8.097825
SLC9A9	0.945995	0.054005	0.451036	0.412469	3.334229	8.097825
LOC10041	0.946014	0.053986	0.704141	0.703773	322.3088	457.977
CD101	0.946046	0.053954	2.572269	3.076539	5.557048	1.799517
GRM1	0.946046	0.053954	2.572269	3.076539	5.557048	1.799517
LOC10106	0.946046	0.053954	2.572269	3.076539	5.557048	1.799517
PXDNL	0.946046	0.053954	2.572269	3.076539	5.557048	1.799517
SPACA6P	0.946046	0.053954	2.572269	3.076539	5.557048	1.799517
KCNA7	0.946086	0.053914	1.575339	1.593079	29.41901	18.46304
DNAL4	0.946146	0.053854	0.698508	0.698251	466.792	668.5204
PEX11A	0.946214	0.053786	0.709617	0.70865	121.1436	170.9541
RHBDD2	0.946218	0.053782	1.464604	1.464835	1680.451	1147.192
FAM35DP	0.94623	0.05377	0.653789	0.648553	24.41767	37.65489
ATP1B2	0.946305	0.053695	2.014588	2.137844	10.0138	4.678743
STK11IP	0.946327	0.053673	0.7045	0.704136	325.643	462.4758
LONP1	0.946344	0.053656	0.656706	0.656668	3392.022	5165.512
FUK	0.946345	0.053655	0.711204	0.710404	148.9289	209.6437
HCG18	0.946378	0.053622	0.696505	0.696278	531.2538	762.995
MYBL1	0.946383	0.053617	1.451064	1.45136	1283.678	884.4624
DUXA	0.946389	0.053611	2.551347	3.039687	5.490363	1.799517
PKP1	0.946403	0.053597	0.675527	0.671856	34.4537	51.28622
ABCC3	0.946416	0.053584	1.428439	1.42894	696.8538	487.669
BEGAIN	0.946441	0.053559	1.954355	2.056761	11.1141	5.39855
LOC10050	0.946441	0.053559	1.954355	2.056761	11.1141	5.39855
MIR4697H	0.946441	0.053559	1.954355	2.056761	11.1141	5.39855
STK32B	0.946441	0.053559	1.954355	2.056761	11.1141	5.39855
EXOC6	0.946442	0.053558	0.705162	0.704786	314.5289	446.2801
MIRLET7E	0.946443	0.053557	1.558204	1.574157	31.36398	19.92065
RIMBP3B	0.946481	0.053519	2.739832	3.463329	4.823518	1.385628
RDH5	0.946486	0.053514	4.394018	197.7195	1.967195	0
LOC10050	0.946497	0.053503	0.450782	0.411556	3.334229	8.11582
RTN4RL2	0.946529	0.053471	0.564575	0.54927	8.891277	16.19565
ZNF135	0.946529	0.053471	0.564575	0.54927	8.891277	16.19565
LDAH	0.946533	0.053467	0.703585	0.703243	342.3142	486.7692
INPP1	0.94654	0.05346	1.40424	1.405399	284.5209	202.4456
ANKS3	0.946558	0.053442	1.407115	1.408145	316.7517	224.9396
ABCA10	0.946586	0.053414	0.407453	0.353949	2.222819	6.298308
ADAMTS1	0.946586	0.053414	0.407453	0.353949	2.222819	6.298308
ANKRD55	0.946586	0.053414	0.407453	0.353949	2.222819	6.298308

FAM124A	0.946586	0.053414	0.407453	0.353949	2.222819	6.298308
HCG25	0.946586	0.053414	0.407453	0.353949	2.222819	6.298308
ISM1	0.946586	0.053414	0.407453	0.353949	2.222819	6.298308
LINC0068	0.946586	0.053414	0.407453	0.353949	2.222819	6.298308
LINC0106	0.946586	0.053414	0.407453	0.353949	2.222819	6.298308
LMCD1-AS	0.946586	0.053414	0.407453	0.353949	2.222819	6.298308
LOC10013	0.946586	0.053414	0.407453	0.353949	2.222819	6.298308
LOC44120	0.946586	0.053414	0.407453	0.353949	2.222819	6.298308
ZNF205-A	0.946586	0.053414	0.407453	0.353949	2.222819	6.298308
NUDCD1	0.946597	0.053403	1.455781	1.45605	1407.045	966.3404
ARSK	0.946669	0.053331	1.409069	1.409936	385.6591	273.5265
MRPL54	0.946701	0.053299	0.711079	0.71047	195.6081	275.326
SETD6	0.946723	0.053277	0.711008	0.710124	132.2577	186.25
ASH2L	0.946728	0.053272	0.698922	0.698672	479.0175	685.6158
CHGA	0.946731	0.053269	1.676784	1.709713	20.00537	11.69686
ADAT1	0.946733	0.053267	0.707013	0.706596	282.6203	399.9786
DARS	0.946738	0.053262	0.666292	0.666235	2208.371	3314.71
DRC1	0.946772	0.053228	0.451716	0.412469	3.334229	8.097825
PRORSD1	0.946772	0.053228	0.451716	0.412469	3.334229	8.097825
VWA5B2	0.946808	0.053192	0.6258	0.617757	16.67114	26.99275
IRF5	0.946812	0.053188	0.711354	0.710545	144.4832	203.3454
AAMDC	0.946839	0.053161	1.405406	1.406484	301.192	214.1425
NOL12	0.946876	0.053124	0.710179	0.709621	210.0786	296.0475
SLC25A51	0.946892	0.053108	1.404743	1.405773	320.3416	227.8728
TNKS2-AS	0.946917	0.053083	0.564854	0.54927	8.891277	16.19565
NEURL4	0.946919	0.053081	0.698838	0.698593	489.0202	700.012
BOK	0.946936	0.053064	1.429227	1.429697	743.533	520.0603
TNFAIP6	0.946995	0.053005	1.676192	1.709713	20.00537	11.69686
LIN9	0.946998	0.053002	0.70991	0.709385	223.3933	314.9154
SYNE2	0.947	0.053	0.683185	0.68307	1090.293	1596.171
LINC0064	0.947039	0.052961	0.701113	0.699232	63.29478	90.52468
CDKL2	0.947044	0.052956	0.689022	0.686288	44.45638	64.7826
WRN	0.94709	0.05291	0.702308	0.702015	406.7759	579.4443
LOC64284	0.947116	0.052884	0.705149	0.70356	74.30884	105.6226
SOCS7	0.947213	0.052787	1.426439	1.426931	703.5778	493.0676
IL13RA1	0.947226	0.052774	1.509468	1.509563	4599.013	3046.582
ENDOV	0.947277	0.052723	0.707847	0.707433	284.5209	402.192
CFLAR	0.94731	0.05269	1.467629	1.467833	1915.092	1304.704
AIMP1	0.947316	0.052684	0.678775	0.678682	1352.585	1992.965
CTSV	0.947354	0.052646	0.697733	0.697513	557.9276	799.8851
COL6A4P	0.947359	0.052641	0.407845	0.353445	2.222819	6.307306
ZBTB42	0.947366	0.052634	1.401526	1.402803	251.1786	179.0519
MFSD14C	0.947409	0.052591	1.565524	1.582757	29.18562	18.43605
ORAOV1	0.947496	0.052504	0.706945	0.706569	313.4175	443.5808
C9orf66	0.947515	0.052485	0.408378	0.353949	2.222819	6.298308
DSCR8	0.947515	0.052485	0.408378	0.353949	2.222819	6.298308
HOXB-AS	0.947515	0.052485	0.408378	0.353949	2.222819	6.298308
ZNF880	0.947535	0.052465	1.439644	1.444894	68.90739	47.68719
PRNCR1	0.947552	0.052448	0.569368	0.554282	9.002418	16.24963
LIMS3	0.947558	0.052442	1.401247	1.403164	167.3116	119.236
LIMS3L	0.947558	0.052442	1.401247	1.403164	167.3116	119.236
MYO1B	0.947591	0.052409	1.489789	1.489921	3205.305	2151.322
TRAF3IP2	0.947603	0.052397	1.429046	1.429494	780.354	545.8924
TNFRSF1	0.947609	0.052391	1.487556	1.497083	44.45638	29.69202
NRTN	0.947632	0.052368	1.398345	1.399902	207.8336	148.4601
LOC40331	0.947661	0.052339	4.336492	194.3853	1.933853	0

FICD	0.947662	0.052338	0.709841	0.708465	86.68995	122.3671
SLC25A26	0.947681	0.052319	0.713114	0.712482	187.8282	263.6292
ZNF75A	0.947709	0.052291	0.713291	0.712645	183.3826	257.3309
NPRL3	0.947715	0.052285	0.701028	0.700776	482.3518	688.3151
ZNF586	0.947717	0.052283	0.671338	0.667099	30.00806	44.98792
KIN	0.947737	0.052263	0.710092	0.709625	251.0007	353.713
UCKL1-AS	0.947759	0.052241	1.575832	1.594788	27.58519	17.29335
IFT52	0.947761	0.052239	0.695087	0.694897	634.6149	913.2547
ANKRD37	0.947771	0.052229	1.542003	1.557196	32.23088	20.69444
TAF1B	0.947812	0.052188	0.709736	0.709289	262.2927	369.8007
MACF1	0.947843	0.052157	1.522046	1.522121	6020.506	3955.338
JPH1	0.947891	0.052109	0.694232	0.694054	691.2968	996.0324
OXSR1	0.947906	0.052094	1.501667	1.501773	4057.756	2701.974
LTB4R	0.947911	0.052089	1.453063	1.459703	57.7933	39.58937
HSFX1	0.947916	0.052084	1.646632	1.675849	21.11678	12.59662
NLRP11	0.947916	0.052084	1.646632	1.675849	21.11678	12.59662
PDZRN3	0.947916	0.052084	1.646632	1.675849	21.11678	12.59662
NMT2	0.94798	0.05202	1.43331	1.433704	898.119	626.4297
CCDC30	0.94798	0.05202	0.602282	0.591258	12.36999	20.92838
CEBPB-AS	0.947989	0.052011	0.682783	0.679437	36.67652	53.9855
ZNF396	0.948004	0.051996	0.601912	0.59096	12.22551	20.69444
RAMP2	0.948026	0.051974	1.862714	1.939586	12.22551	6.298308
PRKAR1B	0.94805	0.05195	0.71247	0.711903	205.6108	288.8224
FAM210B	0.948069	0.051931	0.700617	0.700377	507.9142	725.2052
PPT2	0.948085	0.051915	1.41188	1.412561	486.5418	344.4365
CHST10	0.948086	0.051914	0.691096	0.68825	43.34497	62.98308
LRRC34	0.94815	0.05185	1.646105	1.675849	21.11678	12.59662
AP1S3	0.94817	0.05183	1.412618	1.413292	485.9416	343.8336
FZD1	0.948181	0.051819	0.708782	0.707296	78.91008	111.57
ZNF766	0.94823	0.05177	1.450618	1.450884	1428.161	984.3356
ZFAS1	0.94823	0.05177	0.670914	0.67085	1953.858	2912.518
ST3GAL4	0.948253	0.051747	1.422646	1.423143	690.1853	484.9697
GM2A	0.948303	0.051697	1.491936	1.492059	3389.799	2271.89
DTNB	0.948308	0.051692	0.703977	0.70369	413.4444	587.5422
ZNF503-A	0.948339	0.051661	0.705967	0.704228	67.79598	96.27414
FAM122C	0.948358	0.051642	0.710169	0.708686	80.36603	113.4055
ANK1	0.948367	0.051633	1.861772	1.939586	12.22551	6.298308
LINC0109	0.948367	0.051633	1.861772	1.939586	12.22551	6.298308
PRDM6	0.948367	0.051633	1.861772	1.939586	12.22551	6.298308
S100Z	0.948367	0.051633	1.861772	1.939586	12.22551	6.298308
SBSN	0.948367	0.051633	1.861772	1.939586	12.22551	6.298308
GFM1	0.948375	0.051625	0.665375	0.665324	2572.913	3867.161
AMN	0.948411	0.051589	1.529818	1.543787	33.34229	21.5942
SHPK	0.948414	0.051586	1.39537	1.396888	211.1567	151.1594
KLHDC7B	0.948462	0.051538	0.697255	0.69486	50.01343	71.98066
IQCE	0.948474	0.051526	0.70678	0.706456	371.2108	525.4588
ACACB	0.948529	0.051471	0.713634	0.712651	116.698	163.756
MED11	0.94862	0.05138	0.713837	0.712654	100.0269	140.3623
ABHD3	0.948643	0.051357	0.686967	0.686843	986.9317	1436.914
FBXO24	0.948663	0.051337	0.626215	0.617767	15.55973	25.19323
HCAR2	0.948663	0.051337	0.626215	0.617767	15.55973	25.19323
ANG	0.948677	0.051323	1.429918	1.434805	71.88597	50.09854
ZNF557	0.948691	0.051309	0.714703	0.714106	197.9198	277.1615
CATSPER	0.948825	0.051175	0.558201	0.540733	7.779867	14.39613
LRRC29	0.948825	0.051175	0.558201	0.540733	7.779867	14.39613
NEK10	0.948825	0.051175	0.558201	0.540733	7.779867	14.39613

KRT83	0.948826	0.051174	1.810332	1.875014	13.37026	7.126086
CYP1A2	0.948867	0.051133	1.621124	1.647319	22.23931	13.49637
TXLNA	0.948869	0.051131	1.472549	1.472719	2329.514	1581.775
CSPG5	0.948878	0.051122	1.395036	1.396671	192.2739	137.663
ZNF304	0.948902	0.051098	1.416628	1.417175	614.6095	433.6835
R3HCC1	0.948914	0.051086	0.712139	0.711677	252.29	354.5048
C8orf34	0.948922	0.051078	0.713815	0.712654	100.0269	140.3623
STAC2	0.948931	0.051069	0.690462	0.687497	40.86653	59.44703
CERS2	0.948947	0.051053	0.649602	0.649577	5296.978	8154.51
HOXC9	0.948955	0.051045	1.620335	1.646496	22.22819	13.49637
FAM109A	0.948986	0.051014	1.401735	1.402571	391.2162	278.9251
COG2	0.949003	0.050997	0.708557	0.708205	334.5343	472.3731
MAD2L1	0.949021	0.050979	1.448885	1.449149	1400.376	966.3404
MECOM	0.949025	0.050975	0.68895	0.688818	924.6928	1342.439
ESRG	0.94904	0.05096	1.522829	1.522898	6621.567	4348.001
PNPT1	0.949052	0.050948	0.690817	0.690673	831.1899	1203.454
CCDC78	0.94907	0.05093	1.518331	1.53145	34.4537	22.49396
HCN2	0.94907	0.05093	1.518331	1.53145	34.4537	22.49396
TSR1	0.949082	0.050918	1.471454	1.471626	2303.952	1565.579
TRIM13	0.949126	0.050874	0.715966	0.715187	148.5288	207.6822
LMNTD2	0.949127	0.050873	0.655954	0.650224	22.22819	34.19082
FIG4	0.949162	0.050838	0.701866	0.701628	501.2457	714.4081
URB1-AS1	0.949179	0.050821	0.70764	0.705878	66.68457	94.47462
GOLGA6A	0.949179	0.050821	2.525613	3.033638	5.179169	1.700543
AKTIP	0.949186	0.050814	0.69418	0.694014	716.8592	1032.923
PRPF38A	0.94921	0.05079	0.685061	0.68495	1113.632	1625.863
RAD51L3-	0.949293	0.050707	1.759692	1.813298	14.904	8.214793
SPTBN4	0.949323	0.050677	1.412899	1.416815	86.68995	61.18356
PRRC1	0.949328	0.050672	1.496045	1.496153	4002.186	2674.981
MAPKBP1	0.949332	0.050668	1.417459	1.417963	681.2941	480.4709
MPV17	0.949333	0.050667	0.696864	0.696683	675.737	969.9395
RPS6KA2	0.949334	0.050666	1.494601	1.494713	3713.219	2484.233
SLC10A7	0.949334	0.050666	0.715797	0.714786	114.4752	160.157
BBS7	0.949373	0.050627	0.705867	0.705578	410.1101	581.2439
GABARAP	0.949457	0.050543	1.437006	1.437329	1130.304	786.3888
RBM26	0.949501	0.050499	0.692807	0.692656	800.2149	1155.29
EPB41L2	0.94951	0.05049	1.476779	1.476931	2641.821	1788.72
KIF27	0.949538	0.050462	1.403177	1.406194	107.0176	76.10156
BLM	0.949651	0.050349	0.704174	0.703917	462.3464	656.8236
GPRIN3	0.949684	0.050316	1.507706	1.520062	35.56511	23.39372
WNT10B	0.949735	0.050265	1.460899	1.468783	48.90202	33.29106
YWHAEP1	0.949766	0.050234	2.193037	2.423928	7.124135	2.933212
AQP4-AS1	0.949772	0.050228	2.458196	2.903914	5.401451	1.853502
LANCL2	0.949789	0.050211	0.717163	0.716447	161.1544	224.9396
ZFP69	0.949794	0.050206	0.705986	0.703984	58.90471	83.67752
PCDHGA9	0.949822	0.050178	1.584383	1.606131	24.57327	15.29589
FOXD2	0.949874	0.050126	1.597197	1.62081	23.3396	14.39613
KCNN1	0.949874	0.050126	1.597197	1.62081	23.3396	14.39613
GUF1	0.949904	0.050096	0.704014	0.703765	476.8169	677.527
MYEF2	0.949918	0.050082	0.701068	0.698683	49.88006	71.39582
PTGES	0.949992	0.050008	0.716939	0.716367	205.6108	287.0229
ZNF223	0.950016	0.049984	1.392778	1.394922	145.5947	104.372
MYO3B	0.950019	0.049981	1.399359	1.400183	386.7705	276.2258
GPR155	0.950019	0.049981	1.412344	1.412896	601.3504	425.6127
PARP6	0.950035	0.049965	0.697548	0.697367	665.7343	954.6436
AKAP1	0.950047	0.049953	0.696	0.695832	716.8592	1030.223

SETD9	0.950056	0.049944	0.71776	0.716834	124.4779	173.6534
ZC4H2	0.950056	0.049944	1.596769	1.62081	23.3396	14.39613
ZNF773	0.950063	0.049937	1.398848	1.399683	381.0912	272.2669
ZNF490	0.950065	0.049935	0.713794	0.712367	81.32184	114.1613
SLC2A10	0.950072	0.049928	0.678331	0.678252	1572.645	2318.677
ZNF516	0.950084	0.049916	1.402274	1.40301	436.784	311.3164
DEFB4B	0.950107	0.049893	4.18598	183.2712	1.822712	0
ZNF114	0.950123	0.049877	0.717779	0.717162	190.2622	265.3027
MTUS1	0.950133	0.049867	0.674642	0.674574	1849.386	2741.564
FUT4	0.950233	0.049767	1.409542	1.410124	564.6072	400.3924
USP2-AS1	0.95026	0.04974	1.497849	1.509517	36.67652	24.29347
DPYSL3	0.950263	0.049737	1.789291	1.851664	13.33691	7.198066
GRIP2	0.950263	0.049737	1.789291	1.851664	13.33691	7.198066
DHRS7B	0.950269	0.049731	0.713867	0.713435	268.9611	376.9987
ATP2A3	0.950274	0.049726	1.38796	1.389607	190.051	136.7633
CFD	0.950277	0.049723	0.712172	0.71056	72.24162	101.6727
NEK2	0.950303	0.049697	1.421179	1.421613	786.6557	553.3514
ZBTB45	0.950304	0.049696	0.715835	0.715326	227.839	318.5144
NFKBID	0.950375	0.049625	1.397087	1.399879	113.3638	80.97825
LUZP1	0.950388	0.049612	1.459541	1.459739	1929.407	1321.745
NAT10	0.950409	0.049591	0.688609	0.688492	1058.062	1536.787
ERVV-2	0.950478	0.049522	1.389242	1.390567	232.5847	167.2561
FAM218A	0.950485	0.049515	0.600682	0.588423	11.1141	18.89492
SNAI3-AS	0.950485	0.049515	0.600682	0.588423	11.1141	18.89492
SLC4A8	0.950507	0.049493	1.556201	1.574566	26.79608	17.01443
FABP6	0.950531	0.049469	0.710057	0.70974	376.7678	530.8574
SNHG5	0.950536	0.049464	0.68679	0.686682	1154.755	1681.648
WRB	0.950542	0.049458	0.69451	0.694358	795.3469	1145.446
LOC10028	0.950548	0.049452	0.656971	0.65095	21.11678	32.44528
LOC10192	0.950595	0.049405	0.691745	0.688546	37.78793	54.88526
TRIM73	0.950612	0.049388	0.462178	0.421507	3.123061	7.423006
BCLAF1	0.950613	0.049387	0.665577	0.665531	2801.864	4209.969
KIAA0101	0.950615	0.049385	0.700039	0.699848	639.105	913.2097
ZNF14	0.950654	0.049346	0.719236	0.71839	135.7365	188.9492
CNTD1	0.95068	0.04932	1.788201	1.851664	13.33691	7.198066
FA2H	0.950695	0.049305	1.576682	1.598144	24.45101	15.29589
LGR6	0.950695	0.049305	1.576682	1.598144	24.45101	15.29589
IMPDH2	0.950702	0.049298	0.666418	0.666372	2787.415	4182.976
ABCC9	0.950736	0.049264	0.706246	0.705993	474.2162	671.7056
CPT1A	0.950761	0.049239	0.673639	0.673577	2000.771	2970.372
DLGAP4	0.950779	0.049221	1.479165	1.4793	2984.135	2017.258
FAF2	0.950847	0.049153	0.666465	0.666418	2731.845	4099.299
HIST1H4H	0.950848	0.049152	0.644917	0.637669	17.78255	27.89251
GOLGA8N	0.950888	0.049112	0.631923	0.623211	15.27077	24.50942
SEC16B	0.950891	0.049109	1.403184	1.406728	91.13559	64.7826
MRPS31P	0.950907	0.049093	0.699353	0.696652	44.1563	63.38797
GUSBP2	0.950909	0.049091	1.703258	1.747208	16.21547	9.276508
BRI3BP	0.950909	0.049091	0.719282	0.718607	170.0457	236.6364
FAM50B	0.950919	0.049081	0.719604	0.718877	157.8202	219.541
SLITRK5	0.950936	0.049064	0.716592	0.716129	254.5128	355.4045
RCBTB1	0.950961	0.049039	0.701069	0.700871	603.4954	861.0687
ONECUT2	0.950975	0.049025	0.645051	0.637669	17.78255	27.89251
GCSH	0.951013	0.048987	0.685115	0.685016	1242.556	1813.913
ADAM9	0.95102	0.04898	1.537632	1.53768	9877.097	6423.375
MYD88	0.951027	0.048973	1.418397	1.418839	755.7585	532.6569
CUL5	0.951032	0.048968	0.686274	0.686172	1218.105	1775.223

GRID2IP	0.951125	0.048875	0.550087	0.529758	6.668457	12.59662
CPOX	0.951133	0.048867	1.438063	1.438348	1259.227	875.4648
PHF21A	0.95114	0.04886	1.423078	1.423467	882.4592	619.9335
PCDHB13	0.951153	0.048847	0.639	0.631129	16.25992	25.76908
CCNI2	0.951179	0.048821	2.210529	2.465035	6.668457	2.699275
COX8C	0.951179	0.048821	2.210529	2.465035	6.668457	2.699275
FOXC2	0.951179	0.048821	2.210529	2.465035	6.668457	2.699275
HIST1H3G	0.951179	0.048821	2.210529	2.465035	6.668457	2.699275
LINC0024:	0.951179	0.048821	2.210529	2.465035	6.668457	2.699275
LMO1	0.951179	0.048821	2.210529	2.465035	6.668457	2.699275
COG1	0.951253	0.048747	0.703986	0.703764	535.6994	761.1955
SVOP	0.95128	0.04872	0.429842	0.377537	2.378417	6.316303
TRIAP1	0.951289	0.048711	0.712126	0.711797	361.2081	507.4637
C16orf95	0.95131	0.04869	1.479938	1.490608	38.89934	26.09299
PIWIL2	0.951337	0.048663	1.7076	1.753191	15.78202	8.997583
SLC15A3	0.951339	0.048661	1.420063	1.425176	66.68457	46.78743
CABLES2	0.951378	0.048622	0.699438	0.699258	656.8431	939.3477
ZNF696	0.951405	0.048595	0.717397	0.716939	256.7356	358.1038
FAAH	0.951407	0.048593	0.720916	0.720078	136.7034	189.849
ADCY1	0.951433	0.048567	1.558368	1.577994	25.56242	16.19565
LINC0158	0.951433	0.048567	1.558368	1.577994	25.56242	16.19565
BBS9	0.951446	0.048554	0.718892	0.718344	210.0564	292.4214
YPEL3	0.951472	0.048528	1.402932	1.403532	546.8135	389.5953
ORM2	0.951533	0.048467	0.370844	0.291202	1.300349	4.489794
DCAF16	0.951573	0.048427	1.396459	1.397175	449.0095	321.3667
LOC10050	0.951586	0.048414	0.550454	0.529758	6.668457	12.59662
LOC37519	0.951586	0.048414	0.550454	0.529758	6.668457	12.59662
VASH1	0.951623	0.048377	0.708085	0.705885	53.34766	75.5797
RUNX3	0.951627	0.048373	0.72099	0.719981	113.3638	157.4577
XAB2	0.951649	0.048351	1.456615	1.456809	1958.304	1344.239
MED22	0.95168	0.04832	0.69599	0.69584	805.7719	1157.989
FLJ12825	0.951684	0.048316	2.200361	2.452728	6.635115	2.699275
CBWD6	0.95169	0.04831	1.390029	1.392575	119.8766	86.07988
DHX36	0.95172	0.04828	0.671434	0.671379	2276.167	3390.289
SH3YL1	0.951728	0.048272	1.437212	1.437487	1327.023	923.152
IRF2BPL	0.951733	0.048267	1.441781	1.442035	1425.938	988.8344
ZNF280D	0.951744	0.048256	0.716574	0.716164	286.7437	400.3924
DKFZp434	0.951761	0.048239	1.731841	1.783256	14.44832	8.097825
RPARP-AS	0.951842	0.048158	0.7222	0.721164	112.2524	155.6582
C21orf33	0.951845	0.048155	0.681798	0.681716	1507.071	2210.706
PAXIP1	0.951854	0.048146	0.713581	0.713245	352.3168	493.9673
DYDC1	0.951865	0.048135	0.225697	0.005004	0	1.988466
AURKA	0.951879	0.048121	1.47128	1.471425	2776.279	1886.793
ZSCAN12	0.951883	0.048117	1.413208	1.418142	68.90739	48.58695
FGGY	0.951911	0.048089	0.716735	0.715162	73.35303	102.5724
CCDC33	0.951934	0.048066	2.206572	2.465035	6.668457	2.699275
GPIHBP1	0.951934	0.048066	2.206572	2.465035	6.668457	2.699275
HCG27	0.951934	0.048066	2.206572	2.465035	6.668457	2.699275
HCG4	0.951934	0.048066	2.206572	2.465035	6.668457	2.699275
KCNJ2	0.951934	0.048066	2.206572	2.465035	6.668457	2.699275
LINC0106	0.951934	0.048066	2.206572	2.465035	6.668457	2.699275
NAP1L2	0.951934	0.048066	2.206572	2.465035	6.668457	2.699275
SLC39A2	0.951934	0.048066	2.206572	2.465035	6.668457	2.699275
PPIP5K1	0.951993	0.048007	0.689827	0.689714	1079.179	1564.68
ELMOD3	0.952023	0.047977	0.717891	0.71744	255.6242	356.3043
C10orf10	0.95203	0.04797	0.694412	0.694276	885.7934	1275.857

NOL7	0.952043	0.047957	1.470365	1.470511	2750.739	1870.598
LOC10192	0.952049	0.047951	0.224232	0.004872	0	2.042451
CEP162	0.952084	0.047916	0.722456	0.721586	131.1463	181.7512
CCK	0.952103	0.047897	1.541919	1.559965	26.67383	17.09541
PQLC2	0.952122	0.047878	0.709759	0.709486	430.1155	606.2392
LINC0120	0.952125	0.047875	4.106389	181.0484	1.800484	0
INPP5A	0.952136	0.047864	1.415144	1.415564	811.329	573.146
IL1R1	0.952145	0.047855	0.668886	0.668839	2648.489	3959.836
SSBP3	0.952179	0.047821	1.441911	1.442155	1518.185	1052.717
HOXB3	0.952192	0.047808	1.386797	1.387773	313.4175	225.8393
SOX7	0.952196	0.047804	0.598544	0.585352	10.00269	17.09541
MRPS36	0.952211	0.047789	0.703833	0.703633	604.6068	859.2692
SLC30A9	0.952228	0.047772	0.685282	0.685191	1369.257	1998.363
SPAG16	0.952229	0.047771	0.712098	0.7118	398.996	560.5494
HAUS3	0.95223	0.04777	0.710496	0.710216	417.7455	588.199
LOC28643	0.952234	0.047766	0.718555	0.717071	77.46525	108.034
C4A	0.952238	0.047762	0.690319	0.686563	32.84215	47.84015
OASL	0.952248	0.047752	1.429875	1.436203	55.57048	38.68961
FAM89B	0.952298	0.047702	1.418812	1.419195	900.2418	634.3296
GOLGA8E	0.952304	0.047696	0.708609	0.708356	464.6692	655.9868
PPP4R1	0.95238	0.04762	0.692742	0.692619	1005.826	1452.21
VENTX	0.952389	0.047611	0.22729	0.005049	0	1.970471
FOXD3	0.952415	0.047585	0.598715	0.585352	10.00269	17.09541
LOC10192	0.952453	0.047547	4.125102	185.494	1.84494	0
TSC2	0.952459	0.047541	0.687469	0.687369	1204.768	1752.729
TSSK6	0.952474	0.047526	0.670665	0.66522	23.3396	35.09057
TMEM107	0.952497	0.047503	0.723799	0.723075	160.043	221.3405
MYO5B	0.952516	0.047484	1.399894	1.400479	555.7048	396.7934
SURF4	0.952523	0.047477	1.531291	1.53134	9650.369	6301.907
LINC0089	0.952551	0.047449	0.683675	0.679401	28.62991	42.14468
SEPT5	0.952555	0.047445	1.379082	1.380433	224.8159	162.8563
TBCEL	0.952589	0.047411	0.702071	0.701892	675.737	962.7414
SLC14A2	0.952594	0.047406	0.480278	0.442378	3.44537	7.800905
ZNF470	0.95261	0.04739	1.38396	1.38644	122.2551	88.17631
RNPC3	0.952639	0.047361	1.37936	1.381028	178.9369	129.5652
C16orf46	0.952643	0.047357	1.527276	1.543737	27.78524	17.99517
GALNT15	0.952674	0.047326	2.180023	2.428115	6.568431	2.699275
TRUB1	0.952687	0.047313	0.691154	0.69104	1066.953	1543.985
RILP	0.952697	0.047303	1.407011	1.411611	71.13021	50.38647
LSM8	0.952709	0.047291	0.681375	0.681298	1617.101	2373.562
GATB	0.952716	0.047284	0.719679	0.719216	247.8443	344.6074
LPIN1	0.952723	0.047277	0.703738	0.703546	632.3921	898.8675
NAALADL	0.952726	0.047274	0.598959	0.585352	10.00269	17.09541
FAM109B	0.952739	0.047261	0.627611	0.617793	13.33691	21.5942
LBX2-AS1	0.952739	0.047261	0.627611	0.617793	13.33691	21.5942
EMBP1	0.95277	0.04723	1.401083	1.405378	76.1649	54.19244
ZNF675	0.952796	0.047204	1.377939	1.379306	221.2816	160.4269
SERTAD1	0.952799	0.047201	1.411544	1.411982	754.6471	534.4564
NSUN6	0.952807	0.047193	0.717367	0.716978	296.7464	413.8888
CRIP1	0.952821	0.047179	1.526758	1.543737	27.78524	17.99517
TTYH3	0.952831	0.047169	1.479	1.479119	3437.59	2324.076
PDCD7	0.95287	0.04713	0.705673	0.705467	584.6014	828.6774
IFT27	0.952875	0.047125	0.71377	0.713458	372.3222	521.8598
COIL	0.952912	0.047088	0.705861	0.705651	561.2618	795.3863
LINC0065	0.952975	0.047025	1.685185	1.728514	15.55973	8.997583
PRAP1	0.952975	0.047025	1.685185	1.728514	15.55973	8.997583

FLJ31356	0.952987	0.047013	1.448858	1.457248	44.40081	30.46582
CBWD1	0.952992	0.047008	1.452382	1.452574	1947.278	1340.568
BTBD9	0.953033	0.046967	0.72413	0.723435	163.3772	225.8393
DFNB31	0.953033	0.046967	1.38427	1.385242	307.8605	222.2403
DVL2	0.953098	0.046902	0.69449	0.694363	950.2552	1368.532
IFT20	0.953117	0.046883	1.386667	1.387505	364.5423	262.7294
BNIP3L	0.953121	0.046879	1.459587	1.459753	2348.408	1608.768
TXNDC5	0.953144	0.046856	1.551271	1.551304	14869.12	9584.909
VPS52	0.953146	0.046854	0.700058	0.699898	746.8672	1067.113
GTDC1	0.953166	0.046834	1.379355	1.380532	253.4014	183.5507
C1orf106	0.953264	0.046736	1.463871	1.464025	2518.543	1720.284
SPIB	0.953267	0.046733	0.578072	0.561132	7.846552	13.99124
PBX2	0.953297	0.046703	0.688461	0.688364	1281.455	1861.6
SYP	0.953299	0.046701	1.684417	1.728514	15.55973	8.997583
DNAJC9-A	0.953324	0.046676	1.947858	2.074149	9.024646	4.345833
FXYD6	0.95333	0.04667	1.378209	1.379425	244.5101	177.2524
CMC4	0.953332	0.046668	0.723262	0.722697	201.543	278.8801
RTKN2	0.9534	0.0466	0.696718	0.696581	881.2144	1265.06
TMPPE	0.953405	0.046595	0.717053	0.715167	61.12753	85.47704
ZNF711	0.953406	0.046594	0.712615	0.710298	51.12484	71.98066
CBX3P2	0.953418	0.046582	0.539405	0.515129	5.557048	10.7971
F2RL2	0.953418	0.046582	0.539405	0.515129	5.557048	10.7971
LOC10013	0.953418	0.046582	0.539405	0.515129	5.557048	10.7971
RORA-AS	0.953418	0.046582	0.539405	0.515129	5.557048	10.7971
PPP1R3E	0.953436	0.046564	0.715724	0.713725	57.7933	80.97825
TFAP2A-A	0.953444	0.046556	1.444633	1.453065	44.45638	30.59178
ERCC6-P(	0.953457	0.046543	0.726334	0.725345	116.4535	160.5529
RHCE	0.953477	0.046523	0.71378	0.711676	54.42573	76.47946
LYSMD2	0.953486	0.046514	1.444786	1.453065	44.45638	30.59178
PTGER2	0.95351	0.04649	1.38467	1.387676	101.1383	72.88042
KCNQ4	0.953514	0.046486	1.41343	1.419103	60.01612	42.28864
SLC28A2	0.953561	0.046439	0.452334	0.404054	2.600698	6.451267
TTC28	0.953579	0.046421	0.713281	0.712998	411.2215	576.7541
NIF3L1	0.953579	0.046421	0.708188	0.707966	531.2538	750.3984
FAM193A	0.953588	0.046412	0.718928	0.71854	296.7464	412.9891
KTI12	0.953596	0.046404	1.377563	1.378734	253.1458	183.6047
ALPPL2	0.953606	0.046394	1.818835	1.898501	10.9585	5.767451
SRA1	0.953625	0.046375	1.444945	1.445154	1782.701	1233.569
YJEFN3	0.953634	0.046366	1.413534	1.419103	60.01612	42.28864
EFTUD1P	0.953677	0.046323	0.231372	0.005167	0	1.925483
RGPD3	0.953695	0.046305	1.655178	1.693579	16.44886	9.708392
FAM222B	0.953705	0.046295	0.70998	0.709741	492.3544	693.7137
GRIN2D	0.95371	0.04629	1.374544	1.376136	184.494	134.064
ZNF57	0.95371	0.04629	1.374544	1.376136	184.494	134.064
PROS1	0.95371	0.04629	1.381563	1.382492	323.4202	233.9372
ANXA2P1	0.95371	0.04629	1.752678	1.81368	12.73675	7.018115
RBM10	0.953759	0.046241	0.684427	0.684347	1561.53	2281.787
EMID1	0.95376	0.04624	0.720737	0.720311	268.9611	373.3997
TNS2	0.953768	0.046232	0.707289	0.707077	549.0363	776.4914
PARD6A	0.953769	0.046231	0.681324	0.676522	25.56242	37.78985
B4GALNT	0.953769	0.046231	1.38696	1.387716	404.5531	291.5217
ACAD10	0.953778	0.046222	0.724133	0.723606	220.0591	304.1183
PITPNM3	0.953784	0.046216	0.709942	0.709706	496.8001	700.012
LINC0084	0.953792	0.046208	1.501287	1.515705	30.00806	19.79468
FBXO25	0.953797	0.046203	0.712092	0.711831	446.7867	627.6624
AREG	0.953814	0.046186	0.727063	0.726308	152.2631	209.6437

BLACAT1	0.953828	0.046172	1.386116	1.389575	90.02418	64.7826
SMKR1	0.953834	0.046166	0.706098	0.703182	41.12215	58.48429
TIAM1	0.953834	0.046166	0.701978	0.701815	745.7558	1062.615
MAN1C1	0.95387	0.04613	1.501023	1.515705	30.00806	19.79468
CCNJ	0.95387	0.04613	0.710794	0.71055	480.1289	675.7185
PKN3	0.953893	0.046107	1.376592	1.377738	257.847	187.1497
GVQW2	0.953922	0.046078	0.53984	0.515129	5.557048	10.7971
FAM53B-A	0.953934	0.046066	0.369918	0.282847	1.155866	4.111895
LAMA2	0.953937	0.046063	0.706045	0.703182	41.12215	58.48429
NAGPA	0.953952	0.046048	0.722239	0.72058	70.0188	97.1739
CASP8AP	0.954039	0.045961	0.696566	0.696436	928.027	1332.542
ZNF354C	0.95404	0.04596	1.385469	1.386234	397.8846	287.0229
CENPO	0.954057	0.045943	1.418992	1.41933	1002.491	706.3103
ERCC8	0.954101	0.045899	0.724657	0.724111	207.8336	287.0229
HOXB4	0.954134	0.045866	1.37689	1.37795	278.9638	202.4456
ATP8A2	0.954134	0.045866	1.37264	1.373925	231.1732	168.2548
PGC	0.954146	0.045854	2.002325	2.158436	7.779867	3.599033
IKBIP	0.954148	0.045852	1.397382	1.397923	584.8904	418.3966
CCDC180	0.954167	0.045833	0.668501	0.662427	20.58331	31.07765
MED9	0.954181	0.045819	1.383129	1.38394	372.3222	269.0277
DNA2	0.954216	0.045784	0.710458	0.710222	490.1316	690.1146
COL9A3	0.954254	0.045746	0.719176	0.717265	60.01612	83.67752
ZNF324	0.954264	0.045736	0.712471	0.712216	458.1786	643.3182
BLOC1S5-	0.954264	0.045736	0.710462	0.710235	524.4186	738.3777
PIGN	0.95427	0.04573	1.387144	1.387848	434.5611	313.1159
IGSF9B	0.95427	0.04573	1.64588	1.683715	16.67114	9.897341
RAB9B	0.954272	0.045728	1.490035	1.503516	31.11947	20.69444
CHST5	0.954296	0.045704	2.769696	3.856224	3.567625	0.917753
TGM7	0.954336	0.045664	1.489787	1.503516	31.11947	20.69444
CLCNKA	0.954399	0.045601	1.835229	1.923192	10.39168	5.39855
SNPH	0.954436	0.045564	1.375136	1.37622	271.1839	197.0471
CASP3	0.954438	0.045562	1.413934	1.414292	948.0324	670.3199
TAF3	0.95444	0.04556	0.722828	0.722384	256.7356	355.4045
VPS33B	0.954445	0.045555	0.726993	0.726346	174.4913	240.2355
ACVR2B-A	0.954452	0.045548	0.663124	0.656336	18.89396	28.79227
PHF3	0.954472	0.045528	0.68708	0.686995	1463.726	2130.628
KIAA0513	0.954477	0.045523	1.42662	1.426898	1270.13	890.1309
SEC23IP	0.954522	0.045478	1.458635	1.458791	2443.99	1675.35
CCDC58	0.954539	0.045461	0.709226	0.709015	569.0417	802.5844
EPC1	0.95454	0.04546	0.718555	0.718218	341.2027	475.0724
FARP2	0.954575	0.045425	0.72897	0.728206	150.0403	206.0447
CHP2	0.954586	0.045414	2.000525	2.158436	7.779867	3.599033
FES	0.954586	0.045414	2.000525	2.158436	7.779867	3.599033
LY6G5C	0.954586	0.045414	2.000525	2.158436	7.779867	3.599033
MSS51	0.954586	0.045414	2.000525	2.158436	7.779867	3.599033
ST8SIA6	0.954586	0.045414	2.000525	2.158436	7.779867	3.599033
LIX1	0.954592	0.045408	0.725464	0.724959	228.9504	315.8152
PSRC1	0.954598	0.045402	1.397369	1.397886	611.2753	437.2825
FLJ20021	0.954619	0.045381	0.695651	0.691798	31.11947	44.98792
SLC11A1	0.954627	0.045373	0.648868	0.640638	15.55973	24.29347
ZNF500	0.954648	0.045352	0.728175	0.727086	103.3611	142.1618
KLHDC3	0.95465	0.04535	0.687617	0.687531	1448.167	2106.334
LOC93622	0.954667	0.045333	1.369491	1.370869	213.3906	155.6582
TAF6	0.954749	0.045251	0.677182	0.677126	2238.379	3305.712
RAB11FIP	0.954756	0.045244	0.699988	0.699848	870.2337	1243.466
ZFYVE16	0.954812	0.045188	0.700629	0.700486	850.2283	1213.774

PROM2	0.954826	0.045174	1.377036	1.377914	343.4256	249.2331
C12orf66	0.954832	0.045168	0.726249	0.725708	208.945	287.9227
RHEBL1	0.954834	0.045166	1.369524	1.371366	156.7088	114.2693
DIMT1	0.954851	0.045149	0.7111	0.710873	516.8055	727.0047
TIAM2	0.954886	0.045114	0.725516	0.725035	240.0645	331.1111
SERTM1	0.954894	0.045106	1.423095	1.430142	48.90202	34.19082
CGB8	0.954932	0.045068	0.235603	0.00529	0	1.880495
ZNF292	0.954959	0.045041	0.698644	0.698511	906.9102	1298.351
NFATC1	0.95498	0.04502	1.3693	1.371281	145.5947	106.1715
PRICKLE2	0.954987	0.045013	1.89383	2.005933	8.980189	4.471799
ANXA2P2	0.954994	0.045006	2.722917	3.723098	3.712108	0.989734
NEK11	0.955001	0.044999	1.385752	1.389568	80.02149	57.58453
PLP2	0.955001	0.044999	1.471696	1.471817	3221.976	2189.112
C17orf107	0.955024	0.044976	0.729626	0.728665	116.698	160.157
RAET1K	0.955034	0.044966	1.631135	1.667062	16.67114	9.996315
C6orf47	0.955054	0.044946	0.712879	0.712638	483.4632	678.4178
ZBTB25	0.955062	0.044938	0.728529	0.727825	157.8202	216.8418
ZSCAN22	0.955094	0.044906	0.720564	0.72021	324.4649	450.518
MUC4	0.955118	0.044882	0.729205	0.72807	98.91545	135.8635
CGNL1	0.955131	0.044869	1.376572	1.377458	333.434	242.062
FAM179B	0.955185	0.044815	0.729464	0.728747	156.7088	215.0422
TMPO-AS	0.955186	0.044814	0.719015	0.716778	52.23625	72.88042
TNFSF13I	0.9552	0.0448	1.997957	2.158436	7.779867	3.599033
FAM107A	0.955202	0.044798	1.373661	1.374599	317.8631	231.2379
FAM198B	0.955233	0.044767	0.730236	0.729328	123.3665	169.1546
ZNF566	0.955241	0.044759	0.730362	0.72964	158.2203	216.8507
MED14	0.955254	0.044746	1.478381	1.478487	3816.58	2581.407
HAL	0.955281	0.044719	0.684459	0.679466	24.45101	35.99033
AVEN	0.955299	0.044701	1.384981	1.385624	481.2403	347.3067
DFFB	0.955309	0.044691	0.730061	0.728836	93.20281	127.8826
DNAJC16	0.955325	0.044675	0.70689	0.70671	652.3974	923.152
C22orf39	0.955331	0.044669	0.720702	0.720356	331.2001	459.7765
ADIPOR1	0.955346	0.044654	1.498435	1.498509	5724.871	3820.374
GRAMD1A	0.955426	0.044574	1.471552	1.47167	3362.014	2284.486
DYRK1B	0.955444	0.044556	0.728419	0.72784	194.4967	267.2282
FAM65A	0.955483	0.044517	1.439988	1.440193	1764.918	1225.471
DEXI	0.955567	0.044433	1.381078	1.381772	440.1182	318.5144
CEBPG	0.955602	0.044398	0.664378	0.664345	4005.52	6029.28
STX2	0.955645	0.044355	0.730254	0.728924	85.78971	117.6974
SPAST	0.955661	0.044339	0.713058	0.712832	525.4189	737.091
SYDE2	0.955662	0.044338	0.716468	0.716205	446.7867	623.8294
GPC1	0.95567	0.04433	1.431432	1.431668	1490.4	1041.02
LRRC26	0.955681	0.044319	0.52471	0.494654	4.445638	8.997583
LY86-AS1	0.955681	0.044319	0.52471	0.494654	4.445638	8.997583
SFR1	0.955682	0.044318	0.73143	0.730511	121.5882	166.4463
FH	0.955699	0.044301	0.669973	0.669932	3109.724	4641.853
CASD1	0.9557	0.0443	0.708559	0.708371	624.6122	881.7631
CLTCL1	0.955738	0.044262	1.367481	1.368594	262.2927	191.6485
ADIPOR2	0.955744	0.044256	1.462964	1.463098	2882.996	1970.471
FOXM1	0.955766	0.044234	0.693352	0.69325	1196.988	1726.636
RNF217	0.955769	0.044231	1.366782	1.36803	228.9504	167.355
SNX15	0.955786	0.044214	0.715561	0.715311	472.4046	660.4226
RNASEH1	0.955806	0.044194	0.715118	0.714869	465.6806	651.425
PRKAB1	0.955855	0.044145	1.403181	1.403579	824.6659	587.5422
LOC38964	0.955907	0.044093	0.726813	0.725203	69.16302	95.37438
PRDM5	0.955925	0.044075	0.698933	0.694888	30.00806	43.1884

ANXA8	0.955932	0.044068	2.108841	2.341968	6.335035	2.699275
NDST2	0.955936	0.044064	0.726312	0.725865	253.4014	349.1062
C9orf92	0.955958	0.044042	0.241229	0.005526	0	1.799517
ENPP3	0.955958	0.044042	0.241229	0.005526	0	1.799517
HNF1A	0.955958	0.044042	0.241229	0.005526	0	1.799517
LINC0038	0.955958	0.044042	0.241229	0.005526	0	1.799517
PDZD4	0.955958	0.044042	0.241229	0.005526	0	1.799517
PIRT	0.955958	0.044042	0.241229	0.005526	0	1.799517
TMEM119	0.955958	0.044042	0.241229	0.005526	0	1.799517
TMEM190	0.955958	0.044042	0.241229	0.005526	0	1.799517
C15orf52	0.956005	0.043995	1.440471	1.440666	1847.163	1282.156
FAM74A7	0.956056	0.043944	0.449649	0.395474	2.27839	5.776448
THRA	0.956065	0.043935	1.372083	1.373009	310.0833	225.8393
FAM221A	0.956078	0.043922	1.363587	1.365842	127.8121	93.57486
NUTM2A	0.956083	0.043917	0.716654	0.714078	44.73424	62.65017
FAM212B-	0.956102	0.043898	0.665963	0.658916	17.78255	26.99275
CCDC28A	0.956103	0.043897	0.73214	0.731462	167.8228	229.4384
CCDC71L	0.956122	0.043878	1.42188	1.422153	1251.447	879.9636
C5orf51	0.956169	0.043831	0.686684	0.686608	1614.878	2351.968
PCDH11Y	0.956196	0.043804	1.767718	1.840354	11.06964	6.010385
PDCD4-A	0.956196	0.043804	1.562282	1.587688	20.00537	12.59662
FST	0.956212	0.043788	1.36954	1.372428	100.0269	72.88042
NCR3LG1	0.956212	0.043788	1.36954	1.372428	100.0269	72.88042
SDCCAG8	0.956222	0.043778	0.708049	0.707872	666.8457	942.0469
ATMIN	0.956223	0.043777	0.693139	0.693042	1245.89	1797.717
LOC25489	0.956227	0.043773	2.102062	2.333763	6.312806	2.699275
POPDC3	0.956227	0.043773	1.374946	1.378386	85.57854	62.08332
LOC10272	0.956238	0.043762	0.525243	0.494654	4.445638	8.997583
NIM1K	0.956238	0.043762	0.525243	0.494654	4.445638	8.997583
RASGEF1	0.956238	0.043762	0.525243	0.494654	4.445638	8.997583
BAG2	0.956259	0.043741	0.701671	0.701536	878.0136	1251.564
MAP3K14-	0.956263	0.043737	0.721519	0.719288	51.12484	71.08091
FTH1P3	0.956292	0.043708	0.731099	0.730485	182.2489	249.494
ZKSCAN4	0.956293	0.043707	0.728927	0.728421	222.1263	304.9461
PTMS	0.956306	0.043694	0.688514	0.688433	1512.628	2197.21
CD99	0.956337	0.043663	1.445403	1.445578	2092.784	1447.711
LINC0088	0.956355	0.043645	1.362626	1.364034	200.0537	146.6606
ATG4D	0.956368	0.043632	0.701593	0.701459	888.0163	1265.96
GATSL2	0.956375	0.043625	1.391231	1.391733	617.5881	443.7518
TMEM94	0.956384	0.043616	0.702474	0.702335	856.8968	1220.072
CDK5RAP	0.956388	0.043612	0.69395	0.69385	1193.654	1720.338
FAM133C	0.956396	0.043604	0.240889	0.005445	0	1.826509
MAATS1	0.956401	0.043599	1.561824	1.587688	20.00537	12.59662
LOC1026C	0.956424	0.043576	0.728322	0.726636	66.68457	91.77535
POLR2G	0.956455	0.043545	0.686624	0.686549	1649.332	2402.355
TMEM150	0.956471	0.043529	0.730473	0.729919	202.2765	277.1256
SLC18B1	0.956474	0.043526	0.72165	0.721326	347.8712	482.2705
PHLPP1	0.956476	0.043524	1.36118	1.363378	130.0349	95.37438
RBM3	0.956494	0.043506	0.702394	0.702257	866.9106	1234.468
PLCD3	0.956533	0.043467	0.677458	0.677406	2405.09	3550.446
TAZ	0.956552	0.043448	0.719267	0.718992	425.6699	592.041
DFFA	0.956604	0.043396	0.685859	0.685788	1765.185	2573.957
SDHAP2	0.956624	0.043376	1.365572	1.366629	269.4168	197.137
RPEL1	0.956636	0.043364	0.241793	0.005472	0	1.817512
AP4E1	0.956643	0.043357	0.721088	0.720784	376.7678	522.7236
DDTL	0.956645	0.043355	0.732653	0.73137	86.83443	118.7321

FANCB	0.956649	0.043351	1.374748	1.375497	392.3276	285.2234
TACC3	0.956667	0.043333	1.464579	1.464701	3241.982	2213.405
CDR2	0.956677	0.043323	1.462517	1.462645	3030.814	2072.143
CHN1	0.956681	0.043319	1.371046	1.374354	87.80136	63.88284
ZNF580	0.95671	0.04329	0.731132	0.730603	215.6135	295.1207
AGAP2	0.956739	0.043261	1.864935	1.974205	8.891277	4.498792
FCRLA	0.956739	0.043261	1.864935	1.974205	8.891277	4.498792
LBX2	0.956739	0.043261	1.864935	1.974205	8.891277	4.498792
LINC0112	0.956739	0.043261	1.864935	1.974205	8.891277	4.498792
LOC10028	0.956739	0.043261	1.864935	1.974205	8.891277	4.498792
NCAM2	0.956739	0.043261	1.864935	1.974205	8.891277	4.498792
RCSD1	0.956739	0.043261	1.864935	1.974205	8.891277	4.498792
RGS11	0.956754	0.043246	1.541377	1.564208	21.11678	13.49637
DHX30	0.95676	0.04324	0.701673	0.701544	939.2522	1338.84
CCNC	0.956852	0.043148	1.470183	1.470294	3573.182	2430.247
LOC10050	0.956881	0.043119	0.629548	0.617828	11.1141	17.99517
LOC15368	0.956881	0.043119	0.629548	0.617828	11.1141	17.99517
PCDHGB6	0.956881	0.043119	0.629548	0.617828	11.1141	17.99517
FOLH1B	0.956883	0.043117	3.837936	165.4886	1.644886	0
HMCN1	0.956884	0.043116	0.717019	0.714168	41.12215	57.58453
GS1-594A	0.956916	0.043084	0.593882	0.576755	7.779867	13.49637
TBX15	0.956916	0.043084	0.593882	0.576755	7.779867	13.49637
GUCA1B	0.956934	0.043066	1.431195	1.44046	38.89934	27.00175
PXN	0.95698	0.04302	1.473658	1.47376	3962.175	2688.478
MCM3AP	0.956983	0.043017	0.683129	0.683066	1966.084	2878.327
KIAA0196	0.957016	0.042984	0.702806	0.702674	913.5787	1300.151
CASP4	0.957049	0.042951	1.430901	1.431115	1672.671	1168.786
RNF170	0.957086	0.042914	0.725151	0.724796	319.1746	440.3687
ZNF708	0.957104	0.042896	1.356767	1.3587	145.5057	107.0892
ACTA1	0.957111	0.042889	0.243622	0.005526	0	1.799517
ACTN3	0.957111	0.042889	0.243622	0.005526	0	1.799517
ADGRD1	0.957111	0.042889	0.243622	0.005526	0	1.799517
AGBL4	0.957111	0.042889	0.243622	0.005526	0	1.799517
ALG1L2	0.957111	0.042889	0.243622	0.005526	0	1.799517
APOA1-AS	0.957111	0.042889	0.243622	0.005526	0	1.799517
ASB18	0.957111	0.042889	0.243622	0.005526	0	1.799517
B3GALT5	0.957111	0.042889	0.243622	0.005526	0	1.799517
BAGE	0.957111	0.042889	0.243622	0.005526	0	1.799517
BEST3	0.957111	0.042889	0.243622	0.005526	0	1.799517
BPIFB4	0.957111	0.042889	0.243622	0.005526	0	1.799517
BTNL3	0.957111	0.042889	0.243622	0.005526	0	1.799517
C1QTNF1	0.957111	0.042889	0.243622	0.005526	0	1.799517
C1orf158	0.957111	0.042889	0.243622	0.005526	0	1.799517
C2orf73	0.957111	0.042889	0.243622	0.005526	0	1.799517
C6orf25	0.957111	0.042889	0.243622	0.005526	0	1.799517
C9	0.957111	0.042889	0.243622	0.005526	0	1.799517
C9orf139	0.957111	0.042889	0.243622	0.005526	0	1.799517
C9orf41-A	0.957111	0.042889	0.243622	0.005526	0	1.799517
CACNA2D	0.957111	0.042889	0.243622	0.005526	0	1.799517
CELA1	0.957111	0.042889	0.243622	0.005526	0	1.799517
CNGA3	0.957111	0.042889	0.243622	0.005526	0	1.799517
COL9A1	0.957111	0.042889	0.243622	0.005526	0	1.799517
CRMP1	0.957111	0.042889	0.243622	0.005526	0	1.799517
CTAGE10	0.957111	0.042889	0.243622	0.005526	0	1.799517
DBIL5P2	0.957111	0.042889	0.243622	0.005526	0	1.799517
DCAF8L1	0.957111	0.042889	0.243622	0.005526	0	1.799517

DCAF8L2	0.957111	0.042889	0.243622	0.005526	0	1.799517
DDX39B-A	0.957111	0.042889	0.243622	0.005526	0	1.799517
DNAH8	0.957111	0.042889	0.243622	0.005526	0	1.799517
EIF1B-AS1	0.957111	0.042889	0.243622	0.005526	0	1.799517
ERMN	0.957111	0.042889	0.243622	0.005526	0	1.799517
ERVH48-1	0.957111	0.042889	0.243622	0.005526	0	1.799517
ESRRB	0.957111	0.042889	0.243622	0.005526	0	1.799517
FAM110D	0.957111	0.042889	0.243622	0.005526	0	1.799517
FAM181A-	0.957111	0.042889	0.243622	0.005526	0	1.799517
FAM209B	0.957111	0.042889	0.243622	0.005526	0	1.799517
FAM83E	0.957111	0.042889	0.243622	0.005526	0	1.799517
FAM92A1I	0.957111	0.042889	0.243622	0.005526	0	1.799517
FCER2	0.957111	0.042889	0.243622	0.005526	0	1.799517
FCGR2C	0.957111	0.042889	0.243622	0.005526	0	1.799517
FGB	0.957111	0.042889	0.243622	0.005526	0	1.799517
FGD2	0.957111	0.042889	0.243622	0.005526	0	1.799517
FOXE3	0.957111	0.042889	0.243622	0.005526	0	1.799517
FSTL4	0.957111	0.042889	0.243622	0.005526	0	1.799517
GABRB2	0.957111	0.042889	0.243622	0.005526	0	1.799517
GAL3ST2	0.957111	0.042889	0.243622	0.005526	0	1.799517
GAL3ST3	0.957111	0.042889	0.243622	0.005526	0	1.799517
GCK	0.957111	0.042889	0.243622	0.005526	0	1.799517
GFRA2	0.957111	0.042889	0.243622	0.005526	0	1.799517
HIST1H2A	0.957111	0.042889	0.243622	0.005526	0	1.799517
HP09053	0.957111	0.042889	0.243622	0.005526	0	1.799517
IHH	0.957111	0.042889	0.243622	0.005526	0	1.799517
KCNMB2	0.957111	0.042889	0.243622	0.005526	0	1.799517
KLHL10	0.957111	0.042889	0.243622	0.005526	0	1.799517
KLHL32	0.957111	0.042889	0.243622	0.005526	0	1.799517
KNG1	0.957111	0.042889	0.243622	0.005526	0	1.799517
LEF1	0.957111	0.042889	0.243622	0.005526	0	1.799517
LHFPL3-A	0.957111	0.042889	0.243622	0.005526	0	1.799517
LINC0093	0.957111	0.042889	0.243622	0.005526	0	1.799517
LINC0114	0.957111	0.042889	0.243622	0.005526	0	1.799517
LINC0128	0.957111	0.042889	0.243622	0.005526	0	1.799517
LMAN1L	0.957111	0.042889	0.243622	0.005526	0	1.799517
LMOD2	0.957111	0.042889	0.243622	0.005526	0	1.799517
LOC10013	0.957111	0.042889	0.243622	0.005526	0	1.799517
LOC10050	0.957111	0.042889	0.243622	0.005526	0	1.799517
LOC10106	0.957111	0.042889	0.243622	0.005526	0	1.799517
LOC10192	0.957111	0.042889	0.243622	0.005526	0	1.799517
LOC10192	0.957111	0.042889	0.243622	0.005526	0	1.799517
LOC10192	0.957111	0.042889	0.243622	0.005526	0	1.799517
LOC10192	0.957111	0.042889	0.243622	0.005526	0	1.799517
LOC10192	0.957111	0.042889	0.243622	0.005526	0	1.799517
LOC10192	0.957111	0.042889	0.243622	0.005526	0	1.799517
LOC10192	0.957111	0.042889	0.243622	0.005526	0	1.799517
LOC10192	0.957111	0.042889	0.243622	0.005526	0	1.799517
LOC10192	0.957111	0.042889	0.243622	0.005526	0	1.799517
LOC10192	0.957111	0.042889	0.243622	0.005526	0	1.799517
LOC22194	0.957111	0.042889	0.243622	0.005526	0	1.799517
LOC28329	0.957111	0.042889	0.243622	0.005526	0	1.799517
LOC28358	0.957111	0.042889	0.243622	0.005526	0	1.799517
LOC28400	0.957111	0.042889	0.243622	0.005526	0	1.799517
LOC72817	0.957111	0.042889	0.243622	0.005526	0	1.799517
LRGUK	0.957111	0.042889	0.243622	0.005526	0	1.799517

MEIG1	0.957111	0.042889	0.243622	0.005526	0	1.799517
MLNR	0.957111	0.042889	0.243622	0.005526	0	1.799517
MMP16	0.957111	0.042889	0.243622	0.005526	0	1.799517
MRGPRF	0.957111	0.042889	0.243622	0.005526	0	1.799517
NCF1C	0.957111	0.042889	0.243622	0.005526	0	1.799517
PDE3B	0.957111	0.042889	0.243622	0.005526	0	1.799517
PLA2G4D	0.957111	0.042889	0.243622	0.005526	0	1.799517
PNMT	0.957111	0.042889	0.243622	0.005526	0	1.799517
POM121L	0.957111	0.042889	0.243622	0.005526	0	1.799517
PPP1R1B	0.957111	0.042889	0.243622	0.005526	0	1.799517
PRSS42	0.957111	0.042889	0.243622	0.005526	0	1.799517
PTPN5	0.957111	0.042889	0.243622	0.005526	0	1.799517
RANBP3L	0.957111	0.042889	0.243622	0.005526	0	1.799517
RGMB-AS	0.957111	0.042889	0.243622	0.005526	0	1.799517
RNF225	0.957111	0.042889	0.243622	0.005526	0	1.799517
SELV	0.957111	0.042889	0.243622	0.005526	0	1.799517
SEMA3B-A	0.957111	0.042889	0.243622	0.005526	0	1.799517
SFTPD	0.957111	0.042889	0.243622	0.005526	0	1.799517
SLC22A1	0.957111	0.042889	0.243622	0.005526	0	1.799517
SLC4A4	0.957111	0.042889	0.243622	0.005526	0	1.799517
SNX29P2	0.957111	0.042889	0.243622	0.005526	0	1.799517
SPATA3-A	0.957111	0.042889	0.243622	0.005526	0	1.799517
SSX6	0.957111	0.042889	0.243622	0.005526	0	1.799517
STRA8	0.957111	0.042889	0.243622	0.005526	0	1.799517
TPRG1-AS	0.957111	0.042889	0.243622	0.005526	0	1.799517
TPTE	0.957111	0.042889	0.243622	0.005526	0	1.799517
TTC39A-A	0.957111	0.042889	0.243622	0.005526	0	1.799517
UNC5D	0.957111	0.042889	0.243622	0.005526	0	1.799517
WNT16	0.957111	0.042889	0.243622	0.005526	0	1.799517
LINC0026	0.957139	0.042861	1.362726	1.365615	99.49339	72.85343
CUL9	0.957142	0.042858	0.714243	0.714034	567.9303	795.3863
MRRF	0.95716	0.04284	0.722896	0.722583	363.4309	502.9649
SUCLG2	0.957194	0.042806	1.441933	1.442107	2122.792	1472.005
MIR762HG	0.957205	0.042795	1.42573	1.434306	40.01074	27.89251
DRD5P2	0.957219	0.042781	2.169194	2.463954	5.557048	2.249396
LOC10106	0.957219	0.042781	2.169194	2.463954	5.557048	2.249396
PRR14	0.957234	0.042766	0.704524	0.704383	839.1142	1191.28
RCOR2	0.957251	0.042749	1.522983	1.543661	22.22819	14.39613
ADGRG2	0.957258	0.042742	1.863107	1.974205	8.891277	4.498792
IFFO1	0.957258	0.042742	1.863107	1.974205	8.891277	4.498792
YTHDC2	0.957284	0.042716	0.714394	0.714187	571.2645	799.8851
LOC28629	0.957309	0.042691	2.67567	3.724819	3.378685	0.899758
ZNF775	0.957327	0.042673	0.736801	0.735898	124.4779	169.1546
DCXR	0.95733	0.04267	0.698195	0.698086	1128.081	1615.966
FAAH2	0.957334	0.042666	1.358844	1.36012	217.8363	160.157
MAB21L3	0.957342	0.042658	0.733886	0.733268	179.915	245.3641
NEURL3	0.957353	0.042647	1.37304	1.37691	75.57585	54.88526
ALDH16A1	0.957359	0.042641	0.728821	0.728399	266.7383	366.2016
LRRC8E	0.957383	0.042617	1.420552	1.420807	1335.914	940.2474
C7orf69	0.957403	0.042597	2.654007	3.675953	3.334229	0.899758
KCCAT19	0.957403	0.042597	2.654007	3.675953	3.334229	0.899758
SPTY2D1-	0.957403	0.042597	2.654007	3.675953	3.334229	0.899758
TMEM92-A	0.957403	0.042597	2.654007	3.675953	3.334229	0.899758
UBASH3A	0.957403	0.042597	2.654007	3.675953	3.334229	0.899758
GOLGA8S	0.957405	0.042595	2.538889	3.290235	3.901048	1.178683
OSTF1	0.957407	0.042593	1.407584	1.407909	1005.826	714.4081

LOC10192	0.95741	0.04259	0.735573	0.73455	106.762	145.347
BRCA1	0.957456	0.042544	0.714094	0.713889	567.9303	795.5483
TP53BP2	0.957485	0.042515	0.685246	0.685181	1916.07	2796.449
FAM47E	0.957546	0.042454	1.704785	1.763483	11.89208	6.73919
PNPLA3	0.957607	0.042393	0.734185	0.732717	75.57585	103.1483
ST3GAL2	0.957618	0.042382	1.381602	1.382144	564.5961	408.4903
C19orf35	0.957638	0.042362	0.392296	0.30567	1.211436	3.985929
RMND5B	0.957663	0.042337	0.703737	0.703604	895.7961	1273.158
SPACA6	0.957672	0.042328	1.356688	1.358009	208.945	153.8587
LPP-AS2	0.957677	0.042323	0.712543	0.709174	34.4537	48.58695
FGF19	0.957697	0.042303	1.506675	1.52553	23.3396	15.29589
GOLGA2F	0.957715	0.042285	0.721691	0.719062	43.34497	60.28381
LRFN3	0.957728	0.042272	0.737004	0.736163	131.1463	178.1521
CELSR2	0.957769	0.042231	1.444488	1.44465	2261.718	1565.579
WNT3	0.957784	0.042216	1.368709	1.372413	77.79867	56.68477
SPATA2	0.957795	0.042205	0.711338	0.711161	659.0659	926.7511
DNM1	0.957808	0.042192	1.361466	1.362448	285.6323	209.6437
ZCCHC4	0.957811	0.042189	0.734083	0.733549	212.0014	289.0114
ZNF717	0.957814	0.042186	1.354252	1.355938	162.2658	119.6679
SLC16A13	0.957841	0.042159	0.730333	0.729917	274.5182	376.099
CT45A7	0.957843	0.042157	3.78041	162.1544	1.611544	0
FDX1	0.95785	0.04215	0.717309	0.71708	496.8001	692.8139
KIF9-AS1	0.957858	0.042142	0.669338	0.661865	16.67114	25.19323
CCDC181	0.957867	0.042133	0.503214	0.463956	3.334229	7.198066
EGR3	0.957867	0.042133	0.503214	0.463956	3.334229	7.198066
ENTPD3-A	0.957867	0.042133	0.503214	0.463956	3.334229	7.198066
LOC10272	0.957867	0.042133	0.503214	0.463956	3.334229	7.198066
NPAS3	0.957867	0.042133	0.503214	0.463956	3.334229	7.198066
EHD4	0.957875	0.042125	1.423547	1.423779	1477.063	1037.421
ENSA	0.957875	0.042125	0.666345	0.666314	4121.107	6184.939
USP1	0.957882	0.042118	0.671	0.670963	3340.897	4979.262
C8orf44	0.957902	0.042098	1.44433	1.45573	32.91995	22.61093
PRPF19	0.957914	0.042086	0.675774	0.67573	2881.885	4264.854
C18orf61	0.957917	0.042083	2.653131	3.68817	3.345343	0.899758
SLC6A9	0.95792	0.04208	0.700816	0.700702	1059.173	1511.594
HOMER2	0.957967	0.042033	1.405601	1.405923	1006.937	716.2076
LOC72867	0.957996	0.042004	0.543601	0.51511	4.690148	9.114552
GPATCH2	0.958003	0.041997	1.428382	1.428593	1650.443	1155.29
HAUS7	0.95804	0.04196	1.374739	1.375365	469.0148	341.0084
CTNS	0.958046	0.041954	0.725711	0.725385	347.8712	479.5712
ELOVL7	0.958084	0.041916	0.704613	0.70448	892.4619	1266.842
PPP2CB	0.958115	0.041885	1.464301	1.464413	3444.258	2351.968
ANKDD1B	0.958118	0.041882	2.645619	3.675953	3.334229	0.899758
APOBEC3	0.958118	0.041882	2.645619	3.675953	3.334229	0.899758
BOK-AS1	0.958118	0.041882	2.645619	3.675953	3.334229	0.899758
CBLN3	0.958118	0.041882	2.645619	3.675953	3.334229	0.899758
CDYL2	0.958118	0.041882	2.645619	3.675953	3.334229	0.899758
CLEC4A	0.958118	0.041882	2.645619	3.675953	3.334229	0.899758
DKFZP434	0.958118	0.041882	2.645619	3.675953	3.334229	0.899758
ETNPPL	0.958118	0.041882	2.645619	3.675953	3.334229	0.899758
FAM180A	0.958118	0.041882	2.645619	3.675953	3.334229	0.899758
FLJ26850	0.958118	0.041882	2.645619	3.675953	3.334229	0.899758
GABRA3	0.958118	0.041882	2.645619	3.675953	3.334229	0.899758
GAPLINC	0.958118	0.041882	2.645619	3.675953	3.334229	0.899758
HULC	0.958118	0.041882	2.645619	3.675953	3.334229	0.899758
IGSF1	0.958118	0.041882	2.645619	3.675953	3.334229	0.899758

INSC	0.958118	0.041882	2.645619	3.675953	3.334229	0.899758
KCNH6	0.958118	0.041882	2.645619	3.675953	3.334229	0.899758
KCTD4	0.958118	0.041882	2.645619	3.675953	3.334229	0.899758
KY	0.958118	0.041882	2.645619	3.675953	3.334229	0.899758
LINC0021	0.958118	0.041882	2.645619	3.675953	3.334229	0.899758
LINC0028	0.958118	0.041882	2.645619	3.675953	3.334229	0.899758
LINC0048	0.958118	0.041882	2.645619	3.675953	3.334229	0.899758
LINC0094	0.958118	0.041882	2.645619	3.675953	3.334229	0.899758
LINC0113	0.958118	0.041882	2.645619	3.675953	3.334229	0.899758
LINC0133	0.958118	0.041882	2.645619	3.675953	3.334229	0.899758
LOC10013	0.958118	0.041882	2.645619	3.675953	3.334229	0.899758
LOC40004	0.958118	0.041882	2.645619	3.675953	3.334229	0.899758
LRIT3	0.958118	0.041882	2.645619	3.675953	3.334229	0.899758
MARCO	0.958118	0.041882	2.645619	3.675953	3.334229	0.899758
MEDAG	0.958118	0.041882	2.645619	3.675953	3.334229	0.899758
MMP12	0.958118	0.041882	2.645619	3.675953	3.334229	0.899758
MYBPH	0.958118	0.041882	2.645619	3.675953	3.334229	0.899758
NTNG2	0.958118	0.041882	2.645619	3.675953	3.334229	0.899758
PLXDC2	0.958118	0.041882	2.645619	3.675953	3.334229	0.899758
SCN3A	0.958118	0.041882	2.645619	3.675953	3.334229	0.899758
XKRX	0.958118	0.041882	2.645619	3.675953	3.334229	0.899758
VCL	0.958171	0.041829	1.559614	1.559636	22671.64	14536.5
PRKAR1A	0.958176	0.041824	0.669151	0.669118	3739.293	5588.399
HIST1H2A	0.958182	0.041818	0.539951	0.510399	4.601236	9.024576
COL6A3	0.958216	0.041784	1.77016	1.85127	10.00269	5.39855
FMOD	0.958216	0.041784	1.77016	1.85127	10.00269	5.39855
GPR135	0.958216	0.041784	1.77016	1.85127	10.00269	5.39855
KC6	0.958216	0.041784	1.77016	1.85127	10.00269	5.39855
LINC0068	0.958216	0.041784	1.77016	1.85127	10.00269	5.39855
LOC64624	0.958216	0.041784	1.77016	1.85127	10.00269	5.39855
NUDT9P1	0.958216	0.041784	1.77016	1.85127	10.00269	5.39855
TERT	0.958216	0.041784	1.77016	1.85127	10.00269	5.39855
ZNF528-A	0.958216	0.041784	1.77016	1.85127	10.00269	5.39855
HIRIP3	0.958227	0.041773	0.723553	0.723265	395.6618	547.053
FURIN	0.958271	0.041729	1.425811	1.426027	1594.873	1118.4
BAGE5	0.958279	0.041721	0.248317	0.005667	0	1.754529
LINC0066	0.958282	0.041718	0.72606	0.723536	45.56779	62.98308
OR2A7	0.958292	0.041708	0.706154	0.702101	28.60768	40.75005
NUDT17	0.958305	0.041695	0.691755	0.686336	22.22819	32.3913
TARSL2	0.958316	0.041684	1.350523	1.352051	180.0484	133.1642
HOXC5	0.958357	0.041643	1.955042	2.117521	7.079679	3.338103
PGAP2	0.958382	0.041618	0.723495	0.723213	403.4417	557.8501
CEACAM5	0.958394	0.041606	2.629625	3.639954	3.334229	0.908756
PCDHB8	0.958417	0.041583	1.476745	1.492341	25.97364	17.40133
DENND2C	0.958421	0.041579	0.718317	0.715189	36.67652	51.28622
MGA	0.958425	0.041575	0.704178	0.70405	932.4726	1324.444
ANKRD60	0.958435	0.041565	0.246099	0.005499	0	1.808514
RFPL1S	0.958435	0.041565	0.246099	0.005499	0	1.808514
TAF1L	0.958435	0.041565	0.246099	0.005499	0	1.808514
C22orf46	0.958465	0.041535	0.733359	0.732889	236.7302	323.0132
CEP55	0.958468	0.041532	1.467655	1.467758	3865.483	2633.593
CPEB1	0.958468	0.041532	1.479039	1.49499	25.56242	17.09541
HHEX	0.958475	0.041525	1.350794	1.352414	166.7114	123.2669
FTO-IT1	0.95849	0.04151	0.5039	0.463956	3.334229	7.198066
LEKR1	0.95849	0.04151	0.5039	0.463956	3.334229	7.198066
SYCE3	0.95849	0.04151	0.5039	0.463956	3.334229	7.198066

TFAMP1	0.95849	0.04151	0.5039	0.463956	3.334229	7.198066
RASA1	0.958492	0.041508	1.384326	1.384802	637.9491	460.6763
RPL23AP8	0.958529	0.041471	1.362192	1.363032	334.9455	245.733
MTCP1	0.958557	0.041443	0.731877	0.729858	55.1926	75.62469
GUCY2D	0.95858	0.04142	1.478747	1.49499	25.56242	17.09541
PPM1B	0.958582	0.041418	0.688748	0.688678	1744.913	2533.719
NBPF8	0.958582	0.041418	1.35564	1.356708	257.2802	189.6331
GLIS2	0.958593	0.041407	0.722407	0.72214	422.3356	584.8429
IQCG	0.958597	0.041403	0.739499	0.73867	132.2577	179.0519
TCEAL8	0.95861	0.04139	1.388386	1.388816	713.525	513.762
FAM207A	0.958611	0.041389	0.701802	0.701689	1071.399	1526.89
THAP11	0.958657	0.041343	0.726166	0.725863	380.1021	523.6593
SUPV3L1	0.958659	0.041341	0.697844	0.697747	1258.116	1803.116
PNPLA2	0.958659	0.041341	1.422159	1.422384	1520.408	1068.913
ADD3-AS1	0.958663	0.041337	0.247026	0.005526	0	1.799517
ANKRD20	0.958663	0.041337	0.247026	0.005526	0	1.799517
ARHGEF1	0.958663	0.041337	0.247026	0.005526	0	1.799517
BCAN	0.958663	0.041337	0.247026	0.005526	0	1.799517
BCL11B	0.958663	0.041337	0.247026	0.005526	0	1.799517
CATSPER	0.958663	0.041337	0.247026	0.005526	0	1.799517
CD180	0.958663	0.041337	0.247026	0.005526	0	1.799517
CD52	0.958663	0.041337	0.247026	0.005526	0	1.799517
COL3A1	0.958663	0.041337	0.247026	0.005526	0	1.799517
CYBB	0.958663	0.041337	0.247026	0.005526	0	1.799517
FAM196A	0.958663	0.041337	0.247026	0.005526	0	1.799517
FAM66B	0.958663	0.041337	0.247026	0.005526	0	1.799517
HS3ST5	0.958663	0.041337	0.247026	0.005526	0	1.799517
IBA57-AS1	0.958663	0.041337	0.247026	0.005526	0	1.799517
ITM2A	0.958663	0.041337	0.247026	0.005526	0	1.799517
LINC0044	0.958663	0.041337	0.247026	0.005526	0	1.799517
LINC0059	0.958663	0.041337	0.247026	0.005526	0	1.799517
LINC0063	0.958663	0.041337	0.247026	0.005526	0	1.799517
LINC0143	0.958663	0.041337	0.247026	0.005526	0	1.799517
LOC10050	0.958663	0.041337	0.247026	0.005526	0	1.799517
LOC10192	0.958663	0.041337	0.247026	0.005526	0	1.799517
LOC10192	0.958663	0.041337	0.247026	0.005526	0	1.799517
LOC44108	0.958663	0.041337	0.247026	0.005526	0	1.799517
LOC73115	0.958663	0.041337	0.247026	0.005526	0	1.799517
MMP21	0.958663	0.041337	0.247026	0.005526	0	1.799517
MOGAT3	0.958663	0.041337	0.247026	0.005526	0	1.799517
NACAP1	0.958663	0.041337	0.247026	0.005526	0	1.799517
RAD21-AS	0.958663	0.041337	0.247026	0.005526	0	1.799517
RNVU1-20	0.958663	0.041337	0.247026	0.005526	0	1.799517
RSPH6A	0.958663	0.041337	0.247026	0.005526	0	1.799517
SCARNA1	0.958663	0.041337	0.247026	0.005526	0	1.799517
SLC22A16	0.958663	0.041337	0.247026	0.005526	0	1.799517
SLC24A3	0.958663	0.041337	0.247026	0.005526	0	1.799517
SLC25A18	0.958663	0.041337	0.247026	0.005526	0	1.799517
SLC35G5	0.958663	0.041337	0.247026	0.005526	0	1.799517
SNORA64	0.958663	0.041337	0.247026	0.005526	0	1.799517
SNORA74	0.958663	0.041337	0.247026	0.005526	0	1.799517
SNORA92	0.958663	0.041337	0.247026	0.005526	0	1.799517
SOHLH2	0.958663	0.041337	0.247026	0.005526	0	1.799517
SOX11	0.958663	0.041337	0.247026	0.005526	0	1.799517
SPANXD	0.958663	0.041337	0.247026	0.005526	0	1.799517
SPDYE8P	0.958663	0.041337	0.247026	0.005526	0	1.799517

SRMS	0.958663	0.041337	0.247026	0.005526	0	1.799517
STAR	0.958663	0.041337	0.247026	0.005526	0	1.799517
STPG2	0.958663	0.041337	0.247026	0.005526	0	1.799517
THRSP	0.958663	0.041337	0.247026	0.005526	0	1.799517
TKTL1	0.958663	0.041337	0.247026	0.005526	0	1.799517
TMEM100	0.958663	0.041337	0.247026	0.005526	0	1.799517
TMEM105	0.958663	0.041337	0.247026	0.005526	0	1.799517
TMEM72-/	0.958663	0.041337	0.247026	0.005526	0	1.799517
TNNC2	0.958663	0.041337	0.247026	0.005526	0	1.799517
TRIM72	0.958663	0.041337	0.247026	0.005526	0	1.799517
TTBK1	0.958663	0.041337	0.247026	0.005526	0	1.799517
ZNF671	0.958663	0.041337	0.247026	0.005526	0	1.799517
MINOS1-N	0.958671	0.041329	1.34956	1.351338	151.3073	111.9659
ZNF414	0.958685	0.041315	0.737963	0.73732	171.1571	232.1376
ZNF267	0.958698	0.041302	1.381538	1.382033	606.8296	439.0821
CRIPT	0.958706	0.041294	1.387702	1.388135	708.1791	510.163
DPY19L1	0.958712	0.041288	1.394115	1.394495	825.2883	591.816
TMEM177	0.958741	0.041259	0.738595	0.737872	150.0403	203.3454
PRR29	0.958783	0.041217	0.630662	0.617852	10.00269	16.19565
PSMC3IP	0.958797	0.041203	1.359296	1.360181	314.5289	231.2379
SHKBP1	0.958802	0.041198	1.459126	1.459242	3337.563	2287.186
MRPS28	0.958805	0.041195	0.727776	0.727446	342.3142	470.5736
C1QTNF9	0.958855	0.041145	0.71615	0.712695	33.34229	46.78743
ADAMTS5	0.958874	0.041126	1.35128	1.352522	217.8363	161.0567
UPK3BL	0.958888	0.041112	0.716441	0.716243	582.6787	813.5255
ASPHD2	0.95893	0.04107	0.740711	0.739833	124.4779	168.2548
MRPL46	0.958962	0.041038	0.733769	0.733325	250.0672	341.0084
BMPR1B	0.958976	0.041024	0.630827	0.617852	10.00269	16.19565
PROCA1	0.958976	0.041024	0.630827	0.617852	10.00269	16.19565
KIAA0895I	0.959001	0.040999	0.740555	0.739357	91.13559	123.2669
MST1R	0.959023	0.040977	1.361774	1.362561	356.7625	261.8297
CC2D2A	0.959032	0.040968	0.738554	0.737904	166.7114	225.9293
BCL2L13	0.959034	0.040966	0.685635	0.685575	2034.991	2968.303
EPHX1	0.959047	0.040953	0.695449	0.695363	1421.493	2044.251
CSTA	0.959063	0.040937	1.456686	1.470265	27.78524	18.89492
DAND5	0.95909	0.04091	1.796883	1.89169	9.046874	4.777717
CA14	0.959099	0.040901	2.633742	3.675953	3.334229	0.899758
CERS6-AS	0.959099	0.040901	2.633742	3.675953	3.334229	0.899758
FAM19A2	0.959099	0.040901	2.633742	3.675953	3.334229	0.899758
HS3ST2	0.959099	0.040901	2.633742	3.675953	3.334229	0.899758
LOC10192	0.959099	0.040901	2.633742	3.675953	3.334229	0.899758
LOC10192	0.959099	0.040901	2.633742	3.675953	3.334229	0.899758
PRG2	0.959099	0.040901	2.633742	3.675953	3.334229	0.899758
PTGDR2	0.959099	0.040901	2.633742	3.675953	3.334229	0.899758
SLC10A2	0.959099	0.040901	2.633742	3.675953	3.334229	0.899758
SLC5A1	0.959099	0.040901	2.633742	3.675953	3.334229	0.899758
SLC6A3	0.959099	0.040901	2.633742	3.675953	3.334229	0.899758
TACR2	0.959099	0.040901	2.633742	3.675953	3.334229	0.899758
THRB-AS1	0.959099	0.040901	2.633742	3.675953	3.334229	0.899758
TRPC4	0.959099	0.040901	2.633742	3.675953	3.334229	0.899758
VSIG2	0.959099	0.040901	2.633742	3.675953	3.334229	0.899758
RNF183	0.959114	0.040886	1.387082	1.393482	48.90202	35.09057
ZNF615	0.959117	0.040883	0.738399	0.737832	197.8309	268.128
HPDL	0.959118	0.040882	1.45651	1.470265	27.78524	18.89492
METTL6	0.95914	0.04086	0.722093	0.721848	465.6806	645.1267
MRPS10	0.959167	0.040833	0.694177	0.694095	1507.071	2171.279

PSMB9	0.959179	0.040821	1.36664	1.367311	425.6699	311.3164
ZNF100	0.959185	0.040815	0.740192	0.738697	74.53113	100.8989
FSIP1	0.959206	0.040794	0.590403	0.570474	6.668457	11.69686
GPT	0.959206	0.040794	0.590403	0.570474	6.668457	11.69686
SNAPC4	0.959207	0.040793	1.36604	1.366701	439.0068	321.2137
CILP	0.959225	0.040775	0.722549	0.722301	460.1236	637.0289
STARD10	0.959226	0.040774	1.403237	1.403537	1093.627	779.1907
TMEM5	0.95923	0.04077	0.723106	0.722856	465.6806	644.2269
KLHL3	0.959239	0.040761	0.737854	0.736178	65.57317	89.07607
FBLL1	0.959251	0.040749	0.631064	0.617852	10.00269	16.19565
GBA3	0.959289	0.040711	1.700181	1.763404	11.1141	6.298308
RFPL3S	0.959289	0.040711	1.700181	1.763404	11.1141	6.298308
TTC6	0.959289	0.040711	1.700181	1.763404	11.1141	6.298308
G2E3	0.959298	0.040702	1.421154	1.421372	1561.53	1098.605
SLC26A3	0.95932	0.04068	0.46811	0.412831	2.222819	5.39855
XRCC6BP	0.959325	0.040675	0.742116	0.741156	113.3638	152.9589
MON1A	0.959342	0.040658	0.739062	0.738464	183.3826	248.3333
FAM189A	0.959358	0.040642	0.729172	0.726651	44.45638	61.18356
NPIPA5	0.959366	0.040634	0.73428	0.733863	271.4729	369.9266
SLC35A3	0.959367	0.040633	0.728988	0.728669	358.9853	492.6627
ZNF815P	0.959386	0.040614	0.742834	0.741664	94.60318	127.5587
FAM131C	0.959389	0.040611	1.38346	1.389527	50.01343	35.99033
NSUN3	0.959396	0.040604	0.742246	0.741158	100.0269	134.9637
CORT	0.959399	0.040601	3.684534	156.5973	1.555973	0
LHX9	0.959409	0.040591	1.446738	1.459587	28.89665	19.79468
SERINC5	0.959413	0.040587	0.726782	0.726489	385.6591	530.8574
HNF1B	0.959494	0.040506	1.359598	1.360356	374.545	275.326
GLRX	0.95953	0.04047	1.356472	1.35733	321.1974	236.6364
BBOX1-AS	0.959539	0.040461	1.502449	1.523048	21.15012	13.88327
KIAA1456	0.959554	0.040446	0.39109	0.297378	1.11141	3.76099
LINC0096	0.95956	0.04044	0.739952	0.73934	178.9369	242.026
GSAP	0.959572	0.040428	1.347533	1.350091	104.4725	77.37921
DLG1	0.959576	0.040424	1.454774	1.454893	3224.199	2216.105
SMIM8	0.959578	0.040422	0.743766	0.74272	105.5839	142.1618
LOC10013	0.9596	0.0404	0.590762	0.570474	6.668457	11.69686
SAMD15	0.9596	0.0404	0.590762	0.570474	6.668457	11.69686
ST7-OT4	0.9596	0.0404	0.590762	0.570474	6.668457	11.69686
DTNA	0.959615	0.040385	0.673221	0.665268	15.55973	23.39372
SLC25A25	0.95962	0.04038	0.735897	0.733793	53.46991	72.87143
SLC38A9	0.959626	0.040374	0.730572	0.730238	342.3142	468.7741
LINC0063	0.959647	0.040353	0.737487	0.73562	58.90471	80.07849
SETD2	0.959662	0.040338	0.691602	0.691529	1679.34	2428.448
LSMEM1	0.959679	0.040321	1.437792	1.449837	30.00806	20.69444
MRVI1	0.959682	0.040318	1.699088	1.763404	11.1141	6.298308
FZD2	0.959709	0.040291	1.358383	1.359168	353.4282	260.0302
RAPGEF5	0.959727	0.040273	0.742466	0.741162	83.35572	112.4698
SVBP	0.959728	0.040272	0.744041	0.742886	95.58122	128.6654
BLZF1	0.959729	0.040271	1.396437	1.396768	951.3666	681.117
HNRNPA3	0.959731	0.040269	0.468073	0.412146	2.222819	5.407547
ADPRHL1	0.959732	0.040268	0.728232	0.725502	41.12215	56.68477
MRPS11	0.959735	0.040265	0.728902	0.72859	361.2081	495.7668
ZDHHC20	0.959764	0.040236	1.450318	1.450446	2911.86	2007.559
INPP5E	0.959772	0.040228	0.733548	0.733158	285.6323	389.5953
ABCB1	0.95984	0.04016	0.468778	0.412831	2.222819	5.39855
C10orf131	0.95984	0.04016	0.468778	0.412831	2.222819	5.39855
C22orf23	0.95984	0.04016	0.468778	0.412831	2.222819	5.39855

C9orf50	0.95984	0.04016	0.468778	0.412831	2.222819	5.39855
FMO5	0.95984	0.04016	0.468778	0.412831	2.222819	5.39855
GNAT2	0.95984	0.04016	0.468778	0.412831	2.222819	5.39855
IQSEC3	0.95984	0.04016	0.468778	0.412831	2.222819	5.39855
LINC0114	0.95984	0.04016	0.468778	0.412831	2.222819	5.39855
LOC10013	0.95984	0.04016	0.468778	0.412831	2.222819	5.39855
LOC10192	0.95984	0.04016	0.468778	0.412831	2.222819	5.39855
PSORS1C	0.95984	0.04016	0.468778	0.412831	2.222819	5.39855
RPH3A	0.95984	0.04016	0.468778	0.412831	2.222819	5.39855
SEPT7-AS	0.95984	0.04016	0.468778	0.412831	2.222819	5.39855
UTAT33	0.959856	0.040144	1.647018	1.697474	12.22551	7.198066
POLI	0.959856	0.040144	0.741707	0.740223	73.35303	99.09938
ATF2	0.95986	0.04014	0.702276	0.70217	1121.412	1597.071
KRT86	0.959873	0.040127	1.352691	1.356095	80.02149	59.00615
ARMC2	0.959879	0.040121	0.69614	0.690378	21.11678	30.59178
RPSAP52	0.959881	0.040119	1.354874	1.358688	73.35303	53.9855
ZFP69B	0.9599	0.0401	1.373169	1.378765	53.34766	38.68961
LOC65371	0.959923	0.040077	0.473694	0.418996	2.256161	5.39855
HYKK	0.959933	0.040067	1.429573	1.440899	31.11947	21.5942
SPRY1	0.959935	0.040065	0.72735	0.727071	413.4444	568.6472
SAMD11	0.959946	0.040054	0.744897	0.743962	117.8094	158.3575
PCGF2	0.959974	0.040026	0.72383	0.723591	485.686	671.2197
TRIM6	0.959974	0.040026	1.46617	1.482121	24.66218	16.63653
PRCD	0.960028	0.039972	1.354944	1.358688	73.35303	53.9855
TOB2P1	0.960029	0.039971	0.720041	0.716497	32.23088	44.98792
TRAIP	0.960041	0.039959	0.73746	0.736995	236.7302	321.2137
RIMKLA	0.960049	0.039951	1.370356	1.375784	54.47018	39.58937
KIAA0430	0.960082	0.039918	1.410927	1.411175	1310.352	928.5506
VAMP8	0.960084	0.039916	1.421723	1.421927	1676.006	1178.683
DAGLA	0.960086	0.039914	0.732454	0.732094	305.6376	417.4879
C9orf91	0.96009	0.03991	0.725168	0.724914	446.7867	616.3344
CNTN5	0.960103	0.039897	1.646394	1.697474	12.22551	7.198066
LOC10013	0.960103	0.039897	1.646394	1.697474	12.22551	7.198066
LINC0034	0.960108	0.039892	0.742646	0.741165	73.35303	98.97341
CYB561	0.960119	0.039881	0.697463	0.697376	1379.804	1978.569
TMEM214	0.960141	0.039859	1.452075	1.452195	3176.409	2187.312
UROD	0.960254	0.039746	0.705203	0.705088	1030.277	1461.207
CCDC144	0.960259	0.039741	1.431523	1.443395	29.87469	20.69444
PIGV	0.960271	0.039729	0.731439	0.73111	340.0913	465.175
HERC2P9	0.960285	0.039715	0.744933	0.744071	125.3114	168.4168
SLFN5	0.960292	0.039708	0.688658	0.688596	2021.654	2935.911
CEP170	0.960309	0.039691	1.436192	1.436349	2283.435	1589.747
MPC1	0.960351	0.039649	0.70207	0.70197	1206.991	1719.438
WTH3DI	0.960359	0.039641	0.446389	0.378053	1.711571	4.543779
AKR1B1	0.960361	0.039639	1.519445	1.519483	12001	7898.078
ALPP	0.960375	0.039625	1.480546	1.499217	22.38379	14.92699
ZNF799	0.960383	0.039617	1.338637	1.340751	124.6557	92.97203
PPFIA3	0.960402	0.039598	1.394699	1.395023	970.2606	695.5132
INSL3	0.960409	0.039591	1.339674	1.341354	154.4859	115.1691
RRP36	0.960422	0.039578	0.708472	0.708346	945.8096	1335.241
XBP1	0.960424	0.039576	0.668953	0.668924	4455.641	6660.911
SLC7A6O	0.960436	0.039564	1.348866	1.349773	301.192	223.1401
BRINP3	0.960436	0.039564	0.40361	0.310723	1.11141	3.599033
LGALS4	0.960436	0.039564	0.40361	0.310723	1.11141	3.599033
BCYRN1	0.960451	0.039549	1.645501	1.697474	12.22551	7.198066
CMYA5	0.960451	0.039549	1.645501	1.697474	12.22551	7.198066

ERN2	0.960451	0.039549	1.645501	1.697474	12.22551	7.198066
TEX14	0.960451	0.039549	1.645501	1.697474	12.22551	7.198066
GPR157	0.960498	0.039502	0.736753	0.734502	48.90202	66.58211
VPS8	0.96053	0.03947	0.713328	0.713178	787.9894	1104.903
ZBTB24	0.960551	0.039449	0.728738	0.728456	399.3406	548.2047
SEC1P	0.960553	0.039447	0.401225	0.307655	1.11141	3.635024
CECR2	0.960559	0.039441	0.469732	0.412831	2.222819	5.39855
GOLGA2P	0.960559	0.039441	0.469732	0.412831	2.222819	5.39855
IL21R	0.960559	0.039441	0.469732	0.412831	2.222819	5.39855
LINC0112	0.960559	0.039441	0.469732	0.412831	2.222819	5.39855
LINC0132	0.960559	0.039441	0.469732	0.412831	2.222819	5.39855
MSANTD1	0.960559	0.039441	0.469732	0.412831	2.222819	5.39855
SEC14L5	0.960559	0.039441	0.469732	0.412831	2.222819	5.39855
SLC23A3	0.960559	0.039441	0.469732	0.412831	2.222819	5.39855
PWARSN	0.960572	0.039428	0.706192	0.701167	23.3396	33.29106
TUBB2B	0.960598	0.039402	1.406574	1.406833	1245.712	885.4701
CPT1C	0.960606	0.039394	0.745926	0.745075	126.7007	170.0543
LINC0127	0.960614	0.039386	1.408483	1.418056	34.4537	24.29347
DDHD1	0.96063	0.03937	1.370446	1.370962	561.2618	409.39
CEP104	0.960649	0.039351	0.713577	0.713427	788.0783	1104.642
UBE2D4	0.960676	0.039324	0.730101	0.729806	380.3021	521.104
PCDHA13	0.960726	0.039274	0.625384	0.610043	8.635652	14.1622
PGBD4	0.960735	0.039265	0.731821	0.729038	40.01074	54.88526
BGLAP	0.960744	0.039256	3.564326	147.7061	1.467061	0
ZNF765	0.960772	0.039228	0.719414	0.719229	630.3359	876.4096
OXSM	0.960776	0.039224	0.742572	0.742007	192.2739	259.1304
FAM200A	0.960792	0.039208	0.728544	0.728268	402.3303	552.4516
BMP8A	0.960796	0.039204	0.630851	0.616346	8.924619	14.48611
MTSS1L	0.960801	0.039199	0.699884	0.699794	1358.143	1940.779
DGCR11	0.960819	0.039181	1.402434	1.41153	35.56511	25.19323
SEMA4G	0.960824	0.039176	1.340572	1.343266	96.69263	71.98066
DYNLL1	0.960836	0.039164	0.680305	0.680262	2908.559	4275.651
CES2	0.960844	0.039156	0.731842	0.731536	371.9888	508.5074
NABP2	0.960851	0.039149	0.710908	0.710773	863.5652	1214.971
C1orf174	0.960888	0.039112	1.373278	1.37375	619.3108	450.8149
PPEF2	0.960902	0.039098	0.265628	0.030094	0.044456	1.799517
AP3B2	0.960911	0.039089	1.335872	1.338128	115.5866	86.3768
MINA	0.960912	0.039088	0.704357	0.704251	1120.301	1590.773
MRPL14	0.960921	0.039079	1.405092	1.405348	1267.007	901.5578
DLSTP1	0.960992	0.039008	0.69817	0.692069	19.86089	28.70229
HAUS1	0.960998	0.039002	0.720864	0.720669	586.8243	814.2813
ATP6AP1I	0.961	0.039	0.688877	0.681875	17.24908	25.3012
IPO4	0.961	0.039	0.726859	0.726611	455.6779	627.1315
NEO1	0.961014	0.038986	1.396794	1.405453	36.67652	26.09299
DIRAS1	0.961034	0.038966	1.356587	1.361193	60.01612	44.08816
LOC38843	0.961058	0.038942	2.316731	2.868363	3.889934	1.349637
RICTOR	0.96107	0.03893	1.375357	1.3758	663.5115	482.2705
RIOK1	0.96107	0.03893	0.706948	0.706833	1025.831	1451.31
ARHGAP3	0.961086	0.038914	0.404681	0.310723	1.11141	3.599033
C9orf153	0.961086	0.038914	0.404681	0.310723	1.11141	3.599033
CAND1.11	0.961086	0.038914	0.404681	0.310723	1.11141	3.599033
CATSPER	0.961086	0.038914	0.404681	0.310723	1.11141	3.599033
CFP	0.961086	0.038914	0.404681	0.310723	1.11141	3.599033
FAM87A	0.961086	0.038914	0.404681	0.310723	1.11141	3.599033
HIST1H3F	0.961086	0.038914	0.404681	0.310723	1.11141	3.599033
HLA-DRA	0.961086	0.038914	0.404681	0.310723	1.11141	3.599033

IRX3	0.961086	0.038914	0.404681	0.310723	1.11141	3.599033
KCNAB1	0.961086	0.038914	0.404681	0.310723	1.11141	3.599033
LOC10013	0.961086	0.038914	0.404681	0.310723	1.11141	3.599033
LOC10192	0.961086	0.038914	0.404681	0.310723	1.11141	3.599033
LOC10192	0.961086	0.038914	0.404681	0.310723	1.11141	3.599033
LOC44091	0.961086	0.038914	0.404681	0.310723	1.11141	3.599033
NCOR1P1	0.961086	0.038914	0.404681	0.310723	1.11141	3.599033
NHLRC4	0.961086	0.038914	0.404681	0.310723	1.11141	3.599033
SLC51A	0.961086	0.038914	0.404681	0.310723	1.11141	3.599033
ZG16	0.961086	0.038914	0.404681	0.310723	1.11141	3.599033
LOC10192	0.961089	0.038911	0.632415	0.617881	8.891277	14.39613
PTPN12	0.961102	0.038898	1.443644	1.443775	2800.752	1939.879
PRELID3A	0.961102	0.038898	0.747882	0.746691	90.02418	120.5676
BANK1	0.961127	0.038873	1.335913	1.338128	115.5866	86.3768
THADA	0.961144	0.038856	0.712995	0.712853	840.2256	1178.683
L3HYPDH	0.961157	0.038843	0.745334	0.743689	65.57317	88.17631
MMS22L	0.961164	0.038836	1.380575	1.380964	782.9992	566.9917
DAPK2	0.961201	0.038799	1.391522	1.399781	37.78793	26.99275
LOC10050	0.961215	0.038785	0.747058	0.745616	74.46444	99.87317
ADCK2	0.961225	0.038775	0.714064	0.713916	786.878	1102.204
TDG	0.961247	0.038753	0.722994	0.72279	569.0417	787.2885
CD247	0.961256	0.038744	1.569138	1.605128	14.44832	8.997583
HOXA2	0.961256	0.038744	1.569138	1.605128	14.44832	8.997583
TXK	0.961256	0.038744	1.569138	1.605128	14.44832	8.997583
VWDE	0.961256	0.038744	1.569138	1.605128	14.44832	8.997583
PKN1	0.961291	0.038709	0.668298	0.668271	4721.268	7064.902
L2HGDH	0.961294	0.038706	0.710516	0.710387	909.2775	1279.978
CLK3	0.961295	0.038705	1.382259	1.382632	823.5545	595.64
SENP5	0.961296	0.038704	0.714701	0.714552	796.0916	1114.117
CHAMP1	0.961322	0.038678	0.714853	0.714702	769.0954	1076.111
PAXIP1-A	0.961371	0.038629	1.386437	1.394475	38.89934	27.89251
SHE	0.961385	0.038615	0.632679	0.617881	8.891277	14.39613
PTCD1	0.961396	0.038604	0.714034	0.713889	799.2257	1119.542
PSEN2	0.961402	0.038598	0.74764	0.746897	144.4832	193.448
ZCCHC3	0.961433	0.038567	0.729838	0.729575	434.5611	595.64
CCPG1	0.961479	0.038521	0.690441	0.69038	1996.447	2891.814
AKIP1	0.961488	0.038512	0.723893	0.723683	542.579	749.7506
SULF1	0.961502	0.038498	1.333231	1.33573	103.3611	77.37921
LINC0112	0.961519	0.038481	1.396079	1.396365	1100.54	788.1433
HLA-DRB1	0.961528	0.038472	0.585729	0.561911	5.557048	9.897341
LINC0144	0.961528	0.038472	0.585729	0.561911	5.557048	9.897341
CSDC2	0.961534	0.038466	1.568499	1.605128	14.44832	8.997583
FECH	0.96156	0.03844	0.733035	0.732732	367.8766	502.0651
AHCTF1P	0.961565	0.038435	1.414305	1.42547	30.1192	21.12632
ZNF322	0.961574	0.038426	0.700609	0.700522	1399.265	1997.463
LARS2-AS	0.961587	0.038413	0.403609	0.308416	1.11141	3.626026
MLANA	0.961599	0.038401	0.41	0.316882	1.133638	3.599033
ARPC5	0.961611	0.038389	1.497252	1.497303	8438.933	5636.086
LZIC	0.961618	0.038382	0.702464	0.702371	1308.162	1862.5
CARHSP1	0.961626	0.038374	1.414125	1.414331	1614.878	1141.793
SLMAP	0.961639	0.038361	1.44217	1.442297	2903.002	2012.759
ZNF492	0.961645	0.038355	0.732687	0.729533	35.8763	49.18079
TRNT1	0.961646	0.038354	0.727324	0.727091	494.5773	680.2173
YBX2	0.961671	0.038329	0.72042	0.72024	635.7263	882.6629
GIT2	0.96168	0.03832	0.715339	0.71519	777.9867	1087.808
ACBD4	0.961689	0.038311	0.728072	0.72783	464.5692	638.2975

SHF	0.961697	0.038303	1.377451	1.384827	41.12215	29.69202
POU6F1	0.961704	0.038296	1.338068	1.341213	82.09983	61.21056
BIRC5	0.961723	0.038277	1.468076	1.468159	4623.464	3149.154
RBBP8NL	0.96173	0.03827	0.743384	0.741177	50.01343	67.48187
PLBD1	0.961732	0.038268	0.735651	0.732815	38.89934	53.08574
ZNF215	0.961754	0.038246	1.336563	1.339564	85.57854	63.88284
ZMYM5	0.961757	0.038243	0.731999	0.731711	383.4808	524.0912
DPY19L2P	0.961801	0.038199	0.735135	0.732223	37.93241	51.80808
ZNF763	0.961805	0.038195	0.588406	0.564764	5.59039	9.906339
CAMKK1	0.961812	0.038188	0.750118	0.749254	123.3665	164.6558
SCG2	0.961824	0.038176	0.722394	0.718231	27.78524	38.68961
GAS1	0.961825	0.038175	0.722318	0.718231	27.78524	38.68961
LOC10099	0.961825	0.038175	0.722318	0.718231	27.78524	38.68961
GNAZ	0.961833	0.038167	0.740932	0.740508	257.847	348.2065
HCK	0.961837	0.038163	1.332158	1.334575	104.4725	78.27897
LOC79999	0.961871	0.038129	2.309432	2.868363	3.889934	1.349637
CCDC96	0.961881	0.038119	1.373477	1.380428	42.23356	30.59178
CHRNB4	0.961881	0.038119	1.516663	1.543536	16.67114	10.7971
CXorf23	0.961893	0.038107	0.747771	0.747127	166.7114	223.1401
XRCC2	0.961898	0.038102	0.725005	0.724794	539.356	744.1541
SSRP1	0.961901	0.038099	0.667606	0.667581	5182.503	7763.115
EVA1C	0.96193	0.03807	1.539908	1.571535	15.55973	9.897341
LVCAT5	0.96193	0.03807	1.539908	1.571535	15.55973	9.897341
PI4KAP1	0.961932	0.038068	0.742461	0.740174	47.4683	64.13477
AVPI1	0.96194	0.03806	0.711637	0.711509	918.0243	1290.253
ZNF664	0.961952	0.038048	0.668834	0.668808	4947.918	7398.119
TM6SF2	0.961956	0.038044	0.586154	0.561911	5.557048	9.897341
ERP29	0.961964	0.038036	0.693152	0.693087	1862.722	2687.578
C5orf63	0.961969	0.038031	0.711163	0.70594	22.22819	31.49154
LOC28504	0.961978	0.038022	0.406209	0.310723	1.11141	3.599033
OTOF	0.961978	0.038022	0.406209	0.310723	1.11141	3.599033
STAB1	0.961978	0.038022	0.406209	0.310723	1.11141	3.599033
TFB1M	0.962009	0.037991	0.744775	0.744265	212.2792	285.2234
FRG1	0.962012	0.037988	0.732317	0.732044	415.5672	567.6845
SPPL2A	0.962031	0.037969	0.724066	0.723868	586.8243	810.6822
JUN	0.962035	0.037965	1.454829	1.45493	3819.915	2625.495
MIR17HG	0.962038	0.037962	1.516322	1.543536	16.67114	10.7971
RIPK3	0.962038	0.037962	1.516322	1.543536	16.67114	10.7971
DNAJC17	0.962046	0.037954	0.749815	0.748292	70.0188	93.57486
KAT2B	0.962058	0.037942	1.357849	1.358428	477.9061	351.8055
RPPH1	0.962079	0.037921	0.693459	0.686369	16.67114	24.29347
KLHL30	0.962087	0.037913	0.751709	0.750671	102.2497	136.2144
DMC1	0.962105	0.037895	0.72335	0.719145	27.45182	38.17674
SDCBP	0.962148	0.037852	1.485884	1.485944	6844.06	4605.863
TARBP2	0.962153	0.037847	0.730405	0.730155	457.9007	627.1315
TUBB8	0.962156	0.037844	0.487964	0.432247	2.222819	5.155615
POLR3G	0.962186	0.037814	0.750484	0.749781	154.4859	206.0447
PLAA	0.962197	0.037803	1.435847	1.435985	2599.587	1810.314
SLC26A7	0.962263	0.037737	1.515834	1.543536	16.67114	10.7971
FLJ31662	0.962275	0.037725	0.687913	0.680014	15.293	22.49396
GPR89A	0.962283	0.037717	1.408678	1.408894	1522.798	1080.844
PAXBP1-A	0.96229	0.03771	0.671113	0.661074	12.55893	19.0029
DTX1	0.962299	0.037701	2.115744	2.462336	4.445638	1.799517
FAM106A	0.962299	0.037701	2.115744	2.462336	4.445638	1.799517
HSPB2	0.962299	0.037701	2.115744	2.462336	4.445638	1.799517
LINC0160	0.962299	0.037701	2.115744	2.462336	4.445638	1.799517

PCYT1B	0.962299	0.037701	2.115744	2.462336	4.445638	1.799517
STXBP5-A	0.962299	0.037701	2.115744	2.462336	4.445638	1.799517
TMEM134	0.96233	0.03767	0.740377	0.739994	285.6323	385.9963
RNASEH2	0.962332	0.037668	0.737217	0.736893	346.7598	470.5736
RGPD5	0.962334	0.037666	1.429999	1.430149	2340.762	1636.723
HMGA1	0.962366	0.037634	1.519181	1.519215	13246.89	8719.558
RALGDS	0.962386	0.037614	1.37459	1.374984	743.533	540.7547
SRRM2-A	0.962425	0.037575	2.144424	2.511472	4.534551	1.799517
TTI2	0.962435	0.037565	1.337662	1.338604	273.4068	204.2451
MARC1	0.962436	0.037564	0.753033	0.7519	93.3584	124.1666
TTN-AS1	0.962436	0.037564	0.753033	0.7519	93.3584	124.1666
LOC10192	0.962442	0.037558	0.742523	0.742104	259.9254	350.2579
NAIP	0.962442	0.037558	1.327327	1.329579	110.0629	82.77776
VWA9	0.962456	0.037544	0.718622	0.718465	732.4189	1019.426
CKAP4	0.962461	0.037539	1.433999	1.434138	2556.242	1782.421
TMEM231	0.962482	0.037518	0.746017	0.745533	226.7276	304.1183
HSD17B14	0.96251	0.03749	0.661801	0.650326	11.1141	17.09541
ZSCAN18	0.962517	0.037483	0.744205	0.743767	252.29	339.2089
NFYC	0.962577	0.037423	0.712828	0.712702	924.6928	1297.451
ARMC4	0.962585	0.037415	1.334673	1.335687	251.1786	188.0495
KCNMB2-A	0.962587	0.037413	0.662429	0.650976	11.12521	17.09541
STK11	0.962599	0.037401	1.36297	1.363456	579.0444	424.6859
LOC10013	0.962611	0.037389	1.477933	1.499527	18.89396	12.59662
MAPT	0.962611	0.037389	1.477933	1.499527	18.89396	12.59662
NDUFA6-A	0.962616	0.037384	1.356041	1.361819	47.79061	35.09057
VCX3B	0.962634	0.037366	3.473606	144.3718	1.433718	0
KLB	0.962644	0.037356	0.488634	0.432247	2.222819	5.155615
NPHP4	0.962655	0.037345	1.342232	1.343019	332.3115	247.4335
CENPA	0.962656	0.037344	1.375167	1.375547	773.5411	562.3489
FEM1C	0.962669	0.037331	1.432294	1.432435	2510.674	1752.729
PPP1R13I	0.962712	0.037288	1.412418	1.412613	1699.345	1202.977
WBP4	0.96273	0.03727	0.742784	0.742378	267.8497	360.8031
DBNDD2	0.962745	0.037255	0.753613	0.752504	93.91411	124.8055
SNHG4	0.96275	0.03725	0.754888	0.753618	84.4449	112.0559
DTX2P1-U	0.96277	0.03723	0.731294	0.727513	29.99694	41.23592
KCTD9	0.962781	0.037219	1.400307	1.400545	1337.026	954.6436
IL20RA	0.962793	0.037207	1.477543	1.499527	18.89396	12.59662
C22orf24	0.96281	0.03719	2.112226	2.462336	4.445638	1.799517
C5orf17	0.96281	0.03719	2.112226	2.462336	4.445638	1.799517
CHRDL2	0.96281	0.03719	2.112226	2.462336	4.445638	1.799517
CLEC18B	0.96281	0.03719	2.112226	2.462336	4.445638	1.799517
CTRL	0.96281	0.03719	2.112226	2.462336	4.445638	1.799517
ESPNP	0.96281	0.03719	2.112226	2.462336	4.445638	1.799517
FAM182A	0.96281	0.03719	2.112226	2.462336	4.445638	1.799517
FLJ27354	0.96281	0.03719	2.112226	2.462336	4.445638	1.799517
FRG2	0.96281	0.03719	2.112226	2.462336	4.445638	1.799517
HIST1H3A	0.96281	0.03719	2.112226	2.462336	4.445638	1.799517
HIST1H3J	0.96281	0.03719	2.112226	2.462336	4.445638	1.799517
KCNMB1	0.96281	0.03719	2.112226	2.462336	4.445638	1.799517
LINC0026	0.96281	0.03719	2.112226	2.462336	4.445638	1.799517
LINC0146	0.96281	0.03719	2.112226	2.462336	4.445638	1.799517
LINC0155	0.96281	0.03719	2.112226	2.462336	4.445638	1.799517
LOC10192	0.96281	0.03719	2.112226	2.462336	4.445638	1.799517
PIK3R6	0.96281	0.03719	2.112226	2.462336	4.445638	1.799517
RCAN3AS	0.96281	0.03719	2.112226	2.462336	4.445638	1.799517
ZNF341-A	0.96281	0.03719	2.112226	2.462336	4.445638	1.799517

C1orf145	0.96281	0.03719	0.734609	0.731116	32.23088	44.08816
SUV39H2	0.962812	0.037188	1.351558	1.352178	431.2269	318.9103
LPP	0.962835	0.037165	1.450807	1.450909	3643.434	2511.135
ZC3HAV1	0.962839	0.037161	0.750599	0.749973	170.0457	226.7391
POTEF	0.962845	0.037155	0.59524	0.571365	5.434793	9.519443
NXNL2	0.962846	0.037154	1.462419	1.48192	20.00537	13.49637
TNNT2	0.96287	0.03713	0.7345	0.731116	32.23088	44.08816
MGAT4B	0.962911	0.037089	1.472134	1.472206	5427.013	3686.31
MRPS18A	0.962923	0.037077	0.70999	0.70988	1090.293	1535.887
ZNF316	0.962927	0.037073	0.736565	0.736265	368.988	501.1654
FUT5	0.962938	0.037062	1.422539	1.436377	24.97337	17.38333
HIST1H2E	0.962983	0.037017	1.359346	1.359849	553.482	407.0147
TMC7	0.963013	0.036987	0.755928	0.754645	83.3446	110.4453
PKM	0.963045	0.036955	1.564318	1.564333	34439.25	22015.29
FRK	0.963045	0.036955	1.330132	1.331215	231.1732	173.6534
CDK13	0.963055	0.036945	0.694161	0.694099	1941.633	2797.349
GPD1	0.963056	0.036944	1.448769	1.466513	21.11678	14.39613
TSHZ2	0.963056	0.036944	1.448769	1.466513	21.11678	14.39613
UHRF1BP	0.963058	0.036942	0.730142	0.729913	491.243	673.0192
AMMECR1	0.963077	0.036923	0.717344	0.717202	812.4404	1132.796
ADAMTSL	0.963114	0.036886	1.348171	1.348805	422.3356	313.1159
UBE2B	0.963124	0.036876	1.427093	1.427241	2346.186	1643.858
B3GALT4	0.963126	0.036874	0.683043	0.673927	13.33691	19.79468
LOC10537	0.963126	0.036874	0.683043	0.673927	13.33691	19.79468
ELL3	0.963141	0.036859	0.751865	0.749995	56.68189	75.5797
ABCB5	0.963141	0.036859	0.517791	0.471193	2.76741	5.884419
ZNF428	0.963148	0.036852	0.73703	0.736732	371.2108	503.8647
TRIM33	0.963204	0.036796	0.714903	0.714776	928.027	1298.351
ADAMTSL	0.963213	0.036787	2.098211	2.44391	4.412296	1.799517
HIST1H3E	0.963222	0.036778	0.634436	0.617919	7.779867	12.59662
F2RL3	0.963239	0.036761	0.739333	0.736161	34.4537	46.80543
ZNF565	0.963243	0.036757	1.320955	1.323419	100.0269	75.5797
DSC2	0.96328	0.03672	1.344769	1.34544	393.439	292.4214
PPP2R5D	0.96328	0.03672	1.415342	1.415518	1937.187	1368.532
ACP7	0.963294	0.036706	1.323833	1.325191	180.0484	135.8635
CCDC7	0.963327	0.036673	0.755069	0.753518	67.79598	89.97583
MFI2-AS1	0.963338	0.036662	1.426053	1.440831	23.3396	16.19565
KIRREL2	0.963357	0.036643	0.716723	0.711291	21.11678	29.69202
CGB5	0.963362	0.036638	2.068229	2.387052	4.545665	1.89849
BPHL	0.963384	0.036616	0.724547	0.724367	633.5035	874.5651
RAB31	0.963391	0.036609	1.363945	1.364383	645.729	473.2729
ALDH6A1	0.963398	0.036602	0.70517	0.70508	1318.532	1870.049
ATP7B	0.963434	0.036566	0.75251	0.751891	171.1571	227.6389
FAM126B	0.96345	0.03655	1.378512	1.378843	898.2412	651.443
FOXP1	0.963454	0.036546	1.352748	1.353295	496.8001	367.1014
PAN3	0.963458	0.036542	0.738296	0.737994	364.5423	493.9673
ERCC2	0.963468	0.036532	1.41678	1.416951	1967.195	1388.327
ABL2	0.963497	0.036503	1.445672	1.445778	3460.841	2393.753
MANSC1	0.963497	0.036503	1.32841	1.329448	239.5866	180.2126
NLRP12	0.963497	0.036503	0.651139	0.637119	9.191357	14.43212
USP13	0.963498	0.036502	0.737171	0.736884	384.5477	521.8598
LINC0097	0.963499	0.036501	0.315992	0.111642	0.200054	1.871497
DIAPH1	0.963501	0.036499	1.525716	1.525744	16221.02	10631.54
CBLN1	0.963514	0.036486	2.10722	2.462336	4.445638	1.799517
LINC0069	0.963514	0.036486	2.10722	2.462336	4.445638	1.799517
LSMEM2	0.963514	0.036486	2.10722	2.462336	4.445638	1.799517

NPTX1	0.963514	0.036486	2.10722	2.462336	4.445638	1.799517
PCAT19	0.963514	0.036486	2.10722	2.462336	4.445638	1.799517
RLN1	0.963514	0.036486	2.10722	2.462336	4.445638	1.799517
SLC34A3	0.963514	0.036486	2.10722	2.462336	4.445638	1.799517
TDO2	0.963514	0.036486	2.10722	2.462336	4.445638	1.799517
TMEM255	0.963514	0.036486	2.10722	2.462336	4.445638	1.799517
RNF19A	0.963539	0.036461	1.408283	1.408479	1671.56	1186.781
CYP3A5	0.963541	0.036459	0.634734	0.617919	7.779867	12.59662
PASD1	0.963576	0.036424	1.416158	1.430016	24.45101	17.09541
PLXNC1	0.963576	0.036424	1.416158	1.430016	24.45101	17.09541
DPYSL5	0.963604	0.036396	2.073026	2.402603	4.445638	1.844505
RPF2	0.963617	0.036383	0.704017	0.703932	1400.376	1989.366
C5orf24	0.963619	0.036381	0.707892	0.707794	1212.548	1713.14
MAPRE3	0.963633	0.036367	1.332533	1.333414	286.7437	215.0422
PIAS2	0.963639	0.036361	0.725722	0.725539	620.1665	854.7704
ATF7IP2	0.963658	0.036342	0.756581	0.755687	115.5866	152.9589
KIT	0.963662	0.036338	1.407559	1.420282	25.56242	17.99517
GATA5	0.96368	0.03632	1.415905	1.430016	24.45101	17.09541
DNAJC1	0.963688	0.036312	0.706553	0.706461	1281.455	1813.913
CREG1	0.963694	0.036306	0.669021	0.668998	5390.336	8057.336
NRG4	0.963723	0.036277	1.407394	1.420282	25.56242	17.99517
NDE1	0.963736	0.036264	1.379562	1.379878	943.5867	683.8163
PHIP	0.963744	0.036256	0.708434	0.708335	1175.871	1660.054
DEF6	0.963757	0.036243	1.324192	1.325281	228.9504	172.7536
MIAT	0.963765	0.036235	1.554403	1.594087	12.91458	8.097825
LOC10192	0.96377	0.03623	0.699473	0.691866	15.55973	22.49396
FAM213B	0.963772	0.036228	1.358284	1.358746	611.2753	449.8792
RPUSD4	0.963782	0.036218	1.355433	1.355938	536.8108	395.8937
ZFAT	0.963784	0.036216	1.339812	1.340517	367.8766	274.4263
ZNF829	0.9638	0.0362	1.31587	1.317704	131.7131	99.95415
SMG6	0.963843	0.036157	0.738136	0.737855	398.996	540.7547
CD276	0.963867	0.036133	1.429324	1.429459	2592.919	1813.913
ADAM28	0.963894	0.036106	0.579117	0.549548	4.445638	8.097825
C19orf45	0.963894	0.036106	0.579117	0.549548	4.445638	8.097825
CFAP47	0.963894	0.036106	0.579117	0.549548	4.445638	8.097825
FOXD4L5	0.963894	0.036106	0.579117	0.549548	4.445638	8.097825
PTOV1-AS	0.963894	0.036106	0.579117	0.549548	4.445638	8.097825
RAET1E-A	0.963894	0.036106	0.579117	0.549548	4.445638	8.097825
LZTS1	0.963902	0.036098	0.725961	0.725782	630.1692	868.2668
TDP1	0.963916	0.036084	1.393499	1.393741	1319.243	946.5457
TMEM217	0.96394	0.03606	1.399219	1.411475	26.67383	18.89492
CEP76	0.96394	0.03606	0.753099	0.752508	176.7141	234.8369
LOC40068	0.963958	0.036042	0.751055	0.748667	44.45638	59.38405
TMEM86B	0.963961	0.036039	1.321826	1.32296	217.8363	164.6558
HABP4	0.96397	0.03603	1.364261	1.364671	691.2968	506.5639
HCFC1R1	0.963983	0.036017	0.747385	0.746967	256.7356	343.7077
CPNE4	0.96399	0.03601	1.392188	1.403468	27.78524	19.79468
TSG1	0.963998	0.036002	0.676342	0.66557	11.38083	17.10441
ZNF419	0.964	0.036	1.354566	1.355051	574.7432	424.1461
SH2B2	0.964015	0.035985	0.743052	0.742708	316.7517	426.4854
ZNF16	0.964047	0.035953	0.745636	0.745253	282.298	378.7982
LINC0098	0.964064	0.035936	1.32797	1.33204	61.12753	45.88767
POU2F3	0.964064	0.035936	1.32797	1.33204	61.12753	45.88767
SLC6A12	0.964073	0.035927	1.337458	1.338155	375.6564	280.7246
DSCAM-A	0.964086	0.035914	0.277165	0.006572	0	1.511594
PDE7A	0.964093	0.035907	1.360082	1.36052	636.9599	468.1712

SOWAHD	0.964097	0.035903	0.757542	0.755703	57.7933	76.47946
SNX13	0.964101	0.035899	0.733025	0.732797	487.9088	665.8211
C7orf31	0.964105	0.035895	0.758779	0.757106	63.35035	83.67752
SEL1L3	0.964146	0.035854	1.474514	1.474577	6470.627	4388.121
GPR65	0.964147	0.035853	0.554446	0.517289	3.523168	6.820168
RASIP1	0.964156	0.035844	1.337132	1.337826	376.7678	281.6243
GGA3	0.964162	0.035838	0.723198	0.723037	701.2994	969.9395
ASH1L-AS	0.96421	0.03579	0.66658	0.654172	10.00269	15.29589
PDIA4	0.964222	0.035778	0.650969	0.650958	11608.67	17833.21
IFRD2	0.964226	0.035774	0.717057	0.716931	934.6955	1303.75
TMEM79	0.964238	0.035762	0.75081	0.750349	235.6188	314.0156
DENND1C	0.964243	0.035757	0.761238	0.760161	97.80404	128.6654
TMEM243	0.964269	0.035731	1.388109	1.388365	1219.216	878.1641
GPX7	0.964276	0.035724	1.324512	1.328387	63.35035	47.68719
ZNF599	0.964279	0.035721	0.732962	0.728547	25.56242	35.09057
OGFRL1	0.964286	0.035714	1.408457	1.408639	1832.714	1301.051
C4B	0.964297	0.035703	0.678537	0.667782	11.35861	17.01443
C4B_2	0.964297	0.035703	0.678537	0.667782	11.35861	17.01443
UHRF1	0.964305	0.035695	0.730248	0.730046	555.7048	761.1955
BRE	0.96433	0.03567	0.723906	0.723744	693.5196	958.2426
LOC10012	0.964336	0.035664	1.873568	2.054811	5.557048	2.699275
TNRC18P	0.964336	0.035664	1.873568	2.054811	5.557048	2.699275
SBK3	0.96436	0.03564	0.579638	0.549548	4.445638	8.097825
SPRR2D	0.96436	0.03564	0.579638	0.549548	4.445638	8.097825
CISD2	0.964362	0.035638	0.724588	0.724424	693.5196	957.3428
TMCO6	0.964442	0.035558	0.74727	0.746891	288.9665	386.8961
MAGI1	0.964495	0.035505	0.718081	0.717953	894.6847	1246.165
ARHGEF7	0.964514	0.035486	0.710292	0.710194	1193.654	1680.749
MAML3	0.964546	0.035454	1.363799	1.372326	33.34229	24.29347
NR4A2	0.964546	0.035454	1.363799	1.372326	33.34229	24.29347
TRIM22	0.964546	0.035454	1.363799	1.372326	33.34229	24.29347
TAF4B	0.964575	0.035425	0.756484	0.755898	182.2712	241.1352
LOC10192	0.964586	0.035414	2.37516	3.236158	2.934121	0.899758
PINK1	0.964619	0.035381	1.423743	1.423882	2466.218	1732.035
CXorf36	0.964644	0.035356	0.551804	0.513057	3.434256	6.703199
ANKRD46	0.964652	0.035348	0.735751	0.735512	464.5692	631.6303
CCDC50	0.964661	0.035339	1.418493	1.418644	2235.045	1575.477
STXBP1	0.964669	0.035331	1.390984	1.39122	1338.137	961.8416
CIDEC	0.96468	0.03532	1.35888	1.366991	34.44258	25.19323
STAT5B	0.9647	0.0353	1.397917	1.398127	1540.547	1101.862
TMEM143	0.964722	0.035278	1.309892	1.31239	94.46981	71.98066
PQBP1	0.964727	0.035273	0.714762	0.714649	1031.388	1443.212
NPL	0.964741	0.035259	1.355125	1.362875	35.56511	26.09299
ZNF718	0.964747	0.035253	1.315901	1.3194	69.11856	52.38393
ADGRE1	0.964747	0.035253	1.871569	2.054811	5.557048	2.699275
AKR7A3	0.964747	0.035253	1.871569	2.054811	5.557048	2.699275
CCR10	0.964747	0.035253	1.871569	2.054811	5.557048	2.699275
CRISPLD1	0.964747	0.035253	1.871569	2.054811	5.557048	2.699275
CYP26B1	0.964747	0.035253	1.871569	2.054811	5.557048	2.699275
DACH1	0.964747	0.035253	1.871569	2.054811	5.557048	2.699275
FAM189A1	0.964747	0.035253	1.871569	2.054811	5.557048	2.699275
PPP5D1	0.964747	0.035253	1.871569	2.054811	5.557048	2.699275
PYGM	0.964747	0.035253	1.871569	2.054811	5.557048	2.699275
THSD7B	0.964747	0.035253	1.871569	2.054811	5.557048	2.699275
ZCCHC12	0.964747	0.035253	1.871569	2.054811	5.557048	2.699275
NREP	0.964764	0.035236	1.350962	1.351448	555.7048	411.1895

BEND6	0.964766	0.035234	0.762492	0.761462	100.0269	131.3647
MRPL20	0.964768	0.035232	0.72657	0.726403	690.1853	950.1448
LOC10192	0.96478	0.03522	1.495818	1.525595	14.45944	9.474455
P3H1	0.964814	0.035186	1.357627	1.358052	651.286	479.5712
CDS2	0.96482	0.03518	0.720852	0.720715	831.3344	1153.49
PIGG	0.964824	0.035176	1.340696	1.341293	435.6726	324.8127
TUBA4B	0.964832	0.035168	3.320204	135.4806	1.344806	0
RMRP	0.964834	0.035166	1.351213	1.358622	36.67652	26.99275
DZIP1L	0.964834	0.035166	1.351076	1.358622	36.67652	26.99275
ZNF431	0.96484	0.03516	1.318477	1.319511	235.73	178.647
THOC6	0.964851	0.035149	0.758164	0.757555	174.4913	230.3381
SMC3	0.964871	0.035129	0.70409	0.704014	1584.87	2251.195
FCGBP	0.964874	0.035126	0.689382	0.679555	12.22551	17.99517
WWC2-AS	0.964874	0.035126	0.689382	0.679555	12.22551	17.99517
TRRAP	0.964894	0.035106	0.679714	0.679682	3852.146	5667.578
ZNF282	0.9649	0.0351	0.723823	0.723673	773.5411	1068.913
LILRB3	0.964914	0.035086	0.62376	0.602703	6.123867	10.16727
ALMS1-IT	0.964924	0.035076	1.347548	1.354643	37.78793	27.89251
APOLD1	0.964944	0.035056	1.312105	1.313496	167.8228	127.7657
LOC10302	0.964985	0.035015	1.324648	1.329252	53.381	40.15621
SEC24D	0.96499	0.03501	1.427248	1.427376	2716.285	1902.989
PLOD1	0.964995	0.035005	1.489431	1.489477	8920.173	5988.791
ATL1	0.965002	0.034998	0.744792	0.741203	30.00806	40.48912
PDE4B	0.965013	0.034987	1.344107	1.350912	38.89934	28.79227
GOLGA8C	0.965075	0.034925	0.283258	0.006772	0	1.466606
BFSP1	0.965115	0.034885	0.73169	0.726696	22.22819	30.59178
ZNF337-A	0.965115	0.034885	0.73169	0.726696	22.22819	30.59178
DLX1	0.965119	0.034881	1.309054	1.311437	95.58122	72.88042
MFSD14B	0.965121	0.034879	1.41336	1.413518	2099.164	1485.06
FAM53A	0.965136	0.034864	0.636857	0.617969	6.668457	10.7971
PTCH2	0.965136	0.034864	0.636857	0.617969	6.668457	10.7971
MSL1	0.96515	0.03485	1.403355	1.403543	1696.011	1208.375
SUGT1	0.96516	0.03484	1.395669	1.395882	1458.169	1044.619
OPLAH	0.965189	0.034811	0.731307	0.731115	583.49	798.0856
SLC25A12	0.965194	0.034806	0.735104	0.734888	522.3625	710.8091
ZNF852	0.965202	0.034798	0.762143	0.760176	53.34766	70.18115
SSBP1	0.965212	0.034788	0.685597	0.685559	3229.756	4711.134
CHRFAM7	0.965216	0.034784	1.851249	2.027873	5.557048	2.735265
MMP11	0.965223	0.034777	1.306655	1.308577	118.9208	90.87559
SMG1P3	0.96524	0.03476	0.7358	0.73558	511.1262	694.8653
ZNF445	0.965249	0.034751	0.748543	0.748186	305.6376	408.5083
VPS29	0.965278	0.034722	0.708504	0.708417	1357.031	1915.585
CD96	0.965281	0.034719	0.667243	0.653679	9.124673	13.96425
CDC45	0.965289	0.034711	0.719794	0.719666	901.3532	1252.464
ZNF626	0.965307	0.034693	2.016135	2.323686	4.445638	1.907488
AP4B1-AS	0.965315	0.034685	0.55881	0.519752	3.334229	6.424274
LOC10050	0.965317	0.034683	1.86872	2.054811	5.557048	2.699275
LOC10537	0.965317	0.034683	1.86872	2.054811	5.557048	2.699275
MYH16	0.965317	0.034683	1.86872	2.054811	5.557048	2.699275
MYLK2	0.965317	0.034683	1.86872	2.054811	5.557048	2.699275
PITRM1-A	0.965317	0.034683	1.86872	2.054811	5.557048	2.699275
SCARNA7	0.965317	0.034683	1.86872	2.054811	5.557048	2.699275
TAS2R20	0.965317	0.034683	1.86872	2.054811	5.557048	2.699275
TMEM26	0.965317	0.034683	1.86872	2.054811	5.557048	2.699275
SMC1B	0.965333	0.034667	0.749645	0.746344	32.23088	43.1884
DNAAF2	0.965335	0.034665	0.74321	0.742918	372.3222	501.1654

ACRC	0.965366	0.034634	0.706397	0.69832	14.44832	20.69444
SMIM10	0.965378	0.034622	0.765646	0.764088	66.68457	87.27656
ARHGAP9	0.96538	0.03462	0.637097	0.617969	6.668457	10.7971
CNGA1	0.96538	0.03462	0.637097	0.617969	6.668457	10.7971
CABP4	0.965394	0.034606	0.579128	0.546034	3.945504	7.234057
LINC0135	0.965396	0.034604	1.344112	1.351354	36.56538	27.05573
EFHC1	0.9654	0.0346	0.763479	0.762668	126.7007	166.1314
TEX26-AS	0.965406	0.034594	1.735872	1.850484	6.668457	3.599033
LIN7B	0.965417	0.034583	0.765077	0.763375	61.12753	80.07849
GOLGA8I	0.965423	0.034577	2.152461	2.641749	3.534282	1.331642
SNRNP35	0.965432	0.034568	0.763159	0.762428	143.3718	188.0495
MED7	0.965494	0.034506	0.742256	0.74198	394.5504	531.7572
HOGA1	0.965495	0.034505	0.765788	0.76435	70.93016	92.80107
MON2	0.96551	0.03449	0.711196	0.711103	1254.781	1764.561
CPLX1	0.965515	0.034485	0.706546	0.69832	14.44832	20.69444
FAM104A	0.965523	0.034477	0.72461	0.724463	772.4297	1066.214
CRTAP	0.965525	0.034475	1.462136	1.462206	5505.923	3765.489
RAI2	0.965528	0.034472	0.748188	0.747849	322.3088	430.9842
MAK	0.965553	0.034447	0.667295	0.653384	8.891277	13.61334
PTPRB	0.965558	0.034442	1.330461	1.331123	385.6591	289.7222
CD9	0.96556	0.03444	1.452448	1.452529	4591.233	3160.851
DENR	0.965569	0.034431	0.699726	0.699664	1964.094	2807.201
P3H2-AS1	0.965581	0.034419	0.639522	0.620538	6.7018	10.8061
SARM1	0.965587	0.034413	1.324883	1.330155	46.6792	35.09057
LEO1	0.965588	0.034412	0.739197	0.738954	450.1209	609.1364
CSPP1	0.965595	0.034405	0.735398	0.735186	521.2511	709.0095
TENM2	0.965601	0.034399	0.753992	0.750881	34.4537	45.88767
SETD4	0.965646	0.034354	0.756938	0.756437	208.945	276.2258
SNHG12	0.965654	0.034346	1.312623	1.313752	204.4994	155.6582
ZNF641	0.965669	0.034331	0.75269	0.752291	270.0725	359.0036
TMTC2	0.965688	0.034312	1.325184	1.325917	341.2027	257.3309
HELB	0.965697	0.034303	1.305003	1.307842	80.02149	61.18356
APC	0.965703	0.034297	0.710304	0.710215	1317.02	1854.402
THSD1	0.965714	0.034286	1.341352	1.3488	35.86519	26.58786
AASS	0.965715	0.034285	0.760136	0.757702	42.51142	56.10893
MAFF	0.965716	0.034284	1.367661	1.367984	888.0163	649.1396
LOC64465	0.965724	0.034276	0.63744	0.617969	6.668457	10.7971
ITPR3	0.965737	0.034263	1.506561	1.506595	13069.07	8674.57
PRICKLE4	0.965741	0.034259	1.320577	1.325526	48.90202	36.89009
MIR4435-2	0.96575	0.03425	1.347083	1.34755	569.7308	422.7874
C20orf203	0.965753	0.034247	1.734551	1.850484	6.668457	3.599033
CPNE8	0.965753	0.034247	1.734551	1.850484	6.668457	3.599033
CRHBP	0.965753	0.034247	1.734551	1.850484	6.668457	3.599033
HERC2P4	0.965753	0.034247	1.734551	1.850484	6.668457	3.599033
ITGB1BP2	0.965753	0.034247	1.734551	1.850484	6.668457	3.599033
KDM8	0.965759	0.034241	0.719581	0.712756	16.67114	23.39372
ADORA2B	0.965769	0.034231	1.314508	1.315504	236.7302	179.9517
HOOK2	0.965772	0.034228	0.713271	0.713173	1190.32	1669.052
HES6	0.965775	0.034225	0.750226	0.749871	305.6376	407.5905
ERMP1	0.965781	0.034219	1.484049	1.484097	8516.732	5738.658
TBC1D13	0.965782	0.034218	1.347491	1.347945	597.9384	443.5898
CEBPB	0.965785	0.034215	0.702214	0.702146	1777.144	2531.02
SUMO1P3	0.965798	0.034202	0.566344	0.528904	3.545397	6.712197
PSG5	0.965805	0.034195	0.767213	0.765733	68.58509	89.57094
SLC29A2	0.965816	0.034184	0.723977	0.723838	836.8914	1156.189
IQCD	0.965818	0.034182	1.300222	1.302327	107.8067	82.77776

NSUN7	0.965829	0.034171	0.750749	0.750381	288.9665	385.0966
HENMT1	0.96584	0.03416	1.300813	1.302577	128.9235	98.97341
DCAF15	0.965845	0.034155	1.376203	1.376475	1104.741	802.5844
PCGF5	0.965854	0.034146	1.40331	1.403485	1834.937	1307.412
NOS3	0.96587	0.03413	0.767984	0.766718	80.02149	104.372
ASL	0.965896	0.034104	0.729971	0.7298	657.9545	901.5578
WDR59	0.965897	0.034103	0.7358	0.735591	530.1424	720.7064
TAF1D	0.965929	0.034071	1.396028	1.396222	1648.22	1180.483
FAM63A	0.965956	0.034044	1.307129	1.308302	198.9423	152.0592
KCTD5	0.965958	0.034042	1.392885	1.393091	1510.406	1084.209
LOC1039(	0.965958	0.034042	0.568573	0.530131	3.334229	6.298308
RLF	0.965983	0.034017	0.728063	0.727907	733.5303	1007.729
GLI4	0.965993	0.034007	0.764547	0.763827	142.2604	186.25
LOC28392	0.96602	0.03398	1.334261	1.341111	37.26556	27.78454
MTHFS	0.966028	0.033972	1.340586	1.341099	506.8028	377.8985
GAGE7	0.966037	0.033963	0.289626	0.006985	0	1.421618
LOC10050	0.966058	0.033942	0.727353	0.721178	18.12709	25.13925
ULK1	0.966074	0.033926	1.358977	1.359339	766.8726	564.1485
ZNF141	0.966075	0.033925	0.768391	0.766848	65.57317	85.51303
IRF2BP2	0.966078	0.033922	0.677083	0.677057	4652.361	6871.454
SEMA3E	0.966082	0.033918	0.762819	0.762192	164.0107	215.1862
LOC10272	0.966089	0.033911	0.730096	0.724207	18.89396	26.09299
CEP131	0.966131	0.033869	0.741758	0.74151	449.0095	605.5373
FBXL13	0.966152	0.033848	1.594594	1.656804	8.891277	5.362559
COMMD2	0.966152	0.033848	0.722852	0.722722	872.601	1207.386
RAP1GAP	0.966165	0.033835	0.741019	0.740775	447.8981	604.6376
NR4A3	0.966168	0.033832	1.30822	1.312361	56.68189	43.1884
ELMOD1	0.966168	0.033832	0.508063	0.445915	2.000537	4.498792
LOC10192	0.96617	0.03383	0.439226	0.340428	1.11141	3.284118
PPP5C	0.96617	0.03383	1.412144	1.412293	2233.933	1581.775
FBXL18	0.966174	0.033826	1.377097	1.377359	1125.713	817.2955
AP5M1	0.966181	0.033819	0.713427	0.713332	1225.885	1718.538
RBBP9	0.966191	0.033809	0.745564	0.745282	390.2715	523.6593
EPT1	0.966212	0.033788	0.704875	0.704804	1716.016	2434.746
MTMR9	0.966227	0.033773	0.748311	0.747994	338.3686	452.3715
CD1D	0.966237	0.033763	1.732665	1.850484	6.668457	3.599033
GABRG1	0.966237	0.033763	1.732665	1.850484	6.668457	3.599033
SCARNA9	0.966237	0.033763	1.732665	1.850484	6.668457	3.599033
TMEM155	0.966237	0.033763	1.732665	1.850484	6.668457	3.599033
GIGYF2	0.966245	0.033755	0.718235	0.718125	1068.065	1487.3
WDR62	0.966254	0.033746	1.382733	1.38297	1269.23	917.7535
LOC37444	0.966257	0.033743	0.770406	0.768895	67.79598	88.17631
KIF14	0.96627	0.03373	1.388037	1.388254	1411.479	1016.727
MKL2	0.966281	0.033719	0.718838	0.718725	1019.163	1418.019
MET	0.966282	0.033718	1.497935	1.497972	11519.76	7690.234
TMEM251	0.966283	0.033717	0.737391	0.737182	537.9222	729.704
DNAH5	0.966285	0.033715	1.296869	1.299356	90.02418	69.28139
RTTN	0.966289	0.033711	1.30479	1.306019	184.494	141.2621
RND3	0.966292	0.033708	1.473044	1.4731	7171.926	4868.592
TASP1	0.966318	0.033682	0.767945	0.767157	131.1463	170.9541
HERC6	0.96632	0.03368	1.300757	1.302233	151.1517	116.0688
LINC0034	0.96632	0.03368	1.30062	1.303669	73.19743	56.14492
POC1A	0.966321	0.033679	0.723467	0.723336	866.8995	1198.478
ARHGEF3	0.966327	0.033673	0.569044	0.530131	3.334229	6.298308
GTF2IRD1	0.966327	0.033673	0.569044	0.530131	3.334229	6.298308
IFNB1	0.966327	0.033673	0.569044	0.530131	3.334229	6.298308

KCNK15-A	0.966327	0.033673	0.569044	0.530131	3.334229	6.298308
LOC40112	0.966327	0.033673	0.569044	0.530131	3.334229	6.298308
PLAC4	0.966327	0.033673	0.569044	0.530131	3.334229	6.298308
POTEM	0.966327	0.033673	0.569044	0.530131	3.334229	6.298308
SPATA4	0.966327	0.033673	0.569044	0.530131	3.334229	6.298308
ADGRB3	0.966343	0.033657	1.646085	1.727706	7.779867	4.498792
ADRA2C	0.966343	0.033657	1.646085	1.727706	7.779867	4.498792
FASTK	0.966343	0.033657	0.703023	0.702958	1853.831	2637.192
PDGFA	0.966352	0.033648	1.302502	1.303832	168.9343	129.5652
OR7E37P	0.966375	0.033625	0.72067	0.713426	15.65976	21.9541
TANGO6	0.966387	0.033613	0.765867	0.765199	155.5973	203.3454
CLCN2	0.966397	0.033603	0.752373	0.752011	292.3007	388.6956
PLIN4	0.966439	0.033561	0.738775	0.733511	21.11678	28.79227
MRPS25	0.966451	0.033549	0.72719	0.727042	767.984	1056.316
CRYM-AS	0.966454	0.033546	1.653455	1.737566	7.824323	4.498792
PIGR	0.966465	0.033535	1.32229	1.323016	329.933	249.377
ATP13A1	0.966475	0.033525	0.716581	0.716478	1123.635	1568.279
WASF3	0.966481	0.033519	0.764257	0.76177	41.12215	53.9855
OSBP	0.966497	0.033503	1.432706	1.43281	3393.133	2368.164
ILKAP	0.966503	0.033497	0.746211	0.745928	381.2135	511.0627
TRIM34	0.966509	0.033491	1.315751	1.321035	44.95652	34.02886
ZNF689	0.966514	0.033486	0.760041	0.75956	220.0591	289.7222
RP9P	0.966518	0.033482	1.299678	1.301114	154.686	118.8851
ARSI	0.966533	0.033467	1.302744	1.306429	61.12753	46.78743
NUP153	0.966548	0.033452	0.682226	0.682195	3956.618	5799.842
SLC2A1	0.966551	0.033449	1.466929	1.466989	6676.237	4550.978
SSC5D	0.966566	0.033434	1.300208	1.303793	63.35035	48.58695
ARHGAP1	0.966578	0.033422	1.326362	1.327002	385.6591	290.6219
C10orf12	0.96659	0.03341	0.755817	0.755411	258.9584	342.8079
SVIL-AS1	0.966601	0.033399	1.354203	1.354571	756.8699	558.7499
ARHGAP4	0.966613	0.033387	0.69708	0.686433	11.1141	16.19565
GLYCTK	0.966613	0.033387	0.69708	0.686433	11.1141	16.19565
NDUFAF5	0.966617	0.033383	1.303069	1.30426	188.9396	144.8611
SLC25A15	0.966624	0.033376	0.741196	0.740966	484.808	654.2952
ZNF567	0.966625	0.033375	1.327333	1.327955	397.8846	299.6195
SNX9	0.966635	0.033365	1.409262	1.40941	2257.273	1601.57
LOC40132	0.966641	0.033359	0.745817	0.741221	23.3396	31.49154
MTFR1L	0.966643	0.033357	0.735579	0.73539	596.8269	811.582
CHMP4A	0.966644	0.033356	0.749436	0.749128	354.5397	473.2729
TBXA2R	0.966644	0.033356	1.395298	1.411404	20.09429	14.23418
DDX11L1	0.966654	0.033346	3.185978	127.7007	1.267007	0
AGGF1	0.966664	0.033336	0.734086	0.733906	631.2806	860.1689
EZR	0.966665	0.033335	1.487606	1.487648	9727.057	6538.544
PANX1	0.96667	0.03333	1.366895	1.367189	990.2659	724.3054
KDM3A	0.966678	0.033322	1.416694	1.416826	2540.682	1793.218
TOR3A	0.966704	0.033296	0.70819	0.708115	1587.093	2241.298
GINS2	0.966709	0.033291	0.754566	0.754181	270.0725	358.1038
NR3C1	0.966713	0.033287	1.419561	1.419686	2762.964	1946.177
ACTN1-AS	0.966715	0.033285	1.584248	1.645779	8.891277	5.39855
ASTL	0.966715	0.033285	1.584248	1.645779	8.891277	5.39855
GNMT	0.966715	0.033285	1.584248	1.645779	8.891277	5.39855
HAGLR	0.966715	0.033285	1.584248	1.645779	8.891277	5.39855
LINC0059	0.966715	0.033285	1.584248	1.645779	8.891277	5.39855
ROR2	0.966715	0.033285	1.584248	1.645779	8.891277	5.39855
METTL9	0.966715	0.033285	0.701106	0.701047	1993.869	2844.136
CDCA7	0.966719	0.033281	0.719657	0.719547	1059.173	1472.005

LINC0092	0.966724	0.033276	1.397387	1.413947	19.3163	13.65833
SLC44A5	0.966725	0.033275	1.539178	1.587222	10.00269	6.298308
OBSCN	0.966725	0.033275	1.337901	1.338397	519.0283	387.7958
TAPT1	0.966729	0.033271	0.740074	0.739856	511.2484	691.0144
SREK1	0.966732	0.033268	0.727707	0.727563	797.9921	1096.805
LLGL2	0.966761	0.033239	0.711212	0.711128	1432.607	2014.559
MTMR8	0.966764	0.033236	1.644719	1.727706	7.779867	4.498792
TFF1	0.966764	0.033236	1.644719	1.727706	7.779867	4.498792
DUOX1	0.966781	0.033219	1.947038	2.241223	4.045531	1.799517
MTBP	0.966786	0.033214	0.759744	0.759279	223.3933	294.221
NME4	0.966787	0.033213	1.367168	1.367461	976.929	714.4081
SCN11A	0.966794	0.033206	1.773622	1.923802	5.634847	2.924214
LINC0144	0.966808	0.033192	1.438234	1.462189	15.22631	10.4102
BRE-AS1	0.966809	0.033191	1.590622	1.653998	8.935733	5.39855
NR2C1	0.966813	0.033187	0.749234	0.748925	342.3142	457.0772
UNC5C	0.96682	0.03318	0.697274	0.686433	11.1141	16.19565
MFSD6	0.96682	0.03318	1.34689	1.347305	641.2833	475.9721
ABHD1	0.966839	0.033161	0.569717	0.530131	3.334229	6.298308
DOCK9-A	0.966839	0.033161	0.569717	0.530131	3.334229	6.298308
LOC10537	0.966839	0.033161	0.569717	0.530131	3.334229	6.298308
PDZRN4	0.966839	0.033161	0.569717	0.530131	3.334229	6.298308
PIFO	0.966839	0.033161	0.569717	0.530131	3.334229	6.298308
SPATA9	0.966839	0.033161	0.569717	0.530131	3.334229	6.298308
SPEF1	0.966839	0.033161	0.569717	0.530131	3.334229	6.298308
FUT10	0.966841	0.033159	1.299823	1.301088	175.6027	134.9637
LINC0085	0.966857	0.033143	1.301833	1.302998	192.2739	147.5604
STOML1	0.966866	0.033134	0.758183	0.757754	243.3987	321.2137
RIMS3	0.966882	0.033118	1.315834	1.316609	305.6376	232.1376
ARRDC3-	0.966896	0.033104	1.402244	1.419975	18.33826	12.91153
GREB1	0.966897	0.033103	1.29602	1.299458	64.92855	49.96358
ARC	0.966911	0.033089	0.773237	0.772041	83.35572	107.971
ZNF860	0.966913	0.033087	1.305291	1.306276	234.952	179.8617
CLIC1	0.966916	0.033084	1.481635	1.481681	8846.82	5970.796
CA13	0.966917	0.033083	0.773139	0.77199	86.82332	112.4698
CSAG1	0.96692	0.03308	1.503982	1.543284	11.1141	7.198066
NBPF14	0.966922	0.033078	1.400977	1.401142	1974.23	1409.013
FAM175B	0.966932	0.033068	0.729578	0.729428	761.3156	1043.72
LINC0091	0.966947	0.033053	0.714601	0.706002	13.33691	18.89492
LOC10192	0.966947	0.033053	0.714601	0.706002	13.33691	18.89492
ATP1B4	0.966958	0.033042	1.539386	1.588229	9.980458	6.280313
ADAM21	0.966962	0.033038	1.53859	1.587222	10.00269	6.298308
C16orf71	0.966962	0.033038	1.53859	1.587222	10.00269	6.298308
HOXA-AS	0.966962	0.033038	1.53859	1.587222	10.00269	6.298308
LOC10012	0.966962	0.033038	1.53859	1.587222	10.00269	6.298308
LRRC17	0.966962	0.033038	1.53859	1.587222	10.00269	6.298308
MXRA8	0.966962	0.033038	1.53859	1.587222	10.00269	6.298308
PPP2R3C	0.966968	0.033032	0.755244	0.75487	281.1866	372.4999
U2AF1	0.96697	0.03303	0.698508	0.698455	2295.061	3285.917
RHOQ	0.966971	0.033029	0.706868	0.706796	1656	2342.971
ZNF439	0.966981	0.033019	1.317668	1.323782	39.16607	29.58405
CYP2R1	0.966988	0.033012	0.773539	0.772046	66.68457	86.3768
ASMTL-A	0.966999	0.033001	0.715172	0.70659	13.34803	18.89492
ZNF630	0.967018	0.032982	1.291167	1.292922	124.4779	96.27414
SC5D	0.967047	0.032953	1.44031	1.440397	4197.805	2914.335
MMP23A	0.967062	0.032938	1.859211	2.068258	4.923544	2.375362
P3H3	0.96707	0.03293	1.293838	1.296938	70.0188	53.9855

FPGT-TNI	0.967077	0.032923	1.914264	2.16943	4.501209	2.069444
TIGD5	0.967078	0.032922	0.750861	0.750555	354.5397	472.3731
LINC0046	0.967087	0.032913	1.583199	1.645779	8.891277	5.39855
TMEM185	0.967104	0.032896	0.743487	0.743251	470.1263	632.5301
STRC	0.967109	0.032891	0.714759	0.706002	13.33691	18.89492
LOC10065	0.967109	0.032891	0.757008	0.753257	27.78524	36.89009
ACTR3C	0.967121	0.032879	0.774296	0.772804	66.56232	86.13386
SNIP1	0.967123	0.032877	1.325057	1.325653	419.8572	316.7149
ITPKA	0.967128	0.032872	1.290508	1.292646	100.0269	77.37921
CCND2	0.967132	0.032868	1.503496	1.543284	11.1141	7.198066
LINC0031	0.967132	0.032868	1.503496	1.543284	11.1141	7.198066
ADNP	0.967163	0.032837	0.701721	0.701662	2022.765	2882.826
UBAP1	0.967177	0.032823	1.424689	1.4248	3160.882	2218.471
MVB12A	0.96718	0.03282	0.728677	0.728533	781.2987	1072.431
PADI2	0.967182	0.032818	0.728754	0.728608	762.427	1046.419
DUSP8	0.967184	0.032816	1.312248	1.313067	281.1866	214.1425
MIOS	0.967184	0.032816	0.75124	0.750928	341.2027	454.3779
NOP14	0.967195	0.032805	0.729899	0.729749	749.379	1026.903
VSIG1	0.967207	0.032793	0.647461	0.627045	6.157209	9.825361
FAM90A1	0.967208	0.032792	0.727856	0.720681	15.55973	21.5942
NT5DC3	0.967212	0.032788	0.759862	0.759427	238.9753	314.6815
CYP26A1	0.967253	0.032747	1.47568	1.509098	12.22551	8.097825
IL1RL1	0.967253	0.032747	1.47568	1.509098	12.22551	8.097825
MERTK	0.967263	0.032737	0.769898	0.769224	152.2631	197.9468
TRNAU1A	0.96728	0.03272	1.304534	1.305465	247.8443	189.849
ATAD2B	0.967288	0.032712	0.745192	0.744944	435.6726	584.8429
AIM1L	0.967297	0.032703	1.359106	1.35942	900.2418	662.2221
VMAC	0.967297	0.032703	0.77406	0.773087	102.2497	132.2645
UBE2W	0.967326	0.032674	1.408631	1.408774	2300.618	1633.061
SERPINB8	0.967329	0.032671	1.290212	1.293082	74.46444	57.58453
ERG	0.967361	0.032639	1.290178	1.291863	126.7007	98.07366
KDF1	0.96738	0.03262	0.74388	0.743646	462.3464	621.733
ADAM22	0.967408	0.032592	1.434381	1.459355	14.44832	9.897341
C10orf55	0.967408	0.032592	1.434381	1.459355	14.44832	9.897341
LAIR1	0.967411	0.032589	0.769622	0.768932	144.3943	187.7886
ATP2A1-A	0.967428	0.032572	0.775404	0.774343	93.3584	120.5676
SIX3	0.967431	0.032569	1.502801	1.543284	11.1141	7.198066
YKT6	0.967433	0.032567	1.469075	1.46913	7253.059	4936.974
NDUFC2-I	0.967436	0.032564	1.34776	1.348141	699.8324	519.1066
BOC	0.967448	0.032552	0.774041	0.772055	50.01343	64.7826
C1orf115	0.967448	0.032552	0.774041	0.772055	50.01343	64.7826
PDCD11	0.967452	0.032548	0.729304	0.729161	784.6552	1076.111
FAM27B	0.967459	0.032541	0.766119	0.763014	33.34229	43.70126
CDSN	0.967459	0.032541	1.41863	1.440695	15.55973	10.7971
LOC38990	0.967462	0.032538	1.34002	1.340456	594.6041	443.5808
NUDT16L1	0.967494	0.032506	0.754174	0.753841	316.7517	420.1871
PCDH12	0.967499	0.032501	1.405189	1.424904	16.67114	11.69686
C12orf49	0.967504	0.032496	0.697803	0.697753	2395.088	3432.578
LINC0093	0.967523	0.032477	1.475091	1.509098	12.22551	8.097825
PCBP1-AS	0.967523	0.032477	1.475091	1.509098	12.22551	8.097825
ZNF737	0.967529	0.032471	0.776245	0.775258	101.9718	131.5357
PSG3	0.96753	0.03247	0.765667	0.762541	32.55319	42.69353
USP12	0.96753	0.03247	1.374872	1.375112	1223.662	889.861
PAQR7	0.967536	0.032464	1.35667	1.356994	851.0174	627.1315
ACTR5	0.967543	0.032457	0.768753	0.768138	164.4886	214.1425
SEC14L1F	0.96755	0.03245	1.35977	1.372249	22.22819	16.19565

SKIDA1	0.96755	0.03245	1.35977	1.372249	22.22819	16.19565
POU2F1	0.967553	0.032447	1.350607	1.350966	752.4243	556.9504
FAM26F	0.967559	0.032441	1.383461	1.399633	18.89396	13.49637
KLHDC1	0.967559	0.032441	1.383461	1.399633	18.89396	13.49637
ZNF345	0.967559	0.032441	1.383461	1.399633	18.89396	13.49637
CIB4	0.967574	0.032426	0.640757	0.61804	5.557048	8.997583
CIRBP-AS	0.967574	0.032426	0.640757	0.61804	5.557048	8.997583
MTTP	0.967574	0.032426	0.640757	0.61804	5.557048	8.997583
SULT1E1	0.967574	0.032426	0.640757	0.61804	5.557048	8.997583
FOXS1	0.967582	0.032418	1.374555	1.389365	20.00537	14.39613
PPP4R4	0.967582	0.032418	1.374555	1.389365	20.00537	14.39613
GLUD1P3	0.967586	0.032414	1.452582	1.481742	13.33691	8.997583
HSD17B1	0.967586	0.032414	1.452582	1.481742	13.33691	8.997583
ZNRF2P1	0.967586	0.032414	1.452582	1.481742	13.33691	8.997583
ANO8	0.967592	0.032408	1.324553	1.325117	442.341	333.8103
PRTN3	0.967604	0.032396	1.366659	1.380304	21.11678	15.29589
ZC3H12D	0.967626	0.032374	0.64136	0.618658	5.557048	8.988585
SPAG8	0.967629	0.032371	1.433935	1.459355	14.44832	9.897341
ALDH1A2	0.967631	0.032369	1.342502	1.352686	25.56242	18.89492
RYK	0.967641	0.032359	0.691883	0.691843	3004.14	4342.234
LOC10012	0.967643	0.032357	1.353278	1.365042	23.3396	17.09541
IFIT5	0.967655	0.032345	0.765181	0.764676	202.2765	264.5289
FZD8	0.967661	0.032339	1.296593	1.297668	207.8336	160.157
HIST1H2E	0.967662	0.032338	1.372905	1.387631	20.00537	14.41413
URAHP	0.967662	0.032338	1.34756	1.358555	24.45101	17.99517
NUSAP1	0.967666	0.032334	1.417662	1.417781	2882.996	2033.454
SCART1	0.967681	0.032319	1.404834	1.424904	16.67114	11.69686
FAAHP1	0.967681	0.032319	1.34237	1.352686	25.56242	18.89492
AIFM2	0.967701	0.032299	0.773254	0.772487	130.0349	168.3358
PGPEP1	0.967704	0.032296	1.409874	1.41001	2432.231	1724.972
DISC1-IT1	0.967707	0.032293	0.699485	0.687727	10.00269	14.54909
CEACAM2	0.967716	0.032284	1.501647	1.543648	10.49171	6.793175
GLI3	0.967731	0.032269	0.768822	0.765895	34.4537	44.98792
PHYHD1	0.967734	0.032266	1.524942	1.572963	9.969344	6.334298
BROX	0.967736	0.032264	1.370577	1.370829	1147.03	836.7392
ESD	0.967742	0.032258	0.719369	0.719268	1140.306	1585.374
EXOSC10	0.967747	0.032253	0.717097	0.717004	1234.776	1722.137
RSAD2	0.967748	0.032252	1.347359	1.358555	24.45101	17.99517
WDR97	0.967748	0.032252	1.347359	1.358555	24.45101	17.99517
GABRP	0.967762	0.032238	1.325658	1.333901	30.00806	22.49396
KLK1	0.967762	0.032238	1.325658	1.333901	30.00806	22.49396
LOC10050	0.967762	0.032238	1.325658	1.333901	30.00806	22.49396
CHCHD6	0.967785	0.032215	0.752287	0.751988	354.5397	471.4734
RFC5	0.967789	0.032211	0.727709	0.727579	882.4592	1212.874
TLR6	0.967802	0.032198	1.32366	1.33171	30.48596	22.88985
MAGEF1	0.967803	0.032197	0.75662	0.75627	300.0806	396.7934
PARD6G	0.967806	0.032194	1.319116	1.326595	32.23088	24.29347
LINC0011	0.96781	0.03219	0.622011	0.593633	4.434524	7.476992
FBXL17	0.96782	0.03218	0.731895	0.731743	726.8619	993.3332
VAPA	0.967821	0.032179	0.69924	0.699188	2333.96	3338.103
CHCHD5	0.967835	0.032165	0.775794	0.774912	112.2524	144.8611
MIR4458H	0.967846	0.032154	0.771341	0.770711	162.2658	210.5434
FAM102B	0.967855	0.032145	1.313457	1.320297	34.4537	26.09299
GCH1	0.967873	0.032127	1.364901	1.365174	1039.168	761.1955
ZMYND15	0.96788	0.03212	1.310904	1.317462	35.56511	26.99275
KDM4D	0.967887	0.032113	0.777814	0.776011	54.45907	70.18115

ZFPM1	0.967887	0.032113	0.777814	0.776011	54.45907	70.18115
CKLF-CM1	0.967888	0.032112	0.303261	0.007454	0	1.331642
PYGO2	0.967914	0.032086	0.721024	0.72092	1093.627	1516.993
IDH3A	0.96792	0.03208	0.71516	0.715073	1341.471	1875.996
LINC0144	0.967922	0.032078	0.616004	0.58673	4.456752	7.602958
ADM5	0.967924	0.032076	1.306156	1.312325	37.78793	28.79227
NFE2	0.967924	0.032076	0.771879	0.769155	36.67652	47.68719
KIAA1324	0.967925	0.032075	0.754198	0.749391	22.22819	29.66503
ATXN7L1	0.967928	0.032072	0.776463	0.775625	120.0322	154.7584
SLC16A14	0.967944	0.032056	0.64116	0.61804	5.557048	8.997583
C17orf100	0.967962	0.032038	1.304156	1.309989	38.89934	29.69202
HERC2P2	0.967981	0.032019	0.711015	0.71094	1558.652	2192.387
ALOXE3	0.967991	0.032009	1.302167	1.307791	40.01074	30.59178
LOC10192	0.968	0.032	0.704957	0.693617	10.26942	14.81002
ZNF890P	0.968005	0.031995	0.613184	0.582908	4.312269	7.405011
APLF	0.968009	0.031991	0.775454	0.774651	123.3665	159.2572
PCDHB6	0.968016	0.031984	1.540204	1.595353	8.891277	5.569504
SUN1	0.968017	0.031983	1.4202	1.420311	3113.058	2191.811
CPTP	0.968018	0.031982	0.736936	0.736762	635.7263	862.8682
SP6	0.96802	0.03198	1.300289	1.305719	41.12215	31.49154
SYCP2L	0.968023	0.031977	0.76735	0.766847	205.6108	268.128
SSC4D	0.968034	0.031966	0.702975	0.702919	2133.906	3035.785
MISP	0.968036	0.031964	1.354953	1.355269	865.7881	638.8284
C11orf63	0.968046	0.031954	0.778507	0.776467	48.90202	62.98308
DNAH7	0.968076	0.031924	0.680935	0.665411	7.779867	11.69686
ITGA10	0.968076	0.031924	0.680935	0.665411	7.779867	11.69686
LRRIQ3	0.968076	0.031924	0.680935	0.665411	7.779867	11.69686
UGDH-AS	0.968081	0.031919	0.702094	0.702039	2153.445	3067.42
LOC90246	0.968082	0.031918	0.779218	0.778118	88.91277	114.2693
DMRTA1	0.968082	0.031918	1.296833	1.30191	43.34497	33.29106
PTPN4	0.968084	0.031916	0.738359	0.738178	609.0525	825.0784
EMP2	0.968086	0.031914	0.737703	0.737529	642.9504	871.7668
INO80C	0.968086	0.031914	0.778794	0.777756	94.46981	121.4674
DCAF8	0.968088	0.031912	0.709838	0.709766	1639.329	2309.68
TPTE2P1	0.968088	0.031912	1.930502	2.228939	4.023303	1.799517
LRRC24	0.968113	0.031887	0.774612	0.772065	38.89934	50.38647
LOC39981	0.968114	0.031886	1.295239	1.300156	44.45638	34.19082
UBE2H	0.968116	0.031884	1.501519	1.501549	14149.36	9423.169
LINC0050	0.968125	0.031875	0.643527	0.620514	5.568162	8.979588
ZC3H13	0.968129	0.031871	0.720189	0.720089	1148.086	1594.372
STPG1	0.968161	0.031839	1.337747	1.33816	635.7263	475.0724
BAG1	0.968217	0.031783	0.741948	0.741748	544.5907	734.2028
FAM71F2	0.968219	0.031781	0.67057	0.652566	6.7018	10.27524
ZNF24	0.968239	0.031761	0.714192	0.714111	1427.05	1998.363
ACTB	0.968249	0.031751	1.595559	1.595565	90220.28	56544.41
GALNT4	0.968256	0.031744	0.779181	0.778203	100.1047	128.6384
TRMT2B	0.968258	0.031742	1.338245	1.338655	628.3132	469.3589
RNF139-A	0.968259	0.031741	1.497787	1.541488	9.991572	6.47826
C11orf31	0.968271	0.031729	1.349082	1.349422	787.9894	583.9431
GBX2	0.968284	0.031716	1.288364	1.292608	50.01343	38.68961
DUSP3	0.968286	0.031714	0.710748	0.710676	1659.335	2334.873
TCAM1P	0.968304	0.031696	0.681247	0.665554	7.835438	11.77784
LINC0150	0.968339	0.031661	0.706629	0.695029	10.00269	14.39613
TOB1-AS1	0.968339	0.031661	0.706629	0.695029	10.00269	14.39613
FAM127C	0.968348	0.031652	0.755954	0.755644	344.8482	456.3664
PPP1R15	0.968348	0.031652	1.461326	1.461383	6732.919	4607.221

SRRM5	0.96836	0.03164	0.681214	0.665411	7.779867	11.69686
ZMYND10	0.96836	0.03164	0.681214	0.665411	7.779867	11.69686
MXD4	0.968361	0.031639	1.319106	1.319681	416.7786	315.8152
SPIN2A	0.968362	0.031638	1.541949	1.598871	8.824592	5.515518
ZMYM2	0.96837	0.03163	0.705967	0.705906	1923.85	2725.368
RCBTB2	0.968376	0.031624	0.76346	0.763045	247.8443	324.8127
CFHR1	0.968387	0.031613	3.051751	119.9208	1.189208	0
C11orf45	0.968389	0.031611	1.282833	1.286633	55.57048	43.1884
ENO3	0.968389	0.031611	1.282833	1.286633	55.57048	43.1884
DPCD	0.968394	0.031606	0.762571	0.762171	257.847	338.3091
DNAJC19	0.968416	0.031584	0.744499	0.744289	525.6967	706.3103
PPP2R1B	0.968432	0.031568	1.370594	1.370829	1227.252	895.2595
IMP3	0.968436	0.031564	0.750223	0.749966	415.6672	554.2511
ATP5G2	0.968456	0.031544	0.69826	0.698214	2614.035	3743.894
FBXW10	0.968468	0.031532	0.697171	0.683803	8.813478	12.89354
HDHD3	0.96847	0.03153	0.758581	0.758239	305.6376	403.0917
LINC0096	0.968486	0.031514	0.565161	0.516189	2.600698	5.047644
ZNF112	0.968501	0.031499	0.782504	0.780921	61.12753	78.27897
ADAMTS8	0.968507	0.031493	0.724475	0.7153	12.22551	17.09541
KCNQ1	0.968507	0.031493	0.724475	0.7153	12.22551	17.09541
UBAC2-AS	0.968507	0.031493	0.724475	0.7153	12.22551	17.09541
ALG8	0.968533	0.031467	0.747927	0.747692	456.7893	610.9359
HOXC6	0.968544	0.031456	1.277411	1.280142	74.05322	57.84546
MICALCL	0.968545	0.031455	1.28102	1.284577	57.7933	44.98792
BREA2	0.968562	0.031438	0.70684	0.695029	10.00269	14.39613
FAM110A	0.968587	0.031413	1.320551	1.321093	444.5638	336.5096
AIP	0.968591	0.031409	1.367001	1.367246	1162.534	850.2716
SIX1	0.968596	0.031404	1.292134	1.293116	223.3933	172.7536
CROCCP2	0.9686	0.0314	1.27673	1.278791	97.80404	76.47946
MKKS	0.968609	0.031391	0.716036	0.715953	1383.705	1932.681
LINC0047	0.96861	0.03139	0.768471	0.764764	26.84054	35.09957
M6PR	0.968611	0.031389	0.694399	0.694358	3041.928	4380.923
HSF4	0.968619	0.031381	1.281076	1.284607	57.47099	44.73598
SLC1A4	0.968621	0.031379	0.715245	0.715164	1462.615	2045.151
RHCG	0.968633	0.031367	0.737593	0.730046	14.44832	19.79468
ZFHX4-AS	0.968633	0.031367	0.737593	0.730046	14.44832	19.79468
C7orf55-LU	0.968635	0.031365	0.721454	0.721355	1151.409	1596.18
GMIP	0.968645	0.031355	1.336985	1.337386	640.1719	478.6714
LOC10050	0.968663	0.031337	1.278459	1.281785	61.12753	47.68719
TPBGL	0.968663	0.031337	1.278459	1.281785	61.12753	47.68719
OTUD6A	0.968668	0.031332	0.660596	0.639677	5.901585	9.23152
NOX4	0.968683	0.031317	0.724643	0.7153	12.22551	17.09541
MPI	0.968692	0.031308	0.741906	0.741716	574.5988	774.6919
RBM8A	0.968692	0.031308	0.690733	0.690697	3411.094	4938.629
PTPMT1	0.9687	0.0313	0.738753	0.738582	641.2833	868.2668
SYNGR3	0.968722	0.031278	1.29155	1.292519	225.6161	174.5531
GPR87	0.96873	0.03127	0.782763	0.781689	90.02418	115.1691
LOC38824	0.968734	0.031266	1.87865	2.155235	3.889934	1.799517
LOC61303	0.968734	0.031266	1.87865	2.155235	3.889934	1.799517
RAB30	0.968745	0.031255	1.309802	1.310443	367.8766	280.7246
RAD9A	0.968767	0.031233	0.756676	0.75637	342.3142	452.5784
LDLRAP1	0.968767	0.031233	0.740777	0.740595	602.384	813.3815
CCDC142	0.968769	0.031231	0.757286	0.756973	334.7343	442.2042
AHNAK2	0.968772	0.031228	1.37264	1.372861	1319.243	960.9419
TRMT10A	0.968779	0.031221	0.780623	0.779755	112.2524	143.9613
KLRG1	0.968801	0.031199	0.550857	0.494228	2.222819	4.507789

LOC10192	0.968801	0.031199	0.550857	0.494228	2.222819	4.507789
UBE2R2	0.968814	0.031186	1.402122	1.402261	2313.955	1650.157
FAM47E-S	0.968815	0.031185	0.774699	0.771485	30.79716	39.92228
FAM157A	0.968817	0.031183	1.825445	2.045356	4.445638	2.168418
ID4	0.968818	0.031182	0.682005	0.681979	4763.501	6984.824
MB	0.968826	0.031174	0.783156	0.781138	47.79061	61.18356
ZNF76	0.968827	0.031173	0.753514	0.753245	401.2189	532.6569
C12orf56	0.968843	0.031157	0.755585	0.750061	18.89396	25.19323
KLHL12	0.968848	0.031152	0.713481	0.713405	1543.748	2163.919
MAGEA11	0.968849	0.031151	0.747759	0.741254	16.67114	22.49396
KRTCAP3	0.968858	0.031142	0.758533	0.758217	335.6457	442.6811
RPP25L	0.968863	0.031137	0.773155	0.772607	185.6054	240.2355
MAP3K14	0.96887	0.03113	1.338329	1.338711	675.737	504.7644
BAALC-AS	0.968875	0.031125	0.551833	0.495215	2.222819	4.498792
C15orf56	0.968875	0.031125	0.551833	0.495215	2.222819	4.498792
CECR5-AS	0.968875	0.031125	0.551833	0.495215	2.222819	4.498792
CEMIP	0.968875	0.031125	0.551833	0.495215	2.222819	4.498792
CLDN10-A	0.968875	0.031125	0.551833	0.495215	2.222819	4.498792
CRAT	0.968875	0.031125	0.551833	0.495215	2.222819	4.498792
GAS5-AS1	0.968875	0.031125	0.551833	0.495215	2.222819	4.498792
HEXA-AS1	0.968875	0.031125	0.551833	0.495215	2.222819	4.498792
HIPK1-AS	0.968875	0.031125	0.551833	0.495215	2.222819	4.498792
HIST1H4C	0.968875	0.031125	0.551833	0.495215	2.222819	4.498792
KLF14	0.968875	0.031125	0.551833	0.495215	2.222819	4.498792
LDLRAD1	0.968875	0.031125	0.551833	0.495215	2.222819	4.498792
LIFR-AS1	0.968875	0.031125	0.551833	0.495215	2.222819	4.498792
LINC0094	0.968875	0.031125	0.551833	0.495215	2.222819	4.498792
LOC10012	0.968875	0.031125	0.551833	0.495215	2.222819	4.498792
LOC10050	0.968875	0.031125	0.551833	0.495215	2.222819	4.498792
LOC10192	0.968875	0.031125	0.551833	0.495215	2.222819	4.498792
MAN1A1	0.968875	0.031125	0.551833	0.495215	2.222819	4.498792
NAT2	0.968875	0.031125	0.551833	0.495215	2.222819	4.498792
TTLL13P	0.968875	0.031125	0.551833	0.495215	2.222819	4.498792
U2AF1L4	0.96889	0.03111	0.779844	0.779052	123.3665	158.3575
TTC39C	0.968891	0.031109	0.748089	0.747863	473.7161	633.4298
RELL1	0.968891	0.031109	1.301504	1.302246	306.838	235.6197
MLLT4-AS	0.968891	0.031109	1.304766	1.3123	30.79716	23.4657
LIN7C	0.968919	0.031081	1.32252	1.323021	485.686	367.1014
USP30	0.968926	0.031074	0.7637	0.763316	267.8497	350.9057
RPL39L	0.968936	0.031064	1.342193	1.342548	736.8646	548.8526
PTPRU	0.968939	0.031061	1.374748	1.37496	1367.034	994.2329
UTP3	0.968942	0.031058	0.737069	0.73691	696.8538	945.646
ZC3H6	0.968982	0.031018	0.778582	0.777895	145.5947	187.1677
MMP25-AS	0.968996	0.031004	0.784845	0.782927	50.01343	63.88284
LLPH-AS1	0.969007	0.030993	1.2841	1.288966	42.85595	33.24607
RPL23AP5	0.969011	0.030989	0.755394	0.75511	370.6329	490.8362
DNASE1	0.969022	0.030978	0.785492	0.783696	53.29209	68.00373
LOC10099	0.969043	0.030957	0.767345	0.763014	23.3396	30.59178
FLVCR1-A	0.969059	0.030941	0.782603	0.780189	40.01074	51.28622
TAMM41	0.969065	0.030935	0.762708	0.762353	295.6349	387.7958
SMIM14	0.969084	0.030916	1.360217	1.360478	1070.321	786.7217
KDM4A-AS	0.969086	0.030914	0.760983	0.755842	20.00537	26.47089
PRH1-PRI	0.969086	0.030914	1.282607	1.287564	42.58922	33.07512
QRSL1	0.969101	0.030899	0.752877	0.752619	410.3658	545.2535
RPS14P3	0.969109	0.030891	1.443384	1.476915	11.1141	7.521979
LINC0047	0.969116	0.030884	0.783244	0.782329	105.5839	134.9637

LOC10192	0.969126	0.030874	0.313595	0.007821	0	1.268659
RIPPLY3	0.96913	0.03087	0.784679	0.782521	44.4675	56.82873
MINK1	0.969137	0.030863	1.400596	1.400734	2313.955	1651.956
ARHGAP1	0.969158	0.030842	1.397111	1.397256	2231.244	1596.873
STAG3L5I	0.969162	0.030838	0.786558	0.784554	48.47969	61.7954
ARPC5L	0.969167	0.030833	1.405605	1.405733	2547.351	1812.113
RNF25	0.969175	0.030825	0.747293	0.74708	503.4685	673.919
IFI35	0.969198	0.030802	0.769504	0.769039	214.502	278.9251
LNPEP	0.969204	0.030796	1.380856	1.381041	1652.666	1196.679
F11R	0.96921	0.03079	1.481733	1.481771	10586.04	7144.18
ATP5L2	0.969217	0.030783	2.942671	112.141	1.11141	0
CD80	0.969217	0.030783	2.942671	112.141	1.11141	0
ERICH4	0.969217	0.030783	2.942671	112.141	1.11141	0
ERICH6	0.969217	0.030783	2.942671	112.141	1.11141	0
GJC3	0.969217	0.030783	2.942671	112.141	1.11141	0
H2BFM	0.969217	0.030783	2.942671	112.141	1.11141	0
IQCA1L	0.969217	0.030783	2.942671	112.141	1.11141	0
JSRP1	0.969217	0.030783	2.942671	112.141	1.11141	0
KCNA10	0.969217	0.030783	2.942671	112.141	1.11141	0
KCNK4	0.969217	0.030783	2.942671	112.141	1.11141	0
LHFPL5	0.969217	0.030783	2.942671	112.141	1.11141	0
LINC0058	0.969217	0.030783	2.942671	112.141	1.11141	0
LINC0115	0.969217	0.030783	2.942671	112.141	1.11141	0
LINC0140	0.969217	0.030783	2.942671	112.141	1.11141	0
LINC0147	0.969217	0.030783	2.942671	112.141	1.11141	0
LOC10050	0.969217	0.030783	2.942671	112.141	1.11141	0
MGAT4C	0.969217	0.030783	2.942671	112.141	1.11141	0
MYCT1	0.969217	0.030783	2.942671	112.141	1.11141	0
MYH6	0.969217	0.030783	2.942671	112.141	1.11141	0
NCKAP5	0.969217	0.030783	2.942671	112.141	1.11141	0
NR1H4	0.969217	0.030783	2.942671	112.141	1.11141	0
PLPP4	0.969217	0.030783	2.942671	112.141	1.11141	0
RGS6	0.969217	0.030783	2.942671	112.141	1.11141	0
RPS2P32	0.969217	0.030783	2.942671	112.141	1.11141	0
SAA3P	0.969217	0.030783	2.942671	112.141	1.11141	0
SSMEM1	0.969217	0.030783	2.942671	112.141	1.11141	0
SSX1	0.969217	0.030783	2.942671	112.141	1.11141	0
TCP11	0.969217	0.030783	2.942671	112.141	1.11141	0
TEX28	0.969217	0.030783	2.942671	112.141	1.11141	0
TMEM132	0.969217	0.030783	2.942671	112.141	1.11141	0
TMEM74	0.969217	0.030783	2.942671	112.141	1.11141	0
ZNF350-A	0.969217	0.030783	2.942671	112.141	1.11141	0
ZNF571-A	0.969217	0.030783	2.942671	112.141	1.11141	0
ZNF732	0.969217	0.030783	2.942671	112.141	1.11141	0
ZNF99	0.969217	0.030783	2.942671	112.141	1.11141	0
ARTN	0.969244	0.030756	1.285014	1.286075	196.7195	152.9589
RIBC2	0.96925	0.03075	0.775633	0.772083	27.78524	35.99033
LOC10012	0.969252	0.030748	0.488585	0.394499	1.211436	3.086171
TRAF5	0.969258	0.030742	0.784103	0.783186	104.9949	134.064
LOC10192	0.969277	0.030723	0.724172	0.713889	11.10298	15.55682
KLK4	0.969277	0.030723	0.786497	0.784312	44.45638	56.68477
MMP14	0.969278	0.030722	1.333741	1.334135	644.6176	483.1702
BST2	0.969294	0.030706	1.392879	1.393033	1991.646	1429.716
LINC0026	0.969318	0.030682	0.490413	0.396935	1.222551	3.095169
SMPDL3B	0.969319	0.030681	1.340254	1.3406	763.5384	569.547
ACTR8	0.969322	0.030678	0.740798	0.740627	639.0605	862.8682

EPHB3	0.969329	0.030671	0.783435	0.782584	113.3638	144.8611
LOC10192	0.969338	0.030662	2.97505	115.4752	1.144752	0
GOLGA8D	0.969342	0.030658	1.451606	1.487742	10.78067	7.243054
EPSTI1	0.969344	0.030656	1.322794	1.323258	534.588	403.9915
ANKRD18	0.969365	0.030635	1.465998	1.506852	9.780404	6.487257
CALHM2	0.969372	0.030628	1.268057	1.270481	80.02149	62.98308
XRRA1	0.969373	0.030627	0.756004	0.755728	380.1021	502.9649
P4HA2	0.969395	0.030605	1.432681	1.432763	4296.709	2998.894
SIAH1	0.969397	0.030603	0.73834	0.738184	721.3048	977.1375
LOC10050	0.969397	0.030603	2.242892	3.276682	2.233933	0.674819
FOXL2	0.969407	0.030593	1.266255	1.268576	84.46713	66.58211
TRPV1	0.969417	0.030583	0.78455	0.783664	108.5069	138.4638
PARPBP	0.969419	0.030581	0.765349	0.764972	271.1839	354.5048
CCT6P1	0.969423	0.030577	0.783824	0.781157	36.05413	46.1576
SPCS2	0.96943	0.03057	0.7058	0.705744	2176.14	3083.472
SYT5	0.969443	0.030557	0.781844	0.778786	32.23088	41.38888
NRXN1	0.969449	0.030551	0.55278	0.495215	2.222819	4.498792
RPS7P5	0.969449	0.030551	0.55278	0.495215	2.222819	4.498792
RSPH14	0.969449	0.030551	0.55278	0.495215	2.222819	4.498792
SEMA6A	0.969449	0.030551	0.55278	0.495215	2.222819	4.498792
CT45A4	0.969452	0.030548	2.923244	111.0295	1.100295	0
AICDA	0.969454	0.030546	1.899316	2.237539	3.334229	1.484601
LOC10013	0.969466	0.030534	0.781789	0.778786	32.23088	41.38888
GRAMD4	0.969483	0.030517	0.753005	0.752755	417.89	555.1509
FZD5	0.969495	0.030505	1.35144	1.351727	941.3639	696.4129
PYGB	0.969498	0.030502	1.411501	1.411614	2926.341	2073.043
PSMD5-AS	0.969499	0.030501	0.780202	0.779527	144.4832	185.3502
PRRG1	0.969501	0.030499	1.396264	1.396407	2205.037	1579.076
KPTN	0.969512	0.030488	1.268844	1.270495	120.0322	94.47462
PEX16	0.969522	0.030478	0.770569	0.770121	224.5047	291.5217
ZNF131	0.969523	0.030477	0.736144	0.735999	763.5384	1037.421
NEURL1	0.969533	0.030467	1.269857	1.271532	116.698	91.77535
NOXA1	0.969554	0.030446	0.789684	0.787718	48.90202	62.08332
LINC0062	0.969565	0.030435	0.554964	0.49768	2.233933	4.498792
UCKL1	0.969565	0.030435	0.750064	0.749841	479.0175	638.8284
CES3	0.969571	0.030429	0.790635	0.788948	56.81526	72.01665
MRPL45	0.969575	0.030425	0.749938	0.749719	497.6114	663.7337
ATG4A	0.96958	0.03042	1.339832	1.340179	737.976	550.6521
GANC	0.969585	0.030415	1.269523	1.27118	117.8094	92.67511
BMS1P4	0.969604	0.030396	0.787151	0.784643	38.64371	49.25277
PFN2	0.969613	0.030387	0.730589	0.730468	928.027	1270.459
POLE3	0.969614	0.030386	1.400483	1.400616	2409.536	1720.338
MTA3	0.969618	0.030382	0.726626	0.726519	1060.285	1459.408
TRPV4	0.969637	0.030363	1.280144	1.281245	185.6054	144.8611
POLR3F	0.969645	0.030355	0.767637	0.767251	267.8497	349.1062
CDRT1	0.969655	0.030345	1.267888	1.271445	54.53687	42.89148
C19orf81	0.969672	0.030328	0.786225	0.785328	106.6953	135.8635
RNF41	0.969682	0.030318	0.716604	0.716526	1495.457	2087.097
TMEM52	0.969694	0.030306	0.789867	0.78847	66.68457	84.57728
GTF2A1	0.96972	0.03028	0.714507	0.714435	1611.544	2255.694
SNX33	0.96972	0.03028	1.332773	1.333153	665.7343	499.3659
RFX1	0.969726	0.030274	1.287026	1.287904	244.5101	189.849
KIAA0825	0.969751	0.030249	0.645301	0.617376	4.456752	7.225059
ARFIP1	0.969758	0.030242	1.342152	1.342476	806.8834	601.0385
ME2	0.969767	0.030233	0.741024	0.740859	665.312	898.0308
ZBTB10	0.969768	0.030232	0.734114	0.73398	820.2203	1117.5

IBA57	0.969769	0.030231	0.790532	0.788837	54.77026	69.43435
VN1R2	0.969775	0.030225	2.995547	118.8094	1.178094	0
TMEM131	0.969778	0.030222	0.724082	0.723984	1172.537	1619.565
APOBEC3	0.969783	0.030217	0.78255	0.779277	29.47458	37.82584
BAD	0.969786	0.030214	0.73718	0.737035	757.9813	1028.424
RNF113A	0.969787	0.030213	0.752439	0.752205	453.4551	602.8381
KRT16P3	0.969803	0.030197	2.936699	113.2524	1.122524	0
USP17L1	0.969803	0.030197	2.936699	113.2524	1.122524	0
NPR1	0.969808	0.030192	0.788533	0.786099	38.89934	49.48671
RGS3	0.969812	0.030188	1.361217	1.361455	1174.76	862.8682
DMXL2	0.969818	0.030182	0.729762	0.729646	973.5948	1334.342
C1orf101	0.969824	0.030176	0.646112	0.618146	4.445638	7.198066
CATSPER	0.969824	0.030176	0.646112	0.618146	4.445638	7.198066
CXXC4	0.969824	0.030176	0.646112	0.618146	4.445638	7.198066
DPY19L2	0.969824	0.030176	0.646112	0.618146	4.445638	7.198066
EID3	0.969824	0.030176	0.646112	0.618146	4.445638	7.198066
FGFBP3	0.969824	0.030176	0.646112	0.618146	4.445638	7.198066
SYPL2	0.969824	0.030176	0.646112	0.618146	4.445638	7.198066
ANKS6	0.969873	0.030127	1.374875	1.375065	1573.756	1144.493
CDC20	0.969877	0.030123	1.431223	1.431304	4356.726	3043.882
KIAA1033	0.969883	0.030117	0.719022	0.71894	1403.71	1952.476
DHX16	0.969889	0.030111	0.712754	0.712687	1737.133	2437.445
FSD1	0.96989	0.03011	0.782809	0.782118	140.0376	179.0519
NAGLU	0.969902	0.030098	0.772478	0.772027	222.2819	287.9227
ZNF664-F	0.969928	0.030072	1.920569	2.275891	3.412027	1.493599
SPTLC1	0.969935	0.030065	1.404334	1.404455	2734.345	1946.906
TXLNG	0.969942	0.030058	0.718035	0.717956	1459.281	2032.554
SMIM11B	0.969964	0.030036	0.767244	0.766874	274.6182	358.1038
CLIC4	0.969976	0.030024	1.507901	1.507924	18492.74	12263.71
BISPR	0.969995	0.030005	1.273621	1.274828	164.5108	129.0433
GSG1	0.969995	0.030005	0.776746	0.77247	22.96172	29.72801
TMEM183	0.970002	0.029998	1.399254	1.399383	2504.606	1789.79
KLF5	0.970007	0.029993	0.703972	0.703922	2438.433	3464.069
FOXJ1	0.970016	0.029984	0.691811	0.674092	6.668457	9.897341
LINC0101	0.970016	0.029984	0.691811	0.674092	6.668457	9.897341
LOC10028	0.970016	0.029984	0.691811	0.674092	6.668457	9.897341
MGP	0.970016	0.029984	0.691811	0.674092	6.668457	9.897341
ZDHHC19	0.970016	0.029984	0.691811	0.674092	6.668457	9.897341
EBPL	0.970022	0.029978	1.315934	1.316412	504.5799	383.297
ALOX12-A	0.970025	0.029975	0.75864	0.752	15.55973	20.69444
METTL12	0.970025	0.029975	0.75864	0.752	15.55973	20.69444
LOC28486	0.970029	0.029971	2.08451	2.75688	2.5229	0.908756
ABCA6	0.970033	0.029967	2.917524	12.40958	1.11141	0.908756
ABCD2	0.970033	0.029967	2.917524	112.141	1.11141	0
ACOT6	0.970033	0.029967	2.917524	112.141	1.11141	0
ADCY8	0.970033	0.029967	2.917524	112.141	1.11141	0
AGPAT4-I	0.970033	0.029967	2.917524	112.141	1.11141	0
AMPD1	0.970033	0.029967	2.917524	112.141	1.11141	0
ANGPTL7	0.970033	0.029967	2.917524	112.141	1.11141	0
ANKRD30	0.970033	0.029967	2.917524	112.141	1.11141	0
ANKS1B	0.970033	0.029967	2.917524	112.141	1.11141	0
ANP32C	0.970033	0.029967	2.917524	112.141	1.11141	0
AOX2P	0.970033	0.029967	2.917524	112.141	1.11141	0
AQP10	0.970033	0.029967	2.917524	112.141	1.11141	0
ARHGAP2	0.970033	0.029967	2.917524	112.141	1.11141	0
ASB12	0.970033	0.029967	2.917524	112.141	1.11141	0

ASB16	0.970033	0.029967	2.917524	112.141	1.11141	0
ASTN1	0.970033	0.029967	2.917524	112.141	1.11141	0
ATP13A5	0.970033	0.029967	2.917524	112.141	1.11141	0
ATP8B5P	0.970033	0.029967	2.917524	112.141	1.11141	0
B3GAT2	0.970033	0.029967	2.917524	112.141	1.11141	0
BAAT	0.970033	0.029967	2.917524	112.141	1.11141	0
BATF	0.970033	0.029967	2.917524	112.141	1.11141	0
BCAR4	0.970033	0.029967	2.917524	112.141	1.11141	0
BEAN1-AS	0.970033	0.029967	2.917524	112.141	1.11141	0
BPIFA2	0.970033	0.029967	2.917524	112.141	1.11141	0
BPIFB2	0.970033	0.029967	2.917524	112.141	1.11141	0
BTBD16	0.970033	0.029967	2.917524	112.141	1.11141	0
BTBD17	0.970033	0.029967	2.917524	112.141	1.11141	0
C10orf62	0.970033	0.029967	2.917524	112.141	1.11141	0
C19orf71	0.970033	0.029967	2.917524	112.141	1.11141	0
C1orf105	0.970033	0.029967	2.917524	112.141	1.11141	0
C3orf20	0.970033	0.029967	2.917524	112.141	1.11141	0
C6orf58	0.970033	0.029967	2.917524	112.141	1.11141	0
C9orf131	0.970033	0.029967	2.917524	112.141	1.11141	0
CA7	0.970033	0.029967	2.917524	112.141	1.11141	0
CACNG1	0.970033	0.029967	2.917524	112.141	1.11141	0
CADM3	0.970033	0.029967	2.917524	112.141	1.11141	0
CALCR	0.970033	0.029967	2.917524	112.141	1.11141	0
CALN1	0.970033	0.029967	2.917524	112.141	1.11141	0
CAPN8	0.970033	0.029967	2.917524	112.141	1.11141	0
CASP14	0.970033	0.029967	2.917524	112.141	1.11141	0
CATSPER	0.970033	0.029967	2.917524	112.141	1.11141	0
CBLN2	0.970033	0.029967	2.917524	112.141	1.11141	0
CCL7	0.970033	0.029967	2.917524	112.141	1.11141	0
CD81-AS1	0.970033	0.029967	2.917524	112.141	1.11141	0
CEACAM2	0.970033	0.029967	2.917524	112.141	1.11141	0
CELA2A	0.970033	0.029967	2.917524	112.141	1.11141	0
CERS3	0.970033	0.029967	2.917524	112.141	1.11141	0
CFAP46	0.970033	0.029967	2.917524	112.141	1.11141	0
CH25H	0.970033	0.029967	2.917524	112.141	1.11141	0
CHIT1	0.970033	0.029967	2.917524	112.141	1.11141	0
CHRM2	0.970033	0.029967	2.917524	112.141	1.11141	0
CHRNA4	0.970033	0.029967	2.917524	112.141	1.11141	0
CIDEB	0.970033	0.029967	2.917524	112.141	1.11141	0
CLDN14	0.970033	0.029967	2.917524	112.141	1.11141	0
CLDN2	0.970033	0.029967	2.917524	112.141	1.11141	0
CLDN8	0.970033	0.029967	2.917524	112.141	1.11141	0
CLEC19A	0.970033	0.029967	2.917524	112.141	1.11141	0
CLEC4D	0.970033	0.029967	2.917524	112.141	1.11141	0
CLEC9A	0.970033	0.029967	2.917524	112.141	1.11141	0
CLPSL1	0.970033	0.029967	2.917524	112.141	1.11141	0
CMKLR1	0.970033	0.029967	2.917524	112.141	1.11141	0
CNPY1	0.970033	0.029967	2.917524	112.141	1.11141	0
CNRIP1	0.970033	0.029967	2.917524	112.141	1.11141	0
COL4A6	0.970033	0.029967	2.917524	112.141	1.11141	0
CPB2	0.970033	0.029967	2.917524	112.141	1.11141	0
CPEB1-AS	0.970033	0.029967	2.917524	112.141	1.11141	0
CRYBA2	0.970033	0.029967	2.917524	112.141	1.11141	0
CSE1L-AS	0.970033	0.029967	2.917524	112.141	1.11141	0
CST2	0.970033	0.029967	2.917524	112.141	1.11141	0
CST7	0.970033	0.029967	2.917524	112.141	1.11141	0

CTC-436P	0.970033	0.029967	2.917524	112.141	1.11141	0
CXorf65	0.970033	0.029967	2.917524	112.141	1.11141	0
CYP2C8	0.970033	0.029967	2.917524	112.141	1.11141	0
CYP4F8	0.970033	0.029967	2.917524	112.141	1.11141	0
DCLK1	0.970033	0.029967	2.917524	112.141	1.11141	0
DGKG	0.970033	0.029967	2.917524	112.141	1.11141	0
DNASE2B	0.970033	0.029967	2.917524	112.141	1.11141	0
DPEP1	0.970033	0.029967	2.917524	112.141	1.11141	0
DPYS	0.970033	0.029967	2.917524	112.141	1.11141	0
DUOXA1	0.970033	0.029967	2.917524	112.141	1.11141	0
ELMO1	0.970033	0.029967	2.917524	112.141	1.11141	0
EPHA5	0.970033	0.029967	2.917524	112.141	1.11141	0
EPHX3	0.970033	0.029967	2.917524	112.141	1.11141	0
EQTN	0.970033	0.029967	2.917524	112.141	1.11141	0
EVI2A	0.970033	0.029967	2.917524	112.141	1.11141	0
EYA1	0.970033	0.029967	2.917524	112.141	1.11141	0
F10-AS1	0.970033	0.029967	2.917524	112.141	1.11141	0
FAM19A5	0.970033	0.029967	2.917524	112.141	1.11141	0
FAM83C-A	0.970033	0.029967	2.917524	112.141	1.11141	0
FAM90A1(	0.970033	0.029967	2.917524	112.141	1.11141	0
FAM92B	0.970033	0.029967	2.917524	112.141	1.11141	0
FFAR2	0.970033	0.029967	2.917524	112.141	1.11141	0
FGD5	0.970033	0.029967	2.917524	112.141	1.11141	0
FGFBP2	0.970033	0.029967	2.917524	112.141	1.11141	0
FLJ32255	0.970033	0.029967	2.917524	112.141	1.11141	0
FLJ35934	0.970033	0.029967	2.917524	112.141	1.11141	0
FLJ40194	0.970033	0.029967	2.917524	112.141	1.11141	0
FNDC7	0.970033	0.029967	2.917524	112.141	1.11141	0
FNDC9	0.970033	0.029967	2.917524	112.141	1.11141	0
FOXF1	0.970033	0.029967	2.917524	112.141	1.11141	0
FOXR1	0.970033	0.029967	2.917524	112.141	1.11141	0
FRMD8P1	0.970033	0.029967	2.917524	112.141	1.11141	0
FRMPD1	0.970033	0.029967	2.917524	112.141	1.11141	0
FRMPD2	0.970033	0.029967	2.917524	112.141	1.11141	0
GADL1	0.970033	0.029967	2.917524	112.141	1.11141	0
GAGE12B	0.970033	0.029967	2.917524	112.141	1.11141	0
GAPDHS	0.970033	0.029967	2.917524	112.141	1.11141	0
GBX1	0.970033	0.029967	2.917524	112.141	1.11141	0
GIMAP5	0.970033	0.029967	2.917524	112.141	1.11141	0
GIMAP6	0.970033	0.029967	2.917524	112.141	1.11141	0
GLOD5	0.970033	0.029967	2.917524	112.141	1.11141	0
GLRA3	0.970033	0.029967	2.917524	112.141	1.11141	0
GLT6D1	0.970033	0.029967	2.917524	112.141	1.11141	0
GNG8	0.970033	0.029967	2.917524	112.141	1.11141	0
GPR183	0.970033	0.029967	2.917524	112.141	1.11141	0
GPR26	0.970033	0.029967	2.917524	112.141	1.11141	0
GPR50	0.970033	0.029967	2.917524	112.141	1.11141	0
GRID2	0.970033	0.029967	2.917524	112.141	1.11141	0
GRIK1-AS	0.970033	0.029967	2.917524	112.141	1.11141	0
GRIK2	0.970033	0.029967	2.917524	112.141	1.11141	0
GRM7-AS	0.970033	0.029967	2.917524	112.141	1.11141	0
GRP	0.970033	0.029967	2.917524	112.141	1.11141	0
GSTA3	0.970033	0.029967	2.917524	112.141	1.11141	0
GSTTP2	0.970033	0.029967	2.917524	112.141	1.11141	0
GUCY1B2	0.970033	0.029967	2.917524	112.141	1.11141	0
H2AFB1	0.970033	0.029967	2.917524	112.141	1.11141	0

H2AFB3	0.970033	0.029967	2.917524	112.141	1.11141	0
HCG22	0.970033	0.029967	2.917524	112.141	1.11141	0
HCRTR1	0.970033	0.029967	2.917524	112.141	1.11141	0
HES5	0.970033	0.029967	2.917524	112.141	1.11141	0
HOXC12	0.970033	0.029967	2.917524	112.141	1.11141	0
HOXD11	0.970033	0.029967	2.917524	112.141	1.11141	0
HPR	0.970033	0.029967	2.917524	112.141	1.11141	0
HS1BP3-IT	0.970033	0.029967	2.917524	112.141	1.11141	0
IFT74-AS1	0.970033	0.029967	2.917524	112.141	1.11141	0
IL12A-AS1	0.970033	0.029967	2.917524	112.141	1.11141	0
IL17B	0.970033	0.029967	2.917524	112.141	1.11141	0
IL18RAP	0.970033	0.029967	2.917524	112.141	1.11141	0
IQCF1	0.970033	0.029967	2.917524	112.141	1.11141	0
ITGAD	0.970033	0.029967	2.917524	112.141	1.11141	0
KAAG1	0.970033	0.029967	2.917524	112.141	1.11141	0
KCNC4-AS	0.970033	0.029967	2.917524	112.141	1.11141	0
KCND2	0.970033	0.029967	2.917524	112.141	1.11141	0
KCNJ2-AS	0.970033	0.029967	2.917524	112.141	1.11141	0
KCNQ1-AS	0.970033	0.029967	2.917524	112.141	1.11141	0
KIRREL3	0.970033	0.029967	2.917524	112.141	1.11141	0
KLK3	0.970033	0.029967	2.917524	112.141	1.11141	0
KLKP1	0.970033	0.029967	2.917524	112.141	1.11141	0
KRT12	0.970033	0.029967	2.917524	112.141	1.11141	0
KRT16P2	0.970033	0.029967	2.917524	112.141	1.11141	0
KRT42P	0.970033	0.029967	2.917524	112.141	1.11141	0
KRT72	0.970033	0.029967	2.917524	112.141	1.11141	0
LBX1	0.970033	0.029967	2.917524	112.141	1.11141	0
LBX1-AS1	0.970033	0.029967	2.917524	112.141	1.11141	0
LCA10	0.970033	0.029967	2.917524	112.141	1.11141	0
LCE3D	0.970033	0.029967	2.917524	112.141	1.11141	0
LCN1	0.970033	0.029967	2.917524	112.141	1.11141	0
LCNL1	0.970033	0.029967	2.917524	112.141	1.11141	0
LDHC	0.970033	0.029967	2.917524	112.141	1.11141	0
LEXM	0.970033	0.029967	2.917524	112.141	1.11141	0
LILRB1	0.970033	0.029967	2.917524	112.141	1.11141	0
LINC00158	0.970033	0.029967	2.917524	112.141	1.11141	0
LINC00293	0.970033	0.029967	2.917524	112.141	1.11141	0
LINC00479	0.970033	0.029967	2.917524	112.141	1.11141	0
LINC00539	0.970033	0.029967	2.917524	112.141	1.11141	0
LINC00602	0.970033	0.029967	2.917524	112.141	1.11141	0
LINC00607	0.970033	0.029967	2.917524	112.141	1.11141	0
LINC00619	0.970033	0.029967	2.917524	112.141	1.11141	0
LINC00652	0.970033	0.029967	2.917524	112.141	1.11141	0
LINC00838	0.970033	0.029967	2.917524	112.141	1.11141	0
LINC0086	0.970033	0.029967	2.917524	112.141	1.11141	0
LINC00900	0.970033	0.029967	2.917524	112.141	1.11141	0
LINC00934	0.970033	0.029967	2.917524	112.141	1.11141	0
LINC00964	0.970033	0.029967	2.917524	112.141	1.11141	0
LINC0104	0.970033	0.029967	2.917524	112.141	1.11141	0
LINC01115	0.970033	0.029967	2.917524	112.141	1.11141	0
LINC01163	0.970033	0.029967	2.917524	112.141	1.11141	0
LINC0116	0.970033	0.029967	2.917524	112.141	1.11141	0
LINC0121	0.970033	0.029967	2.917524	112.141	1.11141	0
LINC01219	0.970033	0.029967	2.917524	112.141	1.11141	0
LINC01248	0.970033	0.029967	2.917524	112.141	1.11141	0
LINC01267	0.970033	0.029967	2.917524	112.141	1.11141	0

LINC0127	0.970033	0.029967	2.917524	112.141	1.11141	0
LINC0144	0.970033	0.029967	2.917524	112.141	1.11141	0
LINC0148	0.970033	0.029967	2.917524	112.141	1.11141	0
LINC0148	0.970033	0.029967	2.917524	112.141	1.11141	0
LINC0152	0.970033	0.029967	2.917524	112.141	1.11141	0
LINC0153	0.970033	0.029967	2.917524	112.141	1.11141	0
LINC0158	0.970033	0.029967	2.917524	112.141	1.11141	0
LOC10012	0.970033	0.029967	2.917524	112.141	1.11141	0
LOC10013	0.970033	0.029967	2.917524	112.141	1.11141	0
LOC10024	0.970033	0.029967	2.917524	112.141	1.11141	0
LOC10050	0.970033	0.029967	2.917524	112.141	1.11141	0
LOC10050	0.970033	0.029967	2.917524	112.141	1.11141	0
LOC10050	0.970033	0.029967	2.917524	112.141	1.11141	0
LOC10099	0.970033	0.029967	2.917524	112.141	1.11141	0
LOC10099	0.970033	0.029967	2.917524	112.141	1.11141	0
LOC10192	0.970033	0.029967	2.917524	112.141	1.11141	0
LOC10192	0.970033	0.029967	2.917524	112.141	1.11141	0
LOC10192	0.970033	0.029967	2.917524	112.141	1.11141	0
LOC10192	0.970033	0.029967	2.917524	112.141	1.11141	0
LOC10192	0.970033	0.029967	2.917524	112.141	1.11141	0
LOC10192	0.970033	0.029967	2.917524	112.141	1.11141	0
LOC10192	0.970033	0.029967	2.917524	112.141	1.11141	0
LOC10192	0.970033	0.029967	2.917524	112.141	1.11141	0
LOC10192	0.970033	0.029967	2.917524	112.141	1.11141	0
LOC10192	0.970033	0.029967	2.917524	112.141	1.11141	0
LOC10192	0.970033	0.029967	2.917524	112.141	1.11141	0
LOC10192	0.970033	0.029967	2.917524	112.141	1.11141	0
LOC10192	0.970033	0.029967	2.917524	112.141	1.11141	0
LOC10192	0.970033	0.029967	2.917524	112.141	1.11141	0
LOC10192	0.970033	0.029967	2.917524	112.141	1.11141	0
LOC10192	0.970033	0.029967	2.917524	112.141	1.11141	0
LOC10192	0.970033	0.029967	2.917524	112.141	1.11141	0
LOC10192	0.970033	0.029967	2.917524	112.141	1.11141	0
LOC10192	0.970033	0.029967	2.917524	112.141	1.11141	0
LOC10247	0.970033	0.029967	2.917524	112.141	1.11141	0
LOC10272	0.970033	0.029967	2.917524	112.141	1.11141	0
LOC15727	0.970033	0.029967	2.917524	112.141	1.11141	0
LOC25402	0.970033	0.029967	2.917524	112.141	1.11141	0
LOC28335	0.970033	0.029967	2.917524	112.141	1.11141	0
LOC28559	0.970033	0.029967	2.917524	112.141	1.11141	0
LOC34011	0.970033	0.029967	2.917524	112.141	1.11141	0
LOC38881	0.970033	0.029967	2.917524	112.141	1.11141	0
LOC38894	0.970033	0.029967	2.917524	112.141	1.11141	0
LOC40068	0.970033	0.029967	2.917524	112.141	1.11141	0
LOC40101	0.970033	0.029967	2.917524	112.141	1.11141	0
LOC44070	0.970033	0.029967	2.917524	112.141	1.11141	0
LOC64602	0.970033	0.029967	2.917524	112.141	1.11141	0
LOC72915	0.970033	0.029967	2.917524	112.141	1.11141	0
LOC72973	0.970033	0.029967	2.917524	112.141	1.11141	0
LRRC66	0.970033	0.029967	2.917524	112.141	1.11141	0
LUZP2	0.970033	0.029967	2.917524	112.141	1.11141	0
LYG2	0.970033	0.029967	2.917524	112.141	1.11141	0
LYPD1	0.970033	0.029967	2.917524	112.141	1.11141	0
LYVE1	0.970033	0.029967	2.917524	112.141	1.11141	0
LYZL6	0.970033	0.029967	2.917524	112.141	1.11141	0
MAGEL2	0.970033	0.029967	2.917524	112.141	1.11141	0

MARCH11	0.970033	0.029967	2.917524	112.141	1.11141	0
MGC1602	0.970033	0.029967	2.917524	112.141	1.11141	0
MGC1627	0.970033	0.029967	2.917524	112.141	1.11141	0
MIP	0.970033	0.029967	2.917524	112.141	1.11141	0
MIR4500H	0.970033	0.029967	2.917524	112.141	1.11141	0
MIR5689H	0.970033	0.029967	2.917524	112.141	1.11141	0
MIR7851	0.970033	0.029967	2.917524	112.141	1.11141	0
MIR8072	0.970033	0.029967	2.917524	112.141	1.11141	0
MMP8	0.970033	0.029967	2.917524	112.141	1.11141	0
MOGAT1	0.970033	0.029967	2.917524	112.141	1.11141	0
MS4A12	0.970033	0.029967	2.917524	112.141	1.11141	0
MT1B	0.970033	0.029967	2.917524	112.141	1.11141	0
MT3	0.970033	0.029967	2.917524	112.141	1.11141	0
MTHFD2P	0.970033	0.029967	2.917524	112.141	1.11141	0
MTVR2	0.970033	0.029967	2.917524	112.141	1.11141	0
MUC2	0.970033	0.029967	2.917524	112.141	1.11141	0
MYH13	0.970033	0.029967	2.917524	112.141	1.11141	0
MYL10	0.970033	0.029967	2.917524	112.141	1.11141	0
MYT1L	0.970033	0.029967	2.917524	112.141	1.11141	0
NALCN-AS	0.970033	0.029967	2.917524	112.141	1.11141	0
NBPF22P	0.970033	0.029967	2.917524	112.141	1.11141	0
NCR3	0.970033	0.029967	2.917524	112.141	1.11141	0
NEFL	0.970033	0.029967	2.917524	112.141	1.11141	0
NEUROD4	0.970033	0.029967	2.917524	112.141	1.11141	0
NLRP8	0.970033	0.029967	2.917524	112.141	1.11141	0
NOVA1	0.970033	0.029967	2.917524	112.141	1.11141	0
NR0B2	0.970033	0.029967	2.917524	112.141	1.11141	0
NR5A2	0.970033	0.029967	2.917524	112.141	1.11141	0
NRG2	0.970033	0.029967	2.917524	112.141	1.11141	0
OCSTAMP	0.970033	0.029967	2.917524	112.141	1.11141	0
ODAM	0.970033	0.029967	2.917524	112.141	1.11141	0
OLFM5P	0.970033	0.029967	2.917524	112.141	1.11141	0
OR51B2	0.970033	0.029967	2.917524	112.141	1.11141	0
OSR1	0.970033	0.029967	2.917524	112.141	1.11141	0
OSTN	0.970033	0.029967	2.917524	112.141	1.11141	0
OSTN-AS1	0.970033	0.029967	2.917524	112.141	1.11141	0
OVAAL	0.970033	0.029967	2.917524	112.141	1.11141	0
PACRG	0.970033	0.029967	2.917524	112.141	1.11141	0
PCDHGB8	0.970033	0.029967	2.917524	112.141	1.11141	0
PGM5P3-A	0.970033	0.029967	2.917524	112.141	1.11141	0
PGM5P4-A	0.970033	0.029967	2.917524	112.141	1.11141	0
PIK3IP1-A	0.970033	0.029967	2.917524	112.141	1.11141	0
PLA2G12B	0.970033	0.029967	2.917524	112.141	1.11141	0
PLA2G4E	0.970033	0.029967	2.917524	112.141	1.11141	0
PLCXD2-A	0.970033	0.029967	2.917524	112.141	1.11141	0
PLPPR5	0.970033	0.029967	2.917524	112.141	1.11141	0
PNLDC1	0.970033	0.029967	2.917524	112.141	1.11141	0
PNLIPRP3	0.970033	0.029967	2.917524	112.141	1.11141	0
PPEF1	0.970033	0.029967	2.917524	112.141	1.11141	0
PRKACG	0.970033	0.029967	2.917524	112.141	1.11141	0
PROK2	0.970033	0.029967	2.917524	112.141	1.11141	0
PRORY	0.970033	0.029967	2.917524	112.141	1.11141	0
PRR26	0.970033	0.029967	2.917524	112.141	1.11141	0
PRSS50	0.970033	0.029967	2.917524	112.141	1.11141	0
PTPN7	0.970033	0.029967	2.917524	112.141	1.11141	0
PTTG2	0.970033	0.029967	2.917524	112.141	1.11141	0

RAB44	0.970033	0.029967	2.917524	112.141	1.11141	0
REXO1L2	0.970033	0.029967	2.917524	112.141	1.11141	0
RFPL1	0.970033	0.029967	2.917524	112.141	1.11141	0
RFPL4B	0.970033	0.029967	2.917524	112.141	1.11141	0
RFX4	0.970033	0.029967	2.917524	112.141	1.11141	0
RHO	0.970033	0.029967	2.917524	112.141	1.11141	0
RNU11	0.970033	0.029967	2.917524	112.141	1.11141	0
ROPN1B	0.970033	0.029967	2.917524	112.141	1.11141	0
RPL21P44	0.970033	0.029967	2.917524	112.141	1.11141	0
RUVBL1-A	0.970033	0.029967	2.917524	112.141	1.11141	0
SCML4	0.970033	0.029967	2.917524	112.141	1.11141	0
SCT	0.970033	0.029967	2.917524	112.141	1.11141	0
SCUBE1	0.970033	0.029967	2.917524	112.141	1.11141	0
SEMG1	0.970033	0.029967	2.917524	112.141	1.11141	0
SERPINA6	0.970033	0.029967	2.917524	112.141	1.11141	0
SH3RF3-A	0.970033	0.029967	2.917524	112.141	1.11141	0
SHH	0.970033	0.029967	2.917524	112.141	1.11141	0
SLC14A1	0.970033	0.029967	2.917524	112.141	1.11141	0
SLC39A5	0.970033	0.029967	2.917524	112.141	1.11141	0
SLC5A12	0.970033	0.029967	2.917524	112.141	1.11141	0
SLED1	0.970033	0.029967	2.917524	112.141	1.11141	0
SNORA25	0.970033	0.029967	2.917524	112.141	1.11141	0
SNORA5B	0.970033	0.029967	2.917524	112.141	1.11141	0
SNORA5C	0.970033	0.029967	2.917524	112.141	1.11141	0
SNORD36	0.970033	0.029967	2.917524	112.141	1.11141	0
SNORD83	0.970033	0.029967	2.917524	112.141	1.11141	0
SNX29P1	0.970033	0.029967	2.917524	112.141	1.11141	0
ST3GAL5-	0.970033	0.029967	2.917524	112.141	1.11141	0
SULT1B1	0.970033	0.029967	2.917524	112.141	1.11141	0
SYT6	0.970033	0.029967	2.917524	112.141	1.11141	0
TBC1D3P	0.970033	0.029967	2.917524	112.141	1.11141	0
TLR8	0.970033	0.029967	2.917524	112.141	1.11141	0
TMEM204	0.970033	0.029967	2.917524	112.141	1.11141	0
TNNI2	0.970033	0.029967	2.917524	112.141	1.11141	0
TPRXL	0.970033	0.029967	2.917524	112.141	1.11141	0
TRPC7	0.970033	0.029967	2.917524	112.141	1.11141	0
TRPM3	0.970033	0.029967	2.917524	112.141	1.11141	0
TSPAN11	0.970033	0.029967	2.917524	112.141	1.11141	0
TSPEAR-A	0.970033	0.029967	2.917524	112.141	1.11141	0
TSPY26P	0.970033	0.029967	2.917524	112.141	1.11141	0
TTLL10	0.970033	0.029967	2.917524	112.141	1.11141	0
TULP2	0.970033	0.029967	2.917524	112.141	1.11141	0
TXNDC2	0.970033	0.029967	2.917524	112.141	1.11141	0
TYRO3P	0.970033	0.029967	2.917524	112.141	1.11141	0
UBE2NL	0.970033	0.029967	2.917524	112.141	1.11141	0
USP17L6P	0.970033	0.029967	2.917524	112.141	1.11141	0
USP26	0.970033	0.029967	2.917524	112.141	1.11141	0
VIP	0.970033	0.029967	2.917524	112.141	1.11141	0
VTN	0.970033	0.029967	2.917524	112.141	1.11141	0
WIF1	0.970033	0.029967	2.917524	112.141	1.11141	0
WNT9B	0.970033	0.029967	2.917524	112.141	1.11141	0
ZNF154	0.970033	0.029967	2.917524	112.141	1.11141	0
ZNF32-AS	0.970033	0.029967	2.917524	112.141	1.11141	0
ZNF32-AS	0.970033	0.029967	2.917524	112.141	1.11141	0
ZNF33BP1	0.970033	0.029967	2.917524	112.141	1.11141	0
ZNF385D	0.970033	0.029967	2.917524	112.141	1.11141	0

ZNF486	0.970033	0.029967	2.917524	112.141	1.11141	0
ZNF729	0.970033	0.029967	2.917524	112.141	1.11141	0
NALCN	0.970033	0.029967	0.765941	0.760245	17.78255	23.39372
CEP19	0.970038	0.029962	0.791265	0.790158	84.87835	107.4221
C2orf70	0.97004	0.02996	0.736588	0.726785	11.1141	15.29589
KIAA1143	0.970045	0.029955	0.758415	0.758141	381.2135	502.8299
EFCAB10	0.970047	0.029953	0.718786	0.70608	8.891277	12.59662
CALCRL	0.970054	0.029946	0.771765	0.766785	20.00537	26.09299
CFAP44	0.970054	0.029946	0.771765	0.766785	20.00537	26.09299
CKMT2-AS	0.970055	0.029945	1.272706	1.273967	154.8972	121.5843
UQCRH	0.970056	0.029944	0.692405	0.674705	6.668457	9.888344
KIR3DX1	0.970061	0.029939	1.286324	1.293525	29.28564	22.63792
ARL6	0.970064	0.029936	0.77295	0.772505	224.5047	290.6219
CARD19	0.970076	0.029924	1.300683	1.301319	350.094	269.0277
NFATC2	0.970086	0.029914	0.776518	0.772099	22.22819	28.79227
LINC0067	0.970093	0.029907	0.729258	0.718371	10.35834	14.42313
MT1E	0.970097	0.029903	0.750716	0.750503	499.0229	664.9214
EZH1	0.9701	0.0299	0.76822	0.767853	281.1866	366.2016
SOD3	0.970111	0.029889	0.793604	0.792184	65.57317	82.77776
CAMSAP1	0.970121	0.029879	1.365157	1.365374	1305.906	956.4431
ZGLP1	0.970124	0.029876	0.766046	0.760245	17.78255	23.39372
ETFDH	0.970125	0.029875	1.296971	1.29765	323.4202	249.2331
TNFAIP8L	0.970126	0.029874	1.296591	1.297286	311.0502	239.7676
PDP2	0.97015	0.02985	0.74631	0.746125	581.8007	779.7665
NHLRC2	0.970165	0.029835	0.749657	0.749452	521.2511	695.5132
THAP3	0.970169	0.029831	1.299351	1.300004	334.5343	257.3309
UBE2A	0.97017	0.02983	1.410254	1.410363	3034.148	2151.322
LINC0033	0.97018	0.02982	0.749361	0.741283	13.33691	17.99517
AMOT	0.97018	0.02982	0.783811	0.78021	26.67383	34.19082
LOC10192	0.97018	0.02982	0.783811	0.78021	26.67383	34.19082
FAM98C	0.970195	0.029805	1.277527	1.278555	196.7195	153.8587
KCNH3	0.97021	0.02979	0.794621	0.793016	57.7933	72.88042
RCAN3	0.970218	0.029782	1.336521	1.336858	760.2042	568.6472
PYGO1	0.97022	0.02978	1.282302	1.283186	237.8417	185.3502
ARHGEF4	0.970224	0.029776	1.353357	1.353615	1054.728	779.1907
LINC0018	0.970224	0.029776	0.6466	0.618146	4.445638	7.198066
LOC10012	0.970224	0.029776	0.6466	0.618146	4.445638	7.198066
PHKG1	0.970224	0.029776	0.6466	0.618146	4.445638	7.198066
RPL31P11	0.970224	0.029776	0.6466	0.618146	4.445638	7.198066
THEGL	0.970224	0.029776	0.6466	0.618146	4.445638	7.198066
XYLT1	0.970224	0.029776	0.6466	0.618146	4.445638	7.198066
SLC25A5-	0.97023	0.02977	0.736766	0.726785	11.1141	15.29589
TECTA	0.97023	0.02977	0.736766	0.726785	11.1141	15.29589
FAM160B	0.970235	0.029765	1.312704	1.313204	467.9034	356.3043
UFC1	0.970246	0.029754	1.392896	1.393037	2217.262	1591.672
SDC4	0.970261	0.029739	1.455852	1.455905	7105.241	4880.289
CT45A6	0.970262	0.029738	2.898349	111.0295	1.100295	0
USP17L3	0.970262	0.029738	2.898349	111.0295	1.100295	0
ANO9	0.970277	0.029723	1.301488	1.302096	367.8766	282.5241
ZBTB14	0.970281	0.029719	0.782205	0.781595	158.9316	203.3454
HYAL1	0.970288	0.029712	0.719015	0.70608	8.891277	12.59662
TEX9	0.970288	0.029712	0.719015	0.70608	8.891277	12.59662
GP6	0.970291	0.029709	0.7892	0.78611	31.11947	39.58937
LOC73010	0.970291	0.029709	1.283738	1.284625	231.1732	179.9517
TMIE	0.970298	0.029702	1.261179	1.265287	46.6792	36.89009
INE2	0.970304	0.029696	0.494039	0.393031	1.11141	2.843236

APOA1BP	0.970317	0.029683	0.707079	0.707025	2192.811	3101.467
CNTNAP3	0.970319	0.029681	0.324658	0.008226	0	1.205676
LOC10192	0.970322	0.029678	0.692127	0.674092	6.668457	9.897341
SLC25A40	0.970323	0.029677	1.353418	1.353674	1066.953	788.1883
HSPA5	0.970325	0.029675	0.624068	0.624065	61510.96	98564.92
PLEKHO2	0.970334	0.029666	1.296094	1.296761	327.8658	252.8321
C15orf62	0.970346	0.029654	1.261254	1.265287	46.6792	36.89009
LOC72861	0.970353	0.029647	1.270156	1.275775	35.58733	27.89251
CHRD	0.970361	0.029639	1.266512	1.271473	38.89934	30.59178
PTEN	0.970378	0.029622	0.698314	0.698274	2993.426	4286.898
GABBR2	0.97038	0.02962	0.791306	0.788495	33.34229	42.28864
ITGA7	0.970393	0.029607	0.794482	0.793289	77.79867	98.07366
MCAM	0.970396	0.029604	1.434271	1.434345	4819.072	3359.769
OR2A9P	0.970402	0.029598	1.265539	1.266913	139.4486	110.0674
VWA7	0.970407	0.029593	1.257509	1.260905	54.45907	43.1884
MCM3AP-	0.970415	0.029585	0.796274	0.794973	72.24162	90.87559
AKR1C2	0.970419	0.029581	0.793058	0.792021	90.02418	113.6665
NEGR1	0.970422	0.029578	1.259021	1.262616	51.12484	40.48912
LRRC41	0.970449	0.029551	0.781534	0.780962	170.0457	217.7415
CD177	0.970451	0.029549	1.273144	1.279236	32.23088	25.19323
HMX2	0.970451	0.029549	1.273144	1.279236	32.23088	25.19323
SMAD7	0.970474	0.029526	1.301209	1.301791	391.2162	300.5193
GOLGA6L	0.970476	0.029524	1.551854	1.632891	6.323921	3.868961
PPP1R21	0.970478	0.029522	0.738935	0.738793	769.0954	1041.02
DLX2	0.970478	0.029522	1.257104	1.260381	55.57048	44.08816
PGK1	0.970479	0.029521	1.535828	1.535842	32956.63	21458.34
BSN-AS2	0.970482	0.029518	0.700297	0.683066	6.75737	9.897341
C1orf53	0.970497	0.029503	0.797514	0.796063	64.46176	80.97825
ANKRD54	0.970501	0.029499	0.76704	0.766701	300.0806	391.3949
TPTE2P6	0.970511	0.029489	1.81889	2.081531	3.756564	1.799517
EIF2B3	0.970531	0.029469	0.741065	0.740913	721.3048	973.5385
PDCL3P4	0.970544	0.029456	2.0814	2.751378	2.567356	0.926751
GMPPB	0.970548	0.029452	0.76246	0.762169	354.5397	465.175
KRT75	0.97055	0.02945	1.27749	1.284514	28.89665	22.49396
FAM103A1	0.970555	0.029445	1.324581	1.324973	623.5008	470.5736
CNDP2	0.970584	0.029416	1.383766	1.383921	1986.089	1435.114
SGK3	0.970585	0.029415	0.764064	0.76376	337.1239	441.4034
GTF2A1L	0.970589	0.029411	0.32067	0.007932	0	1.250664
FRAS1	0.970589	0.029411	0.729807	0.7297	1049.171	1437.814
FBXL8	0.97059	0.02941	1.281045	1.288684	26.99614	20.94637
CARD10	0.970606	0.029394	1.347107	1.347381	974.7062	723.4057
GLB1L2	0.970607	0.029393	1.251949	1.254488	72.24162	57.58453
LY75	0.970609	0.029391	1.31469	1.31515	512.9711	390.0452
ZNF287	0.970627	0.029373	0.798346	0.796948	66.68457	83.67752
BHLHA15	0.970628	0.029372	1.281065	1.288797	26.67383	20.69444
BHLHB9	0.970631	0.029369	0.780945	0.780408	181.1598	232.1376
HERC2P7	0.97064	0.02936	0.797624	0.7962	64.47287	80.97825
C12orf76	0.970644	0.029356	0.79811	0.796429	54.45907	68.38163
HPCAL1	0.970654	0.029346	1.314457	1.314916	513.4712	390.4951
SYS1-DBN	0.970655	0.029345	1.256411	1.260557	45.23437	35.88236
PRR7-AS1	0.970661	0.029339	1.50602	1.569947	7.068565	4.498792
SIX4	0.970672	0.029328	1.343285	1.34357	940.2525	699.814
PUS10	0.970678	0.029322	1.251207	1.253625	75.57585	60.28381
LRCH1	0.970682	0.029318	1.324072	1.324462	626.835	473.2729
ADH4	0.970686	0.029314	2.920244	114.3638	1.133638	0
PTK2	0.970695	0.029305	1.473201	1.47324	10436.14	7083.797

TGDS	0.970698	0.029302	1.267003	1.268151	171.1571	134.9637
RSPH3	0.970699	0.029301	1.285293	1.286051	281.1866	218.6413
PFKFB4	0.970701	0.029299	1.30959	1.310085	466.792	356.3043
TRAM2-AS	0.970719	0.029281	0.797781	0.795611	42.23356	53.08574
CCDC24	0.970721	0.029279	0.798088	0.796429	54.45907	68.38163
CIB2	0.970723	0.029277	1.2853	1.293896	24.45101	18.89492
EP400NL	0.970723	0.029277	1.2853	1.293896	24.45101	18.89492
NTMT1	0.970731	0.029269	1.360272	1.36049	1303.683	958.2426
MRPL21	0.97074	0.02926	0.740645	0.7405	770.2068	1040.121
C14orf28	0.970744	0.029256	1.257823	1.259423	115.5866	91.77535
FGFR3	0.970745	0.029255	0.75571	0.75548	456.7893	604.6376
COQ3	0.970747	0.029253	0.787928	0.787232	136.7034	173.6534
PVRL3	0.970755	0.029245	0.746717	0.746542	614.6095	823.2788
AMOTL1	0.970773	0.029227	1.429826	1.429902	4587.899	3208.538
CDH1	0.970773	0.029227	0.6863	0.686275	4893.536	7130.585
HMGA2	0.970775	0.029225	0.706016	0.705967	2369.703	3356.683
NANP	0.970781	0.029219	1.288203	1.288924	293.4121	227.6389
FBXL14	0.970785	0.029215	0.787297	0.786627	142.2604	180.8514
OLFML3	0.970795	0.029205	1.285143	1.293896	24.45101	18.89492
LIMK2	0.97081	0.02919	1.405343	1.405453	2936.344	2089.248
ZNF692	0.970812	0.029188	1.301344	1.301896	412.333	316.7149
DPF1	0.970823	0.029177	1.251018	1.253355	76.68726	61.18356
MIR193BH	0.970838	0.029162	1.290398	1.300068	22.22819	17.09541
STXBP5	0.970847	0.029153	0.761288	0.761016	374.545	492.1678
AGAP4	0.970855	0.029145	1.272948	1.273901	211.5346	166.0504
SBK1	0.970863	0.029137	1.262646	1.263935	146.7061	116.0688
QTRTD1	0.970866	0.029134	0.733218	0.733102	964.9147	1316.211
SWI5	0.970871	0.029129	1.322247	1.322634	639.0605	483.1702
SWT1	0.970901	0.029099	0.781355	0.780851	196.7195	251.9323
ATG10	0.970903	0.029097	0.79591	0.794909	92.26922	116.0778
ANP32AP	0.970909	0.029091	2.901418	113.2524	1.122524	0
SNHG21	0.970914	0.029086	1.249332	1.251448	85.57854	68.38163
LPAL2	0.970917	0.029083	0.5033	0.401902	1.11141	2.780253
PDK3	0.97092	0.02908	1.343378	1.343658	939.1411	698.9412
FAHD1	0.970922	0.029078	0.768183	0.767852	306.749	399.4927
EEA1	0.970932	0.029068	0.734441	0.734322	933.584	1271.358
LINC0100	0.970935	0.029065	1.249094	1.251696	68.25166	54.52535
C3orf70	0.970937	0.029063	2.840823	107.6953	1.066953	0
PDF	0.970941	0.029059	0.798586	0.797384	76.09821	95.43736
MTERF4	0.970967	0.029033	0.79102	0.790295	132.2577	167.355
CFTR	0.970967	0.029033	1.300492	1.312216	18.89396	14.39613
RGPD4	0.970969	0.029031	0.765515	0.758387	14.47055	19.08387
ALK	0.970976	0.029024	1.296651	1.307691	20.00537	15.29589
EHD2	0.970983	0.029017	2.066894	2.747496	2.489557	0.899758
APOL4	0.970991	0.029009	1.258789	1.264038	35.48731	28.07246
LOC10012	0.971002	0.028998	0.507784	0.408582	1.144752	2.816243
SPATC1L	0.971022	0.028978	0.794951	0.794092	110.0295	138.5628
DTD1	0.971033	0.028967	0.744452	0.744293	682.4055	916.8537
NOL3	0.971039	0.028961	1.283895	1.284628	288.9665	224.9396
KRTCAP2	0.971052	0.028948	0.711743	0.711685	2051.662	2882.826
RBPMS-A	0.971054	0.028946	1.300341	1.312216	18.89396	14.39613
DAG1	0.971067	0.028933	0.700183	0.700143	2923.007	4174.879
SRCIN1	0.971072	0.028928	0.801127	0.7993	48.90202	61.18356
LOC10272	0.971077	0.028923	1.249189	1.251237	86.68995	69.28139
SLC2A1-A	0.97108	0.02892	0.782852	0.77783	18.89396	24.29347
KDR	0.971083	0.028917	0.790153	0.786128	23.3396	29.69202

LOC64307	0.971083	0.028917	0.790153	0.786128	23.3396	29.69202
PNPLA5	0.971084	0.028916	0.792882	0.789241	25.56242	32.3913
ZNF789	0.971087	0.028913	0.783626	0.783099	186.7168	238.436
C16orf45	0.97109	0.02891	1.29645	1.307691	20.00537	15.29589
PCSK6-AS	0.971094	0.028906	1.742195	1.951146	4.012189	2.051449
FAM149A	0.971098	0.028902	0.786889	0.782394	21.11678	26.99275
DOCK7	0.97113	0.02887	0.711393	0.711336	2037.214	2863.931
AADACL2	0.971131	0.028869	2.882592	112.141	1.11141	0
AIM2	0.971131	0.028869	2.882592	112.141	1.11141	0
AMPH	0.971131	0.028869	2.882592	112.141	1.11141	0
ANGPT4	0.971131	0.028869	2.882592	112.141	1.11141	0
ANTXRL	0.971131	0.028869	2.882592	112.141	1.11141	0
ATP1A4	0.971131	0.028869	2.882592	112.141	1.11141	0
BMX	0.971131	0.028869	2.882592	112.141	1.11141	0
C16orf82	0.971131	0.028869	2.882592	112.141	1.11141	0
C1orf189	0.971131	0.028869	2.882592	112.141	1.11141	0
C6orf229	0.971131	0.028869	2.882592	112.141	1.11141	0
C9orf135	0.971131	0.028869	2.882592	112.141	1.11141	0
CACNA1B	0.971131	0.028869	2.882592	112.141	1.11141	0
CCDC178	0.971131	0.028869	2.882592	112.141	1.11141	0
CCR4	0.971131	0.028869	2.882592	112.141	1.11141	0
CES5AP1	0.971131	0.028869	2.882592	112.141	1.11141	0
CSF3	0.971131	0.028869	2.882592	112.141	1.11141	0
CXADRP3	0.971131	0.028869	2.882592	112.141	1.11141	0
DGCR9	0.971131	0.028869	2.882592	112.141	1.11141	0
DKK2	0.971131	0.028869	2.882592	112.141	1.11141	0
DSPP	0.971131	0.028869	2.882592	112.141	1.11141	0
DUSP27	0.971131	0.028869	2.882592	112.141	1.11141	0
EGR4	0.971131	0.028869	2.882592	112.141	1.11141	0
FAM150A	0.971131	0.028869	2.882592	112.141	1.11141	0
FAM209A	0.971131	0.028869	2.882592	112.141	1.11141	0
FAM25C	0.971131	0.028869	2.882592	112.141	1.11141	0
FAM9C	0.971131	0.028869	2.882592	112.141	1.11141	0
FLJ33360	0.971131	0.028869	2.882592	112.141	1.11141	0
FSIP2	0.971131	0.028869	2.882592	112.141	1.11141	0
FTCDNL1	0.971131	0.028869	2.882592	112.141	1.11141	0
GABRR1	0.971131	0.028869	2.882592	112.141	1.11141	0
GDPD2	0.971131	0.028869	2.882592	112.141	1.11141	0
GLB1L3	0.971131	0.028869	2.882592	112.141	1.11141	0
GLRA4	0.971131	0.028869	2.882592	112.141	1.11141	0
GUCY2C	0.971131	0.028869	2.882592	112.141	1.11141	0
GXYLT1P3	0.971131	0.028869	2.882592	112.141	1.11141	0
HOTS	0.971131	0.028869	2.882592	112.141	1.11141	0
IL21-AS1	0.971131	0.028869	2.882592	112.141	1.11141	0
KRT32	0.971131	0.028869	2.882592	112.141	1.11141	0
LILRA3	0.971131	0.028869	2.882592	112.141	1.11141	0
LINC0003	0.971131	0.028869	2.882592	112.141	1.11141	0
LINC0034	0.971131	0.028869	2.882592	112.141	1.11141	0
LINC0054	0.971131	0.028869	2.882592	112.141	1.11141	0
LINC0058	0.971131	0.028869	2.882592	112.141	1.11141	0
LINC0064	0.971131	0.028869	2.882592	112.141	1.11141	0
LINC0068	0.971131	0.028869	2.882592	112.141	1.11141	0
LINC0069	0.971131	0.028869	2.882592	112.141	1.11141	0
LINC0070	0.971131	0.028869	2.882592	112.141	1.11141	0
LINC0086	0.971131	0.028869	2.882592	112.141	1.11141	0
LINC0120	0.971131	0.028869	2.882592	112.141	1.11141	0

LINC0123	0.971131	0.028869	2.882592	112.141	1.11141	0
LINC0131	0.971131	0.028869	2.882592	112.141	1.11141	0
LINC0147	0.971131	0.028869	2.882592	112.141	1.11141	0
LINC0159	0.971131	0.028869	2.882592	112.141	1.11141	0
LINC0159	0.971131	0.028869	2.882592	112.141	1.11141	0
LINC0160	0.971131	0.028869	2.882592	112.141	1.11141	0
LINC0161	0.971131	0.028869	2.882592	112.141	1.11141	0
LINC0161	0.971131	0.028869	2.882592	112.141	1.11141	0
LMO7DN	0.971131	0.028869	2.882592	112.141	1.11141	0
LOC10042	0.971131	0.028869	2.882592	112.141	1.11141	0
LOC10050	0.971131	0.028869	2.882592	112.141	1.11141	0
LOC10050	0.971131	0.028869	2.882592	112.141	1.11141	0
LOC10050	0.971131	0.028869	2.882592	112.141	1.11141	0
LOC10192	0.971131	0.028869	2.882592	112.141	1.11141	0
LOC10192	0.971131	0.028869	2.882592	112.141	1.11141	0
LOC10192	0.971131	0.028869	2.882592	112.141	1.11141	0
LOC10192	0.971131	0.028869	2.882592	112.141	1.11141	0
LOC10192	0.971131	0.028869	2.882592	112.141	1.11141	0
LOC10192	0.971131	0.028869	2.882592	112.141	1.11141	0
LOC10272	0.971131	0.028869	2.882592	112.141	1.11141	0
LOC10272	0.971131	0.028869	2.882592	112.141	1.11141	0
LOC10272	0.971131	0.028869	2.882592	112.141	1.11141	0
LOC44098	0.971131	0.028869	2.882592	112.141	1.11141	0
MAGEB17	0.971131	0.028869	2.882592	112.141	1.11141	0
MAGEB18	0.971131	0.028869	2.882592	112.141	1.11141	0
MC4R	0.971131	0.028869	2.882592	112.141	1.11141	0
MSMP	0.971131	0.028869	2.882592	112.141	1.11141	0
MYCNOS	0.971131	0.028869	2.882592	112.141	1.11141	0
MYHAS	0.971131	0.028869	2.882592	112.141	1.11141	0
NAGS	0.971131	0.028869	2.882592	112.141	1.11141	0
NCF4	0.971131	0.028869	2.882592	112.141	1.11141	0
NEUROG3	0.971131	0.028869	2.882592	112.141	1.11141	0
NRK	0.971131	0.028869	2.882592	112.141	1.11141	0
NXF2	0.971131	0.028869	2.882592	112.141	1.11141	0
NXF2B	0.971131	0.028869	2.882592	112.141	1.11141	0
ONECUT1	0.971131	0.028869	2.882592	112.141	1.11141	0
OR51E2	0.971131	0.028869	2.882592	112.141	1.11141	0
PAGE2B	0.971131	0.028869	2.882592	112.141	1.11141	0
PCAT2	0.971131	0.028869	2.882592	112.141	1.11141	0
PCAT5	0.971131	0.028869	2.882592	112.141	1.11141	0
PDE1B	0.971131	0.028869	2.882592	112.141	1.11141	0
PHF24	0.971131	0.028869	2.882592	112.141	1.11141	0
PLP1	0.971131	0.028869	2.882592	112.141	1.11141	0
PON3	0.971131	0.028869	2.882592	112.141	1.11141	0
PRAMEF2	0.971131	0.028869	2.882592	112.141	1.11141	0
PTH2	0.971131	0.028869	2.882592	112.141	1.11141	0
PYCARD-/	0.971131	0.028869	2.882592	112.141	1.11141	0
REG3G	0.971131	0.028869	2.882592	112.141	1.11141	0
RFX8	0.971131	0.028869	2.882592	112.141	1.11141	0
RGS17	0.971131	0.028869	2.882592	112.141	1.11141	0
RLN3	0.971131	0.028869	2.882592	112.141	1.11141	0
RNF128	0.971131	0.028869	2.882592	112.141	1.11141	0
RNF151	0.971131	0.028869	2.882592	112.141	1.11141	0
RNF17	0.971131	0.028869	2.882592	112.141	1.11141	0
RPL19P12	0.971131	0.028869	2.882592	112.141	1.11141	0
RRH	0.971131	0.028869	2.882592	112.141	1.11141	0

RSPH9	0.971131	0.028869	2.882592	112.141	1.11141	0
RYR3	0.971131	0.028869	2.882592	112.141	1.11141	0
S100A7A	0.971131	0.028869	2.882592	112.141	1.11141	0
SACS-AS1	0.971131	0.028869	2.882592	112.141	1.11141	0
SCARNA2	0.971131	0.028869	2.882592	112.141	1.11141	0
SCARNA8	0.971131	0.028869	2.882592	112.141	1.11141	0
SELP	0.971131	0.028869	2.882592	112.141	1.11141	0
SIGLEC17	0.971131	0.028869	2.882592	112.141	1.11141	0
SIGLEC5	0.971131	0.028869	2.882592	112.141	1.11141	0
SLAMF8	0.971131	0.028869	2.882592	112.141	1.11141	0
SLC18A1	0.971131	0.028869	2.882592	112.141	1.11141	0
SLC25A31	0.971131	0.028869	2.882592	112.141	1.11141	0
SLC2A7	0.971131	0.028869	2.882592	112.141	1.11141	0
SLC45A2	0.971131	0.028869	2.882592	112.141	1.11141	0
SLC4A1	0.971131	0.028869	2.882592	112.141	1.11141	0
SLC9C1	0.971131	0.028869	2.882592	112.141	1.11141	0
SLITRK2	0.971131	0.028869	2.882592	112.141	1.11141	0
SMAD1-AS	0.971131	0.028869	2.882592	112.141	1.11141	0
SNORA23	0.971131	0.028869	2.882592	112.141	1.11141	0
SNORA32	0.971131	0.028869	2.882592	112.141	1.11141	0
SNORA51	0.971131	0.028869	2.882592	112.141	1.11141	0
SNORA71	0.971131	0.028869	2.882592	112.141	1.11141	0
SNORA72	0.971131	0.028869	2.882592	112.141	1.11141	0
SNORD11	0.971131	0.028869	2.882592	112.141	1.11141	0
SNORD14	0.971131	0.028869	2.882592	112.141	1.11141	0
SNORD16	0.971131	0.028869	2.882592	112.141	1.11141	0
SNORD22	0.971131	0.028869	2.882592	112.141	1.11141	0
SNORD80	0.971131	0.028869	2.882592	112.141	1.11141	0
SNORD89	0.971131	0.028869	2.882592	112.141	1.11141	0
SOX2-OT	0.971131	0.028869	2.882592	112.141	1.11141	0
SOX21	0.971131	0.028869	2.882592	112.141	1.11141	0
SPACA3	0.971131	0.028869	2.882592	112.141	1.11141	0
SPAG6	0.971131	0.028869	2.882592	112.141	1.11141	0
SPANXN1	0.971131	0.028869	2.882592	112.141	1.11141	0
SPANXN2	0.971131	0.028869	2.882592	112.141	1.11141	0
SPARCL1	0.971131	0.028869	2.882592	112.141	1.11141	0
SPINK1	0.971131	0.028869	2.882592	112.141	1.11141	0
SPINK13	0.971131	0.028869	2.882592	112.141	1.11141	0
SPRR2A	0.971131	0.028869	2.882592	112.141	1.11141	0
SRGAP2-/	0.971131	0.028869	2.882592	112.141	1.11141	0
SSX4	0.971131	0.028869	2.882592	112.141	1.11141	0
STOML3	0.971131	0.028869	2.882592	112.141	1.11141	0
STT3A-AS	0.971131	0.028869	2.882592	112.141	1.11141	0
SYCP3	0.971131	0.028869	2.882592	112.141	1.11141	0
SYNGR4	0.971131	0.028869	2.882592	112.141	1.11141	0
SYT8	0.971131	0.028869	2.882592	112.141	1.11141	0
TAC1	0.971131	0.028869	2.882592	112.141	1.11141	0
TBC1D22/	0.971131	0.028869	2.882592	112.141	1.11141	0
TBR1	0.971131	0.028869	2.882592	112.141	1.11141	0
TCEAL5	0.971131	0.028869	2.882592	112.141	1.11141	0
TCTEX1D	0.971131	0.028869	2.882592	112.141	1.11141	0
TEKT3	0.971131	0.028869	2.882592	112.141	1.11141	0
TEX101	0.971131	0.028869	2.882592	112.141	1.11141	0
TH	0.971131	0.028869	2.882592	112.141	1.11141	0
TIGIT	0.971131	0.028869	2.882592	112.141	1.11141	0
TMEM163	0.971131	0.028869	2.882592	112.141	1.11141	0

TMEM196	0.971131	0.028869	2.882592	112.141	1.11141	0
TMIGD2	0.971131	0.028869	2.882592	112.141	1.11141	0
TPSB2	0.971131	0.028869	2.882592	112.141	1.11141	0
TSGA10IF	0.971131	0.028869	2.882592	112.141	1.11141	0
UPP2	0.971131	0.028869	2.882592	112.141	1.11141	0
WFIKKN2	0.971131	0.028869	2.882592	112.141	1.11141	0
XPNPEP2	0.971131	0.028869	2.882592	112.141	1.11141	0
ZBP1	0.971131	0.028869	2.882592	112.141	1.11141	0
ZMAT4	0.971131	0.028869	2.882592	112.141	1.11141	0
ZNF536	0.971131	0.028869	2.882592	112.141	1.11141	0
ZNF663P	0.971131	0.028869	2.882592	112.141	1.11141	0
ZNF676	0.971131	0.028869	2.882592	112.141	1.11141	0
ZNF683	0.971131	0.028869	2.882592	112.141	1.11141	0
ZNF750	0.971131	0.028869	2.882592	112.141	1.11141	0
GPR20	0.97114	0.02886	1.304503	1.317345	17.78255	13.49637
VGF	0.971161	0.028839	0.798916	0.796088	32.23088	40.48912
MAP1LC3	0.971169	0.028831	1.415545	1.415637	3656.771	2583.125
MTERF3	0.971171	0.028829	0.753201	0.752998	522.3625	693.7137
DGCR14	0.971174	0.028826	0.764619	0.764334	358.9853	469.6738
EIF4E1B	0.971186	0.028814	0.514619	0.413915	1.11141	2.699275
MAGEA8	0.971186	0.028814	0.514619	0.413915	1.11141	2.699275
RTEL1	0.971187	0.028813	0.78003	0.779564	209.2451	268.4159
ANKRD23	0.971188	0.028812	0.777979	0.772125	16.67114	21.5942
C20orf195	0.971188	0.028812	0.777979	0.772125	16.67114	21.5942
FAM174A	0.971191	0.028809	1.323722	1.32409	662.4001	500.2656
ALAD	0.971193	0.028807	1.334161	1.33447	836.8914	627.1315
ZNF175	0.9712	0.0288	1.281766	1.282525	271.1839	211.4432
DCAF12	0.971217	0.028783	1.374131	1.3743	1768.253	1286.654
LINC0066	0.971219	0.028781	1.314849	1.329967	15.55973	11.69686
POM121L	0.971224	0.028776	1.380813	1.408946	11.05853	7.845892
ZNF672	0.971229	0.028771	0.738616	0.738487	865.7881	1172.385
RNASEL	0.971256	0.028744	0.792898	0.792172	131.1463	165.5555
MAMSTR	0.971258	0.028742	0.801771	0.799311	36.67652	45.88767
BAG4	0.971263	0.028737	0.758349	0.758116	446.7867	589.3417
DLGAP1	0.971271	0.028729	0.771671	0.764791	14.44832	18.89492
MT1F	0.971278	0.028722	0.791233	0.79056	142.2604	179.9517
TBC1D24	0.971299	0.028701	0.767055	0.766758	348.6714	454.7378
TXNRD2	0.97131	0.02869	0.762758	0.762493	388.9934	510.163
YIPF1	0.971318	0.028682	1.322153	1.32252	673.5142	509.2632
TMEM102	0.971319	0.028681	1.279341	1.280135	253.4014	197.9468
LOC10192	0.971336	0.028664	1.314657	1.329967	15.55973	11.69686
MPL	0.971336	0.028664	1.314657	1.329967	15.55973	11.69686
CLPTM1L	0.97135	0.02865	1.360334	1.360541	1351.474	993.3332
SPATA31I	0.971351	0.028649	2.863766	111.0295	1.100295	0
CUTC	0.97136	0.02864	1.284863	1.285544	312.3061	242.9347
HCLS1	0.971381	0.028619	0.804031	0.801855	41.12215	51.28622
RBM22	0.971385	0.028615	0.70997	0.709916	2188.365	3082.572
CERK	0.971385	0.028615	0.731309	0.731206	1112.521	1521.491
PLCB1	0.971385	0.028615	0.763333	0.755015	12.22551	16.19565
SLC7A4	0.971387	0.028613	1.308981	1.323205	16.67114	12.59662
TMC5	0.971387	0.028613	1.308981	1.323205	16.67114	12.59662
TVP23A	0.971402	0.028598	0.50895	0.405711	1.089181	2.699275
POTEE	0.971406	0.028594	1.407665	1.444846	9.013532	6.235325
ZBTB34	0.971425	0.028575	1.304375	1.304856	479.0175	367.1014
CACNB1	0.971432	0.028568	1.263953	1.264978	188.9396	149.3599
NDP	0.971448	0.028552	1.320939	1.337854	14.44832	10.7971

LOC10192	0.97145	0.02855	0.514045	0.413873	1.133638	2.75326
ISCA1	0.971474	0.028526	0.72841	0.728316	1199.211	1646.558
COMMD8	0.971482	0.028518	0.768287	0.767986	343.4256	447.1799
INTS7	0.971515	0.028485	0.754857	0.754653	516.1164	683.9153
PAG1	0.971519	0.028481	0.50354	0.400611	1.11141	2.789251
LOC10012	0.971537	0.028463	0.751798	0.74133	10.00269	13.49637
LOC10013	0.971537	0.028463	0.751798	0.74133	10.00269	13.49637
SCIN	0.971537	0.028463	0.751798	0.74133	10.00269	13.49637
CRNDE	0.97157	0.02843	1.328299	1.347174	13.33691	9.897341
RAD51-AS	0.97157	0.02843	1.328299	1.347174	13.33691	9.897341
LOC10192	0.97157	0.02843	2.84494	109.9181	1.089181	0
LOC14366	0.97158	0.02842	0.80584	0.803915	45.56779	56.68477
ARHGAP4	0.971596	0.028404	0.752725	0.752535	557.9276	741.4008
DOK4	0.971613	0.028387	1.319277	1.319647	660.1773	500.2656
EGFL8	0.971619	0.028381	0.791654	0.786943	19.81643	25.18423
PPM1F	0.971627	0.028373	1.371814	1.371983	1717.128	1251.564
PTK2B	0.97163	0.02837	1.352588	1.352811	1240.333	916.8537
HS3ST1	0.971632	0.028368	1.320636	1.337854	14.44832	10.7971
RDH14	0.971635	0.028365	0.773459	0.773115	295.6349	382.3973
PTPN2	0.971643	0.028357	0.742628	0.742488	773.53	1041.812
TRIM21	0.97166	0.02834	1.259304	1.26042	166.7114	132.2645
B4GALT6	0.971671	0.028329	1.246445	1.24807	107.8067	86.3768
AKR7L	0.971675	0.028325	0.51573	0.413915	1.11141	2.699275
ANKS4B	0.971675	0.028325	0.51573	0.413915	1.11141	2.699275
ANPEP	0.971675	0.028325	0.51573	0.413915	1.11141	2.699275
APOC1P1	0.971675	0.028325	0.51573	0.413915	1.11141	2.699275
BDKRB1	0.971675	0.028325	0.51573	0.413915	1.11141	2.699275
C19orf18	0.971675	0.028325	0.51573	0.413915	1.11141	2.699275
CACNA1S	0.971675	0.028325	0.51573	0.413915	1.11141	2.699275
CAHM	0.971675	0.028325	0.51573	0.413915	1.11141	2.699275
CALY	0.971675	0.028325	0.51573	0.413915	1.11141	2.699275
CCDC160	0.971675	0.028325	0.51573	0.413915	1.11141	2.699275
CCER2	0.971675	0.028325	0.51573	0.413915	1.11141	2.699275
CCR2	0.971675	0.028325	0.51573	0.413915	1.11141	2.699275
CPLX3	0.971675	0.028325	0.51573	0.413915	1.11141	2.699275
CPNE6	0.971675	0.028325	0.51573	0.413915	1.11141	2.699275
CTD-2201	0.971675	0.028325	0.51573	0.413915	1.11141	2.699275
CYP21A2	0.971675	0.028325	0.51573	0.413915	1.11141	2.699275
DMRTA2	0.971675	0.028325	0.51573	0.413915	1.11141	2.699275
FBXO47	0.971675	0.028325	0.51573	0.413915	1.11141	2.699275
FLT1	0.971675	0.028325	0.51573	0.413915	1.11141	2.699275
GALNT16	0.971675	0.028325	0.51573	0.413915	1.11141	2.699275
GNA14	0.971675	0.028325	0.51573	0.413915	1.11141	2.699275
GRIN2C	0.971675	0.028325	0.51573	0.413915	1.11141	2.699275
HIST1H3I	0.971675	0.028325	0.51573	0.413915	1.11141	2.699275
LCN10	0.971675	0.028325	0.51573	0.413915	1.11141	2.699275
LINC0117	0.971675	0.028325	0.51573	0.413915	1.11141	2.699275
LINC0157	0.971675	0.028325	0.51573	0.413915	1.11141	2.699275
LINC0159	0.971675	0.028325	0.51573	0.413915	1.11141	2.699275
LINGO4	0.971675	0.028325	0.51573	0.413915	1.11141	2.699275
LOC10012	0.971675	0.028325	0.51573	0.413915	1.11141	2.699275
LOC10192	0.971675	0.028325	0.51573	0.413915	1.11141	2.699275
LOC10272	0.971675	0.028325	0.51573	0.413915	1.11141	2.699275
LOC10280	0.971675	0.028325	0.51573	0.413915	1.11141	2.699275
LOC28304	0.971675	0.028325	0.51573	0.413915	1.11141	2.699275
LOC64333	0.971675	0.028325	0.51573	0.413915	1.11141	2.699275

MIF-AS1	0.971675	0.028325	0.51573	0.413915	1.11141	2.699275
MORN5	0.971675	0.028325	0.51573	0.413915	1.11141	2.699275
OPRD1	0.971675	0.028325	0.51573	0.413915	1.11141	2.699275
PAQR9-AS	0.971675	0.028325	0.51573	0.413915	1.11141	2.699275
PGAM2	0.971675	0.028325	0.51573	0.413915	1.11141	2.699275
PSPN	0.971675	0.028325	0.51573	0.413915	1.11141	2.699275
PVRL3-AS	0.971675	0.028325	0.51573	0.413915	1.11141	2.699275
SLC35G3	0.971675	0.028325	0.51573	0.413915	1.11141	2.699275
TCTE1	0.971675	0.028325	0.51573	0.413915	1.11141	2.699275
TSPEAR-A	0.971675	0.028325	0.51573	0.413915	1.11141	2.699275
ZNF503-A	0.971675	0.028325	0.51573	0.413915	1.11141	2.699275
OTUD1	0.971678	0.028322	0.744706	0.744557	726.8619	976.2378
FITM2	0.971679	0.028321	0.807384	0.805617	50.01343	62.08332
RAD51C	0.97168	0.02832	0.749993	0.74982	614.6095	819.6798
RAD54L	0.971693	0.028307	1.311345	1.311766	552.3706	421.0869
C2orf48	0.971703	0.028297	1.337043	1.358356	12.22551	8.997583
FBXL7	0.971703	0.028297	1.337043	1.358356	12.22551	8.997583
LINC0089	0.971703	0.028297	1.337043	1.358356	12.22551	8.997583
LINC0114	0.971703	0.028297	1.337043	1.358356	12.22551	8.997583
LOC28562	0.971703	0.028297	1.337043	1.358356	12.22551	8.997583
MED14OS	0.971703	0.028297	1.337043	1.358356	12.22551	8.997583
CDKN2AIF	0.97171	0.02829	0.750735	0.750558	602.384	802.5844
INTS4P2	0.971718	0.028282	1.936558	2.456141	2.511786	1.016727
EPPK1	0.971726	0.028274	1.386406	1.386541	2263.063	1632.162
FAM27E2	0.971727	0.028273	0.332029	0.008349	0	1.187681
GRIN3B	0.971728	0.028272	0.706944	0.686627	5.557048	8.097825
CLYBL	0.971731	0.028269	0.734789	0.72081	7.779867	10.7971
LINC0115	0.971731	0.028269	0.734789	0.72081	7.779867	10.7971
PLEKHD1	0.971731	0.028269	0.734789	0.72081	7.779867	10.7971
MYO9A	0.97174	0.02826	0.76038	0.760146	444.5638	584.8429
GRIPAP1	0.971743	0.028257	0.721227	0.721154	1555.973	2157.62
LAMB2P1	0.971743	0.028257	0.751987	0.74133	10.00269	13.49637
LINC0150	0.971757	0.028243	0.517149	0.415294	1.11141	2.690277
SLC35F1	0.971757	0.028243	0.517149	0.415294	1.11141	2.690277
ZNF77	0.971758	0.028242	0.791157	0.790558	160.043	202.4456
FRG1JP	0.97176	0.02824	1.244457	1.249427	34.99829	28.00948
TAF11	0.971763	0.028237	0.740885	0.740755	852.4511	1150.791
WDYHV1	0.97177	0.02823	1.280975	1.281657	306.749	239.3357
SLC22A20	0.971771	0.028229	1.327963	1.347174	13.33691	9.897341
ZNF391	0.971776	0.028224	0.801967	0.800988	92.247	115.1691
LINC0044	0.971784	0.028216	0.735137	0.721071	7.724297	10.71612
SIRPG-AS	0.971787	0.028213	2.826114	108.8067	1.078067	0
CHKB-CPT	0.9718	0.0282	0.80822	0.805787	36.84323	45.72572
RRN3P2	0.971801	0.028199	0.687743	0.663995	5.056914	7.620953
WARS2-IT	0.971808	0.028192	1.367744	1.396804	10.05826	7.198066
FZD3	0.971812	0.028188	0.78094	0.78051	226.4942	290.19
NCBP3	0.971813	0.028187	0.738081	0.73796	910.3222	1233.569
LINC0029	0.971817	0.028183	0.796831	0.796072	121.4993	152.626
BEST1	0.971832	0.028168	0.805249	0.804086	76.68726	95.37438
SIRT6	0.971838	0.028162	0.770727	0.77042	333.4229	432.7837
ARMC3	0.971848	0.028152	1.3476	1.37202	11.1141	8.097825
AVPR2	0.971848	0.028152	1.3476	1.37202	11.1141	8.097825
CYP4X1	0.971848	0.028152	1.3476	1.37202	11.1141	8.097825
ZNF221	0.971848	0.028152	1.3476	1.37202	11.1141	8.097825
CAGE1	0.971857	0.028143	0.654255	0.618323	3.334229	5.39855
RIMS1	0.971857	0.028143	0.654255	0.618323	3.334229	5.39855

SAPCD1-/	0.971857	0.028143	0.654255	0.618323	3.334229	5.39855
GEMIN5	0.971876	0.028124	0.72525	0.725168	1382.594	1906.588
ABHD18	0.971883	0.028117	0.793422	0.792772	143.3718	180.8514
PAK4	0.971889	0.028111	1.377102	1.377253	1993.869	1447.711
ZNF75D	0.971897	0.028103	0.774541	0.7742	296.7464	383.297
POU2AF1	0.971909	0.028091	1.383165	1.417952	8.769022	6.18134
CTPS1	0.97191	0.02809	1.419304	1.419385	4188.903	2951.207
PCDH20	0.971918	0.028082	0.806024	0.802955	28.89665	35.99033
LOC10050	0.971919	0.028081	0.807225	0.804387	31.11947	38.68961
KRBA2	0.97192	0.02808	0.809236	0.806725	35.56511	44.08816
EFCAB3	0.971924	0.028076	1.336665	1.358356	12.22551	8.997583
SOCS2	0.971924	0.028076	1.336665	1.358356	12.22551	8.997583
FAHD2A	0.971946	0.028054	1.320997	1.321348	688.7294	521.23
ALKBH6	0.971949	0.028051	0.784966	0.784489	201.1651	256.4311
SOCS1	0.971958	0.028042	0.803	0.799333	24.45101	30.59178
RSF1	0.971961	0.028039	0.74206	0.741927	839.1142	1130.996
NELFA	0.971964	0.028036	0.769502	0.769206	341.2027	443.5808
PGBD2	0.971964	0.028036	0.782236	0.7818	222.2819	284.3236
DDO	0.971964	0.028036	0.707197	0.686627	5.557048	8.097825
GHR	0.971964	0.028036	0.707197	0.686627	5.557048	8.097825
VAX2	0.971964	0.028036	0.707197	0.686627	5.557048	8.097825
CCNF	0.971978	0.028022	1.328801	1.329111	802.4377	603.7378
LOC10192	0.97199	0.02801	0.735039	0.72081	7.779867	10.7971
LOC10537	0.97199	0.02801	0.735039	0.72081	7.779867	10.7971
RNF32	0.972008	0.027992	1.360599	1.389095	10.00269	7.198066
CARD14	0.972009	0.027991	0.794752	0.794089	140.0376	176.3526
MAP2K5	0.97201	0.02799	0.785747	0.785263	197.8309	251.9323
MFSD13A	0.972026	0.027974	0.810732	0.808554	40.01074	49.48671
PPM1J	0.972028	0.027972	1.232059	1.235177	53.34766	43.1884
HCN3	0.972028	0.027972	0.802926	0.801995	96.69263	120.5676
BSPRY	0.972036	0.027964	1.32924	1.329546	814.6632	612.7354
LRFN1	0.972044	0.027956	1.266343	1.267217	220.0591	173.6534
KGFLP2	0.972046	0.027954	0.78001	0.772791	13.4036	17.34734
KRT6B	0.972058	0.027942	1.948936	2.525952	2.356188	0.926751
TMEM42	0.972069	0.027931	1.252327	1.25351	152.2631	121.4674
GDPD3	0.972073	0.027927	0.798708	0.794159	20.00537	25.19323
C1RL-AS1	0.972088	0.027912	1.232239	1.235179	55.57048	44.98792
THBS4	0.972091	0.027909	1.347169	1.37202	11.1141	8.097825
PROB1	0.972095	0.027905	1.232116	1.235177	53.34766	43.1884
MS4A10	0.972096	0.027904	1.469127	1.533956	6.312806	4.111895
LIN28A	0.972101	0.027899	0.65283	0.616268	3.323115	5.39855
ZNF117	0.972106	0.027894	1.266478	1.267338	223.6378	176.4606
CIB1	0.972108	0.027892	1.369879	1.370045	1739.356	1269.559
TIFA	0.972108	0.027892	1.297596	1.298084	459.0122	353.605
LY6G5B	0.972111	0.027889	1.232691	1.235187	65.57317	53.08574
MOGS	0.972112	0.027888	1.384822	1.384954	2343.963	1692.445
PBX4	0.972118	0.027882	1.231911	1.235173	50.01343	40.48912
FYTTD1	0.972126	0.027874	1.417539	1.417621	4137.778	2918.816
TNFRSF1	0.97213	0.02787	1.422176	1.422251	4619.107	3247.741
FRAT1	0.972156	0.027844	1.231678	1.235169	46.6792	37.78985
TMCO4	0.97216	0.02784	0.761678	0.761448	450.1209	591.1412
DEPDC1-/	0.972172	0.027828	0.654689	0.618323	3.334229	5.39855
HMX3	0.972172	0.027828	0.654689	0.618323	3.334229	5.39855
KIAA2022	0.972172	0.027828	0.654689	0.618323	3.334229	5.39855
LRRC63	0.972172	0.027828	0.654689	0.618323	3.334229	5.39855
MTUS2	0.972172	0.027828	0.654689	0.618323	3.334229	5.39855

PLPPR4	0.972172	0.027828	0.654689	0.618323	3.334229	5.39855
SNORD14	0.972172	0.027828	0.654689	0.618323	3.334229	5.39855
UBXN10-A	0.972172	0.027828	0.654689	0.618323	3.334229	5.39855
UPK3A	0.972172	0.027828	0.654689	0.618323	3.334229	5.39855
ZFR2	0.972172	0.027828	0.654689	0.618323	3.334229	5.39855
ZC3HC1	0.972173	0.027827	0.764333	0.764086	414.5558	542.5543
FAM162A	0.972175	0.027825	1.391163	1.391284	2576.247	1851.703
LINC0118	0.972181	0.027819	1.232958	1.235192	73.35303	59.38405
FCRLB	0.972182	0.027818	1.376999	1.41104	8.891277	6.298308
GPR37L1	0.972182	0.027818	1.376999	1.41104	8.891277	6.298308
PLK5	0.972182	0.027818	1.376999	1.41104	8.891277	6.298308
SMIM6	0.972182	0.027818	1.376999	1.41104	8.891277	6.298308
ITPKB	0.972183	0.027817	1.251957	1.253113	155.5973	124.1666
LOC10013	0.972184	0.027816	0.514498	0.411184	1.11141	2.71727
ATP5A1	0.972186	0.027814	0.678311	0.678294	7441.999	10971.65
YY2	0.972194	0.027806	0.810219	0.808808	61.12753	75.5797
ANP32A-IT	0.972195	0.027805	0.655562	0.619347	3.345343	5.407547
HOXA10	0.972207	0.027793	1.233024	1.235193	75.57585	61.18356
LINC0149	0.972216	0.027784	1.338208	1.362508	10.79179	7.917873
OTUD6B-A	0.972228	0.027772	0.72591	0.725829	1401.487	1930.881
STAT4	0.972229	0.027771	1.231133	1.235161	41.12215	33.29106
GPR137	0.972229	0.027771	1.308087	1.3085	555.7048	424.6859
PI4K2B	0.972233	0.027767	0.756367	0.756172	536.8108	709.9093
ADGRE4P	0.972253	0.027747	0.663841	0.628671	3.367571	5.362559
CMBL	0.972254	0.027746	0.778437	0.770504	12.24773	15.89873
MEF2BNB	0.972255	0.027745	1.236222	1.238018	92.74713	74.91388
OLMALIN	0.972257	0.027743	0.796333	0.795691	146.606	184.2525
LOC28495	0.972268	0.027732	0.607832	0.552553	2.222819	4.030917
CNPY4	0.972272	0.027728	0.787791	0.787299	193.3853	245.634
MEI1	0.972274	0.027726	1.360101	1.389095	10.00269	7.198066
SPINK2	0.972274	0.027726	1.360101	1.389095	10.00269	7.198066
TMEM266	0.972274	0.027726	1.360101	1.389095	10.00269	7.198066
LOC10033	0.972284	0.027716	0.776994	0.768976	12.40333	16.13267
PCP4L1	0.972292	0.027708	0.811115	0.809786	65.57317	80.97825
TCEAL2	0.972293	0.027707	0.70756	0.686627	5.557048	8.097825
ZNF833P	0.972293	0.027707	0.70756	0.686627	5.557048	8.097825
LINC0033	0.972298	0.027702	0.792143	0.786164	15.55973	19.79468
MATN1-AS	0.972304	0.027696	1.231149	1.235159	40.01074	32.3913
LYPD5	0.972314	0.027686	1.23098	1.235157	38.89934	31.49154
ISL2	0.972317	0.027683	1.233228	1.235196	83.35572	67.48187
SMPDL3A	0.972318	0.027682	1.427866	1.481208	6.668457	4.498792
SNORA40	0.972318	0.027682	1.427866	1.481208	6.668457	4.498792
TBC1D10	0.972318	0.027682	1.427866	1.481208	6.668457	4.498792
LINC0151	0.972319	0.027681	0.527305	0.425212	1.11141	2.627294
PCDHA9	0.972334	0.027666	0.529778	0.429247	1.133638	2.654287
AKAP14	0.972345	0.027655	0.517313	0.413915	1.11141	2.699275
CHRNA9	0.972345	0.027655	0.517313	0.413915	1.11141	2.699275
CXorf58	0.972345	0.027655	0.517313	0.413915	1.11141	2.699275
FAM3B	0.972345	0.027655	0.517313	0.413915	1.11141	2.699275
FRG2C	0.972345	0.027655	0.517313	0.413915	1.11141	2.699275
LOC10013	0.972345	0.027655	0.517313	0.413915	1.11141	2.699275
MRVI1-AS	0.972345	0.027655	0.517313	0.413915	1.11141	2.699275
PAEP	0.972345	0.027655	0.517313	0.413915	1.11141	2.699275
PRG4	0.972345	0.027655	0.517313	0.413915	1.11141	2.699275
SNORA84	0.972345	0.027655	0.517313	0.413915	1.11141	2.699275
SOCS2-AS	0.972345	0.027655	0.517313	0.413915	1.11141	2.699275

TBC1D26	0.972345	0.027655	0.517313	0.413915	1.11141	2.699275
UNC13C	0.972345	0.027655	0.517313	0.413915	1.11141	2.699275
ZNF781	0.972345	0.027655	0.517313	0.413915	1.11141	2.699275
LOC10272	0.972346	0.027654	1.230857	1.235154	37.78793	30.59178
CHMP5	0.972347	0.027653	1.381563	1.381699	2201.702	1593.472
ZNF684	0.972355	0.027645	0.809244	0.808115	77.79867	96.27414
PIR-FIGF	0.972358	0.027642	0.338889	0.008608	0	1.151691
EOMES	0.972371	0.027629	1.398336	1.440288	7.779867	5.39855
MMP19	0.972371	0.027629	1.398336	1.440288	7.779867	5.39855
SCN9A	0.972371	0.027629	1.398336	1.440288	7.779867	5.39855
LOC64236	0.972371	0.027629	0.81385	0.812235	53.34766	65.68236
ANKRD36	0.972372	0.027628	1.243408	1.244763	127.412	102.3565
EIF2B5-AS	0.97238	0.02762	0.660267	0.624487	3.367571	5.39855
ADRB1	0.972381	0.027619	1.230727	1.235152	36.67652	29.69202
CTBP1-AS	0.972381	0.027619	0.803329	0.802521	113.3638	141.2621
TRIM52-A	0.972383	0.027617	0.776162	0.775823	292.3007	376.7648
SEC31B	0.972384	0.027616	0.811511	0.810228	67.79598	83.67752
CTAGE8	0.972396	0.027604	0.739609	0.73949	923.0145	1248.181
ARMCX4	0.97241	0.02759	1.268435	1.269215	248.9557	196.1473
HOXA10-A	0.97241	0.02759	1.268435	1.269215	248.9557	196.1473
PDPK1	0.972411	0.027589	0.734855	0.734751	1073.622	1461.207
NPHS1	0.972411	0.027589	0.792252	0.786164	15.55973	19.79468
TMTC4	0.972418	0.027582	1.311469	1.311846	627.9464	478.6714
PIGB	0.972426	0.027574	0.799179	0.798477	130.0349	162.8563
POU5F1P	0.972429	0.027571	2.769636	105.4725	1.044725	0
NRF1	0.972434	0.027566	0.770783	0.770496	350.094	454.3779
CRAT37	0.97244	0.02756	0.611412	0.556268	2.222819	4.003924
FANCD2	0.972447	0.027553	0.734261	0.73416	1098.084	1495.704
EME2	0.972469	0.027531	0.787356	0.780275	13.33691	17.09541
FBXO8	0.972473	0.027527	0.780525	0.780152	266.7383	341.9082
LINC0092	0.972474	0.027526	0.520691	0.418017	1.122524	2.699275
LOC10050	0.972474	0.027526	1.376411	1.41104	8.891277	6.298308
SEPSECS	0.972474	0.027526	1.376411	1.41104	8.891277	6.298308
SUGT1P1	0.972474	0.027526	1.376411	1.41104	8.891277	6.298308
TBX6	0.972474	0.027526	1.376411	1.41104	8.891277	6.298308
ZNF416	0.972479	0.027521	0.784336	0.783904	220.0591	280.7246
ANKRD36	0.972485	0.027515	1.233276	1.235051	92.41371	74.8239
FAAP24	0.972495	0.027505	0.806113	0.805204	97.80404	121.4674
LINC0099	0.972496	0.027504	0.798887	0.798225	140.6378	176.1907
DUSP22	0.9725	0.0275	1.32354	1.323852	796.8807	601.9383
PPP1R2P	0.972501	0.027499	1.915361	2.466666	2.322846	0.935749
LINC0068	0.972501	0.027499	1.46942	1.542532	5.557048	3.599033
LOC10050	0.972501	0.027499	1.46942	1.542532	5.557048	3.599033
ARL14	0.972517	0.027483	1.898903	2.454299	2.222819	0.899758
ATP10B	0.972517	0.027483	1.898903	2.454299	2.222819	0.899758
GPA33	0.972517	0.027483	1.898903	2.454299	2.222819	0.899758
KISS1R	0.972517	0.027483	1.898903	2.454299	2.222819	0.899758
LINC0099	0.972517	0.027483	1.898903	2.454299	2.222819	0.899758
LOC10050	0.972517	0.027483	1.898903	2.454299	2.222819	0.899758
LOC39971	0.972517	0.027483	1.898903	2.454299	2.222819	0.899758
LOC65378	0.972517	0.027483	1.898903	2.454299	2.222819	0.899758
RPS6KA2	0.972517	0.027483	1.898903	2.454299	2.222819	0.899758
ELMO3	0.972547	0.027453	1.278861	1.279494	321.1974	251.0326
GNL3L	0.972547	0.027453	0.735365	0.735263	1101.685	1498.359
GFRA3	0.972547	0.027453	1.230184	1.235141	32.23088	26.09299
PCDHB12	0.972559	0.027441	0.539457	0.442631	1.189208	2.699275

CTAGE15	0.972568	0.027432	0.761188	0.760976	496.9557	653.0536
VCX	0.972574	0.027426	0.349304	0.009177	0	1.07971
PRH2	0.972575	0.027425	0.437696	0.257349	0.455678	1.799517
PIK3C2A	0.972576	0.027424	0.717038	0.716978	1934.964	2698.78
AEBP1	0.972576	0.027424	1.427234	1.481208	6.668457	4.498792
ARMC12	0.972576	0.027424	1.427234	1.481208	6.668457	4.498792
ASGR1	0.972576	0.027424	1.427234	1.481208	6.668457	4.498792
CAPN9	0.972576	0.027424	1.427234	1.481208	6.668457	4.498792
FYB	0.972576	0.027424	1.427234	1.481208	6.668457	4.498792
HCG26	0.972576	0.027424	1.427234	1.481208	6.668457	4.498792
MUSTN1	0.972576	0.027424	1.427234	1.481208	6.668457	4.498792
NKAIN1	0.972576	0.027424	1.427234	1.481208	6.668457	4.498792
RPA4	0.972576	0.027424	1.427234	1.481208	6.668457	4.498792
TRIM9	0.972576	0.027424	1.427234	1.481208	6.668457	4.498792
MOCS2	0.972576	0.027424	1.323548	1.323861	780.2095	589.3417
LOC10192	0.972578	0.027422	0.752894	0.739716	7.891008	10.67113
NPIPB9	0.972586	0.027414	0.799142	0.793558	16.24881	20.4785
ZSWIM3	0.972591	0.027409	0.799199	0.798545	142.2604	178.1521
FAM45BP	0.972603	0.027397	0.734569	0.734469	1105.286	1504.882
AAK1	0.972606	0.027394	0.717255	0.717195	1926.895	2686.714
HYDIN	0.972609	0.027391	0.655309	0.618323	3.334229	5.39855
LOC10012	0.972609	0.027391	0.655309	0.618323	3.334229	5.39855
LOC10537	0.972609	0.027391	0.655309	0.618323	3.334229	5.39855
NKAPP1	0.972609	0.027391	0.655309	0.618323	3.334229	5.39855
PTPRCAP	0.972609	0.027391	0.655309	0.618323	3.334229	5.39855
SLITRK4	0.972609	0.027391	0.655309	0.618323	3.334229	5.39855
ZNF69	0.972609	0.027391	0.655309	0.618323	3.334229	5.39855
ZNF7	0.97261	0.02739	0.75988	0.759676	520.1397	684.6891
LOC28314	0.972612	0.027388	0.787485	0.780275	13.33691	17.09541
SYNE3	0.972614	0.027386	0.806101	0.805204	97.80404	121.4674
SPATA24	0.972621	0.027379	0.818264	0.816171	41.12215	50.38647
ZBTB18	0.972628	0.027372	1.278551	1.279179	323.4202	252.8321
ZNF195	0.972628	0.027372	0.784809	0.784397	237.8417	303.2185
ATL3	0.97263	0.02737	0.723283	0.723211	1587.749	2195.419
ZNF177	0.972634	0.027366	0.803998	0.799037	17.97149	22.49396
ZNF169	0.972637	0.027363	0.817603	0.815294	36.67652	44.98792
ZNF331	0.97264	0.02736	0.729132	0.729046	1317.832	1807.614
ARID5A	0.972643	0.027357	1.288553	1.289084	398.996	309.5169
BLACE	0.972664	0.027336	0.523035	0.419488	1.11141	2.663285
RBAK	0.972666	0.027334	0.753504	0.753333	617.9437	820.2827
CCDC186	0.972668	0.027332	1.391326	1.39144	2781.858	1999.263
PSMC3	0.972669	0.027331	0.71824	0.718179	1877.171	2613.798
HRCT1	0.972673	0.027327	1.533831	1.644587	4.445638	2.699275
SULT1C2	0.972676	0.027324	0.818226	0.816171	41.12215	50.38647
PNPLA1	0.972688	0.027312	1.474929	1.550261	5.557048	3.581038
GSKIP	0.972691	0.027309	0.740723	0.740604	920.2471	1242.566
NOXO1	0.972692	0.027308	0.780843	0.772178	11.1141	14.39613
WDR86	0.972692	0.027308	0.780843	0.772178	11.1141	14.39613
CNTFR	0.972695	0.027305	1.397621	1.440288	7.779867	5.39855
RTCA-AS1	0.972695	0.027305	1.397621	1.440288	7.779867	5.39855
SNORA70	0.972695	0.027305	1.397621	1.440288	7.779867	5.39855
SLC4A2	0.972695	0.027305	0.702562	0.702524	3121.95	4443.906
IRAK1BP1	0.972697	0.027303	0.806191	0.80534	104.4725	129.7272
HEATR5B	0.972714	0.027286	0.760861	0.760655	511.2484	672.1195
TIMM21	0.972718	0.027282	0.786142	0.78572	231.1732	294.221
ANO1	0.972721	0.027279	1.229544	1.235131	28.89665	23.39372

RBM18	0.972748	0.027252	1.33694	1.337192	1020.274	762.995
TYW5	0.972754	0.027246	0.77278	0.772489	344.0702	445.4074
OMA1	0.972757	0.027243	0.774722	0.774411	315.6403	407.5905
ZNF232	0.972757	0.027243	0.812871	0.811743	76.68726	94.47462
PNMAL1	0.972766	0.027234	0.773838	0.77354	333.4229	431.0382
CECR6	0.97277	0.02723	1.647115	1.848134	3.334229	1.799517
LKAAEAR	0.97277	0.02723	1.647115	1.848134	3.334229	1.799517
OTOGL	0.97277	0.02723	1.647115	1.848134	3.334229	1.799517
FFAR4	0.972792	0.027208	1.468577	1.542532	5.557048	3.599033
FLJ45513	0.972792	0.027208	1.468577	1.542532	5.557048	3.599033
GSC	0.972792	0.027208	1.468577	1.542532	5.557048	3.599033
HIST1H4E	0.972792	0.027208	1.468577	1.542532	5.557048	3.599033
LOC10192	0.972792	0.027208	1.468577	1.542532	5.557048	3.599033
OCLM	0.972792	0.027208	1.468577	1.542532	5.557048	3.599033
REM2	0.972792	0.027208	1.468577	1.542532	5.557048	3.599033
SERPINA2	0.972792	0.027208	1.468577	1.542532	5.557048	3.599033
UTS2B	0.972792	0.027208	1.468577	1.542532	5.557048	3.599033
SDHAF4	0.972796	0.027204	0.800327	0.799661	136.7034	170.9541
ICAM2	0.972797	0.027203	0.814358	0.810686	23.3396	28.79227
MSRB3	0.972811	0.027189	1.332744	1.333009	951.3444	713.6793
WASIR2	0.972824	0.027176	1.375563	1.414405	7.779867	5.497523
EFCAB5	0.972825	0.027175	1.229215	1.235127	27.78524	22.49396
ATP6V0E2	0.972834	0.027166	0.819146	0.817462	50.01343	61.18356
ZNF44	0.972836	0.027164	0.801191	0.800497	128.9235	161.0567
ZNF519	0.972851	0.027149	0.813165	0.81207	78.91008	97.1739
PQLC1	0.972858	0.027142	1.279922	1.280513	345.6484	269.9275
PARK2	0.972862	0.027138	0.778096	0.768502	10.11383	13.16346
SCMH1	0.972864	0.027136	0.786487	0.786064	225.6161	287.0229
LOC10050	0.972871	0.027129	0.781	0.772178	11.1141	14.39613
SPNS3	0.972871	0.027129	0.781	0.772178	11.1141	14.39613
LOC72921	0.97288	0.02712	0.806128	0.805326	110.9631	137.789
RPSAP9	0.972881	0.027119	1.236967	1.238314	124.0444	100.1701
LOC40092	0.972886	0.027114	1.220012	1.224148	37.07662	30.28586
PCDHB14	0.972888	0.027112	0.81273	0.808588	21.20569	26.22795
RAB40C	0.97289	0.02711	0.783887	0.783496	246.7329	314.9154
DAGLB	0.972894	0.027106	0.773904	0.77361	338.9799	438.1823
HMGB3P1	0.972898	0.027102	1.901857	2.466516	2.233933	0.899758
CRY2	0.972904	0.027096	1.259464	1.260297	223.3933	177.2524
RAB3A	0.972913	0.027087	1.249848	1.250849	177.8255	142.1618
JDP2	0.972917	0.027083	1.334879	1.335135	980.2632	734.2028
LOC10192	0.972919	0.027081	0.724582	0.705041	5.757102	8.169805
ORM1	0.972922	0.027078	0.73668	0.719732	6.479518	9.006581
ABL1	0.972923	0.027077	1.377592	1.377728	2170.583	1575.477
DIRAS3	0.97293	0.02707	1.228822	1.235118	25.56242	20.69444
GPR132	0.972936	0.027064	1.426331	1.481208	6.668457	4.498792
SHISA7	0.972936	0.027064	1.426331	1.481208	6.668457	4.498792
SLC35E4	0.972959	0.027041	1.233783	1.235206	115.5866	93.57486
ABHD17A	0.972967	0.027033	1.292197	1.292675	450.1209	348.2065
CATIP-AS	0.972986	0.027014	0.771467	0.760347	8.891277	11.69686
IL36RN	0.972986	0.027014	0.771467	0.760347	8.891277	11.69686
DNAJC24	0.972994	0.027006	1.263712	1.26447	250.3006	197.9468
PLS3-AS1	0.972999	0.027001	0.819649	0.818166	56.68189	69.28139
ABALON	0.973004	0.026996	1.894344	2.454299	2.222819	0.899758
ARHGAP2	0.973004	0.026996	1.894344	2.454299	2.222819	0.899758
ARHGEF3	0.973004	0.026996	1.894344	2.454299	2.222819	0.899758
AWAT2	0.973004	0.026996	1.894344	2.454299	2.222819	0.899758

B3GNT6	0.973004	0.026996	1.894344	2.454299	2.222819	0.899758
C11orf91	0.973004	0.026996	1.894344	2.454299	2.222819	0.899758
C2CD4D	0.973004	0.026996	1.894344	2.454299	2.222819	0.899758
C2orf82	0.973004	0.026996	1.894344	2.454299	2.222819	0.899758
C3orf80	0.973004	0.026996	1.894344	2.454299	2.222819	0.899758
C9orf170	0.973004	0.026996	1.894344	2.454299	2.222819	0.899758
CACNA1A	0.973004	0.026996	1.894344	2.454299	2.222819	0.899758
CCDC17	0.973004	0.026996	1.894344	2.454299	2.222819	0.899758
CCNYL2	0.973004	0.026996	1.894344	2.454299	2.222819	0.899758
CHRNA3	0.973004	0.026996	1.894344	2.454299	2.222819	0.899758
CHRNA6	0.973004	0.026996	1.894344	2.454299	2.222819	0.899758
CYP2C18	0.973004	0.026996	1.894344	2.454299	2.222819	0.899758
DQX1	0.973004	0.026996	1.894344	2.454299	2.222819	0.899758
ECEL1	0.973004	0.026996	1.894344	2.454299	2.222819	0.899758
FAM178B	0.973004	0.026996	1.894344	2.454299	2.222819	0.899758
FLJ44635	0.973004	0.026996	1.894344	2.454299	2.222819	0.899758
FMO3	0.973004	0.026996	1.894344	2.454299	2.222819	0.899758
FPR2	0.973004	0.026996	1.894344	2.454299	2.222819	0.899758
FSCN3	0.973004	0.026996	1.894344	2.454299	2.222819	0.899758
FSTL5	0.973004	0.026996	1.894344	2.454299	2.222819	0.899758
GAS2L2	0.973004	0.026996	1.894344	2.454299	2.222819	0.899758
GRM4	0.973004	0.026996	1.894344	2.454299	2.222819	0.899758
GRM8	0.973004	0.026996	1.894344	2.454299	2.222819	0.899758
HIPK4	0.973004	0.026996	1.894344	2.454299	2.222819	0.899758
HIST1H2E	0.973004	0.026996	1.894344	2.454299	2.222819	0.899758
HOXD1	0.973004	0.026996	1.894344	2.454299	2.222819	0.899758
HSPB6	0.973004	0.026996	1.894344	2.454299	2.222819	0.899758
HTR7	0.973004	0.026996	1.894344	2.454299	2.222819	0.899758
KCCAT33	0.973004	0.026996	1.894344	2.454299	2.222819	0.899758
KCNE5	0.973004	0.026996	1.894344	2.454299	2.222819	0.899758
KRTAP5-9	0.973004	0.026996	1.894344	2.454299	2.222819	0.899758
LIMD1-AS	0.973004	0.026996	1.894344	2.454299	2.222819	0.899758
LINC0023	0.973004	0.026996	1.894344	2.454299	2.222819	0.899758
LINC0055	0.973004	0.026996	1.894344	2.454299	2.222819	0.899758
LINC0062	0.973004	0.026996	1.894344	2.454299	2.222819	0.899758
LINC0135	0.973004	0.026996	1.894344	2.454299	2.222819	0.899758
LINC0151	0.973004	0.026996	1.894344	2.454299	2.222819	0.899758
LINC0154	0.973004	0.026996	1.894344	2.454299	2.222819	0.899758
LINC0162	0.973004	0.026996	1.894344	2.454299	2.222819	0.899758
LOC10012	0.973004	0.026996	1.894344	2.454299	2.222819	0.899758
LOC10012	0.973004	0.026996	1.894344	2.454299	2.222819	0.899758
LOC10013	0.973004	0.026996	1.894344	2.454299	2.222819	0.899758
LOC10050	0.973004	0.026996	1.894344	2.454299	2.222819	0.899758
LOC10050	0.973004	0.026996	1.894344	2.454299	2.222819	0.899758
LOC10192	0.973004	0.026996	1.894344	2.454299	2.222819	0.899758
LOC10192	0.973004	0.026996	1.894344	2.454299	2.222819	0.899758
LOC10272	0.973004	0.026996	1.894344	2.454299	2.222819	0.899758
LOC65029	0.973004	0.026996	1.894344	2.454299	2.222819	0.899758
MAGEA4	0.973004	0.026996	1.894344	2.454299	2.222819	0.899758
MDGA2	0.973004	0.026996	1.894344	2.454299	2.222819	0.899758
MMP13	0.973004	0.026996	1.894344	2.454299	2.222819	0.899758
MS4A2	0.973004	0.026996	1.894344	2.454299	2.222819	0.899758
MT1DP	0.973004	0.026996	1.894344	2.454299	2.222819	0.899758
MUC22	0.973004	0.026996	1.894344	2.454299	2.222819	0.899758
MYOC	0.973004	0.026996	1.894344	2.454299	2.222819	0.899758
NRADDP	0.973004	0.026996	1.894344	2.454299	2.222819	0.899758

OSMR-AS	0.973004	0.026996	1.894344	2.454299	2.222819	0.899758
OSTCP1	0.973004	0.026996	1.894344	2.454299	2.222819	0.899758
PLEK	0.973004	0.026996	1.894344	2.454299	2.222819	0.899758
PRR15L	0.973004	0.026996	1.894344	2.454299	2.222819	0.899758
SAP30L-A	0.973004	0.026996	1.894344	2.454299	2.222819	0.899758
SCARNA2	0.973004	0.026996	1.894344	2.454299	2.222819	0.899758
SMOC1	0.973004	0.026996	1.894344	2.454299	2.222819	0.899758
SPOCK3	0.973004	0.026996	1.894344	2.454299	2.222819	0.899758
TRIM31	0.973004	0.026996	1.894344	2.454299	2.222819	0.899758
TWIST2	0.973004	0.026996	1.894344	2.454299	2.222819	0.899758
VRTN	0.973004	0.026996	1.894344	2.454299	2.222819	0.899758
WISP1	0.973004	0.026996	1.894344	2.454299	2.222819	0.899758
C5orf66	0.973005	0.026995	1.532609	1.644587	4.445638	2.699275
C6orf141	0.973005	0.026995	1.532609	1.644587	4.445638	2.699275
CHST13	0.973005	0.026995	1.532609	1.644587	4.445638	2.699275
LINC0109	0.973005	0.026995	1.532609	1.644587	4.445638	2.699275
LOC10192	0.973005	0.026995	1.532609	1.644587	4.445638	2.699275
LRP4-AS1	0.973005	0.026995	1.532609	1.644587	4.445638	2.699275
LRRD1	0.973005	0.026995	1.532609	1.644587	4.445638	2.699275
MYO16-AS	0.973005	0.026995	1.532609	1.644587	4.445638	2.699275
SPTSSB	0.973005	0.026995	1.532609	1.644587	4.445638	2.699275
TMEM150	0.973005	0.026995	1.532609	1.644587	4.445638	2.699275
ZDHHC11	0.973005	0.026995	1.532609	1.644587	4.445638	2.699275
ATP5EP2	0.97301	0.02699	1.692636	1.920448	3.378685	1.754529
LIME1	0.973037	0.026963	1.23149	1.233005	105.9284	85.90892
KLHL28	0.973046	0.026954	1.287802	1.2883	432.1605	335.4479
CNP	0.973052	0.026948	1.357387	1.357567	1529.3	1126.497
SLC4A5	0.973056	0.026944	0.820038	0.816723	25.71802	31.49154
DHDDS	0.973065	0.026935	1.347419	1.347628	1279.232	949.245
LOC64647	0.973065	0.026935	0.550989	0.449763	1.11141	2.483333
CNTNAP2	0.97308	0.02692	0.812386	0.807733	18.89396	23.39372
H1FX-AS1	0.973084	0.026916	1.649746	1.854276	3.345343	1.799517
LIMS3-LO	0.973087	0.026913	0.812272	0.811354	96.23696	118.6151
CRTC3	0.97309	0.02691	0.758742	0.758556	571.2645	753.0977
ACADM	0.973092	0.026908	0.738099	0.737994	1042.502	1412.621
LOC10106	0.973108	0.026892	1.886831	2.442083	2.211705	0.899758
MAN1B1-A	0.973117	0.026883	1.29064	1.308681	12.22551	9.339491
PRELID3E	0.97312	0.02688	0.718791	0.718731	1927.484	2681.793
PITPNC1	0.973135	0.026865	1.248803	1.249747	191.1624	152.9589
ZFYVE27	0.973142	0.026858	1.337347	1.337587	1074.733	803.4842
PHACTR2	0.973149	0.026851	0.727197	0.72712	1475.952	2029.864
BTBD8	0.973158	0.026842	0.810882	0.80568	16.67114	20.69444
SOCS5	0.973158	0.026842	0.758272	0.758087	566.8189	747.6992
SGMS2	0.973158	0.026842	1.314719	1.315047	732.4189	556.9504
ADAMTSL	0.973159	0.026841	1.645075	1.848134	3.334229	1.799517
AQP5	0.973159	0.026841	1.645075	1.848134	3.334229	1.799517
C3orf35	0.973159	0.026841	1.645075	1.848134	3.334229	1.799517
CCDC67	0.973159	0.026841	1.645075	1.848134	3.334229	1.799517
CD4	0.973159	0.026841	1.645075	1.848134	3.334229	1.799517
CPXM2	0.973159	0.026841	1.645075	1.848134	3.334229	1.799517
DEFB103A	0.973159	0.026841	1.645075	1.848134	3.334229	1.799517
DEFB103B	0.973159	0.026841	1.645075	1.848134	3.334229	1.799517
DRD2	0.973159	0.026841	1.645075	1.848134	3.334229	1.799517
FLJ38576	0.973159	0.026841	1.645075	1.848134	3.334229	1.799517
GPR68	0.973159	0.026841	1.645075	1.848134	3.334229	1.799517
GRM2	0.973159	0.026841	1.645075	1.848134	3.334229	1.799517

GRPR	0.973159	0.026841	1.645075	1.848134	3.334229	1.799517
HHIP	0.973159	0.026841	1.645075	1.848134	3.334229	1.799517
IGSF11	0.973159	0.026841	1.645075	1.848134	3.334229	1.799517
INHBC	0.973159	0.026841	1.645075	1.848134	3.334229	1.799517
IRAIN	0.973159	0.026841	1.645075	1.848134	3.334229	1.799517
LNX1-AS2	0.973159	0.026841	1.645075	1.848134	3.334229	1.799517
LOC10099	0.973159	0.026841	1.645075	1.848134	3.334229	1.799517
LOC40158	0.973159	0.026841	1.645075	1.848134	3.334229	1.799517
LRRC39	0.973159	0.026841	1.645075	1.848134	3.334229	1.799517
MRC1	0.973159	0.026841	1.645075	1.848134	3.334229	1.799517
NKX2-3	0.973159	0.026841	1.645075	1.848134	3.334229	1.799517
OR1F1	0.973159	0.026841	1.645075	1.848134	3.334229	1.799517
PSG7	0.973159	0.026841	1.645075	1.848134	3.334229	1.799517
ROBO4	0.973159	0.026841	1.645075	1.848134	3.334229	1.799517
SCOC-AS	0.973159	0.026841	1.645075	1.848134	3.334229	1.799517
SMIM2-AS	0.973159	0.026841	1.645075	1.848134	3.334229	1.799517
TEKT4	0.973159	0.026841	1.645075	1.848134	3.334229	1.799517
TRIM67	0.973159	0.026841	1.645075	1.848134	3.334229	1.799517
ZG16B	0.973159	0.026841	1.645075	1.848134	3.334229	1.799517
C1GALT1	0.973181	0.026819	0.756614	0.741426	6.668457	8.997583
SCHIP1	0.973186	0.026814	1.299957	1.300369	539.5449	414.9145
BCL2L14	0.973196	0.026804	1.467374	1.542532	5.557048	3.599033
BCL6B	0.973196	0.026804	1.467374	1.542532	5.557048	3.599033
PAX8-AS1	0.973196	0.026804	1.467374	1.542532	5.557048	3.599033
SLC26A4-	0.973196	0.026804	1.467374	1.542532	5.557048	3.599033
SPANXB1	0.973196	0.026804	1.467374	1.542532	5.557048	3.599033
SPATA25	0.973196	0.026804	1.467374	1.542532	5.557048	3.599033
ZNF793-A	0.973196	0.026804	1.467374	1.542532	5.557048	3.599033
PPWD1	0.973197	0.026803	0.765491	0.765271	472.3491	617.2342
LENG8	0.9732	0.0268	1.408561	1.408645	3891.045	2762.258
ARRDC1-/	0.973202	0.026798	1.258521	1.259319	232.2846	184.4505
SEPT10	0.973203	0.026797	1.40344	1.40353	3549.842	2529.221
IL2RB	0.973209	0.026791	0.771667	0.760347	8.891277	11.69686
LOC10192	0.973212	0.026788	1.879318	2.429866	2.200591	0.899758
TRIM50	0.973225	0.026775	0.364204	0.009913	0	0.998732
LINC0031	0.973233	0.026767	1.638877	1.83899	3.334229	1.808514
FREM2	0.973234	0.026766	1.640403	1.841992	3.323115	1.799517
FAM21EP	0.973243	0.026757	1.46374	1.537943	5.568162	3.617028
SMARCE1	0.973245	0.026755	0.729858	0.729776	1370.368	1877.796
EVPLL	0.973256	0.026744	0.810978	0.80568	16.67114	20.69444
ARHGAP6	0.973259	0.026741	0.825689	0.823526	37.78793	45.88767
FLJ37453	0.973265	0.026735	0.825825	0.823528	35.56511	43.1884
HPD	0.973265	0.026735	0.825825	0.823528	35.56511	43.1884
LOC10013	0.973265	0.026735	0.825825	0.823528	35.56511	43.1884
LINC0120	0.973274	0.026726	1.446459	1.517632	5.412565	3.563043
RING1	0.973294	0.026706	0.752897	0.752741	677.9598	900.6581
RPA2	0.973296	0.026704	1.35433	1.354512	1527.077	1127.397
PRIM2	0.973297	0.026703	0.749474	0.749332	752.4243	1004.13
TMEM254	0.973315	0.026685	0.767119	0.766892	456.7893	595.64
ZNF767P	0.973337	0.026663	1.251691	1.252547	213.6685	170.5852
RPGRIP1I	0.973339	0.026661	0.792974	0.792504	198.9423	251.0326
DUXAP8	0.97334	0.02666	1.308408	1.308764	646.0068	493.5984
LOC10050	0.973342	0.026658	0.804812	0.804156	138.9262	172.7626
PIEZO1	0.973344	0.026656	1.420397	1.420466	4883.534	3437.976
LINC0113	0.973354	0.026646	0.809051	0.80301	14.44832	17.99517
MX2	0.973354	0.026646	0.809051	0.80301	14.44832	17.99517

RAB11B-A	0.973354	0.026646	0.809051	0.80301	14.44832	17.99517
PLEKHF2	0.973354	0.026646	0.761711	0.761514	523.4739	687.4153
THG1L	0.973354	0.026646	0.780476	0.780152	306.749	393.1944
ANKZF1	0.973354	0.026646	1.335965	1.336203	1073.622	803.4842
ZNF746	0.973363	0.026637	0.763217	0.763011	500.8456	656.4097
GMCL1P1	0.973364	0.026636	0.826618	0.824495	37.83238	45.88767
ZNF197	0.973368	0.026632	1.248268	1.249173	198.9423	159.2572
SPRTN	0.973379	0.026621	0.756243	0.756073	619.0551	818.7801
ACP5	0.97338	0.02662	0.756805	0.741426	6.668457	8.997583
CD8A	0.97338	0.02662	0.756805	0.741426	6.668457	8.997583
CPA2	0.97338	0.02662	0.756805	0.741426	6.668457	8.997583
DUSP26	0.97338	0.02662	0.756805	0.741426	6.668457	8.997583
HIST1H1E	0.97338	0.02662	0.756805	0.741426	6.668457	8.997583
KLHL41	0.97338	0.02662	0.756805	0.741426	6.668457	8.997583
LOC28569	0.97338	0.02662	0.756805	0.741426	6.668457	8.997583
VWA2	0.97338	0.02662	0.756805	0.741426	6.668457	8.997583
RASEF	0.973387	0.026613	0.766218	0.765996	459.0122	599.239
CORO1A	0.973399	0.026601	1.209807	1.212313	58.90471	48.58695
TCF15	0.973425	0.026575	1.208842	1.211432	56.68189	46.78743
TPGS1	0.973428	0.026572	0.826647	0.823542	26.67383	32.3913
MARCH1	0.973439	0.026561	1.213094	1.21527	67.79598	55.78501
LAPTM4A	0.97344	0.02656	1.426292	1.426356	5482.583	3843.767
FAM105A	0.97344	0.02656	1.214106	1.216191	71.13021	58.48429
LINC0089	0.973441	0.026559	1.232094	1.24118	18.04929	14.54009
SLC35B2	0.973441	0.026559	1.370282	1.370425	2061.665	1504.396
C1S	0.973458	0.026542	1.215601	1.217551	76.68726	62.98308
ACBD7	0.973458	0.026542	1.269238	1.269874	306.2711	241.1802
CDK4	0.973461	0.026539	0.699044	0.699013	3857.703	5518.794
CREBBP	0.973463	0.026537	0.730948	0.730866	1391.485	1903.889
HUS1B	0.973464	0.026536	1.530868	1.644587	4.445638	2.699275
MORF4L2	0.973464	0.026536	1.530868	1.644587	4.445638	2.699275
SLCO5A1	0.973464	0.026536	1.530868	1.644587	4.445638	2.699275
SMG7-AS	0.973464	0.026536	1.530868	1.644587	4.445638	2.699275
SOX17	0.973465	0.026535	0.735775	0.735681	1198.1	1628.563
STAG3L1	0.973465	0.026535	0.815523	0.814552	86.95669	106.7563
FRMD6-AS	0.973467	0.026533	1.330781	1.36203	8.202203	6.019383
KCNE4	0.973471	0.026529	1.654545	1.863436	3.378685	1.808514
RAB27A	0.973479	0.026521	1.363951	1.364108	1799.594	1319.244
NRROS	0.973495	0.026505	1.226958	1.235086	20.00537	16.19565
RAD50	0.973501	0.026499	0.702023	0.701989	3495.383	4979.262
C1RL	0.973508	0.026492	0.795315	0.794828	190.4289	239.5876
FLRT3	0.973511	0.026489	1.217371	1.219158	84.46713	69.28139
VWF	0.973512	0.026488	1.208379	1.210965	55.57048	45.88767
C3orf33	0.973523	0.026477	0.813025	0.812179	102.3053	125.9662
MSRA	0.973525	0.026475	1.207849	1.21048	54.45907	44.98792
CAPN10	0.973526	0.026474	1.261239	1.261955	262.2927	207.8442
ZNF865	0.973538	0.026462	0.749999	0.749859	761.36	1015.341
CR1	0.97355	0.02645	1.654631	1.866698	3.334229	1.781521
IGBP1	0.97355	0.02645	1.340348	1.340567	1188.097	886.2619
LOC10192	0.973555	0.026445	1.208541	1.210981	58.93805	48.66793
NETO2	0.973556	0.026444	1.357744	1.357915	1623.769	1195.779
LINC0144	0.973557	0.026443	0.827887	0.824555	24.25096	29.4131
SFTPB	0.973558	0.026442	0.69559	0.663531	3.578739	5.39855
ZNF224	0.973563	0.026437	1.247842	1.248716	205.6108	164.6558
TCTA	0.973564	0.026436	0.755966	0.7558	624.6122	826.428
NFKB1	0.973565	0.026435	1.337051	1.33728	1121.412	838.5747

LAMB2	0.973581	0.026419	0.705921	0.705883	3164.183	4482.596
PODXL2	0.973588	0.026412	1.322197	1.322479	859.1196	649.6255
GAMT	0.973589	0.026411	0.80151	0.800936	157.8202	197.0471
TYW1B	0.973592	0.026408	1.206612	1.209229	54.37016	44.96092
DUSP15	0.973595	0.026405	1.205501	1.208328	50.01343	41.38888
USP32P2	0.973604	0.026396	0.821208	0.8164	17.28242	21.17131
ZNF491	0.973605	0.026395	0.806596	0.799398	12.22551	15.29589
CROCC	0.973608	0.026392	0.800729	0.800171	162.6659	203.2914
AIPL1	0.973609	0.026391	0.815285	0.809479	14.65949	18.11213
ZFAND4	0.973616	0.026384	0.800113	0.799578	173.3799	216.8418
PEX12	0.973625	0.026375	0.824831	0.823511	62.23894	75.5797
LOC44030	0.973626	0.026374	1.25994	1.275275	12.3033	9.645409
GPATCH1	0.973632	0.026368	0.757549	0.757381	632.3921	834.9757
CXCL1	0.973635	0.026365	0.708511	0.70847	2916.339	4116.394
LCMT1-AS	0.973638	0.026362	1.226127	1.235068	17.78255	14.39613
UPB1	0.973638	0.026362	1.226127	1.235068	17.78255	14.39613
DNHD1	0.973638	0.026362	0.804401	0.803797	151.1517	188.0495
PAK1	0.973639	0.026361	1.329039	1.329295	958.0351	720.7064
ST7-AS1	0.97364	0.02636	0.827265	0.823553	22.22819	26.99275
ZNF625	0.973657	0.026343	0.710081	0.683409	4.301155	6.298308
TMPRSS5	0.973659	0.026341	0.757078	0.741426	6.668457	8.997583
IQCC	0.973662	0.026338	0.817653	0.816699	88.91277	108.8708
CHST3	0.973663	0.026337	0.725757	0.725687	1597.096	2200.809
CEACAM8	0.973664	0.026336	0.628318	0.572965	2.222819	3.886956
RASA2	0.973669	0.026331	1.375077	1.375208	2238.379	1627.663
AQP9	0.97367	0.02633	1.887889	2.454299	2.222819	0.899758
ARL9	0.97367	0.02633	1.887889	2.454299	2.222819	0.899758
CYP7B1	0.97367	0.02633	1.887889	2.454299	2.222819	0.899758
FALEC	0.97367	0.02633	1.887889	2.454299	2.222819	0.899758
GABRA5	0.97367	0.02633	1.887889	2.454299	2.222819	0.899758
HIST1H2E	0.97367	0.02633	1.887889	2.454299	2.222819	0.899758
IGFBP7-A	0.97367	0.02633	1.887889	2.454299	2.222819	0.899758
ITGAM	0.97367	0.02633	1.887889	2.454299	2.222819	0.899758
JAML	0.97367	0.02633	1.887889	2.454299	2.222819	0.899758
LINC0136	0.97367	0.02633	1.887889	2.454299	2.222819	0.899758
LOC10012	0.97367	0.02633	1.887889	2.454299	2.222819	0.899758
LOC10028	0.97367	0.02633	1.887889	2.454299	2.222819	0.899758
LOC10192	0.97367	0.02633	1.887889	2.454299	2.222819	0.899758
LOC10272	0.97367	0.02633	1.887889	2.454299	2.222819	0.899758
LOC73066	0.97367	0.02633	1.887889	2.454299	2.222819	0.899758
LUM	0.97367	0.02633	1.887889	2.454299	2.222819	0.899758
MYH11	0.97367	0.02633	1.887889	2.454299	2.222819	0.899758
PDGFRA	0.97367	0.02633	1.887889	2.454299	2.222819	0.899758
PLAC8L1	0.97367	0.02633	1.887889	2.454299	2.222819	0.899758
PLSCR2	0.97367	0.02633	1.887889	2.454299	2.222819	0.899758
SLC17A8	0.97367	0.02633	1.887889	2.454299	2.222819	0.899758
SLC18A3	0.97367	0.02633	1.887889	2.454299	2.222819	0.899758
SLC22A3	0.97367	0.02633	1.887889	2.454299	2.222819	0.899758
SLC26A10	0.97367	0.02633	1.887889	2.454299	2.222819	0.899758
SLC52A1	0.97367	0.02633	1.887889	2.454299	2.222819	0.899758
SNCAIP	0.97367	0.02633	1.887889	2.454299	2.222819	0.899758
SNORA4	0.97367	0.02633	1.887889	2.454299	2.222819	0.899758
SOAT2	0.97367	0.02633	1.887889	2.454299	2.222819	0.899758
SP9	0.97367	0.02633	1.887889	2.454299	2.222819	0.899758
TARID	0.97367	0.02633	1.887889	2.454299	2.222819	0.899758
TMEM132	0.97367	0.02633	1.887889	2.454299	2.222819	0.899758

TMEM240	0.97367	0.02633	1.887889	2.454299	2.222819	0.899758
TMOD4	0.97367	0.02633	1.887889	2.454299	2.222819	0.899758
UBAP1L	0.97367	0.02633	1.887889	2.454299	2.222819	0.899758
ZNF876P	0.973673	0.026327	1.612374	1.80514	3.25643	1.799517
UBL7-AS1	0.973674	0.026326	0.831496	0.829365	37.35448	45.0419
SDR39U1	0.973675	0.026325	0.806539	0.805884	135.592	168.2548
G6PC3	0.973676	0.026324	0.77775	0.777459	337.8685	434.5833
ZNF417	0.97368	0.02632	1.29538	1.295797	516.6499	398.7099
BTRC	0.973683	0.026317	0.759653	0.759474	580.1558	763.8948
HOXC-AS	0.973696	0.026304	1.642172	1.848134	3.334229	1.799517
LINC0088	0.973696	0.026304	1.642172	1.848134	3.334229	1.799517
LOC10013	0.973696	0.026304	1.642172	1.848134	3.334229	1.799517
LOC38924	0.973696	0.026304	1.642172	1.848134	3.334229	1.799517
PCAT1	0.973696	0.026304	1.642172	1.848134	3.334229	1.799517
PFN1P2	0.973696	0.026304	1.642172	1.848134	3.334229	1.799517
PRC1-AS1	0.973696	0.026304	1.642172	1.848134	3.334229	1.799517
SLC26A8	0.973696	0.026304	1.642172	1.848134	3.334229	1.799517
SMAD5-AS	0.973696	0.026304	1.642172	1.848134	3.334229	1.799517
SNORA50	0.973696	0.026304	1.642172	1.848134	3.334229	1.799517
STAM-AS1	0.973696	0.026304	1.642172	1.848134	3.334229	1.799517
TM4SF18	0.973696	0.026304	1.642172	1.848134	3.334229	1.799517
TMEM151	0.973696	0.026304	1.642172	1.848134	3.334229	1.799517
MGAT1	0.973699	0.026301	1.411565	1.411641	4420.076	3131.159
SAMD8	0.973702	0.026298	1.368282	1.368425	2043.882	1493.599
NCKAP5L	0.973706	0.026294	0.739401	0.739298	1070.287	1447.711
CTSZ	0.973706	0.026294	1.414952	1.415025	4583.453	3239.13
LMX1B	0.973707	0.026293	0.832305	0.829758	31.10835	37.49293
ZBTB8A	0.973711	0.026289	0.791391	0.790971	224.0713	283.2889
FOXD4L1	0.973727	0.026273	0.641641	0.587896	2.222819	3.787982
PPARGC1	0.973731	0.026269	0.77589	0.775615	360.0967	464.2753
HSDL2	0.973746	0.026254	1.34212	1.342329	1253.67	933.9491
PAQR3	0.973746	0.026254	1.295139	1.295543	549.0363	423.7862
PPP3CB	0.973752	0.026248	0.759996	0.759819	596.8269	785.489
LOC10192	0.973758	0.026242	1.225965	1.235068	17.78255	14.39613
BHLHE40-	0.973762	0.026238	0.806727	0.799398	12.22551	15.29589
M1AP	0.973766	0.026234	0.832482	0.830619	43.34497	52.18598
TBX2	0.973771	0.026229	0.832966	0.831008	41.12215	49.48671
KLHL31	0.973773	0.026227	1.225545	1.235057	16.67114	13.49637
ZNF213-A	0.973773	0.026227	1.225545	1.235057	16.67114	13.49637
ATXN7L2	0.973784	0.026216	0.833506	0.831442	38.89934	46.78743
CCDC81	0.973785	0.026215	1.200251	1.203501	42.23356	35.09057
SH2D4A	0.973786	0.026214	1.336021	1.336245	1161.423	869.1665
PCDHGA4	0.973791	0.026209	1.239727	1.251994	13.92596	11.12101
ZNF573	0.973792	0.026208	1.199284	1.202666	41.12215	34.19082
ACOXL	0.973795	0.026205	0.831285	0.829661	50.01343	60.28381
VPS16	0.973813	0.026187	0.753674	0.753526	725.0614	962.2285
RPS18P9	0.973815	0.026185	0.814909	0.808112	12.35887	15.29589
ROR1-AS1	0.973817	0.026183	1.234089	1.245426	14.63726	11.75084
MS4A15	0.97382	0.02618	1.199346	1.202666	41.12215	34.19082
CRADD	0.973829	0.026171	0.833469	0.831442	38.89934	46.78743
LARP4B	0.973856	0.026144	0.729636	0.72956	1470.395	2015.459
NSMF	0.973857	0.026143	1.326181	1.326439	953.5894	718.9069
C11orf58	0.973861	0.026139	0.712004	0.711959	2588.784	3636.148
PGM2	0.97387	0.02613	1.342584	1.342787	1318.132	981.6363
SLC7A5	0.973871	0.026129	0.669586	0.669575	12092.85	18060.49
IL1RN	0.973875	0.026125	1.241528	1.242439	191.1624	153.8587

ZNF830	0.973875	0.026125	0.812035	0.811318	123.3665	152.0592
CALML4	0.973901	0.026099	0.830925	0.829397	52.23625	62.98308
TIMM10	0.973902	0.026098	0.743955	0.743843	979.1518	1316.346
GOLGA6C	0.973903	0.026097	1.19915	1.201953	48.6464	40.47113
LINC0132	0.973903	0.026097	0.738241	0.717373	5.212511	7.270047
GRAP	0.973906	0.026094	1.225373	1.235057	16.67114	13.49637
RARRES1	0.973906	0.026094	1.225373	1.235057	16.67114	13.49637
REREP3	0.97391	0.02609	1.396414	1.455447	5.557048	3.814975
FAM153C	0.97391	0.02609	0.76256	0.746966	6.590659	8.826629
PUS3	0.973911	0.026089	0.776572	0.776296	353.4282	455.2777
APITD1	0.973912	0.026088	0.803882	0.803309	156.6976	195.0676
LRRC45	0.97392	0.02608	0.771499	0.771261	421.2242	546.1533
EXOG	0.973921	0.026079	0.792541	0.792124	224.5047	283.4239
NKTR	0.973921	0.026079	0.750099	0.749964	793.5464	1058.116
DLG3-AS1	0.973925	0.026075	0.730635	0.706313	4.445638	6.298308
DPY19L2P	0.973925	0.026075	0.730635	0.706313	4.445638	6.298308
ELN	0.973925	0.026075	0.730635	0.706313	4.445638	6.298308
HIST1H2B	0.973925	0.026075	0.730635	0.706313	4.445638	6.298308
LINC0126	0.973925	0.026075	0.730635	0.706313	4.445638	6.298308
VSTM4	0.973927	0.026073	1.204932	1.21106	23.4952	19.39879
SIGLEC9	0.973932	0.026068	0.803136	0.794241	10.00269	12.59662
SH3RF1	0.973933	0.026067	1.301324	1.301685	631.2806	484.9697
TADA3	0.973943	0.026057	0.73332	0.733237	1359.621	1854.276
AGAP12P	0.973946	0.026054	0.836154	0.833767	32.58653	39.0855
CHRNB1	0.973947	0.026053	1.229766	1.230873	148.1954	120.3967
GOLGA8T	0.973956	0.026044	0.777884	0.765059	7.67984	10.0413
FASTKD2	0.973966	0.026034	0.74404	0.743928	983.742	1322.366
CDKN2C	0.973967	0.026033	1.222348	1.223664	117.8094	96.27414
MRPS23	0.973967	0.026033	0.750324	0.75019	792.435	1056.316
GGT1	0.973967	0.026033	0.836556	0.834116	31.81966	38.14975
SPRY2	0.973969	0.026031	1.294263	1.29466	556.8162	430.0845
ADNP-AS1	0.973969	0.026031	1.224262	1.23503	14.44832	11.69686
CCDC155	0.973969	0.026031	1.224262	1.23503	14.44832	11.69686
DNAH3	0.973969	0.026031	1.224262	1.23503	14.44832	11.69686
KCNJ12	0.973972	0.026028	0.836694	0.834486	35.82073	42.92747
MKLN1-AS	0.973978	0.026022	0.826157	0.824991	70.06326	84.92819
PYCRL	0.973982	0.026018	0.756303	0.756146	664.6229	878.9649
KIAA1958	0.973985	0.026015	1.273119	1.273667	361.2081	283.5948
SENP7	0.97399	0.02601	0.770818	0.770589	447.8981	581.2439
TMEM256	0.973991	0.026009	0.765847	0.765646	517.9169	676.4473
LOC10192	0.973998	0.026002	0.833304	0.829511	21.15012	25.49915
CTAGE9	0.974006	0.025994	0.791902	0.780739	8.280001	10.60815
LOC10050	0.974009	0.025991	0.835801	0.833775	37.96575	45.53677
SAR1A	0.974014	0.025986	1.432389	1.432444	6466.181	4514.087
CAPN12	0.974023	0.025977	0.837446	0.834665	27.78524	33.29106
AIFM1	0.974033	0.025967	0.730822	0.730745	1482.62	2028.919
PNMA6A	0.974033	0.025967	1.194013	1.197733	35.56511	29.69202
LOC44145	0.974038	0.025962	1.34166	1.38193	6.668457	4.822705
THAP8	0.97404	0.02596	0.809276	0.808642	141.149	174.5531
ATP6V0A4	0.974046	0.025954	0.805434	0.804866	160.043	198.8466
A1BG-AS1	0.974067	0.025933	0.806685	0.8061	154.4859	191.6485
UBE2J2	0.974068	0.025932	0.75959	0.759421	627.9464	826.8779
SNCG	0.97408	0.02592	1.224125	1.23503	14.44832	11.69686
LRAT	0.974086	0.025914	0.828741	0.823581	15.55973	18.89492
COX11	0.974093	0.025907	0.766445	0.766243	515.694	673.0192
KATNB1	0.974099	0.025901	0.775353	0.775095	385.6591	497.5663

FAHD2B	0.974103	0.025897	1.235615	1.236597	168.9676	136.6373
C9orf116	0.974108	0.025892	0.838655	0.835651	25.56242	30.59178
NPIPB4	0.974108	0.025892	0.783694	0.783374	302.1589	385.7174
KCNMB3	0.974112	0.025888	1.22239	1.233144	14.51501	11.76884
FRG1HP	0.974113	0.025887	1.237535	1.238483	176.9031	142.8366
TSIX	0.974119	0.025881	1.203287	1.210424	19.62749	16.21364
SLC25A52	0.97412	0.02588	0.377166	0.010474	0	0.944746
EHBP1	0.974123	0.025877	1.356593	1.356755	1737.133	1280.356
PARTICL	0.974126	0.025874	0.803296	0.794241	10.00269	12.59662
SETMAR	0.974128	0.025872	0.814002	0.813271	117.8094	144.8611
SUGCT	0.974129	0.025871	0.83871	0.835651	25.56242	30.59178
ZNF326	0.974136	0.025864	0.777661	0.77739	362.3195	466.0748
LOC10050	0.974137	0.025863	1.191408	1.195315	33.34229	27.89251
NUDT14	0.97414	0.02586	0.800048	0.799558	185.6054	232.1376
TMEM260	0.974155	0.025845	0.772012	0.771781	442.341	573.146
PRKAG2	0.974166	0.025834	0.721602	0.721543	1956.081	2710.972
TMEM69	0.974168	0.025832	0.76547	0.765275	535.6994	700.012
SUZ12P1	0.97418	0.02582	0.833557	0.832171	58.02669	69.73127
N6AMT1	0.974182	0.025818	0.807794	0.807191	146.7061	181.7512
NAGA	0.974183	0.025817	0.737773	0.73768	1184.763	1606.069
FARP1	0.974188	0.025812	1.387226	1.387331	2929.676	2111.733
PLB1	0.974194	0.025806	1.189978	1.193985	32.23088	26.99275
MCOLN2	0.974194	0.025806	0.797747	0.786272	7.779867	9.897341
MAP3K15	0.974195	0.025805	0.828835	0.823581	15.55973	18.89492
MPDU1	0.9742	0.0258	0.772007	0.771775	431.2269	558.7499
FCGRT	0.974201	0.025799	1.31306	1.313358	784.6552	597.4395
COL1A2	0.974213	0.025787	0.840092	0.836828	23.3396	27.89251
FAM81A	0.974213	0.025787	0.840092	0.836828	23.3396	27.89251
KDM6B	0.974213	0.025787	0.755935	0.755785	711.3021	941.1472
SLC1A5	0.974219	0.025781	1.47141	1.471439	13791.48	9372.782
HMGN2P4	0.974222	0.025778	1.257303	1.275959	10.0138	7.845892
PLPP1	0.974229	0.025771	0.801209	0.800712	182.2712	227.6389
PGAP3	0.974234	0.025766	0.821171	0.820286	94.46981	115.1691
FAM133A	0.974239	0.025761	1.223929	1.23503	14.44832	11.69686
CCDC74B	0.974247	0.025753	0.731241	0.70499	4.101101	5.821436
MKRN2	0.97425	0.02575	1.362727	1.362875	1910.513	1401.823
ZNF80	0.974251	0.025749	0.841717	0.839196	30.56376	36.42222
TSPYL4	0.974254	0.025746	0.818512	0.817703	104.4725	127.7657
ARHGAP5	0.974255	0.025745	1.188451	1.192563	31.11947	26.09299
CA3	0.974255	0.025745	1.223247	1.235014	13.33691	10.7971
MIA2	0.974255	0.025745	1.223247	1.235014	13.33691	10.7971
STMND1	0.974255	0.025745	1.223247	1.235014	13.33691	10.7971
ATP5J2-P	0.974265	0.025735	1.232009	1.233014	162.0213	131.4007
SLC6A20	0.97427	0.02573	1.188375	1.192563	31.11947	26.09299
LOC10049	0.97427	0.02573	0.834086	0.829169	15.9265	19.20984
SRGAP2	0.974271	0.025729	1.341303	1.341502	1310.63	976.9846
BHMG1	0.974283	0.025717	0.731055	0.706313	4.445638	6.298308
LOC10192	0.974283	0.025717	0.731055	0.706313	4.445638	6.298308
SNORA67	0.974283	0.025717	0.731055	0.706313	4.445638	6.298308
USH2A	0.974283	0.025717	0.731055	0.706313	4.445638	6.298308
MARCH9	0.97429	0.02571	0.776182	0.775926	386.7705	498.4661
PET117	0.974291	0.025709	0.779442	0.779168	363.6532	466.7226
DYNLT1	0.974298	0.025702	1.366592	1.366731	2056.108	1504.396
CASC5	0.974306	0.025694	1.329912	1.330142	1105.853	831.3767
LOC10246	0.974319	0.025681	1.578111	1.765687	3.089719	1.745531
ZNF709	0.974333	0.025667	0.84203	0.839993	37.78793	44.98792

TAF4	0.974347	0.025653	0.771547	0.771325	460.1236	596.5398
LRP8	0.974356	0.025644	1.306401	1.306718	731.3075	559.6497
CHST7	0.974357	0.025643	1.255217	1.255893	270.0725	215.0422
COLQ	0.974366	0.025634	0.797892	0.786272	7.779867	9.897341
ICAM4	0.974366	0.025634	0.797892	0.786272	7.779867	9.897341
LOC28303	0.974366	0.025634	0.797892	0.786272	7.779867	9.897341
DYX1C1	0.974369	0.025631	0.841338	0.839427	39.68844	47.2823
SKAP2	0.974369	0.025631	1.361633	1.36178	1949.412	1431.515
COX5B	0.974372	0.025628	0.710272	0.710232	2957.461	4164.081
FOXD4L3	0.974377	0.025623	0.373615	0.010186	0	0.971739
GK3P	0.974377	0.025623	0.373615	0.010186	0	0.971739
LINC0082	0.974377	0.025623	0.373615	0.010186	0	0.971739
LOC10019	0.974377	0.025623	0.373615	0.010186	0	0.971739
TRPC6	0.974377	0.025623	0.670063	0.618675	2.222819	3.599033
USP12-AS	0.974377	0.025623	0.670063	0.618675	2.222819	3.599033
LOC10192	0.974379	0.025621	1.193021	1.195314	57.30428	47.93912
STARD3N	0.97438	0.02562	1.34909	1.349264	1564.865	1159.788
FOXL2NB	0.974381	0.025619	0.843373	0.84112	33.29783	39.58937
TXNL4A	0.97439	0.02561	0.771768	0.771542	445.6752	577.6448
ADAMTS3	0.974397	0.025603	0.841898	0.838257	21.11678	25.19323
HAVCR2	0.974399	0.025601	1.313705	1.349035	6.812941	5.047644
ACE2	0.974403	0.025597	0.843158	0.841046	35.56511	42.28864
LOC10012	0.974404	0.025596	0.753358	0.753219	762.427	1012.228
VCX3A	0.974406	0.025594	1.630046	1.854434	3.01192	1.619565
TMEM92	0.974413	0.025587	1.275457	1.275945	417.89	327.512
CCDC12	0.974421	0.025579	0.80475	0.804224	170.0457	211.4432
CDKN2B-/	0.974424	0.025576	1.186253	1.18918	43.10046	36.24226
PTPRA	0.974431	0.025569	1.364487	1.364627	2063.465	1512.107
BMS1P6	0.974434	0.025566	0.822441	0.821603	100.9493	122.871
NDUFS3	0.974437	0.025563	0.761503	0.761332	603.4954	792.6871
ULK4P1	0.974441	0.025559	0.74816	0.725158	4.512323	6.226327
ULK4P2	0.974441	0.025559	0.74816	0.725158	4.512323	6.226327
AS3MT	0.974444	0.025556	0.825432	0.824488	86.89	105.3887
GMPR2	0.974444	0.025556	0.774098	0.773859	410.1101	529.9576
ETV2	0.974447	0.025553	1.222218	1.234994	12.22551	9.897341
NPIPB6	0.974456	0.025544	0.798689	0.786802	7.579813	9.636411
ATP1A1-A	0.974458	0.025542	1.183184	1.187642	27.78524	23.39372
CASC10	0.974461	0.025539	1.196435	1.198326	72.24162	60.28381
ZNF286B	0.974462	0.025538	1.183205	1.186528	37.95464	31.98641
TMEM216	0.974466	0.025534	0.783946	0.783647	327.8658	418.3876
HGSNAT	0.974473	0.025527	1.32299	1.323241	969.1492	732.4033
NFE2L1	0.974483	0.025517	0.683372	0.683356	7607.599	11132.71
PAOX	0.97449	0.02551	0.846036	0.843615	31.11947	36.89009
MYCN	0.974491	0.025509	1.183102	1.187642	27.78524	23.39372
ADAT3	0.974508	0.025492	1.181213	1.185739	26.67383	22.49396
BCL3	0.974508	0.025492	1.306751	1.307065	727.9733	556.9504
NBEA	0.974511	0.025489	1.21235	1.213656	114.5308	94.36665
MED6	0.974511	0.025489	0.773423	0.773192	432.0049	558.7319
LOC10028	0.974511	0.025489	1.221168	1.23497	11.1141	8.997583
UQCRB	0.974516	0.025484	1.401331	1.401413	3991.072	2847.888
TTC28-AS	0.974521	0.025479	1.224466	1.225561	141.149	115.1691
RLN2	0.974523	0.025477	1.191958	1.194021	64.46176	53.9855
ZNF674-A	0.974531	0.025469	1.204108	1.205637	93.3584	77.4332
NPM2	0.974533	0.025467	1.181154	1.185739	26.67383	22.49396
SPOCK1	0.974535	0.025465	1.191232	1.193323	63.35035	53.08574
ZCCHC17	0.974537	0.025463	1.345711	1.345891	1500.403	1114.801

MTMR9LF	0.974541	0.025459	0.830665	0.823618	11.1141	13.49637
COMMD4	0.97456	0.02544	0.7666	0.766408	529.031	690.2766
LINC0120	0.974562	0.025438	1.790583	2.253696	2.222819	0.980737
L1TD1	0.974563	0.025437	0.843184	0.838729	16.80451	20.03762
PPIEL	0.974568	0.025432	0.829499	0.822554	11.49198	13.97325
CCDC148	0.974568	0.025432	0.847739	0.845204	28.89665	34.19082
GLB1	0.974569	0.025431	1.418528	1.418592	5451.464	3842.868
PRPF40B	0.974588	0.025412	0.766948	0.766755	525.6967	685.6158
ERI1	0.974593	0.025407	1.341352	1.341543	1360.365	1014.028
JOSD2	0.974598	0.025402	0.788274	0.787947	295.6349	375.1992
SHPRH	0.9746	0.0254	0.785138	0.784836	323.4202	412.0893
CERS4	0.974602	0.025398	0.747759	0.747642	920.2471	1230.869
WWC3	0.974603	0.025397	0.741387	0.741289	1112.521	1500.797
SNRPD1	0.974622	0.025378	0.73675	0.736664	1317.02	1787.82
CNTD2	0.974633	0.025367	1.19916	1.200888	77.79867	64.7826
PA2G4P4	0.974638	0.025362	0.623399	0.544212	1.444832	2.663285
SLC7A5P2	0.974639	0.025361	0.849032	0.846567	30.08586	35.54045
SCAF4	0.97464	0.02536	0.732968	0.73289	1441.498	1966.872
TOR4A	0.974646	0.025354	1.200554	1.202128	88.86831	73.92414
CRNKL1	0.974647	0.025353	0.766129	0.765941	542.3679	708.1098
MAPK15	0.974658	0.025342	1.220995	1.23497	11.1141	8.997583
PODN	0.974658	0.025342	1.220995	1.23497	11.1141	8.997583
STX19	0.974661	0.025339	1.178873	1.183678	25.56242	21.5942
GOLT1B	0.974661	0.025339	1.382289	1.382395	2891.888	2091.938
HLA-DOA	0.974665	0.025335	0.667995	0.615596	2.211705	3.599033
PDCD6	0.974669	0.025331	1.308662	1.308962	769.0732	587.5422
MRO	0.974683	0.025317	0.786075	0.769454	5.557048	7.225059
GPATCH2	0.974691	0.025309	0.791541	0.791191	273.3956	345.5522
C2orf91	0.974697	0.025303	0.615748	0.536282	1.467061	2.744263
CHD5	0.974698	0.025302	0.846692	0.842283	16.67114	19.79468
LOC10013	0.974698	0.025302	0.846692	0.842283	16.67114	19.79468
SLC35D1	0.974698	0.025302	1.307588	1.307887	780.2095	596.5398
FAM13A	0.974711	0.025289	0.803387	0.802909	187.8282	233.9372
ABHD8	0.974723	0.025277	0.751149	0.751023	844.6713	1124.698
TMEM147	0.974724	0.025276	1.211511	1.212751	120.0322	98.97341
NDUFA4L	0.974725	0.025275	1.185425	1.187682	55.57048	46.78743
GDPD5	0.974727	0.025273	1.32311	1.323353	1001.38	756.6967
ACSS3	0.974728	0.025272	0.670655	0.618675	2.222819	3.599033
ALDH1L1-	0.974728	0.025272	0.670655	0.618675	2.222819	3.599033
BIN2	0.974728	0.025272	0.670655	0.618675	2.222819	3.599033
C21orf62-/	0.974728	0.025272	0.670655	0.618675	2.222819	3.599033
DCST1	0.974728	0.025272	0.670655	0.618675	2.222819	3.599033
DNAH12	0.974728	0.025272	0.670655	0.618675	2.222819	3.599033
FOXP3	0.974728	0.025272	0.670655	0.618675	2.222819	3.599033
GGTA1P	0.974728	0.025272	0.670655	0.618675	2.222819	3.599033
GTSE1-AS	0.974728	0.025272	0.670655	0.618675	2.222819	3.599033
ITFG1-AS	0.974728	0.025272	0.670655	0.618675	2.222819	3.599033
KLHL33	0.974728	0.025272	0.670655	0.618675	2.222819	3.599033
LINC0117	0.974728	0.025272	0.670655	0.618675	2.222819	3.599033
LOC10027	0.974728	0.025272	0.670655	0.618675	2.222819	3.599033
LOC10192	0.974728	0.025272	0.670655	0.618675	2.222819	3.599033
LOC10272	0.974728	0.025272	0.670655	0.618675	2.222819	3.599033
LOC39132	0.974728	0.025272	0.670655	0.618675	2.222819	3.599033
PARM1	0.974728	0.025272	0.670655	0.618675	2.222819	3.599033
RERGL	0.974728	0.025272	0.670655	0.618675	2.222819	3.599033
RNASE10	0.974728	0.025272	0.670655	0.618675	2.222819	3.599033

ST6GALN	0.974728	0.025272	0.670655	0.618675	2.222819	3.599033
TWIST1	0.974728	0.025272	0.670655	0.618675	2.222819	3.599033
CCDC154	0.974732	0.025268	1.219709	1.234941	10.00269	8.097825
CMTM1	0.974735	0.025265	1.178058	1.183295	23.3396	19.7227
OR7E156I	0.974742	0.025258	1.770306	2.213492	2.222819	0.998732
PPCS	0.974743	0.025257	0.740665	0.74057	1154.755	1559.281
PTBP3	0.974751	0.025249	1.408663	1.408735	4538.997	3222.034
FHOD1	0.974751	0.025249	1.283754	1.284172	495.6887	385.9963
DSTN	0.974771	0.025229	1.442778	1.442821	8396.699	5819.637
TMEM121	0.97478	0.02522	1.180153	1.182625	50.01343	42.28864
GDF9	0.974787	0.025213	1.17399	1.178994	23.3396	19.79468
HIST2H2E	0.974788	0.025212	1.182416	1.184772	52.23625	44.08816
FAM133B	0.97479	0.02521	0.756018	0.755875	738.9318	977.5874
NAT1	0.974794	0.025206	0.806088	0.805594	183.3826	227.6389
ARHGAP2	0.974801	0.025199	1.287961	1.288353	539.0336	418.3876
LOC10013	0.974805	0.025195	0.846742	0.84504	43.34497	51.29522
MRPL55	0.974811	0.025189	0.77369	0.773466	446.7867	577.6448
CCDC66	0.974812	0.025188	0.788268	0.787947	295.6349	375.1992
KAZALD1	0.974814	0.025186	0.846889	0.845188	43.34497	51.28622
ZNF713	0.974823	0.025177	0.848545	0.846771	41.95571	49.54969
UVRAG	0.974823	0.025177	0.749163	0.749045	905.7988	1209.275
PRTFDC1	0.974835	0.025165	0.824308	0.823502	102.2497	124.1666
DLEU2L	0.974836	0.025164	1.180198	1.182625	50.01343	42.28864
DCLRE1C	0.974843	0.025157	0.788839	0.788515	292.3007	370.7004
C9orf173	0.974845	0.025155	0.388694	0.010992	0	0.899758
CACNA1C	0.974845	0.025155	0.388694	0.010992	0	0.899758
FAM129C	0.974845	0.025155	0.388694	0.010992	0	0.899758
GALNT13	0.974845	0.025155	0.388694	0.010992	0	0.899758
GJB4	0.974845	0.025155	0.388694	0.010992	0	0.899758
GP2	0.974845	0.025155	0.388694	0.010992	0	0.899758
GUSBP5	0.974845	0.025155	0.388694	0.010992	0	0.899758
HERC2P1	0.974845	0.025155	0.388694	0.010992	0	0.899758
HIF1A-AS	0.974845	0.025155	0.388694	0.010992	0	0.899758
HIST2H2A	0.974845	0.025155	0.388694	0.010992	0	0.899758
ITLN1	0.974845	0.025155	0.388694	0.010992	0	0.899758
KRT36	0.974845	0.025155	0.388694	0.010992	0	0.899758
LINC0116	0.974845	0.025155	0.388694	0.010992	0	0.899758
LOC1005C	0.974845	0.025155	0.388694	0.010992	0	0.899758
LOC1019	0.974845	0.025155	0.388694	0.010992	0	0.899758
LOC4403	0.974845	0.025155	0.388694	0.010992	0	0.899758
MRPL23-A	0.974845	0.025155	0.388694	0.010992	0	0.899758
NLRP6	0.974845	0.025155	0.388694	0.010992	0	0.899758
OR51B4	0.974845	0.025155	0.388694	0.010992	0	0.899758
PRSS57	0.974845	0.025155	0.388694	0.010992	0	0.899758
RASSF1-A	0.974845	0.025155	0.388694	0.010992	0	0.899758
SLA2	0.974845	0.025155	0.388694	0.010992	0	0.899758
SLC4A9	0.974845	0.025155	0.388694	0.010992	0	0.899758
SNORA80	0.974845	0.025155	0.388694	0.010992	0	0.899758
TNIP3	0.974845	0.025155	0.388694	0.010992	0	0.899758
SERHL2	0.974855	0.025145	0.840963	0.839644	57.64881	68.66056
STRIP1	0.974858	0.025142	0.769555	0.769357	521.2511	677.518
CYP1A1	0.974864	0.025136	1.281745	1.282168	485.686	378.7982
LOC64428	0.974865	0.025135	1.220747	1.23497	11.1141	8.997583
TMEM191	0.974866	0.025134	0.848791	0.847065	43.04489	50.81835
TLR5	0.974872	0.025128	0.84064	0.839347	58.90471	70.18115
LDB1	0.974879	0.025121	0.749759	0.749641	921.3585	1229.07

HOXA11	0.974882	0.025118	1.17116	1.176317	22.22819	18.89492
TRPV3	0.974882	0.025118	1.17116	1.176317	22.22819	18.89492
CCL5	0.974883	0.025117	0.837818	0.836645	65.82879	78.68386
MYT1	0.974891	0.025109	1.177787	1.180288	47.79061	40.48912
GPER1	0.974893	0.025107	1.219519	1.234941	10.00269	8.097825
LOC55420	0.974893	0.025107	1.219519	1.234941	10.00269	8.097825
TMEM39A	0.974894	0.025106	1.359596	1.359739	1990.535	1463.907
NRARP	0.974896	0.025104	0.824272	0.823501	108.9181	132.2645
PPIAL4G	0.974903	0.025097	1.169043	1.173391	25.97364	22.13405
FAM43A	0.974903	0.025097	1.211954	1.213152	122.2551	100.7729
LINC0017	0.974909	0.025091	0.853079	0.850974	34.4537	40.48912
PKIB	0.97493	0.02507	0.834532	0.827445	11.20301	13.54136
ST13P4	0.974932	0.025068	1.7715	2.236093	2.14502	0.953744
ARAP1	0.974932	0.025068	1.333113	1.33332	1223.662	917.7535
PRH1	0.974946	0.025054	0.852667	0.848599	17.82701	21.00936
HMGB3	0.974947	0.025053	0.732743	0.732668	1500.392	2047.85
LINC0066	0.974949	0.025051	1.174761	1.177311	46.80146	39.75132
SLC33A1	0.974959	0.025041	0.735636	0.735555	1375.925	1870.598
MN1	0.974966	0.025034	0.831338	0.830423	88.91277	107.0712
NOL11	0.97497	0.02503	0.7385	0.738413	1263.673	1711.34
GALNT9	0.974973	0.025027	0.789009	0.772336	5.557048	7.198066
LOC10013	0.974973	0.025027	0.789009	0.772336	5.557048	7.198066
MYH7B	0.974973	0.025027	0.789009	0.772336	5.557048	7.198066
PCP2	0.974973	0.025027	0.789009	0.772336	5.557048	7.198066
YTHDF3-A	0.974973	0.025027	0.789009	0.772336	5.557048	7.198066
PCGF1	0.974976	0.025024	0.785129	0.784836	323.4202	412.0893
DANT2	0.97498	0.02502	1.217912	1.234905	8.891277	7.198066
ANKRD7	0.974982	0.025018	1.168057	1.173373	21.11678	17.99517
BARX2	0.974982	0.025018	1.168057	1.173373	21.11678	17.99517
MAMDC4	0.974982	0.025018	1.168057	1.173373	21.11678	17.99517
RASGRF1	0.974984	0.025016	1.173746	1.176364	44.45638	37.78985
ADGRG5	0.974989	0.025011	0.85512	0.852937	32.23088	37.78985
NAT8L	0.974997	0.025003	0.838369	0.837232	67.79598	80.97825
MTMR4	0.975004	0.024996	0.738228	0.738143	1313.686	1779.722
USP32P1	0.975005	0.024995	1.167117	1.170513	32.7977	28.01847
PLSCR1	0.975006	0.024994	1.36573	1.365859	2242.825	1642.059
SMDT1	0.975017	0.024983	0.764708	0.764537	610.1639	798.0856
TPD52L2	0.975019	0.024981	1.432072	1.432122	7089.682	4950.47
NHLRC1	0.975021	0.024979	0.809639	0.809113	166.7114	206.0447
PLK2	0.975023	0.024977	1.422649	1.422707	5988.275	4209.069
OPRM1	0.975027	0.024973	0.734315	0.702824	3.367571	4.795712
LOC10050	0.975032	0.024968	0.665781	0.612566	2.222819	3.635024
ZEB1-AS1	0.975033	0.024967	0.858662	0.855222	20.00537	23.39372
MAN1A2	0.975038	0.024962	0.752927	0.7528	832.4458	1105.803
LOC64575	0.975039	0.024961	1.255952	1.281425	7.324189	5.713465
PTGES2	0.975042	0.024958	0.747974	0.747863	989.1545	1322.645
CNBD2	0.975046	0.024954	1.673133	2.022108	2.211705	1.088708
SYCE2	0.975054	0.024946	0.857557	0.855201	30.00806	35.09057
VIM-AS1	0.975054	0.024946	1.17072	1.173422	42.23356	35.99033
KANTR	0.975054	0.024946	1.169564	1.175318	20.09429	17.09541
EXTL2	0.975056	0.024944	0.788457	0.788146	305.6376	387.7958
RAB6C	0.975064	0.024936	0.710695	0.67089	2.76741	4.129891
VAT1	0.975073	0.024927	0.735103	0.735025	1415.936	1926.383
PCDHA12	0.975085	0.024915	1.250119	1.272399	8.280001	6.505253
ILK	0.975091	0.024909	1.363622	1.363754	2181.697	1599.77
ATP1B1	0.975092	0.024908	1.394556	1.394641	3753.23	2691.177

MYO1D	0.975099	0.024901	1.379741	1.379847	2820.758	2044.251
RPS10-NU	0.9751	0.0249	1.234883	1.235662	216.247	175.003
LOC10192	0.975103	0.024897	1.260762	1.288224	6.957424	5.39855
MAGEA2	0.975106	0.024894	1.167327	1.17298	20.13874	17.16739
MFNG	0.975107	0.024893	1.16733	1.170169	40.01074	34.19082
EVC	0.975107	0.024893	1.259705	1.26026	335.6457	266.3285
TBP	0.975107	0.024893	0.772444	0.772239	496.8001	643.3272
SLC6A6	0.975107	0.024893	1.308325	1.308606	832.4458	636.1291
NBPF1	0.975113	0.024887	0.777565	0.77733	417.8455	537.5426
SPAG17	0.97512	0.02488	1.219247	1.234941	10.00269	8.097825
CENPK	0.97512	0.02488	1.292168	1.292518	626.835	484.9697
LOC10192	0.975126	0.024874	0.842951	0.835678	10.26942	12.2907
LOC10049	0.975128	0.024872	1.163922	1.169648	19.06067	16.29462
SLC36A2	0.975128	0.024872	1.776011	2.271051	2.056108	0.899758
CDH15	0.975139	0.024861	0.860299	0.857843	27.78524	32.3913
ZNF436	0.975146	0.024854	0.79016	0.789841	295.6349	374.2995
LOC10050	0.975156	0.024844	1.217701	1.234905	8.891277	7.198066
LOC10537	0.975156	0.024844	1.217701	1.234905	8.891277	7.198066
RHBDF1	0.975171	0.024829	1.273573	1.274026	437.8954	343.7077
AZIN1-AS	0.975173	0.024827	1.165579	1.168411	38.89934	33.29106
REPIN1	0.975185	0.024815	0.712379	0.712339	2996.36	4206.37
OTUD7B	0.975188	0.024812	0.754536	0.754405	809.1062	1072.512
CFAP69	0.975191	0.024809	1.165616	1.168411	38.89934	33.29106
CXCL17	0.9752	0.0248	1.160851	1.166504	18.89396	16.19565
TMEM246	0.975205	0.024795	0.844814	0.843594	62.23894	73.78018
ALG1L9P	0.975211	0.024789	0.6715	0.618675	2.222819	3.599033
CNGA4	0.975211	0.024789	0.6715	0.618675	2.222819	3.599033
FAM215A	0.975211	0.024789	0.6715	0.618675	2.222819	3.599033
HOTTIP	0.975211	0.024789	0.6715	0.618675	2.222819	3.599033
LOC10029	0.975211	0.024789	0.6715	0.618675	2.222819	3.599033
LOC10050	0.975211	0.024789	0.6715	0.618675	2.222819	3.599033
LOC72986	0.975211	0.024789	0.6715	0.618675	2.222819	3.599033
PANO1	0.975211	0.024789	0.6715	0.618675	2.222819	3.599033
PCDHGA1	0.975211	0.024789	0.6715	0.618675	2.222819	3.599033
BECN1	0.975213	0.024787	1.328574	1.328786	1168.091	879.0639
GATA6	0.975215	0.024785	1.252596	1.253188	310.0833	247.4335
ABHD15	0.975223	0.024777	0.805443	0.804987	195.6081	242.9977
RNF222	0.975223	0.024777	1.231543	1.2526	7.835438	6.25332
SH2D3A	0.975228	0.024772	1.341282	1.341462	1431.496	1067.113
MAPK14	0.975229	0.024771	1.404225	1.404299	4403.405	3135.658
ASCL2	0.975234	0.024766	0.855057	0.849326	12.22551	14.39613
CDK5RAP	0.975237	0.024763	0.752213	0.752091	870.2337	1157.089
BDNF	0.975249	0.024751	1.276975	1.277402	471.2377	368.9009
LRRC57	0.975264	0.024736	1.270507	1.270974	420.0128	330.4632
CCDC15	0.975265	0.024735	0.84646	0.845181	57.7933	68.38163
LINC0153	0.975276	0.024724	1.197706	1.211612	9.802632	8.088827
TMEM9B-	0.975277	0.024723	0.7893	0.772336	5.557048	7.198066
ZSWIM8-A	0.975277	0.024723	0.7893	0.772336	5.557048	7.198066
PRDM10	0.97528	0.02472	1.252928	1.253521	304.5262	242.9347
LYPLAL1	0.97529	0.02471	0.832638	0.823651	8.891277	10.7971
EXOSC7	0.975294	0.024706	1.304392	1.304684	772.4297	592.041
ZNF487	0.975297	0.024703	0.863194	0.859359	17.78255	20.69444
HIST1H2B	0.975298	0.024702	1.160748	1.166504	18.89396	16.19565
BSDC1	0.975307	0.024693	1.359584	1.359721	2027.211	1490.9
DUSP5	0.975307	0.024693	1.347603	1.347764	1690.454	1254.263
ADCY5	0.975319	0.024681	1.156635	1.162464	17.78255	15.29589

COLEC12	0.975319	0.024681	1.156635	1.162464	17.78255	15.29589
WBP1	0.975327	0.024673	0.771625	0.771428	507.8142	658.2812
KAT7	0.975338	0.024662	0.758817	0.758675	750.2015	988.8344
MRPS9	0.975338	0.024662	0.748125	0.748017	991.3773	1325.344
ECHDC3	0.975341	0.024659	0.830607	0.829788	97.80404	117.8683
TRAPPC1	0.975342	0.024658	0.782274	0.782021	391.2162	500.2656
FAM231D	0.975349	0.024651	1.157542	1.160609	34.59818	29.80899
AGPAT4	0.975354	0.024646	1.268875	1.269345	413.4444	325.7125
CCDC144	0.975356	0.024644	0.773177	0.749983	4.167786	5.560506
EXOC7	0.97536	0.02464	0.723285	0.723231	2100.564	2904.42
FAM35BP	0.975363	0.024637	0.840093	0.830204	7.535357	9.078561
STARD9	0.975388	0.024612	1.185524	1.187078	82.24431	69.28139
LOC65365	0.975389	0.024611	1.169606	1.171704	54.97032	46.9134
TMOD2	0.975391	0.024609	0.867559	0.864712	23.3396	26.99275
HS3ST3B1	0.975393	0.024607	0.83404	0.822808	6.824055	8.295772
CCDC59	0.975399	0.024601	0.765416	0.76525	627.9464	820.5796
POLN	0.975399	0.024601	1.198833	1.214033	9.03576	7.441001
FBXO48	0.975403	0.024597	0.838937	0.837949	77.79867	92.84606
MCF2L	0.975404	0.024596	0.806417	0.805974	204.4994	253.7318
LRRC69	0.975404	0.024596	1.217401	1.234905	8.891277	7.198066
TNFRSF8	0.975404	0.024596	1.217401	1.234905	8.891277	7.198066
C1orf229	0.975407	0.024593	1.164282	1.172622	13.14798	11.21099
PSG1	0.975425	0.024575	1.149165	1.153527	22.46159	19.47077
TMEM221	0.975427	0.024573	1.156529	1.162464	17.78255	15.29589
GUCY1A3	0.97543	0.02457	0.868885	0.864737	15.55973	17.99517
IL17D	0.97543	0.02457	0.868885	0.864737	15.55973	17.99517
VIPR1	0.97543	0.02457	0.868885	0.864737	15.55973	17.99517
LOC10192	0.975434	0.024566	0.853571	0.846441	9.891545	11.68786
LOC10192	0.975445	0.024555	0.812923	0.798286	5.801558	7.270047
RAMP2-AS	0.97545	0.02455	1.159145	1.161759	39.74401	34.20881
CACNG6	0.975452	0.024548	1.217104	1.218058	157.8202	129.5652
ANAPC7	0.975453	0.024547	0.748809	0.748702	1012.494	1352.337
KIF2C	0.975455	0.024545	1.377672	1.377777	2825.203	2050.549
CDX2	0.975456	0.024544	1.215408	1.234858	7.779867	6.298308
CNIH3	0.975456	0.024544	1.215408	1.234858	7.779867	6.298308
CYP2D7	0.975456	0.024544	1.215408	1.234858	7.779867	6.298308
FAM179A	0.975456	0.024544	1.215408	1.234858	7.779867	6.298308
GPR75	0.975456	0.024544	1.215408	1.234858	7.779867	6.298308
HOXA6	0.975456	0.024544	1.215408	1.234858	7.779867	6.298308
LIPE-AS1	0.975456	0.024544	1.215408	1.234858	7.779867	6.298308
LTC4S	0.975456	0.024544	1.215408	1.234858	7.779867	6.298308
PLCD4	0.975456	0.024544	1.215408	1.234858	7.779867	6.298308
SEC24B-A	0.975456	0.024544	1.215408	1.234858	7.779867	6.298308
VNN3	0.975456	0.024544	1.215408	1.234858	7.779867	6.298308
CYP4F30P	0.975458	0.024542	0.380117	0.01028	0	0.962741
JPX	0.975461	0.024539	0.832885	0.83205	94.83658	113.9814
KLHDC8B	0.975475	0.024525	1.258677	1.259207	350.094	278.0253
GOLGA6L	0.975476	0.024524	1.150248	1.153571	29.69686	25.74209
SP4	0.975482	0.024518	0.799516	0.799144	250.2228	313.1159
TFAM	0.975486	0.024514	0.728722	0.728659	1764.918	2422.149
CDH23	0.975487	0.024513	1.15736	1.164993	13.67034	11.73285
RGS9BP	0.975496	0.024504	0.866807	0.864699	31.11947	35.99033
CSF2RA	0.975497	0.024503	1.212236	1.231346	7.779867	6.316303
LAMTOR4	0.975498	0.024502	0.732096	0.732027	1624.881	2219.704
ZNF568	0.975504	0.024496	1.191221	1.192612	93.3584	78.27897
AIFM3	0.97551	0.02449	1.192203	1.19357	95.58122	80.07849

MED15P9	0.975513	0.024487	0.605445	0.504366	1.11141	2.213405
ADAM20	0.975515	0.024485	0.391794	0.010992	0	0.899758
ADAMTS1	0.975515	0.024485	0.391794	0.010992	0	0.899758
AFF3	0.975515	0.024485	0.391794	0.010992	0	0.899758
ANGPT2	0.975515	0.024485	0.391794	0.010992	0	0.899758
ANKK1	0.975515	0.024485	0.391794	0.010992	0	0.899758
ANKRD66	0.975515	0.024485	0.391794	0.010992	0	0.899758
ANKUB1	0.975515	0.024485	0.391794	0.010992	0	0.899758
ANO1-AS2	0.975515	0.024485	0.391794	0.010992	0	0.899758
APOC4	0.975515	0.024485	0.391794	0.010992	0	0.899758
ARHGAP1	0.975515	0.024485	0.391794	0.010992	0	0.899758
ARHGAP3	0.975515	0.024485	0.391794	0.010992	0	0.899758
ARL11	0.975515	0.024485	0.391794	0.010992	0	0.899758
ARSF	0.975515	0.024485	0.391794	0.010992	0	0.899758
ASXL3	0.975515	0.024485	0.391794	0.010992	0	0.899758
ASZ1	0.975515	0.024485	0.391794	0.010992	0	0.899758
ATP4A	0.975515	0.024485	0.391794	0.010992	0	0.899758
BOLL	0.975515	0.024485	0.391794	0.010992	0	0.899758
BSND	0.975515	0.024485	0.391794	0.010992	0	0.899758
C16orf54	0.975515	0.024485	0.391794	0.010992	0	0.899758
C1QTNF9	0.975515	0.024485	0.391794	0.010992	0	0.899758
C1orf234	0.975515	0.024485	0.391794	0.010992	0	0.899758
C2-AS1	0.975515	0.024485	0.391794	0.010992	0	0.899758
C7	0.975515	0.024485	0.391794	0.010992	0	0.899758
C9orf106	0.975515	0.024485	0.391794	0.010992	0	0.899758
CA5A	0.975515	0.024485	0.391794	0.010992	0	0.899758
CACNA1C	0.975515	0.024485	0.391794	0.010992	0	0.899758
CACNA1G	0.975515	0.024485	0.391794	0.010992	0	0.899758
CADPS	0.975515	0.024485	0.391794	0.010992	0	0.899758
CAMK2A	0.975515	0.024485	0.391794	0.010992	0	0.899758
CAMP	0.975515	0.024485	0.391794	0.010992	0	0.899758
CAPN11	0.975515	0.024485	0.391794	0.010992	0	0.899758
CASC21	0.975515	0.024485	0.391794	0.010992	0	0.899758
CCDC63	0.975515	0.024485	0.391794	0.010992	0	0.899758
CCDC83	0.975515	0.024485	0.391794	0.010992	0	0.899758
CCDC85A	0.975515	0.024485	0.391794	0.010992	0	0.899758
CCEPR	0.975515	0.024485	0.391794	0.010992	0	0.899758
CCL17	0.975515	0.024485	0.391794	0.010992	0	0.899758
CCL18	0.975515	0.024485	0.391794	0.010992	0	0.899758
CCRL2	0.975515	0.024485	0.391794	0.010992	0	0.899758
CDC20B	0.975515	0.024485	0.391794	0.010992	0	0.899758
CDH12	0.975515	0.024485	0.391794	0.010992	0	0.899758
CELA3A	0.975515	0.024485	0.391794	0.010992	0	0.899758
CELA3B	0.975515	0.024485	0.391794	0.010992	0	0.899758
CFHR5	0.975515	0.024485	0.391794	0.010992	0	0.899758
CFL1P1	0.975515	0.024485	0.391794	0.010992	0	0.899758
CHRNA1	0.975515	0.024485	0.391794	0.010992	0	0.899758
CLCA2	0.975515	0.024485	0.391794	0.010992	0	0.899758
CLLU1	0.975515	0.024485	0.391794	0.010992	0	0.899758
CMTM2	0.975515	0.024485	0.391794	0.010992	0	0.899758
COL18A1-	0.975515	0.024485	0.391794	0.010992	0	0.899758
COLEC11	0.975515	0.024485	0.391794	0.010992	0	0.899758
CPA3	0.975515	0.024485	0.391794	0.010992	0	0.899758
CPLX4	0.975515	0.024485	0.391794	0.010992	0	0.899758
CTAGE11	0.975515	0.024485	0.391794	0.010992	0	0.899758
CTD-2151	0.975515	0.024485	0.391794	0.010992	0	0.899758

CTRB1	0.975515	0.024485	0.391794	0.010992	0	0.899758
CTRC	0.975515	0.024485	0.391794	0.010992	0	0.899758
CXCL14	0.975515	0.024485	0.391794	0.010992	0	0.899758
CXCR3	0.975515	0.024485	0.391794	0.010992	0	0.899758
CYP19A1	0.975515	0.024485	0.391794	0.010992	0	0.899758
CYP4F22	0.975515	0.024485	0.391794	0.010992	0	0.899758
CYYR1	0.975515	0.024485	0.391794	0.010992	0	0.899758
DCAF4L1	0.975515	0.024485	0.391794	0.010992	0	0.899758
DENND5E	0.975515	0.024485	0.391794	0.010992	0	0.899758
DISC1FP1	0.975515	0.024485	0.391794	0.010992	0	0.899758
DNTT	0.975515	0.024485	0.391794	0.010992	0	0.899758
DSC1	0.975515	0.024485	0.391794	0.010992	0	0.899758
EBLN1	0.975515	0.024485	0.391794	0.010992	0	0.899758
EGFLAM	0.975515	0.024485	0.391794	0.010992	0	0.899758
EIF1AY	0.975515	0.024485	0.391794	0.010992	0	0.899758
ENAM	0.975515	0.024485	0.391794	0.010992	0	0.899758
ENHO	0.975515	0.024485	0.391794	0.010992	0	0.899758
ENPP7P1	0.975515	0.024485	0.391794	0.010992	0	0.899758
EPPIN	0.975515	0.024485	0.391794	0.010992	0	0.899758
EXTL1	0.975515	0.024485	0.391794	0.010992	0	0.899758
F10	0.975515	0.024485	0.391794	0.010992	0	0.899758
FAM183A	0.975515	0.024485	0.391794	0.010992	0	0.899758
FAM222A-	0.975515	0.024485	0.391794	0.010992	0	0.899758
FAM71D	0.975515	0.024485	0.391794	0.010992	0	0.899758
FAM95A	0.975515	0.024485	0.391794	0.010992	0	0.899758
FBXO15	0.975515	0.024485	0.391794	0.010992	0	0.899758
FBXO40	0.975515	0.024485	0.391794	0.010992	0	0.899758
FCER1G	0.975515	0.024485	0.391794	0.010992	0	0.899758
FCRL6	0.975515	0.024485	0.391794	0.010992	0	0.899758
FENDRR	0.975515	0.024485	0.391794	0.010992	0	0.899758
FKSG29	0.975515	0.024485	0.391794	0.010992	0	0.899758
FLJ13224	0.975515	0.024485	0.391794	0.010992	0	0.899758
FMR1NB	0.975515	0.024485	0.391794	0.010992	0	0.899758
FOXCUT	0.975515	0.024485	0.391794	0.010992	0	0.899758
FPR3	0.975515	0.024485	0.391794	0.010992	0	0.899758
FREM3	0.975515	0.024485	0.391794	0.010992	0	0.899758
FRMPD2E	0.975515	0.024485	0.391794	0.010992	0	0.899758
FRMPD3	0.975515	0.024485	0.391794	0.010992	0	0.899758
FXYD2	0.975515	0.024485	0.391794	0.010992	0	0.899758
GALP	0.975515	0.024485	0.391794	0.010992	0	0.899758
GFAP	0.975515	0.024485	0.391794	0.010992	0	0.899758
GFRAL	0.975515	0.024485	0.391794	0.010992	0	0.899758
GHRL	0.975515	0.024485	0.391794	0.010992	0	0.899758
GIPC2	0.975515	0.024485	0.391794	0.010992	0	0.899758
GLRA2	0.975515	0.024485	0.391794	0.010992	0	0.899758
GNB3	0.975515	0.024485	0.391794	0.010992	0	0.899758
GPR141	0.975515	0.024485	0.391794	0.010992	0	0.899758
GPR142	0.975515	0.024485	0.391794	0.010992	0	0.899758
GPR4	0.975515	0.024485	0.391794	0.010992	0	0.899758
GS1-24F4	0.975515	0.024485	0.391794	0.010992	0	0.899758
GUCY2EF	0.975515	0.024485	0.391794	0.010992	0	0.899758
HFM1	0.975515	0.024485	0.391794	0.010992	0	0.899758
HHATL	0.975515	0.024485	0.391794	0.010992	0	0.899758
HIST1H2E	0.975515	0.024485	0.391794	0.010992	0	0.899758
HIST1H4E	0.975515	0.024485	0.391794	0.010992	0	0.899758
HIST1H4L	0.975515	0.024485	0.391794	0.010992	0	0.899758

HM13-AS1	0.975515	0.024485	0.391794	0.010992	0	0.899758
HNRNPCL	0.975515	0.024485	0.391794	0.010992	0	0.899758
HOXD8	0.975515	0.024485	0.391794	0.010992	0	0.899758
HRC	0.975515	0.024485	0.391794	0.010992	0	0.899758
HS6ST3	0.975515	0.024485	0.391794	0.010992	0	0.899758
HSP90AB	0.975515	0.024485	0.391794	0.010992	0	0.899758
HTT-AS	0.975515	0.024485	0.391794	0.010992	0	0.899758
IFNL4	0.975515	0.024485	0.391794	0.010992	0	0.899758
IGFALS	0.975515	0.024485	0.391794	0.010992	0	0.899758
IL17REL	0.975515	0.024485	0.391794	0.010992	0	0.899758
IMPG2	0.975515	0.024485	0.391794	0.010992	0	0.899758
INPP5D	0.975515	0.024485	0.391794	0.010992	0	0.899758
ISX	0.975515	0.024485	0.391794	0.010992	0	0.899758
ITPRIPL1	0.975515	0.024485	0.391794	0.010992	0	0.899758
KCNA4	0.975515	0.024485	0.391794	0.010992	0	0.899758
KCNB1	0.975515	0.024485	0.391794	0.010992	0	0.899758
KCNB2	0.975515	0.024485	0.391794	0.010992	0	0.899758
KCNC1	0.975515	0.024485	0.391794	0.010992	0	0.899758
KCNF1	0.975515	0.024485	0.391794	0.010992	0	0.899758
KCNJ3	0.975515	0.024485	0.391794	0.010992	0	0.899758
KHDC1L	0.975515	0.024485	0.391794	0.010992	0	0.899758
KIAA0408	0.975515	0.024485	0.391794	0.010992	0	0.899758
KPRP	0.975515	0.024485	0.391794	0.010992	0	0.899758
KRTAP2-2	0.975515	0.024485	0.391794	0.010992	0	0.899758
LACTB2-A	0.975515	0.024485	0.391794	0.010992	0	0.899758
LINC0027	0.975515	0.024485	0.391794	0.010992	0	0.899758
LINC0037	0.975515	0.024485	0.391794	0.010992	0	0.899758
LINC0042	0.975515	0.024485	0.391794	0.010992	0	0.899758
LINC0047	0.975515	0.024485	0.391794	0.010992	0	0.899758
LINC0050	0.975515	0.024485	0.391794	0.010992	0	0.899758
LINC0054	0.975515	0.024485	0.391794	0.010992	0	0.899758
LINC0060	0.975515	0.024485	0.391794	0.010992	0	0.899758
LINC0063	0.975515	0.024485	0.391794	0.010992	0	0.899758
LINC0089	0.975515	0.024485	0.391794	0.010992	0	0.899758
LINC0101	0.975515	0.024485	0.391794	0.010992	0	0.899758
LINC0102	0.975515	0.024485	0.391794	0.010992	0	0.899758
LINC0103	0.975515	0.024485	0.391794	0.010992	0	0.899758
LINC0105	0.975515	0.024485	0.391794	0.010992	0	0.899758
LINC0134	0.975515	0.024485	0.391794	0.010992	0	0.899758
LINC0135	0.975515	0.024485	0.391794	0.010992	0	0.899758
LINC0160	0.975515	0.024485	0.391794	0.010992	0	0.899758
LMNTD1	0.975515	0.024485	0.391794	0.010992	0	0.899758
LMO3	0.975515	0.024485	0.391794	0.010992	0	0.899758
LMOD3	0.975515	0.024485	0.391794	0.010992	0	0.899758
LOC10012	0.975515	0.024485	0.391794	0.010992	0	0.899758
LOC10012	0.975515	0.024485	0.391794	0.010992	0	0.899758
LOC10012	0.975515	0.024485	0.391794	0.010992	0	0.899758
LOC10012	0.975515	0.024485	0.391794	0.010992	0	0.899758
LOC10013	0.975515	0.024485	0.391794	0.010992	0	0.899758
LOC10013	0.975515	0.024485	0.391794	0.010992	0	0.899758
LOC10050	0.975515	0.024485	0.391794	0.010992	0	0.899758
LOC10050	0.975515	0.024485	0.391794	0.010992	0	0.899758
LOC10050	0.975515	0.024485	0.391794	0.010992	0	0.899758
LOC10099	0.975515	0.024485	0.391794	0.010992	0	0.899758
LOC10099	0.975515	0.024485	0.391794	0.010992	0	0.899758
LOC10124	0.975515	0.024485	0.391794	0.010992	0	0.899758

LOC10192	0.975515	0.024485	0.391794	0.010992	0	0.899758
LOC10192	0.975515	0.024485	0.391794	0.010992	0	0.899758
LOC10192	0.975515	0.024485	0.391794	0.010992	0	0.899758
LOC10192	0.975515	0.024485	0.391794	0.010992	0	0.899758
LOC10192	0.975515	0.024485	0.391794	0.010992	0	0.899758
LOC10192	0.975515	0.024485	0.391794	0.010992	0	0.899758
LOC10192	0.975515	0.024485	0.391794	0.010992	0	0.899758
LOC10192	0.975515	0.024485	0.391794	0.010992	0	0.899758
LOC10192	0.975515	0.024485	0.391794	0.010992	0	0.899758
LOC10192	0.975515	0.024485	0.391794	0.010992	0	0.899758
LOC10192	0.975515	0.024485	0.391794	0.010992	0	0.899758
LOC10192	0.975515	0.024485	0.391794	0.010992	0	0.899758
LOC10192	0.975515	0.024485	0.391794	0.010992	0	0.899758
LOC10192	0.975515	0.024485	0.391794	0.010992	0	0.899758
LOC10192	0.975515	0.024485	0.391794	0.010992	0	0.899758
LOC10192	0.975515	0.024485	0.391794	0.010992	0	0.899758
LOC10192	0.975515	0.024485	0.391794	0.010992	0	0.899758
LOC10192	0.975515	0.024485	0.391794	0.010992	0	0.899758
LOC10192	0.975515	0.024485	0.391794	0.010992	0	0.899758
LOC10192	0.975515	0.024485	0.391794	0.010992	0	0.899758
LOC10272	0.975515	0.024485	0.391794	0.010992	0	0.899758
LOC10272	0.975515	0.024485	0.391794	0.010992	0	0.899758
LOC10272	0.975515	0.024485	0.391794	0.010992	0	0.899758
LOC10272	0.975515	0.024485	0.391794	0.010992	0	0.899758
LOC10272	0.975515	0.024485	0.391794	0.010992	0	0.899758
LOC10272	0.975515	0.024485	0.391794	0.010992	0	0.899758
LOC10537	0.975515	0.024485	0.391794	0.010992	0	0.899758
LOC10537	0.975515	0.024485	0.391794	0.010992	0	0.899758
LOC10544	0.975515	0.024485	0.391794	0.010992	0	0.899758
LOC11511	0.975515	0.024485	0.391794	0.010992	0	0.899758
LOC28344	0.975515	0.024485	0.391794	0.010992	0	0.899758
LOC33953	0.975515	0.024485	0.391794	0.010992	0	0.899758
LOC34009	0.975515	0.024485	0.391794	0.010992	0	0.899758
LOC34105	0.975515	0.024485	0.391794	0.010992	0	0.899758
LOC34496	0.975515	0.024485	0.391794	0.010992	0	0.899758
LOC38903	0.975515	0.024485	0.391794	0.010992	0	0.899758
LOC40070	0.975515	0.024485	0.391794	0.010992	0	0.899758
LOC40113	0.975515	0.024485	0.391794	0.010992	0	0.899758
LOC40135	0.975515	0.024485	0.391794	0.010992	0	0.899758
LOC41430	0.975515	0.024485	0.391794	0.010992	0	0.899758
LOC64690	0.975515	0.024485	0.391794	0.010992	0	0.899758
LOC72908	0.975515	0.024485	0.391794	0.010992	0	0.899758
LOC72995	0.975515	0.024485	0.391794	0.010992	0	0.899758
LRRC16B	0.975515	0.024485	0.391794	0.010992	0	0.899758
LRRC2-AS	0.975515	0.024485	0.391794	0.010992	0	0.899758
LRRC25	0.975515	0.024485	0.391794	0.010992	0	0.899758
LRRC3-AS	0.975515	0.024485	0.391794	0.010992	0	0.899758
LVCAT8	0.975515	0.024485	0.391794	0.010992	0	0.899758
LY86	0.975515	0.024485	0.391794	0.010992	0	0.899758
MALRD1	0.975515	0.024485	0.391794	0.010992	0	0.899758
MAMDC2-	0.975515	0.024485	0.391794	0.010992	0	0.899758
MANSC4	0.975515	0.024485	0.391794	0.010992	0	0.899758
MARCH10	0.975515	0.024485	0.391794	0.010992	0	0.899758
MDC1-AS	0.975515	0.024485	0.391794	0.010992	0	0.899758
MIR3681H	0.975515	0.024485	0.391794	0.010992	0	0.899758
MIXL1	0.975515	0.024485	0.391794	0.010992	0	0.899758

MKNK1-AS	0.975515	0.024485	0.391794	0.010992	0	0.899758
MLIP	0.975515	0.024485	0.391794	0.010992	0	0.899758
MOG	0.975515	0.024485	0.391794	0.010992	0	0.899758
MORC1	0.975515	0.024485	0.391794	0.010992	0	0.899758
MPC1L	0.975515	0.024485	0.391794	0.010992	0	0.899758
MSTN	0.975515	0.024485	0.391794	0.010992	0	0.899758
MTNR1A	0.975515	0.024485	0.391794	0.010992	0	0.899758
MUC19	0.975515	0.024485	0.391794	0.010992	0	0.899758
MUC21	0.975515	0.024485	0.391794	0.010992	0	0.899758
MYBPC1	0.975515	0.024485	0.391794	0.010992	0	0.899758
MZB1	0.975515	0.024485	0.391794	0.010992	0	0.899758
NANOGNB	0.975515	0.024485	0.391794	0.010992	0	0.899758
NLRP14	0.975515	0.024485	0.391794	0.010992	0	0.899758
NPB	0.975515	0.024485	0.391794	0.010992	0	0.899758
NPW	0.975515	0.024485	0.391794	0.010992	0	0.899758
NRAP	0.975515	0.024485	0.391794	0.010992	0	0.899758
NTSR2	0.975515	0.024485	0.391794	0.010992	0	0.899758
OOEP	0.975515	0.024485	0.391794	0.010992	0	0.899758
OOSP1	0.975515	0.024485	0.391794	0.010992	0	0.899758
OPALIN	0.975515	0.024485	0.391794	0.010992	0	0.899758
OR13C9	0.975515	0.024485	0.391794	0.010992	0	0.899758
OR1F2P	0.975515	0.024485	0.391794	0.010992	0	0.899758
OR1L3	0.975515	0.024485	0.391794	0.010992	0	0.899758
OR1N1	0.975515	0.024485	0.391794	0.010992	0	0.899758
OR7E24	0.975515	0.024485	0.391794	0.010992	0	0.899758
OR7E5P	0.975515	0.024485	0.391794	0.010992	0	0.899758
OVCH2	0.975515	0.024485	0.391794	0.010992	0	0.899758
PACERR	0.975515	0.024485	0.391794	0.010992	0	0.899758
PAH	0.975515	0.024485	0.391794	0.010992	0	0.899758
PAX5	0.975515	0.024485	0.391794	0.010992	0	0.899758
PCDH11X	0.975515	0.024485	0.391794	0.010992	0	0.899758
PCDH15	0.975515	0.024485	0.391794	0.010992	0	0.899758
PCDH8	0.975515	0.024485	0.391794	0.010992	0	0.899758
PEBP4	0.975515	0.024485	0.391794	0.010992	0	0.899758
PEG3	0.975515	0.024485	0.391794	0.010992	0	0.899758
PEX5L	0.975515	0.024485	0.391794	0.010992	0	0.899758
PF4V1	0.975515	0.024485	0.391794	0.010992	0	0.899758
PGA4	0.975515	0.024485	0.391794	0.010992	0	0.899758
PLBD1-AS	0.975515	0.024485	0.391794	0.010992	0	0.899758
PLCL2	0.975515	0.024485	0.391794	0.010992	0	0.899758
PNOC	0.975515	0.024485	0.391794	0.010992	0	0.899758
POM121L4	0.975515	0.024485	0.391794	0.010992	0	0.899758
PON1	0.975515	0.024485	0.391794	0.010992	0	0.899758
PRAMEF1	0.975515	0.024485	0.391794	0.010992	0	0.899758
PRKG2	0.975515	0.024485	0.391794	0.010992	0	0.899758
PRPH2	0.975515	0.024485	0.391794	0.010992	0	0.899758
PSAPL1	0.975515	0.024485	0.391794	0.010992	0	0.899758
PTCHD1	0.975515	0.024485	0.391794	0.010992	0	0.899758
PTGER4P	0.975515	0.024485	0.391794	0.010992	0	0.899758
PTGFR	0.975515	0.024485	0.391794	0.010992	0	0.899758
PYY2	0.975515	0.024485	0.391794	0.010992	0	0.899758
RAPSN	0.975515	0.024485	0.391794	0.010992	0	0.899758
RAX	0.975515	0.024485	0.391794	0.010992	0	0.899758
RBFOX3	0.975515	0.024485	0.391794	0.010992	0	0.899758
RBM12B-A	0.975515	0.024485	0.391794	0.010992	0	0.899758
RBP3	0.975515	0.024485	0.391794	0.010992	0	0.899758

RGN	0.975515	0.024485	0.391794	0.010992	0	0.899758
RGSL1	0.975515	0.024485	0.391794	0.010992	0	0.899758
RHBDL3	0.975515	0.024485	0.391794	0.010992	0	0.899758
RHOXF1P	0.975515	0.024485	0.391794	0.010992	0	0.899758
RIMBP2	0.975515	0.024485	0.391794	0.010992	0	0.899758
RPL13AP6	0.975515	0.024485	0.391794	0.010992	0	0.899758
RPS15AP	0.975515	0.024485	0.391794	0.010992	0	0.899758
RUNX1T1	0.975515	0.024485	0.391794	0.010992	0	0.899758
SATL1	0.975515	0.024485	0.391794	0.010992	0	0.899758
SEMA6A-A	0.975515	0.024485	0.391794	0.010992	0	0.899758
SFRP2	0.975515	0.024485	0.391794	0.010992	0	0.899758
SFRP5	0.975515	0.024485	0.391794	0.010992	0	0.899758
SLC16A8	0.975515	0.024485	0.391794	0.010992	0	0.899758
SND1-IT1	0.975515	0.024485	0.391794	0.010992	0	0.899758
SNORA26	0.975515	0.024485	0.391794	0.010992	0	0.899758
SNORA57	0.975515	0.024485	0.391794	0.010992	0	0.899758
SPON2	0.975515	0.024485	0.391794	0.010992	0	0.899758
TBX10	0.975515	0.024485	0.391794	0.010992	0	0.899758
TLR8-AS1	0.975515	0.024485	0.391794	0.010992	0	0.899758
TRDN	0.975515	0.024485	0.391794	0.010992	0	0.899758
TREH	0.975515	0.024485	0.391794	0.010992	0	0.899758
TRIM43	0.975515	0.024485	0.391794	0.010992	0	0.899758
TRIM51	0.975515	0.024485	0.391794	0.010992	0	0.899758
TSSK2	0.975515	0.024485	0.391794	0.010992	0	0.899758
TTC29	0.975515	0.024485	0.391794	0.010992	0	0.899758
TXLNGY	0.975515	0.024485	0.391794	0.010992	0	0.899758
UCHL1-AS	0.975515	0.024485	0.391794	0.010992	0	0.899758
UGT2B7	0.975515	0.024485	0.391794	0.010992	0	0.899758
UGT3A1	0.975515	0.024485	0.391794	0.010992	0	0.899758
UNC45B	0.975515	0.024485	0.391794	0.010992	0	0.899758
UNC80	0.975515	0.024485	0.391794	0.010992	0	0.899758
USP17L7	0.975515	0.024485	0.391794	0.010992	0	0.899758
VHLL	0.975515	0.024485	0.391794	0.010992	0	0.899758
VIT	0.975515	0.024485	0.391794	0.010992	0	0.899758
WBP11P1	0.975515	0.024485	0.391794	0.010992	0	0.899758
WDR38	0.975515	0.024485	0.391794	0.010992	0	0.899758
XAGE2	0.975515	0.024485	0.391794	0.010992	0	0.899758
XIRP2-AS	0.975515	0.024485	0.391794	0.010992	0	0.899758
ZACN	0.975515	0.024485	0.391794	0.010992	0	0.899758
ZBBX	0.975515	0.024485	0.391794	0.010992	0	0.899758
ZBTB20-A	0.975515	0.024485	0.391794	0.010992	0	0.899758
ZDHHC22	0.975515	0.024485	0.391794	0.010992	0	0.899758
ZFP92	0.975515	0.024485	0.391794	0.010992	0	0.899758
ZIC4	0.975515	0.024485	0.391794	0.010992	0	0.899758
ZNF132	0.975515	0.024485	0.391794	0.010992	0	0.899758
ZNF197-A	0.975515	0.024485	0.391794	0.010992	0	0.899758
ZNF705B	0.975515	0.024485	0.391794	0.010992	0	0.899758
ZNF790-A	0.975515	0.024485	0.391794	0.010992	0	0.899758
ST3GAL6-	0.975519	0.024481	1.173625	1.175431	65.57317	55.78501
HAGHL	0.975524	0.024476	1.227469	1.228278	196.7195	160.157
NCK1-AS1	0.975524	0.024476	0.830151	0.829383	104.4725	125.9662
ARSE	0.975531	0.024469	1.180631	1.182264	74.46444	62.98308
FTSJ3	0.975542	0.024458	0.742616	0.742524	1192.542	1606.069
SLC22A4	0.975545	0.024455	0.872339	0.869291	21.11678	24.29347
CHRNA5	0.975551	0.024449	1.216211	1.217155	158.7982	130.465
ZFHX3	0.975559	0.024441	0.782269	0.782021	391.2162	500.2656

LOC61303	0.975563	0.024437	0.790942	0.790637	309.1719	391.044
ZNF513	0.975563	0.024437	1.177353	1.179054	70.0188	59.38405
RALGAPA	0.975566	0.024434	0.875035	0.871468	17.38245	19.94764
PDS5B	0.975572	0.024428	1.288889	1.289237	610.1639	473.2729
ZFP82	0.975573	0.024427	0.870259	0.86804	28.89665	33.29106
FOXQ1	0.975573	0.024427	0.816277	0.815728	155.5973	190.7488
LOC10254	0.975581	0.024419	1.146782	1.149985	30.00806	26.09299
LRRC37A	0.975583	0.024417	0.809663	0.809193	186.7502	230.788
ITGAE	0.975584	0.024416	1.248661	1.249257	296.7464	237.5362
LINC0112	0.975587	0.024413	1.212692	1.234796	6.668457	5.39855
LINC0151	0.975587	0.024413	1.212692	1.234796	6.668457	5.39855
MIR600HC	0.975588	0.024412	1.18426	1.185794	80.02149	67.48187
TTC3P1	0.975597	0.024403	0.861641	0.854919	10.0138	11.71485
KPNA5	0.975604	0.024396	0.848581	0.847446	65.57317	77.37921
KMO	0.975606	0.024394	0.861998	0.855284	10.00269	11.69686
LINC0088	0.975606	0.024394	0.861998	0.855284	10.00269	11.69686
MST1P2	0.975606	0.024394	0.861998	0.855284	10.00269	11.69686
CDPF1	0.975607	0.024393	0.819822	0.819231	142.2604	173.6534
PRAM1	0.975622	0.024378	1.140614	1.146884	14.44832	12.59662
RNF150	0.975623	0.024377	1.219094	1.219966	177.8255	145.7608
FAM83G	0.975624	0.024376	1.273873	1.274294	471.2377	369.8007
ICOSLG	0.975624	0.024376	1.287436	1.287788	599.0498	465.175
B3GNT8	0.975629	0.024371	0.770344	0.741713	3.334229	4.498792
TM4SF19	0.975629	0.024371	0.770344	0.741713	3.334229	4.498792
NRXN3	0.975631	0.024369	0.816599	0.816063	162.2769	198.8556
ACTG1P1	0.975633	0.024367	1.139962	1.144761	19.0051	16.60054
APIP	0.975634	0.024366	1.281953	1.282331	544.5907	424.6859
LINC0068	0.975635	0.024365	0.826974	0.813873	6.112753	7.512982
PTGES3L	0.975636	0.024364	1.154897	1.157348	41.64452	35.98133
FIGNL2	0.975642	0.024358	0.835278	0.823705	6.668457	8.097825
OLR1	0.975642	0.024358	0.835278	0.823705	6.668457	8.097825
TATDN1	0.975646	0.024354	0.761427	0.761278	689.0739	905.1569
RBM17	0.975647	0.024353	0.734165	0.734094	1551.528	2113.532
CTCFL	0.975654	0.024346	0.394192	0.011102	0	0.890761
ZFP14	0.975658	0.024342	0.847875	0.846757	65.57317	77.4422
LOC10192	0.975659	0.024341	0.625008	0.534091	1.244779	2.339372
SLC40A1	0.975667	0.024333	0.837977	0.837114	91.13559	108.8708
GCA	0.975669	0.024331	1.292042	1.292371	653.5088	505.6642
LAMTOR5	0.975673	0.024327	1.321901	1.322121	1099.184	831.3767
INO80D	0.975678	0.024322	1.278752	1.279144	517.9169	404.8912
FSCN2	0.975689	0.024311	0.876604	0.872012	13.33691	15.29589
KLHL6	0.975689	0.024311	0.876604	0.872012	13.33691	15.29589
LOC10192	0.975689	0.024311	0.876604	0.872012	13.33691	15.29589
SLC45A4	0.975695	0.024305	0.73832	0.738241	1414.824	1916.485
TRIM44	0.975702	0.024298	0.718276	0.718231	2550.685	3551.346
CTDSP1	0.975706	0.024294	0.761202	0.761057	713.525	937.5482
GNA13	0.97571	0.02429	0.730162	0.730099	1763.807	2415.851
MEAF6	0.975716	0.024284	0.752257	0.75214	901.631	1198.757
CYSRT1	0.975721	0.024279	1.140534	1.146884	14.44832	12.59662
IFIT3	0.975721	0.024279	1.299882	1.300177	754.6582	580.4251
GPR89B	0.975725	0.024275	1.281773	1.282148	549.981	428.9508
HIST1H3H	0.975727	0.024273	1.215072	1.234858	7.779867	6.298308
SYCP2	0.975727	0.024273	1.215072	1.234858	7.779867	6.298308
LOC10028	0.975745	0.024255	0.813144	0.798192	5.77933	7.243054
TTC23	0.97575	0.02425	1.251643	1.252191	333.7896	266.5624
KCNN2	0.975753	0.024247	1.194401	1.211955	7.635384	6.298308

PCDHB10	0.975755	0.024245	1.18395	1.199231	8.213317	6.847161
CHRAC1	0.975756	0.024244	0.742414	0.742326	1229.219	1655.906
HIC2	0.97576	0.02424	0.808803	0.808358	197.8309	244.7343
LOC44115	0.975761	0.024239	1.225292	1.226085	198.4644	161.8665
HLTF	0.975767	0.024233	0.714254	0.714214	2995.249	4193.773
LINC0050	0.975769	0.024231	0.824292	0.809734	5.557048	6.865156
TONSL	0.975772	0.024228	0.732901	0.732833	1677.784	2289.453
GTPBP2	0.975774	0.024226	0.712041	0.712004	3139.732	4409.715
TMEM160	0.975779	0.024221	1.239686	1.240324	270.0725	217.7415
ASPM	0.97578	0.02422	1.382611	1.382706	3188.634	2306.081
XAF1	0.975786	0.024214	1.203522	1.204587	131.1463	108.8708
ANXA2R	0.975801	0.024199	1.212425	1.234796	6.668457	5.39855
HAPLN4	0.975801	0.024199	1.212425	1.234796	6.668457	5.39855
HIST1H3E	0.975801	0.024199	1.212425	1.234796	6.668457	5.39855
KCNIP2	0.975801	0.024199	1.212425	1.234796	6.668457	5.39855
LOC10192	0.975801	0.024199	1.212425	1.234796	6.668457	5.39855
PCBP3	0.975801	0.024199	1.212425	1.234796	6.668457	5.39855
TBX1	0.975806	0.024194	1.136762	1.140154	26.67383	23.39372
PEX13	0.975811	0.024189	0.760759	0.760617	716.8147	942.4158
TEX19	0.975812	0.024188	0.872183	0.870309	34.4537	39.58937
SDHAP3	0.975819	0.024181	1.129688	1.135881	13.35914	11.75984
SMA5	0.975821	0.024179	1.14148	1.144289	33.44231	29.22415
SLC29A1	0.975822	0.024178	1.33014	1.330334	1290.347	969.9395
C1QL1	0.975823	0.024177	1.159068	1.161082	52.23625	44.98792
HIST1H2E	0.975824	0.024176	0.876687	0.872012	13.33691	15.29589
NEB	0.975824	0.024176	0.876687	0.872012	13.33691	15.29589
FOXD4	0.975831	0.024169	0.880643	0.874782	10.00269	11.43593
RAD9B	0.975831	0.024169	1.13631	1.142942	13.37026	11.69686
HSD17B7I	0.975833	0.024167	1.161884	1.163801	55.9817	48.10108
SV2B	0.975833	0.024167	0.863834	0.857047	9.969344	11.63387
LMF1	0.975854	0.024146	1.219402	1.220244	181.1598	148.4601
SLC7A5P1	0.975856	0.024144	0.875127	0.869974	12.22551	14.05422
GUSBP1	0.975858	0.024142	0.84569	0.844702	75.1424	88.9591
LINC0067	0.97586	0.02414	0.735479	0.735407	1568.199	2132.427
B4GALT1-	0.975872	0.024128	1.133594	1.140094	13.33691	11.69686
ITGA2B	0.975872	0.024128	1.133594	1.140094	13.33691	11.69686
LOC10192	0.975872	0.024128	1.133594	1.140094	13.33691	11.69686
ZNF614	0.975875	0.024125	0.772821	0.772632	529.031	684.7161
LRRC32	0.975878	0.024122	1.128881	1.132234	24.45101	21.5942
SPRYD7	0.975892	0.024108	0.787449	0.787186	369.0102	468.7741
HIST1H2E	0.975896	0.024104	1.131934	1.138344	13.33691	11.71485
MGC2734	0.975901	0.024099	0.827351	0.826698	124.4779	150.5746
ID2-AS1	0.975906	0.024094	0.835482	0.823705	6.668457	8.097825
LINC0056	0.975906	0.024094	0.835482	0.823705	6.668457	8.097825
SLC25A47	0.975906	0.024094	0.835482	0.823705	6.668457	8.097825
AKR1D1	0.975912	0.024088	0.770683	0.741713	3.334229	4.498792
ANO5	0.975912	0.024088	0.770683	0.741713	3.334229	4.498792
CASQ2	0.975912	0.024088	0.770683	0.741713	3.334229	4.498792
CD36	0.975912	0.024088	0.770683	0.741713	3.334229	4.498792
CELF2	0.975912	0.024088	0.770683	0.741713	3.334229	4.498792
CHADL	0.975912	0.024088	0.770683	0.741713	3.334229	4.498792
CSF3R	0.975912	0.024088	0.770683	0.741713	3.334229	4.498792
CYP51A1-	0.975912	0.024088	0.770683	0.741713	3.334229	4.498792
GAS1RR	0.975912	0.024088	0.770683	0.741713	3.334229	4.498792
GJC2	0.975912	0.024088	0.770683	0.741713	3.334229	4.498792
LINC0109	0.975912	0.024088	0.770683	0.741713	3.334229	4.498792

LOC10192	0.975912	0.024088	0.770683	0.741713	3.334229	4.498792
LOC10192	0.975912	0.024088	0.770683	0.741713	3.334229	4.498792
LOC10192	0.975912	0.024088	0.770683	0.741713	3.334229	4.498792
MMP9	0.975912	0.024088	0.770683	0.741713	3.334229	4.498792
PCDHB5	0.975912	0.024088	0.770683	0.741713	3.334229	4.498792
USHBP1	0.975912	0.024088	0.770683	0.741713	3.334229	4.498792
WT1	0.975912	0.024088	0.770683	0.741713	3.334229	4.498792
AGAP11	0.975912	0.024088	0.778358	0.751586	3.534282	4.705736
ZFHX2	0.975916	0.024084	1.155963	1.157993	50.01343	43.1884
LYZ	0.975916	0.024084	1.140782	1.147756	13.43694	11.70586
APBB2	0.975919	0.024081	0.766908	0.766745	625.7236	816.0808
C10orf99	0.975921	0.024079	0.809119	0.808684	202.2765	250.1328
TNFRSF1	0.975921	0.024079	0.872753	0.864812	7.779867	8.997583
CD209	0.975932	0.024068	1.201423	1.22253	6.635115	5.425543
C7orf26	0.975932	0.024068	0.802001	0.801632	244.5101	305.0181
ZSCAN16	0.975936	0.024064	0.85516	0.854007	62.23894	72.88042
LRRN4CL	0.975945	0.024055	0.82496	0.811023	5.901585	7.279045
CCDC144	0.975962	0.024038	1.150446	1.162433	8.191089	7.045108
BMS1P5	0.975964	0.024036	0.817245	0.816739	171.0459	209.4277
OR51B5	0.975971	0.024029	1.208692	1.23471	5.557048	4.498792
LOC10028	0.975983	0.024017	0.892111	0.889416	20.00537	22.49396
MRPL34	0.975984	0.024016	0.757754	0.757625	796.8807	1051.817
DDX11	0.975989	0.024011	0.745806	0.745712	1143.985	1534.088
MROH6	0.975991	0.024009	1.2398	1.240432	264.5155	213.2427
CDHR3	0.975992	0.024008	0.836422	0.822653	5.557048	6.757185
LOC10027	0.976004	0.023996	0.887485	0.882401	11.1141	12.59662
LYG1	0.976004	0.023996	0.887485	0.882401	11.1141	12.59662
MAPRE2	0.976004	0.023996	0.887485	0.882401	11.1141	12.59662
OCA2	0.976004	0.023996	0.887485	0.882401	11.1141	12.59662
FHL3	0.976009	0.023991	1.288701	1.289025	665.7343	516.4613
UBALD1	0.97601	0.02399	1.220131	1.220937	190.051	155.6582
MIDN	0.97601	0.02399	0.779756	0.779538	446.7867	573.146
EXPH5	0.97601	0.02399	1.236759	1.237406	257.8804	208.402
SLC12A5	0.97602	0.02398	1.14886	1.150973	45.56779	39.58937
LOC10028	0.976024	0.023976	0.862731	0.861427	52.60302	61.0666
NAT6	0.976025	0.023975	1.169837	1.171405	72.24162	61.66943
RASL11A	0.976025	0.023975	0.892146	0.889416	20.00537	22.49396
CFAP57	0.976028	0.023972	1.133477	1.140094	13.33691	11.69686
RACGAP1	0.976031	0.023969	1.119851	1.12346	21.23904	18.90392
EXD1	0.976036	0.023964	0.894139	0.891157	18.01595	20.21757
TMEM41B	0.976043	0.023957	1.321947	1.322158	1150.82	870.4082
FRS3	0.976044	0.023956	0.842112	0.84125	87.80136	104.372
IL10RB	0.976045	0.023955	0.761465	0.761325	739.0874	970.7942
SEC14L4	0.976046	0.023954	1.127636	1.133333	14.72618	12.99251
TAOK2	0.976046	0.023954	1.339092	1.339258	1588.204	1185.881
B9D1	0.976049	0.023951	0.814047	0.813574	182.2712	224.0398
INMT	0.976056	0.023944	1.124051	1.130622	12.14771	10.74311
MYRF	0.976056	0.023944	1.313114	1.31335	990.2659	753.9975
CCDC114	0.976056	0.023944	0.895839	0.892178	14.44832	16.19565
PIPOX	0.976056	0.023944	0.895839	0.892178	14.44832	16.19565
NR6A1	0.976057	0.023943	0.838498	0.837702	96.81489	115.574
C2CD4A	0.976057	0.023943	1.11944	1.122875	22.22819	19.79468
FERMT3	0.976057	0.023943	1.11944	1.122875	22.22819	19.79468
C11orf49	0.976063	0.023937	0.784697	0.784453	394.5504	502.9649
PSG6	0.976066	0.023934	0.401564	0.011445	0	0.863768
FLJ41941	0.976066	0.023934	1.122891	1.129352	12.22551	10.82409

DARS-AS1	0.976067	0.023933	1.115165	1.118593	21.11678	18.87693
TBC1D15	0.976068	0.023932	1.335608	1.335781	1499.914	1122.871
ATM	0.97607	0.02393	0.75943	0.759297	784.6552	1033.399
ASAP3	0.976072	0.023928	0.763659	0.763512	697.9652	914.1544
LOC10192	0.976085	0.023915	0.863077	0.861778	51.725	60.02288
CD81	0.976085	0.023915	1.406255	1.40632	4988.006	3546.847
CYP46A1	0.976087	0.023913	0.872852	0.864812	7.779867	8.997583
LOC10050	0.976087	0.023913	0.872852	0.864812	7.779867	8.997583
PPP1R36	0.976087	0.023913	0.872852	0.864812	7.779867	8.997583
H3F3C	0.97609	0.02391	1.150822	1.160881	10.0916	8.691665
PRDX5	0.976093	0.023907	0.705542	0.705512	4018.857	5696.37
GYPC	0.9761	0.0239	1.212043	1.234796	6.668457	5.39855
REC8	0.9761	0.0239	1.212043	1.234796	6.668457	5.39855
B3GNT3	0.976109	0.023891	1.336238	1.336411	1482.62	1109.402
FAM74A3	0.976109	0.023891	1.183839	1.202729	6.657343	5.533514
C12orf60	0.976117	0.023883	1.140329	1.142549	41.12215	35.99033
RBMS3-AS	0.97612	0.02388	1.119377	1.122875	22.22819	19.79468
LRR1	0.976121	0.023879	0.77201	0.771831	559.039	724.3054
ATP6V1C2	0.976123	0.023877	0.901205	0.898401	17.78255	19.79468
DGKH	0.976126	0.023874	1.312493	1.31273	988.4988	753.0077
ZGRF1	0.976129	0.023871	0.804025	0.803651	240.0645	298.7198
CARKD	0.976139	0.023861	0.755814	0.755695	900.2418	1191.28
MIR3652	0.976146	0.023854	0.391457	0.010779	0	0.917753
SEH1L	0.976153	0.023847	0.750038	0.749936	1061.396	1415.32
PCF11-AS	0.976162	0.023838	0.877801	0.870026	7.779867	8.943598
TMC3-AS1	0.976171	0.023829	0.887577	0.882401	11.1141	12.59662
ZNF19	0.976171	0.023829	0.887577	0.882401	11.1141	12.59662
LINC0063	0.976176	0.023824	0.895906	0.892178	14.44832	16.19565
SNED1	0.976176	0.023824	0.895906	0.892178	14.44832	16.19565
RUFY2	0.976185	0.023815	0.773798	0.773615	553.482	715.4518
SLC19A3	0.976187	0.023813	0.845897	0.845025	86.68995	102.5904
LRRC37A	0.97619	0.02381	1.140479	1.142595	43.20049	37.80784
FAM131B	0.976203	0.023797	1.125437	1.132173	12.22551	10.7971
KLK14	0.976204	0.023796	1.116136	1.122813	11.1141	9.897341
SEPT1	0.976204	0.023796	1.116136	1.122813	11.1141	9.897341
XPC	0.976205	0.023795	0.749226	0.749125	1063.619	1419.819
ACOX2	0.976208	0.023792	1.208384	1.23471	5.557048	4.498792
CALHM3	0.976208	0.023792	1.208384	1.23471	5.557048	4.498792
DFNB59	0.976208	0.023792	1.208384	1.23471	5.557048	4.498792
FAM150B	0.976208	0.023792	1.208384	1.23471	5.557048	4.498792
FAM167B	0.976208	0.023792	1.208384	1.23471	5.557048	4.498792
LINC0052	0.976208	0.023792	1.208384	1.23471	5.557048	4.498792
LINC0090	0.976208	0.023792	1.208384	1.23471	5.557048	4.498792
LOC10192	0.976208	0.023792	1.208384	1.23471	5.557048	4.498792
LRP2BP	0.976208	0.023792	1.208384	1.23471	5.557048	4.498792
MYLPF	0.976208	0.023792	1.208384	1.23471	5.557048	4.498792
PRRT1	0.976208	0.023792	1.208384	1.23471	5.557048	4.498792
NLGN3	0.976213	0.023787	1.108222	1.111646	20.00537	17.99517
TOE1	0.976216	0.023784	0.808394	0.807984	212.2792	262.7294
TNRC6A	0.976222	0.023778	0.736019	0.735949	1607.098	2183.713
LOC55338	0.976224	0.023776	1.107339	1.112562	13.01461	11.69686
KIF4B	0.976226	0.023774	1.204389	1.229855	5.612618	4.561775
ZNF204P	0.976228	0.023772	0.76812	0.767959	630.1692	820.5796
DDX19B	0.976232	0.023768	0.782469	0.782246	442.441	565.6061
LRRC56	0.976235	0.023765	0.896771	0.894518	23.3396	26.09299
ALKBH3	0.976236	0.023764	0.807745	0.807348	222.2819	275.326

SLC10A4	0.976242	0.023758	0.903905	0.898453	8.891277	9.897341
ST8SIA1	0.976243	0.023757	1.111272	1.114837	20.1832	18.10314
MAP3K1	0.97625	0.02375	0.761753	0.761616	752.4243	987.9346
CDC14C	0.976255	0.023745	0.902319	0.899983	21.15012	23.50169
MTRNR2L	0.976256	0.023744	1.168764	1.17022	77.25408	66.01527
CHD9	0.976273	0.023727	0.765596	0.765445	679.0713	887.1617
LOC10537	0.976281	0.023719	0.393806	0.010884	0	0.908756
PVT1	0.976283	0.023717	1.191176	1.192248	123.3665	103.4722
C19orf48	0.976283	0.023717	0.713671	0.713634	3143.066	4404.317
RIN3	0.976286	0.023714	1.247043	1.247574	336.7571	269.9275
TPRKB	0.97629	0.02371	0.766279	0.766127	667.9572	871.8658
FUT8-AS1	0.976293	0.023707	1.101757	1.105145	18.89396	17.09541
HIST1H2A	0.976293	0.023707	1.101757	1.105145	18.89396	17.09541
TMEM151	0.976295	0.023705	1.126974	1.129313	35.56511	31.49154
CARD16	0.9763	0.0237	1.177363	1.178655	92.19142	78.21599
STEAP1B	0.976301	0.023699	1.166526	1.168018	73.23078	62.69516
ANKRD20	0.976304	0.023696	0.771168	0.741713	3.334229	4.498792
MYO1F	0.976304	0.023696	0.771168	0.741713	3.334229	4.498792
SLC7A9	0.976304	0.023696	0.771168	0.741713	3.334229	4.498792
TIPARP-A	0.976304	0.023696	0.771168	0.741713	3.334229	4.498792
MEIS3P1	0.976306	0.023694	1.225013	1.225724	220.9482	180.2576
DND1	0.976308	0.023692	0.909714	0.905906	12.22551	13.49637
LINC0156	0.976308	0.023692	0.909714	0.905906	12.22551	13.49637
MTMR7	0.976308	0.023692	0.909714	0.905906	12.22551	13.49637
ARFGAP1	0.976313	0.023687	1.364196	1.364311	2455.104	1799.517
PRKCB	0.97632	0.02368	0.872995	0.864812	7.779867	8.997583
SIGLEC1	0.97632	0.02368	0.872995	0.864812	7.779867	8.997583
SLC30A4	0.976332	0.023668	1.172084	1.173446	84.46713	71.98066
HMHA1	0.976336	0.023664	1.242854	1.243409	315.6403	253.8488
LRRC37A	0.976337	0.023663	0.813281	0.812841	196.7195	242.017
ARHGAP4	0.976346	0.023654	0.81448	0.81403	191.1624	234.8369
SUCLA2	0.976347	0.023653	0.743361	0.743276	1314.798	1768.925
LINC0051	0.976353	0.023647	0.913116	0.910223	15.55973	17.09541
MYLK4	0.976353	0.023647	0.913116	0.910223	15.55973	17.09541
C7orf13	0.976365	0.023635	0.867777	0.866528	52.23625	60.28381
FDXACB1	0.976366	0.023634	0.861104	0.859991	61.12753	71.08091
CENPN	0.976377	0.023623	1.32926	1.329442	1395.942	1050.018
POTEJ	0.976378	0.023622	1.455401	1.644928	2.211705	1.34064
SNX32	0.976381	0.023619	0.894008	0.892145	28.89665	32.3913
SELM	0.976385	0.023615	1.228688	1.229365	233.396	189.849
DGUOK-A	0.976387	0.023613	1.104948	1.111584	10.00269	8.997583
EMILIN1	0.976387	0.023613	1.104948	1.111584	10.00269	8.997583
LOC10050	0.976387	0.023613	0.858468	0.857414	65.57317	76.47946
F7	0.976389	0.023611	0.903973	0.898453	8.891277	9.897341
GNGT1	0.976389	0.023611	0.903973	0.898453	8.891277	9.897341
ZNF561	0.976399	0.023601	0.772194	0.772022	594.6041	770.1931
MAP10	0.976401	0.023599	0.905065	0.902711	21.11678	23.39372
LOC10192	0.976409	0.023591	0.800796	0.800464	273.7624	342.0071
RASA4CP	0.976412	0.023588	0.887811	0.886136	34.44258	38.86956
LOC10099	0.976414	0.023586	0.876591	0.866851	6.246122	7.207064
ABCA8	0.976416	0.023584	0.396184	0.010992	0	0.899758
ABCG8	0.976416	0.023584	0.396184	0.010992	0	0.899758
AGR3	0.976416	0.023584	0.396184	0.010992	0	0.899758
ARG1	0.976416	0.023584	0.396184	0.010992	0	0.899758
ARRDC5	0.976416	0.023584	0.396184	0.010992	0	0.899758
ASB14	0.976416	0.023584	0.396184	0.010992	0	0.899758

BEND2	0.976416	0.023584	0.396184	0.010992	0	0.899758
BIN3-IT1	0.976416	0.023584	0.396184	0.010992	0	0.899758
BTNL8	0.976416	0.023584	0.396184	0.010992	0	0.899758
C16orf86	0.976416	0.023584	0.396184	0.010992	0	0.899758
C17orf64	0.976416	0.023584	0.396184	0.010992	0	0.899758
C2orf71	0.976416	0.023584	0.396184	0.010992	0	0.899758
CASC6	0.976416	0.023584	0.396184	0.010992	0	0.899758
CCAT2	0.976416	0.023584	0.396184	0.010992	0	0.899758
CCR7	0.976416	0.023584	0.396184	0.010992	0	0.899758
CCR8	0.976416	0.023584	0.396184	0.010992	0	0.899758
CFAP61	0.976416	0.023584	0.396184	0.010992	0	0.899758
CFAP99	0.976416	0.023584	0.396184	0.010992	0	0.899758
CPXM1	0.976416	0.023584	0.396184	0.010992	0	0.899758
CSH2	0.976416	0.023584	0.396184	0.010992	0	0.899758
CYP27C1	0.976416	0.023584	0.396184	0.010992	0	0.899758
DBX2	0.976416	0.023584	0.396184	0.010992	0	0.899758
DCAF13P3	0.976416	0.023584	0.396184	0.010992	0	0.899758
DLG2	0.976416	0.023584	0.396184	0.010992	0	0.899758
DNAJB5-A	0.976416	0.023584	0.396184	0.010992	0	0.899758
DPRX	0.976416	0.023584	0.396184	0.010992	0	0.899758
DRC7	0.976416	0.023584	0.396184	0.010992	0	0.899758
DSCR4	0.976416	0.023584	0.396184	0.010992	0	0.899758
EPO	0.976416	0.023584	0.396184	0.010992	0	0.899758
EVADR	0.976416	0.023584	0.396184	0.010992	0	0.899758
FABP7	0.976416	0.023584	0.396184	0.010992	0	0.899758
FAIM2	0.976416	0.023584	0.396184	0.010992	0	0.899758
FAM106B	0.976416	0.023584	0.396184	0.010992	0	0.899758
FAM46D	0.976416	0.023584	0.396184	0.010992	0	0.899758
FBXO39	0.976416	0.023584	0.396184	0.010992	0	0.899758
FOCAD-A	0.976416	0.023584	0.396184	0.010992	0	0.899758
GP1BA	0.976416	0.023584	0.396184	0.010992	0	0.899758
GRK7	0.976416	0.023584	0.396184	0.010992	0	0.899758
HVCN1	0.976416	0.023584	0.396184	0.010992	0	0.899758
IFNA1	0.976416	0.023584	0.396184	0.010992	0	0.899758
IGSF5	0.976416	0.023584	0.396184	0.010992	0	0.899758
KCNJ9	0.976416	0.023584	0.396184	0.010992	0	0.899758
LILRA2	0.976416	0.023584	0.396184	0.010992	0	0.899758
LILRA4	0.976416	0.023584	0.396184	0.010992	0	0.899758
LINC0067	0.976416	0.023584	0.396184	0.010992	0	0.899758
LINC0069	0.976416	0.023584	0.396184	0.010992	0	0.899758
LINC0112	0.976416	0.023584	0.396184	0.010992	0	0.899758
LINC0119	0.976416	0.023584	0.396184	0.010992	0	0.899758
LINC0128	0.976416	0.023584	0.396184	0.010992	0	0.899758
LINC0162	0.976416	0.023584	0.396184	0.010992	0	0.899758
LOC1002	0.976416	0.023584	0.396184	0.010992	0	0.899758
LOC1005	0.976416	0.023584	0.396184	0.010992	0	0.899758
LOC1005	0.976416	0.023584	0.396184	0.010992	0	0.899758
LOC1019	0.976416	0.023584	0.396184	0.010992	0	0.899758
LOC1019	0.976416	0.023584	0.396184	0.010992	0	0.899758
LOC1019	0.976416	0.023584	0.396184	0.010992	0	0.899758
LOC1019	0.976416	0.023584	0.396184	0.010992	0	0.899758
LOC1019	0.976416	0.023584	0.396184	0.010992	0	0.899758
LOC1019	0.976416	0.023584	0.396184	0.010992	0	0.899758
LOC1019	0.976416	0.023584	0.396184	0.010992	0	0.899758
LOC1019	0.976416	0.023584	0.396184	0.010992	0	0.899758
LOC1019	0.976416	0.023584	0.396184	0.010992	0	0.899758

LOC10192	0.976416	0.023584	0.396184	0.010992	0	0.899758
LOC10272	0.976416	0.023584	0.396184	0.010992	0	0.899758
LOC10272	0.976416	0.023584	0.396184	0.010992	0	0.899758
LOC10272	0.976416	0.023584	0.396184	0.010992	0	0.899758
LOC10537	0.976416	0.023584	0.396184	0.010992	0	0.899758
LOC25688	0.976416	0.023584	0.396184	0.010992	0	0.899758
LOC44004	0.976416	0.023584	0.396184	0.010992	0	0.899758
LOC64732	0.976416	0.023584	0.396184	0.010992	0	0.899758
LOC72998	0.976416	0.023584	0.396184	0.010992	0	0.899758
LRRC70	0.976416	0.023584	0.396184	0.010992	0	0.899758
LRTM2	0.976416	0.023584	0.396184	0.010992	0	0.899758
LYL1	0.976416	0.023584	0.396184	0.010992	0	0.899758
MAFA-AS1	0.976416	0.023584	0.396184	0.010992	0	0.899758
MARK2P9	0.976416	0.023584	0.396184	0.010992	0	0.899758
MBOAT4	0.976416	0.023584	0.396184	0.010992	0	0.899758
MKRN9P	0.976416	0.023584	0.396184	0.010992	0	0.899758
MT1IP	0.976416	0.023584	0.396184	0.010992	0	0.899758
MYLK-AS2	0.976416	0.023584	0.396184	0.010992	0	0.899758
NAGPA-A	0.976416	0.023584	0.396184	0.010992	0	0.899758
NBPF18P	0.976416	0.023584	0.396184	0.010992	0	0.899758
NECAB1	0.976416	0.023584	0.396184	0.010992	0	0.899758
NOX1	0.976416	0.023584	0.396184	0.010992	0	0.899758
NRG3	0.976416	0.023584	0.396184	0.010992	0	0.899758
NTRK2	0.976416	0.023584	0.396184	0.010992	0	0.899758
NWD2	0.976416	0.023584	0.396184	0.010992	0	0.899758
OLIG1	0.976416	0.023584	0.396184	0.010992	0	0.899758
OR13C5	0.976416	0.023584	0.396184	0.010992	0	0.899758
OR1J2	0.976416	0.023584	0.396184	0.010992	0	0.899758
PCAT18	0.976416	0.023584	0.396184	0.010992	0	0.899758
PCDHGA2	0.976416	0.023584	0.396184	0.010992	0	0.899758
PCSK1	0.976416	0.023584	0.396184	0.010992	0	0.899758
PKLR	0.976416	0.023584	0.396184	0.010992	0	0.899758
PLVAP	0.976416	0.023584	0.396184	0.010992	0	0.899758
PRAMEF4	0.976416	0.023584	0.396184	0.010992	0	0.899758
PRDM14	0.976416	0.023584	0.396184	0.010992	0	0.899758
PWAR1	0.976416	0.023584	0.396184	0.010992	0	0.899758
RFPL3	0.976416	0.023584	0.396184	0.010992	0	0.899758
RNVU1-8	0.976416	0.023584	0.396184	0.010992	0	0.899758
ROBO2	0.976416	0.023584	0.396184	0.010992	0	0.899758
RPS6KA6	0.976416	0.023584	0.396184	0.010992	0	0.899758
SCG3	0.976416	0.023584	0.396184	0.010992	0	0.899758
SCGB1A1	0.976416	0.023584	0.396184	0.010992	0	0.899758
SDR9C7	0.976416	0.023584	0.396184	0.010992	0	0.899758
SH2D4B	0.976416	0.023584	0.396184	0.010992	0	0.899758
SLA	0.976416	0.023584	0.396184	0.010992	0	0.899758
SLC16A11	0.976416	0.023584	0.396184	0.010992	0	0.899758
SLC1A2	0.976416	0.023584	0.396184	0.010992	0	0.899758
SMARCA5	0.976416	0.023584	0.396184	0.010992	0	0.899758
SMPX	0.976416	0.023584	0.396184	0.010992	0	0.899758
SMTNL1	0.976416	0.023584	0.396184	0.010992	0	0.899758
SNORA52	0.976416	0.023584	0.396184	0.010992	0	0.899758
SNORA71	0.976416	0.023584	0.396184	0.010992	0	0.899758
SNORA9	0.976416	0.023584	0.396184	0.010992	0	0.899758
SNORD10	0.976416	0.023584	0.396184	0.010992	0	0.899758
SNORD15	0.976416	0.023584	0.396184	0.010992	0	0.899758
SP7	0.976416	0.023584	0.396184	0.010992	0	0.899758

SPATA1	0.976416	0.023584	0.396184	0.010992	0	0.899758
SPATA21	0.976416	0.023584	0.396184	0.010992	0	0.899758
SPDYE6	0.976416	0.023584	0.396184	0.010992	0	0.899758
SRD5A3-A	0.976416	0.023584	0.396184	0.010992	0	0.899758
STAB2	0.976416	0.023584	0.396184	0.010992	0	0.899758
STXBP5L	0.976416	0.023584	0.396184	0.010992	0	0.899758
SYN3	0.976416	0.023584	0.396184	0.010992	0	0.899758
SYP-AS1	0.976416	0.023584	0.396184	0.010992	0	0.899758
TAC3	0.976416	0.023584	0.396184	0.010992	0	0.899758
TAC4	0.976416	0.023584	0.396184	0.010992	0	0.899758
TAL2	0.976416	0.023584	0.396184	0.010992	0	0.899758
TAS1R1	0.976416	0.023584	0.396184	0.010992	0	0.899758
TAS2R60	0.976416	0.023584	0.396184	0.010992	0	0.899758
TDGF1P3	0.976416	0.023584	0.396184	0.010992	0	0.899758
TDRD1	0.976416	0.023584	0.396184	0.010992	0	0.899758
TDRD10	0.976416	0.023584	0.396184	0.010992	0	0.899758
TEKT5	0.976416	0.023584	0.396184	0.010992	0	0.899758
TEX13B	0.976416	0.023584	0.396184	0.010992	0	0.899758
TFDP3	0.976416	0.023584	0.396184	0.010992	0	0.899758
TGFB2-AS	0.976416	0.023584	0.396184	0.010992	0	0.899758
THCAT15	0.976416	0.023584	0.396184	0.010992	0	0.899758
THEG	0.976416	0.023584	0.396184	0.010992	0	0.899758
THRIL	0.976416	0.023584	0.396184	0.010992	0	0.899758
TMC2	0.976416	0.023584	0.396184	0.010992	0	0.899758
TMEM232	0.976416	0.023584	0.396184	0.010992	0	0.899758
TMPRSS1	0.976416	0.023584	0.396184	0.010992	0	0.899758
TNFSF18	0.976416	0.023584	0.396184	0.010992	0	0.899758
TONSL-AS	0.976416	0.023584	0.396184	0.010992	0	0.899758
TSPAN16	0.976416	0.023584	0.396184	0.010992	0	0.899758
TSPAN7	0.976416	0.023584	0.396184	0.010992	0	0.899758
TUBA3C	0.976416	0.023584	0.396184	0.010992	0	0.899758
UNC93A	0.976416	0.023584	0.396184	0.010992	0	0.899758
YBX3P1	0.976416	0.023584	0.396184	0.010992	0	0.899758
ZNF560	0.976416	0.023584	0.396184	0.010992	0	0.899758
ZNF727	0.976416	0.023584	0.396184	0.010992	0	0.899758
ZNF733P	0.976416	0.023584	0.396184	0.010992	0	0.899758
ZNF735	0.976416	0.023584	0.396184	0.010992	0	0.899758
ZNF835	0.976416	0.023584	0.396184	0.010992	0	0.899758
CEP170P1	0.976419	0.023581	0.917514	0.913824	11.62534	12.72258
C11orf71	0.97642	0.02358	0.856178	0.855178	70.0188	81.87801
KIAA1328	0.976421	0.023579	0.851597	0.850694	81.1329	95.37438
VPS25	0.976422	0.023578	0.734087	0.734021	1696.011	2310.579
RASGRP4	0.976426	0.023574	0.852593	0.840498	5.934927	7.063103
LOC10192	0.976433	0.023567	1.117029	1.119387	32.23088	28.79227
HDAC4	0.976436	0.023564	0.804702	0.804343	248.9557	309.5169
PDCL	0.97644	0.02356	1.277918	1.278269	587.5022	459.6055
PABPC4L	0.97644	0.02356	1.158819	1.160342	68.90739	59.38405
ARX	0.976442	0.023558	1.202967	1.234579	4.445638	3.599033
LOC10013	0.976446	0.023554	1.134613	1.146767	7.224162	6.298308
APITD1-C	0.976448	0.023552	0.894849	0.887623	7.346417	8.277776
DEAF1	0.976451	0.023549	1.243465	1.244013	314.5289	252.8321
LOC10050	0.976457	0.023543	0.909783	0.905906	12.22551	13.49637
SFMBT2	0.976457	0.023543	0.802965	0.777305	3.389799	4.363828
ARHGEF3	0.976467	0.023533	0.840601	0.823814	4.445638	5.39855
C10orf82	0.976467	0.023533	0.840601	0.823814	4.445638	5.39855
C1orf127	0.976467	0.023533	0.840601	0.823814	4.445638	5.39855

EPHB6	0.976467	0.023533	0.840601	0.823814	4.445638	5.39855
HSP90B2I	0.976467	0.023533	0.840601	0.823814	4.445638	5.39855
LINC0113	0.976467	0.023533	0.840601	0.823814	4.445638	5.39855
LINC0162	0.976467	0.023533	0.840601	0.823814	4.445638	5.39855
PKD1L1	0.976467	0.023533	0.840601	0.823814	4.445638	5.39855
PLXDC1	0.976467	0.023533	0.840601	0.823814	4.445638	5.39855
WBP2NL	0.976467	0.023533	0.840601	0.823814	4.445638	5.39855
AGER	0.976472	0.023528	1.113338	1.115651	31.11947	27.89251
VWA5A	0.976472	0.023528	1.113338	1.115651	31.11947	27.89251
LOC10192	0.976474	0.023526	1.116912	1.119154	33.23115	29.69202
GOLGA8J	0.976479	0.023521	1.222699	1.252806	5.368108	4.28285
PIGF	0.976485	0.023515	0.78885	0.788598	376.7678	477.7717
GOLT1A	0.976488	0.023512	1.234612	1.235221	271.1839	219.541
ZNF670-Z	0.976492	0.023508	0.892664	0.886212	8.54674	9.645409
CRYBB2	0.976499	0.023501	1.193037	1.222515	4.490095	3.671014
SKP2	0.9765	0.0235	0.761787	0.761655	798.0699	1047.814
TMEM242	0.976505	0.023495	0.813769	0.813346	207.8336	255.5314
BRF2	0.97651	0.02349	1.234614	1.235221	272.2953	220.4408
BBS4	0.976535	0.023465	1.199262	1.200173	152.2631	126.8659
MRGPRX	0.976538	0.023462	0.925547	0.923102	16.0043	17.33834
FAM212A	0.976538	0.023462	1.207944	1.23471	5.557048	4.498792
LINC0062	0.976539	0.023461	1.077699	1.080771	15.55973	14.39613
TAOK1	0.97654	0.02346	1.370754	1.370856	2819.646	2056.847
ENTPD8	0.976541	0.023459	0.929271	0.926475	13.33691	14.39613
ZNF699	0.976541	0.023459	0.895869	0.894148	31.37509	35.09057
TNFSF14	0.976544	0.023456	1.128139	1.130096	41.58895	36.80011
WDR72	0.976546	0.023454	1.09686	1.099312	24.72886	22.49396
TMEM141	0.97656	0.02344	0.750913	0.750814	1086.959	1447.711
ZNF23	0.976568	0.023432	0.818884	0.818413	178.9369	218.6413
HSPA6	0.976572	0.023428	1.167434	1.168776	81.93311	70.10017
ANKRD65	0.976575	0.023425	0.932293	0.930175	16.70449	17.95918
AVIL	0.976582	0.023418	1.091439	1.097862	8.891277	8.097825
HES7	0.976582	0.023418	1.091439	1.097862	8.891277	8.097825
NUDT11	0.976582	0.023418	1.091439	1.097862	8.891277	8.097825
TREX2	0.976582	0.023418	1.091439	1.097862	8.891277	8.097825
PCDHGA7	0.976585	0.023415	1.165476	1.166821	81.73306	70.04618
MAP3K6	0.976585	0.023415	1.291614	1.291906	734.6417	568.6472
PAM16	0.976588	0.023412	0.797642	0.797345	315.6403	395.8667
GRK4	0.97659	0.02341	1.10483	1.111584	10.00269	8.997583
TMEM254	0.97659	0.02341	1.10483	1.111584	10.00269	8.997583
TUSC3	0.97659	0.02341	1.10483	1.111584	10.00269	8.997583
CREBL2	0.976591	0.023409	0.773124	0.772952	577.933	747.6992
RAB15	0.976592	0.023408	0.824008	0.823496	164.4886	199.7463
PGAM4	0.976593	0.023407	0.850137	0.834195	4.456752	5.344564
HP09025	0.976595	0.023405	0.809402	0.783952	3.334229	4.255857
FOXA2	0.976597	0.023403	0.930172	0.926492	10.00269	10.7971
SNRK-AS	0.976597	0.023403	0.930172	0.926492	10.00269	10.7971
UNC79	0.976597	0.023403	0.930172	0.926492	10.00269	10.7971
CD200R1	0.976597	0.023403	0.904072	0.898453	8.891277	9.897341
LOC28371	0.976597	0.023403	0.904072	0.898453	8.891277	9.897341
HSD17B1	0.9766	0.0234	1.30147	1.301726	870.2337	668.5204
UBE2Q2P	0.976606	0.023394	1.077807	1.080378	18.61611	17.23037
UNC119	0.976606	0.023394	1.273867	1.274232	544.5907	427.3852
ZHX1-C8c	0.976608	0.023392	1.118638	1.120662	37.41005	33.38103
NRIP1	0.97661	0.02339	0.732365	0.732304	1823.823	2490.531
CCNB1IP1	0.976614	0.023386	0.761084	0.760955	802.4377	1054.517

SAP130	0.976615	0.023385	0.763883	0.763745	743.533	973.5385
MRPL27	0.976617	0.023383	0.797132	0.796839	321.1974	403.0917
ACTC1	0.976619	0.023381	1.067629	1.070482	14.44832	13.49637
BTBD19	0.976619	0.023381	1.067629	1.070482	14.44832	13.49637
CEL	0.976619	0.023381	1.067629	1.070482	14.44832	13.49637
GTPBP1	0.976623	0.023377	1.335035	1.3352	1543.748	1156.189
NEK5	0.976624	0.023376	0.921575	0.919558	20.36102	22.14305
CLCNKB	0.976626	0.023374	0.922949	0.920583	17.39356	18.89492
KLF8	0.97663	0.02337	1.273786	1.27415	545.7021	428.285
RCL1	0.976633	0.023367	0.768688	0.768535	673.5142	876.3646
WDR45	0.976638	0.023362	0.777049	0.776863	540.0228	695.1353
RBM47	0.976639	0.023361	0.748699	0.748604	1136.972	1518.792
RGS20	0.976652	0.023348	0.80088	0.800561	284.5209	355.4045
SLC6A4	0.976653	0.023347	0.874566	0.864542	6.26835	7.252052
SLC4A1AI	0.976662	0.023338	0.754321	0.754214	1000.269	1326.244
PRMT9	0.976675	0.023325	0.81154	0.811142	218.9477	269.9275
MAPK10	0.976676	0.023324	0.929322	0.926475	13.33691	14.39613
STX1B	0.976676	0.023324	0.929322	0.926475	13.33691	14.39613
ZCCHC16	0.976683	0.023317	0.401027	0.011214	0	0.881763
LOC1003	0.976685	0.023315	0.903073	0.901415	30.00806	33.29106
MAPKAPK	0.976686	0.023314	0.772189	0.772022	600.1612	777.3912
GORASP1	0.976688	0.023312	1.332604	1.332772	1533.745	1150.791
EDA2R	0.976691	0.023309	0.928761	0.926464	16.67114	17.99517
LOC10012	0.976694	0.023306	1.115084	1.126138	6.668457	5.92041
CUBN	0.976697	0.023303	1.056184	1.058723	13.33691	12.59662
LINC0052	0.976697	0.023303	1.056184	1.058723	13.33691	12.59662
MRPL45P	0.976701	0.023299	1.055886	1.058355	13.637	12.88454
BEX1	0.976703	0.023297	1.09428	1.096525	26.71829	24.36545
ATP6V1B	0.976707	0.023293	1.202603	1.234579	4.445638	3.599033
CTB-178M	0.976707	0.023293	1.202603	1.234579	4.445638	3.599033
HIST1H2A	0.976707	0.023293	1.202603	1.234579	4.445638	3.599033
IRF4	0.976707	0.023293	1.202603	1.234579	4.445638	3.599033
KIAA1755	0.976707	0.023293	1.202603	1.234579	4.445638	3.599033
LINC0087	0.976707	0.023293	1.202603	1.234579	4.445638	3.599033
LOC10013	0.976707	0.023293	1.202603	1.234579	4.445638	3.599033
LOC10192	0.976707	0.023293	1.202603	1.234579	4.445638	3.599033
LOC10192	0.976707	0.023293	1.202603	1.234579	4.445638	3.599033
LOC64662	0.976707	0.023293	1.202603	1.234579	4.445638	3.599033
MGC2889	0.976707	0.023293	1.202603	1.234579	4.445638	3.599033
PDIA2	0.976707	0.023293	1.202603	1.234579	4.445638	3.599033
PAFAH1B	0.976709	0.023291	1.315717	1.315928	1123.635	853.8706
CARD9	0.976711	0.023289	0.928347	0.926457	20.00537	21.5942
UBAP2	0.976715	0.023285	1.328159	1.328336	1427.05	1074.311
FRRS1	0.976717	0.023283	0.837234	0.836582	118.9208	142.1528
PTCHD4	0.976717	0.023283	0.894546	0.886973	7.157478	8.070832
CCDC169	0.976719	0.023281	1.082724	1.084905	23.42851	21.5942
NDUFB2-A	0.976722	0.023278	0.946612	0.944625	14.44832	15.29589
THAP2	0.976729	0.023271	0.779631	0.779436	511.2484	655.9238
LOC10050	0.976736	0.023264	0.942859	0.94116	17.78255	18.89492
C9orf43	0.976738	0.023262	0.95253	0.95022	11.1141	11.69686
DUSP13	0.976738	0.023262	0.95253	0.95022	11.1141	11.69686
RASSF8-A	0.976738	0.023262	0.95253	0.95022	11.1141	11.69686
JMJD1C-A	0.976739	0.023261	1.078741	1.08079	23.3396	21.5942
CAPS2	0.976742	0.023258	0.892226	0.882495	5.557048	6.298308
CDNF	0.976742	0.023258	0.892226	0.882495	5.557048	6.298308
HIST1H2A	0.976742	0.023258	0.892226	0.882495	5.557048	6.298308

IL7R	0.976742	0.023258	0.892226	0.882495	5.557048	6.298308
OR7E91P	0.976742	0.023258	0.892226	0.882495	5.557048	6.298308
PRRT4	0.976742	0.023258	0.892226	0.882495	5.557048	6.298308
SYF2	0.976747	0.023253	0.76709	0.766945	713.525	930.3501
HAS3	0.976749	0.023251	1.234641	1.235221	284.5209	230.3381
GATA6-AS	0.976752	0.023248	1.010062	1.010633	10.00269	9.897341
RGS7	0.976752	0.023248	1.010062	1.010633	10.00269	9.897341
GOLGA8H	0.976756	0.023244	1.064089	1.066175	18.64945	17.4913
WFDC8	0.976767	0.023233	1.15577	1.178878	4.456752	3.778985
RRN3P1	0.976768	0.023232	1.156921	1.158274	77.9765	67.31992
VWCE	0.97677	0.02323	1.043062	1.045157	12.22551	11.69686
ZCWPW2	0.97677	0.02323	1.043062	1.045157	12.22551	11.69686
ITGA9	0.976772	0.023228	1.078715	1.08079	23.3396	21.5942
P2RX7	0.976772	0.023228	1.078715	1.08079	23.3396	21.5942
LOC10192	0.976775	0.023225	1.084778	1.086965	24.45101	22.49396
BNIPL	0.976778	0.023222	0.928506	0.926586	20.01649	21.6032
LURAP1L	0.976785	0.023215	0.806247	0.805898	254.5128	315.8152
LRRC74B	0.976788	0.023212	0.631268	0.530108	1.11141	2.105434
PLGLB1	0.976789	0.023211	0.930973	0.927264	10.00269	10.7881
MR1	0.97679	0.02321	1.322625	1.322814	1311.463	991.4167
ACSBG2	0.97679	0.02321	1.074804	1.080715	7.779867	7.198066
ALDH8A1	0.97679	0.02321	1.074804	1.080715	7.779867	7.198066
CCDC183	0.97679	0.02321	1.074804	1.080715	7.779867	7.198066
FOXP4-AS	0.97679	0.02321	1.074804	1.080715	7.779867	7.198066
GMNC	0.97679	0.02321	1.074804	1.080715	7.779867	7.198066
LINC0085	0.97679	0.02321	1.074804	1.080715	7.779867	7.198066
LOC10192	0.97679	0.02321	1.074804	1.080715	7.779867	7.198066
LY6G6C	0.97679	0.02321	1.074804	1.080715	7.779867	7.198066
TRPV2	0.97679	0.02321	1.074804	1.080715	7.779867	7.198066
UCP3	0.97679	0.02321	1.074804	1.080715	7.779867	7.198066
MECR	0.976791	0.023209	0.766593	0.766448	706.8565	922.2523
TMEM191	0.976794	0.023206	1.069553	1.071834	18.96065	17.68925
LINC0141	0.976794	0.023206	0.942881	0.94116	17.78255	18.89492
MCF2L2	0.976797	0.023203	1.073562	1.079368	7.779867	7.207064
BCO1	0.976802	0.023198	0.840887	0.823814	4.445638	5.39855
DRC3	0.976802	0.023198	0.840887	0.823814	4.445638	5.39855
LOC10012	0.976802	0.023198	0.840887	0.823814	4.445638	5.39855
MATN4	0.976802	0.023198	0.840887	0.823814	4.445638	5.39855
MCPH1-A	0.976802	0.023198	0.840887	0.823814	4.445638	5.39855
N4BP2L2-	0.976802	0.023198	0.840887	0.823814	4.445638	5.39855
WFIKKN1	0.976802	0.023198	0.840887	0.823814	4.445638	5.39855
DUSP19	0.976802	0.023198	1.064914	1.066757	21.11678	19.79468
TBC1D32	0.976805	0.023195	0.886763	0.885428	42.22245	47.68719
DOC2B	0.976805	0.023195	1.091325	1.097862	8.891277	8.097825
LOC10192	0.976805	0.023195	1.091325	1.097862	8.891277	8.097825
DNAAF3	0.976805	0.023195	0.820586	0.820121	180.0484	219.541
REP15	0.976811	0.023189	1.072095	1.074078	22.22819	20.69444
SV2C	0.976813	0.023187	1.090196	1.096645	8.891277	8.106822
ENTPD1-A	0.976816	0.023184	0.942169	0.938208	7.790981	8.304769
GS1-124K	0.976819	0.023181	0.8061	0.805754	256.9468	318.8923
GSTCD	0.976819	0.023181	0.805932	0.805591	266.7383	331.1111
LOC10050	0.976822	0.023178	1.078676	1.08079	23.3396	21.5942
ENTHD2	0.976823	0.023177	0.808888	0.808521	240.0645	296.9202
CANX	0.976825	0.023175	0.64213	0.642127	48885.35	76130.35
C9orf172	0.976831	0.023169	0.971835	0.970562	12.22551	12.59662
LURAP1L-	0.976831	0.023169	0.971835	0.970562	12.22551	12.59662

CYP4B1	0.976835	0.023165	1.027864	1.029332	11.1141	10.7971
DOCK3	0.976835	0.023165	1.027864	1.029332	11.1141	10.7971
GPR85	0.976835	0.023165	1.027864	1.029332	11.1141	10.7971
HBA1	0.976835	0.023165	1.027864	1.029332	11.1141	10.7971
ZKSCAN7	0.976835	0.023165	1.027864	1.029332	11.1141	10.7971
ELFN1-AS	0.976839	0.023161	1.126077	1.127789	46.6792	41.38888
FAM21C	0.976839	0.023161	0.734444	0.734382	1805.785	2458.922
DGCR5	0.976841	0.023159	1.056139	1.058723	13.33691	12.59662
NAT14	0.976844	0.023156	0.763296	0.763164	777.9867	1019.426
FBXO36	0.976846	0.023154	1.064891	1.066757	21.11678	19.79468
EPHA10	0.976849	0.023151	0.944221	0.942335	16.1599	17.14939
IFI27	0.976857	0.023143	0.767564	0.767418	694.631	905.1569
FLJ42627	0.976858	0.023142	0.799754	0.799449	295.6349	369.8007
EREG	0.976858	0.023142	0.929755	0.924218	6.668457	7.216062
MAP1LC3	0.97686	0.02314	1.01734	1.018177	11.99211	11.77784
CFAP43	0.976862	0.023138	0.962092	0.96076	15.55973	16.19565
PLA2G3	0.976864	0.023136	0.928078	0.926453	23.3396	25.19323
ROM1	0.976867	0.023133	1.028358	1.029341	16.67114	16.19565
HPX	0.976873	0.023127	0.931907	0.926526	6.668457	7.198066
POM121L	0.976873	0.023127	0.931907	0.926526	6.668457	7.198066
KGFLP1	0.976873	0.023127	0.849032	0.848251	92.18031	108.6728
PTK6	0.976875	0.023125	0.989913	0.989449	12.25885	12.38967
DDX11-AS	0.976876	0.023124	0.899817	0.898376	35.56511	39.58937
PIPSL	0.976877	0.023123	1.003203	1.003369	11.01407	10.97705
ZNF678	0.976886	0.023114	1.174612	1.175706	108.9181	92.63912
DCST2	0.976887	0.023113	1.010055	1.010633	10.00269	9.897341
MMP23B	0.976889	0.023111	1.26139	1.311403	3.967732	3.023188
NPIPB15	0.976893	0.023107	1.014047	1.01489	9.624807	9.483453
MCEMP1	0.976903	0.023097	0.928108	0.926453	23.3396	25.19323
PAXIP1-A	0.976903	0.023097	0.928108	0.926453	23.3396	25.19323
UBR5-AS	0.976906	0.023094	1.048388	1.049919	18.89396	17.99517
DCHS1	0.976906	0.023094	0.952572	0.95022	11.1141	11.69686
TRIM74	0.976906	0.023094	0.925415	0.920435	7.991035	8.682668
GGT5	0.976908	0.023092	1.0898	1.09166	30.00806	27.48762
TLR10	0.976908	0.023092	0.883371	0.868889	3.923276	4.516787
ST20	0.97691	0.02309	1.171708	1.172826	104.4725	89.07607
HSPA12A	0.976913	0.023087	1.205374	1.206154	184.494	152.9589
LOC72789	0.976915	0.023085	1.010949	1.011641	9.102444	8.997583
FAM90A7I	0.976915	0.023085	0.832642	0.809566	3.334229	4.120893
LINC0096	0.976918	0.023082	1.087593	1.089441	29.4079	26.99275
GBAT2	0.976919	0.023081	0.9889	0.988198	8.891277	8.997583
RAB33A	0.976919	0.023081	0.9889	0.988198	8.891277	8.997583
WDFY3-A	0.976919	0.023081	0.9889	0.988198	8.891277	8.997583
CCDC158	0.976921	0.023079	0.963358	0.960784	7.779867	8.097825
FER1L6	0.976921	0.023079	0.963358	0.960784	7.779867	8.097825
LOC1027	0.976921	0.023079	0.963358	0.960784	7.779867	8.097825
LOC73018	0.976921	0.023079	0.963358	0.960784	7.779867	8.097825
TRIM31-A	0.976921	0.023079	0.963358	0.960784	7.779867	8.097825
TTLL6	0.976921	0.023079	0.963358	0.960784	7.779867	8.097825
ELFN2	0.976923	0.023077	1.329052	1.329224	1445.944	1087.808
C4orf47	0.976924	0.023076	1.003485	1.003623	14.44832	14.39613
PLA2G4F	0.976924	0.023076	1.003485	1.003623	14.44832	14.39613
GPCPD1	0.976927	0.023073	0.800266	0.799965	302.3034	377.8985
HARS2	0.976927	0.023073	0.75027	0.750175	1125.858	1500.797
CCZ1	0.976929	0.023071	0.789556	0.789315	386.7261	489.9544
SYNPO2L	0.976937	0.023063	0.978852	0.976976	6.179437	6.325301

PTGDS	0.976938	0.023062	1.028345	1.029341	16.67114	16.19565
ACTL10	0.976938	0.023062	1.01662	1.017238	15.55973	15.29589
ATP8A1	0.976938	0.023062	1.01662	1.017238	15.55973	15.29589
HCST	0.976938	0.023062	1.01662	1.017238	15.55973	15.29589
GNRHR2	0.976939	0.023061	0.970381	0.96939	16.49332	17.01443
PAQR8	0.976939	0.023061	1.234661	1.235221	294.5235	238.436
RBMX2	0.97695	0.02305	0.775193	0.775021	574.5988	741.4008
SNAP47	0.976952	0.023048	1.270377	1.270735	556.8162	438.1823
CASKIN1	0.976953	0.023047	0.911695	0.910197	31.11947	34.19082
WDR93	0.976954	0.023046	1.167411	1.192942	4.445638	3.724999
SH3BP5-A	0.976958	0.023042	1.106745	1.10851	38.89934	35.09057
TMED10P	0.976959	0.023041	1.036195	1.037977	12.22551	11.77784
MRPL23	0.976961	0.023039	0.793159	0.792905	374.545	472.3731
SLC9A7P1	0.97697	0.02303	0.887773	0.886522	44.66755	50.38647
ANKRD2	0.97697	0.02303	0.835998	0.835398	130.0349	155.6582
LOC10192	0.976973	0.023027	1.078603	1.08036	27.52962	25.48116
IAPP	0.976973	0.023027	0.973822	0.971904	7.546471	7.764914
MIA3	0.976978	0.023022	0.743507	0.743428	1372.591	1846.304
UGT8	0.976982	0.023018	0.787158	0.786933	423.836	538.5953
FBXW2	0.976986	0.023014	1.346399	1.346534	1954.969	1451.85
KLHL6-AS	0.976986	0.023014	1.128853	1.142633	6.190551	5.416545
ABCC6P2	0.976986	0.023014	1.066325	1.067976	24.41767	22.86286
ULK2	0.976988	0.023012	1.175771	1.176856	107.1399	91.03755
GGTLC2	0.976992	0.023008	0.973465	0.972368	13.64811	14.03623
PTOV1-AS	0.976993	0.023007	0.873979	0.872916	58.90471	67.48187
LYSMD1	0.976993	0.023007	0.788152	0.787926	426.7813	541.6545
SLC16A9	0.976997	0.023003	1.153712	1.154999	80.02149	69.28139
ZBTB49	0.976999	0.023001	0.878329	0.877213	54.45907	62.08332
FDPSP2	0.977003	0.022997	1.16376	1.185009	5.29031	4.462801
PMS2P2	0.977004	0.022996	1.02991	1.031065	15.49305	15.02596
CNN1	0.977004	0.022996	0.868611	0.86762	65.57317	75.5797
PRIMPOL	0.977005	0.022995	0.825837	0.825343	165.6	200.6461
C10orf25	0.977007	0.022993	1.103453	1.105176	37.78793	34.19082
SLC5A5	0.977011	0.022989	1.111831	1.127741	4.445638	3.940941
LINC0123	0.977012	0.022988	0.827725	0.827222	164.4886	198.8466
LINC0024	0.977013	0.022987	1.053817	1.058676	6.668457	6.298308
LOC64417	0.977013	0.022987	1.053817	1.058676	6.668457	6.298308
MYOZ1	0.977013	0.022987	1.053817	1.058676	6.668457	6.298308
BCRP3	0.977017	0.022983	0.984084	0.983367	12.67007	12.88454
CHRM3	0.977021	0.022979	1.063467	1.065043	24.41767	22.92584
HACD3	0.977025	0.022975	1.389886	1.38996	4143.335	2980.899
TIGD3	0.977028	0.022972	0.98868	0.988194	13.33691	13.49637
C1orf220	0.977035	0.022965	0.892394	0.882495	5.557048	6.298308
FAM231A	0.977035	0.022965	0.892394	0.882495	5.557048	6.298308
NUGGC	0.977035	0.022965	0.892394	0.882495	5.557048	6.298308
SDK2	0.977035	0.022965	0.892394	0.882495	5.557048	6.298308
SLC2A5	0.977035	0.022965	0.892394	0.882495	5.557048	6.298308
LINC0123	0.977035	0.022965	1.074701	1.080715	7.779867	7.198066
LOC10193	0.977035	0.022965	1.074701	1.080715	7.779867	7.198066
SNORA63	0.977035	0.022965	1.074701	1.080715	7.779867	7.198066
SOX18	0.977035	0.022965	1.074701	1.080715	7.779867	7.198066
TCAP	0.977035	0.022965	1.074701	1.080715	7.779867	7.198066
CHIC1	0.977036	0.022964	0.801138	0.800837	301.192	376.099
VAMP1	0.977038	0.022962	1.16766	1.168801	97.80404	83.67752
FOXD3-AS	0.97704	0.02296	1.172978	1.174062	106.6953	90.87559
PCDHGA8	0.977041	0.022959	0.999341	0.999289	7.201934	7.207064

ING5	0.977044	0.022956	0.809648	0.809296	253.4014	313.1159
C3orf67	0.977047	0.022953	1.096229	1.097953	35.56511	32.3913
TMEM170	0.977048	0.022952	1.269882	1.270237	560.0726	440.9176
WDR73	0.97705	0.02295	0.807599	0.807256	257.847	319.4142
C8orf88	0.977051	0.022949	0.988554	0.988192	17.78255	17.99517
DMRT2	0.977051	0.022949	1.016608	1.017238	15.55973	15.29589
TBX2-AS1	0.977051	0.022949	1.016608	1.017238	15.55973	15.29589
B3GALNT	0.977052	0.022948	0.745555	0.745472	1305.951	1751.847
KHK	0.977054	0.022946	1.176448	1.177492	113.3638	96.27414
LOC64362	0.977054	0.022946	1.206498	1.251587	3.211974	2.564311
PAPLN	0.977055	0.022945	0.998744	0.998677	10.79179	10.8061
TYMP	0.97706	0.02294	1.037638	1.038723	20.50551	19.7407
SGK494	0.977061	0.022939	1.160475	1.161684	87.80136	75.5797
LINC0131	0.977066	0.022934	0.976011	0.975197	16.67114	17.09541
PABPC1P	0.977071	0.022929	1.33714	1.443797	2.589584	1.790519
FLJ10038	0.977073	0.022927	0.938439	0.937096	24.45101	26.09299
SLC35B3	0.977073	0.022927	0.762269	0.762145	846.8941	1111.202
CRYGS	0.977074	0.022926	1.202083	1.234579	4.445638	3.599033
CSRP3	0.977074	0.022926	1.202083	1.234579	4.445638	3.599033
DSCR9	0.977074	0.022926	1.202083	1.234579	4.445638	3.599033
LGI4	0.977074	0.022926	1.202083	1.234579	4.445638	3.599033
NAPSB	0.977074	0.022926	1.202083	1.234579	4.445638	3.599033
SRRM4	0.977074	0.022926	1.202083	1.234579	4.445638	3.599033
TERC	0.977074	0.022926	1.202083	1.234579	4.445638	3.599033
TEX21P	0.977074	0.022926	1.202083	1.234579	4.445638	3.599033
A4GALT	0.977076	0.022924	1.266597	1.266965	532.3652	420.1871
TAS2R5	0.977078	0.022922	1.010045	1.010633	10.00269	9.897341
BATF2	0.977083	0.022917	1.092362	1.094032	34.4537	31.49154
CARD8	0.977089	0.022911	0.824043	0.823567	171.2015	207.8802
ARHGAP1	0.977092	0.022908	0.81325	0.812869	226.7276	278.9251
ADAMTS1	0.977094	0.022906	0.951382	0.950199	22.22819	23.39372
FAM84A	0.977097	0.022903	0.832316	0.810732	3.645423	4.498792
RSPH10B	0.977109	0.022891	0.752285	0.706778	2.222819	3.149154
RSPH10B	0.977109	0.022891	0.752285	0.706778	2.222819	3.149154
KIAA1614	0.977115	0.022885	1.021778	1.023742	6.45729	6.307306
EIF5AL1	0.977119	0.022881	1.007238	1.007454	19.17182	19.02989
LOC38976	0.977127	0.022873	1.038497	1.039538	21.90588	21.07234
TIE1	0.97713	0.02287	0.988912	0.988198	8.891277	8.997583
TNFRSF1	0.97713	0.02287	0.988912	0.988198	8.891277	8.997583
CARMN	0.977133	0.022867	0.932001	0.926526	6.668457	7.198066
DPF3	0.977133	0.022867	0.932001	0.926526	6.668457	7.198066
SLC25A21	0.977133	0.022867	0.932001	0.926526	6.668457	7.198066
ZBTB7C	0.977133	0.022867	0.932001	0.926526	6.668457	7.198066
PKD1P6	0.977136	0.022864	0.755034	0.75493	1039.468	1376.909
FBP1	0.977138	0.022862	0.98856	0.988192	17.78255	17.99517
ZNF790	0.977138	0.022862	0.98856	0.988192	17.78255	17.99517
TSC22D3	0.977151	0.022849	1.339693	1.339838	1779.367	1328.043
NUBPL	0.977152	0.022848	0.78786	0.787637	426.9369	542.0504
LINC0085	0.977154	0.022846	0.963403	0.960784	7.779867	8.097825
TNFRSF1	0.977154	0.022846	0.963403	0.960784	7.779867	8.097825
TCF7L1	0.977156	0.022844	0.811342	0.810979	240.0645	296.0205
FOLR3	0.977157	0.022843	1.010341	1.010638	20.00537	19.79468
TMEM108	0.977157	0.022843	0.913076	0.911742	34.4537	37.78985
INSR	0.977159	0.022841	0.759896	0.759779	888.0163	1168.786
ARHGAP2	0.977165	0.022835	0.75606	0.755953	986.9317	1305.549
LOC44017	0.977167	0.022833	1.004141	1.004526	6.146095	6.118356

ZBED9	0.977171	0.022829	0.822352	0.821911	192.2739	233.9372
C1QTNF2	0.977171	0.022829	1.074303	1.075819	30.00806	27.89251
FBXO43	0.977175	0.022825	0.913052	0.911742	34.4537	37.78985
COX5A	0.977176	0.022824	0.736319	0.736256	1776.033	2412.252
GLCCI1	0.977178	0.022822	1.186423	1.187338	137.8148	116.0688
TMEM229	0.97719	0.02281	0.999951	0.999949	18.89396	18.89492
APBB3	0.977191	0.022809	1.248968	1.249421	391.2162	313.1159
TCP11L2	0.977193	0.022807	0.874703	0.873717	64.46176	73.78018
GOLGA6L	0.977197	0.022803	0.935016	0.928115	4.968001	5.353562
C17orf67	0.977203	0.022797	0.978469	0.977902	21.11678	21.5942
MFSD4	0.977203	0.022797	0.978469	0.977902	21.11678	21.5942
DSG3	0.977208	0.022792	0.758909	0.714365	2.233933	3.131159
DTX3L	0.97721	0.02279	1.371365	1.37146	3072.225	2240.11
ARGLU1	0.977216	0.022784	0.756355	0.756249	1012.494	1338.84
RASL12	0.977219	0.022781	1.03665	1.037578	23.3396	22.49396
LOC10192	0.977221	0.022779	1.051017	1.052213	25.56242	24.29347
LOC44044	0.977222	0.022778	1.179523	1.207621	4.467867	3.698007
WIPF3	0.977223	0.022777	1.044103	1.045176	24.45101	23.39372
TRIM6-TR	0.977224	0.022776	0.816489	0.785184	2.611813	3.329106
SGTB	0.977227	0.022773	0.775134	0.774969	615.2541	793.9107
MBNL1	0.977227	0.022773	1.370422	1.370517	3107.501	2267.391
FCAR	0.977238	0.022762	0.9164	0.906637	4.412296	4.867692
COX18	0.977241	0.022759	1.21347	1.214146	218.3586	179.8437
ACTG1P2	0.977245	0.022755	1.092733	1.094265	37.71013	34.46074
SCAF8	0.977248	0.022752	0.756541	0.756435	1011.383	1337.041
ANKRD45	0.977249	0.022751	1.026513	1.029305	5.557048	5.39855
CXCR2	0.977249	0.022751	1.026513	1.029305	5.557048	5.39855
CYP11A1	0.977249	0.022751	1.026513	1.029305	5.557048	5.39855
LOC10027	0.977249	0.022751	1.026513	1.029305	5.557048	5.39855
LOC10050	0.977249	0.022751	1.026513	1.029305	5.557048	5.39855
MYH15	0.977249	0.022751	1.026513	1.029305	5.557048	5.39855
PAN3-AS1	0.977249	0.022751	1.026513	1.029305	5.557048	5.39855
PCDHGB3	0.977249	0.022751	1.026513	1.029305	5.557048	5.39855
PNPLA7	0.977249	0.022751	1.026513	1.029305	5.557048	5.39855
RHOXF1-A	0.977249	0.022751	1.026513	1.029305	5.557048	5.39855
SLC7A10	0.977249	0.022751	1.026513	1.029305	5.557048	5.39855
YWHAEP7	0.977249	0.022751	1.026513	1.029305	5.557048	5.39855
PIGZ	0.977254	0.022746	0.948125	0.94703	25.56242	26.99275
TSGA10	0.977254	0.022746	0.948125	0.94703	25.56242	26.99275
NLRC3	0.977254	0.022746	1.027119	1.029994	5.523706	5.362559
CLIC6	0.97726	0.02274	0.937033	0.935803	27.78524	29.69202
LINC0088	0.977261	0.022739	0.97848	0.977902	21.11678	21.5942
STAG3	0.977261	0.022739	1.006814	1.007516	5.557048	5.515518
ZNF493	0.977263	0.022737	1.044726	1.045832	24.55104	23.47469
HSPA1L	0.977266	0.022734	1.036633	1.037578	23.3396	22.49396
DPH3P1	0.977268	0.022732	0.790558	0.75027	2.211705	2.951207
LSM6	0.977269	0.022731	0.818101	0.817695	208.945	255.5314
FUT6	0.977273	0.022727	1.191924	1.230482	3.478712	2.825241
SPAG7	0.977277	0.022723	0.779915	0.779733	547.9249	702.7112
ITGBL1	0.977283	0.022717	1.053733	1.058676	6.668457	6.298308
PSD	0.977283	0.022717	1.053733	1.058676	6.668457	6.298308
SMCO2	0.977283	0.022717	1.053733	1.058676	6.668457	6.298308
SNHG25	0.977283	0.022717	1.053733	1.058676	6.668457	6.298308
SRPK3	0.977283	0.022717	1.053733	1.058676	6.668457	6.298308
MIATNB	0.977283	0.022717	1.006788	1.006957	22.65053	22.49396
DLK2	0.977287	0.022713	0.874685	0.873717	64.46176	73.78018

ABCC6	0.977291	0.022709	0.851548	0.850844	104.2058	122.4751
NTN4	0.977292	0.022708	0.764443	0.764315	799.1035	1045.519
UBE2E4P	0.977295	0.022705	0.6368	0.526871	1.000269	1.907488
SH3BP5L	0.977305	0.022695	1.329796	1.329958	1575.979	1184.982
MAN1B1	0.97731	0.02269	1.300633	1.300868	969.1492	744.9999
LOC10028	0.977311	0.022689	0.832049	0.831531	153.3745	184.4505
NEIL3	0.977319	0.022681	1.224381	1.22497	265.6269	216.8418
KIF3A	0.977324	0.022676	0.743448	0.743372	1432.551	1927.102
GOLGA8C	0.977328	0.022672	1.032443	1.033254	23.99533	23.22276
CXorf51A	0.977334	0.022666	1.518706	1.843472	1.667114	0.899758
LOC64236	0.977337	0.022663	1.059743	1.06101	29.04113	27.37065
SARDH	0.977341	0.022659	0.899527	0.898371	44.45638	49.48671
DLEU2	0.977344	0.022656	0.930613	0.929433	31.10835	33.47101
GDF1	0.977347	0.022653	1.143792	1.145028	77.33188	67.53586
CASP10	0.977347	0.022653	1.263755	1.264126	512.8711	405.71
TBC1D1	0.977348	0.022652	1.341658	1.341798	1847.163	1376.63
MROH7	0.977349	0.022651	1.003132	1.003482	5.10137	5.083634
AGBL2	0.97735	0.02265	1.19365	1.234363	3.334229	2.699275
ATP2B3	0.97735	0.02265	1.19365	1.234363	3.334229	2.699275
CALCB	0.97735	0.02265	1.19365	1.234363	3.334229	2.699275
CASC19	0.97735	0.02265	1.19365	1.234363	3.334229	2.699275
COLEC10	0.97735	0.02265	1.19365	1.234363	3.334229	2.699275
FAM25A	0.97735	0.02265	1.19365	1.234363	3.334229	2.699275
GLIS3-AS	0.97735	0.02265	1.19365	1.234363	3.334229	2.699275
GZMM	0.97735	0.02265	1.19365	1.234363	3.334229	2.699275
KRT38	0.97735	0.02265	1.19365	1.234363	3.334229	2.699275
LINC0096	0.97735	0.02265	1.19365	1.234363	3.334229	2.699275
LINC0146	0.97735	0.02265	1.19365	1.234363	3.334229	2.699275
LOC10192	0.97735	0.02265	1.19365	1.234363	3.334229	2.699275
MYCBPAF	0.97735	0.02265	1.19365	1.234363	3.334229	2.699275
MYL4	0.97735	0.02265	1.19365	1.234363	3.334229	2.699275
TSPAN32	0.97735	0.02265	1.19365	1.234363	3.334229	2.699275
ZNF252P-	0.97735	0.02265	1.19365	1.234363	3.334229	2.699275
SLC46A1	0.977356	0.022644	1.251009	1.251434	429.0041	342.8079
ZNF787	0.977364	0.022636	0.750248	0.750159	1220.328	1626.763
CCDC163	0.977372	0.022628	1.119433	1.120833	54.45907	48.58695
RABGGTA	0.97738	0.02262	1.197017	1.197787	177.8255	148.4601
PCDHGA1	0.977382	0.022618	1.114832	1.132955	4.078873	3.599033
PID1	0.977383	0.022617	1.193504	1.194315	162.2658	135.8635
SEPT7P9	0.977384	0.022616	0.940966	0.932371	3.589853	3.850966
LOC10050	0.97741	0.02259	0.964529	0.960301	4.579007	4.768719
ST20-AS1	0.977411	0.022589	0.997744	0.997688	23.3396	23.39372
PCDH18	0.977413	0.022587	0.957198	0.956324	26.67383	27.89251
C2orf27A	0.977437	0.022563	1.072551	1.073865	34.39813	32.0314
LINC0056	0.977438	0.022562	0.911404	0.899319	3.74545	4.165881
LOC72960	0.977441	0.022559	1.038455	1.039289	27.29622	26.26394
HAPLN3	0.977449	0.022551	1.238731	1.23922	344.537	278.0253
KDM6A	0.97745	0.02255	1.355181	1.355295	2396.199	1768.025
GLIPR1L2	0.977452	0.022548	0.92028	0.919132	36.32087	39.51738
NT5C	0.977456	0.022544	0.844967	0.844346	120.0322	142.1618
AVL9	0.977459	0.022541	1.360934	1.36104	2645.155	1943.478
HRG	0.977465	0.022535	0.936515	0.926627	3.334229	3.599033
IFNE	0.977465	0.022535	0.936515	0.926627	3.334229	3.599033
LOC10013	0.977465	0.022535	0.936515	0.926627	3.334229	3.599033
SNORD13	0.977465	0.022535	0.936515	0.926627	3.334229	3.599033
LOC72973	0.977466	0.022534	0.996536	0.996457	24.90669	24.99529

FOLH1	0.977467	0.022533	0.969085	0.968433	26.14035	26.99275
SLC2A3	0.977475	0.022525	0.980094	0.978161	5.8349	5.965398
CCDC116	0.977476	0.022524	1.014325	1.014648	25.56242	25.19323
DHRS2	0.977477	0.022523	0.888311	0.887296	56.68189	63.88284
MGC5734	0.977478	0.022522	0.819972	0.819574	215.3245	262.7294
ARL14EPI	0.97748	0.02252	0.709858	0.619729	1.11141	1.799517
LGALS17A	0.97748	0.02252	0.709858	0.619729	1.11141	1.799517
MIR497HG	0.97748	0.02252	0.709858	0.619729	1.11141	1.799517
PFKFB1	0.97748	0.02252	0.709858	0.619729	1.11141	1.799517
SLC6A1	0.97748	0.02252	0.709858	0.619729	1.11141	1.799517
ZNF541	0.97748	0.02252	0.709858	0.619729	1.11141	1.799517
SLC38A3	0.977492	0.022508	1.09347	1.09484	43.34497	39.58937
SLC6A11	0.977495	0.022505	0.991722	0.990676	4.456752	4.498792
AGBL3	0.977495	0.022505	0.989533	0.988211	4.445638	4.498792
AMY2B	0.977495	0.022505	0.989533	0.988211	4.445638	4.498792
C18orf65	0.977495	0.022505	0.989533	0.988211	4.445638	4.498792
CHST8	0.977495	0.022505	0.989533	0.988211	4.445638	4.498792
CSAG3	0.977495	0.022505	0.989533	0.988211	4.445638	4.498792
HAGLROS	0.977495	0.022505	0.989533	0.988211	4.445638	4.498792
HMP19	0.977495	0.022505	0.989533	0.988211	4.445638	4.498792
LOC10192	0.977495	0.022505	0.989533	0.988211	4.445638	4.498792
MYH3	0.977495	0.022505	0.989533	0.988211	4.445638	4.498792
PODNL1	0.977495	0.022505	0.989533	0.988211	4.445638	4.498792
ZIC5	0.977495	0.022505	0.989533	0.988211	4.445638	4.498792
EPB41L5	0.977499	0.022501	0.765717	0.765588	793.5464	1036.522
PCDHGB4	0.97751	0.02249	0.831184	0.800127	2.467329	3.086171
LINC0127	0.977515	0.022485	1.021777	1.022252	26.67383	26.09299
TPX2	0.977522	0.022478	1.42431	1.424353	8002.149	5618.091
CBARP	0.977529	0.022471	1.093495	1.09484	43.34497	39.58937
THRAP3	0.977534	0.022466	0.722103	0.722062	2835.206	3926.545
ABCC6P1	0.977535	0.022465	0.838115	0.808675	2.5229	3.122161
CHAF1B	0.977536	0.022464	0.772837	0.772685	647.9518	838.5747
SIRT4	0.977544	0.022456	1.028742	1.029349	27.78524	26.99275
FAM231B	0.977548	0.022452	1.026465	1.029305	5.557048	5.39855
FTCD	0.977548	0.022452	1.026465	1.029305	5.557048	5.39855
LINC0122	0.977548	0.022452	1.026465	1.029305	5.557048	5.39855
SCARNA1	0.977548	0.022452	1.026465	1.029305	5.557048	5.39855
FAM173A	0.977558	0.022442	0.823915	0.823495	200.0537	242.9347
ANAPC4	0.977561	0.022439	0.795129	0.794888	391.2162	492.1678
MBLAC1	0.977567	0.022433	1.035268	1.035988	28.89665	27.89251
ULK4P3	0.97757	0.02243	0.986412	0.984983	5.423679	5.506521
KCNE3	0.977571	0.022429	0.986181	0.98589	26.67383	27.05573
LYRM1	0.977573	0.022427	0.753772	0.753677	1135.861	1507.095
FAM222A	0.977574	0.022426	0.897603	0.896557	50.01343	55.78501
KMT2E-AS	0.977574	0.022426	0.87528	0.874393	70.0188	80.07849
HMGN3-A	0.977575	0.022425	0.988433	0.988189	26.67383	26.99275
KMT2C	0.977578	0.022422	0.733572	0.733515	1966.084	2680.362
RBFA	0.977584	0.022416	0.843814	0.843222	126.7007	150.2596
UBE2M	0.977592	0.022408	0.706156	0.706131	4739.05	6711.297
SCAMP1-A	0.977601	0.022399	1.067139	1.068288	35.56511	33.29106
ZNF501	0.977601	0.022399	1.067139	1.068288	35.56511	33.29106
MPP6	0.977601	0.022399	0.807596	0.807284	283.4094	351.0677
PSMB8	0.977602	0.022398	1.307051	1.30726	1089.181	833.1762
SUSD5	0.977605	0.022395	0.842467	0.841899	135.592	161.0567
APBB1IP	0.977608	0.022392	0.943649	0.942693	32.23088	34.19082
OR4F5	0.97761	0.02239	2.261337	75.46444	0.744644	0

PIGL	0.977612	0.022388	0.915164	0.91409	41.12215	44.98792
LENG8-AS	0.977616	0.022384	0.943632	0.942693	32.23088	34.19082
ZNF382	0.977616	0.022384	0.943632	0.942693	32.23088	34.19082
LINC0127	0.977616	0.022384	0.854356	0.824139	2.222819	2.699275
LOC10192	0.977616	0.022384	0.854356	0.824139	2.222819	2.699275
LRRK2	0.977616	0.022384	0.854356	0.824139	2.222819	2.699275
RRS1-AS1	0.977616	0.022384	0.854356	0.824139	2.222819	2.699275
TCF24	0.977616	0.022384	0.854356	0.824139	2.222819	2.699275
THPO	0.977616	0.022384	0.854356	0.824139	2.222819	2.699275
PTENP1	0.977623	0.022377	0.999269	0.999167	4.045531	4.048912
PLA2G4C	0.977625	0.022375	0.927485	0.926442	36.67652	39.58937
ARHGEF1	0.977633	0.022367	0.750943	0.750855	1236.999	1647.457
HIST1H2A	0.977635	0.022365	1.155709	1.188776	3.178631	2.672282
C1orf50	0.977638	0.022362	0.833298	0.832811	165.6	198.8466
B3GLCT	0.977648	0.022352	0.833704	0.833207	158.9316	190.7488
DLG5-AS1	0.977655	0.022345	0.996233	0.996156	27.78524	27.89251
MALSU1	0.97766	0.02234	1.263659	1.264012	536.8108	424.6859
KIAA1522	0.977664	0.022336	1.412659	1.412709	6617.333	4684.142
ZNF124	0.977666	0.022334	0.846739	0.846133	121.7327	143.8714
ERCC6	0.977674	0.022326	0.795146	0.794906	385.9036	485.4736
CHDH	0.977678	0.022322	0.836364	0.835849	151.1628	180.8514
TUBA3FP	0.977678	0.022322	0.738034	0.658632	1.211436	1.844505
JAK3	0.977681	0.022319	1.219971	1.220549	264.4266	216.6438
NYAP1	0.977695	0.022305	1.182197	1.183024	151.1517	127.7657
HNRNPUL	0.977697	0.022303	0.73025	0.730199	2199.813	3012.625
C2orf76	0.977703	0.022297	1.150865	1.151938	92.247	80.07849
INCENP	0.977706	0.022294	1.322554	1.322724	1428.161	1079.71
GDPD1	0.977708	0.022292	0.864046	0.863301	91.52458	106.0185
PLXNB1	0.977713	0.022287	0.747871	0.74779	1324.8	1771.624
LRRC1	0.977714	0.022286	0.772012	0.771867	692.4082	897.059
DNAJC3-A	0.977723	0.022277	1.003554	1.003624	28.89665	28.79227
TRIM46	0.977723	0.022277	1.003554	1.003624	28.89665	28.79227
SNTA1	0.977723	0.022277	1.289119	1.289374	846.8941	656.8236
ZNF333	0.977727	0.022273	1.199807	1.200514	193.3853	161.0837
DNAH17-A	0.977729	0.022271	1.0057	1.005813	29.04113	28.87324
ANKRD20	0.97774	0.02226	0.936629	0.926627	3.334229	3.599033
CALML3-A	0.97774	0.02226	0.936629	0.926627	3.334229	3.599033
CCDC185	0.97774	0.02226	0.936629	0.926627	3.334229	3.599033
CCR1	0.97774	0.02226	0.936629	0.926627	3.334229	3.599033
CD79B	0.97774	0.02226	0.936629	0.926627	3.334229	3.599033
CHRNA10	0.97774	0.02226	0.936629	0.926627	3.334229	3.599033
DKKL1	0.97774	0.02226	0.936629	0.926627	3.334229	3.599033
GGT8P	0.97774	0.02226	0.936629	0.926627	3.334229	3.599033
HECW2	0.97774	0.02226	0.936629	0.926627	3.334229	3.599033
LINC0089	0.97774	0.02226	0.936629	0.926627	3.334229	3.599033
LOC10013	0.97774	0.02226	0.936629	0.926627	3.334229	3.599033
LOC10050	0.97774	0.02226	0.936629	0.926627	3.334229	3.599033
MANEA-A	0.97774	0.02226	0.936629	0.926627	3.334229	3.599033
MIR4737	0.97774	0.02226	0.936629	0.926627	3.334229	3.599033
PDX1	0.97774	0.02226	0.936629	0.926627	3.334229	3.599033
PRR34	0.97774	0.02226	0.936629	0.926627	3.334229	3.599033
PTH1R	0.97774	0.02226	0.936629	0.926627	3.334229	3.599033
SCN2A	0.97774	0.02226	0.936629	0.926627	3.334229	3.599033
ZNF257	0.97774	0.02226	0.936629	0.926627	3.334229	3.599033
PSMB7	0.97774	0.02226	1.36716	1.367254	3038.594	2222.403
HERC2P3	0.977749	0.022251	0.893549	0.892595	57.81553	64.7736

LINC0092(	0.977751	0.022249	0.941949	0.932786	3.356457	3.599033
TBC1D31	0.977752	0.022248	0.791987	0.791767	434.5611	548.8526
PALM2	0.977752	0.022248	1.119727	1.120934	64.53955	57.57553
SCLT1	0.977755	0.022245	1.245316	1.245751	394.5504	316.7149
LINC0097(	0.977755	0.022245	0.876603	0.85057	2.256161	2.654287
ACAD8	0.977757	0.022243	0.783506	0.783321	520.1397	664.0216
TMEM171	0.97776	0.02224	0.906855	0.905854	48.90202	53.9855
ALPK2	0.977762	0.022238	1.193016	1.234363	3.334229	2.699275
BGN	0.977762	0.022238	1.193016	1.234363	3.334229	2.699275
HAND2	0.977762	0.022238	1.193016	1.234363	3.334229	2.699275
MAGEA9	0.977762	0.022238	1.193016	1.234363	3.334229	2.699275
MESP2	0.977762	0.022238	1.193016	1.234363	3.334229	2.699275
NTN5	0.977762	0.022238	1.193016	1.234363	3.334229	2.699275
SNORA44	0.977762	0.022238	1.193016	1.234363	3.334229	2.699275
TCF20	0.977769	0.022231	0.732845	0.732791	2107.233	2875.628
MND1	0.97777	0.02223	1.211725	1.212346	235.6188	194.3478
FGFR4	0.977777	0.022223	1.306284	1.306491	1100.295	842.1738
ANGPT1	0.977779	0.022221	0.946823	0.938324	3.334229	3.554045
PILRA	0.977782	0.022218	1.112995	1.114216	58.74911	52.72584
ODF2	0.97779	0.02221	1.341125	1.341257	1968.306	1467.506
SMARCAL	0.977792	0.022208	0.781122	0.780949	571.2645	731.5035
LRRC37A	0.977794	0.022206	0.705437	0.613626	1.11141	1.817512
RALA	0.977796	0.022204	1.38465	1.384724	4231.136	3055.579
JAKMIP2	0.977797	0.022203	0.838581	0.805415	2.222819	2.762258
TCEA3	0.9778	0.0222	1.118216	1.119408	64.46176	57.58453
ILDR1	0.977806	0.022194	0.942018	0.941144	35.56511	37.78985
PELI2	0.977807	0.022193	1.131122	1.132275	73.35303	64.7826
POU5F1B	0.977807	0.022193	1.180549	1.218347	3.367571	2.762258
KCNK5	0.977807	0.022193	0.787782	0.78758	471.2377	598.3393
MTSS1	0.97781	0.02219	0.893068	0.892128	57.7933	64.7826
HSD17B1	0.977814	0.022186	0.753037	0.752946	1162.534	1543.985
TNNI1	0.977817	0.022183	0.980885	0.978448	4.445638	4.543779
GNPTG	0.977817	0.022183	0.788135	0.787931	467.9034	593.8405
DBR1	0.97782	0.02218	0.782223	0.782046	557.9943	713.5083
DYRK4	0.977825	0.022175	1.141465	1.142569	82.24431	71.98066
SCAND2P	0.977828	0.022172	0.927398	0.92644	40.01074	43.1884
JAM3	0.97783	0.02217	0.989556	0.988211	4.445638	4.498792
LOC10192	0.97783	0.02217	0.989556	0.988211	4.445638	4.498792
MIS18A	0.977831	0.022169	1.290436	1.290686	853.5626	661.3224
THAP9	0.977834	0.022166	0.855822	0.855171	110.0295	128.6654
PABPC3	0.977838	0.022162	0.708095	0.616663	1.11141	1.808514
FAM90A2	0.977838	0.022162	1.070296	1.083266	3.334229	3.077173
DDIT4L	0.977842	0.022158	0.877464	0.87663	73.35303	83.67752
IKZF3	0.977854	0.022146	1.097514	1.098702	51.34712	46.73345
FAM50A	0.977857	0.022143	0.724581	0.724538	2609.59	3601.732
RREB1	0.977865	0.022135	0.75785	0.757749	1054.728	1391.926
SLC25A41	0.977874	0.022126	0.988399	0.988189	31.11947	31.49154
DENND2D	0.977877	0.022123	0.784117	0.783932	521.2511	664.9214
ADGRV1	0.97788	0.02212	0.710773	0.619729	1.11141	1.799517
AHSG	0.97788	0.02212	0.710773	0.619729	1.11141	1.799517
ALB	0.97788	0.02212	0.710773	0.619729	1.11141	1.799517
ANKRD22	0.97788	0.02212	0.710773	0.619729	1.11141	1.799517
ANKRD63	0.97788	0.02212	0.710773	0.619729	1.11141	1.799517
ANO3	0.97788	0.02212	0.710773	0.619729	1.11141	1.799517
ASB11	0.97788	0.02212	0.710773	0.619729	1.11141	1.799517
AXDND1	0.97788	0.02212	0.710773	0.619729	1.11141	1.799517

BEST2	0.97788	0.02212	0.710773	0.619729	1.11141	1.799517
BEST4	0.97788	0.02212	0.710773	0.619729	1.11141	1.799517
BTG4	0.97788	0.02212	0.710773	0.619729	1.11141	1.799517
C2orf78	0.97788	0.02212	0.710773	0.619729	1.11141	1.799517
CADM2	0.97788	0.02212	0.710773	0.619729	1.11141	1.799517
CALML6	0.97788	0.02212	0.710773	0.619729	1.11141	1.799517
CAMK1G	0.97788	0.02212	0.710773	0.619729	1.11141	1.799517
CD79A	0.97788	0.02212	0.710773	0.619729	1.11141	1.799517
CHRNB2	0.97788	0.02212	0.710773	0.619729	1.11141	1.799517
CPXCR1	0.97788	0.02212	0.710773	0.619729	1.11141	1.799517
CRYAA	0.97788	0.02212	0.710773	0.619729	1.11141	1.799517
DIRAS2	0.97788	0.02212	0.710773	0.619729	1.11141	1.799517
DNM1P41	0.97788	0.02212	0.710773	0.619729	1.11141	1.799517
DPEP2	0.97788	0.02212	0.710773	0.619729	1.11141	1.799517
ELOVL2-A	0.97788	0.02212	0.710773	0.619729	1.11141	1.799517
EMILIN3	0.97788	0.02212	0.710773	0.619729	1.11141	1.799517
EML5	0.97788	0.02212	0.710773	0.619729	1.11141	1.799517
ENPEP	0.97788	0.02212	0.710773	0.619729	1.11141	1.799517
EPHA3	0.97788	0.02212	0.710773	0.619729	1.11141	1.799517
FABP5P3	0.97788	0.02212	0.710773	0.619729	1.11141	1.799517
FAM13C	0.97788	0.02212	0.710773	0.619729	1.11141	1.799517
FAM151B	0.97788	0.02212	0.710773	0.619729	1.11141	1.799517
FAM201A	0.97788	0.02212	0.710773	0.619729	1.11141	1.799517
GPHA2	0.97788	0.02212	0.710773	0.619729	1.11141	1.799517
HCN4	0.97788	0.02212	0.710773	0.619729	1.11141	1.799517
HLF	0.97788	0.02212	0.710773	0.619729	1.11141	1.799517
HPCA	0.97788	0.02212	0.710773	0.619729	1.11141	1.799517
IGSF22	0.97788	0.02212	0.710773	0.619729	1.11141	1.799517
IRX6	0.97788	0.02212	0.710773	0.619729	1.11141	1.799517
KCNA6	0.97788	0.02212	0.710773	0.619729	1.11141	1.799517
KLK9	0.97788	0.02212	0.710773	0.619729	1.11141	1.799517
LINC0046	0.97788	0.02212	0.710773	0.619729	1.11141	1.799517
LINC0047	0.97788	0.02212	0.710773	0.619729	1.11141	1.799517
LINC0052	0.97788	0.02212	0.710773	0.619729	1.11141	1.799517
LINC0063	0.97788	0.02212	0.710773	0.619729	1.11141	1.799517
LINC0093	0.97788	0.02212	0.710773	0.619729	1.11141	1.799517
LINC0101	0.97788	0.02212	0.710773	0.619729	1.11141	1.799517
LINC0109	0.97788	0.02212	0.710773	0.619729	1.11141	1.799517
LINC0154	0.97788	0.02212	0.710773	0.619729	1.11141	1.799517
LINC0155	0.97788	0.02212	0.710773	0.619729	1.11141	1.799517
LINC0161	0.97788	0.02212	0.710773	0.619729	1.11141	1.799517
LOC10012	0.97788	0.02212	0.710773	0.619729	1.11141	1.799517
LOC10050	0.97788	0.02212	0.710773	0.619729	1.11141	1.799517
LOC10246	0.97788	0.02212	0.710773	0.619729	1.11141	1.799517
LOC10537	0.97788	0.02212	0.710773	0.619729	1.11141	1.799517
LOC15117	0.97788	0.02212	0.710773	0.619729	1.11141	1.799517
LPAR5	0.97788	0.02212	0.710773	0.619729	1.11141	1.799517
MEIS2	0.97788	0.02212	0.710773	0.619729	1.11141	1.799517
MMRN2	0.97788	0.02212	0.710773	0.619729	1.11141	1.799517
NCRNA00	0.97788	0.02212	0.710773	0.619729	1.11141	1.799517
NR2E3	0.97788	0.02212	0.710773	0.619729	1.11141	1.799517
NTN3	0.97788	0.02212	0.710773	0.619729	1.11141	1.799517
OR2S2	0.97788	0.02212	0.710773	0.619729	1.11141	1.799517
OVCH1	0.97788	0.02212	0.710773	0.619729	1.11141	1.799517
PCDHB7	0.97788	0.02212	0.710773	0.619729	1.11141	1.799517
PKHD1	0.97788	0.02212	0.710773	0.619729	1.11141	1.799517

PLA2G2C	0.97788	0.02212	0.710773	0.619729	1.11141	1.799517
RBP5	0.97788	0.02212	0.710773	0.619729	1.11141	1.799517
RNU6ATA	0.97788	0.02212	0.710773	0.619729	1.11141	1.799517
SCARNA2	0.97788	0.02212	0.710773	0.619729	1.11141	1.799517
SH3GL1P	0.97788	0.02212	0.710773	0.619729	1.11141	1.799517
SLC1A7	0.97788	0.02212	0.710773	0.619729	1.11141	1.799517
SNORA61	0.97788	0.02212	0.710773	0.619729	1.11141	1.799517
TCEAL7	0.97788	0.02212	0.710773	0.619729	1.11141	1.799517
TCHH	0.97788	0.02212	0.710773	0.619729	1.11141	1.799517
UMODL1	0.97788	0.02212	0.710773	0.619729	1.11141	1.799517
UNC5B-AS	0.97788	0.02212	0.710773	0.619729	1.11141	1.799517
WNT2	0.97788	0.02212	0.710773	0.619729	1.11141	1.799517
WT1-AS	0.97788	0.02212	0.710773	0.619729	1.11141	1.799517
ZBTB46-A	0.97788	0.02212	0.710773	0.619729	1.11141	1.799517
ZNF214	0.97788	0.02212	0.710773	0.619729	1.11141	1.799517
ZNF705E	0.97788	0.02212	0.710773	0.619729	1.11141	1.799517
MEX3C	0.977881	0.022119	1.283396	1.283667	765.7612	596.5398
PSPC1	0.977887	0.022113	1.290212	1.290461	856.8968	664.0216
FBXL2	0.977892	0.022108	1.212562	1.213163	244.5101	201.5459
TAL1	0.977894	0.022106	1.101694	1.102865	55.57048	50.38647
OXCT2P1	0.977896	0.022104	0.889396	0.868439	2.600698	2.996195
FAM227A	0.977897	0.022103	1.106295	1.107473	56.94863	51.42119
SLC35G1	0.977901	0.022099	1.114503	1.115672	62.23894	55.78501
ARL17B	0.977902	0.022098	1.025381	1.025839	32.99775	32.16636
ZNF891	0.977904	0.022096	1.038592	1.039252	34.59818	33.29106
LINC0153	0.977906	0.022094	1.028843	1.02935	33.34229	32.3913
HDGFRP2	0.977906	0.022094	0.769347	0.769215	761.3156	989.7341
CXorf51B	0.977914	0.022086	1.514963	1.843472	1.667114	0.899758
LINC0101	0.977915	0.022085	0.948593	0.947824	35.58733	37.54691
LAX1	0.977918	0.022082	0.852351	0.821411	2.222819	2.708272
RHBG	0.977918	0.022082	0.852351	0.821411	2.222819	2.708272
SNTN	0.977919	0.022081	0.8513	0.820037	2.211705	2.699275
LINC0099	0.977923	0.022077	0.713471	0.622826	1.11141	1.790519
CT55	0.977926	0.022074	1.178487	1.233931	2.222819	1.799517
CXorf57	0.977926	0.022074	1.178487	1.233931	2.222819	1.799517
GPAT2	0.977926	0.022074	1.178487	1.233931	2.222819	1.799517
LOC91450	0.977926	0.022074	1.178487	1.233931	2.222819	1.799517
SLC22A18	0.977926	0.022074	1.178487	1.233931	2.222819	1.799517
LINC0050	0.977931	0.022069	0.814795	0.814453	248.3111	304.8831
UBA5	0.977936	0.022064	0.780239	0.78007	579.0444	742.3006
ABCB4	0.977937	0.022063	0.85469	0.824139	2.222819	2.699275
ACRBP	0.977937	0.022063	0.85469	0.824139	2.222819	2.699275
ACY3	0.977937	0.022063	0.85469	0.824139	2.222819	2.699275
ALDH3A1	0.977937	0.022063	0.85469	0.824139	2.222819	2.699275
C10orf91	0.977937	0.022063	0.85469	0.824139	2.222819	2.699275
C8orf48	0.977937	0.022063	0.85469	0.824139	2.222819	2.699275
CD160	0.977937	0.022063	0.85469	0.824139	2.222819	2.699275
CLC	0.977937	0.022063	0.85469	0.824139	2.222819	2.699275
EXOC3L2	0.977937	0.022063	0.85469	0.824139	2.222819	2.699275
FAM228A	0.977937	0.022063	0.85469	0.824139	2.222819	2.699275
FBXL22	0.977937	0.022063	0.85469	0.824139	2.222819	2.699275
GCGR	0.977937	0.022063	0.85469	0.824139	2.222819	2.699275
GNA15	0.977937	0.022063	0.85469	0.824139	2.222819	2.699275
GPC3	0.977937	0.022063	0.85469	0.824139	2.222819	2.699275
GPC5	0.977937	0.022063	0.85469	0.824139	2.222819	2.699275
IRGM	0.977937	0.022063	0.85469	0.824139	2.222819	2.699275

IZUMO1	0.977937	0.022063	0.85469	0.824139	2.222819	2.699275
KLK15	0.977937	0.022063	0.85469	0.824139	2.222819	2.699275
LINC0113(	0.977937	0.022063	0.85469	0.824139	2.222819	2.699275
LOC10012	0.977937	0.022063	0.85469	0.824139	2.222819	2.699275
LOC10050(	0.977937	0.022063	0.85469	0.824139	2.222819	2.699275
LOC10272	0.977937	0.022063	0.85469	0.824139	2.222819	2.699275
NANOS3	0.977937	0.022063	0.85469	0.824139	2.222819	2.699275
NDUFV2-/	0.977937	0.022063	0.85469	0.824139	2.222819	2.699275
NEBL-AS1	0.977937	0.022063	0.85469	0.824139	2.222819	2.699275
NOXRED1	0.977937	0.022063	0.85469	0.824139	2.222819	2.699275
NR2E1	0.977937	0.022063	0.85469	0.824139	2.222819	2.699275
PIP5K1B	0.977937	0.022063	0.85469	0.824139	2.222819	2.699275
PRAMEF1	0.977937	0.022063	0.85469	0.824139	2.222819	2.699275
RDH16	0.977937	0.022063	0.85469	0.824139	2.222819	2.699275
RGAG1	0.977937	0.022063	0.85469	0.824139	2.222819	2.699275
SCARNA1	0.977937	0.022063	0.85469	0.824139	2.222819	2.699275
SCRT1	0.977937	0.022063	0.85469	0.824139	2.222819	2.699275
WFDC12	0.977937	0.022063	0.85469	0.824139	2.222819	2.699275
KRR1	0.977938	0.022062	0.768844	0.768713	774.6525	1007.729
MYL6B	0.977941	0.022059	0.75662	0.756522	1071.399	1416.22
PIK3C3	0.977944	0.022056	0.779051	0.778889	614.6095	789.088
C20orf196	0.97795	0.02205	1.104031	1.105186	56.68189	51.28622
LINC0051	0.977953	0.022047	0.859111	0.829588	2.245047	2.708272
BRICD5	0.977956	0.022044	0.995134	0.995049	32.23088	32.3913
LINC0009	0.977956	0.022044	0.857041	0.826885	2.222819	2.690277
CUEDC2	0.977967	0.022033	0.768814	0.768685	800.2149	1041.02
LOC10013	0.97797	0.02203	0.884691	0.883842	68.37392	77.36122
HECTD2-/	0.97797	0.02203	1.224639	1.295352	2.33396	1.799517
HRH4	0.977971	0.022029	0.720116	0.632013	1.133638	1.799517
ERC2	0.977971	0.022029	0.885169	0.884328	70.0188	79.17873
C19orf24	0.977978	0.022022	0.849343	0.848758	124.4779	146.6606
LOC10193	0.977983	0.022017	1.057866	1.058754	40.01074	37.78985
TAX1BP3	0.977997	0.022003	1.31026	1.31045	1242.656	948.2643
TBC1D3P	0.977998	0.022002	0.713258	0.632882	1.300349	2.060447
ASIC3	0.978003	0.021997	1.053623	1.054451	38.89934	36.89009
HOXA9	0.978003	0.021997	1.053623	1.054451	38.89934	36.89009
DMRT1	0.978007	0.021993	1.085897	1.086984	48.90202	44.98792
HOXA4	0.978007	0.021993	1.085897	1.086984	48.90202	44.98792
NDUFS7	0.978015	0.021985	0.789329	0.789127	467.9034	592.9407
ZSCAN23	0.978019	0.021981	1.001512	1.001538	33.34229	33.29106
RNF44	0.978025	0.021975	1.366399	1.366491	3100.833	2269.19
PMS2P1	0.978028	0.021972	0.785178	0.784995	534.3324	680.6852
LINC0016	0.978033	0.021967	0.875027	0.848752	2.289504	2.699275
RPAIN	0.978037	0.021963	0.791473	0.791262	445.6752	563.2487
NCMAP	0.978037	0.021963	0.839763	0.83926	152.2631	181.4273
KLHL21	0.978038	0.021962	1.308705	1.3089	1184.763	905.1569
GTF2IP4	0.978054	0.021946	1.355485	1.529789	1.767141	1.151691
EIF4E2	0.97806	0.02194	1.313455	1.313637	1284.789	978.0373
TXN	0.978062	0.021938	1.408438	1.408489	6423.947	4560.875
LOC10309	0.978065	0.021935	1.113514	1.114622	65.02857	58.34033
LRRC37A	0.978067	0.021933	0.933313	0.922875	3.412027	3.698007
INO80	0.978069	0.021931	0.782805	0.782631	553.482	707.21
GOLGA6L	0.978074	0.021926	0.885196	0.861059	2.322846	2.699275
CYP51A1	0.978074	0.021926	0.712901	0.712871	3851.034	5402.149
IKZF2	0.978077	0.021923	0.80876	0.808466	298.9692	369.8007
LINC0148	0.978079	0.021921	0.964631	0.964093	35.56511	36.89009

HIST2H3A	0.978082	0.021918	0.590756	0.41607	0.555705	1.349637
HIST2H3C	0.978082	0.021918	0.590756	0.41607	0.555705	1.349637
DOK3	0.978091	0.021909	1.00756	1.007686	34.4537	34.19082
GLUD2	0.978092	0.021908	1.181194	1.234027	2.400645	1.943478
PCDHB15	0.978106	0.021894	0.936066	0.925862	3.356457	3.626026
SLC45A3	0.978106	0.021894	1.226383	1.226877	326.7544	266.3285
UBE3D	0.978107	0.021893	1.205048	1.205669	226.7276	188.0495
ALYREF	0.978109	0.021891	0.756052	0.755957	1111.41	1470.205
KBTBD2	0.978111	0.021889	1.328522	1.328672	1659.335	1248.865
ANKRD62	0.97812	0.02188	0.936792	0.926627	3.334229	3.599033
C1orf162	0.97812	0.02188	0.936792	0.926627	3.334229	3.599033
CEP83-AS	0.97812	0.02188	0.936792	0.926627	3.334229	3.599033
NOTCH4	0.97812	0.02188	0.936792	0.926627	3.334229	3.599033
NPPC	0.97812	0.02188	0.936792	0.926627	3.334229	3.599033
DSCC1	0.978124	0.021876	0.79244	0.792228	441.2296	556.9504
SRSF1	0.978125	0.021875	0.711449	0.711421	4235.582	5953.701
CCBE1	0.978126	0.021874	0.69379	0.598884	1.11141	1.8625
SLC2A11	0.97813	0.02187	0.814913	0.814591	271.1839	332.9106
LOC10050	0.97813	0.02187	1.013299	1.013519	35.56511	35.09057
NTF4	0.97813	0.02187	1.013299	1.013519	35.56511	35.09057
TFAP4	0.978132	0.021868	0.847577	0.847025	133.3691	157.4577
ST8SIA4	0.978132	0.021868	0.945373	0.93661	3.378685	3.608031
LOC10334	0.978143	0.021857	0.841872	0.841373	154.4859	183.6137
LOC10050	0.978151	0.021849	1.000462	1.000536	3.600967	3.599033
FAIM	0.978156	0.021844	0.843314	0.842797	145.5947	172.7536
LOC20218	0.978156	0.021844	1.007728	1.007853	35.42062	35.14456
CXorf49	0.978156	0.021844	1.642583	2.438488	1.11141	0.449879
CXorf49B	0.978156	0.021844	1.642583	2.438488	1.11141	0.449879
TP63	0.978161	0.021839	0.970992	0.970547	36.67652	37.78985
PDE7B	0.978164	0.021836	0.971282	0.970841	36.68763	37.78985
FLJ41200	0.978166	0.021834	0.883863	0.883067	73.37526	83.09268
SPN	0.978167	0.021833	1.132203	1.160523	3.134175	2.699275
CCSER1	0.978168	0.021832	0.933078	0.932264	44.45638	47.68719
NKX2-8	0.978184	0.021816	0.970984	0.970547	36.67652	37.78985
SNCB	0.978184	0.021816	0.970984	0.970547	36.67652	37.78985
ZNF485	0.978189	0.021811	0.909135	0.908273	55.57048	61.18356
LINC0100	0.978193	0.021807	0.939555	0.938789	42.23356	44.98792
SNHG19	0.978203	0.021797	0.849782	0.849231	134.4806	158.3575
CD300LG	0.978205	0.021795	1.102311	1.122862	3.234202	2.879227
ZNF888	0.978209	0.021791	1.228006	1.228489	330.4999	269.0277
LINC0120	0.978214	0.021786	0.915462	0.897203	2.356188	2.627294
RAG1	0.978215	0.021785	0.933064	0.932264	44.45638	47.68719
PGAP1	0.978229	0.021771	1.248856	1.249235	475.6833	380.7777
TTC21A	0.978231	0.021769	0.92726	0.926438	46.6792	50.38647
GAL3ST1	0.978232	0.021768	0.868425	0.867737	94.46981	108.8708
TUBGCP4	0.978235	0.021765	1.314644	1.314818	1377.036	1047.319
MICAL1	0.978239	0.021761	1.18874	1.189443	181.9377	152.9589
RGL1	0.978244	0.021756	1.206422	1.207019	237.8417	197.0471
ZNF563	0.978246	0.021754	1.028908	1.029352	38.89934	37.78985
ZXDA	0.978246	0.021754	0.866537	0.865873	101.0605	116.7166
TNRC6C	0.978251	0.021749	0.854576	0.853997	124.4779	145.7608
LOC10192	0.978253	0.021747	0.975432	0.975067	37.32113	38.27572
PRNP	0.978255	0.021745	1.381439	1.381512	4041.085	2925.114
CLCN7	0.978255	0.021745	0.752491	0.752406	1247.002	1657.355
LOC11323	0.978257	0.021743	1.15425	1.155154	112.2524	97.1739
B3GNT9	0.978259	0.021741	1.150343	1.151267	106.6953	92.67511

FGF5	0.97826	0.02174	1.173948	1.227899	2.245047	1.826509
SSTR2	0.978262	0.021738	1.12042	1.121442	76.68726	68.38163
PBK	0.978267	0.021733	1.306535	1.306728	1178.094	901.5578
ACR	0.978267	0.021733	1.177924	1.233931	2.222819	1.799517
ACSBG1	0.978267	0.021733	1.177924	1.233931	2.222819	1.799517
BAALC	0.978267	0.021733	1.177924	1.233931	2.222819	1.799517
CASP1P2	0.978267	0.021733	1.177924	1.233931	2.222819	1.799517
CCDC168	0.978267	0.021733	1.177924	1.233931	2.222819	1.799517
CD3E	0.978267	0.021733	1.177924	1.233931	2.222819	1.799517
CLEC3B	0.978267	0.021733	1.177924	1.233931	2.222819	1.799517
FAM26D	0.978267	0.021733	1.177924	1.233931	2.222819	1.799517
FGF17	0.978267	0.021733	1.177924	1.233931	2.222819	1.799517
FOXA3	0.978267	0.021733	1.177924	1.233931	2.222819	1.799517
GPR55	0.978267	0.021733	1.177924	1.233931	2.222819	1.799517
GREM2	0.978267	0.021733	1.177924	1.233931	2.222819	1.799517
HTR3A	0.978267	0.021733	1.177924	1.233931	2.222819	1.799517
IL9R	0.978267	0.021733	1.177924	1.233931	2.222819	1.799517
ITPR1-AS	0.978267	0.021733	1.177924	1.233931	2.222819	1.799517
KCNJ10	0.978267	0.021733	1.177924	1.233931	2.222819	1.799517
LINC0037	0.978267	0.021733	1.177924	1.233931	2.222819	1.799517
LINC0063	0.978267	0.021733	1.177924	1.233931	2.222819	1.799517
LINC0065	0.978267	0.021733	1.177924	1.233931	2.222819	1.799517
LINC0092	0.978267	0.021733	1.177924	1.233931	2.222819	1.799517
LINC0095	0.978267	0.021733	1.177924	1.233931	2.222819	1.799517
LINC0151	0.978267	0.021733	1.177924	1.233931	2.222819	1.799517
LINC0151	0.978267	0.021733	1.177924	1.233931	2.222819	1.799517
LOC10013	0.978267	0.021733	1.177924	1.233931	2.222819	1.799517
LOC10028	0.978267	0.021733	1.177924	1.233931	2.222819	1.799517
LOC10049	0.978267	0.021733	1.177924	1.233931	2.222819	1.799517
LOC10192	0.978267	0.021733	1.177924	1.233931	2.222819	1.799517
LOC28357	0.978267	0.021733	1.177924	1.233931	2.222819	1.799517
LOC38878	0.978267	0.021733	1.177924	1.233931	2.222819	1.799517
MFAP4	0.978267	0.021733	1.177924	1.233931	2.222819	1.799517
MKX-AS1	0.978267	0.021733	1.177924	1.233931	2.222819	1.799517
MYL3	0.978267	0.021733	1.177924	1.233931	2.222819	1.799517
NINJ2	0.978267	0.021733	1.177924	1.233931	2.222819	1.799517
NKX2-1-A	0.978267	0.021733	1.177924	1.233931	2.222819	1.799517
PLCG1-AS	0.978267	0.021733	1.177924	1.233931	2.222819	1.799517
PRSS35	0.978267	0.021733	1.177924	1.233931	2.222819	1.799517
PTGER3	0.978267	0.021733	1.177924	1.233931	2.222819	1.799517
QRFPR	0.978267	0.021733	1.177924	1.233931	2.222819	1.799517
RAPGEF4	0.978267	0.021733	1.177924	1.233931	2.222819	1.799517
RNF212B	0.978267	0.021733	1.177924	1.233931	2.222819	1.799517
S100A7	0.978267	0.021733	1.177924	1.233931	2.222819	1.799517
SLC5A2	0.978267	0.021733	1.177924	1.233931	2.222819	1.799517
UROC1	0.978267	0.021733	1.177924	1.233931	2.222819	1.799517
WWTR1-A	0.978267	0.021733	1.177924	1.233931	2.222819	1.799517
SIM1	0.978275	0.021725	1.033634	1.034138	40.01074	38.68961
PPP1R26-	0.978277	0.021723	1.033443	1.033945	40.02186	38.7076
EML2-AS1	0.97828	0.02172	1.139363	1.140332	93.36952	81.87801
GLUD1P7	0.978283	0.021717	1.173486	1.227826	2.222819	1.808514
LINC0154	0.978283	0.021717	1.173486	1.227826	2.222819	1.808514
CT47A12	0.978287	0.021713	0.757488	0.681149	1.222551	1.799517
CT47A2	0.978287	0.021713	0.757488	0.681149	1.222551	1.799517
CT47A4	0.978287	0.021713	0.757488	0.681149	1.222551	1.799517
CT47A5	0.978287	0.021713	0.757488	0.681149	1.222551	1.799517

CT47A6	0.978287	0.021713	0.757488	0.681149	1.222551	1.799517
CT47A8	0.978287	0.021713	0.757488	0.681149	1.222551	1.799517
VPS33A	0.978288	0.021712	0.794337	0.794122	432.3383	544.4258
ARFIP2	0.978289	0.021711	1.298077	1.298292	1029.165	792.7051
IL1RL2	0.978289	0.021711	1.19514	1.195797	202.2765	169.1546
LOC10140	0.978301	0.021699	0.704122	0.61062	1.11141	1.826509
C3orf17	0.978302	0.021698	0.755687	0.755595	1150.309	1522.391
ZNF43	0.978302	0.021698	0.9714	0.970988	38.38809	39.53538
MAGEE1	0.978305	0.021695	1.133688	1.134672	87.80136	77.37921
ECHDC2	0.97831	0.02169	1.132502	1.133489	86.68995	76.47946
FSD1L	0.978313	0.021687	1.108883	1.1099	68.90739	62.08332
TRIM3	0.978327	0.021673	1.207188	1.207771	244.5101	202.4456
NAA16	0.978328	0.021672	1.202664	1.203264	236.0189	196.1473
THEM6	0.978342	0.021658	0.775671	0.775528	706.8565	911.4552
RAVER2	0.978342	0.021658	0.775437	0.775294	691.2968	891.6605
TMEM198	0.978348	0.021652	1.099112	1.10011	63.35035	57.58453
EID2	0.978351	0.021649	0.80999	0.809702	304.5262	376.099
LINC0111	0.978355	0.021645	1.042476	1.043074	42.23356	40.48912
CCDC69	0.978361	0.021639	0.808738	0.80846	323.5758	400.2395
TBX3	0.978362	0.021638	1.05435	1.055081	45.56779	43.1884
ADRA1A	0.978366	0.021634	1.147545	1.192427	2.222819	1.8625
HSPB11	0.978378	0.021622	1.301759	1.301962	1105.853	849.3718
CCDC62	0.97838	0.02162	0.855165	0.824139	2.222819	2.699275
CFAP58-A	0.97838	0.02162	0.855165	0.824139	2.222819	2.699275
CFAP73	0.97838	0.02162	0.855165	0.824139	2.222819	2.699275
DOC2A	0.97838	0.02162	0.855165	0.824139	2.222819	2.699275
DRAIC	0.97838	0.02162	0.855165	0.824139	2.222819	2.699275
HOXA-AS	0.97838	0.02162	0.855165	0.824139	2.222819	2.699275
LINC0136	0.97838	0.02162	0.855165	0.824139	2.222819	2.699275
LL22NC03	0.97838	0.02162	0.855165	0.824139	2.222819	2.699275
LOC10012	0.97838	0.02162	0.855165	0.824139	2.222819	2.699275
LOC10192	0.97838	0.02162	0.855165	0.824139	2.222819	2.699275
SEMA5A	0.97838	0.02162	0.855165	0.824139	2.222819	2.699275
SUMO4	0.97838	0.02162	0.855165	0.824139	2.222819	2.699275
TDRD12	0.97838	0.02162	0.855165	0.824139	2.222819	2.699275
TENM4	0.97838	0.02162	0.855165	0.824139	2.222819	2.699275
TTC22	0.978386	0.021614	1.068091	1.068935	50.01343	46.78743
POMK	0.978387	0.021613	0.938593	0.937874	45.56779	48.58695
DNAJC12	0.978388	0.021612	1.071206	1.072072	51.12484	47.68719
ECD	0.978397	0.021603	0.758328	0.758232	1084.736	1430.616
NT5M	0.978399	0.021601	1.005341	1.005419	38.89934	38.68961
KCTD7	0.978403	0.021597	0.861809	0.861193	110.0295	127.7657
ST7-OT3	0.978405	0.021595	0.86496	0.836318	2.27839	2.726268
SUGP2	0.978409	0.021591	1.361648	1.361741	3071.625	2255.658
ZNF138	0.978417	0.021583	1.209356	1.20991	265.6269	219.541
AUH	0.978417	0.021583	0.843776	0.843291	157.8202	187.1497
NACA2	0.978417	0.021583	0.859868	0.82965	2.222819	2.68128
TSHZ1	0.978421	0.021579	1.101203	1.102189	64.46176	58.48429
GHDC	0.978422	0.021578	1.226625	1.227096	335.6457	273.5265
C20orf166	0.978428	0.021572	0.712074	0.619729	1.11141	1.799517
CALB2	0.978428	0.021572	0.712074	0.619729	1.11141	1.799517
DLX2-AS1	0.978428	0.021572	0.712074	0.619729	1.11141	1.799517
DNALI1	0.978428	0.021572	0.712074	0.619729	1.11141	1.799517
DRICH1	0.978428	0.021572	0.712074	0.619729	1.11141	1.799517
ELAVL3	0.978428	0.021572	0.712074	0.619729	1.11141	1.799517
ENO4	0.978428	0.021572	0.712074	0.619729	1.11141	1.799517

FOXH1	0.978428	0.021572	0.712074	0.619729	1.11141	1.799517
FTH1P18	0.978428	0.021572	0.712074	0.619729	1.11141	1.799517
GPR17	0.978428	0.021572	0.712074	0.619729	1.11141	1.799517
KLF3-AS1	0.978428	0.021572	0.712074	0.619729	1.11141	1.799517
LOC10050	0.978428	0.021572	0.712074	0.619729	1.11141	1.799517
LOC10192	0.978428	0.021572	0.712074	0.619729	1.11141	1.799517
PSCA	0.978428	0.021572	0.712074	0.619729	1.11141	1.799517
PTPRN	0.978428	0.021572	0.712074	0.619729	1.11141	1.799517
PTPRVP	0.978428	0.021572	0.712074	0.619729	1.11141	1.799517
SCARNA9	0.978428	0.021572	0.712074	0.619729	1.11141	1.799517
SNORA5A	0.978428	0.021572	0.712074	0.619729	1.11141	1.799517
TAF7L	0.978428	0.021572	0.712074	0.619729	1.11141	1.799517
TCTEX1D	0.978428	0.021572	0.712074	0.619729	1.11141	1.799517
TDRD6	0.978428	0.021572	0.712074	0.619729	1.11141	1.799517
TMC1	0.978428	0.021572	0.712074	0.619729	1.11141	1.799517
TMPRSS9	0.978428	0.021572	0.712074	0.619729	1.11141	1.799517
TSSK3	0.978428	0.021572	0.712074	0.619729	1.11141	1.799517
USH1C	0.978428	0.021572	0.712074	0.619729	1.11141	1.799517
ZNF730	0.978428	0.021572	0.712074	0.619729	1.11141	1.799517
MAD2L1B	0.97844	0.02156	0.773623	0.773487	733.5303	948.3453
IL17RB	0.978449	0.021551	1.082603	1.08352	55.57048	51.28622
RHD	0.978471	0.021529	0.875436	0.848752	2.289504	2.699275
BOLA1	0.978472	0.021528	0.838457	0.838006	171.1571	204.2451
FAM157C	0.978479	0.021521	0.963039	0.954044	2.222819	2.330374
CTR9	0.97848	0.02152	0.753604	0.753519	1244.779	1651.956
LOC10192	0.978481	0.021519	0.870271	0.869614	97.80404	112.4698
DMD	0.978484	0.021516	0.774017	0.773882	751.3129	970.8392
ZNF10	0.978491	0.021509	0.903466	0.902683	63.35035	70.18115
PVRIG2P	0.978494	0.021506	0.907431	0.906645	60.83856	67.10397
RCCD1	0.978494	0.021506	0.823851	0.823494	231.1732	280.7246
BCAS4	0.978494	0.021506	1.221822	1.222311	315.6403	258.2306
PMS2P3	0.978498	0.021502	0.890576	0.889825	73.80871	82.94872
ZNF8	0.9785	0.0215	1.200944	1.201533	237.8417	197.9468
DCTN6	0.978508	0.021492	1.238187	1.238599	407.8873	329.3115
ICAM1	0.97851	0.02149	1.417863	1.417906	7971.03	5621.69
ZSCAN12	0.978512	0.021488	1.214064	1.214583	290.4002	239.0928
ZNF677	0.978515	0.021485	0.721375	0.632013	1.133638	1.799517
CNTN4	0.978517	0.021483	1.375964	1.611358	1.455947	0.899758
ABCA9	0.978519	0.021481	0.727114	0.640879	1.16698	1.826509
AGO2	0.978531	0.021469	1.307773	1.307956	1279.232	978.0373
SELT	0.978532	0.021468	1.348199	1.34831	2425.096	1798.617
C19orf68	0.978534	0.021466	1.208936	1.209488	261.1813	215.942
SLC39A10	0.97854	0.02146	1.343455	1.343571	2301.729	1713.14
GUK1	0.978541	0.021459	1.328592	1.328732	1800.484	1355.036
ZNF347	0.978552	0.021448	0.826087	0.825725	230.3063	278.9161
CRTAM	0.978554	0.021446	1.03333	1.042191	2.222819	2.132427
TP73	0.978559	0.021441	1.024635	1.024973	43.34497	42.28864
SRGAP2B	0.978564	0.021436	1.355177	1.355276	2807.221	2071.325
PLRG1	0.978565	0.021435	0.748596	0.748521	1442.61	1927.282
LINC0116	0.978573	0.021427	1.02495	1.025289	42.98932	41.92874
LINC0134	0.97858	0.02142	0.887054	0.886326	77.29854	87.21357
FRY-AS1	0.978581	0.021419	1.10563	1.138074	2.100564	1.844505
EHD1	0.978582	0.021418	1.344886	1.345	2369.525	1761.727
DERL3	0.978595	0.021405	0.881382	0.880686	85.57854	97.1739
TUFT1	0.978598	0.021402	1.401493	1.401546	6050.536	4317.04
GXYLT2	0.978598	0.021402	0.812709	0.812419	298.9692	368.0011

PCDHGA1	0.978619	0.021381	1.107851	1.141801	2.056108	1.799517
PIP5KL1	0.978619	0.021381	0.901751	0.901005	68.90739	76.47946
FAM195A	0.97862	0.02138	0.793737	0.793534	461.235	581.2439
RPP14	0.978626	0.021374	0.766787	0.766674	920.2471	1200.314
SYNRG	0.978627	0.021373	0.768044	0.767927	867.1662	1129.233
HACL1	0.978635	0.021365	1.202572	1.203138	250.0672	207.8442
TSTD3	0.978649	0.021351	1.038499	1.038994	46.90148	45.14087
ZNF682	0.978649	0.021351	0.973413	0.967717	2.611813	2.699275
SCX	0.978651	0.021349	1.037107	1.037586	46.6792	44.98792
MRPL39	0.978652	0.021348	0.76314	0.763037	1014.717	1329.843
GIMAP1	0.97866	0.02134	0.816634	0.758626	1.35592	1.790519
RIN2	0.97866	0.02134	1.241137	1.241527	437.8954	352.7053
SNHG10	0.978666	0.021334	1.138409	1.139276	105.5839	92.67511
ZNF321P	0.978667	0.021333	0.91235	0.911605	61.13864	67.06798
HDAC9	0.978672	0.021328	1.331868	1.332002	1881.616	1412.621
DEPDC7	0.978677	0.021323	0.867905	0.8673	110.0295	126.8659
MFSD8	0.978685	0.021315	0.833252	0.832852	197.8309	237.5362
MTRF1	0.978693	0.021307	0.846287	0.845807	154.4859	182.6509
FAXC	0.978695	0.021305	1.163368	1.164126	145.5947	125.0664
ZNF35	0.978702	0.021298	1.164382	1.165134	147.8175	126.8659
LOC10192	0.978702	0.021298	1.003577	1.003625	43.34497	43.1884
DEPTOR	0.978703	0.021297	0.832836	0.832445	206.7222	248.3333
PHF12	0.978707	0.021293	0.780659	0.780506	639.0605	818.7801
FAM73B	0.978709	0.021291	1.254739	1.255069	562.3732	448.0796
NTN1	0.978711	0.021289	1.305695	1.30588	1232.553	943.8465
ZNF488	0.978723	0.021277	0.899089	0.898363	71.13021	79.17873
MIS18BP1	0.978724	0.021276	1.300245	1.300442	1130.304	869.1665
LINC0113	0.978727	0.021273	0.931862	0.931186	54.45907	58.48429
FNIP2	0.978733	0.021267	1.284071	1.284309	872.4565	679.3175
RNF208	0.978733	0.021267	0.896614	0.895895	73.35303	81.87801
TBX20	0.978736	0.021264	0.988338	0.988188	44.45638	44.98792
ATP6V1G	0.978736	0.021264	1.177123	1.233931	2.222819	1.799517
C5orf67	0.978736	0.021264	1.177123	1.233931	2.222819	1.799517
CLCN1	0.978736	0.021264	1.177123	1.233931	2.222819	1.799517
CYP2D6	0.978736	0.021264	1.177123	1.233931	2.222819	1.799517
EFCAB12	0.978736	0.021264	1.177123	1.233931	2.222819	1.799517
ENKUR	0.978736	0.021264	1.177123	1.233931	2.222819	1.799517
ETV1	0.978736	0.021264	1.177123	1.233931	2.222819	1.799517
FAM223A	0.978736	0.021264	1.177123	1.233931	2.222819	1.799517
FAM223B	0.978736	0.021264	1.177123	1.233931	2.222819	1.799517
HCG4B	0.978736	0.021264	1.177123	1.233931	2.222819	1.799517
KCNK7	0.978736	0.021264	1.177123	1.233931	2.222819	1.799517
LOC10050	0.978736	0.021264	1.177123	1.233931	2.222819	1.799517
PXT1	0.978736	0.021264	1.177123	1.233931	2.222819	1.799517
RAB37	0.978736	0.021264	1.177123	1.233931	2.222819	1.799517
SPATA41	0.978736	0.021264	1.177123	1.233931	2.222819	1.799517
SRD5A2	0.978736	0.021264	1.177123	1.233931	2.222819	1.799517
TAS2R31	0.978736	0.021264	1.177123	1.233931	2.222819	1.799517
TBX18	0.978736	0.021264	1.177123	1.233931	2.222819	1.799517
TEX12	0.978736	0.021264	1.177123	1.233931	2.222819	1.799517
TMEM253	0.978736	0.021264	1.177123	1.233931	2.222819	1.799517
USP3-AS1	0.978736	0.021264	1.177123	1.233931	2.222819	1.799517
VAC14-AS	0.978736	0.021264	1.177123	1.233931	2.222819	1.799517
SLC27A5	0.97874	0.02126	1.11381	1.114705	82.24431	73.78018
COX10-AS	0.978745	0.021255	1.112323	1.113218	81.1329	72.88042
LOC10050	0.978748	0.021252	1.176903	1.233925	2.211705	1.790519

H2AFY2	0.978756	0.021244	0.799366	0.799145	413.4444	517.361
SDPR	0.978761	0.021239	1.170338	1.171051	162.2658	138.5628
SLC35D2	0.978763	0.021237	1.246101	1.24646	495.6887	397.6752
OTX1	0.978766	0.021234	0.846621	0.846142	152.2631	179.9517
RUVBL1	0.978768	0.021232	0.730909	0.730863	2402.868	3287.717
SRGAP2C	0.978771	0.021229	1.26347	1.263767	651.086	515.1926
TBC1D17	0.978776	0.021224	1.311217	1.311387	1397.042	1065.314
ZNF222	0.978781	0.021219	1.14847	1.149288	118.9208	103.4722
TNFSF12-	0.978783	0.021217	1.095351	1.120965	2.411759	2.150422
IL17RE	0.978784	0.021216	1.140258	1.141103	107.8067	94.47462
PDZK1	0.978792	0.021208	1.077569	1.078363	61.12753	56.68477
SYS1	0.978794	0.021206	0.777641	0.777501	716.6369	921.7214
CLIP3	0.978799	0.021201	0.974251	0.973938	45.56779	46.78743
NDUFC1	0.978801	0.021199	0.775367	0.775233	737.976	951.9443
GABARAF	0.978803	0.021197	1.320742	1.320893	1593.761	1206.576
KLHL25	0.978804	0.021196	0.853581	0.853069	138.9262	162.8563
AGTPBP1	0.978813	0.021187	0.777744	0.777603	696.8538	896.1593
CT47A10	0.978815	0.021185	0.758579	0.681149	1.222551	1.799517
CT47A11	0.978815	0.021185	0.758579	0.681149	1.222551	1.799517
CT47A3	0.978815	0.021185	0.758579	0.681149	1.222551	1.799517
CT47A9	0.978815	0.021185	0.758579	0.681149	1.222551	1.799517
RAPGEFL	0.978818	0.021182	0.882903	0.882241	88.91277	100.7819
ANKRD20	0.978833	0.021167	0.864933	0.822416	1.478175	1.799517
COMT	0.978835	0.021165	0.748563	0.748491	1493.734	1995.664
ARHGEF2	0.978836	0.021164	0.919683	0.918986	60.32731	65.64637
KBTBD8	0.978838	0.021162	0.881438	0.880785	91.13559	103.4722
HOXA5	0.978844	0.021156	1.067011	1.06773	56.68189	53.08574
NPHP3-AC	0.978848	0.021152	0.885332	0.884666	86.80109	98.11864
LNP1	0.978861	0.021139	1.021174	1.021437	47.79061	46.78743
PTPN9	0.978865	0.021135	1.252434	1.252764	555.7048	443.5808
B9D2	0.978868	0.021132	1.080022	1.080813	62.23894	57.58453
ZNF570	0.978872	0.021128	1.089066	1.089895	66.68457	61.18356
TMEM91	0.978883	0.021117	0.997716	0.997687	46.6792	46.78743
LINC0150	0.978885	0.021115	0.966123	0.965733	47.79061	49.48671
KNTC1	0.978885	0.021115	0.768684	0.768568	865.7881	1126.497
SKA1	0.978886	0.021114	1.244983	1.245339	496.7556	398.8898
C6orf132	0.978892	0.021108	1.338505	1.338625	2158.357	1612.367
DCAF12L1	0.978896	0.021104	1.223378	1.223821	357.8739	292.4214
CMIP	0.978906	0.021094	1.299448	1.299642	1143.64	879.9636
VPS72	0.978911	0.021089	0.753364	0.753284	1323.689	1757.228
LINC0113	0.978911	0.021089	1.021179	1.021437	47.79061	46.78743
TSNAX-DI	0.978922	0.021078	0.894847	0.894168	78.8434	88.17631
SLC38A1	0.978927	0.021073	0.667952	0.667946	21512.44	32206.85
SCAMP5	0.97893	0.02107	0.866965	0.866388	112.2524	129.5652
RRN3P3	0.978939	0.021061	1.016822	1.017027	48.59083	47.77717
POC5	0.978939	0.021061	0.829324	0.828965	224.5047	270.8272
OST4	0.978943	0.021057	1.363795	1.363881	3310.889	2427.548
PHF23	0.978947	0.021053	0.783242	0.783088	639.0605	816.0808
ZWILCH	0.978954	0.021046	1.309923	1.310091	1400.376	1068.913
CELSR3	0.978957	0.021043	0.836561	0.836164	195.6081	233.9372
LINC0095	0.97896	0.02104	1.232343	1.232744	414.2557	336.0417
ABCG1	0.978961	0.021039	1.261026	1.261323	644.6176	511.0627
LRG1	0.978968	0.021032	1.234829	1.235224	417.89	338.3091
CHAF1A	0.978969	0.021031	0.767708	0.767597	915.8015	1193.08
SLC20A2	0.978973	0.021027	1.348285	1.348389	2516.231	1866.099
TTPAL	0.978985	0.021015	0.735879	0.735829	2230.643	3031.475

AGK	0.978988	0.021012	0.7736	0.773475	813.5518	1051.817
TTBK2	0.978989	0.021011	1.252581	1.252908	551.2592	439.9818
ICAM3	0.978993	0.021007	1.231492	1.231895	411.2215	333.8103
CCT6P3	0.978996	0.021004	1.024693	1.024984	49.52441	48.31702
PLXND1	0.978999	0.021001	1.362599	1.362685	3350.9	2459.039
VCPKMT	0.979003	0.020997	1.176088	1.176747	178.9369	152.0592
PARP12	0.979003	0.020997	1.291318	1.291527	1045.859	809.7825
GNAI2	0.979004	0.020996	1.350798	1.350898	2683.787	1986.666
BCORL1	0.979011	0.020989	1.286536	1.286758	943.5867	733.303
TOPORS-	0.979012	0.020988	0.906518	0.905848	73.35303	80.97825
ASAP1	0.979013	0.020987	1.394533	1.394588	5631.512	4038.115
OR2A20P	0.979016	0.020984	0.884721	0.884093	94.15862	106.5044
ARRDC1	0.979019	0.020981	1.285639	1.285864	931.3612	724.3054
TNFRSF1	0.979022	0.020978	1.380709	1.623575	1.467061	0.899758
LOC1004	0.979023	0.020977	1.032719	1.033096	51.12484	49.48671
TRPM2-A	0.979023	0.020977	1.032719	1.033096	51.12484	49.48671
SLC41A2	0.979028	0.020972	0.820554	0.820251	281.1866	342.8079
MED13	0.979031	0.020969	0.745464	0.745399	1711.571	2296.183
ZMYND8	0.979039	0.020961	0.773773	0.773647	793.5464	1025.724
RAD17	0.979043	0.020957	0.789624	0.789449	541.2565	685.6158
ELP4	0.979043	0.020957	0.81114	0.810879	333.4229	411.1895
VTI1B	0.97906	0.02094	0.754222	0.754143	1353.697	1795.018
IL27RA	0.979061	0.020939	1.238003	1.238384	435.6726	351.8055
MAP6D1	0.979062	0.020938	0.946265	0.945733	54.45907	57.58453
ARMC8	0.979077	0.020923	0.760006	0.759915	1133.638	1491.799
PIGBOS1	0.979088	0.020912	0.880019	0.879412	98.93768	112.5058
ZNF277	0.979097	0.020903	1.298606	1.298797	1158.089	891.6605
SGMS1	0.979107	0.020893	1.301621	1.301804	1216.993	934.8489
TRIM17	0.979124	0.020876	1.058164	1.058759	60.01612	56.68477
NFX1	0.979125	0.020875	0.758071	0.757984	1200.322	1583.575
C3orf62	0.979129	0.020871	0.877522	0.87693	103.3611	117.8683
FAF1	0.979132	0.020868	0.737126	0.737075	2136.129	2898.121
PARGP1	0.979134	0.020866	0.917667	0.917024	66.87351	72.92541
DDX1	0.979141	0.020859	0.721572	0.721539	3387.576	4694.939
KIF17	0.979147	0.020853	1.049416	1.049937	56.68189	53.9855
MIR924H	0.979149	0.020851	0.899007	0.898361	80.02149	89.07607
NAAA	0.979156	0.020844	1.19431	1.194867	237.5971	198.8466
LOC6449	0.979165	0.020835	0.993384	0.991033	1.578202	1.592572
PDIA3P1	0.979167	0.020833	0.917869	0.917229	66.16221	72.13362
IFI27L1	0.979174	0.020826	1.182877	1.183483	203.388	171.8538
SEPT14	0.979176	0.020824	0.969243	0.968916	51.9584	53.62559
CLUHP3	0.979187	0.020813	0.97989	0.97967	51.12484	52.18598
LMBRD1	0.9792	0.0208	0.785582	0.785426	626.835	798.0856
PLAGL1	0.9792	0.0208	1.315142	1.315296	1532.534	1165.16
LRRC49	0.97921	0.02079	0.850697	0.850249	164.4775	193.448
COMMD5	0.97921	0.02079	1.250088	1.250413	549.0363	439.0821
IKZF5	0.979211	0.020789	0.821588	0.821285	275.6296	335.6098
P4HTM	0.979213	0.020787	0.813579	0.813314	325.643	400.3924
GTF2F2	0.979219	0.020781	1.2987	1.298887	1179.206	907.8561
MPP3	0.97922	0.02078	1.063569	1.064188	62.23894	58.48429
SLC36A1	0.979224	0.020776	0.78687	0.78671	610.1639	775.5917
C11orf52	0.979225	0.020775	0.85093	0.850477	159.165	187.1497
ERMAP	0.979227	0.020773	0.898999	0.898361	80.02149	89.07607
ALKBH7	0.979229	0.020771	0.779115	0.778977	700.188	898.8585
ZNF280B	0.979231	0.020769	0.799721	0.799513	432.3383	540.7547
PPP1R3C	0.979236	0.020764	0.887452	0.886843	93.3584	105.2717

KATNBL1I	0.979249	0.020751	0.475698	0.015412	0	0.638828
HYLS1	0.979252	0.020748	1.137047	1.137808	116.7091	102.5724
MADD	0.979253	0.020747	1.279394	1.279626	896.9075	700.9117
PACSIN1	0.979253	0.020747	0.803342	0.803122	408.9987	509.2632
KIF9	0.979258	0.020742	0.961133	0.960742	54.45907	56.68477
TMEM128	0.979258	0.020742	0.819704	0.819416	297.8578	363.5024
MED30	0.979262	0.020738	0.840363	0.839964	188.9396	224.9396
LOC73010	0.979262	0.020738	1.127546	1.128323	105.5839	93.57486
TNFSF4	0.979263	0.020737	0.996888	0.996854	51.12484	51.28622
MOB2	0.979267	0.020733	1.189541	1.190099	234.5074	197.0471
ADPRM	0.97927	0.02073	1.080111	1.080815	70.0188	64.7826
NME1-NM	0.979281	0.020719	1.199825	1.200344	263.1596	219.2351
GPR39	0.979282	0.020718	1.108109	1.108887	87.80136	79.17873
TVP23C	0.979285	0.020715	1.058919	1.059494	61.84994	58.37632
GFPT2	0.979286	0.020714	1.095499	1.096254	78.91008	71.98066
GAREM2	0.979286	0.020714	1.09721	1.09797	80.02149	72.88042
FBXW5	0.979287	0.020713	1.306322	1.306492	1344.806	1029.324
MSANTD3	0.979289	0.020711	1.249607	1.24993	551.7482	441.4214
NDUFB6	0.979294	0.020706	1.301944	1.302122	1254.781	963.6411
ZDHHC8P	0.979295	0.020705	1.025591	1.025865	54.45907	53.08574
FAM46B	0.979302	0.020698	0.787569	0.787407	590.1585	749.4987
CLEC2D	0.979306	0.020694	0.940813	0.940286	60.1606	63.98181
LINC0150	0.979307	0.020693	0.959436	0.959038	55.57048	57.94443
EXOSC8	0.979308	0.020692	0.781359	0.781217	684.6283	876.3646
CNIH1	0.979317	0.020683	0.741545	0.741488	1892.731	2552.614
FBXL19-A	0.979318	0.020682	0.935333	0.934779	62.23894	66.58211
SUPT7L	0.979319	0.020681	0.770431	0.77032	921.3585	1196.076
LINC0127	0.97932	0.02068	0.954937	0.954505	56.68189	59.38405
CDKL5	0.979323	0.020677	1.183955	1.184545	207.8336	175.4529
CYP2J2	0.979326	0.020674	0.868326	0.867798	123.3665	142.1618
ANKRD20	0.979327	0.020673	1.176371	1.176986	192.285	163.3691
CD27-AS1	0.979346	0.020654	1.086272	1.086991	73.35303	67.48187
WASH1	0.979346	0.020654	0.825781	0.825469	267.683	324.2819
MRPS14	0.979347	0.020653	0.791044	0.790872	547.9249	692.8139
NDEL1	0.979351	0.020649	1.290679	1.290881	1056.951	818.7801
S1PR5	0.979381	0.020619	0.859782	0.8593	142.2604	165.5555
PDE9A	0.979386	0.020614	0.806213	0.805987	392.3276	486.7692
WDR4	0.979386	0.020614	0.806213	0.805987	392.3276	486.7692
C11orf98	0.979387	0.020613	0.767873	0.767768	965.1703	1257.115
SMCO4	0.979387	0.020613	1.201977	1.20247	285.6323	237.5362
APTR	0.979388	0.020612	0.81967	0.819385	295.6349	360.8031
MLH3	0.979388	0.020612	0.786593	0.786438	629.0578	799.8851
RNMTL1	0.979401	0.020599	0.865919	0.86541	130.0349	150.2596
MAGEA10	0.979402	0.020598	1.240735	1.391461	1.255893	0.899758
ECT2	0.979411	0.020589	1.365921	1.365999	3725.767	2727.5
ZNF367	0.979419	0.020581	1.272196	1.272443	797.9921	627.1315
CCDC102	0.979422	0.020578	0.874455	0.873915	115.5866	132.2645
PRR34-AS	0.979422	0.020578	0.874455	0.873915	115.5866	132.2645
TNFSF12	0.979428	0.020572	1.179543	1.258804	1.644886	1.30465
RNF223	0.979429	0.020571	0.992316	0.992236	54.45907	54.88526
SPANXA1	0.979436	0.020564	0.944599	0.92683	1.667114	1.799517
SPANXA2	0.979436	0.020564	0.944599	0.92683	1.667114	1.799517
ZNF230	0.979437	0.020563	1.16958	1.170208	180.0484	153.8587
C11orf1	0.979439	0.020561	0.85864	0.858169	146.7061	170.9541
EML3	0.979467	0.020533	1.260155	1.260435	666.8457	529.0579
CKLF	0.97947	0.02053	1.24621	1.246534	539.0336	432.4238

CCNO	0.97947	0.02053	0.969484	0.969187	56.68189	58.48429
FRAT2	0.979478	0.020522	0.818808	0.818532	305.6376	373.3997
ARHGAP1	0.97948	0.02052	1.256916	1.257205	635.7263	505.6642
TTC33	0.979484	0.020516	1.214862	1.2153	338.9799	278.9251
DUSP23	0.979489	0.020511	1.234862	1.235225	455.6779	368.9009
CASC11	0.97949	0.02051	1.143798	1.232646	1.11141	0.899758
EFCAB1	0.97949	0.02051	1.143798	1.232646	1.11141	0.899758
INHBA-AS	0.97949	0.02051	1.143798	1.232646	1.11141	0.899758
IYD	0.97949	0.02051	1.143798	1.232646	1.11141	0.899758
LINGO3	0.97949	0.02051	1.143798	1.232646	1.11141	0.899758
LOC10027	0.97949	0.02051	1.143798	1.232646	1.11141	0.899758
LOC10192	0.97949	0.02051	1.143798	1.232646	1.11141	0.899758
LOC33987	0.97949	0.02051	1.143798	1.232646	1.11141	0.899758
NOL4	0.97949	0.02051	1.143798	1.232646	1.11141	0.899758
ROPN1	0.97949	0.02051	1.143798	1.232646	1.11141	0.899758
SLC25A30	0.97949	0.02051	1.143798	1.232646	1.11141	0.899758
SLC4A10	0.97949	0.02051	1.143798	1.232646	1.11141	0.899758
SNORA24	0.97949	0.02051	1.143798	1.232646	1.11141	0.899758
TNFRSF1	0.97949	0.02051	1.143798	1.232646	1.11141	0.899758
ZIC2	0.97949	0.02051	1.143798	1.232646	1.11141	0.899758
ADAMTSL	0.979492	0.020508	0.908121	0.907527	80.02149	88.17631
NDUFB11	0.979493	0.020507	0.744191	0.744132	1879.394	2525.622
HSPA7	0.979494	0.020506	1.281248	1.445999	1.422604	0.980737
LOC10192	0.979496	0.020504	0.973476	0.973217	57.7933	59.38405
NRDE2	0.979498	0.020502	1.201346	1.201838	280.0752	233.0374
MITF	0.979505	0.020495	0.769839	0.769731	933.584	1212.874
ASTN2	0.979508	0.020492	0.849411	0.848991	173.2021	204.0112
MSANTD3	0.979511	0.020489	0.824416	0.824115	274.6071	333.2165
FAM69A	0.979512	0.020488	1.272992	1.273234	819.1089	643.3272
EPS15L1	0.979526	0.020474	0.754472	0.754396	1391.485	1844.505
CBFA2T2	0.979532	0.020468	0.752958	0.752885	1449.923	1925.825
AGAP9	0.979547	0.020453	1.034153	1.034488	60.0828	58.0794
HSPBAP1	0.979552	0.020448	0.823788	0.823493	280.0752	340.1086
VAMP2	0.979553	0.020447	1.305101	1.305267	1367.034	1047.319
APOC1	0.979567	0.020433	0.973471	0.973217	57.7933	59.38405
SRGN	0.979567	0.020433	1.13071	1.131418	121.1436	107.0712
WASH3P	0.979576	0.020424	1.199992	1.200483	278.8304	232.2636
CWC27	0.97958	0.02042	0.797395	0.797209	505.6914	634.3296
SMC6	0.979584	0.020416	0.747291	0.747228	1746.024	2336.672
GATSL3	0.979585	0.020415	0.83645	0.836098	221.1705	264.5289
TRIM58	0.979585	0.020415	1.144167	1.144855	136.7256	119.4249
TUSC2	0.979588	0.020412	1.249037	1.249343	590.1585	472.3731
RGMA	0.979589	0.020411	1.063107	1.063662	68.90739	64.7826
TTC32	0.979589	0.020411	1.063107	1.063662	68.90739	64.7826
CDKN1C	0.979589	0.020411	1.147561	1.148232	146.7061	127.7657
TRIM23	0.97959	0.02041	1.209563	1.210015	320.086	264.5289
VWA1	0.97959	0.02041	0.856076	0.855631	157.8202	184.4505
LOC10192	0.979604	0.020396	1.203171	1.330378	1.200322	0.899758
TBC1D4	0.979608	0.020392	1.201221	1.201709	278.9638	232.1376
MGST1	0.979615	0.020385	1.342768	1.342871	2537.348	1889.492
ZXDB	0.979621	0.020379	1.220016	1.22042	385.7369	316.0671
NDRG2	0.979626	0.020374	0.875826	0.875305	118.9208	135.8635
MESDC1	0.979635	0.020365	1.196128	1.196622	275.6296	230.3381
PAX6	0.979646	0.020354	1.19371	1.194219	258.9584	216.8418
ZNF511	0.979649	0.020351	0.798596	0.798407	483.4632	605.5373
MTHFD2L	0.97965	0.02035	0.967011	0.966708	60.01612	62.08332

UTP14A	0.979654	0.020346	0.76873	0.768626	979.7075	1274.625
AMH	0.979654	0.020346	1.080182	1.080816	77.79867	71.98066
CENPC	0.979664	0.020336	1.210076	1.210519	326.7544	269.9275
MLST8	0.979669	0.020331	0.797615	0.797431	510.137	639.7282
RPE	0.979677	0.020323	1.34377	1.34387	2667.383	1984.849
LOC64551	0.979681	0.020319	1.038418	1.038777	63.29478	60.93163
LOC10192	0.979683	0.020317	1.095748	1.096429	87.80136	80.07849
BAIAP2	0.979684	0.020316	0.749661	0.749595	1615.99	2155.821
TM9SF1	0.979686	0.020314	1.225117	1.225498	420.1128	342.8079
LINC0036	0.97969	0.02031	1.117983	1.118688	106.6953	95.37438
EPM2A	0.97969	0.02031	1.114589	1.115293	103.3611	92.67511
SNHG6	0.979697	0.020303	1.268271	1.268517	804.6605	634.3296
SFT2D3	0.9797	0.0203	1.040742	1.041117	65.57317	62.98308
CAMK1	0.979702	0.020298	0.992006	0.991929	58.90471	59.38405
FBXL15	0.979705	0.020295	0.891552	0.890996	97.80404	109.7705
SCAPER	0.979715	0.020285	0.814811	0.81456	337.8685	414.7886
MTMR6	0.979715	0.020285	0.783518	0.78338	709.0793	905.1569
LINC0119	0.979717	0.020283	1.180633	1.293728	1.16698	0.899758
CCDC112	0.97972	0.02028	0.803804	0.8036	449.0095	558.7499
LYSMD3	0.979722	0.020278	1.279288	1.279506	931.3612	727.9045
CCDC146	0.979723	0.020277	0.961084	0.960741	62.23894	64.7826
VPS37D	0.979724	0.020276	1.035082	1.035409	63.35035	61.18356
NFATC3	0.979724	0.020276	0.787737	0.787585	625.7236	794.4866
LOC28373	0.979727	0.020273	1.192695	1.29284	1.433718	1.106703
LOC10192	0.979736	0.020264	1.121331	1.189238	1.200322	1.007729
BORCS6	0.979748	0.020252	0.847128	0.846738	188.9396	223.1401
INHBB	0.979749	0.020251	1.256244	1.256523	655.7317	521.8598
PDIA5	0.979759	0.020241	1.282745	1.282953	1015.828	791.7873
ESYT3	0.979766	0.020234	1.168228	1.168811	195.6081	167.355
VIMP	0.979768	0.020232	1.284324	1.284527	1042.502	811.582
PET100	0.979772	0.020228	1.268655	1.268899	795.7693	627.1315
ZBTB9	0.979782	0.020218	0.813629	0.813388	357.8739	439.9818
RNF181	0.979784	0.020216	0.755518	0.755442	1373.702	1818.412
PDCD2L	0.979787	0.020213	1.268607	1.268851	796.8807	628.0313
HLA-J	0.979796	0.020204	1.201531	1.338553	1.11141	0.827778
DCUN1D2	0.979797	0.020203	1.240949	1.241273	524.6631	422.6795
PAX8	0.979804	0.020196	1.360037	1.360117	3546.508	2607.5
SPRYD4	0.979805	0.020195	0.882831	0.882317	116.698	132.2645
SMARCAD	0.979825	0.020175	0.780752	0.780623	769.0954	985.2353
ENKD1	0.979825	0.020175	1.017087	1.017246	62.23894	61.18356
PLA2G6	0.97984	0.02016	0.950586	0.950185	66.68457	70.18115
SH2D2A	0.979842	0.020158	0.897945	0.8974	94.46981	105.2717
ZNF846	0.979846	0.020154	0.978091	0.977895	63.35035	64.7826
FAM66A	0.979856	0.020144	1.150582	1.244862	1.122524	0.899758
EXOC1	0.979858	0.020142	0.76806	0.767961	1024.72	1334.342
MED27	0.979864	0.020136	1.261947	1.262205	727.9733	576.7451
SRRM3	0.979868	0.020132	0.863251	0.862807	154.4859	179.0519
LOC40095	0.979868	0.020132	0.986992	0.980514	1.11141	1.133695
BRPF3	0.979872	0.020128	1.312399	1.312544	1603.764	1221.872
PRSS21	0.979875	0.020125	1.220796	1.221188	386.7705	316.7149
NAA35	0.979879	0.020121	1.286218	1.286415	1061.396	825.0784
AGO1	0.979882	0.020118	0.790764	0.790608	614.6095	777.3912
MREG	0.979883	0.020117	1.228066	1.228429	439.9737	358.1578
SNAPC5	0.979887	0.020113	0.892128	0.891605	106.6953	119.6679
ABCA13	0.979888	0.020112	1.143069	1.232646	1.11141	0.899758
ALS2CR1	0.979888	0.020112	1.143069	1.232646	1.11141	0.899758

ANKRD36	0.979888	0.020112	1.143069	1.232646	1.11141	0.899758
C11orf86	0.979888	0.020112	1.143069	1.232646	1.11141	0.899758
C14orf178	0.979888	0.020112	1.143069	1.232646	1.11141	0.899758
C5orf60	0.979888	0.020112	1.143069	1.232646	1.11141	0.899758
C5orf66-A	0.979888	0.020112	1.143069	1.232646	1.11141	0.899758
CACNA1E	0.979888	0.020112	1.143069	1.232646	1.11141	0.899758
CAMKV	0.979888	0.020112	1.143069	1.232646	1.11141	0.899758
CCDC184	0.979888	0.020112	1.143069	1.232646	1.11141	0.899758
CD244	0.979888	0.020112	1.143069	1.232646	1.11141	0.899758
CD300C	0.979888	0.020112	1.143069	1.232646	1.11141	0.899758
CECR7	0.979888	0.020112	1.143069	1.232646	1.11141	0.899758
CFAP52	0.979888	0.020112	1.143069	1.232646	1.11141	0.899758
CFLAR-AS	0.979888	0.020112	1.143069	1.232646	1.11141	0.899758
CHGB	0.979888	0.020112	1.143069	1.232646	1.11141	0.899758
CHRNA7	0.979888	0.020112	1.143069	1.232646	1.11141	0.899758
CLEC4M	0.979888	0.020112	1.143069	1.232646	1.11141	0.899758
COL5A3	0.979888	0.020112	1.143069	1.232646	1.11141	0.899758
COMP	0.979888	0.020112	1.143069	1.232646	1.11141	0.899758
CORIN	0.979888	0.020112	1.143069	1.232646	1.11141	0.899758
CPN2	0.979888	0.020112	1.143069	1.232646	1.11141	0.899758
CPNE9	0.979888	0.020112	1.143069	1.232646	1.11141	0.899758
CRYM	0.979888	0.020112	1.143069	1.232646	1.11141	0.899758
CTNNA3	0.979888	0.020112	1.143069	1.232646	1.11141	0.899758
DCDC2B	0.979888	0.020112	1.143069	1.232646	1.11141	0.899758
DPP6	0.979888	0.020112	1.143069	1.232646	1.11141	0.899758
DPY19L2F	0.979888	0.020112	1.143069	1.232646	1.11141	0.899758
DRD4	0.979888	0.020112	1.143069	1.232646	1.11141	0.899758
DRD5	0.979888	0.020112	1.143069	1.232646	1.11141	0.899758
ERAS	0.979888	0.020112	1.143069	1.232646	1.11141	0.899758
EWSAT1	0.979888	0.020112	1.143069	1.232646	1.11141	0.899758
EXOC3L1	0.979888	0.020112	1.143069	1.232646	1.11141	0.899758
FAM159A	0.979888	0.020112	1.143069	1.232646	1.11141	0.899758
FAM166A	0.979888	0.020112	1.143069	1.232646	1.11141	0.899758
FAM186A	0.979888	0.020112	1.143069	1.232646	1.11141	0.899758
FAM83C	0.979888	0.020112	1.143069	1.232646	1.11141	0.899758
FGR	0.979888	0.020112	1.143069	1.232646	1.11141	0.899758
FITM1	0.979888	0.020112	1.143069	1.232646	1.11141	0.899758
FMR1-AS1	0.979888	0.020112	1.143069	1.232646	1.11141	0.899758
GAS8-AS1	0.979888	0.020112	1.143069	1.232646	1.11141	0.899758
GPR1-AS	0.979888	0.020112	1.143069	1.232646	1.11141	0.899758
GRAP2	0.979888	0.020112	1.143069	1.232646	1.11141	0.899758
GSN-AS1	0.979888	0.020112	1.143069	1.232646	1.11141	0.899758
HBM	0.979888	0.020112	1.143069	1.232646	1.11141	0.899758
HFE2	0.979888	0.020112	1.143069	1.232646	1.11141	0.899758
HIST1H2A	0.979888	0.020112	1.143069	1.232646	1.11141	0.899758
HIST1H4D	0.979888	0.020112	1.143069	1.232646	1.11141	0.899758
HMGCLL1	0.979888	0.020112	1.143069	1.232646	1.11141	0.899758
HSD3BP4	0.979888	0.020112	1.143069	1.232646	1.11141	0.899758
HTR6	0.979888	0.020112	1.143069	1.232646	1.11141	0.899758
IFIT1B	0.979888	0.020112	1.143069	1.232646	1.11141	0.899758
IGLON5	0.979888	0.020112	1.143069	1.232646	1.11141	0.899758
IL1RAPL2	0.979888	0.020112	1.143069	1.232646	1.11141	0.899758
IRX4	0.979888	0.020112	1.143069	1.232646	1.11141	0.899758
IRX5	0.979888	0.020112	1.143069	1.232646	1.11141	0.899758
ISLR2	0.979888	0.020112	1.143069	1.232646	1.11141	0.899758
KCNH1	0.979888	0.020112	1.143069	1.232646	1.11141	0.899758

KCNIP2-A	0.979888	0.020112	1.143069	1.232646	1.11141	0.899758
KIAA2012	0.979888	0.020112	1.143069	1.232646	1.11141	0.899758
KLF1	0.979888	0.020112	1.143069	1.232646	1.11141	0.899758
KLHL38	0.979888	0.020112	1.143069	1.232646	1.11141	0.899758
KRT39	0.979888	0.020112	1.143069	1.232646	1.11141	0.899758
LDLRAD2	0.979888	0.020112	1.143069	1.232646	1.11141	0.899758
LEMD1-AS	0.979888	0.020112	1.143069	1.232646	1.11141	0.899758
LINC0010	0.979888	0.020112	1.143069	1.232646	1.11141	0.899758
LINC0053	0.979888	0.020112	1.143069	1.232646	1.11141	0.899758
LINC0087	0.979888	0.020112	1.143069	1.232646	1.11141	0.899758
LINC0088	0.979888	0.020112	1.143069	1.232646	1.11141	0.899758
LINC0092	0.979888	0.020112	1.143069	1.232646	1.11141	0.899758
LINC0120	0.979888	0.020112	1.143069	1.232646	1.11141	0.899758
LINC0125	0.979888	0.020112	1.143069	1.232646	1.11141	0.899758
LINC0150	0.979888	0.020112	1.143069	1.232646	1.11141	0.899758
LINC0152	0.979888	0.020112	1.143069	1.232646	1.11141	0.899758
LOC10012	0.979888	0.020112	1.143069	1.232646	1.11141	0.899758
LOC10028	0.979888	0.020112	1.143069	1.232646	1.11141	0.899758
LOC10192	0.979888	0.020112	1.143069	1.232646	1.11141	0.899758
LOC10192	0.979888	0.020112	1.143069	1.232646	1.11141	0.899758
LOC10192	0.979888	0.020112	1.143069	1.232646	1.11141	0.899758
LOC10192	0.979888	0.020112	1.143069	1.232646	1.11141	0.899758
LOC10192	0.979888	0.020112	1.143069	1.232646	1.11141	0.899758
LOC10192	0.979888	0.020112	1.143069	1.232646	1.11141	0.899758
LOC10192	0.979888	0.020112	1.143069	1.232646	1.11141	0.899758
LOC10272	0.979888	0.020112	1.143069	1.232646	1.11141	0.899758
LOC44089	0.979888	0.020112	1.143069	1.232646	1.11141	0.899758
LRRC43	0.979888	0.020112	1.143069	1.232646	1.11141	0.899758
LRRTM4	0.979888	0.020112	1.143069	1.232646	1.11141	0.899758
LTA	0.979888	0.020112	1.143069	1.232646	1.11141	0.899758
MAP2K4P	0.979888	0.020112	1.143069	1.232646	1.11141	0.899758
MFSD7	0.979888	0.020112	1.143069	1.232646	1.11141	0.899758
MGC4592	0.979888	0.020112	1.143069	1.232646	1.11141	0.899758
MSMB	0.979888	0.020112	1.143069	1.232646	1.11141	0.899758
MTRNR2L	0.979888	0.020112	1.143069	1.232646	1.11141	0.899758
NEU4	0.979888	0.020112	1.143069	1.232646	1.11141	0.899758
NME9	0.979888	0.020112	1.143069	1.232646	1.11141	0.899758
NPAP1	0.979888	0.020112	1.143069	1.232646	1.11141	0.899758
OR1Q1	0.979888	0.020112	1.143069	1.232646	1.11141	0.899758
OR2W3	0.979888	0.020112	1.143069	1.232646	1.11141	0.899758
OR52N2	0.979888	0.020112	1.143069	1.232646	1.11141	0.899758
OVOL1-AS	0.979888	0.020112	1.143069	1.232646	1.11141	0.899758
PARD3-AS	0.979888	0.020112	1.143069	1.232646	1.11141	0.899758
PCDH17	0.979888	0.020112	1.143069	1.232646	1.11141	0.899758
PCDHB18	0.979888	0.020112	1.143069	1.232646	1.11141	0.899758
PGA5	0.979888	0.020112	1.143069	1.232646	1.11141	0.899758
PKD2L1	0.979888	0.020112	1.143069	1.232646	1.11141	0.899758
PLIN1	0.979888	0.020112	1.143069	1.232646	1.11141	0.899758
PMFBP1	0.979888	0.020112	1.143069	1.232646	1.11141	0.899758
PNMA5	0.979888	0.020112	1.143069	1.232646	1.11141	0.899758
POT1-AS1	0.979888	0.020112	1.143069	1.232646	1.11141	0.899758
POU3F1	0.979888	0.020112	1.143069	1.232646	1.11141	0.899758
PP12613	0.979888	0.020112	1.143069	1.232646	1.11141	0.899758
PPIAP30	0.979888	0.020112	1.143069	1.232646	1.11141	0.899758
PRKG1	0.979888	0.020112	1.143069	1.232646	1.11141	0.899758
PROSER2	0.979888	0.020112	1.143069	1.232646	1.11141	0.899758

PYDC1	0.979888	0.020112	1.143069	1.232646	1.11141	0.899758
RBM5-AS:	0.979888	0.020112	1.143069	1.232646	1.11141	0.899758
RGS18	0.979888	0.020112	1.143069	1.232646	1.11141	0.899758
RNF138P:	0.979888	0.020112	1.143069	1.232646	1.11141	0.899758
RNF148	0.979888	0.020112	1.143069	1.232646	1.11141	0.899758
S100A12	0.979888	0.020112	1.143069	1.232646	1.11141	0.899758
SATB1	0.979888	0.020112	1.143069	1.232646	1.11141	0.899758
SCGB1B2	0.979888	0.020112	1.143069	1.232646	1.11141	0.899758
SDR16C5	0.979888	0.020112	1.143069	1.232646	1.11141	0.899758
SERPINA	0.979888	0.020112	1.143069	1.232646	1.11141	0.899758
SETSIP	0.979888	0.020112	1.143069	1.232646	1.11141	0.899758
SIDT1	0.979888	0.020112	1.143069	1.232646	1.11141	0.899758
SNRPD2P	0.979888	0.020112	1.143069	1.232646	1.11141	0.899758
TEPP	0.979888	0.020112	1.143069	1.232646	1.11141	0.899758
TMEM207	0.979888	0.020112	1.143069	1.232646	1.11141	0.899758
TRIM60	0.979888	0.020112	1.143069	1.232646	1.11141	0.899758
TRPM1	0.979888	0.020112	1.143069	1.232646	1.11141	0.899758
TTC36	0.979888	0.020112	1.143069	1.232646	1.11141	0.899758
TTC9B	0.979888	0.020112	1.143069	1.232646	1.11141	0.899758
UCN2	0.979888	0.020112	1.143069	1.232646	1.11141	0.899758
VSTM5	0.979888	0.020112	1.143069	1.232646	1.11141	0.899758
VWA3B	0.979888	0.020112	1.143069	1.232646	1.11141	0.899758
WFDC5	0.979888	0.020112	1.143069	1.232646	1.11141	0.899758
ZAR1	0.979888	0.020112	1.143069	1.232646	1.11141	0.899758
TAS2R14	0.979892	0.020108	1.002791	1.004215	1.11141	1.106703
NEK6	0.979893	0.020107	1.301977	1.302141	1362.588	1046.419
IWS1	0.979894	0.020106	0.760699	0.760615	1241.445	1632.162
LOXL3	0.979895	0.020105	0.988293	0.988187	62.23894	62.98308
RNF207	0.9799	0.0201	1.166846	1.167418	197.6753	169.3255
SHANK2-/	0.979901	0.020099	0.476795	0.015202	0	0.647826
ZNF530	0.979903	0.020097	1.151441	1.152057	164.433	142.7287
RBMXL1	0.979906	0.020094	0.778176	0.778053	804.9828	1034.614
WDR66	0.979914	0.020086	0.884052	0.883542	114.4752	129.5652
ZNF695	0.979915	0.020085	1.181163	1.181692	230.6286	195.1666
LMTK3	0.97992	0.02008	0.876383	0.8759	130.0349	148.4601
KCNRG	0.979928	0.020072	1.171355	1.264148	1.344806	1.061715
PSME4	0.979935	0.020065	1.34684	1.346935	2774.078	2059.547
FAM225A	0.979937	0.020063	1.141934	1.232593	1.089181	0.881763
FBXL6	0.979946	0.020054	0.803895	0.803696	451.2323	561.4492
ZNF652	0.979959	0.020041	0.800972	0.800785	495.7109	619.0337
CEP112	0.979968	0.020032	0.937535	0.93708	73.35303	78.27897
RPS6KB2	0.979974	0.020026	0.777333	0.777215	852.4511	1096.805
C4orf32	0.979983	0.020017	1.178294	1.178819	232.2846	197.0471
EXO5	0.979985	0.020015	0.874188	0.873709	128.9235	147.5604
TBC1D16	0.979987	0.020013	1.377035	1.377098	4822.406	3501.859
MYO9B	0.979996	0.020004	1.358629	1.358709	3460.929	2547.216
STAT6	0.979997	0.020003	1.364528	1.364602	3915.496	2869.329
SEPT7P2	0.980001	0.019999	1.118907	1.119561	115.9756	103.5892
NGDN	0.980007	0.019993	0.81772	0.817475	352.3168	430.9842
ZNF578	0.980008	0.019992	1.049696	1.050105	73.95319	70.42408
RNF43	0.980008	0.019992	1.198963	1.199419	297.8578	248.3333
AGBL5	0.980008	0.019992	0.772237	0.772132	969.1492	1255.163
SMIM10L2	0.980009	0.019991	1.039851	1.040189	71.13021	68.38163
HMGCL	0.980016	0.019984	0.765374	0.765283	1120.301	1463.907
PLEKHG4	0.980017	0.019983	0.903155	0.902634	95.58122	105.8926
LUADT1	0.980039	0.019961	1.105505	1.171563	1.055839	0.899758

CASP9	0.980051	0.019949	0.820055	0.819799	328.9772	401.2922
DNAJC14	0.980067	0.019933	0.772468	0.772362	945.8096	1224.571
B4GALT5	0.980068	0.019932	1.367438	1.367509	4044.419	2957.506
ZFYVE21	0.980076	0.019924	1.27311	1.273332	891.3505	700.012
HOXB13	0.98008	0.01992	1.11217	1.11281	111.141	99.87317
LINC0127	0.980088	0.019912	1.212699	1.213101	365.6538	301.419
FAM160A	0.980093	0.019907	0.819237	0.818987	336.7571	411.1895
HEY1	0.980105	0.019895	0.930221	0.929752	77.79867	83.67752
ZNF20	0.980121	0.019879	1.135146	1.135771	140.0043	123.2669
POLG2	0.980123	0.019877	0.876623	0.87616	135.592	154.7584
SCIMP	0.980142	0.019858	1.095442	1.157026	1.011383	0.872766
MYOF	0.980151	0.019849	1.419428	1.419462	10181.62	7172.873
GPRASP2	0.980159	0.019841	1.134403	1.135023	140.1265	123.4558
BMP3	0.980164	0.019836	0.706269	0.512795	0.43345	0.85477
EGFEM1F	0.980169	0.019831	1.08779	1.144436	1.000269	0.872766
EXOSC1	0.980171	0.019829	0.782329	0.782201	757.9813	969.0397
FZR1	0.980173	0.019827	1.26239	1.262636	767.984	608.2366
C8orf34-A	0.980176	0.019824	0.913018	0.912522	91.13559	99.87317
NNT-AS1	0.980181	0.019819	1.194936	1.195392	291.1893	243.5916
OSGEPL1	0.980186	0.019814	0.898853	0.898359	106.6953	118.7681
MYZAP	0.980192	0.019808	0.975229	0.975028	68.34058	70.09117
CFI	0.980196	0.019804	0.910675	0.910179	93.3584	102.5724
MCF2L-AS	0.9802	0.0198	0.994647	0.994603	68.90739	69.28139
ADAT2	0.980204	0.019796	0.89163	0.89114	112.2524	125.9662
PGGT1B	0.980204	0.019796	0.825787	0.825516	302.3034	366.2016
ZNF71	0.98021	0.01979	0.842916	0.842581	228.9504	271.727
SERPINB9	0.980215	0.019785	0.908452	0.907957	95.58122	105.2717
IFI44L	0.980218	0.019782	1.054041	1.054458	77.79867	73.78018
DDX11L9	0.980219	0.019781	1.14684	1.232857	1.211436	0.980737
PSTK	0.98022	0.01978	0.991508	0.991437	67.79598	68.38163
GID4	0.98022	0.01978	1.244308	1.244602	590.1585	474.1726
CD84	0.980227	0.019773	1.03284	1.053002	0.966926	0.917753
CTHRC1	0.98023	0.01977	0.858976	0.858576	172.2685	200.6461
POP5	0.980231	0.019769	0.812581	0.812363	405.6645	499.3659
SUSD4	0.980235	0.019765	0.894065	0.89358	113.3638	126.8659
ZCRB1	0.980238	0.019762	0.741265	0.741215	2186.143	2949.408
CEP44	0.980244	0.019756	1.213299	1.213688	378.9351	312.2161
BNIP1	0.980255	0.019745	1.218205	1.218577	406.7759	333.8103
RP2	0.980255	0.019745	1.235677	1.235991	538.2557	435.483
HS3ST3A	0.980255	0.019745	1.005417	1.005462	67.6515	67.28393
ASB2	0.980258	0.019742	0.964363	0.964088	71.13021	73.78018
SPATA2L	0.980259	0.019741	0.858506	0.85811	174.4913	203.3454
LINC0155	0.98026	0.01974	0.767432	0.621819	0.555705	0.899758
SPX	0.980266	0.019734	0.976178	0.975988	71.13021	72.88042
CPEB2	0.980272	0.019728	1.161224	1.161775	193.3853	166.4553
USP16	0.980274	0.019726	0.756921	0.756848	1413.713	1867.898
NDUFB1	0.98028	0.01972	0.755918	0.755848	1519.297	2010.06
SNHG20	0.980281	0.019719	0.904629	0.904145	103.25	114.1973
TUBG2	0.980282	0.019718	1.185627	1.186105	262.4816	221.2956
SMG9	0.980291	0.019709	1.218341	1.218711	410.1101	336.5096
C15orf57	0.980291	0.019709	1.167332	1.167865	208.8894	178.863
HADH	0.980296	0.019704	1.329391	1.329502	2288.392	1721.238
ZNF653	0.980303	0.019697	0.924003	0.923545	88.91277	96.27414
DUSP6	0.980306	0.019694	0.867588	0.867165	154.4859	178.1521
RAD51D	0.980327	0.019673	0.898312	0.897835	110.8742	123.4918
INAFM1	0.980328	0.019672	1.111101	1.111698	120.0322	107.971

LRP5L	0.98033	0.01967	1.071835	1.072335	87.80136	81.87801
ZNF469	0.980335	0.019665	0.90137	0.900891	107.8067	119.6679
TTC5	0.980345	0.019655	0.873162	0.872725	144.4832	165.5555
PRRT3-AS	0.980347	0.019653	1.075325	1.075838	90.02418	83.67752
VARS2	0.980363	0.019637	1.218608	1.218972	416.7786	341.9082
CBLC	0.980363	0.019637	1.29483	1.294998	1300.349	1004.13
ZNF812	0.980366	0.019634	1.156953	1.257079	1.133638	0.899758
ADCK4	0.980366	0.019634	0.827728	0.827461	308.9719	373.3997
TRIM56	0.980368	0.019632	1.285704	1.285888	1160.312	902.3406
C16orf87	0.980368	0.019632	0.789355	0.789214	682.4055	864.6677
TRAF6	0.98037	0.01963	1.222552	1.222904	441.2296	360.8031
SNORA59	0.980378	0.019622	1.941296	56.57048	0.555705	0
SNORA59	0.980378	0.019622	1.941296	56.57048	0.555705	0
RTN4R	0.980379	0.019621	1.188679	1.189133	286.7437	241.1352
PTRHD1	0.980381	0.019619	1.139626	1.140203	160.043	140.3623
SMA4	0.980382	0.019618	0.989607	0.989524	72.33054	73.09636
ABCA11P	0.980382	0.019618	0.954828	0.954503	75.57585	79.17873
UBE2E2	0.980383	0.019617	0.776065	0.775957	931.3612	1200.278
BBS5	0.980399	0.019601	0.802555	0.802376	513.4379	639.8991
PPP1R8	0.9804	0.0196	0.775394	0.775287	923.5814	1191.28
GUSBP4	0.980401	0.019599	0.91429	0.913821	95.23669	104.219
ZBTB41	0.980402	0.019598	1.284808	1.284995	1119.189	870.966
DKFZP586	0.980404	0.019596	1.090753	1.091311	101.1383	92.67511
OTUD4	0.980412	0.019588	1.298631	1.298791	1362.588	1049.118
BTF3P11	0.980424	0.019576	1.148974	1.244958	1.11141	0.890761
MED16	0.980428	0.019572	1.218391	1.218755	411.2215	337.4094
DLL3	0.98043	0.01957	0.882771	0.882316	127.8121	144.8611
ANKEF1	0.980432	0.019568	0.824699	0.824443	321.1974	389.5953
ASIC2	0.980432	0.019568	1.142036	1.232646	1.11141	0.899758
CGA	0.980432	0.019568	1.142036	1.232646	1.11141	0.899758
CRTC3-AS	0.980432	0.019568	1.142036	1.232646	1.11141	0.899758
CTNNA2	0.980432	0.019568	1.142036	1.232646	1.11141	0.899758
ERICH3	0.980432	0.019568	1.142036	1.232646	1.11141	0.899758
GJD3	0.980432	0.019568	1.142036	1.232646	1.11141	0.899758
GPBAR1	0.980432	0.019568	1.142036	1.232646	1.11141	0.899758
HAND1	0.980432	0.019568	1.142036	1.232646	1.11141	0.899758
IL36G	0.980432	0.019568	1.142036	1.232646	1.11141	0.899758
KLHL1	0.980432	0.019568	1.142036	1.232646	1.11141	0.899758
KRTAP3-2	0.980432	0.019568	1.142036	1.232646	1.11141	0.899758
KRTAP5-1	0.980432	0.019568	1.142036	1.232646	1.11141	0.899758
LGI3	0.980432	0.019568	1.142036	1.232646	1.11141	0.899758
LINC0005	0.980432	0.019568	1.142036	1.232646	1.11141	0.899758
LINC0103	0.980432	0.019568	1.142036	1.232646	1.11141	0.899758
LOC10013	0.980432	0.019568	1.142036	1.232646	1.11141	0.899758
LOC10192	0.980432	0.019568	1.142036	1.232646	1.11141	0.899758
LOC10192	0.980432	0.019568	1.142036	1.232646	1.11141	0.899758
LOC10192	0.980432	0.019568	1.142036	1.232646	1.11141	0.899758
LOC10192	0.980432	0.019568	1.142036	1.232646	1.11141	0.899758
LOC28605	0.980432	0.019568	1.142036	1.232646	1.11141	0.899758
LOC40079	0.980432	0.019568	1.142036	1.232646	1.11141	0.899758
LOC79160	0.980432	0.019568	1.142036	1.232646	1.11141	0.899758
MEGF10	0.980432	0.019568	1.142036	1.232646	1.11141	0.899758
NTRK1	0.980432	0.019568	1.142036	1.232646	1.11141	0.899758
NTRK3	0.980432	0.019568	1.142036	1.232646	1.11141	0.899758
NUTM2F	0.980432	0.019568	1.142036	1.232646	1.11141	0.899758
NYX	0.980432	0.019568	1.142036	1.232646	1.11141	0.899758

PIP5K1P1	0.980432	0.019568	1.142036	1.232646	1.11141	0.899758
PRSS27	0.980432	0.019568	1.142036	1.232646	1.11141	0.899758
SEZ6	0.980432	0.019568	1.142036	1.232646	1.11141	0.899758
SHISA3	0.980432	0.019568	1.142036	1.232646	1.11141	0.899758
SHISA8	0.980432	0.019568	1.142036	1.232646	1.11141	0.899758
SHOX	0.980432	0.019568	1.142036	1.232646	1.11141	0.899758
SIRPG	0.980432	0.019568	1.142036	1.232646	1.11141	0.899758
SLAMF6	0.980432	0.019568	1.142036	1.232646	1.11141	0.899758
SLC22A14	0.980432	0.019568	1.142036	1.232646	1.11141	0.899758
SLC22A9	0.980432	0.019568	1.142036	1.232646	1.11141	0.899758
SLC46A2	0.980432	0.019568	1.142036	1.232646	1.11141	0.899758
SLC5A4	0.980432	0.019568	1.142036	1.232646	1.11141	0.899758
SLITRK6	0.980432	0.019568	1.142036	1.232646	1.11141	0.899758
SNORA10	0.980432	0.019568	1.142036	1.232646	1.11141	0.899758
SNORA14	0.980432	0.019568	1.142036	1.232646	1.11141	0.899758
SNORA33	0.980432	0.019568	1.142036	1.232646	1.11141	0.899758
SNORA65	0.980432	0.019568	1.142036	1.232646	1.11141	0.899758
SOX15	0.980432	0.019568	1.142036	1.232646	1.11141	0.899758
SSTR5	0.980432	0.019568	1.142036	1.232646	1.11141	0.899758
SSUH2	0.980432	0.019568	1.142036	1.232646	1.11141	0.899758
SYTL5	0.980432	0.019568	1.142036	1.232646	1.11141	0.899758
TACR3	0.980432	0.019568	1.142036	1.232646	1.11141	0.899758
TBC1D28	0.980432	0.019568	1.142036	1.232646	1.11141	0.899758
TDGF1	0.980432	0.019568	1.142036	1.232646	1.11141	0.899758
TEKT2	0.980432	0.019568	1.142036	1.232646	1.11141	0.899758
THSD4-AS	0.980432	0.019568	1.142036	1.232646	1.11141	0.899758
THSD7A	0.980432	0.019568	1.142036	1.232646	1.11141	0.899758
TLR4	0.980432	0.019568	1.142036	1.232646	1.11141	0.899758
TMEM233	0.980432	0.019568	1.142036	1.232646	1.11141	0.899758
TPTE2	0.980432	0.019568	1.142036	1.232646	1.11141	0.899758
TRIM71	0.980432	0.019568	1.142036	1.232646	1.11141	0.899758
UBE2E1-A	0.980432	0.019568	1.142036	1.232646	1.11141	0.899758
UG0898H	0.980432	0.019568	1.142036	1.232646	1.11141	0.899758
UTF1	0.980432	0.019568	1.142036	1.232646	1.11141	0.899758
ZNF295-A	0.980432	0.019568	1.142036	1.232646	1.11141	0.899758
ZSCAN10	0.980432	0.019568	1.142036	1.232646	1.11141	0.899758
ZSWIM5	0.980432	0.019568	1.142036	1.232646	1.11141	0.899758
PLEKHN1	0.980442	0.019558	1.107947	1.108531	116.698	105.2717
SMC2-AS	0.980445	0.019555	1.128409	1.208737	1.11141	0.917753
ABRACL	0.980448	0.019552	1.282352	1.28254	1114.744	869.1665
ACSM3	0.980449	0.019551	0.843612	0.843288	236.7302	280.7246
IFI6	0.98045	0.01955	1.198595	1.199012	331.2001	276.2258
AK5	0.98045	0.01955	1.203744	1.204149	344.537	286.1231
CORO1C	0.98045	0.01955	1.391251	1.3913	6499.523	4671.545
CEP126	0.980458	0.019542	1.116992	1.11758	126.7007	113.3695
ZNF808	0.980464	0.019536	1.215465	1.215829	418.0011	343.7976
QPRT	0.980464	0.019536	1.371153	1.371217	4525.449	3300.313
MTERF1	0.980469	0.019531	1.23492	1.235226	551.2592	446.2801
CITED4	0.98047	0.01953	0.824691	0.824436	323.4202	392.2946
MAPKAPK	0.980476	0.019524	1.226254	1.226587	473.4605	385.9963
C2orf74	0.980483	0.019517	1.029132	1.029355	77.79867	75.5797
TEAD4	0.980483	0.019517	0.818849	0.818619	373.4336	456.1775
TMEM38A	0.980485	0.019515	1.298826	1.298983	1408.045	1083.957
AKAP13	0.980487	0.019513	1.301284	1.301437	1463.726	1124.698
PABPN1	0.980488	0.019512	0.787327	0.787194	727.3842	924.0248
METTL17	0.980489	0.019511	0.80676	0.80657	476.7947	591.1412

EIF3J-AS1	0.98049	0.01951	0.949526	0.949184	81.1329	85.47704
ZNF217	0.980493	0.019507	0.737525	0.737482	2509.563	3402.886
ZIM2-AS1	0.980495	0.019505	1.127119	1.208213	1.089181	0.899758
PAQR4	0.980496	0.019504	1.247476	1.247746	664.6229	532.6569
ADARB1	0.980504	0.019496	0.786521	0.786389	722.4162	918.6532
CGREF1	0.980504	0.019496	1.006434	1.006484	73.35303	72.88042
PPM1N	0.980504	0.019496	1.006434	1.006484	73.35303	72.88042
ZNF543	0.980523	0.019477	0.860345	0.859964	179.1703	208.348
THAP10	0.980526	0.019474	0.836296	0.836004	272.2953	325.7125
C1orf159	0.980542	0.019458	0.873496	0.873077	150.0403	171.8538
QRICH2	0.980546	0.019454	1.145373	1.145928	171.1571	149.3599
RMDN2	0.980552	0.019448	1.009202	1.009273	74.46444	73.78018
ADD3	0.980559	0.019441	0.732857	0.732818	2838.54	3873.46
AURKB	0.980566	0.019434	1.270351	1.270564	919.1357	723.4057
FGFRL1	0.98057	0.01943	1.260308	1.260547	774.6525	614.5349
RDH13	0.980572	0.019428	0.768747	0.768656	1142.529	1486.401
LOC14841	0.980576	0.019424	0.849564	0.849227	220.0591	259.1304
VAMP3	0.980583	0.019417	1.339659	1.339753	2735.179	2041.552
ZNF772	0.980586	0.019414	1.121093	1.121659	139.3041	124.1936
SUPT3H	0.980591	0.019409	0.844404	0.84408	227.839	269.9275
PMPCA	0.980593	0.019407	1.30177	1.30192	1497.069	1149.891
UPK2	0.980597	0.019403	1.011904	1.011995	75.57585	74.67994
ZNHIT3	0.980598	0.019402	1.25068	1.250937	707.9679	565.948
WDR1	0.980599	0.019401	1.409079	1.409116	8772.356	6225.428
TVP23C-C	0.980604	0.019396	1.18713	1.187575	284.4875	239.5517
FBXL3	0.980606	0.019394	0.805293	0.805109	486.7974	604.6376
MCOLN1	0.980608	0.019392	0.797788	0.797627	571.2645	716.2076
FNTB	0.980615	0.019385	0.92154	0.921104	93.20281	101.1868
ARFRP1	0.980625	0.019375	0.807483	0.807295	480.1289	594.7402
LDLRAD3	0.980645	0.019355	0.845486	0.845164	231.1732	273.5265
LOC10106	0.980649	0.019351	0.909343	0.847705	0.700188	0.827778
TBC1D3K	0.980649	0.019351	0.909343	0.847705	0.700188	0.827778
C1GALT1C	0.980651	0.019349	1.237341	1.237634	571.2645	461.576
LRRC37B	0.980652	0.019348	0.90573	0.905281	108.4625	119.8118
AKAP11	0.980652	0.019348	0.761879	0.761801	1335.914	1753.629
BMS1	0.980655	0.019345	0.739939	0.739893	2395.243	3237.285
VPS13A	0.980661	0.019339	1.291106	1.291273	1280.344	991.5337
ZNF669	0.980663	0.019337	1.139783	1.140336	161.8879	141.9639
CMTR1	0.980667	0.019333	0.764268	0.764187	1260.338	1649.257
TCF7L2	0.980672	0.019328	1.229456	1.229772	501.2457	407.5905
WASH7P	0.980676	0.019324	0.816786	0.816568	390.1826	477.8346
LGALS8	0.980684	0.019316	1.33807	1.338166	2701.837	2019.058
MKI67	0.980688	0.019312	1.396218	1.396262	7111.91	5093.532
ADGRL2	0.98069	0.01931	0.752844	0.752782	1696.011	2252.995
HSPH1	0.980696	0.019304	1.405446	1.405485	8340.017	5933.906
PCOLCE	0.980704	0.019296	0.898047	0.897609	121.1436	134.9637
PACRGL	0.980707	0.019293	1.126901	1.12745	151.1517	134.064
MTFR1	0.980713	0.019287	1.295529	1.295687	1381.482	1066.214
LRRC75B	0.980719	0.019281	1.019637	1.019781	78.91008	77.37921
FAM83H-A	0.980723	0.019277	0.83381	0.833538	294.8014	353.677
CMTM8	0.980725	0.019275	0.86793	0.867541	167.8228	193.448
MRM1	0.980732	0.019268	1.042778	1.043079	84.46713	80.97825
SENP6	0.980741	0.019259	0.755652	0.755586	1628.226	2154.921
DCTN3	0.980749	0.019251	1.275371	1.275568	1019.163	798.9854
SLFN12	0.980763	0.019237	1.141872	1.142401	177.8255	155.6582
GLOD4	0.980765	0.019235	0.791157	0.791018	676.8484	855.6701

ANKRA2	0.980766	0.019234	1.142407	1.142935	178.9369	156.5579
NUP88	0.980768	0.019232	0.795145	0.794996	620.1665	780.0905
CBX1	0.980771	0.019229	1.37119	1.371251	4829.075	3521.654
TMEM9	0.98078	0.01922	1.343963	1.34405	3088.607	2297.983
TRIM68	0.980785	0.019215	0.852878	0.852539	211.1678	247.6945
DENND5B	0.980787	0.019213	1.195484	1.195886	337.8685	282.5241
HEYL	0.98079	0.01921	1.024501	1.024677	81.1329	79.17873
MSL3P1	0.980792	0.019208	1.186786	1.187211	302.3034	254.6316
ADSSL1	0.980797	0.019203	1.111157	1.111699	130.0349	116.9686
FOCAD	0.980805	0.019195	1.30036	1.300506	1549.305	1191.307
IKZF4	0.980812	0.019188	1.117467	1.11801	137.8148	123.2669
MORC3	0.980813	0.019187	1.289007	1.289173	1301.461	1009.529
ABCB9	0.980822	0.019178	0.90182	0.901392	120.0322	133.1642
LOC64340	0.98083	0.01917	0.833068	0.8328	295.6572	355.0176
HAUS4	0.980833	0.019167	0.84234	0.84204	252.29	299.6195
ZNF680	0.980842	0.019158	1.206121	1.206488	392.8944	325.6495
TRNP1	0.980846	0.019154	1.288864	1.28903	1304.795	1012.228
MRPS30	0.98086	0.01914	0.800078	0.799917	565.7075	707.21
DNAJA3	0.980861	0.019139	0.743247	0.743199	2246.159	3022.288
UBTD1	0.980863	0.019137	1.250515	1.250764	715.7478	572.2463
FKBP11	0.980867	0.019133	1.134952	1.135483	164.4886	144.8611
LYSMD4	0.980867	0.019133	0.960994	0.96074	85.57854	89.07607
TIMM23B	0.980869	0.019131	1.177705	1.178154	265.6936	225.5154
RNF31	0.980882	0.019118	0.812691	0.812493	437.8954	538.9552
C3orf18	0.980888	0.019112	1.060161	1.060545	94.46981	89.07607
HAUS5	0.980892	0.019108	0.795616	0.795468	625.7458	786.6407
RTN2	0.980908	0.019092	1.158634	1.159119	220.0591	189.849
E2F6	0.980912	0.019088	0.8165	0.816293	420.1128	514.6618
DNAH1	0.980914	0.019086	1.063605	1.064004	96.69263	90.87559
FAM161B	0.980914	0.019086	1.063605	1.064004	96.69263	90.87559
PPP2R3B	0.980916	0.019084	1.20764	1.208002	394.5504	326.6123
CD109	0.980918	0.019082	1.324042	1.324151	2283.947	1724.837
ZNF785	0.980922	0.019078	0.956975	0.956703	87.80136	91.77535
SAP30BP	0.980928	0.019072	0.793646	0.793504	656.8431	827.7776
TAT	0.980934	0.019066	0.977949	0.963882	0.866899	0.899758
TIMM13	0.980941	0.019059	0.785576	0.785454	787.9894	1003.231
RBM6	0.980942	0.019058	0.792903	0.792763	669.0686	843.9733
FAM220A	0.980947	0.019053	0.800048	0.79989	590.1585	737.8018
ACTRT3	0.980947	0.019053	1.068526	1.068943	100.0269	93.57486
SNX25	0.980948	0.019052	1.227018	1.227325	517.9169	421.9866
RAB11B	0.980952	0.019048	1.322871	1.322982	2195.034	1659.154
SOWAHC	0.980953	0.019047	1.329899	1.33	2519.599	1894.432
KBTBD7	0.980954	0.019046	0.969037	0.968833	83.66691	86.3588
MIR210HG	0.980956	0.019044	1.017921	1.018046	83.35572	81.87801
CLN3	0.980958	0.019042	0.779111	0.779004	916.9351	1177.064
C19orf60	0.980959	0.019041	0.840933	0.840648	272.2953	323.913
TM2D2	0.980971	0.019029	1.266296	1.266503	944.6981	745.9086
ZNF551	0.980974	0.019026	0.853531	0.853207	223.3933	261.8297
MPP2	0.980987	0.019013	0.829419	0.829171	324.5316	391.3949
PLEKHM1	0.980992	0.019008	0.908335	0.907921	114.6197	126.2451
SS18L2	0.980994	0.019006	1.198192	1.198571	363.4309	303.2185
FAM76B	0.980996	0.019004	0.834966	0.834703	303.4148	363.5024
CEP72	0.981008	0.018992	0.924309	0.92392	102.2497	110.6703
RGL2	0.981012	0.018988	1.283706	1.283878	1202.545	936.6484
SMIM1	0.981034	0.018966	1.084383	1.084847	112.2524	103.4722
LINC0112	0.981044	0.018956	1.181867	1.182286	297.8578	251.9323

ANTXR1	0.98105	0.01895	1.249801	1.250042	750.2015	600.1388
SNX21	0.981055	0.018945	0.875843	0.875462	162.2658	185.3502
PLPP3	0.981061	0.018939	0.834799	0.834539	307.8605	368.9009
LIPA	0.981062	0.018938	0.784075	0.783959	830.223	1059.016
C9orf9	0.981062	0.018938	0.969191	0.968993	84.37822	87.07861
DNAJC4	0.981069	0.018931	0.850027	0.849719	240.0645	282.5241
IRF9	0.981072	0.018928	0.869407	0.869041	176.7141	203.3454
TMED8	0.98108	0.01892	0.937805	0.937458	94.46981	100.7729
ZNF662	0.981086	0.018914	0.97749	0.977343	87.14563	89.16605
RASA4B	0.981093	0.018907	0.817285	0.817082	426.5034	521.9858
PGP	0.981095	0.018905	0.802036	0.801875	563.4847	702.7112
XAGE1E	0.9811	0.0189	0.559798	0.021745	0	0.449879
ZNF555	0.981101	0.018899	0.888535	0.88814	145.7169	164.0709
KBTBD4	0.981103	0.018897	0.864014	0.863664	191.1624	221.3405
CHST12	0.981121	0.018879	1.108493	1.108994	136.7034	123.2669
KIF18A	0.981127	0.018873	1.243205	1.243462	671.2914	539.855
CHCHD4	0.98113	0.01887	0.797255	0.797108	626.835	786.3888
TMLHE-AS	0.981132	0.018868	1.103549	1.230116	0.555705	0.449879
MSLN	0.981143	0.018857	1.224365	1.224668	515.694	421.0869
RNF216P1	0.981153	0.018847	0.868419	0.868061	181.9822	209.6437
SERF1B	0.981164	0.018836	0.837662	0.837395	290.0779	346.4069
TMEM238	0.981164	0.018836	0.911914	0.911522	118.9208	130.465
RBFOX2	0.981165	0.018835	1.370054	1.370114	4865.751	3551.346
STK33	0.981166	0.018834	0.988264	0.988186	84.46713	85.47704
COA5	0.981169	0.018831	0.830038	0.829798	341.2027	411.1895
MTURN	0.981174	0.018826	0.833667	0.833414	311.1947	373.3997
DLGAP1-A	0.981174	0.018826	0.966517	0.96631	90.81328	93.97975
IRF7	0.981174	0.018826	0.922059	0.921679	107.8067	116.9686
ISCA2	0.981185	0.018815	0.808094	0.80792	519.0283	642.4274
CCNK	0.981193	0.018807	1.30386	1.303993	1728.242	1325.344
C2orf47	0.981196	0.018804	0.824546	0.824322	367.8766	446.2801
HSD11B1L	0.981197	0.018803	0.980671	0.980547	85.57854	87.27656
ARHGAP1	0.981198	0.018802	0.767732	0.76765	1246.579	1623.893
MORC4	0.981199	0.018801	0.816189	0.815992	443.4524	543.454
TBC1D3I	0.981211	0.018789	0.91003	0.847705	0.700188	0.827778
ZNF438	0.981221	0.018779	0.904623	0.904229	124.4779	137.663
ZBTB46	0.981222	0.018778	1.168428	1.168868	254.5128	217.7415
CYB5R4	0.981224	0.018776	0.832082	0.831838	332.3115	399.4927
ABCA7	0.981229	0.018771	0.822174	0.821961	397.8846	484.07
TSPAN14	0.981232	0.018768	1.351249	1.351324	3601.845	2665.417
DDAH1	0.981241	0.018759	1.366007	1.366069	4711.265	3448.774
NUDT8	0.981243	0.018757	0.915706	0.915322	114.4752	125.0664
MPP5	0.981245	0.018755	1.330634	1.330731	2586.25	1943.478
SKIV2L	0.981246	0.018754	1.326006	1.326108	2465.106	1858.901
ESCO1	0.981255	0.018745	0.792524	0.792391	707.9679	893.46
SHROOM	0.981256	0.018744	1.254675	1.254898	831.6789	662.744
LPAR3	0.981261	0.018739	0.948776	0.948486	95.58122	100.7729
CDR2L	0.981264	0.018736	1.315453	1.315569	2038.325	1549.384
ESCO2	0.981264	0.018736	0.820662	0.820452	400.1074	487.669
NFIC	0.981267	0.018733	1.278285	1.278461	1150.309	899.7583
ARL16	0.981267	0.018733	1.254278	1.254503	805.7719	642.3015
OR4F17	0.981271	0.018729	1.283363	1.640962	0.744644	0.449879
OR4F4	0.981271	0.018729	1.283363	1.640962	0.744644	0.449879
ZNF595	0.981271	0.018729	0.956276	0.956022	95.59234	99.99014
WWTR1	0.981273	0.018727	1.321778	1.321885	2255.05	1705.933
IFT140	0.98128	0.01872	0.880941	0.880567	160.043	181.7512

P2RY6	0.981284	0.018716	1.008298	1.008351	88.91277	88.17631
GAREM1	0.981286	0.018714	0.842633	0.842359	281.1866	333.8103
MPP1	0.981296	0.018704	0.845259	0.844975	262.2927	310.4166
WISP2	0.981313	0.018687	1.044917	1.045191	97.80404	93.57486
ATG16L2	0.981314	0.018686	1.110393	1.110871	148.9289	134.064
C12orf73	0.981316	0.018684	0.868076	0.867731	188.9396	217.7415
ARMC6	0.981318	0.018682	0.772293	0.772205	1165.869	1509.794
LOC72775	0.981324	0.018676	1.148451	1.148918	207.878	180.9324
TAF8	0.981329	0.018671	0.841558	0.84129	288.4219	342.8349
CDYL	0.981339	0.018661	0.759667	0.759601	1554.862	2046.95
ANKRD36	0.981353	0.018647	1.111972	1.112451	148.0398	133.0743
MAPKAPK	0.98136	0.01864	0.802295	0.802141	584.6014	728.8042
INAFM2	0.981364	0.018636	1.20056	1.200913	388.9934	323.913
DUSP18	0.98137	0.01863	0.929171	0.928823	107.8067	116.0688
SSX2IP	0.981372	0.018628	0.763438	0.763366	1458.169	1910.187
CEP83	0.981373	0.018627	0.857075	0.856765	228.9504	267.2282
TMBIM4	0.981375	0.018625	0.797758	0.797617	662.4001	830.4769
UBXN2A	0.98139	0.01861	1.242127	1.242374	709.0793	570.7437
CNTRL	0.981393	0.018607	1.189381	1.189756	348.9826	293.3212
ZNF440	0.981393	0.018607	0.866796	0.866459	195.1413	225.2185
PANK2	0.981396	0.018604	0.82217	0.821961	397.8846	484.07
HN1	0.981397	0.018603	1.350696	1.35077	3742.116	2770.356
CBX2	0.981399	0.018601	0.786559	0.786443	825.7773	1050.018
PRKAG1	0.981406	0.018594	0.761337	0.761268	1504.849	1976.769
SASS6	0.981413	0.018587	0.834319	0.834074	321.1974	385.0966
CCDC90B	0.981416	0.018584	0.781865	0.78176	920.7695	1177.82
RIOK2	0.981418	0.018582	0.780298	0.780196	954.7008	1223.671
PPOX	0.981418	0.018582	0.851402	0.85111	251.1786	295.1207
PLCB4	0.981422	0.018578	1.232699	1.232968	606.8296	492.1678
DNAJC27	0.981434	0.018566	1.108711	1.109179	146.7061	132.2645
USP6NL	0.981439	0.018561	0.821235	0.82103	407.8873	496.8015
TRMU	0.98144	0.01856	1.212088	1.212404	472.3491	389.5953
LOC65227	0.981444	0.018556	0.934053	0.933724	106.6953	114.2693
TCTN3	0.981457	0.018543	0.765434	0.76536	1404.822	1835.507
NBPF15	0.981459	0.018541	1.274804	1.27498	1154.254	905.3098
PPIL1	0.981476	0.018524	0.768342	0.768263	1278.121	1663.653
TOB2	0.981478	0.018522	1.310596	1.310715	1910.513	1457.608
TMEM110	0.981479	0.018521	1.278928	1.279096	1231.442	962.7414
ZNF219	0.981494	0.018506	0.918211	0.917852	118.9208	129.5652
SNHG9	0.981498	0.018502	1.082785	1.083196	126.7007	116.9686
TOMM40L	0.981499	0.018501	0.812785	0.812607	497.9115	612.7354
ZNF432	0.981499	0.018501	1.163337	1.163764	253.4014	217.7415
CCDC127	0.981503	0.018497	1.038746	1.038976	100.0269	96.27414
TRIM52	0.981503	0.018497	1.127032	1.127499	174.4913	154.7584
C12orf65	0.981504	0.018496	0.822808	0.822601	400.6076	487.0032
ACADS	0.981506	0.018494	1.065948	1.066304	112.2524	105.2717
ERVMER3	0.981519	0.018481	0.904204	0.903834	133.3691	147.5604
ALKBH1	0.981521	0.018479	0.835252	0.835009	322.3088	385.9963
UTP11L	0.981545	0.018455	0.768247	0.768168	1293.936	1684.447
MMAA	0.981546	0.018454	0.955216	0.954973	102.2497	107.0712
STRN3	0.981546	0.018454	1.256034	1.256246	863.5652	687.4153
PRR14L	0.98155	0.01845	0.758254	0.758192	1660.446	2190.012
FAM226A	0.98156	0.01844	1.102795	1.230116	0.555705	0.449879
FAM226B	0.98156	0.01844	1.102795	1.230116	0.555705	0.449879
LOC10192	0.98156	0.01844	1.102795	1.230116	0.555705	0.449879
TRIM36	0.981562	0.018438	0.838445	0.838197	316.7517	377.8985

TRPM7	0.981564	0.018436	1.281511	1.281674	1262.372	984.9384
TARS2	0.981566	0.018434	0.787542	0.787428	851.2619	1081.069
USPL1	0.981566	0.018434	1.189667	1.190033	351.2054	295.1207
PALM	0.981577	0.018423	1.247334	1.247562	786.878	630.7306
RDM1	0.981587	0.018413	1.077978	1.07837	122.2551	113.3695
MARC2	0.981601	0.018399	1.143503	1.14395	208.945	182.6509
PLEKHH3	0.981602	0.018398	1.216572	1.216868	515.694	423.7862
C20orf96	0.981603	0.018397	1.108086	1.108533	155.5973	140.3623
GOLGA2F	0.981606	0.018394	0.563006	0.021745	0	0.449879
GOLGA2F	0.981606	0.018394	0.563006	0.021745	0	0.449879
OR2A1	0.981606	0.018394	0.563006	0.021745	0	0.449879
OR2A42	0.981606	0.018394	0.563006	0.021745	0	0.449879
XAGE1B	0.981606	0.018394	0.563006	0.021745	0	0.449879
CACFD1	0.98161	0.01839	1.197345	1.197691	390.1048	325.7125
MDM2	0.981613	0.018387	0.770937	0.770855	1224.54	1588.55
ZNF225	0.981622	0.018378	0.997267	0.997251	97.80404	98.07366
ZNF585B	0.981623	0.018377	1.050974	1.051256	110.2518	104.8758
TRMT44	0.981625	0.018375	1.029185	1.029356	100.0269	97.1739
YTHDF2	0.981635	0.018365	0.74675	0.746702	2327.292	3116.763
DHX9	0.981662	0.018338	0.718394	0.718372	5126.932	7136.883
KLF12	0.981668	0.018332	0.827775	0.82756	376.7678	455.2777
PDZD7	0.98169	0.01831	0.979402	0.979285	97.80404	99.87317
SPAG4	0.98169	0.01831	1.098492	1.098922	143.3718	130.465
PLIN2	0.981693	0.018307	1.283492	1.283648	1325.912	1032.923
TEF	0.981694	0.018306	0.84456	0.844298	285.6323	338.3091
C19orf57	0.981703	0.018297	0.983906	0.983814	100.0269	101.6727
ZNF496	0.981704	0.018296	0.808321	0.808159	546.8135	676.6182
POLR2J4	0.981709	0.018291	1.098125	1.098552	143.5608	130.6809
HIF1AN	0.98171	0.01829	0.782417	0.782315	950.2552	1214.674
TTC7A	0.981712	0.018288	1.298331	1.298463	1666.003	1283.055
ZBTB12	0.98172	0.01828	1.105612	1.106049	152.2631	137.663
HOXB2	0.981722	0.018278	0.877456	0.877124	187.8282	214.1425
KCTD12	0.98173	0.01827	1.136946	1.137384	206.7222	181.7512
C21orf58	0.981731	0.018269	0.834704	0.834471	337.8685	404.8912
LOC72875	0.981738	0.018262	0.945494	0.945223	108.0179	114.2783
NDUFAF6	0.981745	0.018255	0.835837	0.835602	332.3115	397.6932
C1orf233	0.981752	0.018248	0.886104	0.885757	163.3772	184.4505
BANP	0.981755	0.018245	0.83801	0.837769	321.0751	383.252
CEPT1	0.981761	0.018239	1.234977	1.235227	662.4001	536.2559
CLIP2	0.981764	0.018236	1.252942	1.253153	854.674	682.0168
KIAA1841	0.981765	0.018235	0.88767	0.887325	164.4886	185.3772
LINC0050	0.981766	0.018234	0.768041	0.767966	1378.103	1794.487
PEX7	0.981768	0.018232	1.039969	1.040191	106.6953	102.5724
DLG3	0.981784	0.018216	0.792221	0.792101	794.6578	1003.231
NCAPG2	0.981784	0.018216	1.327428	1.327521	2717.396	2046.968
PRMT2	0.981784	0.018216	1.321802	1.321902	2423.984	1833.707
ZNF518A	0.981787	0.018213	0.799805	0.799666	659.0659	824.1786
C1QBP	0.981788	0.018212	0.762762	0.762694	1533.745	2010.96
TMEM187	0.981796	0.018204	0.891759	0.89141	155.5973	174.5531
POT1	0.981801	0.018199	1.251528	1.251741	842.4485	673.0192
SNTB1	0.981802	0.018198	0.792885	0.792763	764.6498	964.5409
NUBP1	0.981811	0.018189	0.856047	0.855761	245.6215	287.0229
ELMOD2	0.981814	0.018186	0.808121	0.807963	559.039	691.9141
KLHL35	0.981827	0.018173	0.965535	0.965352	103.3611	107.0712
C7orf73	0.981828	0.018172	0.744901	0.744857	2449.547	3288.617
PCED1B	0.981833	0.018167	0.97145	0.971296	102.2497	105.2717

HOXA3	0.981835	0.018165	0.933208	0.932907	120.0322	128.6654
TEN1	0.981839	0.018161	0.988932	0.98887	102.8721	104.0301
LACTB2	0.981846	0.018154	1.323279	1.323376	2502.894	1891.292
OR2A4	0.981847	0.018153	0.920362	0.920032	126.6785	137.69
TAPBPL	0.98185	0.01815	1.036137	1.036335	110.0295	106.1715
IRS1	0.981854	0.018146	1.267796	1.267975	1075.844	848.4721
ZNF107	0.981857	0.018143	1.193823	1.194161	390.1048	326.6752
KDM4B	0.981869	0.018131	0.804833	0.804685	602.384	748.5989
TM9SF3	0.98187	0.01813	1.394076	1.394115	7996.592	5735.959
MACC1	0.981876	0.018124	1.302752	1.302876	1826.135	1401.616
LINC0066	0.981878	0.018122	0.914875	0.91454	132.5134	144.8971
ARL8A	0.98188	0.01812	1.249997	1.25021	834.6686	667.6207
CCDC61	0.981884	0.018116	0.928869	0.928559	121.1436	130.465
PLAC1	0.981884	0.018116	0.928869	0.928559	121.1436	130.465
MAPK1	0.981886	0.018114	0.727197	0.727169	4061.091	5584.8
DSTNP2	0.981896	0.018104	0.967771	0.967601	104.4725	107.971
SPIN2B	0.981902	0.018098	0.985791	0.985713	104.5392	106.0545
SCAMP4	0.981903	0.018097	0.796737	0.796609	735.4753	923.26
RASSF8	0.981908	0.018092	0.839049	0.838812	324.5316	386.8961
IFT22	0.981911	0.018089	0.782581	0.782483	1002.347	1280.986
DNMT3A	0.981912	0.018088	1.200767	1.201086	430.1155	358.1038
GYG1	0.981916	0.018084	1.252598	1.252805	871.3451	695.5132
VAV1	0.981916	0.018084	0.798422	0.79829	692.4082	867.367
ZNF628	0.98192	0.01808	0.85098	0.850715	277.8524	326.6123
TRMT12	0.981928	0.018072	1.144561	1.144979	225.6161	197.0471
C19orf47	0.981928	0.018072	0.830213	0.830001	377.8793	455.2777
NSMCE4A	0.981929	0.018071	0.818476	0.818295	466.792	570.4468
WDR74	0.981931	0.018069	0.792365	0.792246	789.1008	996.0324
SEC24B	0.981941	0.018059	1.26174	1.261928	998.0458	790.8875
ORC6	0.981946	0.018054	1.311724	1.311833	2174.317	1657.463
C8orf37	0.981949	0.018051	1.028051	1.028204	107.3622	104.417
PARP15	0.981971	0.018029	1.1012	1.101609	155.4084	141.0731
WDR54	0.981983	0.018017	1.195995	1.196321	410.1101	342.8079
HOXA1	0.981985	0.018015	1.101911	1.102319	156.7088	142.1618
PRSS16	0.981986	0.018014	0.798874	0.798743	693.5196	868.2668
TMEM212	0.98199	0.01801	0.879971	0.879653	192.6517	219.0102
SMC5	0.981993	0.018007	1.306156	1.306272	2008.628	1537.678
SNX18	0.982002	0.017998	0.781137	0.781041	1022.497	1309.148
FAAP20	0.982003	0.017997	1.176502	1.176867	324.765	275.9559
ZNF30	0.982003	0.017997	1.078291	1.078646	137.8148	127.7657
PCDH7	0.982019	0.017981	1.201855	1.202166	444.5638	369.8007
TSC1	0.982021	0.017979	0.834004	0.833785	360.0967	431.884
COMMD3	0.982025	0.017975	0.817239	0.817064	485.586	594.3084
PDE6A	0.982041	0.017959	0.906945	0.906616	145.8281	160.8498
PRICKLE2	0.982044	0.017956	0.856269	0.855998	263.4041	307.7173
AASDH	0.982048	0.017952	0.860966	0.860682	240.0645	278.9251
PIBF1	0.98205	0.01795	1.172047	1.172416	311.1947	265.4287
CCNG1	0.982051	0.017949	1.375217	1.375266	5970.492	4341.334
RPL32P3	0.982054	0.017946	0.986184	0.98611	105.5839	107.0712
SECISBP2	0.982055	0.017945	0.791656	0.791541	816.886	1032.023
MON1B	0.982069	0.017931	1.249628	1.249836	855.7854	684.7161
USP11	0.98207	0.01793	0.763224	0.763159	1579.313	2069.444
DIS3L2	0.982071	0.017929	0.860608	0.860326	242.2873	281.6243
STAG3L5I	0.982076	0.017924	0.782296	0.782201	1034.311	1322.312
TOMM5	0.982076	0.017924	1.321271	1.321368	2506.229	1896.691
MRPL53	0.982084	0.017916	0.812907	0.812744	532.3652	655.024

C16orf13	0.982086	0.017914	1.215344	1.21562	551.2592	453.4782
SEMA4A	0.982094	0.017906	0.815208	0.815038	500.1343	613.6352
PCSK9	0.982104	0.017896	1.230851	1.231096	662.4001	538.0555
LOC10050	0.982105	0.017895	0.988248	0.988186	106.6953	107.971
MVK	0.98211	0.01789	1.174114	1.174476	322.3088	274.4263
RPS6KA5	0.982115	0.017885	0.874685	0.874382	210.0564	240.2355
ZNF45	0.982116	0.017884	0.863366	0.863083	242.2873	280.7246
ZNF300	0.982118	0.017882	0.892438	0.892117	173.3799	194.3478
EPHA1	0.982121	0.017879	0.775563	0.77548	1214.771	1566.479
EMILIN2	0.982126	0.017874	1.202877	1.20318	459.0122	381.4975
CCDC6	0.982133	0.017867	0.735885	0.735851	3216.419	4371.026
NOP9	0.982136	0.017864	1.23299	1.233228	685.7397	556.0506
LOC17139	0.982142	0.017858	1.08153	1.081886	141.149	130.465
CYTH3	0.982147	0.017853	1.309329	1.309438	2157.246	1647.457
GSTM2	0.982154	0.017846	0.934801	0.934522	124.4779	133.2002
CHKB	0.982168	0.017832	0.869093	0.8688	221.0038	254.3797
ARHGEF3	0.982171	0.017829	0.802236	0.802101	666.8457	831.3767
NUDT2	0.982175	0.017825	0.827383	0.827187	414.5558	501.1654
METTL22	0.982184	0.017816	1.088578	1.088947	148.9289	136.7633
ZNF818P	0.982185	0.017815	1.010343	1.010397	112.2301	111.0752
CHTF8	0.982186	0.017814	0.750228	0.75018	2195.034	2926.014
ZNF569	0.982198	0.017802	1.070785	1.071104	137.8148	128.6654
GOLPH3L	0.982205	0.017795	1.311536	1.311642	2193.923	1672.651
THAP4	0.982218	0.017782	0.786682	0.786579	915.8015	1164.287
FRY	0.982221	0.017779	0.861081	0.860807	247.8443	287.9227
SFI1	0.982226	0.017774	0.834561	0.834349	370.0994	443.5808
USP46	0.982231	0.017769	0.810926	0.810772	566.8189	699.1122
SMARCD3	0.982231	0.017769	0.827342	0.827148	419.0014	506.5639
SLC22A23	0.982237	0.017763	0.856229	0.855968	273.4068	319.4142
NR3C2	0.982242	0.017758	0.91938	0.919076	138.9262	151.1594
SLC8B1	0.982253	0.017747	1.172528	1.172879	334.5343	285.2234
OGDH	0.982254	0.017746	0.723249	0.723225	4797.955	6634.116
PAXBP1	0.982256	0.017744	0.811656	0.811503	576.8216	710.8091
FAM118A	0.982263	0.017737	1.239349	1.23957	764.5942	616.8203
PSTPIP2	0.982269	0.017731	0.865512	0.865229	231.0954	267.0933
GRAMD1C	0.982271	0.017729	1.105567	1.105956	171.1571	154.7584
ZNF32	0.982271	0.017729	1.105567	1.105956	171.1571	154.7584
SOS1	0.982273	0.017727	1.311485	1.311589	2271.721	1732.035
UBOX5	0.982275	0.017725	0.892904	0.89259	173.4799	194.3568
PWAR5	0.982276	0.017724	1.004231	1.004253	111.141	110.6703
OAZ2	0.982279	0.017721	1.298154	1.298275	1853.831	1427.916
LOC28441	0.982281	0.017719	1.149478	1.149863	254.5128	221.3405
MTRNR2L	0.982283	0.017717	1.099477	1.099857	163.9885	149.0989
GPX1	0.982284	0.017716	1.329778	1.329862	2951.904	2219.704
UQCC3	0.982284	0.017716	0.811701	0.811548	579.0444	713.5083
GINS3	0.982287	0.017713	0.860055	0.859786	254.5128	296.0205
F8A1	0.982291	0.017709	0.835444	0.835231	368.988	441.7813
ADK	0.9823	0.0177	1.326699	1.326786	2818.535	2124.329
TNXA	0.982305	0.017695	0.661709	0.207127	0.077799	0.413889
RBPMS2	0.982307	0.017693	1.069638	1.069948	136.7034	127.7657
SNX8	0.982319	0.017681	0.824335	0.824153	461.235	559.6497
POLB	0.982319	0.017681	1.199968	1.200267	457.9007	381.4975
LOC10012	0.982324	0.017676	1.110633	1.111022	180.0484	162.0555
SSBP4	0.982335	0.017665	1.228873	1.229112	670.18	545.2535
NCBP2-AS	0.982345	0.017655	0.970031	0.969888	117.8094	121.4674
FMNL2	0.982346	0.017654	1.321571	1.321663	2685.166	2031.654

NMRAL1	0.982351	0.017649	0.805549	0.805411	660.1773	819.6798
PHLDA1	0.982358	0.017642	0.800445	0.800318	732.4189	915.1622
RBM48	0.982362	0.017638	0.838051	0.837835	358.1295	427.4482
PBLD	0.982372	0.017628	1.078432	1.078763	145.5947	134.9637
C1orf216	0.982374	0.017626	0.942923	0.942681	128.9235	136.7633
MYL12A	0.982377	0.017623	1.427741	1.427764	15284.69	10705.33
KDM2A	0.982389	0.017611	1.341106	1.341178	3587.63	2674.981
ALG13	0.98239	0.01761	0.817212	0.817048	516.8055	632.5301
FRMD5	0.982407	0.017593	1.1585	1.158865	286.7437	247.4335
ABCD1	0.982408	0.017592	0.802591	0.802461	706.8565	880.8634
CCNH	0.982423	0.017577	0.825733	0.825549	445.6752	539.855
ARMC7	0.982426	0.017574	0.878681	0.878392	213.3906	242.9347
KCTD20	0.982435	0.017565	1.345242	1.34531	3858.859	2868.375
ZNF527	0.982437	0.017563	0.922369	0.922082	142.0604	154.0656
ZBED8	0.982446	0.017554	0.843323	0.843098	334.5343	396.7934
SHC1	0.982457	0.017543	1.357189	1.357249	4611.238	3397.487
P2RY2	0.98246	0.01754	1.202915	1.2032	487.2864	404.9902
FAM83D	0.982462	0.017538	1.303665	1.303776	2071.667	1588.973
WASH5P	0.982468	0.017532	0.998444	0.998436	115.6088	115.7899
NR1H3	0.982472	0.017528	0.936519	0.936263	132.2577	141.2621
LINC0011(	0.982477	0.017523	1.207109	1.207383	530.1424	439.0821
ZNF670	0.982487	0.017513	0.93902	0.93877	128.7012	137.0962
TTYH2	0.98249	0.01751	0.993788	0.993758	118.9208	119.6679
RPL36A-H	0.982492	0.017508	0.793998	0.793887	855.1296	1077.146
C11orf80	0.982492	0.017508	0.918092	0.917803	147.8175	161.0567
RQCD1	0.982495	0.017505	0.77413	0.774053	1270.341	1641.159
ZNF283	0.982497	0.017503	0.947592	0.947369	126.7007	133.7401
XK	0.982504	0.017496	0.90026	0.899959	170.0457	188.9492
DRAP1	0.982512	0.017488	1.31787	1.317964	2531.791	1920.984
CDAN1	0.982516	0.017484	1.197149	1.197443	457.9007	382.3973
MBNL1-AS	0.982521	0.017479	1.029139	1.029279	122.2551	118.7771
ARHGAP1	0.982524	0.017476	0.7759	0.775821	1248.113	1608.768
APEX1	0.982524	0.017476	0.768365	0.768297	1490.4	1939.879
PPIAL4D	0.982525	0.017475	1.079684	1.227567	0.366765	0.29692
PPIAL4E	0.982525	0.017475	1.079684	1.227567	0.366765	0.29692
PPIAL4F	0.982525	0.017475	1.079684	1.227567	0.366765	0.29692
MOK	0.982528	0.017472	1.007153	1.007188	117.8094	116.9686
DECR1	0.982533	0.017467	0.766269	0.766204	1610.432	2101.835
MAP7D1	0.982558	0.017442	1.353749	1.35381	4504.543	3327.306
ZNF200	0.982559	0.017441	0.883998	0.883708	201.1651	227.6389
ERN1	0.98256	0.01744	1.15873	1.159081	304.5262	262.7294
KITLG	0.982571	0.017429	1.040932	1.04112	131.1463	125.9662
EEPD1	0.982571	0.017429	0.936036	0.935786	138.9262	148.4601
ZNF92	0.982573	0.017427	1.208709	1.208975	550.2255	455.1157
PVRL2	0.98258	0.01742	1.356095	1.356153	4811.292	3547.747
CALCOCO	0.98258	0.01742	0.756886	0.756833	1993.869	2634.492
AZIN2	0.982592	0.017408	1.11809	1.118462	202.2765	180.8514
TBKBP1	0.982592	0.017408	1.160517	1.160864	312.3061	269.0277
BUB1B-PA	0.982599	0.017401	1.134608	1.134979	234.4296	206.5485
SMAGP	0.982599	0.017401	0.863479	0.863222	265.6269	307.7173
CENPP	0.982603	0.017397	0.926698	0.926428	143.3718	154.7584
TMEM18	0.982605	0.017395	0.860755	0.860503	275.6296	320.314
STS	0.982607	0.017393	1.098385	1.098736	178.9369	162.8563
LOC10192	0.98262	0.01738	0.882809	0.882524	206.2554	233.7122
LINS1	0.982621	0.017379	1.044989	1.045192	134.4806	128.6654
HMGN5	0.982635	0.017365	0.953567	0.95337	127.8121	134.064

CCDC130	0.982642	0.017358	0.844118	0.843901	352.3168	417.4879
KLHL18	0.982643	0.017357	1.207172	1.20744	531.2538	439.9818
ARNT	0.982658	0.017342	1.311556	1.311655	2346.186	1788.72
GPD1L	0.98266	0.01734	0.762626	0.762567	1748.247	2292.584
ZSCAN2	0.98266	0.01734	1.015489	1.015561	125.5782	123.6538
DDX51	0.98266	0.01734	0.861871	0.861617	266.9161	309.7868
CCDC64B	0.982675	0.017325	0.840893	0.840683	362.3195	430.9842
OTUB2	0.982677	0.017323	1.131923	1.132289	232.2846	205.1449
VEZF1	0.982678	0.017322	0.78196	0.781872	1113.632	1424.317
CRYBB2P	0.982695	0.017305	0.884127	0.883847	212.2348	240.1275
AMMECR1	0.982698	0.017302	0.79399	0.793881	860.7311	1084.209
ATP5SL	0.982701	0.017299	1.257489	1.257663	1059.173	842.1738
C17orf51	0.982705	0.017295	1.076754	1.077056	158.9316	147.5604
NHEJ1	0.982718	0.017282	0.858803	0.858558	281.1866	327.512
CTAGE4	0.982723	0.017277	0.814879	0.814729	571.1756	701.0647
DNAH14	0.982723	0.017277	1.137883	1.138244	247.8443	217.7415
AKT1	0.982728	0.017272	1.341599	1.341668	3796.575	2829.74
MARS2	0.982734	0.017266	0.839468	0.839264	374.545	446.2801
RASSF6	0.982734	0.017266	0.893862	0.893577	188.9396	211.4432
NMRK1	0.982737	0.017263	0.816261	0.816109	573.4873	702.7112
NUDT16	0.982738	0.017262	0.77558	0.775504	1299.238	1675.35
KREMEN1	0.982739	0.017261	1.25963	1.2598	1098.628	872.0637
C2orf44	0.982739	0.017261	0.826214	0.82604	480.1289	581.2439
LINC0157	0.98274	0.01726	1.173142	1.173464	358.9853	305.9178
ZSCAN9	0.982751	0.017249	1.173504	1.173825	361.2081	307.7173
CHST11	0.982759	0.017241	1.204206	1.204474	522.3625	433.6835
SLC16A3	0.982765	0.017235	1.284367	1.284497	1622.658	1263.261
SNAR-A10	0.982774	0.017226	0.943416	0.829793	0.222282	0.269927
SNAR-A5	0.982774	0.017226	0.943416	0.829793	0.222282	0.269927
YEATS2	0.982778	0.017222	1.303014	1.303122	2096.118	1608.534
CDC14A	0.982781	0.017219	0.981262	0.981178	124.4779	126.8659
MTX3	0.982781	0.017219	1.22207	1.222304	660.1551	540.0889
PTH2R	0.982787	0.017213	1.144606	1.144958	267.8497	233.9372
ADPGK	0.982788	0.017212	1.231351	1.231567	747.9786	607.3369
TRMO	0.982788	0.017212	1.033586	1.033737	131.1463	126.8659
XRCC3	0.982789	0.017211	0.812641	0.812497	602.384	741.4008
TRMT10B	0.982792	0.017208	0.944804	0.944588	140.0376	148.2532
ARL4D	0.982795	0.017205	1.145296	1.145647	270.0725	235.7367
RC3H1	0.982796	0.017204	1.231356	1.231573	749.0901	608.2366
ALPL	0.9828	0.0172	1.061883	1.062137	150.0403	141.2621
CUEDC1	0.982804	0.017196	0.817108	0.816955	555.7048	680.2173
ZNF320	0.982808	0.017192	0.889024	0.888744	200.9762	226.1363
ATP5O	0.982808	0.017192	0.722709	0.722687	5266.97	7288.042
LRRC40	0.982809	0.017191	0.793827	0.793721	879.125	1107.602
ZCCHC11	0.982814	0.017186	0.791528	0.791426	923.5814	1166.987
SESN1	0.982817	0.017183	1.113771	1.114124	204.4994	183.5507
ENDOG	0.982821	0.017179	1.147631	1.147978	277.8524	242.035
CRELD1	0.982826	0.017174	1.288369	1.288494	1693.788	1314.547
NYAP2	0.982833	0.017167	1.148595	1.148941	281.1866	244.7343
BZW2	0.982837	0.017163	1.305332	1.305436	2190.588	1678.049
CTCF	0.982837	0.017163	0.80321	0.803086	729.0847	907.8561
SLC38A7	0.982845	0.017155	0.846165	0.84595	344.8148	407.6085
BBIP1	0.982847	0.017153	0.880934	0.880662	220.2814	250.1328
CENPW	0.982847	0.017153	1.215024	1.215268	609.0525	501.1654
ANKRD9	0.982849	0.017151	0.95219	0.951997	134.4806	141.2621
COA4	0.982856	0.017144	0.780893	0.78081	1172.537	1501.697

L3MBTL1	0.982862	0.017138	1.104856	1.105201	188.9396	170.9541
SPATA5	0.982865	0.017135	1.133736	1.134085	252.1121	222.3033
TCHP	0.982868	0.017132	0.857081	0.856848	304.5262	355.4045
CWF19L2	0.982868	0.017132	1.187768	1.188058	447.8981	376.9987
KMT5C	0.982871	0.017129	0.8511	0.850876	322.3088	378.7982
C6orf62	0.982881	0.017119	1.353089	1.353147	4667.92	3449.673
INPP5B	0.982888	0.017112	1.167528	1.167849	346.7598	296.9202
PFAS	0.982888	0.017112	0.832762	0.832578	441.2296	529.9576
ZNF793	0.982892	0.017108	1.058416	1.058654	150.596	142.2518
SIKE1	0.982892	0.017108	0.783601	0.783514	1089.181	1390.127
BBS12	0.982896	0.017104	1.073189	1.07347	162.2658	151.1594
C17orf62	0.982899	0.017101	0.773181	0.773109	1381.482	1786.92
TRIT1	0.982905	0.017095	0.81581	0.815662	579.0444	709.9093
NOL10	0.982913	0.017087	1.236698	1.236901	821.3317	664.0216
SREK1IP1	0.982915	0.017085	0.84118	0.84098	387.4707	460.7392
ACVR2B	0.982916	0.017084	0.852419	0.852195	318.9745	374.2995
SEC61A2	0.982917	0.017083	1.043082	1.043266	138.9262	133.1642
LMO4	0.982926	0.017074	1.347494	1.347556	4293.375	3186.044
TRIM14	0.982943	0.017057	1.205734	1.20599	550.1477	456.1775
S100A16	0.982943	0.017057	1.397193	1.397226	9768.179	6991.122
KIF16B	0.982946	0.017054	0.832951	0.832767	432.3383	519.1605
KCNQ1OT	0.982947	0.017053	0.763108	0.763051	1803.829	2363.971
VPS51	0.982948	0.017052	0.810936	0.810799	639.0605	788.1883
COPS8	0.982951	0.017049	1.269541	1.26969	1310.352	1032.023
FIBP	0.982954	0.017046	0.783794	0.783708	1145.863	1462.107
CDCA2	0.982954	0.017046	1.259671	1.259835	1138.083	903.3573
CEP70	0.982961	0.017039	1.278701	1.278836	1499.292	1172.385
RFX2	0.982961	0.017039	0.915938	0.915674	165.6	180.8514
TTC27	0.982962	0.017038	0.804955	0.804831	719.082	893.46
ZNF461	0.982979	0.017021	1.007611	1.007645	130.0571	129.0703
FARS2	0.982979	0.017021	0.850151	0.849934	333.4229	392.2946
RUFY3	0.982979	0.017021	0.850151	0.849934	333.4229	392.2946
C21orf2	0.982985	0.017015	1.059617	1.059855	154.4859	145.7608
FAM27C	0.982987	0.017013	0.6041	0.025196	0	0.386896
EFCAB7	0.98299	0.01701	0.887225	0.886956	212.2792	239.3357
HTRA1	0.983003	0.016997	1.127251	1.127591	244.5101	216.8418
IL22RA1	0.983009	0.016991	1.072228	1.072501	161.1544	150.2596
ADCK3	0.98301	0.01699	1.009841	1.009884	134.4806	133.1642
ZNF236	0.983017	0.016983	1.073194	1.07347	162.2658	151.1594
DGKZ	0.98302	0.01698	1.266264	1.266416	1263.673	997.832
CBLB	0.983023	0.016977	0.815066	0.814923	599.0498	735.1025
KLHL13	0.983032	0.016968	1.219087	1.219315	681.2941	558.7499
CHI3L1	0.983037	0.016963	0.828274	0.828102	465.6806	562.3489
CDKN1B	0.983048	0.016952	0.791679	0.791581	955.8122	1207.476
WDSUB1	0.98305	0.01695	0.84197	0.841773	392.3276	466.0748
PURA	0.983056	0.016944	1.243971	1.244157	929.1384	746.7994
DHX37	0.98306	0.01694	0.79762	0.797511	830.223	1041.02
TCTEX1D	0.98307	0.01693	1.068825	1.069085	164.4886	153.8587
MAP4K5	0.98307	0.01693	1.28723	1.287352	1729.353	1343.339
CDK20	0.983073	0.016927	0.91376	0.913501	176.7141	193.448
POMGNT2	0.983073	0.016927	0.93706	0.936837	150.0403	160.157
LINC0064	0.983073	0.016927	1.034559	1.034704	143.3718	138.5628
LOC10192	0.983095	0.016905	1.127706	1.128041	250.0783	221.6914
ZNF836	0.983101	0.016899	1.072614	1.072882	168.9343	157.4577
GPX3	0.983106	0.016894	0.697842	0.697833	12332.2	17672.15
IKBKB	0.983106	0.016894	1.200862	1.201118	547.9249	456.1775

GSTT2B	0.983108	0.016892	0.899068	0.898803	194.63	216.5448
TBC1D7	0.98311	0.01689	1.293659	1.293771	1964.972	1518.792
EXOC3	0.983112	0.016888	0.864904	0.864666	280.0752	323.913
FAM185A	0.983122	0.016878	0.972361	0.972249	135.592	139.4625
ATF7IP	0.983131	0.016869	1.255068	1.255233	1126.969	897.8148
NCOA6	0.983133	0.016867	0.770172	0.770108	1565.976	2033.454
GALNT11	0.983137	0.016863	1.262626	1.26278	1222.551	968.1399
ANKRD33	0.983144	0.016856	0.785784	0.785697	1101.407	1401.823
RPL21	0.983145	0.016855	1.355166	1.355221	5015.314	3700.733
ZNF449	0.983154	0.016846	1.046216	1.046403	147.8175	141.2621
ATAT1	0.983154	0.016846	1.129446	1.129779	250.0672	221.3405
CSNK2A1	0.983156	0.016844	1.355043	1.355098	5013.602	3699.806
LIN37	0.98317	0.01683	1.131548	1.131879	255.6242	225.8393
RGS12	0.983173	0.016827	1.064034	1.064277	160.043	150.3766
NDUFA6	0.983179	0.016821	0.766254	0.766195	1699.345	2217.904
HSF2	0.983187	0.016813	0.853447	0.853233	340.0913	398.5929
ZNF844	0.983195	0.016805	0.94659	0.946396	151.5629	160.148
FRG1BP	0.983199	0.016801	1.055816	1.056034	152.2187	144.1413
SNAR-A6	0.983205	0.016795	0.943916	0.829793	0.222282	0.269927
SNAR-A9	0.983205	0.016795	0.943916	0.829793	0.222282	0.269927
PHC1	0.983209	0.016791	0.81998	0.819832	581.2672	709.0095
ZCCHC9	0.98321	0.01679	0.851336	0.851125	342.3142	402.192
LCORL	0.983213	0.016787	1.156733	1.157047	328.9772	284.3236
CNEP1R1	0.983216	0.016784	1.219711	1.21993	709.0793	581.2439
TRPS1	0.983223	0.016777	1.169553	1.169852	377.8793	323.0132
OGFOD3	0.983224	0.016776	0.811903	0.811771	657.4877	809.9444
C5orf45	0.983224	0.016776	0.882565	0.882312	233.396	264.5289
EXD3	0.983228	0.016772	1.168021	1.168319	382.3249	327.2421
JADE2	0.983233	0.016767	0.78593	0.785844	1113.632	1417.119
GOLGA2P	0.983234	0.016766	0.831234	0.831065	481.2403	579.0664
GALNT12	0.983242	0.016758	0.862625	0.862396	295.6349	342.8079
PICALM	0.983244	0.016756	1.391072	1.391106	9194.691	6609.625
NT5DC1	0.983246	0.016754	0.807393	0.807269	719.082	890.7607
C1orf35	0.983249	0.016751	1.10851	1.10883	214.502	193.448
TRPM2	0.983254	0.016746	1.248615	1.248787	1023.608	819.6798
HEBP1	0.983254	0.016746	1.265447	1.265594	1297.015	1024.825
MRPS18C	0.983258	0.016742	0.818939	0.818793	581.2672	709.9093
TMEM51	0.983259	0.016741	1.202352	1.202598	573.4873	476.8719
DPY19L3	0.983261	0.016739	0.799315	0.799206	829.1115	1037.421
ATAD3C	0.983267	0.016733	1.120046	1.120371	236.0189	210.6604
KANK2	0.98327	0.01673	1.309045	1.309137	2539.571	1939.879
HAUS2	0.98327	0.01673	1.228507	1.228711	781.6988	636.1921
TMEM50A	0.983276	0.016724	1.298224	1.298328	2103.898	1620.465
SLC25A24	0.983276	0.016724	1.27558	1.275713	1505.96	1180.483
CDC7	0.983279	0.016721	0.820019	0.819874	587.9357	717.1074
DENND1A	0.983282	0.016718	1.233437	1.233632	859.1196	696.4129
STON1	0.983289	0.016711	0.883434	0.883186	240.7869	272.6358
ATP8B2	0.983291	0.016709	0.797139	0.797035	887.8162	1113.901
PPME1	0.983295	0.016705	1.305753	1.305848	2425.096	1857.101
C3orf58	0.983305	0.016695	0.83647	0.836291	435.6726	520.9601
ALG14	0.983307	0.016693	1.043252	1.043422	151.1517	144.8611
DLX3	0.98331	0.01669	0.861516	0.861294	312.3061	362.6026
DLX6	0.983321	0.016679	0.943226	0.943029	157.8202	167.355
MED13L	0.983321	0.016679	0.770223	0.77016	1610.432	2091.038
ELF1	0.983324	0.016676	1.243628	1.243806	966.9263	777.3912
KBTBD3	0.983336	0.016664	1.021353	1.02144	143.3718	140.3623

GMEB1	0.983341	0.016659	1.106543	1.106853	221.1705	199.8183
GOPC	0.983346	0.016654	1.292503	1.292613	1952.747	1510.694
BCRP2	0.983348	0.016652	1.04979	1.049981	156.1642	148.73
INO80B-W	0.98336	0.01664	0.898919	0.898669	210.7566	234.522
MFSD2A	0.983361	0.016639	1.158856	1.159156	355.6511	306.8176
TMEM208	0.983369	0.016631	0.847425	0.84723	383.4363	452.5784
SLC35A4	0.98337	0.01663	0.757827	0.757779	2247.27	2965.603
PITPNA	0.983379	0.016621	0.775458	0.775389	1439.275	1856.201
CBR1	0.983385	0.016615	1.294686	1.294793	2029.434	1567.379
TMX2	0.983386	0.016614	0.757628	0.757581	2263.941	2988.385
AP5Z1	0.983387	0.016613	0.992329	0.992298	141.049	142.1438
PMF1-BGI	0.983388	0.016612	0.980153	0.980075	142.0715	144.9601
KIF7	0.983397	0.016603	1.007039	1.007067	142.2604	141.2621
C18orf54	0.983405	0.016595	1.025372	1.025473	146.7061	143.0616
DOHH	0.983407	0.016593	0.894414	0.894164	215.6135	241.1352
CDC42SE	0.983409	0.016591	0.745312	0.745277	3043.039	4083.103
TMEM182	0.983409	0.016591	0.887941	0.887696	235.6188	265.4287
ZBTB7B	0.983413	0.016587	0.771533	0.771469	1554.862	2015.459
SURF6	0.983416	0.016584	1.212983	1.213205	673.5142	555.1509
ARV1	0.983416	0.016584	0.856033	0.855822	333.4229	389.5953
SPTLC3	0.983419	0.016581	1.100914	1.101215	210.0564	190.7488
MAK16	0.983421	0.016579	0.811365	0.811238	686.8511	846.6726
ZNF524	0.983421	0.016579	1.175978	1.176258	421.2242	358.1038
LOC14688	0.983423	0.016577	0.965412	0.965283	150.2626	155.6672
BLOC1S6	0.98343	0.01657	1.228538	1.228737	803.7492	654.1243
CDADC1	0.98343	0.01657	0.905071	0.904824	197.8309	218.6413
EIF3K	0.983443	0.016557	0.733331	0.733305	4244.473	5788.145
SPAG5	0.983444	0.016556	1.298422	1.298523	2166.137	1668.152
MEN1	0.983446	0.016554	0.794504	0.794407	961.3693	1210.175
LSM10	0.983451	0.016549	0.818615	0.818475	604.6068	738.7016
AGFG2	0.983461	0.016539	0.771069	0.771006	1583.759	2054.148
ACOT11	0.983463	0.016537	1.056519	1.056726	164.4886	155.6582
WDR76	0.983482	0.016518	1.217649	1.217863	703.889	577.9687
CORO7	0.983488	0.016512	0.904467	0.904222	201.1651	222.4742
INTS6	0.983489	0.016511	1.236565	1.236749	903.576	730.6037
AUNIP	0.983493	0.016507	1.178018	1.178292	436.7951	370.7004
NLRC5	0.983494	0.016506	1.158411	1.158707	353.4282	305.0181
BORA	0.983501	0.016499	1.190387	1.190643	504.5799	423.7862
MAP3K10	0.983509	0.016491	1.209793	1.210018	640.1719	529.0579
INPP5K	0.983514	0.016486	0.860518	0.860306	328.9772	382.3973
BPTF	0.98352	0.01648	0.783914	0.783835	1218.394	1554.404
APRT	0.98353	0.01647	0.770968	0.770907	1634.883	2120.73
SDR42E1	0.983531	0.016469	1.086724	1.086999	195.6081	179.9517
TEKT4P2	0.983533	0.016467	0.839449	0.839272	433.4497	516.4613
C1D	0.983541	0.016459	0.843924	0.84374	416.7786	493.9673
RAB20	0.983543	0.016457	0.840401	0.840222	427.8927	509.2632
TSFM	0.983546	0.016454	0.816937	0.816803	633.5035	775.5917
GALT	0.983547	0.016453	1.146116	1.146418	315.6403	275.326
BRD3	0.983552	0.016448	1.226499	1.226697	799.1035	651.425
LRIG2	0.983552	0.016448	1.065325	1.065553	174.4913	163.756
ZNF37BP	0.983553	0.016447	0.875042	0.874814	277.8524	317.6147
HCFC2	0.983565	0.016435	0.907059	0.906821	205.6108	226.7391
SLC30A7	0.983566	0.016434	1.208195	1.208419	651.286	538.9552
SYT15	0.983567	0.016433	1.081208	1.081468	195.5859	180.8514
KCTD10	0.983567	0.016433	1.281785	1.281903	1771.665	1382.056
C15orf61	0.983577	0.016423	1.036572	1.03671	156.7088	151.1594

SLC25A22	0.98358	0.01642	1.177432	1.177703	432.3383	367.1014
ZFYVE1	0.98359	0.01641	1.23938	1.239557	954.7008	770.1931
OSR2	0.983592	0.016408	0.926646	0.926427	176.7141	190.7488
ZNF460	0.983603	0.016397	0.989713	0.989674	147.8175	149.3599
FANCA	0.983606	0.016394	1.163874	1.164158	382.3249	328.4118
OVOL2	0.983617	0.016383	0.953439	0.953279	157.8202	165.5555
ZNF441	0.983618	0.016382	0.967131	0.967012	152.2631	157.4577
CHIC2	0.983625	0.016375	0.947842	0.947667	161.1544	170.0543
STAM2	0.983625	0.016375	0.806183	0.806071	805.7719	999.6315
ZNF426	0.983626	0.016374	1.003145	1.003157	148.9289	148.4601
DDX12P	0.983628	0.016372	1.052952	1.05314	172.0795	163.3961
PRPF18	0.98363	0.01637	0.855363	0.855164	360.0967	421.0869
PTPRF	0.983641	0.016359	1.381269	1.381305	8271.11	5987.892
RAD54B	0.983648	0.016352	1.258457	1.258605	1253.037	995.5736
C1QTNF1	0.98365	0.01635	0.940725	0.940535	166.7114	177.2524
SLC39A9	0.98365	0.01635	1.309105	1.309192	2695.168	2058.647
C19orf25	0.983662	0.016338	0.897362	0.897125	221.1705	246.5338
LIX1L	0.983662	0.016338	1.19162	1.191868	516.9277	433.7105
CYP2U1	0.983662	0.016338	1.171533	1.171804	431.2269	368.0011
TRIM7	0.983663	0.016337	1.018453	1.018523	156.7088	153.8587
ST6GALN	0.983664	0.016336	0.845462	0.84528	416.7786	493.0676
ATP11C	0.983665	0.016335	1.262513	1.262655	1330.357	1053.617
TEAD3	0.983665	0.016335	1.25628	1.25643	1251.447	996.0324
STK24	0.983666	0.016334	1.319267	1.319345	3155.292	2391.558
ZNF706	0.983689	0.016311	0.801363	0.80126	894.6847	1116.6
ELOVL3	0.98369	0.01631	1.060476	1.060684	175.6027	165.5555
ARNTL	0.98369	0.01631	0.875038	0.874814	277.8524	317.6147
TEC	0.983695	0.016305	0.998246	0.998239	154.4859	154.7584
ZNF700	0.983696	0.016304	0.862377	0.86217	332.5449	385.7084
TUBB	0.9837	0.0163	1.465348	1.465358	35866.3	24476.13
NUDCD2	0.983703	0.016297	0.801227	0.801123	875.7907	1093.206
C14orf105	0.983704	0.016296	0.689567	0.03691	0	0.26093
ZNF420	0.983705	0.016295	1.030422	1.030534	162.2658	157.4577
ICK	0.983707	0.016293	0.918596	0.918372	190.051	206.9444
ZDHHC24	0.983709	0.016291	0.900259	0.900024	217.8363	242.035
WEE1	0.983713	0.016287	1.226768	1.22696	824.6659	672.1195
ZDHHC12	0.983717	0.016283	0.823633	0.82349	580.1558	704.5108
ZNF140	0.983721	0.016279	0.923862	0.923645	184.494	199.7463
C19orf33	0.983724	0.016276	1.248822	1.248981	1110.298	888.9612
RNF111	0.983731	0.016269	0.816711	0.816581	656.8431	804.3839
LOC10050	0.983735	0.016265	0.896523	0.896288	221.7707	247.4335
APOPT1	0.983737	0.016263	1.256129	1.256277	1263.673	1005.885
SLC25A20	0.983739	0.016261	1.153538	1.153821	362.3195	314.0156
KIF24	0.983745	0.016255	0.979741	0.979667	153.3745	156.5579
DDX26B	0.98375	0.01625	1.049768	1.049943	170.0457	161.9565
SEPT7	0.983752	0.016248	1.367904	1.367947	6747.368	4932.475
HOXB7	0.983754	0.016246	0.879919	0.879694	266.7383	303.2185
BRIP1	0.983756	0.016244	0.837739	0.837574	476.3501	568.7282
MTCH1	0.983764	0.016236	1.388319	1.388352	9472.544	6822.867
TRA2B	0.983767	0.016233	0.748568	0.748532	2948.57	3939.142
HACE1	0.983768	0.016232	1.092648	1.092919	213.3906	195.2476
BRF1	0.983771	0.016229	0.831193	0.83104	530.1424	637.9286
PIGU	0.983779	0.016221	0.832124	0.831967	509.0256	611.8356
SORD	0.983779	0.016221	1.244044	1.244207	1078.067	866.4672
PRPF3	0.983784	0.016216	0.791715	0.791628	1076.956	1360.435
CEP135	0.983785	0.016215	0.981196	0.981128	154.4859	157.4577

MUL1	0.983786	0.016214	0.806262	0.806153	826.8887	1025.724
SNAR-A11	0.983787	0.016213	0.944617	0.829793	0.222282	0.269927
SNAR-A14	0.983787	0.016213	0.944617	0.829793	0.222282	0.269927
SNAR-A3	0.983787	0.016213	0.944617	0.829793	0.222282	0.269927
SNAR-A4	0.983787	0.016213	0.944617	0.829793	0.222282	0.269927
SNAR-A7	0.983787	0.016213	0.944617	0.829793	0.222282	0.269927
SNAR-A8	0.983787	0.016213	0.944617	0.829793	0.222282	0.269927
LYPLA1	0.983789	0.016211	1.364477	1.364521	6416.168	4702.137
NAA30	0.983795	0.016205	1.231989	1.232171	895.7961	727.0047
PEG10	0.983799	0.016201	0.696531	0.696522	13651.44	19599.44
TTC31	0.9838	0.0162	0.846201	0.84602	403.4417	476.8719
CCNB2	0.983801	0.016199	1.25806	1.258205	1278.121	1015.827
DDIT3	0.983803	0.016197	0.795175	0.795083	1005.826	1265.06
SLC39A8	0.98381	0.01619	1.283927	1.284038	1900.51	1480.102
ZBTB39	0.983811	0.016189	0.850477	0.850291	387.8819	456.1775
SPATA5L1	0.983827	0.016173	1.130549	1.13084	288.9665	255.5314
RARG	0.983831	0.016169	0.81722	0.817092	662.4001	810.6822
MAOA	0.983835	0.016165	0.844431	0.844257	436.784	517.361
RILPL2	0.983836	0.016164	1.042931	1.043082	168.9343	161.9565
RINL	0.983839	0.016161	1.057634	1.057828	178.9369	169.1546
ZBTB8OS	0.983841	0.016159	0.809487	0.809373	764.6498	944.7462
LOC10050	0.983849	0.016151	1.05857	1.058766	180.0484	170.0543
ACSF2	0.983864	0.016136	0.763989	0.763939	1996.092	2612.898
FAM69B	0.983868	0.016132	1.195671	1.195903	574.5988	480.4709
TCEAL1	0.983868	0.016132	0.967384	0.967273	164.4886	170.0543
POP7	0.983869	0.016131	0.791224	0.79114	1132.526	1431.515
TRADD	0.983879	0.016121	0.949405	0.949245	173.3799	182.6509
WDR27	0.98388	0.01612	0.979976	0.979905	157.8202	161.0567
CLASP2	0.983881	0.016119	0.776239	0.776175	1531.522	1973.17
ARL6IP4	0.983886	0.016114	0.777612	0.777546	1485.955	1911.087
ALG11	0.983892	0.016108	0.889081	0.888857	249.9227	281.1745
RPL22L1	0.983894	0.016106	1.111913	1.112195	251.1786	225.8393
SCUBE3	0.983895	0.016105	1.021567	1.021644	165.6	162.0915
C17orf49	0.983896	0.016104	1.174771	1.175028	454.4887	386.7881
PRKX	0.9839	0.0161	0.801145	0.801045	906.8991	1132.148
RAB33B	0.9839	0.0161	0.870636	0.870425	304.504	349.835
FAM71E1	0.983919	0.016081	1.115713	1.115995	260.0698	233.0374
SDHAP1	0.983926	0.016074	0.950384	0.950227	174.4913	183.6317
LOC10012	0.98393	0.01607	0.817045	0.816919	676.1371	827.6697
TANK	0.983931	0.016069	1.265954	1.266086	1459.281	1152.59
SYNM	0.983934	0.016066	0.855157	0.854967	372.3222	435.483
RAB18	0.983939	0.016061	1.333106	1.33317	3978.846	2984.498
LPGAT1	0.98394	0.01606	1.24972	1.249873	1165.813	932.7434
ADAM19	0.983944	0.016056	1.109985	1.110261	256.7356	231.2379
CGGBP1	0.983947	0.016053	0.757153	0.757111	2497.337	3298.514
MAX	0.983949	0.016051	1.237879	1.238048	975.8176	788.1883
C19orf43	0.983953	0.016047	0.773113	0.773053	1663.78	2152.222
PRPSAP2	0.983958	0.016042	0.826836	0.826694	573.4873	693.7137
NXF1	0.98396	0.01604	0.791059	0.790976	1153.643	1458.508
HNRNPA1	0.983961	0.016039	0.775422	0.775359	1580.436	2038.33
ZNF687	0.983966	0.016034	0.787743	0.787665	1238.11	1571.878
ZNF605	0.983969	0.016031	0.941309	0.941132	177.8255	188.9492
TMED1	0.983971	0.016029	1.182863	1.183107	504.5799	426.4854
LTBP3	0.983981	0.016019	1.261085	1.261223	1348.14	1068.913
PRSS22	0.983983	0.016017	0.819876	0.819747	649.0632	791.7873
FAM171A1	0.983986	0.016014	1.125049	1.12533	284.5209	252.8321

PACS2	0.983999	0.016001	0.786666	0.78659	1273.698	1619.268
GGPS1	0.983999	0.016001	0.786479	0.786402	1278.821	1626.169
SVIP	0.984001	0.015999	1.118587	1.118864	277.8524	248.3333
SLC25A14	0.984002	0.015998	0.936304	0.93612	187.8282	200.6461
SOCS6	0.984002	0.015998	0.894844	0.894623	242.2873	270.8272
CNTLN	0.984002	0.015998	1.11906	1.119337	278.9971	249.251
STOX2	0.984004	0.015996	0.850257	0.850079	414.5558	487.669
FAM126A	0.984008	0.015992	1.223619	1.223806	833.5572	681.117
SLC37A3	0.984008	0.015992	0.773235	0.773176	1673.783	2164.818
WDR90	0.984012	0.015988	1.057674	1.057861	185.6054	175.4529
PSMA3-AS	0.984016	0.015984	1.070314	1.07053	202.2765	188.9492
PHC3	0.984021	0.015979	1.283384	1.283493	1901.633	1481.605
ARPC2	0.984026	0.015974	1.390345	1.390375	10438.36	7507.583
APOOL	0.984031	0.015969	0.823625	0.82349	613.4981	744.9999
LDB3	0.984031	0.015969	1.033717	1.033833	171.1571	165.5555
TOB1	0.984035	0.015965	0.80418	0.804077	874.6793	1087.808
DTWD1	0.984042	0.015958	0.900475	0.900257	238.9531	265.4287
GAS2L3	0.984046	0.015954	1.225346	1.225529	858.0638	700.1559
RANBP17	0.984052	0.015948	0.941031	0.940857	181.1598	192.5483
TRAPPC2	0.984055	0.015945	0.884629	0.884415	277.0744	313.2868
FADD	0.984061	0.015939	1.215424	1.215621	757.9813	623.5325
C16orf91	0.984062	0.015938	1.082002	1.08224	212.2792	196.1473
PTPRS	0.984069	0.015931	1.277547	1.277661	1807.152	1414.42
ATG5	0.98407	0.01593	1.251108	1.251256	1214.771	970.8392
WTIP	0.984071	0.015929	0.946789	0.946629	182.2712	192.5483
PLLP	0.984076	0.015924	0.992262	0.992235	163.3772	164.6558
CEP57	0.984077	0.015923	0.755448	0.755408	2659.603	3520.754
CNST	0.984078	0.015922	0.799202	0.799108	971.372	1215.573
RALGPS1	0.984079	0.015921	1.110955	1.111226	258.9584	233.0374
ANKRD27	0.984084	0.015916	1.334572	1.334633	4163.34	3119.462
CBFB	0.984087	0.015913	0.804187	0.804086	905.7988	1126.497
SLC35E1	0.984088	0.015912	1.334063	1.334124	4236.693	3175.634
FAM21A	0.984093	0.015907	0.761375	0.76133	2303.085	3025.086
WDR18	0.984094	0.015906	0.858134	0.857946	368.988	430.0845
WDR70	0.984097	0.015903	0.827875	0.827735	577.933	698.2124
SLF1	0.984098	0.015902	0.886202	0.885988	267.8497	302.3188
COG3	0.984099	0.015901	1.23905	1.239213	1036.945	836.7752
ESF1	0.984101	0.015899	0.80315	0.80305	902.4646	1123.798
MARCH2	0.984103	0.015897	1.185583	1.185818	533.4766	449.8792
ZNF649	0.984103	0.015897	0.934666	0.934482	193.3853	206.9444
C17orf53	0.984124	0.015876	0.91448	0.914269	208.945	228.5386
RWDD2A	0.984138	0.015862	0.900054	0.899841	244.5101	271.727
ZNF174	0.984141	0.015859	0.980409	0.980343	166.7114	170.0543
MOB1B	0.98415	0.01585	0.818124	0.818003	708.0346	865.5675
NBAS	0.984159	0.015841	0.791643	0.791561	1148.086	1450.41
TMEM57	0.984168	0.015832	0.82622	0.826083	590.1585	714.4081
GOSR2	0.984169	0.015831	1.260872	1.261004	1413.713	1121.099
SAYSD1	0.984181	0.015819	0.937632	0.937455	188.9396	201.5459
STAP2	0.984188	0.015812	1.195774	1.195993	610.1639	510.172
TXNL1	0.984195	0.015805	0.819167	0.819043	682.4055	833.1762
DPH2	0.984201	0.015799	1.255253	1.255392	1314.798	1047.319
SCD5	0.984219	0.015781	1.288183	1.288282	2160.425	1676.979
SS18	0.984223	0.015777	0.775405	0.775346	1692.021	2182.283
DNAJC3	0.984227	0.015773	1.296783	1.296874	2455.104	1893.091
CBX8	0.984231	0.015769	0.859703	0.859518	371.2108	431.884
EFNA4	0.984231	0.015769	0.961534	0.961414	175.6027	182.6509

HES4	0.984231	0.015769	0.961534	0.961414	175.6027	182.6509
CLCN3	0.984231	0.015769	0.774415	0.774356	1658.223	2141.425
IL17RA	0.984232	0.015768	0.846102	0.845936	446.7867	528.1581
FTO	0.984238	0.015762	1.232188	1.232355	996.112	808.2979
PPP1R3D	0.984243	0.015757	0.911819	0.911613	222.2819	243.8345
MRPL36	0.984246	0.015754	1.01949	1.019555	173.3799	170.0543
FUNDC1	0.984251	0.015749	0.849395	0.849224	427.8927	503.8647
DHX35	0.984253	0.015747	0.84219	0.842031	487.9088	579.4443
HLA-DMA	0.98426	0.01574	0.937436	0.937262	192.2739	205.1449
CNPY2	0.984264	0.015736	0.770188	0.770134	1871.614	2430.247
PRKAR2B	0.984267	0.015733	1.029261	1.029358	177.8255	172.7536
INTU	0.984272	0.015728	0.938525	0.938355	196.7195	209.6437
GLTP	0.98428	0.01572	0.81402	0.813907	759.2595	932.8604
TMEM43	0.984283	0.015717	0.78128	0.781214	1499.292	1919.184
LOC90768	0.984287	0.015713	1.014142	1.014189	173.3799	170.9541
LARP7	0.98429	0.01571	0.82827	0.828134	594.6041	718.0071
ELP6	0.984292	0.015708	0.919443	0.919245	213.3906	232.1376
LOC81691	0.984293	0.015707	0.955153	0.955019	185.6054	194.3478
ZFR	0.984293	0.015707	0.756165	0.756125	2702.948	3574.74
MTFR2	0.9843	0.0157	1.13266	1.132922	332.3115	293.3212
ZBED6	0.984303	0.015697	1.164056	1.164305	437.8954	376.099
ZZEF1	0.984303	0.015697	0.818955	0.818835	716.8592	875.4648
DRAM2	0.984303	0.015697	1.216504	1.216691	802.4377	659.5228
CAPN7	0.984313	0.015687	1.254111	1.254249	1319.243	1051.817
GCHFR	0.984319	0.015681	1.118758	1.11902	288.9665	258.2306
CYB561D1	0.984322	0.015678	0.935946	0.935771	196.1527	209.6167
SLC12A2	0.984323	0.015677	1.265299	1.265422	1583.759	1251.564
C1orf43	0.984324	0.015676	0.726255	0.726235	5932.082	8168.267
FBXL4	0.984326	0.015674	0.897741	0.897534	252.29	281.0935
MAST1	0.984327	0.015673	1.189594	1.189814	582.3786	489.4685
CENPI	0.984327	0.015673	1.195704	1.195915	642.3947	537.1557
KLHL26	0.984329	0.015671	1.113186	1.113443	284.5209	255.5314
PPAN-P2F	0.984334	0.015666	0.860719	0.860538	383.9698	446.1991
ACOT9	0.984338	0.015662	1.27551	1.275623	1754.916	1375.73
ZADH2	0.984344	0.015656	0.926614	0.926427	206.7222	223.1401
GUCY1B3	0.984375	0.015625	0.890301	0.890097	272.2953	305.9178
C17orf89	0.984377	0.015623	0.824569	0.824441	642.3947	779.1907
FER	0.984378	0.015622	0.798108	0.79802	1044.725	1309.148
HECTD2	0.984379	0.015621	1.027169	1.027257	181.1598	176.3526
AKAP10	0.984379	0.015621	0.84417	0.844011	472.3491	559.6497
IRF2BP1	0.984386	0.015614	0.827099	0.826967	616.8323	745.8996
NIPBL	0.984388	0.015612	0.767841	0.76779	2017.208	2627.294
RNF11	0.98439	0.01561	1.321144	1.321212	3637.644	2753.26
GNA12	0.984391	0.015609	1.258683	1.258813	1423.716	1130.996
KBTBD6	0.984393	0.015607	1.122343	1.122602	301.9922	269.0097
C9orf85	0.984405	0.015595	1.027442	1.02753	182.2712	177.3873
FAM127A	0.984407	0.015593	1.271295	1.271411	1703.569	1339.902
ANKRD13	0.984412	0.015588	0.870601	0.87041	336.7571	386.8961
SDHAF3	0.984412	0.015588	0.822255	0.822131	672.4028	817.8803
ZNF702P	0.984412	0.015588	0.944135	0.94398	197.8309	209.5717
KRIT1	0.984417	0.015583	0.786011	0.78594	1349.251	1716.739
DPY19L4	0.984423	0.015577	0.814022	0.813912	778.5535	956.56
MCU	0.984423	0.015577	1.295293	1.295383	2417.316	1866.099
SLX1A	0.984431	0.015569	1.115615	1.115868	296.0239	265.2847
SLX1B	0.984431	0.015569	1.115615	1.115868	296.0239	265.2847
AP2B1	0.984453	0.015547	1.3535	1.353546	5950.487	4396.219

RPP21	0.984458	0.015542	0.851772	0.851606	442.341	519.4215
PROSER3	0.984469	0.015531	1.024109	1.024185	183.3826	179.0519
PAIP2B	0.98447	0.01553	1.114882	1.115135	288.9665	259.1304
TRIM16L	0.984472	0.015528	1.279522	1.279627	1936.72	1513.501
TNNT1	0.984473	0.015527	1.31112	1.311195	3165.294	2414.052
DGKE	0.984474	0.015526	0.854443	0.854273	416.7008	487.786
DALRD3	0.984481	0.015519	1.071781	1.071981	218.9477	204.2451
CHD1	0.984481	0.015519	0.775814	0.775755	1673.783	2157.62
COPG2	0.984487	0.015513	0.873679	0.873488	330.0886	377.8985
SNAPIN	0.984487	0.015513	0.80957	0.809468	876.9022	1083.309
GSTA4	0.98449	0.01551	0.936886	0.93672	202.2765	215.942
C10orf76	0.984493	0.015507	0.835461	0.835319	550.1589	658.6231
RAI1	0.984494	0.015506	0.805601	0.805505	945.8096	1174.185
TCTN2	0.984499	0.015501	1.033214	1.033317	193.3853	187.1497
TMEM206	0.984505	0.015495	1.139685	1.139938	358.9853	314.9154
ZNF180	0.984506	0.015494	0.896499	0.896299	264.5155	295.1207
RBM34	0.984511	0.015489	0.786162	0.786092	1366.311	1738.108
RSBN1	0.984518	0.015482	1.131396	1.131651	327.8658	289.7222
EFCAB14	0.984518	0.015482	1.332127	1.332186	4291.152	3221.135
VSIG10	0.984519	0.015481	0.865616	0.865435	374.545	432.7837
IL6ST	0.98452	0.01548	0.739205	0.73918	4251.142	5751.165
ORAI3	0.984525	0.015475	0.974013	0.973934	182.2712	187.1497
GALNS	0.984542	0.015458	1.192933	1.193141	630.1692	528.1581
LUZP6	0.984544	0.015456	1.354799	1.354844	6162.21	4548.278
MTPN	0.984544	0.015456	1.354799	1.354844	6162.21	4548.278
WIZ	0.984548	0.015452	1.263099	1.26322	1554.862	1230.869
PEMT	0.984549	0.015451	0.858638	0.858465	400.1074	466.0748
COASY	0.984551	0.015449	0.809466	0.809364	849.1169	1049.118
ZNF559	0.984554	0.015446	1.125477	1.125729	313.9954	278.9251
NAB1	0.984554	0.015446	1.19135	1.191558	636.8377	534.4564
C5orf42	0.98456	0.01544	0.846045	0.845889	475.6833	562.3489
RAB43	0.984563	0.015437	1.264735	1.264855	1594.839	1260.885
PRDM15	0.984569	0.015431	0.881182	0.880991	317.8631	360.8031
FERMT1	0.984574	0.015426	1.145326	1.145573	383.4363	334.7101
VSIG10L	0.984575	0.015425	1.029268	1.029358	194.4967	188.9492
TRIM62	0.984584	0.015416	0.911504	0.91131	234.5074	257.3309
FBXO46	0.984587	0.015413	1.193571	1.193777	640.1719	536.2559
LGALSL	0.984587	0.015413	0.836587	0.836445	550.1477	657.7233
LINC0062	0.984593	0.015407	1.040584	1.040706	196.6973	189.0032
SORL1	0.984605	0.015395	1.297181	1.297266	2626.261	2024.456
WBSCR16	0.984619	0.015381	0.84112	0.840973	516.8055	614.5349
CCDC91	0.984622	0.015378	0.832507	0.832373	590.1585	709.0095
SNTB2	0.984624	0.015376	1.219081	1.219254	878.0136	720.1216
FBXO5	0.984627	0.015373	1.180998	1.181215	559.039	473.2729
LINC0049	0.984628	0.015372	0.861965	0.861792	400.1074	464.2753
ACAA1	0.984633	0.015367	0.815203	0.815094	791.3236	970.8392
ZSWIM8	0.984634	0.015366	0.797512	0.797429	1133.638	1421.618
ZNF674	0.984636	0.015364	1.020691	1.020754	193.3853	189.4531
WDFY1	0.984636	0.015364	1.285308	1.285405	2163.914	1683.448
GTF2H5	0.984638	0.015362	0.853487	0.853324	435.517	510.3789
LRRC59	0.984641	0.015359	0.726543	0.726524	6331.7	8715.059
SMIM22	0.984643	0.015357	1.04333	1.043458	205.6108	197.0471
DDHD2	0.984651	0.015349	1.236325	1.236476	1101.407	890.7607
XRCC1	0.984651	0.015349	0.810691	0.81059	861.3424	1062.615
JRK	0.984654	0.015346	0.869722	0.869541	356.7625	410.2898
IL6R	0.984656	0.015344	1.093563	1.093789	257.847	235.7367

RWDD3	0.984656	0.015344	0.883351	0.883162	316.4294	358.2928
SLC34A2	0.984658	0.015342	0.717316	0.717302	8341.129	11628.48
ZSCAN29	0.98466	0.01534	0.835859	0.835722	579.1333	692.9759
ATP6V0A2	0.984661	0.015339	0.798241	0.798158	1122.601	1406.493
NEMF	0.984666	0.015334	1.248611	1.248746	1334.803	1068.913
ZNF668	0.984667	0.015333	1.083142	1.083355	237.8417	219.541
B3GNTL1	0.984673	0.015327	0.947503	0.947362	201.1651	212.343
TMEM218	0.984674	0.015326	0.975849	0.975778	192.2739	197.0471
CEP78	0.984675	0.015325	0.850515	0.850358	469.0148	551.5518
RRP7BP	0.98468	0.01532	0.923017	0.922837	222.1374	240.7123
HSD17B7	0.98468	0.01532	1.021437	1.021502	190.7512	186.7358
ZDHHC7	0.984687	0.015313	1.304139	1.304218	2878.551	2207.107
NPIPB5	0.984688	0.015312	0.849006	0.84885	467.5033	550.7511
ANAPC10	0.98469	0.01531	0.878643	0.878457	325.643	370.7004
DRAM1	0.984694	0.015306	0.797083	0.797001	1125.858	1412.621
IFNLR1	0.9847	0.0153	1.159829	1.160063	452.8438	390.3601
ARMCX3	0.984704	0.015296	1.322263	1.322327	3796.575	2871.129
TXNDC9	0.984705	0.015295	0.818037	0.817925	762.427	932.1496
KLHL11	0.984711	0.015289	1.121142	1.121384	318.9079	284.3866
GK5	0.984713	0.015287	0.782678	0.782615	1523.442	1946.609
AHSA2	0.984716	0.015284	1.164102	1.164331	474.5719	407.5905
UPRT	0.98472	0.01528	0.948714	0.948577	202.2765	213.2427
RAD54L2	0.984723	0.015277	0.868251	0.868074	367.8766	423.7862
HCG11	0.984723	0.015277	1.066514	1.066694	224.3269	210.3005
ERAL1	0.984729	0.015271	0.803801	0.803711	993.6002	1236.268
ZBED4	0.984743	0.015257	0.799498	0.799414	1082.513	1354.136
THUMPD2	0.98475	0.01525	0.898219	0.898031	281.1866	313.1159
NDUFAF3	0.984752	0.015248	0.796102	0.796022	1138.083	1429.716
THUMPD3	0.984754	0.015246	0.968686	0.968597	197.8309	204.2451
DHCR7	0.984757	0.015243	1.310131	1.310203	3185.3	2431.147
TCEA2	0.984759	0.015241	0.84227	0.842126	535.6994	636.1291
STAU2	0.984761	0.015239	1.308541	1.308615	3190.857	2438.345
NR2C2	0.984771	0.015229	1.22853	1.228686	1043.614	849.3718
SAAL1	0.984773	0.015227	0.861369	0.861201	414.5558	481.3707
NAA38	0.984775	0.015225	0.855831	0.855669	433.4497	506.5639
MTL5	0.984776	0.015224	1.054053	1.054203	220.0591	208.7439
PFKFB3	0.984779	0.015221	1.240853	1.240995	1195.877	963.6411
FIGNL1	0.984779	0.015221	0.806374	0.806281	954.7008	1184.082
ALKBH2	0.984781	0.015219	0.936234	0.936076	215.6135	230.3381
ABHD14A	0.984782	0.015218	0.973272	0.973195	192.6406	197.9468
NFYB	0.984789	0.015211	0.846515	0.846365	493.4659	583.0434
LOC15077	0.984794	0.015206	1.047446	1.047582	206.2443	196.8761
ZNF276	0.984794	0.015206	1.055597	1.055751	222.2819	210.5434
ENGASE	0.984799	0.015201	1.205258	1.205442	764.6498	634.3296
MIER2	0.984809	0.015191	1.115066	1.115302	310.0833	278.0253
ZMYM4	0.98481	0.01519	0.7796	0.779541	1656	2124.329
SLC15A4	0.984815	0.015185	0.910569	0.910385	252.29	277.1256
C1orf52	0.984815	0.015185	1.231311	1.231463	1090.293	885.3622
PLPP6	0.984816	0.015184	0.942694	0.942549	211.1678	224.0398
UBE2V2	0.984833	0.015167	1.299049	1.29913	2734.068	2104.535
MED28	0.984833	0.015167	1.168651	1.168872	509.0256	435.483
PEBP1	0.984834	0.015166	0.755735	0.755699	2907.447	3847.367
RAB14	0.984836	0.015164	1.294951	1.295035	2575.136	1988.466
ANKRD13	0.984838	0.015162	0.993818	0.9938	196.7195	197.9468
P3H4	0.984842	0.015158	1.075822	1.076014	241.187	224.1478
XRCC4	0.984844	0.015156	1.062338	1.062505	225.6161	212.343

ATAD5	0.984848	0.015152	0.885176	0.884992	314.5289	355.4045
CASP7	0.98485	0.01515	1.225452	1.22561	991.3773	808.8827
FNBP1L	0.984853	0.015147	1.318707	1.318772	3684.323	2793.75
FBXW9	0.984857	0.015143	0.891558	0.891373	296.7464	332.9106
PARN	0.984868	0.015132	0.782622	0.782559	1537.079	1964.172
C12orf10	0.984872	0.015128	0.838509	0.838372	563.4847	672.1195
PRELID2	0.984874	0.015126	1.135084	1.13532	370.8107	326.6123
MMP24-AS	0.984875	0.015125	0.849994	0.849842	477.9061	562.3489
IFIT1	0.984878	0.015122	0.898216	0.898031	281.1866	313.1159
SLC19A1	0.984884	0.015116	1.160785	1.161008	486.7974	419.2874
LOC44043	0.984886	0.015114	0.947624	0.94749	215.5912	227.5399
ZNF607	0.984886	0.015114	1.053274	1.05342	218.9477	207.8442
KNOP1	0.984894	0.015106	0.83199	0.831863	625.7236	752.1979
TAF1C	0.984895	0.015105	0.853253	0.853097	459.0122	538.0555
PCMT1	0.984899	0.015101	1.278839	1.278937	2113.901	1652.856
ARHGAP8	0.984918	0.015082	1.204408	1.204589	773.8634	642.4274
SLC25A17	0.984925	0.015075	0.835197	0.835067	614.6095	736.0023
ANKRD12	0.984926	0.015074	0.820033	0.819923	766.5614	934.9209
SAMD1	0.984933	0.015067	1.18728	1.187479	635.7263	535.3562
ATG7	0.984937	0.015063	1.191273	1.191468	665.7343	558.7499
TSTD2	0.984946	0.015054	0.848329	0.848182	509.0256	600.1388
CCNJL	0.984948	0.015052	1.252912	1.253036	1485.955	1185.881
SNX11	0.98495	0.01505	0.86074	0.860578	421.2242	489.4685
LATS1	0.984962	0.015038	0.83302	0.832893	623.5008	748.5989
DDI2	0.984968	0.015032	0.873957	0.873784	361.9861	414.2757
POLR3E	0.98497	0.01503	0.786888	0.786822	1454.835	1849.003
FAM136A	0.984977	0.015023	0.764846	0.764803	2385.085	3118.562
CSGALNA	0.984984	0.015016	1.177467	1.177672	591.2699	502.0651
PLOD3	0.984985	0.015015	1.352896	1.352939	6412.833	4739.927
HDAC8	0.984988	0.015012	1.167225	1.16744	516.8055	442.6811
RGMB	0.984994	0.015006	1.201614	1.201796	759.0927	631.6303
RNF166	0.984994	0.015006	0.93106	0.930902	231.1732	248.3333
TSSC1	0.985	0.015	0.864829	0.864665	404.5531	467.8743
MYPOP	0.985006	0.014994	0.995986	0.995975	198.9423	199.7463
LOC72873	0.985006	0.014994	1.042202	1.042318	215.7024	206.9444
RNF168	0.985007	0.014993	0.913174	0.913	264.5155	289.7222
CNOT2	0.985008	0.014992	0.793317	0.793244	1282.567	1616.866
NUTM2B-A	0.985012	0.014988	0.881292	0.881117	346.8932	393.6982
R3HCC1L	0.985013	0.014987	0.864592	0.864429	406.7759	470.5736
RIMS4	0.985022	0.014978	1.101019	1.101236	292.3007	265.4287
EIF2S3	0.98503	0.01497	1.391036	1.391062	12036.84	8652.985
SLC25A38	0.985031	0.014969	0.820904	0.820796	790.2122	962.7414
KIF20B	0.985031	0.014969	1.253837	1.253959	1488.177	1186.781
MBD5	0.985035	0.014965	1.102811	1.103029	296.7464	269.0277
ZNF616	0.985036	0.014964	0.885428	0.885252	334.5343	377.8985
ZDHHC2	0.98504	0.01496	0.977507	0.977445	202.2765	206.9444
C10orf11	0.985044	0.014956	1.044258	1.044378	218.9477	209.6437
RRAGD	0.985045	0.014955	0.814697	0.814598	882.4592	1083.309
CYB5D1	0.985047	0.014953	1.175437	1.175643	570.1531	484.9697
ZDHHC18	0.985048	0.014952	1.261379	1.261493	1654.889	1311.848
TIMM22	0.985049	0.014951	0.887416	0.887238	314.5289	354.5048
RSBN1L	0.98505	0.01495	1.157459	1.157679	464.5692	401.2922
TJP3	0.985051	0.014949	0.817474	0.817371	841.3371	1029.324
ADGRA3	0.985052	0.014948	0.823191	0.823079	743.533	903.3573
SNAP25	0.985059	0.014941	1.035393	1.03549	218.9477	211.4432
TCP11L1	0.985059	0.014941	0.999416	0.999414	197.8309	197.9468

GLYR1	0.985062	0.014938	0.784098	0.784037	1568.199	2000.163
TBC1D3E	0.985064	0.014936	0.985768	0.985728	206.8556	209.8506
FBXL20	0.985067	0.014933	0.911625	0.91145	258.8917	284.0447
BEND7	0.985073	0.014927	1.023727	1.023794	204.4994	199.7463
C15orf41	0.985074	0.014926	0.934438	0.934287	231.1732	247.4335
GSG2	0.985075	0.014925	0.882902	0.882727	336.7571	381.4975
REV1	0.985079	0.014921	0.829155	0.829035	664.6229	801.6846
DOK7	0.98508	0.01492	0.946367	0.946236	225.6161	238.436
COX19	0.985086	0.014914	1.129222	1.129448	367.8766	325.7125
SLC26A6	0.985094	0.014906	0.937679	0.937534	234.5074	250.1328
TRMT61B	0.985096	0.014904	0.880518	0.880346	355.6511	403.9915
TUBD1	0.985097	0.014903	0.967146	0.967059	208.167	215.2582
LNX2	0.985098	0.014902	0.825598	0.825484	720.4824	872.8015
TMEM126	0.985099	0.014901	0.916013	0.915844	264.5155	288.8224
PPP2R5A	0.985102	0.014898	0.756432	0.756397	2986.358	3948.139
PKIG	0.985107	0.014893	1.109587	1.109804	324.5316	292.4214
NOD1	0.985115	0.014885	1.061445	1.061602	237.8417	224.0398
NDUFS6	0.985128	0.014872	0.866027	0.865864	401.2189	463.3755
TRAK1	0.985135	0.014865	0.784654	0.784592	1569.31	2000.163
PSMD4	0.98514	0.01486	0.750615	0.750584	3478.656	4634.601
PEX14	0.985144	0.014856	1.148223	1.148443	441.2296	384.1968
CNKSR1	0.985145	0.014855	0.872241	0.872076	392.3276	449.8792
STX8	0.985146	0.014854	0.868356	0.868194	410.1101	472.3731
TMEM63C	0.985148	0.014852	0.83904	0.838911	604.6068	720.7064
ERCC6L2	0.985148	0.014852	0.925363	0.925203	241.5871	261.1189
VKORC1	0.98515	0.01485	1.24795	1.248075	1403.71	1124.698
CLK4	0.985154	0.014846	0.941487	0.94135	227.839	242.035
C3orf14	0.985154	0.014846	0.899453	0.899279	294.5235	327.512
ACACA	0.985162	0.014838	1.319728	1.319789	3937.724	2983.599
UBA6-AS1	0.985162	0.014838	0.993574	0.993556	205.6108	206.9444
DDX17	0.985164	0.014836	0.728765	0.728747	6300.581	8645.778
ZNF576	0.985165	0.014835	0.932964	0.932815	243.3987	260.9299
CTDP1	0.98517	0.01483	1.065341	1.065502	251.1786	235.7367
MID1IP1	0.985176	0.014824	0.789075	0.789009	1440.42	1825.61
GTF2E1	0.985181	0.014819	0.820274	0.820169	799.8592	975.239
CTNND1	0.985195	0.014805	1.375412	1.375443	9545.897	6940.232
CCDC8	0.985209	0.014791	0.803769	0.803686	1082.513	1346.938
MBNL2	0.985222	0.014778	1.274533	1.27463	2049.439	1607.868
RAB17	0.985229	0.014771	1.15357	1.153784	472.3491	409.39
ARL2	0.985231	0.014769	0.817189	0.817089	851.3397	1041.92
AMN1	0.985236	0.014764	1.109975	1.110189	325.643	293.3212
PREP	0.985239	0.014761	1.198568	1.198745	766.8726	639.7282
ZNF101	0.985244	0.014756	1.025389	1.025457	222.0485	216.5358
SLC25A43	0.985246	0.014754	1.191126	1.191309	723.5276	607.3369
LRP5	0.985247	0.014753	1.280345	1.280437	2235.045	1745.531
DERA	0.985247	0.014753	1.227819	1.227963	1126.969	917.7535
AXIN1	0.985247	0.014753	0.822811	0.822704	775.7639	942.9467
ARL4A	0.985247	0.014753	1.11149	1.111704	330.0886	296.9202
CCNE1	0.98525	0.01475	0.78617	0.786109	1550.416	1972.27
TOR2A	0.98525	0.01475	0.953961	0.953849	222.2819	233.0374
MICALL2	0.98525	0.01475	0.935269	0.935125	238.9531	255.5314
COPRS	0.985255	0.014745	0.835897	0.835773	630.1692	753.9975
PHF13	0.985262	0.014738	1.226241	1.226388	1079.179	879.9636
INTS12	0.985265	0.014735	0.860557	0.860405	449.0095	521.8598
LYPLA2	0.985269	0.014731	1.272806	1.272904	2068.333	1624.892
GRB14	0.985271	0.014729	1.033576	1.033665	222.2819	215.0422

ORC1	0.985272	0.014728	0.875329	0.875167	388.9934	444.4806
LOC10013	0.985273	0.014727	1.006849	1.006868	211.7902	210.3455
MDM4	0.985277	0.014723	0.885747	0.885579	350.1718	395.4168
POLM	0.985289	0.014711	0.92865	0.928499	248.9557	268.128
MCM2	0.985289	0.014711	0.762591	0.762552	2651.823	3477.566
FASTKD5	0.985294	0.014706	1.239383	1.239514	1285.901	1037.421
TDRP	0.985299	0.014701	0.90843	0.908261	277.8524	305.9178
GFER	0.985302	0.014698	1.118548	1.118763	352.3168	314.9154
ZUFSP	0.985308	0.014692	0.950297	0.95018	233.396	245.634
SNUPN	0.985319	0.014681	1.148966	1.149178	460.1236	400.3924
CCDC183	0.985322	0.014678	0.965725	0.965639	218.9477	226.7391
NUP54	0.985325	0.014675	0.822469	0.822364	812.4404	987.9346
TNFAIP1	0.985335	0.014665	1.259161	1.259271	1688.231	1340.64
ARHGEF9	0.985337	0.014663	1.148335	1.148545	471.2377	410.2898
ZNF354B	0.985337	0.014663	0.96076	0.960664	227.7167	237.0413
RAB40B	0.985347	0.014653	0.961366	0.961271	222.2819	231.2379
CTSL	0.985348	0.014652	0.818171	0.818072	839.1142	1025.724
MRFAP1L	0.985351	0.014649	0.822262	0.822158	798.181	970.8392
CDK10	0.985361	0.014639	0.929715	0.929567	246.7329	265.4287
CAMK1D	0.985365	0.014635	0.90587	0.905705	300.0917	331.336
PCSK7	0.985365	0.014635	1.237215	1.237346	1299.238	1050.018
THAP1	0.985368	0.014632	1.025125	1.025191	227.839	222.2403
RRM2B	0.98537	0.01463	1.230503	1.230641	1192.542	969.0397
TBC1D22A	0.985373	0.014627	0.85809	0.857944	485.6971	566.1189
GALC	0.985374	0.014626	0.899108	0.898941	308.9719	343.7077
TAF1A	0.985375	0.014625	1.107115	1.107319	336.7571	304.1183
GNPDA2	0.985378	0.014622	1.090926	1.091118	294.5235	269.9275
AGAP6	0.985383	0.014617	1.017336	1.017381	225.1605	221.3136
E2F8	0.985383	0.014617	1.10824	1.108444	340.0913	306.8176
GORAB	0.985384	0.014616	0.88942	0.889255	345.6484	388.6956
SKA2	0.985386	0.014614	0.820586	0.820485	848.0055	1033.543
ZC3H8	0.985386	0.014614	0.918086	0.917927	270.0725	294.221
CHP1	0.985387	0.014613	1.28619	1.286275	2489.769	1935.641
PDK1	0.985413	0.014587	0.824358	0.824253	800.2149	970.8392
TMEM120	0.985413	0.014587	0.844938	0.844806	557.9276	660.4226
ZSCAN30	0.985414	0.014586	0.992174	0.992154	222.2819	224.0398
TUBA1C	0.985415	0.014585	1.404483	1.404503	16442.66	11707.1
R3HDM4	0.985416	0.014584	0.834851	0.834732	660.1773	790.8875
DHRS1	0.985419	0.014581	1.043952	1.044063	236.7302	226.7391
PPP1R3B	0.985421	0.014579	1.277758	1.277849	2220.318	1737.541
KMT2E	0.985422	0.014578	0.761588	0.761551	2728.511	3582.838
PNPLA8	0.985425	0.014575	1.314909	1.314971	3922.164	2982.699
DLEU1	0.985427	0.014573	1.097347	1.097543	310.0833	282.5241
PRKAR2A	0.985429	0.014571	0.834248	0.83413	667.9572	800.7849
METTL10	0.985435	0.014565	0.881332	0.88117	367.8766	417.4879
CTU1	0.985437	0.014563	0.899391	0.899226	312.3061	347.3067
KIF15	0.985438	0.014562	1.175712	1.175903	616.8323	524.5591
FZD4	0.985438	0.014562	0.959275	0.959178	227.839	237.5362
FBXO41	0.98544	0.01456	1.02255	1.022608	224.5047	219.541
KIAA0895	0.985446	0.014554	0.887	0.886836	342.3142	385.9963
FASTKD1	0.985446	0.014554	0.81725	0.817155	908.0216	1111.202
MTIF3	0.985448	0.014552	1.134617	1.134827	414.5558	365.3019
NUDT4	0.985449	0.014551	1.164812	1.165011	551.7259	473.5788
PDCL3	0.98545	0.01455	0.856503	0.856362	503.124	587.5152
EIF2B1	0.985454	0.014546	0.803811	0.803732	1130.304	1406.322
KATNBL1	0.985456	0.014544	0.901122	0.900957	303.4148	336.7705

ADNP2	0.985457	0.014543	0.852213	0.852075	519.0283	609.1364
PIDD1	0.985458	0.014542	0.993245	0.993227	218.9477	220.4408
SARS2	0.985464	0.014536	0.973998	0.973933	227.839	233.9372
ACER3	0.98547	0.01453	1.22024	1.220387	1039.99	852.1791
NCOA2	0.985471	0.014529	0.809015	0.808929	1029.165	1272.258
TM9SF2	0.985473	0.014527	1.331692	1.331742	5106.927	3834.77
ZNF510	0.985474	0.014526	1.103621	1.10382	326.7544	296.0205
ZSWIM1	0.985476	0.014524	1.009317	1.009341	227.1499	225.0475
LOC64621	0.985481	0.014519	0.981982	0.981936	221.0705	225.1375
PIK3CG	0.985491	0.014509	1.105973	1.106174	333.4229	301.419
MGST2	0.985491	0.014509	1.192764	1.192938	752.4243	630.7306
MRPL11	0.985498	0.014502	0.79756	0.797489	1318.132	1652.856
BRMS1	0.985498	0.014502	0.805073	0.804993	1112.521	1382.029
MTRF1L	0.9855	0.0145	1.177397	1.177584	635.7263	539.855
TP73-AS1	0.985501	0.014499	1.125386	1.125592	397.8846	353.488
INVS	0.98551	0.01449	0.914369	0.914213	291.1893	318.5144
MINCR	0.98551	0.01449	1.061878	1.062023	258.9584	243.8345
STIL	0.985511	0.014489	1.254289	1.2544	1672.671	1333.442
ELP5	0.985513	0.014487	0.854869	0.85473	506.8028	592.9407
FBXW8	0.985514	0.014486	0.886429	0.886267	355.6511	401.2922
AAED1	0.985524	0.014476	1.052107	1.052232	255.6242	242.9347
ULK3	0.985537	0.014463	0.894436	0.894274	332.3115	371.6002
ADA	0.98554	0.01446	1.185568	1.185746	719.082	606.4371
ZNF707	0.985543	0.014457	0.865652	0.865505	457.9007	529.0579
KIAA1586	0.985546	0.014454	1.002275	1.00228	223.6489	223.1401
ZNF611	0.985546	0.014454	0.842563	0.842438	603.6621	716.5675
ZFYVE19	0.985549	0.014451	1.152009	1.152208	509.0256	441.7813
TLE3	0.985552	0.014448	1.211587	1.21174	974.7062	804.3839
FADS3	0.985557	0.014443	1.328229	1.328281	4791.287	3607.131
HPRT1	0.985557	0.014443	1.286984	1.287065	2594.03	2015.459
GRPEL1	0.985558	0.014442	1.236137	1.236264	1327.023	1073.412
ZRSR2	0.985561	0.014439	1.067849	1.068003	269.1612	252.0223
FOXRED2	0.985562	0.014438	0.80231	0.802234	1185.885	1478.231
NCOA7	0.985563	0.014437	0.809682	0.809597	1014.717	1253.363
POLR1C	0.985565	0.014435	1.144441	1.144644	463.4578	404.8912
NDUFS4	0.985568	0.014432	1.204455	1.204616	874.6793	726.105
POLE4	0.985573	0.014427	1.17926	1.179443	657.9545	557.8501
OSBPL10	0.985575	0.014425	1.275952	1.276043	2223.931	1742.832
TOM1	0.985578	0.014422	1.211745	1.211896	981.3747	809.7825
C21orf91	0.985584	0.014416	0.801647	0.801572	1205.879	1504.396
SLC29A4	0.985587	0.014413	0.964827	0.964744	237.8417	246.5338
GOLGA5	0.985591	0.014409	0.772065	0.77202	2239.49	2900.821
CDK16	0.985592	0.014408	0.737175	0.737154	5345.88	7252.052
FAM83B	0.985598	0.014402	1.027216	1.027284	240.3201	233.9372
TAF13	0.985601	0.014399	1.190139	1.190311	765.7612	643.3272
KIAA0556	0.985604	0.014396	1.203703	1.203862	895.7961	744.1001
PCBP1	0.98561	0.01439	0.74889	0.748863	4029.971	5381.454
KLK5	0.985622	0.014378	0.86997	0.869821	431.2269	495.7668
ALS2CL	0.985623	0.014377	1.21875	1.218893	1078.067	884.4624
NIT1	0.985626	0.014374	0.822496	0.822397	836.8914	1017.627
EXOSC3	0.985627	0.014373	1.191625	1.191795	762.427	639.7282
ZNF710	0.98563	0.01437	1.137808	1.13801	442.341	388.6956
CBWD2	0.985632	0.014368	1.220656	1.220798	1077.5	882.6179
TMEM60	0.985634	0.014366	0.979876	0.979827	234.5074	239.3357
GREB1L	0.985635	0.014365	0.98634	0.986307	233.396	236.6364
KRAS	0.985641	0.014359	0.790475	0.790414	1558.196	1971.37

PTCH1	0.985642	0.014358	0.989177	0.98915	227.839	230.3381
NDUFAF2	0.985646	0.014354	0.833927	0.833815	717.9706	861.0687
XIAP	0.985654	0.014346	0.788371	0.788312	1630.882	2068.832
BAGE4	0.985654	0.014346	0.901986	0.137018	0	0.062983
NOP2	0.985656	0.014344	0.813931	0.813843	1000.269	1229.07
IAH1	0.985657	0.014343	1.064119	1.064263	276.741	260.0302
CYB5R2	0.985664	0.014336	1.093548	1.093731	317.8631	290.6219
RAB2B	0.985665	0.014335	1.089939	1.090117	318.7745	292.4214
MRPL52	0.985666	0.014334	0.829342	0.829235	754.3693	909.7186
CEP128	0.985666	0.014334	0.891963	0.891806	357.8739	401.2922
NSMCE3	0.985671	0.014329	0.901043	0.900886	323.4202	359.0036
MEX3A	0.985674	0.014326	0.813489	0.813402	985.8203	1211.974
SLC12A4	0.98569	0.01431	1.206415	1.206571	898.897	744.9999
WDR24	0.985691	0.014309	0.910679	0.910525	302.3034	332.0108
DXO	0.985693	0.014307	0.922017	0.921871	281.1866	305.0181
ALDH18A1	0.985697	0.014303	1.334292	1.334339	5521.483	4137.988
SELO	0.985702	0.014298	1.130152	1.13035	431.2269	381.4975
PNN	0.985708	0.014292	1.302214	1.302282	3324.226	2552.614
ZNF317	0.985715	0.014285	0.795918	0.795851	1354.808	1702.343
CCS	0.985721	0.014279	0.855859	0.855724	521.2511	609.1364
CCDC92	0.985723	0.014277	0.931371	0.931236	268.9611	288.8224
TK2	0.985723	0.014277	0.986375	0.986342	237.8417	241.1352
EFCAB11	0.985724	0.014276	0.888679	0.888524	363.5198	409.1291
FAM118B	0.985725	0.014275	0.830125	0.830019	753.5357	907.8561
CTBP1	0.98573	0.01427	1.236134	1.236258	1334.803	1079.71
FGFR1	0.985732	0.014268	0.816536	0.816445	944.6981	1157.089
SPATA31I	0.985733	0.014267	1.019427	2.11141	0.011114	0
FDFT1	0.985735	0.014265	1.301601	1.301669	3309.778	2542.717
TOP3A	0.985736	0.014264	0.840221	0.840103	647.7962	771.0929
TMEM11	0.985737	0.014263	1.145152	1.145346	495.6887	432.7837
CDC37L1	0.985738	0.014262	1.230711	1.230841	1247.002	1013.128
FBXL19	0.98574	0.01426	1.203706	1.203862	895.7961	744.1001
CDC16	0.985741	0.014259	0.784522	0.784468	1794.926	2288.085
SMIM10L1	0.985743	0.014257	0.900661	0.900507	337.8685	375.1992
TRIM39	0.985746	0.014254	0.91794	0.917794	298.9692	325.7485
DNM3	0.985747	0.014253	1.104457	1.104646	347.8712	314.9154
C6orf1	0.98575	0.01425	0.98634	0.986307	233.396	236.6364
MITD1	0.985756	0.014244	1.208132	1.208283	946.921	783.6895
POLR2J3	0.985757	0.014243	0.83163	0.831523	740.4766	890.5088
NECAP1	0.985759	0.014241	1.179428	1.179604	683.5169	579.4443
NUDT4P2	0.985759	0.014241	1.202665	1.202821	888.1385	738.3777
CMTR2	0.985764	0.014236	0.861296	0.861157	491.243	570.4468
DDT	0.985767	0.014233	0.793745	0.793681	1454.691	1832.844
NKIRAS1	0.98577	0.01423	0.855296	0.855162	520.1397	608.2366
NDUFS1	0.985773	0.014227	0.772965	0.772921	2265.053	2930.513
FNIP1	0.985775	0.014225	0.794584	0.794519	1432.607	1803.116
GABPB1	0.985784	0.014216	0.846781	0.846657	595.7155	703.611
PPP1R14I	0.985785	0.014215	0.769578	0.769537	2451.77	3186.035
RAPGEF6	0.985792	0.014208	0.840247	0.84013	654.6202	779.1907
CHURC1-	0.985795	0.014205	1.140788	1.140983	469.7261	411.6844
SNAPC2	0.985806	0.014194	1.186227	1.186398	729.0847	614.5349
RPA3	0.985808	0.014192	0.858795	0.858661	524.5853	610.9359
PPP3CC	0.98581	0.01419	1.047151	1.047259	260.0698	248.3333
HSPA9	0.985813	0.014187	0.684891	0.684886	28373.18	41427.57
ZNRF2	0.985827	0.014173	0.854245	0.854115	557.9276	653.2245
INO80B	0.985829	0.014171	0.99982	0.99982	237.2415	237.2843

CPSF1	0.985839	0.014161	1.308266	1.308328	3719.888	2843.236
NAIF1	0.985849	0.014151	0.942717	0.942601	269.1056	285.4933
PPP1R10	0.985851	0.014149	0.80593	0.805854	1168.091	1449.511
SHISA4	0.985855	0.014145	1.171908	1.172085	660.1773	563.2487
RWDD4	0.985858	0.014142	0.826503	0.826404	840.2256	1016.727
ING2	0.985861	0.014139	0.871561	0.871418	444.5638	510.163
FAM225B	0.985864	0.014136	1.007083	1.151206	0.022228	0.017995
INIP	0.985866	0.014134	1.127695	1.127888	425.7921	377.5116
ATP11B	0.985871	0.014129	1.280981	1.281063	2516.231	1964.172
CHST2	0.98588	0.01412	0.963218	0.963136	247.8443	257.3309
AZIN1	0.985883	0.014117	1.37245	1.372479	10191.63	7425.705
WDR12	0.985884	0.014116	0.808377	0.808298	1123.635	1390.127
PIGO	0.985887	0.014113	0.832681	0.832575	746.8672	897.059
MTCH2	0.985895	0.014105	0.784397	0.784344	1803.818	2299.782
REEP6	0.985895	0.014105	0.980318	0.980273	247.8443	252.8321
ATG4B	0.985896	0.014104	1.224823	1.224954	1192.542	973.5385
NAT9	0.985897	0.014103	1.177733	1.177905	707.9679	601.0385
GXYLT1	0.985907	0.014093	0.776647	0.776601	2190.588	2820.742
RNGTT	0.985907	0.014093	0.836248	0.836138	710.1907	849.3718
TADA1	0.985907	0.014093	0.94608	0.94597	268.9611	284.3236
CCDC64	0.985911	0.014089	0.996459	0.996451	241.1759	242.035
BIN3	0.985911	0.014089	1.019364	1.019409	246.7329	242.035
METTL5	0.985914	0.014086	0.789655	0.789597	1622.658	2055.048
INTS8	0.985918	0.014082	0.790301	0.790242	1602.653	2028.055
CASKIN2	0.985918	0.014082	0.849952	0.849828	585.7128	689.2149
BICD1	0.985924	0.014076	1.067734	1.067875	297.8578	278.9251
PSMD5	0.985924	0.014076	0.799889	0.79982	1327.023	1659.154
GMDS	0.98593	0.01407	1.24547	1.24558	1604.875	1288.454
ACTR2	0.985934	0.014066	1.362869	1.362901	8803.475	6459.365
FBXO45	0.985936	0.014064	1.210741	1.210884	1011.094	835.0027
C14orf142	0.985945	0.014055	0.883482	0.883336	410.1101	464.2753
CTXN1	0.985954	0.014046	0.890545	0.890398	384.5477	431.884
ARID3A	0.985955	0.014045	0.994556	0.994543	243.3987	244.7343
DOCK11	0.985962	0.014038	1.084427	1.08459	320.086	295.1207
NUDT1	0.985967	0.014033	1.007311	1.007329	245.6215	243.8345
ATAD3A	0.985967	0.014033	0.818398	0.818309	956.3235	1168.66
TMEM183	0.98597	0.01403	1.163111	1.163289	617.3436	530.6864
GTSE1	0.985973	0.014027	1.19901	1.199163	886.9048	739.6013
FUOM	0.985975	0.014025	0.931908	0.931781	290.0779	311.3164
CEP152	0.985982	0.014018	1.160185	1.160366	585.7128	504.7644
UBXN7	0.985983	0.014017	1.191012	1.191171	811.329	681.117
COX17	0.985989	0.014011	1.26413	1.264223	2083.893	1648.357
TRIM2	0.985989	0.014011	0.786795	0.786741	1744.913	2217.904
RNF135	0.985991	0.014009	0.897259	0.897113	365.6538	407.5905
MIEF2	0.985992	0.014008	1.088011	1.088177	328.9772	302.3188
C17orf75	0.985992	0.014008	0.980697	0.980653	246.8552	251.7254
ZW10	0.985999	0.014001	0.820094	0.820005	959.1465	1169.686
CCDC138	0.986001	0.013999	0.966961	0.966889	252.29	260.9299
CDC42BP	0.986006	0.013994	1.248558	1.248665	1652.666	1323.544
FAM200B	0.986009	0.013991	1.055144	1.055262	286.7437	271.727
BTN2A2	0.986012	0.013988	0.853521	0.853395	558.5167	654.4662
PMF1	0.98602	0.01398	0.88262	0.882477	419.9461	475.8732
XPO6	0.986024	0.013976	0.737611	0.737592	5684.86	7707.33
KLF4	0.986028	0.013972	0.991684	0.991665	253.4014	255.5314
SLC25A11	0.986028	0.013972	0.804377	0.804304	1242.556	1544.885
MGC7208	0.98603	0.01397	0.925103	0.92497	296.6463	320.7099

TESK1	0.986033	0.013967	1.187935	1.188095	812.4404	683.8163
YAF2	0.986034	0.013966	1.181786	1.181952	740.1988	626.2498
RBM12B	0.986038	0.013962	1.206294	1.206439	978.0404	810.6822
RBM4B	0.986039	0.013961	0.958267	0.95818	259.5697	270.8992
GAB1	0.986041	0.013959	0.884543	0.884399	403.4417	456.1775
OIP5	0.986055	0.013945	0.988212	0.988185	248.9557	251.9323
TRAPPC9	0.986056	0.013944	0.820045	0.819956	945.8096	1153.49
TRMT11	0.986056	0.013944	1.126544	1.126728	450.1209	399.4927
GTF3C5	0.986057	0.013943	1.199954	1.200104	911.3559	759.396
ZNF584	0.986059	0.013941	0.851899	0.851777	602.384	707.21
SARAF	0.98606	0.01394	1.288519	1.288592	2898.556	2249.396
C18orf25	0.986061	0.013939	1.27536	1.275444	2361.745	1851.703
KIF1C	0.986061	0.013939	1.329068	1.329115	5199.185	3911.762
NUP205	0.986063	0.013937	0.734528	0.734511	6379.491	8685.367
PTK7	0.986064	0.013936	1.337981	1.338024	6076.076	4541.08
FAM156A	0.986068	0.013932	1.120722	1.120904	430.649	384.1968
FIGN	0.986074	0.013926	0.865572	0.865439	496.8001	574.0458
NOB1	0.986074	0.013926	0.833886	0.833783	780.2095	935.7486
PSMG4	0.986076	0.013924	0.871947	0.87181	474.5719	544.3538
SH3BP4	0.986082	0.013918	1.308425	1.308484	3891.045	2973.701
CDCA4	0.986083	0.013917	1.200244	1.200393	919.1357	765.6943
ABHD12	0.986083	0.013917	0.824622	0.824529	881.3478	1068.913
POLE2	0.986093	0.013907	0.98647	0.98644	251.1786	254.6316
MDK	0.986096	0.013904	0.781937	0.781888	1970.529	2520.223
BRAP	0.986097	0.013903	0.848485	0.848367	619.0551	729.704
GATC	0.986106	0.013894	0.841993	0.841881	678.8379	806.3364
COPS3	0.986109	0.013891	0.785718	0.785665	1823.823	2321.376
PPIL4	0.986109	0.013891	0.848393	0.848275	621.278	732.4033
EHD3	0.986113	0.013887	1.103646	1.103821	373.4336	338.3091
LMBR1L	0.986117	0.013883	0.911637	0.911496	322.3088	353.605
ABCC5	0.986117	0.013883	1.186311	1.186469	811.329	683.8163
VASP	0.986118	0.013882	1.271192	1.271277	2351.743	1849.903
SDF2	0.986123	0.013877	1.179393	1.179556	753.5357	638.8284
RECQL	0.986125	0.013875	1.235113	1.235229	1418.159	1148.092
BNC2	0.986126	0.013874	1.10537	1.105545	378.9907	342.8079
AGO4	0.986126	0.013874	0.860831	0.860703	531.2538	617.2342
FANCL	0.986129	0.013871	1.105709	1.105885	380.1021	343.7077
FBXO17	0.986133	0.013867	0.826792	0.826698	859.1196	1039.221
TMEM62	0.986137	0.013863	1.027887	1.027948	270.0725	262.7294
EDEM3	0.98614	0.01386	1.293199	1.293267	3145.289	2432.047
ILF3	0.986141	0.013859	0.732266	0.732249	6719.582	9176.635
RASGEF1	0.986143	0.013857	0.868565	0.868431	486.7974	560.5494
FAM46A	0.986144	0.013856	0.938454	0.938338	284.5209	303.2185
PLGLB2	0.986146	0.013854	0.984714	0.526383	0	0.008998
PCTP	0.986146	0.013854	0.887736	0.887594	400.1074	450.7789
CYB5R3	0.986147	0.013853	1.313242	1.313298	4229.225	3220.307
CENPH	0.986148	0.013852	1.108758	1.108933	400.1074	360.8031
PPP2CA	0.986149	0.013851	0.742759	0.742738	5060.248	6812.97
INTS9	0.986152	0.013848	0.917738	0.917603	317.8631	346.4069
PTBP2	0.986155	0.013845	1.027857	1.027919	264.5155	257.3309
ZNF337	0.986159	0.013841	0.964353	0.964279	268.9611	278.9251
RPL23AP7	0.986159	0.013841	1.103222	1.103395	375.9899	340.7565
BCAS3	0.986159	0.013841	0.989073	0.989049	254.5128	257.3309
PDSS2	0.986161	0.013839	0.974179	0.974123	257.9026	264.7539
SPNS1	0.986166	0.013834	0.806464	0.806392	1258.116	1560.181
TEAD2	0.986167	0.013833	0.796486	0.796424	1482.62	1861.6

CLDN10	0.986169	0.013831	1.008244	1.008263	256.7356	254.6316
SRGAP3	0.986169	0.013831	1.036188	1.036266	277.8524	268.128
LPAR2	0.986172	0.013828	1.082038	1.082191	330.0886	305.0181
TMEM99	0.98618	0.01382	0.9416	0.941489	277.8524	295.1207
GSTM4	0.986195	0.013805	1.140123	1.140303	506.8028	444.4446
TMEM38B	0.986195	0.013805	1.195982	1.19613	918.0243	767.4938
ZNF408	0.986197	0.013803	1.019238	1.019281	262.2927	257.3309
KIAA0040	0.986203	0.013797	1.216645	1.216776	1172.537	963.6411
DDX55	0.986206	0.013794	0.873533	0.873398	466.792	534.4564
SHROOM	0.986211	0.013789	1.143997	1.144174	540.2006	472.1302
CIDECP	0.986212	0.013788	0.959959	0.959877	263.4152	274.4263
MNT	0.986215	0.013785	1.130369	1.130548	480.1289	424.6859
TICRR	0.986218	0.013782	0.893715	0.893574	377.8793	422.8864
TMEM222	0.986222	0.013778	0.864611	0.864483	531.2538	614.5349
RAB3IP	0.986222	0.013778	0.805607	0.805536	1259.216	1563.204
TOPORS	0.986223	0.013777	1.212131	1.212265	1114.744	919.553
CAPRIN2	0.986228	0.013772	1.118244	1.118421	425.6699	380.5978
KCTD11	0.986229	0.013771	1.104379	1.104549	394.5504	357.204
C10orf88	0.986231	0.013769	1.076058	1.076202	323.4202	300.5193
MCPH1	0.986237	0.013763	0.873096	0.872964	484.319	554.8
OSBPL7	0.986245	0.013755	0.98318	0.983144	260.0698	264.5289
RHOBTB1	0.986245	0.013755	1.146306	1.146483	531.2538	463.3755
CNIH4	0.986245	0.013755	0.826717	0.826624	879.125	1063.514
MTDH	0.986246	0.013754	0.76541	0.765375	2929.676	3827.77
PPP4R3A	0.986246	0.013754	0.766567	0.766531	2851.877	3720.501
ZNF526	0.986251	0.013749	1.133631	1.133809	484.5746	427.3852
TMPRSS1	0.986253	0.013747	0.933331	0.933212	305.6376	327.512
HDAC11	0.986256	0.013744	0.853931	0.853812	594.6041	696.4129
WBP1L	0.986258	0.013742	1.092541	1.092702	357.8739	327.512
TMEM129	0.986259	0.013741	1.178923	1.179082	770.2068	653.2245
PGBD1	0.986263	0.013737	1.058088	1.058206	298.9692	282.5241
COMMD9	0.986266	0.013734	0.889986	0.889849	412.333	463.3755
ZDHHC17	0.986274	0.013726	1.035521	1.035597	276.741	267.2282
C5orf22	0.986274	0.013726	0.809111	0.809037	1223.662	1512.494
PRMT7	0.986275	0.013725	0.929941	0.929819	304.5262	327.512
LINC0009	0.986276	0.013724	0.906047	0.905911	362.3195	399.9516
ANAPC15	0.986281	0.013719	1.153988	1.15416	601.2726	520.9601
MRPL32	0.986282	0.013718	0.824921	0.82483	911.3559	1104.903
LY75-CD3	0.986283	0.013717	0.962728	0.962653	270.5171	281.0125
PSMG3	0.986286	0.013714	0.88941	0.889271	405.6645	456.1775
EPHA2	0.986287	0.013713	1.314367	1.31442	4408.962	3354.299
PPA1	0.986287	0.013713	1.358288	1.35832	8695.669	6401.78
RNMT	0.986288	0.013712	0.805903	0.805832	1268.118	1573.677
PTPN20	0.98629	0.01371	0.914681	0.914549	342.3142	374.2995
PRR12	0.986291	0.013709	1.190132	1.190283	853.5626	717.1074
B3GNT2	0.986297	0.013703	0.819335	0.81925	1002.491	1223.671
PIGC	0.986298	0.013702	0.902491	0.902354	364.5423	403.9915
MTRR	0.986304	0.013696	0.852537	0.85242	611.2753	717.1074
ZNF335	0.986305	0.013695	1.126936	1.127112	463.4578	411.1895
ZNRD1	0.986308	0.013692	1.172045	1.172206	723.5276	617.2342
MTRNR2L	0.986312	0.013688	1.323248	1.323296	5062.682	3825.808
PLEKHM1	0.986314	0.013686	1.177184	1.177343	764.5053	649.3466
MAP4K2	0.986314	0.013686	1.184356	1.18451	804.6605	679.3175
ZNF350	0.986315	0.013685	0.959523	0.959442	274.5182	286.1231
PYGL	0.986318	0.013682	0.730293	0.730277	7323.078	10027.81
RBM38	0.986318	0.013682	0.819724	0.819641	1025.831	1251.564

GNB1L	0.986333	0.013667	1.04415	1.044241	295.0237	282.5241
BNIP2	0.986345	0.013655	1.224901	1.225021	1333.691	1088.708
FAM217B	0.986347	0.013653	0.885624	0.885489	425.5143	480.5429
TMEM130	0.98635	0.01365	0.984228	0.984194	265.8381	270.1074
SMNDC1	0.986352	0.013648	1.218678	1.218803	1236.999	1014.927
PKP3	0.986352	0.013648	0.825198	0.825109	943.5867	1143.593
PPP1R9A	0.98636	0.01364	0.815001	0.814922	1092.516	1340.64
NPEPL1	0.986365	0.013635	0.947542	0.947444	287.8106	303.7764
ECH1	0.986366	0.013634	0.774823	0.774782	2416.204	3118.562
CDK19	0.986373	0.013627	0.857421	0.857302	582.3786	679.3175
SNHG7	0.986376	0.013624	1.116915	1.117085	445.8864	399.1508
AKIRIN2	0.986377	0.013623	0.826991	0.8269	898.0189	1086.008
PRR11	0.986378	0.013622	1.298899	1.298961	3566.202	2745.424
STAG3L2	0.986382	0.013618	0.966212	0.966144	281.0866	290.9368
PELI3	0.986388	0.013612	1.051405	1.051508	298.9692	284.3236
ZNF266	0.986389	0.013611	0.877357	0.877226	465.6806	530.8574
MRPL35	0.986391	0.013609	0.819682	0.819599	1014.717	1238.067
GMCL1	0.986392	0.013608	1.165397	1.165561	670.1355	574.9456
TIMM17B	0.986393	0.013607	0.816914	0.816834	1091.404	1336.141
TMEM30A	0.986396	0.013604	1.342922	1.342959	6955.201	5179.009
STIM2	0.986404	0.013596	1.025884	1.025939	277.8524	270.8272
STX7	0.986407	0.013593	0.833547	0.833451	836.8914	1004.13
BCAR1	0.986408	0.013592	1.247452	1.247551	1800.484	1443.212
ANXA5	0.986408	0.013592	1.364789	1.364818	9855.98	7221.46
METRNL	0.986417	0.013583	0.80176	0.801695	1403.71	1750.93
NCL	0.986424	0.013576	0.714389	0.714379	11897.64	16654.53
MARVELD	0.986429	0.013571	1.117553	1.117724	433.4497	387.7958
TIMM8A	0.986429	0.013571	0.879767	0.879637	470.1263	534.4564
BMS1P20	0.98643	0.01357	1.10173	1.101892	394.117	357.6719
ANAPC16	0.986434	0.013566	0.769544	0.769508	2754.962	3580.165
ABCA2	0.98644	0.01356	1.239451	1.239557	1591.539	1283.955
TRIM29	0.986442	0.013558	0.955573	0.955489	292.3007	305.9178
ELOVL6	0.986449	0.013551	0.877415	0.877285	471.2377	537.1557
INF2	0.98645	0.01355	1.271902	1.271982	2461.772	1935.38
RFFL	0.986451	0.013549	1.108802	1.108966	426.5812	384.6647
PWP2	0.986453	0.013547	1.235121	1.235229	1548.194	1253.363
MTM1	0.986457	0.013543	0.891467	0.891334	423.4471	475.0724
DENND4A	0.986459	0.013541	0.930638	0.930521	315.6403	339.2089
UPF3B	0.986459	0.013541	0.823574	0.823489	989.1545	1201.177
IARS2	0.98646	0.01354	0.778317	0.778274	2333.96	2998.894
LOC64676	0.986461	0.013539	0.931551	0.931436	322.2866	346.0111
ASNSD1	0.986461	0.013539	0.829538	0.829446	876.9022	1057.216
MAP1S	0.986462	0.013538	1.241825	1.241929	1648.22	1327.143
KCTD2	0.986466	0.013534	0.870491	0.870365	509.0256	584.8429
SMIM4	0.986469	0.013531	1.035926	1.035999	288.9665	278.9251
SUPT16H	0.986473	0.013527	0.750475	0.750451	4388.956	5848.429
BIVM-ERC	0.986475	0.013525	0.922295	0.922172	331.2779	359.2375
ZFAND2A	0.986477	0.013523	1.015059	1.01509	276.741	272.6268
CTBS	0.986481	0.013519	0.837056	0.836957	805.7719	962.7414
MFSD5	0.986483	0.013517	0.868678	0.868553	521.2511	600.1388
GLRX5	0.986486	0.013514	0.811864	0.81179	1182.54	1456.709
C4orf33	0.986488	0.013512	0.951946	0.951856	291.1893	305.9178
TAF9	0.986489	0.013511	0.814988	0.814912	1149.231	1410.254
ING4	0.986489	0.013511	1.109545	1.109708	432.3383	389.5953
SP2	0.986494	0.013506	0.941715	0.941612	310.0833	329.3115
LSM14B	0.986497	0.013503	0.804114	0.804048	1363.7	1696.044

RASSF4	0.986498	0.013502	0.875985	0.875858	495.6887	565.948
LAMC1	0.986498	0.013502	1.401277	1.401295	17931.48	12796.36
AP3S1	0.986509	0.013491	1.271775	1.271854	2547.351	2002.862
PIP4K2C	0.986513	0.013487	1.303426	1.303483	3905.493	2996.195
HELZ	0.986514	0.013486	0.816253	0.816175	1101.407	1349.476
DTX2	0.986514	0.013486	1.178441	1.178592	806.4832	684.2752
CXXC1	0.986514	0.013486	0.942054	0.941952	303.4148	322.1135
TRIM35	0.986515	0.013485	1.097477	1.097633	390.1048	355.4045
SOCS3	0.986522	0.013478	1.204596	1.204729	1053.616	874.5651
ALDH7A1	0.986523	0.013477	1.270393	1.270473	2443.99	1923.683
CPPED1	0.986529	0.013471	0.86127	0.861151	573.4873	665.9561
NDUFA3	0.98653	0.01347	0.793497	0.793442	1682.674	2120.73
TMEM261	0.986531	0.013469	1.160264	1.160424	672.4028	579.4443
PYROXD1	0.986536	0.013464	0.851146	0.851036	652.3974	766.5941
PACS1	0.986537	0.013463	1.28107	1.281142	2915.227	2275.489
NOM1	0.98654	0.01346	0.865819	0.865697	544.813	629.3359
CCDC88A	0.986547	0.013453	1.185857	1.186004	856.8968	722.5059
NME6	0.986547	0.013453	0.897306	0.897175	400.3742	446.2621
HEATR1	0.986553	0.013447	0.766211	0.766177	3115.281	4066.008
SERAC1	0.986554	0.013446	0.921262	0.92114	338.9799	368.0011
MAD1L1	0.986556	0.013444	1.002617	1.002623	277.8524	277.1256
TLE2	0.986558	0.013442	0.89643	0.896298	396.7732	442.6811
GALK1	0.986559	0.013441	1.103152	1.103311	408.9987	370.7004
GOLGA2P	0.98656	0.01344	0.836765	0.836668	801.3152	957.7477
WDR13	0.986561	0.013439	1.196661	1.196799	969.1492	809.7825
RAP1GAP	0.986562	0.013438	0.941882	0.94178	306.749	325.7125
CHST14	0.986564	0.013436	1.129615	1.129782	500.1343	442.6811
RRP8	0.986567	0.013433	0.971151	0.971095	277.8524	286.1231
PRPF40A	0.986575	0.013425	0.752228	0.752203	4276.704	5685.573
SCNM1	0.986576	0.013424	0.834002	0.833907	837.7027	1004.553
IFT80	0.986579	0.013421	0.802808	0.802744	1419.27	1768.025
PTPN18	0.986583	0.013417	0.880649	0.880522	469.0148	532.6569
FBXO3	0.986586	0.013414	0.91001	0.909882	366.7652	403.0917
APTX	0.986594	0.013406	1.163078	1.163236	682.4055	586.6424
ASB13	0.986596	0.013404	0.864116	0.863998	576.8216	667.6207
GTF2H4	0.986606	0.013394	0.86172	0.861604	594.6041	690.1146
WRAP53	0.986608	0.013392	0.973036	0.972983	284.5209	292.4214
DNMT3B	0.986613	0.013387	0.784558	0.784511	2050.551	2613.798
IDUA	0.986613	0.013387	1.001642	1.001646	281.1866	280.7246
OIP5-AS1	0.986614	0.013386	0.835273	0.835178	828.0001	991.4077
KIAA0930	0.986618	0.013382	1.268367	1.268447	2419.55	1907.488
DNAJC11	0.986619	0.013381	0.795676	0.795619	1639.329	2060.447
C11orf84	0.98662	0.01338	1.195111	1.195249	963.5921	806.1834
KCTD21	0.986628	0.013372	1.03617	1.036241	306.749	296.0205
ECI1	0.986633	0.013367	0.822668	0.822586	1012.494	1230.869
PARP8	0.986633	0.013367	0.90721	0.907083	377.8793	416.5881
TAB1	0.986634	0.013366	0.868342	0.868221	539.8005	621.733
NUP85	0.986634	0.013366	0.842878	0.842777	744.6444	883.5627
SNRNP25	0.986634	0.013366	1.006326	1.006338	283.4094	281.6243
MRPS27	0.986634	0.013366	1.213751	1.213874	1200.322	988.8344
DBT	0.986638	0.013362	0.83232	0.832229	892.4619	1072.377
NLE1	0.986641	0.013359	1.049471	1.049566	307.8605	293.3212
HACD1	0.986648	0.013352	0.858083	0.85797	626.835	730.6037
NBPF12	0.98665	0.01335	1.06316	1.063275	332.2114	312.4411
C8orf76	0.986651	0.013349	1.005276	1.005286	284.1985	282.7041
TTC39B	0.986657	0.013343	0.883958	0.883831	461.235	521.8598

METTL2A	0.986659	0.013341	0.870187	0.870066	531.6317	611.0259
NUP35	0.986662	0.013338	1.020711	1.020753	285.6323	279.8248
MAPK7	0.986663	0.013337	1.067619	1.06774	340.0913	318.5144
RXRB	0.986666	0.013334	1.2001	1.200233	1029.165	857.4697
CRLF3	0.986669	0.013331	1.102163	1.102317	415.9006	377.2956
FBXO6	0.98667	0.01333	1.006849	1.006863	285.7767	283.8288
EXTL3	0.986671	0.013329	1.223063	1.223178	1353.697	1106.703
TMEM265	0.986674	0.013326	1.028048	1.028103	303.4148	295.1207
ANKRD42	0.986676	0.013324	1.104591	1.104746	423.4471	383.297
ARMC9	0.986677	0.013323	0.997774	0.997769	284.632	285.2684
FAM102A	0.986685	0.013315	1.241421	1.24152	1754.916	1413.52
GMPR	0.986687	0.013313	0.889138	0.889011	442.341	497.5663
ZCCHC2	0.986687	0.013313	0.95765	0.957573	298.9692	312.2161
SUV39H1	0.98669	0.01331	1.141556	1.141716	583.49	511.0627
TTC14	0.986693	0.013307	1.095858	1.096006	411.2215	375.1992
CEP57L1	0.986695	0.013305	0.927302	0.927188	337.8685	364.4021
UBN1	0.986701	0.013299	1.22648	1.226591	1420.381	1157.989
E4F1	0.986705	0.013295	0.997256	0.997251	293.4121	294.221
C7orf50	0.986708	0.013292	0.844262	0.844161	741.3102	878.1641
ZSCAN32	0.986708	0.013292	0.918842	0.918723	364.5423	396.7934
KLHL42	0.986709	0.013291	0.827334	0.827249	978.0404	1182.282
LSM7	0.98671	0.01329	1.157158	1.157314	676.8484	584.8429
EMC3	0.98671	0.01329	0.81167	0.8116	1239.222	1526.89
HSPA4L	0.986713	0.013287	1.196368	1.196503	995.823	832.2764
PIAS1	0.986714	0.013286	1.150521	1.15068	620.1665	538.9552
CRTC1	0.986715	0.013285	0.94045	0.94035	318.9745	339.2089
PPP1R35	0.986717	0.013283	0.857871	0.857759	621.278	724.3054
PPIL3	0.98672	0.01328	1.084686	1.084825	376.7678	347.3067
EWSR1	0.986724	0.013276	0.753125	0.7531	4296.709	5705.367
IPW	0.986726	0.013274	1.097831	1.097981	408.9987	372.4999
CXorf40A	0.986732	0.013268	0.920239	0.92012	347.8934	378.0964
DCAKD	0.986735	0.013265	0.981604	0.981568	296.7464	302.3188
RAD1	0.986754	0.013246	0.831009	0.830921	908.3217	1093.152
RNF130	0.986761	0.013239	0.797777	0.79772	1617.101	2027.155
HCCS	0.986766	0.013234	1.239206	1.239306	1689.343	1363.134
NUDT19	0.986767	0.013233	1.170013	1.170162	758.1369	647.889
UNC93B1	0.986772	0.013228	0.851616	0.851511	697.9652	819.6798
SPIN3	0.986774	0.013226	0.95052	0.950434	318.9745	335.6098
PUS7L	0.986778	0.013222	0.851229	0.851123	684.6283	804.3839
PARP4	0.986786	0.013214	1.265578	1.265657	2403.979	1899.39
ZNF749	0.986789	0.013211	1.000128	1.000128	299.1359	299.0977
MSI2	0.98679	0.01321	0.909465	0.909343	384.5477	422.8864
SIN3A	0.98679	0.01321	0.826298	0.826216	1010.271	1222.772
PACSIN2	0.986804	0.013196	1.288835	1.288899	3336.452	2588.605
ITSN1	0.986817	0.013183	0.799425	0.799367	1583.759	1981.268
UTP14C	0.986818	0.013182	0.841437	0.84134	788.6896	937.4222
ZNF585A	0.986819	0.013181	1.116396	1.11655	487.6865	436.7787
CCDC120	0.986819	0.013181	0.873504	0.873386	530.3202	607.2019
ENY2	0.986822	0.013178	1.182365	1.182505	872.4565	737.8018
KANSL1	0.98683	0.01317	0.851072	0.850968	711.3021	835.8755
MANBA	0.986835	0.013165	0.826024	0.825942	998.0458	1208.375
GATA2	0.986839	0.013161	1.162948	1.163098	716.8592	616.3344
STAG1	0.98684	0.01316	0.82856	0.828476	984.7089	1188.581
CPQ	0.986846	0.013154	1.010957	1.010978	305.6376	302.3188
RASL10B	0.986849	0.013151	0.938018	0.937919	340.0913	362.6026
CRAMP1	0.986849	0.013151	0.864128	0.864015	592.3813	685.6158

PAPOLG	0.98685	0.01315	1.101003	1.10115	429.0041	389.5953
ARRDC3	0.986851	0.013149	1.172498	1.172644	791.3236	674.8187
FNDC3A	0.986852	0.013148	0.797134	0.797079	1666.003	2090.139
WASH2P	0.986853	0.013147	0.964967	0.964903	304.5485	315.6262
TEX30	0.986853	0.013147	1.015279	1.015308	307.8605	303.2185
CHD8	0.986853	0.013147	0.799788	0.79973	1583.759	1980.368
PSMD14	0.986854	0.013146	1.295654	1.295712	3714.331	2866.63
DPM3	0.986858	0.013142	0.959275	0.959203	308.9719	322.1135
SGK223	0.98686	0.01314	0.902699	0.902577	410.1101	454.3779
BLOC1S3	0.986863	0.013137	0.968127	0.968068	297.8911	307.7173
LRRC58	0.986864	0.013136	1.274074	1.274147	2741.836	2151.898
AEN	0.986867	0.013133	0.874087	0.873969	532.3652	609.1364
USP43	0.986874	0.013126	1.039548	1.039621	318.9745	306.8176
SH3GLB1	0.986881	0.013119	1.307615	1.307666	4554.556	3482.964
DNAJB1	0.986884	0.013116	1.315847	1.315894	5149.161	3913.049
CCDC84	0.986888	0.013112	0.994131	0.99412	297.8578	299.6195
PCCA	0.986889	0.013111	1.064742	1.064853	361.2081	339.2089
RAB4B	0.986889	0.013111	1.016243	1.016274	303.2703	298.4138
PPHLN1	0.986895	0.013105	0.789187	0.789139	1966.084	2491.431
ETAA1	0.986898	0.013102	0.876316	0.876198	523.4739	597.4395
RMI2	0.986898	0.013102	0.917018	0.916903	374.545	408.4903
BCHE	0.986898	0.013102	1.035347	1.035413	316.7517	305.9178
WDR92	0.9869	0.0131	1.073866	1.073988	369.2547	343.8156
C5orf34	0.986902	0.013098	1.011973	1.011996	302.3034	298.7198
PIAS3	0.986903	0.013097	1.282443	1.282509	3105.278	2421.25
RNF126	0.986905	0.013095	0.888009	0.887889	480.1289	540.7547
ARF6	0.986906	0.013094	1.267113	1.26719	2511.786	1982.168
CLCC1	0.986909	0.013091	0.875176	0.875059	530.9426	606.752
HRSP12	0.986912	0.013088	0.864135	0.864024	600.1612	694.6134
STX10	0.986913	0.013087	1.217847	1.21796	1332.58	1094.106
KRBA1	0.986914	0.013086	1.051385	1.051476	343.4256	326.6123
RNF215	0.986915	0.013085	0.974314	0.974268	311.1947	319.4142
PLEKHM3	0.98692	0.01308	1.120151	1.120304	512.7822	457.716
DHX32	0.98692	0.01308	0.820812	0.820737	1104.741	1346.038
NDUFS2	0.986922	0.013078	0.782382	0.782339	2275.055	2908.019
TP53INP1	0.986926	0.013074	1.10088	1.101024	446.7867	405.791
BRAT1	0.986927	0.013073	1.110762	1.110912	466.792	420.1871
SCO1	0.98693	0.01307	0.863822	0.863711	604.6068	700.012
TPP1	0.986933	0.013067	0.795956	0.795903	1731.576	2175.616
ERBB2IP	0.986936	0.013064	1.254188	1.254274	2122.792	1692.445
SPDL1	0.986939	0.013061	0.784337	0.784293	2246.159	2863.931
GPBP1L1	0.986944	0.013056	1.277165	1.277235	2906.336	2275.489
PDXDC2P	0.986954	0.013046	0.978385	0.978346	304.7596	311.5053
FAM234B	0.986957	0.013043	1.039312	1.039384	324.5316	312.2341
CABLES1	0.986958	0.013042	0.90779	0.907672	403.4417	444.4806
APOO	0.986959	0.013041	0.861261	0.861152	626.835	727.9045
NSMCE2	0.986961	0.013039	0.964231	0.964168	312.3061	323.913
OGG1	0.986963	0.013037	0.986044	0.986018	303.4148	307.7173
HPS6	0.986964	0.013036	1.061054	1.061159	352.3168	332.0108
GRHPR	0.986976	0.013024	1.250778	1.250866	2044.994	1634.861
CLN8	0.986977	0.013023	0.954652	0.954575	321.1974	336.4826
MAP4	0.986978	0.013022	1.344546	1.344579	8117.736	6037.378
PDDC1	0.98698	0.01302	0.893514	0.893395	464.747	520.2043
SLC26A11	0.986983	0.013017	0.84199	0.841897	830.223	986.1351
DENND6A	0.986983	0.013017	1.15156	1.15171	662.4001	575.1435
C2orf49	0.986983	0.013017	0.865093	0.864983	602.384	696.4129

TRIM5	0.986986	0.013014	0.921573	0.921462	371.4331	403.0917
LEPROTL	0.986998	0.013002	1.18016	1.180297	883.5706	748.5989
ANAPC2	0.987004	0.012996	1.095948	1.096088	429.0041	391.3949
MRPL43	0.987006	0.012994	1.147699	1.147849	642.3947	559.6497
LIN52	0.987007	0.012993	0.901612	0.901494	426.7813	473.4168
CLK2	0.987008	0.012992	0.852777	0.852675	703.5223	825.0784
MCM6	0.987011	0.012989	1.279262	1.279329	3030.814	2369.064
PDE6D	0.987011	0.012989	1.16022	1.160366	723.5276	623.5325
SMYD4	0.987012	0.012988	1.076779	1.076901	393.8502	365.7248
IFT57	0.987017	0.012983	0.811733	0.811667	1348.14	1660.954
TNIP2	0.987019	0.012981	1.148534	1.148684	649.0632	565.0482
CASP8	0.987021	0.012979	0.862614	0.862506	624.9567	724.5844
QDPR	0.987023	0.012977	0.969344	0.96929	307.8605	317.6147
MESDC2	0.987024	0.012976	1.186338	1.18647	973.5948	820.5796
POP1	0.987025	0.012975	1.086988	1.08712	407.8873	375.1992
ATXN7L3I	0.987025	0.012975	0.812446	0.812379	1300.349	1600.67
PRKRIR	0.987027	0.012973	0.802529	0.802471	1555.973	1938.979
RBM7	0.987028	0.012972	1.240318	1.240412	1803.573	1454.009
PNKD	0.987031	0.012969	0.76398	0.763951	3498.717	4579.77
UBE2S	0.987032	0.012968	1.375403	1.375425	12855.67	9346.689
USP20	0.987034	0.012966	1.166088	1.166231	770.2068	660.4226
ALG12	0.987036	0.012964	0.866378	0.866268	600.1612	692.8139
PCDH1	0.987042	0.012958	1.218905	1.219014	1420.381	1165.187
PSMG1	0.987049	0.012951	0.791262	0.791213	1907.179	2410.452
IPPK	0.987049	0.012951	1.124916	1.125066	533.4766	474.1726
C6orf136	0.987051	0.012949	0.998243	0.998239	308.9719	309.5169
ACYP1	0.987055	0.012945	0.915486	0.915373	391.2162	427.3852
PAAF1	0.987057	0.012943	0.887817	0.8877	496.8001	559.6497
GPR137C	0.98706	0.01294	0.907089	0.906973	414.5558	457.0772
METTL16	0.987064	0.012936	1.144588	1.144737	632.2587	552.3166
RBM42	0.987065	0.012935	0.809967	0.809903	1369.257	1690.646
GEMIN2	0.987066	0.012934	0.899295	0.899178	440.1182	489.4685
COX10	0.987068	0.012932	0.918155	0.918044	395.6618	430.9842
MYEOV2	0.987071	0.012929	1.136773	1.136923	591.2699	520.0603
NXT1	0.987072	0.012928	0.967764	0.967709	325.643	336.5096
NAA25	0.987081	0.012919	0.837267	0.837179	883.5706	1055.416
SPOP	0.987081	0.012919	0.844112	0.844019	822.4431	974.4382
DCLRE1B	0.987087	0.012913	0.993876	0.993864	311.1947	313.1159
ACD	0.987089	0.012911	1.052144	1.052232	357.8739	340.1086
TCEAL3	0.987094	0.012906	1.08744	1.087569	425.6699	391.3949
LYPD6	0.987095	0.012905	0.896518	0.896402	464.5692	518.2608
RHOBTB2	0.987101	0.012899	1.01424	1.014266	311.1947	306.8176
APBA3	0.987103	0.012897	0.991729	0.991714	312.3061	314.9154
PLEKHG6	0.987104	0.012896	0.953701	0.953625	331.2001	347.3067
RTF1	0.987106	0.012894	0.812974	0.812908	1309.24	1610.567
MRPL47	0.987111	0.012889	0.818366	0.818296	1225.885	1498.098
SLC22A5	0.987112	0.012888	0.860894	0.86079	666.8457	774.6919
COMMD1(	0.987115	0.012885	1.188311	1.18844	988.0431	831.3767
RCE1	0.987116	0.012884	1.01159	1.011611	316.7517	313.1159
ERICH1	0.987117	0.012883	0.989608	0.989589	313.4175	316.7149
SRM	0.987117	0.012883	0.79051	0.790463	2048.328	2591.304
MAP3K4	0.987118	0.012882	1.159958	1.160102	737.976	636.1291
WDR83	0.987124	0.012876	0.927888	0.927786	378.9907	408.4903
CHUK	0.987126	0.012874	0.820915	0.820843	1149.198	1400.024
ETV3	0.987131	0.012869	1.008697	1.008713	316.7517	314.0156
S100A4	0.987131	0.012869	1.008697	1.008713	316.7517	314.0156

MAP2K4	0.987134	0.012866	0.856109	0.856007	692.4082	808.8827
NCKIPSD	0.987138	0.012862	0.937266	0.937172	363.4309	387.7958
C19orf53	0.987139	0.012861	1.263711	1.263787	2508.451	1984.867
SLC39A3	0.987146	0.012854	0.928618	0.928516	370.0994	398.5929
AP3B1	0.987149	0.012851	0.836598	0.836513	928.027	1109.402
HNRNPM	0.987149	0.012851	0.76349	0.763462	3618.75	4739.927
FAM214A	0.987153	0.012847	1.048193	1.048276	348.9826	332.9106
TM2D3	0.987156	0.012844	1.044612	1.044689	353.4282	338.3091
NARF	0.98716	0.01284	0.826681	0.826604	1081.402	1308.249
TAF5L	0.987161	0.012839	1.118553	1.118698	533.4766	476.8719
FOXO1	0.987162	0.012838	0.967048	0.966992	324.5316	335.6098
IPMK	0.987171	0.012829	1.212622	1.212733	1318.132	1086.908
ATP6V0E1	0.987177	0.012823	1.350066	1.350096	8804.587	6521.448
FEZ2	0.987177	0.012823	0.805963	0.805903	1477.063	1832.808
SYNJ1	0.987181	0.012819	0.86269	0.862585	645.729	748.5989
PWP1	0.987181	0.012819	0.827882	0.827805	1065.842	1287.554
ATP5C1	0.987182	0.012818	0.760741	0.760714	3982.181	5234.794
RANBP1	0.987183	0.012817	0.774849	0.774814	2812.978	3630.525
MSH3	0.987189	0.012811	0.9337	0.933603	357.7961	383.2431
BARD1	0.987189	0.012811	0.873892	0.873783	585.7128	670.3199
IQSEC2	0.987193	0.012807	0.907053	0.90694	426.7813	470.5736
RBMS1	0.987194	0.012806	1.28085	1.280914	3209.751	2505.827
UXT	0.987194	0.012806	1.195098	1.195222	1062.508	888.9612
C14orf80	0.987196	0.012804	1.05166	1.051747	356.7625	339.2089
ERCC6L	0.987197	0.012803	1.185525	1.185654	956.9236	807.0832
GTF2F1	0.987197	0.012803	0.77854	0.778502	2602.921	3343.502
PLD1	0.987199	0.012801	0.993042	0.993029	318.9745	321.2137
SGSM2	0.9872	0.0128	0.868655	0.868548	621.278	715.3079
DERL1	0.987202	0.012798	1.264265	1.264339	2558.465	2023.556
EVL	0.987203	0.012797	1.097846	1.097981	453.4551	412.9891
KANSL2	0.987207	0.012793	0.87117	0.871061	590.1585	677.518
CSRNP1	0.987211	0.012789	0.884601	0.884489	515.694	583.0434
MARS	0.987218	0.012782	1.354364	1.354392	9663.706	7135.083
MRPL33	0.987218	0.012782	1.263356	1.263432	2489.557	1970.471
DNAJB12	0.987223	0.012777	0.822777	0.822704	1165.869	1417.119
IP6K2	0.987223	0.012777	0.861996	0.861893	656.8431	762.0953
KRBOX4	0.987225	0.012775	1.015906	1.015934	334.4676	329.2216
FOXN2	0.987229	0.012771	0.880602	0.880491	537.9222	610.9359
NARFL	0.987231	0.012769	1.083165	1.083287	427.8927	394.9939
CENPQ	0.987232	0.012768	0.993795	0.993784	315.6403	317.6147
WDR47	0.987233	0.012767	1.010311	1.010329	324.5316	321.2137
SMCR8	0.987237	0.012763	0.839751	0.839664	880.2475	1048.335
FANCC	0.987238	0.012762	1.124433	1.124577	553.482	492.1678
ZFX	0.987241	0.012759	0.901923	0.90181	447.8981	496.6666
PPL	0.987242	0.012758	0.809833	0.809771	1421.493	1755.428
C14orf2	0.987245	0.012755	0.794866	0.794817	1879.394	2364.565
SLC2A13	0.987246	0.012754	1.149642	1.149784	687.9625	598.3393
ENOPH1	0.987254	0.012746	0.810365	0.810303	1411.49	1741.932
GNE	0.987262	0.012738	0.818187	0.818118	1235.932	1510.703
SPNS2	0.987264	0.012736	1.223346	1.223448	1500.403	1226.371
PLAG1	0.987266	0.012734	1.144232	1.144374	657.9545	574.9456
PRKCD	0.987269	0.012731	1.215169	1.215276	1421.493	1169.686
TSSC4	0.98727	0.01273	0.88087	0.88076	541.2565	614.5349
BTBD10	0.98727	0.01273	1.177614	1.177744	933.584	792.6871
RBM19	0.987277	0.012723	1.05179	1.051875	363.4309	345.5072
SAC3D1	0.987279	0.012721	1.002552	1.002556	325.643	324.8127

SS18L1	0.987282	0.012718	0.846472	0.846381	830.1896	980.8715
DCAF17	0.987283	0.012717	0.912389	0.912281	430.1155	471.4734
APOBEC3	0.987286	0.012714	1.16902	1.169156	820.6759	701.9374
HPSE	0.987288	0.012712	0.922029	0.921926	395.684	429.1937
CLCN6	0.987291	0.012709	0.932608	0.932513	374.545	401.6521
QARS	0.987291	0.012709	1.263896	1.263969	2590.696	2049.649
IFNGR1	0.987293	0.012707	1.195649	1.195769	1111.41	929.4503
CASC4	0.987293	0.012707	0.821588	0.821517	1205.602	1467.533
EXOC6B	0.987293	0.012707	0.984196	0.984168	326.7544	332.0108
PARP2	0.987298	0.012702	1.146452	1.146594	675.737	589.3417
MPHOSPH	0.9873	0.0127	0.836501	0.836418	934.6955	1117.5
DUSP11	0.987308	0.012692	1.131623	1.131765	595.7155	526.3586
GDI1	0.987309	0.012691	1.2896	1.289658	3713.219	2879.227
SNRK	0.987316	0.012684	0.989514	0.989496	335.6457	339.2089
ZBTB6	0.987329	0.012671	0.901853	0.901743	456.7893	506.5639
CENPJ	0.98733	0.01267	1.135793	1.135934	635.7263	559.6497
PDCD2	0.987337	0.012663	0.818237	0.81817	1253.67	1532.288
SMO	0.987341	0.012659	1.128133	1.128273	597.9384	529.9576
GLRB	0.987349	0.012651	0.940422	0.940335	375.6564	399.4927
PDLIM2	0.987351	0.012649	0.996918	0.996912	330.0886	331.1111
SLC37A4	0.987353	0.012647	0.85516	0.855065	752.4243	879.9636
AP5B1	0.987353	0.012647	0.865983	0.86588	644.7287	744.595
METTL14	0.987353	0.012647	1.039613	1.039679	354.5397	341.0084
TMEM127	0.987353	0.012647	0.812143	0.812082	1431.329	1762.546
SPRED1	0.987355	0.012645	0.946604	0.946524	367.2875	388.0388
SH2D5	0.987361	0.012639	1.019522	1.019555	346.7598	340.1086
RPRD2	0.987362	0.012638	0.79241	0.792364	2018.32	2547.216
NFXL1	0.987362	0.012638	1.042942	1.043013	367.8766	352.7053
NSA2	0.987365	0.012635	0.821275	0.821205	1199.211	1460.308
USF2	0.987368	0.012632	1.234371	1.234463	1789.369	1449.511
ESPL1	0.98737	0.01263	0.854862	0.854766	739.0874	864.6677
MXD1	0.987372	0.012628	1.151195	1.151333	716.8592	622.6327
ZFAND2B	0.987376	0.012624	0.893002	0.892892	495.6887	555.1509
TBL3	0.987377	0.012623	1.194555	1.194673	1145.863	959.1424
KLRG2	0.987377	0.012623	0.906247	0.906139	446.7867	493.0676
VPS13B	0.987384	0.012616	0.836632	0.83655	949.1438	1134.595
FGF2	0.987388	0.012612	0.985582	0.985557	333.4229	338.3091
NUP155	0.987389	0.012611	0.794417	0.794369	1950.524	2455.44
NDUFB8	0.987392	0.012608	1.203995	1.204107	1247.002	1035.622
KAT8	0.987393	0.012607	0.889102	0.888994	527.9196	593.8405
RAPGEF1	0.987393	0.012607	1.224916	1.225014	1599.318	1305.549
FAM117B	0.987395	0.012605	1.055457	1.055545	387.8819	367.4703
C7orf43	0.987397	0.012603	0.912387	0.912281	430.1155	471.4734
SF3A2	0.987398	0.012602	1.174564	1.174693	905.7988	771.0929
ZNF701	0.987403	0.012597	0.992604	0.992592	341.9918	344.5444
TMEM25	0.987406	0.012594	1.159403	1.159537	800.2149	690.1146
SLC39A1	0.987406	0.012594	0.759801	0.759776	4250.03	5593.797
UCK1	0.987407	0.012593	0.864764	0.864664	661.2887	764.7946
RBM41	0.987409	0.012591	0.877302	0.877197	592.737	675.7185
SPC24	0.987409	0.012591	1.180717	1.180844	950.2996	804.7618
PHF6	0.987414	0.012586	0.795172	0.795124	1931.63	2429.347
EMC3-AS1	0.98742	0.01258	1.073123	1.073232	412.333	384.1968
RAB25	0.98742	0.01258	0.840414	0.84033	905.7988	1077.91
RAB23	0.987427	0.012573	1.193501	1.193619	1115.855	934.8489
KIF23	0.987432	0.012568	1.264093	1.264164	2670.717	2112.632
UNG	0.987434	0.012566	1.189136	1.189256	1092.516	918.6532

RHPN2	0.987436	0.012564	1.302976	1.303025	4613.461	3540.576
SYT17	0.98744	0.01256	1.006235	1.006246	346.7598	344.6074
USE1	0.987444	0.012556	0.978839	0.978804	347.8712	355.4045
HEATR5A	0.987445	0.012555	0.798796	0.798745	1813.042	2269.865
JMJD8	0.987446	0.012554	0.904631	0.904524	459.0122	507.4637
PVR	0.987446	0.012554	1.339551	1.339583	8004.372	5975.268
ECE2	0.987447	0.012553	1.0761	1.076211	421.2242	391.3949
MEX3D	0.987454	0.012546	0.958123	0.958059	353.4282	368.9009
GFOD2	0.987454	0.012546	1.021634	1.02167	346.8265	339.4698
FAM131A	0.987454	0.012546	0.949806	0.949731	362.3195	381.4975
ACTR10	0.987457	0.012543	0.813397	0.813335	1394.819	1714.939
STK19	0.987468	0.012532	0.908036	0.90793	450.1209	495.7668
TINF2	0.987471	0.012529	1.058257	1.058347	398.996	376.9987
PREX2	0.987473	0.012527	0.912598	0.912494	436.784	478.6714
PDPN	0.987479	0.012521	1.110633	1.110766	525.6967	473.2729
COMMD7	0.987484	0.012516	1.261918	1.26199	2620.704	2076.642
DUS1L	0.987485	0.012515	0.794909	0.794861	1933.853	2432.946
CNN2	0.987492	0.012508	1.338602	1.338634	7956.581	5943.803
LRSAM1	0.987494	0.012506	0.895552	0.895445	497.9115	556.0506
SHROOM:	0.987495	0.012505	0.892115	0.892009	525.6967	589.3417
KIAA0100	0.987503	0.012497	1.282539	1.282598	3490.937	2721.769
CBX5	0.987504	0.012496	0.745603	0.745585	6162.644	8265.522
BTBD7	0.987505	0.012495	1.227898	1.227992	1697.122	1382.029
NUTM2A-/	0.987507	0.012493	0.928683	0.928589	400.5742	431.3801
FANCM	0.987511	0.012489	1.017982	1.018012	348.9826	342.8079
KIFAP3	0.987511	0.012489	0.885507	0.885401	545.7021	616.3344
TERF2IP	0.987512	0.012488	1.164127	1.164256	856.8968	736.0023
NDUFAF7	0.987514	0.012486	0.968959	0.96891	349.7161	360.938
CDC5L	0.987515	0.012485	0.795316	0.795269	2014.986	2533.719
DNPH1	0.987516	0.012484	0.871218	0.871116	630.1692	723.4057
AKAP17A	0.987517	0.012483	0.834273	0.834195	1010.271	1211.075
WWP1	0.987517	0.012483	1.235141	1.23523	1909.402	1545.785
LMAN1	0.987519	0.012481	1.297108	1.297159	4306.712	3320.108
CDH6	0.987519	0.012481	0.822306	0.822237	1216.993	1480.102
GCDH	0.987522	0.012478	0.867704	0.867605	655.7317	755.797
CDC25C	0.987523	0.012477	1.04128	1.041346	370.0994	355.4045
EMG1	0.987523	0.012477	0.98188	0.981851	344.537	350.9057
ASCC3	0.987525	0.012475	1.232872	1.232962	1812.709	1470.205
RANGRF	0.987528	0.012472	1.095559	1.095684	471.2377	430.0845
UGGT2	0.98753	0.01247	0.868624	0.868524	650.1746	748.5989
VRK2	0.98753	0.01247	0.980025	0.979993	345.6484	352.7053
ZNF776	0.987534	0.012466	1.080713	1.080825	451.2323	417.4879
SLC12A9	0.987538	0.012462	0.806692	0.806637	1595.984	1978.569
LSM11	0.987541	0.012459	1.078977	1.079088	436.6617	404.6573
CD151	0.987542	0.012458	1.314021	1.314064	5539.265	4215.368
TAF7	0.987542	0.012458	1.281852	1.28191	3570.959	2785.652
RSC1A1	0.987544	0.012456	0.930121	0.930028	393.7724	423.3993
ACAD9	0.987544	0.012456	0.806471	0.806416	1644.886	2039.752
FAM107B	0.987552	0.012448	1.222781	1.222877	1650.443	1349.637
TMEM87B	0.987552	0.012448	0.912616	0.912515	455.6779	499.3659
HRH1	0.987553	0.012447	0.921352	0.921254	421.2242	457.2302
ARSA	0.987556	0.012444	1.169144	1.169271	886.4158	758.0914
POLR1B	0.987557	0.012443	0.853481	0.85339	782.4323	916.8537
CNOT11	0.987559	0.012441	0.796853	0.796806	1924.961	2415.851
SNRNP27	0.987562	0.012438	0.851538	0.851449	823.5545	967.2402
ZFHX4	0.987566	0.012434	1.050472	1.050551	385.6591	367.1014

RPAP1	0.987572	0.012428	0.857717	0.857623	744.6444	868.2668
GINS1	0.987573	0.012427	1.131139	1.131274	616.8323	545.2535
AIF1L	0.987575	0.012425	1.304241	1.304288	4827.963	3701.606
REXO2	0.987575	0.012425	1.324345	1.324383	6505.08	4911.781
BLMH	0.987577	0.012423	1.169862	1.169988	896.9075	766.5941
COL1A1	0.987578	0.012422	1.001623	1.001625	347.8712	347.3067
PDK2	0.987578	0.012422	0.88468	0.884575	557.9276	630.7306
ZKSCAN8	0.987579	0.012421	0.810717	0.810659	1498.18	1848.104
POLR2M	0.987579	0.012421	0.88679	0.886686	561.3174	633.0519
SCO2	0.987586	0.012414	0.949071	0.948997	374.0449	394.1481
STXBP3	0.987587	0.012413	0.884867	0.884764	572.3759	646.9262
BLOC1S5	0.987588	0.012412	0.9263	0.926206	423.3804	457.1132
GCC1	0.987591	0.012409	1.244269	1.244351	2122.792	1705.942
MCM7	0.987593	0.012407	0.750036	0.750017	5464.801	7286.243
EMC9	0.987599	0.012401	1.094226	1.094347	483.4632	441.7813
POLQ	0.987602	0.012398	1.185353	1.185472	1059.173	893.46
SRRD	0.987603	0.012397	1.002825	1.002829	350.094	349.1062
LLGL1	0.987607	0.012393	1.155923	1.156053	808.1392	699.0492
NOL6	0.987608	0.012392	1.221597	1.221693	1604.875	1313.647
CHMP3	0.987612	0.012388	0.794916	0.794871	2070.556	2604.899
CDC42BP	0.987613	0.012387	0.865889	0.865792	682.4055	788.1883
PHLDA3	0.987623	0.012377	0.815197	0.815135	1373.702	1685.247
TEX10	0.987624	0.012376	1.171972	1.172095	949.1438	809.7825
CLK1	0.987634	0.012366	1.024531	1.024571	356.7625	348.2065
ZNF254	0.987635	0.012365	0.949017	0.948944	387.6597	408.5173
STOML2	0.987652	0.012348	0.781092	0.781057	2761.853	3536.05
LOC10028	0.987653	0.012347	0.969804	0.969758	360.2745	371.5102
MRPS35	0.98766	0.01234	0.792418	0.792374	2141.686	2702.874
BDH2	0.987661	0.012339	0.985121	0.985097	354.5397	359.9033
ZBED5	0.987664	0.012336	0.883141	0.883039	576.8216	653.2245
CHFR	0.987667	0.012333	0.873466	0.873367	635.7263	727.9045
FAM132B	0.987667	0.012333	0.897413	0.89731	522.3625	582.1436
ACTR6	0.987671	0.012329	1.123181	1.123312	602.384	536.2559
NDUFV1	0.987673	0.012327	0.773606	0.773574	3180.854	4111.895
TCFL5	0.987675	0.012325	0.861977	0.861883	723.5276	839.4745
NBPF11	0.987678	0.012322	0.880953	0.880852	590.9031	670.8328
NPM3	0.987678	0.012322	1.114429	1.114558	564.5961	506.5639
TRIM47	0.987682	0.012318	1.14036	1.140491	695.7424	610.0361
PWWP2A	0.987684	0.012316	0.829279	0.829207	1128.081	1360.435
STXBP2	0.987684	0.012316	1.273035	1.273097	3250.873	2553.514
PEX6	0.987686	0.012314	1.195344	1.195454	1202.545	1005.93
SCML2	0.987687	0.012313	1.133282	1.133414	655.7317	578.5446
AP1S2	0.987698	0.012302	1.117559	1.117687	595.2043	532.531
CYBA	0.987699	0.012301	1.262982	1.263051	2775.19	2197.21
NUPL2	0.987702	0.012298	0.910272	0.910172	466.792	512.8622
PIN1	0.987704	0.012296	1.22473	1.224823	1700.457	1388.327
VCPIP1	0.987708	0.012292	1.182656	1.182772	1052.505	889.861
SYNGAP1	0.98771	0.01229	1.048002	1.048074	404.5531	385.9963
SARNP	0.987711	0.012289	0.826126	0.826057	1191.431	1442.313
LHPP	0.98772	0.01228	0.851151	0.851065	834.6686	980.7366
MSH2	0.987725	0.012275	0.785179	0.785141	2510.674	3197.741
SPIN4	0.987726	0.012274	0.997228	0.997223	367.8766	368.9009
LZTR1	0.987728	0.012272	1.192721	1.192833	1156.977	969.9395
RRAGB	0.98773	0.01227	0.890741	0.890641	560.1504	628.9311
C9orf142	0.987735	0.012265	1.022328	1.022363	378.9907	370.7004
SPTY2D1	0.987737	0.012263	1.206344	1.206447	1351.474	1120.208

TXNL4B	0.98774	0.01226	1.023499	1.023536	371.2108	362.6746
CCDC167	0.98774	0.01226	0.895646	0.895544	515.694	575.8453
C1orf186	0.987742	0.012258	0.902606	0.902506	497.4447	551.1829
TMEM104	0.987742	0.012258	0.922338	0.922244	429.0041	465.175
S1PR2	0.98775	0.01225	0.9489	0.94883	397.9513	419.4133
ZNF791	0.987752	0.012248	1.042645	1.042709	391.3273	375.2982
PPP1R12/	0.987757	0.012243	1.206926	1.207028	1427.05	1182.282
IL17RC	0.987762	0.012238	0.945135	0.94506	394.5504	417.4879
LTN1	0.987763	0.012237	1.277723	1.277782	3437.59	2690.277
CCL28	0.987771	0.012229	0.956461	0.956399	381.2135	398.5929
LCA5	0.987774	0.012226	0.917895	0.917799	443.4524	483.1702
ATAD3B	0.987774	0.012226	0.902664	0.902564	500.79	554.854
NUAK2	0.987776	0.012224	0.843344	0.843264	937.3073	1111.525
AFAP1	0.987778	0.012222	1.24672	1.246797	2276.167	1825.61
RAB11FIP	0.987778	0.012222	0.916993	0.916897	454.5665	495.7668
MFSD11	0.987781	0.012219	0.962436	0.962382	385.6591	400.7344
HSPA2	0.987782	0.012218	1.143603	1.14373	750.2015	655.9238
DOLPP1	0.987787	0.012213	1.108015	1.108139	552.3706	498.4661
NFKBIL1	0.987787	0.012213	1.098125	1.098244	516.8055	470.5736
PIAS4	0.987789	0.012211	1.054724	1.054803	422.3356	400.3924
TM2D1	0.987792	0.012208	1.150081	1.150207	796.8807	692.8139
SPIDR	0.987792	0.012208	1.207573	1.207674	1412.602	1169.686
CHTF18	0.987793	0.012207	1.173616	1.173735	975.8176	831.3767
LZTS3	0.987793	0.012207	1.235146	1.23523	1970.529	1595.271
LAPTM4B	0.987794	0.012206	0.740948	0.740933	7185.263	9697.595
KLC4	0.987798	0.012202	1.135693	1.135821	685.7397	603.7378
RANGAP1	0.987801	0.012199	1.303874	1.303919	5021.348	3850.966
C1orf123	0.987803	0.012197	1.058684	1.058768	420.1128	396.7934
CXorf40B	0.987803	0.012197	0.900979	0.90088	523.4517	581.0459
ARHGEF1	0.987805	0.012195	0.917569	0.917474	455.6779	496.6666
WDR33	0.987807	0.012193	0.830005	0.829935	1144.752	1379.329
WDR41	0.987808	0.012192	1.081994	1.082102	471.2377	435.483
PRPS1	0.987808	0.012192	1.2077	1.207801	1419.27	1175.084
COX20	0.987808	0.012192	0.944202	0.944127	400.1074	423.7862
RFX7	0.987809	0.012191	1.110469	1.110593	554.5934	499.3659
SUSD2	0.987812	0.012188	0.916767	0.916673	470.1263	512.8622
RABGEF1	0.987812	0.012188	1.111147	1.111271	567.9303	511.0627
OAT	0.98782	0.01218	1.306193	1.306237	5213.622	3991.328
FBXW7	0.987824	0.012176	0.962354	0.9623	380.1021	394.9939
ZDHHC23	0.987825	0.012175	1.138009	1.138137	703.5223	618.134
TMUB2	0.987829	0.012171	0.931261	0.931176	435.6726	467.8743
COA3	0.987829	0.012171	1.174728	1.174846	994.7116	846.6726
USP18	0.98783	0.01217	1.093876	1.093992	507.9142	464.2753
CISD3	0.987831	0.012169	0.902769	0.902671	506.8028	561.4492
OPTN	0.987837	0.012163	1.253891	1.253962	2529.568	2017.258
KIFC2	0.987838	0.012162	0.80705	0.806997	1686.008	2089.239
ATP5D	0.987841	0.012159	0.914846	0.914752	479.0175	523.6593
PLEKHA3	0.987842	0.012158	1.022329	1.022363	378.9907	370.7004
MCTS1	0.987846	0.012154	0.813451	0.813395	1547.593	1902.638
RRAGA	0.987848	0.012152	1.235814	1.235896	2060.553	1667.252
DNAAF5	0.98785	0.01215	0.884057	0.883959	595.7155	673.919
TAF10	0.987851	0.012149	0.852958	0.852873	860.231	1008.629
FOXO3	0.987851	0.012149	1.219557	1.219649	1646.953	1350.348
TMEM170	0.987853	0.012147	1.055959	1.056039	430.5934	407.7435
TRIM32	0.987854	0.012146	1.133247	1.133373	680.1827	600.1388
KIAA1429	0.987858	0.012142	1.236469	1.23655	2081.67	1683.448

ACAP3	0.987861	0.012139	0.995968	0.995961	370.0994	371.6002
GMEB2	0.987866	0.012134	0.868239	0.868147	720.1934	829.5772
TXNDC15	0.987866	0.012134	0.877715	0.877619	635.7263	724.3774
MED17	0.987866	0.012134	0.849305	0.849223	904.6874	1065.314
XPNPEP1	0.987867	0.012133	0.83143	0.83136	1134.749	1364.933
PTPN21	0.987873	0.012127	1.144157	1.144282	769.0954	672.1195
POMT2	0.987875	0.012125	1.127357	1.127483	651.286	577.6448
KLHL5	0.987888	0.012112	1.279346	1.279402	3605.413	2818.043
PTPN6	0.987892	0.012108	1.033688	1.033739	393.439	380.5978
AKR1A1	0.987893	0.012107	1.194607	1.194713	1278.121	1069.813
SLMO2-A1	0.987896	0.012104	0.827224	0.827158	1248.68	1509.605
ABHD17B	0.987897	0.012103	0.952528	0.952463	396.7843	416.5881
GOT1	0.9879	0.0121	0.769196	0.769168	3672.097	4774.118
TCEAL4	0.987901	0.012099	0.803355	0.803306	1872.725	2331.274
CDIPT	0.987902	0.012098	1.255854	1.255923	2630.706	2094.637
NBPF20	0.987905	0.012095	1.080878	1.080982	478.7841	442.915
UQCC1	0.987907	0.012093	1.150083	1.150207	796.8807	692.8139
RABIF	0.987908	0.012092	1.02549	1.025529	396.7732	386.8961
CDK11A	0.987918	0.012082	1.066997	1.067088	458.612	429.7786
VHL	0.987919	0.012081	0.834778	0.834707	1119.189	1340.82
RABEPK	0.987919	0.012081	1.13154	1.131665	667.9572	590.2414
WDR37	0.987921	0.012079	0.958381	0.958323	392.3276	409.39
POLK	0.987921	0.012079	0.940966	0.94089	415.6672	441.7813
KDM2B	0.987926	0.012074	0.899649	0.899552	530.1424	589.3417
WNK2	0.987927	0.012073	1.031733	1.031781	394.5504	382.3973
PIK3C2B	0.987929	0.012071	0.863628	0.863539	749.9903	868.5097
HSDL1	0.987929	0.012071	0.842129	0.842052	984.9423	1169.695
C9orf72	0.987929	0.012071	1.039548	1.039605	413.4444	397.6932
EIF4EBP2	0.987933	0.012067	0.769186	0.769159	3697.66	4807.409
ZNF827	0.987935	0.012065	1.031201	1.031247	404.5531	392.2946
RBM14	0.987937	0.012063	1.170289	1.170406	973.4392	831.7096
NXPE3	0.987938	0.012062	1.145521	1.145644	789.6787	689.2868
FOXRED1	0.987938	0.012062	0.848772	0.848691	885.7934	1043.72
GID8	0.987938	0.012062	0.808258	0.808206	1684.897	2084.74
TNRC6B	0.987939	0.012061	0.921596	0.921507	470.1263	510.172
RAD51AP	0.98794	0.01206	1.089095	1.089204	505.6914	464.2753
CTC1	0.987942	0.012058	0.92885	0.928764	441.2296	475.0724
PDCD6IP	0.987948	0.012052	0.767868	0.767842	3915.474	5099.326
PEX26	0.987951	0.012049	0.830806	0.830737	1195.588	1439.19
CCDC85B	0.987955	0.012045	1.082263	1.082366	487.9088	450.7789
NCAPD2	0.987958	0.012042	1.261912	1.261977	2884.108	2285.386
POLR2C	0.987963	0.012037	1.229393	1.229478	1900.51	1545.785
ELF2	0.987966	0.012034	1.075338	1.075436	471.2377	438.1823
PRKCA	0.987967	0.012033	1.025409	1.025447	392.3498	382.6132
BMI1	0.987967	0.012033	0.883295	0.8832	615.8098	697.2497
FBXO31	0.987967	0.012033	0.969226	0.969182	396.7732	409.39
SF3B4	0.987972	0.012028	0.786465	0.786428	2570.69	3268.822
ZNF319	0.987976	0.012024	1.023027	1.023062	391.2162	382.3973
TPP2	0.987983	0.012017	0.830026	0.829959	1188.097	1431.515
HINFP	0.987986	0.012014	0.932423	0.932341	437.8954	469.6738
CDK9	0.987987	0.012013	1.182145	1.182256	1091.404	923.152
CEP192	0.987988	0.012012	0.860274	0.860187	790.2122	918.6532
TUBGCP6	0.987992	0.012008	1.171378	1.171492	1021.385	871.8658
MAU2	0.987996	0.012004	0.852644	0.852562	869.1223	1019.426
SSNA1	0.987996	0.012004	1.123513	1.123635	649.0632	577.6448
IFT122	0.987998	0.012002	1.078791	1.078891	483.4298	448.0796

CDIP1	0.988	0.012	1.105994	1.106111	571.2645	516.4613
PNPLA6	0.988	0.012	1.25594	1.256008	2687.388	2139.625
ZNF286A	0.988004	0.011996	0.942725	0.942653	431.0935	457.3202
HIST1H2E	0.988009	0.011991	1.148069	1.14819	800.2149	696.9348
CEBPD	0.988013	0.011987	0.902765	0.902671	527.9196	584.8429
MTO1	0.988018	0.011982	0.878643	0.878551	667.9572	760.2958
PM20D2	0.988018	0.011982	1.227015	1.2271	1867.224	1521.653
ZNF816	0.988022	0.011978	1.053355	1.053428	442.8745	420.4121
CEBPZOS	0.988024	0.011976	0.820653	0.820593	1389.073	1692.769
DR1	0.988026	0.011974	1.27015	1.270209	3349.788	2637.192
ZDHHC21	0.988028	0.011972	0.912201	0.912109	495.6887	543.454
METTL21/	0.988029	0.011971	0.884692	0.884598	616.2433	696.6379
MSL2	0.988031	0.011969	0.831195	0.831128	1209.214	1454.909
SRSF3	0.988032	0.011968	0.758964	0.758942	4700.151	6193.036
C11orf95	0.988035	0.011965	0.974343	0.974307	390.1048	400.3924
MFSD9	0.988035	0.011965	1.061319	1.061402	447.8981	421.9866
WDR48	0.988038	0.011962	1.178381	1.178491	1108.075	940.2474
STX17	0.988038	0.011962	0.853361	0.853279	891.3505	1044.619
USF3	0.98804	0.01196	0.89222	0.892126	592.4702	664.1116
ZNF184	0.98804	0.01196	1.08402	1.084123	502.3571	463.3755
DSTYK	0.988041	0.011959	1.118517	1.118636	646.9404	578.3286
HRAS	0.988041	0.011959	1.030238	1.030282	413.4444	401.2922
GRHL1	0.988042	0.011958	0.943206	0.943134	423.4471	448.9794
NFKBIB	0.988042	0.011958	0.829154	0.829088	1250.169	1507.887
PPP1R16/	0.988043	0.011957	1.165657	1.165773	951.3666	816.0808
ASB7	0.988043	0.011957	0.915543	0.915452	486.7974	531.7572
TMEM161	0.988044	0.011956	1.010117	1.010132	398.996	394.9939
ZNF562	0.988044	0.011956	1.138813	1.138933	762.4159	669.4112
ZC3H3	0.988044	0.011956	1.200004	1.200104	1367.034	1139.094
PPFIBP1	0.988044	0.011956	1.314319	1.314357	6295.002	4789.413
EAPP	0.988045	0.011955	0.930548	0.930465	447.8981	481.3707
INTS10	0.988046	0.011954	0.848397	0.848318	911.3559	1074.311
FBXO30	0.988049	0.011951	1.09382	1.09393	533.4766	487.669
TMEM164	0.988051	0.011949	0.836242	0.836171	1099.184	1314.547
MYNN	0.988055	0.011945	1.162452	1.162569	924.6928	795.3863
ZNF462	0.988056	0.011944	1.13691	1.137031	733.5303	645.1267
DCP2	0.988057	0.011943	0.80913	0.809078	1700.634	2101.943
PAN2	0.988057	0.011943	0.905805	0.905712	534.588	590.2414
SLC25A42	0.98806	0.01194	1.028373	1.028414	403.4417	392.2946
STK16	0.988063	0.011937	0.930429	0.930348	461.235	495.7668
CSNK1G2	0.988064	0.011936	0.872736	0.872646	716.8592	821.4793
CNOT6L	0.988065	0.011935	0.899945	0.899851	545.7021	606.4371
UBE2D1	0.988068	0.011932	1.176004	1.176116	1061.396	902.4576
LMAN2	0.988069	0.011931	0.766094	0.766069	4077.762	5322.97
PRPF4	0.988069	0.011931	1.204622	1.204719	1448.167	1202.077
ARAP3	0.988071	0.011929	1.193745	1.193848	1282.567	1074.311
EIF3H	0.988071	0.011929	1.276807	1.276863	3613.193	2829.74
CDK8	0.988074	0.011926	0.943073	0.943001	426.7813	452.5784
HAUS8	0.988075	0.011925	1.052954	1.053027	436.784	414.7886
NDUFA9	0.988077	0.011923	0.845491	0.845414	971.372	1148.991
TRUB2	0.988079	0.011921	0.99616	0.996154	388.9934	390.4951
ZNF467	0.98808	0.01192	0.993873	0.993864	388.9934	391.3949
RGP1	0.988085	0.011915	0.902692	0.902599	536.8108	594.7402
SMYD5	0.988087	0.011913	1.165022	1.165136	979.1518	840.3743
SOX13	0.988089	0.011911	1.186493	1.186598	1220.328	1028.424
ZNF212	0.988093	0.011907	0.978268	0.978237	393.439	402.192

SLC22A15	0.988103	0.011897	0.933869	0.933791	457.9007	490.3683
MPG	0.988104	0.011896	0.939043	0.938968	436.784	465.175
C4orf46	0.988111	0.011889	0.890727	0.890633	594.6041	667.6207
ANKFY1	0.988112	0.011888	0.821796	0.821736	1389.262	1690.646
CRBN	0.988116	0.011884	0.953879	0.953819	425.6699	446.2801
XPNPEP3	0.988118	0.011882	0.926936	0.926852	463.8801	500.4906
BRAF	0.988119	0.011881	1.085118	1.08522	514.5826	474.1726
IQCJ-SCH	0.98812	0.01188	1.089841	1.089946	519.6284	476.7459
WDR20	0.988121	0.011879	0.92326	0.923174	473.4605	512.8622
CCM2	0.988121	0.011879	1.203768	1.203864	1450.39	1204.776
ISG15	0.988122	0.011878	1.157462	1.157578	894.6847	772.8924
LYRM4	0.988124	0.011876	1.159134	1.159249	932.4726	804.3749
CACTIN	0.988124	0.011876	0.96791	0.967866	402.3303	415.6883
ATP6AP1	0.988125	0.011875	0.772252	0.772224	3677.654	4762.421
NXPH4	0.988128	0.011872	0.886846	0.886755	633.5035	714.4081
NENF	0.988129	0.011871	0.991559	0.991546	393.439	396.7934
ALPK1	0.988134	0.011866	0.950996	0.950933	431.2269	453.4782
METTL2B	0.988135	0.011865	0.865455	0.865369	768.7176	888.3134
SSSCA1	0.988136	0.011864	1.145781	1.145898	826.8887	721.6062
TYSND1	0.988137	0.011863	0.895976	0.895884	586.8243	655.024
PCM1	0.98814	0.01186	0.791135	0.791096	2379.528	3007.892
YIPF6	0.98815	0.01185	1.262874	1.262936	3039.705	2406.853
TRAPPC1	0.988151	0.011849	0.8352	0.835132	1169.203	1400.024
HMGCR	0.988151	0.011849	0.794931	0.794889	2255.05	2836.938
ZBTB22	0.988152	0.011848	0.980544	0.980517	397.8846	405.791
TMEM68	0.988156	0.011844	1.170799	1.17091	1052.505	898.8765
CAP2	0.988158	0.011842	1.150238	1.150354	843.5599	733.303
KLHDC4	0.988164	0.011836	1.030661	1.030704	425.6699	412.9891
CRLS1	0.988172	0.011828	0.860431	0.860348	821.3317	954.6526
H2AFJ	0.988176	0.011824	0.845735	0.84566	989.1545	1169.686
SCARB1	0.988176	0.011824	0.850795	0.850717	949.1438	1115.7
IRAK4	0.988179	0.011821	0.860347	0.860264	844.349	981.5013
ZDHHC9	0.98818	0.01182	1.261009	1.261072	2983.023	2365.465
PLK3	0.98818	0.01182	0.903115	0.903024	546.8135	605.5373
TIMM10B	0.988181	0.011819	0.914278	0.91419	519.0283	567.7475
ABHD17C	0.988184	0.011816	0.956284	0.956227	419.0014	438.1823
TSNARE1	0.988184	0.011816	0.986061	0.986042	408.9987	414.7886
SOCS4	0.988184	0.011816	0.829446	0.829381	1251.447	1508.895
UBAC1	0.988186	0.011814	1.156483	1.156598	899.1304	777.3912
C18orf8	0.988189	0.011811	0.857159	0.857079	879.125	1025.724
SPG20	0.988191	0.011809	0.821864	0.821805	1447.055	1760.827
CORO2A	0.988193	0.011807	0.87104	0.870953	732.4189	840.9411
TP53BP1	0.988194	0.011806	0.80421	0.804163	1911.624	2377.161
LOC10050	0.988199	0.011801	1.184435	1.18454	1192.631	1006.83
MED26	0.988201	0.011799	1.062906	1.062986	480.1289	451.6787
PPM1H	0.988203	0.011797	1.188729	1.188831	1281.455	1077.91
SENP1	0.988203	0.011797	0.87419	0.874102	710.1907	812.4817
RHOG	0.988205	0.011795	1.104767	1.104878	593.4927	537.1557
FAM45A	0.988211	0.011789	0.942937	0.942868	440.685	467.3884
PLPP5	0.988212	0.011788	1.048566	1.048631	443.4524	422.8864
CLPB	0.988213	0.011787	1.088345	1.088447	535.6994	492.1678
TMEM109	0.988216	0.011784	1.210533	1.210624	1640.441	1355.036
RAPGEF2	0.988224	0.011776	1.151517	1.151631	888.0163	771.0929
SNRPA	0.988228	0.011772	1.21817	1.218256	1754.916	1440.513
NOTUM	0.988228	0.011772	1.095526	1.095631	575.7102	525.4588
MGMT	0.98823	0.01177	0.900375	0.900285	564.5961	627.1315

DPP7	0.988235	0.011765	1.189108	1.189209	1263.673	1062.615
USP42	0.988238	0.011762	0.970392	0.970352	411.2215	423.7862
TBC1D12	0.988239	0.011761	0.953022	0.952963	439.0068	460.6763
NDOR1	0.988241	0.011759	0.874253	0.874165	715.7478	818.7801
THOC1	0.988241	0.011759	1.045941	1.046003	442.341	422.8864
BEX4	0.988243	0.011757	0.927589	0.927508	475.6833	512.8622
PIGX	0.988244	0.011756	0.906719	0.90663	540.4563	596.1169
USP45	0.988244	0.011756	0.92131	0.921226	492.3544	534.4564
TMCO3	0.988248	0.011752	1.24269	1.242762	2383.974	1918.285
FLNB	0.988249	0.011751	1.365189	1.36521	14008.21	10260.84
AP4M1	0.988251	0.011749	1.033349	1.033395	426.7813	412.9891
TXNDC11	0.988254	0.011746	1.126282	1.126398	690.1853	612.7354
GTF3C2	0.988255	0.011745	0.828555	0.828492	1288.124	1554.782
FGD1	0.988255	0.011745	1.004648	1.004655	406.7759	404.8912
AIDA	0.988256	0.011744	1.269154	1.269212	3320.892	2616.497
CCDC18	0.988257	0.011743	1.085543	1.085642	532.3652	490.3683
HOMER1	0.98826	0.01174	0.905057	0.904967	539.0336	595.64
IRGQ	0.988264	0.011736	1.132691	1.132807	741.9659	654.9791
GRWD1	0.988269	0.011731	0.915588	0.915502	512.3598	559.6497
MRPL42	0.988273	0.011727	0.842884	0.842812	1050.282	1246.165
NF1	0.988274	0.011726	1.224469	1.224551	1911.624	1561.081
NET1	0.988275	0.011725	0.833907	0.83384	1193.654	1431.515
TMEM55B	0.988278	0.011722	1.093413	1.093516	574.5988	525.4588
FHDC1	0.988279	0.011721	1.054331	1.054402	460.1236	436.3828
SPSB3	0.98828	0.01172	1.014342	1.014362	413.4444	407.5905
SUGP1	0.988284	0.011716	0.897084	0.896995	601.2726	670.3199
DNAJC21	0.988285	0.011715	0.820026	0.819969	1483.899	1809.702
SORT1	0.988296	0.011704	1.248696	1.248764	2666.272	2135.126
KLF3	0.988296	0.011704	1.175373	1.175479	1136.972	967.2402
MSL3	0.988301	0.011699	1.104889	1.104997	622.3894	563.2487
EIF2AK2	0.98831	0.01169	1.242674	1.242745	2415.149	1943.397
C2CD2	0.988311	0.011689	1.132739	1.132853	750.2015	662.2221
ACVR1	0.988314	0.011686	1.057623	1.057697	470.1263	444.4806
HIP1R	0.988317	0.011683	0.830879	0.830815	1260.338	1516.993
NPIPA1	0.988318	0.011682	0.912711	0.912626	541.0008	592.7968
PPM1K	0.988318	0.011682	0.98327	0.983247	422.6579	429.8595
ZDHHC3	0.988319	0.011681	1.180933	1.181036	1180.295	999.3705
GPANK1	0.988321	0.011679	0.981778	0.981754	423.4471	431.3171
RASAL1	0.988323	0.011677	1.099229	1.099334	602.384	547.9528
TARBP1	0.988329	0.011671	0.829343	0.829281	1325.912	1598.871
EEF2	0.988329	0.011671	1.421615	1.421624	36271.96	25514.45
OCEL1	0.988336	0.011664	1.048861	1.048925	456.7893	435.483
CWC25	0.988336	0.011664	0.998478	0.998476	423.1581	423.8042
TMED4	0.988338	0.011662	1.286196	1.286244	4273.459	3322.43
FBRSL1	0.988339	0.011661	0.938938	0.938867	461.235	491.268
ZNF37A	0.988342	0.011658	0.836511	0.836445	1195.877	1429.716
MX1	0.988345	0.011655	0.962729	0.962681	427.8927	444.4806
ZNF275	0.988347	0.011653	0.876747	0.876661	714.6364	815.181
DVL1	0.988347	0.011653	1.204693	1.204784	1539.302	1277.657
TBCC	0.988351	0.011649	0.921071	0.92099	517.9169	562.3489
RGS14	0.988351	0.011649	1.081297	1.081391	531.2538	491.268
CLSPN	0.988352	0.011648	0.836494	0.836427	1169.225	1397.882
EMC6	0.988356	0.011644	0.981406	0.981381	416.7786	424.6859
URB2	0.988362	0.011638	1.019106	1.019132	424.5585	416.5881
RNF2	0.988363	0.011637	0.85966	0.859581	864.6767	1005.93
CPSF4	0.988368	0.011632	0.813668	0.813617	1679.34	2064.046

NDFIP2	0.988368	0.011632	1.18778	1.187879	1282.567	1079.71
SEMA4D	0.988368	0.011632	0.991356	0.991344	415.8006	419.4313
REL	0.988371	0.011629	1.037129	1.037179	443.4524	427.5561
ZMYM6	0.988372	0.011628	1.091696	1.091797	566.8189	519.1605
EP400	0.988372	0.011628	0.828166	0.828105	1329.246	1605.169
PCYT1A	0.988373	0.011627	0.893183	0.893095	617.9437	691.9141
ACTR3B	0.988373	0.011627	0.891278	0.89119	628.0687	704.7537
CISD1	0.988374	0.011626	0.829181	0.829119	1309.24	1579.076
MTFP1	0.988375	0.011625	1.052259	1.052326	466.792	443.5808
STX3	0.988377	0.011623	0.81215	0.8121	1770.475	2180.123
PPP1R9B	0.988379	0.011621	0.843069	0.842999	1072.51	1272.258
NDUFS8	0.988383	0.011617	0.802425	0.802381	2056.108	2562.512
EHHADH	0.988385	0.011615	1.043367	1.043424	453.4551	434.5833
AP3S2	0.988387	0.011613	1.161691	1.161799	1013.217	872.1087
SPTSSA	0.988388	0.011612	1.222409	1.222489	1959.004	1602.47
TRAPPC1	0.988388	0.011612	0.976667	0.976637	432.3383	442.6811
SMIM12	0.98839	0.01161	1.127945	1.128057	734.6528	651.2541
ATXN2L	0.988392	0.011608	0.756841	0.756822	5417.01	7157.577
MRPL41	0.988399	0.011601	0.871195	0.871111	765.7612	879.0639
HNRNPH2	0.988403	0.011597	0.844941	0.84487	1051.06	1244.051
CLMN	0.988406	0.011594	0.918639	0.918557	519.0283	565.0482
USP21	0.988411	0.011589	1.096732	1.096834	590.1585	538.0555
DOCK9	0.988416	0.011584	1.18871	1.188807	1309.24	1101.304
KLC3	0.988417	0.011583	1.147097	1.147208	869.1223	757.5965
TMEM106	0.988417	0.011583	1.07964	1.079731	547.9249	507.4637
ZNF721	0.98842	0.01158	0.943159	0.943094	473.4938	502.0651
DUSP12	0.988422	0.011578	1.051247	1.051312	470.1263	447.1799
ST6GALN	0.988424	0.011576	1.165413	1.165518	1059.173	908.7559
TBC1D25	0.988425	0.011575	1.095862	1.095963	603.4954	550.6521
CENPT	0.988425	0.011575	0.979693	0.979667	434.5611	443.5808
AP3M2	0.988433	0.011567	0.960259	0.960209	450.1209	468.7741
EIF3C	0.988433	0.011567	0.92403	0.923951	506.4471	548.1328
KAT2A	0.988433	0.011567	1.176883	1.176985	1190.32	1011.328
SFSWAP	0.988434	0.011566	0.931917	0.931842	477.9061	512.8622
VPS18	0.98844	0.01156	0.896364	0.896277	611.2753	682.0168
MRPL15	0.98844	0.01156	0.808458	0.808411	1867.168	2309.68
EIF1B	0.98844	0.01156	0.86271	0.862631	849.1169	984.3356
NUBP2	0.988441	0.011559	1.108923	1.109029	644.6176	581.2439
TMEM63A	0.988448	0.011552	1.009699	1.009712	437.8954	433.6835
SOS2	0.98845	0.01155	0.833734	0.83367	1243.667	1491.799
CDC40	0.988452	0.011548	1.109883	1.10999	666.868	600.7866
RBSN	0.988456	0.011544	1.173953	1.174056	1132.426	964.5409
LIFR	0.988456	0.011544	0.932887	0.932814	486.7974	521.8598
BPGM	0.988458	0.011542	1.236352	1.236424	2301.729	1861.6
CENPE	0.988458	0.011542	1.216443	1.216526	1807.152	1485.501
NSUN2	0.988458	0.011542	0.781708	0.781677	3137.509	4013.822
CSTF2T	0.988462	0.011538	1.021962	1.021991	436.784	427.3852
TFAP2A	0.988462	0.011538	0.886075	0.88599	687.9625	776.4914
KIF18B	0.988462	0.011538	1.137337	1.137448	806.7389	709.2525
ZNF468	0.988474	0.011526	1.209686	1.209773	1675.861	1385.268
ANKRD49	0.988477	0.011523	0.919094	0.919013	516.8055	562.3489
SERTAD3	0.988478	0.011522	1.016768	1.01679	434.5611	427.3852
PUSL1	0.988479	0.011521	0.981094	0.981069	429.0041	437.2825
F2R	0.988483	0.011517	1.04762	1.04768	470.6042	449.1863
PRPF38B	0.988486	0.011514	1.163279	1.163384	1025.831	881.7631
ZGPAT	0.98849	0.01151	1.053038	1.053105	480.8958	456.6453

COPS7B	0.988493	0.011507	0.911022	0.910939	555.7048	610.0361
ERO1B	0.988496	0.011504	0.893043	0.892958	652.3974	730.6037
LMF2	0.988502	0.011498	1.199724	1.199814	1543.748	1286.654
CNNM3	0.988503	0.011497	1.005664	1.005671	441.8075	439.316
PRICKLE3	0.988504	0.011496	0.897287	0.897203	631.2806	703.611
NUFIP2	0.988506	0.011494	1.28979	1.289834	4856.86	3765.489
C1orf112	0.988506	0.011494	1.035628	1.035674	455.6779	439.9818
COX8A	0.988509	0.011491	0.774244	0.774218	3719.888	4804.709
PCED1A	0.988513	0.011487	0.938412	0.938344	481.2403	512.8622
DHX34	0.988519	0.011481	0.992696	0.992686	441.2296	444.4806
POLR3K	0.988519	0.011481	0.977712	0.977684	434.5611	444.4806
SNN	0.988527	0.011473	0.875722	0.87564	771.3182	880.8634
WIPI2	0.988528	0.011472	1.161719	1.161824	1020.274	878.1641
IMPA1	0.988528	0.011472	0.973397	0.973363	437.8954	449.8792
ACAA2	0.988532	0.011468	0.905004	0.90492	584.6014	646.0265
C10orf2	0.988532	0.011468	1.041707	1.04176	466.792	448.0796
ZMYND19	0.988535	0.011465	0.914043	0.913962	550.1477	601.9383
GRSF1	0.988536	0.011464	0.789907	0.789872	2698.502	3416.382
PHKG2	0.988537	0.011463	1.133129	1.133238	777.9867	686.5156
SYNJ2	0.988537	0.011463	0.980872	0.980847	445.6752	454.3779
KIAA0922	0.98854	0.01146	0.929051	0.928977	505.6914	544.3538
ARL6IP6	0.98854	0.01146	1.056659	1.056728	493.4659	466.9746
N4BP3	0.98854	0.01146	1.16696	1.167063	1103.63	945.646
ELL	0.98854	0.01146	1.096992	1.097092	609.0525	555.1509
POLD4	0.988541	0.011459	1.156464	1.156569	998.3014	863.1561
DENND1B	0.988541	0.011459	0.93473	0.93466	504.5799	539.855
C19orf70	0.988543	0.011457	0.91472	0.914638	539.0336	589.3417
GSTK1	0.988545	0.011455	0.788048	0.788014	2806.309	3561.243
TSPAN1	0.988548	0.011452	1.185643	1.18574	1304.795	1100.404
METTL15	0.988549	0.011451	0.946625	0.946564	470.1263	496.6666
CLP1	0.988555	0.011445	1.018276	1.0183	443.4524	435.483
CSTF1	0.988555	0.011445	0.850285	0.850213	1013.606	1192.18
MAFG	0.988556	0.011444	0.807309	0.807263	1953.858	2420.35
TFIP11	0.98856	0.01144	0.872865	0.872785	800.2149	916.8537
SLC35C1	0.988561	0.011439	1.007611	1.007621	437.8954	434.5833
SDF4	0.988564	0.011436	1.2276	1.227675	2167.249	1765.326
CUL3	0.988565	0.011435	0.799215	0.799174	2228.376	2788.351
MAN2C1	0.988567	0.011433	1.053933	1.053999	491.243	466.0748
TMEM259	0.988573	0.011427	1.240057	1.240125	2534.014	2043.351
SEMA4C	0.988574	0.011426	0.909695	0.909613	571.2645	628.0313
USP38	0.988578	0.011422	0.852972	0.852899	966.9263	1133.695
ZFP91	0.98858	0.01142	0.785983	0.785951	3021.011	3843.767
C16orf52	0.988582	0.011418	1.055321	1.055388	495.6887	469.6738
SMAD6	0.988582	0.011418	0.974837	0.974806	453.4551	465.175
ARL3	0.988588	0.011412	0.972658	0.972624	444.5638	457.0772
CCNG2	0.988595	0.011405	0.899761	0.899678	616.8323	685.6158
B4GAT1	0.988598	0.011402	0.925027	0.924952	523.4739	565.948
RBM12	0.988599	0.011401	1.25783	1.257888	3204.127	2547.225
TRAPPC6	0.988601	0.011399	0.832225	0.832164	1314.798	1579.976
CCDC22	0.988601	0.011399	1.110536	1.110638	693.5196	624.4323
AFMID	0.988604	0.011396	0.914546	0.914466	557.3052	609.4333
ARHGAP2	0.988605	0.011395	0.866453	0.866375	844.8824	975.194
CD3EAP	0.988608	0.011392	1.180965	1.181062	1259.138	1066.106
RASSF7	0.988612	0.011388	1.14056	1.140666	858.0082	752.1979
CCZ1B	0.988615	0.011385	0.847934	0.847865	1059.218	1249.278
AXL	0.988618	0.011382	1.181695	1.181791	1271.719	1076.093

BCAS2	0.988619	0.011381	0.874207	0.874127	780.2095	892.5602
TUT1	0.988624	0.011376	0.926993	0.926919	521.2511	562.3489
CFAP97	0.988627	0.011373	0.863251	0.863175	902.4646	1045.519
YWHAE	0.988629	0.011371	1.337996	1.338022	9893.312	7393.98
NECAB3	0.988632	0.011368	0.893205	0.893122	655.7317	734.2028
TRIB1	0.988633	0.011367	0.966026	0.965985	454.5665	470.5736
SH3KBP1	0.988638	0.011362	1.257452	1.257511	3136.398	2494.13
ZNF813	0.988638	0.011362	1.01788	1.017903	462.9799	454.8368
TULP4	0.98864	0.01136	1.079406	1.079492	562.3732	520.9601
CCAR1	0.988641	0.011359	0.813232	0.813184	1840.372	2263.171
POC1B	0.988642	0.011358	1.037839	1.037886	473.4605	456.1775
CAD	0.988645	0.011355	0.801611	0.80157	2208.371	2755.06
GJB2	0.988659	0.011341	0.858525	0.858451	936.9183	1091.407
ZNF142	0.98866	0.01134	0.911548	0.911468	575.7102	631.6303
RUNDC1	0.988661	0.011339	1.071674	1.071754	556.5383	519.2775
CDK17	0.988666	0.011334	0.920409	0.920333	545.7021	592.9407
CXorf56	0.988666	0.011334	1.131614	1.131719	826.5442	730.3428
LRP3	0.988671	0.011329	1.164715	1.164815	1084.736	931.2498
POLR1D	0.988672	0.011328	1.189947	1.190038	1404.822	1180.483
MRPL44	0.988673	0.011327	1.058699	1.058768	513.4712	484.9697
TADA2B	0.988681	0.011319	0.911737	0.911659	592.1701	649.5535
FGD4	0.988682	0.011318	1.083664	1.083753	581.2672	536.3459
GNB4	0.988685	0.011315	1.1324	1.132505	814.9411	719.5907
MBIP	0.988689	0.011311	1.084012	1.0841	597.9384	551.5518
DEF8	0.98869	0.01131	1.153375	1.153477	1003.603	870.0663
WSB1	0.98869	0.01131	0.826023	0.825968	1473.729	1784.248
SLPI	0.988691	0.011309	0.75773	0.757712	5782.664	7631.75
MSRB1	0.988693	0.011307	1.055411	1.055476	509.0256	482.2705
PUS1	0.988694	0.011306	0.989154	0.989141	460.1236	465.175
MCM10	0.988696	0.011304	0.889618	0.889537	703.5223	790.8875
MED23	0.988697	0.011303	0.884942	0.884861	715.7478	808.8827
NUTF2	0.988706	0.011294	1.19549	1.195578	1541.525	1289.354
GAS2L1	0.988707	0.011293	1.051741	1.051803	503.4685	478.6714
CRCP	0.98871	0.01129	1.177584	1.177679	1275.898	1083.399
PEX2	0.988712	0.011288	1.158406	1.158507	1056.951	912.3369
EMC2	0.988713	0.011287	1.103447	1.103546	661.2887	599.239
ORC2	0.988714	0.011286	0.86744	0.867363	856.8968	987.9346
SGPL1	0.988716	0.011284	0.791574	0.79154	2781.858	3514.492
PCNXL2	0.988716	0.011284	0.904032	0.903951	603.4954	667.6207
ST7	0.988723	0.011277	1.151202	1.151303	991.3218	861.0417
RARA	0.988729	0.011271	1.026084	1.026117	469.0148	457.0772
CERS1	0.98873	0.01127	0.990794	0.990782	452.8105	457.0232
TSKU	0.988732	0.011268	0.848203	0.848135	1083.624	1277.657
CYB5A	0.988733	0.011267	1.090863	1.090955	613.4981	562.3489
ABCF3	0.988739	0.011261	0.864005	0.863931	918.0243	1062.615
KAT5	0.988742	0.011258	0.962297	0.962253	470.1263	488.5688
ZNF329	0.988743	0.011257	1.065595	1.065669	537.9222	504.7734
KLHL20	0.988747	0.011253	0.995317	0.995311	465.6806	467.8743
DCTN4	0.988748	0.011252	0.804379	0.804337	2147.243	2669.583
SLC4A11	0.988754	0.011246	1.083847	1.083935	581.2672	536.2559
RIC8A	0.988754	0.011246	1.230485	1.230556	2340.629	1902.089
IQCB1	0.988765	0.011235	1.159078	1.159178	1050.282	906.0566
CEP97	0.988771	0.011229	1.0924	1.092492	624.7233	571.8324
C19orf52	0.988771	0.011229	0.963297	0.963254	472.3491	490.3683
NGRN	0.988778	0.011222	1.232841	1.23291	2362.857	1916.485
MED15	0.988778	0.011222	1.230512	1.230582	2353.965	1912.886

TMEM55A	0.988778	0.011222	0.963479	0.963437	484.5746	502.9649
PEAK1	0.988779	0.011221	1.141605	1.141707	922.4811	807.983
TROAP	0.98878	0.01122	0.887947	0.887867	710.1907	799.8851
C20orf194	0.988784	0.011216	1.152522	1.152623	992.4888	861.0687
UCP2	0.988784	0.011216	0.807242	0.807199	2056.108	2547.216
ARRB2	0.988784	0.011216	0.926972	0.926901	541.2565	583.9431
CWC22	0.988787	0.011213	0.991477	0.991467	470.1263	474.1726
B3GAT3	0.988789	0.011211	0.869862	0.869786	849.1169	976.2378
WBP5	0.988791	0.011209	1.244723	1.244786	2750.739	2209.806
NUP37	0.988796	0.011204	1.119477	1.119577	774.6525	691.9141
FKTN	0.9888	0.0112	0.85889	0.858819	963.5921	1121.999
FBXO28	0.988804	0.011196	0.878547	0.878469	799.1035	909.6556
RAI14	0.988806	0.011194	1.341261	1.341285	10795.12	8048.338
PPP1R14A	0.988807	0.011193	1.059024	1.059091	521.2511	492.1678
DDX31	0.98881	0.01119	1.067009	1.067083	550.1477	515.5615
USP14	0.988811	0.011189	0.794955	0.794919	2595.164	3264.692
NOL4L	0.988814	0.011186	1.123126	1.123227	780.2095	694.6134
SFT2D1	0.988817	0.011183	1.18929	1.189378	1441.498	1211.974
RNPEP	0.98882	0.01118	1.239926	1.239991	2605.144	2100.936
MOCS1	0.98882	0.01118	0.886949	0.88687	710.1907	800.7849
FAM57A	0.988822	0.011178	0.952884	0.952832	491.243	515.5615
C11orf57	0.988824	0.011176	0.865922	0.865848	893.5733	1032.023
HSP90B1	0.988824	0.011176	0.683351	0.683348	52241.81	76449.75
C9orf114	0.988826	0.011174	1.074757	1.074837	573.4873	533.5567
NIPA1	0.988829	0.011171	1.234031	1.234099	2425.096	1965.072
RANBP6	0.98883	0.01117	1.211832	1.21191	1906.067	1572.778
TM7SF3	0.988838	0.011162	0.8099	0.809856	2035.58	2513.511
PLPP2	0.988845	0.011155	1.054668	1.05473	539.0336	511.0627
STK38L	0.988848	0.011152	0.872536	0.872461	858.0082	983.4358
FAM127B	0.988851	0.011149	0.858634	0.858564	1002.403	1167.535
USP53	0.988852	0.011148	1.148401	1.1485	971.372	845.7728
ELK1	0.988856	0.011144	0.839525	0.839465	1284.789	1530.489
MB21D2	0.988858	0.011142	0.952033	0.951981	496.8001	521.8598
SMIM7	0.98886	0.01114	0.852603	0.852535	1054.728	1237.168
ZBTB33	0.988861	0.011139	1.176755	1.176848	1277.01	1085.109
YIF1A	0.988861	0.011139	0.817497	0.817449	1754.916	2146.823
SLAIN2	0.988864	0.011136	0.827325	0.827271	1539.302	1860.7
ZNF654	0.988866	0.011134	1.036103	1.036146	509.0256	491.268
IPO11	0.988867	0.011133	0.872207	0.872132	843.5599	967.2402
TMEM41A	0.988869	0.011131	0.962958	0.962917	495.6887	514.7787
ITGA6	0.988869	0.011131	1.100911	1.101005	674.6256	612.7354
LRIF1	0.98887	0.01113	1.086603	1.086689	617.9437	568.6472
ACAT1	0.988871	0.011129	0.824296	0.824244	1615.99	1960.573
COX14	0.988872	0.011128	1.092968	1.093058	657.9545	601.9383
PXK	0.988873	0.011127	0.817341	0.817293	1808.263	2212.506
PTDSS2	0.988873	0.011127	0.95981	0.959765	487.9088	508.3634
PFDN4	0.988875	0.011125	0.877305	0.877229	822.4431	937.5482
SLC36A4	0.988878	0.011122	0.94232	0.942261	529.031	561.4492
TIGAR	0.988883	0.011117	0.906487	0.906409	616.6767	680.3522
SLC39A14	0.988884	0.011116	1.289188	1.28923	5094.702	3951.738
CDKN3	0.988884	0.011116	1.185431	1.18552	1404.822	1184.982
AK1	0.988888	0.011112	1.081985	1.082069	604.6068	558.7499
DIP2A	0.98889	0.01111	1.19654	1.196625	1585.726	1325.164
BAZ2B	0.988891	0.011109	0.888412	0.888334	725.7505	816.9805
LPCAT3	0.988896	0.011104	1.064783	1.064853	555.7048	521.8598
QRICH1	0.988896	0.011104	0.844845	0.844781	1175.871	1391.926

OXNAD1	0.988896	0.011104	0.944888	0.94483	513.4712	543.454
PHLDB1	0.988899	0.011101	1.23182	1.231887	2457.327	1994.764
DCAF6	0.9889	0.0111	1.170801	1.170895	1213.659	1036.522
GALNT6	0.988908	0.011092	1.155783	1.15588	1052.505	910.5644
BCL7B	0.988912	0.011088	0.926955	0.926886	557.9276	601.9383
MGRN1	0.988912	0.011088	1.133165	1.133265	864.6767	762.995
ZKSCAN5	0.988913	0.011087	0.883863	0.883786	760.2042	860.1689
SLC35A5	0.988916	0.011084	0.867677	0.867604	918.0243	1058.116
RAB3GAP	0.988918	0.011082	0.84358	0.843518	1232.553	1461.207
RHPN1	0.988921	0.011079	1.000841	1.000842	474.5719	474.1726
FPGS	0.988921	0.011079	1.121692	1.12179	791.3236	705.4105
TMEM39B	0.988923	0.011077	1.017641	1.017662	483.4632	475.0724
UBE2L6	0.988925	0.011075	0.838278	0.838219	1328.134	1584.474
SMARCA2	0.988925	0.011075	1.214264	1.21434	2002.76	1649.257
EXOSC2	0.988928	0.011072	1.115526	1.115623	756.8699	678.4268
EIF4EBP1	0.988929	0.011071	0.80434	0.8043	2236.156	2780.253
SIRT7	0.988931	0.011069	0.960779	0.960736	505.6914	526.3586
SEC31A	0.988931	0.011069	1.300357	1.300394	6032.731	4639.154
NEDD8	0.988933	0.011067	1.164783	1.164878	1152.921	989.7341
DNAJA1	0.988935	0.011065	1.314733	1.314765	7440.887	5659.48
YRDC	0.988935	0.011065	0.894748	0.894672	712.4135	796.2861
GALK2	0.988936	0.011064	0.913145	0.913071	607.941	665.8211
ASB1	0.98894	0.01106	1.054032	1.054092	536.8108	509.2632
SZT2	0.988941	0.011059	0.897261	0.897184	699.0766	779.1907
ANKMY2	0.988942	0.011058	0.980474	0.980451	479.0175	488.5688
C14orf159	0.988945	0.011055	0.869311	0.869238	908.0216	1044.619
PELO	0.988946	0.011054	1.141368	1.141466	933.584	817.8803
RALGAPA	0.988947	0.011053	0.926928	0.926859	562.7734	607.1839
CRY1	0.988948	0.011052	0.866312	0.86624	915.8015	1057.216
SLC25A25	0.988951	0.011049	1.192115	1.192199	1570.422	1317.246
NMU	0.988951	0.011049	0.974972	0.974943	474.5719	486.7692
CCDC14	0.988952	0.011048	1.226794	1.226864	2281.724	1859.8
DAPK1	0.988952	0.011048	0.915366	0.915293	616.8323	673.919
PHKA2	0.988954	0.011046	1.129684	1.129783	833.5572	737.8018
NUDT15	0.98896	0.01104	0.851166	0.851101	1098.984	1291.252
DPP3	0.98896	0.01104	1.183478	1.183566	1400.376	1183.182
TYW1	0.988963	0.011037	0.932247	0.932182	550.2367	590.2684
ACOT8	0.988965	0.011035	0.901781	0.901705	679.0713	753.0977
PARVB	0.988967	0.011033	1.056554	1.056617	545.7021	516.4613
PRRC2B	0.988967	0.011033	1.322383	1.322411	8597.865	6501.654
TFAP2C	0.988973	0.011027	1.044478	1.044528	535.6994	512.8622
UNC13B	0.988974	0.011026	0.964387	0.964348	494.5773	512.8622
NDUFAB1	0.988975	0.011025	0.822619	0.822569	1657.112	2014.559
UBN2	0.988976	0.011024	1.132891	1.132989	875.8019	773.0004
AP1G2	0.988978	0.011022	1.08646	1.086545	633.5035	583.0434
TTLL5	0.988982	0.011018	1.144171	1.144268	964.7035	843.0735
SLC25A28	0.988984	0.011016	1.058408	1.058471	565.7075	534.4564
ZNF532	0.988992	0.011008	0.94238	0.942323	543.4793	576.7451
FBXL12	0.988992	0.011008	0.887858	0.887783	746.8672	841.274
EML4	0.988993	0.011007	0.808249	0.808207	2116.124	2618.297
GLMP	0.988995	0.011005	0.895585	0.895508	700.188	781.89
GNAI3	0.988998	0.011002	1.26568	1.26573	3784.405	2989.897
GDAP2	0.989001	0.010999	1.061264	1.06133	551.2592	519.4035
C4orf3	0.989002	0.010998	1.169343	1.169436	1224.773	1047.319
MKNK1	0.989004	0.010996	0.949918	0.949865	517.9169	545.2535
TMEM147	0.989011	0.010989	0.792681	0.792648	2904.113	3663.816

NAA40	0.989013	0.010987	0.915393	0.915321	625.7458	683.6364
MCM5	0.989014	0.010986	0.85142	0.851355	1109.187	1302.85
SAMM50	0.989014	0.010986	0.843301	0.84324	1233.665	1463.007
BYSL	0.989016	0.010984	0.918012	0.917941	601.2726	655.024
TAF12	0.989022	0.010978	0.862802	0.862733	993.6002	1151.691
TCAIM	0.989024	0.010976	1.053028	1.053086	559.039	530.8574
POLR3D	0.989026	0.010974	0.929224	0.929157	566.8189	610.0361
POLR3GL	0.98903	0.01097	0.952592	0.952542	516.8055	542.5543
SLIT3	0.989031	0.010969	0.908751	0.908677	640.1719	704.5108
RNF8	0.989033	0.010967	0.876159	0.876085	826.8887	943.8465
FAM189B	0.989036	0.010964	0.891236	0.891161	751.3129	843.0735
MED24	0.989037	0.010963	1.086919	1.087002	660.1773	607.3369
RAB4A	0.989038	0.010962	1.132659	1.132755	909.133	802.5844
PUDP	0.989048	0.010952	0.88707	0.886996	781.3209	880.8634
FKBP2	0.98905	0.01095	0.835616	0.835559	1384.816	1657.355
WDR75	0.989052	0.010948	1.171159	1.171249	1261.45	1077.011
MPPE1	0.989053	0.010947	0.980838	0.980816	503.8353	513.69
GZF1	0.989054	0.010946	0.933929	0.933866	557.9276	597.4395
FAM219B	0.989055	0.010945	0.993037	0.993029	501.2457	504.7644
KCTD3	0.989057	0.010943	0.895448	0.895373	729.0847	814.2813
ALKBH5	0.98906	0.01094	1.234646	1.23471	2637.375	2136.026
DIEXF	0.989068	0.010932	0.944071	0.944015	536.8108	568.6472
MRPL50	0.989074	0.010926	0.931126	0.931061	557.9276	599.239
HNMT	0.989075	0.010925	0.969015	0.968981	493.4659	509.2632
SUPT5H	0.989081	0.010919	1.293321	1.293359	5638.181	4359.329
ATF1	0.989083	0.010917	0.904012	0.903939	670.18	741.4008
OFD1	0.989083	0.010917	0.976012	0.975986	497.9115	510.163
SERINC3	0.989083	0.010917	1.254456	1.254511	3326.449	2651.588
MTG2	0.989088	0.010912	1.069343	1.069414	583.9013	546.0003
PLK4	0.989089	0.010911	1.068994	1.069064	593.4927	555.1509
LSM3	0.98909	0.01091	1.195459	1.19554	1640.441	1372.131
GAR1	0.989092	0.010908	1.087436	1.087519	654.6202	601.9383
NACC2	0.989094	0.010906	0.904833	0.90476	684.6283	756.6967
MFAP3	0.989094	0.010906	1.212894	1.212967	1998.314	1647.457
PNO1	0.989097	0.010903	0.834654	0.834599	1419.27	1700.543
RNF220	0.989102	0.010898	1.128724	1.128819	872.4565	772.8924
TSTA3	0.989103	0.010897	0.791039	0.791008	2986.358	3775.386
SLC35F2	0.989103	0.010897	1.235167	1.23523	2680.72	2170.217
RNASEH2	0.989103	0.010897	1.102612	1.102701	721.3048	654.1243
RBX1	0.989104	0.010896	1.095738	1.095825	690.1853	629.8308
SLC30A1	0.989104	0.010896	1.051159	1.051215	552.3706	525.4588
POMT1	0.989105	0.010895	1.089181	1.089265	663.5115	609.1364
PPAN	0.989106	0.010894	0.91886	0.91879	611.253	665.2813
MOB3A	0.98911	0.01089	0.993846	0.993839	507.9142	511.0627
MYCBP2	0.989116	0.010884	0.841684	0.841626	1323.689	1572.778
MAP3K13	0.989117	0.010883	0.921866	0.921798	602.3395	653.4405
RB1CC1	0.989124	0.010876	1.260889	1.26094	3652.092	2896.322
NEK4	0.989127	0.010873	1.067807	1.067876	595.7155	557.8501
TAF2	0.989127	0.010873	0.860959	0.860891	1014.717	1178.683
ENPP5	0.989131	0.010869	0.98351	0.983492	512.3598	520.9601
MAST3	0.989134	0.010866	0.997845	0.997842	499.0785	500.1576
SEC61G	0.989138	0.010862	1.259488	1.25954	3685.434	2926.014
OSER1	0.989138	0.010862	1.190551	1.190634	1572.645	1320.845
ZNF302	0.989144	0.010856	1.00788	1.007889	503.4685	499.5278
GNB5	0.989144	0.010856	1.081217	1.081295	640.1719	592.041
NARS2	0.989147	0.010853	1.120845	1.120938	850.2283	758.4963

NPC2	0.989148	0.010852	1.284902	1.284943	5084.699	3957.137
EMC8	0.989154	0.010846	1.095436	1.095521	714.6364	652.3248
BAMBI	0.989161	0.010839	1.083293	1.083372	650.1746	600.1388
SLC41A3	0.989163	0.010837	1.128508	1.128601	905.7988	802.5844
CCNDBP1	0.989165	0.010835	1.128913	1.129007	886.9048	785.561
SELK	0.989165	0.010835	1.165161	1.165251	1221.439	1048.218
SMG1P5	0.989165	0.010835	0.853442	0.853379	1148.564	1345.903
SLC27A4	0.989176	0.010824	1.111617	1.111707	800.2149	719.8066
CLPX	0.989176	0.010824	0.885197	0.885124	784.833	886.6938
TNPO2	0.989178	0.010822	1.248418	1.248474	3143.066	2517.524
CD99L2	0.989182	0.010818	1.092416	1.0925	689.0739	630.7306
CCDC137	0.989185	0.010815	0.852783	0.85272	1134.749	1330.743
SLC12A6	0.989188	0.010812	1.074582	1.074655	640.3497	595.8649
OSBPL1A	0.98919	0.01081	0.914142	0.914072	657.9545	719.8066
NMI	0.98919	0.01081	0.906706	0.906634	674.6256	744.1001
FCHO2	0.989192	0.010808	0.946107	0.946054	549.0363	580.3441
ZYG11A	0.989193	0.010807	1.012793	1.012807	524.9076	518.2698
ZBTB17	0.989197	0.010803	0.962135	0.962095	524.5853	545.2535
GLMN	0.989197	0.010803	1.047074	1.047124	556.8162	531.7572
RIC8B	0.989204	0.010796	1.028272	1.028303	541.2565	526.3586
CBX7	0.989208	0.010792	0.914998	0.914929	641.2833	700.9117
MIB2	0.989216	0.010784	0.997773	0.997771	509.0256	510.163
ZNF800	0.989218	0.010782	1.058147	1.058207	597.9384	565.0482
COMMD6	0.989223	0.010777	1.148455	1.148547	1060.285	923.152
SQLE	0.989225	0.010775	0.834939	0.834885	1492.623	1787.82
DST	0.989226	0.010774	1.23031	1.230373	2534.014	2059.547
GTPBP10	0.989227	0.010773	0.839358	0.839302	1368.356	1630.353
ATP5F1	0.989228	0.010772	0.796283	0.79625	2726.31	3423.94
FRS2	0.989229	0.010771	0.848456	0.848396	1211.436	1427.916
LYST	0.989229	0.010771	0.942071	0.942016	562.3732	596.9896
RSL24D1	0.98923	0.01077	1.217405	1.217475	2133.906	1752.729
GEMIN7	0.98923	0.01077	1.006477	1.006485	513.4712	510.163
DCP1A	0.989231	0.010769	1.068715	1.068784	613.5314	574.0458
DUSP16	0.989235	0.010765	0.924389	0.924324	611.2753	661.3224
HEXA	0.989237	0.010763	0.87683	0.87676	878.0136	1001.431
ABCC4	0.989238	0.010762	1.099754	1.099839	731.3075	664.9214
CWF19L1	0.989239	0.010761	0.877084	0.877014	898.0189	1023.952
SAMHD1	0.98924	0.01076	0.829563	0.829512	1581.536	1906.588
GFM2	0.98924	0.01076	0.854957	0.854894	1119.189	1309.157
ARL13B	0.98924	0.01076	0.981002	0.980982	514.5826	524.5591
CAPG	0.989241	0.010759	1.182979	1.183062	1487.066	1256.962
SEC22A	0.989242	0.010758	1.090842	1.090923	680.2271	623.5325
UBQLN2	0.989245	0.010755	1.14995	1.150041	1080.29	939.3477
LIMD1	0.989246	0.010754	1.008398	1.008408	529.0421	524.6311
RIPK2	0.989248	0.010752	1.226161	1.226226	2421.761	1974.969
CDK5	0.989251	0.010749	0.926488	0.926425	606.8296	655.024
MRPS34	0.989251	0.010749	0.872995	0.872926	915.8015	1049.118
SRFBP1	0.989252	0.010748	0.898666	0.898595	744.6444	828.6774
DAB2IP	0.989253	0.010747	1.157453	1.157542	1159.2	1001.431
CIT	0.989257	0.010743	1.15999	1.160079	1218.105	1050.018
RHBDD1	0.98926	0.01074	1.07727	1.077343	644.6176	598.3393
TULP3	0.989261	0.010739	1.044829	1.044877	561.2618	537.1557
CFL1	0.989262	0.010738	1.372701	1.372716	20007.6	14575.18
DPP8	0.989271	0.010729	1.122973	1.123063	890.2391	792.6871
TBC1D22I	0.989272	0.010728	1.128723	1.128814	931.3612	825.0784
RMDN3	0.989276	0.010724	0.893561	0.89349	761.3156	852.0711

RNF139	0.989281	0.010719	0.982733	0.982714	519.0283	528.1581
CPNE1	0.989284	0.010716	0.90002	0.89995	724.7057	805.2747
GOLIM4	0.989285	0.010715	0.847575	0.847515	1241.445	1464.807
STMN1	0.989292	0.010708	1.332315	1.332339	10460.59	7851.291
MRGBP	0.989293	0.010707	0.876898	0.876829	889.1277	1014.028
MELK	0.989294	0.010706	1.229722	1.229785	2555.142	2077.713
TP53I13	0.989294	0.010706	1.009111	1.009121	535.6994	530.8574
DIAPH2	0.989294	0.010706	1.010048	1.010059	523.4739	518.2608
SMG1P1	0.989299	0.010701	1.025141	1.025168	536.9775	523.7943
KIF13B	0.989306	0.010694	1.095469	1.095551	723.5276	660.4226
SPATA20	0.989308	0.010692	0.947001	0.946951	576.8216	609.1364
DRG2	0.989309	0.010691	1.089421	1.0895	697.9652	640.6279
CYB5R1	0.98931	0.01069	1.218191	1.218259	2233.933	1833.707
SNRPF	0.989312	0.010688	0.858033	0.85797	1096.961	1278.557
SLC2A4R(	0.989313	0.010687	1.21171	1.21178	2125.015	1753.629
CINP	0.989316	0.010684	0.957105	0.957062	546.8135	571.3465
UEVLD	0.989319	0.010681	0.999241	0.99924	522.3625	522.7596
MAML2	0.989321	0.010679	1.188054	1.188134	1598.207	1345.139
ARHGEF1	0.989323	0.010677	1.123104	1.123194	880.2364	783.6895
FAM193B	0.989327	0.010673	1.085588	1.085665	685.7397	631.6303
RSAD1	0.98933	0.01067	0.91808	0.918014	636.8377	693.7137
BCS1L	0.989332	0.010668	1.023158	1.023183	552.3706	539.855
VRK1	0.989334	0.010666	0.877389	0.877321	893.5733	1018.526
SLC39A4	0.989337	0.010663	0.886137	0.886067	823.5545	929.4503
ATG4C	0.989339	0.010661	0.908959	0.908891	705.7451	776.4914
UBE3B	0.98934	0.01066	0.854651	0.85459	1150.309	1346.038
PHB2	0.989344	0.010656	0.813548	0.813507	2143.909	2635.392
AP2A1	0.989345	0.010655	1.285391	1.28543	5350.326	4162.282
RLIM	0.989347	0.010653	1.243347	1.243403	3138.443	2524.074
FYN	0.989348	0.010652	1.145284	1.145373	1062.508	927.6508
ZMPSTE2	0.989349	0.010651	0.824229	0.824182	1760.473	2136.026
RNF121	0.989349	0.010651	1.004678	1.004683	527.9196	525.4588
SMIM13	0.989351	0.010649	0.829811	0.829761	1617.101	1948.876
TMEM70	0.989352	0.010648	0.892224	0.892154	803.5491	900.6851
CSNK1G1	0.989355	0.010645	0.922927	0.922863	650.1746	704.5198
PCBP4	0.989358	0.010642	1.172579	1.172662	1374.814	1172.385
HUS1	0.989358	0.010642	0.948078	0.948028	557.8165	588.3969
RPP30	0.989364	0.010636	0.871099	0.871033	983.5975	1129.233
NSRP1	0.989365	0.010635	0.909316	0.909248	691.2968	760.2958
TSPAN12	0.989373	0.010627	0.913327	0.91326	674.6256	738.7016
LIG1	0.989379	0.010621	0.908412	0.908345	697.9652	768.3936
OCLN	0.989381	0.010619	1.135128	1.135217	982.4861	865.4595
HAGH	0.989383	0.010617	0.911993	0.911927	699.0766	766.5941
CCNE2	0.989385	0.010615	0.97489	0.974864	536.8108	550.6521
MAZ	0.98939	0.01061	1.279663	1.279703	5116.93	3998.526
FOXJ2	0.989393	0.010607	0.936471	0.936414	599.0498	639.7282
ITGB3BP	0.989396	0.010604	0.976289	0.976265	527.9196	540.7547
MOB3B	0.9894	0.0106	1.001537	1.001539	533.4766	532.6569
ZNF544	0.989404	0.010596	0.852959	0.852899	1192.542	1398.224
MBD2	0.989411	0.010589	1.132206	1.132294	990.2659	874.5651
HYOU1	0.989412	0.010588	0.716006	0.716001	20297.67	28348.68
SBDSP1	0.989412	0.010588	1.060338	1.060397	632.7699	596.7287
NBEAL1	0.989417	0.010583	1.056019	1.056075	622.3894	589.3417
BCL2L11	0.989418	0.010582	0.920988	0.920924	651.286	707.21
KLHL9	0.98942	0.01058	1.133174	1.133262	975.8176	861.0687
HYAL2	0.98942	0.01058	0.991815	0.991806	547.9249	552.4516

MIER1	0.989422	0.010578	0.847359	0.847302	1318.132	1555.682
PNRC2	0.989423	0.010577	0.803911	0.803876	2520.677	3135.658
DONSON	0.989427	0.010573	1.171634	1.171716	1384.816	1181.869
ACP6	0.989428	0.010572	0.980523	0.980503	539.0336	549.7523
HMOX2	0.98943	0.01057	0.890919	0.89085	828.0001	929.4503
PTRH2	0.989432	0.010568	0.949264	0.949216	590.1585	621.733
NUDT9	0.989434	0.010566	0.958247	0.958206	561.2618	585.7427
MGME1	0.989434	0.010566	0.985023	0.985008	551.2592	559.6497
FRA10AC	0.989435	0.010565	0.971982	0.971953	545.7021	561.4492
YIPF4	0.989436	0.010564	1.15468	1.154766	1180.317	1022.125
TMEM203	0.989437	0.010563	1.076064	1.076134	669.0686	621.733
GPN2	0.989439	0.010561	0.988198	0.988185	537.9222	544.3538
CHM	0.989441	0.010559	0.854125	0.854065	1185.774	1388.39
BTF3L4	0.989441	0.010559	0.799244	0.799211	2756.296	3448.774
NF2	0.989441	0.010559	1.254561	1.254611	3701.75	2950.514
FOXP4	0.989442	0.010558	1.166684	1.166768	1323.811	1134.595
YARS2	0.989443	0.010557	0.910238	0.910172	700.188	769.2934
TWF2	0.989446	0.010554	1.199311	1.199385	1859.388	1550.284
POGK	0.989447	0.010553	0.801769	0.801734	2635.152	3286.817
GALM	0.989448	0.010552	0.939884	0.939831	598.5719	636.8939
NSUN4	0.989449	0.010551	1.049801	1.049851	597.9495	569.556
QKI	0.989449	0.010551	1.256601	1.256651	3716.554	2957.506
FAM111B	0.989467	0.010533	1.127558	1.127645	943.5867	836.7752
WDR11	0.989468	0.010532	0.851388	0.85133	1234.776	1450.41
AAR2	0.989472	0.010528	0.932585	0.932527	636.8377	682.9166
SWAP70	0.989472	0.010528	1.152593	1.152678	1197.433	1038.825
PLCXD1	0.989475	0.010525	0.978028	0.978006	560.1615	572.7591
SMAD2	0.989476	0.010524	0.833337	0.833288	1620.224	1944.378
ATXN7L3	0.989478	0.010522	1.133791	1.133877	994.7116	877.2643
SORBS3	0.989479	0.010521	0.972528	0.9725	551.2592	566.8477
NR2C2AP	0.989481	0.010519	0.958896	0.958856	566.8189	591.1412
SLC7A11	0.989483	0.010517	1.167774	1.167856	1351.263	1157.044
PSMD9	0.989483	0.010517	0.889722	0.889655	826.8887	929.4503
PPP2R5C	0.989485	0.010515	1.267567	1.267611	4437.858	3500.96
GTPBP3	0.989485	0.010515	0.973999	0.973973	564.3627	579.4443
PPIP5K2	0.989486	0.010514	0.830839	0.830791	1643.775	1978.569
TTC13	0.989487	0.010513	1.077763	1.077832	700.188	649.6255
PAPD4	0.989488	0.010512	1.113323	1.113407	873.5679	784.5892
PRKCE	0.98949	0.01051	1.082469	1.082542	701.2994	647.826
ADCY9	0.989496	0.010504	1.175091	1.175171	1457.058	1239.867
ZNRF1	0.989496	0.010504	0.95635	0.956309	586.8243	613.6352
ARF5	0.989497	0.010503	0.830607	0.830559	1653.777	1991.165
CERCAM	0.989499	0.010501	1.227734	1.227795	2569.579	2092.838
LARS2	0.989501	0.010499	0.944538	0.944488	610.1639	646.0265
MAP9	0.989501	0.010499	1.008966	1.008976	550.1477	545.2535
FAM84B	0.989503	0.010497	0.796892	0.796861	2994.137	3757.418
MAP3K2	0.989506	0.010494	0.864276	0.864214	1069.176	1237.168
CDCA7L	0.989506	0.010494	0.859139	0.859078	1162.534	1353.236
KIAA0391	0.989508	0.010492	1.084766	1.084839	713.525	657.7233
CBS	0.98951	0.01049	0.767597	0.767578	5498.143	7162.976
C7orf60	0.989512	0.010488	1.013153	1.013167	567.9303	560.5494
MASTL	0.989527	0.010473	1.226207	1.226268	2585.139	2108.134
UNC119B	0.989527	0.010473	1.14256	1.142646	1083.624	948.3453
TOMM70A	0.989528	0.010472	1.246915	1.246967	3424.253	2746.062
RUBCN	0.989529	0.010471	0.867399	0.867336	1038.835	1197.731
ANKRD10	0.989531	0.010469	0.910893	0.910828	730.1961	801.6846

IGHMBP2	0.989532	0.010468	0.951751	0.951707	585.7128	615.4347
MRPS17	0.989534	0.010466	0.902537	0.902471	774.6525	858.3694
TXNDC16	0.989539	0.010461	0.889434	0.889368	840.2256	944.7462
CCDC117	0.989539	0.010461	0.847235	0.847179	1324.8	1563.78
CENPL	0.989541	0.010459	0.994455	0.994449	564.5961	567.7475
TSEN15	0.989542	0.010458	0.804469	0.804435	2565.133	3188.743
RAB11FIP	0.989543	0.010457	1.227798	1.227858	2645.177	2154.3
FBXO44	0.989545	0.010455	0.939154	0.939101	614.854	654.7271
BCL9	0.989546	0.010454	0.849006	0.84895	1297.015	1527.79
TMLHE	0.989552	0.010448	0.947575	0.947528	611.2753	645.1267
PLCG1	0.989552	0.010448	1.241605	1.241659	3219.754	2593.103
SMURF1	0.989555	0.010445	1.207657	1.207725	2147.243	1777.922
ISOC1	0.989556	0.010444	0.865084	0.865022	1072.51	1239.867
GGT7	0.989556	0.010444	1.121531	1.121615	943.5867	841.274
ANKH	0.989556	0.010444	0.922564	0.922503	667.9572	724.0715
EGLN2	0.989558	0.010442	0.837272	0.837221	1523.165	1819.311
MAGOHB	0.989558	0.010442	0.970978	0.97095	563.4847	580.3441
PIP5K1C	0.989559	0.010441	0.93623	0.936176	640.1719	683.8163
SEC22C	0.989562	0.010438	0.960788	0.960751	575.7102	599.23
ALG9	0.989565	0.010435	0.95454	0.954498	585.7128	613.6352
PICK1	0.989567	0.010433	1.022031	1.022054	570.1531	557.8501
TRAPPC4	0.989569	0.010431	1.158961	1.159044	1268.118	1094.106
VPS37B	0.989569	0.010431	0.909806	0.909741	742.4216	816.0808
RTCB	0.989571	0.010429	1.250855	1.250905	3636.532	2907.119
BTG2	0.989571	0.010429	1.045885	1.04593	607.941	581.2439
FGFR1OP	0.989577	0.010423	1.140411	1.140495	1076.834	944.1794
TBC1D2	0.989578	0.010422	1.153144	1.153227	1234.776	1070.712
AMER1	0.989579	0.010421	1.018051	1.018069	567.9303	557.8501
SPG7	0.989579	0.010421	1.058187	1.058242	637.9491	602.8381
SDC2	0.989581	0.010419	1.26661	1.266653	4480.092	3536.95
JRKL	0.989582	0.010418	0.894209	0.894143	815.7746	912.3549
UTP6	0.989582	0.010418	1.175874	1.175953	1499.292	1274.958
MRPS22	0.989582	0.010418	0.831701	0.831653	1660.446	1996.564
C11orf54	0.989583	0.010417	0.871036	0.870973	1012.494	1162.488
ERC1	0.989591	0.010409	0.863533	0.863472	1128.081	1306.449
ARHGEF1	0.989594	0.010406	1.296213	1.296246	6752.925	5209.601
MBP	0.989601	0.010399	1.09221	1.092285	781.3209	715.3079
ZBTB7A	0.989602	0.010398	1.018708	1.018727	585.7128	574.9456
RNF141	0.989604	0.010396	1.050355	1.050403	637.9491	607.3369
ANGEL1	0.989607	0.010393	1.115654	1.115736	892.4619	799.8851
STAMBP	0.989607	0.010393	0.837983	0.837932	1525.965	1821.111
LIN54	0.989608	0.010392	0.929152	0.929095	653.5199	703.3951
HADHB	0.989608	0.010392	0.805696	0.805661	2611.813	3241.829
PDGFC	0.98961	0.01039	1.132003	1.132087	1012.494	894.3598
TWISTNB	0.989613	0.010387	0.942148	0.942098	617.9437	655.9238
KIAA0232	0.989613	0.010387	0.853922	0.853865	1239.222	1451.31
UNC50	0.989614	0.010386	1.00326	1.003263	576.8216	574.9456
RNF149	0.989615	0.010385	0.882848	0.882783	907.9772	1028.541
TRMT2A	0.989616	0.010384	1.161016	1.161097	1340.36	1154.39
FNBP4	0.989617	0.010383	1.154496	1.154579	1209.214	1047.319
FAM206A	0.989618	0.010382	0.916013	0.91595	704.6337	769.2934
PHPT1	0.989618	0.010382	1.100373	1.10045	825.7773	750.3984
SNX5	0.989618	0.010382	0.820331	0.820289	1994.98	2432.047
METTL3	0.98962	0.01038	0.926097	0.926038	665.7343	718.9069
SCRN2	0.989622	0.010378	1.067051	1.067111	670.18	628.0313
LYAR	0.989625	0.010375	0.926483	0.926424	653.5088	705.4105

MPZL1	0.989627	0.010373	0.797954	0.797923	2948.57	3695.307
LRRC8B	0.989629	0.010371	0.872001	0.871939	1013.617	1162.488
TOLLIP	0.989631	0.010369	0.903273	0.903208	789.1008	873.6653
TOM1L2	0.989634	0.010366	1.16901	1.169089	1453.724	1243.466
AKAP8L	0.989635	0.010365	1.202742	1.202811	2020.543	1679.849
CAAP1	0.989636	0.010364	0.951808	0.951764	615.7209	646.9262
DCAF4	0.989637	0.010363	1.006697	1.006704	567.9303	564.1485
METTL4	0.989639	0.010361	1.019536	1.019556	577.933	566.8477
GPATCH1	0.989641	0.010359	0.91346	0.913398	737.976	807.947
TNRC18	0.989643	0.010357	0.829379	0.829334	1787.147	2154.921
TRIM11	0.989643	0.010357	1.064795	1.064853	666.8457	626.2318
REXO1	0.989645	0.010355	1.003143	1.003146	581.2672	579.4443
LSM1	0.989653	0.010347	0.987819	0.987807	581.2672	588.4419
LGALS3BI	0.989653	0.010347	1.345193	1.345212	13811.49	10267.14
RHOT2	0.989653	0.010347	0.931728	0.931672	641.2833	688.3151
RPL26L1	0.989656	0.010344	0.878157	0.878094	959.1465	1092.307
RTCA	0.989658	0.010342	1.087043	1.087115	750.2348	690.1146
SYNC	0.98966	0.01034	0.981486	0.981468	584.6014	595.64
TMEM165	0.989662	0.010338	1.172037	1.172116	1444.832	1232.669
BUD31	0.989662	0.010338	0.782838	0.782814	3999.963	5109.727
FAM49A	0.989664	0.010336	0.935548	0.935495	659.0659	704.5108
SDCCAG3	0.98967	0.01033	1.139857	1.139941	1076.956	944.7462
MARCH5	0.98967	0.01033	1.217143	1.217206	2401.756	1973.17
KDM4A	0.989672	0.010328	0.825502	0.825458	1862.722	2256.594
PHF2	0.989676	0.010324	0.951529	0.951486	622.3894	654.1243
CYTH2	0.989678	0.010322	0.936163	0.936111	659.4993	704.5108
DDX59	0.989679	0.010321	0.978605	0.978584	589.0471	601.9383
ESRP2	0.98968	0.01032	0.897414	0.89735	814.6632	907.8561
GCC2	0.98968	0.01032	1.19242	1.192492	1824.068	1529.625
LPIN3	0.98968	0.01032	1.040801	1.04084	614.6095	590.4934
OTUD3	0.989685	0.010315	0.977331	0.977309	590.6364	604.3497
PCID2	0.989685	0.010315	0.844601	0.844548	1455.947	1723.937
AACS	0.989688	0.010312	0.894104	0.89404	859.1196	960.9419
CHMP1A	0.989691	0.010309	1.187031	1.187104	1727.13	1454.909
AAAS	0.989693	0.010307	0.908724	0.908661	739.0874	813.3815
TERF2	0.989693	0.010307	1.001077	1.001078	587.68	587.0473
RABL3	0.989695	0.010305	1.078178	1.078244	721.1492	668.8173
ZNF574	0.989697	0.010303	1.072462	1.072525	717.9706	669.4202
NOA1	0.989698	0.010302	0.999141	0.99914	587.9357	588.4419
ZDHHC8	0.989698	0.010302	0.980272	0.980253	576.8216	588.4419
ZNF248	0.9897	0.0103	1.006406	1.006413	576.8216	573.146
UBE2G1	0.989702	0.010298	1.17701	1.177086	1561.497	1326.577
TOMM34	0.989703	0.010297	1.19133	1.191402	1812.709	1521.491
LDOC1L	0.989703	0.010297	0.913295	0.913233	750.2015	821.4793
AIG1	0.989704	0.010296	1.116221	1.116302	918.0243	822.3791
PSMD10	0.989705	0.010295	1.182295	1.182369	1690.454	1429.716
HSPA14	0.989706	0.010294	0.886803	0.886739	895.9851	1010.429
ZNF518B	0.989707	0.010293	0.993187	0.99318	574.5988	578.5446
NAPRT	0.989709	0.010291	0.834337	0.83429	1688.231	2023.556
RAB35	0.98971	0.01029	1.112437	1.112516	916.9129	824.1786
MED20	0.989712	0.010288	1.038083	1.038119	614.6095	592.041
RPS19BP1	0.989712	0.010288	0.85768	0.857623	1191.431	1389.227
KIAA1109	0.989713	0.010287	0.84483	0.844778	1425.938	1687.947
ARFGAP2	0.989713	0.010287	0.908589	0.908527	775.7639	853.8706
B4GALT2	0.989716	0.010284	1.106183	1.106261	858.0082	775.5917
NEDD1	0.989718	0.010282	0.901881	0.901818	814.6632	903.3573

BAX	0.989721	0.010279	1.180004	1.180078	1617.101	1370.332
PDZD8	0.989724	0.010276	0.838863	0.838814	1522.831	1815.46
OPA3	0.989727	0.010273	1.071529	1.071591	720.2379	672.1195
DEDD	0.98973	0.01027	0.911232	0.911171	746.8672	819.6798
DOT1L	0.989735	0.010265	0.88806	0.887996	892.4619	1005.03
TMSB4X	0.989736	0.010264	1.405296	1.405304	37092.18	26394.41
PSENEN	0.989736	0.010264	1.166832	1.16691	1423.716	1220.072
B2M	0.989738	0.010262	1.357983	1.357999	17186.84	12656
UBP1	0.989746	0.010254	0.817355	0.817316	2158.357	2640.791
GINM1	0.989747	0.010253	1.055608	1.055659	646.8404	612.7354
KLHL24	0.989749	0.010251	1.136097	1.136178	1083.624	953.7438
N4BP1	0.989751	0.010249	0.846477	0.846425	1408.156	1663.653
ZFPL1	0.989758	0.010242	0.849537	0.849484	1355.92	1596.171
PRPF39	0.989768	0.010232	1.010287	1.010297	589.0471	583.0434
GATAD2B	0.989768	0.010232	0.844733	0.844681	1447.055	1713.14
FAM92A1	0.989772	0.010228	0.951528	0.951486	622.3894	654.1243
TMEM97	0.989772	0.010228	0.915347	0.915287	753.5357	823.2788
FMNL3	0.989777	0.010223	1.085674	1.085743	766.8726	706.3103
PISD	0.989778	0.010222	0.900421	0.900358	815.7746	906.0566
LHFPL2	0.98978	0.01022	0.998417	0.998415	585.7128	586.6424
SMIM20	0.989781	0.010219	1.001845	1.001847	586.8243	585.7427
MPDZ	0.989782	0.010218	1.181311	1.181384	1633.772	1382.929
RNF115	0.989783	0.010217	0.889753	0.88969	890.1613	1000.531
LETMD1	0.989786	0.010214	1.081559	1.081626	751.3129	694.6134
CNPPD1	0.989787	0.010213	1.156474	1.156553	1307.018	1130.096
COX16	0.989787	0.010213	1.077729	1.077794	754.4693	700.012
GCN1	0.989788	0.010212	1.260631	1.260675	4240.028	3363.297
VEZT	0.989789	0.010211	0.882078	0.882016	954.7008	1082.409
ZHX1	0.98979	0.01021	0.904397	0.904336	814.252	900.3881
DDAH2	0.989792	0.010208	0.912657	0.912597	770.2068	843.9733
ARHGDIA	0.989798	0.010202	1.274786	1.274825	5260.302	4126.292
SH3GLB2	0.989799	0.010201	0.838641	0.838593	1583.759	1888.593
FBXW11	0.9898	0.0102	1.273194	1.273233	5026.906	3948.139
COG7	0.989802	0.010198	0.924291	0.924234	703.5223	761.1955
CCHCR1	0.989803	0.010197	1.039387	1.039424	631.2806	607.3369
SF3B5	0.989805	0.010195	0.858941	0.858885	1222.551	1423.418
IGF1R	0.989808	0.010192	0.919589	0.919531	741.3102	806.1834
UFL1	0.989809	0.010191	0.94264	0.942592	647.9518	687.4153
VAMP4	0.98981	0.01019	1.039965	1.040002	633.5035	609.1364
WDR55	0.989812	0.010188	1.170095	1.170171	1497.058	1279.348
HSPBP1	0.989813	0.010187	0.85012	0.850067	1398.153	1644.758
CHID1	0.989814	0.010186	1.175785	1.17586	1584.87	1347.838
CRYBG3	0.989814	0.010186	1.039126	1.039162	647.9518	623.5325
ISY1	0.989824	0.010176	1.043255	1.043295	642.3058	615.6506
ELOF1	0.989825	0.010175	1.17944	1.179513	1649.332	1398.314
COG5	0.989825	0.010175	1.288059	1.288093	6201.665	4814.607
MGAT5	0.989826	0.010174	0.820565	0.820525	2051.662	2500.428
RAD51	0.989828	0.010172	1.027478	1.027504	615.7209	599.239
PREX1	0.989832	0.010168	1.027789	1.027816	616.8323	600.1388
GPAA1	0.989838	0.010162	0.771122	0.771103	5256.967	6817.469
C4orf27	0.989844	0.010156	0.932161	0.932108	683.5169	733.303
SLC35F5	0.989847	0.010153	0.830157	0.830113	1810.486	2181.014
DOCK1	0.989848	0.010152	1.081146	1.081211	780.2095	721.6062
DTYMK	0.989849	0.010151	0.934532	0.93448	676.8484	724.3054
SFN	0.989849	0.010151	1.127687	1.127766	1038.057	920.4527
TSPAN6	0.989849	0.010151	1.144615	1.144694	1223.662	1068.985

PMM2	0.989853	0.010147	1.103104	1.103179	891.3505	807.983
ZNF862	0.989861	0.010139	0.965695	0.965665	629.0578	651.425
MED4	0.989861	0.010139	0.947709	0.947665	644.6176	680.2173
BFAR	0.989863	0.010137	0.821336	0.821296	2081.67	2534.619
GPKOW	0.989864	0.010136	0.922572	0.922515	721.3048	781.89
ATP1B3	0.989867	0.010133	1.306273	1.306301	8109.956	6208.332
MAGOH	0.98987	0.01013	0.882514	0.882454	970.2606	1099.505
PPTC7	0.989873	0.010127	0.921666	0.92161	744.6444	807.983
ING1	0.989875	0.010125	0.935276	0.935226	679.0713	726.105
LMBR1	0.989875	0.010125	0.846709	0.846657	1421.493	1678.949
AP1AR	0.989878	0.010122	0.99705	0.997048	600.1612	601.9383
PPA2	0.989881	0.010119	1.176742	1.176815	1627.104	1382.632
TRIM26	0.989893	0.010107	0.861559	0.861503	1242.556	1442.313
ZMIZ1	0.989895	0.010105	1.1424	1.142478	1163.646	1018.526
CEP89	0.989903	0.010097	1.080956	1.081021	771.3182	713.5083
ZNF318	0.989907	0.010093	0.866476	0.866419	1151.42	1328.943
INPP5F	0.98991	0.01009	0.937328	0.937279	695.7424	742.3006
EIF2B4	0.98991	0.01009	0.903621	0.903561	823.5545	911.4552
RIPK1	0.989914	0.010086	0.907202	0.907143	823.5545	907.8561
POLR2K	0.989915	0.010085	1.201272	1.201337	2127.238	1770.724
RNF123	0.989917	0.010083	0.953263	0.953223	642.3947	673.919
INPP4A	0.989922	0.010078	1.161041	1.161116	1410.379	1214.674
UBE2T	0.989923	0.010077	1.170055	1.170129	1538.191	1314.547
GOLGA8A	0.989924	0.010076	0.959083	0.959048	633.359	660.4046
NAA20	0.989928	0.010072	1.170101	1.170174	1579.313	1349.637
PIN4	0.98993	0.01007	0.935147	0.935097	689.0739	736.9021
CDC42EP	0.989937	0.010063	0.903241	0.903181	831.3344	920.4527
PLBD2	0.989937	0.010063	1.01659	1.016606	620.1665	610.0361
OXR1	0.989939	0.010061	1.233863	1.233916	3129.729	2536.419
PCBP2	0.98994	0.01006	0.743636	0.743626	10835.13	14570.69
ZNF480	0.989942	0.010058	0.909099	0.90904	799.1035	879.0639
CSTF2	0.989943	0.010057	0.897061	0.897001	872.4565	972.6387
CEP63	0.989943	0.010057	0.911655	0.911597	785.7666	861.9685
KLHDC2	0.989944	0.010056	0.924415	0.924361	746.8672	807.983
SSH3	0.989944	0.010056	0.988196	0.988185	600.1612	607.3369
KRT19	0.989945	0.010055	0.774199	0.774179	5130.267	6626.72
CTTNBP2	0.989945	0.010055	0.948983	0.948941	655.7317	691.0144
PACSIN3	0.98995	0.01005	0.908577	0.908518	803.5491	884.4624
DIRC2	0.989951	0.010049	1.067987	1.068044	731.3075	684.7161
SEC63	0.989951	0.010049	0.798852	0.798823	3130.863	3919.347
IMPAD1	0.989953	0.010047	1.273743	1.273781	5251.41	4122.693
KLF11	0.989954	0.010046	0.958549	0.958513	639.0605	666.7209
SMAD4	0.989954	0.010046	1.033657	1.033688	661.2887	639.7372
KIAA0141	0.989955	0.010045	1.145993	1.146069	1271.453	1109.402
MED1	0.989956	0.010044	0.834734	0.834689	1739.356	2083.84
EPHB4	0.989961	0.010039	1.271661	1.271699	5115.818	4022.819
EIF3D	0.989961	0.010039	1.292759	1.292791	6839.615	5290.579
NHP2	0.989964	0.010036	1.24873	1.248776	3791.018	3035.785
PHF10	0.989965	0.010035	1.029927	1.029954	641.2833	622.6327
MIIP	0.989967	0.010033	0.901414	0.901354	849.1169	942.0469
ARID1A	0.989969	0.010031	0.82721	0.827169	1998.314	2415.851
SLC35C2	0.989976	0.010024	0.871525	0.871467	1110.298	1274.058
SPECC1	0.989977	0.010023	1.148694	1.14877	1309.24	1139.688
PDXP	0.989977	0.010023	1.07793	1.077991	792.435	735.1025
DPY30	0.989978	0.010022	0.870163	0.870106	1154.755	1327.143
FAM134C	0.98998	0.01002	0.95461	0.954572	650.1746	681.117

MEMO1	0.989982	0.010018	0.87547	0.875412	1068.065	1220.072
RAB32	0.989985	0.010015	0.831987	0.831943	1822.712	2190.911
BCL7C	0.989987	0.010013	0.920372	0.920316	753.5357	818.7801
DHTKD1	0.989987	0.010013	0.823639	0.823599	2069.389	2512.62
RPS6KC1	0.989989	0.010011	1.04203	1.042067	683.5169	655.9238
CERS5	0.989991	0.010009	0.951591	0.951551	657.9545	691.4553
SART1	0.989998	0.010002	0.845816	0.845767	1547.082	1829.209
SCAMP3	0.990001	0.009999	0.826857	0.826816	2024.988	2449.142
LSG1	0.990001	0.009999	0.857711	0.857658	1279.244	1491.556
RXRA	0.990004	0.009996	0.967542	0.967514	634.6149	655.9238
NRBF2	0.990006	0.009994	1.168434	1.168507	1518.185	1299.251
B4GALT3	0.990006	0.009994	1.192082	1.192148	1968.306	1651.056
ALMS1	0.990007	0.009993	0.948158	0.948116	667.9572	704.5108
NFRKB	0.990007	0.009993	0.853877	0.853825	1365.922	1599.77
FAM195B	0.990009	0.009991	1.058247	1.058296	711.3021	672.1195
PPP1CA	0.990012	0.009988	0.809752	0.809719	2679.608	3309.311
TTC21B	0.990018	0.009982	1.052002	1.052047	695.7424	661.3224
MRTO4	0.990022	0.009978	0.845334	0.845285	1565.976	1852.602
MYO1C	0.990022	0.009978	1.277092	1.277128	5723.759	4481.741
SDE2	0.990028	0.009972	0.995386	0.995382	623.5008	626.3937
ZPR1	0.99003	0.00997	0.801766	0.801737	3099.721	3866.261
DNTTIP1	0.990033	0.009967	0.945343	0.9453	695.7424	736.0023
POM121	0.990035	0.009965	1.111007	1.11108	961.1359	865.0456
KIF4A	0.990036	0.009964	1.213105	1.213163	2503.95	2063.983
IER5L	0.990036	0.009964	1.023013	1.023033	659.0659	644.2269
SCAMP2	0.990039	0.009961	0.932834	0.932784	733.5303	786.3888
PSMF1	0.990039	0.009961	0.823673	0.823634	2095.007	2543.617
MTMR14	0.990044	0.009956	0.94664	0.946598	677.9598	716.2076
SHQ1	0.990045	0.009955	1.068419	1.068474	769.0954	719.8066
RPUSD1	0.990045	0.009955	1.096859	1.096928	899.1304	819.6798
EMD	0.990048	0.009952	0.825476	0.825435	2041.659	2473.436
NCAPG	0.990049	0.009951	1.14228	1.142355	1230.33	1077.011
DCUN1D4	0.990054	0.009946	0.939962	0.939916	696.8538	741.4008
VTI1A	0.990057	0.009943	1.016118	1.016133	654.6202	644.2269
FAN1	0.990061	0.009939	0.956656	0.95662	660.1773	690.1146
TFCP2	0.990064	0.009936	0.880656	0.880598	1062.508	1206.576
FKBP9	0.990064	0.009936	1.200394	1.200456	2185.442	1820.508
MED10	0.990064	0.009936	1.119868	1.119942	1058.062	944.7462
FKBP3	0.990065	0.009935	0.831985	0.831942	1859.388	2235
DCUN1D1	0.990067	0.009933	1.140137	1.140212	1213.659	1064.414
INTS4	0.990067	0.009933	0.995999	0.995995	629.8803	632.4131
RSPRY1	0.990068	0.009932	1.137892	1.137968	1170.314	1028.424
RECQL4	0.990071	0.009929	1.188525	1.188592	1926.073	1620.465
NBR1	0.990072	0.009928	1.209725	1.209784	2430.653	2009.16
SLBP	0.990073	0.009927	1.16154	1.161613	1508.183	1298.351
SPRED2	0.990075	0.009925	1.056442	1.056489	735.7531	696.4129
DGKQ	0.990076	0.009924	0.983369	0.983355	633.5035	644.2269
AKAP8	0.990078	0.009922	0.917294	0.917239	804.6605	877.2643
PRKRIP1	0.990082	0.009918	0.947992	0.947951	681.3941	718.8079
IKBKG	0.990082	0.009918	0.961492	0.96146	655.7317	682.0168
PTPN13	0.990084	0.009916	0.897007	0.896949	904.6874	1008.629
CEP68	0.990089	0.009911	1.09515	1.095217	878.0691	801.7296
CEP350	0.99009	0.00991	0.822539	0.8225	2159.469	2625.495
AEBP2	0.990095	0.009905	0.903039	0.902982	866.8995	960.0421
JUND	0.990095	0.009905	0.875917	0.875861	1121.412	1280.356
TMEM59	0.990101	0.009899	1.244283	1.24433	3796.553	3051.08

SMAP2	0.990102	0.009898	0.875697	0.875641	1125.858	1285.755
USF1	0.990105	0.009895	1.082053	1.082114	816.886	754.8972
FADS2	0.990106	0.009894	0.942171	0.942126	700.188	743.2004
PRAF2	0.990109	0.009891	0.949252	0.949212	671.2914	707.21
SMS	0.990111	0.009889	1.283499	1.283532	6231.674	4855.096
RASSF3	0.990112	0.009888	1.176553	1.176622	1762.696	1498.098
AHDC1	0.990115	0.009885	0.941727	0.941683	720.1934	764.7946
DCK	0.99012	0.00988	1.008735	1.008743	643.5061	637.9286
ZCCHC14	0.990122	0.009878	1.082195	1.082256	841.3371	777.3912
HIBCH	0.990125	0.009875	0.899014	0.898956	900.2418	1001.431
SLC9A3R1	0.990127	0.009873	0.863013	0.862961	1295.904	1501.697
TRMT61A	0.990128	0.009872	1.0014	1.001401	656.8431	655.9238
TCF12	0.990133	0.009867	0.878938	0.878881	1099.184	1250.664
SIMC1	0.990136	0.009864	1.087085	1.087149	847.0386	779.1367
HMG20A	0.990139	0.009861	1.077038	1.077097	802.4377	744.9999
SPTBN2	0.990143	0.009857	0.95516	0.955124	693.5196	726.105
ZDHHC13	0.990143	0.009857	1.057348	1.057396	733.5303	693.7137
ARCN1	0.990144	0.009856	1.335019	1.335039	13250.23	9924.973
JMY	0.990145	0.009855	1.149509	1.149582	1342.583	1167.886
CST3	0.990146	0.009854	1.087507	1.08757	851.3397	782.7897
RAP2B	0.99015	0.00985	1.139981	1.140055	1238.11	1086.008
GPN1	0.990156	0.009844	1.191447	1.191511	2029.434	1703.242
BRPF1	0.990157	0.009843	0.95207	0.952033	702.4109	737.8018
HMCES	0.990157	0.009843	1.148064	1.148136	1362.588	1186.781
GSK3B	0.990158	0.009842	1.237181	1.23723	3438.701	2779.353
RPS6KA4	0.99016	0.00984	1.112239	1.112309	1025.831	922.2523
KDSR	0.990164	0.009836	0.991808	0.991801	634.6704	639.9171
CCDC43	0.990165	0.009835	0.978189	0.97817	651.286	665.8211
MSRB2	0.990166	0.009834	1.008345	1.008352	666.8457	661.3224
RBM33	0.990167	0.009833	1.165802	1.165872	1569.31	1346.038
TRAPPC1	0.990171	0.009829	1.145109	1.145181	1300.349	1135.495
TNS3	0.990172	0.009828	0.802091	0.802062	3189.745	3976.932
MCRS1	0.990173	0.009827	1.116547	1.116619	1035.834	927.6508
TPRN	0.990178	0.009822	1.101976	1.102044	938.0297	851.1714
UQCRC1	0.990179	0.009821	0.810465	0.810433	2690.723	3320.108
GPRC5C	0.990179	0.009821	0.909473	0.909418	846.8941	931.2498
PIK3R2	0.990183	0.009817	1.140161	1.140234	1281.455	1123.852
ZNF394	0.990187	0.009813	0.998082	0.998081	650.1746	651.425
EPAS1	0.990188	0.009812	1.308759	1.308784	9294.718	7101.792
CTDSPL2	0.990189	0.009811	0.88646	0.886403	996.9344	1124.698
TMEM189	0.990189	0.009811	1.152145	1.152216	1424.449	1236.268
ZNF3	0.990189	0.009811	0.9251	0.925049	792.435	856.6419
LIG3	0.990191	0.009809	1.018836	1.018853	664.6229	652.3248
SCCPDH	0.990193	0.009807	1.129919	1.129991	1181.428	1045.519
CARS	0.990197	0.009803	1.212183	1.212239	2578.47	2127.029
MFSD14A	0.990197	0.009803	1.049258	1.049299	721.3048	687.4153
PFDN6	0.990197	0.009803	0.852269	0.85222	1461.504	1714.939
CIR1	0.990201	0.009799	0.985516	0.985504	653.5088	663.1219
BUD13	0.990202	0.009798	1.053018	1.053062	732.4189	695.5132
AGPAT5	0.990203	0.009797	0.908652	0.908597	878.0136	966.3404
POLR2D	0.990207	0.009793	0.957145	0.957111	684.6283	715.3079
TRIM41	0.990209	0.009791	1.129962	1.130035	1158.089	1024.825
SNHG8	0.990212	0.009788	1.123368	1.123439	1128.081	1004.13
ZNF618	0.990216	0.009784	0.924549	0.924498	800.2149	865.5675
RAP1A	0.990218	0.009782	1.159208	1.159279	1493.679	1288.454
TSPAN31	0.990218	0.009782	0.999143	0.999142	655.7205	656.2837

RALGAPA	0.990221	0.009779	1.14569	1.145762	1323.689	1155.29
KDELC2	0.990221	0.009779	0.964647	0.964618	675.0702	699.832
ZNF646	0.990226	0.009774	0.9471	0.94706	726.8619	767.4938
RC3H2	0.990228	0.009772	0.899592	0.899536	920.2471	1023.025
HERC2	0.990232	0.009768	0.869061	0.869008	1245.701	1433.477
TSR3	0.990232	0.009768	0.924188	0.924137	785.7666	850.2716
CDC25A	0.990232	0.009768	1.05603	1.056075	746.8672	707.21
NUDCD3	0.990233	0.009767	1.228569	1.22862	3098.61	2522.023
CREBRF	0.99024	0.00976	0.989863	0.989854	659.0659	665.8211
SGTA	0.990241	0.009759	1.156243	1.156312	1498.18	1295.652
TLK1	0.990244	0.009756	1.150415	1.150486	1388.151	1206.576
LINC0142	0.99025	0.00975	1.073226	1.073281	810.2176	754.8972
MAPK8	0.990251	0.009749	1.122447	1.122518	1084.736	966.3404
FAM117A	0.990253	0.009747	1.069384	1.069437	795.7693	744.1001
CTSH	0.990254	0.009746	1.085527	1.085588	886.9048	816.9805
LARP1B	0.990257	0.009743	1.126555	1.126626	1141.418	1013.128
ZNF621	0.990257	0.009743	1.073753	1.073808	833.8128	776.5004
CCSAP	0.990258	0.009742	1.054571	1.054614	765.7612	726.105
LZTS2	0.990259	0.009741	0.903152	0.903097	903.576	1000.531
CLIP1	0.990269	0.009731	0.827172	0.827133	2101.676	2540.917
SMU1	0.990274	0.009726	1.196263	1.196323	2256.161	1885.911
DSN1	0.990275	0.009725	0.883283	0.883228	1068.065	1209.275
FBXO21	0.990277	0.009723	1.09344	1.093504	934.6955	854.7704
PLEKHJ1	0.990279	0.009721	0.929401	0.929353	793.5464	853.8706
GOLM1	0.990286	0.009714	1.129648	1.129718	1178.094	1042.82
ST5	0.990286	0.009714	0.945964	0.945924	741.3102	783.6895
MTCL1	0.990287	0.009713	1.045526	1.045563	729.0847	697.3127
DHX33	0.990287	0.009713	0.929807	0.929759	774.6525	833.1762
TTC9C	0.99029	0.00971	1.002167	1.002169	669.0686	667.6207
LINC0110	0.99029	0.00971	1.061149	1.061197	774.408	729.749
LRCH4	0.990291	0.009709	1.174403	1.174469	1761.584	1499.897
BRSK1	0.990295	0.009705	0.925839	0.92579	811.329	876.3646
COMMD3-	0.990302	0.009698	0.972271	0.972248	680.1827	699.5981
EIF2AK1	0.990305	0.009695	1.252291	1.252333	4313.381	3444.275
MOSPD1	0.990309	0.009691	0.854461	0.854412	1463.726	1713.14
FBXO27	0.990313	0.009687	0.875593	0.87554	1189.475	1358.563
SIAH2	0.990315	0.009685	1.159843	1.159911	1540.414	1328.043
CTNNBIP1	0.990317	0.009683	0.905076	0.905022	904.6874	999.6315
TRMT6	0.990317	0.009683	1.072989	1.073043	823.5545	767.4938
BLOC1S1	0.990325	0.009675	0.932852	0.932806	757.459	812.0229
FAM210A	0.990326	0.009674	0.991065	0.991058	690.1853	696.4129
MSANTD4	0.990329	0.009671	0.902142	0.902088	926.9156	1027.524
WDTC1	0.99033	0.00967	1.192242	1.192302	2191.7	1838.206
NPAT	0.990331	0.009669	0.958584	0.958552	704.6337	735.1025
RHOD	0.990332	0.009668	1.107757	1.107824	1014.717	915.954
TJAP1	0.990332	0.009668	1.052472	1.052513	774.6525	736.0023
ZBTB11	0.990333	0.009667	0.919093	0.919042	830.223	903.3573
ERBB2	0.990336	0.009664	0.861332	0.861282	1359.254	1578.176
SPAG1	0.990336	0.009664	1.022956	1.022975	712.4135	696.4129
EEF1D	0.990341	0.009659	1.323915	1.323935	11760.94	8883.314
DDX54	0.990343	0.009657	0.862088	0.862038	1350.363	1566.479
DYNC1I2	0.990344	0.009656	1.222579	1.222631	3019.7	2469.837
ARID4B	0.99035	0.00965	0.830627	0.830587	2037.214	2452.741
MXI1	0.990352	0.009648	1.065263	1.065312	801.3263	752.1979
SDHAF2	0.990353	0.009647	1.058292	1.058337	777.9867	735.1025
THYN1	0.990354	0.009646	1.035316	1.035345	736.8646	711.7088

MUS81	0.990354	0.009646	0.884048	0.883995	1082.513	1224.571
LEMD3	0.990358	0.009642	0.920905	0.920855	826.8887	897.9588
BTAF1	0.990358	0.009642	0.862225	0.862176	1387.039	1608.768
TTLL12	0.990365	0.009635	1.201637	1.201695	2389.531	1988.466
POLR2H	0.990367	0.009633	1.115117	1.115185	1104.741	990.6339
LUC7L3	0.990372	0.009628	0.862449	0.862399	1354.808	1570.978
KMT5B	0.990373	0.009627	1.148704	1.148773	1388.151	1208.375
TELO2	0.990373	0.009627	1.007598	1.007605	703.5223	698.2124
MZT2B	0.990373	0.009627	1.093619	1.093681	936.3626	856.156
AKR7A2	0.990374	0.009626	0.964149	0.964121	703.5223	729.704
HOOK3	0.990374	0.009626	1.056159	1.056202	794.5689	752.2879
UBAC2	0.990376	0.009624	0.898311	0.898257	965.8149	1075.211
GGA1	0.990378	0.009622	0.876642	0.87659	1168.091	1332.542
RASA4	0.990379	0.009621	0.893958	0.893905	1025.198	1146.877
RPRD1B	0.990381	0.009619	0.855064	0.855016	1517.074	1774.323
PLEKHA6	0.990383	0.009617	1.072759	1.072812	836.8914	780.0905
SGOL1	0.990386	0.009614	0.978346	0.978328	706.8565	722.5149
TENM3	0.990387	0.009613	1.051696	1.051736	783.5438	744.9999
PLEKHA5	0.990387	0.009613	1.000181	1.000181	685.7397	685.6158
HPS5	0.990389	0.009611	0.859935	0.859886	1400.376	1628.563
MOV10	0.99039	0.00961	1.065811	1.065861	811.329	761.1955
DERL2	0.990391	0.009609	0.95745	0.957418	717.9706	749.9036
NIP7	0.990392	0.009608	0.964815	0.964788	705.7451	731.5035
MRPL16	0.990392	0.009608	0.91834	0.91829	846.8941	922.2523
ANKRD40	0.990395	0.009605	0.903384	0.903331	934.6955	1034.722
POGZ	0.990406	0.009594	0.850544	0.850498	1574.867	1851.703
MFSD1	0.990406	0.009594	0.879852	0.8798	1169.203	1328.943
NCK2	0.990407	0.009593	1.112909	1.112976	1072.51	963.6411
MBD1	0.990415	0.009585	1.001071	1.001072	707.9679	707.21
EIF4ENIF1	0.990418	0.009582	0.968385	0.968361	705.7451	728.8042
EPOR	0.990418	0.009582	1.090538	1.090599	930.2498	852.9709
RSRC1	0.990419	0.009581	1.081619	1.081676	884.682	817.8803
WDFY3	0.990423	0.009577	0.917486	0.917436	879.125	958.2426
GSTT2	0.990425	0.009575	1.042639	1.042673	748.9567	718.304
USP3	0.990427	0.009573	0.99812	0.998118	692.4082	693.7137
B4GALNT	0.990427	0.009573	1.055952	1.055995	785.7666	744.1001
CCDC82	0.990427	0.009573	0.906766	0.906713	920.2471	1014.927
KNSTRN	0.990428	0.009572	0.950764	0.950728	739.0874	777.3912
PIK3CA	0.990428	0.009572	1.052918	1.052959	776.8753	737.8018
MANBAL	0.990429	0.009571	1.143078	1.143146	1365.922	1194.879
MARK2	0.990433	0.009567	0.874421	0.874369	1241.445	1419.819
NUMBL	0.990435	0.009565	1.129314	1.129382	1221.439	1081.509
TBC1D9	0.990435	0.009565	0.962056	0.962028	735.7531	764.7946
EXOSC5	0.990436	0.009564	0.932665	0.93262	794.6578	852.0711
DPF2	0.990436	0.009564	1.12949	1.129558	1223.662	1083.309
NAV2	0.990436	0.009564	1.091772	1.091833	964.7035	883.5627
BCKDHA	0.990437	0.009563	1.093086	1.093147	949.1438	868.2668
IMP4	0.990439	0.009561	1.009325	1.009333	700.188	693.7137
ROMO1	0.990452	0.009548	1.13845	1.138518	1321.466	1160.688
ZNF845	0.990453	0.009547	0.935324	0.93528	788.7452	843.3255
JARID2	0.99046	0.00954	1.161454	1.16152	1663.78	1432.415
TIMMDC1	0.990463	0.009537	0.819042	0.819009	2506.229	3060.078
ITPKC	0.990469	0.009531	1.047946	1.047983	771.3182	736.0023
ITGB4	0.990469	0.009531	1.125062	1.125129	1192.542	1059.915
BIN1	0.990469	0.009531	0.864641	0.864592	1355.92	1568.279
TMC4	0.990477	0.009523	0.865268	0.865219	1382.594	1597.971

ORC4	0.990481	0.009519	0.847554	0.84751	1667.803	1967.888
TMEM120	0.990483	0.009517	0.929894	0.929849	835.191	898.2017
MEST	0.990483	0.009517	0.825126	0.82509	2289.504	2774.855
UBL7	0.990486	0.009514	1.012215	1.012225	711.3021	702.7112
GTPBP4	0.990489	0.009511	0.813151	0.81312	2847.431	3501.859
PPT1	0.99049	0.00951	1.240832	1.240876	3908.827	3150.054
PWWP2B	0.99049	0.00951	1.0125	1.01251	712.4135	703.611
CDC14B	0.990495	0.009505	1.115498	1.115564	1118.045	1002.223
FZD6	0.990496	0.009504	0.83341	0.833371	2030.545	2436.545
ARHGEF1	0.990499	0.009501	0.916363	0.916314	881.3478	961.8416
TTC7B	0.9905	0.0095	0.975442	0.975423	713.525	731.5035
RNF213	0.990501	0.009499	0.784086	0.784066	4774.616	6089.564
ANKS1A	0.990504	0.009496	1.158767	1.158832	1601.541	1382.029
FBXL5	0.990508	0.009492	1.076187	1.07624	880.2364	817.8803
MPV17L2	0.99051	0.00949	1.084854	1.084911	922.47	850.2716
UBE2O	0.99051	0.00949	1.140289	1.140355	1362.588	1194.879
CTIF	0.990513	0.009487	0.828418	0.828382	2195.034	2649.788
FAM49B	0.990516	0.009484	1.257589	1.257627	4867.974	3870.76
MMGT1	0.990518	0.009482	0.857776	0.857729	1484.843	1731.135
SDAD1	0.990519	0.009481	1.069649	1.069698	854.674	798.9854
NFYA	0.990522	0.009478	1.082091	1.082146	911.3559	842.1738
TMEM87A	0.990526	0.009474	0.990309	0.990302	727.9733	735.1025
METAP2	0.99053	0.00947	0.840483	0.840441	1854.943	2207.107
SKA3	0.990532	0.009468	0.996696	0.996693	729.0847	731.5035
VGLL4	0.990533	0.009467	0.857159	0.857113	1501.514	1751.829
NDUFA8	0.990533	0.009467	0.884346	0.884294	1131.415	1279.456
DPM2	0.990535	0.009465	1.00567	1.005675	715.7478	711.7088
CLASRP	0.990536	0.009464	0.936939	0.936898	820.2203	875.4648
THOC5	0.990539	0.009461	0.895407	0.895355	1056.951	1180.483
TMEM168	0.990541	0.009459	1.09536	1.09542	1013.606	925.3114
POLRMT	0.990541	0.009459	1.040324	1.040356	753.5357	724.3054
PLD3	0.990541	0.009459	1.272283	1.272316	5948.264	4675.144
LAMTOR3	0.990542	0.009458	1.08043	1.080484	908.0216	840.3833
MPLKIP	0.990544	0.009456	0.964323	0.964297	735.7531	762.995
CDK12	0.990545	0.009455	1.121435	1.1215	1183.651	1055.416
C3	0.990547	0.009453	1.418729	1.418735	56359.58	39725.23
KLF16	0.990548	0.009452	1.004983	1.004987	717.9706	714.4081
FYCO1	0.990551	0.009449	0.915962	0.915913	895.7961	978.0373
ASNA1	0.990553	0.009447	0.842474	0.842432	1861.611	2209.806
FAM91A1	0.990553	0.009447	0.877168	0.877117	1216.738	1387.202
POLR3H	0.990554	0.009446	0.927081	0.927036	842.4485	908.7559
CDK11B	0.990554	0.009446	0.97229	0.972269	727.262	748.0051
PHTF2	0.990557	0.009443	1.07828	1.078333	901.3532	835.8755
WFS1	0.990558	0.009442	1.104124	1.104186	1076.956	975.338
ABI2	0.990559	0.009441	0.830767	0.83073	2144.854	2581.892
ATXN3	0.990566	0.009434	1.045843	1.045878	785.7666	751.2982
CTPS2	0.99057	0.00943	0.904918	0.904868	968.0377	1069.813
KRT10	0.990576	0.009424	0.956703	0.956672	755.7585	789.9878
C1orf27	0.990577	0.009423	0.865127	0.865079	1387.039	1603.369
ABHD5	0.990577	0.009423	0.983959	0.983947	722.4162	734.2028
ABHD10	0.990578	0.009422	1.127838	1.127904	1284.789	1139.094
SCFD1	0.990582	0.009418	0.843161	0.84312	1813.82	2151.322
RAP2C	0.990582	0.009418	1.116887	1.116951	1155.866	1034.839
PAGR1	0.990583	0.009417	0.909318	0.909268	942.4753	1036.522
MRPL13	0.990584	0.009416	1.044821	1.044855	786.878	753.0977
SLC5A6	0.990585	0.009415	1.011894	1.011904	730.1961	721.6062

ABCB7	0.99059	0.00941	1.108449	1.108512	1118.078	1008.629
LATS2	0.990591	0.009409	0.964601	0.964575	744.6444	771.9926
PRPF6	0.990599	0.009401	1.229731	1.229777	3508.72	2853.134
SPSB1	0.990599	0.009401	0.958779	0.958749	774.6525	807.983
UCHL3	0.990599	0.009401	1.131421	1.131486	1297.015	1146.292
BPNT1	0.9906	0.0094	1.006843	1.006848	729.0847	724.1255
KLHL23	0.9906	0.0094	0.9676	0.967576	760.0374	785.507
RITA1	0.990601	0.009399	0.882117	0.882066	1154.755	1309.148
TRMT1	0.990604	0.009396	1.147852	1.147916	1490.4	1298.351
ABHD16A	0.990604	0.009396	0.939069	0.939029	828.0001	881.7631
PDZD11	0.990605	0.009395	0.874367	0.874318	1268.118	1450.41
SNRPB	0.990605	0.009395	1.253721	1.253759	4737.939	3778.985
ECHDC1	0.990606	0.009394	1.058726	1.058769	833.5572	787.2885
SDC3	0.990607	0.009393	0.799451	0.799426	3643.201	4557.276
TSPYL2	0.990607	0.009393	0.96523	0.965204	746.8672	773.7921
FBRS	0.990608	0.009392	0.835786	0.835747	2020.543	2417.651
ZNF592	0.990609	0.009391	1.062309	1.062354	846.8941	797.1859
CHD6	0.990611	0.009389	0.866242	0.866195	1382.594	1596.171
RCN2	0.990611	0.009389	0.865518	0.865471	1428.161	1650.157
ABT1	0.990611	0.009389	0.886328	0.886277	1134.749	1280.356
ASB6	0.990613	0.009387	1.02121	1.021227	746.2226	730.7117
PARP10	0.990616	0.009384	1.014696	1.014707	756.8699	745.8996
ENTPD6	0.990617	0.009383	0.910164	0.910115	945.8096	1039.221
EIF2AK3	0.990619	0.009381	1.133496	1.133561	1325.912	1169.686
TXNDC17	0.990624	0.009376	0.926171	0.926126	862.4538	931.2498
PGS1	0.990631	0.009369	0.894497	0.894446	1064.73	1190.38
WDR61	0.990632	0.009368	1.115179	1.115242	1156.977	1037.421
BAZ2A	0.990633	0.009367	1.264411	1.264446	5483.695	4336.835
RNF145	0.990634	0.009366	1.286991	1.287019	7623.158	5923.109
FRYL	0.990636	0.009364	0.937245	0.937204	821.3317	876.3646
UFD1L	0.990637	0.009363	0.871158	0.87111	1321.466	1516.993
ARPC1A	0.990638	0.009362	0.768587	0.768572	6875.18	8945.397
VLDLR	0.990638	0.009362	1.228542	1.228587	3494.272	2844.136
RRP9	0.990639	0.009361	1.072472	1.072522	879.125	819.6798
ANKHD1	0.990639	0.009361	0.785626	0.785606	4792.72	6100.667
KMT5A	0.99064	0.00936	0.899665	0.899615	1006.937	1119.299
UNC45A	0.990646	0.009354	1.169488	1.169549	1840.494	1573.677
MAEA	0.990647	0.009353	0.938916	0.938876	838.0028	892.5602
BTBD6	0.990647	0.009353	0.923268	0.923223	881.3478	954.6436
GPN3	0.990649	0.009351	0.953354	0.953321	777.9867	816.0808
NDC80	0.990653	0.009347	1.109102	1.109164	1114.744	1005.03
BCL2L2	0.990653	0.009347	1.139597	1.139662	1406.744	1234.351
USP8	0.990656	0.009344	0.861715	0.861669	1471.506	1707.741
SYVN1	0.990662	0.009338	0.84287	0.84283	1904.956	2260.193
COX7C	0.990665	0.009335	0.811875	0.811845	2970.798	3659.317
ADRM1	0.990667	0.009333	0.826111	0.826076	2365.08	2863.031
C1orf109	0.990669	0.009331	1.03739	1.037418	786.878	758.4963
SLFN13	0.99067	0.00933	1.116331	1.116394	1178.583	1055.704
CENPF	0.990671	0.009329	1.203126	1.203179	2628.484	2184.613
CRIP2	0.990675	0.009325	1.167832	1.167893	1792.704	1534.988
SCAP	0.990676	0.009324	0.885005	0.884955	1168.091	1319.945
MRPS26	0.990676	0.009324	0.917524	0.917477	940.2525	1024.825
ADI1	0.990678	0.009322	0.899901	0.899851	1033.678	1148.721
MARCH8	0.990679	0.009321	0.924148	0.924102	884.682	957.3428
EIF2B5	0.990679	0.009321	0.948281	0.948246	796.8807	840.3743
ZNF263	0.990689	0.009311	1.019633	1.019648	777.9867	762.995

ZNF281	0.990691	0.009309	1.159314	1.159376	1732.688	1494.499
VPS26B	0.990691	0.009309	0.863134	0.863087	1461.504	1693.345
MUC20	0.990691	0.009309	1.075754	1.075804	922.47	857.4697
NCDN	0.990693	0.009307	0.882609	0.88256	1199.211	1358.788
KIAA1549	0.990693	0.009307	0.974671	0.974652	752.4243	771.9926
RRNAD1	0.990698	0.009302	0.97609	0.976072	770.2068	789.088
LSR	0.990698	0.009302	1.246741	1.246781	4438.97	3560.344
IRF3	0.990704	0.009296	1.202778	1.202831	2640.709	2195.41
CAMK2D	0.990704	0.009296	1.138356	1.13842	1411.49	1239.867
RYBP	0.990706	0.009294	0.843454	0.843414	1866.057	2212.506
CAPN15	0.990706	0.009294	0.937406	0.937366	856.8968	914.1544
PPP1R11	0.990713	0.009287	0.841359	0.84132	1922.739	2285.386
UIMC1	0.990716	0.009284	0.958398	0.95837	800.2149	834.9757
PRADC1	0.990718	0.009282	1.010771	1.010779	755.7585	747.6992
PPP4R3B	0.990718	0.009282	0.857482	0.857438	1609.321	1876.896
CADM1	0.99072	0.00928	0.922707	0.922662	885.7934	960.0421
PHRF1	0.990723	0.009277	0.946036	0.946001	832.4458	879.9636
NSUN5	0.990726	0.009274	1.028521	1.028542	781.2654	759.585
ZNF384	0.990728	0.009272	0.969355	0.969333	765.7612	789.9878
TBRG1	0.99073	0.00927	0.887864	0.887815	1184.04	1333.658
CCNT2	0.990732	0.009268	1.016541	1.016554	764.6498	752.1979
EIF3F	0.990734	0.009266	1.203581	1.203633	2751.85	2286.286
VPS37A	0.990734	0.009266	0.875461	0.875413	1330.357	1519.692
POLE	0.990738	0.009262	0.913229	0.913182	958.0351	1049.118
RIOK3	0.990741	0.009259	0.849046	0.849005	1751.604	2063.128
BCL10	0.990749	0.009251	0.926733	0.92669	911.3559	983.4538
CSNK1E	0.99075	0.00925	1.191457	1.191512	2422.928	2033.49
MAP2K7	0.990751	0.009249	0.891022	0.890973	1159.2	1301.051
TRABD	0.99076	0.00924	1.105766	1.105825	1149.198	1039.221
ACLY	0.99076	0.00924	1.267063	1.267096	6010.503	4743.526
ZFP64	0.99076	0.00924	1.101906	1.101964	1093.627	992.4334
CAMSAP3	0.990762	0.009238	1.048378	1.048412	835.78	797.1859
LMNB2	0.990766	0.009234	1.224513	1.224558	3443.147	2811.745
MAP4K3	0.99077	0.00923	0.853125	0.853082	1719.351	2015.459
PPRC1	0.990773	0.009227	0.879052	0.879004	1267.007	1441.413
FKBP15	0.990774	0.009226	1.090828	1.090883	1024.72	939.3477
TYW3	0.990776	0.009224	1.021341	1.021357	780.2095	763.8948
RPN2	0.990778	0.009222	0.755778	0.755767	9819.304	12992.51
STAT2	0.990781	0.009219	0.820791	0.82076	2711.428	3303.562
LRPAP1	0.990783	0.009217	0.874251	0.874204	1331.28	1522.85
UBTF	0.990784	0.009216	0.857811	0.857768	1631.549	1902.089
IL17RD	0.990785	0.009215	1.082018	1.08207	977.8181	903.6543
ORMDL1	0.990785	0.009215	1.045377	1.04541	832.4458	796.2861
PPP2R2D	0.990787	0.009213	0.991081	0.991075	762.427	769.2934
ATIC	0.990787	0.009213	0.80959	0.809562	3187.523	3937.342
PPP1R26	0.990789	0.009211	1.013811	1.013822	773.5411	762.995
VPS4B	0.99079	0.00921	1.050442	1.050477	833.5794	793.5238
XPO4	0.990796	0.009204	0.892707	0.892659	1109.187	1242.566
LIG4	0.9908	0.0092	1.022292	1.022309	805.7719	788.1883
PLXNA3	0.990803	0.009197	1.090798	1.090853	1058.062	969.9395
XPO5	0.990805	0.009195	0.876114	0.876067	1314.798	1500.797
MYSM1	0.990805	0.009195	0.963317	0.963291	791.3236	821.4793
SLC12A8	0.990806	0.009194	0.985458	0.985447	767.984	779.3257
TTC37	0.990806	0.009194	0.86087	0.860826	1545.971	1795.918
CAMKK2	0.990806	0.009194	1.09538	1.095436	1062.508	969.9395
SLC35A2	0.990814	0.009186	1.149172	1.149233	1633.772	1421.618

PSEN1	0.990815	0.009185	1.166586	1.166645	1871.614	1604.269
SH3D19	0.990821	0.009179	0.89216	0.892112	1170.314	1311.848
SLF2	0.990826	0.009174	0.944919	0.944884	839.1142	888.0614
PRKCZ	0.990828	0.009172	1.007053	1.007058	794.6578	789.088
ANP32A	0.990828	0.009172	0.84733	0.84729	1832.703	2163.019
ZNF358	0.99083	0.00917	0.897724	0.897676	1075.844	1198.478
OGFR	0.990831	0.009169	1.094626	1.094681	1064.73	972.6387
GPR137B	0.990832	0.009168	1.021183	1.021199	811.329	794.4866
HECTD4	0.990833	0.009167	0.888314	0.888266	1183.651	1332.542
SAFB2	0.990834	0.009166	0.971048	0.971028	785.9889	809.4406
C5orf28	0.990835	0.009165	1.029803	1.029825	807.1279	783.7525
POLDIP3	0.990836	0.009164	1.181678	1.181734	2185.031	1849.003
CFAP20	0.990838	0.009162	1.001083	1.001084	793.5464	792.6871
FBXO22	0.99084	0.00916	1.00946	1.009467	780.2095	772.8924
PA2G4	0.990843	0.009157	0.775278	0.775262	6259.125	8073.567
ERCC1	0.990845	0.009155	1.121904	1.121965	1287.09	1147.174
CCDC25	0.990845	0.009155	0.907725	0.907679	1022.497	1126.497
SETD1A	0.990845	0.009155	0.914763	0.914717	976.929	1068.013
PHF5A	0.990866	0.009134	1.116136	1.116195	1271.453	1139.094
TOMM7	0.990868	0.009132	0.900583	0.900536	1082.513	1202.077
TUBGCP2	0.990868	0.009132	0.849855	0.849815	1793.815	2110.833
MFAP1	0.990869	0.009131	0.979872	0.979858	784.6552	800.7849
EED	0.990873	0.009127	1.014643	1.014654	792.435	780.9902
PLXNA1	0.990873	0.009127	1.228574	1.228617	3716.554	3024.987
ATP9A	0.990874	0.009126	1.17785	1.177907	2123.904	1803.116
ZNF503	0.990875	0.009125	1.017988	1.018001	796.8807	782.7897
PHAX	0.990876	0.009124	0.874037	0.873992	1401.81	1603.918
NPEPPS	0.990878	0.009122	1.225256	1.225299	3577.65	2919.815
IMPA2	0.990879	0.009121	0.931738	0.931698	883.5706	948.3453
MRPL30	0.990881	0.009119	0.865026	0.864982	1505.96	1741.032
MANF	0.990884	0.009116	0.822597	0.822566	2642.932	3213.037
BRIX1	0.990884	0.009116	0.900488	0.900441	1088.07	1208.375
PUS7	0.990884	0.009116	0.879018	0.878971	1338.137	1522.391
TSG101	0.990885	0.009115	0.976138	0.976121	791.3236	810.6822
RAP2A	0.990888	0.009112	0.978523	0.978508	809.1062	826.8779
PPP6R2	0.990894	0.009106	0.970559	0.970539	819.1089	843.9733
ADAMTS1	0.990895	0.009105	1.245431	1.245469	4596.79	3690.809
CASC3	0.990896	0.009104	1.150296	1.150355	1687.12	1466.606
FAM63B	0.990898	0.009102	1.048197	1.04823	866.8439	826.9589
POLR2I	0.990903	0.009097	1.013585	1.013595	797.9921	787.2885
ITGB1BP1	0.990904	0.009096	1.049176	1.04921	871.3451	830.4769
SMAD5	0.990904	0.009096	0.838288	0.838252	2161.703	2578.824
ANKRD28	0.990906	0.009094	0.851667	0.851626	1771.587	2080.241
BBS2	0.990913	0.009087	0.981485	0.981472	812.4404	827.7776
NAA60	0.990914	0.009086	1.074653	1.074699	974.7062	906.9564
HMGCS1	0.990915	0.009085	0.83487	0.834835	2180.586	2611.998
PSMG2	0.990916	0.009084	0.985979	0.98597	791.3236	802.5844
HECTD3	0.990916	0.009084	1.10256	1.102617	1145.863	1039.221
RPIA	0.990917	0.009083	1.017649	1.017662	805.7719	791.7873
GLE1	0.990918	0.009082	0.907199	0.907153	1072.51	1182.282
E2F4	0.990918	0.009082	0.901061	0.901015	1093.627	1213.774
POM121C	0.99092	0.00908	0.911733	0.911688	1016.251	1114.693
PNMA1	0.990928	0.009072	1.041732	1.041761	855.7854	821.4793
LOC10272	0.990932	0.009068	0.990194	0.990187	793.6576	801.5227
UBL4A	0.990932	0.009068	1.151194	1.151253	1676.006	1455.809
CDKN2AIP	0.990936	0.009064	0.990131	0.990124	794.6578	802.5844

TTC19	0.990937	0.009063	0.873526	0.873481	1398.153	1600.67
TMCC1	0.990937	0.009063	1.10574	1.105796	1205.879	1090.507
FBXO2	0.990939	0.009061	0.928128	0.928087	930.2498	1002.331
MPST	0.990943	0.009057	1.065743	1.065785	964.7035	905.1569
PRPF31	0.990943	0.009057	0.819432	0.819402	2822.98	3445.175
ATP13A2	0.990953	0.009047	1.091311	1.091364	1079.179	988.8344
NAPA	0.990954	0.009046	1.064006	1.064047	960.2579	902.4576
C19orf12	0.990956	0.009044	1.020573	1.020588	838.3918	821.4793
FNTA	0.990958	0.009042	1.130734	1.130794	1395.93	1234.468
SLC38A10	0.990959	0.009041	1.186268	1.186321	2426.207	2045.151
NSMAF	0.990959	0.009041	0.914398	0.914354	1010.271	1104.903
PDP1	0.99096	0.00904	1.155267	1.155325	1751.581	1516.093
CDS1	0.990963	0.009037	0.871222	0.871178	1441.498	1654.656
HTATIP2	0.990963	0.009037	1.087741	1.087792	1041.391	957.3428
GLA	0.990964	0.009036	1.088991	1.089042	1068.065	980.7366
ETHE1	0.990968	0.009032	0.895504	0.895457	1156.977	1292.053
CCDC93	0.990968	0.009032	1.028426	1.028446	834.6686	811.582
LRIG1	0.990971	0.009029	1.056156	1.056193	911.3559	862.8682
ZNF609	0.990972	0.009028	1.143818	1.143876	1628.215	1423.418
MRPS21	0.990973	0.009027	1.144322	1.144381	1595.984	1394.625
ZNF260	0.990974	0.009026	0.924351	0.92431	981.3524	1061.715
USP4	0.990975	0.009025	1.00538	1.005384	806.9056	802.5844
GALNT7	0.990976	0.009024	0.880762	0.880716	1317.02	1495.398
FAM96A	0.990978	0.009022	0.9843	0.984289	825.3994	838.5747
ARID4A	0.990978	0.009022	0.918973	0.91893	988.0431	1075.211
TRA2A	0.990979	0.009021	1.004228	1.004231	806.8834	803.4842
INO80E	0.99098	0.00902	0.900462	0.900417	1144.752	1271.358
TTLL4	0.990982	0.009018	0.968317	0.968296	822.4431	849.3718
NTPCR	0.990982	0.009018	0.980655	0.980642	809.1062	825.0784
RAP1GDS	0.990984	0.009016	1.156756	1.156814	1787.147	1544.885
SNX4	0.990989	0.009011	0.900179	0.900133	1122.524	1247.065
ERCC3	0.99099	0.00901	1.08107	1.081118	1029.165	951.9443
ZNF622	0.990991	0.009009	1.003791	1.003794	829.1115	825.9781
SAP30L	0.990992	0.009008	0.959249	0.959223	861.3424	897.9588
NADSYN1	0.990992	0.009008	1.055617	1.055654	914.6901	866.4672
KCNAB2	0.990992	0.009008	0.937706	0.937669	903.576	963.6411
SHARPIN	0.990996	0.009004	0.85801	0.857969	1723.796	2009.16
SZRD1	0.991004	0.008996	1.242027	1.242065	4645.692	3740.295
B4GALT7	0.991007	0.008993	0.970933	0.970913	824.6659	849.3718
RHOF	0.991008	0.008992	1.120783	1.120841	1334.581	1190.695
OSBPL3	0.991011	0.008989	0.953102	0.953072	844.6713	886.2619
SYT13	0.991012	0.008988	0.875652	0.875608	1431.496	1634.861
RER1	0.991024	0.008976	0.872202	0.872159	1487.066	1705.042
WDR77	0.991024	0.008976	1.121194	1.121252	1344.806	1199.378
SLC25A29	0.991025	0.008975	1.121318	1.121376	1379.259	1229.97
CAPZA1	0.991026	0.008974	1.215786	1.215831	3413.139	2807.246
CHCHD1	0.991027	0.008973	1.059448	1.059486	938.0297	885.3622
MORC2	0.991027	0.008973	1.10606	1.106116	1209.214	1093.206
DIS3	0.991028	0.008972	1.121724	1.121782	1351.507	1204.785
FXR2	0.991037	0.008963	1.08749	1.08754	1080.29	993.3332
RNF5P1	0.991037	0.008963	1.007465	1.00747	822.3764	816.2787
STUB1	0.99104	0.00896	1.099794	1.099848	1164.757	1059.016
HHLA3	0.991045	0.008955	1.058552	1.05859	939.1411	887.1617
STX16	0.991045	0.008955	1.214495	1.21454	3380.752	2783.564
SLC7A6	0.991046	0.008954	0.96957	0.96955	855.7854	882.6629
IL1R2	0.991052	0.008948	1.022384	1.022399	843.5599	825.0784

KRI1	0.991056	0.008944	0.939315	0.939279	913.5787	972.6387
PHACTR4	0.991057	0.008943	1.218118	1.218162	3451.238	2833.15
EXOC8	0.991061	0.008939	1.11471	1.114767	1295.904	1162.488
TCF19	0.991063	0.008937	1.197189	1.197238	2801.864	2340.271
SLC25A37	0.991065	0.008935	1.040084	1.040111	905.7988	870.8671
USMG5	0.991066	0.008934	1.087839	1.087889	1091.404	1003.231
GLT8D1	0.991066	0.008934	0.947892	0.947861	909.133	959.1424
CNOT4	0.991068	0.008932	1.065346	1.065386	996.9344	935.7486
CYHR1	0.991069	0.008931	0.908678	0.908635	1106.964	1218.273
ARMT1	0.991069	0.008931	0.958289	0.958263	861.3424	898.8585
SPICE1	0.99107	0.00893	0.937605	0.937569	923.7258	985.2353
GPS1	0.991072	0.008928	0.813575	0.813548	3214.197	3950.839
POLA1	0.991072	0.008928	0.92061	0.920569	1004.714	1091.407
GTF3C4	0.991073	0.008927	1.17417	1.174224	2160.58	1840.006
NMD3	0.991073	0.008927	0.817581	0.817553	3010.809	3682.711
CSK	0.991073	0.008927	1.001102	1.001102	826.8887	825.9781
WDR83OS	0.991073	0.008927	0.854426	0.854387	1792.704	2098.236
XPO7	0.991074	0.008926	1.192481	1.192531	2607.367	2186.413
TAF1	0.991076	0.008924	0.877623	0.877579	1429.184	1628.554
BOLA2	0.991078	0.008922	0.945794	0.945761	896.9075	948.3453
BOLA2B	0.991078	0.008922	0.945794	0.945761	896.9075	948.3453
SBF2	0.991083	0.008917	1.016607	1.016618	842.4485	828.6774
FANCG	0.991083	0.008917	1.006729	1.006734	832.4458	826.8779
RPL13A	0.991086	0.008914	1.250424	1.250458	5277.039	4220.082
RERE	0.991087	0.008913	1.084097	1.084145	1074.733	991.3177
SMIM15	0.991087	0.008913	0.905644	0.905601	1135.861	1254.263
ORMDL2	0.991088	0.008912	0.905218	0.905174	1112.521	1229.07
PARP9	0.99109	0.00891	1.052619	1.052653	929.1384	882.6629
DUT	0.991092	0.008908	1.063935	1.063974	973.5948	915.0542
CAMK2G	0.991094	0.008906	1.132657	1.132714	1498.18	1322.645
MLX	0.991097	0.008903	0.932851	0.932813	973.5948	1043.72
NGLY1	0.991103	0.008897	0.97358	0.973563	843.5599	866.4672
RUFY1	0.991105	0.008895	0.851957	0.851918	1909.402	2241.298
FAAP100	0.991107	0.008893	0.980397	0.980384	838.0028	854.7704
TMEM9B	0.991108	0.008892	0.945665	0.945632	889.1277	940.2474
TLE1	0.99111	0.00889	1.187172	1.187224	2446.212	2060.447
IL1RAP	0.99111	0.00889	1.007122	1.007127	839.1142	833.1762
SIK2	0.991111	0.008889	1.087723	1.087772	1131.415	1040.121
UCA1	0.991112	0.008888	1.015594	1.015605	848.0055	834.9757
YDJC	0.991113	0.008887	0.978122	0.978107	841.3371	860.1689
VPS53	0.991119	0.008881	1.038257	1.038282	893.1732	860.2409
ECSIT	0.991122	0.008878	0.975199	0.975183	866.8995	888.9612
ARID1B	0.991126	0.008874	0.974662	0.974645	848.0055	870.0663
NFKBIA	0.991127	0.008873	1.138841	1.138898	1629.326	1430.616
POLR1E	0.991129	0.008871	1.071581	1.071624	1019.163	951.0445
NT5C2	0.991129	0.008871	1.122515	1.122571	1398.153	1245.49
RNF144B	0.99113	0.00887	0.787298	0.78728	5268.081	6691.503
ATPAF1	0.991132	0.008868	0.96076	0.960736	871.3451	906.9564
ADPRHL2	0.991134	0.008866	1.072197	1.072241	1023.608	954.6436
FAM53B	0.991136	0.008864	0.96928	0.96926	842.4485	869.1665
NFS1	0.991136	0.008864	0.914709	0.914667	1059.173	1157.989
ATXN2	0.991144	0.008856	0.895057	0.895013	1219.216	1362.234
KIAA1217	0.991147	0.008853	0.882455	0.882412	1359.254	1540.386
RWDD1	0.991147	0.008853	0.946742	0.946711	933.584	986.1351
PRPF4B	0.991152	0.008848	0.831877	0.831845	2470.663	2970.102
GPR108	0.991154	0.008846	0.840608	0.840573	2187.254	2602.101

ZNF160	0.991154	0.008846	1.104647	1.1047	1241.033	1123.411
NCAPH2	0.991156	0.008844	0.900805	0.900762	1170.314	1299.251
MEA1	0.991157	0.008843	0.860451	0.860411	1709.348	1986.666
EIF2AK4	0.991158	0.008842	1.173386	1.173439	2195.034	1870.598
KIFC1	0.99116	0.00884	1.179586	1.179638	2336.183	1980.422
MTMR3	0.991161	0.008839	1.127509	1.127565	1501.514	1331.642
USP31	0.991161	0.008839	1.089985	1.090035	1134.749	1041.02
FAM98B	0.991164	0.008836	1.000511	1.000511	848.0055	847.5723
TBL2	0.991165	0.008835	0.967619	0.967598	888.0163	917.7535
RAB24	0.991166	0.008834	0.900168	0.900125	1179.206	1310.048
KIAA2013	0.991169	0.008831	1.169649	1.169702	2122.792	1814.813
NUF2	0.991169	0.008831	1.048421	1.048452	935.8069	892.5602
MTHFR	0.99117	0.00883	0.978277	0.978263	854.674	873.6653
GPATCH8	0.991171	0.008829	0.905699	0.905656	1114.744	1230.869
POLR2A	0.991175	0.008825	1.225359	1.225399	3878.819	3165.35
ELOVL1	0.991176	0.008824	1.16697	1.167024	2072.779	1776.123
MRPS15	0.99118	0.00882	0.846433	0.846397	2039.437	2409.553
MRPS12	0.991181	0.008819	1.15877	1.158824	1921.627	1658.255
SCYL1	0.991186	0.008814	1.206876	1.20692	3221.976	2669.583
JADE1	0.991187	0.008813	0.975685	0.975669	861.3535	882.8339
GSE1	0.991189	0.008811	0.869531	0.86949	1561.53	1795.918
TIPARP	0.991192	0.008808	0.855199	0.855161	1880.505	2199.009
DHX8	0.991193	0.008807	1.071736	1.071778	1020.274	951.9443
SASH1	0.991193	0.008807	0.929638	0.9296	991.3773	1066.457
GGCT	0.991195	0.008805	0.836662	0.836629	2335.072	2791.05
UBR1	0.991196	0.008804	0.958174	0.958149	914.6901	954.6436
CMC2	0.991197	0.008803	1.042081	1.042108	923.5814	886.2619
TRAPPC5	0.991199	0.008801	0.971279	0.971261	854.674	879.9636
PEX5	0.991202	0.008798	0.945685	0.945654	929.1384	982.5361
EHMT1	0.991207	0.008793	1.012051	1.012059	866.8995	856.5699
CSTF3	0.99121	0.00879	0.99749	0.997488	858.0082	860.1689
PIK3R4	0.991211	0.008789	0.896877	0.896834	1254.781	1399.124
APMAP	0.991213	0.008787	0.848436	0.8484	2006.094	2364.565
HIRA	0.991215	0.008785	0.948105	0.948075	924.6928	975.338
LRRC16A	0.991216	0.008784	1.138964	1.139019	1631.549	1432.415
GAK	0.991219	0.008781	0.877372	0.87733	1454.835	1658.255
TRIOBP	0.99122	0.00878	1.153907	1.153961	1858.555	1610.585
MTMR12	0.99122	0.00878	0.904718	0.904676	1158.322	1280.374
PKNOX1	0.991226	0.008774	1.005498	1.005502	865.7881	861.0507
TGFBRAP	0.991226	0.008774	0.936879	0.936844	991.7441	1058.602
HNRNPA3	0.991226	0.008774	0.785903	0.785886	5559.271	7073.891
SPATS2L	0.99123	0.00877	0.872764	0.872723	1565.154	1793.416
VPS39	0.991232	0.008768	1.132372	1.132426	1591.539	1405.422
DAAM1	0.991233	0.008767	1.052515	1.052547	966.9263	918.6532
GCAT	0.991233	0.008767	0.996612	0.996609	863.5652	866.5032
GINS4	0.991239	0.008761	1.035613	1.035636	916.9129	885.3622
KLHL8	0.991241	0.008759	1.06696	1.066999	1029.165	964.5409
SHOC2	0.991241	0.008759	0.90622	0.906178	1181.428	1303.75
MYH10	0.991244	0.008756	1.225565	1.225604	3962.175	3232.832
ACOT7	0.991248	0.008752	0.881853	0.881811	1405.933	1594.372
HMGXB4	0.991251	0.008749	1.03098	1.030999	931.3612	903.3573
AGAP1	0.991253	0.008747	0.913358	0.913318	1103.63	1208.375
PRPS2	0.991255	0.008745	1.175684	1.175735	2306.175	1961.473
PIGS	0.991255	0.008745	0.924234	0.924195	1036.945	1121.999
LINC0099	0.991258	0.008742	1.166212	1.166264	2105.01	1804.915
UBR3	0.991258	0.008742	0.894152	0.894109	1299.238	1453.11

IPO13	0.991259	0.008741	0.923026	0.922987	1044.725	1131.896
FAM35A	0.991262	0.008738	0.868878	0.868837	1644.053	1892.246
RNF187	0.991266	0.008734	1.19904	1.199086	2949.681	2459.939
XPO1	0.991269	0.008731	1.277033	1.277059	7668.726	6004.987
ODF2L	0.99127	0.00873	0.942616	0.942584	958.0017	1016.358
CCSER2	0.991271	0.008729	1.18645	1.186499	2647.567	2231.41
PLEKHG2	0.991271	0.008729	0.964646	0.964624	898.5746	931.5288
CCNT1	0.991271	0.008729	1.150782	1.150836	1833.826	1593.472
POLD3	0.991272	0.008728	0.935689	0.935654	985.8203	1053.617
ESRRA	0.991273	0.008727	1.148717	1.148771	1801.595	1568.279
TTL	0.991273	0.008727	0.899135	0.899092	1224.773	1362.234
RHEB	0.991273	0.008727	1.266806	1.266835	6860.731	5415.645
NRBP2	0.991275	0.008725	1.14434	1.144394	1778.255	1553.883
PARL	0.991279	0.008721	0.955753	0.955727	942.4753	986.1351
HIGD2A	0.991279	0.008721	0.886597	0.886555	1359.254	1533.188
GEMIN4	0.991281	0.008719	1.023246	1.023261	901.3532	880.8634
CTNNBL1	0.991283	0.008717	0.891104	0.891062	1309.24	1469.305
KANK1	0.991286	0.008714	1.149729	1.149783	1869.391	1625.863
PKD1	0.991287	0.008713	0.839517	0.839483	2302.674	2742.967
ACVR1B	0.991287	0.008713	1.032244	1.032264	921.3585	892.5602
AK6	0.991295	0.008705	1.026627	1.026644	933.5507	909.3227
TOP1	0.991296	0.008704	0.803739	0.803717	4144.369	5156.506
SLC37A1	0.991297	0.008703	1.087538	1.087585	1162.534	1068.913
RCC1	0.991299	0.008701	1.181687	1.181736	2541.127	2150.332
PSMD13	0.9913	0.0087	1.170996	1.171047	2230.599	1904.788
NEMP1	0.9913	0.0087	0.850377	0.850341	2006.094	2359.166
ZNF22	0.991301	0.008699	0.946306	0.946276	953.5894	1007.729
TIAL1	0.991303	0.008697	0.85725	0.857213	1892.731	2208.007
NUP98	0.991304	0.008696	1.207783	1.207826	3281.992	2717.27
RAE1	0.991306	0.008694	1.151176	1.151229	1858.277	1614.166
BCL2L2-P	0.991308	0.008692	0.961418	0.961395	914.4678	951.1885
PDHX	0.991308	0.008692	1.054964	1.054997	1021.385	968.1399
TGIF2	0.99131	0.00869	0.898197	0.898155	1277.132	1421.951
SACM1L	0.991313	0.008687	0.94867	0.94864	949.1438	1000.531
CSNK2A2	0.991316	0.008684	1.026022	1.026039	915.8015	892.5602
NCBP1	0.991321	0.008679	1.165838	1.16589	2134.829	1831.071
RPL23	0.991322	0.008678	1.304639	1.304659	11424.18	8756.448
SH3GL1	0.991324	0.008676	1.107584	1.107635	1360.365	1228.17
TOR1A	0.991324	0.008676	1.061927	1.061963	1055.839	994.2329
ATP6V0A1	0.991325	0.008675	0.930497	0.930461	1024.72	1101.304
PCNT	0.991328	0.008672	1.086836	1.086882	1196.988	1101.304
TMEM19	0.991331	0.008669	1.00162	1.001621	911.3559	909.8806
GRINA	0.991332	0.008668	0.78784	0.787822	5409.23	6866.056
GMFB	0.991337	0.008663	0.851096	0.85106	2058.331	2418.55
METAP1	0.991343	0.008657	1.138123	1.138176	1720.462	1511.594
RGL3	0.991343	0.008657	0.932554	0.932519	1020.274	1094.106
DIABLO	0.991344	0.008656	0.933344	0.933309	1016.94	1089.607
ZNF28	0.991345	0.008655	1.122222	1.122275	1456.613	1297.91
BDP1	0.991346	0.008654	1.008143	1.008148	898.0189	890.7607
TYK2	0.991347	0.008653	0.891863	0.891821	1324.8	1485.501
MAD2L2	0.991347	0.008653	0.90989	0.90985	1159.2	1274.058
ZDHHC16	0.991347	0.008653	1.052549	1.05258	999.1572	949.245
ARG2	0.991348	0.008652	1.086192	1.086238	1199.211	1104.003
BRD2	0.991348	0.008652	1.248778	1.24881	5417.01	4337.735
PIH1D1	0.991351	0.008649	0.883931	0.88389	1420.381	1606.968
WNT7A	0.991351	0.008649	0.904542	0.904501	1203.657	1330.743

NIT2	0.991351	0.008649	0.969965	0.969947	910.2444	938.4479
ADAM15	0.991354	0.008646	1.242621	1.242655	5022.46	4041.714
SMOX	0.991354	0.008646	0.966474	0.966454	939.1411	971.739
DHX38	0.991356	0.008644	1.029341	1.029359	955.8122	928.5506
POLA2	0.991357	0.008643	0.935131	0.935097	1012.561	1082.841
SRPK1	0.991362	0.008638	1.245549	1.245583	5349.214	4294.546
PROSER1	0.991363	0.008637	0.869473	0.869434	1640.441	1886.793
PDE12	0.991364	0.008636	0.94711	0.94708	951.3666	1004.526
SMC2	0.991366	0.008634	1.113469	1.11352	1433.718	1287.554
LSS	0.991367	0.008633	1.212654	1.212695	3528.725	2909.818
ANAPC5	0.991369	0.008631	0.875585	0.875545	1582.647	1807.614
OSBPL11	0.991371	0.008629	1.132999	1.133051	1663.78	1468.406
ALDH2	0.991374	0.008626	0.928378	0.928342	1075.844	1158.889
ATP6AP2	0.991378	0.008622	1.250617	1.250649	5589.279	4469.099
CDT1	0.991378	0.008622	0.976227	0.976213	909.133	931.2858
ATR	0.991381	0.008619	0.843726	0.843693	2289.504	2713.671
DYNC1LI1	0.991382	0.008618	0.90246	0.902419	1232.553	1365.833
CARS2	0.991383	0.008617	1.159898	1.15995	2054.996	1771.624
KANSL3	0.991389	0.008611	0.907473	0.907433	1221.439	1346.038
DCAF5	0.99139	0.00861	0.851418	0.851382	2080.559	2443.744
ZFAND6	0.99139	0.00861	1.112011	1.112062	1394.819	1254.263
GTF2E2	0.991394	0.008606	1.101795	1.101844	1303.683	1183.182
CCNB1	0.991396	0.008604	1.190731	1.190777	2798.529	2350.169
URM1	0.991397	0.008603	1.014207	1.014216	918.0243	905.1569
MNAT1	0.991399	0.008601	1.055798	1.055831	1026.942	972.6387
RPS21	0.991402	0.008598	1.266257	1.266285	6888.517	5439.939
GBF1	0.991404	0.008596	1.184674	1.184721	2632.929	2222.403
LRRC14	0.991409	0.008591	1.100344	1.100392	1297.015	1178.683
TFB2M	0.991409	0.008591	1.014219	1.014227	943.5867	930.3501
BRWD1	0.99141	0.00859	1.166097	1.166147	2195.034	1882.294
FIP1L1	0.991412	0.008588	1.000647	1.000647	910.2444	909.6556
GTF2B	0.991414	0.008586	1.011415	1.011422	919.1357	908.7559
ICE2	0.991415	0.008585	0.970059	0.970041	926.9156	955.5433
ATG12	0.991415	0.008585	1.141859	1.141911	1768.253	1548.502
MINOS1	0.991418	0.008582	0.86718	0.867142	1707.459	1969.067
KLC2	0.991419	0.008581	0.887468	0.887428	1403.71	1581.775
CLINT1	0.99142	0.00858	0.774932	0.774919	7353.086	9488.851
CHEK2	0.99142	0.00858	0.943953	0.943923	1019.163	1079.71
MEF2A	0.991421	0.008579	0.925272	0.925235	1081.402	1168.786
ZNF33A	0.991429	0.008571	1.061858	1.061894	1061.885	999.9914
ALG3	0.991433	0.008567	1.081911	1.081954	1200.322	1109.402
AZI2	0.991438	0.008562	0.969076	0.969058	934.6955	964.5409
UBR7	0.991447	0.008553	1.109534	1.109584	1393.708	1256.063
TRIP12	0.991447	0.008553	1.223328	1.223366	4011.077	3278.719
PKMYT1	0.991449	0.008551	0.969571	0.969553	936.9183	966.3404
SNU13	0.99145	0.00855	1.185911	1.185958	2701.837	2278.188
SECISBP2	0.99145	0.00855	0.904204	0.904164	1241.445	1373.031
GBA2	0.991456	0.008544	0.992986	0.992982	920.2471	926.7511
N4BP2L2	0.991457	0.008543	0.988431	0.988424	921.3585	932.1496
METTL13	0.991459	0.008541	0.86541	0.865373	1760.473	2034.354
SMARCA5	0.99146	0.00854	0.818	0.817974	3407.582	4165.881
ZDHHC5	0.99146	0.00854	0.820293	0.820267	3207.528	3910.35
THOC7	0.991461	0.008539	0.861744	0.861707	1879.394	2181.014
EPB41	0.991461	0.008539	0.871306	0.871268	1696.111	1946.717
ACAT2	0.991462	0.008538	1.097516	1.097563	1293.681	1178.683
MRPL22	0.991464	0.008536	1.085369	1.085413	1183.651	1090.507

ROCK1	0.991468	0.008532	0.845569	0.845536	2235.856	2644.309
PRDX1	0.99147	0.00853	1.307054	1.307073	12314.42	9421.369
EPN2	0.991474	0.008526	1.09928	1.099327	1312.575	1193.979
KDM4C	0.991474	0.008526	0.998011	0.998009	925.8042	927.6508
SRSF6	0.991475	0.008525	0.809383	0.809361	3947.727	4877.59
TANC2	0.991476	0.008524	1.108557	1.108606	1430.384	1290.253
SEPHS1	0.991477	0.008523	0.926001	0.925965	1121.412	1211.075
SLC44A1	0.991478	0.008522	1.168447	1.168495	2344.563	2006.479
MYO19	0.991478	0.008522	1.095049	1.095095	1311.463	1197.578
POLR2F	0.991478	0.008522	0.99824	0.998239	926.9156	928.5506
APOE	0.991478	0.008522	0.923304	0.923268	1138.083	1232.669
TOP1MT	0.991478	0.008522	1.044177	1.044204	990.2659	948.3453
LSM2	0.991483	0.008517	0.938285	0.938253	1035.834	1104.003
HTRA2	0.991483	0.008517	1.05069	1.050719	1056.951	1005.93
S100PBP	0.991484	0.008516	1.048667	1.048695	1024.72	977.1375
TSPAN4	0.991484	0.008516	1.068501	1.068538	1111.41	1040.121
CENPU	0.991485	0.008515	0.92532	0.925285	1128.081	1219.173
CPSF3L	0.991485	0.008515	1.068129	1.068166	1136.972	1064.414
ZNF581	0.991486	0.008514	0.997509	0.997508	951.3666	953.7438
CUL7	0.991488	0.008512	1.001727	1.001728	953.5894	951.9443
VPS28	0.99149	0.00851	0.938526	0.938495	1036.945	1104.903
RTKN	0.99149	0.00851	1.037292	1.037314	990.2659	954.6436
VANGL1	0.991491	0.008509	1.01822	1.018231	949.1438	932.1496
PGRMC2	0.991492	0.008508	1.175985	1.176032	2472.886	2102.735
MFN1	0.991494	0.008506	0.870747	0.870709	1680.451	1929.982
MRPL17	0.991494	0.008506	0.922795	0.922759	1119.189	1212.874
ASXL2	0.991495	0.008505	1.085251	1.085295	1214.771	1119.299
MTHFD1L	0.991496	0.008504	0.870681	0.870643	1682.674	1932.681
ZNF451	0.991496	0.008504	1.112233	1.112282	1440.131	1294.752
ATG2A	0.991497	0.008503	1.021289	1.021302	939.1411	919.553
PIGA	0.991504	0.008496	0.986551	0.986543	934.6955	947.4455
EXOSC4	0.991505	0.008495	0.981683	0.981672	938.0297	955.5433
TMEM258	0.991506	0.008494	0.931151	0.931117	1102.518	1184.082
QSER1	0.991508	0.008492	0.869928	0.86989	1701.568	1956.075
FBXO34	0.991509	0.008491	1.02245	1.022464	961.3693	940.2474
FAM134A	0.99151	0.00849	1.166573	1.166622	2268.387	1944.405
SPRYD3	0.991513	0.008487	0.998133	0.998132	959.1465	960.9419
TTF2	0.991514	0.008486	0.937371	0.937339	1049.171	1119.308
SEC23B	0.991514	0.008486	0.860161	0.860125	1896.065	2204.408
CFDP1	0.991514	0.008486	0.880886	0.880847	1530.411	1737.433
MTF1	0.991515	0.008485	1.052198	1.052228	1047.381	995.3936
RPS16	0.991515	0.008485	1.324318	1.324334	16039.86	12111.65
TLN1	0.991516	0.008484	1.296315	1.296335	10964.06	8457.728
THOC2	0.991518	0.008482	0.847943	0.84791	2199.48	2594.003
SLC52A2	0.991518	0.008482	1.208766	1.208807	3609.858	2986.298
ZNF274	0.99152	0.00848	1.04005	1.040073	1006.937	968.1399
CHORDC1	0.99152	0.00848	1.265676	1.265704	7062.952	5580.256
ZNF410	0.991521	0.008479	0.880619	0.88058	1537.079	1745.531
SCYL2	0.991523	0.008477	0.815412	0.815388	3617.638	4436.708
ZNF354A	0.991524	0.008476	0.970676	0.970659	955.9345	984.8305
RARS2	0.991528	0.008472	1.046817	1.046844	1031.388	985.2353
LMAN2L	0.991531	0.008469	1.018997	1.019008	984.7089	966.3404
FAM120B	0.991533	0.008467	1.081035	1.081077	1200.322	1110.302
SPCS1	0.991536	0.008464	0.839327	0.839296	2537.348	3023.188
EIF6	0.991537	0.008463	0.815267	0.815243	3553.176	4358.429
EIF2S1	0.991538	0.008462	0.801076	0.801056	4537.885	5664.878

MTX1	0.99154	0.00846	0.935992	0.93596	1063.619	1136.395
SYNJ2BP	0.991542	0.008458	0.95428	0.954256	1020.874	1069.813
CHD4	0.991542	0.008458	0.795949	0.795931	5114.707	6426.074
VPS4A	0.991544	0.008456	0.911261	0.911223	1215.882	1334.342
OPN3	0.991544	0.008456	0.951418	0.951391	1005.826	1057.216
LGMN	0.991549	0.008451	0.823503	0.823476	3127.973	3798.501
AURKAIP1	0.991553	0.008447	0.893383	0.893344	1388.151	1553.883
MAN2A2	0.991555	0.008445	0.925543	0.925508	1122.524	1212.874
ALG2	0.991555	0.008445	1.001693	1.001694	972.4834	970.8392
GPAT4	0.991558	0.008442	1.108293	1.108341	1427.05	1287.554
USP47	0.991565	0.008435	0.873682	0.873644	1664.892	1905.688
ZNFX1	0.991565	0.008435	1.133188	1.133238	1719.462	1517.298
MRPL9	0.991568	0.008432	1.091705	1.091749	1319.243	1208.375
ASMTL	0.99157	0.00843	1.049087	1.049115	1052.505	1003.231
FKBP5	0.991572	0.008428	0.933383	0.93335	1085.847	1163.387
RFC3	0.991572	0.008428	1.045337	1.045363	1064.73	1018.526
LMTK2	0.991578	0.008422	0.863665	0.863629	1857.165	2150.422
RUNX1	0.991589	0.008411	0.8848	0.884762	1545.971	1747.331
HERC1	0.99159	0.00841	0.999734	0.999733	981.3747	981.6363
POLR3A	0.991591	0.008409	0.861106	0.861071	1918.293	2227.802
CCP110	0.991595	0.008405	0.914758	0.914721	1237.01	1352.337
MTMR1	0.991597	0.008403	1.000164	1.000164	983.5975	983.4358
C11orf24	0.9916	0.0084	1.025135	1.02515	992.4888	968.1399
ZNF608	0.991601	0.008399	0.912718	0.912681	1254.781	1374.831
UBR2	0.991606	0.008394	1.110594	1.110642	1505.96	1355.936
SLC30A5	0.991606	0.008394	1.101385	1.101431	1382.605	1255.28
MECP2	0.991611	0.008389	0.885418	0.88538	1510.406	1705.942
SKI	0.991613	0.008387	1.065694	1.065729	1166.98	1095.006
VKORC1L	0.991613	0.008387	0.967907	0.967889	985.8203	1018.526
ARHGEF3	0.991615	0.008385	1.156762	1.15681	2134.284	1844.972
VPS50	0.991617	0.008383	0.916308	0.916271	1203.657	1313.647
NFATC2IF	0.991617	0.008383	0.891589	0.891551	1470.395	1649.257
STRBP	0.991619	0.008381	0.86985	0.869814	1801.595	2071.244
SSU72	0.991631	0.008369	0.873075	0.873038	1749.359	2003.762
BRD1	0.991632	0.008368	0.944325	0.944297	1056.951	1119.299
RRM2	0.991635	0.008365	0.884868	0.88483	1565.809	1769.618
RIF1	0.991637	0.008363	0.85648	0.856446	2050.551	2394.257
C8orf33	0.991639	0.008361	0.844584	0.844553	2377.705	2815.344
PROSC	0.991639	0.008361	0.970322	0.970306	989.1545	1019.426
REXO4	0.991642	0.008358	0.978644	0.978632	979.1518	1000.531
MAF1	0.991644	0.008356	1.219102	1.219138	4210.019	3453.272
RHOT1	0.991645	0.008355	0.925566	0.925532	1152.532	1245.265
RBL2	0.991647	0.008353	0.900445	0.900407	1327.023	1473.804
STK40	0.991651	0.008349	0.89943	0.899392	1395.93	1552.083
SCARA3	0.991654	0.008346	1.098131	1.098175	1407.045	1281.256
TMEM184	0.991654	0.008346	1.214904	1.214941	3926.61	3231.932
EGFR	0.991656	0.008344	1.188541	1.188584	2945.235	2477.934
EMC7	0.991656	0.008344	0.939191	0.939161	1085.847	1156.189
DHFR	0.991662	0.008338	0.883607	0.883569	1558.196	1763.526
SLC12A7	0.991663	0.008337	1.050809	1.050837	1114.744	1060.815
MED21	0.991663	0.008337	0.94176	0.941731	1076.956	1143.593
ATRX	0.991663	0.008337	0.841518	0.841488	2494.003	2963.804
ZYX	0.991664	0.008336	1.27233	1.272355	8227.765	6466.563
REST	0.991664	0.008336	0.895563	0.895526	1409.267	1573.677
ANAPC13	0.991666	0.008334	1.2188	1.218837	4048.865	3321.908
HNRNPUL	0.991667	0.008333	0.804421	0.8044	4434.635	5512.972

MAPK3	0.991671	0.008329	0.897154	0.897116	1394.819	1554.782
BCAP29	0.991673	0.008327	1.175875	1.17592	2666.272	2267.391
NAGK	0.991677	0.008323	0.998122	0.998121	982.4861	984.3356
POP4	0.991677	0.008323	0.908434	0.908397	1288.124	1418.019
IVNS1ABF	0.991678	0.008322	0.782487	0.782472	6749.59	8625.983
CHMP2A	0.991679	0.008321	0.836586	0.836557	2679.608	3203.14
ARMCX6	0.991679	0.008321	1.038495	1.038517	1050.282	1011.328
BCL2L12	0.99168	0.00832	0.965475	0.965456	1010.271	1046.419
API5	0.991682	0.008318	0.829067	0.82904	2987.469	3603.532
ATF7	0.991683	0.008317	0.983763	0.983754	986.9317	1003.231
ARFGEF1	0.991685	0.008315	1.246673	1.246703	5797.112	4649.951
PITHD1	0.991692	0.008308	1.10139	1.101435	1418.159	1287.554
POLR2J	0.991696	0.008304	0.865851	0.865816	1876.693	2167.545
ERAP1	0.991697	0.008303	0.856032	0.855998	2097.23	2450.042
GTF2A2	0.991698	0.008302	0.921952	0.921917	1193.654	1294.752
TJP2	0.991699	0.008301	1.194976	1.195017	3269.767	2736.165
NKIRAS2	0.9917	0.0083	0.911311	0.911275	1303.683	1430.616
TCEA1	0.991702	0.008298	0.771847	0.771835	8707.894	11282.07
FBXO38	0.991709	0.008291	0.924756	0.924723	1208.102	1306.449
ARHGAP1	0.991709	0.008291	0.972934	0.97292	1005.826	1033.822
SEC61B	0.99171	0.00829	1.153036	1.153083	2121.681	1840.006
RPS6KA3	0.991712	0.008288	1.178145	1.178189	2757.963	2340.847
TAOK3	0.991713	0.008287	1.029163	1.029179	1060.285	1030.223
NT5C3A	0.991715	0.008285	0.955676	0.955653	1044.725	1093.206
LRWD1	0.991716	0.008284	1.048363	1.048389	1122.524	1070.712
GRB10	0.991717	0.008283	0.869038	0.869002	1824.935	2100.036
ZNF598	0.991717	0.008283	0.916991	0.916956	1264.784	1379.329
BRCC3	0.991717	0.008283	1.001598	1.001599	995.823	994.2329
SRCAP	0.991717	0.008283	1.151005	1.151053	2051.662	1782.421
SGPP1	0.991717	0.008283	1.026642	1.026657	1055.839	1028.424
SIPA1L2	0.991718	0.008282	0.884903	0.884866	1538.191	1738.333
NUP160	0.991718	0.008282	0.896521	0.896484	1455.947	1624.064
MRPL18	0.991718	0.008282	1.013888	1.013896	1008.048	994.2329
MFSD3	0.991721	0.008279	1.038917	1.038939	1064.73	1024.825
BTBD1	0.991721	0.008279	1.077384	1.077422	1274.787	1183.182
VRK3	0.991723	0.008277	1.030807	1.030824	1042.502	1011.328
RAB9A	0.991723	0.008277	1.113526	1.113573	1525.965	1370.332
ZBTB1	0.991728	0.008272	1.067436	1.067471	1188.097	1113.001
ERH	0.99173	0.00827	0.831167	0.83114	2937.456	3534.251
OXA1L	0.991732	0.008268	0.921583	0.921549	1208.102	1310.948
DMWD	0.991733	0.008267	0.955282	0.955259	1076.956	1127.397
XYLT2	0.991735	0.008265	1.021599	1.021611	1025.831	1004.13
FUT8	0.991735	0.008265	1.126084	1.126132	1709.348	1517.892
FBLN2	0.991736	0.008264	1.071087	1.071123	1211.436	1130.996
TNKS	0.991737	0.008263	0.916112	0.916077	1279.232	1396.425
NUP43	0.991737	0.008263	1.083917	1.083957	1294.926	1194.627
WDR3	0.991739	0.008261	0.901089	0.901053	1381.482	1533.188
ELF4	0.99174	0.00826	1.155111	1.155158	2180.586	1887.693
PAPD7	0.99174	0.00826	1.008369	1.008374	1032.5	1023.925
THUMPD3	0.991741	0.008259	0.897083	0.897046	1459.281	1626.763
ARL2BP	0.991743	0.008257	1.089602	1.089643	1339.249	1229.07
MYO6	0.991743	0.008257	1.19269	1.192732	3155.047	2645.226
ENTPD4	0.991745	0.008255	1.101464	1.101508	1441.087	1308.285
OAF	0.991746	0.008254	0.946843	0.946817	1083.624	1144.493
SUCLG1	0.991746	0.008254	0.832937	0.83291	2947.458	3538.749
DDX41	0.991748	0.008252	0.854453	0.85442	2169.472	2539.118

SMN1	0.991748	0.008252	0.983427	0.983417	1030.677	1048.056
FSTL1	0.991749	0.008251	1.30454	1.304557	12841.23	9843.356
FAM122B	0.991751	0.008249	1.056444	1.056474	1169.203	1106.703
SLC10A3	0.991752	0.008248	1.043589	1.043613	1071.399	1026.624
FN3KRP	0.991755	0.008245	1.101144	1.101188	1442.61	1310.048
SLC9A6	0.991758	0.008242	1.013147	1.013155	1019.163	1005.93
TYMS	0.991758	0.008242	1.101602	1.101646	1448.167	1314.547
NCAPH	0.991759	0.008241	1.076977	1.077014	1255.893	1166.087
NDUFA12	0.991759	0.008241	0.973356	0.973341	1020.274	1048.218
MCMBP	0.991762	0.008238	1.177064	1.177108	2768.521	2351.968
TBC1D14	0.991762	0.008238	1.074811	1.074848	1273.675	1184.982
PTPN14	0.991768	0.008232	1.121407	1.121454	1666.003	1485.573
ANKHD1-I	0.991769	0.008231	1.043206	1.043229	1094.349	1049.001
ARAF	0.991769	0.008231	0.907693	0.907657	1330.357	1465.706
PNKP	0.991771	0.008229	1.065403	1.065436	1192.542	1119.299
BRD7	0.991772	0.008228	0.824512	0.824487	3364.237	4080.404
EPS15	0.991772	0.008228	0.887701	0.887664	1552.639	1749.13
OTUD6B	0.991773	0.008227	0.919047	0.919013	1240.333	1349.637
HERPUD1	0.991774	0.008226	1.235727	1.235759	5195.84	4204.571
NEK7	0.991775	0.008225	1.187219	1.18726	3080.827	2594.903
COPS4	0.991775	0.008225	1.010838	1.010844	1021.385	1010.429
YIPF3	0.991776	0.008224	1.185868	1.18591	2966.352	2501.328
ERBB3	0.99178	0.00822	1.189185	1.189226	3074.159	2585.006
THRB	0.991781	0.008219	1.002753	1.002755	1040.279	1037.421
RBBP5	0.991783	0.008217	1.078152	1.07819	1303.683	1209.14
EDC3	0.991787	0.008213	0.96907	0.969054	1035.834	1068.913
CEP295	0.991788	0.008212	0.882689	0.882652	1630.438	1847.204
ATXN7	0.991791	0.008209	0.987976	0.987969	1016.94	1029.324
TTC17	0.991792	0.008208	0.855496	0.855464	2170.583	2537.318
ZNF330	0.991795	0.008205	1.10704	1.107084	1517.074	1370.332
DPP9	0.991795	0.008205	1.139359	1.139405	1981.488	1739.053
LRRC47	0.991795	0.008205	0.975083	0.975069	1029.165	1055.479
CCNYL1	0.991798	0.008202	1.11678	1.116825	1624.881	1454.909
UBE4B	0.9918	0.0082	0.857683	0.85765	2120.569	2472.536
CIZ1	0.991801	0.008199	0.817304	0.817282	3707.662	4536.581
TBC1D2B	0.991801	0.008199	0.987352	0.987345	1044.725	1058.116
NDUFV2	0.991806	0.008194	0.876724	0.876689	1733.799	1977.669
TXN2	0.991806	0.008194	0.938842	0.938813	1136.972	1211.075
CLOCK	0.991808	0.008192	0.980555	0.980545	1026.942	1047.319
CDC34	0.991809	0.008191	1.078457	1.078494	1284.789	1191.28
SRP19	0.99181	0.00819	0.889211	0.889175	1588.004	1785.93
DDX52	0.991812	0.008188	1.0462	1.046224	1146.563	1095.906
SH3BGRL	0.991813	0.008187	1.143021	1.143067	2053.885	1796.817
C1orf122	0.991815	0.008185	0.984567	0.984558	1025.831	1041.92
AHCYL2	0.991817	0.008183	1.014873	1.014881	1064.73	1049.118
FAM96B	0.991817	0.008183	0.984447	0.984439	1051.393	1068.013
TBCE	0.991819	0.008181	0.899747	0.899711	1403.71	1560.181
NDUFA4	0.99182	0.00818	0.854565	0.854533	2212.816	2589.504
TRAF7	0.99182	0.00818	1.164434	1.164479	2432.876	2089.239
DLAT	0.991823	0.008177	0.869583	0.869549	1839.383	2115.332
EDEM1	0.991827	0.008173	0.895579	0.895543	1514.851	1691.546
TNFRSF1	0.991829	0.008171	1.039742	1.039763	1129.192	1086.008
SMG5	0.991832	0.008168	1.234943	1.234975	5362.551	4342.234
EDEM2	0.991834	0.008166	0.93114	0.931109	1214.771	1304.65
KCTD15	0.99184	0.00816	1.119591	1.119636	1679.34	1499.897
PTPRG	0.991841	0.008159	0.899841	0.899806	1437.053	1597.071

MTX2	0.991841	0.008159	1.033391	1.03341	1086.959	1051.817
SIRPA	0.991842	0.008158	1.083256	1.083295	1363.611	1258.762
UBE2Z	0.991844	0.008156	0.825551	0.825526	3300.886	3998.526
PPP1R37	0.991847	0.008153	0.915282	0.915248	1297.015	1417.119
TMUB1	0.991848	0.008152	0.938135	0.938106	1182.54	1260.561
TBK1	0.991849	0.008151	0.926869	0.926837	1245.89	1344.239
TMEM126	0.991851	0.008149	0.984076	0.984068	1062.508	1079.71
SNRNP40	0.991854	0.008146	1.022946	1.022959	1065.842	1041.92
CAMTA1	0.991854	0.008146	1.082147	1.082185	1328.134	1227.27
DLD	0.991855	0.008145	0.789612	0.789597	6309.472	7990.754
UBL5	0.991855	0.008145	1.206516	1.206554	3787.684	3139.257
CTTN	0.991858	0.008142	0.806459	0.806439	4521.214	5606.394
ACTR1B	0.991859	0.008141	0.923563	0.92353	1241.445	1344.239
VPRBP	0.99186	0.00814	1.022064	1.022076	1065.842	1042.82
ILVBL	0.991861	0.008139	0.924175	0.924143	1238.11	1339.74
MKL1	0.991862	0.008138	0.981914	0.981904	1042.502	1061.715
NOMO3	0.991864	0.008136	0.82053	0.820507	3524.98	4296.103
ATL2	0.991866	0.008134	1.133996	1.134042	1890.919	1667.414
NLN	0.991867	0.008133	1.035709	1.035728	1102.518	1064.486
NFE2L2	0.991869	0.008131	0.852413	0.852382	2360.634	2769.456
JUP	0.991872	0.008128	1.239389	1.23942	5590.39	4510.488
SMAP1	0.991872	0.008128	1.053437	1.053464	1198.1	1137.294
SLIRP	0.991873	0.008127	0.907787	0.907752	1404.822	1547.584
PPIE	0.991873	0.008127	1.037971	1.037992	1111.41	1070.73
PLEC	0.991876	0.008124	1.323775	1.323788	18039.06	13626.84
PAF1	0.991882	0.008118	0.887839	0.887804	1641.552	1849.003
NUP133	0.991883	0.008117	0.953076	0.953053	1133.638	1189.48
PRKCI	0.991884	0.008116	1.232785	1.232817	5176.946	4199.28
CPSF7	0.991889	0.008111	0.86179	0.861758	2077.225	2410.452
TMEM50B	0.991891	0.008109	1.085127	1.085166	1398.187	1288.454
ITSN2	0.991891	0.008109	1.024607	1.02462	1081.402	1055.416
TFPT	0.991897	0.008103	1.109885	1.109928	1594.873	1436.914
USO1	0.991898	0.008102	0.824121	0.824097	3510.943	4260.356
NDC1	0.991898	0.008102	0.868956	0.868922	1927.184	2217.904
SIVA1	0.991906	0.008094	1.019235	1.019246	1101.407	1080.61
CRB3	0.991906	0.008094	0.958173	0.958152	1099.184	1147.192
MRPL2	0.991908	0.008092	1.003237	1.003239	1081.402	1077.91
GMPPA	0.991909	0.008091	0.937905	0.937877	1205.879	1285.755
YIF1B	0.991913	0.008087	0.961952	0.961933	1091.404	1134.595
DCTPP1	0.991913	0.008087	0.910832	0.910798	1360.365	1493.599
MLF1	0.991914	0.008086	1.1002	1.100241	1503.737	1366.733
XRN2	0.991919	0.008081	1.157368	1.157412	2347.297	2028.055
DBNL	0.99192	0.00808	1.143369	1.143414	2076.113	1815.712
STIM1	0.991925	0.008075	1.1799	1.179942	2917.45	2472.536
IDH2	0.991926	0.008074	1.194513	1.194552	3394.245	2841.437
PHF1	0.991927	0.008073	1.111219	1.111262	1663.78	1497.198
DDX10	0.991928	0.008072	1.059154	1.059184	1216.993	1148.991
WHSC1L1	0.991929	0.008071	0.8601	0.860068	2142.798	2491.431
GLTSCR2	0.99193	0.00807	0.970983	0.970968	1078.067	1110.302
LRRC20	0.991934	0.008066	0.96446	0.964442	1092.516	1132.796
SENP2	0.991936	0.008064	0.921668	0.921636	1283.678	1392.826
YIPF5	0.991937	0.008063	1.198915	1.198953	3569.892	2977.507
PTAR1	0.991944	0.008056	0.977271	0.977259	1073.622	1098.605
SNHG1	0.991945	0.008055	0.933334	0.933305	1243.667	1332.542
SHCBP1	0.991947	0.008053	1.216384	1.216418	4455.641	3662.916
FAR1	0.991949	0.008051	0.859174	0.859143	2231.71	2597.602

MRPS5	0.991951	0.008049	1.13289	1.132934	1969.418	1738.333
SF3B3	0.991952	0.008048	0.84157	0.841542	2712.428	3223.168
E2F1	0.991957	0.008043	0.90891	0.908876	1395.93	1535.887
PKD1P1	0.991958	0.008042	0.813017	0.812996	4164.785	5122.765
MOAP1	0.991959	0.008041	1.06387	1.063901	1283.678	1206.576
TUBGCP3	0.991959	0.008041	1.07257	1.072604	1304.795	1216.473
IDH3G	0.991959	0.008041	0.959405	0.959385	1140.306	1188.581
CAMLG	0.99196	0.00804	0.903719	0.903685	1448.167	1602.515
HMG20B	0.991962	0.008038	1.052749	1.052776	1199.211	1139.094
BRD4	0.991966	0.008034	0.856078	0.856047	2230.599	2605.7
TIA1	0.991968	0.008032	1.076223	1.076259	1309.24	1216.473
PYCR2	0.991968	0.008032	0.942469	0.942443	1176.983	1248.865
ZNF740	0.99197	0.00803	1.063078	1.063108	1283.678	1207.476
GABPA	0.99197	0.00803	1.020577	1.020588	1099.184	1077.011
GPAM	0.991976	0.008024	0.990443	0.990438	1074.733	1085.109
KIAA2026	0.991978	0.008022	1.018695	1.018705	1098.073	1077.91
MYBBP1A	0.99198	0.00802	0.925275	0.925244	1307.018	1412.621
GIT1	0.991982	0.008018	1.136721	1.136765	2001.649	1760.827
POLD2	0.991983	0.008017	0.810699	0.810679	4361.171	5379.655
SUPT4H1	0.991987	0.008013	0.995991	0.995989	1078.067	1082.409
PPP6C	0.991988	0.008012	1.135067	1.135111	1978.309	1742.832
CCNL2	0.991989	0.008011	1.01718	1.017189	1099.184	1080.61
FAM60A	0.99199	0.00801	1.208065	1.208101	4107.77	3400.187
SSH2	0.991993	0.008007	0.97428	0.974267	1120.301	1149.891
ASCC1	0.991995	0.008005	0.919688	0.919656	1321.466	1436.914
PAWR	0.991995	0.008005	1.103566	1.103608	1574.867	1427.017
SETX	0.991997	0.008003	0.879596	0.879562	1830.492	2081.141
CCDC124	0.991998	0.008002	0.868126	0.868094	1968.306	2267.391
EHMT2	0.991998	0.008002	0.882102	0.882068	1746.024	1979.468
TDP2	0.992	0.008	1.101776	1.101816	1597.096	1449.511
PCF11	0.992003	0.007997	0.940864	0.940838	1196.988	1272.258
MRPL49	0.992004	0.007996	1.129893	1.129936	1908.29	1688.846
RNF40	0.992005	0.007995	0.843695	0.843667	2679.608	3176.147
RAB1B	0.992005	0.007995	1.208709	1.208745	4057.756	3356.998
RETSAT	0.992006	0.007994	1.096895	1.096935	1551.528	1414.42
THAP9-AS	0.992007	0.007993	0.935784	0.935757	1253.67	1339.74
FAM73A	0.992008	0.007992	0.951204	0.951181	1184.207	1244.987
RFWD2	0.992012	0.007988	1.125839	1.125882	1853.831	1646.558
NR1D2	0.992016	0.007984	0.877769	0.877735	1828.269	2082.94
VAC14	0.992017	0.007983	1.014896	1.014903	1131.415	1114.801
APPL1	0.992019	0.007981	0.86603	0.865998	2111.967	2438.768
TRAFD1	0.992021	0.007979	1.068743	1.068775	1305.906	1221.872
NUMB	0.992022	0.007978	1.152911	1.152953	2382.862	2066.745
MGAT2	0.992024	0.007976	1.057368	1.057396	1222.551	1156.189
MLLT10	0.992029	0.007971	0.924871	0.92484	1298.126	1403.623
GET4	0.99203	0.00797	1.00814	1.008144	1100.295	1091.407
BAIAP2L1	0.992035	0.007965	1.226518	1.226549	5024.683	4096.6
ACTL6A	0.992038	0.007962	1.146089	1.146132	2201.702	1920.984
LAS1L	0.992039	0.007961	0.90769	0.907657	1478.175	1628.563
APEX2	0.992041	0.007959	1.001155	1.001155	1098.073	1096.805
RAD18	0.992047	0.007953	1.038519	1.038538	1173.649	1130.096
HDAC6	0.992048	0.007952	0.927655	0.927626	1287.012	1387.427
ELAC2	0.992051	0.007949	1.045619	1.045642	1201.434	1148.991
SNHG3	0.992052	0.007948	0.937847	0.93782	1259.894	1343.429
PPP1R7	0.992052	0.007948	0.950755	0.950732	1173.649	1234.468
GAB2	0.992052	0.007948	1.061382	1.061411	1275.898	1202.077

RAB29	0.992054	0.007946	0.952507	0.952485	1168.125	1226.398
NCLN	0.992054	0.007946	0.903714	0.90368	1490.4	1649.257
C8orf59	0.992058	0.007942	0.988191	0.988185	1102.518	1115.7
TM9SF4	0.992059	0.007941	1.222211	1.222243	4811.292	3936.443
TBL1X	0.99206	0.00794	1.108183	1.108224	1697.122	1531.389
BTBD2	0.992061	0.007939	0.979372	0.979362	1110.298	1133.695
SEC16A	0.992063	0.007937	1.195359	1.195396	3568.736	2985.398
CHCHD7	0.992067	0.007933	1.085596	1.085632	1443.721	1329.843
MED25	0.99207	0.00793	0.957641	0.957621	1184.996	1237.438
PSMA2	0.992072	0.007928	1.230169	1.230199	5434.793	4417.813
BET1L	0.992073	0.007927	1.089036	1.089074	1449.278	1330.743
TRMT1L	0.992074	0.007926	0.944973	0.944948	1204.768	1274.958
PRKRA	0.992075	0.007925	0.917356	0.917325	1372.591	1496.298
THUMPD1	0.992076	0.007924	0.945416	0.945391	1232.553	1303.75
TFDP2	0.992076	0.007924	0.853049	0.85302	2492.792	2922.316
SRSF10	0.992078	0.007922	0.853606	0.853577	2477.676	2902.701
EDC4	0.99208	0.00792	0.946027	0.946002	1181.428	1248.865
PNISR	0.992081	0.007919	0.891148	0.891115	1654.889	1857.101
JKAMP	0.992084	0.007916	1.032177	1.032193	1146.975	1111.202
PPP3CA	0.992086	0.007914	0.927056	0.927026	1283.678	1384.728
PUM2	0.992088	0.007912	0.856598	0.856569	2340.629	2732.566
WTAP	0.992088	0.007912	0.894682	0.894649	1611.544	1801.316
EXO1	0.992089	0.007911	1.005276	1.005279	1098.073	1092.307
NUP214	0.992091	0.007909	1.133208	1.133251	1976.086	1743.732
GNPTAB	0.992091	0.007909	0.95068	0.950657	1188.097	1249.764
SYT7	0.992091	0.007909	0.977741	0.97773	1122.524	1148.092
MAP7	0.992093	0.007907	1.018235	1.018244	1136.972	1116.6
RBBP8	0.992094	0.007906	0.935759	0.935732	1257.004	1343.339
NOL8	0.992095	0.007905	0.955919	0.955899	1171.426	1225.471
PIK3R3	0.992096	0.007904	1.011169	1.011175	1153.643	1140.894
ZBED1	0.992098	0.007902	1.00213	1.002131	1118.078	1115.7
PTPN3	0.992099	0.007901	1.078282	1.078316	1435.941	1331.651
KIF21A	0.992099	0.007901	0.867618	0.867587	2077.225	2394.257
OCIAD1	0.992101	0.007899	0.893839	0.893806	1629.326	1822.91
RRP12	0.992103	0.007897	0.89041	0.890376	1647.109	1849.903
PSMB6	0.992103	0.007897	0.875666	0.875633	1916.07	2188.212
TCEB2	0.992105	0.007895	0.902161	0.902129	1568.199	1738.333
STK35	0.992106	0.007894	0.91406	0.914029	1413.713	1546.685
MINPP1	0.992109	0.007891	1.001082	1.001083	1121.412	1120.199
MUT	0.992113	0.007887	0.94426	0.944236	1222.551	1294.752
DDIAS	0.992114	0.007886	1.021319	1.02133	1178.094	1153.49
MRPS2	0.992123	0.007877	0.980673	0.980664	1130.304	1152.59
ORC3	0.992123	0.007877	1.066113	1.066143	1331.469	1248.865
RHBDF2	0.992125	0.007875	1.086214	1.08625	1474.841	1357.735
SYAP1	0.992125	0.007875	1.160238	1.160279	2627.372	2264.431
RNH1	0.992131	0.007869	0.861001	0.860971	2255.05	2619.196
GCNT1	0.992133	0.007867	1.025112	1.025124	1165.869	1137.294
YTHDC1	0.992134	0.007866	0.894921	0.894889	1671.56	1867.898
PPARA	0.992138	0.007862	1.102474	1.102514	1634.017	1482.082
GADD45G	0.992144	0.007856	1.041821	1.041841	1221.439	1172.385
C9orf16	0.992149	0.007851	1.104114	1.104154	1657.112	1500.797
PRRC2C	0.99215	0.00785	0.793933	0.793918	6336.146	7980.856
MAP7D3	0.992152	0.007848	1.109647	1.109688	1721.34	1551.192
S100A10	0.992153	0.007847	1.230701	1.230731	5471.469	4445.706
CDC42SE	0.992154	0.007846	1.126293	1.126334	1942.744	1724.837
CENPBD1	0.992158	0.007842	0.970275	0.97026	1157.955	1193.448

RIPK4	0.992159	0.007841	0.99032	0.990315	1136.972	1148.092
AHR	0.992161	0.007839	1.066658	1.066688	1350.385	1265.96
ELMO2	0.992165	0.007835	1.087031	1.087067	1500.403	1380.229
NIFK	0.992166	0.007834	0.922618	0.922589	1403.71	1521.491
SLC25A44	0.992167	0.007833	1.035074	1.035091	1235.887	1193.988
NOSIP	0.992168	0.007832	1.167602	1.167642	2784.081	2384.36
HERPUD2	0.992171	0.007829	1.065763	1.065792	1349.251	1265.96
TSC22D2	0.992172	0.007828	0.992635	0.992631	1141.418	1149.891
BACE1	0.992176	0.007824	1.048584	1.048607	1261.45	1202.977
ZWINT	0.992178	0.007822	0.88969	0.889658	1768.253	1987.566
EFHD1	0.992178	0.007822	1.029025	1.029039	1192.542	1158.889
TSR2	0.992179	0.007821	0.966758	0.966743	1172.537	1212.874
KIAA1715	0.992179	0.007821	0.963596	0.963579	1180.317	1224.931
WDHD1	0.992183	0.007817	1.015131	1.015139	1163.646	1146.292
GNG12	0.992184	0.007816	1.283101	1.28312	10809.57	8424.437
NELFCD	0.992184	0.007816	0.991073	0.991069	1145.863	1156.189
LAMTOR2	0.992187	0.007813	0.945388	0.945364	1275.898	1349.637
HS6ST1	0.992189	0.007811	1.038591	1.03861	1226.996	1181.383
DYM	0.992192	0.007808	0.919026	0.918996	1443.721	1570.978
HSBP1	0.992193	0.007807	1.156123	1.156164	2584.027	2235
DIAPH3	0.992194	0.007806	1.141628	1.141669	2278.39	1995.664
CASP2	0.992194	0.007806	1.070807	1.070838	1424.827	1330.572
CTDSPL	0.992195	0.007805	0.899515	0.899483	1604.875	1784.221
TMEM161	0.992197	0.007803	0.949429	0.949407	1262.561	1329.843
CEP164	0.992197	0.007803	0.910776	0.910745	1484.843	1630.362
PAK1IP1	0.992199	0.007801	0.914334	0.914304	1488.177	1627.663
DPH3	0.992209	0.007791	0.965758	0.965743	1214.771	1257.862
GOSR1	0.992212	0.007788	0.89796	0.897928	1603.975	1786.308
SPATS2	0.992213	0.007787	0.918566	0.918536	1457.247	1586.49
H1FX	0.992215	0.007785	0.980004	0.979995	1143.64	1166.987
SENP3	0.99222	0.00778	0.898405	0.898373	1631.016	1815.523
PELI1	0.992222	0.007778	1.043473	1.043494	1258.116	1205.676
NCK1	0.992223	0.007777	1.080181	1.080215	1471.506	1362.234
NDUFA1	0.992226	0.007774	1.151339	1.151379	2441.767	2120.73
CTSF	0.992228	0.007772	1.032164	1.032179	1220.328	1182.282
RPAP3	0.992228	0.007772	0.970163	0.970149	1183.651	1220.072
DHX40	0.992228	0.007772	0.944941	0.944917	1294.603	1370.071
SBNO1	0.99223	0.00777	0.915764	0.915733	1428.717	1560.19
LINC0129	0.992231	0.007769	0.924195	0.924166	1420.537	1537.102
TECR	0.992233	0.007767	1.188928	1.188965	3598.744	3026.787
PRKCH	0.992235	0.007765	1.038614	1.038633	1244.779	1198.478
UPF2	0.992239	0.007761	1.011654	1.01166	1206.991	1193.08
DCAF10	0.992244	0.007756	0.889483	0.889451	1764.896	1984.255
SOGA1	0.992246	0.007754	1.108015	1.108054	1752.693	1581.775
CLUH	0.992246	0.007754	0.892817	0.892786	1718.239	1924.583
C14orf119	0.992248	0.007752	0.955676	0.955656	1208.102	1264.16
ZNF638	0.992249	0.007751	0.85516	0.855132	2562.91	2997.095
EEF1B2	0.992251	0.007749	0.810167	0.810149	4770.17	5888.018
TPD52L1	0.992253	0.007747	1.124563	1.124603	1977.198	1758.128
HIVEP1	0.992253	0.007747	1.061287	1.061314	1390.373	1310.048
C14orf1	0.992258	0.007742	1.009432	1.009436	1182.54	1171.485
LAP3	0.992261	0.007739	1.066877	1.066906	1394.819	1307.349
JAK1	0.992262	0.007738	1.270657	1.270678	9362.514	7368.121
TMEM184	0.992266	0.007734	1.044896	1.044916	1281.455	1226.371
NDUFB9	0.992266	0.007734	1.031668	1.031683	1233.665	1195.779
SERPINB6	0.992267	0.007733	1.174157	1.174195	3057.488	2603.901

TBC1D10I	0.992268	0.007732	0.924483	0.924455	1434.83	1552.083
TRAM2	0.992268	0.007732	0.902261	0.90223	1609.321	1783.717
UBE2L3	0.992269	0.007731	0.855674	0.855646	2562.91	2995.295
SNRPN	0.992271	0.007729	1.087805	1.08784	1557.907	1432.109
ACTR1A	0.992275	0.007725	1.15908	1.159119	2657.38	2292.584
NFAT5	0.992275	0.007725	0.94621	0.946188	1277.01	1349.637
PLCG2	0.992278	0.007722	0.959404	0.959386	1229.219	1281.256
WDR5	0.992282	0.007718	0.953315	0.953295	1281.455	1344.239
PGAM5	0.992283	0.007717	0.968068	0.968054	1208.48	1248.361
C20orf27	0.992283	0.007717	0.946158	0.946135	1280.344	1353.236
MTR	0.992284	0.007716	0.936596	0.936571	1329.246	1419.27
TMEM33	0.992286	0.007714	0.916933	0.916903	1469.317	1602.479
GGNBP2	0.992286	0.007714	0.888025	0.887993	1810.486	2038.852
HEBP2	0.992287	0.007713	1.048943	1.048966	1308.129	1247.065
DMXL1	0.992287	0.007713	0.918733	0.918704	1454.835	1583.575
ZNF525	0.992288	0.007712	1.052857	1.052881	1327.145	1260.489
FOXJ3	0.992291	0.007709	0.869058	0.869029	2171.183	2498.404
KIAA1279	0.992294	0.007706	1.095371	1.095407	1680.451	1534.088
NUP93	0.992299	0.007701	1.116014	1.116053	1883.839	1687.947
RPA1	0.9923	0.0077	1.172725	1.172763	3046.374	2597.602
TSC22D4	0.992302	0.007698	1.144531	1.144571	2357.3	2059.547
SHTN1	0.992302	0.007698	1.072163	1.072193	1483.732	1383.828
MDC1	0.992307	0.007693	0.899341	0.89931	1704.902	1895.791
IDH3B	0.992308	0.007692	0.911104	0.911074	1533.745	1683.448
IPO7	0.992308	0.007692	1.25892	1.258942	8143.298	6468.362
RFC2	0.992308	0.007692	0.960642	0.960624	1267.107	1319.046
CEP250	0.992308	0.007692	0.934319	0.934293	1352.585	1447.711
RAB21	0.99231	0.00769	0.843785	0.843759	2937.733	3481.723
ATG13	0.992313	0.007687	0.92925	0.929223	1387.05	1492.699
NUP58	0.992313	0.007687	1.114048	1.114087	1911.624	1715.866
BACH1	0.992317	0.007683	1.065924	1.065952	1413.713	1326.244
DYNLL2	0.992318	0.007682	0.871179	0.87115	2193.923	2518.423
SLC25A1	0.992318	0.007682	1.122228	1.122267	2031.657	1810.314
KLF13	0.992319	0.007681	0.912942	0.912913	1558.196	1706.842
WIPF2	0.992323	0.007677	0.96443	0.964415	1262.561	1309.148
GNL2	0.992326	0.007674	1.155752	1.155791	2619.592	2266.491
DMTF1	0.992326	0.007674	0.922631	0.922603	1475.952	1599.77
EMC4	0.992331	0.007669	1.052275	1.052298	1342.583	1275.857
RPF1	0.992333	0.007667	0.973718	0.973706	1195.877	1228.17
ZER1	0.992336	0.007664	1.052789	1.052813	1347.028	1279.456
CDK2AP1	0.992336	0.007664	1.079908	1.07994	1522.631	1409.921
HNRNPLL	0.992339	0.007661	0.958212	0.958194	1257.004	1311.848
TCEB3	0.99234	0.00766	1.127396	1.127435	2127.238	1886.793
HIPK2	0.992341	0.007659	0.986192	0.986185	1205.879	1222.772
NCOR1	0.992343	0.007657	0.896486	0.896455	1720.462	1919.184
NAA10	0.992343	0.007657	0.8398	0.839776	3075.27	3662.016
LAMB3	0.992344	0.007656	1.15143	1.151469	2597.364	2255.694
SNRPD2	0.992346	0.007654	1.218505	1.218535	5029.128	4127.191
FAM3A	0.992348	0.007652	1.004017	1.004019	1240.333	1235.368
BMPR1A	0.992349	0.007651	0.932645	0.932619	1380.371	1480.102
WASL	0.992349	0.007651	0.85669	0.856663	2535.125	2959.305
CHD7	0.99235	0.00765	1.052691	1.052714	1352.585	1284.855
CYC1	0.992356	0.007644	0.807183	0.807166	5184.726	6423.375
SRF	0.992356	0.007644	0.870257	0.870228	2241.713	2576.008
OTUB1	0.992357	0.007643	0.934942	0.934916	1345.917	1439.613
C11orf73	0.992357	0.007643	0.97725	0.97724	1250.336	1279.456

ATXN1L	0.992358	0.007642	0.994213	0.994211	1210.325	1217.373
SV2A	0.992362	0.007638	0.8973	0.89727	1761.584	1963.273
RCN1	0.992363	0.007637	1.210812	1.210844	4745.719	3919.347
TOX4	0.992366	0.007634	0.918424	0.918395	1530.411	1666.397
EEF1E1	0.992368	0.007632	0.875071	0.875041	2092.662	2391.504
AMD1	0.99237	0.00763	1.150386	1.150425	2532.902	2201.709
HACD2	0.99237	0.00763	0.945784	0.945762	1318.132	1393.726
MXRA7	0.992374	0.007626	1.282341	1.282359	11333.39	8837.921
ZXDC	0.992374	0.007626	0.967781	0.967767	1274.787	1317.246
ERLIN1	0.992374	0.007626	0.883497	0.883467	1937.187	2192.711
KMT2B	0.992375	0.007625	0.942948	0.942925	1333.691	1414.42
EDRF1	0.992375	0.007625	0.930872	0.930846	1403.71	1507.995
ITM2B	0.992378	0.007622	1.256502	1.256525	8263.33	6576.333
VASN	0.992379	0.007621	0.948447	0.948425	1341.471	1414.42
UCK2	0.992379	0.007621	1.115154	1.115192	1920.516	1722.137
GSDMD	0.99238	0.00762	0.987112	0.987106	1220.328	1236.268
EAF1	0.992382	0.007618	1.070612	1.070641	1474.841	1377.53
GRK6	0.992385	0.007615	0.876943	0.876913	2116.124	2413.152
PSMB5	0.992387	0.007613	1.130314	1.130352	2156.135	1907.488
IMMP2L	0.992388	0.007612	0.976072	0.976061	1234.776	1265.06
SRSF2	0.992388	0.007612	0.831721	0.831699	3639.866	4376.424
TRMT112	0.99239	0.00761	0.888549	0.888519	1862.722	2096.437
TMEM8A	0.992393	0.007607	0.946619	0.946597	1355.92	1432.415
CAPN1	0.992394	0.007606	1.2288	1.228828	5759.324	4686.841
VBP1	0.992397	0.007603	1.107643	1.10768	1833.826	1655.555
PLEKHA2	0.992397	0.007603	1.071545	1.071574	1488.544	1389.119
MTHFD1	0.992398	0.007602	0.829802	0.82978	3664.317	4416.014
RAPH1	0.992398	0.007602	0.887185	0.887154	1889.396	2129.728
ATP5J	0.9924	0.0076	0.826675	0.826653	3773.236	4564.474
CKAP2	0.992401	0.007599	1.139799	1.139838	2337.294	2050.549
DDX19A	0.992402	0.007598	1.043722	1.043742	1365.211	1307.997
HOOK1	0.992406	0.007594	0.856953	0.856926	2572.913	3002.493
TRAF4	0.992409	0.007591	0.956379	0.95636	1322.577	1382.929
CDK5RAP	0.992413	0.007587	1.099655	1.099691	1749.359	1590.773
LCMT1	0.992418	0.007582	1.030233	1.030247	1290.347	1252.464
PPP2R5E	0.992423	0.007577	1.141967	1.142006	2396.199	2098.236
THAP5	0.992423	0.007577	0.941045	0.941022	1397.042	1484.601
PRMT5	0.992423	0.007577	0.891512	0.891482	1836.049	2059.547
FOPNL	0.992423	0.007577	1.162495	1.162533	2872.994	2471.321
FAM111A	0.992425	0.007575	1.044711	1.044731	1347.028	1289.354
CDC42EP	0.992426	0.007574	0.982738	0.98273	1241.445	1263.261
PSPH	0.992426	0.007574	0.894621	0.894591	1792.103	2003.267
ACBD6	0.992428	0.007572	0.93061	0.930585	1429.273	1535.887
ZYG11B	0.992428	0.007572	1.032137	1.032151	1331.802	1290.316
TOMM6	0.992429	0.007571	1.066727	1.066754	1505.96	1411.721
H2AFX	0.99243	0.00757	1.017557	1.017565	1262.561	1240.767
ELMSAN1	0.992431	0.007569	1.024153	1.024164	1278.121	1247.965
UBE3A	0.992432	0.007568	1.113084	1.113121	1924.961	1729.335
MCM8	0.992433	0.007567	0.9295	0.929474	1439.275	1548.484
SLC16A7	0.992434	0.007566	0.952175	0.952155	1349.54	1417.353
DDX47	0.992434	0.007566	1.085076	1.085108	1654.889	1525.09
XRN1	0.99244	0.00756	1.091279	1.091313	1677.117	1536.787
WSB2	0.99244	0.00756	0.92018	0.920152	1551.528	1686.165
CTDNEP1	0.992442	0.007558	0.948319	0.948299	1337.026	1409.921
SAV1	0.992444	0.007556	1.104745	1.104782	1826.046	1652.856
RNF20	0.992446	0.007554	1.018775	1.018783	1302.572	1278.557

LSM5	0.992447	0.007553	0.993392	0.993389	1223.662	1231.805
NPAS2	0.992448	0.007552	0.952912	0.952893	1320.355	1385.628
ADGRE5	0.992448	0.007552	1.07312	1.073149	1524.354	1420.448
ARL5B	0.992449	0.007551	0.991905	0.991901	1245.89	1256.063
UFM1	0.992449	0.007551	1.120401	1.120439	2039.437	1820.211
CLASP1	0.992449	0.007551	0.956065	0.956047	1309.24	1369.432
BCOR	0.992456	0.007544	0.90909	0.909061	1629.326	1792.319
C6orf89	0.992457	0.007543	0.868597	0.868568	2298.395	2646.189
DHX29	0.992458	0.007542	0.901898	0.901868	1683.786	1866.998
WBSCR22	0.992464	0.007536	0.962127	0.962111	1295.904	1346.938
SERPINA1	0.992464	0.007536	0.927327	0.927301	1469.283	1584.474
BLCAP	0.992465	0.007535	1.146434	1.146471	2584.027	2253.895
TOMM40	0.992465	0.007535	0.845072	0.845047	3107.501	3677.312
DOPEY2	0.992466	0.007534	1.026148	1.02616	1298.126	1265.033
ZNF512B	0.992468	0.007532	0.902952	0.902923	1676.006	1856.201
USP32	0.99247	0.00753	0.891586	0.891556	1862.6	2089.158
DAXX	0.992471	0.007529	0.961574	0.961558	1300.349	1352.337
PAPSS1	0.992472	0.007528	1.011189	1.011194	1299.238	1284.855
PRDM2	0.992473	0.007527	0.971942	0.97193	1275.898	1312.747
MEGF8	0.992473	0.007527	1.168218	1.168254	3080.827	2637.12
KPNA4	0.992474	0.007526	1.246401	1.246425	7492.068	6010.844
CTBP2	0.992475	0.007525	0.839315	0.839292	3364.237	4008.423
ARL6IP1	0.992476	0.007524	1.258873	1.258894	8573.414	6810.271
TSPYL1	0.992477	0.007523	0.988464	0.988459	1258.393	1273.086
FLAD1	0.992482	0.007518	0.925075	0.925048	1494.846	1615.966
MMP24	0.992484	0.007516	1.004065	1.004067	1264.784	1259.662
EIF3J	0.992484	0.007516	0.850502	0.850477	2918.562	3431.678
DESI2	0.992489	0.007511	1.219244	1.219273	5434.793	4457.403
DCTD	0.99249	0.00751	0.997787	0.997786	1293.681	1296.552
PEPD	0.992496	0.007504	0.89762	0.897591	1790.481	1994.764
ZMYM3	0.992498	0.007502	0.894396	0.894366	1837.16	2054.148
NCSTN	0.992498	0.007502	1.211775	1.211805	4886.868	4032.717
CHMP2B	0.9925	0.0075	0.901703	0.901674	1781.59	1975.869
C22orf29	0.992505	0.007495	1.078675	1.078706	1596.595	1480.102
PPP4R2	0.992505	0.007495	1.033353	1.033368	1337.026	1293.852
HIP1	0.992505	0.007495	1.02999	1.030004	1326.056	1287.428
BAHD1	0.992505	0.007495	1.019602	1.019611	1298.126	1273.158
BCAT2	0.992511	0.007489	1.10524	1.105275	1871.614	1693.345
RPL14	0.992512	0.007488	1.280414	1.280431	11523.09	8999.383
WBP11	0.992517	0.007483	0.86122	0.861193	2538.459	2947.608
SNX1	0.992518	0.007482	1.079081	1.079111	1645.742	1525.09
ICE1	0.992518	0.007482	0.908323	0.908294	1670.449	1839.106
NUDT5	0.992519	0.007481	0.907668	0.90764	1678.228	1849.003
CLDN12	0.992519	0.007481	1.116387	1.116423	2026.1	1814.813
ZC3H7A	0.992522	0.007478	0.954097	0.954078	1380.371	1446.811
BRWD3	0.992522	0.007478	0.942325	0.942303	1401.487	1487.3
ARRB1	0.992523	0.007477	1.054443	1.054465	1439.275	1364.933
RACGAP1	0.992524	0.007476	1.186804	1.186837	3764.222	3171.639
EARS2	0.992525	0.007475	1.02561	1.025621	1321.466	1288.454
YLPM1	0.992526	0.007474	1.095112	1.095146	1763.807	1610.567
RCOR1	0.992528	0.007472	1.119969	1.120006	2085.004	1861.6
ZNF644	0.992529	0.007471	1.102757	1.102792	1897.176	1720.338
SEC11A	0.992529	0.007471	1.110596	1.110632	1951.635	1757.228
SHMT1	0.992539	0.007461	0.890603	0.890575	1967.195	2208.907
ATF3	0.992542	0.007458	0.942816	0.942794	1408.156	1493.599
CNOT3	0.992543	0.007457	0.862523	0.862497	2522.9	2925.114

MMS19	0.992549	0.007451	0.883171	0.883142	2018.32	2285.386
E2F3	0.992549	0.007451	0.882135	0.882106	2073.89	2351.068
RUSC1	0.992555	0.007445	1.052417	1.052439	1478.175	1404.523
LARP4	0.992558	0.007442	0.845629	0.845605	3101.944	3668.315
NEK9	0.992558	0.007442	0.964867	0.964852	1360.365	1409.921
LUC7L2	0.992559	0.007441	0.952042	0.952023	1372.369	1441.53
BOD1L1	0.992562	0.007438	1.024514	1.024525	1334.803	1302.85
TPGS2	0.992565	0.007435	0.890272	0.890243	1941.633	2181.014
GRPEL2	0.992566	0.007434	0.887308	0.887279	2040.548	2299.782
NOP16	0.992567	0.007433	1.045366	1.045385	1413.713	1352.337
ERGIC2	0.992568	0.007432	0.917392	0.917365	1603.764	1748.23
SH2B1	0.992571	0.007429	1.032292	1.032306	1362.588	1319.945
GAPVD1	0.992572	0.007428	1.057156	1.057179	1478.175	1398.224
TMEM237	0.992573	0.007427	0.959382	0.959366	1383.705	1442.313
MAP2K2	0.992574	0.007426	0.86765	0.867622	2410.647	2778.454
UTP4	0.992578	0.007422	0.924457	0.924432	1547.082	1673.55
RABGGTE	0.992582	0.007418	1.085222	1.085253	1737.133	1600.67
RABAC1	0.992583	0.007417	1.067909	1.067936	1553.751	1454.909
CIC	0.992586	0.007414	1.109801	1.109836	1977.198	1781.521
TRAPPC1	0.992587	0.007413	0.883899	0.883871	2066.11	2337.572
ASAP2	0.992589	0.007411	1.07285	1.072878	1593.761	1485.501
ROCK2	0.992591	0.007409	1.036815	1.03683	1388.151	1338.84
MBD4	0.992592	0.007408	0.891459	0.891431	1939.41	2175.616
FXR1	0.992592	0.007408	1.197468	1.1975	4303.378	3593.635
PSMD6	0.992593	0.007407	0.885222	0.885193	2046.105	2311.479
NDUFA10	0.992594	0.007406	0.915128	0.915101	1639.329	1791.419
CCDC88C	0.992596	0.007404	0.924632	0.924606	1554.862	1681.648
IPO8	0.992597	0.007403	1.029631	1.029644	1341.471	1302.85
CHPF	0.992602	0.007398	0.986853	0.986847	1311.463	1328.943
CITED2	0.992605	0.007395	0.987021	0.987015	1312.575	1329.843
RINT1	0.992605	0.007395	1.077923	1.077952	1614.878	1498.098
RNF216	0.992611	0.007389	0.953819	0.953801	1388.44	1455.692
LLPH	0.992611	0.007389	0.948763	0.948743	1410.212	1486.401
CENPB	0.992612	0.007388	1.037578	1.037594	1400.376	1349.637
KLHDC10	0.992614	0.007386	1.143107	1.143143	2635.152	2305.181
CMPK1	0.992615	0.007385	1.219839	1.219867	5559.271	4557.276
CSNK1G3	0.992617	0.007383	1.097803	1.097836	1847.163	1682.548
STRN	0.992617	0.007383	0.874012	0.873985	2250.604	2575.108
HEXIM1	0.992618	0.007382	1.212944	1.212972	5269.193	4344.033
AP2A2	0.992618	0.007382	0.923838	0.923812	1572.645	1702.343
SH3BP2	0.992621	0.007379	1.052189	1.05221	1509.105	1434.224
MAGED2	0.992623	0.007377	1.219888	1.219915	5577.053	4571.672
CD2BP2	0.992624	0.007376	0.890416	0.890388	1977.198	2220.603
XPR1	0.992626	0.007374	0.858518	0.858493	2711.017	3157.882
MRPL19	0.992628	0.007372	0.901866	0.901838	1854.943	2056.847
DESI1	0.992633	0.007367	1.10225	1.102284	1955.859	1774.368
SLC25A32	0.99264	0.00736	0.963339	0.963324	1401.532	1454.891
NDUFA5	0.99264	0.00736	1.027802	1.027813	1354.808	1318.146
URGCP	0.992643	0.007357	1.01283	1.012835	1376.447	1359.004
NOP10	0.992645	0.007355	0.983435	0.983428	1332.58	1355.036
YBX3	0.992646	0.007354	0.863133	0.863107	2590.696	3001.594
KIF2A	0.992652	0.007348	1.008741	1.008744	1341.471	1329.843
UBTD2	0.992654	0.007346	1.087247	1.087278	1756.027	1615.066
RFK	0.992654	0.007346	1.132566	1.132602	2391.753	2111.733
EPG5	0.992659	0.007341	1.053961	1.053982	1538.191	1459.408
KDM3B	0.992664	0.007336	0.847279	0.847256	3143.066	3709.703

ITPK1	0.992665	0.007335	0.872046	0.872019	2372.859	2721.112
OGFOD1	0.992667	0.007333	0.958946	0.95893	1394.819	1454.558
LRP6	0.992667	0.007333	0.990163	0.990159	1338.137	1351.437
EPB41L1	0.992668	0.007332	0.898993	0.898966	1919.404	2135.126
C6orf48	0.992668	0.007332	1.053536	1.053557	1540.414	1462.107
DNAJC7	0.992672	0.007328	0.914278	0.914252	1690.454	1849.003
MED8	0.992673	0.007327	1.063269	1.063294	1567.088	1473.804
KIAA1804	0.992678	0.007322	1.007111	1.007114	1383.705	1373.931
CCNL1	0.992679	0.007321	0.999752	0.999752	1344.806	1345.139
MAGED1	0.992679	0.007321	1.207843	1.207871	4955.775	4102.898
SAFB	0.99268	0.00732	0.944174	0.944154	1465.727	1552.425
MRPL51	0.992682	0.007318	0.921978	0.921953	1661.557	1802.216
ACO2	0.992685	0.007315	0.835767	0.835746	3687.657	4412.415
MOB1A	0.992686	0.007314	1.21193	1.211958	5324.763	4393.52
RABGAP1	0.992687	0.007313	1.124848	1.124883	2277.278	2024.456
WIPI1	0.99269	0.00731	0.957566	0.95755	1410.379	1472.904
BCKDK	0.992692	0.007308	0.913602	0.913576	1679.34	1838.206
SSH1	0.992694	0.007306	0.935784	0.935761	1559.308	1666.352
MRPS33	0.992695	0.007305	1.060902	1.060925	1562.642	1472.904
PLGRKT	0.992695	0.007305	1.025541	1.025552	1397.042	1362.234
FUCA2	0.992696	0.007304	0.985504	0.985498	1385.928	1406.322
BRD8	0.992697	0.007303	1.021893	1.021902	1421.493	1391.026
PLEKHA8	0.992698	0.007302	0.951228	0.951209	1439.964	1513.825
TBC1D5	0.992698	0.007302	1.042639	1.042657	1498.18	1436.887
MAVS	0.992699	0.007301	0.850478	0.850455	3054.798	3591.961
COA1	0.992699	0.007301	1.068295	1.06832	1656.056	1550.149
C20orf24	0.992703	0.007297	1.201987	1.202016	4682.558	3895.585
ACP1	0.992704	0.007296	1.117444	1.117479	2130.572	1906.588
RASAL2	0.992706	0.007294	1.116734	1.116768	2158.357	1932.681
TANC1	0.99271	0.00729	0.921132	0.921107	1684.897	1829.209
GLG1	0.992711	0.007289	1.143	1.143035	2652.912	2320.936
AATF	0.992716	0.007284	0.89725	0.897223	1929.407	2150.422
LOC64285	0.992717	0.007283	0.967354	0.967341	1392.596	1439.613
CSRP2	0.992717	0.007283	1.107923	1.107957	2002.76	1807.614
F2RL1	0.992718	0.007282	1.069733	1.069759	1638.218	1531.389
PLEKHG3	0.99272	0.00728	0.986101	0.986096	1363.7	1382.929
CYSTM1	0.992721	0.007279	0.895648	0.895621	1922.739	2146.823
CREB1	0.992724	0.007276	0.990439	0.990436	1363.722	1376.891
KEAP1	0.992725	0.007275	0.932248	0.932225	1562.642	1676.25
WDR46	0.992732	0.007268	0.923469	0.923444	1635.995	1771.624
SMC1A	0.992735	0.007265	0.847198	0.847175	3219.754	3800.579
CDKN2B	0.992736	0.007264	1.086988	1.087018	1801.595	1657.373
AGTRAP	0.992736	0.007264	0.977802	0.977793	1380.371	1411.721
RTFDC1	0.992738	0.007262	1.174353	1.174385	3560.956	3032.185
STRA13	0.99274	0.00726	1.044639	1.044657	1492.623	1428.816
TMA7	0.992741	0.007259	1.090509	1.09054	1809.375	1659.154
UMPS	0.992743	0.007257	1.050768	1.050788	1525.965	1452.21
NXN	0.992746	0.007254	1.164846	1.164879	3259.32	2797.987
LETM1	0.992749	0.007251	1.000937	1.000938	1377.048	1375.757
CC2D1A	0.992749	0.007251	0.908252	0.908226	1802.706	1984.867
PELP1	0.992751	0.007249	0.929851	0.929828	1593.761	1714.04
SF3A3	0.992752	0.007248	1.136714	1.136749	2552.908	2245.797
C16orf72	0.992753	0.007247	0.973317	0.973307	1395.93	1434.215
TMEM248	0.992753	0.007247	1.06654	1.066565	1672.671	1568.279
TRIM38	0.992754	0.007246	0.905291	0.905264	1887.24	2084.74
NDUFA7	0.992757	0.007243	1.00156	1.001561	1381.482	1379.329

PEF1	0.992757	0.007243	1.077588	1.077616	1724.908	1600.67
RANBP3	0.992758	0.007242	1.037453	1.037468	1469.283	1416.22
DNAJC9	0.99276	0.00724	1.060961	1.060984	1597.096	1505.296
CD320	0.99276	0.00724	1.073934	1.073961	1694.9	1578.176
NPTN	0.992774	0.007226	1.126593	1.126627	2375.082	2108.134
NIN	0.992776	0.007224	1.09361	1.09364	1897.176	1734.734
MID1	0.992782	0.007218	1.060529	1.060552	1605.987	1514.293
SMPD4	0.992783	0.007217	1.060677	1.060699	1646.475	1552.254
GMNN	0.992786	0.007214	0.911179	0.911153	1790.481	1965.072
CDC123	0.992787	0.007213	1.068321	1.068346	1664.892	1558.381
ERF	0.992788	0.007212	1.026716	1.026727	1444.832	1407.222
BRK1	0.992788	0.007212	1.192596	1.192626	4355.614	3652.119
SPAG9	0.992789	0.007211	0.881367	0.881341	2313.955	2625.495
WRNIP1	0.992789	0.007211	1.164523	1.164556	3296.441	2830.64
CWC15	0.99279	0.00721	1.008444	1.008447	1438.164	1426.117
PHB	0.992792	0.007208	0.839377	0.839356	3723.222	4435.808
CEP120	0.992792	0.007208	1.005567	1.005569	1401.487	1393.726
DDA1	0.992794	0.007206	1.070646	1.070671	1728.242	1614.166
RNASEK	0.992797	0.007203	0.949036	0.949018	1498.502	1579.004
PTPRK	0.992806	0.007194	1.005076	1.005078	1441.498	1434.215
PSMD11	0.992808	0.007192	0.896346	0.89632	2051.662	2288.985
SIPA1L1	0.992813	0.007187	0.912494	0.912469	1834.937	2010.96
HMMR	0.992814	0.007186	0.954937	0.954921	1480.398	1550.284
PIGK	0.992818	0.007182	0.883845	0.883818	2230.599	2523.822
FCF1	0.99282	0.00718	0.978484	0.978475	1452.646	1484.601
PAK2	0.992824	0.007176	1.136837	1.13687	2681.587	2358.743
TDRKH	0.992826	0.007174	1.026438	1.026448	1437.053	1400.024
PRDX2	0.99283	0.00717	0.813547	0.813531	5418.122	6660.011
DSCR3	0.992834	0.007166	0.886735	0.886709	2239.49	2525.622
ATAD2	0.992834	0.007166	1.167972	1.168005	3456.484	2959.305
PLCB3	0.992834	0.007166	1.0836	1.083628	1827.157	1686.147
SIRT2	0.992835	0.007165	0.978365	0.978357	1460.392	1492.699
RAB5A	0.992842	0.007158	1.138465	1.138499	2668.494	2343.87
GOLGA7	0.992844	0.007156	0.99913	0.99913	1420.381	1421.618
ATP6V0D1	0.992845	0.007155	0.947824	0.947805	1502.626	1585.374
TCERG1	0.992846	0.007154	1.161493	1.161526	3345.343	2880.126
ANAPC11	0.992848	0.007152	0.921361	0.921338	1761.584	1911.986
LRRC42	0.99285	0.00715	1.088383	1.088412	1853.831	1703.242
NAE1	0.992851	0.007149	1.010853	1.010857	1437.053	1421.618
CDKAL1	0.992853	0.007147	0.985007	0.985001	1426.883	1448.611
ATN1	0.992853	0.007147	1.115428	1.115461	2246.159	2013.659
DROSHA	0.992854	0.007146	0.926908	0.926885	1673.783	1805.815
ZNF761	0.992854	0.007146	1.086511	1.08654	1914.392	1761.916
TUBG1	0.992854	0.007146	0.915358	0.915333	1784.735	1949.821
GFRA1	0.992857	0.007143	1.105051	1.105083	2095.007	1895.791
PES1	0.992858	0.007142	0.898331	0.898305	2006.094	2233.2
SPTBN1	0.992861	0.007139	1.220183	1.220208	6048.291	4956.769
VPS13D	0.992866	0.007134	1.055515	1.055535	1658.223	1570.978
RAB34	0.992872	0.007128	1.072874	1.072899	1750.47	1631.532
PTPN23	0.992872	0.007128	0.969586	0.969574	1435.941	1481.002
MAGT1	0.992875	0.007125	0.837335	0.837315	3841.776	4588.21
OSTC	0.992876	0.007124	0.913216	0.913191	1821.6	1994.764
EIF4E	0.992879	0.007121	0.992841	0.992838	1470.395	1481.002
EDF1	0.992882	0.007118	0.870676	0.870651	2585.139	2969.202
ADAM10	0.992883	0.007117	1.182748	1.182778	4059.979	3432.578
NDUFB2	0.992883	0.007117	0.879623	0.879597	2369.525	2693.876

GBAS	0.992886	0.007114	1.06966	1.069684	1773.81	1658.255
DENND5A	0.992886	0.007114	0.969017	0.969005	1504.849	1552.983
PREB	0.992886	0.007114	1.077643	1.077669	1801.595	1671.751
BMPR2	0.992887	0.007113	1.104337	1.104368	2157.246	1953.375
KIAA0355	0.992888	0.007112	1.075799	1.075825	1785.657	1659.802
ADCY7	0.992895	0.007105	1.127568	1.127601	2491.78	2209.806
PITRM1	0.9929	0.0071	0.907237	0.907212	1954.969	2154.921
MYO18A	0.992904	0.007096	1.079683	1.07971	1800.15	1667.252
DBI	0.992907	0.007093	0.891906	0.891881	2145.02	2405.054
BAK1	0.992908	0.007092	0.967345	0.967333	1484.843	1534.988
TRIM27	0.99291	0.00709	1.121865	1.121897	2398.422	2137.826
SNURF	0.99291	0.00709	1.052033	1.052052	1621.836	1541.592
USP48	0.99291	0.00709	0.899726	0.8997	2020.543	2245.797
TWSG1	0.992915	0.007085	1.030352	1.030363	1520.408	1475.604
KIAA1524	0.992915	0.007085	1.062039	1.062061	1690.454	1591.672
ERI3	0.992917	0.007083	1.031601	1.031613	1525.965	1479.203
MOB4	0.992922	0.007078	1.107895	1.107926	2182.319	1969.733
GLIS3	0.992925	0.007075	0.952988	0.952971	1545.971	1622.264
IQSEC1	0.992926	0.007074	0.942112	0.942093	1603.764	1702.343
SNRPC	0.992927	0.007073	0.873772	0.873747	2544.017	2911.618
DCTN1	0.992931	0.007069	1.158978	1.15901	3363.125	2901.721
CCDC86	0.992932	0.007068	0.950354	0.950337	1534.857	1615.066
C15orf39	0.992935	0.007065	1.026089	1.026099	1515.963	1477.403
CRK	0.992937	0.007063	0.988444	0.98844	1465.66	1482.802
ZHX3	0.992939	0.007061	1.00238	1.002381	1469.439	1465.949
MTA1	0.992941	0.007059	0.921787	0.921764	1813.82	1967.771
TRAP1	0.992941	0.007059	0.901963	0.901939	2057.219	2280.887
PDLIM1	0.992942	0.007058	0.986151	0.986146	1504.849	1525.99
VTA1	0.992943	0.007057	1.069431	1.069455	1763.807	1649.257
AGFG1	0.992944	0.007056	1.14035	1.140383	2872.994	2519.323
PAIP1	0.992947	0.007053	0.869799	0.869774	2666.272	3065.477
GGCX	0.992948	0.007052	1.10424	1.104271	2148.71	1945.817
DDX50	0.992948	0.007052	0.895216	0.895191	2118.347	2366.364
NOC3L	0.992952	0.007048	1.005559	1.005561	1479.286	1471.105
OPHN1	0.992954	0.007046	1.159669	1.1597	3329.839	2871.291
CDK2	0.992956	0.007044	1.205601	1.205628	5295.867	4392.62
SPTLC2	0.99296	0.00704	0.895995	0.89597	2165.448	2416.877
FTSJ1	0.99296	0.00704	1.079676	1.079702	1867.168	1729.335
STRAP	0.992962	0.007038	1.237313	1.237335	7844.329	6339.697
FEN1	0.992962	0.007038	1.100722	1.100752	2110.567	1917.385
R3HDM1	0.992962	0.007038	0.931318	0.931297	1699.345	1824.71
ACSS2	0.992962	0.007038	0.892137	0.892112	2181.697	2445.543
TOR1AIP1	0.992963	0.007037	1.092557	1.092586	2056.863	1882.564
ANXA3	0.992964	0.007036	1.228475	1.228498	6895.185	5612.692
STAM	0.992966	0.007034	1.04829	1.048307	1670.449	1593.472
PIM3	0.992966	0.007034	1.035584	1.035598	1568.199	1514.293
ADAM17	0.992967	0.007033	0.956567	0.956551	1552.639	1623.164
RAVER1	0.992968	0.007032	1.106121	1.106152	2190.588	1980.368
UGDH	0.992968	0.007032	0.946111	0.946093	1603.764	1695.145
TMED10	0.992973	0.007027	0.804986	0.804972	6568.431	8159.827
IDI1	0.992974	0.007026	1.04044	1.040455	1632.661	1569.178
CXXC5	0.992974	0.007026	1.098537	1.098567	2090.561	1902.989
MRPL37	0.99298	0.00702	0.889852	0.889827	2236.156	2513.025
PGM1	0.99298	0.00702	0.870889	0.870865	2730.733	3135.658
IFRD1	0.992983	0.007017	0.817039	0.817024	5384.779	6590.73
ZNF148	0.992983	0.007017	0.895417	0.895392	2140.575	2390.658

CREB3	0.992984	0.007016	0.983942	0.983936	1492.623	1516.993
FLOT2	0.992984	0.007016	1.061233	1.061255	1694.9	1597.071
ARID5B	0.992984	0.007016	0.947072	0.947055	1607.098	1696.944
STX4	0.992986	0.007014	1.01064	1.010644	1540.414	1524.191
FERMT2	0.992989	0.007011	0.840643	0.840623	3816.58	4540.18
EIF2B2	0.992994	0.007006	1.0151	1.015106	1517.074	1494.499
KPNA3	0.992994	0.007006	0.996788	0.996787	1468.172	1472.904
AGPAT3	0.992995	0.007005	1.068518	1.068541	1788.258	1673.55
ALS2	0.992995	0.007005	0.922411	0.922388	1841.606	1996.564
STK4	0.992996	0.007004	1.062981	1.063003	1787.224	1681.297
SAP18	0.993002	0.006998	1.059627	1.059648	1723.796	1626.763
MTRNR2L	0.993006	0.006994	1.064285	1.064307	1803.351	1694.389
RNF167	0.993007	0.006993	0.97217	0.97216	1523.743	1567.379
ATP2C2	0.993007	0.006993	1.072075	1.072099	1826.046	1703.242
PARK7	0.993007	0.006993	0.846135	0.846114	3574.293	4224.365
LIMK1	0.993008	0.006992	0.964214	0.964202	1545.971	1603.369
APLP1	0.99301	0.00699	1.110924	1.110954	2295.061	2065.845
ZC3H11A	0.99301	0.00699	1.19311	1.193138	4715.077	3951.828
UACA	0.99301	0.00699	0.974453	0.974443	1557.14	1597.98
CLPP	0.993011	0.006989	1.040443	1.040458	1614.878	1552.083
PODXL	0.993012	0.006988	1.187966	1.187994	4472.312	3764.589
SETD5	0.993015	0.006985	1.132613	1.132644	2699.614	2383.46
MTF2	0.993019	0.006981	1.035592	1.035605	1597.096	1542.186
USP25	0.993021	0.006979	0.900734	0.90071	2082.782	2312.379
RNF14	0.993021	0.006979	0.880228	0.880204	2529.079	2873.288
MRE11A	0.993023	0.006977	1.0442	1.044216	1640.441	1570.978
SF1	0.993025	0.006975	0.833593	0.833575	4255.587	5105.229
ATP5I	0.993025	0.006975	0.989208	0.989204	1510.406	1526.89
IER3IP1	0.993026	0.006974	1.00166	1.00166	1487.066	1484.601
ELP2	0.993027	0.006973	1.027044	1.027054	1568.199	1526.89
EGLN1	0.993028	0.006972	0.934316	0.934295	1714.905	1835.507
HMGN3	0.99303	0.00697	1.103947	1.103977	2206.148	1998.363
SERPINB1	0.993035	0.006965	1.049677	1.049695	1678.251	1598.799
TIMM50	0.993037	0.006963	0.941399	0.94138	1711.071	1817.62
CHTOP	0.993038	0.006962	1.130463	1.130494	2675.163	2366.364
CHKA	0.993041	0.006959	1.148437	1.148468	3104.167	2702.874
TOMM22	0.993043	0.006957	1.034833	1.034846	1607.098	1552.983
SERINC1	0.993049	0.006951	1.144354	1.144386	2954.127	2581.407
IST1	0.993049	0.006951	1.080646	1.080672	1934.964	1790.519
MIB1	0.993051	0.006949	0.893423	0.893398	2187.254	2448.242
ADRBK1	0.993054	0.006946	0.937915	0.937895	1703.791	1816.612
AFTPH	0.993054	0.006946	0.940381	0.940362	1687.12	1794.118
ARL5A	0.993056	0.006944	1.022227	1.022235	1568.199	1534.088
UCHL5	0.993058	0.006942	0.874887	0.874863	2630.706	3006.992
RRAS2	0.993059	0.006941	1.11219	1.11222	2411.759	2168.418
OSBPL8	0.99306	0.00694	1.080492	1.080517	1940.521	1795.918
BHLHE40	0.993062	0.006938	1.157068	1.157099	3456.484	2987.198
ISG20L2	0.993063	0.006937	0.933375	0.933355	1742.824	1867.268
LRRFIP2	0.993063	0.006937	1.050307	1.050324	1698.234	1616.866
IFITM3	0.993065	0.006935	1.19363	1.193657	4830.342	4046.672
CASK	0.993069	0.006931	1.108469	1.108499	2303.952	2078.442
STK3	0.993073	0.006927	1.125589	1.12562	2670.717	2372.663
NISCH	0.993075	0.006925	0.927564	0.927543	1842.717	1986.666
KDM5A	0.993076	0.006924	1.045392	1.045409	1677.117	1604.269
EIF3G	0.993076	0.006924	0.85054	0.850519	3462.041	4070.507
SERPINB9	0.993076	0.006924	0.954095	0.95408	1622.591	1700.687

ETNK1	0.993076	0.006924	1.021433	1.021441	1577.09	1543.985
CHMP4B	0.993078	0.006922	1.173327	1.173356	4046.642	3448.774
GIGYF1	0.993078	0.006922	1.024329	1.024338	1587.093	1549.384
PPID	0.993083	0.006917	1.046773	1.04679	1729.798	1652.478
NUP50	0.993089	0.006911	0.931402	0.931381	1774.921	1905.688
NUP188	0.993092	0.006908	0.894303	0.894279	2241.713	2506.727
HNRNPH1	0.993093	0.006907	0.8243	0.824283	4991.34	6055.373
SLC9A1	0.993094	0.006906	0.999115	0.999115	1584.87	1586.274
CHEK1	0.993095	0.006905	0.934088	0.934068	1799.372	1926.383
MTMR2	0.993099	0.006901	1.134829	1.13486	2827.426	2491.431
PHF8	0.9931	0.0069	1.016996	1.017002	1615.99	1588.973
ASF1B	0.993103	0.006897	1.097705	1.097733	2173.917	1980.368
SPCS3	0.993104	0.006896	1.096717	1.096745	2213.928	2018.635
HIPK1	0.993106	0.006894	0.983837	0.983831	1557.085	1582.675
LRBA	0.993108	0.006892	1.059759	1.059779	1832.714	1729.335
RABL6	0.993113	0.006887	0.987345	0.98734	1558.196	1578.176
COX6C	0.993117	0.006883	0.854825	0.854804	3276.435	3832.97
PIP4K2A	0.993118	0.006882	1.012714	1.012718	1578.202	1558.381
ARMC10	0.993119	0.006881	0.890159	0.890135	2339.951	2628.761
TTI1	0.99312	0.00688	0.94879	0.948774	1674.894	1765.326
NSFL1C	0.99312	0.00688	1.057808	1.057827	1782.701	1685.247
POLR2L	0.993123	0.006877	0.997551	0.99755	1562.642	1566.479
TXNDC12	0.993124	0.006876	1.151643	1.151674	3227.789	2802.693
GSK3A	0.993127	0.006873	1.005535	1.005537	1571.533	1562.88
KIAA0319	0.99313	0.00687	0.90312	0.903096	2176.829	2410.408
NDUFB5	0.99313	0.00687	0.926173	0.926152	1891.619	2042.451
RPL7L1	0.993137	0.006863	1.049766	1.049783	1709.326	1628.266
LSM12	0.993139	0.006861	0.98051	0.980503	1617.101	1649.257
SMARCD1	0.99314	0.00686	0.963982	0.96397	1619.324	1679.849
USP54	0.993141	0.006859	0.964898	0.964886	1616.756	1675.593
SPTAN1	0.993151	0.006849	1.234531	1.234552	8086.616	6550.24
PAIP2	0.993154	0.006846	1.186399	1.186427	4620.13	3894.154
STAT1	0.993155	0.006845	1.159784	1.159813	3657.649	3153.653
DGKD	0.993155	0.006845	1.039246	1.03926	1691.565	1627.663
CDC73	0.993158	0.006842	0.931306	0.931286	1862.722	2000.163
POLR2E	0.993159	0.006841	0.934554	0.934535	1792.704	1918.285
EPS8L2	0.993161	0.006839	0.867503	0.867481	3009.697	3469.468
KIAA1671	0.993163	0.006837	1.094098	1.094125	2171.694	1984.867
CCAR2	0.993164	0.006836	0.970689	0.970679	1651.555	1701.443
PGD	0.993164	0.006836	0.856669	0.856648	3397.579	3966.135
GNA11	0.993165	0.006835	1.043833	1.043848	1721.573	1649.257
UBA3	0.993165	0.006835	1.037089	1.037102	1687.12	1626.763
GGH	0.993166	0.006834	1.023548	1.023557	1633.772	1596.171
DAZAP1	0.993168	0.006832	0.998422	0.998422	1625.992	1628.563
ATP6V1H	0.993171	0.006829	0.916387	0.916365	2024.988	2209.806
SHISA5	0.993171	0.006829	0.936845	0.936826	1751.581	1869.698
NDUFB3	0.993174	0.006826	1.039181	1.039195	1702.679	1638.46
ATP6V0C	0.993176	0.006824	1.013053	1.013057	1611.544	1590.773
HTATSF1	0.993179	0.006821	0.916667	0.916645	1979.42	2159.42
CXADR	0.993179	0.006821	0.896269	0.896245	2235.867	2494.706
ACAP2	0.99318	0.00682	0.866061	0.866039	3076.382	3552.246
DNPEP	0.99318	0.00682	1.049057	1.049074	1803.818	1719.438
CETN2	0.99318	0.00682	0.949813	0.949797	1704.902	1795.018
SAMD4A	0.993181	0.006819	1.095251	1.095278	2200.591	2009.16
SNRPA1	0.993189	0.006811	1.051434	1.051451	1824.935	1735.634
PAPOLA	0.993189	0.006811	0.806031	0.806018	6939.641	8609.787

CYB5B	0.993193	0.006807	0.906715	0.906692	2119.458	2337.572
ANAPC1	0.993196	0.006804	1.060203	1.060223	1848.363	1743.372
ACBD5	0.993196	0.006804	0.972716	0.972707	1625.992	1671.616
SETD3	0.993196	0.006804	1.101966	1.101994	2307.286	2093.738
RALBP1	0.993196	0.006804	1.107933	1.107961	2399.533	2165.718
RRN3	0.9932	0.0068	0.881676	0.881654	2654.813	3011.176
IKBKAP	0.993201	0.006799	1.043336	1.04335	1740.467	1668.152
KIF1A	0.993201	0.006799	1.044516	1.044531	1747.136	1672.651
TRAPPC3	0.993203	0.006797	1.048292	1.048308	1813.82	1730.235
RBM4	0.993204	0.006796	0.985259	0.985254	1610.088	1634.186
TMEM219	0.993209	0.006791	0.972811	0.972801	1672.671	1719.438
DRG1	0.99321	0.00679	0.971822	0.971812	1635.995	1683.448
UBE2N	0.993211	0.006789	1.058395	1.058414	1889.396	1785.12
PCNXL3	0.993214	0.006786	0.967479	0.967468	1650.443	1705.942
ANKRD13	0.993217	0.006783	0.992325	0.992323	1652.666	1665.453
UBE3C	0.993219	0.006781	1.172491	1.172519	4114.438	3509.057
MAPK1IP1	0.99322	0.00678	0.94621	0.946193	1749.514	1849.003
NPC1	0.993225	0.006775	0.908141	0.908119	2122.792	2337.572
MYDGF	0.993225	0.006775	0.976379	0.97637	1634.883	1674.45
NUDC	0.993227	0.006773	1.163076	1.163104	3866.06	3323.914
DOCK6	0.993236	0.006764	0.987111	0.987106	1627.104	1648.357
RB1	0.993238	0.006762	0.918894	0.918873	1994.98	2171.117
COPS2	0.993239	0.006761	0.930999	0.930979	1873.837	2012.759
PUM1	0.993245	0.006755	0.906418	0.906396	2162.803	2386.159
HNRNPF	0.993245	0.006755	0.830854	0.830837	4774.616	5746.756
ADD1	0.993247	0.006753	0.92631	0.92629	1922.739	2075.742
DBN1	0.993247	0.006753	1.193289	1.193315	5125.821	4295.446
ARHGEF5	0.993254	0.006746	1.204863	1.204887	5817.384	4828.157
SDHD	0.993255	0.006745	1.158516	1.158545	3744.339	3231.932
PPP1R2	0.993256	0.006744	0.954168	0.954154	1728.142	1811.177
DCUN1D5	0.993257	0.006743	1.144894	1.144923	3325.337	2904.42
USP5	0.993258	0.006742	1.03987	1.039883	1757.139	1689.746
FIS1	0.993263	0.006737	0.946277	0.946261	1776.033	1876.896
TBCA	0.993263	0.006737	0.927124	0.927104	1892.731	2041.552
USP39	0.993264	0.006736	1.103992	1.104019	2393.976	2168.418
ID3	0.993267	0.006733	1.007885	1.007888	1625.992	1613.267
PRPF8	0.993268	0.006732	0.831816	0.831799	4745.719	5705.367
PIKFYVE	0.993271	0.006729	1.039181	1.039194	1761.584	1695.145
ATG101	0.993274	0.006726	1.01248	1.012485	1667.114	1646.558
TOP2B	0.993274	0.006726	0.833316	0.833299	4656.806	5588.399
SERTAD2	0.993276	0.006724	1.065275	1.065295	1943.855	1824.71
WDR36	0.993276	0.006724	0.978117	0.97811	1661.557	1698.744
SLC17A5	0.993279	0.006721	0.983181	0.983176	1656	1684.339
EI24	0.993281	0.006719	1.186775	1.186801	4847.969	4084.903
TMPO	0.993281	0.006719	0.84385	0.843832	4144.457	4911.475
PRCC	0.993281	0.006719	0.951396	0.951381	1758.25	1848.104
SKIV2L2	0.993282	0.006718	0.973069	0.97306	1716.016	1763.526
ANKRD11	0.993284	0.006716	1.10789	1.107917	2534.014	2287.186
FUBP3	0.993284	0.006716	1.010518	1.010521	1669.337	1651.956
SRP72	0.993286	0.006714	1.153185	1.153213	3609.858	3130.259
SNRPG	0.993289	0.006711	1.046953	1.046969	1817.155	1735.634
RSU1	0.993289	0.006711	1.137057	1.137086	3077.493	2706.473
SREBF2	0.99329	0.00671	1.205101	1.205125	6050.514	5020.651
DIP2B	0.993293	0.006707	0.918966	0.918946	2082.782	2266.491
USP33	0.993294	0.006706	0.844992	0.844973	4004.798	4739.558
CP	0.993299	0.006701	1.083885	1.083909	2149.466	1983.067

RFC4	0.9933	0.0067	0.949437	0.949422	1823.823	1920.984
TFPI2	0.993301	0.006699	0.822657	0.822642	5404.785	6570.035
RNF26	0.993302	0.006698	0.928035	0.928016	1943.855	2094.637
ASUN	0.993303	0.006697	0.973941	0.973932	1686.008	1731.135
NUP107	0.993304	0.006696	0.907174	0.907152	2197.257	2422.149
VPS45	0.993306	0.006694	0.952822	0.952807	1766.03	1853.502
BCL9L	0.993306	0.006694	1.171221	1.171248	4191.126	3578.339
DNAJC2	0.993311	0.006689	1.089565	1.08959	2228.376	2045.151
VDAC3	0.993313	0.006687	1.000012	1.000012	1672.671	1672.651
ARL1	0.993315	0.006685	1.054038	1.054055	1881.616	1785.12
AGPAT1	0.993316	0.006684	0.890226	0.890204	2506.229	2815.344
MRPS24	0.99332	0.00668	0.932152	0.932133	1918.304	2057.972
GNPAT	0.99332	0.00668	1.0867	1.086724	2252.827	2073.043
CMTM4	0.993322	0.006678	0.919821	0.9198	2044.994	2223.303
DYRK2	0.993328	0.006672	1.0699	1.069921	2071.667	1936.28
MED29	0.993332	0.006668	0.94447	0.944453	1800.717	1906.624
POFUT1	0.993335	0.006665	0.888155	0.888133	2567.523	2890.923
HIPK3	0.993339	0.006661	1.053453	1.05347	1893.842	1797.717
PFKM	0.993341	0.006659	0.923145	0.923125	2021.654	2190.012
UBE2I	0.993349	0.006651	0.904649	0.904627	2271.721	2511.225
SMARCA4	0.993349	0.006651	0.831892	0.831876	4996.897	6006.786
EEF2K	0.993357	0.006643	1.028852	1.028861	1768.731	1719.114
UBE2K	0.993361	0.006639	0.913861	0.913841	2199.48	2406.853
GATAD1	0.993361	0.006639	0.969494	0.969484	1703.246	1756.859
PSMC6	0.993362	0.006638	1.124037	1.124064	2921.896	2599.402
MARCKS	0.993363	0.006637	1.066476	1.066496	2016.097	1890.392
PPM1A	0.993364	0.006636	1.026223	1.026232	1762.696	1717.639
PFKL	0.993365	0.006635	1.16951	1.169537	4313.381	3688.109
AAGAB	0.993375	0.006625	0.950323	0.950309	1868.28	1965.972
VDAC2	0.993377	0.006623	0.802015	0.802004	7952.136	9915.337
KXD1	0.993381	0.006619	1.04137	1.041383	1844.94	1771.624
NBN	0.993383	0.006617	0.938749	0.938732	1907.179	2031.654
PPP1CC	0.993386	0.006614	0.877215	0.877194	2886.331	3290.416
UXS1	0.993387	0.006613	0.896011	0.895989	2453.992	2738.864
ITGB2	0.993393	0.006607	1.095049	1.095074	2370.637	2164.818
NT5DC2	0.993395	0.006605	1.171105	1.171132	4426.744	3779.885
GON4L	0.993396	0.006604	1.052784	1.052801	1895.12	1800.074
ARIH1	0.993397	0.006603	1.028712	1.028722	1793.815	1743.732
RFWD3	0.993399	0.006601	1.001401	1.001401	1726.353	1723.937
HGS	0.993401	0.006599	0.86269	0.86267	3368.682	3904.951
ACBD3	0.993403	0.006597	1.087701	1.087725	2278.39	2094.637
WDR82	0.993403	0.006597	0.861497	0.861477	3498.917	4061.536
PSMD3	0.993404	0.006596	1.108764	1.10879	2660.715	2399.655
CUL4B	0.993405	0.006595	1.085894	1.085917	2311.732	2128.828
DPAGT1	0.993405	0.006595	1.04318	1.043194	1871.614	1794.118
NNT	0.993412	0.006588	1.055761	1.055778	1961.638	1858.001
COL4A3B	0.993414	0.006586	1.079364	1.079386	2186.143	2025.356
GOT2	0.993414	0.006586	1.153934	1.153961	3803.244	3295.815
COPZ1	0.993416	0.006584	0.842309	0.842292	4331.163	5142.119
NQO1	0.993416	0.006584	0.83106	0.831044	5056.914	6085.011
WDR91	0.993418	0.006582	0.892681	0.89266	2609.556	2923.351
OAZ1	0.993418	0.006582	1.240418	1.240436	9534.783	7686.635
RAB22A	0.993419	0.006581	0.985689	0.985684	1738.289	1763.535
CARM1	0.99342	0.00658	1.002687	1.002688	1782.701	1777.922
USP28	0.993424	0.006576	0.898186	0.898165	2446.212	2723.568
CSNK2B	0.993427	0.006573	0.86715	0.86713	3319.78	3828.472

ORAI2	0.993429	0.006571	0.977674	0.977666	1756.116	1796.232
ARIH2	0.993431	0.006569	0.920181	0.920161	2122.792	2306.98
KDELR1	0.993433	0.006567	1.121569	1.121596	2874.105	2562.512
NDUFA13	0.993433	0.006567	0.871582	0.871561	3103.056	3560.344
CBX6	0.993434	0.006566	0.883545	0.883524	2769.633	3134.758
PSME2	0.993441	0.006559	1.171375	1.171401	4405.628	3760.99
EIF4G3	0.993442	0.006558	0.858777	0.858757	3572.07	4159.583
SLC1A3	0.993443	0.006557	0.944005	0.943989	1956.081	2072.143
NSDHL	0.993445	0.006555	1.026734	1.026743	1817.155	1769.825
APP	0.993446	0.006554	1.3274	1.327408	31353.98	23620.46
PPP6R3	0.993449	0.006551	0.903324	0.903303	2377.305	2631.793
RSL1D1	0.99345	0.00655	0.86734	0.86732	3204.194	3694.363
NDRG3	0.99345	0.00655	0.972551	0.972542	1781.512	1831.809
OTUD5	0.993451	0.006549	1.085802	1.085825	2293.949	2112.632
MICU1	0.993453	0.006547	1.091122	1.091146	2423.984	2221.503
DNAJC13	0.993456	0.006544	0.865607	0.865587	3328.672	3845.567
NDUFB4	0.99346	0.00654	1.122483	1.12251	2921.896	2603.001
CPSF6	0.993463	0.006537	0.964022	0.964011	1817.155	1884.994
CBL	0.993464	0.006536	1.186628	1.186653	5076.919	4278.351
PRKAB2	0.993464	0.006536	1.084595	1.084618	2345.074	2162.119
HMGB2	0.993464	0.006536	0.994579	0.994577	1763.807	1773.424
NAP1L4	0.993464	0.006536	1.074975	1.074997	2177.251	2025.356
TRIM37	0.99347	0.00653	0.995643	0.995642	1810.486	1818.412
MLLT6	0.993472	0.006528	1.028855	1.028864	1843.895	1792.166
SAR1B	0.993479	0.006521	0.896256	0.896236	2533.08	2826.357
IER2	0.99348	0.00652	1.053977	1.053993	1997.203	1894.891
SPG21	0.99348	0.00652	0.951961	0.951947	1886.062	1981.268
USP15	0.993484	0.006516	1.01225	1.012254	1841.606	1819.311
ATP6V1G	0.993485	0.006515	1.086753	1.086776	2335.072	2148.623
BAP1	0.993486	0.006514	1.083541	1.083564	2255.05	2081.141
PAPD5	0.993486	0.006514	1.088502	1.088525	2416.204	2219.704
PLS1	0.993486	0.006514	0.897893	0.897872	2463.995	2744.263
ZFP36L2	0.993486	0.006514	1.019212	1.019218	1861.611	1826.509
RRBP1	0.993486	0.006514	1.141686	1.141713	3514.277	3078.073
PSMA4	0.993491	0.006509	0.89801	0.897989	2509.563	2794.649
VMP1	0.993492	0.006508	0.968563	0.968554	1820.489	1879.595
ACSL1	0.993496	0.006504	0.896665	0.896644	2601.81	2901.721
POMGNT1	0.993499	0.006501	1.064798	1.064816	2152.8	2021.757
H3F3B	0.993499	0.006501	0.817008	0.816995	6336.057	7755.323
ARMCX2	0.993502	0.006498	0.918549	0.918529	2198.368	2393.357
G3BP2	0.993507	0.006493	1.190321	1.190344	5452.575	4580.67
SLC25A4	0.993511	0.006489	0.996454	0.996453	1794.926	1801.316
DLST	0.993514	0.006486	1.137202	1.137229	3339.93	2936.901
LTA4H	0.993516	0.006484	1.090654	1.090677	2415.093	2214.305
GNL1	0.993518	0.006482	0.927543	0.927524	2107.233	2271.89
MAT2B	0.993518	0.006482	1.09446	1.094483	2471.775	2258.393
DPM1	0.993527	0.006473	0.925152	0.925134	2191.7	2369.064
SIGMAR1	0.993527	0.006473	1.11186	1.111886	2775.19	2495.93
BACE2	0.993529	0.006471	1.152242	1.152268	3813.391	3309.464
EIF3M	0.993529	0.006471	0.862937	0.862918	3518.723	4077.705
WFDC2	0.993533	0.006467	1.012148	1.012152	1830.492	1808.514
ARHGEF1	0.993534	0.006466	0.988307	0.988303	1854.943	1876.896
NDUFB7	0.993535	0.006465	0.949272	0.949258	1940.521	2044.251
AP3D1	0.993536	0.006464	0.833495	0.83348	5222.514	6265.917
PRCP	0.99354	0.00646	0.956772	0.956759	1901.622	1987.566
RNASEH2	0.993541	0.006459	0.999892	0.999892	1860.5	1860.7

DAZAP2	0.993547	0.006453	1.194956	1.194979	5807.115	4859.595
TBC1D9B	0.993549	0.006451	1.209303	1.209324	6951.867	5748.556
CPEB4	0.993553	0.006447	0.951009	0.950995	1989.423	2091.938
UBXN6	0.993553	0.006447	1.002939	1.002939	1828.269	1822.91
FAM8A1	0.993554	0.006446	1.082687	1.082708	2398.422	2215.205
ECI2	0.993554	0.006446	1.037654	1.037665	1943.855	1873.297
SNRPB2	0.993557	0.006443	1.036085	1.036096	1937.187	1869.698
PRKD3	0.99356	0.00644	1.017697	1.017702	1908.29	1875.096
FBXO18	0.99356	0.00644	0.950234	0.95022	1952.747	2055.048
NRAS	0.993561	0.006439	0.927973	0.927955	2188.365	2358.267
WDR6	0.993562	0.006438	1.050964	1.050979	2034.991	1936.28
HK1	0.993562	0.006438	1.183929	1.183952	5209.177	4399.818
RNF4	0.993562	0.006438	1.003624	1.003625	1834.937	1828.309
NUP62	0.993563	0.006437	0.867061	0.867042	3408.393	3931.062
PIK3CB	0.993563	0.006437	1.010309	1.010312	1847.163	1828.309
DDR1	0.993564	0.006436	1.123744	1.123769	3137.509	2791.95
SDC1	0.993564	0.006436	1.049038	1.049053	2022.765	1928.182
USP24	0.993564	0.006436	0.934377	0.934361	2126.127	2275.489
DDX24	0.993567	0.006433	1.176411	1.176435	4959.11	4215.368
SLU7	0.993568	0.006432	1.052613	1.052628	2101.498	1996.429
NUS1	0.993573	0.006427	1.076782	1.076802	2341.74	2174.716
STAG2	0.993578	0.006422	0.855407	0.855389	3834.363	4482.596
GABARAF	0.993582	0.006418	0.909259	0.909239	2455.104	2700.175
MYC	0.993588	0.006412	1.044738	1.044751	2011.651	1925.483
NCBP2	0.993588	0.006412	1.003127	1.003128	1820.489	1814.813
TUFM	0.993589	0.006411	0.81153	0.811518	7284.178	8975.989
RAB7A	0.99359	0.00641	1.238211	1.238229	9640.367	7785.609
SYNGR2	0.99359	0.00641	0.881414	0.881394	3061.933	3473.967
ENDOD1	0.993591	0.006409	1.084667	1.084689	2458.438	2266.491
POLDIP2	0.993592	0.006408	0.966343	0.966334	1897.176	1963.273
KIAA0368	0.993597	0.006403	1.091937	1.09196	2507.34	2296.183
CNOT7	0.993597	0.006403	1.052386	1.052401	2072.779	1969.571
FEM1B	0.993598	0.006402	1.022755	1.022762	1952.747	1909.287
UBA6	0.993598	0.006402	0.982789	0.982784	1860.5	1893.091
GNAI1	0.993601	0.006399	1.096013	1.096037	2572.913	2347.469
ZMIZ2	0.993604	0.006396	0.919575	0.919556	2269.498	2468.037
SCP2	0.993605	0.006395	1.10864	1.108665	2794.084	2520.223
FUBP1	0.993605	0.006395	0.936402	0.936385	2089.45	2231.401
ARMC1	0.99361	0.00639	1.040517	1.040529	2001.649	1923.683
HK2	0.993615	0.006385	1.157614	1.157639	4128.887	3566.642
KDM1B	0.993616	0.006384	1.037467	1.037478	1988.312	1916.485
GPBP1	0.993618	0.006382	1.042511	1.042524	2068.333	1983.967
RBMX	0.993623	0.006377	0.865788	0.86577	3531.949	4079.549
GLUL	0.993624	0.006376	0.86743	0.867411	3556.588	4100.235
DGCR2	0.993624	0.006376	1.081237	1.081257	2442.878	2259.293
IRS2	0.993625	0.006375	1.050453	1.050467	2129.461	2027.155
ATOX1	0.993626	0.006374	0.960138	0.960127	1947.19	2028.055
PUM3	0.993627	0.006373	0.897684	0.897665	2645.155	2946.708
MAPKAP1	0.993627	0.006373	1.082681	1.082702	2406.202	2222.403
MICALL1	0.993627	0.006373	1.005516	1.005517	1882.728	1872.397
TBL1XR1	0.993628	0.006372	1.049905	1.049919	2128.349	2027.155
AP1G1	0.993629	0.006371	0.98737	0.987367	1877.171	1901.189
ISYNA1	0.993632	0.006368	1.008809	1.008812	1891.619	1875.096
RAB8A	0.993633	0.006367	1.039488	1.0395	2012.763	1936.28
HINT1	0.993636	0.006364	1.22386	1.223879	8267.776	6755.385
UBQLN4	0.99364	0.00636	1.126968	1.126993	3228.645	2864.83

AMFR	0.993643	0.006357	1.065296	1.065313	2222.819	2086.54
GORASP2	0.993646	0.006354	1.145605	1.14563	3851.034	3361.497
MARCH7	0.993648	0.006352	0.889206	0.889187	2852.988	3208.538
CNOT6	0.993651	0.006349	0.917395	0.917376	2338.406	2549.015
YY1	0.993653	0.006347	0.907777	0.907757	2486.223	2738.864
FGD5-AS1	0.993654	0.006346	0.996023	0.996022	1892.731	1900.29
SNAP23	0.993654	0.006346	1.055008	1.055023	2101.676	1992.065
CLNS1A	0.993656	0.006344	1.047247	1.047261	2080.559	1986.666
TBCB	0.993656	0.006344	0.994255	0.994253	1893.842	1904.788
FBXO11	0.993657	0.006343	1.057159	1.057175	2210.594	2091.038
CBWD5	0.993659	0.006341	1.051868	1.051883	2117.424	2012.984
KLC1	0.99366	0.00634	1.050928	1.050942	2110.567	2008.261
AJUBA	0.99366	0.00634	0.885758	0.885739	2951.904	3332.705
CSNK1A1	0.993662	0.006338	1.247137	1.247153	11045.19	8856.321
CACYBP	0.993663	0.006337	1.198966	1.198987	6323.921	5274.383
PDLIM7	0.993665	0.006335	1.16289	1.162915	4414.519	3796.08
TUBB6	0.993669	0.006331	1.074398	1.074417	2398.422	2232.3
RNPS1	0.993672	0.006328	0.904962	0.904943	2551.796	2819.843
DDX39A	0.993673	0.006327	1.013713	1.013717	1932.741	1906.588
PSMD12	0.993674	0.006326	0.946735	0.946722	2060.553	2176.515
PFDN2	0.993677	0.006323	0.993496	0.993494	1954.969	1967.771
COX6A1	0.993678	0.006322	0.873841	0.873822	3318.669	3797.88
GTF3C3	0.993679	0.006321	0.954318	0.954306	2016.097	2112.632
TOPBP1	0.993679	0.006321	1.11824	1.118264	3070.825	2746.062
ASH1L	0.993682	0.006318	0.931141	0.931124	2201.702	2364.565
MRPL4	0.993683	0.006317	1.073987	1.074006	2406.202	2240.398
PRDX4	0.993684	0.006316	1.094726	1.094748	2632.929	2405.054
RELA	0.993687	0.006313	1.036701	1.036712	2037.214	1965.072
COPS6	0.99369	0.00631	0.844865	0.844849	4630.132	5480.428
DNAJC8	0.993692	0.006308	1.121911	1.121936	3169.74	2825.241
LANCL1	0.993693	0.006307	0.992322	0.99232	1920.516	1935.38
PDHB	0.993695	0.006305	1.061631	1.061648	2283.947	2151.322
BOD1	0.993695	0.006305	0.87716	0.877141	3234.202	3687.21
SESN2	0.993698	0.006302	1.044586	1.044599	2095.007	2005.561
GNB2	0.993701	0.006299	1.242521	1.242538	10583.95	8518.012
TBC1D20	0.993702	0.006298	1.079909	1.079929	2497.604	2312.748
NSF	0.993703	0.006297	0.960367	0.960356	2003.06	2085.748
PLEKHM2	0.993704	0.006296	1.138332	1.138357	3617.638	3177.946
SRSF7	0.993707	0.006293	0.916694	0.916676	2456.215	2679.48
MFF	0.993711	0.006289	1.06822	1.068238	2310.621	2163.019
ATAD1	0.993713	0.006287	0.892266	0.892247	2850.766	3195.042
HDAC1	0.993715	0.006285	1.17591	1.175934	5091.367	4329.637
CFL2	0.993718	0.006282	1.036891	1.036901	2062.776	1989.366
MIEF1	0.993719	0.006281	0.915441	0.915422	2427.396	2651.669
DICER1	0.993726	0.006274	0.936186	0.936171	2188.365	2337.572
PPIC	0.993729	0.006271	1.048686	1.048699	2149.466	2049.649
INTS1	0.99373	0.00627	1.032247	1.032256	2046.105	1982.168
SNW1	0.993731	0.006269	1.024359	1.024366	1976.086	1929.082
FAM199X	0.993734	0.006266	1.012007	1.01201	2020.543	1996.564
EBP	0.993736	0.006264	1.022371	1.022377	2007.206	1963.273
HTT	0.993737	0.006263	1.011986	1.01199	1974.975	1951.576
SRPK2	0.99374	0.00626	1.130633	1.130657	3388.688	2997.095
SRP9	0.993741	0.006259	1.173687	1.17371	5152.495	4389.921
KPNA1	0.993744	0.006256	1.139716	1.139741	3714.331	3258.925
EVI5L	0.993746	0.006254	1.059872	1.059888	2257.273	2129.728
DNAJC10	0.993747	0.006253	1.098452	1.098475	2758.519	2511.225

NDUFA2	0.993747	0.006253	1.012908	1.012912	1984.978	1959.674
GOLPH3	0.993748	0.006252	1.086041	1.086062	2565.133	2361.866
PSMB3	0.993751	0.006249	1.021192	1.021198	2062.776	2019.957
KAZN	0.993752	0.006248	1.022865	1.022872	1986.089	1941.678
FANCI	0.993752	0.006248	0.947173	0.94716	2119.458	2237.699
ZFAND3	0.993753	0.006247	0.873295	0.873276	3515.389	4025.519
DAD1	0.993754	0.006246	1.069946	1.069964	2367.302	2212.506
URI1	0.993754	0.006246	1.159834	1.159858	4361.171	3760.09
ANKRD13	0.993755	0.006245	1.022893	1.022899	2023.877	1978.569
MLLT1	0.99376	0.00624	0.895211	0.895192	2835.206	3167.149
PTDSS1	0.993763	0.006237	1.010179	1.010182	1990.535	1970.471
CEP170B	0.993771	0.006229	1.048143	1.048156	2179.474	2079.341
ALDH9A1	0.993772	0.006228	0.98841	0.988407	1980.532	2003.762
UQCR10	0.993777	0.006223	0.925891	0.925874	2341.74	2529.221
UBE2D2	0.993784	0.006216	0.831417	0.831403	5759.324	6927.239
CUX1	0.993785	0.006215	0.854824	0.854807	4347.834	5086.334
PSMA1	0.993785	0.006215	0.922362	0.922345	2450.658	2656.986
BANF1	0.99379	0.00621	1.141789	1.141813	3844.366	3366.896
DDX46	0.99379	0.00621	0.923828	0.923811	2436.265	2637.192
NOC2L	0.993792	0.006208	1.085049	1.085069	2594.03	2390.658
ITPRIPL2	0.993793	0.006207	1.001441	1.001442	2047.216	2044.269
SEC24C	0.993796	0.006204	1.162723	1.162747	4742.385	4078.604
DDX27	0.993799	0.006201	0.903358	0.90334	2707.394	2997.095
SFPQ	0.993803	0.006197	0.862668	0.862651	3908.827	4531.183
PMPCB	0.993805	0.006195	0.916243	0.916225	2496.226	2724.468
HBS1L	0.993807	0.006193	1.019226	1.019231	2050.551	2011.86
CABIN1	0.993807	0.006193	1.067067	1.067084	2383.974	2234.1
MAN2B1	0.993809	0.006191	0.942616	0.942602	2201.702	2335.773
ATP6V1F	0.993814	0.006186	1.083304	1.083324	2591.807	2392.457
ZNF207	0.993815	0.006185	1.114246	1.114269	3143.066	2820.742
TBRG4	0.993817	0.006183	0.931688	0.931673	2367.302	2540.917
SCPEP1	0.993819	0.006181	0.957051	0.95704	2110.567	2205.308
ZKSCAN1	0.993819	0.006181	0.839106	0.839091	5244.364	6250.054
IFNAR1	0.993828	0.006172	1.033604	1.033613	2131.695	2062.372
PEX19	0.993831	0.006169	0.93156	0.931545	2325.069	2495.93
HDAC3	0.993833	0.006167	0.932131	0.932116	2320.623	2489.631
PPIB	0.993833	0.006167	0.827236	0.827223	6235.008	7537.275
SCOC	0.993836	0.006164	0.934103	0.934088	2302.841	2465.338
MICAL3	0.993838	0.006162	1.073149	1.073167	2482.322	2313.081
LTBR	0.993843	0.006157	1.044215	1.044227	2211.705	2118.031
MARK3	0.993845	0.006155	1.018808	1.018813	2129.461	2090.139
TSN	0.993846	0.006154	0.987137	0.987134	2087.227	2114.432
USP10	0.993847	0.006153	1.084974	1.084994	2712.951	2500.428
SOAT1	0.99385	0.00615	0.947177	0.947164	2253.283	2378.979
C5orf15	0.993851	0.006149	0.979288	0.979283	2053.885	2097.337
HDAC2	0.993855	0.006145	0.883991	0.883973	3227.578	3651.219
PRPSAP1	0.993855	0.006145	0.989803	0.9898	2043.882	2064.945
MROH1	0.993866	0.006134	1.004877	1.004879	2060.553	2050.549
SFXN1	0.993867	0.006133	0.936001	0.935986	2311.732	2469.837
GYS1	0.993868	0.006132	1.110302	1.110324	3121.95	2811.745
LMNB1	0.993871	0.006129	1.048855	1.048868	2270.61	2164.818
NUCB1	0.993873	0.006127	1.213765	1.213784	8051.051	6633.018
RRP7A	0.993877	0.006123	0.886203	0.886186	3270.389	3690.413
TMC6	0.99388	0.00612	1.032793	1.032802	2222.819	2152.222
ATF6	0.99388	0.00612	0.887924	0.887906	3150.079	3547.765
JOSD1	0.993882	0.006118	1.11449	1.114512	3309.244	2969.229

RAP1B	0.993883	0.006117	1.154875	1.154898	4457.864	3859.963
GDI2	0.993888	0.006112	0.890266	0.890248	3097.499	3479.365
FAM20B	0.993888	0.006112	1.126658	1.126681	3478.134	3087.062
KIF22	0.993893	0.006107	1.002118	1.002118	2078.336	2073.943
PPIG	0.993895	0.006105	1.030494	1.030502	2170.583	2106.334
TACC1	0.993895	0.006105	0.932185	0.93217	2433.987	2611.099
ABCE1	0.993898	0.006102	1.137715	1.137738	3968.844	3488.363
HIBADH	0.993901	0.006099	0.879909	0.879891	3409.805	3875.259
SNX6	0.993906	0.006094	1.054913	1.054927	2353.965	2231.401
DYNLRB1	0.993909	0.006091	0.94097	0.940956	2302.841	2447.343
CHERP	0.99391	0.00609	1.052387	1.052401	2335.072	2218.804
RAB2A	0.993911	0.006089	1.202929	1.202948	7247.502	6024.782
CHML	0.993919	0.006081	1.031785	1.031794	2198.368	2130.628
ABHD2	0.993919	0.006081	0.989694	0.989692	2095.307	2117.131
CHD2	0.99392	0.00608	1.03215	1.032158	2253.939	2183.713
UBFD1	0.99392	0.00608	1.131228	1.131251	3712.108	3281.419
DNM1L	0.993922	0.006078	0.900056	0.900039	2907.381	3230.285
PSIP1	0.993923	0.006077	1.032484	1.032493	2258.384	2187.312
DBF4	0.993924	0.006076	1.000352	1.000352	2101.676	2100.936
WDR43	0.993924	0.006076	0.978926	0.978921	2113.901	2159.42
NAA15	0.993925	0.006075	1.070974	1.070991	2601.81	2429.347
PARP1	0.993925	0.006075	1.146851	1.146874	4327.829	3773.586
TEAD1	0.993926	0.006074	1.114845	1.114867	3294.218	2954.806
FKBP1A	0.993926	0.006074	1.186414	1.186434	6127.368	5164.523
ESYT2	0.993934	0.006066	0.903684	0.903667	2846.053	3149.451
SPEN	0.993934	0.006066	0.940968	0.940954	2285.058	2428.448
MAP3K7	0.99394	0.00606	0.993205	0.993203	2111.678	2126.129
PSMA3	0.993941	0.006059	0.937636	0.937622	2419.539	2580.507
NASP	0.993946	0.006054	0.877973	0.877956	3618.75	4121.793
CAV2	0.993947	0.006053	1.024537	1.024544	2186.609	2134.227
RFC1	0.993954	0.006046	1.067586	1.067602	2590.696	2426.648
CORO1B	0.993957	0.006043	0.931189	0.931175	2505.117	2690.277
MCUR1	0.993959	0.006041	0.923225	0.923209	2547.217	2759.091
RBBP6	0.99396	0.00604	1.061687	1.061702	2529.568	2382.56
GNG11	0.993962	0.006038	1.116733	1.116755	3467.598	3105.066
DENND4B	0.993963	0.006037	0.984705	0.984701	2136.129	2169.317
RPL19	0.993965	0.006035	0.789912	0.789904	12652.29	16017.5
BAG5	0.993973	0.006027	1.072467	1.072484	2607.367	2431.147
DEPDC1	0.993973	0.006027	1.117568	1.11759	3420.919	3060.978
PSMD7	0.993976	0.006024	0.987472	0.987468	2143.909	2171.117
COX7B	0.993981	0.006019	0.870907	0.87089	3838.809	4407.916
DDX39B	0.993983	0.006017	0.895753	0.895736	3076.293	3434.377
OCRL	0.993983	0.006017	1.116598	1.11662	3410.916	3054.679
RPS6KA1	0.99399	0.00601	0.939901	0.939887	2443.99	2600.302
ITM2C	0.993993	0.006007	1.096836	1.096856	3004.14	2738.864
MALAT1	0.993996	0.006004	1.084627	1.084645	2801.864	2583.206
SCAF1	0.993997	0.006003	1.051167	1.05118	2462.884	2342.971
AGPS	0.993997	0.006003	1.039979	1.03999	2373.971	2282.687
NDFIP1	0.993998	0.006002	1.147229	1.147251	4362.283	3802.379
UHRF1BP	0.993999	0.006001	0.91685	0.916834	2681.842	2925.114
TCAF1	0.994001	0.005999	0.987298	0.987294	2217.262	2245.797
KMT2D	0.994002	0.005998	0.998949	0.998948	2166.137	2168.418
SP3	0.994007	0.005993	1.016774	1.016779	2260.607	2223.303
RPS19	0.994009	0.005991	1.282585	1.282595	20435.49	15932.92
NDUFA11	0.994011	0.005989	0.907968	0.907951	2799.641	3083.472
SNX14	0.994014	0.005986	0.972329	0.972322	2207.259	2270.09

CLDN4	0.994018	0.005982	0.876403	0.876386	3688.768	4209.069
POMP	0.994022	0.005978	1.007257	1.007259	2195.034	2179.215
ARPP19	0.994029	0.005971	0.939098	0.939085	2431.975	2589.729
PTPN1	0.994034	0.005966	1.083577	1.083596	2827.426	2609.299
JTB	0.994039	0.005961	0.839773	0.83976	5659.298	6739.19
ID1	0.99404	0.00596	1.133	1.133022	4041.085	3566.642
AGAP3	0.994042	0.005958	0.986297	0.986293	2201.702	2232.3
NOP58	0.994045	0.005955	0.876112	0.876095	3738.782	4267.554
SLC5A3	0.994047	0.005953	1.05615	1.056164	2560.088	2423.949
BCCIP	0.99405	0.00595	0.985118	0.985114	2210.594	2243.997
SNX3	0.994051	0.005949	1.115118	1.115139	3467.598	3109.565
STX6	0.994053	0.005947	1.050863	1.050875	2515.12	2393.357
MAN2A1	0.994053	0.005947	1.104265	1.104285	3224.199	2919.716
SNRPD3	0.994058	0.005942	0.878547	0.878531	3760.243	4280.15
ANXA6	0.994059	0.005941	0.873514	0.873498	3856.591	4415.114
ETS2	0.994059	0.005941	0.967174	0.967166	2321.735	2400.555
ETFA	0.994061	0.005939	1.097921	1.09794	3181.966	2898.121
HNRNPA0	0.994062	0.005938	0.88205	0.882033	3560.956	4037.216
CUL1	0.994063	0.005937	0.934205	0.934191	2472.886	2647.089
TCEB1	0.994068	0.005932	1.118187	1.118208	3653.203	3267.013
DNAJB9	0.994068	0.005932	1.074413	1.074429	2799.641	2605.7
COPE	0.994073	0.005927	0.886842	0.886826	3428.699	3866.261
CELF1	0.994091	0.005909	0.954467	0.954456	2365.08	2477.934
STRN4	0.994095	0.005905	1.078204	1.078221	2816.312	2611.998
JMJD1C	0.994095	0.005905	0.96105	0.961041	2329.514	2423.949
CANT1	0.994099	0.005901	1.106646	1.106666	3415.362	3086.171
ATRN	0.994099	0.005901	0.981719	0.981714	2259.496	2301.582
BABAM1	0.994101	0.005899	0.992992	0.992991	2250.604	2266.491
RPL35	0.994102	0.005898	1.228297	1.228312	10655.08	8674.57
EIF3E	0.994102	0.005898	1.188212	1.188231	6687.351	5627.988
RPS15	0.994107	0.005893	1.039841	1.039851	2478.443	2383.46
WAPL	0.994108	0.005892	0.869244	0.869228	4104.436	4721.932
AKAP9	0.994108	0.005892	0.949217	0.949205	2419.539	2549.015
SYMPK	0.994109	0.005891	1.075803	1.075819	2865.214	2663.285
ATF6B	0.994111	0.005889	0.917324	0.917309	2796.307	3048.381
AKT2	0.994112	0.005888	1.121914	1.121935	3731.002	3325.507
ATP6V1C1	0.994117	0.005883	1.120297	1.120318	3629.864	3240.03
IQGAP1	0.994118	0.005882	1.215088	1.215104	8991.304	7399.612
ZFAND1	0.994122	0.005878	1.03808	1.038089	2422.873	2333.973
AKT1S1	0.994123	0.005877	1.070112	1.070128	2737.402	2558.013
ELAVL1	0.994124	0.005876	0.888555	0.888539	3532.06	3975.132
PIGT	0.994124	0.005876	0.941547	0.941534	2458.438	2611.099
RBM25	0.994128	0.005872	0.953886	0.953876	2403.979	2520.223
BOP1	0.994134	0.005866	1.042729	1.04274	2468.441	2367.264
GNL3	0.994137	0.005863	1.1147	1.114721	3577.627	3209.438
ASXL1	0.994139	0.005861	1.063988	1.064002	2679.608	2518.423
RPL36AL	0.99414	0.00586	0.917978	0.917963	2886.675	3144.655
VDAC1	0.994142	0.005858	1.240515	1.240529	12232.17	9860.451
YTHDF1	0.994143	0.005857	1.080911	1.080928	2981.912	2758.659
TMF1	0.994143	0.005857	1.023219	1.023225	2317.289	2264.692
ATP11A	0.994144	0.005856	1.149401	1.149422	4823.518	4196.473
MPC2	0.994146	0.005854	0.936862	0.936849	2571.802	2745.163
FAM208B	0.994151	0.005849	0.867902	0.867886	4337.832	4998.157
CDK2AP2	0.994155	0.005845	1.011959	1.011962	2327.292	2299.782
ICMT	0.994156	0.005844	0.901465	0.901449	3157.792	3503.02
PFDN1	0.994159	0.005841	1.03617	1.036178	2505.117	2417.651

ZBTB4	0.99416	0.00584	0.942264	0.942252	2530.68	2685.779
ZC3H15	0.99416	0.00584	0.98206	0.982056	2315.066	2357.367
EFTUD2	0.994161	0.005839	1.062276	1.06229	2682.943	2525.622
XRCC6	0.994161	0.005839	0.804176	0.804167	10269.42	12770.27
EIF4A2	0.99417	0.00583	1.133357	1.133378	4167.786	3677.312
KDM1A	0.994171	0.005829	1.078804	1.07882	2913.005	2700.175
CCT2	0.994174	0.005826	0.812183	0.812174	9228.034	11362.15
TNKS1BP	0.994181	0.005819	1.157961	1.157981	5064.693	4373.725
TSPAN17	0.994184	0.005816	0.965416	0.965408	2449.547	2537.318
ZMAT2	0.994185	0.005815	1.119593	1.119613	3777.681	3374.094
SRRT	0.994187	0.005813	1.071505	1.071521	2895.222	2701.974
RMND5A	0.994189	0.005811	0.973719	0.973713	2363.613	2427.422
CRTC2	0.994196	0.005804	0.993475	0.993473	2338.406	2353.768
ABI1	0.994196	0.005804	0.998617	0.998617	2340.629	2343.87
C16orf58	0.994198	0.005802	1.040306	1.040316	2515.12	2417.651
DCTN2	0.994199	0.005801	0.89041	0.890394	3584.296	4025.519
LAD1	0.994199	0.005801	0.883114	0.883098	3726.556	4219.866
HARS	0.994202	0.005798	1.075085	1.075101	2895.222	2692.977
TNPO1	0.994203	0.005797	1.158762	1.158782	5239.185	4521.285
IDS	0.994204	0.005796	0.971826	0.971819	2384.207	2453.344
ZBTB44	0.994204	0.005796	1.021735	1.02174	2409.536	2358.267
COPS5	0.994205	0.005795	0.962212	0.962203	2428.43	2523.822
NIPA2	0.994207	0.005793	1.170025	1.170045	5824.909	4978.363
TFE3	0.99421	0.00579	1.032147	1.032155	2530.68	2451.841
GGA2	0.994211	0.005789	0.909647	0.909632	3053.042	3356.35
DMKN	0.994211	0.005789	0.898586	0.89857	3298.664	3671.014
ZNF106	0.994214	0.005786	1.050596	1.050608	2681.854	2552.668
EIF4B	0.994217	0.005783	1.220105	1.22012	10140.5	8311.067
BIRC6	0.994221	0.005779	1.059072	1.059085	2666.272	2517.524
AES	0.994221	0.005779	1.069458	1.069473	2839.651	2655.187
BRI3	0.994222	0.005778	0.901426	0.90141	3244.205	3599.033
RNF10	0.994224	0.005776	1.13272	1.132741	4237.805	3741.195
TNKS2	0.994226	0.005774	1.087153	1.08717	3190.857	2935.012
USP7	0.994229	0.005771	0.941141	0.941129	2614.035	2777.554
BET1	0.994229	0.005771	0.933988	0.933975	2646.266	2833.339
RRM1	0.994231	0.005769	1.106071	1.10609	3588.742	3244.528
CMTM6	0.994231	0.005769	1.158203	1.158223	5399.228	4661.648
DDX18	0.994233	0.005767	0.969339	0.969332	2422.873	2499.529
YY1AP1	0.994234	0.005766	0.992528	0.992526	2374.916	2392.799
TMEM14B	0.994237	0.005763	0.902232	0.902217	3324.226	3684.51
IGF2BP2	0.994238	0.005762	0.904096	0.90408	3204.194	3544.148
RBBP7	0.994239	0.005761	1.182271	1.18229	6651.786	5626.189
CDC27	0.99424	0.00576	0.986166	0.986163	2385.085	2418.55
RNF114	0.99424	0.00576	0.881092	0.881076	3859.925	4380.923
KRT17	0.994241	0.005759	1.237131	1.237144	12216.61	9874.847
TOR1AIP2	0.994241	0.005759	1.058946	1.058959	2800.13	2644.228
HN1L	0.994244	0.005756	1.106179	1.106198	3525.391	3186.944
EIF5B	0.994245	0.005755	1.175049	1.175068	6208.334	5283.381
SLC25A36	0.994246	0.005754	0.891595	0.891579	3618.75	4058.81
DHCR24	0.994249	0.005751	1.269458	1.269469	18420.5	14510.4
BAZ1B	0.994251	0.005749	0.963784	0.963776	2466.218	2558.913
ZBTB38	0.994252	0.005748	0.932693	0.932679	2734.068	2931.413
POLD1	0.994254	0.005746	1.127641	1.127661	4124.441	3657.518
ARHGAP5	0.994254	0.005746	1.118037	1.118056	3934.39	3518.955
ACSL3	0.994255	0.005745	0.97671	0.976705	2477.332	2536.419
MCM4	0.994257	0.005743	0.89105	0.891035	3650.98	4097.463

MAT2A	0.994257	0.005743	0.903195	0.90318	3249.762	3598.133
CEBPZ	0.994259	0.005741	1.040775	1.040784	2581.804	2480.634
ANXA11	0.994266	0.005734	0.870885	0.87087	4303.378	4941.473
PSMD1	0.994266	0.005734	0.935794	0.935781	2778.524	2969.202
AP1B1	0.994271	0.005729	0.870809	0.870794	4418.964	5074.637
DCDC2	0.994272	0.005728	0.938561	0.938549	2752.962	2933.212
EMC1	0.994274	0.005726	0.959217	0.959209	2576.247	2685.806
DNTTIP2	0.994274	0.005726	1.066721	1.066735	2934.121	2750.561
DCAF7	0.994281	0.005719	1.023337	1.023342	2495.115	2438.201
RPSAP58	0.994283	0.005717	1.038159	1.038168	2650.267	2552.83
MBTPS1	0.994285	0.005715	1.005555	1.005556	2496.226	2482.433
CDC23	0.994288	0.005712	1.031901	1.031908	2549.574	2470.736
SRSF4	0.99429	0.00571	1.040754	1.040763	2569.579	2468.937
AFF1	0.99429	0.00571	1.002168	1.002169	2437.321	2432.047
ZNHIT6	0.994291	0.005709	1.038943	1.038952	2665.16	2565.238
PDCD5	0.994296	0.005704	0.879164	0.879149	4019.968	4572.572
ANXA7	0.994296	0.005704	0.889028	0.889013	3684.323	4144.287
DDX23	0.994304	0.005696	1.028896	1.028903	2505.117	2434.746
ITFG1	0.994311	0.005689	1.011218	1.011221	2478.443	2450.942
NDUFC2	0.994312	0.005688	0.966328	0.96632	2516.976	2604.701
ARHGEF3	0.994313	0.005687	1.001501	1.001501	2459.216	2455.53
ISOC2	0.994315	0.005685	0.908644	0.908629	3206.417	3528.852
PLEKHB2	0.994315	0.005685	1.094991	1.095009	3464.441	3163.847
SUMO2	0.994315	0.005685	0.94206	0.942049	2764.076	2934.112
CIAO1	0.994316	0.005684	1.056106	1.056118	2787.437	2639.324
CDC42EP	0.994317	0.005683	1.055047	1.055059	2844.097	2695.676
KRT80	0.994321	0.005679	1.253566	1.253578	15461.93	12334.24
LEMD2	0.994324	0.005676	0.989168	0.989166	2466.218	2493.23
SDHC	0.994328	0.005672	0.893958	0.893943	3586.519	4012.022
PPP3R1	0.994329	0.005671	0.951581	0.951571	2628.484	2762.258
TAB2	0.994332	0.005668	1.022812	1.022817	2606.255	2548.116
SRSF5	0.994333	0.005667	0.972315	0.972309	2572.913	2646.189
RHOB	0.994333	0.005667	1.038371	1.03838	2644.043	2546.316
MTA2	0.994335	0.005665	0.929331	0.929318	2944.124	3168.049
GRB2	0.994336	0.005664	1.041619	1.041628	2671.995	2565.211
SIN3B	0.994338	0.005662	0.994891	0.99489	2539.571	2552.614
SRRM1	0.994341	0.005659	0.936104	0.936092	2861.769	3057.145
ARHGEF2	0.994342	0.005658	0.861269	0.861255	4832.409	5610.893
SLC39A6	0.994343	0.005657	0.986075	0.986072	2489.557	2524.722
SRI	0.994349	0.005651	0.96414	0.964133	2628.484	2726.268
HAT1	0.994351	0.005649	1.002177	1.002177	2499.56	2494.13
YWHAG	0.994351	0.005649	0.82597	0.82596	7798.761	9442.064
HMGB1	0.994351	0.005649	1.207877	1.207893	9117.549	7548.306
CTSD	0.994352	0.005648	1.149646	1.149665	5136.935	4468.2
HPS3	0.994356	0.005644	0.982357	0.982353	2508.451	2553.514
NELFE	0.994358	0.005642	0.942156	0.942145	2747.404	2916.117
RCC2	0.994358	0.005642	1.157826	1.157845	5670.412	4897.384
NACC1	0.994361	0.005639	0.897738	0.897723	3615.415	4027.318
RBM27	0.994366	0.005634	0.986399	0.986395	2572.913	2608.399
GUCD1	0.994368	0.005632	0.96824	0.968234	2567.356	2651.588
RANBP9	0.99437	0.00563	0.91099	0.910976	3309.778	3633.224
TMED5	0.994371	0.005629	1.150261	1.15028	5206.954	4526.684
PI4KB	0.994372	0.005628	1.013894	1.013897	2550.685	2515.724
SBDS	0.994373	0.005627	0.974264	0.974259	2548.084	2615.408
OS9	0.994375	0.005625	0.873096	0.873082	4402.293	5042.246
MYL6	0.994377	0.005623	1.294136	1.294144	26611.59	20563.08

ADCY6	0.994378	0.005622	0.948631	0.94862	2707.394	2854.033
UTRN	0.994378	0.005622	0.967925	0.967918	2579.582	2665.084
SRPRB	0.994381	0.005619	1.020366	1.02037	2586.25	2534.619
BUB1	0.994382	0.005618	1.109591	1.109609	3818.803	3441.576
TTC3	0.994387	0.005613	1.203183	1.203199	8641.198	7181.853
TIMM23	0.99439	0.00561	1.052178	1.05219	2832.916	2692.401
PPP4C	0.994392	0.005608	1.032649	1.032657	2668.494	2584.106
PAFAH1B	0.994399	0.005601	1.068973	1.068986	3123.061	2921.515
AHSA1	0.994399	0.005601	1.145834	1.145852	5072.473	4426.811
GNPNAT1	0.994406	0.005594	0.932841	0.932829	2913.005	3122.764
SRSF9	0.994407	0.005593	1.019007	1.019011	2608.478	2559.812
SGCE	0.994414	0.005586	1.028567	1.028573	2666.272	2592.204
CAMSAP2	0.994415	0.005585	1.078012	1.078027	3208.639	2976.4
SSR2	0.994415	0.005585	1.157343	1.157362	5781.553	4995.458
RPL10A	0.994416	0.005584	0.818264	0.818255	8863.491	10832.19
CLDN7	0.994419	0.005581	1.153691	1.15371	5473.692	4744.426
FDPS	0.994423	0.005577	0.909237	0.909223	3422.03	3763.689
ST14	0.994426	0.005574	1.152032	1.15205	5541.488	4810.108
SRSF11	0.994427	0.005573	1.070095	1.070109	3176.409	2968.303
KPNA6	0.994427	0.005573	1.096673	1.09669	3582.429	3266.582
S100A11	0.99443	0.00557	1.231906	1.231919	12402.22	10067.4
PPP2R3A	0.994434	0.005566	1.030181	1.030188	2764.076	2683.079
UBQLN1	0.994441	0.005559	1.172998	1.173015	6600.661	5627.088
HEXB	0.994441	0.005559	1.0659	1.065913	3008.586	2822.542
DDIT4	0.994447	0.005553	0.846654	0.846642	6109.418	7216.062
UQCRFS1	0.994451	0.005549	0.864136	0.864123	4986.895	5771.05
ADH5	0.994454	0.005546	0.922275	0.922262	3078.605	3338.103
GDE1	0.994454	0.005546	0.939148	0.939137	2894.111	3081.672
SSB	0.99446	0.00554	0.865264	0.865251	5065.805	5854.727
GSTM3	0.99446	0.00554	1.075745	1.075759	3233.735	3006.003
BCR	0.994464	0.005536	0.895362	0.895348	3755.553	4194.52
SF3A1	0.994464	0.005536	1.049493	1.049503	2895.222	2758.659
ANKRD17	0.994465	0.005535	0.97668	0.976675	2640.709	2703.774
ATP6V1B2	0.994467	0.005533	0.997411	0.99741	2619.592	2626.394
VAMP7	0.994473	0.005527	1.182009	1.182026	7185.263	6078.767
HNRNPR	0.994486	0.005514	0.917524	0.917511	3260.876	3554.045
INTS3	0.994487	0.005513	0.962923	0.962915	2727.399	2832.439
DAP	0.994488	0.005512	0.944192	0.944182	2880.774	3051.08
NR2F6	0.994491	0.005509	1.039369	1.039377	2901.89	2791.95
ATG3	0.994495	0.005505	0.916177	0.916164	3299.775	3601.732
ATP6V1D	0.994495	0.005505	1.001006	1.001007	2655.157	2652.487
DYNC1LI2	0.994497	0.005503	1.073845	1.073859	3254.207	3030.386
ISY1-RAB	0.994508	0.005492	0.997285	0.997284	2665.294	2672.552
URB1	0.994508	0.005492	0.993554	0.993553	2728.511	2746.215
EXOC5	0.99451	0.00549	0.98023	0.980226	2682.943	2737.065
EXOC4	0.994511	0.005489	1.089972	1.089988	3653.203	3351.6
PSMB2	0.994513	0.005487	1.127756	1.127773	4712.377	4178.478
EXOC2	0.994516	0.005484	1.055212	1.055222	3023.034	2864.83
NUMA1	0.99452	0.00548	1.134007	1.134025	4962.444	4375.957
SPIN1	0.994523	0.005477	0.999988	0.999988	2749.627	2749.661
EIF5	0.994524	0.005476	1.190274	1.19029	8124.404	6825.566
ADGRL1	0.994527	0.005473	0.922673	0.92266	3303.109	3579.985
ATPIF1	0.994531	0.005469	1.091623	1.091638	3633.198	3328.206
NR1H2	0.994534	0.005466	0.910108	0.910094	3484.269	3828.472
RAF1	0.994536	0.005464	0.975313	0.975308	2727.399	2796.449
KRT8	0.994538	0.005462	1.299122	1.299129	31042.09	23894.53

ABCF1	0.994539	0.005461	0.969122	0.969115	2756.296	2844.136
RPS25	0.99454	0.00546	1.236297	1.236309	14021.54	11341.45
CNOT8	0.994541	0.005459	1.060269	1.060281	3118.615	2941.31
ANKIB1	0.994542	0.005458	1.156517	1.156534	5912.699	5112.427
RAB5C	0.994546	0.005454	0.984882	0.984879	2715.174	2756.859
WAC-AS1	0.994547	0.005453	0.925311	0.925299	3208.528	3467.561
ARPC4	0.994552	0.005448	1.021885	1.021889	2741.258	2682.539
KCMF1	0.994555	0.005445	1.075205	1.075218	3358.98	3123.997
MVP	0.994557	0.005443	0.988107	0.988104	2724.065	2756.859
FBXO7	0.99456	0.00544	1.000517	1.000517	2795.195	2793.75
MDH2	0.99456	0.00544	0.87291	0.872897	4771.281	5466.032
LONP2	0.994561	0.005439	0.984903	0.984899	2798.529	2841.437
MTRNR2L	0.994564	0.005436	1.084341	1.084356	3621.673	3339.93
BNIP3	0.994568	0.005432	0.980524	0.980521	2815.2	2871.129
HSF1	0.99457	0.00543	0.913946	0.913934	3537.617	3870.76
TALDO1	0.994574	0.005426	1.115395	1.115412	4417.853	3960.736
CDC42EP	0.994575	0.005425	1.160017	1.160034	6189.44	5335.567
VPS41	0.994576	0.005424	0.954702	0.954693	2893.088	3030.386
PKN2	0.994577	0.005423	0.942481	0.942471	3084.162	3272.421
MFN2	0.994582	0.005418	0.882322	0.882308	4406.739	4994.558
DEGS1	0.994583	0.005417	1.102013	1.102029	3958.496	3592.006
ACIN1	0.994583	0.005417	1.025485	1.02549	2847.431	2776.654
SSR3	0.994583	0.005417	1.225224	1.225237	12531.65	10227.94
SEC24A	0.99459	0.00541	1.076854	1.076868	3438.701	3193.242
GNAQ	0.994591	0.005409	0.98779	0.987788	2764.076	2798.248
MRS2	0.994591	0.005409	0.939372	0.939362	3066.379	3264.323
TMEM106	0.994592	0.005408	0.984792	0.984789	2836.317	2880.126
CUL2	0.994593	0.005407	1.000024	1.000024	2835.206	2835.138
CBLL1	0.994594	0.005406	1.020213	1.020217	2831.872	2775.754
FARSA	0.994596	0.005404	1.008523	1.008524	2788.527	2764.957
IPO9	0.994599	0.005401	0.887872	0.887859	4228.291	4762.349
SRPRA	0.9946	0.0054	1.184579	1.184595	8112.179	6848.06
IGF2BP3	0.994601	0.005399	0.983475	0.983472	2782.97	2829.74
G3BP1	0.994602	0.005398	1.179211	1.179227	7512.017	6370.289
CSRP1	0.994603	0.005397	1.247811	1.247822	16191.01	12975.41
SNHG16	0.994605	0.005395	1.074811	1.074825	3507.609	3263.423
DNAJA2	0.994608	0.005392	0.911198	0.911185	3572.07	3920.247
ALAS1	0.994608	0.005392	1.033749	1.033756	3005.252	2907.119
CDC25B	0.99461	0.00539	0.913411	0.913398	3526.503	3860.863
PSMA6	0.99461	0.00539	0.976385	0.976381	2812.978	2881.026
FBLN1	0.994611	0.005389	1.10666	1.106676	4228.913	3821.274
PKP4	0.994611	0.005389	0.91312	0.913107	3619.861	3964.335
CACUL1	0.994613	0.005387	1.037796	1.037803	2973.476	2865.163
ATP6V1A	0.994618	0.005382	0.896656	0.896643	3972.389	4430.293
P4HA1	0.994623	0.005377	1.108854	1.108871	4213.354	3799.679
RAN	0.994628	0.005372	1.173089	1.173105	7156.366	6100.361
PCNP	0.994629	0.005371	0.92044	0.920427	3412.027	3707.004
CYBRD1	0.994629	0.005371	0.977553	0.977548	2900.779	2967.403
RNF7	0.994632	0.005368	1.031139	1.031145	2946.002	2857.021
NOMO2	0.994632	0.005368	0.853659	0.853647	5964.224	6986.758
TMEM167	0.994632	0.005368	0.982522	0.982519	2890.776	2942.21
HSPA13	0.994632	0.005368	0.915848	0.915835	3508.72	3831.171
CLTA	0.994634	0.005366	1.1022	1.102215	4052.199	3676.412
ZRANB2	0.994637	0.005363	0.975481	0.975476	2917.45	2990.797
PPFIA1	0.994638	0.005362	1.039361	1.039369	3019.7	2905.32
PRKDC	0.99464	0.00536	0.837365	0.837355	7494.235	8949.896

PSME3	0.994641	0.005359	0.901784	0.901771	3862.148	4282.85
ANXA4	0.994642	0.005358	0.996813	0.996812	2825.203	2834.239
WAC	0.994642	0.005358	0.845525	0.845514	6899.909	8160.61
FAM213A	0.99465	0.00535	0.940535	0.940525	3131.952	3330.005
DNASE2	0.994653	0.005347	1.085752	1.085767	3703.817	3411.245
EIF3B	0.994653	0.005347	1.123089	1.123105	4720.156	4202.771
RBM39	0.994654	0.005346	1.083729	1.083743	3665.429	3382.191
NCAPD3	0.994656	0.005344	1.092726	1.092741	3861.037	3533.351
PPP6R1	0.994657	0.005343	1.199737	1.199751	9733.725	8113.121
ARFGEF2	0.994665	0.005335	0.970094	0.970089	2907.447	2997.095
HIGD1A	0.994667	0.005333	1.101588	1.101604	4095.544	3717.801
RAD23A	0.994668	0.005332	0.976157	0.976152	2885.219	2955.706
PAFAH1B	0.994668	0.005332	1.034313	1.03432	3089.719	2987.198
SP1	0.994672	0.005328	1.016734	1.016737	2984.135	2935.012
ITGB8	0.994674	0.005326	1.162419	1.162435	6605.096	5682.118
TJP1	0.99468	0.00532	1.064913	1.064925	3460.929	3249.927
RPL34	0.99468	0.00532	1.094799	1.094814	3882.154	3545.947
STK38	0.994684	0.005316	0.976089	0.976084	2976.355	3049.281
SRP14	0.994685	0.005315	1.064996	1.065007	3469.821	3258.025
PPM1G	0.994687	0.005313	0.894587	0.894575	4261.144	4763.32
PRKACA	0.994701	0.005299	0.948805	0.948796	3110.835	3278.719
PARD3	0.994702	0.005298	1.059213	1.059224	3330.895	3144.655
COPB1	0.994702	0.005298	1.159289	1.159305	6499.523	5606.394
PITPNB	0.994703	0.005297	1.043444	1.043452	3223.088	3088.87
KIF11	0.994704	0.005296	1.04577	1.045778	3171.963	3033.112
VARS	0.994706	0.005294	1.016684	1.016687	2957.461	2908.919
TIPRL	0.994707	0.005293	0.947023	0.947014	3213.207	3392.989
CCDC85C	0.99471	0.00529	1.005153	1.005154	2923.007	2908.019
PPP2R1A	0.99471	0.00529	1.197637	1.197651	9740.394	8132.915
CD2AP	0.99471	0.00529	1.111775	1.111791	4464.532	4015.621
RPS26	0.994712	0.005288	0.896233	0.89622	4081.096	4553.677
TTC1	0.994713	0.005287	0.954756	0.954748	3071.936	3217.536
YTHDF3	0.994716	0.005284	0.990914	0.990912	2988.58	3015.99
CPA4	0.994717	0.005283	1.10947	1.109485	4409.773	3974.61
FAM83H	0.994721	0.005279	0.944378	0.944369	3186.389	3374.094
CDV3	0.994722	0.005278	0.818879	0.81887	10183.85	12436.46
KMT2A	0.994723	0.005277	0.938594	0.938584	3259.764	3473.067
KIF1B	0.994724	0.005276	1.040505	1.040512	3148.323	3025.743
SLC48A1	0.994726	0.005274	1.081558	1.081571	3739.827	3457.771
GNG5	0.99473	0.00527	1.099182	1.099197	4247.807	3864.462
VPS26A	0.994731	0.005269	0.926658	0.926647	3454.261	3727.699
CCNI	0.994735	0.005265	1.082606	1.082619	3776.57	3488.363
MARCH6	0.994737	0.005263	0.9498	0.949791	3150.846	3317.409
CTSC	0.994737	0.005263	0.934766	0.934756	3334.173	3566.894
SSR4	0.994741	0.005259	0.940855	0.940845	3335.34	3545.048
LITAF	0.994742	0.005258	0.853468	0.853457	6382.825	7478.791
FKBP8	0.994744	0.005256	1.16738	1.167395	7134.138	6111.158
TRPC4AP	0.994746	0.005254	0.928186	0.928175	3534.282	3807.777
APEH	0.994751	0.005249	0.989671	0.989669	2965.241	2996.195
MCM3	0.994755	0.005245	0.905358	0.905345	3962.175	4376.424
SNRPE	0.994763	0.005237	1.021002	1.021006	3056.376	2993.496
TGOLN2	0.994764	0.005236	1.173563	1.173578	7501.67	6392.135
SBF1	0.994765	0.005235	0.942265	0.942256	3274.213	3474.867
C6orf106	0.994769	0.005231	1.13312	1.133136	5365.885	4735.428
CLPTM1	0.994781	0.005219	1.02538	1.025385	3106.39	3029.486
GTF2I	0.994781	0.005219	0.892955	0.892942	4395.103	4922.047

EIF1AX	0.994783	0.005217	1.057977	1.057987	3436.478	3248.127
NDUFS5	0.994785	0.005215	1.038563	1.03857	3216.419	3096.968
WDR26	0.994785	0.005215	1.029539	1.029545	3087.496	2998.894
GPX4	0.994791	0.005209	0.898277	0.898265	4236.693	4716.533
MMADHC	0.994794	0.005206	1.044128	1.044136	3285.327	3146.455
PPP1CB	0.994795	0.005205	0.831533	0.831524	8701.226	10464.19
TIMM17A	0.994797	0.005203	1.057095	1.057105	3528.725	3338.103
PPDPF	0.994799	0.005201	0.943721	0.943712	3306.444	3503.659
OSBPL9	0.994799	0.005201	1.045215	1.045223	3246.427	3105.966
RPS29	0.994802	0.005198	1.016699	1.016702	3035.26	2985.398
PDCD10	0.994804	0.005196	1.021276	1.02128	3114.17	3049.281
UGGT1	0.994809	0.005191	0.956581	0.956574	3190.835	3335.692
GTF3C1	0.994811	0.005189	0.90496	0.904948	4074.428	4502.391
ABCD3	0.994818	0.005182	1.079921	1.079934	3864.371	3578.339
COX6B1	0.994834	0.005166	1.101875	1.10189	4527.883	4109.196
SUMO1	0.99484	0.00516	1.111264	1.111279	4829.975	4346.318
LMNA	0.994841	0.005159	1.17447	1.174485	7972.141	6787.777
FLNA	0.994842	0.005158	1.355946	1.355949	82093.16	60542.94
EPRS	0.994846	0.005154	0.866797	0.866785	5762.659	6648.314
ARHGAP3	0.994847	0.005153	0.865646	0.865635	5836.012	6741.889
UBR5	0.994849	0.005151	1.084602	1.084615	4017.746	3704.305
PCYOX1	0.994851	0.005149	1.076832	1.076844	3861.037	3585.51
ANP32E	0.994853	0.005147	0.923732	0.923721	3704.328	4010.223
SLC16A1	0.994857	0.005143	1.028295	1.0283	3236.425	3147.355
AP3M1	0.994859	0.005141	0.982361	0.982358	3199.159	3256.612
ASAH1	0.994859	0.005141	1.02901	1.029015	3322.003	3228.333
MTRNR2L	0.994861	0.005139	1.265186	1.265194	22616.1	17875.59
CTSA	0.994866	0.005134	0.949821	0.949813	3339.786	3516.255
TFG	0.994869	0.005131	0.924677	0.924667	3802.132	4111.895
PANK3	0.994872	0.005128	0.893835	0.893823	4547.888	5088.133
ZNHIT1	0.994881	0.005119	1.045162	1.045169	3428.699	3280.519
PHKB	0.994883	0.005117	0.949012	0.949004	3316.446	3494.661
NUDT21	0.994884	0.005116	1.006627	1.006629	3166.406	3145.555
LAMP2	0.994885	0.005115	1.151682	1.151697	6595.104	5726.422
KHDRBS1	0.994886	0.005114	0.905369	0.905358	4203.351	4642.753
SDHB	0.994888	0.005112	0.961505	0.961499	3267.544	3398.387
PLXNB2	0.994889	0.005111	0.867008	0.866996	5861.574	6760.784
PSME1	0.994894	0.005106	1.01242	1.012423	3277.547	3237.33
SF3B6	0.994896	0.005104	1.056756	1.056765	3599.856	3406.485
ARL8B	0.994897	0.005103	0.917321	0.91731	3915.496	4268.453
KDM5C	0.994899	0.005101	1.027355	1.027359	3235.313	3149.154
CELSR1	0.994901	0.005099	0.92027	0.920259	3857.703	4191.974
UBE2E1	0.994904	0.005096	0.916623	0.916612	3945.504	4304.444
PUF60	0.994905	0.005095	1.120143	1.120158	5171.389	4616.66
HADHA	0.994905	0.005095	0.905848	0.905836	4327.829	4777.717
TSEN34	0.994907	0.005093	0.991095	0.991094	3180.854	3209.438
AUP1	0.994908	0.005092	0.942725	0.942716	3488.715	3700.706
GMPS	0.994909	0.005091	0.856165	0.856154	6553.982	7655.144
ZC3H7B	0.994911	0.005089	1.069998	1.070009	3929.944	3672.813
CHD3	0.994912	0.005088	1.007607	1.007608	3286.438	3261.624
C14orf166	0.994915	0.005085	0.997674	0.997674	3271.99	3279.619
TMCO1	0.994919	0.005081	1.000998	1.000998	3281.992	3278.719
RHOBTB3	0.99492	0.00508	0.802498	0.802491	14612.81	18209.31
DCTN5	0.994923	0.005077	1.036815	1.036821	3489.759	3365.825
RDH11	0.994923	0.005077	0.978358	0.978354	3229.756	3301.213
VAPB	0.994925	0.005075	0.985937	0.985934	3213.085	3258.925

CUTA	0.994927	0.005073	1.000818	1.000818	3293.107	3290.416
SHROOM3	0.994927	0.005073	1.020434	1.020438	3368.682	3301.213
ATP6V0B	0.994934	0.005066	1.000868	1.000868	3227.533	3224.734
RALGAPB	0.994936	0.005064	0.963712	0.963706	3325.337	3450.573
PDS5A	0.994941	0.005059	0.988867	0.988865	3234.202	3270.621
SUMO3	0.994946	0.005054	0.993513	0.993512	3316.446	3338.103
CPNE3	0.994946	0.005054	0.911869	0.911858	4140.001	4540.18
TARDBP	0.994946	0.005054	0.921109	0.921099	4015.356	4359.311
PSMC2	0.994947	0.005053	0.902027	0.902016	4425.633	4906.382
LRRC75A-	0.994952	0.005048	0.955323	0.955316	3417.584	3577.439
H2AFV	0.994957	0.005043	0.983082	0.983079	3266.599	3322.825
PFDN5	0.994961	0.005039	1.005435	1.005436	3355.346	3337.204
UBE4A	0.994962	0.005038	0.974997	0.974993	3301.998	3386.69
NCOR2	0.994964	0.005036	0.960981	0.960975	3469.821	3610.73
IPO5	0.994964	0.005036	1.112333	1.112347	5030.24	4522.185
AP2S1	0.994968	0.005032	1.045798	1.045806	3663.206	3502.759
MTOR	0.994971	0.005029	0.931523	0.931513	3763.233	4039.915
DHX15	0.994974	0.005026	0.980786	0.980783	3298.664	3363.297
OSGIN2	0.994978	0.005022	0.996738	0.996738	3367.571	3378.592
DDX6	0.99499	0.00501	0.937713	0.937704	3695.437	3940.941
GATAD2A	0.994996	0.005004	0.981599	0.981596	3410.916	3474.867
AKIRIN1	0.994997	0.005003	1.023475	1.023479	3417.584	3339.183
GLRX3	0.994998	0.005002	0.984017	0.984014	3408.693	3464.069
HMGXB3	0.995004	0.004996	0.989243	0.989242	3329.783	3365.996
RPS3	0.995007	0.004993	1.250104	1.250113	19606.38	15683.69
SCRN1	0.995007	0.004993	0.858195	0.858184	6817.386	7943.966
ESRP1	0.995007	0.004993	0.873666	0.873655	5787.11	6624.021
MFGE8	0.995015	0.004985	0.885674	0.885663	5198.063	5869.123
INPPL1	0.995017	0.004983	0.886662	0.886651	5283.641	5959.099
NIPSNAP1	0.995019	0.004981	0.919206	0.919196	4098.879	4459.202
ACADVL	0.995019	0.004981	1.110385	1.110399	5091.367	4585.168
TMED7	0.995021	0.004979	1.028205	1.02821	3490.415	3394.653
UBE2V1	0.995023	0.004977	1.063675	1.063685	3930.644	3695.307
SYPL1	0.995029	0.004971	1.125514	1.125528	5838.235	5187.107
SMARCC2	0.995033	0.004967	0.966749	0.966743	3457.595	3576.539
IK	0.995038	0.004962	0.983547	0.983544	3392.022	3448.774
RAD21	0.995046	0.004954	1.044196	1.044203	3778.793	3618.828
SLC7A1	0.995051	0.004949	0.922415	0.922405	4017.746	4355.73
UBXN4	0.995053	0.004947	0.981682	0.981679	3420.919	3484.764
HSPE1	0.995072	0.004928	1.116553	1.116567	5449.764	4880.82
FBL	0.995076	0.004924	1.095897	1.09591	4764.613	4347.632
NUP210	0.995077	0.004923	1.069393	1.069403	4135.555	3867.161
KDELR2	0.99508	0.00492	1.15183	1.151844	7230.831	6277.614
OPA1	0.995083	0.004917	0.970522	0.970518	3602.078	3711.503
TPR	0.995083	0.004917	0.855092	0.855082	7499.792	8770.844
GNB1	0.995086	0.004914	1.158162	1.158176	7667.615	6620.422
PCBD1	0.995086	0.004914	1.097084	1.097097	4740.162	4320.639
TPD52	0.995087	0.004913	1.173442	1.173455	8845.709	7538.175
RAB1A	0.99509	0.00491	0.972937	0.972933	3516.5	3614.329
HMGN4	0.995092	0.004908	1.045259	1.045266	3785.461	3621.527
WHSC1	0.995094	0.004906	1.062721	1.062731	4046.642	3807.777
MCL1	0.995095	0.004905	1.20888	1.208891	12803.44	10591.06
TMEM14C	0.995101	0.004899	0.948625	0.948617	3741.005	3943.641
GOLGB1	0.995103	0.004897	0.986165	0.986163	3577.627	3627.825
FAM53C	0.995111	0.004889	0.981551	0.981548	3517.611	3583.737
PSMC1	0.995113	0.004887	0.92446	0.92445	4165.563	4505.99

NMT1	0.995117	0.004883	1.138931	1.138944	6599.55	5794.443
DKC1	0.995118	0.004882	0.97284	0.972836	3564.291	3663.816
CRKL	0.99512	0.00488	1.017825	1.017828	3524.28	3462.549
SMC4	0.995123	0.004877	1.195126	1.195138	11463.08	9591.424
ECHS1	0.995124	0.004876	0.99851	0.99851	3525.391	3530.652
SMG7	0.995126	0.004874	0.946211	0.946203	3907.716	4129.891
PTP4A2	0.995126	0.004874	0.859395	0.859385	7113.021	8276.877
MRPL3	0.995126	0.004874	1.052149	1.052157	3941.058	3745.694
TPI1	0.995127	0.004873	1.226271	1.226281	15836.48	12914.23
ABCF2	0.995139	0.004861	1.011443	1.011445	3586.53	3545.947
FADS1	0.995139	0.004861	1.002021	1.002021	3557.622	3550.446
NRBP1	0.995145	0.004855	1.014826	1.014828	3699.882	3645.821
MAPK9	0.995146	0.004854	1.008115	1.008116	3586.519	3557.644
COX7A2	0.995147	0.004853	0.997237	0.997236	3502.052	3511.757
USP34	0.995148	0.004852	1.024093	1.024096	3676.543	3590.036
XIST	0.995149	0.004851	1.137589	1.137602	6630.67	5828.634
SARS	0.99515	0.00485	0.912049	0.912039	4553.59	4992.759
NUB1	0.99515	0.00485	0.968183	0.968178	3646.535	3766.388
POLR2B	0.995151	0.004849	0.942081	0.942073	4014.411	4261.255
DLG5	0.995156	0.004844	0.944343	0.944335	3896.602	4126.292
TCOF1	0.995156	0.004844	0.887262	0.887252	5613.73	6327.1
RBCK1	0.995159	0.004841	0.926067	0.926058	4224.468	4561.775
MGEA5	0.995163	0.004837	1.05155	1.051558	4097.767	3896.853
PCNA	0.995167	0.004833	0.955716	0.95571	3781.015	3956.237
DDB1	0.995168	0.004832	1.164005	1.164018	8411.148	7225.959
SF3B1	0.995171	0.004829	1.161132	1.161145	8204.426	7065.802
ARHGAP2	0.995174	0.004826	1.008709	1.008711	3637.644	3606.231
FAM168B	0.995178	0.004822	1.035462	1.035467	3831.029	3699.806
BSG	0.995183	0.004817	1.135951	1.135965	6651.786	5855.627
PATL1	0.995184	0.004816	1.002727	1.002728	3724.334	3714.202
PIP5K1A	0.995188	0.004812	1.00657	1.006571	3653.348	3629.499
CDC42BP	0.995189	0.004811	1.139333	1.139346	6854.063	6015.784
ABCC1	0.995194	0.004806	0.905267	0.905257	4979.115	5500.223
PSMC4	0.995195	0.004805	1.031304	1.031309	3822.138	3706.104
SLC39A7	0.995197	0.004803	1.11706	1.117073	5947.153	5323.87
ABLIM1	0.995207	0.004793	0.923723	0.923714	4376.731	4738.19
TGFBR2	0.995208	0.004792	0.95328	0.953274	3975.512	4170.38
UGP2	0.995208	0.004792	1.086816	1.086827	4932.436	4538.381
RAC1	0.99522	0.00478	1.099582	1.099594	5225.848	4752.523
CSTB	0.995224	0.004776	0.907713	0.907703	4971.335	5476.829
SAE1	0.995226	0.004774	1.040449	1.040455	3977.735	3823.073
EBNA1BP	0.995226	0.004774	0.977083	0.97708	3735.448	3823.073
RPL29	0.995231	0.004769	1.189444	1.189456	11378.61	9566.23
CKS1B	0.995233	0.004767	1.052277	1.052285	4251.142	4039.915
ATP6V1E1	0.995237	0.004763	0.933349	0.93334	4236.693	4539.281
EIF3A	0.995238	0.004762	0.849384	0.849375	8447.824	9945.928
REEP5	0.995239	0.004761	0.95565	0.955643	3916.607	4098.399
EIF4A3	0.995242	0.004758	0.944685	0.944678	4155.56	4398.918
SYNPO	0.995243	0.004757	0.97853	0.978527	3851.034	3935.543
G6PD	0.995243	0.004757	1.09422	1.094232	5114.707	4674.244
MSH6	0.995245	0.004755	0.975909	0.975906	3709.885	3801.479
SLC25A6	0.995251	0.004749	1.088259	1.08827	5078.03	4666.147
INADL	0.995253	0.004747	0.979263	0.97926	3775.492	3855.455
SLC25A39	0.995256	0.004744	1.03177	1.031775	4035.528	3911.249
H2AFZ	0.995258	0.004742	0.891341	0.891331	5568.162	6247.022
SHFM1	0.995258	0.004742	1.056923	1.056931	4381.177	4145.187

ARPC3	0.995259	0.004741	1.104595	1.104607	5498.143	4977.463
KARS	0.995266	0.004734	1.00868	1.008681	3894.379	3860.863
ARF3	0.995274	0.004726	1.119136	1.119149	6118.31	5466.931
NARS	0.995279	0.004721	0.959629	0.959623	4047.754	4218.067
NDRG1	0.995279	0.004721	0.837362	0.837354	10067.15	12022.57
PGRMC1	0.995286	0.004714	1.088971	1.088982	5064.693	4650.851
KIRREL	0.995289	0.004711	1.123797	1.12381	6378.38	5675.675
CDC42	0.995289	0.004711	1.033812	1.033817	4124.441	3989.528
NGFRAP1	0.99529	0.00471	1.048657	1.048664	4316.715	4116.394
UBAP2L	0.995291	0.004709	1.076753	1.076763	4674.589	4341.334
USP22	0.995293	0.004707	0.99286	0.992859	3824.36	3851.865
SNX19	0.995295	0.004705	1.04332	1.043326	4151.115	3978.731
PSMB1	0.995295	0.004705	1.033434	1.033439	4035.528	3904.951
HDAC7	0.995297	0.004703	0.985053	0.985051	3842.143	3900.452
RBBP4	0.995298	0.004702	0.928388	0.928379	4468.978	4813.743
DAP3	0.995303	0.004697	1.016432	1.016434	4006.632	3941.85
IMMT	0.995303	0.004697	1.027354	1.027358	3993.295	3886.956
CLTC	0.995306	0.004694	0.82407	0.824063	12636.73	15334.66
MKLN1	0.995308	0.004692	1.032845	1.03285	4055.534	3926.545
TFDP1	0.995308	0.004692	0.954457	0.954451	4163.34	4362.028
ANP32B	0.995313	0.004687	1.037829	1.037835	4218.911	4065.108
YES1	0.99532	0.00468	1.056916	1.056924	4411.874	4174.258
SEP15	0.995323	0.004677	1.048358	1.048365	4383.399	4181.177
CSNK1D	0.995323	0.004677	1.012802	1.012804	3932.167	3882.457
SERPINH1	0.995323	0.004677	0.95475	0.954744	4093.321	4287.348
CD74	0.995325	0.004675	1.067385	1.067394	4735.716	4436.708
YWHAQ	0.995326	0.004674	1.135135	1.135148	7260.839	6396.382
TPM3	0.995332	0.004668	1.188558	1.188569	11572.25	9736.285
PPIF	0.995342	0.004658	1.023442	1.023445	4038.862	3946.34
PRELID1	0.995343	0.004657	0.928009	0.928	4579.007	4934.275
CAPNS1	0.995352	0.004648	1.073459	1.073469	4823.518	4493.393
LEPROT	0.995357	0.004643	0.985778	0.985776	3956.251	4013.336
PTGES3	0.995364	0.004636	1.217646	1.217656	16093.21	13216.55
LY6E	0.99537	0.00463	0.885641	0.885632	6177.214	6974.926
TNPO3	0.995374	0.004626	1.023862	1.023865	4107.77	4012.022
ZFAND5	0.995382	0.004618	0.963069	0.963064	4227.802	4389.948
RUVBL2	0.995384	0.004616	1.038748	1.038753	4383.399	4219.866
MYO10	0.995391	0.004609	0.999397	0.999397	4022.191	4024.619
OGT	0.995391	0.004609	0.91479	0.914781	4955.775	5417.445
PDHA1	0.995391	0.004609	0.983954	0.983952	4029.971	4095.7
H3F3AP4	0.995397	0.004603	1.085529	1.085539	5252.777	4838.864
UBA1	0.995397	0.004603	0.828301	0.828294	12129.92	14644.47
ERGIC3	0.995402	0.004598	0.939286	0.939278	4486.76	4776.817
PTP4A1	0.995403	0.004597	0.930762	0.930754	4657.918	5004.456
UBE2G2	0.995404	0.004596	0.973732	0.973728	4097.367	4207.918
AK2	0.995406	0.004594	0.863286	0.863277	7821	9059.666
LINC0065	0.995408	0.004592	1.044811	1.044817	4415.119	4225.733
STARD7	0.995409	0.004591	0.93742	0.937412	4645.692	4955.869
NME2	0.995412	0.004588	0.891378	0.891369	6011.148	6743.724
TAX1BP1	0.995418	0.004582	1.148842	1.148853	8514.509	7411.309
MLLT4	0.995421	0.004579	0.880851	0.880842	6770.707	7686.635
OLA1	0.995427	0.004573	1.026982	1.026986	4247.807	4136.189
PAICS	0.995435	0.004565	1.098423	1.098434	5773.795	5256.388
GNPDA1	0.995439	0.004561	0.9531	0.953094	4358.948	4573.471
IRAK1	0.995448	0.004552	1.149713	1.149724	8712.34	7577.764
AAMP	0.995451	0.004549	1.035344	1.035349	4386.734	4236.962

MLF2	0.995452	0.004548	1.002502	1.002503	4155.56	4145.187
SEPT11	0.995455	0.004545	1.049084	1.04909	4697.928	4478.097
ERP44	0.995459	0.004541	0.967102	0.967098	4261.144	4406.116
PFKP	0.995469	0.004531	0.919046	0.919038	5116.93	5567.704
COX7A2L	0.995471	0.004529	0.977044	0.977041	4151.115	4248.659
NDST1	0.99548	0.00452	1.100544	1.100555	6130.535	5570.404
ANKRD52	0.995484	0.004516	0.965898	0.965893	4324.495	4477.197
UBA2	0.995484	0.004516	0.879502	0.879493	6795.158	7726.225
PSMD2	0.995485	0.004515	1.146557	1.146569	8436.71	7358.223
HNRNPL	0.995492	0.004508	1.088529	1.088539	5608.173	5152.016
SUB1	0.995493	0.004507	0.945073	0.945067	4521.214	4784.015
LIMCH1	0.995499	0.004501	0.973506	0.973503	4406.739	4526.684
COX4I1	0.995502	0.004498	0.987516	0.987515	4257.81	4311.642
TPMT	0.995514	0.004486	0.969583	0.969579	4288.863	4423.428
YARS	0.995517	0.004483	0.871885	0.871877	7749.859	8888.712
CBX3	0.995521	0.004479	1.134017	1.134028	7758.75	6841.762
UBC	0.995528	0.004472	1.292384	1.292389	44844.99	34699.3
HSD17B4	0.995531	0.004469	1.009261	1.009262	4353.391	4313.441
EPCAM	0.99554	0.00446	1.139929	1.13994	8216.651	7207.964
RDX	0.99554	0.00446	0.940492	0.940485	4794.621	5098.031
ATXN10	0.995543	0.004457	1.006688	1.006688	4474.535	4444.806
WNK1	0.995543	0.004457	0.946837	0.946831	4692.371	4955.869
BAG6	0.995546	0.004454	1.033985	1.03399	4591.233	4440.307
PRKCSH	0.995552	0.004448	1.184892	1.184902	12768.98	10776.41
ARF1	0.995562	0.004438	1.115925	1.115935	6924.082	6204.733
SNX2	0.995566	0.004434	1.140944	1.140955	8400	7362.254
STAU1	0.995575	0.004425	1.09874	1.09875	6370.6	5798.043
ATP2A2	0.995578	0.004422	1.152693	1.152704	9562.568	8295.772
KPNB1	0.99558	0.00442	1.130441	1.130452	7782.09	6884.051
MDH1	0.995581	0.004419	0.920089	0.920082	5522.594	6002.288
RAD23B	0.995596	0.004404	1.070643	1.070651	5393.671	5037.747
BCAP31	0.995597	0.004403	0.945479	0.945473	4964.667	5250.989
ZNF146	0.995597	0.004403	0.936871	0.936864	5011.346	5349.063
UQCRC2	0.9956	0.0044	0.90018	0.900171	6192.774	6879.552
AHCTF1	0.995605	0.004395	1.020222	1.020224	4596.679	4505.558
SEPT2	0.995607	0.004393	1.141829	1.14184	8642.321	7568.767
SEC62	0.995609	0.004391	1.019777	1.01978	4602.458	4513.188
MORF4L2	0.995614	0.004386	1.149916	1.149927	9513.666	8273.278
CYFIP1	0.995623	0.004377	1.112148	1.112159	7130.804	6411.678
UBE2D3	0.995631	0.004369	1.013484	1.013485	4612.35	4550.978
ANXA1	0.995636	0.004364	1.081696	1.081704	5826.009	5385.953
CD47	0.995645	0.004355	1.123314	1.123324	7630.938	6793.175
HNRNPUL	0.995646	0.004354	0.946465	0.946459	4961.332	5241.992
RPL38	0.995654	0.004346	0.934393	0.934387	5346.991	5722.463
APH1A	0.995658	0.004342	0.944325	0.944319	5029.128	5325.669
GIPC1	0.995663	0.004337	1.080204	1.080213	5862.686	5427.342
TK1	0.995669	0.004331	0.947241	0.947235	5008.012	5286.98
FKBP4	0.995684	0.004316	1.050883	1.050888	5208.065	4955.869
NPTX2	0.995689	0.004311	1.003495	1.003495	4822.406	4805.609
CAPZA2	0.995691	0.004309	1.064818	1.064825	5528.151	5191.605
CNN3	0.995696	0.004304	1.16367	1.16368	10971.84	9428.567
WASF2	0.995709	0.004291	1.033604	1.033608	5002.455	4839.8
PYCR1	0.995718	0.004282	0.89474	0.894732	6871.845	7680.337
MATR3	0.995718	0.004282	0.875545	0.875538	8122.237	9276.859
STT3A	0.995718	0.004282	0.882766	0.882759	7779.867	8813.133
KPNA2	0.995722	0.004278	0.897334	0.897327	6752.424	7525.048

LSM4	0.995724	0.004276	0.990441	0.99044	4784.618	4830.802
RPL36	0.995726	0.004274	0.990346	0.990344	4791.287	4838
FLII	0.995728	0.004272	1.027254	1.027257	4979.115	4846.998
HNRNPH3	0.99573	0.00427	1.038247	1.038252	5125.821	4936.974
DDX21	0.995735	0.004265	1.147758	1.147768	9728.168	8475.723
PRMT1	0.99574	0.00426	1.034353	1.034357	5099.147	4929.776
EIF3L	0.995741	0.004259	1.104529	1.104538	7054.117	6386.484
COPA	0.995756	0.004244	1.19104	1.191049	14800.64	12426.56
RPS13	0.99577	0.00423	1.06614	1.066147	5942.707	5574.003
SCRIB	0.99577	0.00423	0.929174	0.929167	5833.789	6278.513
WLS	0.995771	0.004229	0.911303	0.911296	6443.953	7071.201
PRSS8	0.995772	0.004228	1.051073	1.051079	5468.135	5202.403
UQCRQ	0.995774	0.004226	0.931001	0.930994	5659.298	6078.767
CS	0.99578	0.00422	1.071125	1.071132	5970.492	5574.003
UHMK1	0.995785	0.004215	1.090141	1.090149	6615.11	6068.078
GHITM	0.995796	0.004204	0.874217	0.87421	8792.361	10057.5
SERF2	0.995806	0.004194	1.017722	1.017724	5105.327	5016.413
S100A14	0.995808	0.004192	0.882421	0.882413	8005.483	9072.263
TOMM20	0.995815	0.004185	0.892703	0.892696	7365.1	8250.406
NPLOC4	0.995821	0.004179	1.039954	1.039959	5415.899	5207.801
MORF4L1	0.995821	0.004179	1.047884	1.047889	5688.194	5428.242
CKAP5	0.995822	0.004178	1.005676	1.005677	5073.585	5044.945
RAB6A	0.995823	0.004177	0.897485	0.897478	7298.593	8132.339
UBR4	0.995825	0.004175	0.959771	0.959767	5262.524	5483.127
PTPN11	0.995828	0.004172	1.025078	1.025081	5236.962	5108.828
PDAP1	0.995832	0.004168	0.954817	0.954812	5351.437	5604.702
TCP1	0.995836	0.004164	1.10108	1.101088	7444.221	6760.784
GAA	0.995836	0.004164	0.914769	0.914762	6386.159	6981.225
CNOT1	0.995841	0.004159	0.948497	0.948492	5385.891	5678.375
RALY	0.99585	0.00415	0.99357	0.993569	5250.299	5284.281
AFF4	0.995866	0.004134	0.916377	0.91637	6432.839	7019.914
LAMP1	0.995875	0.004125	1.180639	1.180647	14231.6	12054.06
PSMD8	0.995892	0.004108	0.978768	0.978766	5289.198	5403.948
ENAH	0.995897	0.004103	0.89859	0.898583	7545.915	8397.57
TAGLN2	0.995898	0.004102	1.147016	1.147025	10839.58	9450.161
PFN1	0.995901	0.004099	1.168949	1.168957	12899.02	11034.64
WDR45B	0.995904	0.004096	0.993366	0.993365	5285.864	5321.171
SEC61A1	0.995907	0.004093	1.199056	1.199064	17502.48	14596.78
DNM2	0.995911	0.004089	1.086231	1.086238	6944.087	6392.783
NOLC1	0.995912	0.004088	0.955668	0.955664	5584.833	5843.93
RANBP2	0.995922	0.004078	1.039684	1.039688	5726.916	5508.302
MRFAP1	0.995926	0.004074	0.995633	0.995632	5353.471	5376.956
PSMA7	0.995926	0.004074	1.026376	1.026379	5549.268	5406.648
U2AF2	0.995929	0.004071	1.017844	1.017845	5469.247	5373.357
SCAF11	0.995929	0.004071	1.097843	1.097851	7380.049	6722.265
MCFD2	0.995932	0.004068	1.032255	1.032259	5644.216	5467.831
RAB10	0.995934	0.004066	0.971402	0.971399	5467.024	5627.988
ATP5E	0.995945	0.004055	1.027758	1.027761	5761.747	5606.115
PSMB4	0.995947	0.004053	1.024336	1.024338	5592.613	5459.733
SF3B2	0.995948	0.004052	0.974915	0.974912	5485.918	5627.088
MAPRE1	0.995954	0.004046	1.060257	1.060263	6425.059	6059.872
CD164	0.99596	0.00404	0.999156	0.999156	5465.912	5470.53
CYR61	0.995961	0.004039	1.313169	1.313172	75969.29	57851.76
GSPT1	0.995962	0.004038	1.010162	1.010163	5517.037	5461.533
FAM120A	0.995966	0.004034	0.953346	0.953342	5796.001	6079.667
ATP2B4	0.995967	0.004033	1.025537	1.025539	5669.3	5528.115

YWHAZ	0.995971	0.004029	1.25087	1.250875	33857.98	27067.43
TIMP3	0.995977	0.004023	1.04896	1.048965	6233.896	5942.904
UBA52	0.995977	0.004023	1.142186	1.142194	10632.79	9309.088
USP9X	0.995978	0.004022	0.956413	0.956409	5787.11	6050.875
RPL39	0.995982	0.004018	1.193352	1.193359	17621.4	14766.21
TUG1	0.995994	0.004006	1.013619	1.01362	5642.626	5566.805
RPLP1	0.996001	0.003999	0.859648	0.859642	11495.31	13372.21
PRDX3	0.996002	0.003998	0.994033	0.994032	5592.613	5626.189
HP1BP3	0.996009	0.003991	0.97405	0.974048	5688.35	5839.908
EIF3CL	0.996013	0.003987	0.841634	0.841629	14065.24	16711.93
ACTR3	0.996016	0.003984	1.100046	1.100054	8004.372	7276.345
FAU	0.996017	0.003983	0.95504	0.955036	6083.856	6370.289
PABPC4	0.996019	0.003981	0.971778	0.971776	5739.319	5906.014
CDC37	0.99602	0.00398	1.053466	1.053471	6345.037	6022.982
DUSP1	0.996023	0.003977	1.07312	1.073126	7085.236	6602.426
TRAM1	0.996027	0.003973	1.192161	1.192168	17445.8	14633.67
SMG1	0.99604	0.00396	1.011169	1.01117	5916.767	5851.407
RAB13	0.996041	0.003959	0.989493	0.989492	5723.759	5784.546
DNMT1	0.996042	0.003958	1.008234	1.008235	5667.077	5620.79
TUBA1B	0.996044	0.003956	1.268194	1.268198	44047.04	34731.98
MIF	0.996046	0.003954	1.085421	1.085428	7473.118	6884.951
RPL21P28	0.996047	0.003953	1.023194	1.023197	5913.177	5779.121
GART	0.996049	0.003951	0.942051	0.942046	6467.292	6865.156
CCT3	0.996056	0.003944	1.140657	1.140666	10992.95	9637.311
FUS	0.996068	0.003932	1.020597	1.020599	5956.044	5835.832
RHOA	0.996077	0.003923	1.051093	1.051097	6395.051	6084.166
BBX	0.996095	0.003905	1.038709	1.038713	6315.029	6079.667
TMED2	0.996095	0.003905	0.930771	0.930766	6968.538	7486.889
LSM14A	0.996097	0.003903	0.925108	0.925103	7005.215	7572.366
DSP	0.996116	0.003884	1.164572	1.16458	14362.75	12332.99
AP1M2	0.996121	0.003879	0.98793	0.987929	6002.723	6076.068
CTDSP2	0.996123	0.003877	1.016237	1.016238	6111.641	6013.985
NAMPT	0.996124	0.003876	1.199036	1.199043	19848.66	16553.75
NRDC	0.996128	0.003872	0.979074	0.979072	6066.073	6195.736
KIAA1191	0.996131	0.003869	1.082522	1.082529	7737.122	7147.266
EIF3I	0.996131	0.003869	1.010888	1.010889	6097.526	6031.845
HDGF	0.996133	0.003867	1.130362	1.13037	10602.85	9379.98
EPN1	0.996138	0.003862	1.057842	1.057847	6919.636	6541.243
BTF3	0.996147	0.003853	0.981097	0.981096	6122.755	6240.733
PTBP1	0.996154	0.003846	1.023328	1.02333	6193.886	6052.674
CALU	0.996167	0.003833	0.864651	0.864646	11720.93	13555.76
TES	0.996176	0.003824	0.964232	0.964229	6386.159	6623.076
HMGN1	0.996204	0.003796	0.883737	0.883732	9956.007	11265.87
NUCKS1	0.996213	0.003787	0.959198	0.959195	6766.262	7054.105
MLEC	0.996217	0.003783	1.131562	1.131569	11258.58	9949.527
LARS	0.996242	0.003758	0.890155	0.890149	9876.13	11094.92
ESYT1	0.996242	0.003758	1.027797	1.027799	6712.914	6531.346
COL18A1	0.996248	0.003752	1.122059	1.122067	10678.42	9516.744
HECTD1	0.996248	0.003752	0.934679	0.934674	7334.192	7846.792
CNBP	0.996248	0.003752	1.117396	1.117403	10326.05	9241.112
SERP1	0.996249	0.003751	1.004161	1.004161	6510.637	6483.658
RPL23A	0.996253	0.003747	1.068979	1.068984	7975.475	7460.796
CAND1	0.996254	0.003746	0.980409	0.980407	6696.243	6830.065
AHNAK	0.996273	0.003727	1.123034	1.123041	10925.16	9728.187
SYNCRIP	0.996281	0.003719	1.028794	1.028797	6774.041	6584.431
PDXDC1	0.996287	0.003713	1.11724	1.117248	10818.25	9682.947

KHSRP	0.996287	0.003713	0.964591	0.964588	6832.946	7083.797
FAM3C	0.996305	0.003695	1.021449	1.02145	7057.451	6909.244
CCT4	0.996307	0.003693	1.1148	1.114807	10515.05	9432.166
SEPT9	0.996307	0.003693	1.034803	1.034805	7109.687	6870.554
HNRNPAE	0.996316	0.003684	1.067877	1.067882	8056.608	7544.473
SEL1L	0.996318	0.003682	0.917202	0.917197	8368.914	9124.449
CAPRIN1	0.996321	0.003679	0.966961	0.966958	6946.31	7183.67
TARS	0.996323	0.003677	0.913702	0.913696	8776.801	9605.82
NAA50	0.996323	0.003677	1.097931	1.097938	9509.32	8661.073
RPL27	0.996324	0.003676	0.989027	0.989026	6799.604	6875.053
RPL24	0.996325	0.003675	1.116507	1.116514	10766.22	9642.71
BCAM	0.996327	0.003673	1.001026	1.001026	6982.986	6975.826
DYNC1H1	0.996334	0.003666	1.125421	1.125428	11824.29	10506.48
NACA	0.996335	0.003665	0.86699	0.866985	12685.63	14631.89
SSR1	0.99634	0.00366	0.939859	0.939855	7782.468	8280.503
CLDN1	0.996347	0.003653	1.123927	1.123933	11515.31	10245.55
GOLGA4	0.996357	0.003643	1.111322	1.111329	10417.24	9373.682
DDOST	0.996371	0.003629	0.938394	0.93839	7987.856	8512.298
MYBL2	0.996378	0.003622	0.996724	0.996723	7031.888	7055.005
GLUD1	0.996382	0.003618	1.093939	1.093945	9870.251	9022.623
COPB2	0.996383	0.003617	1.176867	1.176873	18580.55	15788.06
CTNNB1	0.996387	0.003613	0.964866	0.964863	7274.176	7539.075
ST13	0.996392	0.003608	0.991559	0.991558	7095.317	7155.724
FASN	0.996393	0.003607	0.895896	0.895891	10378.34	11584.39
SOD1	0.996395	0.003605	1.038363	1.038366	7773.199	7485.989
EIF1	0.996397	0.003603	0.899328	0.899323	9902.659	11011.24
MTRNR2L	0.996399	0.003601	1.243434	1.243439	39052.18	31406.59
NONO	0.996403	0.003597	0.905665	0.90566	9501.441	10491.18
BZW1	0.99641	0.00359	1.061318	1.061323	8315.566	7835.095
STT3B	0.996412	0.003588	1.049664	1.049667	8147.744	7762.215
AHCY	0.996412	0.003588	0.938044	0.93804	8212.205	8754.648
MTHFD2	0.996414	0.003586	1.084666	1.084672	9328.061	8599.89
KIF5B	0.996417	0.003583	0.894263	0.894258	10420.58	11652.77
SND1	0.996418	0.003582	1.044919	1.044923	7859.889	7521.979
SNRNP20	0.996419	0.003581	0.915669	0.915664	9009.086	9838.857
XRCC5	0.996421	0.003579	1.105729	1.105736	10638.41	9621.116
COPG1	0.996429	0.003571	0.936671	0.936667	8155.524	8706.961
AHCYL1	0.996434	0.003566	0.981293	0.981291	7498.68	7641.647
RPL11	0.996434	0.003566	1.165124	1.16513	17585.83	15093.45
NCKAP1	0.996437	0.003563	0.998037	0.998037	7183.04	7197.167
YME1L1	0.99644	0.00356	1.090098	1.090103	10013.3	9185.642
AP2M1	0.996442	0.003558	0.922031	0.922026	9030.203	9793.869
ADGRG1	0.996448	0.003552	0.919565	0.91956	8822.369	9594.123
GLO1	0.996453	0.003547	0.959858	0.959855	7674.283	7995.252
NOMO1	0.996473	0.003527	0.967296	0.967294	7836.56	8101.532
RTN3	0.996475	0.003525	1.019094	1.019095	7650.944	7507.583
STIP1	0.99648	0.00352	1.035932	1.035935	7966.584	7690.234
ATP5L	0.996489	0.003511	1.005372	1.005373	7593.15	7552.571
RARS	0.996505	0.003495	0.925971	0.925966	9004.64	9724.588
RPLP2	0.99651	0.00349	0.995404	0.995404	7846.552	7882.783
SLC25A3	0.99651	0.00349	0.953322	0.953319	8302.23	8708.761
RPS28	0.996514	0.003486	0.993245	0.993244	7549.805	7601.158
GFPT1	0.996525	0.003475	0.976089	0.976088	7814.676	8006.121
TWF1	0.996537	0.003463	0.911759	0.911755	9964.898	10929.36
ARPC1B	0.996542	0.003458	1.076191	1.076196	9942.67	9238.718
LARP1	0.996544	0.003456	0.871412	0.871407	13884.84	15933.82

YAP1	0.996544	0.003456	0.940576	0.940573	8865.714	9425.868
IGF2R	0.99655	0.00345	0.970211	0.970209	8011.974	8257.991
CD63	0.996558	0.003442	0.949004	0.949001	8478.944	8934.6
TFRC	0.996559	0.003441	1.147063	1.147069	15892.05	13854.48
SKP1	0.996564	0.003436	1.073974	1.073979	9978.235	9290.904
CALM1	0.996566	0.003434	0.916593	0.916589	9865.983	10763.81
PRDX6	0.996569	0.003431	1.113197	1.113203	12328.87	11075.12
ADAR	0.99657	0.00343	1.081605	1.08161	10172.73	9405.174
H2AFY	0.996594	0.003406	1.039162	1.039165	8676.775	8349.757
RPL18A	0.996597	0.003403	1.164281	1.164286	18987.32	16308.12
TKT	0.99661	0.00339	1.148982	1.148988	17116.82	14897.3
RPL27A	0.99661	0.00339	1.027146	1.027148	8711.339	8481.095
LAMA5	0.996615	0.003385	0.879787	0.879782	13438.05	15274.3
MAL2	0.996617	0.003383	1.044894	1.044897	8969.075	8583.694
RPL13	0.996622	0.003378	1.091997	1.092002	11430.85	10467.79
VPS35	0.996624	0.003376	0.927922	0.927918	9658.149	10408.4
RPS12	0.996626	0.003374	1.079688	1.079693	10708.43	9918.036
EIF5A	0.996667	0.003333	1.088282	1.088287	11269.42	10355.18
SRRM2	0.996691	0.003309	0.969715	0.969713	9043.54	9325.995
SON	0.996697	0.003303	0.97552	0.975518	9004.64	9230.62
PRRC2A	0.996718	0.003282	0.953738	0.953736	9557.011	10020.61
MYL12B	0.996726	0.003274	0.987257	0.987256	8887.353	9002.073
LASP1	0.996729	0.003271	1.039513	1.039516	9763.733	9392.577
RPS9	0.996734	0.003266	1.030515	1.030517	9567.014	9283.706
PDIA3	0.996741	0.003259	0.856148	0.856144	18723.33	21869.37
RPL9	0.996778	0.003222	1.102161	1.102166	13016.83	11810.23
RPS7	0.996784	0.003216	1.063671	1.063674	11087.42	10423.7
RPS15A	0.99679	0.00321	0.90405	0.904046	12493.36	13819.39
GNS	0.996794	0.003206	1.039413	1.039415	9986.182	9607.502
PDIA6	0.996799	0.003201	1.091641	1.091646	12911.25	11827.32
CHCHD2	0.996808	0.003192	1.022057	1.022059	9905.994	9692.196
SLC26A2	0.996811	0.003189	1.07675	1.076754	12017.07	11160.46
IARS	0.996818	0.003182	1.073525	1.073529	11606.45	10811.5
ATP1A1	0.996819	0.003181	0.916324	0.91632	11793.17	12870.14
RPL31	0.996824	0.003176	1.072834	1.072838	11417.51	10642.34
TRIM28	0.99683	0.00317	0.888788	0.888784	14447.21	16255.03
NAP1L1	0.996833	0.003167	1.018531	1.018532	10019.36	9837.058
RPL35A	0.996835	0.003165	1.112683	1.112688	15147.4	13613.34
CCT5	0.996844	0.003156	1.034368	1.03437	10210.52	9871.248
RPS23	0.996856	0.003144	0.975235	0.975234	9686.724	9932.72
ATF4	0.996856	0.003144	0.986882	0.986881	9760.399	9890.143
NCOA4	0.996857	0.003143	1.044621	1.044624	10622.85	10169.07
DEK	0.996863	0.003137	1.128849	1.128854	16983.45	15044.86
SCD	0.996869	0.003131	1.071466	1.071469	12216.61	11401.74
RPS17	0.996877	0.003123	1.074813	1.074817	12197.72	11348.65
SET	0.996877	0.003123	0.927427	0.927424	11589.78	12496.74
UBB	0.996882	0.003118	1.073745	1.073749	12173.67	11337.54
HM13	0.996885	0.003115	0.954127	0.954125	10513.86	11019.38
ILF2	0.996891	0.003109	0.955763	0.95576	10509.49	10995.95
RPL36A	0.996906	0.003094	0.997211	0.99721	10121.14	10149.45
RPL22	0.996908	0.003092	1.032785	1.032787	10659.53	10321.13
HUWE1	0.996917	0.003083	0.88542	0.885417	16234.36	18335.27
EIF4H	0.996931	0.003069	1.036439	1.036441	10951.83	10566.76
ATP5B	0.996935	0.003065	0.846048	0.846045	24955.59	29496.78
AARS	0.996942	0.003058	0.9635	0.963498	10722.88	11129.11
RPN1	0.996953	0.003047	0.92965	0.929647	12422.22	13362.31

SLC38A2	0.996954	0.003046	0.983316	0.983315	10771.78	10954.56
RPS10	0.996967	0.003033	0.915324	0.915321	13555.37	14809.43
RPL30	0.996967	0.003033	1.002681	1.002681	10623.96	10595.55
SLC25A5	0.996972	0.003028	1.029492	1.029494	11361.94	11036.44
CAST	0.996973	0.003027	0.994149	0.994149	10894.04	10958.16
PSAT1	0.996976	0.003024	1.057581	1.057584	12458.9	11780.54
RPLP0	0.996977	0.003023	0.853039	0.853036	22694.98	26604.94
EIF4A1	0.996984	0.003016	0.96361	0.963608	11066.23	11484.16
PGAM1	0.996988	0.003012	1.095043	1.095047	14811.74	13526.12
ETF1	0.99699	0.00301	0.950788	0.950786	11494.2	12089.15
DDX3X	0.996999	0.003001	0.93452	0.934517	12288.87	13149.97
CSDE1	0.997002	0.002998	1.014842	1.014843	11305.26	11139.91
CSE1L	0.997009	0.002991	0.999619	0.999619	11213.01	11217.29
RPL5	0.997016	0.002984	1.160945	1.160949	25516.85	21979.3
RPS3A	0.997017	0.002983	1.133512	1.133517	19767.53	17439.12
GAPDH	0.997042	0.002958	1.239421	1.239424	58480.15	47183.33
CCT7	0.997049	0.002951	1.009863	1.009864	11379.72	11268.57
NOTCH2	0.997071	0.002929	1.017523	1.017524	11976.45	11770.19
RPL18	0.997072	0.002928	1.085853	1.085856	14728.4	13563.86
ERGIC1	0.997083	0.002917	1.074155	1.074158	14230.49	13248.04
HSPA4	0.997092	0.002908	0.954947	0.954945	12586.71	13180.56
KTN1	0.997105	0.002895	0.990781	0.990781	11793.17	11902.9
YWHAB	0.99711	0.00289	1.084904	1.084907	15368.57	14165.79
PHGDH	0.997124	0.002876	1.050793	1.050795	13620.32	12961.92
RPS27A	0.99713	0.00287	1.039316	1.039318	13189.14	12690.19
XPOT	0.997143	0.002857	0.939647	0.939644	13468.06	14333.15
RPL26	0.997147	0.002853	1.006562	1.006562	12259.96	12180.03
TXNRD1	0.997167	0.002833	0.909709	0.909706	16042.09	17634.36
RPS2	0.997168	0.002832	0.863734	0.863731	24346.54	28187.63
CYCS	0.997176	0.002824	1.018897	1.018898	12756.37	12519.78
TIMP2	0.997177	0.002823	1.002415	1.002415	12824.56	12793.66
EIF4G1	0.997189	0.002811	0.934876	0.934874	14262.72	15256.3
LDHB	0.997205	0.002795	0.905729	0.905726	16985.67	18753.66
EIF2S2	0.997211	0.002789	0.977421	0.97742	12956.81	13256.14
HNRNPC	0.997219	0.002781	1.015677	1.015677	12925.69	12726.18
RPL6	0.997239	0.002761	0.924499	0.924496	15994.3	17300.55
RPL37	0.997244	0.002756	1.015736	1.015737	13742.58	13529.67
CCT8	0.997247	0.002753	0.998099	0.998099	13561.42	13587.25
CCT6A	0.997262	0.002738	1.003392	1.003392	13449.17	13403.7
SERBP1	0.997264	0.002736	0.97192	0.971919	13640.33	14034.43
GSTP1	0.997271	0.002729	0.955031	0.95503	14220.49	14890.1
HNRNPK	0.997293	0.002707	1.014832	1.014833	13975.98	13771.7
KRT18	0.997296	0.002704	1.129002	1.129005	23931.98	21197.41
HDLBP	0.997299	0.002701	0.951752	0.95175	14701.73	15447.05
RPL12	0.9973	0.0027	0.962684	0.962683	14650.6	15218.51
RPL32	0.997301	0.002699	0.962646	0.962644	14074.89	14621.07
NPM1	0.997305	0.002695	0.83039	0.830388	40390.7	48640.76
GANAB	0.997312	0.002688	0.991797	0.991797	13978.2	14093.81
RPL37A	0.997315	0.002685	1.009024	1.009024	14476.11	14346.65
CTNNA1	0.997321	0.002679	0.912488	0.912485	18353.82	20114.1
PTMA	0.997358	0.002642	1.066005	1.066007	17192.39	16127.84
KRT7	0.997377	0.002623	1.196947	1.19695	47691.27	39844
RPS11	0.997397	0.002603	1.099556	1.099559	21806.97	19832.47
HNRNPA1	0.997404	0.002596	1.011976	1.011977	15326.12	15144.73
RPS8	0.997451	0.002549	0.940808	0.940806	17139.05	18217.41
RPS20	0.997458	0.002542	1.013347	1.013347	16105.44	15893.3

APLP2	0.997565	0.002435	0.897878	0.897876	24669.96	27475.92
RPL15	0.997569	0.002431	1.063803	1.063805	20627.76	19390.55
GPI	0.997688	0.002312	0.967444	0.967443	20722.29	21419.65
RPS14	0.997698	0.002302	0.99485	0.99485	19956.47	20059.79
PSAP	0.997698	0.002302	0.942147	0.942146	21911.44	23256.95
EIF4G2	0.99772	0.00228	0.991771	0.991771	20409.93	20579.27
RPS6	0.997721	0.002279	1.081319	1.081321	25580.2	23656.45
RPS24	0.997738	0.002262	1.022647	1.022648	21899.24	21414.26
RPL17	0.99775	0.00225	1.020451	1.020452	21549.56	21117.67
HNRNPA2	0.997756	0.002244	0.95728	0.957279	22162.62	23151.68
GNB2L1	0.997759	0.002241	1.150753	1.150755	43966.25	38206.44
RPS27	0.997772	0.002228	0.940951	0.940949	23326.26	24790.14
YBX1	0.997784	0.002216	1.032779	1.03278	22928.38	22200.64
RPSA	0.997796	0.002204	1.02874	1.028741	23547.19	22889.33
HSP90AB	0.997834	0.002166	1.125239	1.125241	38692.61	34386.06
RPS5	0.997844	0.002156	0.991794	0.991794	23215.12	23407.21
RPL13AP5	0.997849	0.002151	1.058636	1.058638	26710.12	25230.65
RPL28	0.997855	0.002145	0.959404	0.959403	25038.01	26097.49
RPL7	0.997865	0.002135	0.924881	0.92488	28795.51	31134.34
RPS18	0.997869	0.002131	1.01589	1.01589	24235.26	23856.19
RPL41	0.997884	0.002116	0.966595	0.966594	25425.72	26304.43
RPL4	0.997886	0.002114	1.040112	1.040113	26696.06	25666.51
ACTG1	0.997888	0.002112	1.237462	1.237463	123588.2	99872.22
RPL10	0.997907	0.002093	0.994068	0.994068	24825.56	24973.69
TMBIM6	0.997907	0.002093	0.983907	0.983906	25505.74	25922.94
P4HB	0.997943	0.002057	0.933366	0.933365	28917.77	30982.28
TPT1	0.997944	0.002056	1.043278	1.043279	28058.65	26894.68
PPIA	0.99796	0.00204	1.068679	1.068681	30970.13	28979.78
PTTG1IP	0.997963	0.002037	1.034874	1.034875	27945.28	27003.55
GNAS	0.998021	0.001979	1.001828	1.001828	28303.16	28251.51
PABPC1	0.998074	0.001926	1.115227	1.115229	48266.27	43279.27
HSP90AA	0.998112	0.001888	1.14035	1.140351	58627.97	51412.19
EEF1G	0.998173	0.001827	1.026415	1.026416	35138.33	34234
HSPD1	0.998181	0.001819	0.968011	0.968011	35014.96	36172.08
ENO1	0.998187	0.001813	1.104196	1.104198	51473.8	46616.47
RPS4X	0.998214	0.001786	0.964225	0.964225	36174.16	37516.32
ALDOA	0.998222	0.001778	1.061655	1.061656	41874.58	39442.7
RPL3	0.998281	0.001719	1.003575	1.003575	39120.51	38981.13
HSPA8	0.998314	0.001686	1.058286	1.058287	46623.63	44055.77
RPL8	0.998318	0.001682	1.075875	1.075876	51955.06	48290.93
CALR	0.998328	0.001672	0.910799	0.910798	53366.55	58593.16
EEF1A1	0.99893	0.00107	1.140513	1.140513	218926.6	191954.4

Supplementary Table 3 SKOV3-shSIK3 61 vs SKOV3-shLacz.result

Gene	PPEE	PPDE	PostFC	RealFC	C1Mean	C2Mean
ADD2	0	1	0.245165501	0.244405	118.1257024	483.3495068
ALDH3A1	0	1	0.207050804	0.205822	64.61576884	313.9790853
ANPEP	0	1	0.12415151	0.12364	102.9813816	832.985523
BEND4	0	1	0.231234419	0.230276	89.85630355	390.2452985
CDH2	0	1	0.090788607	0.090288	79.76008966	883.4995083
CFH	0	1	0.165239794	0.164763	140.337373	851.8044587
CMTM3	0	1	0.221672887	0.220836	99.95251743	452.6449275
COL1A1	0	1	0.120511013	0.119718	64.61576884	539.8063139
COL5A2	0	1	0.121117396	0.120591	97.93327465	812.1856466
COL6A2	0	1	0.269075321	0.269014	1565.922773	5820.993958
COL6A3	0	1	0.062498654	0.059161	8.076971105	136.6849015
CRLF1	0	1	0.127668971	0.125712	27.25977748	216.9129959
DSC2	0	1	0.21207228	0.211095	82.78895383	392.2262391
FLJ26245	0	1	0.339475854	0.339333	764.2833908	2252.329464
FN1	0	1	0.116202849	0.116181	2311.023358	19891.61505
IGFBP2	0	1	0.091544164	0.088949	15.14432082	170.3608917
KIF1A	0	1	0.191925188	0.190995	80.76971105	422.9308184
KRT17	0	1	0.037802878	0.037638	107.0198671	2843.640234
KRT81	0	1	0.019497434	0.019198	30.60162428	1594.498709
LAPTM5	0	1	0.231853273	0.230699	74.71198272	323.8837884
LCN2	0	1	0.359380316	0.359338	2640.15993	7347.308691
LOC10106	0	1	0.02492014	0.021016	2.544245898	121.5307059
LYNX1	0	1	0.110553351	0.107983	18.17318499	168.3799511
MME	0	1	0.067283887	0.063228	7.067349717	111.923144
MYEF2	0	1	0.225425007	0.224513	92.78420557	413.3034471
NID1	0	1	0.06234064	0.058564	7.067349717	120.8373767
NID2	0	1	0.11120069	0.110974	212.0204915	1910.61721
NREP	0	1	0.180501748	0.179792	100.9621388	561.5966605
PDPN	0	1	0.082343572	0.07849	9.086592493	115.8850252
RFTN1	0	1	0.123813165	0.122201	32.30788442	264.4555703
SERF1A	0	1	164.4277197	8118.356	81.17355961	0
SLC22A2	0	1	0.177684223	0.176819	81.77933244	462.5496305
TBC1D3K	0	1	0.02492014	0.021016	2.544245898	121.5307059
VASH1	0	1	0.24657598	0.24575	109.0391099	443.7306947
WFDC2	0	1	0.139495497	0.138132	42.4040983	307.0457932
ALPK2	0	1	0.036330057	0.034024	7.067349717	207.9987632
C14orf132	0	1	0.093784589	0.092409	30.28864164	327.865479
CHKB-CP	0	1	0.006151097	0.000122	0	82.03075031
CPAMD8	0	1	0.247391167	0.246629	121.1545666	491.2732692
FEZ1	0	1	0.252362588	0.251877	192.8376851	765.6335425
KCTD12	0	1	0.178747278	0.177964	92.88516771	521.9778485
PLVAP	0	1	0.111151954	0.108361	17.1635636	158.4752481
SAA1	0	1	0.233194817	0.232925	329.0860915	1412.87617
SAA2	0	1	0.150734477	0.150074	96.0048978	639.7744814
SIK3	0	1	0.30465919	0.304287	282.6939887	929.0611421
SLC22A17	0	1	0.114645471	0.111933	18.17318499	162.4371293
TBC1D3E	0	1	512.9099485	4793.212	285.2988119	0.049523515
TBC1D3I	0	1	0.025008093	0.021016	2.544245898	121.5307059
TGFB2	0	1	0.145920553	0.144234	36.34636997	252.0548821
TMEM59L	0	1	0.104407177	0.102728	27.25977748	265.4460406
TMTC1	0	1	0.282641419	0.282211	234.2321621	830.014112
TNFSF10	0	1	0.316308671	0.31596	307.9345234	974.622776
MMP2	0	1	0.281423846	0.28122	481.5894022	1712.52315
RPS6KA2	0	1	0.110631892	0.108971	28.26939887	259.5032188
SHC2	0	1	0.148866303	0.147825	58.55804051	396.1881203

SPARC	0	1	0.124694841	0.12459	505.8203155	4059.937763
FBN2	1.11E-16	1	0.007112941	0.000144	0	69.33292105
HS6ST2	1.11E-16	1	0.089495351	0.085736	10.09621388	117.8659658
SERPINE1	1.11E-16	1	0.329812956	0.329559	423.0313616	1283.64951
SDK1	1.11E-16	1	0.0485642	0.043479	4.038485553	93.10420827
ITGB6	2.22E-16	1	0.265739607	0.264903	113.0775955	426.8926996
TPM1	2.22E-16	1	0.354341657	0.354223	940.9671338	2656.441347
HS3ST3A1	3.33E-16	1	0.031180667	0.025322	2.019242776	80.12904733
LINC0129	7.77E-16	1	0.307740063	0.307217	202.4795694	659.0985569
JMJD7-PL	9.99E-16	1	0.007788583	0.000155	0	64.67771064
ABCG2	1.55E-15	1	4.591410469	4.624085	247.3572401	53.48539624
GALNT14	1.78E-15	1	0.051737153	0.046443	4.038485553	87.16138647
MFAP2	2E-15	1	0.120086131	0.116786	15.14432082	129.7516094
NPIPA8	2.44E-15	1	6.472143777	6.5833	157.7230533	23.94957187
KIF5C	2.78E-15	1	0.20006126	0.198242	42.4040983	213.941585
DMBT1	3E-15	1	5.286526508	5.343438	195.8665493	36.64740113
GYG2	3.33E-15	1	0.158927354	0.156392	25.2405347	161.446659
SERPINA3	3.33E-15	1	0.338090278	0.337769	338.223165	1001.365474
TIMP3	4.33E-15	1	0.033344285	0.026954	2.019242776	75.27574286
KRT16	4.77E-15	1	0.112595346	0.108933	13.12507805	120.5699497
MSC-AS1	1.14E-14	1	0.243527725	0.24221	67.64463301	279.3126248
LDOC1	2.36E-14	1	0.093579006	0.089157	9.086592493	102.018441
P2RY6	2.6E-14	1	0.177356559	0.174965	29.27902026	167.3894808
CCBE1	3.73E-14	1	2.972136545	2.976117	733.8634984	246.5775814
SLC44A2	3.96E-14	1	0.273671761	0.272634	92.88516771	340.7217835
CAMK1D	4.6E-14	1	0.29041683	0.289531	115.4502057	398.7732478
TNC	4.77E-14	1	0.056305824	0.050582	4.048581767	80.22809436
COL16A1	5.27E-14	1	0.222153898	0.220432	48.46182663	219.8844068
TNFRSF6	8.32E-14	1	0.232393836	0.230746	53.49983736	231.8889068
F2R	1.04E-13	1	0.373250139	0.373057	589.8410074	1581.117455
OBSL1	1.38E-13	1	0.342089035	0.341647	247.3572401	724.0337899
ROBO1	2.26E-13	1	0.175981048	0.173275	26.25015609	151.541956
LAMC3	2.75E-13	1	0.196768009	0.194487	33.31750581	171.351362
C1QTNF1	2.98E-13	1	0.263433975	0.262141	72.69273995	277.3316842
KIAA1462	3.01E-13	1	0.282204391	0.281111	91.87554632	326.8551993
ANXA8L1	3.05E-13	1	0.312073058	0.311321	138.5705355	445.1272579
GPC6	3.67E-13	1	0.085326197	0.080277	7.067349717	88.15185677
NLGN4X	4.06E-13	1	0.148302838	0.144997	18.17318499	125.3935401
STEAP3	5.07E-13	1	0.327396872	0.326748	168.6067718	516.0350267
ZBED2	6.47E-13	1	2.881841959	2.885828	660.2923879	228.7986395
MAN1A1	7.81E-13	1	0.186769051	0.184191	28.26939887	153.5228966
EML5	9.28E-13	1	0.198918384	0.196547	32.30788442	164.4180699
CACNA1H	1.43E-12	1	0.334589267	0.333966	173.6548788	519.9969079
BLOC1S5	1.44E-12	1	2.804918499	2.808471	710.1878769	252.8670678
ALOX5AP	1.53E-12	1	0.071444131	0.065463	5.048106941	77.25668346
NPDC1	1.59E-12	1	0.355134198	0.354686	248.3668615	700.2625026
COL27A1	2.54E-12	1	0.303043097	0.302045	105.0006244	347.6550756
TGFBI	3.2E-12	1	0.411364249	0.411309	2161.599392	5255.435416
CDC42EP	4.54E-12	1	0.213490483	0.211152	35.33674859	167.3894808
SALL2	4.82E-12	1	0.131696542	0.127502	13.12507805	103.0089113
SLIT3	6.2E-12	1	0.07414623	0.068081	5.048106941	74.28527256
PNPLA4	6.44E-12	1	0.259793204	0.258205	58.55804051	226.8176989
TNS1	7.73E-12	1	0.257682876	0.25601	56.53879774	220.8748771
FCGRT	9.47E-12	1	0.084297589	0.07853	6.057728329	77.25668346
MMP11	9.47E-12	1	0.084297589	0.07853	6.057728329	77.25668346
RUNX3	1.06E-11	1	0.32915228	0.32836	135.289266	412.0356451

CD74	1.1E-11	1	0.115724148	0.110895	10.09621388	91.12326767
KCNMA1	1.13E-11	1	0.140018592	0.135994	14.13469943	103.9993816
STC1	1.26E-11	1	0.361423725	0.360923	224.1359482	621.0248786
JPH3	1.35E-11	1	0.027401222	0.018714	1.009621388	54.47586654
FFAR4	1.96E-11	1	3.075092468	3.083474	371.5507671	120.4907121
NOTCH3	1.99E-11	1	0.287668038	0.286357	75.72160411	264.4555703
SSC4D	2.41E-11	1	0.260203374	0.258458	54.51955496	210.9701741
DTX1	2.44E-11	1	0.212979245	0.210495	32.30788442	153.5228966
RIMS2	2.49E-11	1	0.251613637	0.249775	49.47144802	198.0940602
TBC1D4	2.8E-11	1	0.233841176	0.231711	40.38485553	174.3227729
DSC3	2.87E-11	1	0.245039606	0.243082	45.62479053	187.7238361
ISYNA1	6.67E-11	1	0.365783328	0.365228	202.933899	555.6538387
FA2H	7.09E-11	1	2.633727463	2.636827	676.4463301	256.5318079
ADAMTS2	8.15E-11	1	0.162915408	0.159057	17.1635636	107.9612628
EEF1E1-B	9.86E-11	1	3.719780944	3.743944	205.0944888	54.77300763
EEF1A2	1.18E-10	1	0.433714791	0.433666	2468.524294	5692.232819
EREG	1.28E-10	1	0.335080841	0.334181	120.1449452	359.5407192
MARCKSL	1.37E-10	1	0.39489973	0.39455	332.1654367	841.8997557
BRSK1	1.51E-10	1	0.1793737	0.175819	20.19242776	114.8945549
INHBE	1.72E-10	1	0.334961579	0.334038	117.116081	350.6264865
OSCAR	1.74E-10	1	0.155579053	0.151471	15.14432082	100.0375004
EGLN3	1.85E-10	1	0.117276585	0.111988	9.086592493	81.21856466
VNN2	1.85E-10	1	0.117276585	0.111988	9.086592493	81.21856466
SLC43A3	1.86E-10	1	0.332870699	0.331896	113.0775955	340.7217835
MT1X	2.01E-10	1	0.236634874	0.234305	37.35599136	159.4657184
SMOC1	2.46E-10	1	0.193893929	0.190674	23.22129193	121.827847
NFKBIZ	2.72E-10	1	0.417006738	0.416791	549.2340352	1317.781116
LTBP2	2.81E-10	1	0.366774011	0.366142	177.6933643	485.3304474
MIR205HG	2.96E-10	1	0.268476625	0.266633	51.4906908	193.1417087
GLIPR2	3.59E-10	1	0.367281302	0.366635	174.6645002	476.4162147
PTGS2	3.66E-10	1	0.030722484	0.021004	1.009621388	48.53304474
FAM25A	4.69E-10	1	2.936324027	2.944528	338.2130688	114.8549361
TRO	5E-10	1	0.218631825	0.21583	29.27902026	135.6944312
DENND2A	5.56E-10	1	0.249392867	0.247158	40.38485553	163.4275996
COL6A1	6.51E-10	1	0.432368122	0.432222	819.8125672	1896.750626
KIAA1324	7.01E-10	1	0.224181404	0.221377	30.47037349	137.6753718
SMARCD3	7.61E-10	1	0.277485178	0.275674	53.50993357	194.132179
SRRM3	9.47E-10	1	0.261441218	0.259296	44.42334108	171.351362
UCA1	1.1E-09	1	2.227930583	2.228188	5175.319236	2322.652855
C6orf223	1.19E-09	1	0.302888635	0.301399	68.65425439	227.8081692
HGD	1.47E-09	1	2.573465547	2.577112	541.1570641	209.9797038
KCNJ15	1.56E-09	1	0.012381595	0.000252	0	39.61881203
CCDC80	1.75E-09	1	0.386694367	0.386124	201.9242776	522.9683188
RBP1	1.81E-09	1	0.140286887	0.135198	11.10583527	82.20903496
GATM	1.85E-09	1	0.153765485	0.148988	13.12507805	88.15185677
FGFBP1	1.99E-09	1	4.922515619	5.00628	114.0872169	22.78081692
CLDN2	2.82E-09	1	0.012695041	0.000259	0	38.62834173
VASN	3.23E-09	1	0.198336042	0.194674	21.20204915	108.9517331
RPS6KA6	3.62E-09	1	0.160790218	0.156064	13.78133195	88.35985553
FXYD6	3.7E-09	1	0.186906415	0.18301	18.57703354	101.5529199
RAB4B-E(	4.53E-09	1	0.012957461	0.000264	0	37.83596549
CLIP3	4.79E-09	1	0.326233877	0.324915	80.76971105	248.6080455
LMO2	5.84E-09	1	0.272383345	0.27023	44.42334108	164.4180699
AKR1C1	6.76E-09	1	2.177240248	2.177582	3647.600536	1675.063563
C2CD4C	8.23E-09	1	0.092529145	0.085099	5.048106941	59.42821805
PGRMC1	8.92E-09	1	0.460869512	0.4608	1751.693108	3801.425014

SAA2-SAA	1.4E-08	1	0.162548176	0.157634	13.06450076	82.93207828
HCLS1	1.61E-08	1	0.082498968	0.074303	4.038485553	54.47586654
CDC20	1.75E-08	1	2.132523712	2.13274	5424.695719	2543.527732
DCHS1	1.88E-08	1	0.416507744	0.416031	253.4149684	609.139235
LTBP3	3.18E-08	1	0.448852668	0.448619	511.8780438	1141.021786
ITGAX	3.29E-08	1	0.252322355	0.249513	32.30788442	129.5138965
AIF1L	3.37E-08	1	0.386435557	0.385638	144.3758585	374.3977737
ALDH2	3.37E-08	1	0.355747929	0.354575	96.92365326	273.369803
LRP1	3.41E-08	1	0.454652097	0.454451	614.8594254	1352.982431
ADGRB2	3.53E-08	1	0.42437881	0.423938	269.5689106	635.8819331
P2RY11	3.72E-08	1	0.014257942	0.000291	0	34.33960533
IFITM1	3.83E-08	1	0.430528115	0.430134	302.7349732	703.8281957
STMN3	4.05E-08	1	0.378690379	0.377772	127.2122949	336.7599023
CNRIP1	4.38E-08	1	0.085582957	0.077107	4.038485553	52.49492594
AMTN	4.49E-08	1	0.45550746	0.455302	587.5996479	1290.582802
FTCD	4.51E-08	1	0.226007153	0.222472	24.23091332	108.9517331
KRT86	5.29E-08	1	0.26049025	0.257761	33.98385593	131.8712158
TP63	5.33E-08	1	0.435474259	0.43509	318.0307373	730.967082
ZSCAN12	5.54E-08	1	2.563735636	2.569208	365.3617879	142.2018211
PCSK6	5.97E-08	1	0.361812241	0.360655	98.94289604	274.3602733
FAM184A	6.02E-08	1	0.176909068	0.172037	14.13469943	82.20903496
GPR75-AS	6.3E-08	1	0.284113589	0.281808	42.57573394	151.1061491
HMOX1	6.79E-08	1	2.131110235	2.131728	1942.511551	911.2326767
FBXL21	7.55E-08	1	0.122186245	0.11523	7.067349717	61.40915865
ALDH4A1	7.84E-08	1	0.338205215	0.336734	73.70236134	218.8939365
RAI2	8.64E-08	1	0.278637773	0.276121	39.37523414	142.6277233
AP1G2	9.1E-08	1	0.404050803	0.403349	166.587529	413.0261154
SCARF2	1E-07	1	0.291642075	0.289406	44.42334108	153.5228966
FLRT3	1.02E-07	1	0.460593667	0.460376	556.3013849	1208.373767
CDH13	1.06E-07	1	0.470698875	0.470552	844.0434805	1793.741715
ATP10A	1.09E-07	1	0.113116552	0.105604	6.057728329	57.44727744
MRC2	1.18E-07	1	0.484144354	0.484118	4543.296247	9384.7061
KRT14	1.2E-07	1	0.058031941	0.047337	2.019242776	42.85764991
ANXA8	1.28E-07	1	0.382518159	0.381554	118.8829185	311.5920519
PALM2-AK	1.28E-07	1	0.281525673	0.278994	39.39542657	141.2311602
SLC2A10	1.44E-07	1	0.387476291	0.386556	125.1930521	323.8837884
BTNL9	1.46E-07	1	0.17519203	0.169997	13.12507805	77.25668346
NBEA	1.5E-07	1	0.338150213	0.336559	69.66387578	207.0082929
RHBDL3	1.85E-07	1	0.393638248	0.392762	132.2604018	336.7599023
DDIT4	1.94E-07	1	0.488140095	0.488117	5316.66623	10892.2019
UNC5B	2.14E-07	1	0.482800672	0.482708	1310.488562	2714.879094
PDZD4	2.22E-07	1	0.355742132	0.354389	81.77933244	230.7795801
BCAT1	2.46E-07	1	2.037950819	2.038031	12892.86513	6326.133811
FAT2	2.67E-07	1	0.269874639	0.267028	33.31750581	124.7992579
SLAIN1	2.67E-07	1	0.423528283	0.422928	196.8761707	465.5210414
EMILIN3	3.24E-07	1	0.268176591	0.265253	32.30788442	121.827847
DPYSL4	3.24E-07	1	0.383320133	0.382272	109.0391099	285.2554466
SLCO2B1	3.25E-07	1	0.092460863	0.0834	4.038485553	48.53304474
CHD5	3.3E-07	1	0.436048387	0.435545	237.2610262	544.7586654
HES2	3.32E-07	1	2.164183953	2.16554	904.7318221	417.7803729
SOX7	3.37E-07	1	0.307419373	0.305261	47.46230146	155.5038372
STAG3L1	3.88E-07	1	3.804487239	3.849688	115.7530922	30.06077363
CDH6	3.89E-07	1	0.255880931	0.252645	28.26939887	111.923144
FAM72A	4.62E-07	1	2.272133818	2.274722	544.1455434	239.2084823
UGT8	4.72E-07	1	0.38985213	0.388836	113.622791	292.2283575
ECEL1	4.74E-07	1	0.140747127	0.133827	8.076971105	60.41868835

BVES	4.77E-07	1	0.392630091	0.391645	117.5704107	300.2115482
GALNT16	5.06E-07	0.999999	0.421563449	0.420885	174.6645002	415.007056
NPIPA7	5.11E-07	0.999999	0.341575029	0.339901	65.04990604	191.3984809
RET	5.29E-07	0.999999	0.258155399	0.254901	28.26939887	110.9326737
LTF	5.3E-07	0.999999	2.350761698	2.354369	429.08909	182.2465353
KCNS3	5.7E-07	0.999999	0.323639683	0.321617	53.50993357	166.3990105
ANXA6	5.83E-07	0.999999	0.47468496	0.474462	544.1859282	1146.964608
DMKN	5.87E-07	0.999999	0.443291373	0.442806	247.3572401	558.6252496
NEDD4	6E-07	0.999999	0.47662448	0.476415	578.5130554	1214.316589
RGPD6	6.01E-07	0.999999	0.142154247	0.135034	8.087067319	59.95316731
ANK3	6.09E-07	0.999999	0.285035445	0.282333	36.34636997	128.7611391
IL33	6.4E-07	0.999999	0.423993323	0.423318	175.6741215	415.007056
SEPT3	6.89E-07	0.999999	0.366420363	0.36503	82.78895383	226.8176989
SRCIN1	6.94E-07	0.999999	0.334011553	0.332181	58.55804051	176.3037135
ZFP91-CN	7.17E-07	0.999999	58.74924011	2932.941	29.31940511	0
SYTL4	7.99E-07	0.999999	0.231912276	0.227807	21.20204915	93.10420827
OSGIN2	8.06E-07	0.999999	0.490586278	0.490461	992.4578246	2023.530824
ADAMTS1	8.44E-07	0.999999	2.716046942	2.726205	224.1359482	82.20903496
SLC16A9	9.24E-07	0.999999	0.17114046	0.165016	11.10583527	67.35198045
ERG	9.89E-07	0.999999	0.04283789	0.029404	1.009621388	34.66646053
FOXS1	1.09E-06	0.999999	0.154804207	0.148107	9.086592493	61.40915865
C4orf19	1.17E-06	0.999999	0.264264989	0.260887	28.26939887	108.387165
DPY19L2	1.17E-06	0.999999	0.12365917	0.115525	6.047632115	52.42559302
SIRPA	1.22E-06	0.999999	0.461284073	0.460888	311.9730089	676.907213
SEZ6L2	1.37E-06	0.999999	0.463453863	0.463077	321.0596014	693.3292105
CACHD1	1.41E-06	0.999999	0.316992856	0.314741	46.44258386	147.5800748
CD200	1.43E-06	0.999999	2.113357337	2.114713	842.0242377	398.1690609
UBE2C	1.46E-06	0.999999	1.994345825	1.994664	3020.787193	1514.42909
LOC10192	1.49E-06	0.999999	2.415586077	2.420816	326.1077084	134.7039609
C1orf116	1.68E-06	0.999998	2.083154426	2.084289	968.2269112	464.5305711
CLIC6	1.73E-06	0.999998	0.418675184	0.417826	142.3566157	340.7217835
ANTXR1	1.94E-06	0.999998	0.495086206	0.494933	813.7548389	1644.180699
SMIM11A	2.11E-06	0.999998	0.017582291	0.00036	0	27.75297783
H19	2.12E-06	0.999998	0.06597523	0.053901	2.019242776	37.63787143
ARNT2	2.38E-06	0.999998	0.464764941	0.464355	294.8094453	634.8914628
ACSS3	2.45E-06	0.999998	0.218771184	0.214033	17.1635636	80.22809436
LOC10026	2.71E-06	0.999997	2.240478178	2.24348	451.2098946	201.1149946
CLDN10	2.89E-06	0.999997	0.225738642	0.221155	18.17318499	82.20903496
ATP2A3	2.96E-06	0.999997	0.201098646	0.1956	14.13469943	72.30433196
PTPRR	3.87E-06	0.999996	1.961977893	1.962323	2724.968127	1388.639362
OLFML2A	3.91E-06	0.999996	0.449223881	0.448612	200.9146562	447.8708606
CDCA3	4.1E-06	0.999996	2.222346113	2.225319	445.2430322	200.0750008
GPR63	4.32E-06	0.999996	0.203749898	0.198317	14.13469943	71.31386166
IRX3	4.34E-06	0.999996	0.210549239	0.205343	15.14432082	73.79003741
SUSD2	4.52E-06	0.999995	0.415660226	0.414665	121.1545666	292.1887387
KRT20	4.57E-06	0.999995	0.117852126	0.108631	5.048106941	46.55210414
PLEKHO2	4.58E-06	0.999995	0.413689462	0.412693	118.1257024	286.2459169
SERPING	5.09E-06	0.999995	0.351754601	0.34987	58.55804051	167.3894808
MDGA1	5.49E-06	0.999995	0.256193317	0.252175	23.22129193	92.11373797
TUB	5.77E-06	0.999994	0.488890338	0.488603	433.1275755	886.4709192
EPHA10	6.27E-06	0.999994	0.464117913	0.463604	240.7644124	519.3431975
NSG1	6.52E-06	0.999993	0.357455462	0.35562	60.57728329	170.3608917
KIAA1549	7.34E-06	0.999993	0.450154461	0.449503	184.760714	411.0451748
MAP2	7.42E-06	0.999993	2.138985875	2.141293	526.0127432	245.6465393
FOLR3	7.48E-06	0.999993	1.931988628	1.932252	3327.944308	1722.308997
FAM133D	7.85E-06	0.999992	2.933987333	2.952779	147.8893409	50.07817841

FOLR2	7.93E-06	0.999992	2.107266878	2.109195	589.3866778	279.4314813
CACNA2D	8.09E-06	0.999992	0.359539733	0.357699	60.57728329	169.3704214
FAM20A	8.36E-06	0.999992	0.176769685	0.170029	10.09621388	59.42821805
PRSS33	8.66E-06	0.999991	0.250462589	0.246134	21.20204915	86.17091617
TEX19	9.21E-06	0.999991	0.445016414	0.444271	164.5682863	370.4358925
TMEM98	9.52E-06	0.99999	0.168235367	0.161096	9.086592493	56.45680714
CENPE	9.8E-06	0.99999	1.93183744	1.932189	2491.745586	1289.592332
RTN4RL2	1.07E-05	0.999989	0.397251951	0.39593	87.83706077	221.8653474
TMPRSS4	1.1E-05	0.999989	1.997370407	1.998361	1001.544417	501.1779722
LRRC75B	1.11E-05	0.999989	0.107733072	0.097297	4.038485553	41.59975263
H3F3A	1.13E-05	0.999989	0.409596121	0.40843	100.63906	246.4191061
TNFSF15	1.16E-05	0.999988	0.109470195	0.099156	4.139447691	41.8374655
SYNGR1	1.22E-05	0.999988	0.475684374	0.475216	264.5208037	556.644309
COCH	1.23E-05	0.999988	0.408116634	0.40692	97.93327465	240.6842831
XYLT1	1.32E-05	0.999987	0.225759876	0.220503	16.15394221	73.29480226
FAM72C	1.38E-05	0.999986	1.991669118	1.992654	976.2634975	489.9262296
CNTF	1.45E-05	0.999986	0.020091526	0.000404	0	24.76175752
NME3	1.45E-05	0.999986	0.437281169	0.436421	138.3181302	316.9504962
MSC	1.51E-05	0.999985	0.233069679	0.228112	17.1635636	75.27574286
PRSS12	1.54E-05	0.999985	0.484109297	0.483706	300.8671737	622.0153489
COL25A1	1.55E-05	0.999984	0.09245046	0.080718	3.028864164	37.63787143
TP53INP1	1.56E-05	0.999984	0.486899459	0.486509	318.0307373	653.7103985
ALPP	1.64E-05	0.999984	1.900252966	1.900479	3759.45649	1978.15738
GAMT	1.66E-05	0.999983	0.414644217	0.413489	101.9717602	246.6271049
RASSF2	1.72E-05	0.999983	0.344420004	0.342177	48.46182663	141.6471577
PTX3	1.93E-05	0.999981	0.379043432	0.377358	67.64463301	179.2751244
FAM72B	2.31E-05	0.999977	1.998340039	1.999561	795.4604993	397.8124916
FMNL1	2.35E-05	0.999976	0.503614091	0.503338	441.2045466	876.5662162
VEGFC	2.37E-05	0.999976	0.259256015	0.254923	21.20204915	83.19950527
CD82	2.47E-05	0.999975	0.389615797	0.388049	73.55091813	189.5562062
COL9A2	2.51E-05	0.999975	0.282762046	0.279053	26.25015609	94.09467857
CAMK4	2.7E-05	0.999973	0.020911167	0.00042	0	23.77128722
TYMP	2.76E-05	0.999972	0.46623887	0.465608	191.8280638	412.005931
LOXL1	2.79E-05	0.999972	0.504640328	0.504358	431.1083327	854.7758696
CD40	3.16E-05	0.999968	0.466706949	0.466066	188.7991996	405.102353
LRRN1	3.34E-05	0.999967	0.51097158	0.510719	492.6952374	964.718073
NUF2	3.4E-05	0.999966	1.94954327	1.950441	1004.573281	515.0445564
CORO2B	3.66E-05	0.999963	0.213192266	0.207178	13.12507805	63.39009925
LINC0155	3.74E-05	0.999963	2.39241179	2.399538	228.1744337	95.08514887
STARD4	4.21E-05	0.999958	0.391613798	0.389943	70.67349717	181.256065
RAB6B	4.23E-05	0.999958	0.470359148	0.469707	189.808821	404.1118827
MTSS1	4.4E-05	0.999956	0.349744529	0.347378	46.44258386	133.7134906
PAPPA	4.49E-05	0.999955	0.242697047	0.237485	17.1635636	72.30433196
CDHR1	4.49E-05	0.999955	0.189284027	0.182172	10.09621388	55.46633684
ITGA11	4.75E-05	0.999953	0.021337932	0.000439	0	22.78081692
ATHL1	4.95E-05	0.99995	0.493211225	0.492777	279.6651245	567.5394823
ANK2	5E-05	0.99995	0.230527935	0.224968	15.14432082	67.35198045
PIR	5.09E-05	0.999949	1.8741493	1.874503	2253.505227	1202.183328
TMOD2	5.17E-05	0.999948	0.501685659	0.501312	333.1750581	664.6154765
AURKA	5.6E-05	0.999944	1.867269169	1.867603	2414.004739	1292.563743
P4HA3	5.7E-05	0.999943	0.078154721	0.064004	2.019242776	31.69504962
TRPC6	5.72E-05	0.999943	1.979532161	1.98089	692.6002723	349.6360162
LY6E	5.93E-05	0.999941	0.519624018	0.519399	539.1378213	1038.012875
FAM20C	6E-05	0.99994	0.531834351	0.531708	962.1691829	1809.58924
SIRPB1	6.55E-05	0.999935	0.022197028	0.000447	0	22.36481939
ADRBK2	6.82E-05	0.999932	0.326553465	0.32359	35.780982	110.5959138

PLK1	6.95E-05	0.99993	1.863031406	1.863385	2211.07084	1186.58342
FRMPD3	7.07E-05	0.999929	0.253008543	0.248049	18.17318499	73.29480226
ATP6V0E2	7.19E-05	0.999928	0.417749433	0.416376	87.83706077	210.9701741
SLC8A1	7.31E-05	0.999927	2.001750565	2.003486	575.4841913	287.2363872
KIF20A	7.78E-05	0.999922	1.88642175	1.887022	1356.931146	719.0814384
SDK2	7.79E-05	0.999922	0.259825987	0.254933	19.18280638	75.27574286
ROS1	8.08E-05	0.999919	0.2201788	0.21386	13.12507805	61.40915865
PGBD5	8.25E-05	0.999917	0.290328815	0.286411	25.2405347	88.15185677
BIRC7	8.26E-05	0.999917	0.241998653	0.236479	16.15394221	68.34245075
C1S	8.44E-05	0.999916	0.529164833	0.528986	677.4559515	1280.678099
LARGE	8.55E-05	0.999915	0.119179951	0.107536	4.038485553	37.63787143
DAB2	9.03E-05	0.99991	0.35045357	0.347921	43.41371969	124.7992579
DYSF	9.11E-05	0.999909	0.435020094	0.433873	103.991003	239.6938128
SH3PXD2	9.11E-05	0.999909	0.515734471	0.515429	406.8774194	789.4048297
GJB2	9.26E-05	0.999907	0.229020626	0.223102	14.13469943	63.39009925
SMOC2	9.63E-05	0.999904	1.839260494	1.839492	3244.923142	1764.027606
CYP26B1	9.64E-05	0.999904	0.277896257	0.27357	22.21167054	81.21856466
CX3CL1	0.000101	0.999899	0.214003056	0.207458	12.11545666	58.43774775
G3BP2	0.000103	0.999897	0.545817358	0.54578	3219.682607	5899.241111
ARG2	0.000105	0.999895	0.196393914	0.188917	10.09621388	53.48539624
TM6SF1	0.00011	0.99989	0.330377599	0.327372	35.33674859	107.9612628
KNSTRN	0.000111	0.999889	1.834002753	1.834231	3261.077084	1777.89419
GFPT2	0.000112	0.999888	0.528723316	0.528515	580.5322982	1098.431564
CLU	0.000113	0.999887	1.855535274	1.855981	1733.519923	934.0134936
RTN1	0.000116	0.999884	0.350771306	0.348119	42.4040983	121.827847
TNFRSF1	0.000117	0.999883	0.298384004	0.294554	26.25015609	89.14232707
C12orf75	0.000119	0.999881	1.827547938	1.827724	4172.765197	2283.034043
NPEPL1	0.000123	0.999877	0.490775506	0.49022	218.7344737	446.2068705
LRCH2	0.000126	0.999874	0.481726886	0.481077	186.7799568	388.2643579
KIF26A	0.000127	0.999873	0.15212094	0.142434	6.057728329	42.59022293
LIMS2	0.000129	0.999871	0.454019274	0.453058	125.1930521	276.3412139
AIM1L	0.000135	0.999865	2.062135849	2.064887	396.7812055	192.1512384
WBSCR17	0.000137	0.999863	0.138220889	0.127637	5.048106941	39.61881203
CREB3L1	0.000138	0.999862	0.122096917	0.110441	4.038485553	36.64740113
RIMKLA	0.000139	0.999861	0.205305426	0.198048	10.78275643	54.48577125
ICA1	0.000156	0.999844	0.430792984	0.429469	91.87554632	213.941585
GNB3	0.000157	0.999843	0.199897792	0.192481	10.09621388	52.49492594
TTK	0.000164	0.999836	1.839349991	1.839767	1793.087585	974.622776
FBLIM1	0.000164	0.999836	0.548032312	0.547954	1532.968731	2797.632888
SSTR1	0.000177	0.999823	0.083613281	0.068269	2.019242776	29.71410902
FILIP1L	0.000182	0.999818	0.057385799	0.039578	1.009621388	25.75222782
KCNH3	0.000187	0.999813	0.439368445	0.43816	98.94289604	225.8272286
ADCY1	0.000189	0.999811	0.336536878	0.33349	35.33674859	105.9803222
CXADR	0.000191	0.999809	0.490475309	0.489867	198.9458945	406.1324421
FBLN5	0.000191	0.999809	0.438747706	0.437527	97.93327465	223.846288
SNAP91	0.000199	0.999801	0.236484881	0.230298	14.13469943	61.40915865
GPRC5B	0.000206	0.999794	1.834122928	1.834571	1660.827184	905.2898549
EFNB3	0.000214	0.999786	0.10624843	0.092944	3.028864164	32.68551993
S1PR3	0.000219	0.999781	0.270466106	0.265444	19.18280638	72.29442725
CHPF	0.000221	0.999779	0.546562338	0.546439	975.294261	1784.827482
BORA	0.000221	0.999779	2.004414936	2.006628	443.2237894	220.8748771
IL27RA	0.000229	0.999771	0.424889146	0.423446	81.77933244	193.1417087
SPTBN5	0.000234	0.999766	0.125708108	0.113508	4.038485553	35.65693083
SPATC1L	0.000246	0.999754	0.252913819	0.247227	16.15394221	65.37103985
ADAMTS1	0.000247	0.999753	2.102514882	2.106102	315.0018731	149.5610154
ARID5B	0.000252	0.999748	0.520809137	0.520453	341.2520292	655.6913391

CENPF	0.000267	0.999733	1.797326399	1.797485	4386.804932	2440.518821
TROAP	0.000272	0.999728	1.842020036	1.842632	1233.757336	669.5579233
TSPAN18	0.000275	0.999725	0.468141977	0.467214	133.2700232	285.2554466
NMU	0.000283	0.999717	1.843564735	1.8442	1187.314752	643.8056955
CDK7	0.000285	0.999715	1.816671372	1.817068	1821.356984	1002.355944
LRRC4B	0.000298	0.999702	0.086102262	0.070623	2.019242776	28.72363872
NEK2	0.000317	0.999683	1.831276581	1.831842	1311.42751	715.9020287
PTTG1	0.000325	0.999675	1.818370914	1.818827	1587.124822	872.604335
CKS2	0.000338	0.999662	1.794580478	1.794818	2927.902026	1631.304585
SERPINB3	0.00036	0.99964	0.14540092	0.134353	5.048106941	37.63787143
EFNA2	0.000363	0.999637	0.186147988	0.177456	8.076971105	45.56163384
SRGN	0.000364	0.999636	0.207831541	0.200028	10.09621388	50.51398534
BGN	0.000367	0.999633	0.14564754	0.134353	5.048106941	37.63787143
GPSM2	0.000465	0.999535	1.800159354	1.800569	1710.298632	949.8610184
SKA3	0.000479	0.999521	1.800674148	1.801114	1630.538542	905.2898549
ANGPT2	0.000492	0.999508	2.17297224	2.178211	237.3316997	108.9517331
FAM69B	0.000501	0.999499	0.473949855	0.47302	130.2411591	275.3507436
ZSWIM5	0.000505	0.999495	0.260754578	0.254951	16.15394221	63.39009925
HLX	0.000531	0.999469	0.08947829	0.073144	2.019242776	27.73316842
L3MBTL4	0.000548	0.999452	0.23914637	0.232616	13.12507805	56.45680714
RINL	0.00055	0.99945	0.239173567	0.232616	13.12507805	56.45680714
KLF10	0.000561	0.999439	1.769747922	1.769892	4613.969744	2606.917832
SPECC1L	0.000581	0.999419	0.414033348	0.412211	64.49461428	156.4744981
APH1B	0.000581	0.999419	0.48591457	0.485102	149.4239654	308.0362635
PCDH7	0.000584	0.999416	0.062070072	0.042875	1.009621388	23.77128722
IFT27	0.00059	0.99941	0.025714613	0.000531	0	18.81893571
NPAS3	0.00059	0.99941	0.025714613	0.000531	0	18.81893571
GUCY1B2	0.000611	0.999389	0.112994046	0.098939	3.028864164	30.70457932
PLA2G4A	0.000611	0.999389	0.112994046	0.098939	3.028864164	30.70457932
AATK	0.000615	0.999385	0.248405967	0.242006	14.13469943	58.43774775
MKX	0.000617	0.999383	0.439822527	0.438396	83.79857522	191.160768
DLL1	0.000626	0.999374	0.113306879	0.098939	3.028864164	30.70457932
SEPT4	0.000632	0.999368	0.288216083	0.283249	20.19242776	71.31386166
DDR2	0.000637	0.999363	0.132992877	0.120183	4.038485553	33.67599023
CCNB1	0.000639	0.999361	1.764790015	1.764929	4712.91264	2670.307931
TMEM151	0.000641	0.999359	0.223285051	0.215781	11.10583527	51.50445564
RPL17-C1	0.000642	0.999358	2.031839049	2.034992	324.0985618	159.2577197
MMP1	0.000648	0.999352	0.562445086	0.562347	1244.863172	2213.701122
UBE2S	0.000658	0.999342	1.767745088	1.767949	3230.788442	1827.417705
NDC80	0.000658	0.999342	1.780502708	1.780848	1959.675114	1100.412504
SMPDL3A	0.000687	0.999313	0.203653773	0.195365	9.086592493	46.55210414
PSRC1	0.000697	0.999303	1.827926024	1.828732	914.7169777	500.1875019
RCN2	0.000704	0.999296	0.561254581	0.561135	1023.756088	1824.446294
NBPF25P	0.000716	0.999284	0.281218913	0.276065	18.72847675	67.86702501
GPR17	0.000729	0.999271	0.233792079	0.226664	12.11545666	53.48539624
P3H3	0.000815	0.999185	0.532219411	0.531835	315.0018731	592.3012399
CENPA	0.000815	0.999185	1.798496931	1.799084	1190.343617	661.6341609
PIANP	0.000818	0.999182	0.216050669	0.208191	10.09621388	48.53304474
IGFBP3	0.000855	0.999145	0.566177921	0.566168	12266.89987	21666.53783
KYNU	0.000865	0.999135	0.459931949	0.458726	99.95251743	217.9034662
PDZD2	0.000892	0.999108	1.771398507	1.771765	1816.308877	1025.136761
VWA5B2	0.000931	0.999069	0.303763457	0.299099	22.21167054	74.28527256
FKBP10	0.000941	0.999059	0.153367772	0.141815	5.048106941	35.65693083
FAXC	0.00095	0.99905	0.353422514	0.350141	34.3271272	98.05655978
FZD8	0.00095	0.99905	0.353422514	0.350141	34.3271272	98.05655978
ST5	0.000964	0.999036	0.549229001	0.548968	472.5028097	860.7186914

HMMR	0.000996	0.999004	1.768431719	1.768809	1753.712351	991.4607711
RDM1	0.001052	0.998948	2.285025513	2.293333	172.6452574	75.27574286
SFRP1	0.001103	0.998897	0.549885947	0.549623	458.3681102	833.9759933
COL15A1	0.001113	0.998887	0.027104482	0.000561	0	17.82846541
LDLRAD4	0.001116	0.998884	0.510320761	0.509681	189.808821	372.4168331
LGI2	0.001133	0.998867	0.37101387	0.368152	39.37523414	106.9707925
RBMS3	0.001155	0.998845	0.393910163	0.391474	48.46182663	123.8087876
PGPEP1	0.001165	0.998835	0.538150517	0.537778	324.0985618	602.6714639
ZNF488	0.001172	0.998828	2.028085232	2.031569	291.7805812	143.6181936
PLAT	0.001191	0.998809	0.527796284	0.527324	256.4438326	486.3209177
NOV	0.00122	0.99878	1.75824939	1.758615	1813.280013	1031.079583
C9orf84	0.001267	0.998733	2.188795245	2.195359	197.8857921	90.13279737
RAB15	0.001282	0.998718	0.513024691	0.512377	191.8280638	374.3977737
RBP7	0.001293	0.998707	0.458311227	0.45697	91.87554632	201.0654711
DIRAS1	0.001319	0.998681	0.327317694	0.323287	26.25015609	81.21856466
JPH1	0.00132	0.99868	0.322880826	0.318628	25.2405347	79.23762406
NEURL1	0.001342	0.998658	1.760557019	1.760966	1594.192172	905.2898549
ABLIM2	0.001532	0.998468	0.280783625	0.275175	17.1635636	62.39962895
BMP8A	0.00154	0.99846	0.275142273	0.269322	16.31548163	60.6068777
RARRES2	0.001545	0.998455	0.520368649	0.519772	207.982006	400.1500015
PET117	0.001557	0.998443	1.794007207	1.794806	888.3254746	494.9380093
CD52	0.001637	0.998363	34.80637334	1717.356	17.1635636	0
MAPK15	0.001723	0.998277	0.385004359	0.382306	42.4040983	110.9326737
SNAP25-A	0.001736	0.998264	0.252491161	0.245537	13.12507805	53.48539624
MYD88	0.001763	0.998237	0.401968794	0.399628	49.47144802	123.8087876
ZNF165	0.001783	0.998217	1.889926306	1.891779	442.214168	233.750991
LOC10050	0.001826	0.998174	1.726199555	1.726344	4238.743955	2455.326352
ITGA2	0.001831	0.998169	0.546375322	0.546022	341.2520292	624.9867598
VNN1	0.001854	0.998146	0.26144748	0.254968	14.13469943	55.46633684
HEG1	0.001878	0.998122	0.56959274	0.569435	774.3796047	1359.915723
PTGFR	0.001881	0.998119	0.067587051	0.046771	1.009621388	21.79034662
CAMK2N1	0.001973	0.998027	0.548978925	0.54864	355.3867286	647.7675767
DACT1	0.001974	0.998026	0.269933427	0.263749	15.14432082	57.44727744
EDARADD	0.002005	0.997995	0.203139725	0.193821	8.056778678	41.60965734
LINC0127	0.002057	0.997943	0.176459334	0.165525	6.057728329	36.64740113
NPFFR2	0.002058	0.997942	3.255105546	3.307847	68.82589003	20.79987632
PLEKHA7	0.002084	0.997916	1.737153374	1.737496	1817.318499	1045.936638
ARL14EPL	0.002104	0.997896	1.862621649	1.864227	485.6278877	260.4936891
C3orf80	0.002105	0.997895	0.028653179	0.000594	0	16.83799511
LRRC38	0.002105	0.997895	0.028653179	0.000594	0	16.83799511
VWA1	0.002205	0.997795	0.495503414	0.494585	132.2604018	267.4269812
REEP2	0.002222	0.997778	0.535050934	0.534566	249.3764829	466.5115117
MYLK2	0.002231	0.997769	0.029269623	0.000594	0	16.83799511
COL7A1	0.002237	0.997763	0.581093631	0.581039	2153.522421	3706.339865
BACE1	0.002284	0.997716	0.568729517	0.568547	653.2250381	1148.945549
XAF1	0.002307	0.997693	0.347533943	0.343806	29.27902026	85.18044587
CCNA2	0.002311	0.997689	1.724993185	1.725249	2378.667991	1378.734659
EVA1C	0.002333	0.997667	0.247407707	0.239994	12.11545666	50.51398534
CDKL5	0.002382	0.997618	0.335432694	0.331368	26.25015609	79.23762406
KHK	0.002404	0.997596	0.321418959	0.316914	23.22129193	73.29480226
LAMA2	0.002446	0.997554	1.774004543	1.774756	889.476443	501.1779722
KCNJ4	0.002448	0.997552	0.162258278	0.150155	5.048106941	33.67599023
SLITRK5	0.00246	0.99754	0.217614963	0.208682	9.086592493	43.58069323
BNIP3	0.002477	0.997523	0.579182274	0.579082	1204.478316	2079.987632
CFI	0.002494	0.997506	0.533259102	0.53274	233.2225407	437.7878729
PIK3CD	0.002508	0.997492	0.530731195	0.530186	222.1268016	418.9689372

C19orf66	0.002632	0.997368	0.536646858	0.536154	245.3379973	457.597279
PLS1	0.002673	0.997327	1.72620267	1.726527	1870.828432	1083.574509
NKAIN4	0.002747	0.997253	0.466161741	0.464803	88.84668216	191.160768
CHPF2	0.002834	0.997166	0.557492493	0.557185	390.7234772	701.2529729
ANKRD34	0.002848	0.997152	0.406822801	0.404414	48.46182663	119.8469064
MED28	0.002889	0.997111	1.73709001	1.737553	1345.82531	774.5477752
AXIN2	0.002952	0.997048	0.405101569	0.402645	47.45220524	117.8659658
PCDH11X	0.00307	0.99693	0.450392042	0.44876	73.56101434	163.9327395
KCNT2	0.003135	0.996865	0.241695535	0.233759	11.10583527	47.54257444
AKAP5	0.003233	0.996767	1.868110264	1.869999	418.3971995	223.7373362
NUP37	0.003295	0.996705	1.727674864	1.728112	1432.65275	829.0236417
LINC0060	0.003375	0.996625	0.070730419	0.048997	1.009621388	20.79987632
PGR	0.003375	0.996625	0.070730419	0.048997	1.009621388	20.79987632
ALDH1A2	0.003381	0.996619	0.453316401	0.451715	74.71198272	165.4085402
PALM2	0.003407	0.996593	0.225145263	0.21629	9.409671338	43.54107442
AMPH	0.003499	0.996501	0.071210504	0.048997	1.009621388	20.79987632
RBFA	0.003532	0.996468	0.493867963	0.49284	118.1257024	239.6938128
PDGFB	0.003554	0.996446	0.500862943	0.499923	129.2315377	258.5127485
SUSD6	0.003601	0.996399	0.570279606	0.570049	529.0416074	928.0706718
TMEM256	0.003665	0.996335	0.366350558	0.362832	32.5098087	89.61775281
KIF14	0.00383	0.99617	1.756705027	1.757459	858.1781799	488.3018583
ZEB2	0.003857	0.996143	0.271041624	0.26441	14.13469943	53.48539624
FAM19A5	0.003949	0.996051	0.359317966	0.355658	30.28864164	85.18044587
SYT7	0.004041	0.995959	0.355759455	0.351991	29.27902026	83.19950527
KLHDC7B	0.00406	0.99594	0.19835138	0.187988	7.067349717	37.63787143
UNC5A	0.00406	0.99594	0.19835138	0.187988	7.067349717	37.63787143
PTPRS	0.004193	0.995807	0.588830403	0.588761	1743.616137	2961.506199
BCL2	0.004302	0.995698	0.401197302	0.398523	43.41371969	108.9517331
SECTM1	0.004346	0.995654	0.339664788	0.335396	25.2405347	75.27574286
DPP4	0.004369	0.995631	1.835542791	1.837236	451.3007605	245.6366346
DEPDC1	0.004399	0.995601	1.711035068	1.711403	1610.346114	940.9467857
SOX17	0.004399	0.995601	1.701899978	1.702185	2083.858545	1224.221292
NAV2	0.004447	0.995553	1.746964967	1.747704	879.3802291	503.1589128
NEIL3	0.004483	0.995517	1.796089064	1.797308	571.4457057	317.9409665
ZNF117	0.004547	0.995453	0.560206733	0.559863	349.076595	623.510959
HNF4G	0.00463	0.99537	1.848155989	1.850033	415.9640119	224.8367583
ATP8B2	0.004687	0.995313	0.575723659	0.575515	570.516854	991.3221052
NRG2	0.004835	0.995165	0.213724465	0.204068	8.076971105	39.61881203
LMO7	0.004855	0.995145	1.719455941	1.719943	1236.7862	719.0814384
CSF2RA	0.004894	0.995106	0.129428664	0.113591	3.028864164	26.74269812
UGT1A3	0.004912	0.995088	5.236551755	5.56814	35.39732587	6.348914628
SREBF1	0.005075	0.994925	0.570694631	0.570424	450.2911391	789.4048297
RELL2	0.005082	0.994918	0.430146642	0.428009	55.52917635	129.7516094
PBK	0.005223	0.994777	1.695354242	1.695627	2101.022109	1239.078346
MN1	0.005283	0.994717	0.483093619	0.481801	95.91403188	199.0845305
TMEM130	0.005314	0.994686	0.130415789	0.11452	3.038960378	26.61393698
LINC0111	0.005418	0.994582	1.822649624	1.824254	455.3392461	249.5985158
KCNK5	0.005443	0.994557	1.698245639	1.698567	1795.106828	1056.831811
PANX2	0.005616	0.994384	0.517604965	0.516763	147.4047227	285.2554466
PLLP	0.005674	0.994326	1.772664135	1.773737	621.9267751	350.6264865
ARHGAP1	0.00584	0.99416	1.684002851	1.684199	2928.225105	1738.641852
FBXO9	0.005845	0.994155	0.589937572	0.589827	1088.371856	1845.24617
FIBCD1	0.005965	0.994035	0.292491667	0.286256	16.15394221	56.45680714
ARHGEF3	0.006082	0.993918	1.782350985	1.783556	563.3990232	315.8807883
KCTD4	0.006134	0.993866	0.324548698	0.319596	21.20204915	66.36151015
TSC2	0.006162	0.993838	0.588597253	0.588472	934.9094054	1588.714362

LIMA1	0.006181	0.993819	1.679482841	1.679601	4717.960747	2808.973773
SH3GL2	0.006199	0.993801	0.240325221	0.231843	10.09621388	43.58069323
SKAP2	0.0062	0.9938	1.68722994	1.687499	2144.435828	1270.773396
COPZ2	0.00627	0.99373	0.074682056	0.051446	1.009621388	19.80940602
DOC2B	0.00627	0.99373	0.074682056	0.051446	1.009621388	19.80940602
UPK3B	0.006288	0.993712	1.760944227	1.761939	656.1832288	372.4168331
SPON1	0.006378	0.993622	1.682194089	1.6824	2712.85267	1612.48565
CCR7	0.00643	0.99357	0.172525907	0.159536	5.048106941	31.69504962
SEPT5	0.006509	0.993491	0.471814345	0.470296	81.39567631	173.0846851
AKR1C2	0.006776	0.993224	1.71407303	1.714634	1061.899584	619.311365
TLN2	0.007406	0.992594	0.554709517	0.554232	257.453454	464.5305711
C1R	0.007529	0.992471	0.581132285	0.580904	531.0608502	914.2040876
NFATC4	0.007554	0.992446	0.032350012	0.000673	0	14.85705451
SOX9	0.007599	0.992401	0.155524428	0.140897	4.038485553	28.72363872
RASGRF2	0.007633	0.992367	0.272325372	0.265176	13.12507805	49.52351504
LOXL4	0.007814	0.992186	0.348783125	0.344459	25.2405347	73.29480226
SLC22A3	0.007848	0.992152	0.476442427	0.474948	82.78895383	174.3227729
FAM189A1	0.007945	0.992055	0.281366729	0.274577	14.13469943	51.50445564
EMP1	0.008079	0.991921	1.672382056	1.672443	8916.9761	5331.701629
CPVL	0.008112	0.991888	0.512671855	0.511718	127.2122949	248.6080455
TLL1	0.008477	0.991523	0.233341395	0.223948	9.086592493	40.60928233
PRR11	0.008585	0.991415	1.667724839	1.66788	3492.865962	2094.190976
FNDC4	0.008665	0.991335	0.51068164	0.509688	122.164188	239.6938128
ADAM19	0.008828	0.991172	0.557286253	0.556808	256.4438326	460.5686899
ACSL5	0.008933	0.991067	0.372227366	0.368511	30.28864164	82.20903496
SLC16A2	0.008986	0.991014	0.475273297	0.473722	79.76008966	168.3799511
TMEM132	0.009194	0.990806	0.59883839	0.598761	1506.355111	2515.794564
ZNF579	0.009232	0.990768	0.479157004	0.477662	82.78895383	173.3323026
DEPDC1B	0.009238	0.990762	1.725266296	1.726058	769.3314978	445.7116353
CORO7-P	0.009268	0.990732	0.033031896	0.000687	0	14.54010402
FAM222B	0.009587	0.990413	1.742883079	1.74387	639.0903387	366.4740113
CKAP2L	0.009728	0.990272	1.664126067	1.664307	2977.100876	1788.789363
RHOF	0.009764	0.990236	1.66533688	1.665397	9023.804139	5418.407399
SELM	0.009845	0.990155	0.406631214	0.403757	40.38485553	100.0375004
CRABP2	0.010059	0.989941	0.257627097	0.249339	11.10583527	44.57116353
RASEF	0.010066	0.989934	0.529607038	0.528818	153.462451	290.2077981
GPR153	0.01014	0.98986	0.578567082	0.578283	416.9736333	721.062379
WNK2	0.010401	0.989599	0.554776353	0.554259	232.2129193	418.9689372
LETM2	0.010621	0.989379	1.72918043	1.730069	690.5810295	399.1595312
S100A10	0.010697	0.989303	1.661513228	1.661712	2692.660242	1620.409412
COL23A1	0.010716	0.989284	0.463890985	0.462135	68.65425439	148.5705451
GPR135	0.010818	0.989182	0.077984273	0.054152	1.009621388	18.81893571
LOC10050	0.010818	0.989182	0.077984273	0.054152	1.009621388	18.81893571
CCDC3	0.010972	0.989028	0.277796743	0.270586	13.12507805	48.53304474
COX6B2	0.011256	0.988744	1.893246253	1.896223	275.7780822	145.4307543
BUB1B	0.011354	0.988646	1.656109374	1.656243	3945.509519	2382.19993
ADGRA2	0.011647	0.988353	0.59753545	0.597406	900.5822782	1507.495798
ARSG	0.011762	0.988238	0.397828443	0.394647	36.34636997	92.11373797
LYPD1	0.011762	0.988238	0.397828443	0.394647	36.34636997	92.11373797
COL5A1	0.011831	0.988169	0.602042163	0.602024	6396.961115	10625.76539
RXFP1	0.011873	0.988127	1.654404731	1.654503	5343.926008	3229.923651
SERF2-C1	0.012097	0.987903	0.211580369	0.20064	7.026964862	35.06264865
RGAG4	0.012292	0.987708	0.395383909	0.392119	35.33674859	90.13279737
CDC25C	0.01248	0.98752	1.721601757	1.722482	687.5521653	399.1595312
PHOSPHO	0.012573	0.987427	3.854540493	3.981938	43.41371969	10.89517331
CLMN	0.012862	0.987138	0.582161614	0.581876	414.9543905	713.1386166

SLC2A12	0.01291	0.98709	2.098324512	2.105585	158.5105579	75.27574286
GOLGA8E	0.012973	0.987027	0.598986427	0.598855	888.062973	1482.942039
AP4S1	0.013093	0.986907	0.501532731	0.500318	99.95251743	199.7877644
PADI2	0.013399	0.986601	0.524473743	0.523568	133.2700232	254.5508673
NIPAL1	0.013432	0.986568	1.67233794	1.672759	1299.614939	776.9249039
SDR42E1	0.013649	0.986351	0.139579345	0.122675	3.028864164	24.76175752
AUTS2	0.01371	0.98629	0.112887878	0.093083	2.019242776	21.79034662
LOC64285	0.013929	0.986071	0.597966548	0.597815	770.3411192	1288.601861
GK	0.014106	0.985894	0.113325826	0.093083	2.019242776	21.79034662
HOXA9	0.014304	0.985696	0.034580821	0.000721	0	13.86658421
ADAMTSL	0.014478	0.985522	0.531065119	0.530221	143.3662371	270.3983921
TRIM59	0.014713	0.985287	1.661523345	1.661872	1535.230283	923.7918401
FLRT1	0.014959	0.985041	0.215112773	0.204097	7.067349717	34.66646053
SBK1	0.015072	0.984928	0.263764314	0.255527	11.10583527	43.49155091
ABCA3	0.015322	0.984678	0.603952892	0.603848	1100.487313	1822.465353
TAGLN	0.015719	0.984281	0.590392664	0.590146	488.6567519	828.0331714
KCNQ1	0.015734	0.984266	0.50035459	0.499076	94.90441049	190.1702977
LACTB	0.015764	0.984236	0.563939438	0.56345	243.3187545	431.8450511
ASAP3	0.01587	0.98413	0.593285973	0.593072	549.2340352	926.0897312
TMEM74B	0.01601	0.98399	0.5712578	0.570836	282.6939887	495.2351504
CCDC181	0.016022	0.983978	0.183537025	0.170168	5.048106941	29.71410902
CASC5	0.016374	0.983626	1.654896359	1.655235	1595.201793	963.7276027
MMP7	0.016714	0.983286	0.576530773	0.576154	315.0018731	546.739606
ASPM	0.017187	0.982813	1.650053947	1.650346	1785.010614	1081.593568
SOX4	0.017204	0.982796	0.609703297	0.609653	2336.263892	3832.129594
STAT5A	0.017337	0.982663	0.489074776	0.48756	81.8197173	167.8252878
NES	0.017616	0.982384	0.478308812	0.476607	72.69273995	152.5324263
HRASLS2	0.017649	0.982351	1.742131633	1.743389	500.7722085	287.2363872
SNED1	0.017993	0.982007	0.569716617	0.569249	260.4823181	457.597279
ARL14	0.018052	0.981948	3.242833008	3.311381	52.50031218	15.84752481
CALM3	0.018136	0.981864	1.639807733	1.639877	7406.582504	4516.544572
PLOD2	0.019231	0.980769	0.61093098	0.610874	2032.367854	3326.98974
IGF2	0.019616	0.980384	0.166493418	0.15133	4.038485553	26.74269812
IGFN1	0.020223	0.979777	0.607849939	0.607739	1047.987001	1724.408794
ALPPL2	0.020262	0.979738	1.85920892	1.862092	268.9328492	144.4204746
LOC61326	0.020598	0.979402	0.569295849	0.568813	247.0038726	434.251894
KIF18A	0.020779	0.979221	1.646775422	1.647116	1520.489811	923.1183203
ARHGEF4	0.020796	0.979204	0.585595754	0.585271	363.4636997	621.0248786
MDK	0.021086	0.978914	0.602224125	0.602051	674.4270873	1120.22191
DNM1	0.021105	0.978895	0.515678196	0.514545	107.0198671	207.9987632
PTGES	0.021626	0.978374	1.730191937	1.731448	500.7722085	289.2173278
LOC10050	0.021749	0.978251	4.067927992	4.234541	37.78003234	8.914232707
CUZD1	0.021977	0.978023	3.285259225	3.36161	50.06712464	14.88676862
FRMD6	0.02225	0.97775	0.602937474	0.602757	660.2923879	1095.460153
TRIM17	0.0226	0.9774	0.32793905	0.322015	18.17318499	56.45680714
FAM155B	0.022621	0.977379	0.145276119	0.127784	3.028864164	23.77128722
PRRX1	0.022888	0.977112	0.22134766	0.210098	7.067349717	33.67599023
RARRES3	0.022941	0.977059	1.629311567	1.629497	2750.208661	1687.761393
DAW1	0.023033	0.976967	1.662217985	1.662759	991.4482032	596.2631211
MFSD2A	0.023358	0.976642	0.396722449	0.393071	32.30788442	82.20903496
CD70	0.023566	0.976434	0.610030872	0.609918	1022.746466	1676.866219
C1QTNF3	0.023703	0.976297	27.09965076	1297.354	12.96353862	0
OPHN1	0.023734	0.976266	1.636305025	1.636591	1773.147563	1083.435843
RHOU	0.023824	0.976176	2.33131583	2.346526	99.95251743	42.59022293
TRIM31	0.023938	0.976062	1.714810732	1.715912	542.1666854	315.9600259
KLHDC8B	0.024242	0.975758	0.546972119	0.546212	158.5105579	290.2077981

FOXL1	0.024257	0.975743	0.502519461	0.501154	88.84668216	177.2941838
TNXA	0.024329	0.975671	27.01834344	1293.315	12.92315377	0
NSUN7	0.025034	0.974966	0.470107542	0.468179	62.59652607	133.7134906
PCDHGA1	0.025059	0.974941	0.249573804	0.239731	9.086592493	37.93501252
IRF1	0.02523	0.97477	1.6333534	1.633648	1748.098856	1070.054589
CDRT4	0.025494	0.974506	0.036896136	0.000771	0	12.96525624
LFNG	0.025613	0.974387	0.237145379	0.226736	8.076971105	35.65693083
PLAG1	0.026717	0.973283	0.538444256	0.537558	136.2988874	253.560397
MPPED2	0.026815	0.973185	0.037114594	0.000775	0	12.88601861
DOK5	0.026985	0.973015	0.037142084	0.000776	0	12.87611391
LOC28562	0.026985	0.973015	0.037142084	0.000776	0	12.87611391
NR2F1-AS	0.026985	0.973015	0.037142084	0.000776	0	12.87611391
TMEM150	0.027013	0.972987	0.406454058	0.403063	34.3271272	85.18044587
CD24	0.027054	0.972946	0.614335677	0.614247	1301.270719	2118.487212
KIAA1524	0.027213	0.972787	1.637929534	1.638295	1392.267894	849.8235181
MFAP5	0.027374	0.972626	1.620878757	1.621011	3692.690227	2278.012359
CLSTN3	0.027697	0.972303	0.58144094	0.58103	287.7420956	495.2351504
MCAM	0.027723	0.972277	0.57297477	0.572478	239.2903652	417.9982763
RBM24	0.027811	0.972189	1.717378552	1.718582	498.7529658	290.2077981
LINC0113	0.027823	0.972177	0.25130184	0.241623	9.086592493	37.63787143
ITPKA	0.028026	0.971974	0.510972111	0.509694	94.90441049	186.2084165
RAB11FIP	0.02849	0.97151	0.615538447	0.61545	1324.623261	2152.291964
PCDHA8	0.028621	0.971379	26.51017267	1268.075	12.67074842	0
GJA3	0.028815	0.971185	0.567175897	0.56661	211.0108701	372.4168331
MT2A	0.029169	0.970831	0.615924232	0.615841	1379.142816	2239.45335
ARHGEF9	0.029412	0.970588	0.505316724	0.503904	87.83706077	174.3227729
B4GALNT	0.029459	0.970541	0.61422812	0.614122	1083.323749	1764.027606
TTC3P1	0.029556	0.970444	0.285121032	0.277181	12.13564909	43.8085014
ID3	0.029712	0.970288	1.623261599	1.623498	2072.75271	1276.716218
VSTM4	0.030892	0.969108	0.290456439	0.282556	12.62026735	44.69001997
PTGIS	0.031137	0.968863	0.617785303	0.617757	4122.17307	6672.808321
SYS1-DBN	0.031395	0.968605	0.406548522	0.402994	33.47904523	83.09055353
AFF3	0.031411	0.968589	0.590125876	0.589778	338.223165	573.4823041
ANTXR2	0.031444	0.968556	0.586471688	0.586077	305.9152806	521.9778485
SPEG	0.0315	0.9685	0.53112256	0.530075	118.1257024	222.8558177
ANKRD30	0.031538	0.968462	1.82879271	1.831554	267.5698603	146.0844647
IGFBP5	0.031625	0.968375	0.28613653	0.278166	12.11545666	43.58069323
RAET1L	0.031686	0.968314	8.099684872	9.760856	20.2933899	2.070082929
FBN1	0.032521	0.967479	0.615252575	0.61514	1018.707981	1656.066343
PSTPIP2	0.032564	0.967436	0.54547588	0.544637	143.0027734	262.5736767
LRP4	0.032625	0.967375	0.594262537	0.59395	373.5599136	628.948641
PMEPA1	0.033066	0.966934	0.619751106	0.619698	2194.916898	3541.921796
IL1A	0.033258	0.966742	1.636522203	1.636538	31144.80058	19030.89636
ICAM1	0.033456	0.966544	0.600964384	0.600709	456.3488674	759.6907207
SPNS2	0.033729	0.966271	0.086788763	0.060519	1.009621388	16.83799511
TCAIM	0.033939	0.966061	1.76623091	1.768195	343.271272	194.132179
MED12L	0.034209	0.965791	0.46013138	0.457883	53.50993357	116.8754955
ENPP2	0.034506	0.965494	2.506979482	2.531399	77.74084689	30.70457932
NR2F1	0.035276	0.964724	0.087475492	0.060519	1.009621388	16.83799511
NTN5	0.035276	0.964724	0.087475492	0.060519	1.009621388	16.83799511
WSCD1	0.035276	0.964724	0.087475492	0.060519	1.009621388	16.83799511
LHX1	0.035894	0.964106	0.580989463	0.580528	256.4438326	441.7497541
RASGRP1	0.036313	0.963687	0.564924266	0.564288	187.7895782	332.7980211
ZNF699	0.037037	0.962963	1.753362021	1.755209	356.39635	203.0464117
COL1A2	0.037181	0.962819	0.151457694	0.133337	3.028864164	22.78081692
ITGB3	0.037407	0.962593	0.613719443	0.613568	757.2160411	1234.125995

AASS	0.037792	0.962208	0.283833166	0.275526	11.55006868	41.94641724
LIMS3-LO	0.037872	0.962128	2.55786712	2.585305	73.04610743	28.24821298
MND1	0.038052	0.961948	1.70788992	1.709146	468.9287499	274.3602733
MAPK12	0.038313	0.961687	0.442514851	0.439769	44.42334108	101.0279707
GHDC	0.038507	0.961493	0.578784544	0.578287	238.2706476	412.0356451
ITGA7	0.039372	0.960628	0.513876865	0.512542	90.86592493	177.2941838
GDAP1	0.039487	0.960513	0.196358196	0.182319	5.048106941	27.73316842
MR1	0.039606	0.960394	0.508053613	0.506591	84.79810039	167.3993855
LINC0045	0.040451	0.959549	1.630327463	1.630788	1086.938194	666.5072748
SNN	0.040562	0.959438	0.614973896	0.614814	735.0043706	1195.497653
PURG	0.040607	0.959393	0.383127425	0.3787	26.25015609	69.33292105
HOXA10-H	0.040821	0.959179	25.39219697	1212.546	12.11545666	0
BTBD11	0.041551	0.958449	0.258222793	0.248152	9.086592493	36.64740113
CADPS2	0.041938	0.958062	0.339621782	0.333723	18.17318499	54.47586654
APOE	0.042223	0.957777	0.339753994	0.333723	18.17318499	54.47586654
CHSY3	0.042395	0.957605	0.379314191	0.374848	25.2405347	67.35198045
NCAPG	0.042447	0.957553	1.603049814	1.603199	3163.143809	1973.016839
KIFC2	0.042667	0.957333	0.61296007	0.612778	633.0326104	1033.060524
TPD52L1	0.043163	0.956837	1.602732119	1.602821	5261.137054	3282.418577
RPL21	0.043719	0.956281	0.624074834	0.624026	2317.797917	3714.273533
SGK1	0.045231	0.954769	0.497755615	0.49611	73.70236134	148.5705451
ARMT1	0.045952	0.954048	1.600399742	1.600567	2788.574274	1742.237259
RAB27A	0.047363	0.952637	0.591076601	0.590666	286.389203	484.8649263
C10orf90	0.047512	0.952488	11.1213137	16.15634	16.15394221	0.990470301
XRCC4	0.048784	0.951216	1.631464175	1.632019	903.6111424	553.6728981
TM4SF19-	0.049033	0.950967	0.039891429	0.000839	0	11.90545302
SLC13A3	0.049252	0.950748	0.569753288	0.56909	183.7510926	322.8933181
LOC10012	0.049576	0.950424	7.942858367	9.64007	19.18280638	1.980940602
IQGAP2	0.05022	0.94978	0.179550972	0.163432	4.038485553	24.76175752
ABCC8	0.050449	0.949551	0.0401131	0.000841	0	11.88564361
BZRAP1	0.050449	0.949551	0.0401131	0.000841	0	11.88564361
PRDM1	0.050449	0.949551	0.0401131	0.000841	0	11.88564361
VNN3	0.050449	0.949551	0.0401131	0.000841	0	11.88564361
LINC0031	0.050723	0.949277	0.362098873	0.356876	21.20204915	59.42821805
COL8A2	0.051803	0.948197	0.409004261	0.405197	31.29826303	77.25668346
ING5	0.052104	0.947896	0.59134244	0.590905	274.6473062	464.797998
MROH8	0.052342	0.947658	0.234968437	0.223225	7.067349717	31.69504962
HERC5	0.052461	0.947539	1.594297245	1.594454	2939.007861	1843.26523
LRRC6	0.052492	0.947508	0.460714395	0.458283	49.47144802	107.9612628
MPP2	0.052681	0.947319	0.388541289	0.384187	26.25015609	68.34245075
SPATA20	0.052834	0.947166	0.619267113	0.619103	716.8311856	1157.859782
AXL	0.053935	0.946065	1.60491138	1.604944	14636.46107	9119.606724
MSH5-SAI	0.054881	0.945119	1.798851674	1.801583	256.464025	142.3503916
STK32A	0.056367	0.943633	0.466602813	0.46421	51.4906908	110.9326737
TRIM6-TR	0.056506	0.943494	24.3555286	1161.055	11.60054975	0
PIK3IP1	0.05671	0.94329	0.25089235	0.240069	8.076971105	33.67599023
KIF15	0.056738	0.943262	1.625508081	1.626071	879.3802291	540.7967842
DCLK2	0.056977	0.943023	0.522466218	0.521148	91.87554632	176.3037135
EGFL7	0.056983	0.943017	0.606308249	0.606013	393.7523414	649.7485173
S100P	0.056984	0.943016	0.605891318	0.605583	385.6753703	636.8724034
PCDHGA1	0.057477	0.942523	0.602383165	0.602049	348.4506297	578.7813203
CDCA8	0.05756	0.94244	1.596833329	1.59711	1716.35636	1074.660276
PCDH1	0.059148	0.940852	0.626198696	0.626088	1056.063972	1686.770922
TPPP	0.059192	0.940808	2.101615536	2.11128	117.116081	55.46633684
FAM24B	0.059636	0.940364	1.843593133	1.847149	213.2421334	115.4393136
SEMA4G	0.059748	0.940252	0.496907791	0.495142	68.65425439	138.6658421

F11R	0.059807	0.940193	1.590137117	1.590212	6139.689393	3860.922565
NCAN	0.059882	0.940118	0.265071248	0.255043	9.086592493	35.65693083
TMEM164	0.060901	0.939099	1.589159652	1.589346	2446.312624	1539.190847
GTSE1	0.061233	0.938767	1.640410384	1.641163	679.4751942	414.0165857
GRK5	0.062615	0.937385	2.002207991	2.009401	139.3277516	69.33292105
GPM6B	0.062777	0.937223	0.317670964	0.310384	14.13469943	45.56163384
WNT11	0.062777	0.937223	0.317670964	0.310384	14.13469943	45.56163384
DENND6E	0.063012	0.936988	0.496763916	0.494932	67.64463301	136.6849015
LRRN2	0.063204	0.936796	0.289228041	0.280499	11.10583527	39.61881203
PLPPR2	0.063851	0.936149	0.598087289	0.597691	294.8094453	493.2542098
TRIM16L	0.064269	0.935731	1.738937604	1.740903	318.5052593	182.9497693
DISC1	0.065667	0.934333	0.496390958	0.494578	66.8369359	135.1496725
GPNMB	0.066215	0.933785	0.130253933	0.107773	2.019242776	18.81893571
OPRK1	0.066215	0.933785	0.130253933	0.107773	2.019242776	18.81893571
ZNF467	0.066215	0.933785	0.130253933	0.107773	2.019242776	18.81893571
TNFRSF1	0.066806	0.933194	0.45691553	0.454214	45.43296247	100.0375004
ANKRD33	0.066857	0.933143	1.584197126	1.584366	2672.467814	1686.770922
MEGF6	0.066872	0.933128	0.627252104	0.627134	962.1691829	1534.238496
PLOD3	0.067117	0.932883	0.609396816	0.609102	392.74272	644.7961658
SCG2	0.067886	0.932114	2.289522309	2.30657	86.82743938	37.63787143
SMR3B	0.068025	0.931975	0.130749322	0.107773	2.019242776	18.81893571
PDLIM1	0.068196	0.931804	0.617791374	0.617567	522.9838791	846.8521072
ARSD	0.069162	0.930838	0.619706299	0.619505	569.4365591	919.1861532
RHOBTB1	0.069292	0.930708	0.596689537	0.596265	274.6170176	460.5686899
PDE8B	0.070283	0.929717	0.363263387	0.357775	20.19242776	56.45680714
CACNG6	0.070734	0.929266	0.224310324	0.211172	6.057728329	28.72363872
OSBPL3	0.071183	0.928817	1.604913308	1.605366	1039.91003	647.7675767
CCDC69	0.07151	0.92849	0.544498064	0.543409	113.0775955	208.0978102
PSD	0.072243	0.927757	0.390842873	0.386206	25.2405347	65.37103985
CPEB4	0.072727	0.927273	0.617445363	0.617205	489.6663733	793.3667109
SLC7A8	0.073769	0.926231	0.358116232	0.352253	19.18280638	54.47586654
SPIRE2	0.074187	0.925813	0.446274417	0.44325	40.38485553	91.12326767
FAM171A	0.074962	0.925038	0.593683887	0.59321	247.3572401	416.9879966
FAM167A	0.076054	0.923946	1.578773036	1.578857	5307.579638	3361.656201
PKP2	0.076571	0.923429	1.578456062	1.578649	2359.485184	1494.619684
SKA1	0.078312	0.921688	1.587257506	1.587585	1418.659397	893.5924007
FOXE1	0.078716	0.921284	1.582875873	1.583138	1712.317874	1081.593568
CXCR4	0.079154	0.920846	0.346207065	0.339909	17.1635636	50.51398534
TUBA4A	0.079823	0.920177	0.614041009	0.61375	404.8581767	659.6532203
BEX4	0.07998	0.92002	0.548830907	0.547795	116.1064596	211.9606444
MAL2	0.080839	0.919161	1.586506014	1.586541	13126.08767	8273.398422
SHROOM	0.081382	0.918618	0.474918229	0.47252	51.4906908	108.9814472
ARHGAP	0.081839	0.918161	0.339602792	0.332982	16.15394221	48.53304474
FMNL3	0.082873	0.917127	0.602267548	0.601861	286.7324742	476.4162147
TIAM1	0.08308	0.91692	0.403303092	0.398958	27.25977748	68.34245075
CFB	0.083344	0.916656	0.635121265	0.635079	2676.5063	4214.45113
ANOS1	0.084126	0.915874	0.378946981	0.373862	22.21167054	59.42821805
NUPR1	0.084492	0.915508	0.635071697	0.635004	1654.769455	2605.927361
TBC1D3	0.085138	0.914862	0.043651949	0.000898	0	11.12298148
VANGL2	0.08579	0.91421	0.547695854	0.546622	112.0679741	205.0273523
MB21D2	0.085928	0.914072	1.700463029	1.70211	352.3578645	207.0082929
ATP6V0E	0.08638	0.91362	0.272537734	0.262328	9.086592493	34.66646053
CACNG4	0.08638	0.91362	0.272537734	0.262328	9.086592493	34.66646053
DBF4	0.08645	0.91355	1.612419794	1.613048	765.2930122	474.4352741
PI16	0.086501	0.913499	0.611924228	0.611608	363.4636997	594.2821805
DHX58	0.088231	0.911769	0.560059971	0.559148	131.2507805	234.7414613

KIAA1644	0.09009	0.90991	0.296747861	0.287689	11.10583527	38.62834173
UPP1	0.091015	0.908985	1.573901883	1.574148	1830.443577	1162.812133
GPSM3	0.091114	0.908886	0.451072067	0.448121	40.38485553	90.13279737
ASMTL-AS	0.092167	0.907833	0.503917201	0.502098	66.63501162	132.7230203
PAX6	0.092585	0.907415	0.043600749	0.000917	0	10.89517331
MYLK	0.093345	0.906655	0.621566229	0.621327	477.5509166	768.6049534
FAM225B	0.093539	0.906461	0.115087448	0.088925	1.453854799	16.4517117
IGDCC4	0.093768	0.906232	0.568021712	0.567196	144.3758585	254.5508673
ITGB2	0.093902	0.906098	0.626661536	0.62647	595.676619	950.8514887
FER1L4	0.093975	0.906025	0.430061291	0.426517	33.36798688	78.24715376
GPR37	0.097599	0.902401	0.044524034	0.000917	0	10.89517331
BEAN1	0.097924	0.902076	0.393129052	0.388416	24.23091332	62.39962895
S100A14	0.098294	0.901706	1.676925186	1.678359	385.6753703	229.7891098
NRG1	0.098295	0.901705	0.582697614	0.582021	178.7029857	307.0457932
SUSD4	0.099642	0.900358	0.412039578	0.40782	28.26939887	69.33292105
SYNPO2L	0.099682	0.900318	22.53306397	1066.151	10.65150565	0
RUNDC3A	0.099726	0.900274	0.358733221	0.352686	18.19337741	51.60350267
BMP2	0.100193	0.899807	1.649019535	1.65016	467.4547027	283.274506
SERPINB4	0.101794	0.898206	0.098107335	0.068583	1.009621388	14.85705451
EPCAM	0.10189	0.89811	1.564159251	1.564277	3648.771697	2332.557558
LOC40092	0.102734	0.897266	2.031167582	2.039895	117.2978129	57.49680096
POPDC3	0.104257	0.895743	0.425168774	0.421403	31.29826303	74.28527256
SYNC	0.105604	0.894396	0.581230488	0.580517	169.6163932	292.1887387
BSPRY	0.105718	0.894282	1.714145812	1.716128	300.8671737	175.3132432
STX16-NP	0.106019	0.893981	0.429923805	0.426201	32.46942384	76.19688024
ASB3	0.10638	0.89362	1.646732717	1.64785	464.2542029	281.7293724
PECAM1	0.107146	0.892854	0.232479744	0.218711	6.057728329	27.73316842
TUBB4A	0.107211	0.892789	0.534999869	0.533657	89.93707326	168.5384264
OSCP1	0.1075	0.8925	0.570712187	0.569882	143.3662371	251.5794564
PNMA2	0.107677	0.892323	0.628959726	0.628764	580.3202777	922.9598451
IL11	0.110118	0.889882	0.346877602	0.3402	16.18423085	47.59209795
ARF4-AS1	0.111798	0.888202	4.3103352	4.580983	27.25977748	5.942821805
GPR173	0.112386	0.887614	0.544149244	0.542932	98.94289604	182.2465353
EDAR	0.115093	0.884907	2.498026048	2.527335	62.59652607	24.76175752
SCUBE3	0.11547	0.88453	0.250648504	0.238101	7.067349717	29.71410902
OSBP2	0.115556	0.884444	0.542270374	0.541014	95.91403188	177.2941838
LOC10028	0.115728	0.884272	1.602784872	1.603461	695.6291364	433.8259917
GPR155	0.116966	0.883034	0.537071108	0.535728	89.85630355	167.7361454
LOC10063	0.117051	0.882949	0.547352029	0.546169	101.6587776	186.1390836
JAK2	0.118001	0.881999	0.565769175	0.564846	129.2315377	228.7986395
BEX2	0.11844	0.88156	0.61431803	0.613947	318.0307373	518.0159673
EFEMP1	0.120522	0.879478	0.638714816	0.638598	960.1499401	1503.533917
SPTB	0.120972	0.879028	0.578406392	0.577639	154.4720724	267.4269812
GJB3	0.121065	0.878935	1.590623521	1.591195	800.6297608	503.1589128
GUCY1A2	0.121191	0.878809	0.194830948	0.177637	4.038485553	22.78081692
GJA1	0.121616	0.878384	1.560600682	1.560852	1731.500681	1109.326737
SLC9A4	0.121692	0.878308	1.803487453	1.807153	197.1689609	109.1003036
ADCYAP1	0.122544	0.877456	0.3143959	0.305976	12.11545666	39.61881203
KIF17	0.12281	0.87719	0.521866116	0.520246	74.71198272	143.6181936
SH3PXD2	0.123749	0.876251	0.634014611	0.633834	639.0903387	1008.298766
IGF2BP3	0.123754	0.876246	1.564977915	1.565282	1415.489186	904.2993846
CCDC74A	0.12385	0.87615	0.380767078	0.375341	20.83858545	55.53566976
LHX2	0.124034	0.875966	0.292997117	0.28335	10.09621388	35.65693083
SORBS2	0.125313	0.874687	1.689964955	1.69174	320.04998	189.1798274
DUSP1	0.125884	0.874116	1.564053832	1.564093	11160.35482	7135.348047
ITM2C	0.126896	0.873104	0.641276215	0.641179	1146.929897	1788.789363

SFR1	0.127252	0.872748	1.605843728	1.606604	622.7748571	387.6304569
SLC1A3	0.12804	0.87196	0.494059496	0.49183	55.52917635	112.9136143
CSPG4	0.128746	0.871254	2.21011954	2.226117	83.79857522	37.63787143
SLC16A3	0.129193	0.870807	0.642349121	0.642321	4133.389963	6435.085544
CCDC40	0.12951	0.87049	0.585709151	0.584994	168.6067718	288.2268575
RCAN2	0.130131	0.869869	0.430960512	0.427097	31.29826303	73.29480226
ATF5	0.133871	0.866129	0.641033083	0.640918	971.2557754	1515.41956
NRP1	0.134119	0.865881	0.644242345	0.644198	2526.072713	3921.271921
SPC24	0.135076	0.864924	1.57533024	1.575794	947.9436176	601.5621372
GALNT12	0.136945	0.863055	0.590286982	0.589629	178.7029857	303.083912
IDH1	0.138325	0.861675	0.644668015	0.644599	1613.374978	2502.91845
MUC13	0.138373	0.861627	5.093188678	5.594747	22.21167054	3.961881203
CDH23	0.139637	0.860363	0.423005206	0.418921	28.79440199	68.74854358
ZNF711	0.140004	0.859996	0.619844433	0.619493	333.1750581	537.8253733
ABCB6	0.140743	0.859257	0.482232773	0.479739	48.46182663	101.0279707
CPEB1	0.141215	0.858785	0.412268468	0.407826	26.25015609	64.38056955
CTSO	0.142977	0.857023	0.574265692	0.573394	136.2988874	237.7128722
ACADSB	0.145349	0.854651	0.620570356	0.620228	334.1846795	538.8158436
ARHGDIG	0.146741	0.853259	0.354052547	0.347148	16.15394221	46.55210414
IFI6	0.147204	0.852796	1.738292208	1.741	236.2514048	135.6944312
MAL	0.147572	0.852428	1.547193284	1.54736	2467.514673	1594.657184
PTAFR	0.148126	0.851874	2.221836887	2.238509	79.83076316	35.65693083
BUB1	0.148405	0.851595	1.558070559	1.5584	1284.238406	824.0712902
ELOVL2	0.148416	0.851584	0.561243191	0.560152	112.0679741	200.0750008
EFNB2	0.149058	0.850942	0.625950343	0.625656	386.6849917	618.0534677
ST3GAL6	0.149626	0.850374	1.669992822	1.671676	331.1558153	198.0940602
LINC0111(	0.150275	0.849725	1.643003543	1.644333	395.7715842	240.6842831
NMUR2	0.151652	0.848348	0.534511488	0.533017	80.76971105	151.541956
NFATC2IF	0.151848	0.848152	0.643053922	0.642924	880.4706202	1369.483666
NCF2	0.152502	0.847498	1.577308719	1.577864	798.610518	506.1303237
ARVCF	0.152567	0.847433	0.626579254	0.626286	387.6946131	619.043938
ARPC1A	0.153208	0.846792	1.55426177	1.554565	1381.162059	888.4518598
MGC3280	0.153612	0.846388	1.897803973	1.903733	140.2969881	73.69099038
LAMA3	0.153926	0.846074	1.562239756	1.562265	17047.45714	10912.0113
CRADD	0.154792	0.845208	1.771713335	1.774982	203.9435204	114.8945549
ROBO3	0.156413	0.843587	0.383258996	0.377648	20.19242776	53.48539624
AVPR2	0.156982	0.843018	0.339578565	0.332033	14.13469943	42.59022293
PAK3	0.158253	0.841747	0.339747249	0.332033	14.13469943	42.59022293
C16orf62	0.160111	0.839889	0.639304142	0.639127	633.0326104	990.4703008
BACH2	0.162966	0.837034	0.258862339	0.246309	7.067349717	28.72363872
A4GALT	0.163702	0.836298	0.63093771	0.630663	421.0121189	667.5769827
PHOSPH(	0.163753	0.836247	1.670407329	1.672123	318.0610259	190.2099166
COL13A1	0.164178	0.835822	0.047752619	0.001009	0	9.904703008
KIF5A	0.164178	0.835822	0.047752619	0.001009	0	9.904703008
GAS2L3	0.166464	0.833536	1.541318333	1.541491	2347.218284	1522.689612
COL4A5	0.167571	0.832429	0.322545599	0.313819	12.11545666	38.62834173
NKD2	0.167752	0.832248	0.274738422	0.263294	8.076971105	30.70457932
C9orf172	0.168506	0.831494	0.104872671	0.073478	1.009621388	13.86658421
RASGRP3	0.168506	0.831494	0.104872671	0.073478	1.009621388	13.86658421
CIT	0.169144	0.830856	1.539836237	1.540014	2329.196542	1512.448149
SMC4	0.169636	0.830364	1.54662102	1.546669	8750.388571	5657.566358
CTH	0.170343	0.829657	1.544183347	1.544442	1615.394221	1045.936638
CCM2L	0.170765	0.829235	0.288807928	0.278221	9.086592493	32.68551993
FAR2P2	0.172328	0.827672	0.048759341	0.001009	0	9.904703008
TENM2	0.172328	0.827672	0.048759341	0.001009	0	9.904703008
TMEM200	0.172328	0.827672	0.048759341	0.001009	0	9.904703008

VAC14-AS	0.172355	0.827645	1.883132417	1.889048	140.337373	74.28527256
SMG9	0.172461	0.827539	1.570907283	1.571464	784.4758186	499.1970316
ZNF625-Z	0.172502	0.827498	7.851350114	10.08676	15.37653374	1.51541956
RNASEK-(	0.172752	0.827248	3.917202217	4.12831	27.75449196	6.715388639
ONECUT2	0.173307	0.826693	0.620500933	0.620104	294.8094453	475.4257444
GOLGA8A	0.175873	0.824127	0.649717299	0.649645	1540.207716	2370.84914
PTPN20	0.175976	0.824024	0.145681362	0.120444	2.019242776	16.83799511
TMEM159	0.176614	0.823386	0.583912942	0.583077	144.3758585	247.6175752
CCDC184	0.176854	0.823146	0.511708216	0.509709	60.57728329	118.8564361
MAPT	0.177292	0.822708	0.640883484	0.640698	604.7632115	943.9181966
FBXO41	0.177739	0.822261	0.535522074	0.53397	77.74084689	145.5991342
FRMD3	0.177739	0.822261	0.535522074	0.53397	77.74084689	145.5991342
ADAMTS5	0.178602	0.821398	0.550207546	0.5489	91.87554632	167.3894808
GALNS	0.181947	0.818053	0.64329875	0.643132	671.3982231	1043.955697
EPS8L3	0.182587	0.817413	1.700999049	1.703316	256.4438326	150.5514857
AAMDC	0.183356	0.816644	1.646569128	1.648054	349.3290003	211.9606444
TLE2	0.184114	0.815886	0.619618115	0.61921	279.6651245	451.6544572
SH3TC2	0.184279	0.815721	1.551329774	1.551728	1068.179429	688.376859
BBC3	0.184623	0.815377	0.601363482	0.600749	189.808821	315.9600259
SLC25A42	0.185137	0.814863	0.562066075	0.560928	105.0006244	187.1988868
IPO11-LRI	0.187828	0.812172	0.04951292	0.001025	0	9.74622776
ZDHHC1	0.188321	0.811679	0.486053158	0.48345	46.44258386	96.07561918
THBS1	0.189279	0.810721	0.651905209	0.651839	1717.365981	2634.651
SLC9A9	0.190113	0.809887	0.361511956	0.354693	16.15394221	45.56163384
NKX3-1	0.191246	0.808754	0.594125702	0.593413	164.5682863	277.3316842
OR7E37P	0.192352	0.807648	2.219967705	2.237703	74.90381079	33.46799146
ABCG1	0.192684	0.807316	1.570168084	1.570803	701.6868648	446.7021056
CXCL1	0.194248	0.805752	0.652587275	0.652535	2152.230106	3298.266102
USP2	0.194778	0.805222	1.62791306	1.629204	385.6753703	236.7224019
ZCCHC24	0.195023	0.804977	0.634666719	0.634396	415.9640119	655.6913391
VDR	0.196201	0.803799	0.641308782	0.641103	544.1859282	848.8330478
TRIQK	0.196298	0.803702	1.588354007	1.589206	546.205171	343.6931944
C12orf66	0.196586	0.803414	1.604249154	1.605259	467.4547027	291.1982684
UBE2F-SC	0.197152	0.802848	0.049350408	0.001044	0	9.567943105
CXCL16	0.197352	0.802648	0.618733138	0.618294	261.4919395	422.9308184
ACP6	0.19783	0.80217	1.613120427	1.61424	430.0987114	266.4365109
PLB1	0.199599	0.800401	2.433442405	2.462778	58.55804051	23.77128722
GPRC5A	0.199823	0.800177	1.53315787	1.533362	1951.598143	1272.754336
FAR2	0.201236	0.798764	0.593496363	0.59274	158.5105579	267.4269812
HAS3	0.203049	0.796951	1.54902777	1.549444	994.4770673	641.8247549
ACSL4	0.203475	0.796525	0.652807668	0.65273	1416.498808	2170.120429
MFI2	0.203523	0.796477	0.608611204	0.608036	205.9627632	338.7408429
GALNT6	0.203547	0.796453	0.228482166	0.212693	5.048106941	23.77128722
PVRL1	0.203783	0.796217	0.646052551	0.64588	658.2731451	1019.193939
LY6K	0.204177	0.795823	2.242184004	2.261252	71.68311856	31.69504962
MYPN	0.204583	0.795417	2.160751164	2.176098	79.76008966	36.64740113
SLIT2	0.204615	0.795385	0.633170051	0.632872	378.6080206	598.2440617
TMEM139	0.205959	0.794041	1.580012922	1.580772	585.5804051	370.4358925
KLHL14	0.206752	0.793248	0.228831971	0.212693	5.048106941	23.77128722
APOBEC3	0.207199	0.792801	0.535591594	0.533961	73.99515154	138.5866045
RHPN1	0.208788	0.791212	0.587482247	0.586655	142.3566157	242.6652237
TRIP13	0.209538	0.790462	1.539562193	1.539906	1203.488887	781.5305908
P2RY1	0.211185	0.788815	0.547302255	0.545867	83.79857522	153.5228966
ADAM23	0.211536	0.788464	0.62466409	0.624269	288.751717	462.5496305
LINC0133	0.213089	0.786911	2.200455241	2.218192	74.71198272	33.67599023
RIMS1	0.217418	0.782582	0.249644338	0.235528	6.057728329	25.75222782

FNDC1	0.217625	0.782375	0.59473762	0.593989	156.4913152	263.4651
ZNF512B	0.218044	0.781956	0.652805657	0.652702	1055.054351	1616.447531
LOC28629	0.21856	0.78144	1.7953849	1.799577	166.1836805	92.34154614
NOL4L	0.219607	0.780393	0.63289267	0.632573	353.3674859	558.6252496
TPST2	0.22015	0.77985	0.629483933	0.629128	319.0403587	507.120794
KREMEN2	0.221347	0.778653	0.250017056	0.235528	6.057728329	25.75222782
MID1	0.222093	0.777907	1.622524799	1.623849	371.5406708	228.7986395
PPFIA4	0.223121	0.776879	0.405774387	0.400561	22.21167054	55.46633684
FAM182A	0.223321	0.776679	0.050544193	0.001071	0	9.330230233
MAMDC2	0.224735	0.775265	0.182523592	0.161393	3.028864164	18.81893571
PLPPR3	0.224735	0.775265	0.182523592	0.161393	3.028864164	18.81893571
ATXN7L3	0.224971	0.775029	0.655331448	0.655256	1460.922149	2229.548647
CAPN3	0.225909	0.774091	0.48450469	0.481721	43.41371969	90.13279737
LGR6	0.225923	0.774077	0.320922986	0.311657	11.10583527	35.65693083
PID1	0.225923	0.774077	0.320922986	0.311657	11.10583527	35.65693083
LOC10192	0.228381	0.771619	1.655255368	1.657095	293.799824	177.2941838
PLK4	0.229027	0.770973	1.543314212	1.543743	951.0633476	616.0725271
LIMD2	0.231265	0.768735	0.594080174	0.593301	150.4335868	253.560397
FCGBP	0.231271	0.768729	0.283794676	0.272068	8.076971105	29.71410902
ENG	0.231835	0.768165	3.378515012	3.50823	31.29826303	8.914232707
RGS2	0.233753	0.766247	0.468910308	0.465689	37.35599136	80.22809436
FNDC5	0.236009	0.763991	0.401961892	0.396521	21.20204915	53.48539624
STC2	0.236101	0.763899	0.656358716	0.656279	1385.200545	2110.692211
PHC1	0.237144	0.762856	0.651524638	0.65137	730.965885	1122.202851
SGOL2	0.237534	0.762466	1.535911296	1.536297	1062.1217	691.3482699
CDK1	0.238148	0.761852	1.521095742	1.521196	3935.504171	2587.108426
SNURF	0.239407	0.760593	0.637092812	0.636792	374.3373221	587.8540282
ESRG	0.240175	0.759825	1.536638841	1.536668	13811.42876	8987.903888
SCML1	0.242237	0.757763	1.569381939	1.570135	577.503434	367.8012415
HIST1H1D	0.242747	0.757253	19.29414773	909.6592	9.086592493	0
SEMA3C	0.244875	0.755125	0.658136145	0.658076	1855.684111	2819.868946
MIR22HG	0.245797	0.754203	0.60034585	0.599624	161.5394221	269.4079218
MIR100HG	0.246712	0.753288	0.633250602	0.632902	324.0884656	512.0731455
TNXB	0.248082	0.751918	0.574893422	0.573792	110.2506556	192.1512384
MXD3	0.251096	0.748904	1.655243507	1.657126	280.6747459	169.3704214
CCDC113	0.252215	0.747785	0.649658253	0.649472	595.676619	917.1754985
RNF145	0.254309	0.745691	0.657497885	0.657467	3676.031474	5591.204848
BACE2	0.254454	0.745546	1.521212063	1.521279	5795.236864	3809.447824
TUBAL3	0.259157	0.740843	2.906069324	2.978059	38.36561275	12.87611391
BCL11A	0.259824	0.740176	0.475652643	0.47244	38.36561275	81.21856466
ASRGL1	0.259975	0.740025	1.516707603	1.5169	2025.300505	1335.153965
JAKMIP1	0.260857	0.739143	0.153934352	0.127967	2.019242776	15.84752481
KCNJ18	0.261757	0.738243	0.052829912	0.001097	0	9.102422064
MKI67	0.264558	0.735442	1.519891668	1.519953	6274.796927	4128.280214
CFAP46	0.265533	0.734467	0.112640169	0.079126	1.009621388	12.87611391
CNKSR2	0.265533	0.734467	0.112640169	0.079126	1.009621388	12.87611391
C17orf107	0.265559	0.734441	0.355943216	0.348226	14.13469943	40.60928233
MCMDC2	0.267767	0.732233	1.830339023	1.835681	138.8633257	75.64221687
SLC4A3	0.267831	0.732169	0.614083722	0.613501	196.8761707	320.9123775
HJURP	0.269002	0.730998	1.525024222	1.525369	1155.006868	757.1947355
HOMEZ	0.269471	0.730529	0.580001468	0.578965	114.1175055	197.1134946
PDE5A	0.269672	0.730328	0.570663883	0.569485	100.9621388	177.2941838
C3orf52	0.270168	0.729832	1.682007996	1.684385	235.2417834	139.6563124
PLXNC1	0.270979	0.729021	0.561363027	0.560033	89.85630355	160.4561887
NCKAP5	0.271528	0.728472	0.052573482	0.001121	0	8.914232707
NOP9	0.273456	0.726544	0.652928933	0.652754	631.0133676	966.6990136

DARS-AS1	0.27404	0.72596	0.23702805	0.22088	5.048106941	22.88976865
SERTAD2	0.274862	0.725138	1.512582175	1.512712	2906.699977	1921.512384
ADGRB1	0.274888	0.725112	0.052778432	0.001121	0	8.914232707
CARD11	0.274888	0.725112	0.052778432	0.001121	0	8.914232707
FLJ16779	0.274888	0.725112	0.052778432	0.001121	0	8.914232707
MPC1	0.275965	0.724035	0.413202947	0.407843	22.21167054	54.47586654
GEN1	0.277127	0.722873	1.534822201	1.535286	862.2166655	561.5966605
IKZF2	0.278185	0.721815	1.93745641	1.94583	106.0102458	54.47586654
DDIT4L	0.279606	0.720394	0.569490904	0.568275	97.93327465	172.3418323
CHRD	0.281169	0.718831	0.429491305	0.42482	25.2405347	59.42821805
UTRN	0.281499	0.718501	1.51145453	1.511565	3413.529913	2258.272286
MXD4	0.282001	0.717999	0.648532283	0.648302	481.5894022	742.8527256
EFR3B	0.282731	0.717269	0.544290043	0.542635	72.69273995	133.9710129
KIF20B	0.285024	0.714976	1.533040248	1.533504	858.1781799	559.6157199
SLC1A7	0.285573	0.714427	0.339546584	0.330778	12.11545666	36.64740113
LINC0092	0.286643	0.713357	0.053885112	0.001121	0	8.914232707
SLC45A1	0.286643	0.713357	0.053885112	0.001121	0	8.914232707
PRSS3	0.292803	0.707197	0.383579778	0.376848	17.1635636	45.56163384
TACSTD2	0.2932	0.7068	1.508027605	1.508179	2523.043849	1672.904338
SLC1A1	0.293589	0.706411	0.536246062	0.534367	65.62539023	122.8183173
ELMOD1	0.293652	0.706348	0.053567728	0.001138	0	8.775566865
NUDT15	0.295922	0.704078	1.51627141	1.516566	1293.324998	852.794929
TGM1	0.296428	0.703572	0.501541799	0.498878	46.44258386	93.10420827
BDH1	0.296711	0.703289	0.474624286	0.471315	36.40694726	77.25668346
PRICKLE1	0.297254	0.702746	0.628167304	0.627692	244.3283759	389.2548282
TESK2	0.29732	0.70268	1.921025736	1.929289	107.0198671	55.46633684
NAP1L5	0.297746	0.702254	0.426750302	0.421895	24.23091332	57.44727744
RCAN3	0.300637	0.699363	0.646796219	0.646528	414.9543905	641.8247549
PPP1R3F	0.301071	0.698929	0.319234511	0.309101	10.09621388	32.68551993
DHRS1	0.301165	0.698835	0.593235321	0.592335	130.2411591	219.8844068
LSAMP	0.302829	0.697171	0.259816809	0.244945	6.057728329	24.76175752
TAL1	0.302829	0.697171	0.259816809	0.244945	6.057728329	24.76175752
GCNT3	0.303592	0.696408	1.577965819	1.579022	430.0987114	272.3793327
USP22	0.305934	0.694066	0.662571338	0.662537	3183.336237	4804.771429
CCDC136	0.305937	0.694063	0.307144082	0.296165	9.086592493	30.70457932
KCNH1	0.306106	0.693894	0.277687683	0.264547	7.067349717	26.74269812
RRAGD	0.307908	0.692092	0.661780172	0.661664	958.1306974	1448.06758
DOCK2	0.308712	0.691288	0.548806922	0.547177	73.70236134	134.7039609
DPH6	0.309847	0.690153	1.663790376	1.66604	239.280269	143.6181936
EDEM2	0.313267	0.686733	1.531521162	1.532056	757.2160411	494.2446801
LINC-ROR	0.31419	0.68581	1.676903968	1.679387	222.8335366	132.6834015
IL18	0.314603	0.685397	1.507242579	1.507317	5017.818299	3328.970681
SNRPN	0.314709	0.685291	0.644059432	0.643748	358.6478057	557.1296395
MAGED4	0.314855	0.685145	0.158744358	0.132092	2.019242776	15.35228966
MAGED4B	0.314855	0.685145	0.158744358	0.132092	2.019242776	15.35228966
FRMD5	0.314949	0.685051	0.42381525	0.41876	23.22129193	55.46633684
DYRK1B	0.317672	0.682328	0.620368637	0.619793	198.8954135	320.9123775
ADA	0.318887	0.681113	1.515709551	1.516075	1066.160186	703.2339135
APIP	0.319411	0.680589	1.505718657	1.505931	1746.645002	1159.840722
PER3	0.319645	0.680355	1.502507229	1.502659	2476.601265	1648.14258
MYO7B	0.319683	0.680317	0.371551901	0.364201	15.14432082	41.59975263
ARPC4-TT	0.319897	0.680103	0.600167272	0.599334	139.8830433	233.4043264
LRRC16A	0.320245	0.679755	1.511250081	1.511547	1268.084464	838.9283448
RAMP2	0.320364	0.679636	0.192299217	0.170355	3.028864164	17.82846541
C2	0.321437	0.678563	0.48077916	0.477477	37.35599136	78.24715376
SH3PXD2	0.324526	0.675474	3.835132781	4.072172	24.23091332	5.942821805

TINAGL1	0.325216	0.674784	1.503009453	1.503098	4247.47718	2825.811768
LINC0064	0.326761	0.673239	6.289381519	7.611639	15.14432082	1.980940602
OXCT1	0.328197	0.671803	0.646920314	0.646625	376.5887778	582.3965369
SLC38A5	0.330194	0.669806	1.983393623	1.994134	90.86592493	45.56163384
OSTF1	0.330889	0.669111	1.530585503	1.531133	721.8792925	471.4638632
FAM83D	0.332042	0.667958	1.513804296	1.51418	1031.833059	681.4435669
GAREM2	0.336528	0.663472	0.629685976	0.629196	231.2032979	367.4644816
RABGGTE	0.336557	0.663443	1.508699038	1.509022	1182.266646	783.4620079
RPL22L1	0.337249	0.662751	1.559631834	1.560528	474.5220524	304.0743823
CENPL	0.337969	0.662031	1.565485498	1.566482	442.214168	282.2939404
ENTPD7	0.338346	0.661654	0.601999421	0.601164	139.3277516	231.7700504
C15orf59	0.338592	0.661408	0.365434433	0.357631	14.14479565	39.56928852
SLC35A1	0.34094	0.65906	1.500375865	1.500588	1759.77008	1172.716836
ZMIZ2	0.34097	0.65903	0.665327925	0.665226	1062.1217	1596.638125
SYDE1	0.341811	0.658189	0.650299596	0.650017	399.8100697	615.0820568
SLC37A1	0.343123	0.656877	1.528439196	1.528992	711.7830787	465.5210414
AREG	0.343681	0.656319	1.560854544	1.56181	456.3488674	292.1887387
RAB26	0.343852	0.656148	0.571150544	0.569842	90.86592493	159.4657184
TEC	0.346082	0.653918	1.54376097	1.544518	552.2628993	357.5597786
DNAJB5	0.349833	0.650167	0.610797216	0.610042	156.4913152	256.5318079
HLA-DMB	0.35045	0.64955	1.646547602	1.64868	243.3187545	147.5800748
CACNB4	0.351059	0.648941	0.417274296	0.411769	21.20204915	51.50445564
TMX1	0.352431	0.647569	0.664765388	0.664633	838.5713326	1261.710593
ZNF580	0.353291	0.646709	0.646930964	0.6466	343.271272	530.8920812
BSCL2	0.354315	0.645685	0.568067399	0.566687	86.28224383	152.2649993
CPM	0.35591	0.64409	1.496840051	1.49702	2008.91435	1341.938687
EML2-AS1	0.356048	0.643952	1.937527621	1.946739	96.41884257	49.52351504
INHBB	0.359847	0.640153	0.223340745	0.204269	4.038485553	19.80940602
MAD2L1	0.361976	0.638024	1.495930225	1.496117	1929.386473	1289.592332
SYTL5	0.36346	0.63654	2.308040003	2.335415	55.52917635	23.77128722
LDOC1L	0.363542	0.636458	0.668463243	0.668373	1226.689987	1835.341467
SH3BP5-A	0.367019	0.632981	0.469863884	0.466059	32.30788442	69.33292105
SCYL3	0.368273	0.631727	1.600975407	1.602532	307.9345234	192.1512384
USP35	0.37054	0.62946	0.594356492	0.593365	118.1257024	199.0845305
KITLG	0.370809	0.629191	0.618800504	0.618121	172.6452574	279.3126248
IL1B	0.370837	0.629163	1.493363401	1.493462	3733.579893	2499.947039
CFAP70	0.371812	0.628188	0.551243579	0.549521	69.66387578	126.7801985
HSF2BP	0.373193	0.626807	0.605397511	0.604558	138.3181302	228.7986395
NLGN4Y	0.373773	0.626227	0.057512686	0.0012	0	8.319950527
HLA-DPA1	0.374077	0.625923	0.163864657	0.136493	2.019242776	14.85705451
SPOCK1	0.374301	0.625699	0.576661654	0.575357	92.88516771	161.446659
ADHFE1	0.374379	0.625621	0.386442971	0.379433	16.15394221	42.59022293
FAM81A	0.374379	0.625621	0.386442971	0.379433	16.15394221	42.59022293
DNER	0.374641	0.625359	0.339739113	0.329984	11.10583527	33.67599023
PPP1R14	0.375961	0.624039	1.840982794	1.847412	117.116081	63.39009925
CEP72	0.376525	0.623475	1.565019664	1.566083	404.8581767	258.5127485
MIR210HC	0.377452	0.622548	0.635694944	0.635226	240.3403715	378.3596549
LOC64491	0.377664	0.622336	6.737338254	8.466803	13.66017738	1.604561887
ZNF672	0.377834	0.622166	0.661646848	0.66145	565.3879774	854.7758696
ECM2	0.378118	0.621882	1.701953426	1.705177	180.7222285	105.9803222
MUC16	0.37889	0.62111	1.898193527	1.906365	101.9717602	53.48539624
NPY4R	0.379133	0.620867	2.054051082	2.067967	76.22641481	36.85539989
GALM	0.379191	0.620809	1.5448444	1.54567	496.1986236	321.0213292
C5orf34	0.380804	0.619196	1.705232061	1.708532	177.6933643	103.9993816
TFCP2L1	0.381856	0.618144	0.595228046	0.594206	117.116081	197.1035899
MMEL1	0.381979	0.618021	0.248790362	0.23202	5.048106941	21.79034662

MGAT5B	0.382462	0.617538	0.591220421	0.590163	111.0583527	188.1893571
TMEM178	0.382862	0.617138	0.601124822	0.600188	127.2122949	211.9606444
ICAM2	0.383893	0.616107	1.671293534	1.673975	203.9435204	121.827847
DDIAS	0.386223	0.613777	1.499237562	1.499567	1131.785576	754.7383692
ACY1	0.388536	0.611464	1.498187714	1.498493	1190.343617	794.3571812
PABPN1	0.389556	0.610444	0.666758562	0.666609	737.7404445	1106.711895
FLNC	0.391353	0.608647	0.121650321	0.085714	1.009621388	11.88564361
LINC0009	0.391353	0.608647	0.121650321	0.085714	1.009621388	11.88564361
LOC39981	0.391743	0.608257	1.719691661	1.723337	165.5779077	96.07561918
MARCKS	0.391803	0.608197	0.671321399	0.671262	1797.126071	2677.241223
C10orf55	0.395102	0.604898	2.001558747	2.013559	81.77933244	40.60928233
NDRG2	0.395316	0.604684	0.428527464	0.42323	22.21167054	52.49492594
RAPGEFL	0.3964	0.6036	1.525795502	1.526441	604.7632115	396.1881203
ATP2B4	0.396878	0.603122	1.488297552	1.488412	3091.460691	2077.016221
GCOM1	0.397137	0.602863	1.500824177	1.5012	994.6184143	662.5453936
UCN2	0.397166	0.602834	0.288165881	0.274718	7.067349717	25.75222782
LOC15222	0.400002	0.599998	0.122430492	0.085714	1.009621388	11.88564361
EGFL8	0.401188	0.598812	2.977388777	3.069574	32.03528665	10.42965227
CENPM	0.406057	0.593943	1.547431182	1.548383	443.2237894	286.2459169
PTP4A3	0.407473	0.592527	0.581982709	0.580738	94.90441049	163.4275996
PRKG1	0.407502	0.592498	3.296314387	3.437181	27.25977748	7.923762406
CLDN1	0.407886	0.592114	0.666403623	0.666388	6931.05083	10400.92863
TNRC6C	0.408071	0.591929	0.606007081	0.605103	131.2507805	216.9129959
SPATA18	0.409264	0.590736	0.517805675	0.515201	47.45220524	92.11373797
LRP10	0.410832	0.589168	0.672943019	0.672883	1798.135692	2672.288871
C9orf3	0.412992	0.587008	0.640906801	0.640458	249.3865791	389.393494
ZNF853	0.416508	0.583492	0.658022637	0.657747	405.867798	617.0629974
SMC2	0.418972	0.581028	1.483584469	1.483697	3174.249644	2139.41585
INO80C	0.419141	0.580859	1.571143259	1.572402	347.3097575	220.8748771
LINC0042	0.420578	0.579422	1.670459815	1.673284	193.302111	115.5185512
P2RX5-TA	0.422116	0.577884	0.369416835	0.36118	13.44815689	37.25158801
LINC0136	0.422176	0.577824	3.976105933	4.274596	21.20204915	4.952351504
C20orf202	0.423124	0.576876	0.058986583	0.00126	0	7.923762406
CXXC4	0.423124	0.576876	0.058986583	0.00126	0	7.923762406
DEGS2	0.423124	0.576876	0.058986583	0.00126	0	7.923762406
EGR3	0.423124	0.576876	0.058986583	0.00126	0	7.923762406
LRRC10B	0.423124	0.576876	0.058986583	0.00126	0	7.923762406
NHSL2	0.423124	0.576876	0.058986583	0.00126	0	7.923762406
PODN	0.423124	0.576876	0.058986583	0.00126	0	7.923762406
MEX3A	0.42319	0.57681	0.626307202	0.625674	179.7126071	287.2363872
POC5	0.426666	0.573334	1.562082071	1.563243	368.5118067	235.7319316
TMEM265	0.427034	0.572966	1.600874072	1.602619	274.6170176	171.351362
INO80B-W	0.427147	0.572853	1.536669479	1.537542	470.8066457	306.2038935
ZSCAN12	0.428891	0.571109	1.748083249	1.752667	142.3566157	81.21856466
SARM1	0.429477	0.570523	0.466069945	0.461971	29.27902026	63.39009925
RASD2	0.430245	0.569755	0.466161929	0.461971	29.27902026	63.39009925
NUAK2	0.430377	0.569623	1.517907832	1.518541	604.5209024	398.0898233
COLQ	0.430601	0.569399	2.812378598	2.88653	34.3271272	11.88564361
MCM5	0.434893	0.565107	0.67301856	0.672906	953.0825904	1416.37253
KIAA2022	0.435684	0.564316	0.45384073	0.449293	26.25015609	58.43774775
UBXN10	0.435684	0.564316	0.45384073	0.449293	26.25015609	58.43774775
EPHB1	0.436048	0.563952	0.203381676	0.180369	3.028864164	16.83799511
ZNF414	0.437013	0.562987	0.604646463	0.603703	123.1738094	204.036882
EVPL	0.437388	0.562612	0.060215163	0.00126	0	7.923762406
PNMA6A	0.437597	0.562403	0.506508696	0.503587	41.39447691	82.20903496
LOC38869	0.438129	0.561871	0.635310749	0.634769	207.9517173	327.6079567

ARHGEF1	0.439903	0.560097	0.67486909	0.674773	1138.852926	1687.761393
TNFSF13	0.440309	0.559691	0.190307675	0.164895	2.604823181	15.84752481
ATG12	0.44062	0.55938	1.488435812	1.488485	7140.042457	4796.847667
AKR1C3	0.442026	0.557974	1.48590313	1.485961	6027.823343	4056.510736
STX1B	0.4422	0.5578	0.203887232	0.180369	3.028864164	16.83799511
NPSR1-AS	0.44621	0.55379	2.595741501	2.649162	39.37523414	14.85705451
SORL1	0.447638	0.552362	1.48055218	1.480634	4342.38159	2932.782561
TGFBR3	0.44931	0.55069	1.621778917	1.623934	233.2326369	143.6181936
INPP5J	0.449863	0.550137	0.395693455	0.388465	16.15394221	41.59975263
DTX3	0.450969	0.549031	0.570320972	0.568791	77.74084689	136.6849015
PICK1	0.453809	0.546191	0.619801463	0.619045	151.4432082	244.6461643
ADAMTS7	0.454259	0.545741	0.544494256	0.542441	58.55804051	107.9612628
LYSMD2	0.454259	0.545741	0.544494256	0.542441	58.55804051	107.9612628
TACC3	0.45462	0.54538	1.476628974	1.476754	2796.651245	1893.779215
VEGFA	0.455541	0.544459	0.666229942	0.66622	11179.53763	16780.54784
NECAB2	0.458988	0.541012	0.45200489	0.447175	25.2405347	56.45680714
EPHA6	0.460247	0.539753	0.436667609	0.431368	22.21167054	51.50445564
MFSD4	0.460614	0.539386	0.280825406	0.265908	6.229363965	23.45433672
FAAH2	0.462367	0.537633	1.706045715	1.709776	157.5009366	92.11373797
LOC10192	0.46434	0.53566	2.218103923	2.242036	55.52917635	24.76175752
SCIN	0.46434	0.53566	2.218103923	2.242036	55.52917635	24.76175752
HMGB2	0.465045	0.534955	1.47779046	1.477873	4158.630498	2813.926124
GNRHR2	0.466974	0.533026	1.577669217	1.579157	298.4844672	189.0114475
CSGALNA	0.468769	0.531231	0.234712593	0.215014	4.038485553	18.81893571
MAP3K15	0.468936	0.531064	0.585685055	0.584359	90.86592493	155.5038372
CCDC18	0.471727	0.528273	1.560444762	1.561708	337.2135436	215.9225256
IL1RAP	0.473057	0.526943	0.670951841	0.67077	590.6285121	880.5280974
CCSER1	0.47585	0.52415	0.235229098	0.215014	4.038485553	18.81893571
OLFM2	0.476439	0.523561	0.523706309	0.521047	46.44258386	89.14232707
MKLN1-AS	0.477441	0.522559	2.103875064	2.121783	63.64653231	29.99144071
SPSB4	0.479557	0.520443	0.414261515	0.407867	18.17318499	44.57116353
WASF3	0.479933	0.520067	0.676669583	0.676552	908.6592493	1343.077728
SELT	0.481458	0.518542	1.472698209	1.472811	3003.62363	2039.378349
DZIP3	0.481753	0.518247	1.481346962	1.481665	1090.391099	735.9194335
PLGRKT	0.483039	0.516961	1.516397496	1.517135	516.9261507	340.7217835
NTF4	0.483891	0.516109	0.328219447	0.316583	9.086592493	28.72363872
RUNDC3B	0.483891	0.516109	0.328219447	0.316583	9.086592493	28.72363872
CCDC102	0.484352	0.515648	0.638456096	0.637897	200.9146562	314.9695556
LIPG	0.486468	0.513532	0.260290503	0.243063	5.048106941	20.79987632
NOVA2	0.487339	0.512661	0.512648085	0.509728	41.39447691	81.21856466
AMOT	0.487886	0.512114	0.260361214	0.243063	5.048106941	20.79987632
GIMAP2	0.487886	0.512114	0.260361214	0.243063	5.048106941	20.79987632
PRRT2	0.489217	0.510783	0.314752438	0.302286	8.076971105	26.74269812
RNF152	0.489217	0.510783	0.314752438	0.302286	8.076971105	26.74269812
CD177	0.4895	0.5105	1.552693331	1.553895	346.3001361	222.8558177
HSF4	0.48987	0.51013	0.576594991	0.575108	79.35624111	137.9923223
CCDC88A	0.491251	0.508749	1.474911244	1.47498	4948.154423	3354.722909
CFAP47	0.491417	0.508583	1.542345944	1.543425	377.5983992	244.6461643
LINC0060	0.492831	0.507169	2.224871681	2.250506	53.50993357	23.77128722
SLC8A2	0.492849	0.507151	0.260743331	0.243063	5.048106941	20.79987632
UBE2T	0.493963	0.506037	1.516020651	1.51678	501.7818299	330.8170805
DNAH17	0.494754	0.505246	0.281577455	0.266236	6.057728329	22.78081692
CELF6	0.495553	0.504447	0.49928604	0.495962	36.34636997	73.29480226
MOB3B	0.497087	0.502913	1.640077778	1.642677	196.8761707	119.8469064
CKS1B	0.497909	0.502091	1.468563434	1.468693	2643.188794	1799.684537
KIF2C	0.498675	0.501325	1.469309217	1.469429	2801.699352	1906.655329

TRIM3	0.499289	0.500711	0.63221297	0.631558	172.6452574	273.369803
NPIPB11	0.499913	0.500087	1.865840673	1.874154	94.99527641	50.68236529
AGBL3	0.501634	0.498366	0.17516452	0.146235	2.019242776	13.86658421
GDF6	0.501634	0.498366	0.17516452	0.146235	2.019242776	13.86658421
MAGEA1	0.501634	0.498366	0.17516452	0.146235	2.019242776	13.86658421
DLGAP1-A	0.502336	0.497664	1.550717017	1.551936	341.2520292	219.8844068
FAM222A	0.5041	0.4959	0.575425179	0.57388	76.7312255	133.7134906
NFAM1	0.505036	0.494964	0.175433541	0.145923	2.019242776	13.89629832
KDELC2	0.505098	0.494902	0.664928554	0.664647	385.5643119	580.1085505
LINGO1	0.505366	0.494634	0.376774724	0.36827	13.12507805	35.65693083
RAMP2-AS	0.506968	0.493032	0.381125791	0.372883	13.55921524	36.37997415
JUND	0.50719	0.49281	0.681234711	0.681151	1295.344241	1901.702977
REEP4	0.507774	0.492226	1.470349545	1.470578	1474.047227	1002.355944
TENM4	0.508923	0.491077	0.175796192	0.146235	2.019242776	13.86658421
RNF5	0.50898	0.49102	1.703849257	1.707787	148.5556911	86.98310181
GRIN3B	0.509475	0.490525	0.627709948	0.626987	156.4913152	249.5985158
ARHGAP2	0.50969	0.49031	1.479065378	1.479423	964.1884257	651.7294579
ZNF444	0.509977	0.490023	0.633605284	0.632958	173.6548788	274.3602733
OSER1	0.510917	0.489083	1.46924572	1.469465	1532.605267	1042.965227
RASA4CP	0.511508	0.488492	0.063575974	0.001335	0	7.478050771
SLC36A4	0.512511	0.487489	0.647613531	0.647116	228.1744337	352.6074271
NAT8L	0.513685	0.486315	0.672259264	0.67205	511.8780438	761.6716613
MGME1	0.514528	0.485472	1.470661991	1.470934	1266.065221	860.7186914
DNAH5	0.51505	0.48495	0.61358121	0.612625	123.1738094	201.0654711
DBF4B	0.516723	0.483277	1.48774043	1.48822	737.0236134	495.2351504
LINC0052	0.518186	0.481814	2.396181816	2.434274	43.41371969	17.82846541
SNX5	0.51834	0.48166	1.475109188	1.475157	7191.533148	4875.09482
TMEM121	0.520551	0.479449	0.568992482	0.567246	69.66387578	122.8183173
LRRC73	0.5212	0.4788	0.582924754	0.581482	81.77933244	140.6467827
ANKRD18	0.523299	0.476701	1.563400115	1.564857	294.55704	188.228976
TICAM2	0.524433	0.475567	2.648477168	2.711951	35.04395838	12.91573272
CTSV	0.525416	0.474584	1.566204751	1.567751	285.7228528	182.2465353
ARRB1	0.526695	0.473305	1.463965226	1.46412	2130.301129	1455.000872
MIPEPP3	0.527914	0.472086	0.427273444	0.421157	19.18280638	45.56163384
PRRT1	0.527914	0.472086	0.427273444	0.421157	19.18280638	45.56163384
PMP22	0.529768	0.470232	0.644013306	0.643467	203.9435204	316.9504962
ENPEP	0.532498	0.467502	0.132227255	0.093499	1.009621388	10.89517331
HELB	0.533387	0.466613	1.734752878	1.739566	129.2315377	74.28527256
MMRN2	0.534292	0.465708	0.470971992	0.466567	27.25977748	58.43774775
GRIN2B	0.534598	0.465402	2.311585085	2.343793	46.44258386	19.80940602
KIF11	0.535278	0.464722	1.462444823	1.462606	2045.523221	1398.544065
AP1M2	0.535611	0.464389	2.130925423	2.1515	57.54841913	26.74269812
MAP1LC3	0.536458	0.463542	0.554670283	0.552579	58.55804051	105.9803222
RIMS3	0.537093	0.462907	0.554624243	0.552579	58.55804051	105.9803222
PALD1	0.537403	0.462597	0.647828732	0.647304	216.0589771	333.7884914
UBE2QL1	0.540528	0.459472	0.36041941	0.350601	11.10583527	31.69504962
ZNF655	0.540652	0.459348	0.677580585	0.677395	586.3981985	865.6710429
ZNF416	0.540736	0.459264	1.505842952	1.506588	495.7241016	329.0342339
CYB5R4	0.541195	0.458805	1.467432227	1.467733	1136.833683	774.5477752
LRFN1	0.541731	0.458269	0.631749927	0.631032	157.5009366	249.5985158
TM7SF2	0.542418	0.457582	0.662823459	0.662468	312.9826303	472.4543335
SYTL2	0.542554	0.457446	1.576923505	1.578691	256.4438326	162.4371293
EMID1	0.543438	0.456562	0.399769981	0.392209	15.14432082	38.62834173
NPIPB4	0.544373	0.455627	1.492212806	1.49281	599.2304863	401.4078988
NUP50-AS	0.547172	0.452828	1.640897608	1.643734	180.7222285	109.9422034
CRISPLD1	0.550103	0.449897	0.657492536	0.65708	265.5304251	404.1118827

FAM231D	0.550601	0.449399	1.77907676	1.785341	110.4929647	61.88458439
POU4F1	0.553845	0.446155	0.512868751	0.509733	38.36561275	75.27574286
KCTD13	0.554016	0.445984	1.550885027	1.552259	302.8864164	195.1226493
MUC5B	0.555783	0.444217	0.215706738	0.191635	3.028864164	15.84752481
MLXIPL	0.557695	0.442305	0.46962609	0.465055	26.25015609	56.45680714
CREB5	0.557907	0.442093	0.630385528	0.629605	148.4143441	235.7319316
C4orf48	0.558705	0.441295	0.616716308	0.61576	120.1449452	195.1226493
EMP2	0.560488	0.439512	0.667699176	0.667379	344.5131063	516.2232161
IMP3	0.561009	0.438991	0.671295859	0.671025	396.7812055	591.3107696
LYN	0.561134	0.438866	1.459244349	1.459455	1546.739967	1059.803222
ST6GALN	0.561961	0.438039	0.216234615	0.191635	3.028864164	15.84752481
TNFAIP6	0.561961	0.438039	0.216234615	0.191635	3.028864164	15.84752481
TNFAIP8L	0.561961	0.438039	0.216234615	0.191635	3.028864164	15.84752481
FOSB	0.562692	0.437308	0.645934892	0.64537	196.8761707	305.0648526
PCDHGA7	0.563228	0.436772	0.525339912	0.522502	42.52525287	81.39684932
LPIN3	0.563511	0.436489	0.658299497	0.657881	261.1688607	396.9904013
LOC72874	0.563752	0.436248	0.63426184	0.633546	157.5009366	248.6080455
DUXAP10	0.563805	0.436195	0.581092533	0.579523	75.28746692	129.9199894
RDH10	0.563869	0.436131	0.673658827	0.673411	432.2088201	641.8247549
KLF4	0.564489	0.435511	1.462561284	1.462857	1138.852926	778.5096564
CLIP4	0.56517	0.43483	1.463474466	1.463533	5664.985609	3870.757935
SGSM2	0.566989	0.433011	0.686129743	0.686033	1105.53542	1611.495179
UGT1A5	0.567774	0.432226	14.94015748	708.7446	7.077445931	0
IL1R2	0.567877	0.432123	1.45741005	1.457609	1628.519299	1117.250499
PDE2A	0.569316	0.430684	0.480035629	0.475778	28.26939887	59.42821805
ACRV1	0.569447	0.430553	14.92027138	707.735	7.067349717	0
FTH1P3	0.570445	0.429555	0.653365087	0.65288	227.1547161	347.9324073
SLC6A12	0.571561	0.428439	0.606790729	0.605629	101.9717602	168.3799511
FOXM1	0.574478	0.425522	1.455752483	1.455942	1700.202418	1167.764485
IGFBP4	0.576561	0.423439	1.761939556	1.767789	112.0679741	63.39009925
TLR7	0.578127	0.421873	6.798810917	9.115215	11.09573906	1.208373767
NHLRC2	0.578544	0.421456	1.455069216	1.455262	1670.580126	1147.955079
CCDC180	0.57998	0.42002	0.386980868	0.378399	13.14527047	34.75560285
ZNF541	0.580626	0.419374	0.24740016	0.226953	4.038485553	17.82846541
CAMK2N2	0.582018	0.417982	0.326629228	0.313908	8.076971105	25.75222782
SLC4A4	0.582018	0.417982	0.326629228	0.313908	8.076971105	25.75222782
TRIL	0.583137	0.416863	0.066594166	0.00144	0	6.933292105
SLC4A8	0.583538	0.416462	1.537318054	1.538586	316.0518793	205.4136357
PWAR5	0.584854	0.415146	0.525384451	0.52247	41.39447691	79.23762406
FAR2P1	0.585552	0.414448	0.619903092	0.618924	119.6098459	193.2605651
FAM19A2	0.585732	0.414268	0.326911502	0.313908	8.076971105	25.75222782
HPCAL4	0.585732	0.414268	0.326911502	0.313908	8.076971105	25.75222782
PCOLCE	0.58679	0.41321	0.387579378	0.378789	13.12507805	34.66646053
LOC72839	0.586814	0.413186	0.498037149	0.494301	32.30788442	65.37103985
LRRC4	0.58724	0.41276	0.066849917	0.00144	0	6.933292105
NDST4	0.58724	0.41276	0.066849917	0.00144	0	6.933292105
NRN1	0.58724	0.41276	0.066849917	0.00144	0	6.933292105
PKNOX2	0.58724	0.41276	0.066849917	0.00144	0	6.933292105
SAMSN1	0.58724	0.41276	0.066849917	0.00144	0	6.933292105
TRABD2B	0.58724	0.41276	0.066849917	0.00144	0	6.933292105
C19orf57	0.589358	0.410642	0.587316105	0.585769	77.74084689	132.7230203
DLGAP1-A	0.58957	0.41043	1.570566662	1.572405	242.8543287	154.444034
CHCHD10	0.590081	0.409919	0.606618994	0.605448	98.94289604	163.4275996
TMED9	0.590501	0.409499	1.452246174	1.452426	1780.972129	1226.202232
ADAMTS1	0.590637	0.409363	0.311688212	0.297602	7.067349717	23.77128722
LRIG1	0.59076	0.40924	0.676067126	0.675816	425.0506044	628.948641

ATF3	0.591577	0.408423	1.46448701	1.464865	876.3513649	598.2440617
DRAXIN	0.593241	0.406759	0.273060849	0.25521	5.048106941	19.80940602
RBAK-RB/	0.593487	0.406513	14.76126446	684.5137	6.835136798	0
CBLN3	0.594997	0.405003	0.29409119	0.278332	6.057728329	21.79034662
TMEM246	0.595962	0.404038	0.627810844	0.626933	132.2604018	210.9701741
ARL3	0.596047	0.403953	1.457025006	1.457315	1118.660498	767.6144831
ZNF423	0.596875	0.403125	0.567240666	0.565308	61.58690468	108.9517331
RPS6KL1	0.597258	0.402742	0.436801329	0.430514	19.18280638	44.57116353
STRADB	0.597622	0.402378	0.688790475	0.688688	1040.919651	1511.457679
MMP10	0.598167	0.401833	1.757581686	1.76349	110.0487313	62.39962895
NXPH4	0.598211	0.401789	0.656629669	0.656134	226.1551909	344.6836647
SIX4	0.598715	0.401285	0.679573045	0.679344	472.8359847	696.0232898
LOC81691	0.598775	0.401225	1.557638964	1.559307	259.4726968	166.3990105
SRRM2-A	0.601262	0.398738	0.0682304	0.00144	0	6.933292105
RBP4	0.602011	0.397989	0.48880452	0.484687	29.27902026	60.41868835
CDKN3	0.602069	0.397931	1.471867662	1.472363	682.5040584	463.5401008
SOGA3	0.603222	0.396778	0.226825767	0.203096	3.210596014	15.84752481
SH3D21	0.6033	0.3967	0.519745537	0.516528	38.36561275	74.28527256
TNS4	0.603355	0.396645	0.64028746	0.639597	161.5394221	252.5699267
TTYH3	0.604987	0.395013	0.690091649	0.690005	1222.651501	1771.951368
TMEM163	0.605612	0.394388	0.466743184	0.461688	24.23091332	52.49492594
MYO5B	0.605698	0.394302	1.452489788	1.452758	1221.64188	840.9092854
AGPAT4	0.606459	0.393541	1.448486081	1.448635	2129.291508	1469.857926
RMDN2	0.60664	0.39336	0.525784871	0.522798	40.38485553	77.25668346
CPNE7	0.606756	0.393244	0.525851999	0.522798	40.38485553	77.25668346
NLRC5	0.606979	0.393021	1.454085838	1.454365	1150.968383	791.3857703
CENPI	0.607703	0.392297	1.562449814	1.564227	246.3476187	157.4847778
NRBP2	0.610053	0.389947	0.688166887	0.688032	792.5527897	1151.91696
LINC0067	0.610935	0.389065	1.463656456	1.464093	774.3796047	528.9111406
CCDC15	0.614664	0.385336	1.746136268	1.751864	111.0583527	63.39009925
EBF4	0.615917	0.384083	0.690136789	0.690035	1019.717602	1477.781689
HRK	0.616405	0.383595	0.41015322	0.402528	15.14432082	37.63787143
C16orf95	0.616405	0.383595	0.505841279	0.502136	33.31750581	66.36151015
TMEM241	0.61796	0.38204	1.477662144	1.47829	559.330249	378.3596549
C7orf60	0.618073	0.381927	0.65989302	0.65941	231.2032979	350.6264865
EGLN1	0.619809	0.380191	0.691411636	0.691346	1579.047851	2284.024514
SCHIP1	0.619858	0.380142	1.48544833	1.486154	497.4202655	334.699724
THRB	0.621218	0.378782	1.483570559	1.484281	502.7914513	338.7408429
SMIM10L2	0.623821	0.376179	0.589695904	0.58811	75.72160411	128.7611391
GALNT3	0.624836	0.375164	1.53022801	1.531537	301.8767951	197.1035899
PBX1	0.625488	0.374512	0.188014642	0.157475	2.019242776	12.87611391
LRRC27	0.626243	0.373757	0.644017472	0.643338	163.9625134	254.8678178
EBF3	0.627455	0.372545	0.188138254	0.157475	2.019242776	12.87611391
SCN5A	0.627455	0.372545	0.188138254	0.157475	2.019242776	12.87611391
PARPBP	0.627535	0.372465	1.460677651	1.461102	771.3507406	527.9206703
PTGS1	0.627936	0.372064	0.628587054	0.627688	126.2026735	201.0654711
TM4SF1	0.628537	0.371463	1.445256038	1.445361	2983.431202	2064.140107
THSD4	0.630374	0.369626	0.689184682	0.689035	709.7638359	1030.089113
FAM35BP	0.631337	0.368663	3.294777312	3.472446	21.72705227	6.249867598
ZC3H6	0.632543	0.367457	1.458935912	1.459371	766.3026336	525.0879253
ZNF219	0.633984	0.366016	0.631896139	0.631035	131.2507805	207.9987632
TOX	0.634266	0.365734	0.188806003	0.157475	2.019242776	12.87611391
EPHB3	0.636288	0.363712	0.44919394	0.443311	20.19242776	45.56163384
MMP17	0.636288	0.363712	0.44919394	0.443311	20.19242776	45.56163384
SAMD14	0.637818	0.362182	0.449325552	0.443311	20.19242776	45.56163384
MIR3945H	0.637904	0.362096	5.284495363	6.32154	12.70103706	2.000750008

GGT1	0.638261	0.361739	0.505640599	0.501904	32.30788442	64.38056955
TAOK3	0.639457	0.360543	1.441961924	1.44216	1601.259522	1110.317207
JAK3	0.640082	0.359918	1.512947166	1.514066	337.7385468	223.0638164
P2RY2	0.640195	0.359805	0.685001027	0.684793	505.2246388	737.7815176
FAM124A	0.640483	0.359517	0.429627493	0.422793	17.1635636	40.60928233
MUC5AC	0.640483	0.359517	0.429627493	0.422793	17.1635636	40.60928233
CDH24	0.641853	0.358147	0.63436895	0.633529	134.2796446	211.9606444
ALPK1	0.642112	0.357888	1.444694894	1.44496	1206.497559	834.9664636
GPR137B	0.642507	0.357493	0.666828481	0.666396	255.4342112	383.3120064
PBRM1	0.644539	0.355461	0.689676773	0.689523	675.4367087	979.5751275
LINC0096	0.646316	0.353684	0.549072382	0.546555	47.63393709	87.16138647
CHAC1	0.646499	0.353501	0.692224755	0.692103	854.1396944	1234.125995
DUXAP8	0.648733	0.351267	0.649612655	0.648971	172.5140066	265.832324
ZGPAT	0.649506	0.350494	0.680739804	0.680471	393.2273383	577.8799923
CLCA2	0.650342	0.349658	0.463154916	0.457772	22.21167054	48.53304474
RPL26L1	0.650408	0.349592	1.448433332	1.448764	957.121076	660.6436906
RAET1E	0.650638	0.349362	2.419420108	2.46598	36.57858289	14.8273404
HERC1	0.651192	0.348808	0.686950452	0.686743	514.906908	749.7860177
MFSD1	0.65147	0.34853	0.656359559	0.655784	194.8569279	297.1410902
KCNAB2	0.651882	0.348118	0.532569063	0.529587	40.38485553	76.26621316
IQCH-AS1	0.652272	0.347728	0.573588362	0.57163	60.57728329	105.9803222
C19orf18	0.652821	0.347179	0.610222822	0.608979	92.88516771	152.5324263
F2RL2	0.654363	0.345637	0.351817192	0.340025	9.086592493	26.74269812
BLM	0.655329	0.344671	1.463119655	1.46365	621.9267751	424.911759
CLTCL1	0.656903	0.343097	0.674696013	0.674337	303.8960378	450.6639869
DCK	0.658051	0.341949	1.438968709	1.439169	1536.643753	1067.726984
SLC38A3	0.66012	0.33988	0.398809466	0.389927	13.12507805	33.67599023
TGFB2-OT	0.661835	0.338165	0.071419892	0.001546	0	6.457866361
CHEK2	0.661928	0.338072	1.509102559	1.510276	326.1077084	215.9225256
HAPLN3	0.664138	0.335862	0.585498543	0.583758	67.64463301	115.8850252
FAM46A	0.664521	0.335479	1.450751886	1.451155	790.5335469	544.7586654
CALHM3	0.665292	0.334708	1.505330074	1.506429	337.2135436	223.846288
SNX24	0.665463	0.334537	1.439777177	1.440027	1233.757336	856.7568102
PBXIP1	0.666532	0.333468	0.691662661	0.691501	643.1288243	930.0516124
PGM2L1	0.667027	0.332973	0.670860708	0.670455	264.5006113	394.5142255
GPR68	0.667491	0.332509	0.144818561	0.102839	1.009621388	9.904703008
TSLP	0.667491	0.332509	0.144818561	0.102839	1.009621388	9.904703008
CDH5	0.667713	0.332287	0.229621979	0.204403	3.028864164	14.85705451
IL17D	0.667713	0.332287	0.229621979	0.204403	3.028864164	14.85705451
KCNK13	0.667713	0.332287	0.229621979	0.204403	3.028864164	14.85705451
LRRC24	0.667848	0.332152	0.339437478	0.326459	8.076971105	24.76175752
COMMD3-	0.668394	0.331606	1.435590175	1.435725	2254.383598	1570.202473
SMO	0.66867	0.33133	0.695501561	0.695387	916.7362205	1318.31597
SCAMP5	0.669701	0.330299	0.486530088	0.481963	26.25015609	54.47586654
KCNA7	0.670139	0.329861	0.285821612	0.267714	5.128876652	19.18540973
FGF5	0.67097	0.32903	0.284148785	0.265838	5.048106941	19.01702977
LINC0112	0.672437	0.327563	1.559544954	1.561495	218.0782198	139.6563124
ENTPD2	0.672998	0.327002	0.595540482	0.593976	74.71198272	125.7897282
DEPTOR	0.673007	0.326993	1.456654224	1.457176	635.0518532	435.8069323
CGGBP1	0.673592	0.326408	0.694090084	0.694059	3446.847419	4966.218088
C3	0.67417	0.32583	0.677681279	0.677676	21221.23196	31314.70903
C15orf62	0.674831	0.325169	2.567766937	2.63191	31.29826303	11.88564361
KLF6	0.675238	0.324762	1.43573413	1.435831	3142.951381	2188.939365
LINC0105	0.675731	0.324269	1.437184319	1.437266	3738.183767	2600.895772
TPRN	0.676084	0.323916	0.666292294	0.665818	228.1744337	342.7027241
ALG14	0.677123	0.322877	1.610152299	1.613063	164.5682863	102.018441

APLF	0.67813	0.32187	1.485172774	1.486046	401.8293125	270.3983921
ADGRF1	0.678527	0.321473	1.654019795	1.657875	136.2988874	82.20903496
RABEPK	0.678813	0.321187	1.443837544	1.444188	885.4379574	613.1011162
KCNMB2-/	0.679049	0.320951	0.146692297	0.103858	1.019717602	9.904703008
ACOT13	0.681497	0.318503	1.452105875	1.452576	680.5252005	468.4924523
TMEM229	0.682322	0.317678	0.443647975	0.436993	18.17318499	41.59975263
PER2	0.682765	0.317235	0.678954923	0.678621	317.2331364	467.4722679
FIGN	0.68307	0.31693	1.469957712	1.470653	483.6086449	328.8361399
NFATC2	0.683327	0.316673	0.421090209	0.413404	15.14432082	36.64740113
PIR-FIGF	0.683471	0.316529	2.696693369	2.779789	28.23911023	10.15232058
SLC6A17	0.685133	0.314867	0.625672959	0.624586	107.0198671	171.351362
SGOL1	0.6854	0.3146	1.471669845	1.472419	461.3363971	313.3154702
MFAP1	0.685945	0.314055	0.695235181	0.695093	722.8889139	1039.993816
ZC3H12A	0.686111	0.313889	0.697718933	0.69762	1039.91003	1490.657803
CCDC36	0.686299	0.313701	0.307768913	0.291579	6.057728329	20.79987632
IL11RA	0.686588	0.313412	0.578974093	0.577023	60.57728329	104.9898519
VASH2	0.689022	0.310978	0.287062912	0.268635	5.048106941	18.81893571
PLEKHB1	0.68926	0.31074	0.262093102	0.240295	4.038485553	16.83799511
RASA4B	0.689519	0.310481	0.51609008	0.512339	32.88336861	64.19238019
SHF	0.690608	0.309392	0.569738712	0.56751	54.51955496	96.07561918
TIE1	0.69279	0.30721	0.4857917	0.480917	25.2405347	52.49492594
FKBP9P1	0.692994	0.307006	0.201839868	0.17108	2.150493557	12.61859163
CHRM4	0.693299	0.306701	0.287468986	0.268635	5.048106941	18.81893571
TNFRSF1	0.693955	0.306045	0.539518244	0.536554	40.38485553	75.27574286
CRB3	0.694439	0.305561	1.458244103	1.458829	556.3013849	381.3310658
SEPT6	0.694582	0.305418	0.694660478	0.694496	625.9652607	901.3279737
PYROXD1	0.694745	0.305255	1.445101367	1.445523	743.0813417	514.0540861
GBP3	0.695151	0.304849	1.457832783	1.458437	550.2436565	377.2800423
TMEM86A	0.698254	0.301746	0.627025691	0.62593	106.0102458	169.3704214
PPIH	0.699067	0.300933	1.449824846	1.450319	642.1192029	442.7402244
IL6R	0.699192	0.300808	1.552164351	1.554116	213.9690608	137.6753718
CHMP3	0.6992	0.3008	1.433220496	1.433291	4406.442068	3074.350481
RAB39B	0.699932	0.300068	0.620547333	0.619367	96.92365326	156.4943075
TOX4	0.70028	0.29972	0.698536592	0.698411	831.5140791	1190.58492
RASA4	0.700919	0.299081	0.619704263	0.618477	95.33854768	154.1567976
TAF6L	0.701826	0.298174	0.670171089	0.669689	228.1744337	340.7217835
KCNJ12	0.702262	0.297738	0.487522681	0.482647	25.2405347	52.30673658
RIMBP3B	0.70322	0.29678	0.075020899	0.001595	0	6.259772301
LINC0126	0.703238	0.296762	1.544693981	1.54654	222.1167054	143.6181936
NEURL3	0.703678	0.296322	0.416100137	0.407905	14.13469943	34.66646053
OBSCN	0.704448	0.295552	0.695336368	0.695164	599.7151046	862.699632
LOC2843	0.704739	0.295261	2.703263028	2.788145	27.21939262	9.756132463
BTBD10	0.704888	0.295112	1.42706121	1.427179	2568.476811	1799.684537
PORCN	0.70499	0.29501	1.430126614	1.430208	3770.935885	2636.631941
EFHC2	0.705078	0.294922	0.472704212	0.467307	22.21167054	47.54257444
ENPP6	0.705078	0.294922	0.472704212	0.467307	22.21167054	47.54257444
CDKN2AIF	0.706424	0.293576	1.427619375	1.427812	1545.730345	1082.584039
MAPK11	0.706452	0.293548	0.596336557	0.594646	70.67349717	118.8564361
THAP9	0.70741	0.29259	1.498661975	1.499855	311.9730089	207.9987632
NEMP1	0.707503	0.292497	1.428217781	1.428317	3000.594766	2100.787508
METTL4	0.708331	0.291669	1.432777608	1.433076	1010.63101	705.2148542
PIM1	0.709673	0.290327	1.426121007	1.426282	1836.501305	1287.611391
AKT3	0.709805	0.290195	1.426459027	1.426668	1449.816313	1016.222529
GALNT15	0.711962	0.288038	13.19609849	606.7728	6.057728329	0
IL31RA	0.711962	0.288038	13.19609849	606.7728	6.057728329	0
LOC10192	0.711962	0.288038	13.19609849	606.7728	6.057728329	0

RAD51AP	0.712352	0.287648	1.461168766	1.461847	483.6086449	330.8170805
TMED10	0.712513	0.287487	0.693377708	0.693361	6077.769314	8765.672067
ANKDD1A	0.713087	0.286913	0.484753538	0.47979	24.23091332	50.51398534
GPRIN2	0.713729	0.286271	1.866290868	1.87749	70.67349717	37.63787143
CENPH	0.71374	0.28626	1.425306909	1.425511	1481.114576	1039.003346
RNF40	0.714059	0.285941	0.700736726	0.700688	2075.781574	2962.49667
CENPW	0.714798	0.285202	1.469990147	1.470789	421.0121189	286.2459169
PDGFRA	0.715367	0.284633	0.375481199	0.364278	10.09621388	27.73316842
METTL10	0.715418	0.284582	1.432215333	1.432532	952.072969	664.6055718
C18orf54	0.715993	0.284007	1.434387177	1.434737	868.2743938	605.1773538
CLDN15	0.716201	0.283799	0.620838735	0.619621	93.8947891	151.541956
TSPYL4	0.716434	0.283566	0.688377667	0.688103	379.6176419	551.6919575
KIF18B	0.717269	0.282731	1.427285357	1.42753	1213.74664	850.2395156
PLS3-AS1	0.717317	0.282683	1.884771648	1.896845	67.64463301	35.65693083
CDH16	0.717957	0.282043	1.437949655	1.43799	7577.208518	5269.302
TBX19	0.718073	0.281927	1.679183155	1.684019	115.0968383	68.34245075
DUSP5	0.719767	0.280233	1.423242304	1.423367	2407.947011	1691.723274
LINC0066	0.719983	0.280017	1.559146623	1.561318	194.8569279	124.7992579
KCNRG	0.721239	0.278761	0.471017008	0.465288	21.37368479	45.94791725
KIAA1107	0.722208	0.277792	1.545606899	1.547564	210.0012487	135.6944312
LOC10272	0.722255	0.277745	0.630678917	0.629611	106.0102458	168.3799511
ACSF3	0.722285	0.277715	0.686596399	0.686291	343.271272	500.1875019
AMMECR	0.722415	0.277585	0.699708514	0.699566	717.840807	1026.127232
GNG7	0.723167	0.276833	0.410514261	0.401739	13.12507805	32.68551993
PDCL2	0.723736	0.276264	12.93166118	606.7728	6.057728329	0
BACH1	0.724297	0.275703	0.702594895	0.702534	1656.788698	2358.309786
TMEM92	0.724992	0.275008	0.638756174	0.637786	118.1257024	185.2179462
ARHGDIB	0.728023	0.271977	3.735768729	4.069594	16.15394221	3.961881203
IKZF3	0.728137	0.271863	1.711405294	1.717256	101.6183927	59.17069577
CHRNA3	0.728379	0.271621	0.365092575	0.353098	9.086592493	25.75222782
MAGEA6	0.728578	0.271422	0.076604613	0.001674	0	5.962631211
TMEM9B-	0.730321	0.269679	0.560887018	0.558261	46.44258386	83.19950527
SHC3	0.731205	0.268795	0.076840341	0.00168	0	5.942821805
TNNT2	0.731205	0.268795	0.076840341	0.00168	0	5.942821805
HDGF	0.731248	0.268752	1.444651936	1.444676	12773.7298	8841.928375
C1QL1	0.73204	0.26796	0.687440103	0.687129	336.2039223	489.2923286
GDF1	0.73234	0.26766	0.610716313	0.60923	79.40672218	130.3458916
ABHD10	0.733294	0.266706	1.419855681	1.420042	1592.172929	1121.21238
ADAT3	0.733433	0.266567	0.4837255	0.478571	23.22129193	48.53304474
LINC0089	0.733639	0.266361	0.445543906	0.438599	17.03231282	38.8462452
HSPBAP1	0.733716	0.266284	1.494804956	1.496015	297.8383095	199.0845305
BARX2	0.734513	0.265487	0.07713219	0.00168	0	5.942821805
CDC42EP	0.734513	0.265487	0.07713219	0.00168	0	5.942821805
CDH15	0.734513	0.265487	0.07713219	0.00168	0	5.942821805
CHRDL2	0.734513	0.265487	0.07713219	0.00168	0	5.942821805
CXCL6	0.734513	0.265487	0.07713219	0.00168	0	5.942821805
DCLK3	0.734513	0.265487	0.07713219	0.00168	0	5.942821805
DUSP15	0.734513	0.265487	0.07713219	0.00168	0	5.942821805
FGF14-AS	0.734513	0.265487	0.07713219	0.00168	0	5.942821805
FMOD	0.734513	0.265487	0.07713219	0.00168	0	5.942821805
IGSF10	0.734513	0.265487	0.07713219	0.00168	0	5.942821805
IL3RA	0.734513	0.265487	0.07713219	0.00168	0	5.942821805
IL7R	0.734513	0.265487	0.07713219	0.00168	0	5.942821805
NINJ2	0.734513	0.265487	0.07713219	0.00168	0	5.942821805
NYNRIN	0.734513	0.265487	0.07713219	0.00168	0	5.942821805
SLC6A13	0.734513	0.265487	0.07713219	0.00168	0	5.942821805

VCAM1	0.734513	0.265487	0.07713219	0.00168	0	5.942821805
TMEM104	0.734906	0.265094	0.632110753	0.631039	105.0006244	166.3990105
TRERF1	0.736214	0.263786	1.466518901	1.467378	396.7812055	270.3983921
NCMAP	0.73658	0.26342	0.203187541	0.170587	2.019242776	11.88564361
BIRC5	0.737029	0.262971	1.419603966	1.419738	2151.503178	1515.41956
CBX4	0.73744	0.26256	0.691446326	0.691174	380.6272633	550.7014872
RRP36	0.737738	0.262262	0.699512524	0.69933	571.4457057	817.1379981
MAPK8IP2	0.737743	0.262257	0.454335047	0.447649	18.17318499	40.60928233
ASIC1	0.738098	0.261902	0.671398933	0.670883	207.982006	310.0172041
FHOD1	0.738098	0.261902	0.671398933	0.670883	207.982006	310.0172041
XK	0.73838	0.26162	1.63205723	1.635891	131.2507805	80.22809436
GLIS3	0.738757	0.261243	0.701754479	0.70161	706.7349717	1007.308296
RRAS2	0.739051	0.260949	1.428101329	1.428153	5847.72708	4094.604223
GCAT	0.739609	0.260391	0.624935631	0.623723	93.90488531	150.5613904
PDK1	0.739879	0.260121	0.693278285	0.693013	398.8004483	575.4632447
DAPK2	0.741214	0.258786	0.576988322	0.574777	53.50993357	93.10420827
ENTPD4	0.741384	0.258616	0.704808163	0.704744	1584.86327	2248.852913
KCNJ16	0.741687	0.258313	0.4042191	0.394779	12.11545666	30.70457932
SLC40A1	0.741906	0.258094	0.642772574	0.641823	120.1449452	187.1988868
NOTCH4	0.742233	0.257767	0.203895289	0.170587	2.019242776	11.88564361
SLC26A1	0.742233	0.257767	0.203895289	0.170587	2.019242776	11.88564361
MON1B	0.7428	0.2572	0.688889823	0.688578	334.1846795	485.3304474
IAPP	0.743171	0.256829	12.72858138	583.5515	5.82551541	0
IL6	0.743807	0.256193	0.683268258	0.682888	276.6362604	405.102353
HR	0.743826	0.256174	1.41746465	1.417616	1908.184424	1346.049139
PDE10A	0.744031	0.255969	1.417415782	1.41757	1868.809189	1318.31597
ARHGAP1	0.744174	0.255826	1.62948933	1.633409	130.5339493	79.91114387
DDX26B	0.744885	0.255115	0.570034636	0.567634	49.47144802	87.16138647
VPS39	0.744891	0.255109	0.704774846	0.704659	897.5534141	1273.744807
STIL	0.745199	0.254801	1.416319165	1.416478	1844.235005	1301.983115
CHRNE	0.745693	0.254307	0.078707098	0.00168	0	5.942821805
KANK4	0.745693	0.254307	0.078707098	0.00168	0	5.942821805
KIAA1257	0.745693	0.254307	0.078707098	0.00168	0	5.942821805
LOC10050	0.745693	0.254307	0.078707098	0.00168	0	5.942821805
ZNF821	0.74591	0.25409	0.614619315	0.613163	80.76971105	131.73255
AMIGO2	0.745984	0.254016	0.700785769	0.70076	3915.311743	5587.242967
GABRP	0.746275	0.253725	0.469381763	0.463457	20.19242776	43.58069323
LOC64624	0.746488	0.253512	0.566191816	0.563684	47.45220524	84.18997557
BTC	0.746623	0.253377	1.432067017	1.432514	689.5714081	481.3685662
TPTEP1	0.746835	0.253165	1.45183504	1.452515	469.4436569	323.1904591
FKBP2	0.748553	0.251447	1.417138941	1.417339	1441.739342	1017.212999
NCR3LG1	0.749476	0.250524	1.442580197	1.443152	543.1763068	376.3787143
ZNF257	0.751799	0.248201	1.450726526	1.451411	464.9609379	320.3478094
ABHD3	0.75254	0.24746	1.415177842	1.415339	1778.952886	1256.906812
EME2	0.754335	0.245665	0.339392018	0.324644	7.067349717	21.79034662
SYT12	0.754725	0.245275	0.482776586	0.477248	22.21167054	46.55210414
GADD45B	0.754744	0.245256	1.414587927	1.41475	1762.798944	1246.011638
ZNF551	0.755406	0.244594	1.44294964	1.443571	511.8780438	354.5883677
WDR31	0.755612	0.244388	1.474287505	1.475333	333.1750581	225.8272286
SPAG5	0.756001	0.243999	1.414215725	1.414374	1800.154935	1272.754336
CHST10	0.756213	0.243787	0.676946066	0.67645	219.0878412	323.8837884
PDLIM7	0.757105	0.242895	0.706957777	0.706871	1167.122325	1651.113991
AK8	0.757202	0.242798	0.617596479	0.616194	81.77933244	132.7230203
ATP5G1	0.758377	0.241623	1.414526069	1.414728	1412.460322	998.3940632
HIST1H3H	0.758529	0.241471	6.40399936	9.092316	9.086592493	0.990470301
RNF217-A	0.758826	0.241174	2.816710268	2.928156	23.22129193	7.923762406

ADGRE5	0.760608	0.239392	1.413018556	1.413185	1706.260146	1207.383297
IER5L	0.760797	0.239203	0.707091549	0.70704	2000.05997	2828.783179
HPSE	0.761209	0.238791	1.434418194	1.43493	592.6679473	413.0261154
CASKIN1	0.761406	0.238594	0.683602895	0.68318	254.4245898	372.4168331
IPW	0.761407	0.238593	0.57143038	0.568982	48.46182663	85.18044587
HSD11B1	0.761866	0.238134	0.24545637	0.218992	3.028864164	13.86658421
NTNG2	0.761866	0.238134	0.24545637	0.218992	3.028864164	13.86658421
SHISA2	0.761866	0.238134	0.24545637	0.218992	3.028864164	13.86658421
SSH2	0.762776	0.237224	1.411521462	1.411675	1889.001617	1338.125376
SMG1P1	0.762897	0.237103	1.415386226	1.415636	1146.314028	809.7490897
TPPP3	0.763653	0.236347	0.322780952	0.306151	6.057728329	19.80940602
KCNK1	0.764819	0.235181	1.411922332	1.412037	2523.043849	1786.808423
FBXL8	0.76556	0.23444	0.530660914	0.526982	32.71173298	62.08267845
MEGF11	0.766419	0.233581	0.246034211	0.218992	3.028864164	13.86658421
TMEM198	0.766419	0.233581	0.246034211	0.218992	3.028864164	13.86658421
ADSSL1	0.76703	0.23297	0.665278062	0.66462	164.5682863	247.6175752
MTFR2	0.767065	0.232935	1.450474368	1.451242	424.040983	292.1887387
RALGPS1	0.767251	0.232749	1.423584887	1.423978	746.1102059	523.9587891
ZNF850	0.768448	0.231552	1.42365245	1.424071	717.4268622	503.7829091
MFGE8	0.76939	0.23061	1.410908417	1.411095	1516.451325	1074.660276
ZNF462	0.769431	0.230569	0.30257859	0.283551	5.048106941	17.82846541
HUNK	0.769992	0.230008	0.277479124	0.255304	4.038485553	15.84752481
TPBGL	0.769992	0.230008	0.277479124	0.255304	4.038485553	15.84752481
ZNF280A	0.769992	0.230008	0.277479124	0.255304	4.038485553	15.84752481
FOXO1	0.770738	0.229262	1.41924357	1.419299	5237.915762	3690.492341
MICALCL	0.771724	0.228276	1.593650935	1.596892	142.3566157	89.14232707
LOC10192	0.773311	0.226689	12.24073744	559.3206	5.583206277	0
MCF2L-AS	0.773931	0.226069	0.277965424	0.255304	4.038485553	15.84752481
CYP2U1	0.776058	0.223942	0.696712816	0.696422	361.444457	519.0064376
ZNRF1	0.776144	0.223856	1.407762184	1.407932	1679.000369	1192.526242
PRC1	0.776261	0.223739	1.414447145	1.414516	4141.466934	2927.830209
CIITA	0.776366	0.223634	0.389252595	0.378142	10.1063101	26.74269812
THPO	0.776681	0.223319	0.15987852	0.114253	1.009621388	8.914232707
SLC6A6	0.776804	0.223196	1.484306575	1.485601	276.6362604	186.2084165
SLC7A2	0.776822	0.223178	1.42555495	1.42559	8371.780551	5872.498413
GOLGA8J	0.777144	0.222856	0.081979958	0.001756	0	5.685299526
DPP7	0.777153	0.222847	0.707398414	0.707255	704.7157289	996.4131226
CERKL	0.777797	0.222203	0.493720797	0.488539	23.22129193	47.54257444
CEP57L1	0.778394	0.221606	1.454777906	1.455624	380.6272633	261.4841594
LOC11511	0.778495	0.221505	0.160060257	0.114253	1.009621388	8.914232707
LZTS1	0.778495	0.221505	0.160060257	0.114253	1.009621388	8.914232707
PTN	0.778495	0.221505	0.160060257	0.114253	1.009621388	8.914232707
TMEM154	0.778694	0.221306	0.622254066	0.620827	81.77933244	131.73255
BATF3	0.780269	0.219731	0.590645456	0.588525	56.53879774	96.07561918
CA2	0.780886	0.219114	1.408422515	1.40866	1205.487937	855.7663399
HECW2	0.781035	0.218965	0.564739631	0.561998	43.41371969	77.25668346
SEMA3B	0.782372	0.217628	1.409329163	1.409421	3064.200913	2174.08231
LINC0064	0.782609	0.217391	0.637456681	0.636296	98.94289604	155.5038372
ADAM10	0.783321	0.216679	0.702135058	0.702118	5854.79443	8338.769462
TARBP1	0.783513	0.216487	0.705886982	0.705698	545.1955496	772.5668346
HAUS8	0.783667	0.216333	1.437338219	1.437978	478.560538	332.7980211
ANO9	0.78393	0.21607	0.555237705	0.552203	39.37523414	71.31386166
WDR62	0.784077	0.215923	1.42471605	1.425194	616.8786682	432.8355214
PDGFD	0.784705	0.215295	0.161041595	0.114253	1.009621388	8.914232707
ZNF469	0.784705	0.215295	0.161041595	0.114253	1.009621388	8.914232707
MTMR6	0.784763	0.215237	0.710337641	0.710216	842.0242377	1185.59295

TSNAXIP1	0.785715	0.214285	0.58476557	0.582524	52.50031218	90.13279737
MMP13	0.785891	0.214109	0.530579953	0.526736	31.29826303	59.42821805
GLMP	0.78604	0.21396	0.537618071	0.534012	33.31750581	62.39962895
BID	0.787047	0.212953	0.693677674	0.693339	304.9056592	439.7688135
DAZAP1	0.787052	0.212948	1.408942244	1.409022	3610.406084	2562.346668
TIAF1	0.787325	0.212675	4.431078805	5.148476	11.97410966	2.317700504
CCDC106	0.787789	0.212211	0.673891372	0.6733	180.7222285	268.4174515
CNGA1	0.789548	0.210452	1.506624208	1.508306	221.107084	146.5896045
COL12A1	0.789961	0.210039	0.705148409	0.705127	4631.133307	6567.808564
SKAP1	0.790259	0.209741	1.768746552	1.777728	75.72160411	42.59022293
DACT2	0.790308	0.209692	0.578168493	0.575675	48.46182663	84.18997557
HACL1	0.79058	0.20942	1.452947727	1.453854	361.444457	248.6080455
DLGAP5	0.791353	0.208647	1.404176428	1.40439	1325.632883	943.9181966
LRRC37A	0.791471	0.208529	1.744343382	1.752231	80.46682464	45.91820314
UTP18	0.791871	0.208129	0.711839555	0.711779	1635.586649	2297.891098
PDZK1	0.792154	0.207846	0.480304558	0.474233	20.19242776	42.59022293
CSPG5	0.792179	0.207821	0.672292038	0.671668	171.635636	255.5413376
LOC10192	0.792336	0.207664	1.582387745	1.585567	141.3469943	89.14232707
HSH2D	0.792436	0.207564	3.052544097	3.224153	19.18280638	5.942821805
MAN1C1	0.792703	0.207297	0.37967945	0.367216	9.086592493	24.76175752
MMP14	0.792704	0.207296	0.444812617	0.43702	15.14432082	34.66646053
PDZK1IP1	0.793515	0.206485	0.712439676	0.712358	1220.632258	1713.51362
BCHE	0.793759	0.206241	1.62121686	1.625341	119.1353238	73.29480226
GTF2IRD2	0.793971	0.206029	0.58965707	0.587499	54.13589883	92.15335678
C2orf72	0.794279	0.205721	0.683227267	0.682742	217.0685985	317.9409665
RBM47	0.794453	0.205547	1.403220968	1.403352	2103.041352	1498.581565
SP140	0.794629	0.205371	1.77294727	1.782193	74.14659475	41.59975263
HSD17B1	0.794645	0.205355	2.016027125	2.038215	46.44258386	22.78081692
SERTAD4	0.795279	0.204721	1.780505915	1.789858	72.69273995	40.60928233
OBFC1	0.795994	0.204006	1.40334613	1.40355	1349.863796	961.7466621
CXCL3	0.797043	0.202957	0.603785546	0.601932	62.59652607	103.9993816
ADAM11	0.797766	0.202234	0.621275253	0.619787	76.7312255	123.8087876
B3GNT7	0.797868	0.202132	0.61328752	0.611635	69.66387578	113.9040846
BMS1P5	0.799155	0.200845	1.433436872	1.434118	453.8954875	316.4948799
RPL39L	0.79933	0.20067	1.409403145	1.409739	836.3905504	593.2917102
MAGOHB	0.799495	0.200505	1.455837256	1.456799	336.2039223	230.7795801
ITGA4	0.799851	0.200149	0.658005533	0.657153	128.2219163	195.1226493
UBA6-AS1	0.799862	0.200138	1.434056124	1.434725	453.3200033	315.9600259
PXN-AS1	0.800269	0.199731	1.670035212	1.675741	97.93327465	58.43774775
PLEKHM3	0.801272	0.198728	0.695801308	0.695444	295.0517545	424.2679533
CYP2J2	0.802178	0.197822	0.523280934	0.519023	28.26939887	54.47586654
YJEFN3	0.802178	0.197822	0.523280934	0.519023	28.26939887	54.47586654
DCUN1D2	0.803034	0.196966	1.427614568	1.428213	496.3601631	347.5362191
CDHR3	0.803056	0.196944	0.214511088	0.179926	1.978857921	11.04374385
IL15RA	0.803212	0.196788	1.414094054	1.414535	661.3020092	467.501982
NYAP1	0.803315	0.196685	0.463258617	0.456163	17.1635636	37.63787143
CASP1	0.804644	0.195356	0.448256346	0.440457	15.15441704	34.41884295
OSBPL11	0.804824	0.195176	1.400392535	1.4005	2607.852046	1862.084165
PLEKHA4	0.805016	0.194984	0.579753057	0.577265	47.45220524	82.20903496
PTRF	0.805474	0.194526	0.704218568	0.704203	6529.221517	9271.792486
TTC27	0.805617	0.194383	1.405395749	1.405703	903.6111424	642.8152252
C4orf33	0.805751	0.194249	1.508540557	1.510364	204.9531418	135.6944312
CR2	0.806251	0.193749	0.368622545	0.354835	8.076971105	22.78081692
SHE	0.806251	0.193749	0.368622545	0.354835	8.076971105	22.78081692
FAM131C	0.806813	0.193187	0.682390781	0.681864	199.9050349	293.179209
TMEM87A	0.80714	0.19286	0.713843867	0.713717	801.6393822	1123.193321

FAM89A	0.807276	0.192724	0.576092252	0.57343	45.43296247	79.23762406
LRP4-AS1	0.807664	0.192336	3.927727845	4.405658	13.12507805	2.971410902
DAAM2	0.80824	0.19176	0.629858005	0.628491	82.78895383	131.73255
BOLA2	0.808427	0.191573	1.425869977	1.426471	491.685616	344.6836647
BOLA2B	0.808427	0.191573	1.425869977	1.426471	491.685616	344.6836647
WNT7A	0.808799	0.191201	1.399322969	1.399533	1293.324998	924.1087906
NBPF8	0.809886	0.190114	0.713046133	0.712906	709.7739321	995.6108416
TSNARE1	0.810061	0.189939	0.677593937	0.676976	175.6741215	259.5032188
PPIB	0.810179	0.189821	0.711233266	0.711202	3169.201537	4456.125883
TMEM54	0.810334	0.189666	0.710190199	0.709996	526.0127432	740.871785
MYL12B	0.810365	0.189635	1.42891701	1.428936	15782.2501	11044.75413
MTMR11	0.810643	0.189357	1.422076001	1.422638	519.9550149	365.483541
ARHGAP1	0.811026	0.188974	1.416740991	1.417234	582.551541	411.0451748
TMEM132	0.811213	0.188787	0.441126248	0.432619	14.13469943	32.68551993
ATP6V0D1	0.811288	0.188712	0.714961263	0.714861	981.3519893	1372.791837
RIBC2	0.811346	0.188654	0.628715514	0.627312	80.76971105	128.7611391
ZFPM2-AS	0.811681	0.188319	1.564364112	1.567413	144.3859547	92.11373797
CGN	0.811862	0.188138	1.408783255	1.409192	700.6772434	497.216091
RAD18	0.812118	0.187882	1.409568878	1.409971	699.667622	496.2256207
CD99	0.812317	0.187683	0.715162735	0.715058	949.0441049	1327.230203
ZFP36	0.812534	0.187466	1.401126563	1.401402	992.4578246	708.1862651
ZNF733P	0.812702	0.187298	0.50171484	0.496356	22.95879037	46.26486775
ZNF844	0.812835	0.187165	1.426128015	1.426778	465.5768069	326.3104406
FHOD3	0.81319	0.18681	0.712029274	0.711863	600.724726	843.8806963
TLCD2	0.81347	0.18653	0.677276753	0.676666	173.3418961	256.1752386
SMIM11B	0.814031	0.185969	1.42585878	1.426487	470.4835669	329.8167055
GPR176	0.814774	0.185226	0.7140055	0.713856	679.4751942	951.841959
HTATIP2	0.814904	0.185096	1.396521912	1.396695	1550.778452	1110.317207
VPS18	0.814907	0.185093	0.713134948	0.712981	646.1576884	906.2803252
PEX11G	0.815433	0.184567	0.601098828	0.599033	57.54841913	96.07561918
SPATA5L1	0.815487	0.184513	0.700977726	0.700663	324.0884656	462.5496305
SLC29A4	0.81551	0.18449	0.671354218	0.67063	151.4432082	225.8272286
CFAP221	0.815614	0.184386	0.355176612	0.340096	7.067349717	20.79987632
SMARCA2	0.816281	0.183719	0.709654895	0.709437	471.4931883	664.6055718
PRR29-AS	0.81681	0.18319	11.44799104	519.9454	5.189453935	0
FLJ22447	0.816905	0.183095	0.699984225	0.699642	304.9056592	435.8069323
PARP11	0.816952	0.183048	1.474915669	1.476251	254.4245898	172.3418323
BCL2L12	0.817072	0.182928	1.41981012	1.420374	514.906908	362.5121301
DDX10	0.817425	0.182575	1.398063082	1.398314	1080.294885	772.5668346
BARX1	0.817461	0.182539	0.514536608	0.509767	25.2405347	49.52351504
LOC90768	0.817898	0.182102	1.467459435	1.468694	270.578532	184.2274759
TTLL7	0.818296	0.181704	0.539497042	0.535662	31.29826303	58.43774775
PRKAR2B	0.818307	0.181693	0.701657249	0.701344	325.098087	463.5401008
PHF24	0.818723	0.181277	0.539583041	0.535662	31.29826303	58.43774775
MDH1B	0.818741	0.181259	0.460552331	0.453191	16.15394221	35.65693083
SLMO2-AT	0.819074	0.180926	1.405427094	1.405826	711.2984604	505.9619437
CAMKK1	0.819088	0.180912	0.649181892	0.648118	103.991003	160.4561887
GAA	0.819116	0.180884	0.714919893	0.714876	2261.551909	3163.562141
KBTBD6	0.81926	0.18074	0.699998591	0.699662	303.8960378	434.350941
PAG1	0.820769	0.179231	0.551978974	0.548467	34.81174546	63.47924158
ADAP2	0.821078	0.178922	0.667308368	0.666505	137.3085088	206.0178226
SYNE2	0.821485	0.178515	1.397626635	1.397708	3412.520292	2441.509291
WIPF1	0.822574	0.177426	0.717833356	0.717743	1119.670119	1559.990724
ANKZF1	0.822779	0.177221	0.714056455	0.713893	608.8016971	852.794929
HERC2P3	0.823804	0.176196	0.714780643	0.714615	614.0820169	859.3221282
CH17-360	0.824042	0.175958	2.436693596	2.502059	26.75496679	10.68717455

SYT3	0.824125	0.175875	0.64804236	0.646909	99.95251743	154.5133669
RASSF1	0.824356	0.175644	1.404464503	1.404858	702.6964862	500.1875019
HCP5	0.824616	0.175384	1.393000767	1.393185	1444.768206	1037.022405
ANLN	0.824645	0.175355	1.398549142	1.398621	3752.7627	2683.184045
CCDC120	0.824757	0.175243	0.670066874	0.669314	142.7200794	213.2383511
WNT6	0.825426	0.174574	0.339332571	0.322256	6.057728329	18.81893571
PCDHGA6	0.825603	0.174397	0.595145339	0.592917	52.40944626	88.39947434
SNORA81	0.825665	0.174335	0.22160593	0.186081	2.019242776	10.89517331
TMEM63C	0.825665	0.174335	0.22160593	0.186081	2.019242776	10.89517331
LOC10192	0.825805	0.174195	1.798611242	1.809554	64.26240136	35.50836028
AHRR	0.826059	0.173941	0.689758751	0.689275	215.0493557	311.9981447
OCA2	0.826387	0.173613	1.638921499	1.643984	100.9621388	61.40915865
COX7B2	0.82654	0.17346	1.435671523	1.436498	368.5118067	256.5318079
CCDC74B	0.826567	0.173433	0.430490427	0.421144	12.47892036	29.6447761
CCDC64	0.826984	0.173016	0.590035507	0.587666	49.47144802	84.18997557
ST14	0.82716	0.17284	0.436776149	0.42765	13.12507805	30.70457932
SMIM20	0.827748	0.172252	1.39264929	1.392862	1249.911279	897.3660925
NEO1	0.828067	0.171933	0.717525093	0.717472	1840.539791	2565.318079
DEF6	0.828067	0.171933	0.339708874	0.322256	6.057728329	18.81893571
TM4SF19	0.828384	0.171616	11.20524359	505.8107	5.048106941	0
NECAB1	0.828533	0.171467	0.403805949	0.392288	10.09621388	25.75222782
SNAPC3	0.828592	0.171408	1.411272842	1.411804	544.1859282	385.4514222
TCN2	0.828724	0.171276	1.41248753	1.413036	529.0416074	374.3977737
VRK2	0.828899	0.171101	1.391138818	1.391297	1672.94264	1202.430945
PTPN6	0.828934	0.171066	1.458787246	1.459961	277.6458817	190.1702977
FDXR	0.829916	0.170084	0.668817238	0.668027	136.2988874	204.036882
C1orf21	0.830688	0.169312	0.712193498	0.711971	458.3681102	643.8056955
CCL26	0.830772	0.169228	11.16341541	505.8107	5.048106941	0
CSF1R	0.830772	0.169228	11.16341541	505.8107	5.048106941	0
LOC10012	0.830772	0.169228	11.16341541	505.8107	5.048106941	0
LOC10099	0.830772	0.169228	11.16341541	505.8107	5.048106941	0
LURAP1L-	0.830772	0.169228	11.16341541	505.8107	5.048106941	0
LYG2	0.830772	0.169228	11.16341541	505.8107	5.048106941	0
PTHLH	0.831038	0.168962	0.553670223	0.55019	34.3271272	62.39962895
GLIPR1	0.831055	0.168945	0.600483213	0.59835	54.51955496	91.12326767
GLRX	0.831164	0.168836	0.650374919	0.649281	100.9621388	155.5038372
BTN3A2	0.831404	0.168596	1.39386099	1.394131	989.600596	709.8304458
CSRP2BP	0.831776	0.168224	1.389265391	1.389436	1576.160334	1134.385635
ST20-MTH	0.831786	0.168214	0.089048256	0.001964	0	5.081112643
ATP6V1C2	0.831898	0.168102	0.319787456	0.30022	5.048106941	16.83799511
HMGN2P4	0.832157	0.167843	0.319684679	0.300044	5.048106941	16.84789982
LXN	0.832193	0.167807	1.458050078	1.459235	274.6170176	188.1893571
TIMELESS	0.832329	0.167671	1.395733261	1.395803	3930.456064	2815.907065
FAM47E	0.832592	0.167408	0.528063971	0.52366	27.18910398	51.93035787
HOXB-AS3	0.832592	0.167408	0.619003913	0.617248	66.63501162	107.9612628
LINC0109	0.832707	0.167293	0.319867341	0.30022	5.048106941	16.83799511
RASL11A	0.832707	0.167293	0.319867341	0.30022	5.048106941	16.83799511
TLR9	0.832707	0.167293	0.319867341	0.30022	5.048106941	16.83799511
ZNF433	0.832962	0.167038	1.496271239	1.49809	198.8954135	132.7626391
FAM166B	0.833583	0.166417	2.784543815	2.909632	20.19242776	6.933292105
LINC0089	0.833583	0.166417	2.784543815	2.909632	20.19242776	6.933292105
TRMU	0.834045	0.165955	0.694356799	0.693894	228.1744337	328.8361399
EIF3C	0.834369	0.165631	0.694859606	0.694417	233.2023482	335.8288602
PDE3A	0.834495	0.165505	0.26363634	0.235825	3.028864164	12.87611391
DKK3	0.834925	0.165075	0.718637166	0.718589	2081.839302	2897.12563
AP4B1-AS	0.835062	0.164938	0.207732012	0.168685	1.655779077	9.865084196

PPP4R4	0.835164	0.164836	0.668495854	0.667681	132.2604018	198.0940602
SNX18	0.835314	0.164686	0.7187949	0.718671	791.5431683	1101.402974
C12orf80	0.835511	0.164489	0.320298972	0.30022	5.048106941	16.83799511
TCAF2	0.835511	0.164489	0.320298972	0.30022	5.048106941	16.83799511
TAPT1	0.835961	0.164039	0.716504711	0.716328	570.4360843	796.3381218
SVIL-AS1	0.835981	0.164019	0.708441287	0.708155	358.4155928	506.1303237
LOC72873	0.836211	0.163789	1.405615237	1.40609	585.0453058	416.0767639
ELL3	0.836447	0.163553	0.295379302	0.272313	4.038485553	14.85705451
ESM1	0.836447	0.163553	0.295379302	0.272313	4.038485553	14.85705451
JAKMIP3	0.836447	0.163553	0.295379302	0.272313	4.038485553	14.85705451
LHX9	0.836447	0.163553	0.295379302	0.272313	4.038485553	14.85705451
MKS1	0.836536	0.163464	0.704612176	0.704275	306.924902	435.8069323
ZNF823	0.837481	0.162519	1.452018093	1.453183	280.6747459	193.1417087
LPAR2	0.837801	0.162199	0.696641508	0.696212	239.280269	343.6931944
AQP7P1	0.837941	0.162059	1.794299202	1.805447	62.59652607	34.66646053
BEGAIN	0.837972	0.162028	0.264241991	0.235825	3.028864164	12.87611391
NAP1L3	0.837972	0.162028	0.264241991	0.235825	3.028864164	12.87611391
SNHG3	0.838313	0.161687	1.387046065	1.387238	1389.713552	1001.781472
CBS	0.838358	0.161642	0.720756262	0.720655	991.4482032	1375.763248
PDE4A	0.838692	0.161308	0.630135771	0.628621	74.71198272	118.8564361
CXCL10	0.838784	0.161216	10.94305098	505.8107	5.048106941	0
TBX20	0.838784	0.161216	10.94305098	505.8107	5.048106941	0
TCTEX1D	0.838784	0.161216	10.94305098	505.8107	5.048106941	0
WISP2	0.838944	0.161056	1.415282677	1.415903	461.3969744	325.864729
SBF2-AS1	0.839151	0.160849	1.444208996	1.445234	304.9056592	210.9701741
NRSN2	0.839495	0.160505	0.591960451	0.589545	48.46182663	82.20903496
OTOF	0.839502	0.160498	0.295884126	0.272313	4.038485553	14.85705451
REC8	0.839502	0.160498	0.295884126	0.272313	4.038485553	14.85705451
SLC10A5	0.839502	0.160498	0.295884126	0.272313	4.038485553	14.85705451
FAM25G	0.84004	0.15996	0.090494504	0.001999	0	4.991970316
KIT	0.84006	0.15994	0.475423875	0.468488	17.1635636	36.64740113
ANKMY2	0.840472	0.159528	1.391086647	1.391372	928.8516771	667.5769827
LRRC3	0.840519	0.159481	0.68157593	0.680935	164.5682863	241.6846581
HACD3	0.841106	0.158894	0.717073499	0.71704	2974.34461	4148.08962
SLC2A11	0.841247	0.158753	0.628909071	0.627315	72.69273995	115.8850252
ECM1	0.842561	0.157439	1.903247661	1.920698	49.47144802	25.75222782
DCAF4L1	0.842627	0.157373	5.765109706	8.08317	8.076971105	0.990470301
LINC0094	0.842627	0.157373	5.765109706	8.08317	8.076971105	0.990470301
PLEKHG4	0.842718	0.157282	0.090987083	0.002011	0	4.962256207
KRT5	0.842832	0.157168	1.472205743	1.473693	227.7100079	154.5133669
MPST	0.843027	0.156973	0.718231834	0.718057	577.503434	804.2618842
PAQR4	0.843201	0.156799	0.720482169	0.720346	737.0236134	1023.155821
CCDC168	0.843602	0.156398	0.09115247	0.002015	0	4.952351504
FUT9	0.843602	0.156398	0.09115247	0.002015	0	4.952351504
KRT23	0.843602	0.156398	0.09115247	0.002015	0	4.952351504
LOC10272	0.843602	0.156398	0.09115247	0.002015	0	4.952351504
MIR503HC	0.843602	0.156398	0.09115247	0.002015	0	4.952351504
P2RX3	0.843602	0.156398	0.09115247	0.002015	0	4.952351504
PLA2G7	0.843602	0.156398	0.09115247	0.002015	0	4.952351504
RELN	0.843602	0.156398	0.09115247	0.002015	0	4.952351504
RNF43	0.843602	0.156398	0.09115247	0.002015	0	4.952351504
RUNDC3A	0.843602	0.156398	0.09115247	0.002015	0	4.952351504
RPS14P3	0.843661	0.156339	0.117896367	0.045777	0.262501561	5.942821805
NRXN3	0.844285	0.155715	10.82373437	499.753	4.987529658	0
NR1D1	0.844442	0.155558	1.385262543	1.385405	1826.405091	1318.31597
ORAI2	0.844591	0.155409	0.716361769	0.716165	502.4582762	701.5996376

RBM14	0.844994	0.155006	0.72020072	0.720063	711.7931749	988.5190743
WNT4	0.845158	0.154842	0.55518155	0.551518	33.31750581	60.41868835
SLC15A1	0.845277	0.154723	0.627751246	0.626148	70.73407445	112.9730425
ANKRD6	0.846203	0.153797	0.718837146	0.718671	593.6573762	826.0522308
IGFBPL1	0.84649	0.15351	0.503603986	0.497933	21.20204915	42.59022293
PHACTR2	0.84655	0.15345	1.384248098	1.384404	1657.798319	1197.478594
PCDH18	0.846708	0.153292	2.89926753	3.054549	18.17318499	5.942821805
RASGEF1	0.846859	0.153141	0.619317584	0.617519	63.60614745	103.0089113
FAM135A	0.846925	0.153075	1.384397389	1.384533	1906.165181	1376.753718
KIF7	0.846949	0.153051	0.71060991	0.71033	357.4059714	503.1589128
TAS2R14	0.8471	0.1529	0.091820073	0.002031	0	4.912732692
PLCL2	0.847312	0.152688	0.581378539	0.578599	42.4040983	73.29480226
EFCAB12	0.847407	0.152593	5.730097311	8.08317	8.076971105	0.990470301
MESP2	0.847407	0.152593	5.730097311	8.08317	8.076971105	0.990470301
MTMR2	0.847634	0.152366	1.389104196	1.389181	3420.597263	2462.309168
ZFP36L1	0.848551	0.151449	0.702666165	0.702659	14565.80777	20729.55292
MSRB3	0.848627	0.151373	0.721106735	0.721058	2009.025408	2786.22267
ZNF611	0.848765	0.151235	1.390432461	1.390765	795.2686712	571.818314
PPARG	0.849006	0.150994	1.383481639	1.383668	1384.190923	1000.375004
EIF2A	0.84903	0.15097	1.385053804	1.38516	2450.351109	1768.999767
TSPAN33	0.849654	0.150346	0.700313685	0.699894	243.3187545	347.6550756
SLC6A15	0.849815	0.150185	0.577324951	0.574334	40.38485553	70.32339135
C9orf16	0.850066	0.149934	0.722967147	0.722889	1240.824686	1716.485031
MXRA7	0.850201	0.149799	0.722927785	0.722832	1018.28394	1408.745909
MZT1	0.850258	0.149742	1.383041187	1.383232	1350.873417	976.6037166
SFMBT2	0.850535	0.149465	0.092497528	0.002048	0	4.87311388
GGH	0.850672	0.149328	1.383998871	1.38411	2329.196542	1682.809041
ZNF141	0.850996	0.149004	1.517234829	1.519631	159.6413339	105.0492801
FAM155A	0.851116	0.148884	0.092984804	0.002015	0	4.952351504
GP1BA	0.851116	0.148884	0.092984804	0.002015	0	4.952351504
KCNJ8	0.851116	0.148884	0.092984804	0.002015	0	4.952351504
LINC0148	0.851116	0.148884	0.092984804	0.002015	0	4.952351504
LINC0160	0.851116	0.148884	0.092984804	0.002015	0	4.952351504
NAP1L2	0.851116	0.148884	0.092984804	0.002015	0	4.952351504
PDGFRB	0.851116	0.148884	0.092984804	0.002015	0	4.952351504
UNC5C	0.851116	0.148884	0.092984804	0.002015	0	4.952351504
CCDC61	0.851521	0.148479	1.516941066	1.519337	159.5201793	104.9898519
ADAM9	0.851654	0.148346	1.417951831	1.417968	17586.59496	12402.66911
GPAM	0.851799	0.148201	0.722121555	0.721991	750.1486914	1039.003346
PNPLA3	0.85197	0.14803	0.578257182	0.575339	40.45552902	70.32339135
HCN3	0.851977	0.148023	0.636489974	0.635026	76.7312255	120.8373767
ZNF519	0.852052	0.147948	1.497540349	1.499561	179.7227033	119.8469064
HSPB8	0.852354	0.147646	0.384692846	0.370956	8.076971105	21.79034662
DHRS4	0.852457	0.147543	0.68875082	0.688149	177.0673991	257.3142794
TGFBR1	0.853215	0.146785	0.724019697	0.72392	999.5251743	1380.715599
NUDT12	0.8534	0.1466	1.397471046	1.397936	581.5419196	415.9975263
PTGDS	0.854083	0.145917	0.590709471	0.588131	45.43296247	77.25668346
C7orf31	0.854496	0.145504	0.635442714	0.633903	74.71198272	117.8659658
IRS2	0.854798	0.145202	0.716802016	0.716567	430.0987114	600.2250023
LY6G5B	0.85492	0.14508	0.473525098	0.466136	16.15394221	34.66646053
INCENP	0.855045	0.144955	1.395734142	1.395777	6277.825792	4497.725636
SRGAP3	0.855632	0.144368	0.699090401	0.698619	222.1167054	317.9409665
LOC10013	0.855657	0.144343	0.616011627	0.614072	59.16381335	96.35295086
WDR92	0.855689	0.144311	1.42578118	1.426652	339.2933637	237.8218239
PLEKHM1	0.855758	0.144242	1.380019374	1.380223	1283.299458	929.7742807
PTPRE	0.855784	0.144216	0.717705133	0.71749	458.3681102	638.853344

KIAA1456	0.855797	0.144203	0.640019958	0.638567	78.74037206	123.3135524
PCDHAC2	0.856144	0.143856	1.423441387	1.424283	348.9150555	244.9730195
PIGK	0.856265	0.143735	1.385028856	1.385327	871.303258	628.948641
SLIT1	0.856344	0.143656	0.328501511	0.308565	5.048106941	16.38237877
MYLIP	0.856591	0.143409	1.388111884	1.388462	748.1294486	538.8158436
LIAS	0.856594	0.143406	1.398177426	1.398673	547.2147924	391.2357688
PTK6	0.856626	0.143374	0.438055867	0.428401	12.12555287	28.3175459
CAPS2	0.856901	0.143099	1.572742473	1.576494	117.116081	74.28527256
GABRE	0.856909	0.143091	0.688267201	0.687659	171.635636	249.5985158
CDYL2	0.85754	0.14246	0.648601402	0.647341	87.83706077	135.6944312
STAC2	0.857833	0.142167	0.178467762	0.128197	1.009621388	7.943571812
POTEM	0.858057	0.141943	0.607795687	0.605636	53.50993357	88.35985553
IMPA2	0.858474	0.141526	1.391815204	1.391865	5348.974114	3843.024767
PSG9	0.858956	0.141044	1.543445319	1.546514	133.2700232	86.17091617
ARMC12	0.859161	0.140839	0.178887613	0.128517	1.009621388	7.923762406
C5orf49	0.859161	0.140839	0.178887613	0.128517	1.009621388	7.923762406
GPC3	0.859161	0.140839	0.178887613	0.128517	1.009621388	7.923762406
GUCY1A3	0.859161	0.140839	0.178887613	0.128517	1.009621388	7.923762406
HOXC6	0.859161	0.140839	0.178887613	0.128517	1.009621388	7.923762406
LAYN	0.859161	0.140839	0.178887613	0.128517	1.009621388	7.923762406
LOC10050	0.859161	0.140839	0.178887613	0.128517	1.009621388	7.923762406
MEF2C	0.859161	0.140839	0.178887613	0.128517	1.009621388	7.923762406
MTNR1A	0.859161	0.140839	0.178887613	0.128517	1.009621388	7.923762406
PTPN5	0.859161	0.140839	0.178887613	0.128517	1.009621388	7.923762406
SEMA3D	0.859161	0.140839	0.178887613	0.128517	1.009621388	7.923762406
SNORA57	0.859161	0.140839	0.178887613	0.128517	1.009621388	7.923762406
KIF6	0.859268	0.140732	1.537334982	1.540268	137.3085088	89.14232707
STPG1	0.859548	0.140452	1.383540376	1.383859	827.8895383	598.2440617
B3GNTL1	0.859574	0.140426	1.43946689	1.440569	279.6651245	194.132179
TPX2	0.860198	0.139802	1.391770291	1.391817	5628.639239	4044.090238
SCN1B	0.860476	0.139524	0.647910952	0.64662	85.81781799	132.7230203
WDR77	0.860689	0.139311	1.380907405	1.381166	990.4385818	717.1004978
DPF1	0.860736	0.139264	1.929761102	1.949616	44.42334108	22.78081692
CRELD2	0.860857	0.139143	0.64350865	0.642129	80.76971105	125.7897282
BCAS4	0.860985	0.139015	1.404076048	1.404681	456.3488674	324.8742587
DNAH17-A	0.861262	0.138738	0.094769991	0.002103	0	4.744352741
KLHL7-AS	0.861416	0.138584	0.534720877	0.530149	26.25015609	49.52351504
MCPH1-A	0.861423	0.138577	1.929321621	1.949616	44.42334108	22.78081692
UBL7-AS1	0.861848	0.138152	1.546877513	1.55007	129.2315377	83.36788522
MYBBP1A	0.862042	0.137958	0.723173802	0.723006	593.6573762	821.0998793
APOL4	0.86246	0.13754	0.472751579	0.465129	15.64913152	33.65618082
ITGA1	0.862567	0.137433	1.388256643	1.388652	664.3308734	478.3971553
EMP3	0.862943	0.137057	0.695704304	0.695176	194.8569279	280.3030951
CCDC169	0.863228	0.136772	1.674704232	1.681736	78.52835157	46.69076998
KLHL31	0.863229	0.136771	0.372501055	0.357092	7.067349717	19.80940602
FAM69A	0.863387	0.136613	0.68858914	0.687959	165.5779077	240.6842831
HDAC10	0.863387	0.136613	0.68858914	0.687959	165.5779077	240.6842831
MAGEA11	0.863519	0.136481	0.179959651	0.128517	1.009621388	7.923762406
KPNA2	0.863665	0.136335	1.393869079	1.393908	6825.494914	4896.657359
ARID3B	0.863666	0.136334	1.435209491	1.436314	281.6843673	196.1131196
ZNF114	0.864251	0.135749	1.431653631	1.432708	291.8613509	203.7100268
LINC0026	0.864427	0.135573	10.39099584	467.4451	4.664450813	0
ABCA9	0.864559	0.135441	0.180944535	0.129627	1.019717602	7.933667109
DMXL2	0.864711	0.135289	0.722596716	0.722419	550.2436565	761.6716613
GJB4	0.864721	0.135279	2.012068348	2.038119	38.36561275	18.81893571
LOC10192	0.864869	0.135131	2.576908208	2.673643	21.20204915	7.923762406

PIF1	0.86487	0.13513	1.698467897	1.706628	72.69273995	42.59022293
RAD51L3-	0.865192	0.134808	0.637760503	0.636196	73.31870521	115.2511242
ITPKC	0.86522	0.13478	1.385281628	1.385651	702.6964862	507.120794
CLIC2	0.865224	0.134776	0.372841042	0.357092	7.067349717	19.80940602
HTRA1	0.865553	0.134447	0.726752406	0.726682	1406.402594	1935.378968
POLR2M	0.865803	0.134197	0.727016969	0.72694	1274.929697	1753.835666
CNTRL	0.865893	0.134107	1.411187595	1.411953	377.5983992	267.4269812
VPS53	0.866656	0.133344	0.71512789	0.714841	345.2602261	482.9929375
REREP3	0.867613	0.132387	4.198197677	4.950086	10.23756088	2.060178226
CBX2	0.867632	0.132368	0.686266821	0.685585	153.462451	223.846288
WDR12	0.867712	0.132288	1.376059779	1.376282	1132.795198	823.0808199
IQGAP3	0.868956	0.131044	1.374935196	1.375064	1943.521172	1413.401119
SLC25A46	0.869118	0.130882	0.72761668	0.727505	883.4187146	1214.316589
MPHOSPH	0.869842	0.130158	1.375181713	1.375408	1107.554663	805.2523545
CERS1	0.870051	0.129949	0.721625657	0.721413	459.7310991	637.2685915
SPANXD	0.870342	0.129658	0.097217329	0.002117	0	4.714638632
COQ3	0.87041	0.12959	1.433305458	1.434403	275.626639	192.1512384
FAM110B	0.870453	0.129547	0.601358366	0.59891	47.45220524	79.23762406
ARPP19	0.870787	0.129213	0.721003169	0.720978	3879.056239	5380.274293
SPINT2	0.87093	0.12907	1.40548553	1.405505	13937.82326	9916.588651
KIF12	0.871132	0.128868	0.718561794	0.718303	380.6272633	529.9016109
ANKS1B	0.871787	0.128213	0.4884782	0.481498	17.1635636	35.65693083
RAPGEF4	0.871787	0.128213	0.4884782	0.481498	17.1635636	35.65693083
NTHL1	0.871999	0.128001	1.387915288	1.388395	558.3206277	402.1309421
GRB10	0.872494	0.127506	0.726880498	0.726741	694.6195151	955.8038402
SDHB	0.872576	0.127424	1.374618917	1.374725	2370.591019	1724.408794
CRMP1	0.872585	0.127415	0.357673412	0.340149	6.057728329	17.82846541
GSC	0.872585	0.127415	0.357673412	0.340149	6.057728329	17.82846541
ISM2	0.872585	0.127415	0.357673412	0.340149	6.057728329	17.82846541
BOC	0.873006	0.126994	0.634064583	0.6324	67.64463301	106.9707925
KCNG1	0.873064	0.126936	0.711588216	0.711231	279.6651245	393.2167094
HDAC9	0.873387	0.126613	1.391997534	1.392524	504.8106941	362.5121301
RCAN1	0.873396	0.126604	0.728962993	0.728861	965.1980471	1324.258792
TRHDE	0.873474	0.126526	1.379888475	1.38026	702.6964862	509.1017346
PGF	0.873627	0.126373	0.502989965	0.49673	19.18280638	38.62834173
MLLT3	0.873691	0.126309	1.375022874	1.375313	885.4379574	643.8056955
PPM1E	0.87391	0.12609	0.7193449	0.719086	379.6176419	527.9206703
RND2	0.874031	0.125969	0.503248015	0.496991	19.19290259	38.62834173
RHBDD2	0.874118	0.125882	0.722901775	0.722691	462.4065958	639.8438143
CEBPA	0.874335	0.125665	0.686544471	0.685835	147.4047227	214.9320553
TFEB	0.8744	0.1256	0.713643337	0.713295	291.7805812	409.0642342
PARTICL	0.874423	0.125577	1.715623431	1.724841	66.63501162	38.62834173
MORC4	0.874953	0.125047	0.729444865	0.729348	1010.63101	1385.667951
CHN1	0.875119	0.124881	0.665294939	0.664234	101.9717602	153.5228966
RALY-AS1	0.875626	0.124374	1.81339661	1.827486	52.50031218	28.72363872
HIST1H4J	0.875747	0.124253	10.08266679	450.2815	4.492815177	0
EPPK1	0.875899	0.124101	0.44676628	0.437061	12.11545666	27.73316842
KNOP1	0.876021	0.123979	1.370821147	1.370984	1515.441704	1105.364856
TNNT1	0.876112	0.123888	0.727762992	0.727609	642.1192029	882.509038
SIPA1L1	0.876147	0.123853	0.72969096	0.729612	1250.9209	1714.504091
AP4E1	0.876253	0.123747	0.719243835	0.718975	364.7964	507.388221
TNFSF12	0.876381	0.123619	0.611306916	0.609009	51.26857409	84.18997557
PKDCC	0.876568	0.123432	0.686642198	0.685923	145.3854799	211.9606444
TPGS2	0.87695	0.12305	1.373193825	1.373292	2542.226655	1851.188992
RBM8A	0.877021	0.122979	0.724915088	0.724882	2932.950133	4046.110797
LANCL2	0.87729	0.12271	0.659081404	0.657893	91.87554632	139.6563124

LINC0068(	0.877583	0.122417	2.434796127	2.512016	22.70638502	9.033089143
ECT2	0.87801	0.12199	1.381769196	1.38182	5142.617599	3721.622822
SCLT1	0.878024	0.121976	1.38522754	1.385688	561.3494918	405.102353
MGST3	0.878523	0.121477	1.37066109	1.370781	2055.589146	1499.572035
LINC0109	0.878577	0.121423	2.241618055	2.292417	27.25977748	11.88564361
LOC65316	0.878581	0.121419	0.591515397	0.588689	41.39447691	70.32339135
EFCAB11	0.878609	0.121391	1.403370351	1.404096	379.6277382	270.368678
ZNF83	0.878705	0.121295	1.392186706	1.392217	9026.923869	6483.846397
RGS3	0.879205	0.120795	0.729555751	0.729492	1509.383975	2069.092458
PPP6R2	0.879648	0.120352	0.729313153	0.72917	687.5521653	942.9277263
SNX30	0.879982	0.120018	1.368785008	1.368954	1450.825935	1059.803222
AP1S2	0.880014	0.119986	0.73040431	0.730336	1432.824385	1961.874048
HSD17B3	0.880142	0.119858	0.33925151	0.318972	5.048106941	15.84752481
LOC10012	0.880242	0.119758	0.712868378	0.712499	269.5991993	378.389369
PHF11	0.880254	0.119746	1.396708263	1.397354	417.9832547	299.1220308
THAP7-AS	0.880426	0.119574	0.60375049	0.601198	46.44258386	77.25668346
GULP1	0.880458	0.119542	1.378018092	1.378077	4401.949252	3194.26672
MRPL37	0.880805	0.119195	1.371660396	1.371755	2629.054095	1916.560032
PSD4	0.880846	0.119154	1.412873613	1.413756	322.0692228	227.8081692
DMC1	0.881113	0.118887	0.34816833	0.328614	5.441859282	16.58047283
LRAT	0.881131	0.118869	0.468935779	0.460521	14.13469943	30.70457932
SFTA1P	0.881134	0.118866	0.636438582	0.634715	66.63501162	104.9898519
FLOT1	0.881458	0.118542	1.379291215	1.379346	4657.383464	3376.513255
MITD1	0.881591	0.118409	1.369572264	1.369811	1033.852301	754.7383692
LARP6	0.881697	0.118303	0.730868892	0.730785	1137.843304	1557.019313
TMC6	0.881772	0.118228	0.706434441	0.705957	216.0589771	306.0553229
SLC46A1	0.881782	0.118218	0.725015966	0.724792	442.214168	610.1297053
CGB8	0.881875	0.118125	0.227837636	0.187301	1.726452574	9.260897312
LOC10272	0.882019	0.117981	0.241884502	0.20467	2.019242776	9.904703008
MXRA8	0.882019	0.117981	0.241884502	0.20467	2.019242776	9.904703008
STRA6	0.882019	0.117981	0.241884502	0.20467	2.019242776	9.904703008
ZNF843	0.882019	0.117981	0.241884502	0.20467	2.019242776	9.904703008
DOC2A	0.882277	0.117723	0.339696233	0.318972	5.048106941	15.84752481
PSORS1C	0.882277	0.117723	0.339696233	0.318972	5.048106941	15.84752481
PSG1	0.882409	0.117591	0.540836507	0.536131	25.4525552	47.48314622
NSUN3	0.882424	0.117576	1.459851395	1.461513	196.8761707	134.7039609
TIMM8A	0.882444	0.117556	1.419099126	1.420121	289.7613384	204.036882
ATP13A1	0.882688	0.117312	0.731150587	0.731066	1136.833683	1555.038372
PKIA	0.88273	0.11727	0.700402775	0.699853	185.7703354	265.4460406
HERC2P2	0.883117	0.116883	0.730138822	0.730082	1690.742265	2315.828515
FAM198B	0.883634	0.116366	0.71970269	0.719409	334.1846795	464.5305711
AGAP2-AS	0.883746	0.116254	0.701341416	0.700804	188.7991996	269.4079218
ABCA11P	0.883831	0.116169	1.397781127	1.398471	392.2076207	280.4516657
URGCP-M	0.88387	0.11613	2.73422361	2.87365	17.77943265	6.180534677
OR7E156I	0.883997	0.116003	4.0361928	4.742485	10.13659874	2.129511147
FXR2	0.884255	0.115745	0.729588143	0.729436	630.0037462	863.6901023
HIVEP3	0.884924	0.115076	0.702518427	0.701971	189.808821	270.3983921
ORAI3	0.884936	0.115064	0.717903819	0.717581	304.9056592	424.911759
LRRC1	0.884943	0.115057	1.368398666	1.368653	965.1980471	705.2148542
C14orf37	0.884978	0.115022	0.682226061	0.681391	126.2026735	185.2179462
MT1F	0.885066	0.114934	0.242685987	0.20467	2.019242776	9.904703008
ESCO2	0.885355	0.114645	1.369114505	1.369389	896.5437927	654.7008688
CASC18	0.885355	0.114645	0.315748219	0.291749	4.038485553	13.86658421
DLGAP3	0.885355	0.114645	0.315748219	0.291749	4.038485553	13.86658421
NFIB	0.885482	0.114518	0.731473257	0.73136	841.0146163	1149.936019
KAT2A	0.885684	0.114316	0.73184651	0.731781	1490.201169	2036.406938

ITGA9	0.885831	0.114169	0.502633834	0.49603	18.17318499	36.64740113
PRKD1	0.885842	0.114158	0.691231038	0.690532	148.4143441	214.9320553
SUGT1P1	0.886533	0.113467	1.885968869	1.905317	43.41371969	22.78081692
SLC17A7	0.886634	0.113366	0.502781191	0.49603	18.17318499	36.64740113
TSHZ3	0.886634	0.113366	0.502781191	0.49603	18.17318499	36.64740113
OASL	0.887025	0.112975	0.284724762	0.25546	3.028864164	11.88564361
E2F8	0.887031	0.112969	1.405547596	1.406407	330.1461939	234.7414613
KIAA1551	0.88709	0.11291	1.434096029	1.434101	65359.85981	45575.50042
TTLL12	0.887114	0.112886	1.368377584	1.368472	2607.852046	1905.664859
PLAUR	0.887183	0.112817	1.369582002	1.369885	812.7452175	593.2917102
SLC35F1	0.887244	0.112756	0.187988045	0.134717	0.98942896	7.40871785
BMP8B	0.887385	0.112615	0.726921271	0.726699	443.960813	610.9319862
DGCR14	0.887391	0.112609	0.708432952	0.707972	218.0782198	308.0362635
LINC0017	0.887633	0.112367	0.31627223	0.291749	4.038485553	13.86658421
WIPI1	0.887851	0.112149	0.720369841	0.720067	323.0788442	448.6830463
NBPF20	0.888003	0.111997	0.723936627	0.723681	379.8498549	524.8898312
TRA2B	0.888302	0.111698	0.728244812	0.72821	2790.593517	3832.129594
CHRNA7	0.88832	0.11168	0.10189487	0.002279	0	4.377878729
SLC9A7	0.888445	0.111555	0.732247343	0.73213	808.7168281	1104.612098
TBC1D28	0.888545	0.111455	9.71011266	443.2142	4.42214168	0
AIMP2	0.888593	0.111407	1.366872258	1.367139	916.7362205	670.5483936
APOL1	0.888675	0.111325	0.724630473	0.724607	4199.853339	5796.044011
LOC10192	0.888695	0.111305	0.451494233	0.441607	11.91353238	26.9903157
NUDT16L	0.888835	0.111165	0.696251681	0.695611	160.5298007	230.7795801
APBB1IP	0.88898	0.11102	0.442253173	0.431478	11.10583527	25.75222782
CCDC134	0.889164	0.110836	1.480479167	1.482625	161.5394221	108.9517331
CASP4	0.889188	0.110812	0.727855435	0.727638	453.3200033	623.0058192
CCNA1	0.889316	0.110684	1.370177994	1.370253	3311.558153	2416.747534
FAM231C	0.889422	0.110578	0.399996003	0.385543	7.955816539	20.65130577
SYN1	0.88956	0.11044	0.285360086	0.25546	3.028864164	11.88564361
SYT5	0.88956	0.11044	0.285360086	0.25546	3.028864164	11.88564361
TBC1D13	0.889959	0.110041	1.363643343	1.363876	1059.092836	776.5287158
TXNDC16	0.890053	0.109947	1.392877983	1.393553	394.7619628	283.274506
LTB4R	0.890299	0.109701	0.625062579	0.622969	55.52917635	89.14232707
NDUFC2-I	0.890568	0.109432	1.385546833	1.386122	452.1589387	326.2014889
LINC0095	0.890616	0.109384	1.364909457	1.365183	906.0544262	663.6844344
PITRM1-A	0.891104	0.108896	0.402892951	0.388612	8.076971105	20.79987632
CEP70	0.891239	0.108761	1.364500455	1.364748	977.3135037	716.1100275
MALL	0.891559	0.108441	1.363149217	1.363364	1120.407143	821.7932086
PSG3	0.892169	0.107831	0.289616995	0.260553	3.089441448	11.88564361
SLC12A9	0.892288	0.107712	0.666285113	0.665122	92.88516771	139.6563124
ZNF484	0.892427	0.107573	1.403452044	1.404303	324.0884656	230.7795801
PADI1	0.892761	0.107239	1.368689549	1.368762	3389.299	2476.175752
LOC1005C	0.893117	0.106883	2.008909477	2.038054	34.3271272	16.83799511
DDO	0.893246	0.106754	0.614020685	0.61165	48.46182663	79.23762406
TAF1A	0.893397	0.106603	1.406475164	1.407412	303.8960378	215.9225256
GPM6A	0.893543	0.106457	0.585774529	0.582544	36.34636997	62.39962895
PCDH10	0.893876	0.106124	0.466375463	0.457132	13.12507805	28.72363872
PC	0.894023	0.105977	0.729440028	0.729233	464.4258386	636.8724034
RGL1	0.894504	0.105496	0.671540788	0.670458	98.94289604	147.5800748
ENC1	0.89477	0.10523	1.385117968	1.38575	420.0024975	303.083912
HLA-DRB	0.894801	0.105199	0.104008226	0.002332	0	4.278831699
ATP8B3	0.894818	0.105182	0.732655311	0.732497	600.724726	820.109409
ZYG11A	0.895389	0.104611	1.371833221	1.372275	574.3736077	418.5529397
SNHG19	0.895571	0.104429	1.388322994	1.38901	390.7234772	281.2935654
TMEM175	0.895609	0.104391	0.629370978	0.627325	56.53879774	90.13279737

ZNF582-A	0.895645	0.104355	1.401244375	1.402125	318.0307373	226.8176989
NAAA	0.895699	0.104301	0.485870383	0.477978	15.14432082	31.69504962
SNHG1	0.895754	0.104246	1.372377004	1.372428	4960.26988	3614.226128
CABLES1	0.895766	0.104234	1.363039761	1.363316	872.3128794	639.8438143
TRAK2	0.896116	0.103884	0.73472979	0.734662	1404.383351	1911.60768
PWARSN	0.896161	0.103839	0.458217026	0.448473	12.12555287	27.04974391
TPBG	0.896375	0.103625	0.735028087	0.734896	732.9851278	997.4035929
POU6F1	0.896387	0.103613	0.719448148	0.719092	275.626639	383.3021017
PCDHGB2	0.896452	0.103548	0.660982835	0.659649	83.40482288	126.4434386
LINC0057	0.896549	0.103451	0.527364911	0.521707	21.20204915	40.64890114
NGFR	0.896604	0.103396	0.486046131	0.477978	15.14432082	31.69504962
GLRB	0.896661	0.103339	0.726327955	0.726051	357.4059714	492.2637395
GGT7	0.896824	0.103176	0.715491469	0.715065	237.2610262	331.8075508
NCOA5	0.897181	0.102819	0.73239183	0.732202	514.906908	703.2339135
ABHD4	0.897281	0.102719	0.732821738	0.732651	557.3110063	760.681191
PRIMA1	0.897614	0.102386	0.51605414	0.509798	19.18280638	37.63787143
UBE2Q2P	0.89776	0.10224	1.618194813	1.624317	79.82066695	49.13723162
SULT4A1	0.897983	0.102017	0.527999597	0.522216	21.20204915	40.60928233
ERICH5	0.898229	0.101771	0.436450007	0.424965	10.09621388	23.77128722
LRRK2	0.898261	0.101739	1.424593792	1.42583	237.2610262	166.3990105
COG7	0.898279	0.101721	1.364425565	1.364757	729.9764561	534.8737718
AQP7P3	0.898421	0.101579	1.471254535	1.473359	160.5298007	108.9517331
LOC10050	0.898582	0.101418	0.436497282	0.424965	10.09621388	23.77128722
CSTA	0.898651	0.101349	0.391538101	0.375876	7.067349717	18.81893571
CMPK1	0.899	0.101	0.727087985	0.727066	4372.670232	6014.135666
LCP1	0.899095	0.100905	0.391602231	0.375876	7.067349717	18.81893571
LOC10192	0.899095	0.100905	0.391602231	0.375876	7.067349717	18.81893571
TMEFF1	0.899484	0.100516	0.715378321	0.714936	228.6994368	319.892193
FAM126A	0.89957	0.10043	0.729845708	0.729616	416.9736333	571.5013635
RAP2B	0.899675	0.100325	1.358082433	1.358294	1120.679741	825.0617605
PRPF40B	0.899798	0.100202	1.357502915	1.357687	1284.238406	945.8991372
DCAF12L2	0.899818	0.100182	0.436752774	0.424965	10.09621388	23.77128722
TMEM221	0.899818	0.100182	0.436752774	0.424965	10.09621388	23.77128722
BLOC1S2	0.899827	0.100173	0.725327673	0.725036	331.1255267	456.7058557
ACSM3	0.89991	0.10009	1.519182845	1.522485	119.1353238	78.24715376
MSRB1	0.900038	0.099962	0.716950264	0.716541	241.2995118	336.7599023
CENPO	0.900164	0.099836	1.358035877	1.358257	1072.217914	789.4048297
PPIEL	0.900281	0.099719	0.618696929	0.616358	48.89596383	79.33667109
PRMT7	0.900316	0.099684	1.364921893	1.365284	669.3789804	490.2827989
PTGR2	0.900542	0.099458	1.357297379	1.357441	1641.644377	1209.364237
MEIOB	0.900555	0.099445	1.464369546	1.466371	165.5779077	112.9136143
BCL2L11	0.900602	0.099398	0.731372542	0.731159	448.2718963	613.1011162
NEIL1	0.900734	0.099266	0.633542211	0.631585	57.54841913	91.12326767
GCH1	0.900745	0.099255	0.708824989	0.708299	190.8184424	269.4079218
ZBTB46	0.901	0.099	0.722687184	0.722351	289.7613384	401.1404718
NLRP1	0.901089	0.098911	0.716716307	0.716298	236.2514048	329.8266102
PCSK5	0.901093	0.098907	1.359209418	1.359307	2478.6407	1823.455824
SPATA12	0.901115	0.098885	2.866642753	3.053859	15.14432082	4.952351504
CCDC77	0.901126	0.098874	1.424512746	1.425792	230.1936765	161.446659
CROCC	0.901151	0.098849	0.7343068	0.734137	560.7033341	763.7615536
FZD1	0.901174	0.098826	0.727349349	0.727076	352.3578645	484.6272135
OPN3	0.90123	0.09877	0.703024882	0.702412	165.5779077	235.7319316
MCTP1	0.901372	0.098628	1.356515581	1.356673	1498.27814	1104.374385
TMEM94	0.901699	0.098301	0.736940857	0.736829	840.0049949	1140.031316
PRH1-TAS	0.902104	0.097896	2.300325557	2.366392	23.17081086	9.785846572
C7orf26	0.902224	0.097776	0.7143334	0.713876	217.0685985	304.0743823

DBNDD2	0.902228	0.097772	0.688897343	0.688062	124.1329497	180.4141653
ATP6AP1	0.902388	0.097612	0.737622239	0.737545	1249.911279	1694.694685
TMEM68	0.902488	0.097512	1.356103988	1.356344	1003.56366	739.9011241
APBB1	0.902497	0.097503	0.73130311	0.731078	425.0506044	581.4060666
TMEM64	0.902517	0.097483	1.354859388	1.355024	1450.825935	1070.698395
KLHL6-AS	0.902806	0.097194	0.333183883	0.309471	4.321179541	13.98544065
CPS1	0.902988	0.097012	0.727467492	0.727189	344.2808934	473.4448038
LINC0108	0.90321	0.09679	1.818387658	1.834467	45.43296247	24.76175752
ZCWPW2	0.90321	0.09679	1.818387658	1.834467	45.43296247	24.76175752
NKX3-2	0.903299	0.096701	0.462971321	0.453242	12.11545666	26.74269812
CNGB1	0.903385	0.096615	1.955529395	1.98149	35.33674859	17.82846541
C19orf71	0.903457	0.096543	5.086219403	7.074023	7.067349717	0.990470301
LOC10192	0.903457	0.096543	5.086219403	7.074023	7.067349717	0.990470301
HID1	0.903909	0.096091	0.732113295	0.73188	418.9928761	572.4918338
DGUOK	0.903933	0.096067	1.355247425	1.355456	1123.708605	829.0236417
SGSM1	0.90413	0.09587	0.461395477	0.451301	11.97410966	26.54460406
TBC1D19	0.904421	0.095579	1.373843675	1.374408	453.3200033	329.8266102
FAM210A	0.904555	0.095445	1.354500726	1.35463	1843.568655	1360.935907
PDZD7	0.904693	0.095307	1.637570127	1.644689	71.68311856	43.58069323
MEGF8	0.905113	0.094887	0.7367994	0.736744	1708.279389	2318.690974
LOC10192	0.905335	0.094665	1.718618936	1.729558	56.53879774	32.68551993
IL13RA2	0.905773	0.094227	9.16419487	404.8486	4.038485553	0
BLOC1S6	0.905851	0.094149	0.73863127	0.738544	1077.286214	1458.665612
ACTRT3	0.906019	0.093981	0.675990863	0.674893	96.92365326	143.6181936
GPA33	0.906089	0.093911	2.160271596	2.207544	26.25015609	11.88564361
MNX1	0.906212	0.093788	0.630350495	0.628239	53.50993357	85.18044587
ZBED8	0.906237	0.093763	1.421892324	1.423196	224.1359482	157.4847778
ZNRF2P2	0.906343	0.093657	0.254297077	0.214979	2.019242776	9.429277263
ZNF80	0.906395	0.093605	1.541899677	1.545988	101.9717602	65.95541733
PROK1	0.906449	0.093551	2.073044459	2.110679	29.27902026	13.86658421
ADRA1B	0.906633	0.093367	1.770045539	1.783554	49.47144802	27.73316842
BAALC	0.906727	0.093273	0.539436611	0.53405	22.21167054	41.59975263
MXRA5	0.906766	0.093234	0.738842123	0.738743	940.9671338	1273.744807
VAC14	0.906877	0.093123	1.361984879	1.362048	3904.205908	2866.42105
HCG4	0.90691	0.09309	1.407749748	1.408865	256.4438326	182.0187272
COL14A1	0.907091	0.092909	3.055185015	3.307017	13.12507805	3.961881203
MGAT3	0.90713	0.09287	0.738954794	0.738843	843.0338591	1141.021786
ANKRD36	0.907204	0.092796	9.130732325	404.8486	4.038485553	0
ASPN	0.907204	0.092796	9.130732325	404.8486	4.038485553	0
CALCB	0.907204	0.092796	9.130732325	404.8486	4.038485553	0
CIB3	0.907204	0.092796	9.130732325	404.8486	4.038485553	0
IL37	0.907204	0.092796	9.130732325	404.8486	4.038485553	0
LINC0086	0.907204	0.092796	9.130732325	404.8486	4.038485553	0
LOC10192	0.907204	0.092796	9.130732325	404.8486	4.038485553	0
LOC10496	0.907204	0.092796	9.130732325	404.8486	4.038485553	0
OVOL1	0.907204	0.092796	9.130732325	404.8486	4.038485553	0
PCDH12	0.907204	0.092796	9.130732325	404.8486	4.038485553	0
SCARNA1	0.907204	0.092796	9.130732325	404.8486	4.038485553	0
UFSP1	0.907454	0.092546	0.378110173	0.360145	6.057728329	16.83799511
C10orf11	0.907574	0.092426	0.528794961	0.52286	20.19242776	38.62834173
CDCA2	0.907629	0.092371	1.352362137	1.352522	1450.825935	1072.679336
KCTD17	0.907896	0.092104	0.732128219	0.731884	390.7234772	533.8634921
AZIN2	0.908007	0.091993	0.711791935	0.711264	188.7991996	265.4460406
C6orf136	0.908071	0.091929	1.374999314	1.375596	421.0121189	306.0553229
PSD2	0.908165	0.091835	0.5289275	0.52286	20.19242776	38.62834173
DTYMK	0.908182	0.091818	1.356406328	1.356715	763.2737694	562.5871308

TGIF2	0.908641	0.091359	0.737362994	0.737184	539.8041714	732.2546934
LOC10050	0.908826	0.091174	1.64267899	1.650196	68.65425439	41.59975263
ALX1	0.908873	0.091127	1.642510976	1.650196	68.65425439	41.59975263
GABPB1-A	0.908913	0.091087	0.614959462	0.612378	44.44353351	72.58166364
SCN8A	0.909055	0.090945	0.678334488	0.677252	97.93327465	144.6086639
DHRS4L2	0.909103	0.090897	0.692249096	0.691386	122.7194797	177.5021826
STK36	0.909301	0.090699	0.736796951	0.736604	499.7625871	678.472156
RPL21P28	0.909435	0.090565	1.376339584	1.376369	8614.382474	6258.771926
ZNF865	0.909462	0.090538	0.72118117	0.720776	241.2692231	334.7393428
LRRC46	0.909677	0.090323	1.872222973	1.892622	39.37523414	20.79987632
TIMM10	0.91034	0.08966	1.35731273	1.357688	644.1384456	474.4352741
UGT1A6	0.910447	0.089553	1.363630291	1.363681	4875.532357	3575.270931
AURKB	0.910488	0.089512	1.356173595	1.356508	702.6964862	518.0159673
NPPA-AS1	0.910531	0.089469	0.353838318	0.332443	4.876471305	14.68867456
KCTD15	0.910531	0.089469	0.740378124	0.740274	894.5245499	1208.373767
MYBL1	0.910672	0.089328	1.349561428	1.349713	1546.739967	1145.974138
GINS4	0.910977	0.089023	1.3499779	1.350159	1269.094085	939.9563154
RRN3P3	0.911014	0.088986	1.822245995	1.839701	43.12092949	23.43452732
LCAT	0.911294	0.088706	0.644411013	0.642581	60.75901514	94.56019961
ECHDC3	0.911344	0.088656	0.724927399	0.724562	265.5304251	366.4740113
ENTPD8	0.911447	0.088553	0.566115019	0.561765	27.25977748	48.53304474
TUBA1C	0.911617	0.088383	1.392461273	1.392475	19435.09057	13957.22215
NT5M	0.911742	0.088258	0.660017455	0.658539	73.70236134	111.923144
PDE3B	0.911808	0.088192	0.703840198	0.703166	150.4335868	213.941585
DNAJC17	0.911826	0.088174	1.387939246	1.388772	315.0018731	226.8176989
HSPA6	0.911958	0.088042	0.202516103	0.14685	1.009621388	6.933292105
NEGR1	0.911958	0.088042	0.202516103	0.14685	1.009621388	6.933292105
CALB2	0.911979	0.088021	8.954440786	404.8486	4.038485553	0
LOC10013	0.911979	0.088021	8.954440786	404.8486	4.038485553	0
MMP23B	0.911979	0.088021	8.954440786	404.8486	4.038485553	0
TMEM88	0.911979	0.088021	8.954440786	404.8486	4.038485553	0
GIMAP8	0.912026	0.087974	0.110996025	0.002518	0	3.961881203
PI15	0.912026	0.087974	0.110996025	0.002518	0	3.961881203
RHOJ	0.912026	0.087974	0.110996025	0.002518	0	3.961881203
SLC43A1	0.912304	0.087696	0.711268831	0.710704	176.6837429	248.6080455
SMG7	0.912727	0.087273	1.349756902	1.349873	2016.213912	1493.629214
PLA2G6	0.9128	0.0872	0.712909906	0.712365	182.7414713	256.5318079
EIF1B-AS1	0.912802	0.087198	0.202734612	0.14685	1.009621388	6.933292105
FAM132A	0.912802	0.087198	0.202734612	0.14685	1.009621388	6.933292105
FST	0.912802	0.087198	0.202734612	0.14685	1.009621388	6.933292105
GRIK1-AS	0.912802	0.087198	0.202734612	0.14685	1.009621388	6.933292105
HIC1	0.912802	0.087198	0.202734612	0.14685	1.009621388	6.933292105
LRRC26	0.912802	0.087198	0.202734612	0.14685	1.009621388	6.933292105
TEX40	0.912802	0.087198	0.202734612	0.14685	1.009621388	6.933292105
GSTM1	0.912854	0.087146	8.927464017	394.7523	3.937523414	0
RALGAPA	0.913181	0.086819	0.740801718	0.740671	713.8023214	963.7276027
CATSPER	0.913226	0.086774	3.867917663	4.568992	9.086592493	1.980940602
HIST1H4E	0.913226	0.086774	3.867917663	4.568992	9.086592493	1.980940602
LOC10272	0.913226	0.086774	3.867917663	4.568992	9.086592493	1.980940602
TRIM31-A	0.913226	0.086774	3.867917663	4.568992	9.086592493	1.980940602
UBE2Q2P	0.913236	0.086764	1.720212032	1.731555	53.49983736	30.89276868
ABCB1	0.913371	0.086629	0.111401946	0.002518	0	3.961881203
ATP2C2	0.913371	0.086629	0.111401946	0.002518	0	3.961881203
C12orf42	0.913371	0.086629	0.111401946	0.002518	0	3.961881203
CCKBR	0.913371	0.086629	0.111401946	0.002518	0	3.961881203
CLDN14	0.913371	0.086629	0.111401946	0.002518	0	3.961881203

CPB2	0.913371	0.086629	0.111401946	0.002518	0	3.961881203
DUSP26	0.913371	0.086629	0.111401946	0.002518	0	3.961881203
FAM43B	0.913371	0.086629	0.111401946	0.002518	0	3.961881203
FES	0.913371	0.086629	0.111401946	0.002518	0	3.961881203
FGD5	0.913371	0.086629	0.111401946	0.002518	0	3.961881203
FGF13	0.913371	0.086629	0.111401946	0.002518	0	3.961881203
FLRT2	0.913371	0.086629	0.111401946	0.002518	0	3.961881203
FOXL2NB	0.913371	0.086629	0.111401946	0.002518	0	3.961881203
FRY	0.913371	0.086629	0.111401946	0.002518	0	3.961881203
GRIA2	0.913371	0.086629	0.111401946	0.002518	0	3.961881203
GSTT2	0.913371	0.086629	0.111401946	0.002518	0	3.961881203
IFNB1	0.913371	0.086629	0.111401946	0.002518	0	3.961881203
ILDR1	0.913371	0.086629	0.111401946	0.002518	0	3.961881203
ILDR2	0.913371	0.086629	0.111401946	0.002518	0	3.961881203
KLHDC8A	0.913371	0.086629	0.111401946	0.002518	0	3.961881203
KRT75	0.913371	0.086629	0.111401946	0.002518	0	3.961881203
LINC0035	0.913371	0.086629	0.111401946	0.002518	0	3.961881203
LOC10192	0.913371	0.086629	0.111401946	0.002518	0	3.961881203
LOC10192	0.913371	0.086629	0.111401946	0.002518	0	3.961881203
LOC10272	0.913371	0.086629	0.111401946	0.002518	0	3.961881203
MARCO	0.913371	0.086629	0.111401946	0.002518	0	3.961881203
METTL24	0.913371	0.086629	0.111401946	0.002518	0	3.961881203
OCM	0.913371	0.086629	0.111401946	0.002518	0	3.961881203
OR10H1	0.913371	0.086629	0.111401946	0.002518	0	3.961881203
PIK3R5	0.913371	0.086629	0.111401946	0.002518	0	3.961881203
PTGER4P	0.913371	0.086629	0.111401946	0.002518	0	3.961881203
RTN4RL1	0.913371	0.086629	0.111401946	0.002518	0	3.961881203
UPK1B	0.913371	0.086629	0.111401946	0.002518	0	3.961881203
MYL6B	0.913497	0.086503	0.739918643	0.739764	603.7535901	816.1475278
FLJ10038	0.913687	0.086313	0.666128123	0.664757	78.75046828	118.4701527
NINJ1	0.913732	0.086268	0.741668306	0.741564	897.5534141	1210.354708
KRT83	0.914052	0.085948	0.204905997	0.149332	1.029813816	6.953101511
TRHDE-AS	0.914111	0.085889	1.414031076	1.415356	220.0974626	155.5038372
EVL	0.914149	0.085851	0.741544492	0.741425	778.4180903	1049.898519
PJA1	0.914183	0.085817	0.740323891	0.740158	575.4841913	777.5191861
CLIP2	0.914192	0.085808	0.740646243	0.740586	1539.672617	2078.997161
HAGLR	0.914251	0.085749	1.352986866	1.353315	707.7445931	522.9683188
GCNT1	0.914269	0.085731	1.349223331	1.349342	1936.43363	1435.092419
ZNF485	0.914326	0.085674	1.420057349	1.421493	206.9723846	145.5991342
RLTPR	0.914439	0.085561	0.716317879	0.715794	192.8376851	269.4079218
PCDHA10	0.914443	0.085557	0.370471195	0.350793	5.451955496	15.56028843
MEX3B	0.91452	0.08548	0.361136624	0.340223	5.048106941	14.85705451
MIOX	0.91452	0.08548	0.361136624	0.340223	5.048106941	14.85705451
LINC0033	0.914773	0.085227	1.950666645	1.978129	33.31750581	16.83799511
LINC0067	0.915049	0.084951	1.454027194	1.456084	156.4408341	107.4363135
SLC35D1	0.915489	0.084511	0.738627143	0.73842	464.4258386	628.948641
ALDH1A1	0.915559	0.084441	3.855231951	4.568992	9.086592493	1.980940602
HOXA-AS	0.915675	0.084325	0.20391407	0.14685	1.009621388	6.933292105
KRT34	0.915675	0.084325	0.20391407	0.14685	1.009621388	6.933292105
RASIP1	0.915675	0.084325	0.20391407	0.14685	1.009621388	6.933292105
FAM106C	0.915735	0.084265	3.414525867	3.847824	10.66160186	2.763412139
POLI	0.915817	0.084183	0.738345052	0.738144	466.4450813	631.9200519
ZBED6	0.915826	0.084174	0.725577477	0.725192	251.3957257	346.6646053
BZW2	0.91586	0.08414	1.346897206	1.347112	1058.083215	785.4429485
LOC33980	0.915929	0.084071	1.395221931	1.396256	265.5304251	190.1702977
CHST2	0.915957	0.084043	1.358630456	1.358686	4284.833171	3153.657438

UBE2D4	0.916061	0.083939	1.418630711	1.420027	207.2651748	145.9557035
ADCY3	0.916122	0.083878	0.737884248	0.737847	2543.236277	3446.836647
PRICKLE4	0.916468	0.083532	0.68069253	0.67958	94.90441049	139.6563124
ZHX1-C8o	0.916513	0.083487	0.532714893	0.526644	19.71790571	37.44968207
GPR35	0.916565	0.083435	2.005069685	2.037972	30.28864164	14.85705451
TPI1P2	0.916891	0.083109	2.304171258	2.376905	21.20204915	8.914232707
WNT5A	0.916926	0.083074	0.682996533	0.681922	97.93327465	143.6181936
CYB561	0.916929	0.083071	0.742698436	0.742578	769.3314978	1036.031935
CD27-AS1	0.917004	0.082996	0.501261577	0.493392	15.14432082	30.70457932
RRAD	0.917039	0.082961	0.743568341	0.743473	990.4385818	1332.182555
LRRC70	0.917258	0.082742	2.718689208	2.881328	15.14432082	5.249492594
CCNB2	0.917436	0.082564	1.345365212	1.345519	1465.970256	1089.517331
KCTD14	0.917459	0.082541	0.591914592	0.588398	33.22663988	56.47661655
PPA1	0.917466	0.082534	1.357559576	1.357615	4270.698472	3145.733675
HOXC9	0.917479	0.082521	0.422221159	0.408033	8.076971105	19.80940602
PRDM9	0.917479	0.082521	0.422221159	0.408033	8.076971105	19.80940602
LOC10050	0.917555	0.082445	2.170636886	2.222882	24.23091332	10.89517331
ATG14	0.917643	0.082357	0.741059744	0.740878	526.0127432	709.988921
HES4	0.917737	0.082263	0.687244309	0.68624	103.991003	151.541956
SEMA6B	0.917743	0.082257	0.743382029	0.743284	946.0152407	1272.754336
APC2	0.917857	0.082143	0.113590459	0.002518	0	3.961881203
CDK5R2	0.917857	0.082143	0.113590459	0.002518	0	3.961881203
CPXM1	0.917857	0.082143	0.113590459	0.002518	0	3.961881203
FAM150A	0.917857	0.082143	0.113590459	0.002518	0	3.961881203
MAOA	0.917857	0.082143	0.113590459	0.002518	0	3.961881203
NDNF	0.917857	0.082143	0.113590459	0.002518	0	3.961881203
PHYHIPL	0.917857	0.082143	0.113590459	0.002518	0	3.961881203
PRG2	0.917857	0.082143	0.113590459	0.002518	0	3.961881203
RAG2	0.917857	0.082143	0.113590459	0.002518	0	3.961881203
SLC16A12	0.917857	0.082143	0.113590459	0.002518	0	3.961881203
SLITRK2	0.917857	0.082143	0.113590459	0.002518	0	3.961881203
SNORA65	0.917857	0.082143	0.113590459	0.002518	0	3.961881203
TCF4	0.917857	0.082143	0.113590459	0.002518	0	3.961881203
TECTB	0.917857	0.082143	0.113590459	0.002518	0	3.961881203
TFR2	0.917857	0.082143	0.113590459	0.002518	0	3.961881203
TGFA	0.917917	0.082083	1.347908665	1.348006	2409.966254	1787.798893
SHISA8	0.917956	0.082044	0.501470004	0.493392	15.14432082	30.70457932
LRG1	0.917973	0.082027	0.704644904	0.703934	142.3566157	202.234226
FRAT1	0.918026	0.081974	0.575089417	0.570914	28.26939887	49.52351504
LOC10537	0.918459	0.081541	2.074705724	2.116214	27.25977748	12.87611391
GPR179	0.918676	0.081324	0.422534206	0.408033	8.076971105	19.80940602
IL20RA	0.918676	0.081324	0.422534206	0.408033	8.076971105	19.80940602
FAM78A	0.918691	0.081309	0.683093662	0.681972	95.91403188	140.6467827
STAG3L3	0.918935	0.081065	0.687021722	0.685972	101.8102208	148.4219746
NARF	0.918955	0.081045	0.742659676	0.742498	587.5996479	791.3857703
SND1	0.919039	0.080961	0.741385616	0.741336	1873.857296	2527.680208
CCNF	0.919256	0.080744	1.34532738	1.345578	903.6111424	671.5388639
CEP55	0.91932	0.08068	1.34430948	1.344434	1849.626383	1375.763248
TRIM4	0.919397	0.080603	0.741256183	0.741076	518.9453935	700.2625026
DNM3	0.919408	0.080592	0.593715911	0.590214	33.31750581	56.45680714
LINC0144	0.919438	0.080562	0.437186582	0.424239	8.965437927	21.14654092
LOC55422	0.919621	0.080379	1.532851547	1.537007	95.91403188	62.39962895
WWOX	0.919623	0.080377	0.672598967	0.671297	81.77933244	121.827847
VGLL3	0.919715	0.080285	0.608299537	0.605293	38.36561275	63.39009925
KDM4A-AS	0.919757	0.080243	1.866415193	1.887777	37.25502922	19.73016839
CD109	0.919796	0.080204	1.353913242	1.353973	3987.004958	2944.668204

ERCC6L	0.919873	0.080127	1.378551427	1.37934	321.0596014	232.7605207
EEPD1	0.919911	0.080089	1.449896476	1.452029	152.4528296	104.9898519
RGS11	0.919975	0.080025	0.568491675	0.563981	26.25015609	46.55210414
PIGP	0.91998	0.08002	1.346555145	1.346848	776.3988475	576.4537151
AOX1	0.919981	0.080019	1.864104155	1.885323	37.35599136	19.80940602
ALX4	0.920034	0.079966	0.339134425	0.314174	4.038485553	12.87611391
MIA2	0.920034	0.079966	0.339134425	0.314174	4.038485553	12.87611391
DLEU2L	0.920054	0.079946	1.778416032	1.79371	44.42334108	24.76175752
IP6K1	0.920096	0.079904	0.744405133	0.744305	920.774706	1237.097406
ZNF468	0.920427	0.079573	1.348814382	1.348896	2791.996891	2069.835311
NOL6	0.920495	0.079505	1.346120678	1.346221	2249.436453	1670.923397
ZNF816	0.920639	0.079361	1.3426851	1.342922	966.8841148	719.9827663
ZNF862	0.920703	0.079297	0.719396322	0.71887	190.8184424	265.4460406
CCNL2	0.920808	0.079192	0.743519521	0.743457	1480.104955	1990.845305
CSTF3-AS	0.920829	0.079171	2.896580951	3.11534	13.13517426	4.209498778
ZNF34	0.921438	0.078562	0.697118227	0.696251	118.3074343	169.9250848
FAM64A	0.921497	0.078503	1.433758725	1.435527	170.6260146	118.8564361
LINC0049	0.921508	0.078492	0.672461536	0.67109	79.76008966	118.8564361
RAB37	0.921696	0.078304	0.33967798	0.314174	4.038485553	12.87611391
CECR2	0.921818	0.078182	0.115640557	0.002569	0	3.882643579
C12orf45	0.921878	0.078122	1.37175694	1.372476	345.2905148	251.5794564
IL2RG	0.921919	0.078081	0.617482685	0.614656	41.39447691	67.35198045
ORC3	0.922017	0.077983	1.343709151	1.343823	1981.886785	1474.810278
HAGHL	0.922104	0.077896	1.390437702	1.391453	260.4823181	187.1988868
LPAR1	0.922298	0.077702	0.732502655	0.732169	285.7228528	390.2452985
RBM43	0.922351	0.077649	1.372067973	1.372799	340.2726965	247.8651928
FAM117A	0.922429	0.077571	0.661756415	0.660174	68.65425439	103.9993816
CLEC4E	0.922506	0.077494	0.455070471	0.443434	10.09621388	22.78081692
RILP	0.922506	0.077494	0.455070471	0.443434	10.09621388	22.78081692
PLCD1	0.922593	0.077407	0.634565664	0.632293	49.47144802	78.24715376
LOC10013	0.922676	0.077324	0.650520317	0.648666	59.4969884	91.72745455
SLC6A9	0.922809	0.077191	0.737981473	0.73771	354.3771072	480.3780959
GPAT3	0.922919	0.077081	1.341445336	1.341585	1597.221036	1190.545302
RPL36AL	0.922924	0.077076	0.745045745	0.74498	1444.768206	1939.340849
CD163L1	0.922971	0.077029	0.267342082	0.227386	2.019242776	8.914232707
CD22	0.922971	0.077029	0.267342082	0.227386	2.019242776	8.914232707
CYP2E1	0.922971	0.077029	0.267342082	0.227386	2.019242776	8.914232707
EOMES	0.922971	0.077029	0.267342082	0.227386	2.019242776	8.914232707
GABBR2	0.922971	0.077029	0.267342082	0.227386	2.019242776	8.914232707
MYCBPAP	0.922971	0.077029	0.267342082	0.227386	2.019242776	8.914232707
RNF175	0.922971	0.077029	0.267342082	0.227386	2.019242776	8.914232707
RPL13P5	0.923365	0.076635	1.799641341	1.816718	41.39447691	22.78081692
ACE	0.923393	0.076607	0.309480266	0.278663	3.028864164	10.89517331
AGAP2	0.923393	0.076607	0.309480266	0.278663	3.028864164	10.89517331
DNAH11	0.923393	0.076607	0.309480266	0.278663	3.028864164	10.89517331
SYPL2	0.923393	0.076607	0.309480266	0.278663	3.028864164	10.89517331
CCDC7	0.923485	0.076515	1.698552673	1.709622	52.50031218	30.70457932
FAM35DP	0.923655	0.076345	1.484388211	1.487353	118.2569532	79.50505104
SLC39A4	0.923832	0.076168	1.340634337	1.340775	1575.009366	1174.697777
NDST3	0.923902	0.076098	0.116853317	0.002657	0	3.75388244
FAM76B	0.924111	0.075889	1.343425062	1.34375	706.7349717	525.9397297
C22orf39	0.92422	0.07578	0.74170636	0.741494	439.1853039	592.3012399
MST1	0.924478	0.075522	0.636183369	0.633918	49.54212152	78.15801143
MAFB	0.92451	0.07549	0.600991273	0.597611	34.3271272	57.44727744
ZNF486	0.924543	0.075457	0.745596911	0.745528	1346.461372	1806.053261
RIPPLY3	0.924802	0.075198	0.586164114	0.58222	29.72325367	51.058744

LOC64307	0.9249	0.0751	1.644244586	1.652798	60.57728329	36.64740113
RGS20	0.924973	0.075027	1.426985433	1.428705	172.6452574	120.8373767
LINC0106	0.925063	0.074937	0.268198078	0.227386	2.019242776	8.914232707
SPX	0.925063	0.074937	0.268198078	0.227386	2.019242776	8.914232707
TMC8	0.925063	0.074937	0.268198078	0.227386	2.019242776	8.914232707
TMEM125	0.925063	0.074937	0.268198078	0.227386	2.019242776	8.914232707
FOXO6	0.925096	0.074904	0.632801335	0.630428	47.45220524	75.27574286
TMEM56	0.925189	0.074811	1.355984115	1.356028	5501.214924	4056.8574
MYO1F	0.925193	0.074807	0.310146877	0.278663	3.028864164	10.89517331
HIBADH	0.925431	0.074569	1.347902388	1.34797	3379.202786	2506.880331
ARL10	0.925454	0.074546	0.530922901	0.524367	18.17318499	34.66646053
FERMT1	0.925457	0.074543	1.341038621	1.341313	811.7355961	605.1773538
KCNJ11	0.925467	0.074533	0.696024907	0.695095	110.6343117	159.1685773
TIPARP	0.925473	0.074527	1.337833224	1.337998	1365.008117	1020.18441
TMEM120	0.925542	0.074458	1.339064753	1.339193	1766.251849	1318.890443
HCN4	0.925548	0.074452	0.412768234	0.396747	7.067349717	17.82846541
LRP3	0.925575	0.074425	0.746817313	0.746703	804.6682464	1077.631687
KIFC1	0.925626	0.074374	1.338727115	1.338882	1425.5854	1064.755573
FBXW10	0.925649	0.074351	8.419293246	369.5118	3.685118067	0
ZNF503	0.925964	0.074036	0.705644715	0.704875	131.2507805	186.2084165
SPRYD4	0.925983	0.074017	1.459961907	1.462474	133.2700232	91.12326767
LOC10272	0.92603	0.07397	0.500726448	0.49227	14.13469943	28.72363872
DUSP9	0.926523	0.073477	1.497225028	1.500618	107.0198671	71.31386166
SPOCD1	0.926748	0.073252	0.500914466	0.49227	14.13469943	28.72363872
ABCA7	0.927107	0.072893	0.73665744	0.736332	296.8286881	403.1214124
GLA	0.927336	0.072664	1.347635173	1.348062	534.0897143	396.1881203
POLE4	0.927336	0.072664	1.347635173	1.348062	534.0897143	396.1881203
GPR158	0.927484	0.072516	1.340720334	1.341028	723.8985353	539.8063139
GPR89B	0.927489	0.072511	0.667045757	0.665505	69.9667622	105.1384224
USP18	0.927579	0.072421	1.452313745	1.454629	138.3181302	95.08514887
SLC1A4	0.92764	0.07236	0.7484312	0.748336	981.3519893	1311.382678
PAAF1	0.927651	0.072349	0.742766949	0.74253	400.8196911	539.8063139
UBALD2	0.927754	0.072246	1.338306028	1.338564	855.1493158	638.853344
ADAMTS9	0.927883	0.072117	0.7453149	0.745132	504.8106941	677.4816857
CBFB	0.92791	0.07209	0.744569386	0.744526	2163.618635	2906.039862
PCDHGC4	0.927993	0.072007	8.317659092	364.4637	3.634636997	0
LOC44089	0.928048	0.071952	0.119356439	0.002721	0	3.664740113
EED	0.928061	0.071939	1.338110803	1.338371	847.0723447	632.9105222
SH3BP1	0.928071	0.071929	0.650702554	0.648708	56.53879774	87.16138647
FDXACB1	0.928079	0.071921	1.564118632	1.569661	77.74084689	49.52351504
DDX60	0.928223	0.071777	1.341642455	1.341981	659.2827665	491.2732692
FYB	0.928415	0.071585	0.690222102	0.68915	96.92365326	140.6467827
ID4	0.928428	0.071572	1.341196909	1.341286	2506.889907	1869.017458
TTF2	0.928488	0.071512	1.336221021	1.336429	1051.015865	786.4334188
TP53I3	0.92872	0.07128	0.637840711	0.635478	48.46182663	76.26621316
CD3EAP	0.928792	0.071208	1.339232014	1.339534	733.3082066	547.4329352
ADAM32	0.928831	0.071169	2.685129627	2.850403	14.13469943	4.952351504
LRRIQ3	0.928831	0.071169	2.685129627	2.850403	14.13469943	4.952351504
TSPAN15	0.928892	0.071108	1.342825321	1.342903	2899.632627	2159.225256
MANEA	0.928969	0.071031	1.336359262	1.336497	1591.163308	1190.545302
ACTBL2	0.929018	0.070982	0.592012597	0.588158	30.28864164	51.50445564
TAF4B	0.929055	0.070945	1.339413298	1.339748	675.4367087	504.1493831
ITCH	0.929104	0.070896	1.356774291	1.356811	6348.499289	4678.981701
MSR1	0.929169	0.070831	1.339557936	1.339649	2478.620508	1850.198522
AMIGO1	0.929325	0.070675	0.733486594	0.733111	253.4149684	345.674135
ARID5A	0.929345	0.070655	0.703843426	0.703007	121.1545666	172.3418323

ZCCHC9	0.929366	0.070634	1.338604806	1.338905	736.013992	549.7110169
OR7E2P	0.929383	0.070617	0.120214815	0.002743	0	3.635026004
STX18	0.929392	0.070608	1.334744034	1.33497	986.4000962	738.8908444
EEA1	0.929418	0.070582	0.748461632	0.748383	1160.054975	1550.086021
BLACE	0.92943	0.07057	0.272204201	0.230975	2.019242776	8.775566865
LSS	0.929448	0.070552	0.747326306	0.747167	576.4938126	771.5763643
RAB4B	0.92948	0.07052	1.378638757	1.379584	268.973234	194.964174
TIMM17B	0.929845	0.070155	1.334912567	1.33516	900.5822782	674.5102748
PRKAG2	0.929854	0.070146	1.335252827	1.335398	1497.268519	1121.21238
ATOH8	0.929991	0.070009	0.628822222	0.626217	43.41371969	69.33292105
PIGL	0.929991	0.070009	0.628822222	0.626217	43.41371969	69.33292105
NDUFA7	0.930048	0.069952	1.338765862	1.339082	697.6483792	520.9873782
GOT1	0.93012	0.06988	1.340004347	1.340346	649.1865526	484.3399771
MAST1	0.930221	0.069779	0.730845309	0.730424	227.1648123	311.0076744
MYO5A	0.930224	0.069776	0.74859245	0.748519	1252.940143	1673.894808
C5	0.930308	0.069692	0.668124219	0.666521	68.65425439	103.0089113
FBXO5	0.930482	0.069518	1.334498188	1.334713	1012.650252	758.7002504
UHMK1	0.930566	0.069434	0.738357083	0.738335	4337.333484	5874.479354
STS	0.930598	0.069402	0.749809857	0.749688	766.3026336	1022.16535
LINC0094	0.930672	0.069328	1.34001337	1.340365	630.609519	470.4733929
LAMA4	0.930687	0.069313	0.694467693	0.693446	100.9621388	145.5991342
MROH7	0.930695	0.069305	0.121085627	0.002766	0	3.605311895
INCA1	0.930713	0.069287	0.449992009	0.437129	9.086592493	20.79987632
KATNAL2	0.930713	0.069287	0.449992009	0.437129	9.086592493	20.79987632
GCSH	0.93077	0.06923	0.749516392	0.749406	826.8799169	1103.383915
FBXO43	0.930952	0.069048	1.382330234	1.383362	249.3764829	180.2655947
SNX10	0.930982	0.069018	0.745050059	0.744836	430.0987114	577.4441854
RGS16	0.93114	0.06886	0.586707039	0.582563	28.26939887	48.53304474
VWA9	0.931158	0.068842	0.749750968	0.749644	853.130073	1138.050376
GYLTL1B	0.931174	0.068826	0.636106872	0.633691	46.44258386	73.29480226
PTPN2	0.931276	0.068724	1.335713741	1.335828	1906.508452	1427.208275
FAM47E-S	0.931354	0.068646	0.703530623	0.702665	117.1564659	166.7357704
APITD1-C	0.931356	0.068644	1.735991821	1.75002	44.67574643	25.52441965
ERRFI1	0.931467	0.068533	0.743959856	0.743925	2637.131066	3544.893206
ERV3-1	0.931488	0.068512	0.720306354	0.719716	165.8505054	230.4428202
AJUBA	0.93152	0.06848	0.74070762	0.740682	3641.704347	4916.694573
HSD11B1L	0.931524	0.068476	0.722076049	0.721516	173.6548788	240.6842831
FXYD2	0.931558	0.068442	0.121673211	0.002781	0	3.585502489
DLX3	0.931604	0.068396	0.554725238	0.54899	21.20204915	38.62834173
CD1D	0.931665	0.068335	0.450276158	0.437129	9.086592493	20.79987632
HHLA3	0.931741	0.068259	1.344844054	1.345296	499.6818174	371.4263628
LNP1	0.932012	0.067988	1.392888414	1.394162	214.0397343	153.5228966
RNF207	0.932147	0.067853	0.743294449	0.743025	352.5295001	474.4550835
MIA-RAB4	0.932216	0.067784	8.139110605	363.4541	3.624540783	0
SEPW1	0.932269	0.067731	0.74890086	0.748742	575.4841913	768.6049534
MILR1	0.932379	0.067621	0.626567396	0.623829	41.39447691	66.36151015
C1orf233	0.932446	0.067554	0.745986987	0.745775	432.1179541	579.425126
DZIP1	0.932457	0.067543	0.706113655	0.705255	120.1449452	170.3608917
ANKRD63	0.932699	0.067301	0.401023896	0.38264	6.057728329	15.84752481
CLDN11	0.932699	0.067301	0.401023896	0.38264	6.057728329	15.84752481
UGT3A2	0.932699	0.067301	0.401023896	0.38264	6.057728329	15.84752481
MTRNR2L	0.932833	0.067167	0.122565359	0.002804	0	3.55578838
HK2	0.932844	0.067156	0.748004011	0.74782	497.7433444	665.5960421
SLC25A27	0.932892	0.067108	0.737343933	0.736978	263.5111823	357.5597786
MARK1	0.932894	0.067106	0.737040082	0.73667	260.4823181	353.5978974
WRAP53	0.932905	0.067095	1.4057728	1.407261	186.7799568	132.7230203

IQSEC1	0.932987	0.067013	0.750306192	0.750227	1153.997247	1538.200377
CERK	0.933107	0.066893	0.747999422	0.74795	1917.291209	2563.396567
SH2B2	0.933127	0.066873	0.671418227	0.669881	69.66387578	103.9993816
WDYHV1	0.93313	0.06687	1.336198358	1.336549	638.0807173	477.406685
CGREF1	0.933148	0.066852	0.478100306	0.467419	11.10583527	23.77128722
HOXD11	0.933148	0.066852	0.478100306	0.467419	11.10583527	23.77128722
KCNE1	0.933148	0.066852	0.478100306	0.467419	11.10583527	23.77128722
BOLA3-AS	0.933156	0.066844	0.531887608	0.524949	17.15346738	32.68551993
POLR2J4	0.933283	0.066717	1.532380933	1.537115	84.12165406	54.72348412
S1PR1	0.933519	0.066481	0.532220593	0.525257	17.1635636	32.68551993
MTMR9LF	0.933578	0.066422	0.59998435	0.596292	31.29826303	52.49492594
DGCR8	0.933588	0.066412	0.751102131	0.751022	1167.122325	1554.047902
ANKRD20	0.933748	0.066252	2.635845203	2.794035	13.99335244	5.001875019
RAPGEF3	0.933807	0.066193	1.336236202	1.336322	2604.823181	1949.245552
DBNDD1	0.933871	0.066129	0.750941115	0.750815	719.8600498	958.7752511
HOXD3	0.934084	0.065916	0.500076212	0.490981	13.12507805	26.74269812
GUSBP11	0.934244	0.065756	0.702171214	0.701218	109.0391099	155.5038372
SNX6	0.93437	0.06563	0.747632437	0.747587	2041.454447	2730.726619
RNASE4	0.934424	0.065576	0.685437276	0.68415	83.18270617	121.5901341
PRKCH	0.934499	0.065501	0.749882849	0.749717	550.2436565	733.9384929
LINC0068	0.934646	0.065354	0.641302455	0.638952	47.44210903	74.25555845
NFATC1	0.934899	0.065101	0.744839515	0.744582	358.4155928	481.3685662
FAM221A	0.934908	0.065092	1.515882643	1.520276	88.84668216	58.43774775
C11orf72	0.935011	0.064989	1.366255744	1.3671	288.5699852	211.0791258
SLC9A2	0.935016	0.064984	1.332507466	1.332823	699.667622	524.9492594
MAP4K5	0.935132	0.064868	0.749208618	0.749156	1749.673866	2335.528969
DUSP22	0.935171	0.064829	0.750095605	0.749911	505.8203155	674.5102748
TRIM5	0.935291	0.064709	1.335635933	1.335994	610.0536276	456.6266181
LOC10012	0.93531	0.06469	0.422902757	0.406454	7.087542145	17.4520867
CAPN10	0.935499	0.064501	0.74187521	0.741567	301.8767951	407.0832936
VRK1	0.935518	0.064482	1.329268603	1.329459	1116.641255	839.9188151
RNF115	0.935608	0.064392	1.335933003	1.336302	591.6381335	442.7402244
VANGL1	0.935623	0.064377	1.332464401	1.33257	2044.483311	1534.238496
ATG5	0.935721	0.064279	1.332308708	1.332415	2032.367854	1525.324263
CTRL	0.935805	0.064195	2.828740164	3.052825	12.11545666	3.961881203
ENTPD3-A	0.935805	0.064195	2.828740164	3.052825	12.11545666	3.961881203
PRKG1-AS	0.935805	0.064195	2.828740164	3.052825	12.11545666	3.961881203
LOC44091	0.935883	0.064117	0.384413755	0.363276	5.138972866	14.1637253
ARTN	0.93589	0.06411	0.632301112	0.629644	42.4040983	67.35198045
ZNF860	0.936092	0.063908	1.498034856	1.501932	95.51018332	63.58819331
CDK14	0.936158	0.063842	0.751569432	0.75142	608.8016971	810.204706
SDHAF3	0.9363	0.0637	0.688885081	0.687672	85.81781799	124.7992579
MACROD1	0.936359	0.063641	0.7439433	0.743657	324.0884656	435.8069323
TTC28	0.936362	0.063638	0.742965505	0.742665	308.9441448	415.9975263
KCNE4	0.936381	0.063619	0.124942342	0.002804	0	3.55578838
ZNF324	0.936599	0.063401	0.723531256	0.722936	163.4778952	226.1342744
ARHGAP5	0.936606	0.063394	0.748231687	0.748191	2303.956008	3079.372165
PCDHB5	0.936624	0.063376	0.648925845	0.646742	50.60222397	78.24715376
ITGB1BP2	0.936642	0.063358	2.198414935	2.263772	20.19242776	8.914232707
NXNL2	0.936729	0.063271	1.3931383	1.39448	198.8954135	142.6277233
SAMD4A	0.936769	0.063231	0.751723783	0.751569	586.5900265	780.490597
PMS2P2	0.936799	0.063201	1.96309339	1.99776	27.61314497	13.8170607
NEDD1	0.936857	0.063143	1.332760682	1.33285	2476.601265	1858.122284
GUSBP3	0.936937	0.063063	1.826927084	1.848302	34.79155304	18.81893571
CEP97	0.936975	0.063025	1.32776256	1.327951	1125.90958	847.8524822
BICC1	0.936994	0.063006	0.747712756	0.747675	2489.726343	3329.961151

LINC0141(	0.93721	0.06279	1.363732658	1.364578	285.6723718	209.3458028
ARHGAP1	0.937248	0.062752	1.33231415	1.332649	644.1384456	483.3495068
C8orf44-S	0.937271	0.062729	0.728278792	0.72775	181.974159	250.0541321
RIMS4	0.937431	0.062569	0.751528366	0.751467	1484.143441	1974.99778
TWSG1	0.937458	0.062542	1.356656364	1.356684	8644.378325	6371.695445
HBQ1	0.937495	0.062505	2.080965833	2.1303	23.22129193	10.89517331
SLC12A3	0.937495	0.062505	2.080965833	2.1303	23.22129193	10.89517331
TACC2	0.937671	0.062329	1.326104336	1.326291	1156.016489	871.6138647
GLDC	0.937688	0.062312	1.335960972	1.336395	514.906908	385.292947
COL8A1	0.937758	0.062242	0.607693742	0.604125	32.30788442	53.48539624
TCEANC	0.937926	0.062074	1.387426081	1.388733	204.9531418	147.5800748
C14orf105	0.938104	0.061896	1.843241064	1.866222	33.31750581	17.84827482
APOBEC3	0.938135	0.061865	0.44387189	0.429497	8.076971105	18.81893571
RAB9B	0.938135	0.061865	0.44387189	0.429497	8.076971105	18.81893571
THNSL2	0.938171	0.061829	0.704625834	0.70369	108.0294885	153.5228966
TOM1	0.938171	0.061829	0.752886561	0.752723	564.378356	749.7860177
TICRR	0.938284	0.061716	1.331210044	1.331539	650.196174	488.3018583
ZNF594	0.938353	0.061647	0.726294146	0.725727	169.6365856	233.750991
SOBP	0.938384	0.061616	0.733674741	0.733216	206.9723846	282.2840357
ACOT7	0.938402	0.061598	1.333615264	1.333694	2737.083583	2052.254463
RFX7	0.938457	0.061543	0.749828984	0.749619	432.1179541	576.4537151
KCNN3	0.938479	0.061521	0.358281818	0.332562	4.028389339	12.13326118
PLEKHH3	0.938534	0.061466	0.752084062	0.751897	496.733723	660.6436906
FAM131B	0.938593	0.061407	0.728664363	0.728108	176.6837429	242.6652237
MCM6	0.938611	0.061389	0.75164106	0.751585	1615.394221	2149.320553
GEMIN8P4	0.938766	0.061234	1.675494976	1.686937	48.46182663	28.72363872
LOC64284	0.938774	0.061226	1.44210394	1.44453	128.1007617	88.67680603
CCDC183	0.938901	0.061099	0.386040069	0.364507	5.048106941	13.86658421
KIF26B	0.938901	0.061099	0.386040069	0.364507	5.048106941	13.86658421
RAB31	0.938914	0.061086	0.750857889	0.750808	1840.539791	2451.413994
NCAPD3	0.938978	0.061022	1.328836469	1.328944	1971.790571	1483.724511
ZC3HAV1	0.939041	0.060959	1.475396372	1.478686	103.991003	70.32339135
RAC2	0.939109	0.060891	0.637869832	0.635291	43.41371969	68.34245075
EFNA3	0.939309	0.060691	0.714330607	0.713551	127.2122949	178.2846541
PRDM11	0.939375	0.060625	0.66660121	0.664821	60.57728329	91.12326767
RNF7	0.939391	0.060609	1.326858972	1.326985	1670.358009	1258.758991
SNCA	0.939394	0.060606	0.618487849	0.615183	35.33674859	57.44727744
TC2N	0.939479	0.060521	0.754029405	0.753873	590.6285121	783.4620079
DNAJC1	0.939574	0.060426	0.754471403	0.754351	750.1486914	994.432182
GPR143	0.939811	0.060189	0.583910034	0.579264	25.2405347	43.58069323
PAK1IP1	0.93983	0.06017	1.323977057	1.324192	995.4866887	751.7669583
GCHFR	0.939873	0.060127	1.356787797	1.357574	299.8575523	220.8748771
VPS37D	0.939902	0.060098	0.705118989	0.704166	106.0102458	150.5514857
WFS1	0.939968	0.060032	0.750632152	0.750401	402.8389339	536.834903
HIST1H2E	0.940002	0.059998	0.722491838	0.721839	149.4239654	207.0082929
COX7A1	0.940016	0.059984	1.883671225	1.910679	30.28864164	15.84752481
PABPC1L	0.940029	0.059971	0.731440754	0.730929	186.7799568	255.5413376
RRN3P2	0.940062	0.059938	7.761274668	344.2713	3.43271272	0
LINC0141	0.940079	0.059921	0.386510249	0.364507	5.048106941	13.86658421
PCDHGA2	0.940079	0.059921	0.386510249	0.364507	5.048106941	13.86658421
ZNF789	0.940103	0.059897	0.584013924	0.579264	25.2405347	43.58069323
TSPAN12	0.940144	0.059856	0.68400548	0.682619	75.72160411	110.9326737
BNIP1	0.940287	0.059713	1.352660169	1.353388	319.0403587	235.7319316
SOCS7	0.940402	0.059598	0.754189608	0.754117	1239.976604	1644.279746
LOC10272	0.940443	0.059557	0.627764663	0.624815	38.36561275	61.40915865
DERL3	0.940551	0.059449	0.475294501	0.46358	10.09621388	21.79034662

PARM1	0.940551	0.059449	0.475294501	0.46358	10.09621388	21.79034662
DPCD	0.940616	0.059384	0.728057158	0.727486	168.6067718	231.7700504
JPH4	0.940726	0.059274	3.055585987	3.389742	10.09621388	2.971410902
PMS2P7	0.940782	0.059218	1.483424135	1.487114	96.95394191	65.1927552
HOXB4	0.940797	0.059203	0.754947301	0.754813	668.369359	885.4804489
SAPCD1-/	0.940958	0.059042	4.411999828	6.064876	6.057728329	0.990470301
SLC4A2	0.940987	0.059013	0.752959743	0.752903	1589.144065	2110.692211
LOC64673	0.94114	0.05886	3.054379346	3.389742	10.09621388	2.971410902
CCDC138	0.941253	0.058747	1.327509977	1.327839	643.1288243	484.3399771
XKR5	0.941257	0.058743	0.533816931	0.526263	16.15394221	30.70457932
FBXO48	0.941355	0.058645	1.447210449	1.449858	119.1959011	82.20903496
IQUB	0.941371	0.058629	0.518294889	0.509844	14.13469943	27.73316842
CYP2C18	0.941546	0.058454	4.407329101	6.064876	6.057728329	0.990470301
ELOVL2-A	0.941546	0.058454	4.407329101	6.064876	6.057728329	0.990470301
AKAP12	0.941582	0.058418	1.349213535	1.349245	7288.658725	5402.02502
AGPAT5	0.941611	0.058389	1.336212216	1.336268	4035.456688	3019.943947
TMEM50B	0.941615	0.058385	0.756168323	0.756037	698.6580006	924.1087906
UBE2B	0.941617	0.058383	1.323632577	1.323774	1475.056848	1114.279088
RNF103-C	0.941641	0.058359	1.619994243	1.629085	55.28686722	33.9335125
HIST2H2E	0.941661	0.058339	0.638044107	0.63539	42.16178917	66.36151015
ALDH1L2	0.941661	0.058339	0.576996048	0.571928	23.22129193	40.60928233
FAM210B	0.941797	0.058203	0.756520626	0.756406	801.6393822	1059.803222
INHBA	0.941882	0.058118	0.700494282	0.69942	94.90441049	135.6944312
TRAF3IP2	0.942076	0.057924	0.694374141	0.693173	85.81781799	123.8087876
AQP1	0.942081	0.057919	0.499542092	0.489487	12.11545666	24.76175752
INSRR	0.942123	0.057877	1.605512958	1.613775	57.54841913	35.65693083
GCKR	0.942251	0.057749	1.93246076	1.965165	27.25977748	13.86658421
LINC0118	0.942251	0.057749	1.93246076	1.965165	27.25977748	13.86658421
FAM213B	0.942318	0.057682	0.756130111	0.756052	1174.189674	1553.057432
RWDD3	0.942336	0.057664	1.321772203	1.322017	863.7613862	653.3637339
LOC43999	0.94237	0.05763	0.68664958	0.685241	75.84275868	110.6850561
ACYP1	0.942401	0.057599	1.361695023	1.362616	260.4823181	191.160768
GRIP1	0.942552	0.057448	0.732161313	0.731631	179.7126071	245.6366346
MAGED2	0.942634	0.057366	0.751772187	0.751729	2124.243401	2825.811768
PSTK	0.942671	0.057329	1.479029892	1.482596	96.92365326	65.37103985
LSM3	0.942674	0.057326	1.325632777	1.325958	646.1576884	487.311388
GPC2	0.94272	0.05728	1.802883041	1.823636	34.3271272	18.81893571
LOC40065	0.942757	0.057243	1.322981604	1.323277	720.8696711	544.7586654
MRE11A	0.942775	0.057225	1.321474265	1.321648	1187.314752	898.3565628
PRKAR1B	0.942807	0.057193	0.711909653	0.711036	114.0872169	160.4561887
MCRS1	0.942879	0.057121	1.321349424	1.321524	1183.276267	895.3851519
CCDC112	0.942976	0.057024	1.322941746	1.323242	707.7445931	534.8539624
ACVR2A	0.943048	0.056952	0.729935765	0.729364	167.5971504	229.7891098
TSPYL5	0.943164	0.056836	0.75513136	0.754958	518.9453935	687.3863887
SLC26A4	0.94321	0.05679	1.487767856	1.491622	91.85535389	61.5775386
HS3ST3B1	0.943252	0.056748	0.230134373	0.16848	1.009621388	6.041868835
TXNIP	0.943363	0.056637	1.39269617	1.392702	47975.18912	34447.56659
ACBD5	0.943419	0.056581	1.327346002	1.32743	2509.918771	1890.807804
ZNF22	0.943543	0.056457	0.751125223	0.750874	361.444457	481.3685662
KCCAT21	0.94356	0.05644	0.633930988	0.631076	39.37523414	62.39962895
ANKLE1	0.943572	0.056428	0.433492793	0.416885	7.067349717	16.96675625
LOC10192	0.943572	0.056428	0.696969597	0.695761	86.82743938	124.7992579
FRG1BP	0.943581	0.056419	1.32399148	1.324298	678.4554766	512.3108584
ARHGAP4	0.943606	0.056394	0.611427963	0.607757	31.29826303	51.50445564
NAV1	0.943632	0.056368	0.757608931	0.757482	718.7797549	948.910167
CMBL	0.943642	0.056358	1.323979631	1.324086	1968.135742	1486.408685

IGSF9	0.943657	0.056343	0.365676968	0.339768	4.038485553	11.90545302
ARHGAP1	0.943719	0.056281	0.131569306	0.003041	0	3.278456696
NANOS1	0.943734	0.056266	0.738227799	0.73778	208.9916273	283.274506
CCT6B	0.943778	0.056222	0.611510202	0.607757	31.29826303	51.50445564
LOC10050	0.943783	0.056217	0.719848134	0.719099	130.9579903	182.1177742
TOR1B	0.94399	0.05601	1.319090662	1.319271	1159.045354	878.5471568
LMF1	0.94399	0.05601	0.692382117	0.691101	80.76971105	116.8754955
DUSP11	0.944044	0.055956	1.323778308	1.3241	649.1865526	490.2827989
GLIS3-AS	0.944054	0.055946	0.366261983	0.340333	4.038485553	11.88564361
HGFAC	0.944054	0.055946	0.366261983	0.340333	4.038485553	11.88564361
RENBP	0.944054	0.055946	0.366261983	0.340333	4.038485553	11.88564361
RGCC	0.944054	0.055946	0.366261983	0.340333	4.038485553	11.88564361
CA3	0.94406	0.05594	3.460423593	4.061885	8.076971105	1.980940602
CCDC116	0.94406	0.05594	3.460423593	4.061885	8.076971105	1.980940602
LINC0031	0.94406	0.05594	3.460423593	4.061885	8.076971105	1.980940602
LINC0096	0.94406	0.05594	3.460423593	4.061885	8.076971105	1.980940602
IPMK	0.944165	0.055835	0.757559196	0.757445	785.48544	1037.022405
CASC8	0.944192	0.055808	1.456963693	1.460066	107.0198671	73.29480226
PMFBP1	0.944197	0.055803	1.743717681	1.760321	38.36561275	21.79034662
TAF3	0.944215	0.055785	1.336883165	1.337439	394.7619628	295.1601496
KCNN4	0.944227	0.055773	0.751569286	0.751317	359.4252142	478.3971553
HMGB1	0.944313	0.055687	1.358550327	1.35857	12259.97386	9024.17491
LBHD1	0.944325	0.055675	1.535363092	1.540895	72.56148917	47.0869581
TXNDC12	0.944332	0.055668	1.338544331	1.338589	4925.458135	3679.587263
LYAR	0.944553	0.055447	1.32003662	1.320263	903.6111424	684.4149778
RAB30	0.944557	0.055443	1.343333349	1.344029	330.1461939	245.6366346
CREBRF	0.944564	0.055436	1.319363393	1.319539	1167.122325	884.4899786
RNASET2	0.944575	0.055425	0.751758911	0.751721	2404.918147	3199.219072
GNGT2	0.944686	0.055314	2.217205119	2.291874	18.17318499	7.923762406
CELF2	0.944803	0.055197	0.436353005	0.420071	7.067349717	16.83799511
GCNT4	0.944803	0.055197	0.436353005	0.420071	7.067349717	16.83799511
TRIM46	0.944813	0.055187	0.622264746	0.618951	34.3271272	55.46633684
ETFDH	0.94489	0.05511	1.361865681	1.362848	244.3283759	179.2751244
EN2	0.944919	0.055081	0.559662539	0.553482	19.18280638	34.66646053
PLXNB3	0.944919	0.055081	0.559662539	0.553482	19.18280638	34.66646053
HMGN3-A	0.944984	0.055016	1.420625047	1.422778	135.289266	95.08514887
GPLD1	0.945161	0.054839	0.622805972	0.619497	34.35741584	55.46633684
HOXA13	0.945213	0.054787	0.726634372	0.725979	147.4047227	203.0464117
HUS1	0.94525	0.05475	1.344858498	1.345559	321.0596014	238.6042955
SLITRK4	0.945257	0.054743	0.366824883	0.340333	4.038485553	11.88564361
ZNF668	0.945287	0.054713	0.737314277	0.736833	194.8569279	264.4555703
MAPRE3	0.94543	0.05457	1.323228272	1.323574	601.7343473	454.6258681
TOM1L2	0.945508	0.054492	1.318210297	1.318465	818.8029458	621.0248786
CYGB	0.945528	0.054472	2.215615945	2.291874	18.17318499	7.923762406
ZDHHC13	0.945598	0.054402	1.318326156	1.318546	929.8612985	705.2148542
SOX2-OT	0.945625	0.054375	3.449540357	4.061885	8.076971105	1.980940602
LOC44017	0.945705	0.054295	2.099823442	2.155768	20.61646875	9.558038402
NACAD	0.945705	0.054295	0.436710711	0.420071	7.067349717	16.83799511
ZNF771	0.945705	0.054295	0.436710711	0.420071	7.067349717	16.83799511
NRTN	0.945807	0.054193	0.711824092	0.710871	107.0198671	150.5514857
SDF2L1	0.945848	0.054152	0.673925957	0.672126	60.57728329	90.13279737
IDUA	0.94586	0.05414	0.730053601	0.729445	157.5009366	215.9225256
METTL17	0.946015	0.053985	0.748407293	0.748067	275.626639	368.4549519
C17orf53	0.946089	0.053911	1.350274996	1.351083	283.7036101	209.9797038
CHST1	0.946231	0.053769	0.233558247	0.170999	1.009621388	5.952726508
TEX30	0.946381	0.053619	1.322576259	1.322959	554.2821421	418.9689372

CEND1	0.946493	0.053507	0.233917486	0.171284	1.009621388	5.942821805
CPNE5	0.946493	0.053507	0.233917486	0.171284	1.009621388	5.942821805
EPHA4	0.946493	0.053507	0.233917486	0.171284	1.009621388	5.942821805
FABP4	0.946493	0.053507	0.233917486	0.171284	1.009621388	5.942821805
GIPC3	0.946493	0.053507	0.233917486	0.171284	1.009621388	5.942821805
HAPLN2	0.946493	0.053507	0.233917486	0.171284	1.009621388	5.942821805
LINC0109	0.946493	0.053507	0.233917486	0.171284	1.009621388	5.942821805
LINC0160	0.946493	0.053507	0.233917486	0.171284	1.009621388	5.942821805
LRP1B	0.946493	0.053507	0.233917486	0.171284	1.009621388	5.942821805
PARD6G-/	0.946493	0.053507	0.233917486	0.171284	1.009621388	5.942821805
ZFPM2	0.946493	0.053507	0.233917486	0.171284	1.009621388	5.942821805
PRR29	0.946572	0.053428	0.70888142	0.707892	100.9621388	142.6277233
THOC3	0.946682	0.053318	1.316986182	1.317174	1075.872744	816.8012382
PARP10	0.946701	0.053299	0.759126406	0.758972	588.6092693	775.5382455
LOC65371	0.946771	0.053229	7.347661511	314.9923	3.139922517	0
SPTSSA	0.946798	0.053202	0.759879249	0.759753	718.8504284	946.1665642
PAQR7	0.946907	0.053093	0.727398827	0.726731	144.3758585	198.6685329
C15orf57	0.946917	0.053083	1.339440405	1.340097	337.0116194	251.4804094
RORA	0.946962	0.053038	0.717482274	0.716641	117.116081	163.4275996
HTATSF1	0.947043	0.052957	1.411959104	1.413994	139.3277516	98.53198552
DHCR7	0.947047	0.052953	0.751511619	0.751222	313.9922517	417.9784669
APOL2	0.947306	0.052694	0.758666183	0.758592	1190.343617	1569.152574
HOXB3	0.94734	0.05266	0.759780443	0.759679	874.3321221	1150.926489
SCARF1	0.947415	0.052585	0.237176837	0.174967	1.029813816	5.932917102
ZNF441	0.947446	0.052554	1.351398567	1.352266	266.5400465	197.1035899
SLFN12	0.947472	0.052528	1.340062215	1.340771	320.04998	238.7033425
PARP3	0.947507	0.052493	0.746677173	0.746307	248.3668615	332.7980211
LOC38970	0.947526	0.052474	0.338821036	0.306501	3.028864164	9.904703008
FIRRE	0.947536	0.052464	0.472402109	0.459433	9.086592493	19.78959661
RAB9A	0.947644	0.052356	1.32578048	1.32585	2991.508173	2256.291345
DEPDC4	0.947664	0.052336	1.459113966	1.462457	99.95251743	68.34245075
DEFB1	0.947687	0.052313	0.602637411	0.598394	27.25977748	45.56163384
SLC2A3	0.947821	0.052179	0.234504676	0.170716	1.009621388	5.962631211
TRAF1	0.947838	0.052162	0.629170145	0.625974	35.33674859	56.45680714
HCN2	0.947871	0.052129	0.75906673	0.758904	547.2147924	721.062379
CHRNA4	0.947895	0.052105	0.338950444	0.306501	3.028864164	9.904703008
IFITM2	0.947933	0.052067	0.748539697	0.748516	3890.545731	5197.681093
SLCO2A1	0.947972	0.052028	0.518945963	0.509858	13.12507805	25.75222782
PCYT2	0.947975	0.052025	0.74578735	0.745397	236.2514048	316.9504962
GATA6	0.947995	0.052005	1.314124321	1.314309	1115.631634	848.8330478
WDR97	0.948016	0.051984	0.580522975	0.57512	22.21167054	38.62834173
SEC24B-/	0.948059	0.051941	2.274976209	2.364346	16.40634756	6.933292105
PELI1	0.948089	0.051911	1.321336404	1.321714	547.2147924	414.0165857
TLR5	0.948093	0.051907	0.709507363	0.708495	98.94289604	139.6563124
NUTM2D	0.94815	0.05185	1.567086292	1.574402	60.7287265	38.56891351
RACGAP1	0.948166	0.051834	1.329099752	1.329156	3780.961425	2844.630704
IFI30	0.948248	0.051752	0.756969664	0.756756	418.9928761	553.6728981
EPHA2	0.948275	0.051725	1.3488491	1.348873	9575.249245	7098.700646
ACSS1	0.948287	0.051713	0.73602835	0.735481	172.6452574	234.7414613
ADAM33	0.948329	0.051671	0.235224834	0.171284	1.009621388	5.942821805
DES	0.948329	0.051671	0.235224834	0.171284	1.009621388	5.942821805
DNM1P46	0.948329	0.051671	0.235224834	0.171284	1.009621388	5.942821805
PLAC8L1	0.948329	0.051671	0.235224834	0.171284	1.009621388	5.942821805
SSPO	0.948329	0.051671	0.235224834	0.171284	1.009621388	5.942821805
STARD4-/	0.948329	0.051671	0.235224834	0.171284	1.009621388	5.942821805
TEX36	0.948329	0.051671	0.235224834	0.171284	1.009621388	5.942821805

TP73	0.948329	0.051671	0.235224834	0.171284	1.009621388	5.942821805
EFCAB6	0.948341	0.051659	1.672846375	1.685558	43.41371969	25.75222782
H1F0	0.948355	0.051645	0.760503423	0.760404	889.476443	1169.745425
PCDHGB6	0.948368	0.051632	0.739453552	0.738959	189.6371853	256.6308549
GAREM1	0.948506	0.051494	0.755862838	0.755611	364.4733211	482.3590365
LINC0113	0.948578	0.051422	1.354607953	1.355595	242.490865	178.8789363
LINC0070	0.948597	0.051403	1.463624398	1.467154	95.91403188	65.37103985
DYNC2LI1	0.948664	0.051336	1.315398274	1.315692	703.7061075	534.8539624
BNIPL	0.948706	0.051294	0.498680801	0.487733	11.10583527	22.78081692
LINC0141	0.948706	0.051294	0.498680801	0.487733	11.10583527	22.78081692
WDR60	0.948734	0.051266	0.756326793	0.756094	385.6753703	510.0922049
SYTL3	0.94878	0.05122	1.334743265	1.335395	341.2520292	255.5413376
ZNF700	0.94881	0.05119	1.325878016	1.326381	427.9280254	322.6258911
MATK	0.948984	0.051016	0.757176377	0.756955	404.8581767	534.8539624
EFHD1	0.949015	0.050985	0.645698795	0.643021	41.39447691	64.38056955
ST20-AS1	0.949055	0.050945	0.645758901	0.643021	41.39447691	64.38056955
ATP8A2	0.949161	0.050839	0.339649309	0.306501	3.028864164	9.904703008
IL23A	0.949161	0.050839	0.339649309	0.306501	3.028864164	9.904703008
ST8SIA5	0.949161	0.050839	0.339649309	0.306501	3.028864164	9.904703008
TNFSF14	0.949397	0.050603	0.425496637	0.406917	6.088016971	14.97591095
NLGN2	0.949443	0.050557	0.761333997	0.761234	881.3994719	1157.859782
CDON	0.949518	0.050482	1.315026645	1.31531	711.3186528	540.7967842
LINC0052	0.94959	0.05041	1.924594216	1.959515	25.2405347	12.87611391
NUDT11	0.949655	0.050345	0.730226056	0.729562	144.2446077	197.7176814
ABCA12	0.949665	0.050335	0.298788643	0.255773	2.019242776	7.923762406
MYB	0.949665	0.050335	0.298788643	0.255773	2.019242776	7.923762406
ECI1	0.949822	0.050178	1.327367908	1.327423	3806.272633	2867.411521
MS4A10	0.949946	0.050054	0.238211985	0.174226	0.999525174	5.784346557
CCDC84	0.949999	0.050001	1.356100116	1.357117	231.2032979	170.3608917
FAM98C	0.950016	0.049984	1.364581168	1.365752	206.9723846	151.541956
ZDHHC21	0.950071	0.049929	0.759026385	0.758823	438.1756825	577.4441854
FBXO44	0.950166	0.049834	0.761920903	0.761819	862.2166655	1131.790603
CIB2	0.950171	0.049829	0.741477011	0.740988	190.8184424	257.5222782
TPM3P9	0.950184	0.049816	1.362285012	1.363419	212.0204915	155.5038372
ANKRD20	0.950214	0.049786	1.883940641	1.914658	26.80544786	13.99534535
PANK1	0.950227	0.049773	0.735297901	0.734709	160.7115326	218.7453659
LOC10012	0.950419	0.049581	2.496821693	2.646946	13.12507805	4.952351504
LOC28392	0.950436	0.049564	1.395334585	1.397134	149.3734844	106.9113643
SULT1C2	0.950439	0.049561	1.312961782	1.313102	1421.546915	1082.584039
CGB	0.950461	0.049539	0.138855491	0.003236	0	3.080362635
LOC10027	0.950483	0.049517	7.123146152	303.8864	3.028864164	0
LOC64362	0.950483	0.049517	7.123146152	303.8864	3.028864164	0
TMEM150	0.950483	0.049517	7.123146152	303.8864	3.028864164	0
PARD6G	0.950551	0.049449	0.757252617	0.757009	367.5021853	485.4691132
CRABP1	0.950632	0.049368	0.732380217	0.731715	146.3951013	200.0750008
LOC10013	0.950632	0.049368	1.885365221	1.916418	26.58333115	13.86658421
SMG1P3	0.950703	0.049297	1.310821656	1.310988	1218.562534	929.4969491
TXNL4B	0.950706	0.049294	1.312327167	1.312579	793.0979853	604.2265023
FAM26F	0.950744	0.049256	0.426893947	0.408133	6.057728329	14.85705451
PBX2	0.950849	0.049151	0.760730086	0.760668	1451.835556	1908.63627
RPIA	0.950853	0.049147	1.311381853	1.311598	919.7650846	701.2529729
DHRS3	0.950857	0.049143	0.761561525	0.761491	1276.161435	1675.875749
CALHM2	0.950887	0.049113	0.562647176	0.556137	18.17318499	32.68551993
ABHD13	0.950998	0.049002	0.762065783	0.761917	594.6669976	780.490597
ENDOV	0.951036	0.048964	0.712372418	0.711356	97.93327465	137.6753718
FAM53A	0.951055	0.048945	0.644061939	0.641253	39.37523414	61.40915865

AUNIP	0.951062	0.048938	1.317838726	1.31823	520.9646363	395.19765
BREA2	0.951081	0.048919	0.299704139	0.255773	2.019242776	7.923762406
TFAMP1	0.951081	0.048919	0.299704139	0.255773	2.019242776	7.923762406
CAMK2B	0.95112	0.04888	0.72078072	0.719923	114.0872169	158.4752481
ZCCHC7	0.951126	0.048874	1.311319187	1.311478	1250.9209	953.8228996
NNT-AS1	0.951195	0.048805	1.467741867	1.471469	89.85630355	61.06249404
CDH3	0.951247	0.048753	0.731717957	0.731052	143.3662371	196.1131196
ABI3	0.951271	0.048729	7.098049244	303.8864	3.028864164	0
ADAM20	0.951271	0.048729	7.098049244	303.8864	3.028864164	0
AXDND1	0.951271	0.048729	7.098049244	303.8864	3.028864164	0
CALCR	0.951271	0.048729	7.098049244	303.8864	3.028864164	0
CD53	0.951271	0.048729	7.098049244	303.8864	3.028864164	0
CWH43	0.951271	0.048729	7.098049244	303.8864	3.028864164	0
FAM83E	0.951271	0.048729	7.098049244	303.8864	3.028864164	0
HIST1H3J	0.951271	0.048729	7.098049244	303.8864	3.028864164	0
HIST4H4	0.951271	0.048729	7.098049244	303.8864	3.028864164	0
HMX3	0.951271	0.048729	7.098049244	303.8864	3.028864164	0
KIF25	0.951271	0.048729	7.098049244	303.8864	3.028864164	0
LINC0062	0.951271	0.048729	7.098049244	303.8864	3.028864164	0
LINC0151	0.951271	0.048729	7.098049244	303.8864	3.028864164	0
LINC0154	0.951271	0.048729	7.098049244	303.8864	3.028864164	0
LOC10192	0.951271	0.048729	7.098049244	303.8864	3.028864164	0
LOC10192	0.951271	0.048729	7.098049244	303.8864	3.028864164	0
LOC10193	0.951271	0.048729	7.098049244	303.8864	3.028864164	0
MFSD7	0.951271	0.048729	7.098049244	303.8864	3.028864164	0
NAP1L6	0.951271	0.048729	7.098049244	303.8864	3.028864164	0
OR1F2P	0.951271	0.048729	7.098049244	303.8864	3.028864164	0
PLPP7	0.951271	0.048729	7.098049244	303.8864	3.028864164	0
RFPL4B	0.951271	0.048729	7.098049244	303.8864	3.028864164	0
ZFP92	0.951271	0.048729	7.098049244	303.8864	3.028864164	0
PCAT6	0.951274	0.048726	0.562792218	0.556137	18.17318499	32.68551993
UBQLNL	0.951274	0.048726	0.562792218	0.556137	18.17318499	32.68551993
HSPA1B	0.951445	0.048555	0.760043213	0.759986	1539.470693	2025.660336
SNX12	0.951533	0.048467	0.763053686	0.762934	736.013992	964.718073
FOXD4L6	0.951591	0.048409	7.077722413	302.8768	3.018767951	0
LINC0099	0.951591	0.048409	7.077722413	302.8768	3.018767951	0
NICN1	0.951671	0.048329	0.741572552	0.741062	182.6607015	246.4884391
ALG1L	0.951724	0.048276	0.378994704	0.352666	4.038485553	11.46964608
MTRNR2L	0.951789	0.048211	1.567127537	1.5747	57.3868797	36.43940237
IL1RAPL1	0.951822	0.048178	2.241005035	2.327994	16.15394221	6.933292105
PABPC4L	0.951827	0.048173	0.677486587	0.675644	57.54841913	85.18044587
LINC0026	0.951909	0.048091	7.057395582	301.8672	3.008671737	0
SULT1A3	0.951922	0.048078	1.313708856	1.31407	568.063474	432.2907628
SULT1A4	0.951922	0.048078	1.313708856	1.31407	568.063474	432.2907628
PAX8	0.952012	0.047988	1.338700446	1.33873	7625.670345	5696.1947
LOC10027	0.952026	0.047974	2.09113311	2.150639	19.18280638	8.914232707
FOXA1	0.952094	0.047906	0.737300259	0.736714	160.5298007	217.9034662
BORCS7-/	0.952152	0.047848	7.008085532	157.2357	3.119730089	0.009904703
MBP	0.952174	0.047826	1.320539515	1.320605	3208.576772	2429.623648
BAMBI	0.95218	0.04782	0.761345274	0.761152	458.3681102	602.2059429
XPA	0.952238	0.047762	1.317583448	1.318003	485.6278877	368.4549519
NUP43	0.952319	0.047681	1.31347528	1.313579	1926.458571	1466.569565
NR2C2AP	0.952348	0.047652	1.317196396	1.317277	2522.034228	1914.579091
BTN3A1	0.952405	0.047595	1.310412645	1.310714	670.87322	511.8354326
ZNF670	0.952419	0.047581	1.314316801	1.314672	563.9745074	428.982592
RPF2	0.952486	0.047514	1.322084846	1.322146	3394.347107	2567.29902

MAP2K5	0.952576	0.047424	0.761786373	0.761595	462.4065958	607.1582944
HAGLROS	0.952603	0.047397	1.562700961	1.570172	57.54841913	36.64740113
LOC73020	0.952625	0.047375	2.239169117	2.327994	16.15394221	6.933292105
LOC14870	0.952636	0.047364	0.14160008	0.00331	0	3.011029714
POTEE	0.952678	0.047322	0.63399152	0.630733	34.46847419	54.6541512
B3GNT4	0.952796	0.047204	0.696855133	0.695463	73.70236134	105.9803222
CEP128	0.952814	0.047186	1.33127515	1.331975	313.9922517	235.7319316
UGT1A4	0.952822	0.047178	0.14110793	0.003226	0	3.090267338
SSBP2	0.952825	0.047175	0.752881887	0.752517	253.4149684	336.7599023
VIPAS39	0.95284	0.04716	1.311447573	1.311761	634.0422318	483.3495068
MAPK7	0.952853	0.047147	0.744334325	0.743849	190.8184424	256.5318079
AGAP12P	0.952971	0.047029	3.583477317	4.397184	6.784655728	1.535228966
PRRG1	0.952973	0.047027	1.308362312	1.308538	1114.622013	851.8044587
RHOD	0.952978	0.047022	1.308397719	1.308571	1132.795198	865.6710429
ZNF565	0.95301	0.04699	1.444449926	1.447686	98.94289604	68.34245075
CHI3L1	0.953089	0.046911	0.142714454	0.003354	0	2.971410902
IQCA1	0.953089	0.046911	0.142714454	0.003354	0	2.971410902
PTPN22	0.953089	0.046911	0.142714454	0.003354	0	2.971410902
RSPO3	0.953089	0.046911	0.142714454	0.003354	0	2.971410902
SPTY2D1-	0.953089	0.046911	0.142714454	0.003354	0	2.971410902
TMSB15B	0.953089	0.046911	0.142714454	0.003354	0	2.971410902
INADL	0.953127	0.046873	1.313822682	1.313916	2144.910351	1632.453531
LOC10050	0.953179	0.046821	1.580403437	1.588789	53.50993357	33.67599023
RNF208	0.953198	0.046802	0.58417746	0.578657	21.20204915	36.64740113
AVPR1A	0.953241	0.046759	0.142404289	0.003332	0	2.991220308
SAPCD2	0.953256	0.046744	1.308904132	1.309048	1363.998495	1041.974756
RBM20	0.953294	0.046706	0.467863056	0.453345	8.076971105	17.82846541
TRMO	0.953351	0.046649	1.343738249	1.344637	250.3861043	186.2084165
PCDHGC3	0.95339	0.04661	0.741016299	0.740469	170.3837055	230.1060603
LUC7L3	0.95348	0.04652	0.762596194	0.762532	1367.02736	1792.751244
CFAP57	0.953521	0.046479	1.404870196	1.407081	128.2219163	91.12326767
GGT8P	0.95354	0.04646	0.142809829	0.003343	0	2.981315605
COL11A2	0.953556	0.046444	0.706267771	0.705065	83.79857522	118.8564361
HEYL	0.953608	0.046392	0.681393365	0.679594	58.55804051	86.17091617
ZNF573	0.953631	0.046369	0.677455137	0.675503	55.52917635	82.20903496
MAMLD1	0.953759	0.046241	0.673030436	0.670997	52.50031218	78.24715376
TLR2	0.95382	0.04618	0.754667455	0.754315	261.4919395	346.6646053
ADGRV1	0.953838	0.046162	0.143217685	0.003354	0	2.971410902
C1orf140	0.953838	0.046162	0.143217685	0.003354	0	2.971410902
CACNA1F	0.953838	0.046162	0.143217685	0.003354	0	2.971410902
CCDC79	0.953838	0.046162	0.143217685	0.003354	0	2.971410902
CCER2	0.953838	0.046162	0.143217685	0.003354	0	2.971410902
CD69	0.953838	0.046162	0.143217685	0.003354	0	2.971410902
CHST9	0.953838	0.046162	0.143217685	0.003354	0	2.971410902
CILP	0.953838	0.046162	0.143217685	0.003354	0	2.971410902
CLEC4F	0.953838	0.046162	0.143217685	0.003354	0	2.971410902
COL18A1-	0.953838	0.046162	0.143217685	0.003354	0	2.971410902
CRYM	0.953838	0.046162	0.143217685	0.003354	0	2.971410902
CX3CR1	0.953838	0.046162	0.143217685	0.003354	0	2.971410902
CXCL14	0.953838	0.046162	0.143217685	0.003354	0	2.971410902
CYP4F3	0.953838	0.046162	0.143217685	0.003354	0	2.971410902
DNAH8	0.953838	0.046162	0.143217685	0.003354	0	2.971410902
ENTPD3	0.953838	0.046162	0.143217685	0.003354	0	2.971410902
FCMR	0.953838	0.046162	0.143217685	0.003354	0	2.971410902
FCRLB	0.953838	0.046162	0.143217685	0.003354	0	2.971410902
FRMD1	0.953838	0.046162	0.143217685	0.003354	0	2.971410902

GAS2L2	0.953838	0.046162	0.143217685	0.003354	0	2.971410902
GGT5	0.953838	0.046162	0.143217685	0.003354	0	2.971410902
GUCY2F	0.953838	0.046162	0.143217685	0.003354	0	2.971410902
HBE1	0.953838	0.046162	0.143217685	0.003354	0	2.971410902
HHIP	0.953838	0.046162	0.143217685	0.003354	0	2.971410902
HS3ST6	0.953838	0.046162	0.143217685	0.003354	0	2.971410902
ITGA9-AS	0.953838	0.046162	0.143217685	0.003354	0	2.971410902
KRT17P5	0.953838	0.046162	0.143217685	0.003354	0	2.971410902
LCN12	0.953838	0.046162	0.143217685	0.003354	0	2.971410902
LINC0065	0.953838	0.046162	0.143217685	0.003354	0	2.971410902
LINC0120	0.953838	0.046162	0.143217685	0.003354	0	2.971410902
LINC0139	0.953838	0.046162	0.143217685	0.003354	0	2.971410902
LINC0151	0.953838	0.046162	0.143217685	0.003354	0	2.971410902
LINC0154	0.953838	0.046162	0.143217685	0.003354	0	2.971410902
LINC0160	0.953838	0.046162	0.143217685	0.003354	0	2.971410902
LNX1-AS2	0.953838	0.046162	0.143217685	0.003354	0	2.971410902
LOC10192	0.953838	0.046162	0.143217685	0.003354	0	2.971410902
LOC38989	0.953838	0.046162	0.143217685	0.003354	0	2.971410902
LOC40004	0.953838	0.046162	0.143217685	0.003354	0	2.971410902
MARCH10	0.953838	0.046162	0.143217685	0.003354	0	2.971410902
MEPE	0.953838	0.046162	0.143217685	0.003354	0	2.971410902
MIR155HC	0.953838	0.046162	0.143217685	0.003354	0	2.971410902
MIR9-3HG	0.953838	0.046162	0.143217685	0.003354	0	2.971410902
MLC1	0.953838	0.046162	0.143217685	0.003354	0	2.971410902
MUC7	0.953838	0.046162	0.143217685	0.003354	0	2.971410902
MYT1	0.953838	0.046162	0.143217685	0.003354	0	2.971410902
NCAM2	0.953838	0.046162	0.143217685	0.003354	0	2.971410902
NKX6-1	0.953838	0.046162	0.143217685	0.003354	0	2.971410902
NLRP3	0.953838	0.046162	0.143217685	0.003354	0	2.971410902
NME5	0.953838	0.046162	0.143217685	0.003354	0	2.971410902
NTSR1	0.953838	0.046162	0.143217685	0.003354	0	2.971410902
PPP1R1A	0.953838	0.046162	0.143217685	0.003354	0	2.971410902
PPP2R2B	0.953838	0.046162	0.143217685	0.003354	0	2.971410902
PSPN	0.953838	0.046162	0.143217685	0.003354	0	2.971410902
ROBO4	0.953838	0.046162	0.143217685	0.003354	0	2.971410902
S1PR5	0.953838	0.046162	0.143217685	0.003354	0	2.971410902
SARDH	0.953838	0.046162	0.143217685	0.003354	0	2.971410902
SLC15A3	0.953838	0.046162	0.143217685	0.003354	0	2.971410902
SNORA6	0.953838	0.046162	0.143217685	0.003354	0	2.971410902
TMEM30B	0.953838	0.046162	0.143217685	0.003354	0	2.971410902
VGF	0.953838	0.046162	0.143217685	0.003354	0	2.971410902
VWA2	0.953838	0.046162	0.143217685	0.003354	0	2.971410902
WDR38	0.953838	0.046162	0.143217685	0.003354	0	2.971410902
ZNF197-A	0.953838	0.046162	0.143217685	0.003354	0	2.971410902
STK33	0.953851	0.046149	1.309965877	1.310273	641.1095815	489.2923286
TMED10P	0.953883	0.046117	0.467929759	0.453093	8.076971105	17.83837012
CCDC33	0.953892	0.046108	6.965830589	303.8864	3.028864164	0
LOC10012	0.953892	0.046108	6.965830589	303.8864	3.028864164	0
LOC10192	0.953892	0.046108	6.965830589	303.8864	3.028864164	0
LOC64493	0.953892	0.046108	6.965830589	303.8864	3.028864164	0
MRVI1-AS	0.953892	0.046108	6.965830589	303.8864	3.028864164	0
MSMP	0.953892	0.046108	6.965830589	303.8864	3.028864164	0
SIX3	0.953892	0.046108	6.965830589	303.8864	3.028864164	0
SMAD5-AS	0.953892	0.046108	6.965830589	303.8864	3.028864164	0
SNORA21	0.953892	0.046108	6.965830589	303.8864	3.028864164	0
SUMO1P1	0.953892	0.046108	6.965830589	303.8864	3.028864164	0

TEKT5	0.953892	0.046108	6.965830589	303.8864	3.028864164	0
TNNI1	0.953892	0.046108	6.965830589	303.8864	3.028864164	0
ENOX1	0.95395	0.04605	0.519703329	0.509874	12.11545666	23.77128722
PPCDC	0.954079	0.045921	1.353690234	1.35479	212.0204915	156.4943075
MARCH9	0.95409	0.04591	0.762464383	0.762259	428.0794686	561.5966605
KIAA0040	0.954108	0.045892	0.741510268	0.740938	166.587529	224.8367583
JAG2	0.954125	0.045875	0.763033856	0.76297	1382.17168	1811.57018
VIM	0.954168	0.045832	0.727355226	0.727351	25618.1331	35221.1239
DCAF15	0.954298	0.045702	1.307446525	1.307746	668.369359	511.0826752
PDGFRL	0.954334	0.045666	0.756934203	0.756626	290.7709598	384.3024767
FAM27B	0.954339	0.045661	1.658214291	1.671439	41.39447691	24.76175752
KLHL2	0.95438	0.04562	1.305408346	1.305584	1132.795198	867.6519835
PCK2	0.954428	0.045572	0.763427665	0.763363	1351.883039	1770.960898
ZNF470	0.954482	0.045518	0.756881991	0.756571	287.7521918	380.3405955
KCNQ3	0.954494	0.045506	0.697351726	0.695922	71.68311856	103.0089113
QRICH2	0.954494	0.045506	0.697351726	0.695922	71.68311856	103.0089113
CEP19	0.954533	0.045467	0.753174991	0.752803	243.3187545	323.2201733
IFITM10	0.954573	0.045427	1.323409083	1.323995	355.3867286	268.4174515
MELK	0.954594	0.045406	1.325464389	1.32551	4603.87353	3473.282204
PPIP5K1	0.954634	0.045366	0.764996804	0.76484	558.3206277	729.9865164
MIS18A	0.954668	0.045332	1.307535501	1.307821	686.5425439	524.9492594
PROSER3	0.954677	0.045323	1.355682383	1.35684	202.933899	149.5610154
WBP2	0.954692	0.045308	1.318887852	1.31895	3315.596639	2513.813623
TSPAN7	0.954695	0.045305	0.497659365	0.485645	10.09621388	20.79987632
NDUFAF5	0.954729	0.045271	1.331193367	1.331917	296.8286881	222.8558177
SLC39A3	0.954794	0.045206	0.755514927	0.755177	265.5304251	351.6169568
PCGF1	0.954843	0.045157	1.326107659	1.326741	331.1558153	249.5985158
TNFRSF1	0.954877	0.045123	0.582996598	0.577155	20.49531418	35.51826499
TMEM144	0.95498	0.04502	1.337317663	1.338187	258.4630754	193.1417087
SGK3	0.955048	0.044952	1.385450359	1.387227	146.4455824	105.5643247
HAUS6	0.955095	0.044905	1.309121488	1.309229	1828.424334	1396.563124
CLK1	0.955098	0.044902	0.765029429	0.764866	534.0897143	698.281562
KLF2	0.955106	0.044894	0.688640542	0.686978	62.59652607	91.12326767
SH2D5	0.955147	0.044853	0.640376348	0.63715	35.33674859	55.46633684
DNAAF5	0.955176	0.044824	0.766198673	0.76608	736.013992	960.7561918
NGDN	0.955189	0.044811	0.763276444	0.76305	396.7812055	519.9969079
TRIM16	0.955219	0.044781	1.304996177	1.305213	892.0307851	683.4344122
HN1	0.955242	0.044758	1.329685234	1.329721	6059.747572	4557.153854
NDUFB4	0.955268	0.044732	1.310657838	1.310752	2097.993245	1600.600006
MIR3681H	0.955299	0.044701	2.602295312	2.798632	11.10583527	3.961881203
USP11	0.955389	0.044611	0.765713949	0.765629	1029.813816	1345.058668
FKBPL	0.95559	0.04441	1.374931342	1.376506	159.5201793	115.8850252
ALDH6A1	0.955818	0.044182	1.30398256	1.304171	1022.120501	783.7294349
OSR2	0.955949	0.044051	1.457225892	1.46097	86.82743938	59.42821805
IRGM	0.955967	0.044033	0.494600655	0.482221	9.57121076	19.85892953
SLC30A3	0.955972	0.044028	0.41463251	0.392524	5.048106941	12.87611391
WDR78	0.956056	0.043944	0.753246825	0.752851	228.1744337	303.083912
MLLT10P1	0.956128	0.043872	1.915469172	1.952924	23.22129193	11.88564361
ARHGEF1	0.956232	0.043768	0.70961311	0.708377	82.78895383	116.8754955
CCDC34	0.956246	0.043754	1.30653975	1.306893	564.378356	431.8450511
PPT2-EGF	0.956278	0.043722	0.728039167	0.727207	115.7026111	159.1091491
TRAF2	0.956313	0.043687	1.303596018	1.303834	809.7163533	621.0248786
AQP9	0.956327	0.043673	0.14592858	0.003354	0	2.971410902
BEX1	0.956327	0.043673	0.14592858	0.003354	0	2.971410902
C15orf54	0.956327	0.043673	0.14592858	0.003354	0	2.971410902
CYP3A5	0.956327	0.043673	0.14592858	0.003354	0	2.971410902

FAM172BI	0.956327	0.043673	0.14592858	0.003354	0	2.971410902
FSCB	0.956327	0.043673	0.14592858	0.003354	0	2.971410902
HLA-DQB:	0.956327	0.043673	0.14592858	0.003354	0	2.971410902
ITGAM	0.956327	0.043673	0.14592858	0.003354	0	2.971410902
KLF15	0.956327	0.043673	0.14592858	0.003354	0	2.971410902
LOC44145	0.956327	0.043673	0.14592858	0.003354	0	2.971410902
MSANTD1	0.956327	0.043673	0.14592858	0.003354	0	2.971410902
NTRK2	0.956327	0.043673	0.14592858	0.003354	0	2.971410902
SIRPG	0.956327	0.043673	0.14592858	0.003354	0	2.971410902
SLC5A4	0.956327	0.043673	0.14592858	0.003354	0	2.971410902
SNAP25	0.956327	0.043673	0.14592858	0.003354	0	2.971410902
SNCAIP	0.956327	0.043673	0.14592858	0.003354	0	2.971410902
TEKT3	0.956327	0.043673	0.14592858	0.003354	0	2.971410902
TESC	0.956327	0.043673	0.14592858	0.003354	0	2.971410902
UPK1A	0.956327	0.043673	0.14592858	0.003354	0	2.971410902
ZNF814	0.956361	0.043639	1.32905044	1.329818	283.8348609	213.4364451
GTF2IRD2	0.956446	0.043554	0.74906692	0.748596	194.2309627	259.4636
C1QL4	0.956456	0.043544	0.718356257	0.717331	95.91403188	133.7134906
ME1	0.956463	0.043537	1.306366311	1.306481	1704.240903	1304.449386
HSD17B6	0.956521	0.043479	0.692213914	0.690551	63.60614745	92.11373797
SIAE	0.956565	0.043435	0.768057708	0.767937	733.9947492	955.8038402
TFAP2A-A	0.956611	0.043389	1.535039267	1.541931	59.5676619	38.62834173
EPT1	0.956651	0.043349	1.30810293	1.308196	2144.435828	1639.228348
SSR4P1	0.956671	0.043329	0.566163858	0.559134	17.1635636	30.70457932
TMEM161	0.956747	0.043253	1.310534837	1.310986	449.2815177	342.7027241
FKBP5	0.956778	0.043222	1.303409328	1.303671	736.013992	564.5680714
RPH3AL	0.956832	0.043168	0.715485702	0.71436	89.85630355	125.7897282
FAM181B	0.956845	0.043155	0.415113969	0.392524	5.048106941	12.87611391
PHYHIP	0.956845	0.043155	0.415113969	0.392524	5.048106941	12.87611391
SYCE1L	0.956845	0.043155	0.415113969	0.392524	5.048106941	12.87611391
SYNDIG1I	0.956845	0.043155	0.415113969	0.392524	5.048106941	12.87611391
TMEM71	0.956845	0.043155	0.415113969	0.392524	5.048106941	12.87611391
PATZ1	0.956856	0.043144	0.767754042	0.767637	742.0717203	966.6990136
DGCR2	0.956858	0.043142	0.765652254	0.765589	1422.556536	1858.122284
GNB5	0.956879	0.043121	0.767709487	0.767603	812.7452175	1058.812752
CEP152	0.956881	0.043119	1.309756209	1.31017	477.5509166	364.4930707
FLJ23867	0.956902	0.043098	0.75929692	0.758968	276.6362604	364.4930707
ATL2	0.95694	0.04306	1.306535823	1.306642	1833.896482	1403.516226
ADCY10P	0.956968	0.043032	0.646335246	0.64329	36.34636997	56.50633066
SPRED1	0.956988	0.043012	0.766878296	0.766805	1205.911978	1572.648934
PTS	0.956994	0.043006	1.306580596	1.306962	522.9838791	400.1500015
LUADT1	0.957055	0.042945	3.942487371	5.31938	5.522628993	1.030089113
MYZAP	0.957086	0.042914	1.303898778	1.304031	1459.993297	1119.597914
BSN	0.957122	0.042878	1.379563408	1.381306	146.3951013	105.9803222
NME4	0.957152	0.042848	0.763689966	0.763449	364.4733211	477.406685
SLCO1B3	0.957176	0.042824	1.30413468	1.304262	1520.489811	1165.783544
CNIH3	0.957269	0.042731	0.703398284	0.702023	73.70236134	104.9898519
LOC10028	0.957276	0.042724	0.758176295	0.757822	257.453454	339.7313132
ANKRD36	0.957355	0.042645	1.327575425	1.328318	285.2382346	214.7339612
C8orf82	0.957363	0.042637	0.751759182	0.751297	200.9146562	267.4269812
YPEL3	0.957371	0.042629	0.757066499	0.756692	244.3283759	322.8933181
LINC0148	0.957471	0.042529	1.511212875	1.517029	64.61576884	42.59022293
EPS8	0.957497	0.042503	1.301410034	1.301632	861.2070441	661.6341609
APOBEC3	0.957533	0.042467	1.389828275	1.391813	132.4825186	95.1841959
TNFAIP3	0.957544	0.042456	1.309209238	1.30929	2502.851421	1911.60768
RTKN2	0.957552	0.042448	1.323571297	1.324251	306.924902	231.7700504

DPP3	0.957554	0.042446	1.303381966	1.303511	1491.21079	1143.993197
FRRS1	0.957581	0.042419	1.415190093	1.417825	108.7261273	76.68221069
NPTN-IT1	0.957595	0.042405	0.554059193	0.546236	15.14432082	27.73316842
ZNF233	0.957633	0.042367	0.611387201	0.606842	25.2405347	41.59975263
SMPD1	0.957648	0.042352	0.712177889	0.71099	83.79857522	117.8659658
PSMG2	0.957685	0.042315	1.30222695	1.302371	1328.661747	1020.18441
SPIN2A	0.957697	0.042303	6.707311264	290.7613	2.897613384	0
LINC0152	0.957701	0.042299	0.733957271	0.733202	125.6978628	171.4405044
ZNF728	0.957714	0.042286	0.148035799	0.003411	0	2.921887387
FKBP1A-S	0.957775	0.042225	0.65785543	0.655168	40.5867798	61.95391731
CACNA2D	0.9578	0.0422	1.332122887	1.33296	257.453454	193.1417087
FAM24B-C	0.957882	0.042118	0.538620134	0.529503	13.32700232	25.17775505
ATAD5	0.957903	0.042097	1.300842843	1.301072	829.9087811	637.8628737
ACCS	0.957949	0.042051	0.710365839	0.709128	80.76971105	113.9040846
ZNF781	0.957966	0.042034	1.331683103	1.332551	253.4149684	190.1702977
ALKBH7	0.957973	0.042027	0.76522682	0.765003	388.7042344	508.1112643
BLOC1S1	0.957976	0.042024	1.300798584	1.301007	911.0621482	700.2724074
BCL6	0.958004	0.041996	0.768550072	0.768405	596.6862404	776.5287158
METTL20	0.95801	0.04199	1.36240339	1.363822	169.5457197	124.3139275
STAG3L2	0.958027	0.041973	1.322403925	1.323109	301.260926	227.6893127
DPP8	0.958041	0.041959	0.769466487	0.769351	761.2545267	989.4798305
LNX1	0.958165	0.041835	1.988025796	2.037623	20.19242776	9.904703008
CD274	0.958179	0.041821	1.491229799	1.496364	69.66387578	46.55210414
FAAP20	0.958187	0.041813	0.768026934	0.767857	510.5251511	664.8729988
FAT4	0.958192	0.041808	0.58837502	0.582598	20.19242776	34.66646053
NUCB2	0.958293	0.041707	1.302305012	1.302433	1500.297383	1151.91696
AGAP7P	0.958299	0.041701	1.692006465	1.708354	35.18530538	20.59187755
ZNF665	0.958346	0.041654	1.331230335	1.332104	251.4563029	188.7638299
EPHB2	0.958387	0.041613	0.768141837	0.768062	1089.381478	1418.353471
SMIM2-AS	0.958419	0.041581	2.097304089	2.164618	17.1635636	7.923762406
LINC0095	0.958474	0.041526	0.689310529	0.687495	58.55804051	85.18044587
TUBB2B	0.958518	0.041482	0.756527662	0.756144	232.4754208	307.4518861
HOXB5	0.958554	0.041446	0.768681619	0.768499	485.6278877	631.9200519
WDR93	0.958598	0.041402	0.462515994	0.44603	7.067349717	15.85742952
TMEM242	0.958606	0.041394	1.301414186	1.301742	595.676619	457.597279
MMP28	0.958681	0.041319	1.318031108	1.318651	329.1365725	249.5985158
C6orf52	0.958716	0.041284	0.462796281	0.446309	7.067349717	15.84752481
DCN	0.958716	0.041284	0.462796281	0.446309	7.067349717	15.84752481
FAM186B	0.958716	0.041284	0.462796281	0.446309	7.067349717	15.84752481
MRPS36	0.958797	0.041203	1.301273424	1.301606	586.5900265	450.6639869
EPB41L4A	0.958805	0.041195	0.766212693	0.765991	390.7234772	510.0922049
ZNF664-F	0.958854	0.041146	0.453076776	0.435138	6.592827665	15.1641003
GSAP	0.958854	0.041146	0.635642226	0.632062	31.29826303	49.52351504
SLC9A5	0.958854	0.041146	0.635642226	0.632062	31.29826303	49.52351504
EVA1B	0.958871	0.041129	0.756258654	0.755842	220.0974626	291.1982684
ZNF48	0.958878	0.041122	0.769480154	0.769337	602.7439687	783.4620079
RAD21-AS	0.958883	0.041117	2.269955413	2.376134	14.13469943	5.942821805
IL17RC	0.958984	0.041016	0.73903677	0.738344	135.289266	183.2370056
GLIDR	0.959008	0.040992	0.729767981	0.728902	110.7453701	151.9381441
PPM1H	0.959084	0.040916	0.770481241	0.770349	665.3404948	863.6901023
ANGPTL4	0.95909	0.04091	0.754835431	0.754391	206.9723846	274.3602733
ECSIT	0.959109	0.040891	1.299651157	1.299956	636.0614745	489.2923286
NOVA1	0.95913	0.04087	0.677153121	0.67501	49.47144802	73.29480226
PGM2	0.959149	0.040851	1.298825404	1.298973	1308.469319	1007.308296
ARHGAP3	0.959201	0.040799	0.520551534	0.509893	11.10583527	21.79034662
MPPE1	0.959261	0.040739	1.301231351	1.301583	555.2008976	426.5559397

FBXO2	0.959272	0.040728	0.764178636	0.763905	320.04998	418.9689372
FAM227B	0.959347	0.040653	0.520595329	0.509893	11.10583527	21.79034662
LOC55310	0.959347	0.040653	0.520595329	0.509893	11.10583527	21.79034662
SCAND1	0.959391	0.040609	0.770738584	0.770601	638.0807173	828.0331714
DGCR5	0.959408	0.040592	0.463157852	0.446309	7.067349717	15.84752481
SLC23A3	0.959408	0.040592	0.463157852	0.446309	7.067349717	15.84752481
SCFD1	0.959544	0.040456	0.769695017	0.769608	989.4289604	1285.63045
TCF7	0.959554	0.040446	0.736808399	0.736061	126.2026735	171.4603138
LOC72908	0.959558	0.040442	1.411069275	1.413796	106.0506306	75.00831588
GAS6	0.959572	0.040428	0.768977959	0.768903	1146.929897	1491.648273
U2AF2	0.959732	0.040268	0.762484594	0.762448	2436.21641	3195.25719
MARVELD	0.959795	0.040205	0.646930136	0.643659	34.56943633	53.71320441
BMPR1A	0.9598	0.0402	1.305851724	1.305934	2355.446699	1803.646418
C9orf163	0.959858	0.040142	0.520832001	0.509893	11.10583527	21.79034662
CECR1	0.959858	0.040142	0.520832001	0.509893	11.10583527	21.79034662
RHBDL2	0.959917	0.040083	1.550681355	1.558818	52.50031218	33.67599023
JHDM1D-A	0.959975	0.040025	0.693042715	0.691309	59.5676619	86.17091617
OLMALIN(	0.960013	0.039987	0.682131597	0.680043	51.51088322	75.7511686
ARHGEF3	0.960043	0.039957	1.298037274	1.298187	1254.959385	966.6990136
HOXA1	0.960079	0.039921	1.418371793	1.421267	99.95251743	70.32339135
MIR31HG	0.960087	0.039913	0.766422283	0.766172	348.3193789	454.6258681
C1orf115	0.960093	0.039907	1.296887287	1.297083	957.121076	737.9003741
AQP7	0.960116	0.039884	2.76326318	3.051103	9.086592493	2.971410902
HS1BP3-IT	0.960116	0.039884	2.76326318	3.051103	9.086592493	2.971410902
LOC10192	0.960116	0.039884	2.76326318	3.051103	9.086592493	2.971410902
AKAP8L	0.960146	0.039854	0.77028823	0.770201	989.4289604	1284.63998
PRR5-ARI	0.960174	0.039826	2.38322065	2.522212	12.20632258	4.833495068
FHIT	0.96019	0.03981	0.620765002	0.616432	26.25015609	42.59022293
LOC10050	0.960191	0.039809	0.542045851	0.532801	13.12507805	24.64290108
LIMK2	0.960195	0.039805	0.770676215	0.770524	565.4081698	733.799827
LDAH	0.9602	0.0398	1.297558225	1.297808	748.1294486	576.4537151
ATPAF2	0.960243	0.039757	0.732689793	0.731848	113.0775955	154.5133669
FAM8A1	0.96025	0.03975	0.768099832	0.768042	1533.614889	1996.788126
CBX8	0.960252	0.039748	0.715624828	0.714431	82.78895383	115.8850252
ANKRD49	0.960281	0.039719	1.305756971	1.306205	432.1179541	330.8170805
CD9	0.96031	0.03969	1.301551248	1.301654	1852.655247	1423.305822
B3GALT6	0.960319	0.039681	0.766456316	0.766202	342.2616506	446.7021056
WDR4	0.960322	0.039678	1.307699752	1.308192	397.7908269	304.0743823
ZNF677	0.960324	0.039676	0.747577645	0.746982	157.662476	211.0692211
LRRC23	0.960423	0.039577	1.363438166	1.364991	155.4816938	113.9040846
ATE1-AS1	0.960471	0.039529	0.398106772	0.371244	4.038485553	10.89517331
CTNND2	0.960471	0.039529	0.398106772	0.371244	4.038485553	10.89517331
PIK3CD-A	0.960471	0.039529	0.398106772	0.371244	4.038485553	10.89517331
MAP2K3	0.960484	0.039516	1.296759775	1.297011	745.1005845	574.4727744
EIF2B5-AS	0.960485	0.039515	0.320665561	0.275739	2.019242776	7.349289632
ZBTB37	0.960538	0.039462	1.489449941	1.494914	66.63501162	44.57116353
CFAP44	0.960564	0.039436	0.731855395	0.730989	110.0487313	150.5514857
ASB16-AS	0.960578	0.039422	0.748997894	0.748442	164.5682863	219.8844068
PIM3	0.960614	0.039386	1.299963805	1.300076	1697.173553	1305.439856
PPARGC1	0.960656	0.039344	1.308084244	1.308596	383.6561275	293.179209
MIA3	0.960671	0.039329	0.769153636	0.769088	1312.507805	1706.580328
MTIF2	0.960676	0.039324	1.304292287	1.304375	2324.289783	1781.915499
CDK5RAP	0.96074	0.03926	1.306183548	1.306258	2619.967502	2005.702359
MCM3	0.960768	0.039232	0.763214966	0.763179	2475.591644	3243.790235
ALDOC	0.960774	0.039226	0.748432709	0.747842	158.5105579	211.9606444
ZNF671	0.960833	0.039167	0.671526351	0.669065	44.42334108	66.40112896

CRYM-AS	0.960854	0.039146	2.171317801	2.258127	15.15441704	6.705483936
C6orf106	0.960894	0.039106	1.32693748	1.326967	7168.311856	5402.02502
CTXN1	0.960937	0.039063	0.767632472	0.76737	338.223165	440.7592838
APBA1	0.960977	0.039023	0.643092365	0.639654	32.30788442	50.51398534
LINC-PINT	0.960977	0.039023	0.643092365	0.639654	32.30788442	50.51398534
SPSB2	0.960979	0.039021	1.307477146	1.308017	370.5310495	283.274506
RBCK1	0.960998	0.039002	0.753718024	0.753701	5336.858658	7080.87218
AVPI1	0.961012	0.038988	1.297451966	1.297584	1426.595021	1099.422034
LOC10013	0.961031	0.038969	0.600159161	0.594726	21.20204915	35.65693083
CTIF	0.961039	0.038961	0.7713703	0.77128	948.0344835	1229.173643
SDHAP3	0.961106	0.038894	0.63477156	0.630993	29.55161803	46.83934052
LOC10192	0.961107	0.038893	2.757684143	3.051103	9.086592493	2.971410902
SCARA5	0.961107	0.038893	2.757684143	3.051103	9.086592493	2.971410902
NKX2-8	0.961196	0.038804	0.569830479	0.562544	16.15394221	28.72363872
CCS	0.961269	0.038731	0.762608677	0.762267	257.453454	337.7503726
NUDT4P1	0.961334	0.038666	1.295454996	1.295613	1181.570007	911.9755294
POLR3GL	0.961338	0.038662	1.298287695	1.298631	549.2340352	422.9308184
THEG	0.961344	0.038656	0.398687825	0.371244	4.038485553	10.89517331
RILPL2	0.961419	0.038581	1.313534708	1.314171	315.0018731	239.6938128
STX18-AS	0.961439	0.038561	1.498647416	1.504612	62.59652607	41.59975263
ZNF775	0.961458	0.038542	0.707952219	0.706499	70.67349717	100.0375004
ZNF137P	0.961586	0.038414	1.327197215	1.328085	238.1091082	179.2850291
TTC31	0.961654	0.038346	1.294959838	1.295213	729.9562636	563.5776011
MX1	0.961658	0.038342	0.747796653	0.747195	152.4528296	204.036882
PIGA	0.96168	0.03832	0.754800002	0.754318	186.7799568	247.6175752
AK4	0.961684	0.038316	0.761032385	0.761005	3185.35548	4185.727491
PTER	0.961718	0.038282	1.294082954	1.294267	1007.602145	778.5096564
WASH5P	0.961766	0.038234	0.716484007	0.715257	80.42643978	112.4480932
SLC15A2	0.961785	0.038215	0.400458644	0.373096	4.05867798	10.89517331
F2RL1	0.961806	0.038194	0.772614614	0.772511	827.8895383	1071.688865
GAS6-AS2	0.961838	0.038162	0.526902426	0.516219	11.11593148	21.54272904
ZNF98	0.961875	0.038125	1.408313519	1.411136	101.5881041	71.98738146
OAF	0.961875	0.038125	1.307610938	1.307674	3122.758954	2388.023895
EIF4EBP3	0.961878	0.038122	6.345956504	266.5304	2.655304251	0
RNF26	0.961878	0.038122	1.304163312	1.304238	2583.621132	1980.940602
NCAPD2	0.96188	0.03812	1.303792837	1.303869	2529.101577	1939.687514
SDHAF4	0.96191	0.03809	1.331503196	1.332484	219.0878412	164.4180699
LINC0093	0.96193	0.03807	1.904757368	1.945136	21.20204915	10.89517331
IFNLR1	0.961981	0.038019	0.641862766	0.638295	31.17710847	48.84999523
EPC2	0.962	0.038	0.772675485	0.772523	559.330249	724.0337899
MGC2734	0.962036	0.037964	0.700894181	0.699278	62.99027841	90.08327386
ACAP3	0.962079	0.037921	0.764108022	0.763774	262.5015609	343.6931944
DDX17	0.962103	0.037897	0.749648595	0.749637	7649.901258	10204.81551
GLIPR1L2	0.962151	0.037849	1.494892759	1.500669	62.56623742	41.68889496
CLDN4	0.962152	0.037848	1.29345085	1.293626	1057.073593	817.1379981
HRH4	0.962295	0.037705	2.494614661	2.678215	10.60102458	3.9519765
DXO	0.962331	0.037669	1.300098859	1.300523	448.2718963	344.6836647
DLG1-AS1	0.962373	0.037627	1.55201489	1.56068	49.47144802	31.69504962
AFTPH	0.962422	0.037578	1.304212407	1.304283	2721.939262	2086.920924
ETNK2	0.962436	0.037564	0.770027783	0.769793	367.5021853	477.406685
HDDC2	0.962447	0.037553	1.297756066	1.29786	1811.26077	1395.572654
PROC	0.962462	0.037538	0.55740931	0.549048	14.13469943	25.75222782
LINC0124	0.962526	0.037474	0.407197544	0.380649	4.220217403	11.10317207
PFKM	0.962593	0.037407	0.768399956	0.76813	321.0596014	417.9784669
C2CD2L	0.962605	0.037395	1.305814767	1.306357	358.4155928	274.3602733
ZWINT	0.962614	0.037386	1.293260114	1.293399	1357.940767	1049.898519

PTPRD	0.962632	0.037368	0.629813794	0.625587	27.25977748	43.58069323
SLC44A5	0.962634	0.037366	0.593048792	0.587016	19.18280638	32.68551993
NFXL1	0.962635	0.037365	1.291628465	1.291803	1072.217914	830.014112
MED30	0.962666	0.037334	1.29743072	1.297806	498.7529658	384.3024767
WDFY2	0.962682	0.037318	0.764860229	0.764509	254.4245898	332.7980211
ZFPM1	0.962709	0.037291	0.744091696	0.743397	133.2700232	179.2751244
MTHFD2L	0.962737	0.037263	1.322107959	1.32295	246.3476187	186.2084165
ADAM22	0.962774	0.037226	0.755567649	0.755073	181.7318499	240.6842831
APOLD1	0.962798	0.037202	1.487756517	1.493329	63.60614745	42.59022293
WNK4	0.962838	0.037162	1.417648047	1.420827	92.88516771	65.37103985
NQO1	0.962841	0.037159	1.334344706	1.334365	10941.25689	8199.59848
HSD11B2	0.962853	0.037147	0.557594843	0.549048	14.13469943	25.75222782
LOC90246	0.962868	0.037132	0.665364817	0.662625	39.37523414	59.42821805
SLC47A2	0.962868	0.037132	0.665364817	0.662625	39.37523414	59.42821805
DPH3	0.96287	0.03713	0.768600057	0.768311	305.9152806	398.1690609
SUSD3	0.962892	0.037108	0.640919177	0.637161	30.28864164	47.54257444
LINC0089	0.962902	0.037098	0.699966882	0.698288	60.70853407	86.943483
RCC1	0.962992	0.037008	1.307952685	1.308008	3641.229825	2783.796018
NDUFV2	0.963026	0.036974	1.302121894	1.302197	2556.361355	1963.112136
DGAT2	0.96303	0.03697	1.34482515	1.346118	174.6645002	129.7516094
DPYSL5	0.963087	0.036913	0.772887876	0.7727	454.8142429	588.6067856
NT5DC2	0.96321	0.03679	0.755116314	0.754584	172.6452574	228.7986395
LOC64377	0.963221	0.036779	3.775961119	5.12637	5.118780438	0.990470301
VAMP5	0.963311	0.036689	1.983851285	2.037507	18.17318499	8.914232707
TMEM133	0.963324	0.036676	0.733141042	0.732218	102.9813816	140.6467827
DDX60L	0.963338	0.036662	1.29249708	1.29264	1289.286513	997.4035929
TPRA1	0.963342	0.036658	1.313357748	1.313401	4622.046715	3519.140979
ZBTB7C	0.963372	0.036628	1.318910664	1.319754	248.3668615	188.1893571
CENPJ	0.963377	0.036623	1.293410253	1.293765	533.0800929	412.0356451
USP44	0.963415	0.036585	0.773475413	0.773293	466.4450813	603.1964132
F12	0.963482	0.036518	1.595919681	1.607178	41.39447691	25.75222782
ADCY5	0.963557	0.036443	0.456331918	0.437264	6.057728329	13.86658421
HHIP-AS1	0.963557	0.036443	0.456331918	0.437264	6.057728329	13.86658421
ALOXE3	0.963584	0.036416	3.052929522	3.554777	7.067349717	1.980940602
CELA1	0.963584	0.036416	3.052929522	3.554777	7.067349717	1.980940602
FLJ27354	0.963584	0.036416	3.052929522	3.554777	7.067349717	1.980940602
HP09053	0.963584	0.036416	3.052929522	3.554777	7.067349717	1.980940602
LAT2	0.963584	0.036416	3.052929522	3.554777	7.067349717	1.980940602
MRC1	0.963584	0.036416	3.052929522	3.554777	7.067349717	1.980940602
OR7E91P	0.963584	0.036416	3.052929522	3.554777	7.067349717	1.980940602
EIF2B3	0.963609	0.036391	1.291679992	1.291966	641.1095815	496.2256207
ANKRD31	0.963631	0.036369	2.314561461	2.44349	12.11545666	4.952351504
GTSE1-AS	0.963631	0.036369	2.314561461	2.44349	12.11545666	4.952351504
PYGM	0.963631	0.036369	2.314561461	2.44349	12.11545666	4.952351504
RASAL2-A	0.963631	0.036369	2.314561461	2.44349	12.11545666	4.952351504
EMG1	0.963648	0.036352	1.30929435	1.309941	304.9056592	232.7605207
HAUS7	0.963714	0.036286	1.301958278	1.302473	371.5406708	285.2554466
DOCK9-AS	0.963849	0.036151	1.982701808	2.037507	18.17318499	8.914232707
LOC10050	0.963849	0.036151	1.982701808	2.037507	18.17318499	8.914232707
OAS3	0.963881	0.036119	1.291738429	1.291877	1325.632883	1026.127232
C11orf1	0.963887	0.036113	0.749540249	0.748912	145.3854799	194.132179
VCAN	0.963889	0.036111	0.751971188	0.751959	7217.783304	9598.647685
ANXA9	0.963916	0.036084	1.397231202	1.399833	103.991003	74.28527256
ZSWIM6	0.963931	0.036069	1.289334752	1.289503	1103.516177	855.7663399
MPZL3	0.964019	0.035981	1.304649029	1.305256	326.0774197	249.8164193
SSPN	0.96402	0.03598	0.68191985	0.679604	46.44258386	68.34245075

LOC55338	0.96402	0.03598	0.521614746	0.509914	10.13659874	19.88864364
FKRP	0.964033	0.035967	0.769719251	0.769439	307.9345234	400.2094297
PRICKLE2	0.964034	0.035966	0.754603258	0.75404	163.5586649	216.9129959
TMEM171	0.964069	0.035931	1.320232701	1.321132	234.2321621	177.2941838
RNF39	0.964091	0.035909	1.477230547	1.482559	64.61576884	43.58069323
ERICH6	0.964094	0.035906	3.732178911	5.055729	5.048106941	0.990470301
INHBA-AS	0.964094	0.035906	3.732178911	5.055729	5.048106941	0.990470301
SNORA40	0.964094	0.035906	3.732178911	5.055729	5.048106941	0.990470301
ZNF235	0.964097	0.035903	0.701054633	0.699347	59.5676619	85.18044587
FAM92A1I	0.964104	0.035896	6.163015026	257.4438	2.564438326	0
EDNRA	0.964132	0.035868	0.744286227	0.74353	125.1930521	168.3799511
ADAM28	0.964152	0.035848	0.374623907	0.340518	3.028864164	8.914232707
ARID3C	0.964152	0.035848	0.374623907	0.340518	3.028864164	8.914232707
CHN2	0.964152	0.035848	0.374623907	0.340518	3.028864164	8.914232707
CHP2	0.964152	0.035848	0.374623907	0.340518	3.028864164	8.914232707
HS6ST3	0.964152	0.035848	0.374623907	0.340518	3.028864164	8.914232707
IL17RE	0.964152	0.035848	0.374623907	0.340518	3.028864164	8.914232707
PPP5D1	0.964152	0.035848	0.374623907	0.340518	3.028864164	8.914232707
RYR1	0.964152	0.035848	0.374623907	0.340518	3.028864164	8.914232707
ZPLD1	0.964152	0.035848	0.374623907	0.340518	3.028864164	8.914232707
C1orf168	0.964205	0.035795	0.521661365	0.509915	10.09621388	19.80940602
MIB2	0.96421	0.03579	0.77488877	0.774798	930.8709199	1201.440475
HRCT1	0.964215	0.035785	0.494536476	0.479996	8.076971105	16.83799511
KDM1B	0.96422	0.03578	1.289123906	1.289325	901.5918996	699.2720323
TNFAIP2	0.964281	0.035719	0.744105175	0.744098	12804.01844	17207.44054
HNRNPA1	0.964281	0.035719	0.494313396	0.479714	8.076971105	16.84789982
SNORA18	0.964366	0.035634	2.311911163	2.44349	12.11545666	4.952351504
STAT4	0.964368	0.035632	1.717239847	1.738428	29.27902026	16.83799511
TMCC2	0.964374	0.035626	0.698019157	0.696169	56.53879774	81.21856466
MNX1-AS1	0.964376	0.035624	0.494595838	0.479996	8.076971105	16.83799511
PAN3-AS1	0.964376	0.035624	0.494595838	0.479996	8.076971105	16.83799511
LOC10361	0.96445	0.03555	0.697970952	0.696169	56.53879774	81.21856466
SVBP	0.964454	0.035546	1.293571591	1.293981	461.3969744	356.5693083
C10orf111	0.964463	0.035537	3.728438798	5.055729	5.048106941	0.990470301
HIST1H4C	0.964463	0.035537	3.728438798	5.055729	5.048106941	0.990470301
IQCF1	0.964463	0.035537	3.728438798	5.055729	5.048106941	0.990470301
LAG3	0.964463	0.035537	3.728438798	5.055729	5.048106941	0.990470301
LOC10099	0.964463	0.035537	3.728438798	5.055729	5.048106941	0.990470301
PNLDC1	0.964463	0.035537	3.728438798	5.055729	5.048106941	0.990470301
RHOXF1	0.964463	0.035537	3.728438798	5.055729	5.048106941	0.990470301
SFTPB	0.964463	0.035537	3.728438798	5.055729	5.048106941	0.990470301
TM4SF4	0.964463	0.035537	3.728438798	5.055729	5.048106941	0.990470301
SLC41A2	0.964494	0.035506	1.288744153	1.288899	1192.362859	925.0992609
WNT3A	0.964495	0.035505	0.63828401	0.634335	28.26939887	44.57116353
TRABD	0.964543	0.035457	0.776176715	0.776077	863.2262869	1112.298148
EIF3G	0.964559	0.035441	0.768424282	0.768384	2148.474314	2796.097659
EXOSC4	0.964605	0.035395	0.761094198	0.76065	198.8954135	261.4841594
PCDHB14	0.964633	0.035367	0.698302294	0.696446	56.32677725	80.88180476
USP33	0.964637	0.035363	1.296345458	1.296433	2130.533342	1643.378418
LOC10013	0.964647	0.035353	0.657157136	0.653979	34.3271272	52.49492594
TPRG1L	0.964649	0.035351	1.289473148	1.289611	1348.854175	1045.936638
ITGB7	0.964654	0.035346	1.381108501	1.383338	115.0968383	83.19950527
MITF	0.964747	0.035253	1.288672236	1.288839	1086.352614	842.890226
GDF11	0.964757	0.035243	1.290312358	1.290662	533.0800929	413.0261154
GRAMD4	0.964774	0.035226	0.775902546	0.775797	797.6008966	1028.108172
COX17	0.964775	0.035225	1.288257069	1.288549	633.0326104	491.2732692

NEK11	0.964903	0.035097	0.76784197	0.767506	258.4630754	336.7599023
ENKUR	0.964935	0.035065	0.494916581	0.479996	8.076971105	16.83799511
SCLY	0.96495	0.03505	0.752126976	0.751516	148.4143441	197.4898733
NTN4	0.965004	0.034996	1.329489662	1.32951	10247.65709	7707.839881
SQRDL	0.965036	0.034964	0.777090041	0.776978	770.3411192	991.4607711
LOC10050	0.965047	0.034953	0.375354561	0.340518	3.028864164	8.914232707
TMEM255	0.965047	0.034953	0.375354561	0.340518	3.028864164	8.914232707
ZCCHC18	0.965047	0.034953	0.375354561	0.340518	3.028864164	8.914232707
TPST1	0.965049	0.034951	0.76909591	0.768779	272.5977748	354.5883677
HPS5	0.965111	0.034889	1.294649883	1.294742	2017.223534	1558.009783
PFDN4	0.965114	0.034886	1.287870348	1.288019	1243.85355	965.7085433
AIM1	0.965136	0.034864	1.3486048	1.350141	152.4528296	112.9136143
BOLA3	0.96517	0.03483	1.291660752	1.292033	492.6952374	381.3310658
SH2B3	0.965175	0.034825	0.776779023	0.77666	708.7542145	912.5698116
ZNF569	0.965197	0.034803	1.286454864	1.286635	1015.679116	789.4048297
CEP83	0.965198	0.034802	1.286237762	1.28643	953.0825904	740.871785
SHROOM	0.965199	0.034801	0.768083484	0.767727	248.6596517	323.8936931
DRAM2	0.965262	0.034738	1.297004221	1.297083	2371.600641	1828.408175
MGC7208	0.96528	0.03472	1.350199293	1.351731	150.3831058	111.2496242
PART1	0.965286	0.034714	6.061380872	252.3957	2.513957257	0
SRSF8	0.965293	0.034707	0.775646359	0.775463	459.0748452	592.0040988
COMMD1(	0.965317	0.034683	1.290403764	1.290524	1513.422461	1172.716836
PTPN4	0.965344	0.034656	1.28705761	1.2873	742.0717203	576.4537151
RAP1GAP	0.965382	0.034618	0.721362851	0.720107	77.74084689	107.9612628
MARCH1	0.965396	0.034604	1.335216699	1.336437	178.7029857	133.7134906
FBXL14	0.965408	0.034592	0.767263932	0.766905	242.3091332	315.9600259
CGB7	0.965431	0.034569	0.428505337	0.404192	4.613969744	11.43002727
B4GALT3	0.965468	0.034532	1.289672574	1.290013	534.0897143	414.0165857
CTSL	0.965472	0.034528	1.306271387	1.306322	3855.744081	2951.601496
FUT3	0.965493	0.034507	0.381060817	0.347306	3.089441448	8.914232707
ORC6	0.965496	0.034504	1.28548683	1.285693	886.7504652	689.7040892
CEBPB	0.965501	0.034499	0.771886294	0.771836	1700.202418	2202.805949
GLYCTK	0.965504	0.034496	1.451571904	1.456099	70.67349717	48.53304474
CENPC	0.965562	0.034438	1.298122967	1.298644	361.444457	278.3221545
ADAMTS4	0.965603	0.034397	0.335505624	0.289583	2.02933899	7.032339135
CEACAM8	0.965604	0.034396	0.329701259	0.281926	1.958665493	6.972910917
FAM151B	0.965611	0.034389	0.574205721	0.56646	15.14432082	26.74269812
UNKL	0.965618	0.034382	1.286942353	1.287209	674.4472797	523.9587891
OIP5-AS1	0.965691	0.034309	0.777256618	0.777106	560.3398704	721.062379
CRTC3-AS	0.965715	0.034285	3.708391163	5.055729	5.048106941	0.990470301
C16orf74	0.965746	0.034254	1.288614923	1.288944	550.2436565	426.8926996
TATDN3	0.965759	0.034241	1.296830263	1.297365	358.4155928	276.2619763
CA11	0.965777	0.034223	0.749707823	0.749028	134.2796446	179.2751244
KCNE3	0.965779	0.034221	0.621690307	0.6168	23.21119571	37.63787143
BROX	0.965799	0.034201	1.292849798	1.292944	1946.550036	1505.514857
SETD4	0.965837	0.034163	1.2864924	1.28676	670.3886017	520.9873782
PCSK4	0.965899	0.034101	0.698715735	0.6968	54.51955496	78.24715376
SETD9	0.965948	0.034052	1.305905294	1.306589	284.7132315	217.9034662
C19orf33	0.965993	0.034007	1.293313168	1.293403	2053.569904	1587.723892
AGO2	0.965999	0.034001	1.28449864	1.284696	923.8035702	719.0814384
COPS2	0.966013	0.033987	0.769648669	0.76961	2206.022733	2866.42105
NHSL1	0.966015	0.033985	1.289881406	1.290256	485.6278877	376.3787143
EPM2A	0.966015	0.033985	1.324737823	1.325781	200.9146562	151.541956
LINC0122	0.966031	0.033969	1.294440169	1.294911	393.7523414	304.0743823
SIRT2	0.966051	0.033949	0.77834846	0.778237	772.3603619	992.4512414
DLK2	0.966084	0.033916	0.755110015	0.75452	152.4528296	202.0559414

HSPA1A	0.966089	0.033911	0.771944387	0.771896	1772.087461	2295.761587
LTBP4	0.966096	0.033904	0.775148514	0.775085	1368.036981	1765.018076
HYI	0.966118	0.033882	1.298885965	1.299446	337.2135436	259.5032188
ZNF670-Z	0.96615	0.03385	1.847390342	1.884417	21.4544545	11.38050376
MIR548XH	0.966156	0.033844	1.474575394	1.480112	61.73834789	41.70870437
ASUN	0.966181	0.033819	1.297217988	1.297289	2634.102202	2030.464117
BTBD19	0.966181	0.033819	0.635370731	0.631106	26.25015609	41.59975263
AACS	0.966198	0.033802	0.775866165	0.775632	369.5214281	476.4162147
ABCC9	0.966207	0.033793	1.288076144	1.288194	1560.642453	1211.493748
NCAPG2	0.966218	0.033782	1.288031777	1.28815	1557.845802	1209.364237
RUNX2	0.966225	0.033775	0.775803433	0.775586	388.7042344	501.1779722
STARD7-A	0.966234	0.033766	0.708283932	0.706618	61.58690468	87.16138647
ATP8A1	0.966254	0.033746	0.775584774	0.775361	378.6080206	488.3018583
CCDC170	0.966327	0.033673	0.655826496	0.652445	32.30788442	49.52351504
ARHGEF5	0.966364	0.033636	1.28479393	1.285032	749.13907	582.9710096
IL22RA1	0.966392	0.033608	1.679949134	1.6985	30.28864164	17.82846541
GPRIN1	0.9664	0.0336	0.770690198	0.770366	265.5304251	344.6836647
ELOVL4	0.966425	0.033575	0.776766154	0.776545	390.7234772	503.1589128
LEFTY1	0.966434	0.033566	0.33843804	0.292259	2.019242776	6.933292105
SEC14L1P	0.966434	0.033566	0.33843804	0.292259	2.019242776	6.933292105
EDN1	0.966441	0.033559	1.319385268	1.319413	7448.986602	5645.680714
FDX1L	0.966489	0.033511	0.765824589	0.765417	213.0301129	278.3221545
VPS9D1	0.96649	0.03351	0.771776882	0.77147	279.6651245	362.5121301
NCK1-AS1	0.966558	0.033442	1.443013014	1.447366	71.68311856	49.52351504
TRPC7-AS	0.966567	0.033433	3.653346205	4.947673	5.038010727	1.010279707
HMHA1	0.966571	0.033429	1.295196692	1.295737	352.0751705	271.7157176
ARHGEF2	0.966587	0.033413	0.774673525	0.77442	335.1943009	432.8355214
BLNK	0.966606	0.033394	0.276166123	0.205471	1.009621388	4.952351504
CXorf57	0.966606	0.033394	0.276166123	0.205471	1.009621388	4.952351504
LINC0046	0.966606	0.033394	0.276166123	0.205471	1.009621388	4.952351504
SLC16A6	0.966606	0.033394	0.276166123	0.205471	1.009621388	4.952351504
TMEM74	0.966606	0.033394	0.276166123	0.205471	1.009621388	4.952351504
TNRC18P	0.966606	0.033394	0.276166123	0.205471	1.009621388	4.952351504
HNRNPKP	0.966613	0.033387	0.69130841	0.689172	48.46182663	70.32339135
PARS2	0.966641	0.033359	0.76748829	0.767101	224.1359482	292.1887387
BEST1	0.96665	0.03335	1.41992384	1.423481	81.77933244	57.44727744
SDC1	0.966676	0.033324	0.765799625	0.765772	3195.451694	4172.851377
CARD14	0.966716	0.033284	0.338619307	0.292259	2.019242776	6.933292105
DUOXA1	0.966716	0.033284	0.338619307	0.292259	2.019242776	6.933292105
HIST1H2B	0.966716	0.033284	0.338619307	0.292259	2.019242776	6.933292105
KLKB1	0.966716	0.033284	0.338619307	0.292259	2.019242776	6.933292105
POU2F3	0.966716	0.033284	0.338619307	0.292259	2.019242776	6.933292105
SGCG	0.966716	0.033284	0.338619307	0.292259	2.019242776	6.933292105
TLX2	0.966716	0.033284	0.338619307	0.292259	2.019242776	6.933292105
TMEM52	0.966721	0.033279	0.598342929	0.592005	18.17318499	30.70457932
SAMD10	0.966797	0.033203	0.598369136	0.592005	18.17318499	30.70457932
FLT4	0.966842	0.033158	0.561306353	0.552328	13.12507805	23.77128722
RHBDL1	0.966842	0.033158	0.561306353	0.552328	13.12507805	23.77128722
ZNF454	0.966842	0.033158	0.561306353	0.552328	13.12507805	23.77128722
C3orf70	0.966854	0.033146	0.342649913	0.297348	2.089916273	7.052148542
ZNF717	0.966882	0.033118	1.304416955	1.305116	276.6362604	211.9606444
TP53BP1	0.96691	0.03309	0.770097784	0.77006	2307.994493	2997.16313
RPS6KA5	0.966912	0.033088	1.306837865	1.307617	256.4438326	196.1131196
ATP1B2	0.96695	0.03305	0.276436552	0.205471	1.009621388	4.952351504
CDHR2	0.96695	0.03305	0.276436552	0.205471	1.009621388	4.952351504
COL11A1	0.96695	0.03305	0.276436552	0.205471	1.009621388	4.952351504

EFHB	0.96695	0.03305	0.276436552	0.205471	1.009621388	4.952351504
ERVH48-1	0.96695	0.03305	0.276436552	0.205471	1.009621388	4.952351504
FLT3	0.96695	0.03305	0.276436552	0.205471	1.009621388	4.952351504
GNA14	0.96695	0.03305	0.276436552	0.205471	1.009621388	4.952351504
GSG1L	0.96695	0.03305	0.276436552	0.205471	1.009621388	4.952351504
HCK	0.96695	0.03305	0.276436552	0.205471	1.009621388	4.952351504
LOC10272	0.96695	0.03305	0.276436552	0.205471	1.009621388	4.952351504
MANEA-A	0.96695	0.03305	0.276436552	0.205471	1.009621388	4.952351504
MATN1	0.96695	0.03305	0.276436552	0.205471	1.009621388	4.952351504
MB	0.96695	0.03305	0.276436552	0.205471	1.009621388	4.952351504
MYRIP	0.96695	0.03305	0.276436552	0.205471	1.009621388	4.952351504
NR5A2	0.96695	0.03305	0.276436552	0.205471	1.009621388	4.952351504
OR2L1P	0.96695	0.03305	0.276436552	0.205471	1.009621388	4.952351504
PEX5L	0.96695	0.03305	0.276436552	0.205471	1.009621388	4.952351504
POSTN	0.96695	0.03305	0.276436552	0.205471	1.009621388	4.952351504
PPEF1	0.96695	0.03305	0.276436552	0.205471	1.009621388	4.952351504
SAMD12-/	0.96695	0.03305	0.276436552	0.205471	1.009621388	4.952351504
SLC2A9	0.96695	0.03305	0.276436552	0.205471	1.009621388	4.952351504
VSIG8	0.96695	0.03305	0.276436552	0.205471	1.009621388	4.952351504
SYNPO	0.96699	0.03301	0.779498477	0.77939	789.7157536	1013.251118
ZNF835	0.967014	0.032986	0.442979277	0.419962	5.048106941	12.03421415
ISG15	0.967063	0.032937	0.750574738	0.74987	129.2315377	172.3418323
RABEP2	0.967077	0.032923	0.7675714	0.767173	218.0782198	284.2649763
ANK1	0.967096	0.032904	1.483579812	1.489671	57.54841913	38.62834173
C11orf45	0.967173	0.032827	1.333487232	1.334817	166.587529	124.7992579
GPN3	0.967223	0.032777	1.284728868	1.285043	566.3975988	440.7592838
PTPN21	0.967266	0.032734	0.779593978	0.779494	853.130073	1094.469682
FTSJ1	0.967286	0.032714	1.305241582	1.305287	4309.064085	3301.237512
ZNF845	0.967311	0.032689	1.28370431	1.283992	615.596449	479.4371491
FHAD1	0.967344	0.032656	1.43897616	1.443289	71.48119428	49.52351504
WDR45	0.96738	0.03262	0.780050192	0.779941	779.6902132	999.6816746
TOX2	0.967382	0.032618	0.778851784	0.778768	1019.717602	1309.401738
NKILA	0.967383	0.032617	1.295987684	1.296583	321.0596014	247.6175752
LOC49414	0.967477	0.032523	1.612345281	1.626016	35.43771072	21.79034662
ZSCAN16-	0.967562	0.032438	1.412705604	1.416219	82.77885762	58.44765245
SIDT2	0.967562	0.032438	1.281980436	1.282188	847.0723447	660.6436906
FBXW9	0.967575	0.032425	0.772812339	0.772494	268.5592893	347.6550756
ADAMTS1	0.967578	0.032422	0.747471387	0.746663	116.1064596	155.5038372
BRSK2	0.967608	0.032392	0.780133269	0.779961	496.733723	636.8724034
GPD2	0.967623	0.032377	1.282114051	1.282298	955.1018332	744.8336662
BRE	0.967656	0.032344	0.772912154	0.772868	1912.222909	2474.194811
HLA-F-AS	0.967668	0.032332	1.314912176	1.31585	215.0493557	163.4275996
KCNJ1	0.967678	0.032322	0.33959774	0.292259	2.019242776	6.933292105
ZNF300P1	0.967678	0.032322	0.33959774	0.292259	2.019242776	6.933292105
SULT1A1	0.967681	0.032319	0.513679985	0.499858	8.743321221	17.50161021
CRIP1	0.967686	0.032314	1.578305864	1.589925	39.37523414	24.76175752
NUDT1	0.967736	0.032264	1.350644533	1.35235	135.289266	100.0375004
MAP6	0.967741	0.032259	0.768499491	0.768099	216.0589771	281.2935654
PIAS1	0.967759	0.032241	1.281696844	1.281888	921.7843274	719.0814384
ADRA1A	0.967795	0.032205	0.282308463	0.212338	1.050006244	4.982065613
LRRC37B	0.967805	0.032195	1.293029864	1.293558	349.2280382	269.9724899
LINC0155	0.967814	0.032186	0.168470725	0.003975	0	2.505889861
FBXO33	0.967823	0.032177	0.777096498	0.776855	349.3290003	449.6735166
PSMD6-A	0.96785	0.03215	0.447799203	0.425207	5.048106941	11.88564361
SLC28A3	0.96785	0.03215	0.447799203	0.425207	5.048106941	11.88564361
VILL	0.96785	0.03215	0.447799203	0.425207	5.048106941	11.88564361

C11orf96	0.967861	0.032139	0.544807407	0.534162	11.10583527	20.79987632
LOC10028	0.967861	0.032139	0.544807407	0.534162	11.10583527	20.79987632
PLIN2	0.967866	0.032134	0.662884718	0.659637	33.31750581	50.51398534
TMEM56-I	0.967907	0.032093	1.363563353	1.365659	117.9136819	86.33929612
RAD54B	0.967935	0.032065	1.281345611	1.281533	935.1113297	729.6794706
ZNF205	0.967952	0.032048	0.773511692	0.773196	269.5689106	348.6455459
RGS9	0.96796	0.03204	0.644197205	0.640132	27.25977748	42.59022293
GPATCH1	0.967961	0.032039	1.290593453	1.291112	360.4348356	279.1640543
TMEM231	0.967978	0.032022	1.280342828	1.280528	965.1980471	753.7478989
BHMT2	0.967982	0.032018	2.384908255	2.554608	10.13659874	3.961881203
PPP2R5B	0.967988	0.032012	0.767752313	0.767331	205.9627632	268.4174515
TNF	0.967993	0.032007	1.283628582	1.283964	529.0416074	412.0356451
IL36G	0.968117	0.031883	0.277895334	0.205471	1.009621388	4.952351504
NUPR2	0.968117	0.031883	0.277895334	0.205471	1.009621388	4.952351504
ORM1	0.968117	0.031883	0.277895334	0.205471	1.009621388	4.952351504
SHISA3	0.968117	0.031883	0.277895334	0.205471	1.009621388	4.952351504
SLC25A21	0.968117	0.031883	0.277895334	0.205471	1.009621388	4.952351504
SPATA25	0.968117	0.031883	0.277895334	0.205471	1.009621388	4.952351504
RAB8B	0.968127	0.031873	0.780041166	0.779941	828.8991597	1062.774633
C21orf58	0.968197	0.031803	1.298314087	1.298963	290.7709598	223.846288
CC2D1B	0.968232	0.031768	0.780430034	0.780328	814.7644602	1044.133982
SEC13	0.968232	0.031768	1.287251687	1.287351	1799.145314	1397.553594
DCST2	0.968245	0.031755	0.589823498	0.582628	16.15394221	27.73316842
SLC25A5-	0.968273	0.031727	0.545042571	0.534162	11.10583527	20.79987632
COX7B	0.968284	0.031716	1.284989653	1.285103	1571.980501	1223.230821
UBAC2-AS	0.968315	0.031685	1.537286914	1.546388	44.42334108	28.72363872
EID1	0.968333	0.031667	0.77456893	0.774522	1795.106828	2317.700504
UPF3B	0.968333	0.031667	1.280688982	1.280843	1162.074218	907.2707955
DTX2	0.968344	0.031656	0.780141854	0.779935	412.4909143	528.8814265
GPR156	0.968357	0.031643	1.364955463	1.367065	115.0968383	84.18997557
PRR34-AS	0.9684	0.0316	1.336962261	1.338402	152.4528296	113.9040846
LOC10192	0.968412	0.031588	2.375850461	2.54444	10.09621388	3.961881203
NEK3	0.968431	0.031569	1.304355268	1.305164	244.3283759	187.1988868
CDKN2AIF	0.968449	0.031551	1.282287938	1.282617	536.1089571	417.9784669
COBLL1	0.968481	0.031519	1.282444632	1.28278	526.0127432	410.0547045
PAPD5	0.968491	0.031509	1.284005205	1.284117	1625.490435	1265.840854
SLC25A13	0.968493	0.031507	0.781505892	0.781337	503.8010727	644.7961658
SLC19A1	0.96851	0.03149	0.781452992	0.781281	493.7048588	631.9200519
MCC	0.968556	0.031444	0.766020564	0.765551	185.7703354	242.6652237
EGR2	0.968564	0.031436	0.522957877	0.509943	9.086592493	17.82846541
HOXC8	0.968564	0.031436	0.522957877	0.509943	9.086592493	17.82846541
SDR16C5	0.968564	0.031436	0.522957877	0.509943	9.086592493	17.82846541
LYST	0.968578	0.031422	1.281140296	1.281444	577.503434	450.6639869
ELP6	0.968647	0.031353	1.292438418	1.292998	329.1365725	254.5508673
KGFLP1	0.968659	0.031341	1.381813321	1.384398	99.11453168	71.59119334
PSG5	0.968721	0.031279	0.618132857	0.612616	20.62656496	33.67599023
CAMKMT	0.968732	0.031268	1.34163517	1.343204	142.3566157	105.9803222
NR2E1	0.968737	0.031263	0.492651269	0.476042	7.067349717	14.85705451
CTSB	0.968738	0.031262	0.740192173	0.740188	21928.63328	29625.76898
FAM207A	0.968753	0.031247	0.779809152	0.779735	1149.958761	1474.810278
ZNF776	0.968758	0.031242	0.780858583	0.780654	415.9640119	532.8433077
MTMR7	0.96883	0.03117	1.280739506	1.28105	563.3687346	439.7688135
ENO3	0.968841	0.031159	0.756785387	0.756114	136.2988874	180.2655947
NXF1	0.968859	0.031141	0.77766887	0.777611	1497.268519	1925.474265
ZNF833P	0.968863	0.031137	1.976126969	2.037362	16.15394221	7.923762406
TP53INP2	0.968891	0.031109	0.775270128	0.77494	262.5015609	338.7408429

ZNF479	0.968913	0.031087	0.458007984	0.436374	5.310608502	12.1827847
NUDT8	0.969	0.031	1.360959923	1.363022	116.1064596	85.18044587
TMEM120	0.969013	0.030987	0.75310053	0.752381	125.1930521	166.3990105
OLFML3	0.969028	0.030972	0.523244268	0.509943	9.086592493	17.82846541
CETN4P	0.969038	0.030962	2.115341047	2.20653	13.12507805	5.942821805
LOC10192	0.969038	0.030962	2.115341047	2.20653	13.12507805	5.942821805
SCPEP1	0.969049	0.030951	1.280152742	1.280462	564.378356	440.7592838
HOXB13	0.969056	0.030944	0.739444986	0.738437	92.88516771	125.7897282
GLE1	0.969078	0.030922	1.287338632	1.287423	2181.79182	1694.694685
LOC25739	0.969078	0.030922	1.347059491	1.348782	132.2604018	98.05655978
ZNF85	0.969102	0.030898	1.278579846	1.278828	699.7584879	547.1853177
C2orf88	0.969122	0.030878	0.78161286	0.781439	477.5509166	611.1201756
PSCA	0.969123	0.030877	2.372461903	2.54444	10.09621388	3.961881203
ELMO3	0.969125	0.030875	1.481157336	1.487546	54.51955496	36.64740113
C17orf96	0.969218	0.030782	0.777653474	0.777378	304.9056592	392.2262391
FLJ41200	0.969231	0.030769	0.199546138	0.063452	0.171635636	2.852554466
SMIM10L2	0.969236	0.030764	0.69658389	0.694375	47.45220524	68.34245075
PPM1N	0.96927	0.03073	0.669674063	0.666553	34.3271272	51.50445564
NPTX1	0.969273	0.030727	0.493014758	0.476042	7.067349717	14.85705451
SV2A	0.969273	0.030727	0.493014758	0.476042	7.067349717	14.85705451
TMEM40	0.969273	0.030727	0.493014758	0.476042	7.067349717	14.85705451
DYRK4	0.969322	0.030678	1.322412924	1.32359	175.6741215	132.7230203
NTNG1	0.969365	0.030635	0.77988623	0.779634	338.223165	433.8259917
BOLA1	0.969372	0.030628	1.29282534	1.29343	304.9056592	235.7319316
C14orf178	0.969446	0.030554	5.675171087	233.2129	2.322129193	0
ATP6V0E1	0.96946	0.03054	1.298429571	1.29848	3747.714593	2886.230456
RPSAP52	0.969483	0.030517	0.7294176	0.728123	75.72160411	103.9993816
RGPD3	0.969502	0.030498	0.528872982	0.516118	9.268324343	17.96713126
ACOT11	0.96958	0.03042	0.579267237	0.571001	14.13469943	24.76175752
MIR17HG	0.96958	0.03042	0.579267237	0.571001	14.13469943	24.76175752
ZFP2	0.969623	0.030377	0.758201435	0.757552	137.3085088	181.256065
ERBB3	0.969672	0.030328	1.314428204	1.315472	192.8376851	146.5896045
PTPRH	0.969686	0.030314	0.652713729	0.648749	28.26939887	43.58069323
KCNMB3	0.969708	0.030292	1.416531501	1.420372	74.97448428	52.78216233
MIEN1	0.969712	0.030288	1.296938494	1.296989	3787.089827	2919.906447
LINC0097	0.969769	0.030231	5.612770101	229.1744	2.281744337	0
DNLZ	0.969851	0.030149	0.737767582	0.736685	86.82743938	117.8659658
RAE1	0.969861	0.030139	1.281913965	1.282023	1606.307629	1252.94493
MUT	0.96988	0.03012	0.783087053	0.782927	514.906908	657.6722797
C1orf145	0.969903	0.030097	0.579450519	0.571001	14.13469943	24.76175752
ST8SIA1	0.969946	0.030054	0.288989966	0.217679	1.070198671	4.952351504
TUBE1	0.969952	0.030048	1.276709169	1.276891	946.0152407	740.871785
USP45	0.969967	0.030033	1.277129093	1.277296	1029.813816	806.2428248
GATA5	0.969973	0.030027	0.687750886	0.685179	41.39447691	60.41868835
THEMIS2	0.969973	0.030027	0.687750886	0.685179	41.39447691	60.41868835
B4GALNT	0.96999	0.03001	0.765046985	0.764513	163.5586649	213.941585
PHLDB2	0.970001	0.029999	1.303181223	1.303221	4857.288498	3727.139742
ADAMTS1	0.970011	0.029989	1.816130863	1.852554	20.19242776	10.89517331
HIST1H3D	0.970025	0.029975	3.490827192	4.702528	4.694739455	0.990470301
COL17A1	0.970036	0.029964	1.27619615	1.276394	867.2647724	679.4626263
CBX6	0.970038	0.029962	0.781256782	0.781181	1099.477692	1407.458297
STAG3	0.970052	0.029948	1.896627667	1.945256	17.44625759	8.963756222
HDAC5	0.970062	0.029938	0.783132608	0.783028	789.5239255	1008.298766
PNN	0.970094	0.029906	0.777299273	0.77725	1708.279389	2197.853597
IQCE	0.970135	0.029865	0.783146549	0.782944	417.9832547	533.8634921
FRA10AC	0.9702	0.0298	1.291114681	1.291731	296.8286881	229.7891098

ELFN2	0.970205	0.029795	1.275647549	1.275875	753.1775556	590.3202993
SSBP4	0.970207	0.029793	0.783837565	0.7837	597.6958618	762.6621316
COQ7	0.970223	0.029777	1.275788734	1.276038	687.5521653	538.8158436
FAM63B	0.970276	0.029724	0.780926867	0.780857	1197.299908	1533.317359
XIST	0.970292	0.029708	0.762804944	0.762788	5268.254884	6906.579121
MIF	0.970293	0.029707	0.77324776	0.773213	2464.485809	3187.333428
SNPH	0.970348	0.029652	0.77438731	0.774012	226.1551909	292.1887387
MID1IP1	0.970349	0.029651	1.279731279	1.279849	1478.085712	1154.888371
MST1L	0.970356	0.029644	2.181996848	2.295433	11.63083839	5.061303237
ZNF316	0.970374	0.029626	0.782066056	0.781845	375.5791564	480.3780959
CENPN	0.970387	0.029613	1.278436362	1.278558	1455.066345	1138.050376
SPAG8	0.970401	0.029599	1.615170151	1.630618	32.30788442	19.80940602
SMG1P2	0.970405	0.029595	1.275142858	1.275372	746.7159787	585.4868042
ATP8B5P	0.970406	0.029594	0.369075954	0.326782	2.362514048	7.250242602
TMEM182	0.97042	0.02958	1.328105388	1.329539	151.4432082	113.9040846
KIF21A	0.970434	0.029566	1.2814752	1.281578	1702.22166	1328.220673
AHSA2	0.970449	0.029551	0.784031805	0.783874	520.9646363	664.6055718
RPL4	0.970461	0.029539	0.741578411	0.741574	23030.47349	31056.19628
SNRPD1	0.970494	0.029506	1.28543649	1.285515	2316.071464	1801.665477
ITPR1	0.9705	0.0295	0.749342025	0.748481	106.0102458	141.637253
BLOC1S4	0.970518	0.029482	0.783875767	0.783677	423.0313616	539.8063139
EID2	0.970541	0.029459	0.783614406	0.783431	449.2815177	573.4823041
TNRC18	0.970544	0.029456	0.781337229	0.781271	1282.219163	1641.199384
PLEKHG1	0.970596	0.029404	0.784425625	0.784297	640.0999601	816.1475278
SPSB1	0.970645	0.029355	1.282833072	1.282922	2034.387097	1585.742952
SLC26A6	0.970659	0.029341	0.78301538	0.782785	367.5021853	469.4829226
ZNF808	0.970664	0.029336	1.273490409	1.273734	710.5210519	557.8229687
NEB	0.970704	0.029296	0.748927352	0.74802	102.9813816	137.6753718
LINC0093	0.970735	0.029265	0.459185666	0.436473	5.048106941	11.57859782
SP6	0.970773	0.029227	0.565896157	0.556205	12.11545666	21.79034662
S100A2	0.970777	0.029223	0.784398787	0.784231	491.685616	626.9677004
MALT1	0.970786	0.029214	0.778854727	0.778803	1608.326871	2065.130577
CD34	0.9708	0.0292	1.365642705	1.368006	102.9813816	75.27574286
SLC35G1	0.9708	0.0292	1.365642705	1.368006	102.9813816	75.27574286
ADAMTS9	0.970802	0.029198	0.669075122	0.665757	32.30788442	48.53304474
LOC22072	0.970818	0.029182	1.301819423	1.302717	217.896488	167.2607197
SOX13	0.970853	0.029147	1.277189987	1.277313	1434.671993	1123.193321
C10orf88	0.970872	0.029128	1.279047233	1.279454	427.0698472	333.7884914
LPCAT3	0.970879	0.029121	1.273957175	1.274165	817.7933244	641.8247549
ACAA1	0.970891	0.029109	0.78158848	0.781335	328.1269512	419.9594075
WDR72	0.970896	0.029104	0.754935137	0.754171	117.7521425	156.1377382
P3H2	0.970901	0.029099	1.284575269	1.284656	2194.916898	1708.561269
RAB11FIP	0.970917	0.029083	1.273824994	1.274055	737.0236134	578.4841792
SLC38A7	0.970932	0.029068	0.784829586	0.784672	522.1559895	665.4474716
SIPA1L2	0.971017	0.028983	0.781090198	0.780826	313.9922517	402.1309421
SEPT5-GF	0.97102	0.02898	0.469396522	0.44813	5.431763068	12.13326118
MAML3	0.971041	0.028959	0.693000588	0.690568	42.4040983	61.40915865
LINC0112	0.971044	0.028956	1.288974775	1.289598	291.1142311	225.7380862
TENM1	0.971079	0.028921	1.2740103	1.274166	1115.631634	875.5757459
NPIPB9	0.971089	0.028911	0.616601778	0.610548	18.80924646	30.81353106
EML6	0.971097	0.028903	0.638539876	0.633741	23.22129193	36.64740113
CSNK2A3	0.971119	0.028881	5.573803452	233.2129	2.322129193	0
ZMYND12	0.971127	0.028873	0.566110465	0.556205	12.11545666	21.79034662
LOC1019	0.971128	0.028872	0.705641639	0.703617	49.78443065	70.75919829
ZNF554	0.971131	0.028869	1.273846771	1.274169	539.4810925	423.3963395
KLF3-AS1	0.971134	0.028866	0.623625203	0.617896	20.19242776	32.68551993

ADGRL3	0.971136	0.028864	0.75927698	0.758589	129.2315377	170.3608917
JRK	0.971138	0.028862	0.772063824	0.771618	191.8280638	248.6080455
ZFP90	0.971182	0.028818	0.772124441	0.772095	2925.882783	3789.539371
PIGH	0.971183	0.028817	1.291758651	1.292445	267.5496679	207.0082929
TOP2A	0.9712	0.0288	1.334292292	1.334304	18425.59033	13809.13693
LOC11323	0.971257	0.028743	0.705564555	0.703527	49.47144802	70.32339135
SRPX	0.971266	0.028734	0.779464927	0.779154	268.5592893	344.6836647
SLC7A5P2	0.971288	0.028712	1.395621029	1.399053	80.26490036	57.36803982
IL1R1	0.971296	0.028704	1.296862398	1.296907	4174.78444	3219.028478
ADAM15	0.971335	0.028665	0.779319296	0.779269	1651.740591	2119.606444
SASS6	0.971336	0.028664	1.272940021	1.273195	663.321252	520.9873782
PLAGL1	0.971337	0.028663	0.785432859	0.785316	704.6753441	897.316569
COTL1	0.971382	0.028618	1.30519859	1.30523	6236.431315	4778.028731
ZNF276	0.97144	0.02856	0.785216293	0.78501	405.867798	517.025497
SLC25A37	0.971441	0.028559	0.778415716	0.778371	1913.303204	2458.089764
ZNF282	0.971458	0.028542	0.786242258	0.786074	496.733723	631.9200519
CHD8	0.971471	0.028529	0.781417945	0.781357	1369.046602	1752.141962
LOC14366	0.971481	0.028519	0.745286194	0.744297	92.88516771	124.7992579
TM2D1	0.971525	0.028475	1.271353768	1.271553	860.1974227	676.4912154
PDCD2L	0.971567	0.028433	1.288635381	1.289283	279.6651245	216.9129959
C4BPB	0.971579	0.028421	1.876569459	1.924374	17.1635636	8.914232707
LINC0157	0.971579	0.028421	1.876569459	1.924374	17.1635636	8.914232707
CCNI2	0.971617	0.028383	0.436016385	0.408332	4.038485553	9.904703008
CDIPT-AS	0.971617	0.028383	0.436016385	0.408332	4.038485553	9.904703008
CSF2	0.971617	0.028383	0.436016385	0.408332	4.038485553	9.904703008
FJX1	0.971617	0.028383	0.436016385	0.408332	4.038485553	9.904703008
GAS2	0.971617	0.028383	0.436016385	0.408332	4.038485553	9.904703008
HIST1H2A	0.971617	0.028383	0.436016385	0.408332	4.038485553	9.904703008
LOC64236	0.971617	0.028383	0.436016385	0.408332	4.038485553	9.904703008
MS4A15	0.971617	0.028383	0.436016385	0.408332	4.038485553	9.904703008
NDUFB2-A	0.971617	0.028383	0.436016385	0.408332	4.038485553	9.904703008
REP15	0.971617	0.028383	0.436016385	0.408332	4.038485553	9.904703008
SERPINB5	0.971641	0.028359	0.760775863	0.760096	130.2411591	171.351362
SLX4IP	0.971648	0.028352	1.352765002	1.354926	110.0487313	81.21856466
EMC2	0.97167	0.02833	1.278917215	1.279018	1730.491059	1352.982431
IDI1	0.971731	0.028269	0.786658672	0.786487	487.6471305	620.0344083
ZNF350	0.971778	0.028222	1.271607328	1.271813	818.8029458	643.8056955
LOC10050	0.97184	0.02816	1.407371762	1.411308	72.69273995	51.50445564
GOLGA3	0.971841	0.028159	0.784106445	0.784029	1064.161136	1357.300881
ZC3H12B	0.971843	0.028157	0.760968279	0.760284	129.2416339	169.9944177
AGBL2	0.971857	0.028143	0.595872658	0.588238	15.14432082	25.75222782
GMPR2	0.971965	0.028035	0.784826614	0.784741	959.1403188	1222.240351
PHF7	0.971967	0.028033	1.326439621	1.327911	143.3662371	107.9612628
PRUNE2	0.972021	0.027979	1.316359015	1.317651	160.5298007	121.827847
JMJD8	0.97205	0.02795	1.274748946	1.274874	1365.008117	1070.698395
FIZ1	0.972062	0.027938	0.763754374	0.763091	135.289266	177.2941838
BRAF	0.972166	0.027834	1.286203794	1.28684	281.6843673	218.8939365
PI3	0.972169	0.027831	0.70989234	0.707913	50.48106941	71.31386166
RTF1	0.972182	0.027818	0.779237424	0.779192	1847.60714	2371.1859
MTRF1	0.972184	0.027816	1.328421411	1.329958	138.3181302	103.9993816
TSNAX-DI	0.972195	0.027805	0.492108744	0.473443	6.259652607	13.23268322
DLG4	0.972239	0.027761	0.784529828	0.784288	340.2424078	433.8259917
HERC2P9	0.972259	0.027741	0.775664741	0.775244	201.0761957	259.3744577
EFCAB3	0.972259	0.027741	0.436612632	0.408332	4.038485553	9.904703008
LOC44108	0.972259	0.027741	0.436612632	0.408332	4.038485553	9.904703008
HAP1	0.972265	0.027735	0.668384242	0.664857	30.28864164	45.56163384

OAZ1	0.97231	0.02769	1.317946843	1.317966	11135.11429	8448.711666
VPS37C	0.972339	0.027661	1.27116611	1.271331	1018.707981	801.2904733
OSGIN1	0.97236	0.02764	1.279313388	1.279822	342.2616506	267.4269812
SEMA4A	0.972361	0.027639	1.538629686	1.549168	38.36561275	24.76175752
LINC0088(	0.972377	0.027623	1.680398266	1.703176	25.3112082	14.85705451
BCL2L13	0.972377	0.027623	1.27624931	1.276355	1619.432707	1268.792455
NUDT16	0.97238	0.02762	0.787264574	0.787105	509.858801	647.7675767
ALKBH1	0.972417	0.027583	1.27718403	1.27765	369.5214281	289.2173278
EPHX2	0.97244	0.02756	1.352525463	1.354767	106.0102458	78.24715376
GYG1	0.972459	0.027541	1.273035601	1.273167	1286.257649	1010.279707
TRMT2B	0.972463	0.027537	1.270699339	1.270999	558.6840914	439.5608148
CYLD	0.972464	0.027536	0.786224492	0.78614	990.4385818	1259.878223
ZFP42	0.972478	0.027522	0.489405629	0.470151	6.057728329	12.89592332
HS3ST1	0.972478	0.027522	1.341679092	1.34363	117.116081	87.16138647
C2orf48	0.972481	0.027519	2.472147014	2.712464	8.076971105	2.971410902
LINC0037	0.972481	0.027519	2.472147014	2.712464	8.076971105	2.971410902
LOC40112	0.972481	0.027519	2.472147014	2.712464	8.076971105	2.971410902
TXNDC2	0.972481	0.027519	2.472147014	2.712464	8.076971105	2.971410902
GTF2H2C	0.972495	0.027505	1.427919086	1.432786	62.68739199	43.74907319
TRAM2-AS	0.972517	0.027483	1.28873525	1.289445	255.4342112	198.0940602
ARHGEF3	0.972521	0.027479	1.284323212	1.284944	286.2074711	222.7369612
APOL3	0.972523	0.027477	0.758742848	0.757983	117.116081	154.5133669
EGFEM1P	0.972526	0.027474	0.60622011	0.599143	16.21451949	27.06955332
KDM7A	0.972531	0.027469	0.78806699	0.787896	484.6182663	615.0820568
HLA-G	0.972536	0.027464	2.685271811	3.053302	6.794751942	2.218653474
SPSB3	0.972547	0.027453	0.787574848	0.787451	658.2731451	835.9569339
VAMP2	0.972547	0.027453	0.785830427	0.785607	366.4925639	466.5115117
RTN4IP1	0.972548	0.027452	1.28207426	1.282652	304.9056592	237.7128722
CCNE2	0.972556	0.027444	0.778844814	0.778473	225.1455696	289.2173278
ZNF41	0.972565	0.027435	0.783778145	0.783505	301.8767951	385.292947
NLRC3	0.972575	0.027425	0.18562942	0.004568	0	2.179034662
LOC72867	0.972578	0.027422	0.606438006	0.599362	16.21451949	27.05964862
ABCC6	0.972593	0.027407	0.424213451	0.392969	3.594252142	9.161850282
CLUHP3	0.972595	0.027405	1.400203569	1.403913	73.70236134	52.49492594
USP2-AS1	0.972595	0.027405	1.400203569	1.403913	73.70236134	52.49492594
CFAP45	0.972604	0.027396	0.648430289	0.643885	24.23091332	37.63787143
APLP1	0.972605	0.027395	0.780772178	0.780436	247.3572401	316.9504962
ITGA5	0.97261	0.02739	1.290343264	1.290395	3532.665237	2737.659911
OVOL2	0.972617	0.027383	1.295909521	1.29677	217.0685985	167.3894808
ZNF701	0.972623	0.027377	1.26813312	1.268354	766.3632109	604.2165976
C16orf71	0.97263	0.02737	0.490130595	0.470873	6.057728329	12.87611391
FCGR2A	0.97263	0.02737	0.490130595	0.470873	6.057728329	12.87611391
GHR	0.97263	0.02737	0.490130595	0.470873	6.057728329	12.87611391
KCND3	0.97263	0.02737	0.490130595	0.470873	6.057728329	12.87611391
WT1	0.97263	0.02737	0.490130595	0.470873	6.057728329	12.87611391
RAB3B	0.972697	0.027303	1.275304929	1.275412	1600.966731	1255.252726
LOC38964	0.97273	0.02727	1.301658863	1.302636	194.8266393	149.5610154
RAD51C	0.972741	0.027259	1.269387034	1.269571	906.6400066	714.1290869
BMPR1B	0.972751	0.027249	1.273020369	1.273414	430.0987114	337.7503726
FAM179B	0.972767	0.027233	0.787786205	0.787671	703.7061075	893.4042113
CXorf38	0.972778	0.027222	1.27318112	1.273305	1358.132595	1066.617657
CDSN	0.972797	0.027203	1.969235645	2.037175	14.13469943	6.933292105
LINC0063	0.972797	0.027203	1.969235645	2.037175	14.13469943	6.933292105
ARSE	0.972813	0.027187	0.688574172	0.685792	37.35599136	54.47586654
ADRB2	0.972879	0.027121	1.26846805	1.268704	703.7061075	554.6633684
TRAF3IP1	0.972897	0.027103	0.787443739	0.787344	812.7553137	1032.278052

PSMC2	0.972897	0.027103	0.784518842	0.784452	1230.728472	1568.904956
CD101	0.972907	0.027093	0.524888661	0.509977	8.076971105	15.84752481
FAM66E	0.972933	0.027067	0.187013953	0.00461	0	2.159225256
SLC38A6	0.972941	0.027059	0.756464196	0.755608	107.0198671	141.637253
LIN9	0.972954	0.027046	1.27297853	1.273386	414.9543905	325.864729
ALG10	0.972979	0.027021	1.292684654	1.293505	224.7215286	173.7284908
ACOT1	0.972989	0.027011	0.748864801	0.747868	91.69381447	122.6103185
AAAS	0.972994	0.027006	1.268804084	1.268995	867.2647724	683.4245075
CLN8	0.973003	0.026997	0.78632171	0.786088	350.3588141	445.7017306
ASB8	0.973014	0.026986	0.787850672	0.787663	433.1174793	549.8793969
LRWD1	0.97305	0.02695	1.275513866	1.276019	346.3001361	271.3888624
GABPB2	0.973051	0.026949	1.281219883	1.281813	295.8190667	230.7795801
ABR	0.973085	0.026915	0.789314315	0.789166	557.3110063	706.2053245
SPC25	0.973088	0.026912	1.269650823	1.269967	524.8718711	413.2935424
CES1	0.9731	0.0269	0.763746445	0.763036	126.2026735	165.3986355
MSLN	0.973118	0.026882	0.786228975	0.786151	1044.958137	1329.211144
EFTUD1P	0.973125	0.026875	5.288961301	214.0301	2.130301129	0
SUGT1	0.973144	0.026856	1.269166739	1.269333	994.4770673	783.4620079
LOC79160	0.973144	0.026856	0.490549958	0.470873	6.057728329	12.87611391
ITGB4	0.973153	0.026847	1.314063788	1.314084	9880.154905	7518.660053
FAXDC2	0.973182	0.026818	2.46748908	2.712464	8.076971105	2.971410902
NOS1AP	0.973186	0.026814	1.304238306	1.305314	179.7126071	137.6753718
DKK1	0.973202	0.026798	1.269563721	1.269938	455.3392461	358.5502489
SDCBP	0.973221	0.026779	0.763234222	0.763221	6465.61537	8471.492483
SYNE4	0.973234	0.026766	2.129699441	2.240034	11.10583527	4.952351504
ZNF439	0.973235	0.026765	0.733734116	0.732353	68.70473546	93.81734689
HEXDC	0.973268	0.026732	0.785050446	0.784755	283.7036101	361.5216598
GOLGA8M	0.973303	0.026697	0.60791454	0.600742	15.97221036	26.59412758
DAB2IP	0.97331	0.02669	0.785394301	0.785326	1199.430209	1527.305204
S100A1	0.973384	0.026616	0.720558671	0.718799	55.52917635	77.25668346
KIF24	0.973412	0.026588	1.266936334	1.267176	686.5425439	541.7872545
LRRC8E	0.973424	0.026576	0.78739696	0.78717	359.4252142	456.6068087
SHOX2	0.973427	0.026573	0.732751316	0.731292	66.63501162	91.12326767
ZNF784	0.973427	0.026573	0.732751316	0.731292	66.63501162	91.12326767
HMCN1	0.973446	0.026554	0.585386305	0.576332	13.12507805	22.78081692
RNF168	0.973446	0.026554	0.789528987	0.789415	724.0297861	917.1754985
COG8	0.97346	0.02654	0.789289531	0.789157	611.3863278	774.7359646
USP51	0.973473	0.026527	0.675786806	0.672398	31.29826303	46.55210414
SLC25A25	0.973489	0.026511	0.755931143	0.755034	102.2645504	135.4468136
PROM2	0.973494	0.026506	1.27066667	1.271093	401.617292	315.9600259
AGER	0.973502	0.026498	0.732719206	0.731292	66.63501162	91.12326767
NARS	0.973518	0.026482	1.317681286	1.317698	12110.40855	9190.573921
ENDOG	0.973531	0.026469	0.78128633	0.780922	228.1744337	292.1887387
TTI2	0.973552	0.026448	1.275565722	1.276075	336.2039223	263.4651
PRR15	0.973567	0.026433	1.518992987	1.528798	39.37523414	25.75222782
KLRG2	0.973693	0.026307	1.593804402	1.609153	30.28864164	18.81893571
SUMO1P3	0.973699	0.026301	0.450986538	0.424046	4.2908909	10.13251118
AMPD3	0.973742	0.026258	0.667629408	0.663832	28.26939887	42.59022293
DTX3L	0.973768	0.026232	1.277847376	1.277927	2182.114899	1707.541084
ZNF136	0.973769	0.026231	1.268860948	1.269228	452.5829797	356.579213
SLC4A5	0.973793	0.026207	1.426715636	1.43182	59.5676619	41.59975263
AP5M1	0.97383	0.02617	0.78998932	0.789854	597.6958618	756.7193098
SCO2	0.973832	0.026168	0.77815911	0.777721	191.8280638	246.656819
ZNF766	0.97384	0.02616	1.268560329	1.268691	1300.584176	1025.136761
C5orf17	0.973843	0.026157	5.207653978	209.9916	2.089916273	0
RBKS	0.973862	0.026138	0.767615954	0.766977	135.2185925	176.3037135

CD36	0.973867	0.026133	2.113907621	2.222291	11.10583527	4.991970316
SNX22	0.973882	0.026118	0.688026519	0.685089	35.39732587	51.67283559
TTLL11	0.97389	0.02611	0.707946882	0.705739	45.43296247	64.38056955
CD83	0.973911	0.026089	1.289533226	1.290356	221.107084	171.351362
HNF1A-AS	0.973937	0.026063	0.486500552	0.465631	5.542821421	11.91535772
ZFYVE27	0.973943	0.026057	1.266569938	1.266882	527.0223646	415.9975263
LINC0111	0.973944	0.026056	2.127000634	2.240034	11.10583527	4.952351504
OLFML2B	0.973944	0.026056	2.127000634	2.240034	11.10583527	4.952351504
STARD5	0.973944	0.026056	2.127000634	2.240034	11.10583527	4.952351504
EPS8L1	0.973946	0.026054	0.657819941	0.65351	25.2405347	38.62834173
SNHG21	0.973947	0.026053	1.462075825	1.468903	49.47144802	33.67599023
LLGL2	0.973949	0.026051	1.28469355	1.284749	3279.250269	2552.441965
GFOD1	0.97395	0.02605	1.291922932	1.292798	210.0012487	162.4371293
GTF2IP20	0.973973	0.026027	0.778587113	0.778152	192.5045101	247.389767
COA1	0.973979	0.026021	1.264158805	1.264385	736.488514	582.4856792
RARB	0.974011	0.025989	0.645779473	0.640829	22.21167054	34.66646053
CES3	0.974013	0.025987	0.725449502	0.723736	57.70995855	79.74276392
SOWAHA	0.974025	0.025975	0.611427507	0.604199	16.15394221	26.74269812
PUSL1	0.974122	0.025878	0.783696522	0.783355	241.2995118	308.0362635
LOC10537	0.97414	0.02586	1.636471613	1.656006	26.25015609	15.84752481
RDH5	0.974162	0.025838	2.735498403	3.172802	6.118305612	1.921512384
LOC10192	0.974166	0.025834	0.735784571	0.734389	67.64463301	92.11373797
RAB30-AS	0.974178	0.025822	1.285918497	1.286686	233.2225407	181.256065
SERHL	0.974192	0.025808	0.374448408	0.327232	2.100012487	6.438056955
LOC1005C	0.974194	0.025806	0.571338493	0.560856	11.10583527	19.80940602
ROM1	0.974194	0.025806	0.571338493	0.560856	11.10583527	19.80940602
SUGCT	0.974229	0.025771	0.645910831	0.640829	22.21167054	34.66646053
BANK1	0.974291	0.025709	0.571381469	0.560856	11.10583527	19.80940602
C2orf70	0.974291	0.025709	0.571381469	0.560856	11.10583527	19.80940602
LOC10192	0.974291	0.025709	0.571381469	0.560856	11.10583527	19.80940602
CYP1A1	0.974315	0.025685	1.539401817	1.550921	35.33674859	22.78081692
GMFB	0.974337	0.025663	0.782372062	0.782329	1949.578901	2492.023277
ABCG4	0.974339	0.025661	1.69809996	1.724466	22.21167054	12.87611391
RASGEF1	0.974348	0.025652	1.405298867	1.409631	65.62539023	46.55210414
GPR1-AS	0.974369	0.025631	5.146673486	206.9628	2.059627632	0
DNAJA1	0.974377	0.025623	0.77837902	0.778346	2540.207413	3263.599641
LOC10192	0.974387	0.025613	3.258668055	4.34666	4.381756825	1.000375004
DMPK	0.974449	0.025551	0.790427774	0.790232	410.915905	519.9969079
SENP8	0.974466	0.025534	1.336566666	1.338604	110.0487313	82.20903496
LZTR1	0.974515	0.025485	0.790440424	0.790333	749.13907	947.8800778
CREB3L4	0.974517	0.025483	1.286454073	1.287258	223.1263268	173.3323026
SDHD	0.97453	0.02547	1.283302309	1.283358	3278.240647	2554.422906
GPR27	0.974549	0.025451	0.682870066	0.679624	32.30788442	47.54257444
PHLPP2	0.974553	0.025447	0.789807867	0.789717	885.4379574	1121.21238
ZNF81	0.974575	0.025425	0.77081713	0.770177	137.3085088	178.2846541
FAT3	0.9746	0.0254	0.573370273	0.562894	11.14622013	19.80940602
ST13P4	0.974601	0.025399	5.195967515	214.0301	2.130301129	0
SERPINA5	0.97461	0.02539	0.734501154	0.733035	64.61576884	88.15185677
PLXNA3	0.974635	0.025365	0.790997607	0.790899	831.9280238	1051.879459
FAM49A	0.974637	0.025363	1.262249495	1.262511	628.9941248	498.2065613
XDH	0.97464	0.02536	1.291094003	1.292005	200.9146562	155.5038372
ANO2	0.974642	0.025358	5.082097435	202.9243	2.019242776	0
CLDN34	0.974642	0.025358	5.082097435	202.9243	2.019242776	0
DYDC2	0.974642	0.025358	5.082097435	202.9243	2.019242776	0
LOC10317	0.974642	0.025358	5.082097435	202.9243	2.019242776	0
LOC38933	0.974642	0.025358	5.082097435	202.9243	2.019242776	0

OR51B5	0.974642	0.025358	5.082097435	202.9243	2.019242776	0
RPS2P32	0.974642	0.025358	5.082097435	202.9243	2.019242776	0
SNORA49	0.974642	0.025358	5.082097435	202.9243	2.019242776	0
TPH1	0.974642	0.025358	5.082097435	202.9243	2.019242776	0
SPA17	0.974652	0.025348	1.270734673	1.271252	331.1558153	260.4936891
TMEM147	0.974707	0.025293	0.759819844	0.758939	102.9813816	135.6944312
MTMR4	0.974717	0.025283	0.790154391	0.790069	967.2172899	1224.221292
TPGS1	0.974717	0.025283	0.71267733	0.71049	46.44258386	65.37103985
SLC47A1	0.974726	0.025274	0.773160867	0.772561	145.3854799	188.1893571
DHRS13	0.974739	0.025261	0.791737461	0.7916	582.551541	735.9194335
GLI4	0.974756	0.025244	0.757699781	0.756798	98.94289604	130.7420797
NBR2	0.974813	0.025187	0.78370336	0.78333	221.1272764	282.2939404
DPY19L2F	0.974826	0.025174	0.195013857	0.004854	0	2.050273523
PMEL	0.974827	0.025173	1.471877816	1.479524	45.43296247	30.70457932
LOC44046	0.974841	0.025159	0.727981558	0.726311	57.54841913	79.23762406
DHRS4-AS	0.974854	0.025146	0.791950115	0.79175	409.9567647	517.7881591
AMBP	0.974855	0.025145	0.418689673	0.383029	3.028864164	7.923762406
APLN	0.974855	0.025145	0.418689673	0.383029	3.028864164	7.923762406
BDKRB2	0.974855	0.025145	0.418689673	0.383029	3.028864164	7.923762406
CARD9	0.974855	0.025145	0.418689673	0.383029	3.028864164	7.923762406
CCDC160	0.974855	0.025145	0.418689673	0.383029	3.028864164	7.923762406
CXCL5	0.974855	0.025145	0.418689673	0.383029	3.028864164	7.923762406
CYP46A1	0.974855	0.025145	0.418689673	0.383029	3.028864164	7.923762406
LOC38828	0.974855	0.025145	0.418689673	0.383029	3.028864164	7.923762406
PLCL1	0.974855	0.025145	0.418689673	0.383029	3.028864164	7.923762406
EXOC3L2	0.974885	0.025115	5.085692993	203.9339	2.02933899	0
MIRLET7E	0.974894	0.025106	0.751479515	0.750426	86.12070441	114.7657938
PCDHGA1	0.974924	0.025076	0.632645595	0.626682	18.77895782	29.9716313
LINC0131	0.974943	0.025057	5.156195311	212.0109	2.110108701	0
RPS6KA1	0.974988	0.025012	1.282346613	1.282401	3302.471561	2575.222782
C20orf196	0.975017	0.024983	1.397433971	1.401502	66.63501162	47.54257444
RFFL	0.975046	0.024954	1.264837504	1.26497	1257.503631	994.0954221
ADIPOQ	0.975055	0.024945	5.065366162	202.9243	2.019242776	0
ANKRD18	0.975055	0.024945	5.065366162	202.9243	2.019242776	0
ARHGAP3	0.975055	0.024945	5.065366162	202.9243	2.019242776	0
ASCL5	0.975055	0.024945	5.065366162	202.9243	2.019242776	0
BMPR1B-A	0.975055	0.024945	5.065366162	202.9243	2.019242776	0
C8G	0.975055	0.024945	5.065366162	202.9243	2.019242776	0
CNNM1	0.975055	0.024945	5.065366162	202.9243	2.019242776	0
CTD-3080	0.975055	0.024945	5.065366162	202.9243	2.019242776	0
CYP21A1F	0.975055	0.024945	5.065366162	202.9243	2.019242776	0
EMILIN1	0.975055	0.024945	5.065366162	202.9243	2.019242776	0
EQTN	0.975055	0.024945	5.065366162	202.9243	2.019242776	0
EYA1	0.975055	0.024945	5.065366162	202.9243	2.019242776	0
GALNT8	0.975055	0.024945	5.065366162	202.9243	2.019242776	0
GP5	0.975055	0.024945	5.065366162	202.9243	2.019242776	0
GPR82	0.975055	0.024945	5.065366162	202.9243	2.019242776	0
GSDMC	0.975055	0.024945	5.065366162	202.9243	2.019242776	0
HIST1H2A	0.975055	0.024945	5.065366162	202.9243	2.019242776	0
HIST1H4E	0.975055	0.024945	5.065366162	202.9243	2.019242776	0
HLA-DOB	0.975055	0.024945	5.065366162	202.9243	2.019242776	0
KCNN1	0.975055	0.024945	5.065366162	202.9243	2.019242776	0
LINC0041	0.975055	0.024945	5.065366162	202.9243	2.019242776	0
LINC0054	0.975055	0.024945	5.065366162	202.9243	2.019242776	0
LINC0108	0.975055	0.024945	5.065366162	202.9243	2.019242776	0
LINC0113	0.975055	0.024945	5.065366162	202.9243	2.019242776	0

LINC0134	0.975055	0.024945	5.065366162	202.9243	2.019242776	0
LINCR-00	0.975055	0.024945	5.065366162	202.9243	2.019242776	0
LOC10012	0.975055	0.024945	5.065366162	202.9243	2.019242776	0
LOC10013	0.975055	0.024945	5.065366162	202.9243	2.019242776	0
LOC10155	0.975055	0.024945	5.065366162	202.9243	2.019242776	0
LOC10192	0.975055	0.024945	5.065366162	202.9243	2.019242776	0
LOC10192	0.975055	0.024945	5.065366162	202.9243	2.019242776	0
LOC10192	0.975055	0.024945	5.065366162	202.9243	2.019242776	0
LOC10192	0.975055	0.024945	5.065366162	202.9243	2.019242776	0
LOC10537	0.975055	0.024945	5.065366162	202.9243	2.019242776	0
LOC44070	0.975055	0.024945	5.065366162	202.9243	2.019242776	0
LRRC15	0.975055	0.024945	5.065366162	202.9243	2.019242776	0
MESTIT1	0.975055	0.024945	5.065366162	202.9243	2.019242776	0
METTL21I	0.975055	0.024945	5.065366162	202.9243	2.019242776	0
MMD2	0.975055	0.024945	5.065366162	202.9243	2.019242776	0
MTUS2	0.975055	0.024945	5.065366162	202.9243	2.019242776	0
NANOG	0.975055	0.024945	5.065366162	202.9243	2.019242776	0
OR2L2	0.975055	0.024945	5.065366162	202.9243	2.019242776	0
PLIN5	0.975055	0.024945	5.065366162	202.9243	2.019242776	0
POTEB	0.975055	0.024945	5.065366162	202.9243	2.019242776	0
POTEB2	0.975055	0.024945	5.065366162	202.9243	2.019242776	0
PROSER2	0.975055	0.024945	5.065366162	202.9243	2.019242776	0
RDH16	0.975055	0.024945	5.065366162	202.9243	2.019242776	0
ROPN1L-/	0.975055	0.024945	5.065366162	202.9243	2.019242776	0
SERINC4	0.975055	0.024945	5.065366162	202.9243	2.019242776	0
SERPINC1	0.975055	0.024945	5.065366162	202.9243	2.019242776	0
SNTN	0.975055	0.024945	5.065366162	202.9243	2.019242776	0
SNX29P1	0.975055	0.024945	5.065366162	202.9243	2.019242776	0
TXLNB	0.975055	0.024945	5.065366162	202.9243	2.019242776	0
ZNF341-A	0.975055	0.024945	5.065366162	202.9243	2.019242776	0
UXS1	0.97507	0.02493	1.288753362	1.288796	4225.265509	3278.456696
MED31	0.975076	0.024924	0.733091236	0.731555	61.58690468	84.18997557
NOL9	0.975078	0.024922	0.791640559	0.791441	402.2634497	508.2697395
LYPD5	0.975105	0.024895	0.746123065	0.744924	76.7312255	103.0089113
LOC10192	0.975114	0.024886	1.309189641	1.310546	145.3854799	110.9326737
BRMS1L	0.975116	0.024884	0.781384459	0.780951	191.8280638	245.6366346
TRADD	0.975129	0.024871	0.781670585	0.781221	188.7991996	241.6747534
MBIP	0.975139	0.024861	0.791232536	0.79099	339.2327864	428.8736402
TBC1D31	0.975147	0.024853	1.260549635	1.260804	643.1288243	510.0922049
LOC28329	0.975155	0.024845	0.196542494	0.004901	0	2.030464117
B9D2	0.975205	0.024795	1.326730939	1.328535	117.116081	88.15185677
ZNF221	0.975207	0.024793	1.298471104	1.299591	168.6269643	129.7516094
LOC28304	0.975219	0.024781	0.397049725	0.354786	2.453379973	6.933292105
LINC0155	0.975227	0.024773	0.685880456	0.682595	32.46942384	47.57228855
WDPCP	0.975238	0.024762	1.298316073	1.299435	168.6067718	129.7516094
JKAMP	0.975244	0.024756	0.790893998	0.790803	880.3898505	1113.288618
PDCD1LG	0.975262	0.024738	0.55283867	0.539921	9.086592493	16.83799511
VN1R1	0.975262	0.024738	0.55283867	0.539921	9.086592493	16.83799511
GSDMB	0.975311	0.024689	0.784764911	0.78439	219.0878412	279.3126248
LINC0119	0.975319	0.024681	0.197315835	0.004925	0	2.020559414
LOC10192	0.975319	0.024681	0.197315835	0.004925	0	2.020559414
SATB2	0.975328	0.024672	0.783324703	0.78292	203.9435204	260.4936891
PIK3C3	0.975336	0.024664	0.791818883	0.791727	895.5341713	1131.117083
ARV1	0.975348	0.024652	1.277280889	1.277959	254.4245898	199.0845305
MCM8	0.97535	0.02465	1.266552725	1.266669	1411.450701	1114.298898
PTGR1	0.975354	0.024646	1.283541586	1.283593	3472.087954	2704.974391

LOC10050	0.975362	0.024638	0.603089416	0.594783	14.13469943	23.77128722
MTMR14	0.975391	0.024609	1.261248571	1.261443	825.8702955	654.7008688
MFSD13A	0.97541	0.02459	0.625587802	0.61902	17.1635636	27.73316842
SPAG4	0.97541	0.02459	0.625587802	0.61902	17.1635636	27.73316842
AASDH	0.975414	0.024586	1.262193317	1.26252	492.6952374	390.2452985
LINC0029	0.975431	0.024569	0.78537848	0.78501	222.5811312	283.541933
MMP16	0.975486	0.024514	0.717173006	0.715099	47.45220524	66.36151015
ADAMTS3	0.975497	0.024503	0.419448627	0.383029	3.028864164	7.923762406
CASS4	0.975497	0.024503	0.419448627	0.383029	3.028864164	7.923762406
LL22NC03	0.975497	0.024503	0.419448627	0.383029	3.028864164	7.923762406
LOC10012	0.975497	0.024503	0.419448627	0.383029	3.028864164	7.923762406
MEI1	0.975497	0.024503	0.419448627	0.383029	3.028864164	7.923762406
MKRN3	0.975497	0.024503	0.419448627	0.383029	3.028864164	7.923762406
RUSC1-AS	0.975497	0.024503	0.419448627	0.383029	3.028864164	7.923762406
SYCE3	0.975497	0.024503	0.419448627	0.383029	3.028864164	7.923762406
SBDSP1	0.975499	0.024501	1.263707607	1.264128	394.9840795	312.4537611
MCM10	0.975517	0.024483	1.260023694	1.260212	866.255151	687.3863887
COMMD8	0.975517	0.024483	1.259626841	1.259831	798.610518	633.9009925
CYP4F35F	0.975547	0.024453	1.85750203	1.910105	15.14432082	7.923762406
PCDH15	0.975547	0.024453	1.85750203	1.910105	15.14432082	7.923762406
MPP3	0.975552	0.024448	0.773526638	0.772915	139.3277516	180.2655947
B3GNT8	0.975563	0.024437	0.199813541	0.005023	0	1.980940602
BRINP3	0.975563	0.024437	0.199813541	0.005023	0	1.980940602
CSAG1	0.975563	0.024437	0.199813541	0.005023	0	1.980940602
HMSD	0.975563	0.024437	0.199813541	0.005023	0	1.980940602
JSRP1	0.975563	0.024437	0.199813541	0.005023	0	1.980940602
KISS1R	0.975563	0.024437	0.199813541	0.005023	0	1.980940602
LOC10192	0.975563	0.024437	0.199813541	0.005023	0	1.980940602
OR51B4	0.975563	0.024437	0.199813541	0.005023	0	1.980940602
PGC	0.975563	0.024437	0.199813541	0.005023	0	1.980940602
SLC25A30	0.975563	0.024437	0.199813541	0.005023	0	1.980940602
TF	0.975563	0.024437	0.199813541	0.005023	0	1.980940602
TUBA3FP	0.975563	0.024437	0.199813541	0.005023	0	1.980940602
UGT2A2	0.975563	0.024437	0.199813541	0.005023	0	1.980940602
VSTM2L	0.975563	0.024437	0.199813541	0.005023	0	1.980940602
CNTD2	0.975573	0.024427	2.646788568	3.047669	6.057728329	1.980940602
SNAP23	0.975574	0.024426	0.793091246	0.792963	618.8979109	780.490597
DSG3	0.975621	0.024379	2.296587029	2.483801	8.551493158	3.436931944
ASPHD1	0.975632	0.024368	0.625733745	0.61902	17.1635636	27.73316842
HSD17B14	0.975632	0.024368	0.625733745	0.61902	17.1635636	27.73316842
MAFA	0.975632	0.024368	0.625733745	0.61902	17.1635636	27.73316842
SVOPL	0.975634	0.024366	0.553122451	0.539921	9.086592493	16.83799511
LINC0155	0.975643	0.024357	0.198880919	0.004973	0	2.000750008
TGM5	0.975687	0.024313	1.483742473	1.492421	41.39447691	27.73316842
GJA9-MYC	0.975732	0.024268	0.388797242	0.342876	2.21107084	6.467771064
ANKRD36	0.975738	0.024262	1.26102793	1.261353	493.3514913	391.1268171
COX19	0.975739	0.024261	1.266483177	1.266958	346.3506172	273.369803
MRPS5	0.975769	0.024231	1.274732757	1.274801	2539.197791	1991.835775
MLH3	0.975772	0.024228	1.26021794	1.260382	992.4578246	787.4238891
RFC5	0.975776	0.024224	1.266340868	1.266445	1614.3846	1274.735277
C11orf91	0.975783	0.024217	2.645435452	3.047669	6.057728329	1.980940602
CCNB3	0.975783	0.024217	2.645435452	3.047669	6.057728329	1.980940602
CD79A	0.975783	0.024217	2.645435452	3.047669	6.057728329	1.980940602
CPO	0.975783	0.024217	2.645435452	3.047669	6.057728329	1.980940602
EDN2	0.975783	0.024217	2.645435452	3.047669	6.057728329	1.980940602
HCG17	0.975783	0.024217	2.645435452	3.047669	6.057728329	1.980940602

HIST1H2A	0.975783	0.024217	2.645435452	3.047669	6.057728329	1.980940602
KCCAT33	0.975783	0.024217	2.645435452	3.047669	6.057728329	1.980940602
RPRM	0.975783	0.024217	2.645435452	3.047669	6.057728329	1.980940602
FAM174B	0.975795	0.024205	0.787093613	0.786743	232.2129193	295.1601496
SNRPG	0.975842	0.024158	1.266711038	1.266816	1565.922773	1236.106935
EXD1	0.975884	0.024116	0.630145981	0.62374	17.51693108	28.08973773
L1TD1	0.975913	0.024087	0.604650056	0.596348	13.81162059	23.16710034
DUSP23	0.975922	0.024078	0.788013371	0.787675	240.2898904	305.0648526
HAPLN4	0.975928	0.024072	0.791705155	0.791622	965.1980471	1219.26894
P2RX7	0.975948	0.024052	0.526623768	0.510021	7.067349717	13.86658421
ACHE	0.975962	0.024038	0.20047103	0.005023	0	1.980940602
ADGRF4	0.975962	0.024038	0.20047103	0.005023	0	1.980940602
ADH1C	0.975962	0.024038	0.20047103	0.005023	0	1.980940602
ADRA1D	0.975962	0.024038	0.20047103	0.005023	0	1.980940602
AFF2	0.975962	0.024038	0.20047103	0.005023	0	1.980940602
AFP	0.975962	0.024038	0.20047103	0.005023	0	1.980940602
ALK	0.975962	0.024038	0.20047103	0.005023	0	1.980940602
AOC4P	0.975962	0.024038	0.20047103	0.005023	0	1.980940602
APCDD1	0.975962	0.024038	0.20047103	0.005023	0	1.980940602
ARHGAP1	0.975962	0.024038	0.20047103	0.005023	0	1.980940602
ATP12A	0.975962	0.024038	0.20047103	0.005023	0	1.980940602
ATP2B3	0.975962	0.024038	0.20047103	0.005023	0	1.980940602
C10orf105	0.975962	0.024038	0.20047103	0.005023	0	1.980940602
C11orf85	0.975962	0.024038	0.20047103	0.005023	0	1.980940602
C12orf50	0.975962	0.024038	0.20047103	0.005023	0	1.980940602
C15orf56	0.975962	0.024038	0.20047103	0.005023	0	1.980940602
C1QTNF2	0.975962	0.024038	0.20047103	0.005023	0	1.980940602
C1QTNF5	0.975962	0.024038	0.20047103	0.005023	0	1.980940602
C2CD4B	0.975962	0.024038	0.20047103	0.005023	0	1.980940602
CA8	0.975962	0.024038	0.20047103	0.005023	0	1.980940602
CABP1	0.975962	0.024038	0.20047103	0.005023	0	1.980940602
CASQ2	0.975962	0.024038	0.20047103	0.005023	0	1.980940602
CCDC162	0.975962	0.024038	0.20047103	0.005023	0	1.980940602
CD28	0.975962	0.024038	0.20047103	0.005023	0	1.980940602
CDH19	0.975962	0.024038	0.20047103	0.005023	0	1.980940602
CDKL2	0.975962	0.024038	0.20047103	0.005023	0	1.980940602
CEMIP	0.975962	0.024038	0.20047103	0.005023	0	1.980940602
CLDN9	0.975962	0.024038	0.20047103	0.005023	0	1.980940602
CLEC4A	0.975962	0.024038	0.20047103	0.005023	0	1.980940602
CNN1	0.975962	0.024038	0.20047103	0.005023	0	1.980940602
CNTN6	0.975962	0.024038	0.20047103	0.005023	0	1.980940602
COL2A1	0.975962	0.024038	0.20047103	0.005023	0	1.980940602
COL5A3	0.975962	0.024038	0.20047103	0.005023	0	1.980940602
COMP	0.975962	0.024038	0.20047103	0.005023	0	1.980940602
CPZ	0.975962	0.024038	0.20047103	0.005023	0	1.980940602
CRTAC1	0.975962	0.024038	0.20047103	0.005023	0	1.980940602
CSNK1A1	0.975962	0.024038	0.20047103	0.005023	0	1.980940602
CST7	0.975962	0.024038	0.20047103	0.005023	0	1.980940602
CTCFL	0.975962	0.024038	0.20047103	0.005023	0	1.980940602
CYP21A2	0.975962	0.024038	0.20047103	0.005023	0	1.980940602
DUOX2	0.975962	0.024038	0.20047103	0.005023	0	1.980940602
ENPP7P1	0.975962	0.024038	0.20047103	0.005023	0	1.980940602
EPHA7	0.975962	0.024038	0.20047103	0.005023	0	1.980940602
EPPIN	0.975962	0.024038	0.20047103	0.005023	0	1.980940602
ERAS	0.975962	0.024038	0.20047103	0.005023	0	1.980940602
ESPNL	0.975962	0.024038	0.20047103	0.005023	0	1.980940602

FADS6	0.975962	0.024038	0.20047103	0.005023	0	1.980940602
FAM26E	0.975962	0.024038	0.20047103	0.005023	0	1.980940602
FAM71A	0.975962	0.024038	0.20047103	0.005023	0	1.980940602
FOXC2	0.975962	0.024038	0.20047103	0.005023	0	1.980940602
FOXF1	0.975962	0.024038	0.20047103	0.005023	0	1.980940602
FREM1	0.975962	0.024038	0.20047103	0.005023	0	1.980940602
GPR20	0.975962	0.024038	0.20047103	0.005023	0	1.980940602
GRIN1	0.975962	0.024038	0.20047103	0.005023	0	1.980940602
GRPR	0.975962	0.024038	0.20047103	0.005023	0	1.980940602
HAO2	0.975962	0.024038	0.20047103	0.005023	0	1.980940602
HHATL	0.975962	0.024038	0.20047103	0.005023	0	1.980940602
HIST1H1E	0.975962	0.024038	0.20047103	0.005023	0	1.980940602
HPN	0.975962	0.024038	0.20047103	0.005023	0	1.980940602
IKZF1	0.975962	0.024038	0.20047103	0.005023	0	1.980940602
INSL4	0.975962	0.024038	0.20047103	0.005023	0	1.980940602
ISL1	0.975962	0.024038	0.20047103	0.005023	0	1.980940602
ITFG1-AS	0.975962	0.024038	0.20047103	0.005023	0	1.980940602
ITIH3	0.975962	0.024038	0.20047103	0.005023	0	1.980940602
KIAA0087	0.975962	0.024038	0.20047103	0.005023	0	1.980940602
KLK11	0.975962	0.024038	0.20047103	0.005023	0	1.980940602
KRT16P2	0.975962	0.024038	0.20047103	0.005023	0	1.980940602
KRT79	0.975962	0.024038	0.20047103	0.005023	0	1.980940602
KY	0.975962	0.024038	0.20047103	0.005023	0	1.980940602
LINC0030	0.975962	0.024038	0.20047103	0.005023	0	1.980940602
LINC0055	0.975962	0.024038	0.20047103	0.005023	0	1.980940602
LINC0062	0.975962	0.024038	0.20047103	0.005023	0	1.980940602
LINC0119	0.975962	0.024038	0.20047103	0.005023	0	1.980940602
LINC0119	0.975962	0.024038	0.20047103	0.005023	0	1.980940602
LINC0119	0.975962	0.024038	0.20047103	0.005023	0	1.980940602
LINC0126	0.975962	0.024038	0.20047103	0.005023	0	1.980940602
LINC0150	0.975962	0.024038	0.20047103	0.005023	0	1.980940602
LOC10192	0.975962	0.024038	0.20047103	0.005023	0	1.980940602
LOC10192	0.975962	0.024038	0.20047103	0.005023	0	1.980940602
LOC10192	0.975962	0.024038	0.20047103	0.005023	0	1.980940602
LOC10272	0.975962	0.024038	0.20047103	0.005023	0	1.980940602
LOC28357	0.975962	0.024038	0.20047103	0.005023	0	1.980940602
LOC33953	0.975962	0.024038	0.20047103	0.005023	0	1.980940602
LOC39236	0.975962	0.024038	0.20047103	0.005023	0	1.980940602
LOC40332	0.975962	0.024038	0.20047103	0.005023	0	1.980940602
LOC41430	0.975962	0.024038	0.20047103	0.005023	0	1.980940602
LOC72873	0.975962	0.024038	0.20047103	0.005023	0	1.980940602
LRRC16B	0.975962	0.024038	0.20047103	0.005023	0	1.980940602
MARCH4	0.975962	0.024038	0.20047103	0.005023	0	1.980940602
MEP1B	0.975962	0.024038	0.20047103	0.005023	0	1.980940602
MIR5047	0.975962	0.024038	0.20047103	0.005023	0	1.980940602
MKX-AS1	0.975962	0.024038	0.20047103	0.005023	0	1.980940602
MRGPRE	0.975962	0.024038	0.20047103	0.005023	0	1.980940602
MUC4	0.975962	0.024038	0.20047103	0.005023	0	1.980940602
NAALADL	0.975962	0.024038	0.20047103	0.005023	0	1.980940602
NEUROG2	0.975962	0.024038	0.20047103	0.005023	0	1.980940602
NPY1R	0.975962	0.024038	0.20047103	0.005023	0	1.980940602
NXF3	0.975962	0.024038	0.20047103	0.005023	0	1.980940602
OR2B6	0.975962	0.024038	0.20047103	0.005023	0	1.980940602
P2RX6	0.975962	0.024038	0.20047103	0.005023	0	1.980940602
PLA2G4E	0.975962	0.024038	0.20047103	0.005023	0	1.980940602
PLCG1-AS	0.975962	0.024038	0.20047103	0.005023	0	1.980940602

PLEKHD1	0.975962	0.024038	0.20047103	0.005023	0	1.980940602
PLXDC2	0.975962	0.024038	0.20047103	0.005023	0	1.980940602
PNMA3	0.975962	0.024038	0.20047103	0.005023	0	1.980940602
POU3F1	0.975962	0.024038	0.20047103	0.005023	0	1.980940602
PPFIA2	0.975962	0.024038	0.20047103	0.005023	0	1.980940602
PRORY	0.975962	0.024038	0.20047103	0.005023	0	1.980940602
PTCHD1	0.975962	0.024038	0.20047103	0.005023	0	1.980940602
RAB19	0.975962	0.024038	0.20047103	0.005023	0	1.980940602
RAMP1	0.975962	0.024038	0.20047103	0.005023	0	1.980940602
RHOH	0.975962	0.024038	0.20047103	0.005023	0	1.980940602
SCARNA5	0.975962	0.024038	0.20047103	0.005023	0	1.980940602
SCIMP	0.975962	0.024038	0.20047103	0.005023	0	1.980940602
SEC1P	0.975962	0.024038	0.20047103	0.005023	0	1.980940602
SERPINA4	0.975962	0.024038	0.20047103	0.005023	0	1.980940602
SNORA61	0.975962	0.024038	0.20047103	0.005023	0	1.980940602
SNX32	0.975962	0.024038	0.20047103	0.005023	0	1.980940602
SPOCK3	0.975962	0.024038	0.20047103	0.005023	0	1.980940602
SST	0.975962	0.024038	0.20047103	0.005023	0	1.980940602
TCHH	0.975962	0.024038	0.20047103	0.005023	0	1.980940602
TMEM156	0.975962	0.024038	0.20047103	0.005023	0	1.980940602
TP53AIP1	0.975962	0.024038	0.20047103	0.005023	0	1.980940602
TSHZ2	0.975962	0.024038	0.20047103	0.005023	0	1.980940602
TSPAN11	0.975962	0.024038	0.20047103	0.005023	0	1.980940602
UOX	0.975962	0.024038	0.20047103	0.005023	0	1.980940602
UPK3A	0.975962	0.024038	0.20047103	0.005023	0	1.980940602
VMO1	0.975962	0.024038	0.20047103	0.005023	0	1.980940602
VWA3A	0.975962	0.024038	0.20047103	0.005023	0	1.980940602
ZBBX	0.975962	0.024038	0.20047103	0.005023	0	1.980940602
P3H2-AS1	0.975969	0.024031	1.327630917	1.329555	110.2001745	82.88255477
SMG6	0.97598	0.02402	0.794499658	0.794381	684.5233012	861.7091617
HECW1	0.97599	0.02401	1.374526579	1.37792	73.70236134	53.48539624
HERPUD1	0.976015	0.023985	0.778724677	0.778696	2963.238774	3805.386896
KCNC3	0.976018	0.023982	0.790956957	0.790679	289.7613384	366.4740113
INPP1	0.97602	0.02398	1.258162349	1.258359	823.8510527	654.7008688
HEXB	0.976023	0.023977	1.282943887	1.282993	3596.271385	2803.030951
PDE6B	0.976024	0.023976	0.486310453	0.463405	5.048106941	10.90507801
GKAP1	0.976029	0.023971	1.259912234	1.260068	1045.876892	830.014112
PPP3CB-A	0.976068	0.023932	1.298164503	1.299404	155.5523673	119.7082406
PPIG	0.97607	0.02393	0.788022774	0.787967	1457.893285	1850.198522
DNM1P41	0.976093	0.023907	0.486733278	0.463826	5.048106941	10.89517331
INHA	0.976093	0.023907	0.486733278	0.463826	5.048106941	10.89517331
LINC0010	0.976093	0.023907	0.486733278	0.463826	5.048106941	10.89517331
PDIA2	0.976093	0.023907	0.486733278	0.463826	5.048106941	10.89517331
SCN9A	0.976093	0.023907	0.486733278	0.463826	5.048106941	10.89517331
CDKN2D	0.976108	0.023892	1.289601262	1.290648	177.6933643	137.6753718
HRASLS5	0.976141	0.023859	1.540369081	1.553007	32.30788442	20.79987632
NUDT10	0.976144	0.023856	0.388384462	0.341353	2.150493557	6.319200519
WHSC1	0.976145	0.023855	1.308614439	1.308633	10312.27286	7880.181713
PLA2G16	0.976171	0.023829	1.276750328	1.276812	2803.718595	2195.872657
LINC0051	0.976188	0.023812	1.328101805	1.330068	108.0294885	81.21856466
AKR1A1	0.97619	0.02381	1.269192198	1.269276	2030.348612	1599.609536
NR4A1	0.976203	0.023797	0.734418222	0.732807	58.75996479	80.18847555
ZNF395	0.976204	0.023796	1.288075386	1.288115	4615.988987	3583.521548
FAM196B	0.976212	0.023788	0.77019375	0.769449	118.1257024	153.5228966
LOC10192	0.976222	0.023778	0.602880499	0.594307	13.38757961	22.53319934
STAU2-AS	0.976224	0.023776	0.432087574	0.397623	3.25098087	8.191189387

LOC10192	0.976243	0.023757	0.680282798	0.67663	28.81459442	42.59022293
FAM195B	0.976251	0.023749	0.792616905	0.792531	927.8420557	1170.735896
NUTM2A-/	0.976267	0.023733	1.29250382	1.293589	169.6163932	131.1184584
IKBKE	0.976289	0.023711	0.77619121	0.775594	141.3469943	182.2465353
THY1	0.976313	0.023687	1.540089912	1.553007	32.30788442	20.79987632
NPAS2	0.976317	0.023683	0.79479564	0.794618	446.2526536	561.5966605
STMND1	0.976318	0.023682	0.592215106	0.582678	12.11545666	20.79987632
TTPA	0.976318	0.023682	0.592215106	0.582678	12.11545666	20.79987632
CMTR2	0.97632	0.02368	1.258833565	1.259009	901.5918996	716.1100275
DNAJC9-/	0.976326	0.023674	3.181928292	4.256727	4.2908909	1.000375004
AP1S3	0.976326	0.023674	1.258007379	1.258243	669.0155167	531.7042669
DANCR	0.976337	0.023663	0.777227536	0.776648	145.3854799	187.1988868
MRPL52	0.976342	0.023658	0.794915419	0.794742	457.3584888	575.4830542
SMAD3	0.976358	0.023642	0.776650217	0.776627	3686.127688	4746.333681
C2orf27B	0.976367	0.023633	0.199249566	0.004877	0	2.04036882
SLC26A7	0.976369	0.023631	0.526986287	0.510021	7.067349717	13.86658421
TNFRSF1	0.976369	0.023631	0.526986287	0.510021	7.067349717	13.86658421
SEC31B	0.976374	0.023626	0.770177326	0.769449	118.1257024	153.5228966
NPIPB3	0.976413	0.023587	1.258021433	1.258212	830.6962858	660.2177884
AKAP14	0.976428	0.023572	4.977220393	202.9243	2.019242776	0
BCO1	0.976428	0.023572	4.977220393	202.9243	2.019242776	0
CR1	0.976428	0.023572	4.977220393	202.9243	2.019242776	0
FAM3B	0.976428	0.023572	4.977220393	202.9243	2.019242776	0
FOCAD-A	0.976428	0.023572	4.977220393	202.9243	2.019242776	0
GPIHBP1	0.976428	0.023572	4.977220393	202.9243	2.019242776	0
HAND2	0.976428	0.023572	4.977220393	202.9243	2.019242776	0
HOTS	0.976428	0.023572	4.977220393	202.9243	2.019242776	0
LINC0011	0.976428	0.023572	4.977220393	202.9243	2.019242776	0
LINC0062	0.976428	0.023572	4.977220393	202.9243	2.019242776	0
LINC0070	0.976428	0.023572	4.977220393	202.9243	2.019242776	0
LINC0123	0.976428	0.023572	4.977220393	202.9243	2.019242776	0
LOC10192	0.976428	0.023572	4.977220393	202.9243	2.019242776	0
LOC10272	0.976428	0.023572	4.977220393	202.9243	2.019242776	0
LOC15093	0.976428	0.023572	4.977220393	202.9243	2.019242776	0
NCF1	0.976428	0.023572	4.977220393	202.9243	2.019242776	0
PAEP	0.976428	0.023572	4.977220393	202.9243	2.019242776	0
PIH1D3	0.976428	0.023572	4.977220393	202.9243	2.019242776	0
RFPL1S	0.976428	0.023572	4.977220393	202.9243	2.019242776	0
SIGLEC10	0.976428	0.023572	4.977220393	202.9243	2.019242776	0
SLC25A47	0.976428	0.023572	4.977220393	202.9243	2.019242776	0
SPANXC	0.976428	0.023572	4.977220393	202.9243	2.019242776	0
SPDYE6	0.976428	0.023572	4.977220393	202.9243	2.019242776	0
STARD6	0.976428	0.023572	4.977220393	202.9243	2.019242776	0
SYT8	0.976428	0.023572	4.977220393	202.9243	2.019242776	0
TACR2	0.976428	0.023572	4.977220393	202.9243	2.019242776	0
TBPL2	0.976428	0.023572	4.977220393	202.9243	2.019242776	0
TEX29	0.976428	0.023572	4.977220393	202.9243	2.019242776	0
THRIL	0.976428	0.023572	4.977220393	202.9243	2.019242776	0
TLDC2	0.976428	0.023572	4.977220393	202.9243	2.019242776	0
TMPRSS7	0.976428	0.023572	4.977220393	202.9243	2.019242776	0
TPTE2	0.976428	0.023572	4.977220393	202.9243	2.019242776	0
UBE2U	0.976428	0.023572	4.977220393	202.9243	2.019242776	0
TVP23C	0.976429	0.023571	0.71973562	0.717636	46.60412328	64.94513762
FAM95B1	0.976498	0.023502	2.638157168	3.047669	6.057728329	1.980940602
LOC10050	0.976498	0.023502	2.638157168	3.047669	6.057728329	1.980940602
SLC22A14	0.976498	0.023502	2.638157168	3.047669	6.057728329	1.980940602

KIF23	0.976503	0.023497	1.276456536	1.276516	2881.459442	2257.281815
KMT2C	0.976504	0.023496	1.273323465	1.273392	2484.678236	1951.226493
CCDC82	0.97652	0.02348	0.79464191	0.794533	727.9370209	916.1850282
AICDA	0.976523	0.023477	1.958981495	2.036929	12.13564909	5.952726508
ARGFX	0.976523	0.023477	0.200027099	0.004901	0	2.030464117
PRR23D2	0.976534	0.023466	4.882424685	193.8377	1.928376851	0
LOXL2	0.976548	0.023452	0.777036775	0.777013	3480.164925	4478.9067
KLF8	0.976562	0.023438	1.307988586	1.309472	132.2704981	101.0081613
LIF	0.976583	0.023417	1.270397333	1.270474	2170.685985	1708.561269
COL26A1	0.976588	0.023412	0.487228773	0.463826	5.048106941	10.89517331
SNAI3	0.976588	0.023412	0.487228773	0.463826	5.048106941	10.89517331
TMSB15A	0.976588	0.023412	0.487228773	0.463826	5.048106941	10.89517331
CTHRC1	0.976588	0.023412	0.789404465	0.789061	235.2417834	298.1315605
LOC10192	0.97661	0.02339	0.592425778	0.582678	12.11545666	20.79987632
ZC3H8	0.976646	0.023354	1.261588052	1.262025	367.5021853	291.1982684
HFE	0.976656	0.023344	1.267909103	1.268494	282.6939887	222.8558177
SLC15A4	0.976668	0.023332	0.795648587	0.795516	595.676619	748.7955474
DKKL1	0.976688	0.023312	1.95855575	2.036926	12.11545666	5.942821805
IMPG2	0.976688	0.023312	1.95855575	2.036926	12.11545666	5.942821805
POLR2J2	0.97669	0.02331	0.771286465	0.770562	118.388204	153.6417531
ABCC6P1	0.976693	0.023307	4.862097854	192.8281	1.918280638	0
MAST3	0.976714	0.023286	0.788899256	0.788538	224.1359482	284.2451669
ZNF654	0.976715	0.023285	0.796313751	0.796127	432.1179541	542.7777248
KBTBD8	0.97673	0.02327	0.619238863	0.611758	15.14432082	24.76175752
HSPB1	0.976734	0.023266	0.783012916	0.782978	2374.629505	3032.820061
ABHD14A	0.976743	0.023257	0.738449174	0.736901	60.57728329	82.20903496
MRPL36	0.976763	0.023237	1.256224613	1.256482	609.8113184	485.3304474
MRPS27	0.976776	0.023224	1.285678217	1.285718	4439.305244	3452.779468
RBPMS-A	0.9768	0.0232	1.56975949	1.585304	28.26939887	17.82846541
CHD3	0.976803	0.023197	0.784635091	0.784597	2227.224782	2838.687882
SMIM15	0.976805	0.023195	1.277889684	1.277943	3332.760202	2607.908302
ITGA10	0.976818	0.023182	0.63893451	0.632819	18.17318499	28.72363872
LINC0102	0.976818	0.023182	0.63893451	0.632819	18.17318499	28.72363872
NOS3	0.976818	0.023182	0.63893451	0.632819	18.17318499	28.72363872
PTGER2	0.976818	0.023182	0.63893451	0.632819	18.17318499	28.72363872
CCDC183	0.976834	0.023166	0.690319395	0.687012	31.29826303	45.56163384
SESN3	0.976834	0.023166	0.690319395	0.687012	31.29826303	45.56163384
TSPAN1	0.976868	0.023132	1.302461584	1.302484	8707.984473	6685.67453
LOC28495	0.976868	0.023132	0.321423843	0.242785	1.009621388	4.189689372
IL18BP	0.976878	0.023122	0.72591197	0.723916	49.47144802	68.34245075
TSEN15	0.976879	0.023121	1.259363259	1.259502	1148.94914	912.223147
FAM133C	0.976897	0.023103	0.205397671	0.005177	0	1.921512384
MPC2	0.976911	0.023089	1.257654836	1.257815	985.3904748	783.4124844
MRAP2	0.976917	0.023083	0.690393392	0.687012	31.29826303	45.56163384
RECQL4	0.976949	0.023051	0.796366609	0.796201	475.5316738	597.2535914
PDF	0.97695	0.02305	0.77811602	0.77752	140.7816064	181.0678757
MACC1	0.976953	0.023047	1.527121182	1.539276	32.47952006	21.09701741
DNAH7	0.976958	0.023042	0.746686382	0.745348	68.65425439	92.11373797
NSUN5P1	0.976988	0.023012	0.776151627	0.775512	132.0685738	170.3014635
ESRP2	0.977023	0.022977	1.255316739	1.25554	697.6483792	555.6538387
SMYD5	0.977037	0.022963	1.263037553	1.263138	1647.702105	1304.449386
NT5C	0.977039	0.022961	0.794477328	0.794227	317.0211159	399.1595312
C12orf56	0.977041	0.022959	0.750177797	0.748925	72.69273995	97.06608948
RHOC	0.977043	0.022957	1.295421592	1.295449	6745.280494	5206.902371
ARHGAP4	0.977046	0.022954	0.796622134	0.796461	490.6759946	616.0725271
TSSC1	0.97707	0.02293	1.258953484	1.259364	386.6849917	307.0457932

TMEM260	0.977076	0.022924	0.795531703	0.795284	326.1077084	410.0547045
COMMD3	0.977077	0.022923	1.257727489	1.257878	1048.693736	833.6986616
CENPBD1	0.977082	0.022918	1.257715491	1.257866	1049.26922	834.1641826
PLEKHH2	0.977093	0.022907	1.266386245	1.267012	268.5592893	211.9606444
NKAIN1	0.977111	0.022889	0.753331881	0.752155	76.7312255	102.018441
ENPP4	0.977135	0.022865	0.792061715	0.791724	242.3091332	306.0553229
C1orf216	0.977136	0.022864	0.78009452	0.779504	144.3758585	185.2179462
SLC35F3	0.977146	0.022854	0.781526135	0.780994	155.4816938	199.0845305
TCEAL1	0.977154	0.022846	1.265402615	1.266012	274.6170176	216.9129959
ZNF442	0.977169	0.022831	1.679826605	1.707641	20.30348612	11.88564361
CORO2A	0.977174	0.022826	0.796499087	0.796378	649.1865526	815.1768669
C1orf54	0.977194	0.022806	2.149405609	2.290248	9.086592493	3.961881203
VWF	0.977194	0.022806	2.149405609	2.290248	9.086592493	3.961881203
PXDC1	0.9772	0.0228	0.796975731	0.796829	535.0993357	671.5388639
WWP2	0.9772	0.0228	1.263107572	1.263209	1600.2499	1266.811515
PYROXD2	0.977234	0.022766	0.7770044	0.776367	132.2604018	170.3608917
KIF21B	0.977249	0.022751	0.79671755	0.796594	639.0903387	802.2809436
CHURC1-	0.977259	0.022741	1.262676492	1.263198	309.802323	245.2503512
PDSS2	0.977262	0.022738	1.255239043	1.255551	499.9241266	398.1690609
GABRG3	0.977273	0.022727	1.610606713	1.630512	24.23091332	14.85705451
ABCA2	0.977276	0.022724	0.788047643	0.788001	1743.616137	2212.710652
C1orf106	0.97728	0.02272	0.791214274	0.791154	1333.709854	1685.780452
ABHD1	0.977286	0.022714	0.204007601	0.005023	0	1.980940602
BMX	0.977286	0.022714	0.204007601	0.005023	0	1.980940602
C19orf84	0.977286	0.022714	0.204007601	0.005023	0	1.980940602
C5orf67	0.977286	0.022714	0.204007601	0.005023	0	1.980940602
CD300LG	0.977286	0.022714	0.204007601	0.005023	0	1.980940602
CSF3	0.977286	0.022714	0.204007601	0.005023	0	1.980940602
FAM107A	0.977286	0.022714	0.204007601	0.005023	0	1.980940602
FAM209A	0.977286	0.022714	0.204007601	0.005023	0	1.980940602
GOLGA2F	0.977286	0.022714	0.204007601	0.005023	0	1.980940602
HLA-DPB1	0.977286	0.022714	0.204007601	0.005023	0	1.980940602
KCNH5	0.977286	0.022714	0.204007601	0.005023	0	1.980940602
KCNS1	0.977286	0.022714	0.204007601	0.005023	0	1.980940602
KRTDAP	0.977286	0.022714	0.204007601	0.005023	0	1.980940602
LINC0016	0.977286	0.022714	0.204007601	0.005023	0	1.980940602
LINC0057	0.977286	0.022714	0.204007601	0.005023	0	1.980940602
LINC0120	0.977286	0.022714	0.204007601	0.005023	0	1.980940602
LINC0161	0.977286	0.022714	0.204007601	0.005023	0	1.980940602
LINGO2	0.977286	0.022714	0.204007601	0.005023	0	1.980940602
LOC1005C	0.977286	0.022714	0.204007601	0.005023	0	1.980940602
LOC10192	0.977286	0.022714	0.204007601	0.005023	0	1.980940602
LOC10192	0.977286	0.022714	0.204007601	0.005023	0	1.980940602
LOC64335	0.977286	0.022714	0.204007601	0.005023	0	1.980940602
LOC64732	0.977286	0.022714	0.204007601	0.005023	0	1.980940602
LOC73066	0.977286	0.022714	0.204007601	0.005023	0	1.980940602
LOC73115	0.977286	0.022714	0.204007601	0.005023	0	1.980940602
MIR4307H	0.977286	0.022714	0.204007601	0.005023	0	1.980940602
MMP23A	0.977286	0.022714	0.204007601	0.005023	0	1.980940602
MYO18B	0.977286	0.022714	0.204007601	0.005023	0	1.980940602
NBPF4	0.977286	0.022714	0.204007601	0.005023	0	1.980940602
NKAIN2	0.977286	0.022714	0.204007601	0.005023	0	1.980940602
NPPC	0.977286	0.022714	0.204007601	0.005023	0	1.980940602
NRXN1	0.977286	0.022714	0.204007601	0.005023	0	1.980940602
NTRK1	0.977286	0.022714	0.204007601	0.005023	0	1.980940602
ONECUT1	0.977286	0.022714	0.204007601	0.005023	0	1.980940602

OR1J4	0.977286	0.022714	0.204007601	0.005023	0	1.980940602
PCDHB1	0.977286	0.022714	0.204007601	0.005023	0	1.980940602
PROM1	0.977286	0.022714	0.204007601	0.005023	0	1.980940602
RBMS3-AS	0.977286	0.022714	0.204007601	0.005023	0	1.980940602
RFX8	0.977286	0.022714	0.204007601	0.005023	0	1.980940602
RSPH9	0.977286	0.022714	0.204007601	0.005023	0	1.980940602
SCARNA1	0.977286	0.022714	0.204007601	0.005023	0	1.980940602
SLC24A2	0.977286	0.022714	0.204007601	0.005023	0	1.980940602
SLC4A1	0.977286	0.022714	0.204007601	0.005023	0	1.980940602
SLURP1	0.977286	0.022714	0.204007601	0.005023	0	1.980940602
SNORA52	0.977286	0.022714	0.204007601	0.005023	0	1.980940602
SP7	0.977286	0.022714	0.204007601	0.005023	0	1.980940602
SPATA32	0.977286	0.022714	0.204007601	0.005023	0	1.980940602
SRD5A2	0.977286	0.022714	0.204007601	0.005023	0	1.980940602
SVEP1	0.977286	0.022714	0.204007601	0.005023	0	1.980940602
TAS2R3	0.977286	0.022714	0.204007601	0.005023	0	1.980940602
TAT	0.977286	0.022714	0.204007601	0.005023	0	1.980940602
TDRD10	0.977286	0.022714	0.204007601	0.005023	0	1.980940602
TMC3-AS1	0.977286	0.022714	0.204007601	0.005023	0	1.980940602
TMEM155	0.977286	0.022714	0.204007601	0.005023	0	1.980940602
TMPRSS1	0.977286	0.022714	0.204007601	0.005023	0	1.980940602
TNFRSF1	0.977286	0.022714	0.204007601	0.005023	0	1.980940602
XPNPEP2	0.977286	0.022714	0.204007601	0.005023	0	1.980940602
USP12-AS	0.977292	0.022708	0.390510098	0.340888	2.019242776	5.942821805
EFCAB5	0.977317	0.022683	1.516958205	1.52876	33.31750581	21.79034662
CRLF3	0.977322	0.022678	1.254876294	1.255067	815.1077315	649.4513762
LOC10013	0.977335	0.022665	1.635050908	1.657696	22.61551909	13.63877604
F3	0.977354	0.022646	1.338481959	1.338488	34987.41959	26139.50171
ZBED5-AS	0.977377	0.022623	0.767294962	0.766435	100.9621388	131.73255
NUBPL	0.977379	0.022621	0.792285331	0.79196	245.9740588	310.5916769
C6orf132	0.977391	0.022609	1.256182971	1.25634	995.4967849	792.3762406
DUSP28	0.977395	0.022605	1.480328204	1.489608	38.36561275	25.75222782
MCF2L2	0.977409	0.022591	0.482148912	0.456754	4.523103819	9.914607711
HAUS3	0.977415	0.022585	1.253999949	1.254216	717.6085941	572.1550739
GBP6	0.977436	0.022564	0.204822787	0.005048	0	1.971035899
BCAR3	0.977438	0.022562	1.258040073	1.258173	1191.353238	946.8896075
ZNF492	0.97745	0.02255	1.257568837	1.258019	357.8502048	284.4531657
DHRS2	0.977457	0.022543	0.75855621	0.758549	12962.529	17088.5841
PRRX2	0.97746	0.02254	0.784244609	0.783745	164.5682863	209.9797038
LAIR1	0.977467	0.022533	1.348660721	1.351448	81.91058322	60.6068777
LINC0100	0.977476	0.022524	1.26945909	1.270152	240.2898904	189.1798274
ARHGAP4	0.977487	0.022513	0.390702783	0.340888	2.019242776	5.942821805
CACNA1A	0.977487	0.022513	0.390702783	0.340888	2.019242776	5.942821805
DCDC5	0.977487	0.022513	0.390702783	0.340888	2.019242776	5.942821805
IL10RA	0.977487	0.022513	0.390702783	0.340888	2.019242776	5.942821805
KCNQ2	0.977487	0.022513	0.390702783	0.340888	2.019242776	5.942821805
LOC38878	0.977487	0.022513	0.390702783	0.340888	2.019242776	5.942821805
RNF222	0.977487	0.022513	0.390702783	0.340888	2.019242776	5.942821805
TTLL9	0.977487	0.022513	0.390702783	0.340888	2.019242776	5.942821805
NT5C1B-R	0.9775	0.0225	0.208818866	0.005286	0	1.881893571
BHLHB9	0.977524	0.022476	0.730030709	0.728136	50.48106941	69.33292105
QTRT1	0.977537	0.022463	0.797761452	0.7976	486.6375091	610.1297053
LOC10537	0.977548	0.022452	0.674905519	0.670703	25.2405347	37.63787143
SERPINA1	0.977567	0.022433	0.796884242	0.796775	722.8889139	907.2707955
IRF9	0.977578	0.022422	0.778401108	0.777772	133.2700232	171.351362
DOCK4	0.97758	0.02242	0.712469063	0.709947	39.37523414	55.46633684

ZNF679	0.977585	0.022415	0.205644515	0.005073	0	1.961131196
MOCS1	0.977594	0.022406	0.797433912	0.797249	424.040983	531.8825515
KHNYN	0.977655	0.022345	0.791191907	0.791135	1424.575779	1800.675007
OGT	0.977674	0.022326	0.782405205	0.782375	2740.112447	3502.302984
AKAP6	0.977682	0.022318	1.670857511	1.698305	20.19242776	11.88564361
NT5C3A	0.977684	0.022316	1.259798863	1.25991	1427.604643	1133.098024
LOC65434	0.97769	0.02231	0.75522474	0.754011	75.65093061	100.3346415
ENKD1	0.977698	0.022302	0.795353743	0.79527	946.0152407	1189.554831
ROR1	0.977703	0.022297	1.252766464	1.253012	627.9845034	501.1779722
ZNF473	0.977714	0.022286	1.253935704	1.254114	868.2743938	692.3387402
SELO	0.977714	0.022286	0.796378988	0.796109	298.8479309	375.388244
GRM1	0.97772	0.02228	2.146574759	2.290248	9.086592493	3.961881203
SPAG17	0.97772	0.02228	2.146574759	2.290248	9.086592493	3.961881203
TMEM253	0.97772	0.02228	2.146574759	2.290248	9.086592493	3.961881203
LRFN3	0.977731	0.022269	0.765450062	0.764521	93.8947891	122.8183173
XRCC6BP	0.97774	0.02226	1.27190798	1.27268	218.0782198	171.351362
PON2	0.977755	0.022245	0.795049956	0.79497	994.4770673	1250.96399
GUCY1B3	0.977767	0.022233	0.740643878	0.739051	58.55804051	79.23762406
C4orf3	0.977774	0.022226	0.794285296	0.794212	1086.352614	1367.839485
ISG20	0.977804	0.022196	1.28941719	1.290594	154.4720724	119.6884311
TRAIP	0.977806	0.022194	1.276578089	1.277461	194.8569279	152.5324263
GBP1	0.977813	0.022187	0.780384576	0.779782	138.3181302	177.3833262
ADIPOR2	0.977822	0.022178	1.26775581	1.267829	2271.648123	1791.760774
CLCN2	0.977854	0.022146	0.787925803	0.787479	181.7318499	230.7795801
SNHG9	0.977863	0.022137	1.420905188	1.426946	49.47144802	34.66646053
TSG101	0.977882	0.022118	1.266530916	1.266607	2156.551285	1702.618447
RASSF5	0.97791	0.02209	1.321670786	1.32377	100.9621388	76.26621316
KIAA0408	0.977927	0.022073	4.699483208	184.7511	1.837510926	0
CDK5R1	0.97794	0.02206	1.252667365	1.25298	492.6952374	393.2167094
ANGPTL6	0.977942	0.022058	3.049548495	4.046582	4.038485553	0.990470301
CDO1	0.977942	0.022058	3.049548495	4.046582	4.038485553	0.990470301
GAS1RR	0.977942	0.022058	3.049548495	4.046582	4.038485553	0.990470301
HIST1H2E	0.977942	0.022058	3.049548495	4.046582	4.038485553	0.990470301
HIST1H3E	0.977942	0.022058	3.049548495	4.046582	4.038485553	0.990470301
IL1RL1	0.977942	0.022058	3.049548495	4.046582	4.038485553	0.990470301
LINC0055	0.977942	0.022058	3.049548495	4.046582	4.038485553	0.990470301
LINC0115	0.977942	0.022058	3.049548495	4.046582	4.038485553	0.990470301
LOC10192	0.977942	0.022058	3.049548495	4.046582	4.038485553	0.990470301
LOC10192	0.977942	0.022058	3.049548495	4.046582	4.038485553	0.990470301
OIT3	0.977942	0.022058	3.049548495	4.046582	4.038485553	0.990470301
OR2L13	0.977942	0.022058	3.049548495	4.046582	4.038485553	0.990470301
PIP5K1B	0.977942	0.022058	3.049548495	4.046582	4.038485553	0.990470301
RPA4	0.977942	0.022058	3.049548495	4.046582	4.038485553	0.990470301
SLC5A2	0.977942	0.022058	3.049548495	4.046582	4.038485553	0.990470301
WAS	0.977942	0.022058	3.049548495	4.046582	4.038485553	0.990470301
ALKBH5	0.977942	0.022058	0.790705118	0.790655	1640.634756	2075.03528
BAZ1A	0.977943	0.022057	0.795662117	0.79558	969.2365326	1218.27847
ANKRD20	0.977962	0.022038	1.643836886	1.668342	21.32320372	12.77706688
C12orf57	0.977963	0.022037	0.797754677	0.797646	722.8889139	906.2803252
NBR1	0.977975	0.022025	0.77664807	0.776628	4203.053839	5411.929723
POU2F1	0.977977	0.022023	1.263282834	1.26337	1848.556184	1463.192061
GCDH	0.977977	0.022023	0.78345134	0.782903	150.4335868	192.1512384
DPAGT1	0.977979	0.022021	1.257699439	1.257819	1320.584776	1049.898519
COQ2	0.97798	0.02202	1.253537112	1.253891	437.1660611	348.6455459
C10orf91	0.977994	0.022006	1.321716915	1.32377	100.9621388	76.26621316
USP46-AS	0.978031	0.021969	0.708504797	0.705751	36.34636997	51.50445564

CCDC62	0.978059	0.021941	1.439530889	1.446653	44.42334108	30.70457932
IL1RN	0.978062	0.021938	1.541226296	1.555532	29.27902026	18.81893571
NOTUM	0.978081	0.021919	0.70856649	0.705751	36.34636997	51.50445564
PLAC8	0.978102	0.021898	1.255015448	1.25543	375.5791564	299.1616496
FAM133B	0.978114	0.021886	0.798798879	0.798606	405.3831798	507.6160291
BCL9	0.978118	0.021882	1.251636321	1.251841	750.1486914	599.234532
CBX3P2	0.97812	0.02188	0.634336005	0.627428	16.15394221	25.75222782
FNIP2	0.978151	0.021849	1.251128854	1.251391	582.551541	465.5210414
ACAP1	0.978153	0.021847	0.391742571	0.340888	2.019242776	5.942821805
DMD	0.978153	0.021847	0.391742571	0.340888	2.019242776	5.942821805
TNFRSF1	0.978153	0.021847	0.391742571	0.340888	2.019242776	5.942821805
TNFSF13I	0.978153	0.021847	0.391742571	0.340888	2.019242776	5.942821805
UBAP1L	0.978153	0.021847	0.391742571	0.340888	2.019242776	5.942821805
AGO4	0.978162	0.021838	0.794500542	0.794432	1151.978004	1450.06833
CPTP	0.978204	0.021796	0.799343909	0.799176	465.4354599	582.3965369
CNN2	0.978215	0.021785	0.788958631	0.788915	1859.722597	2357.319316
ANKRD20	0.978219	0.021781	0.442289406	0.406681	3.160114945	7.785096564
AADAT	0.978235	0.021765	0.795024819	0.794686	239.280269	301.1029714
DAPP1	0.978244	0.021756	0.501574614	0.478664	5.058203155	10.57822281
TCEAL3	0.978247	0.021753	1.24980239	1.250023	705.7253503	564.5680714
TFAP4	0.978257	0.021743	1.251101165	1.251306	746.1102059	596.2631211
CNTNAP1	0.97829	0.02171	0.799351653	0.799127	355.3867286	444.721165
ATP6AP1I	0.978305	0.021695	0.744063343	0.742511	59.5676619	80.22809436
RAB11FIP	0.978309	0.021691	0.792747201	0.79269	1391.258273	1755.113373
ZNF431	0.978328	0.021672	1.25036757	1.250703	464.6580515	371.5155051
CD180	0.978332	0.021668	0.634489289	0.627428	16.15394221	25.75222782
PSMG3-A	0.978332	0.021668	0.634489289	0.627428	16.15394221	25.75222782
SCART1	0.978332	0.021668	0.634489289	0.627428	16.15394221	25.75222782
PCBP4	0.978332	0.021668	0.795276194	0.79496	250.3861043	314.9695556
TTC21B	0.978334	0.021666	1.250700143	1.25091	725.9884516	580.3660727
CCL20	0.978361	0.021639	1.250543822	1.250757	714.8119428	571.5013635
TRPV1	0.978371	0.021629	0.75929332	0.758157	78.12450302	103.0485301
FLJ30679	0.978373	0.021627	0.558408592	0.543952	8.076971105	14.85705451
GALNT18	0.978373	0.021627	0.558408592	0.543952	8.076971105	14.85705451
PCDHA2	0.978385	0.021615	0.637552892	0.630771	16.39625134	25.9998454
IGIP	0.978394	0.021606	0.789891052	0.789452	183.7510926	232.7605207
POLQ	0.978397	0.021603	1.250560653	1.250766	742.0717203	593.2917102
IBTK	0.97844	0.02156	1.289646988	1.289676	6444.413321	4996.922667
PVRL4	0.978474	0.021526	0.703886651	0.700856	33.31750581	47.54257444
PCBD2	0.978505	0.021495	1.325421729	1.327674	93.36978598	70.32339135
MAN2A1	0.978512	0.021488	0.793130059	0.793073	1394.287137	1758.084784
LOC34496	0.978541	0.021459	3.008815077	3.986034	3.977908269	0.990470301
ITSN1	0.978583	0.021417	1.266651223	1.26672	2403.908525	1897.741096
CEP44	0.978593	0.021407	1.25033582	1.250524	811.7355961	649.1146163
SIDT1	0.978634	0.021366	0.399301167	0.349018	2.019242776	5.804155963
CFD	0.97864	0.02136	0.795084371	0.794743	232.2129193	292.1887387
HIST2H4A	0.978683	0.021317	0.649950063	0.643917	18.17318499	28.22840357
HIST2H4E	0.978683	0.021317	0.649950063	0.643917	18.17318499	28.22840357
MRPL2	0.978721	0.021279	1.248264852	1.248471	748.1294486	599.234532
MASTL	0.978722	0.021278	1.258666822	1.258764	1629.52892	1294.544683
BHLHE40-	0.978726	0.021274	0.558724969	0.543952	8.076971105	14.85705451
NPTX2	0.978726	0.021274	0.558724969	0.543952	8.076971105	14.85705451
SLC51B	0.978729	0.021271	1.57431242	1.592338	25.2405347	15.84752481
DNAJA2	0.978736	0.021264	0.786939529	0.786904	2314.052222	2940.706323
HIST1H2E	0.978738	0.021262	3.034489114	4.046582	4.038485553	0.990470301
LCT	0.978738	0.021262	3.034489114	4.046582	4.038485553	0.990470301

LINC0122(	0.978738	0.021262	3.034489114	4.046582	4.038485553	0.990470301
OR52E6	0.978738	0.021262	3.034489114	4.046582	4.038485553	0.990470301
SUN3	0.978738	0.021262	3.034489114	4.046582	4.038485553	0.990470301
TEX12	0.978738	0.021262	3.034489114	4.046582	4.038485553	0.990470301
PVT1	0.978748	0.021252	1.292207329	1.293619	133.2700232	103.018816
TRIM35	0.978771	0.021229	0.800545158	0.800411	579.5226768	724.0337899
CASP3	0.978774	0.021226	0.797418185	0.797341	1047.987001	1314.354089
TLR3	0.978783	0.021217	0.714373887	0.711666	37.35599136	52.49492594
SVOP	0.978818	0.021182	0.458260333	0.425424	3.483193789	8.20109409
EFCAB2	0.978819	0.021181	0.765574399	0.764523	84.80819661	110.9326737
LOC10192	0.97883	0.02117	1.267303821	1.268086	211.0108701	166.3990105
VRK3	0.978836	0.021164	1.248446206	1.248681	643.1288243	515.0445564
NDUFB9	0.97886	0.02114	1.261890404	1.261971	1984.915649	1572.866838
GAD1	0.978861	0.021139	0.783830567	0.78323	137.3085088	175.3132432
SETDB2	0.978878	0.021122	1.248255515	1.248517	577.503434	462.5496305
CCDC73	0.978906	0.021094	0.337544319	0.25671	1.009621388	3.961881203
TMEM237	0.978923	0.021077	0.801207461	0.801086	652.2154168	814.1665872
SNORA10	0.978923	0.021077	2.524563521	2.905679	5.77503434	1.980940602
HDAC8	0.978932	0.021068	1.248119863	1.248394	550.2436565	440.7592838
HIF1A-AS:	0.978932	0.021068	0.529259028	0.51008	6.057728329	11.88564361
LIPE	0.978932	0.021068	0.529259028	0.51008	6.057728329	11.88564361
KLLN	0.97895	0.02105	1.62269667	1.646117	21.20204915	12.87611391
PITX2	0.97895	0.02105	1.62269667	1.646117	21.20204915	12.87611391
GOLGA6A	0.978955	0.021045	4.557195392	177.6837	1.766837429	0
WDR91	0.978973	0.021027	0.800420825	0.800317	767.3223512	958.7752511
DDAH2	0.978981	0.021019	0.801492257	0.801367	631.0133676	787.4238891
CHKB	0.978998	0.021002	0.798659473	0.798381	280.6747459	351.5575286
FLJ20021	0.97901	0.02099	1.322823279	1.325089	91.87554632	69.33292105
L3MBTL2	0.979013	0.020987	0.801236142	0.801049	414.9543905	518.0159673
LTA	0.979018	0.020982	1.83335095	1.891765	13.12507805	6.933292105
XKRX	0.979018	0.020982	1.83335095	1.891765	13.12507805	6.933292105
JAZF1	0.979018	0.020982	0.800497093	0.800379	653.2250381	816.1475278
HEPH	0.979021	0.020979	0.481793633	0.453651	4.038485553	8.914232707
ULK4	0.979024	0.020976	1.289743642	1.29113	134.2796446	103.9993816
PCDHGA4	0.979024	0.020976	0.801309244	0.801168	549.0119185	685.2667823
NDUFA12	0.979027	0.020973	1.25179412	1.251933	1108.564284	885.4804489
AFMID	0.979035	0.020965	1.25176194	1.252197	352.9939259	281.8977523
PAIP2	0.979035	0.020965	1.268628533	1.268688	2804.728216	2210.729711
SUPT3H	0.979043	0.020957	0.800810218	0.800607	380.6272633	475.4257444
B4GALT4	0.979044	0.020956	1.254247319	1.254359	1422.556536	1134.088494
LINC0048:	0.979049	0.020951	0.529336424	0.51008	6.057728329	11.88564361
LOC10669	0.979049	0.020951	0.529336424	0.51008	6.057728329	11.88564361
WBSCR27	0.979051	0.020949	1.316225999	1.318298	97.93327465	74.28527256
FAM86C2	0.979059	0.020941	1.26327961	1.263995	226.4176925	179.1265539
EXOSC7	0.979064	0.020936	1.247476664	1.247729	595.676619	477.406685
SLC6A10F	0.979073	0.020927	0.529728063	0.510504	6.067824543	11.89554831
LINC0121:	0.97908	0.02092	1.781359047	1.829411	14.46787449	7.903953
GCLM	0.979087	0.020913	1.260011248	1.260098	1840.085461	1460.270174
USP6	0.979093	0.020907	0.605615347	0.595808	11.65103082	19.56178844
PCDHA11	0.979098	0.020902	4.536868561	176.6741	1.756741215	0
ZNF763	0.979104	0.020896	1.383138432	1.387705	56.6599523	40.8271858
GEM	0.979109	0.020891	1.355617085	1.359043	68.65425439	50.51398534
OSBPL10	0.979109	0.020891	1.260047343	1.260132	1880.924646	1492.638743
GNG2	0.979113	0.020887	0.663085815	0.657747	20.19242776	30.70457932
ABCB4	0.979128	0.020872	0.337846798	0.25671	1.009621388	3.961881203
ACP5	0.979128	0.020872	0.337846798	0.25671	1.009621388	3.961881203

C20orf203	0.979128	0.020872	0.337846798	0.25671	1.009621388	3.961881203
CA7	0.979128	0.020872	0.337846798	0.25671	1.009621388	3.961881203
COLEC12	0.979128	0.020872	0.337846798	0.25671	1.009621388	3.961881203
FAM178B	0.979128	0.020872	0.337846798	0.25671	1.009621388	3.961881203
GPER1	0.979128	0.020872	0.337846798	0.25671	1.009621388	3.961881203
HPX	0.979128	0.020872	0.337846798	0.25671	1.009621388	3.961881203
IL18RAP	0.979128	0.020872	0.337846798	0.25671	1.009621388	3.961881203
IL34	0.979128	0.020872	0.337846798	0.25671	1.009621388	3.961881203
IRF4	0.979128	0.020872	0.337846798	0.25671	1.009621388	3.961881203
LINC0116	0.979128	0.020872	0.337846798	0.25671	1.009621388	3.961881203
LIPE-AS1	0.979128	0.020872	0.337846798	0.25671	1.009621388	3.961881203
LOC10192	0.979128	0.020872	0.337846798	0.25671	1.009621388	3.961881203
LOC10192	0.979128	0.020872	0.337846798	0.25671	1.009621388	3.961881203
LOC10192	0.979128	0.020872	0.337846798	0.25671	1.009621388	3.961881203
LOC39132	0.979128	0.020872	0.337846798	0.25671	1.009621388	3.961881203
LRRC43	0.979128	0.020872	0.337846798	0.25671	1.009621388	3.961881203
MROH2A	0.979128	0.020872	0.337846798	0.25671	1.009621388	3.961881203
MYOZ3	0.979128	0.020872	0.337846798	0.25671	1.009621388	3.961881203
NPC1L1	0.979128	0.020872	0.337846798	0.25671	1.009621388	3.961881203
OXCT1-AS	0.979128	0.020872	0.337846798	0.25671	1.009621388	3.961881203
PCDHGA1	0.979128	0.020872	0.337846798	0.25671	1.009621388	3.961881203
PHF21B	0.979128	0.020872	0.337846798	0.25671	1.009621388	3.961881203
PRKAG2-/	0.979128	0.020872	0.337846798	0.25671	1.009621388	3.961881203
RBP5	0.979128	0.020872	0.337846798	0.25671	1.009621388	3.961881203
SCGB2A1	0.979128	0.020872	0.337846798	0.25671	1.009621388	3.961881203
SLC30A2	0.979128	0.020872	0.337846798	0.25671	1.009621388	3.961881203
SSBP3-AS	0.979128	0.020872	0.337846798	0.25671	1.009621388	3.961881203
ST6GAL2	0.979128	0.020872	0.337846798	0.25671	1.009621388	3.961881203
SULT1C4	0.979128	0.020872	0.337846798	0.25671	1.009621388	3.961881203
ZAP70	0.979128	0.020872	0.337846798	0.25671	1.009621388	3.961881203
HPS4	0.979142	0.020858	0.795754113	0.79569	1242.843929	1561.971664
CIDEC	0.979149	0.020851	0.600680869	0.590359	11.10583527	18.81893571
C6orf163	0.979161	0.020839	0.481905732	0.453651	4.038485553	8.914232707
DLL4	0.979161	0.020839	0.481905732	0.453651	4.038485553	8.914232707
GRIK5	0.979161	0.020839	0.481905732	0.453651	4.038485553	8.914232707
PRDM5	0.979213	0.020787	0.76780993	0.766778	85.81781799	111.923144
KCNK3	0.97924	0.02076	0.662994249	0.657535	20.19242776	30.71448403
GOLGA8T	0.979254	0.020746	0.522002309	0.501314	5.754841913	11.48945549
LRRC14	0.979265	0.020735	0.801441696	0.801229	363.4636997	453.6353978
ANKRD54	0.979278	0.020722	0.802072561	0.801922	513.8972866	640.8342846
GALK1	0.979293	0.020707	0.802001523	0.80186	547.2147924	682.4340372
TNFSF4	0.9793	0.0207	0.628530999	0.620632	14.13469943	22.78081692
FAS	0.979301	0.020699	1.246442345	1.246783	448.2718963	359.5407192
SLC25A34	0.979316	0.020684	1.341035225	1.343974	75.89323975	56.46671185
RASGEF1	0.979365	0.020635	0.628563486	0.620632	14.13469943	22.78081692
GSTP1	0.979373	0.020627	1.30238064	1.302398	11074.53701	8503.187532
CGB5	0.979381	0.020619	4.4962149	174.6549	1.736548788	0
SLC22A16	0.979391	0.020609	1.832118108	1.891765	13.12507805	6.933292105
CYB5R1	0.979399	0.020601	0.792775586	0.792331	179.7126071	226.8176989
RBM45	0.979401	0.020599	0.791188696	0.790709	167.5971504	211.9606444
MRPL47	0.979417	0.020583	1.248745171	1.248891	1060.102458	848.8330478
YBEY	0.979421	0.020579	0.788692099	0.788157	151.4432082	192.1512384
ARHGEF2	0.979422	0.020578	1.27323663	1.274224	171.6457322	134.7039609
CLCF1	0.979429	0.020571	1.249758868	1.250186	355.3867286	284.2649763
TECTA	0.979434	0.020566	0.719968218	0.717363	38.36561275	53.48539624
JAM2	0.97945	0.02055	0.529754478	0.51008	6.057728329	11.88564361

MYH11	0.97945	0.02055	0.529754478	0.51008	6.057728329	11.88564361
ZNF888	0.979465	0.020535	1.245856897	1.246098	618.7060829	496.5128571
PRR7	0.979473	0.020527	0.788689866	0.788157	151.4432082	192.1512384
ST3GAL4	0.979513	0.020487	0.795756996	0.795698	1332.700232	1674.885279
TGFBR2	0.979519	0.020481	0.784479974	0.784453	3145.980246	4010.414248
LOC10192	0.979533	0.020467	1.364533603	1.368436	62.20277372	45.4526821
EXOC6B	0.97954	0.02046	1.252289145	1.252407	1307.459698	1043.955697
C1orf162	0.979579	0.020421	1.697135828	1.732131	17.1635636	9.904703008
LOC44002	0.979579	0.020421	1.697135828	1.732131	17.1635636	9.904703008
LOC10192	0.979595	0.020405	2.128997504	2.284826	8.046682464	3.516169568
CGRRF1	0.979598	0.020402	0.799662564	0.799363	260.4823181	325.864729
GOLGA6L	0.979606	0.020394	0.77717163	0.776363	104.0616765	134.0403458
CDKAL1	0.979613	0.020387	1.245299801	1.245532	639.0398576	513.0636158
WARS	0.979618	0.020382	1.316549592	1.31656	19127.2772	14528.21837
PPP2R2A	0.979625	0.020375	1.272507093	1.272554	3582.136685	2814.916595
FSCN1	0.979629	0.020371	0.785310831	0.785283	2899.632627	3692.473281
ECSCR	0.979637	0.020363	1.45864302	1.467654	36.34636997	24.76175752
ATP6V0A4	0.979642	0.020358	0.482511044	0.453651	4.038485553	8.914232707
NFASC	0.979642	0.020358	0.482511044	0.453651	4.038485553	8.914232707
SLC22A31	0.979642	0.020358	0.482511044	0.453651	4.038485553	8.914232707
CNPY4	0.979645	0.020355	0.773637398	0.77274	94.90441049	122.8183173
FAM188B	0.979646	0.020354	0.705490759	0.702276	31.29826303	44.57116353
PKN3	0.979661	0.020339	1.245063789	1.245336	545.1955496	437.7878729
PRDM10	0.979663	0.020337	0.791695812	0.791208	164.5682863	207.9987632
PTBP2	0.979666	0.020334	1.250691172	1.251174	316.0114945	252.5699267
PDE1C	0.979666	0.020334	0.771887174	0.770945	90.86592493	117.8659658
FANCD2	0.979667	0.020333	1.253632017	1.253738	1461.962059	1166.080685
CMAHP	0.97968	0.02032	1.492961382	1.504491	31.29826303	20.79987632
CLASRP	0.979685	0.020315	0.803893535	0.80374	511.8780438	636.8724034
MICU1	0.979695	0.020305	1.259206978	1.259285	2092.945138	1662.009165
TANC2	0.979701	0.020299	0.793010918	0.792966	1800.154935	2270.157929
ACSL3	0.979707	0.020293	1.273213284	1.273258	3883.003859	3049.658056
LOC10192	0.979716	0.020284	0.753868515	0.752396	62.59652607	83.19950527
SMA5	0.979728	0.020272	1.292492428	1.294057	120.3569657	93.00516124
ZNF76	0.97974	0.02026	0.804010087	0.803823	417.9832547	519.9969079
CNTLN	0.979746	0.020254	1.243532629	1.243808	548.23451	440.7691885
TMEM150	0.979762	0.020238	0.799854811	0.799547	253.4149684	316.9504962
STBD1	0.97978	0.02022	1.255377814	1.256028	245.368286	195.3504574
SPN	0.979793	0.020207	0.338726976	0.256071	1.009621388	3.971785906
LCORL	0.979802	0.020198	1.24675969	1.246924	912.6977349	731.9575523
BPHL	0.979818	0.020182	0.803525491	0.803346	428.0794686	532.8730218
TVP23C-C	0.979823	0.020177	0.79954362	0.799224	243.5913523	304.787521
RPL23AP7	0.979846	0.020154	1.24851423	1.248961	338.1524915	270.7450567
TRDMT1	0.979848	0.020152	1.250074523	1.250602	294.8094453	235.7319316
ASAH2	0.979864	0.020136	0.65987567	0.653974	18.74866918	28.67411521
CABP4	0.979876	0.020124	0.623828549	0.615207	13.05440455	21.22577855
ADD3-AS1	0.979883	0.020117	0.339477603	0.25671	1.009621388	3.961881203
ADGRG3	0.979883	0.020117	0.339477603	0.25671	1.009621388	3.961881203
CNGA4	0.979883	0.020117	0.339477603	0.25671	1.009621388	3.961881203
CTNNA2	0.979883	0.020117	0.339477603	0.25671	1.009621388	3.961881203
EVPLL	0.979883	0.020117	0.339477603	0.25671	1.009621388	3.961881203
IBA57-AS1	0.979883	0.020117	0.339477603	0.25671	1.009621388	3.961881203
RSPH14	0.979883	0.020117	0.339477603	0.25671	1.009621388	3.961881203
SBK3	0.979883	0.020117	0.339477603	0.25671	1.009621388	3.961881203
SLC16A14	0.979883	0.020117	0.339477603	0.25671	1.009621388	3.961881203
SNORA67	0.979883	0.020117	0.339477603	0.25671	1.009621388	3.961881203

TIPARP-A	0.979883	0.020117	0.339477603	0.25671	1.009621388	3.961881203
TKTL1	0.979883	0.020117	0.339477603	0.25671	1.009621388	3.961881203
TMEM145	0.979883	0.020117	0.339477603	0.25671	1.009621388	3.961881203
TMPRSS2	0.979883	0.020117	0.339477603	0.25671	1.009621388	3.961881203
TRIM74	0.979941	0.020059	0.579635498	0.566359	8.914956857	15.74847778
RECK	0.979946	0.020054	1.249447903	1.249569	1282.219163	1026.127232
FAHD2B	0.979951	0.020049	0.680271875	0.675674	22.85782823	33.83446547
LOC28333	0.979957	0.020043	1.308150198	1.310148	98.32702699	75.04793469
PCED1B	0.97996	0.02004	1.253954316	1.254554	258.4630754	206.0178226
ANKRD13	0.979961	0.020039	1.243417718	1.243619	750.1486914	603.1964132
CCDC91	0.979978	0.020022	1.250928103	1.251043	1324.623261	1058.812752
VAV3	0.980023	0.019977	1.245822012	1.246212	382.6465061	307.0457932
ZNF600	0.980029	0.019971	1.243495727	1.243733	621.0181159	499.315888
CHMP1B2	0.980066	0.019934	1.310436264	1.312526	94.90441049	72.30433196
YIF1A	0.980076	0.019924	0.800867099	0.800783	924.8131916	1154.888371
AURKAPS	0.98009	0.01991	1.943993296	2.036578	10.09621388	4.952351504
ENO1-AS1	0.98009	0.01991	1.943993296	2.036578	10.09621388	4.952351504
FAM95A	0.98009	0.01991	1.943993296	2.036578	10.09621388	4.952351504
LOC73018	0.98009	0.01991	1.943993296	2.036578	10.09621388	4.952351504
SPIN2B	0.980108	0.019892	0.776670778	0.775773	96.04528266	123.8087876
PPP1R14(	0.980111	0.019889	1.247524606	1.247664	1081.304507	866.6615132
SLC24A1	0.980126	0.019874	0.797801217	0.797392	195.8665493	245.6366346
SGPP2	0.98013	0.01987	0.803820471	0.803709	703.7061075	875.5757459
C11orf98	0.980145	0.019855	1.246002449	1.246157	963.3100551	773.0224509
CYSRT1	0.980147	0.019853	1.581001512	1.601379	22.21167054	13.86658421
ETV4	0.980152	0.019848	1.248767533	1.248894	1197.410966	958.7752511
ABCB7	0.980156	0.019844	1.2417086	1.241969	574.4745699	462.5496305
CATSPER	0.980157	0.019843	0.585723329	0.573008	9.07649628	15.84752481
GBP4	0.980175	0.019825	1.279192016	1.280426	140.953242	110.0808692
PCSK6-AS	0.980195	0.019805	0.417069775	0.368494	2.190878412	5.962631211
LINC0033(	0.980201	0.019799	0.347099763	0.264629	1.009621388	3.843024767
SOS2	0.980207	0.019793	0.802035651	0.801943	837.9857522	1044.946167
CCL22	0.980223	0.019777	1.938616641	2.03049	10.08611767	4.962256207
TFPI2	0.980247	0.019753	0.790901939	0.790864	2135.349236	2700.02204
CARD8	0.980249	0.019751	1.244335605	1.244696	408.8966622	328.5092847
MKRN1	0.980274	0.019726	0.790011943	0.789977	2309.004115	2922.877858
LINC0092(	0.98028	0.01972	0.433641106	0.389677	2.433187545	6.259772301
LMTK3	0.980297	0.019703	1.301246269	1.303162	101.9717602	78.24715376
PCSK1N	0.9803	0.0197	0.799108822	0.798741	212.0204915	265.4460406
LENG8-AS	0.980311	0.019689	1.442066293	1.450418	37.35599136	25.75222782
STAC3	0.980325	0.019675	0.712239891	0.709167	32.30788442	45.56163384
CCDC155	0.980344	0.019656	2.181724527	2.373826	7.067349717	2.971410902
LGALS4	0.980344	0.019656	2.181724527	2.373826	7.067349717	2.971410902
HSD17B8	0.980372	0.019628	1.242010351	1.242247	618.8979109	498.2065613
PBLD	0.980375	0.019625	1.267512505	1.268486	169.6163932	133.7134906
LOC15368	0.980406	0.019594	1.319447494	1.3219	83.79857522	63.39009925
GBP2	0.980437	0.019563	1.26921443	1.270228	163.5586649	128.7611391
SLF1	0.980446	0.019554	1.241998924	1.242309	472.5028097	380.3405955
GLT8D2	0.980448	0.019552	0.782057852	0.781259	106.0102458	135.6944312
METAP1	0.980459	0.019541	1.259454077	1.259522	2387.754583	1895.760156
STAM	0.980474	0.019526	1.254111707	1.254197	1854.67449	1478.772159
ALS2CL	0.980474	0.019526	1.25093752	1.251037	1569.961259	1254.925871
INPP5K	0.98048	0.01952	0.802641897	0.802322	246.3476187	307.0457932
ZNF230	0.980483	0.019517	1.251290425	1.25189	255.4342112	204.036882
ZSCAN25	0.980487	0.019513	0.795485612	0.794994	164.5682863	207.0082929
TBX2	0.980488	0.019512	0.765752264	0.764526	72.69273995	95.08514887

ADAM20P	0.980493	0.019507	2.181030848	2.373826	7.067349717	2.971410902
CTD-2201	0.980493	0.019507	2.181030848	2.373826	7.067349717	2.971410902
FLJ38576	0.980493	0.019507	2.181030848	2.373826	7.067349717	2.971410902
KLRG1	0.980493	0.019507	2.181030848	2.373826	7.067349717	2.971410902
PAQR5	0.980505	0.019495	1.264591254	1.264648	2883.478685	2280.062632
CFAP58	0.980511	0.019489	1.942090104	2.036578	10.09621388	4.952351504
SNHG25	0.980511	0.019489	1.942090104	2.036578	10.09621388	4.952351504
SNORD17	0.980511	0.019489	1.942090104	2.036578	10.09621388	4.952351504
ZFP37	0.980517	0.019483	0.661484108	0.655411	18.17318499	27.73316842
ZNF341	0.980518	0.019482	0.768175644	0.767048	76.7312255	100.0375004
ORAOV1	0.980521	0.019479	1.241388734	1.241622	625.9652607	504.1493831
PRMT3	0.980539	0.019461	1.253573961	1.253663	1728.471817	1378.734659
LOC10192	0.98054	0.01946	1.270915453	1.27204	152.4528296	119.8469064
PINK1-AS	0.980546	0.019454	0.797697363	0.797276	186.456878	233.8698474
HLA-DRB5	0.980554	0.019446	0.229878102	0.005974	0	1.663990105
NEURL4	0.980556	0.019444	0.805197016	0.804983	356.39635	442.7402244
TPRG1	0.980561	0.019439	1.371445967	1.376008	54.51955496	39.61881203
KRBA2	0.980564	0.019436	1.323847536	1.326559	78.85143042	59.43812275
GSTO2	0.980575	0.019425	0.730509926	0.728146	40.38485553	55.46633684
BABAM1	0.980581	0.019419	1.252117015	1.252212	1612.365357	1287.611391
AKR7A3	0.98059	0.01941	2.174819691	2.365966	7.067349717	2.981315605
VPS11	0.980595	0.019405	0.805097862	0.804986	697.6483792	866.6615132
LINC0024	0.980603	0.019397	1.638877851	1.667391	18.17318499	10.89517331
PCDHGB3	0.980603	0.019397	1.638877851	1.667391	18.17318499	10.89517331
PLPP5	0.98063	0.01937	0.805767627	0.805575	395.7715842	491.2930786
TMEM126	0.980653	0.019347	1.242770919	1.242928	954.0922118	767.6144831
CCDC157	0.980657	0.019343	0.743328231	0.741374	48.46182663	65.37103985
EHD3	0.980658	0.019342	0.795474763	0.794994	164.5682863	207.0082929
HMGA2	0.980665	0.019335	0.797665378	0.79761	1408.583376	1766.008546
DNAJC6	0.980669	0.019331	1.240324773	1.24067	430.0987114	346.6646053
HKR1	0.980675	0.019325	1.24545838	1.245595	1089.381478	874.5852756
ZNF777	0.980695	0.019305	0.806851603	0.806684	463.4162172	574.4727744
ROPN1L	0.980748	0.019252	1.737601	1.782849	14.13469943	7.923762406
ZNF32-AS	0.980748	0.019252	1.737601	1.782849	14.13469943	7.923762406
ERVV-1	0.980755	0.019245	0.642280268	0.634641	14.78085712	23.29586147
OR2A1-AS	0.980758	0.019242	0.629667188	0.621006	12.91305755	20.79987632
EMX2OS	0.980767	0.019233	0.352250237	0.271285	1.070198671	3.971785906
RASSF9	0.98078	0.01922	0.707321321	0.703898	29.27902026	41.59975263
EZH1	0.980786	0.019214	0.805317191	0.80521	728.9466423	905.2898549
C19orf54	0.980791	0.019209	1.242558013	1.242723	887.4672964	714.1290869
GBAP1	0.980798	0.019202	1.293782269	1.295518	106.7371732	82.38731962
ZNF583	0.980802	0.019198	0.782583835	0.781762	102.9813816	131.73255
KLHL30	0.980807	0.019193	1.735559519	1.780626	14.13469943	7.933667109
PLEKHA3	0.980822	0.019178	0.805103306	0.804991	682.5040584	847.8425775
NOCT	0.980831	0.019169	1.265496888	1.266485	165.5981001	130.7519844
ZNF624	0.980856	0.019144	0.78130441	0.780446	98.94289604	126.7801985
RNASEH2	0.980873	0.019127	1.242513705	1.242663	1005.582903	809.2142357
C1orf131	0.980879	0.019121	1.244474407	1.244949	311.9730089	250.5889861
UPF2	0.980892	0.019108	1.253476635	1.253558	1934.43458	1543.152729
GUCD1	0.980892	0.019108	0.799932619	0.799869	1221.64188	1527.305204
LEMD1	0.980898	0.019102	0.684353509	0.679655	22.21167054	32.68551993
MIS12	0.980907	0.019093	0.806450716	0.806246	370.5310495	459.5782196
PIGBOS1	0.980908	0.019092	1.247976526	1.248548	263.4707975	211.0196976
METTL12	0.980925	0.019075	1.426695082	1.434458	38.36561275	26.74269812
PRPH	0.980928	0.019072	0.768326375	0.767153	73.70236134	96.07561918
AQP4-AS1	0.980932	0.019068	0.499357661	0.471339	4.088966622	8.686424538

WSB1	0.980935	0.019065	0.801222346	0.801152	1100.487313	1373.633737
YY2	0.980949	0.019051	0.782562388	0.781762	102.9813816	131.73255
CDNF	0.980949	0.019051	0.718704858	0.715765	33.31750581	46.55210414
TMEM110	0.980955	0.019045	0.807635074	0.807467	460.3469681	570.1147051
PNO1	0.980956	0.019044	1.245761466	1.245885	1201.449452	964.3317895
HYPK	0.980958	0.019042	0.802521989	0.802182	227.0133691	282.9971743
VASP	0.980971	0.019029	1.267009616	1.267057	3552.857665	2804.021421
ENTPD5	0.980974	0.019026	1.239191929	1.239468	522.9838791	421.9403481
COX6C	0.980994	0.019006	1.266037228	1.266088	3242.903899	2561.356198
EYS	0.981002	0.018998	2.177294018	2.373826	7.067349717	2.971410902
SPAG5-AS	0.981002	0.018998	2.177294018	2.373826	7.067349717	2.971410902
STMN2	0.981002	0.018998	2.177294018	2.373826	7.067349717	2.971410902
MKNK2	0.98101	0.01899	1.298165574	1.298182	11257.27848	8671.567483
LOC10050	0.981014	0.018986	0.488014808	0.457185	3.685118067	8.072332951
LOC10028	0.981017	0.018983	0.76293882	0.7616	65.62539023	86.17091617
SLC25A45	0.981022	0.018978	1.426522714	1.434458	38.36561275	26.74269812
FSIP2	0.981025	0.018975	1.310369894	1.312733	85.81781799	65.37103985
ZNF529-A	0.981056	0.018944	1.252936343	1.253651	216.0589771	172.3418323
SLF2	0.981056	0.018944	0.805583683	0.805475	699.667622	868.6424538
LOC64465	0.981068	0.018932	1.736637994	1.782849	14.13469943	7.923762406
PKM	0.981089	0.018911	0.754160441	0.754157	24104.71064	31962.47661
NEBL-AS1	0.981107	0.018893	1.544768428	1.562602	23.22129193	14.85705451
LOC73010	0.981129	0.018871	0.741919045	0.739883	45.43296247	61.40915865
ZDHHC15	0.981129	0.018871	0.236756101	0.006232	0	1.594657184
NSFP1	0.981133	0.018867	4.231966099	161.5298	1.605298007	0
ALDH8A1	0.981136	0.018864	0.565628681	0.549223	7.067349717	12.87611391
LINC0153	0.981136	0.018864	0.565628681	0.549223	7.067349717	12.87611391
LOC10192	0.981136	0.018864	0.565628681	0.549223	7.067349717	12.87611391
NKIRAS1	0.981204	0.018796	1.238844076	1.239159	455.3493423	367.4644816
GPD1L	0.981205	0.018795	0.805728729	0.805624	726.9273995	902.318444
GGACT	0.981209	0.018791	0.789007017	0.7883	117.116081	148.5705451
CHRFAM7	0.98122	0.01878	0.627795662	0.618637	12.22651501	19.7697872
CHERP	0.981221	0.018779	0.801021176	0.800956	1192.362859	1488.676862
RAD21L1	0.981223	0.018777	0.701380749	0.69752	26.25015609	37.63787143
TBC1D10/	0.981266	0.018734	0.800405298	0.799993	187.7895782	234.7414613
NR3C2	0.981282	0.018718	0.803356304	0.803009	221.107084	275.3507436
DNAJB9	0.981286	0.018714	0.808224256	0.808085	552.2628993	683.4245075
M1AP	0.981289	0.018711	1.238561068	1.23874	820.8221886	662.6246312
TMEM44	0.981291	0.018709	0.80393043	0.803571	218.0782198	271.3888624
RNF125	0.981303	0.018697	0.683260604	0.678274	21.43426207	31.6059073
CEP85L	0.981315	0.018685	1.238485455	1.238831	415.9640119	335.769432
CNTROB	0.981316	0.018684	1.236419248	1.236673	574.4745699	464.5305711
C18orf8	0.981319	0.018681	1.240502106	1.240652	990.4385818	798.3190624
SNIP1	0.981363	0.018637	0.808876543	0.808687	405.2620252	501.1383534
SERTAD3	0.981385	0.018615	1.237838784	1.238169	434.1371969	350.6264865
TRIB2	0.981386	0.018614	1.241113535	1.241264	971.2557754	782.4715376
HEBP2	0.981386	0.018614	1.254675567	1.254749	2120.204915	1689.742333
DSTNP2	0.981408	0.018592	1.280875101	1.282359	118.1257024	92.11373797
TSEN2	0.981414	0.018586	1.23905932	1.23945	369.5214281	298.1315605
PRDX3	0.981422	0.018578	1.279995977	1.280025	6013.304988	4697.800637
TLE6	0.981426	0.018574	1.262897767	1.263955	156.4913152	123.8087876
ETS2	0.98143	0.01857	1.257531088	1.257594	2547.274762	2025.511765
LOC10099	0.981435	0.018565	1.35487663	1.359027	56.53879774	41.59975263
PPARA	0.981444	0.018556	0.807367582	0.807246	623.905633	772.8837851
SNX16	0.981471	0.018529	1.236913636	1.237155	590.6285121	477.406685
RRM1	0.981474	0.018526	1.261126754	1.261182	2990.498552	2371.1859

LOC10192	0.981487	0.018513	1.412402496	1.419637	39.37523414	27.73316842
CDRT1	0.9815	0.0185	0.557874451	0.539785	6.411095815	11.88564361
D2HGDH	0.981504	0.018496	0.808423326	0.808272	498.7529658	617.0629974
CFAP54	0.98153	0.01847	1.258205776	1.25916	169.6163932	134.7039609
LOC64690	0.981535	0.018465	0.65921049	0.652515	16.15394221	24.76175752
CYP27C1	0.981541	0.018459	0.774503971	0.773388	77.74084689	100.5228308
TNFSF12-	0.981557	0.018443	4.261320722	166.5779	1.655779077	0
LOC44145	0.981569	0.018431	0.620216513	0.610036	11.06545041	18.14541591
PCDHGA3	0.981574	0.018426	1.528029818	1.544791	23.67562155	15.32257555
MTHFD1	0.981598	0.018402	1.258515088	1.258576	2607.852046	2072.063869
WASIR2	0.981624	0.018376	0.610732541	0.599494	10.09621388	16.84789982
NEU3	0.981628	0.018372	1.256989201	1.257885	175.6741215	139.6563124
TBXA2R	0.981646	0.018354	0.646846702	0.63904	14.37700857	22.50348523
MYOM3	0.981649	0.018351	0.694062713	0.689643	23.22129193	33.67599023
ARGLU1	0.981659	0.018341	0.801006753	0.800949	1381.162059	1724.408794
C9orf50	0.981661	0.018339	0.611081502	0.599847	10.09621388	16.83799511
DNAH14	0.981672	0.018328	1.2365659	1.236906	418.9928761	338.7408429
LAP3	0.981679	0.018321	1.249058301	1.249147	1701.212039	1361.896664
C10orf25	0.981688	0.018312	1.38416492	1.389883	45.43296247	32.68551993
EGOT	0.981688	0.018312	1.58932029	1.613432	19.18280638	11.88564361
WDR11-A	0.981699	0.018301	1.828840127	1.899301	10.86352614	5.715013635
POU2F2	0.981706	0.018294	0.639622804	0.631194	13.12507805	20.79987632
OGG1	0.98171	0.01829	1.246401947	1.247046	232.2129193	186.2084165
ELOVL6	0.98171	0.01829	0.807611693	0.807342	280.6747459	347.6550756
SYN2	0.981712	0.018288	0.81002014	0.809831	405.867798	501.1779722
LOC10050	0.981727	0.018273	0.36490607	0.281911	1.03991003	3.714263628
OSBPL6	0.981734	0.018266	0.807157453	0.806842	245.3379973	304.0743823
MREG	0.981741	0.018259	1.237911896	1.238083	835.9362208	675.1837946
DENND4A	0.981747	0.018253	0.809148223	0.808999	504.8106941	623.9962895
PPL	0.981762	0.018238	0.804599898	0.804522	979.3327465	1217.288
NPHP3-AC	0.981763	0.018237	1.245288426	1.245912	238.6038227	191.5074327
CHGA	0.981803	0.018197	0.533082327	0.510162	5.048106941	9.904703008
GHRLOS	0.981803	0.018197	0.533082327	0.510162	5.048106941	9.904703008
OLFM1	0.981803	0.018197	0.533082327	0.510162	5.048106941	9.904703008
PPP1R36	0.981803	0.018197	0.533082327	0.510162	5.048106941	9.904703008
DNAJC27-	0.981807	0.018193	0.699864822	0.695714	24.47322245	35.18150508
KIF9	0.98182	0.01818	1.323993678	1.326997	69.66387578	52.49492594
AMN1	0.981835	0.018165	1.267503901	1.267546	3913.292501	3087.295928
PPP1R15A	0.98184	0.01816	1.272442607	1.272478	4750.268631	3733.082564
ZCRB1	0.981849	0.018151	1.258196578	1.258256	2674.487057	2125.549265
ALG2	0.981851	0.018149	0.80394248	0.803873	1133.804819	1410.429708
HMGCLL1	0.981865	0.018135	0.474504001	0.437669	3.028864164	6.933292105
LAD1	0.981865	0.018135	0.474504001	0.437669	3.028864164	6.933292105
LOC10192	0.981865	0.018135	0.474504001	0.437669	3.028864164	6.933292105
MYL9	0.981865	0.018135	0.474504001	0.437669	3.028864164	6.933292105
RAET1E-A	0.981865	0.018135	0.474504001	0.437669	3.028864164	6.933292105
WIF1	0.981865	0.018135	0.474504001	0.437669	3.028864164	6.933292105
ZC2HC1A	0.981874	0.018126	0.809062152	0.808931	574.4745699	710.1672057
NCDN	0.98189	0.01811	0.808652056	0.808532	627.0455555	775.5382455
MIAT	0.981892	0.018108	0.470273563	0.432731	3.028864164	7.012529729
KDM4D	0.981912	0.018088	1.292846864	1.294791	94.90441049	73.29480226
NAB1	0.981918	0.018082	0.788756682	0.788731	3270.163676	4146.108679
ETV7	0.981923	0.018077	0.639808985	0.631194	13.12507805	20.79987632
LTB4R2	0.981925	0.018075	0.611328321	0.599847	10.09621388	16.83799511
TCEA3	0.981925	0.018075	0.611328321	0.599847	10.09621388	16.83799511
FAM50B	0.981937	0.018063	0.795449447	0.794851	132.2604018	166.3990105

VPS9D1-A	0.981944	0.018056	0.75924562	0.757647	55.52917635	73.29480226
BANP	0.981948	0.018052	0.788934576	0.788178	106.9491936	135.6944312
PNKP	0.981969	0.018031	1.235406413	1.235608	703.7061075	569.5204229
LOC10028	0.98197	0.01803	0.479672025	0.444483	3.261077084	7.349289632
LGALS3BI	0.981973	0.018027	0.780909189	0.780893	5098.58801	6529.180223
PKI55	0.981982	0.018018	0.721331088	0.718233	31.29826303	43.58069323
BRF2	0.982004	0.017996	1.237057017	1.237487	332.1654367	268.4174515
THBS3	0.982007	0.017993	0.810964971	0.810797	455.3392461	561.5966605
IDNK	0.982012	0.017988	1.248609313	1.249354	202.9439952	162.4371293
WBP4	0.982016	0.017984	1.235667724	1.235857	749.13907	606.1678241
LINC0026	0.98202	0.01798	1.875140938	1.961144	9.70246154	4.942446801
CXorf23	0.982041	0.017959	0.810327119	0.81014	398.8004483	492.2637395
HOXA2	0.982043	0.017957	1.801770453	1.867322	11.10583527	5.942821805
KRT38	0.982043	0.017957	1.801770453	1.867322	11.10583527	5.942821805
PATL2	0.982045	0.017955	1.466090658	1.477795	29.27902026	19.80940602
CCDC96	0.982046	0.017954	0.684825284	0.679665	20.19242776	29.71410902
LENG9	0.982057	0.017943	1.254777139	1.255684	171.635636	136.6849015
ATP5S	0.98206	0.01794	0.789527638	0.788773	107.0299634	135.6944312
TRIM13	0.98206	0.01794	0.809079434	0.808961	635.8797427	786.0471354
PRPF38A	0.982065	0.017935	0.803035275	0.802971	1206.497559	1502.543446
RGS17	0.98207	0.01793	1.310345885	1.312989	76.7312255	58.43774775
SUOX	0.982082	0.017918	1.262155228	1.263256	146.3951013	115.8850252
LINC0135	0.982082	0.017918	1.662005809	1.698109	15.14432082	8.914232707
LIPH	0.982082	0.017918	1.662005809	1.698109	15.14432082	8.914232707
MCM7	0.982095	0.017905	0.796885076	0.796844	1909.194045	2395.947658
MFAP3L	0.982105	0.017895	1.252957156	1.253027	2214.099704	1766.999017
PAXIP1-A	0.982123	0.017877	0.79284536	0.792136	115.0968383	145.3019931
BLZF1	0.982138	0.017862	1.2351952	1.235584	363.473796	294.1696793
PHLDB3	0.982139	0.017861	0.76889323	0.767527	64.61576884	84.18997557
CCDC30	0.98214	0.01786	0.772416679	0.771156	69.29031587	89.85546569
CDKL1	0.982141	0.017859	1.373590601	1.378988	46.44258386	33.67599023
GEMIN4	0.982141	0.017859	0.809765538	0.809638	588.6092693	727.0052008
CNTN4	0.982143	0.017857	2.730470053	3.572283	3.5639635	0.990470301
ANGEL2	0.982152	0.017848	1.236827445	1.236987	895.1202265	723.627697
RNF182	0.982153	0.017847	1.235706741	1.235882	806.6874891	652.7199282
RRAGA	0.982156	0.017844	0.804333314	0.804267	1174.189674	1459.953223
USP54	0.982158	0.017842	1.246063227	1.246156	1606.307629	1289.007954
PGBD4	0.982183	0.017817	0.768863189	0.767527	64.61576884	84.18997557
CCDC115	0.982183	0.017817	0.810576371	0.810364	352.3578645	434.816462
TCF15	0.982186	0.017814	0.684940083	0.679665	20.19242776	29.71410902
GSTCD	0.982187	0.017813	1.23608554	1.23624	937.9382696	758.7002504
LIN7A	0.982187	0.017813	0.533575955	0.510162	5.048106941	9.904703008
C14orf80	0.982194	0.017806	1.233228704	1.233519	482.5990235	391.2357688
SPDL1	0.982202	0.017798	1.257137109	1.257194	2822.901401	2245.396172
SGK494	0.982212	0.017788	1.316151566	1.318983	71.67302235	54.3372007
CHAMP1	0.982225	0.017775	0.809369399	0.809255	657.2635237	812.1856466
SLC24A4	0.982247	0.017753	0.605196235	0.592672	9.288516771	15.67914486
LINC0062	0.982255	0.017745	1.264106629	1.26529	137.4195671	108.6050685
KIF9-AS1	0.982256	0.017744	0.765968516	0.764531	60.57728329	79.23762406
TBC1D26	0.982258	0.017742	1.604615534	1.63221	17.78952886	10.89517331
SSX2IP	0.982264	0.017736	0.810601794	0.810479	622.9363965	768.6049534
MANF	0.982268	0.017732	0.80810752	0.808012	787.5046828	974.622776
CDK13	0.982278	0.017722	0.805615741	0.805541	1015.679116	1260.868693
LCAL1	0.98228	0.01772	1.278471762	1.280063	109.0391099	85.18044587
RHBG	0.982284	0.017716	0.245642669	0.006513	0	1.525324263
CTNNAL1	0.982291	0.017709	1.2599991	1.260051	3086.412584	2449.433054

ST3GAL1	0.982294	0.017706	0.806362461	0.805994	205.9627632	255.5413376
DNAJC2	0.982294	0.017706	1.233914985	1.234113	707.7445931	573.4823041
ZNF619	0.982294	0.017706	1.256667289	1.257707	154.4720724	122.8183173
PEMT	0.982296	0.017704	1.233569635	1.233925	394.7619628	319.9219072
COL18A1	0.982299	0.017701	0.756363866	0.75636	23120.32979	30567.89442
TNFRSF1	0.982306	0.017694	0.791113284	0.79037	108.0294885	136.6849015
OSGEPL1	0.982308	0.017692	1.237221508	1.237754	274.6170176	221.8653474
SLC43A2	0.982314	0.017686	0.642632399	0.634012	12.78180677	20.16597532
FBLL1	0.982338	0.017662	0.47528141	0.437669	3.028864164	6.933292105
LEAP2	0.982338	0.017662	0.47528141	0.437669	3.028864164	6.933292105
UBE2E1-A	0.982338	0.017662	0.47528141	0.437669	3.028864164	6.933292105
SLC25A23	0.982342	0.017658	0.805128452	0.805058	1077.266021	1338.125376
LINC0086	0.982349	0.017651	0.240359175	0.006194	0	1.604561887
DDX41	0.982351	0.017649	0.808217987	0.808124	799.6201394	989.4798305
SFT2D2	0.982353	0.017647	1.261731834	1.26287	141.3469943	111.923144
PEG10	0.982353	0.017647	0.672773973	0.666618	17.1635636	25.75222782
B4GALNT	0.982359	0.017641	0.776288185	0.775143	73.70236134	95.08514887
LOH12CR	0.982381	0.017619	1.522995201	1.540585	22.78715473	14.78772159
RAB6C	0.982386	0.017614	0.480186507	0.443169	3.018767951	6.824340372
DNMT3A	0.982396	0.017604	1.233218088	1.233583	383.6561275	311.0076744
LOC10192	0.982401	0.017599	0.246851854	0.006556	0	1.51541956
RRN3P1	0.982414	0.017586	0.618709668	0.607558	10.3284268	17.00637506
LOC10192	0.982419	0.017581	4.122118008	159.5106	1.585105579	0
NUDT18	0.982441	0.017559	1.238978176	1.239582	244.3283759	197.1035899
EMILIN2	0.982442	0.017558	1.232458692	1.23268	630.0037462	511.0826752
MRPL9	0.982444	0.017556	1.232074994	1.232275	712.7927	578.4346557
DDX39A	0.982452	0.017548	1.233176966	1.233374	709.7638359	575.4632447
BLOC1S5	0.982475	0.017525	0.810196338	0.810091	723.3432435	892.9188809
NET1	0.982483	0.017517	1.265238017	1.26528	3916.321365	3095.21969
MCM2	0.982497	0.017503	0.791515724	0.791488	2872.372849	3629.083182
SDCBP2	0.982503	0.017497	0.730488164	0.727655	33.53962251	46.0964878
STAMBPL	0.982503	0.017497	0.74959685	0.747555	44.42334108	59.42821805
EXOC5	0.982509	0.017491	0.798004618	0.797963	1896.068967	2376.138252
ZNF788	0.982516	0.017484	0.794357336	0.79364	113.2795198	142.736675
MIR762HG	0.982521	0.017479	0.727880716	0.724922	32.30788442	44.57116353
SCEL	0.982521	0.017479	1.445553684	1.455974	30.28864164	20.79987632
CREG2	0.982523	0.017477	1.29006649	1.292074	90.86592493	70.32339135
HSPB11	0.982525	0.017475	1.242209519	1.242313	1417.508429	1141.021786
C20orf96	0.982527	0.017473	1.231316846	1.231689	380.6272633	309.0267338
ZNF605	0.982528	0.017472	0.809784995	0.809679	705.7253503	871.6138647
GPR1	0.982532	0.017468	1.39630856	1.403261	38.92090451	27.73316842
DOCK6	0.98254	0.01746	0.804918138	0.804852	1147.939518	1426.277233
SLC29A3	0.982541	0.017459	1.231490873	1.231693	702.6964862	570.5108932
ENOPH1	0.982552	0.017448	1.250223696	1.250296	2115.156808	1691.723274
OR2A20P	0.98256	0.01744	0.630085198	0.62	11.30775955	18.24446294
SHKBP1	0.982572	0.017428	1.249262077	1.249335	2133.329993	1707.570799
NOP10	0.982581	0.017419	1.241645111	1.24175	1392.267894	1121.21238
LOC10192	0.982596	0.017404	1.547103789	1.567767	20.19242776	12.87611391
TAF1D	0.982608	0.017392	1.247273154	1.247351	1960.684736	1571.876367
GOLT1A	0.982612	0.017388	1.238991489	1.239582	244.3283759	197.1035899
DENND2D	0.982617	0.017383	1.232095787	1.232299	683.5136798	554.6633684
FAM84A	0.982624	0.017376	0.401993977	0.335219	1.484143441	4.44721165
CLEC18A	0.982655	0.017345	0.467390327	0.425383	2.614919395	6.160725271
HIST2H2E	0.982656	0.017344	0.392193562	0.317716	1.251930521	3.961881203
NLRP12	0.982664	0.017336	0.542444505	0.519726	5.199550149	10.01365474
CISD1	0.982668	0.017332	1.231793373	1.231998	678.4655728	550.7014872

CH17-340	0.982679	0.017321	0.656497813	0.648829	14.13469943	21.79034662
ETV2	0.982685	0.017315	0.596915618	0.582778	8.076971105	13.86658421
LOC10192	0.982685	0.017315	0.596915618	0.582778	8.076971105	13.86658421
PRKG2	0.982685	0.017315	0.596915618	0.582778	8.076971105	13.86658421
ROR2	0.982685	0.017315	0.596915618	0.582778	8.076971105	13.86658421
ZMYND15	0.982685	0.017315	0.596915618	0.582778	8.076971105	13.86658421
IP6K2	0.982685	0.017315	0.812280517	0.812129	490.6759946	604.1868835
LOC40092	0.982694	0.017306	0.69545612	0.69066	21.33329993	30.89276868
DIAPH3	0.982701	0.017299	1.233106021	1.233267	890.4860644	722.0528493
SLC11A2	0.982706	0.017294	0.799572374	0.799529	1848.616762	2312.134061
GPRASP2	0.982712	0.017288	0.701955262	0.697548	22.94869415	32.90342339
CEP131	0.982719	0.017281	0.813241957	0.813085	481.5894022	592.3012399
C21orf62	0.98272	0.01728	1.582692713	1.608303	17.79962507	11.06355326
LOC10050	0.982727	0.017273	0.368021671	0.281258	1.009621388	3.615216598
DDIT3	0.982737	0.017263	1.23866943	1.238787	1219.622637	984.527479
MRI1	0.982739	0.017261	0.812504412	0.812287	340.2726965	418.909509
ARRDC2	0.982747	0.017253	0.812708825	0.812544	448.2718963	551.6919575
PNPLA7	0.982753	0.017247	0.695444154	0.690618	21.20204915	30.70457932
MRPL22	0.982765	0.017235	1.230537472	1.2308	522.9838791	424.911759
HLA-DMA	0.98277	0.01723	0.762589134	0.760946	53.50993357	70.32339135
A1BG	0.982772	0.017228	0.797254937	0.79658	119.1353238	149.5610154
WLS	0.982773	0.017227	0.811370894	0.811262	700.6772434	863.6901023
NKAP	0.982775	0.017225	0.810090383	0.80999	746.1102059	921.1373797
SCAF1	0.982778	0.017222	0.803842972	0.803786	1389.23903	1728.370675
SLC39A10	0.982788	0.017212	0.802902883	0.80285	1490.201169	1856.141344
UBR5-AS1	0.982798	0.017202	1.403602946	1.411228	36.34636997	25.75222782
ID2-AS1	0.98282	0.01718	1.484416368	1.498726	25.2405347	16.83799511
RSC1A1	0.982821	0.017179	1.231209385	1.231418	661.8875897	537.4985181
ZDHHC11	0.982823	0.017177	0.633147521	0.623139	11.10583527	17.82846541
MARVELD	0.98283	0.01717	1.228596737	1.228888	479.5701594	390.2452985
UBE3D	0.982838	0.017162	1.275574494	1.277206	105.0006244	82.20903496
ZNF703	0.982842	0.017158	0.810768377	0.810432	227.1648123	280.3030951
ZSWIM3	0.982842	0.017158	0.758653681	0.756816	49.47144802	65.37103985
APOOL	0.98285	0.01715	1.230789801	1.230996	669.3789804	543.7681951
NT5C3B	0.982852	0.017148	0.805614212	0.805549	1164.093461	1445.096169
WHAMMP	0.982873	0.017127	1.248705436	1.249595	170.0606266	136.0906193
TAPT1-AS	0.98288	0.01712	0.695551566	0.690618	21.20204915	30.70457932
ESPL1	0.982884	0.017116	1.233087572	1.233249	867.2647724	703.2339135
MAPK8IP3	0.982889	0.017111	0.804499126	0.804439	1281.209542	1592.676244
CPT1B	0.98289	0.01711	0.81329706	0.813121	418.9928761	515.292174
PRPF8	0.982893	0.017107	0.791887882	0.791861	2973.334988	3754.87291
MIATNB	0.982893	0.017107	0.600169893	0.586125	8.076971105	13.78734659
NME1-NM	0.982903	0.017097	1.232007225	1.232183	793.4715452	643.954266
FAM227A	0.982905	0.017095	0.78624475	0.785298	86.24185898	109.8233469
MRPL45P	0.982913	0.017087	1.337091813	1.341071	55.28686722	41.22337392
BGLAP	0.982925	0.017075	3.898289179	144.3662	1.433662371	0
IGFLR1	0.982932	0.017068	0.711885152	0.707954	25.2405347	35.65693083
IFITM3	0.982949	0.017051	0.776091677	0.776081	7640.501683	9844.987553
CASP2	0.982959	0.017041	0.812765898	0.812645	627.8128678	772.5569299
PCDHB11	0.982968	0.017032	0.712530473	0.70862	25.34149684	35.76588256
FOCAD	0.98297	0.01703	0.81415044	0.813992	475.5316738	584.1991928
ST7-AS1	0.982973	0.017027	0.597224306	0.582778	8.076971105	13.86658421
ARSJ	0.982979	0.017021	0.813064143	0.812826	309.9537662	381.3310658
C15orf52	0.98301	0.01699	1.246426318	1.246504	1933.424958	1551.076491
SEC14L2	0.98302	0.01698	1.237646123	1.237762	1235.776579	998.3940632
LYRM4-AS	0.983034	0.016966	0.734160387	0.731321	33.31750581	45.56163384

PPFIBP2	0.983034	0.016966	0.734160387	0.731321	33.31750581	45.56163384
BMP7	0.983045	0.016955	0.674154711	0.667624	16.56788698	24.82118574
EXOSC2	0.98305	0.01695	1.233507014	1.233654	953.0825904	772.5668346
LOC10192	0.983056	0.016944	0.633367759	0.623139	11.10583527	17.82846541
CECR7	0.983057	0.016943	1.922960758	2.036056	8.076971105	3.961881203
DHDH	0.983057	0.016943	1.922960758	2.036056	8.076971105	3.961881203
DRD4	0.983057	0.016943	1.922960758	2.036056	8.076971105	3.961881203
MTTP	0.983057	0.016943	1.922960758	2.036056	8.076971105	3.961881203
OR2A9P	0.983069	0.016931	1.614921181	1.646428	15.62893909	9.488705481
TRPM7	0.983076	0.016924	0.805570315	0.805508	1228.052975	1524.571506
LINC0108	0.983078	0.016922	1.426611886	1.436136	31.29826303	21.79034662
NDUFAF4	0.983079	0.016921	1.231840807	1.23201	818.8029458	664.6055718
C8orf58	0.983079	0.016921	0.807776204	0.807358	180.7222285	223.846288
TMEM189	0.983079	0.016921	0.635933638	0.625948	11.3481444	18.13551121
ULK4P3	0.98308	0.01692	0.623856917	0.612566	10.14669495	16.57056813
FOXN2	0.983093	0.016907	1.240571525	1.24067	1477.076091	1190.545302
PCDHB6	0.983093	0.016907	1.342483029	1.346805	51.93492421	38.55900881
C1orf112	0.983106	0.016894	1.232098428	1.232576	291.7805812	236.7224019
GCC1	0.983122	0.016878	0.811033483	0.810935	758.2256625	935.0039639
SLC25A33	0.983136	0.016864	0.813981514	0.813794	391.7330986	481.3685662
QPRT	0.98315	0.01685	1.262446146	1.262488	3864.951828	3061.37532
ADPRH	0.983154	0.016846	0.685399986	0.679677	18.17318499	26.74269812
C11orf70	0.983155	0.016845	1.230123939	1.230542	329.3384968	267.63498
LINC0151	0.983155	0.016845	2.250166204	2.555775	5.078395582	1.980940602
CEP295	0.983158	0.016842	1.236919136	1.237035	1227.699608	992.4512414
SNHG6	0.983158	0.016842	1.232964426	1.233103	1030.823437	835.9569339
TMEM17	0.983167	0.016833	1.25346465	1.254525	145.3955761	115.8949299
ZNF252P	0.983176	0.016824	0.811033859	0.810937	772.693537	952.842334
GTPBP8	0.983185	0.016815	1.233869048	1.23446	243.3187545	197.1035899
LMF2	0.983201	0.016799	0.808380437	0.808308	1071.208293	1325.249262
TMEM91	0.983204	0.016796	0.79365582	0.792833	98.94289604	124.7992579
MTERF3	0.983211	0.016789	1.233187194	1.23333	983.3712321	797.3285921
PDXP	0.983225	0.016775	0.814418217	0.814282	553.2725207	679.4626263
PAFAH1B	0.983243	0.016757	0.788991956	0.788971	3865.840295	4899.856578
SUSD1	0.983253	0.016747	0.804698491	0.804153	143.3662371	178.2846541
LYPLA1	0.983262	0.016738	1.274245788	1.274274	6131.43069	4811.704721
MMP24	0.983271	0.016729	1.227912493	1.22826	391.7330986	318.9314368
BARD1	0.983272	0.016728	1.227670796	1.227873	689.5714081	561.5966605
FABP3	0.983286	0.016714	1.3308931	1.334763	55.52917635	41.59975263
SLCO1B7	0.983286	0.016714	1.3308931	1.334763	55.52917635	41.59975263
LOC10050	0.983292	0.016708	1.252726276	1.25385	140.337373	111.923144
SPRED3	0.983298	0.016702	1.228091048	1.228519	326.1077084	265.4460406
PLEKHA8	0.983303	0.016697	0.811207907	0.811114	791.765285	976.1481002
ALDH1A3	0.983305	0.016695	0.809049163	0.808971	955.1018332	1180.640599
TLR1	0.983313	0.016687	0.465305907	0.420601	2.493764829	5.942821805
PARP16	0.983313	0.016687	0.813286344	0.81301	266.5400465	327.8456696
IFNGR1	0.983319	0.016681	0.791053874	0.79103	3326.702474	4205.536897
ZNF879	0.983319	0.016681	1.232799122	1.233384	244.3283759	198.0940602
GAB2	0.983328	0.016672	1.241700605	1.241789	1645.682863	1325.249262
FLJ21408	0.983332	0.016668	2.237941381	2.540561	5.048106941	1.980940602
HIST1H2A	0.983332	0.016668	2.237941381	2.540561	5.048106941	1.980940602
LINC0053	0.983332	0.016668	2.237941381	2.540561	5.048106941	1.980940602
LINC0065	0.983332	0.016668	2.237941381	2.540561	5.048106941	1.980940602
LINC0114	0.983332	0.016668	2.237941381	2.540561	5.048106941	1.980940602
LINC0114	0.983332	0.016668	2.237941381	2.540561	5.048106941	1.980940602
LOC10012	0.983332	0.016668	2.237941381	2.540561	5.048106941	1.980940602

MAGEB16	0.983332	0.016668	2.237941381	2.540561	5.048106941	1.980940602
NUDT9P1	0.983332	0.016668	2.237941381	2.540561	5.048106941	1.980940602
NXPE2	0.983332	0.016668	2.237941381	2.540561	5.048106941	1.980940602
OR2H1	0.983332	0.016668	2.237941381	2.540561	5.048106941	1.980940602
PCDHB19	0.983332	0.016668	2.237941381	2.540561	5.048106941	1.980940602
TRPM8	0.983332	0.016668	2.237941381	2.540561	5.048106941	1.980940602
MAP3K3	0.983344	0.016656	0.811256448	0.811163	798.610518	984.527479
PLXNA2	0.983357	0.016643	0.796866774	0.796138	108.0294885	135.6944312
CLCN4	0.983373	0.016627	1.241302631	1.242088	185.7703354	149.5610154
HEXIM2	0.983394	0.016606	0.802398485	0.801806	130.2411591	162.4371293
PLEKHG4	0.983405	0.016595	0.815238497	0.815051	390.7234772	479.3876256
FOXD1	0.983406	0.016594	1.231291837	1.231434	996.4963101	809.2142357
MAPK6	0.983426	0.016574	0.797760826	0.797726	2242.369103	2810.954714
OPLAH	0.983429	0.016571	0.787359965	0.786365	81.77933244	103.9993816
ABHD17A	0.98343	0.01657	0.814660399	0.814519	517.9357721	635.8819331
TMEM232	0.98344	0.01656	1.459293945	1.472108	26.25015609	17.82846541
PRR19	0.983447	0.016553	1.697466256	1.746356	12.11545666	6.933292105
FOXRED2	0.983457	0.016543	0.811590344	0.811497	800.5792797	986.5480384
TDRD9	0.983457	0.016543	1.920687614	2.036056	8.076971105	3.961881203
DLL3	0.98346	0.01654	0.720068159	0.716367	26.25015609	36.64740113
POLA2	0.983466	0.016534	1.236597895	1.236707	1304.36016	1054.7023
CEP192	0.983469	0.016531	1.241331585	1.241419	1664.865669	1341.096787
TNNC1	0.983469	0.016531	0.785985229	0.78491	77.74084689	99.04703008
NAA30	0.983483	0.016517	0.80942812	0.809352	988.419339	1221.249881
LRRC32	0.983494	0.016506	0.731291856	0.728162	30.28864164	41.59975263
DCP1B	0.983494	0.016506	1.237056746	1.23775	205.9627632	166.3990105
DPYSL2	0.983504	0.016496	0.799294695	0.799257	2060.637253	2578.194193
SEMA3E	0.983507	0.016493	1.746895599	1.807961	10.75246778	5.942821805
ASH1L-AS	0.983507	0.016493	0.67193674	0.664931	15.14432082	22.78081692
GOLGA6L	0.983516	0.016484	1.254160922	1.255324	133.5022362	106.3467962
RBM3	0.983525	0.016475	0.806573658	0.806513	1255.363234	1556.533982
SNAI1	0.983531	0.016469	0.777991203	0.776663	64.61576884	83.19950527
LDLRAP1	0.983531	0.016469	0.811864183	0.811771	797.6008966	982.5465384
SLC25A12	0.983536	0.016464	0.816554351	0.816355	373.5599136	457.597279
PPTC7	0.983548	0.016452	1.243511111	1.243588	1970.78095	1584.752481
LOC10192	0.983552	0.016448	0.703749116	0.699034	21.36358857	30.56591348
IL2RB	0.983556	0.016444	1.510318553	1.528622	21.20204915	13.86658421
SLC2A13	0.983557	0.016443	1.23420309	1.234324	1165.103082	943.9181966
TMEM61	0.983574	0.016426	0.720166433	0.716367	26.25015609	36.64740113
KLF14	0.983582	0.016418	0.575359783	0.556408	6.057728329	10.89517331
MGARP	0.983582	0.016418	0.575359783	0.556408	6.057728329	10.89517331
PAPPA2	0.983582	0.016418	0.575359783	0.556408	6.057728329	10.89517331
RNF112	0.983582	0.016418	0.575359783	0.556408	6.057728329	10.89517331
VWA3B	0.983582	0.016418	0.575359783	0.556408	6.057728329	10.89517331
LOC10013	0.983588	0.016412	0.77300717	0.771546	58.30563517	75.57288395
ESYT2	0.983589	0.016411	0.796736835	0.796705	2454.318921	3080.590444
PITX1	0.983598	0.016402	0.815603306	0.815471	565.3879774	693.3292105
AVEN	0.983607	0.016393	0.800604872	0.799909	114.0872169	142.6277233
MAP3K14	0.983611	0.016389	1.232441635	1.232573	1062.1217	861.7091617
MRPL53	0.983617	0.016383	1.225647453	1.225892	551.2532779	449.6735166
SNAPIN	0.983622	0.016378	0.812740135	0.812647	813.7548389	1001.365474
CYSTM1	0.983626	0.016374	1.24837616	1.248441	2327.1773	1864.065106
PLCE1	0.983649	0.016351	1.235964377	1.236072	1318.565533	1066.736514
SIMC1	0.983657	0.016343	0.813847544	0.813737	668.3188779	821.2979734
POMK	0.983665	0.016335	1.247650882	1.248665	148.4143441	118.8564361
MBNL1-AS	0.983667	0.016333	0.76234821	0.760495	47.45220524	62.39962895

CCDC148	0.983674	0.016326	1.336786269	1.34114	50.48106941	37.63787143
DCUN1D4	0.983679	0.016321	1.239207591	1.239299	1569.961259	1266.811515
ITIH4	0.983679	0.016321	0.775603162	0.774208	60.57728329	78.24715376
MON1A	0.983679	0.016321	0.775603162	0.774208	60.57728329	78.24715376
LILRB3	0.983682	0.016318	1.998987653	2.155813	6.673597376	3.090267338
TMEM217	0.983682	0.016318	0.67209181	0.664931	15.14432082	22.78081692
ZMYND10	0.983682	0.016318	0.67209181	0.664931	15.14432082	22.78081692
B4GALT6	0.983692	0.016308	0.816188578	0.815969	332.1654367	407.0832936
ARHGEF1	0.983698	0.016302	1.236154324	1.236862	200.9146562	162.4371293
PAQR6	0.983701	0.016299	1.230522879	1.23107	252.405347	205.0273523
HIP1	0.983702	0.016298	0.814074872	0.813964	662.0390329	813.3544016
TTLL3	0.983707	0.016293	0.810101116	0.80964	165.7798319	204.7599253
APAF1	0.983717	0.016283	1.224528088	1.224913	355.5179794	290.2375122
ARIH2OS	0.983726	0.016274	1.343032385	1.347734	47.84595758	35.49845558
GLIS2	0.983741	0.016259	0.799433555	0.799397	2099.002866	2625.736767
HIST2H3A	0.983742	0.016258	3.022876812	5.015592	2.52405347	0.49523515
HIST2H3C	0.983742	0.016258	3.022876812	5.015592	2.52405347	0.49523515
SLC3A1	0.983748	0.016252	1.696434438	1.746356	12.11545666	6.933292105
TMEM218	0.983748	0.016252	1.227128123	1.227624	279.6651245	227.8081692
GLMN	0.983755	0.016245	1.248709244	1.249772	142.3566157	113.9040846
TRIB3	0.983765	0.016235	0.782834483	0.78282	5727.582135	7316.604112
COLGALT	0.983776	0.016224	0.652950529	0.64398	12.11545666	18.81893571
RAB42	0.983777	0.016223	0.455297554	0.40292	2.02933899	5.051398534
GSR	0.983782	0.016218	1.257895886	1.25794	3560.934636	2830.76412
LOC1005C	0.98379	0.01621	0.772927703	0.77142	56.53879774	73.29480226
ZNF628	0.983791	0.016209	0.809811422	0.809331	159.5201793	197.1035899
DTX2P1-U	0.983792	0.016208	0.729451174	0.726056	28.78430578	39.64852614
MAGEH1	0.983798	0.016202	0.788260778	0.787231	78.75046828	100.0375004
RBM48	0.983798	0.016202	1.238505629	1.239297	181.7318499	146.639128
C1orf198	0.983802	0.016198	0.814240873	0.814139	741.0620989	910.2422064
EPHX4	0.983812	0.016188	1.30742776	1.31051	63.60614745	48.53304474
KDM8	0.983812	0.016188	1.30742776	1.31051	63.60614745	48.53304474
CLIC5	0.983817	0.016183	0.69711641	0.691803	19.18280638	27.73316842
TMPO-AS	0.98382	0.01618	1.28869178	1.291109	76.7312255	59.42821805
SMPX	0.983836	0.016164	2.232465573	2.540561	5.048106941	1.980940602
TAS2R31	0.983836	0.016164	2.232465573	2.540561	5.048106941	1.980940602
SOD2	0.983837	0.016163	0.767208926	0.767203	14337.63333	18688.19364
NACC2	0.983848	0.016152	0.813203677	0.813113	835.9665094	1028.108172
PGGT1B	0.98385	0.01615	1.224306659	1.224551	548.2244138	447.692576
NUFIP1	0.983861	0.016139	1.222861414	1.223196	405.867798	331.8075508
COX15	0.983861	0.016139	1.229872423	1.230017	949.0542011	771.5763643
LBX2	0.983866	0.016134	0.796408852	0.795596	96.92365326	121.827847
SLC6A4	0.983872	0.016128	1.825650825	1.911724	9.116881135	4.764162147
A2M-AS1	0.983878	0.016122	0.624165914	0.611862	9.086592493	14.85705451
APOC1	0.983878	0.016122	0.624165914	0.611862	9.086592493	14.85705451
TYW5	0.983883	0.016117	1.224749378	1.224967	615.1421194	502.1684425
CES4A	0.983903	0.016097	0.575769721	0.556408	6.057728329	10.89517331
DNALI1	0.983903	0.016097	0.575769721	0.556408	6.057728329	10.89517331
MIR99AH(	0.983903	0.016097	0.575769721	0.556408	6.057728329	10.89517331
COX20	0.983908	0.016092	1.226707854	1.227176	289.2868163	235.7319316
PRKRIP1	0.983912	0.016088	1.225290882	1.225705	324.2197164	264.5149985
MST1R	0.983924	0.016076	1.235228806	1.235333	1356.931146	1098.431564
LOC10028	0.983927	0.016073	1.246740394	1.247785	143.3662371	114.8945549
PTCH1	0.983931	0.016069	0.809800827	0.809331	159.5201793	197.1035899
TGDS	0.983936	0.016064	1.222481537	1.222735	534.0897143	436.7974026
MYOM1	0.983938	0.016062	1.269107058	1.270824	96.92365326	76.26621316

SLC52A2	0.983939	0.016061	0.813581758	0.813491	829.9087811	1020.18441
USP20	0.983943	0.016057	0.815075999	0.814963	648.1769312	795.3476515
BNC2	0.98395	0.01605	1.223840612	1.224078	561.3494918	458.5877493
ACRC	0.983951	0.016049	0.714780092	0.710534	23.22129193	32.68551993
CACNA2D	0.983956	0.016044	0.804335693	0.803719	124.1834307	154.5133669
TNS3	0.983963	0.016037	0.806981741	0.806927	1411.450701	1749.170551
PMS2CL	0.983967	0.016033	0.81231525	0.81188	174.0789197	214.4170107
LOC10065	0.983968	0.016032	1.293010705	1.295579	71.91533148	55.50595566
CDK2	0.983971	0.016029	1.239073924	1.239161	1661.836805	1341.096787
MAGOH	0.983977	0.016023	1.232662286	1.232779	1195.391724	969.6704245
IL20RB	0.983977	0.016023	1.242900638	1.242973	2081.839302	1674.885279
ZNF790	0.983981	0.016019	1.225340755	1.225829	281.6843673	229.7891098
LOC10192	0.983984	0.016016	0.653148201	0.64398	12.11545666	18.81893571
PTPRCAP	0.983984	0.016016	0.653148201	0.64398	12.11545666	18.81893571
CCDC102	0.983989	0.016011	0.727855979	0.724338	27.25977748	37.63787143
VWA5A	0.983992	0.016008	0.798866149	0.7981	101.9717602	127.7706688
TIA1	0.983995	0.016005	0.806369298	0.806315	1387.219787	1720.446912
ERO1A	0.983995	0.016005	0.794960666	0.794934	3032.90265	3815.291599
FANCE	0.984003	0.015997	0.809351547	0.808831	147.4047227	182.2465353
PRSS16	0.984009	0.015991	1.223404698	1.223646	550.2436565	449.6735166
LRRK1	0.984012	0.015988	0.817401457	0.817211	379.6176419	464.5305711
GNAL	0.984015	0.015985	1.30383584	1.306857	63.95951494	48.93913756
MALAT1	0.984025	0.015975	0.789392053	0.789372	4061.706845	5145.493213
SKIDA1	0.984027	0.015973	0.738592809	0.735616	31.29826303	42.55060412
MYH7B	0.984027	0.015973	1.479011106	1.494692	22.21167054	14.85705451
TST	0.984036	0.015964	0.816928919	0.816666	276.6362604	338.7408429
TYMSOS	0.984038	0.015962	1.39336682	1.401417	33.31750581	23.77128722
FKBP1B	0.984056	0.015944	0.714874681	0.710534	23.22129193	32.68551993
CALCOCO	0.984058	0.015942	0.812004742	0.811924	924.8131916	1139.040846
INPP5F	0.984061	0.015939	1.234994283	1.235093	1467.989498	1188.564361
RPS27L	0.984066	0.015934	0.817581498	0.817373	347.3097575	424.911759
AZIN1	0.984067	0.015933	1.281125959	1.281146	8759.475164	6837.216486
CYB5D2	0.984069	0.015931	2.392393269	2.899164	3.866849917	1.327230203
CELF4	0.984088	0.015912	0.461519455	0.408928	2.019242776	4.952351504
CFTR	0.984088	0.015912	0.461519455	0.408928	2.019242776	4.952351504
ZNF674-A	0.984093	0.015907	1.347491825	1.352756	44.43343729	32.84399517
ANKRD20	0.984097	0.015903	1.223016078	1.223376	369.3094076	301.8755383
LOC28445	0.984108	0.015892	0.813676938	0.813291	190.8386348	234.652319
LOC28486	0.984109	0.015891	1.545732746	1.569957	17.20394845	10.95460153
ZNF429	0.984113	0.015887	1.224562196	1.225001	305.178257	249.1230901
RGS5	0.98412	0.01588	1.227952364	1.228555	231.2032979	188.1893571
PRSS27	0.98413	0.01587	0.62443518	0.611862	9.086592493	14.85705451
PIK3R2	0.984131	0.015869	1.244433307	1.244499	2287.781873	1838.312878
TEX22	0.984138	0.015862	0.538479121	0.510286	4.038485553	7.923762406
TRIM36	0.984139	0.015861	1.221685191	1.221918	578.5130554	473.4448038
CEP135	0.984162	0.015838	1.224100854	1.224295	687.5622616	561.5966605
FTCDNL1	0.9842	0.0158	1.478667919	1.494692	22.21167054	14.85705451
ANO7	0.984201	0.015799	1.310351496	1.31374	58.55804051	44.57116353
LINC0096	0.984216	0.015784	1.223477239	1.2239	315.0018731	257.3737077
LIPT2	0.984221	0.015779	0.686115423	0.679692	16.15394221	23.77128722
LOC44044	0.984222	0.015778	0.456753279	0.403749	2.049531418	5.091017346
LOC10050	0.984225	0.015775	1.549720147	1.574809	17.1635636	10.89517331
LOC10050	0.984225	0.015775	1.549720147	1.574809	17.1635636	10.89517331
APOBR	0.984228	0.015772	0.461720635	0.408928	2.019242776	4.952351504
CACNA1G	0.984228	0.015772	0.461720635	0.408928	2.019242776	4.952351504
CHST8	0.984228	0.015772	0.461720635	0.408928	2.019242776	4.952351504

CIDEB	0.984228	0.015772	0.461720635	0.408928	2.019242776	4.952351504
COL24A1	0.984228	0.015772	0.461720635	0.408928	2.019242776	4.952351504
HIST1H2E	0.984228	0.015772	0.461720635	0.408928	2.019242776	4.952351504
IL12A	0.984228	0.015772	0.461720635	0.408928	2.019242776	4.952351504
MYO16-AS	0.984228	0.015772	0.461720635	0.408928	2.019242776	4.952351504
SCGB1B2	0.984228	0.015772	0.461720635	0.408928	2.019242776	4.952351504
SH3GL1P	0.984228	0.015772	0.461720635	0.408928	2.019242776	4.952351504
SLCO4C1	0.984228	0.015772	0.461720635	0.408928	2.019242776	4.952351504
CORO1B	0.984245	0.015755	0.811456582	0.811386	1083.323749	1335.153965
GJC2	0.984246	0.015754	0.538590702	0.510286	4.038485553	7.923762406
HSPB6	0.984246	0.015754	0.538590702	0.510286	4.038485553	7.923762406
LINC0142	0.984246	0.015754	0.538590702	0.510286	4.038485553	7.923762406
LOC10014	0.984246	0.015754	0.538590702	0.510286	4.038485553	7.923762406
LOC10192	0.984246	0.015754	0.538590702	0.510286	4.038485553	7.923762406
PRRT4	0.984246	0.015754	0.538590702	0.510286	4.038485553	7.923762406
UPK1A-AS	0.984246	0.015754	0.538590702	0.510286	4.038485553	7.923762406
SPICE1	0.984255	0.015745	1.22213734	1.222506	358.4155928	293.179209
EVC	0.984264	0.015736	0.813963734	0.813874	823.8510527	1012.260647
FSBP	0.984282	0.015718	1.222280642	1.222667	341.9688604	279.6890035
DDX11L2	0.984282	0.015718	0.763353447	0.761372	44.24160923	58.11089255
FRMD6-AS	0.984291	0.015709	0.568859082	0.546996	5.239935005	9.587752511
KIAA0895I	0.984308	0.015692	0.813025643	0.812592	170.6260146	209.9797038
WARS2-IT	0.984312	0.015688	1.859556199	1.964882	7.794277117	3.961881203
C14orf28	0.984317	0.015683	0.751443204	0.748951	36.34636997	48.53304474
ENHO	0.984317	0.015683	0.751443204	0.748951	36.34636997	48.53304474
PPP1R32	0.984317	0.015683	0.751443204	0.748951	36.34636997	48.53304474
OTX1	0.98433	0.01567	0.796687576	0.795815	89.85630355	112.9136143
AQP3	0.98434	0.01566	1.224525917	1.225021	270.578532	220.8748771
TTC33	0.984353	0.015647	1.2240051	1.224182	754.187177	616.0725271
BST1	0.984356	0.015644	0.751498997	0.748951	36.34636997	48.53304474
R3HCC1L	0.984359	0.015641	1.220881028	1.221153	482.5990235	395.19765
DUSP2	0.984366	0.015634	0.779401657	0.777943	58.55804051	75.27574286
TMUB1	0.984379	0.015621	0.820002183	0.81981	381.6368847	465.5210414
FBLN7	0.984379	0.015621	0.686257738	0.679692	16.15394221	23.77128722
TMIE	0.984379	0.015621	0.686257738	0.679692	16.15394221	23.77128722
MLLT1	0.984382	0.015618	0.804176483	0.804133	1761.789322	2190.920305
KATNAL1	0.984391	0.015609	0.816042722	0.815938	697.6685716	855.0532013
RHBDF2	0.984403	0.015597	0.816044959	0.815941	700.6772434	858.7377508
HIST1H2A	0.984405	0.015595	0.708038541	0.703093	20.19242776	28.72363872
R3HDM4	0.984427	0.015573	0.802530336	0.802491	1972.800192	2458.347287
LOC10050	0.98443	0.01557	1.617849346	1.655593	13.12507805	7.923762406
CATSPER	0.984432	0.015568	0.708384061	0.703445	20.20252398	28.72363872
EMBP1	0.984434	0.015566	1.23637265	1.237287	159.267774	128.7215203
ZNF608	0.984437	0.015563	0.820175362	0.819993	402.8389339	491.2732692
TSSC2	0.984439	0.015561	0.322719251	0.157155	0.302886416	1.980940602
EDEM3	0.984448	0.015552	0.816848627	0.816737	651.2057954	797.3285921
LOC15077	0.98445	0.01555	0.818577373	0.818428	481.2764195	588.0521223
SLC27A5	0.984453	0.015547	0.798638232	0.797758	90.86592493	113.9040846
FLJ31356	0.984461	0.015539	1.309487412	1.31297	56.78110687	43.24393333
AQP11	0.984462	0.015538	0.723607403	0.719614	24.23091332	33.67599023
GAB3	0.984462	0.015538	0.723607403	0.719614	24.23091332	33.67599023
NUDT17	0.984462	0.015538	0.723607403	0.719614	24.23091332	33.67599023
PILRB	0.984465	0.015535	1.238005492	1.238926	155.8956385	125.829347
ATP1A1-A	0.984479	0.015521	1.265482697	1.267245	92.88516771	73.29480226
NCKIPSD	0.984502	0.015498	0.820331306	0.820158	424.040983	517.025497
MEIS2	0.984504	0.015496	0.812840128	0.812373	158.5105579	195.1226493

AACSP1	0.984512	0.015488	0.811384265	0.810846	141.3469943	174.3227729
CC2D1A	0.984518	0.015482	0.812459179	0.812386	1010.63101	1244.030698
LOC10050(	0.98453	0.01547	0.798618856	0.797758	90.86592493	113.9040846
MLLT10	0.984537	0.015463	1.239900148	1.239975	1940.492308	1564.943075
FGFR3	0.984545	0.015455	0.819813236	0.819633	397.7908269	485.3304474
CAMK1	0.984554	0.015446	0.819321087	0.819061	276.6362604	337.7503726
DNAJB13	0.984572	0.015428	0.670821675	0.662738	13.12507805	19.80940602
GABRR2	0.984572	0.015428	0.670821675	0.662738	13.12507805	19.80940602
GBX2	0.984572	0.015428	0.670821675	0.662738	13.12507805	19.80940602
ZNF883	0.984572	0.015428	1.220462069	1.220659	679.4751942	556.644309
SLC2A4	0.984575	0.015425	1.421543449	1.432582	27.21939262	18.99722037
PLCG2	0.984586	0.015414	1.253252936	1.253299	3380.212408	2697.050629
FARS2	0.984591	0.015409	1.223641255	1.224167	253.4149684	207.0082929
ZNF287	0.984599	0.015401	0.809707067	0.809109	128.2219163	158.4752481
GGT6	0.9846	0.0154	0.273808503	0.007534	0	1.3173255
HSPE1-M(	0.984608	0.015392	1.572201277	1.602089	15.25537918	9.51841959
SLC7A14	0.984608	0.015392	0.567930075	0.544953	4.977433444	9.142040876
REEP1	0.98461	0.01539	0.81943731	0.819169	268.5592893	327.8456696
CYP27A1	0.984617	0.015383	0.539193115	0.510286	4.038485553	7.923762406
FAM74A7	0.984618	0.015382	0.500452491	0.45961	2.725977748	5.942821805
RGL3	0.984637	0.015363	0.820306419	0.820106	357.4059714	435.8069323
WNT10A	0.984641	0.015359	1.221602079	1.22179	700.6772434	573.4823041
SLC7A7	0.984649	0.015351	1.230875048	1.231681	175.6741215	142.6277233
EBPL	0.984657	0.015343	1.218857257	1.219079	597.6958618	490.2827989
GUSBP2	0.984666	0.015334	0.715751525	0.711113	21.6765712	30.48667586
SNHG10	0.984666	0.015334	1.280477779	1.282907	73.70236134	57.44727744
ALMS1-IT	0.984669	0.015331	1.758709213	1.833121	9.086592493	4.952351504
ANKRD2	0.984669	0.015331	1.758709213	1.833121	9.086592493	4.952351504
ESR2	0.984669	0.015331	1.758709213	1.833121	9.086592493	4.952351504
LINC0117(	0.984669	0.015331	1.758709213	1.833121	9.086592493	4.952351504
PKD1L1	0.984669	0.015331	1.758709213	1.833121	9.086592493	4.952351504
SSSCA1-/	0.984669	0.015331	1.758709213	1.833121	9.086592493	4.952351504
ZNF396	0.98467	0.01533	0.782465882	0.781078	59.5676619	76.26621316
OR2T33	0.984689	0.015311	2.486069544	3.208991	3.2004998	0.990470301
TECPR1	0.984691	0.015309	0.808647052	0.808006	120.0439831	148.5705451
FOXD4L1	0.984693	0.015307	0.52368408	0.489616	3.321654367	6.794626263
HCFC1R1	0.984698	0.015302	1.238023689	1.238101	1845.587898	1490.657803
LOC10192	0.984702	0.015298	0.275311729	0.007591	0	1.307420797
HVCN1	0.984706	0.015294	0.462805864	0.408928	2.019242776	4.952351504
RSPH4A	0.984706	0.015294	0.462805864	0.408928	2.019242776	4.952351504
SNORA33	0.984706	0.015294	0.462805864	0.408928	2.019242776	4.952351504
ZMAT4	0.984706	0.015294	0.462805864	0.408928	2.019242776	4.952351504
DDX59	0.98471	0.01529	1.218256171	1.218487	574.4745699	471.4638632
ANKRD36	0.984712	0.015288	1.233397108	1.234243	165.6485812	134.2087258
ADIRF	0.98472	0.01528	1.50778307	1.528558	18.17318499	11.88564361
ST8SIA4	0.98472	0.01528	0.561372013	0.537125	4.825990235	8.993470331
ANXA2R	0.984721	0.015279	1.232697908	1.233526	168.6067718	136.6849015
FUT8-AS1	0.984723	0.015277	1.237382275	1.238346	148.4143441	119.8469064
PURB	0.984738	0.015262	0.808606491	0.808553	1411.652625	1745.901999
TAPBPL	0.98474	0.01526	1.298028834	1.301214	60.57728329	46.55210414
DMBX1	0.984745	0.015255	1.24678338	1.248031	120.1550414	96.27371324
STK3	0.984749	0.015251	1.233429084	1.233519	1593.182551	1291.573272
CYP2D7	0.984753	0.015247	0.711074475	0.7061	19.95011863	28.25811768
MIR181A2	0.984759	0.015241	0.671000028	0.662738	13.12507805	19.80940602
KRT13	0.98476	0.01524	1.450850801	1.465001	23.22129193	15.84752481
GLUD1P3	0.984769	0.015231	1.616893506	1.655593	13.12507805	7.923762406

NFYC-AS1	0.984769	0.015231	1.616893506	1.655593	13.12507805	7.923762406
C2orf91	0.984772	0.015228	1.633498011	1.676081	12.12555287	7.230433196
WNT2B	0.984778	0.015222	0.770565267	0.768717	46.44258386	60.41868835
GOLGA8K	0.984779	0.015221	1.507253601	1.528129	18.06212663	11.81631069
TMCC1-AS	0.984784	0.015216	1.237061807	1.23808	143.4772955	115.8850252
ZEB1-AS1	0.984801	0.015199	1.217976453	1.218298	401.8293125	329.8266102
DYX1C1	0.984801	0.015199	0.783761822	0.782386	59.83016346	76.47421192
HIST1H2A	0.984804	0.015196	0.276831551	0.007648	0	1.297516094
TRAF4	0.984806	0.015194	1.242049587	1.242113	2347.369727	1889.817334
SDCBP2-A	0.98481	0.01519	0.78018367	0.77869	55.52917635	71.31386166
PDP2	0.984812	0.015188	0.820683838	0.820521	438.4179916	534.3191085
FAHD2CP	0.984813	0.015187	0.756322297	0.753873	36.34636997	48.21609424
LOC64455	0.984814	0.015186	1.218449602	1.218711	493.7048588	405.102353
TCAP	0.984814	0.015186	0.807378586	0.806682	111.0583527	137.6753718
ANP32E	0.984823	0.015177	1.252247829	1.252293	3381.222029	2700.02204
EPHB6	0.984825	0.015175	0.648113439	0.637313	10.09621388	15.84752481
LOC72968	0.984825	0.015175	0.648113439	0.637313	10.09621388	15.84752481
FLJ42627	0.984828	0.015172	0.820683339	0.820446	299.8575523	365.483541
POU2AF1	0.984832	0.015168	0.554302072	0.527311	4.210121189	7.993095327
TBC1D3G	0.984861	0.015139	0.779197492	0.777608	53.74214649	69.11501759
DMRTA1	0.984861	0.015139	0.610873644	0.594953	7.067349717	11.88564361
CHMP5	0.98487	0.01513	1.269525758	1.269552	6454.509535	5084.084054
ZNF321P	0.984901	0.015099	1.232503379	1.233369	161.1456698	130.6529374
TCEAL8	0.984913	0.015087	0.82151635	0.821323	368.5118067	448.6830463
C1orf74	0.984916	0.015084	1.226277289	1.226964	196.8761707	160.4561887
NYX	0.984919	0.015081	1.507332045	1.528558	18.17318499	11.88564361
HLA-H	0.984927	0.015073	1.289321256	1.292157	64.16143922	49.65227618
SLC9A1	0.984928	0.015072	1.242744976	1.242806	2470.543537	1987.873894
LRRC29	0.98493	0.01507	0.718244501	0.713631	21.20204915	29.71410902
WNT5B	0.98493	0.01507	0.718244501	0.713631	21.20204915	29.71410902
PPP1R3E	0.984936	0.015064	0.785483271	0.784132	60.57728329	77.25668346
RAB36	0.984939	0.015061	0.822496144	0.822276	330.196675	401.566374
CREBZF	0.984943	0.015057	0.812966833	0.812899	1085.342992	1335.153965
DCLK1	0.984946	0.015054	0.817667261	0.817273	183.7510926	224.8367583
ZNF609	0.984947	0.015053	0.802060392	0.802026	2325.158057	2899.10657
COA6	0.984963	0.015037	1.216864709	1.217152	447.262275	367.4644816
MRPS9	0.984979	0.015021	1.223322675	1.223466	925.8228129	756.7193098
NELL2	0.984986	0.015014	0.699313161	0.693272	17.1635636	24.76175752
TMEM51-A	0.984986	0.015014	0.699313161	0.693272	17.1635636	24.76175752
WEE2-AS	0.984986	0.015014	0.699313161	0.693272	17.1635636	24.76175752
TRAPPC1	0.984986	0.015014	1.221681657	1.221831	901.5918996	737.9003741
SSNA1	0.984994	0.015006	1.222488103	1.222638	880.3898505	720.0719087
ABI3BP	0.985	0.015	1.757179574	1.833121	9.086592493	4.952351504
LOC10013	0.985	0.015	1.757179574	1.833121	9.086592493	4.952351504
LOC10050	0.985	0.015	1.757179574	1.833121	9.086592493	4.952351504
HOMER3	0.985006	0.014994	1.229308027	1.229413	1311.498183	1066.766228
EPB41L1	0.985012	0.014988	0.807117838	0.807073	1727.462195	2140.40632
NARFL	0.985012	0.014988	1.217107624	1.217317	626.974882	515.0445564
LOC72848	0.985013	0.014987	1.245753525	1.247033	116.1064596	93.10420827
S100A5	0.985016	0.014984	1.233851193	1.234785	150.4335868	121.827847
PYGO1	0.98502	0.01498	0.81985713	0.819744	649.1865526	791.9404337
LCLAT1	0.985021	0.014979	1.223577564	1.223717	955.1018332	780.490597
ZNF815P	0.985028	0.014972	0.728976924	0.724943	24.23091332	33.42837265
ATP5L	0.985028	0.014972	1.253180457	1.253223	3624.540783	2892.173278
TMEM9B	0.98503	0.01497	0.8175754	0.817478	740.0524775	905.2898549
C19orf60	0.98504	0.01496	0.822942634	0.822717	321.0596014	390.2452985

CMYA5	0.985043	0.014957	0.718347128	0.713631	21.20204915	29.71410902
KCNQ4	0.985043	0.014957	0.718347128	0.713631	21.20204915	29.71410902
SYT15	0.985047	0.014953	0.822290365	0.822134	464.2138181	564.6473091
NDE1	0.985064	0.014936	1.221257582	1.221416	829.9087811	679.4626263
TTC39C	0.985068	0.014932	1.2210512	1.221212	818.8837155	670.5483936
LRRC49	0.985074	0.014926	0.822375112	0.82222	467.4547027	568.5299526
LIPC	0.985075	0.014925	0.810696505	0.810081	121.1545666	149.5610154
NDUFB6	0.985085	0.014915	1.216182871	1.216453	473.512431	389.2548282
SLC25A53	0.985102	0.014898	0.577636616	0.555078	5.048106941	9.102422064
KRT15	0.985106	0.014894	1.426481154	1.438801	24.23091332	16.83799511
SNAPC5	0.98511	0.01489	0.808644212	0.807932	108.0294885	133.7134906
IGSF5	0.985136	0.014864	0.611219275	0.594953	7.067349717	11.88564361
LOC10050	0.985136	0.014864	0.611219275	0.594953	7.067349717	11.88564361
ITGB3BP	0.985137	0.014863	1.219188757	1.219372	707.7445931	580.4155963
SEPP1	0.985155	0.014845	1.222053727	1.222192	979.3327465	801.2904733
FAM71F2	0.985163	0.014837	1.605182056	1.644404	12.28709229	7.468146068
TMEM53	0.985166	0.014834	1.232480032	1.233462	145.3854799	117.8659658
TPCN1	0.985182	0.014818	0.793712281	0.793691	3767.907021	4747.324152
LOC10192	0.9852	0.0148	1.374073336	1.38202	31.65163052	22.89967335
KRBOX4	0.985204	0.014796	1.214137758	1.214496	361.1314743	297.349089
FKBP11	0.985208	0.014792	1.381311058	1.389824	30.28864164	21.79034662
MEFV	0.985219	0.014781	0.71310392	0.707918	18.97078588	26.80212634
ANAPC15	0.985224	0.014776	1.222081423	1.222216	1003.56366	821.0998793
FAM86FP	0.985245	0.014755	0.726477864	0.722187	22.39340239	31.01162512
CSRNP3	0.985247	0.014753	0.687030503	0.679711	14.13469943	20.79987632
LINC0092	0.985247	0.014753	0.687030503	0.679711	14.13469943	20.79987632
EIF3J-AS1	0.985249	0.014751	0.811963406	0.811321	118.1257024	145.5991342
HNRNPH2	0.98525	0.01475	0.813620953	0.813555	1117.943667	1374.148781
CEP83-AS	0.98525	0.01475	1.426210498	1.438801	24.23091332	16.83799511
DPM2	0.985256	0.014744	1.232031324	1.232119	1590.153686	1290.582802
CYP4F62F	0.98526	0.01474	0.46771735	0.409075	1.797126071	4.407592838
INO80B	0.985261	0.014739	0.818418497	0.817993	170.0101456	207.8402879
ZNF213-A	0.985262	0.014738	0.748926029	0.745918	30.28864164	40.60928233
ZMYM5	0.985265	0.014735	1.216180023	1.216409	555.4230143	456.6068087
CRIP3	0.985266	0.014734	1.554844009	1.584977	14.13469943	8.914232707
CNTD1	0.985267	0.014733	1.35242315	1.35898	36.34636997	26.74269812
TMC5	0.985295	0.014705	0.744054375	0.740732	28.39055344	38.33120064
DDX18	0.985305	0.014695	0.812354254	0.812294	1239.815065	1526.314733
ZNF506	0.985309	0.014691	1.218228586	1.218393	806.1927747	661.6836844
GSTO1	0.98531	0.01469	1.243444923	1.243501	2609.871288	2098.806567
FBXL4	0.985314	0.014686	1.223771842	1.223897	1063.131322	868.6424538
DHX37	0.985314	0.014686	1.221715491	1.221858	919.7650846	752.7574286
SOWAHB	0.985315	0.014685	0.786641615	0.785193	57.54841913	73.29480226
PRICKLE2	0.985321	0.014679	0.493209089	0.444298	2.221167054	5.011779722
SENCR	0.985324	0.014676	1.471605704	1.489418	19.18280638	12.87611391
ACADL	0.985327	0.014673	0.739796369	0.736261	26.25015609	35.65693083
CYP4F29F	0.985334	0.014666	1.554723984	1.584977	14.13469943	8.914232707
PLEKHJ1	0.985335	0.014665	1.219047767	1.219202	862.2166655	707.1957948
SMUG1	0.985344	0.014656	1.213793587	1.214035	535.0993357	440.7592838
APBB3	0.985352	0.014648	0.818845419	0.818417	168.6067718	206.0178226
ODF3B	0.985359	0.014641	0.766750897	0.764547	39.37523414	51.50445564
LRRC37A4	0.98536	0.01464	0.824374679	0.824186	381.4046718	462.7675339
TMEM69	0.985381	0.014619	1.218674964	1.21883	856.1589372	702.4415373
KCP	0.985387	0.014613	0.711262699	0.705808	18.17318499	25.75222782
GTF2IP4	0.985392	0.014608	2.476450962	3.300785	2.867324742	0.861709162
CLIC3	0.9854	0.0146	1.221305755	1.221961	200.9146562	164.4180699

CHCHD7	0.985402	0.014598	0.82376405	0.823573	369.5214281	448.6830463
ANKAR	0.985403	0.014597	1.323311754	1.328124	43.41371969	32.68551993
WDR53	0.985406	0.014594	1.212645732	1.213002	360.4348356	297.1410902
KCNC4	0.985411	0.014589	1.213771399	1.214106	377.5983992	311.0076744
THSD7A	0.985416	0.014584	0.687192191	0.679711	14.13469943	20.79987632
THAP7	0.985428	0.014572	0.82187492	0.821497	192.8376851	234.7414613
ZNF787	0.98543	0.01457	0.822448418	0.822323	581.5419196	707.1957948
ELMSAN1	0.98545	0.01455	1.249748396	1.249793	3416.558778	2733.69803
CUTC	0.985455	0.014545	1.212329226	1.212606	462.4065958	381.3310658
PIGY	0.985456	0.014544	0.81463507	0.814568	1094.429585	1343.572963
PYURF	0.985456	0.014544	0.81463507	0.814568	1094.429585	1343.572963
FLJ42102	0.985459	0.014541	1.40381308	1.41462	25.71505676	18.17513002
UQCC3	0.985469	0.014531	1.213149172	1.213384	549.2340352	452.6449275
RASGRP4	0.985469	0.014531	2.106701179	2.383358	4.73512431	1.980940602
MOK	0.985477	0.014523	0.805593603	0.804756	90.86592493	112.9136143
HNMT	0.985478	0.014522	0.799685843	0.798675	76.7312255	96.07561918
SLC29A2	0.985478	0.014522	1.217246017	1.217409	806.6874891	662.6246312
IDH3G	0.985479	0.014521	0.821785338	0.821672	642.1192029	781.4810673
ADPRM	0.985483	0.014517	1.235908898	1.237034	126.2026735	102.018441
B4GALT7	0.985483	0.014517	0.823817737	0.823554	267.5496679	324.8742587
TTC25	0.985484	0.014516	0.78453368	0.782999	53.50993357	68.34245075
UBE2G1	0.985484	0.014516	0.78453368	0.782999	53.50993357	68.34245075
GALK2	0.985493	0.014507	0.819802405	0.819379	169.6163932	207.0082929
ERCC5	0.985497	0.014503	0.819430355	0.818994	165.0428083	201.5210874
ALPK3	0.985505	0.014495	0.727898493	0.723489	22.21167054	30.70457932
IRAK3	0.985505	0.014495	0.727898493	0.723489	22.21167054	30.70457932
TSEN34	0.985506	0.014494	1.234173777	1.23425	1849.626383	1498.581565
SAMD12	0.985517	0.014483	0.818071795	0.817987	853.130073	1042.965227
GRAMD1E	0.985522	0.014478	0.795326818	0.794158	67.64463301	85.18044587
TRAPPC2	0.985524	0.014476	1.223200681	1.223998	170.5654373	139.3492666
HHIPL1	0.985533	0.014467	1.890437374	2.035187	6.057728329	2.971410902
KCCAT19	0.985533	0.014467	1.890437374	2.035187	6.057728329	2.971410902
LINC0093	0.985543	0.014457	1.214719972	1.215181	275.626639	226.8176989
PKNOX1	0.985544	0.014456	1.229164872	1.229257	1490.201169	1212.27622
ANKMY1	0.985551	0.014449	0.816027413	0.81548	133.2700232	163.4275996
ZNF713	0.985554	0.014446	0.733263904	0.729161	23.58475563	32.34876002
LOC55424	0.98556	0.01444	0.730697407	0.726459	22.45397967	30.91257809
DMGDH	0.985563	0.014437	0.818298064	0.817782	143.3662371	175.3132432
GDAP1L1	0.985564	0.014436	2.372537075	3.037436	3.028864164	0.990470301
MGAT4C	0.985564	0.014436	2.372537075	3.037436	3.028864164	0.990470301
PPP1R35	0.985566	0.014434	1.215785605	1.216298	249.3764829	205.0273523
LOC10050	0.985577	0.014423	1.365908802	1.373723	31.29826303	22.78081692
ATP1B4	0.985581	0.014419	0.714983258	0.709638	18.40539791	25.94041718
ANKRD24	0.985585	0.014415	1.340741018	1.346854	37.35599136	27.73316842
NLE1	0.98559	0.01441	1.21638471	1.216566	703.7061075	578.4346557
MAGEE1	0.985591	0.014409	0.82289537	0.822559	211.0108701	256.5318079
MAP3K7C	0.985595	0.014405	0.669324334	0.659772	11.10583527	16.83799511
GDPGP1	0.985608	0.014392	1.287949246	1.291093	57.54841913	44.57116353
INIP	0.985612	0.014388	1.212352463	1.212639	437.1963497	360.5311895
RFESD	0.985618	0.014382	1.28787893	1.291093	57.54841913	44.57116353
TM2D3	0.985619	0.014381	0.824589187	0.82444	482.5990235	585.3679478
C1orf109	0.98562	0.01438	1.21479592	1.214994	641.4225641	527.9206703
MAP1LC3	0.985621	0.014379	0.63338979	0.619073	7.67312255	12.40068817
MICAL1	0.985626	0.014374	0.818799238	0.818712	831.1001343	1015.133011
GPR65	0.985628	0.014372	1.737575485	1.816534	8.248606741	4.536353978
DQX1	0.985645	0.014355	1.889914682	2.035187	6.057728329	2.971410902

LOC10013	0.985645	0.014355	1.889914682	2.035187	6.057728329	2.971410902
RNF138P1	0.985645	0.014355	1.889914682	2.035187	6.057728329	2.971410902
SCGB1D2	0.985645	0.014355	1.889914682	2.035187	6.057728329	2.971410902
NGLY1	0.985647	0.014353	1.21357521	1.213795	573.4649485	472.4543335
TMEM213	0.985649	0.014351	0.413149268	0.321856	1.019717602	3.189314368
RNPS1	0.985649	0.014351	0.795068929	0.795048	3757.810807	4726.524275
NCAPH	0.985652	0.014348	1.217113196	1.217276	788.5143042	647.7675767
KIAA1328	0.985656	0.014344	1.219778799	1.220505	183.7510926	150.5514857
YAE1D1	0.98566	0.01434	1.217887859	1.218503	210.0012487	172.3418323
CACNB3	0.985663	0.014337	0.821239517	0.821133	673.4174659	820.109409
LOC10037	0.985663	0.014337	0.771471972	0.769354	40.38485553	52.49492594
LOC10013	0.985671	0.014329	0.821911197	0.821523	183.2664744	223.0836258
ZFP69B	0.985681	0.014319	1.248685057	1.250351	92.88516771	74.28527256
NEXN	0.985687	0.014313	0.771519434	0.769354	40.38485553	52.49492594
GOLGA8II	0.985692	0.014308	0.29130453	0.008208	0	1.208373767
LINC0146	0.985694	0.014306	1.645189127	1.697718	10.09621388	5.942821805
CTSH	0.985696	0.014304	0.811673769	0.811623	1491.21079	1837.322408
CLYBL	0.985697	0.014303	0.821545558	0.82114	175.6741215	213.941585
SYCE2	0.985704	0.014296	1.225577264	1.226499	149.4239654	121.827847
LOC10192	0.985708	0.014292	0.627201396	0.612082	7.501486914	12.26202232
CCDC122	0.985713	0.014287	1.212774077	1.213184	305.178257	251.5497423
GVQW2	0.985717	0.014283	1.382036226	1.391391	28.26939887	20.31454587
TBC1D24	0.985719	0.014281	0.82502754	0.824879	483.3461434	585.9622299
ARAP1	0.985719	0.014281	0.810677208	0.810628	1521.499432	1876.94122
ANKRD33	0.98572	0.01428	2.370658193	3.037436	3.028864164	0.990470301
CD81-AS1	0.98572	0.01428	2.370658193	3.037436	3.028864164	0.990470301
CTAGE10	0.98572	0.01428	2.370658193	3.037436	3.028864164	0.990470301
DACT3	0.98572	0.01428	2.370658193	3.037436	3.028864164	0.990470301
DTNA	0.98572	0.01428	2.370658193	3.037436	3.028864164	0.990470301
ERP27	0.98572	0.01428	2.370658193	3.037436	3.028864164	0.990470301
FAM65C	0.98572	0.01428	2.370658193	3.037436	3.028864164	0.990470301
FRMPD1	0.98572	0.01428	2.370658193	3.037436	3.028864164	0.990470301
HAS2-AS1	0.98572	0.01428	2.370658193	3.037436	3.028864164	0.990470301
HCN1	0.98572	0.01428	2.370658193	3.037436	3.028864164	0.990470301
HDAC11-A	0.98572	0.01428	2.370658193	3.037436	3.028864164	0.990470301
LINC0034	0.98572	0.01428	2.370658193	3.037436	3.028864164	0.990470301
LINC0093	0.98572	0.01428	2.370658193	3.037436	3.028864164	0.990470301
LINC0146	0.98572	0.01428	2.370658193	3.037436	3.028864164	0.990470301
LOC10192	0.98572	0.01428	2.370658193	3.037436	3.028864164	0.990470301
LOC10192	0.98572	0.01428	2.370658193	3.037436	3.028864164	0.990470301
LOC28562	0.98572	0.01428	2.370658193	3.037436	3.028864164	0.990470301
MDC1-AS	0.98572	0.01428	2.370658193	3.037436	3.028864164	0.990470301
PARD3-AS	0.98572	0.01428	2.370658193	3.037436	3.028864164	0.990470301
RGAG1	0.98572	0.01428	2.370658193	3.037436	3.028864164	0.990470301
RPS15AP	0.98572	0.01428	2.370658193	3.037436	3.028864164	0.990470301
SNRK-AS	0.98572	0.01428	2.370658193	3.037436	3.028864164	0.990470301
SPDYA	0.98572	0.01428	2.370658193	3.037436	3.028864164	0.990470301
UQCRH	0.98572	0.01428	2.370658193	3.037436	3.028864164	0.990470301
EEF2KMT	0.985727	0.014273	1.211420216	1.211722	413.2582266	341.0486387
NANS	0.985727	0.014273	1.22286877	1.222985	1145.920276	936.9849045
C20orf24	0.985727	0.014273	1.235493202	1.235562	2049.602091	1658.83966
METTL25	0.98573	0.01427	1.248721664	1.250351	92.88516771	74.28527256
LINC0048	0.985735	0.014265	1.879632645	2.021809	6.077920757	3.001125011
ZNF702P	0.985736	0.014264	1.211435383	1.211666	553.7369466	457.0029968
LINC0033	0.98574	0.01426	0.589187557	0.566783	5.048106941	8.914232707
NBPF7	0.98574	0.01426	0.589187557	0.566783	5.048106941	8.914232707

PLCB2	0.98574	0.01426	0.589187557	0.566783	5.048106941	8.914232707
TRIML2	0.98574	0.01426	0.589187557	0.566783	5.048106941	8.914232707
GOLGA6L	0.985742	0.014258	0.594984175	0.573767	5.320704716	9.280706718
RFC4	0.985762	0.014238	1.233596729	1.233668	1999.050349	1620.409412
NMNAT2	0.985768	0.014232	1.217572093	1.217715	921.7843274	756.9768321
BDNF-AS	0.985769	0.014231	0.803217016	0.802241	78.75046828	98.16551151
GNPTG	0.985776	0.014224	0.82179272	0.821686	667.3597376	812.1856466
LOC10050	0.985776	0.014224	1.644985157	1.697718	10.09621388	5.942821805
TMCO4	0.98578	0.01422	0.824230239	0.823906	217.0685985	263.4651
SLC48A1	0.985781	0.014219	1.255787515	1.255822	4517.682152	3597.388132
FAM212B	0.985803	0.014197	1.225597564	1.226499	149.4239654	121.827847
PSG7	0.985808	0.014192	0.424632059	0.333139	1.009621388	3.050648526
C4orf26	0.985813	0.014187	2.354973768	3.00766	3.028864164	1.000375004
MPL	0.985814	0.014186	1.512382716	1.537686	15.14432082	9.84527479
COPS7B	0.985824	0.014176	0.820175328	0.820084	789.5239255	962.7371324
NLGN3	0.985824	0.014176	0.641126766	0.627573	8.076971105	12.87611391
SERPINA6	0.985824	0.014176	0.641126766	0.627573	8.076971105	12.87611391
SPTSSB	0.985824	0.014176	0.641126766	0.627573	8.076971105	12.87611391
KANSL1-A	0.985826	0.014174	0.736772329	0.732732	23.22129193	31.69504962
VEPH1	0.985826	0.014174	0.736772329	0.732732	23.22129193	31.69504962
UBLCP1	0.98583	0.01417	0.81170338	0.811652	1459.912527	1798.694066
TENM3	0.985834	0.014166	1.222694253	1.222803	1243.85355	1017.212999
HOOK1	0.985853	0.014147	1.24283232	1.242884	2817.853294	2267.186518
ZBED3	0.985879	0.014121	1.263106966	1.265336	72.69273995	57.44727744
ACOX1	0.985883	0.014117	0.820737873	0.820645	767.312255	935.0138686
NCALD	0.985887	0.014113	1.212922344	1.213121	632.022989	520.9873782
ACSF2	0.985889	0.014111	0.823608749	0.823483	560.3398704	680.4530966
LINC0047	0.985906	0.014094	0.816324286	0.815725	121.2151439	148.6002592
CA5BP1	0.985922	0.014078	1.235062129	1.236301	114.1276017	92.31183203
PLSCR4	0.985925	0.014075	0.826047684	0.825873	400.8196911	485.3304474
ENTPD1-A	0.985933	0.014067	1.589903735	1.630615	11.4894914	7.042243839
PCDHGA9	0.985938	0.014062	0.813508885	0.812811	105.6467821	129.9794176
C6orf99	0.98595	0.01405	1.384725764	1.39467	26.25015609	18.81893571
LOC10012	0.98595	0.01405	1.384725764	1.39467	26.25015609	18.81893571
AGMO	0.985966	0.014034	1.290014241	1.293489	52.53060083	40.60928233
LOC10192	0.985971	0.014029	0.425600367	0.331834	0.979332747	2.971410902
ZNF26	0.985974	0.014026	1.209332725	1.209575	519.9550149	429.8641105
TMEM254	0.98598	0.01402	0.701945856	0.695141	15.14432082	21.79034662
THOC5	0.985981	0.014019	1.213398641	1.213568	763.2737694	628.948641
BDH2	0.985996	0.014004	0.826078577	0.825916	431.1083327	521.9778485
CA9	0.985999	0.014001	1.503741741	1.528469	15.14432082	9.904703008
OLFM5P	0.985999	0.014001	1.503741741	1.528469	15.14432082	9.904703008
SCOC-AS	0.985999	0.014001	1.503741741	1.528469	15.14432082	9.904703008
PSMC3IP	0.986004	0.013996	1.212189755	1.212387	634.0422318	522.9683188
LOC10028	0.986005	0.013995	1.440080534	1.455865	20.19242776	13.86658421
TMEM256	0.986015	0.013985	1.210388601	1.210598	606.6310111	501.0987346
C3orf67	0.986017	0.013983	1.247259623	1.249009	87.83706077	70.32339135
SHTN1	0.98602	0.01398	1.247589727	1.247632	3619.492677	2901.087511
LINC0066	0.986025	0.013975	1.236299108	1.237669	106.21217	85.81434686
LRRC69	0.986029	0.013971	1.887098955	2.035187	6.057728329	2.971410902
FAM157B	0.98603	0.01397	1.666410568	1.7261	9.581306974	5.546633684
ADGRG6	0.986032	0.013968	1.210910569	1.21113	567.4072201	468.4924523
RPL13AP2	0.986037	0.013963	0.540458902	0.503862	3.06924902	6.101297053
CHAC2	0.986041	0.013959	1.215183675	1.215821	199.9050349	164.4180699
ZNF625	0.986046	0.013954	1.249628358	1.251477	84.08126921	67.1836005
FOPNL	0.986047	0.013953	1.24368172	1.243731	3017.586694	2426.236239

ADGRG2	0.986048	0.013952	0.589667528	0.566783	5.048106941	8.914232707
TMEM266	0.986048	0.013952	0.589667528	0.566783	5.048106941	8.914232707
CTSK	0.986055	0.013945	0.783294211	0.781527	46.44258386	59.42821805
EMC3-AS1	0.986056	0.013944	1.211163278	1.211653	254.4245898	209.9797038
HAGH	0.986059	0.013941	1.219060145	1.219181	1102.506556	904.2993846
ANKRD61	0.98606	0.01394	1.643884466	1.697718	10.09621388	5.942821805
SNORA8	0.98606	0.01394	1.643884466	1.697718	10.09621388	5.942821805
ABHD11-A	0.986065	0.013935	0.641421936	0.627573	8.076971105	12.87611391
LINC0151	0.986074	0.013926	1.319811962	1.325027	39.37523414	29.71410902
MINOS1-N	0.986078	0.013922	1.213411796	1.213998	215.2007989	177.2644697
HIPK2	0.986085	0.013915	0.818181102	0.818109	1001.544417	1224.221292
WBP2NL	0.986091	0.013909	1.319790412	1.325027	39.37523414	29.71410902
ART5	0.986091	0.013909	0.434001607	0.341993	1.009621388	2.971410902
GFRA3	0.986091	0.013909	0.434001607	0.341993	1.009621388	2.971410902
LINC0068	0.986091	0.013909	0.434001607	0.341993	1.009621388	2.971410902
LINC0112	0.986091	0.013909	0.434001607	0.341993	1.009621388	2.971410902
MYCT1	0.986091	0.013909	0.434001607	0.341993	1.009621388	2.971410902
CCDC68	0.986094	0.013906	1.214494782	1.214646	860.1974227	708.1862651
PSD3	0.986102	0.013898	0.827352258	0.82715	343.271272	415.007056
TMEM187	0.986112	0.013888	0.822337024	0.821882	155.4816938	189.1798274
FAM83H	0.986125	0.013875	0.811064503	0.811018	1620.442328	1998.036119
LRRC2	0.986135	0.013865	0.431867078	0.339736	1.009621388	2.991220308
AFG3L2	0.986144	0.013856	1.256254459	1.256287	4876.471305	3881.653109
LOC72777	0.98615	0.01385	0.823566486	0.823135	163.659627	198.8270082
RPS6KB2	0.986162	0.013838	0.826629403	0.826491	514.906908	623.0058192
ANAPC4	0.986169	0.013831	1.209508114	1.209713	615.8690468	509.1017346
DPY19L3	0.986173	0.013827	0.822641212	0.822541	704.7157289	856.7568102
PTGES3L	0.986174	0.013826	3.42610444	124.1738	1.231738094	0
ALKBH6	0.986175	0.013825	1.220706027	1.221567	152.4528296	124.7992579
ZNF684	0.986181	0.013819	1.230492858	1.231673	117.116081	95.08514887
ZNF446	0.986197	0.013803	0.825554933	0.825184	188.7991996	228.7986395
SLFN5	0.986198	0.013802	0.802242741	0.802215	2866.315121	3573.002754
KRT6A	0.9862	0.0138	1.306881399	1.31145	42.86852414	32.68551993
DLEU2	0.986201	0.013799	0.768139465	0.765623	34.3271272	44.83859052
PAIP2B	0.986206	0.013794	0.794249414	0.792845	56.53879774	71.31386166
LOC10192	0.986208	0.013792	1.503209182	1.528469	15.14432082	9.904703008
HCAR1	0.986211	0.013789	0.493285053	0.436826	1.89808821	4.358069323
ZNF890P	0.986213	0.013787	0.29081507	0.008012	0	1.238087876
ARHGAP3	0.986218	0.013782	0.826688576	0.826539	467.4547027	565.5585417
STON1	0.986223	0.013777	0.81664674	0.815947	106.3939019	130.3954151
SLX1A-SU	0.986227	0.013773	0.825431312	0.82501	170.030338	206.0970602
SLX1B-SU	0.986227	0.013773	0.825431312	0.82501	170.030338	206.0970602
PTOV1-AS	0.986233	0.013767	0.688242343	0.679737	12.11545666	17.82846541
RNF111	0.986238	0.013762	0.820409253	0.820328	886.4475788	1080.603098
ABCC11	0.986242	0.013758	0.434333852	0.341993	1.009621388	2.971410902
ADGRB3	0.986242	0.013758	0.434333852	0.341993	1.009621388	2.971410902
ANKK1	0.986242	0.013758	0.434333852	0.341993	1.009621388	2.971410902
ARHGAP2	0.986242	0.013758	0.434333852	0.341993	1.009621388	2.971410902
ARHGAP9	0.986242	0.013758	0.434333852	0.341993	1.009621388	2.971410902
ASCL2	0.986242	0.013758	0.434333852	0.341993	1.009621388	2.971410902
ASXL3	0.986242	0.013758	0.434333852	0.341993	1.009621388	2.971410902
AZU1	0.986242	0.013758	0.434333852	0.341993	1.009621388	2.971410902
BTK	0.986242	0.013758	0.434333852	0.341993	1.009621388	2.971410902
CAMK1G	0.986242	0.013758	0.434333852	0.341993	1.009621388	2.971410902
CCL17	0.986242	0.013758	0.434333852	0.341993	1.009621388	2.971410902
CDCP2	0.986242	0.013758	0.434333852	0.341993	1.009621388	2.971410902

CDH26	0.986242	0.013758	0.434333852	0.341993	1.009621388	2.971410902
CHRDL1	0.986242	0.013758	0.434333852	0.341993	1.009621388	2.971410902
CLEC2B	0.986242	0.013758	0.434333852	0.341993	1.009621388	2.971410902
CLPSL2	0.986242	0.013758	0.434333852	0.341993	1.009621388	2.971410902
CSAG3	0.986242	0.013758	0.434333852	0.341993	1.009621388	2.971410902
CYP26A1	0.986242	0.013758	0.434333852	0.341993	1.009621388	2.971410902
FGF1	0.986242	0.013758	0.434333852	0.341993	1.009621388	2.971410902
FRG2	0.986242	0.013758	0.434333852	0.341993	1.009621388	2.971410902
GALNT5	0.986242	0.013758	0.434333852	0.341993	1.009621388	2.971410902
GAS1	0.986242	0.013758	0.434333852	0.341993	1.009621388	2.971410902
HAR1B	0.986242	0.013758	0.434333852	0.341993	1.009621388	2.971410902
IGFL1	0.986242	0.013758	0.434333852	0.341993	1.009621388	2.971410902
KLHL10	0.986242	0.013758	0.434333852	0.341993	1.009621388	2.971410902
KLHL32	0.986242	0.013758	0.434333852	0.341993	1.009621388	2.971410902
LCNL1	0.986242	0.013758	0.434333852	0.341993	1.009621388	2.971410902
LINC0044	0.986242	0.013758	0.434333852	0.341993	1.009621388	2.971410902
LINC0090	0.986242	0.013758	0.434333852	0.341993	1.009621388	2.971410902
LINC0113	0.986242	0.013758	0.434333852	0.341993	1.009621388	2.971410902
LINC0127	0.986242	0.013758	0.434333852	0.341993	1.009621388	2.971410902
LOC10012	0.986242	0.013758	0.434333852	0.341993	1.009621388	2.971410902
LOC10106	0.986242	0.013758	0.434333852	0.341993	1.009621388	2.971410902
LOC10144	0.986242	0.013758	0.434333852	0.341993	1.009621388	2.971410902
LOC10192	0.986242	0.013758	0.434333852	0.341993	1.009621388	2.971410902
LOC10192	0.986242	0.013758	0.434333852	0.341993	1.009621388	2.971410902
LOC10544	0.986242	0.013758	0.434333852	0.341993	1.009621388	2.971410902
LRRC2-AS	0.986242	0.013758	0.434333852	0.341993	1.009621388	2.971410902
MUC3A	0.986242	0.013758	0.434333852	0.341993	1.009621388	2.971410902
NANOS3	0.986242	0.013758	0.434333852	0.341993	1.009621388	2.971410902
PNPLA1	0.986242	0.013758	0.434333852	0.341993	1.009621388	2.971410902
PPP4R1-A	0.986242	0.013758	0.434333852	0.341993	1.009621388	2.971410902
SERPINF1	0.986242	0.013758	0.434333852	0.341993	1.009621388	2.971410902
SFTPD	0.986242	0.013758	0.434333852	0.341993	1.009621388	2.971410902
SPON2	0.986242	0.013758	0.434333852	0.341993	1.009621388	2.971410902
SPRY4-IT	0.986242	0.013758	0.434333852	0.341993	1.009621388	2.971410902
TEKT4	0.986242	0.013758	0.434333852	0.341993	1.009621388	2.971410902
TMEM31	0.986242	0.013758	0.434333852	0.341993	1.009621388	2.971410902
WDR63	0.986242	0.013758	0.434333852	0.341993	1.009621388	2.971410902
ZAR1	0.986242	0.013758	0.434333852	0.341993	1.009621388	2.971410902
ZDHHC8P	0.986242	0.013758	0.434333852	0.341993	1.009621388	2.971410902
ZP1	0.986242	0.013758	0.434333852	0.341993	1.009621388	2.971410902
NHLRC3	0.986243	0.013757	1.212002991	1.212179	710.7734573	586.3584181
CFAP58-A	0.986249	0.013751	2.360587065	3.037436	3.028864164	0.990470301
HSD3B2	0.986249	0.013751	2.360587065	3.037436	3.028864164	0.990470301
LINC0132	0.986249	0.013751	2.360587065	3.037436	3.028864164	0.990470301
LOC10192	0.986249	0.013751	2.360587065	3.037436	3.028864164	0.990470301
RDH12	0.986249	0.013751	2.360587065	3.037436	3.028864164	0.990470301
TAGLN3	0.986249	0.013751	2.360587065	3.037436	3.028864164	0.990470301
USH2A	0.986249	0.013751	2.360587065	3.037436	3.028864164	0.990470301
ZRANB2-A	0.986249	0.013751	2.360587065	3.037436	3.028864164	0.990470301
FAM57A	0.986249	0.013751	0.827611179	0.82744	404.8581767	489.2923286
ORC1	0.986254	0.013746	1.206046369	1.206363	390.7234772	323.8837884
MCOLN2	0.986254	0.013746	0.767144513	0.764556	33.31750581	43.58069323
RCC2	0.986259	0.013741	1.272752906	1.272772	9197.650846	7226.471314
NAPA-AS1	0.986267	0.013733	0.767155268	0.764556	33.31750581	43.58069323
PXN	0.986271	0.013729	1.245810427	1.245853	3542.761451	2843.640234
GRASP	0.986305	0.013695	0.787139428	0.785425	47.45220524	60.41868835

SOX6	0.986309	0.013691	0.547344977	0.510491	3.028864164	5.942821805
DDX12P	0.98631	0.01369	0.821302584	0.820728	126.9497933	154.6817469
ANKRD20	0.986327	0.013673	1.578302779	1.618495	11.58035732	7.151195572
C9orf43	0.986329	0.013671	0.715309994	0.70923	16.15394221	22.78081692
LSMEM1	0.986329	0.013671	0.715309994	0.70923	16.15394221	22.78081692
SBSPON	0.986329	0.013671	0.715309994	0.70923	16.15394221	22.78081692
LOC34105	0.986329	0.013671	0.437245013	0.345379	1.019717602	2.971410902
ZNF669	0.986329	0.013671	1.210651011	1.211202	224.3984497	185.2674698
GIPC1	0.986342	0.013658	1.258463738	1.258493	5450.945875	4331.326625
KLHL6	0.986347	0.013653	0.436828967	0.34428	1.009621388	2.951601496
ZNF28	0.986353	0.013647	1.225078183	1.225168	1484.880464	1211.979079
C1orf229	0.986354	0.013646	0.686837409	0.677984	11.66112703	17.20446912
PLEKHA6	0.986356	0.013644	1.220219949	1.220329	1201.449452	984.527479
TBC1D2B	0.986368	0.013632	0.815897961	0.815842	1327.652125	1627.342704
JAKMIP2	0.986375	0.013625	0.449743572	0.366036	1.161064596	3.189314368
STX2	0.986382	0.013618	0.821906878	0.821824	880.884565	1071.86715
PDP1	0.986387	0.013613	1.23794961	1.238005	2583.621132	2086.920924
CCDC121	0.986393	0.013607	1.245705727	1.247507	82.78895383	66.36151015
RNMTL1	0.986409	0.013591	0.827799102	0.827467	208.9916273	252.5699267
AGAP1-IT	0.986415	0.013585	0.54748808	0.510491	3.028864164	5.942821805
C10orf67	0.986415	0.013585	0.54748808	0.510491	3.028864164	5.942821805
DKFZp434	0.986415	0.013585	0.54748808	0.510491	3.028864164	5.942821805
DPCR1	0.986415	0.013585	0.54748808	0.510491	3.028864164	5.942821805
GPR162	0.986415	0.013585	0.54748808	0.510491	3.028864164	5.942821805
HOXA-AS:	0.986415	0.013585	0.54748808	0.510491	3.028864164	5.942821805
LOC10192	0.986415	0.013585	0.54748808	0.510491	3.028864164	5.942821805
DR1	0.986417	0.013583	1.234852949	1.234913	2405.927768	1948.255082
MRM1	0.986417	0.013583	1.207007499	1.207444	278.6555031	230.7795801
ARRDC3	0.98643	0.01357	0.818440183	0.818373	1081.304507	1321.287381
NRM	0.986431	0.013569	1.215760707	1.215892	974.2846396	801.2904733
PVRIG	0.986438	0.013562	0.305501757	0.008779	0	1.129136143
L3MBTL1	0.986441	0.013559	1.216310644	1.2171	161.5394221	132.7230203
DIS3L	0.986445	0.013555	0.824693632	0.824588	665.2900137	806.8172976
HDDC3	0.986448	0.013552	1.205787486	1.206102	384.6657489	318.9314368
HAVCR2	0.986466	0.013534	0.549055933	0.512187	3.038960378	5.942821805
LOC10192	0.986466	0.013534	0.549055933	0.512187	3.038960378	5.942821805
SNHG12	0.986474	0.013526	0.829004904	0.828809	351.3482431	423.9212887
NR2F2	0.986478	0.013522	0.826110234	0.825991	586.5900265	710.1672057
DYRK3	0.986481	0.013519	0.826508747	0.826105	172.6452574	208.9892335
PPIL6	0.986503	0.013497	1.205412606	1.205778	330.1865788	273.8353241
RHCE	0.986514	0.013486	1.561735475	1.600946	11.10583527	6.933292105
GPATCH3	0.986524	0.013476	1.20532217	1.205699	320.04998	265.4460406
CALM2	0.986524	0.013476	1.290657292	1.290668	17197.89073	13324.79696
FGFR4	0.986527	0.013473	0.828249651	0.8281	463.4162172	559.6157199
HECTD2-/	0.986528	0.013472	1.355122163	1.363516	28.06747459	20.58197285
TARBP2	0.986528	0.013472	1.203938665	1.204318	322.0692228	267.4269812
TBCE	0.986532	0.013468	1.215518903	1.215649	975.294261	802.2809436
FGD5P1	0.986534	0.013466	1.564299255	1.603854	11.1260277	6.933292105
ARMC1	0.986535	0.013465	1.229621655	1.229694	1912.222909	1555.038372
CDC37L1	0.986538	0.013462	0.828363668	0.828213	459.3777316	554.6633684
FBXL15	0.98654	0.01346	0.827470192	0.8271	186.7799568	225.8272286
APBA2	0.986551	0.013449	0.82669157	0.82657	571.4457057	691.3482699
LOC72855	0.986553	0.013447	1.205111806	1.205404	412.3091825	342.0490137
COX10	0.986556	0.013444	1.206550411	1.207097	227.1648123	188.1893571
TAF1B	0.986557	0.013443	1.21039254	1.210561	735.0043706	607.1582944
FAIM	0.986559	0.013441	1.205380461	1.205791	293.799824	243.655694

TWIST2	0.986563	0.013437	0.753152094	0.749586	25.2405347	33.67599023
ERVMER3	0.986576	0.013424	0.667207467	0.655535	9.086592493	13.86658421
GPR146	0.986576	0.013424	0.667207467	0.655535	9.086592493	13.86658421
LINC0046	0.986576	0.013424	0.667207467	0.655535	9.086592493	13.86658421
IL21R-AS1	0.986577	0.013423	0.297568055	0.008275	0	1.198469064
FLJ42351	0.986586	0.013414	1.561581561	1.600946	11.10583527	6.933292105
LGALS8-A	0.986586	0.013414	1.561581561	1.600946	11.10583527	6.933292105
PRSS36	0.986586	0.013414	1.561581561	1.600946	11.10583527	6.933292105
WDR43	0.986591	0.013409	1.242586499	1.242632	3202.519043	2577.203723
SULF2	0.986591	0.013409	0.799859061	0.799836	3502.376596	4378.8692
ZNF438	0.986591	0.013409	1.204638326	1.204949	386.6849917	320.9123775
KIAA0141	0.986593	0.013407	0.829232574	0.829086	479.5701594	578.4346557
NSUN6	0.986598	0.013402	1.205895727	1.20636	260.4823181	215.9225256
SLC12A8	0.986598	0.013402	0.761251412	0.758136	28.26939887	37.29120682
CENPK	0.986605	0.013395	1.204852029	1.205087	523.9935005	434.816462
CD55	0.986607	0.013393	1.259815873	1.259843	5970.90089	4739.400389
NUDCD2	0.986613	0.013387	1.210394273	1.210559	754.187177	623.0058192
UBL7	0.986616	0.013384	1.210495203	1.210658	760.2449053	627.9581707
HSPA12A	0.986625	0.013375	1.207581488	1.207765	679.4751942	562.5871308
PRSS8	0.986629	0.013371	1.350738286	1.358941	28.26939887	20.79987632
ST7-OT3	0.98663	0.01337	0.434082428	0.34085	1.019717602	3.011029714
DDX56	0.986633	0.013367	0.825670632	0.82557	709.7638359	859.7282211
ARHGAP3	0.986643	0.013357	1.215753523	1.216608	149.4239654	122.8183173
TAF1L	0.986649	0.013351	0.433654497	0.339736	1.009621388	2.991220308
SLC28A2	0.98665	0.01335	0.517746399	0.467436	2.170685985	4.655210414
GOLT1B	0.986666	0.013334	0.824960247	0.824866	762.2641481	924.1087906
ZNF527	0.986672	0.013328	1.209919755	1.210582	186.7294757	154.2459399
SYBU	0.986679	0.013321	1.207109664	1.207294	674.4270873	558.6252496
KTN1-AS1	0.98668	0.01332	1.38938207	1.401333	22.21167054	15.84752481
ZFP69	0.986683	0.013317	1.206562462	1.207097	227.1648123	188.1893571
APCDD1L	0.986698	0.013302	0.822856066	0.822276	122.164188	148.5705451
OGFR	0.986708	0.013292	0.81929654	0.819231	1099.477692	1342.087258
PGAM4	0.986708	0.013292	0.311189133	0.009014	0	1.099422034
NCCRP1	0.986713	0.013287	0.628950435	0.610773	6.057728329	9.924512414
STAG3L4	0.986714	0.013286	1.20221049	1.202543	364.4733211	303.083912
MRPL34	0.986715	0.013285	1.20998713	1.210152	749.13907	619.043938
ARHGAP2	0.98672	0.01328	0.809927519	0.809889	1963.7136	2424.671296
VSIG1	0.986721	0.013279	0.718693557	0.712416	15.52797695	21.80025132
B3GNT3	0.986723	0.013277	0.767417358	0.764561	30.28864164	39.61881203
IFIT1	0.986725	0.013275	1.224175281	1.225347	114.0872169	93.10420827
HOGA1	0.98673	0.01327	0.776487565	0.774096	35.20549781	45.48239621
FAM201A	0.986732	0.013268	1.243912795	1.245815	77.74084689	62.39962895
ALG1L9P	0.986736	0.013264	1.461179199	1.482227	16.15394221	10.89517331
TIRAP	0.98674	0.01326	1.210972743	1.211698	171.635636	141.6471577
HACE1	0.986742	0.013258	1.212516227	1.212655	905.6303852	746.8146068
CHORDC1	0.986747	0.013253	1.228566338	1.228638	1920.279688	1562.932421
DCDC1	0.986752	0.013248	0.436123643	0.341993	1.009621388	2.971410902
DLEC1	0.986752	0.013248	0.436123643	0.341993	1.009621388	2.971410902
DLX6	0.986752	0.013248	0.436123643	0.341993	1.009621388	2.971410902
HIST1H2E	0.986752	0.013248	0.436123643	0.341993	1.009621388	2.971410902
LINC0033	0.986752	0.013248	0.436123643	0.341993	1.009621388	2.971410902
LINC0123	0.986752	0.013248	0.436123643	0.341993	1.009621388	2.971410902
MAGEA12	0.986752	0.013248	0.436123643	0.341993	1.009621388	2.971410902
MYO1A	0.986752	0.013248	0.436123643	0.341993	1.009621388	2.971410902
NOX4	0.986752	0.013248	0.436123643	0.341993	1.009621388	2.971410902
PXT1	0.986752	0.013248	0.436123643	0.341993	1.009621388	2.971410902

RGS4	0.986752	0.013248	0.436123643	0.341993	1.009621388	2.971410902
RP1L1	0.986752	0.013248	0.436123643	0.341993	1.009621388	2.971410902
SCN4B	0.986752	0.013248	0.436123643	0.341993	1.009621388	2.971410902
SEPSECS	0.986752	0.013248	0.436123643	0.341993	1.009621388	2.971410902
SEZ6	0.986752	0.013248	0.436123643	0.341993	1.009621388	2.971410902
SIX2	0.986752	0.013248	0.436123643	0.341993	1.009621388	2.971410902
SLC36A2	0.986752	0.013248	0.436123643	0.341993	1.009621388	2.971410902
SOX5	0.986752	0.013248	0.436123643	0.341993	1.009621388	2.971410902
SPATA9	0.986752	0.013248	0.436123643	0.341993	1.009621388	2.971410902
TNFRSF8	0.986752	0.013248	0.436123643	0.341993	1.009621388	2.971410902
EFEMP2	0.986755	0.013245	0.812268361	0.811327	78.75046828	97.06608948
CYCS	0.986757	0.013243	1.232735233	1.232797	2273.606789	1844.265605
LUC7L	0.986765	0.013235	0.830259095	0.83011	469.4739455	565.5585417
ASB12	0.986766	0.013234	0.630148268	0.611993	6.057728329	9.904703008
PNMA5	0.986766	0.013234	0.630148268	0.611993	6.057728329	9.904703008
RND1	0.986766	0.013234	0.630148268	0.611993	6.057728329	9.904703008
TOB1-AS1	0.986766	0.013234	0.630148268	0.611993	6.057728329	9.904703008
ABCC2	0.986769	0.013231	1.315500817	1.321241	35.33674859	26.74269812
ATAD3B	0.986771	0.013229	0.830769106	0.830582	365.4021728	439.9371935
TBC1D2	0.986773	0.013227	1.231217497	1.231281	2246.407589	1824.446294
C11orf84	0.986774	0.013226	1.219744226	1.219843	1320.584776	1082.584039
CBLN1	0.986778	0.013222	0.548260308	0.510491	3.028864164	5.942821805
FAM212A	0.986778	0.013222	0.548260308	0.510491	3.028864164	5.942821805
LOC10192	0.986778	0.013222	0.548260308	0.510491	3.028864164	5.942821805
NMB	0.986778	0.013222	0.548260308	0.510491	3.028864164	5.942821805
RASL10A	0.986778	0.013222	0.548260308	0.510491	3.028864164	5.942821805
SEC14L5	0.986778	0.013222	0.548260308	0.510491	3.028864164	5.942821805
SIGLEC1	0.986778	0.013222	0.548260308	0.510491	3.028864164	5.942821805
STAB1	0.986778	0.013222	0.548260308	0.510491	3.028864164	5.942821805
FANCI	0.986784	0.013216	1.236600821	1.236654	2654.294629	2146.349142
MESP1	0.98679	0.01321	0.667462325	0.655535	9.086592493	13.86658421
SLC6A8	0.986793	0.013207	0.826273078	0.826173	716.8210894	867.6420788
C4orf47	0.986804	0.013196	0.799200065	0.79777	54.51955496	68.34245075
SAFB	0.986809	0.013191	0.808569556	0.808535	2161.12487	2672.893058
ADPRHL1	0.986812	0.013188	1.232398819	1.233901	92.88516771	75.27574286
LOC10013	0.986821	0.013179	1.202890853	1.203286	300.4835175	249.7173722
MPP7	0.986822	0.013178	1.207646294	1.208337	180.7222285	149.5610154
MAP3K14-	0.986826	0.013174	0.778001379	0.77563	35.33674859	45.56163384
SEC16B	0.986826	0.013174	0.778001379	0.77563	35.33674859	45.56163384
ARL17B	0.986834	0.013166	1.223132731	1.224368	110.07902	89.9049892
CPED1	0.986835	0.013165	1.560750768	1.600946	11.10583527	6.933292105
DUSP27	0.986835	0.013165	1.560750768	1.600946	11.10583527	6.933292105
LINC0093	0.986835	0.013165	1.560750768	1.600946	11.10583527	6.933292105
RTCA-AS1	0.986835	0.013165	1.560750768	1.600946	11.10583527	6.933292105
SEL1L3	0.986839	0.013161	1.261957446	1.261981	6926.002723	5488.195937
SYNGAP1	0.986842	0.013158	0.830084303	0.829945	504.8106941	608.2478117
SOHLH2	0.986844	0.013156	0.302842255	0.008484	0	1.168754955
AGTRAP	0.986848	0.013152	1.238151575	1.238202	2850.161179	2301.852979
TCTA	0.986849	0.013151	1.205054965	1.205275	543.1763068	450.6639869
TM6SF2	0.986851	0.013149	0.823355037	0.823276	915.7265991	1112.298148
LMX1B	0.986854	0.013146	0.793591235	0.791915	47.45220524	59.9234532
DLG5	0.986856	0.013144	0.797311869	0.797292	4056.658738	5088.045935
PLAGL2	0.986856	0.013144	0.828060749	0.827941	576.4938126	696.3006214
LOC10192	0.986857	0.013143	0.449747272	0.356193	1.009621388	2.852554466
RNASEH1	0.986858	0.013142	0.830607604	0.830272	203.9435204	245.6366346
KCND1	0.986861	0.013139	0.806598124	0.805425	64.61576884	80.22809436

PMF1	0.986867	0.013133	0.832633209	0.832413	315.1230277	378.5676537
GOLGA8S	0.986874	0.013126	1.712509332	1.799197	7.350043706	4.080737639
FRMD4B	0.986884	0.013116	0.809536223	0.808461	69.66387578	86.17091617
ZNF785	0.986886	0.013114	0.814513738	0.813573	79.79037831	98.07636918
TUBG1	0.986898	0.013102	1.234073262	1.23413	2466.010337	1998.174785
CWF19L2	0.9869	0.0131	1.201470959	1.201737	452.3103819	376.3787143
GTF3A	0.986901	0.013099	1.226513438	1.226587	1828.424334	1490.657803
GPR180	0.986904	0.013096	0.824476704	0.824393	858.1781799	1040.984286
SMC6	0.986911	0.013089	1.22758238	1.227652	2005.108077	1633.285526
PCDHGB7	0.986915	0.013085	0.782423785	0.780218	37.44685729	47.99819078
UTS2B	0.986916	0.013084	0.59070248	0.563	4.038485553	7.180909681
HIST1H4K	0.986918	0.013082	0.604536269	0.580277	4.593777316	7.923762406
ATP2A1-A	0.986923	0.013077	1.245682869	1.247764	71.68311856	57.44727744
TTN-AS1	0.986926	0.013074	1.206308368	1.20693	194.8569279	161.446659
LINC0158	0.986927	0.013073	1.206959173	1.207607	187.7895782	155.5038372
PLEKHA8	0.986928	0.013072	1.330174714	1.337244	31.07614633	23.23643326
LOC10192	0.986937	0.013063	1.696515906	1.781863	7.067349717	3.961881203
LOC10192	0.986937	0.013063	1.696515906	1.781863	7.067349717	3.961881203
SAP25	0.986937	0.013063	1.696515906	1.781863	7.067349717	3.961881203
FBXW8	0.986945	0.013055	0.829452179	0.82932	519.9550149	626.9677004
HEIH	0.986945	0.013055	1.207109425	1.207272	765.2930122	633.9009925
TCEB3-AS	0.986947	0.013053	0.823379298	0.822762	114.0872169	138.6658421
SNRPF	0.986951	0.013049	1.233469967	1.233528	2436.21641	1974.99778
TBXAS1	0.986959	0.013041	0.76072481	0.757291	26.25015609	34.66646053
KATNBL1	0.986966	0.013034	0.454432326	0.361994	1.029813816	2.862459169
PCGF3	0.986966	0.013034	1.221391487	1.221477	1573.999744	1288.601861
ZNF791	0.986967	0.013033	1.203998825	1.204214	554.7566642	460.6776416
ING4	0.986969	0.013031	0.832035519	0.831682	196.8761707	236.7224019
C8orf76	0.986971	0.013029	1.20880823	1.208963	795.1273242	657.6920891
DUSP19	0.986976	0.013024	1.229442895	1.2309	93.91498153	76.29592727
ANAPC1P	0.986977	0.013023	3.134317235	107.0102	1.060102458	0
LOC10192	0.986977	0.013023	3.134317235	107.0102	1.060102458	0
PRR7-AS1	0.986978	0.013022	1.692755458	1.777431	7.067349717	3.971785906
PLXNB1	0.986989	0.013011	0.824919111	0.824831	800.6297608	970.6608948
ZBED1	0.987003	0.012997	0.82460394	0.824519	824.1236505	999.5231993
LOC10012	0.98701	0.01299	1.43910264	1.458384	16.15394221	11.07345796
SLC27A2	0.987011	0.012989	0.802316723	0.800935	55.52917635	69.33292105
CATSPER	0.987012	0.012988	1.305109584	1.310462	36.34636997	27.73316842
ETAA1	0.987024	0.012976	1.209501128	1.209647	847.0723447	700.2625026
SMIM24	0.987026	0.012974	1.368238322	1.378876	23.22129193	16.83799511
C7orf55	0.987027	0.012973	1.253976292	1.256486	61.87969488	49.24618335
CCDC144	0.98703	0.01297	3.081869692	103.9814	1.029813816	0
GABRQ	0.987031	0.012969	0.630539278	0.611993	6.057728329	9.904703008
LGI4	0.987031	0.012969	0.630539278	0.611993	6.057728329	9.904703008
SLC7A9	0.987031	0.012969	0.630539278	0.611993	6.057728329	9.904703008
SLC9B1	0.987031	0.012969	0.630539278	0.611993	6.057728329	9.904703008
RNGTT	0.987033	0.012967	1.217273875	1.217376	1266.065221	1039.993816
LOC10574	0.987034	0.012966	1.284956529	1.289074	43.41371969	33.67599023
HIST2H2A	0.987046	0.012954	0.778772876	0.776313	33.8223165	43.57078853
ATG16L2	0.987053	0.012947	0.833000798	0.832822	387.6946131	465.5210414
HIST2H2A	0.987058	0.012942	0.778783096	0.776313	33.8223165	43.57078853
GRM6	0.987059	0.012941	0.319110088	0.009348	0	1.059803222
LOC72898	0.987059	0.012941	0.319110088	0.009348	0	1.059803222
C3orf14	0.98706	0.01294	0.811906795	0.810857	70.67349717	87.16138647
HERC6	0.987066	0.012934	1.211136559	1.211269	942.9863765	778.5096564
BMP6	0.987074	0.012926	1.209639409	1.210444	153.462451	126.7801985

STK19	0.987074	0.012926	1.202238584	1.202474	503.8010727	418.9689372
LOC10050	0.98708	0.01292	0.816307814	0.815405	80.76971105	99.05693478
SEPSECS	0.98708	0.01292	1.206989854	1.207144	804.6682464	666.5865124
LINC0144	0.987085	0.012915	0.513037341	0.458783	2.039435204	4.457116353
BMS1P6	0.987089	0.012911	1.200686011	1.201198	234.4542788	195.1820775
NCAPH2	0.987098	0.012902	0.826164695	0.826072	755.1967983	914.2040876
ZNF397	0.987101	0.012899	1.200651928	1.200935	413.9447691	344.6836647
TINCR	0.987103	0.012897	0.807212465	0.805937	60.61766815	75.21631464
DDX11L9	0.987106	0.012894	0.308306793	0.008703	0	1.139040846
SLC16A4	0.987114	0.012886	0.751507878	0.747348	22.21167054	29.72401373
KSR2	0.987114	0.012886	0.800463575	0.798957	51.4906908	64.44990247
FRS3	0.987115	0.012885	1.202171423	1.202697	225.1455696	187.1988868
GNAO1	0.987128	0.012872	0.790524444	0.788583	41.39447691	52.49492594
PLEKHA2	0.987129	0.012871	1.243197255	1.243238	3621.582593	2913.022678
TMEM110	0.987131	0.012869	0.598763748	0.572949	4.42214168	7.725668346
HABP4	0.987133	0.012867	1.213748103	1.213863	1102.506556	908.2612658
CACNG8	0.987133	0.012867	0.779982434	0.777526	33.88289379	43.58069323
SNRPE	0.987151	0.012849	1.22172145	1.221804	1590.153686	1301.477975
CFAP61	0.987154	0.012846	1.368004628	1.378876	23.22129193	16.83799511
LGALS8	0.987156	0.012844	0.826142718	0.826052	772.3603619	935.0039639
PPP1R2P	0.987156	0.012844	3.093663574	104.991	1.03991003	0
C1orf226	0.987156	0.012844	1.202590871	1.202811	536.1089571	445.7116353
ZNF275	0.987166	0.012834	0.829712165	0.829591	569.4264629	686.3959184
SLC35B3	0.987177	0.012823	1.202735486	1.202927	635.0518532	527.9206703
INTS6-AS	0.98718	0.01282	0.767736889	0.764568	27.25977748	35.65693083
MYO9A	0.987187	0.012813	1.212774643	1.212892	1069.18905	881.5185677
AKTIP	0.987188	0.012812	0.824747814	0.824666	851.1108302	1032.070053
RPS26P1	0.98719	0.01281	0.326540965	0.009707	0	1.02018441
TRIM50	0.98719	0.01281	0.326540965	0.009707	0	1.02018441
FSD1	0.98719	0.01281	0.832972799	0.832794	378.6080206	454.6258681
PRR36	0.987193	0.012807	0.818408363	0.817505	81.77933244	100.0375004
TNFRSF2	0.987196	0.012804	0.829772061	0.8293	145.3854799	175.3132432
C11orf86	0.987206	0.012794	1.49779894	1.528336	12.11545666	7.923762406
CLDND2	0.987206	0.012794	1.49779894	1.528336	12.11545666	7.923762406
RORC	0.987206	0.012794	1.49779894	1.528336	12.11545666	7.923762406
SEMA3B-/	0.987206	0.012794	1.49779894	1.528336	12.11545666	7.923762406
ACSM1	0.987207	0.012793	3.041048717	101.9621	1.009621388	0
AGBL5-AS	0.987207	0.012793	3.041048717	101.9621	1.009621388	0
C7orf69	0.987207	0.012793	3.041048717	101.9621	1.009621388	0
CFAP65	0.987207	0.012793	3.041048717	101.9621	1.009621388	0
FAS-AS1	0.987207	0.012793	3.041048717	101.9621	1.009621388	0
GPAT2	0.987207	0.012793	3.041048717	101.9621	1.009621388	0
HERC2P1	0.987207	0.012793	3.041048717	101.9621	1.009621388	0
IYD	0.987207	0.012793	3.041048717	101.9621	1.009621388	0
LAMB4	0.987207	0.012793	3.041048717	101.9621	1.009621388	0
LHFPL5	0.987207	0.012793	3.041048717	101.9621	1.009621388	0
LINC0116	0.987207	0.012793	3.041048717	101.9621	1.009621388	0
LINC0150	0.987207	0.012793	3.041048717	101.9621	1.009621388	0
LOC10192	0.987207	0.012793	3.041048717	101.9621	1.009621388	0
LOC33987	0.987207	0.012793	3.041048717	101.9621	1.009621388	0
MAGEA8-/	0.987207	0.012793	3.041048717	101.9621	1.009621388	0
MROH5	0.987207	0.012793	3.041048717	101.9621	1.009621388	0
MS4A7	0.987207	0.012793	3.041048717	101.9621	1.009621388	0
PTPRT	0.987207	0.012793	3.041048717	101.9621	1.009621388	0
RALYL	0.987207	0.012793	3.041048717	101.9621	1.009621388	0
RGS7	0.987207	0.012793	3.041048717	101.9621	1.009621388	0

ROPN1	0.987207	0.012793	3.041048717	101.9621	1.009621388	0
SNORA12	0.987207	0.012793	3.041048717	101.9621	1.009621388	0
RNF5P1	0.987213	0.012787	1.205875004	1.206039	734.8630236	609.3175196
ATP5SL	0.987219	0.012781	1.211468532	1.211592	1011.640631	834.9664636
ULK2	0.987225	0.012775	0.820992902	0.820929	1113.733546	1356.676885
N6AMT2	0.987226	0.012774	1.204601916	1.205281	176.6837429	146.5896045
NPHP4	0.987228	0.012772	0.829265556	0.829153	611.8305612	737.9003741
IGSF11	0.987231	0.012769	0.720541779	0.713679	14.13469943	19.80940602
ZBTB8B	0.987231	0.012769	0.720541779	0.713679	14.13469943	19.80940602
RBMXL1	0.987236	0.012764	0.834071201	0.833834	283.8146684	340.3751189
SNRNP35	0.987241	0.012759	1.19762004	1.198016	297.8383095	248.6080455
YPEL2	0.987242	0.012758	0.835043473	0.834805	287.7420956	344.6836647
RAET1G	0.987244	0.012756	3.073336743	103.9814	1.029813816	0
BLACAT1	0.987249	0.012751	0.767807587	0.764568	27.25977748	35.65693083
TSSK3	0.987252	0.012748	1.69480047	1.781863	7.067349717	3.961881203
MATN1-AS	0.987253	0.012747	0.779261236	0.77669	32.30788442	41.59975263
BRICD5	0.987265	0.012735	0.779271911	0.77669	32.30788442	41.59975263
ZNF682	0.987267	0.012733	0.809769167	0.808527	61.63738575	76.23649905
GOLGA6L	0.98727	0.01273	1.198728312	1.199045	366.7146806	305.8374195
ZNF43	0.987271	0.012729	1.201644048	1.201857	553.2422321	460.3210723
TMEM183	0.987271	0.012729	0.835202962	0.834971	295.4656992	353.8653244
POTEJ	0.987275	0.012725	0.734479134	0.728791	16.47702105	22.61243697
ALDH9A1	0.987278	0.012722	1.22177047	1.221851	1644.673241	1346.049139
PLCB4	0.987278	0.012722	1.233878115	1.233931	2647.22728	2145.358671
ZBTB49	0.987288	0.012712	1.219551646	1.220806	103.991003	85.18044587
PCDHB15	0.987298	0.012702	0.829292561	0.828733	125.7584401	151.7499548
FNTB	0.987306	0.012694	0.833205003	0.833032	389.7643369	467.8882654
NMUR1	0.987317	0.012683	0.325320579	0.009615	0	1.030089113
PRRG4	0.987329	0.012671	0.834200557	0.834006	345.2905148	414.0165857
LOC10192	0.987331	0.012669	3.053009912	102.9718	1.019717602	0
TBC1D3P	0.987331	0.012669	3.053009912	102.9718	1.019717602	0
ZNF837	0.987335	0.012665	1.349380203	1.358912	24.23091332	17.82846541
APOBEC3	0.987343	0.012657	0.73750211	0.732002	16.99192796	23.21662385
ITGA2B	0.987347	0.012653	0.794722696	0.792855	42.4040983	53.48539624
MCAT	0.987348	0.012652	0.834811832	0.834567	273.6073962	327.8456696
DGCR11	0.987351	0.012649	0.760160833	0.75636	23.22129193	30.70457932
MYLK3	0.987353	0.012647	1.361019924	1.371685	23.10013736	16.83799511
TMEM18	0.987355	0.012645	1.198224462	1.198471	479.5701594	400.1500015
ZSCAN23	0.987362	0.012638	0.811479801	0.810265	62.59652607	77.25668346
CBR1	0.987364	0.012636	1.24223121	1.242271	3656.848668	2943.677734
SPRY3	0.987367	0.012633	0.808338306	0.807001	57.54841913	71.31386166
GPR37L1	0.987371	0.012629	0.659554964	0.644361	7.016868648	10.89517331
GOLGA8F	0.987372	0.012628	0.744759042	0.739834	19.07174802	25.78194193
FOXD3-AS	0.987383	0.012617	0.808308926	0.807001	57.54841913	71.31386166
SF3A3	0.987385	0.012615	1.231867476	1.231923	2509.918771	2037.397409
LTA4H	0.987387	0.012613	1.259109477	1.259133	6637.251006	5271.282941
KDF1	0.987387	0.012613	1.224436448	1.225927	89.85630355	73.29480226
DCPS	0.987391	0.012609	1.205774662	1.205928	789.5239255	654.7008688
FAAP100	0.987405	0.012595	0.821894692	0.821831	1121.689362	1364.868074
TMEM128	0.987409	0.012591	1.203143632	1.20331	732.9851278	609.139235
A1CF	0.987418	0.012582	3.032683081	101.9621	1.009621388	0
A3GALT2	0.987418	0.012582	3.032683081	101.9621	1.009621388	0
ABHD12B	0.987418	0.012582	3.032683081	101.9621	1.009621388	0
ACP7	0.987418	0.012582	3.032683081	101.9621	1.009621388	0
ACTA1	0.987418	0.012582	3.032683081	101.9621	1.009621388	0
ADAMTSL	0.987418	0.012582	3.032683081	101.9621	1.009621388	0

ANGPTL7	0.987418	0.012582	3.032683081	101.9621	1.009621388	0
ANGPTL8	0.987418	0.012582	3.032683081	101.9621	1.009621388	0
ANKRD22	0.987418	0.012582	3.032683081	101.9621	1.009621388	0
ANKRD36	0.987418	0.012582	3.032683081	101.9621	1.009621388	0
ANKRD55	0.987418	0.012582	3.032683081	101.9621	1.009621388	0
ANO3	0.987418	0.012582	3.032683081	101.9621	1.009621388	0
ANXA13	0.987418	0.012582	3.032683081	101.9621	1.009621388	0
APOL5	0.987418	0.012582	3.032683081	101.9621	1.009621388	0
ARHGEF3	0.987418	0.012582	3.032683081	101.9621	1.009621388	0
ASCL1	0.987418	0.012582	3.032683081	101.9621	1.009621388	0
BAALC-AS	0.987418	0.012582	3.032683081	101.9621	1.009621388	0
BDKRB1	0.987418	0.012582	3.032683081	101.9621	1.009621388	0
BEND3P3	0.987418	0.012582	3.032683081	101.9621	1.009621388	0
BEST2	0.987418	0.012582	3.032683081	101.9621	1.009621388	0
BRCAT10	0.987418	0.012582	3.032683081	101.9621	1.009621388	0
C10orf82	0.987418	0.012582	3.032683081	101.9621	1.009621388	0
C17orf77	0.987418	0.012582	3.032683081	101.9621	1.009621388	0
C1QTNF8	0.987418	0.012582	3.032683081	101.9621	1.009621388	0
C1orf100	0.987418	0.012582	3.032683081	101.9621	1.009621388	0
C20orf141	0.987418	0.012582	3.032683081	101.9621	1.009621388	0
C21orf91-(	0.987418	0.012582	3.032683081	101.9621	1.009621388	0
C2orf61	0.987418	0.012582	3.032683081	101.9621	1.009621388	0
C2orf78	0.987418	0.012582	3.032683081	101.9621	1.009621388	0
C6orf58	0.987418	0.012582	3.032683081	101.9621	1.009621388	0
C7	0.987418	0.012582	3.032683081	101.9621	1.009621388	0
CACNA1C	0.987418	0.012582	3.032683081	101.9621	1.009621388	0
CACNA1I	0.987418	0.012582	3.032683081	101.9621	1.009621388	0
CAMK2A	0.987418	0.012582	3.032683081	101.9621	1.009621388	0
CAPN8	0.987418	0.012582	3.032683081	101.9621	1.009621388	0
CCDC152	0.987418	0.012582	3.032683081	101.9621	1.009621388	0
CCK	0.987418	0.012582	3.032683081	101.9621	1.009621388	0
CD2	0.987418	0.012582	3.032683081	101.9621	1.009621388	0
CD300A	0.987418	0.012582	3.032683081	101.9621	1.009621388	0
CDH7	0.987418	0.012582	3.032683081	101.9621	1.009621388	0
CDKN2A	0.987418	0.012582	3.032683081	101.9621	1.009621388	0
CFAP74	0.987418	0.012582	3.032683081	101.9621	1.009621388	0
CFL1P1	0.987418	0.012582	3.032683081	101.9621	1.009621388	0
CHADL	0.987418	0.012582	3.032683081	101.9621	1.009621388	0
CLDN10-A	0.987418	0.012582	3.032683081	101.9621	1.009621388	0
CLDN19	0.987418	0.012582	3.032683081	101.9621	1.009621388	0
CLVS1	0.987418	0.012582	3.032683081	101.9621	1.009621388	0
CMKLR1	0.987418	0.012582	3.032683081	101.9621	1.009621388	0
CNPY1	0.987418	0.012582	3.032683081	101.9621	1.009621388	0
CSAG4	0.987418	0.012582	3.032683081	101.9621	1.009621388	0
CT45A1	0.987418	0.012582	3.032683081	101.9621	1.009621388	0
CXCL11	0.987418	0.012582	3.032683081	101.9621	1.009621388	0
CXCR2P1	0.987418	0.012582	3.032683081	101.9621	1.009621388	0
CXorf21	0.987418	0.012582	3.032683081	101.9621	1.009621388	0
CYP1A2	0.987418	0.012582	3.032683081	101.9621	1.009621388	0
CYP2C19	0.987418	0.012582	3.032683081	101.9621	1.009621388	0
DDX25	0.987418	0.012582	3.032683081	101.9621	1.009621388	0
DEPDC1-A	0.987418	0.012582	3.032683081	101.9621	1.009621388	0
DIRAS2	0.987418	0.012582	3.032683081	101.9621	1.009621388	0
DKFZP434	0.987418	0.012582	3.032683081	101.9621	1.009621388	0
DMP1	0.987418	0.012582	3.032683081	101.9621	1.009621388	0
DMRTA2	0.987418	0.012582	3.032683081	101.9621	1.009621388	0

DSCAS	0.987418	0.012582	3.032683081	101.9621	1.009621388	0
DTHD1	0.987418	0.012582	3.032683081	101.9621	1.009621388	0
EIF1AY	0.987418	0.012582	3.032683081	101.9621	1.009621388	0
ENDOU	0.987418	0.012582	3.032683081	101.9621	1.009621388	0
EPN3	0.987418	0.012582	3.032683081	101.9621	1.009621388	0
EZR-AS1	0.987418	0.012582	3.032683081	101.9621	1.009621388	0
FAM19A3	0.987418	0.012582	3.032683081	101.9621	1.009621388	0
FAM26D	0.987418	0.012582	3.032683081	101.9621	1.009621388	0
FAM81B	0.987418	0.012582	3.032683081	101.9621	1.009621388	0
FAM83C-/	0.987418	0.012582	3.032683081	101.9621	1.009621388	0
FAM90A1	0.987418	0.012582	3.032683081	101.9621	1.009621388	0
FAM92B	0.987418	0.012582	3.032683081	101.9621	1.009621388	0
FER1L5	0.987418	0.012582	3.032683081	101.9621	1.009621388	0
FITM1	0.987418	0.012582	3.032683081	101.9621	1.009621388	0
FKSG29	0.987418	0.012582	3.032683081	101.9621	1.009621388	0
FLJ46284	0.987418	0.012582	3.032683081	101.9621	1.009621388	0
FOLH1B	0.987418	0.012582	3.032683081	101.9621	1.009621388	0
FOXP1-AS	0.987418	0.012582	3.032683081	101.9621	1.009621388	0
FRMD8P1	0.987418	0.012582	3.032683081	101.9621	1.009621388	0
GALNTL5	0.987418	0.012582	3.032683081	101.9621	1.009621388	0
GBP7	0.987418	0.012582	3.032683081	101.9621	1.009621388	0
GDF5	0.987418	0.012582	3.032683081	101.9621	1.009621388	0
GLYAT	0.987418	0.012582	3.032683081	101.9621	1.009621388	0
GLYATL1	0.987418	0.012582	3.032683081	101.9621	1.009621388	0
GNLY	0.987418	0.012582	3.032683081	101.9621	1.009621388	0
GOLGA6L	0.987418	0.012582	3.032683081	101.9621	1.009621388	0
GRIA3	0.987418	0.012582	3.032683081	101.9621	1.009621388	0
GRM7-AS	0.987418	0.012582	3.032683081	101.9621	1.009621388	0
GYPA	0.987418	0.012582	3.032683081	101.9621	1.009621388	0
HAMP	0.987418	0.012582	3.032683081	101.9621	1.009621388	0
HCG22	0.987418	0.012582	3.032683081	101.9621	1.009621388	0
HELT	0.987418	0.012582	3.032683081	101.9621	1.009621388	0
HIF3A	0.987418	0.012582	3.032683081	101.9621	1.009621388	0
HIST1H2A	0.987418	0.012582	3.032683081	101.9621	1.009621388	0
HIST1H2E	0.987418	0.012582	3.032683081	101.9621	1.009621388	0
HIST1H3A	0.987418	0.012582	3.032683081	101.9621	1.009621388	0
HIST1H3F	0.987418	0.012582	3.032683081	101.9621	1.009621388	0
HLA-DRA	0.987418	0.012582	3.032683081	101.9621	1.009621388	0
HMGB3P1	0.987418	0.012582	3.032683081	101.9621	1.009621388	0
HSD52	0.987418	0.012582	3.032683081	101.9621	1.009621388	0
IGFALS	0.987418	0.012582	3.032683081	101.9621	1.009621388	0
IL1RL2	0.987418	0.012582	3.032683081	101.9621	1.009621388	0
IRAIN	0.987418	0.012582	3.032683081	101.9621	1.009621388	0
IRX4	0.987418	0.012582	3.032683081	101.9621	1.009621388	0
IRX6	0.987418	0.012582	3.032683081	101.9621	1.009621388	0
JARID2-AS	0.987418	0.012582	3.032683081	101.9621	1.009621388	0
KCNB1	0.987418	0.012582	3.032683081	101.9621	1.009621388	0
KCNH4	0.987418	0.012582	3.032683081	101.9621	1.009621388	0
KCNH6	0.987418	0.012582	3.032683081	101.9621	1.009621388	0
KCNIP4	0.987418	0.012582	3.032683081	101.9621	1.009621388	0
KLRC2	0.987418	0.012582	3.032683081	101.9621	1.009621388	0
KRT18P55	0.987418	0.012582	3.032683081	101.9621	1.009621388	0
KRT33B	0.987418	0.012582	3.032683081	101.9621	1.009621388	0
KRT37	0.987418	0.012582	3.032683081	101.9621	1.009621388	0
KRTAP8-1	0.987418	0.012582	3.032683081	101.9621	1.009621388	0
LDLRAD2	0.987418	0.012582	3.032683081	101.9621	1.009621388	0

LINC0020	0.987418	0.012582	3.032683081	101.9621	1.009621388	0
LINC0024	0.987418	0.012582	3.032683081	101.9621	1.009621388	0
LINC0026	0.987418	0.012582	3.032683081	101.9621	1.009621388	0
LINC0032	0.987418	0.012582	3.032683081	101.9621	1.009621388	0
LINC0036	0.987418	0.012582	3.032683081	101.9621	1.009621388	0
LINC0054	0.987418	0.012582	3.032683081	101.9621	1.009621388	0
LINC0057	0.987418	0.012582	3.032683081	101.9621	1.009621388	0
LINC0085	0.987418	0.012582	3.032683081	101.9621	1.009621388	0
LINC0089	0.987418	0.012582	3.032683081	101.9621	1.009621388	0
LINC0090	0.987418	0.012582	3.032683081	101.9621	1.009621388	0
LINC0101	0.987418	0.012582	3.032683081	101.9621	1.009621388	0
LINC0108	0.987418	0.012582	3.032683081	101.9621	1.009621388	0
LINC0109	0.987418	0.012582	3.032683081	101.9621	1.009621388	0
LINC0109	0.987418	0.012582	3.032683081	101.9621	1.009621388	0
LINC0112	0.987418	0.012582	3.032683081	101.9621	1.009621388	0
LINC0124	0.987418	0.012582	3.032683081	101.9621	1.009621388	0
LINC0137	0.987418	0.012582	3.032683081	101.9621	1.009621388	0
LINC0139	0.987418	0.012582	3.032683081	101.9621	1.009621388	0
LINC0144	0.987418	0.012582	3.032683081	101.9621	1.009621388	0
LINC0152	0.987418	0.012582	3.032683081	101.9621	1.009621388	0
LINC0155	0.987418	0.012582	3.032683081	101.9621	1.009621388	0
LINC0161	0.987418	0.012582	3.032683081	101.9621	1.009621388	0
LOC1001	0.987418	0.012582	3.032683081	101.9621	1.009621388	0
LOC1001	0.987418	0.012582	3.032683081	101.9621	1.009621388	0
LOC1001	0.987418	0.012582	3.032683081	101.9621	1.009621388	0
LOC1001	0.987418	0.012582	3.032683081	101.9621	1.009621388	0
LOC1001	0.987418	0.012582	3.032683081	101.9621	1.009621388	0
LOC1001	0.987418	0.012582	3.032683081	101.9621	1.009621388	0
LOC1002	0.987418	0.012582	3.032683081	101.9621	1.009621388	0
LOC1005	0.987418	0.012582	3.032683081	101.9621	1.009621388	0
LOC1005	0.987418	0.012582	3.032683081	101.9621	1.009621388	0
LOC1006	0.987418	0.012582	3.032683081	101.9621	1.009621388	0
LOC1009	0.987418	0.012582	3.032683081	101.9621	1.009621388	0
LOC1009	0.987418	0.012582	3.032683081	101.9621	1.009621388	0
LOC1009	0.987418	0.012582	3.032683081	101.9621	1.009621388	0
LOC1010	0.987418	0.012582	3.032683081	101.9621	1.009621388	0
LOC1019	0.987418	0.012582	3.032683081	101.9621	1.009621388	0
LOC1019	0.987418	0.012582	3.032683081	101.9621	1.009621388	0
LOC1019	0.987418	0.012582	3.032683081	101.9621	1.009621388	0
LOC1019	0.987418	0.012582	3.032683081	101.9621	1.009621388	0
LOC1019	0.987418	0.012582	3.032683081	101.9621	1.009621388	0
LOC1019	0.987418	0.012582	3.032683081	101.9621	1.009621388	0
LOC1019	0.987418	0.012582	3.032683081	101.9621	1.009621388	0
LOC1019	0.987418	0.012582	3.032683081	101.9621	1.009621388	0
LOC1019	0.987418	0.012582	3.032683081	101.9621	1.009621388	0
LOC1019	0.987418	0.012582	3.032683081	101.9621	1.009621388	0
LOC1019	0.987418	0.012582	3.032683081	101.9621	1.009621388	0
LOC1019	0.987418	0.012582	3.032683081	101.9621	1.009621388	0
LOC1019	0.987418	0.012582	3.032683081	101.9621	1.009621388	0
LOC1019	0.987418	0.012582	3.032683081	101.9621	1.009621388	0
LOC1019	0.987418	0.012582	3.032683081	101.9621	1.009621388	0
LOC1019	0.987418	0.012582	3.032683081	101.9621	1.009621388	0
LOC1025	0.987418	0.012582	3.032683081	101.9621	1.009621388	0
LOC1027	0.987418	0.012582	3.032683081	101.9621	1.009621388	0
LOC1027	0.987418	0.012582	3.032683081	101.9621	1.009621388	0
LOC2556	0.987418	0.012582	3.032683081	101.9621	1.009621388	0

LOC34009	0.987418	0.012582	3.032683081	101.9621	1.009621388	0
LOC39070	0.987418	0.012582	3.032683081	101.9621	1.009621388	0
LOC64292	0.987418	0.012582	3.032683081	101.9621	1.009621388	0
LOC72995	0.987418	0.012582	3.032683081	101.9621	1.009621388	0
LPAR4	0.987418	0.012582	3.032683081	101.9621	1.009621388	0
LRRTM2	0.987418	0.012582	3.032683081	101.9621	1.009621388	0
LVCAT8	0.987418	0.012582	3.032683081	101.9621	1.009621388	0
MAGOH2F	0.987418	0.012582	3.032683081	101.9621	1.009621388	0
MAPK4	0.987418	0.012582	3.032683081	101.9621	1.009621388	0
MASP2	0.987418	0.012582	3.032683081	101.9621	1.009621388	0
MDS2	0.987418	0.012582	3.032683081	101.9621	1.009621388	0
MEG3	0.987418	0.012582	3.032683081	101.9621	1.009621388	0
MIP	0.987418	0.012582	3.032683081	101.9621	1.009621388	0
MIR210	0.987418	0.012582	3.032683081	101.9621	1.009621388	0
MIR4290H	0.987418	0.012582	3.032683081	101.9621	1.009621388	0
MIR658	0.987418	0.012582	3.032683081	101.9621	1.009621388	0
MIRLET7D	0.987418	0.012582	3.032683081	101.9621	1.009621388	0
MORC2-A	0.987418	0.012582	3.032683081	101.9621	1.009621388	0
MPP4	0.987418	0.012582	3.032683081	101.9621	1.009621388	0
MRLN	0.987418	0.012582	3.032683081	101.9621	1.009621388	0
MSMB	0.987418	0.012582	3.032683081	101.9621	1.009621388	0
MX2	0.987418	0.012582	3.032683081	101.9621	1.009621388	0
MYT1L	0.987418	0.012582	3.032683081	101.9621	1.009621388	0
NLRP5	0.987418	0.012582	3.032683081	101.9621	1.009621388	0
NOXO1	0.987418	0.012582	3.032683081	101.9621	1.009621388	0
NPW	0.987418	0.012582	3.032683081	101.9621	1.009621388	0
NR0B2	0.987418	0.012582	3.032683081	101.9621	1.009621388	0
OPALIN	0.987418	0.012582	3.032683081	101.9621	1.009621388	0
OPN5	0.987418	0.012582	3.032683081	101.9621	1.009621388	0
OR2J3	0.987418	0.012582	3.032683081	101.9621	1.009621388	0
OR2L3	0.987418	0.012582	3.032683081	101.9621	1.009621388	0
[truncated: 995,488 more chars]
